# Interpretable Machine Learning Models for Molecular Design of Tyrosine Kinase Inhibitors Using Variational Autoencoders and Perturbation-Based Approach of Chemical Space Exploration

**DOI:** 10.3390/ijms231911262

**Published:** 2022-09-24

**Authors:** Keerthi Krishnan, Ryan Kassab, Steve Agajanian, Gennady Verkhivker

**Affiliations:** 1 Keck Center for Science and Engineering, Graduate Program in Computational and Data Sciences, Schmid College of Science and Technology, Chapman University, Orange, CA 92866, USA; 2 Department of Biomedical and Pharmaceutical Sciences, School of Pharmacy, Chapman University, Irvine, CA 92618, USA

**Keywords:** autonomous molecular design, deep learning models, latent space landscapes, protein kinases, kinase inhibitors, perturbation chemical modeling, kinase inhibition likelihood classifiers, explainable machine learning

## Abstract

In the current study, we introduce an integrative machine learning strategy for the autonomous molecular design of protein kinase inhibitors using variational autoencoders and a novel cluster-based perturbation approach for exploration of the chemical latent space. The proposed strategy combines autoencoder-based embedding of small molecules with a cluster-based perturbation approach for efficient navigation of the latent space and a feature-based kinase inhibition likelihood classifier that guides optimization of the molecular properties and targeted molecular design. In the proposed generative approach, molecules sharing similar structures tend to cluster in the latent space, and interpolating between two molecules in the latent space enables smooth changes in the molecular structures and properties. The results demonstrated that the proposed strategy can efficiently explore the latent space of small molecules and kinase inhibitors along interpretable directions to guide the generation of novel family-specific kinase molecules that display a significant scaffold diversity and optimal biochemical properties. Through assessment of the latent-based and chemical feature-based binary and multiclass classifiers, we developed a robust probabilistic evaluator of kinase inhibition likelihood that is specifically tailored to guide the molecular design of novel SRC kinase molecules. The generated molecules originating from LCK and ABL1 kinase inhibitors yielded ~40% of novel and valid SRC kinase compounds with high kinase inhibition likelihood probability values (*p* > 0.75) and high similarity (Tanimoto coefficient > 0.6) to the known SRC inhibitors. By combining the molecular perturbation design with the kinase inhibition likelihood analysis and similarity assessments, we showed that the proposed molecular design strategy can produce novel valid molecules and transform known inhibitors of different kinase families into potential chemical probes of the SRC kinase with excellent physicochemical profiles and high similarity to the known SRC kinase drugs. The results of our study suggest that task-specific manipulation of a biased latent space may be an important direction for more effective task-oriented and target-specific autonomous chemical design models.

## 1. Introduction

The recent advances in machine learning (ML) have driven the design of new expert systems and automated workflows capable of modeling complex chemical and biological phenomena [1,2,3,4,5]. Generative deep learning models are often used for navigating an enormous chemical space of small molecules and molecular design [6,7,8]. Numerous generative-based approaches employing different neural network architectures, molecular representations, and analysis metrics have been developed and deployed in recent years, leading to significant progress in enabling autonomous molecular design. Deep neural network (DNN) models, such as variational autoencoders (VAE) [9], have been particularly useful in the molecular design of novel chemical probes. VAEs are a special type of autoencoders’ neural networks that compress molecular space into a continuous vector space representation. In this approach, the encoded data points are sampled from multidimensional statistical distribution and are then fed into the decoder that reconstructs the original input molecules. The objective function used for training includes a term penalizing reconstruction error and a term constraining the encoded parameters to be close to a normal distribution, which in turn facilitates proper sampling of the latent space [9]. Chemical variational autoencoder (ChemVAE) is a deep learning framework that converts discrete representations of molecules to and from a multidimensional continuous representation, enabling the generation of new molecules for efficient exploration and optimization via open-ended chemical spaces [9]. In this approach, the VAE is trained jointly with a model for property prediction that include the quantitative estimate of druglikeness (QED) [10], synthetic accessibility score (SAS) [11], and water–octanol partition coefficient (logP) [12]. This architecture ensures a smooth and continuous representation of both chemical structures and biochemical properties, which facilitates navigation in the latent space and the design of novel molecules with the desired properties.

Generative adversarial networks (GAN) represent another class of ML approaches successfully applied for de novo molecular design [13,14,15,16,17,18]. GANs are defined by a pair of neural networks, a generator, and a discriminator, trained in competition with each other. An approach termed LatentGAN combines an autoencoder and a generative adversarial neural network for de novo molecular design [14]. Another approach DruGAN integrates GAN and VAE architectural frameworks by training an adversarial autoencoder to efficiently sample molecules from the latent space [15]. MolGAN is an implicit generative model for molecular graphs that circumvents the need for expensive graph matching procedures and adapts the GAN approach to operate directly on graph-structured data [17]. The MolCycleGAN approach employs cycle-consistent adversarial networks to learn transformation rules between sets of compounds with desired and undesired values of the considered property [18]. The progress in GAN applications to molecular discovery has been further catalyzed by the development of several comprehensive benchmarking sets and cheminformatics’ infrastructure [19,20]. These validation platforms can evaluate the diversity and quality of the generated molecules by measuring the fidelity of GAN models in reproducing the property distribution of the training sets and assessing the ability to generate valid novel molecules for the goal-oriented and target-specific molecule generation processes. 

Generative-based ML models that enable the generation of a three-dimensional (3D) representation of small molecules have also received considerable attention for their unique advantages and potential to explicitly design drug-like molecules in a target-conditioning manner [21]. A novel molecular deep generative model adopts a recurrent neural network architecture coupled with a ligand–protein interaction fingerprint as constraints [22]. DeepLigBuilder is a novel deep learning-based method for de novo drug design that combines a graph-based generative model for the design of chemically and conformationally valid molecules with a Monte Carlo tree search to optimize the molecular and structural parameters [23]. A comprehensive review of generative models categorizes them into three types, depending on featurization of the 3D representation of small molecules: cubic grid-based, Euclidean distance matrix (EDM)-based, and Cartesian coordinate-based, where each type of featurization requires distinct generative architectures and optimization strategies [24]. Attention-based generative models for de novo molecular design offer new architectures that enable a more accurate sampling from the latent space and exploration of novel chemistry space not present in the training data [25]. Efficient multiobjective molecular design approaches combine in silico prediction of molecular property-defined desirability ranges and substructure constraints with particle swarm optimization for effective navigation in a continuous latent space [26,27,28,29]. The performances of various VAE and GAN models are typically evaluated based on goal-directed tasks (rediscovery, optimization, and scaffold hopping of active compounds) and target-specific objectives (generation of novel compounds for a given target) [30]. Various ML methods such as Extreme Gradient Boosting (XGBoost) [31], DNN models [32], and graph-based attention models [33] are applied to predict the selectivity of small molecules towards the proteins that belong to the same family. An extensive comparative analysis of the predictive capacity of the classical feature-based models (SVM, XGBoost, RF, and DNN) and four different graph-based neural network models has demonstrated a superior performance and exceptional computational efficiency of the XGBoost and RF methods [34]. By learning molecular descriptors and fingerprint features of over 8 million small molecules, a new feature-generation convolutional neural network (CNN) approach MolMap has outperformed existing ML models on several relevant benchmark datasets [35].

ML models have been actively deployed to help in the discovery of novel inhibitors targeting protein kinases, which are important oncology targets [36,37]. The generative tensorial reinforcement learning (GENTRL) approach has proposed a VAE model that compresses the space of small molecule structures onto the latent space in a high-dimensional lattice followed by exploration and optimization by reinforcement learning to discover novel kinase inhibitors [38]. A computational approach combining fragment-based design and deep generative modeling augmented by three-dimensional pharmacophore screening has been proposed for the systematic design of covalent protein kinase inhibitors [39]. ML models have shown the ability to distinguish between multitarget and single-target kinase inhibitors [40] as well as robust performance in predicting different chemical classes of kinase inhibitors [41]. By representing kinase inhibitors as a large number of molecular descriptors, feature-based ML models can provide an accurate classification of kinase probes according to their binding modes [42]. 

The design of novel and selective chemical probes to interrogate kinase functions remains a considerable challenge. The objective of the current investigation is the development and validation of an integrated ML approach for the autonomous molecular design of chemical probes for protein kinase target-specific tasks. Proto-oncogene tyrosine-protein kinase SRC (short for sarcoma) is a non-receptor tyrosine kinase protein that plays a vital role in regulating diverse cellular processes and is frequently overexpressed in various cancers [43]. We develop and implement a perturbation-based deep learning approach for the guided chemical transformation of small molecules and generic kinase molecules into potential chemical probes of SRC kinase. The proposed strategy combines ChemVAE embedding architecture with cluster decomposition and perturbation-based exploration of the continuous latent space to facilitate sampling along the interpretable controllable directions and allow for the efficient molecular design of specific chemical probes. The important feature of this generative approach is that molecules sharing similar structures tend to cluster in the latent space and interpolating between two molecules in the latent space could lead to smooth changes in the molecular structures and properties. Another advantage of the cluster-based perturbation strategy is a guided control over the navigation of the latent space that can increase both the performance and interpretability of the design predictions. A robust chemical feature-based machine learning predictor of the kinase inhibition likelihood is introduced to aid in the perturbation-based transformation of small molecules. By combining molecular perturbation design with the kinase inhibition likelihood analysis and similarity assessments, we demonstrate that the proposed strategy can morph kinase inhibitors into novel chemical probes of the SRC kinase that exhibit desirable ranges for all included chemical properties and similarity to the known potent SRC kinase inhibitors.

## 2. Results and Discussion

### 2.1. Integrative Machine Learning Model for Targeted Exploration of the Chemical Space 

**Figure 1:**
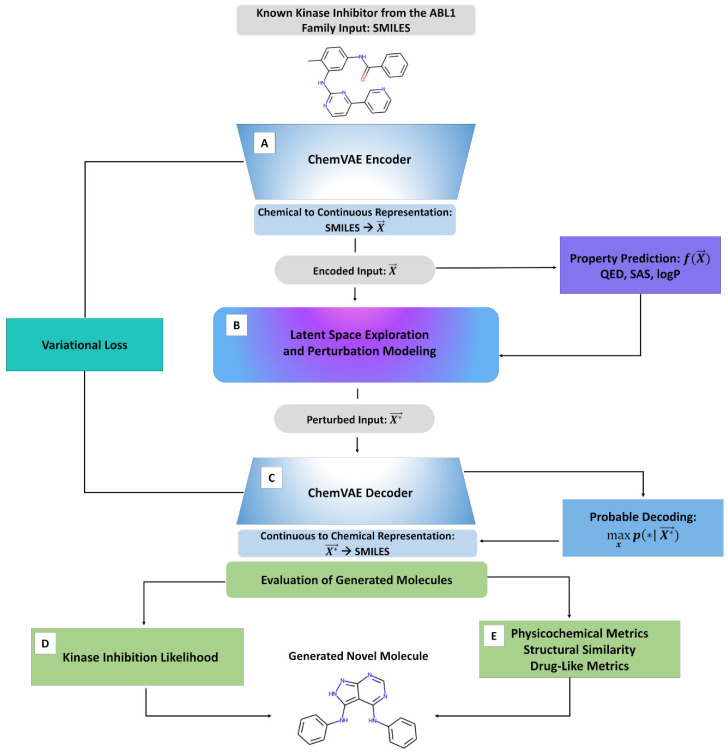
A schematic flow-chart of the generative molecular design approach that includes (**A**) ChemVAE encoding mechanism, (**B**) analysis and clustering of the latent space landscapes, (**C**) cluster-based and perturbation-driven controlled navigation of the latent space along interpretable directions, (**D**) kinase inhibition likelihood classifier, and (**E**) cheminformatics-based analysis of structural similarity, physicochemical properties and drug-like metrics of the generated molecules.

We began by introducing the proposed ML model and describing the components and main stages of the generative molecular design pipeline (Figure 1). The approach synergistically combined the following modules: (A) data mining and latent space analysis of small molecules and kinase inhibitors; (B) ChemVAE embedding of small molecules into the latent space; (C) clustering of molecule-specific embeddings; (D) cluster-based perturbation approach for efficient navigation of the continuous latent space; and (E) feature-based kinase inhibition likelihood classifier to guide optimization of the molecular properties and targeted exploration of the latent space (Figure 1). 

Many well-established and large datasets of generic small molecules and specialized collections of kinase inhibitors were utilized in the development of the generative approach presented in our study. The employed databases of small molecules and drug-like inhibitors included DrugBank (DrugBank Release Version 5.1.9) [44], BindingDB (2022 release version with 1.1 M compounds [45], BindingMoad (release version 2020) [46], ChEBI (ChEBI Release 213 with 60,229 fully annotated entities) [47], and ZINC15 database release of over 230 million compounds in ready-to-dock, 3D formats [48]. For datasets of kinase chemical probes, we used (a) ~20,000 known kinase inhibitors representing 10 major kinase families SRC, ABL1, CSF1R, EGFR, FLT3, KDR, LCK, MAPK10, MAPK14, and MET that were obtained from the ZINC15 database [48]; and (b) a dataset of competitive and allosteric protein kinase inhibitors confirmed by X-ray crystallography [49]. We also leveraged the size and synthetic feasibility of the GDB-17 [50] and FDB-17 [51] enumerated datasets of small molecules. For computational reasons, we utilized the GDB-17 lead set (~11 million compounds) and FDB-17 subset (~10 million molecules) selected from GDB-17 by applying fragment-likeness criteria and complexity reduction filters. The GDB-17 dataset has the most uniform distribution for the distinct chemical categories (heteroaromatic, aromatic, heterocyclic, carboxylic, acyclic) and provided an exceptionally high quality, large set of random small molecules to serve as a baseline for molecular generation experiments. 

An adaptation of the ChemVAE approach [9] was used for the embedding of small molecules into the latent space (Figure 1). An important component of the proposed methodology comes from a non-trivial targeted utilization of the ChemVAE framework that generates latent landscapes for small molecules and kinase inhibitors. This strategy enabled the model to generate more accurate and informative latent spaces while allowing for control over a modelled distribution. By performing cluster-based perturbation in the latent representation of the molecules, the generative design approach encouraged ChemVAE to explore the high-density distinct areas of the latent space for molecule generation, while also facilitating morphing of the kinase molecules from different families into SRC kinase-specific chemical probes (Figure 1). In this approach, the properties of generated molecules were controlled by sampling latent representations along linear interpretable directions that optimize the kinase inhibition likelihood metric. Based on the latent space analysis, it was assumed that molecules with similar structures would tend to cluster together in the latent space and that interpolating two molecules x1 and x2, represented by latent vectors z1 and z2, could lead to the intermediate molecules representing gradual transformation from molecule x1 to molecule x2. Given that molecular structures of small molecules generally have a tendency to correlate with their respective molecular properties, these assumptions implied that molecules with comparable properties would cluster together and interpolating between the two molecules with different values of the molecular property could lead to gradual changes in the molecular structures and property profiles of generated molecules.

After evaluating the optimal parameters, we examined each stage of the generative learning process and monitored the number of produced valid molecules when the chemical transformation was initiated from different kinase families (Figure 1). Several quantitative metrics assessed whether a combination of clustering and perturbation-based targeted exploration of the latent space could allow for efficient chemical transformation of the existing kinase inhibitors from different families into SRC chemical probes. We evaluated similarity between the generated novel molecules and known potent SRC kinase inhibitors. In addition, the main physicochemical properties of generated chemical probes were evaluated at the last stage of the process (Figure 1). Through this analysis, the chemical feasibility and property profiles of designed kinase probes were examined in greater detail.

### 2.2. Principal Component Analysis of the Latent Space Landscapes for Small Molecules and Kinase Inhibitors

**Figure 2:**
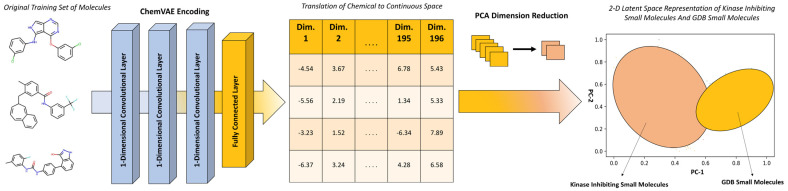
A schematic overview of the adaptation of ChemVAE-enabled transformation of chemical structures to the latent continuous space. PCA dimensionality reduction is used to transform the input from 196 dimensions to two dimensions. The 2D visualization of the latent space highlights differences between the spaces of GDB-17 small molecules and kinase inhibitors.

Using ChemVAE, the training sets of GDB-17 molecules and kinase inhibitors were encoded into a 196-dimensional vectorial representation in the continuous latent space. The 196-dimensional vectors representing these molecules were then fed through principal component analysis (PCA) to facilitate visualization and analysis of the latent space in two dimensions (Figure 2). For the latent space analysis, we employed a sample of the GDB-17 database corresponding to 163,953 random small molecules from a variety of domains, with the following atoms (C, N, O, S, F, Cl, Br, P, I) [50,51]. The set of kinase inhibitors included 1883 unique ABL1 kinase inhibitors and 3477 unique SRC kinase inhibitors. The chemical properties for these molecules were calculated using RDKit software (release 2022.03.1, T5 Informatics GmbH, Basel, Switzerland) for chemical data analysis [52]. The composite data included molecules with a molecular weight less than 700 Daltons, calculated logP < 6, and fewer than 10 rotatable bonds.

**Figure 3:**
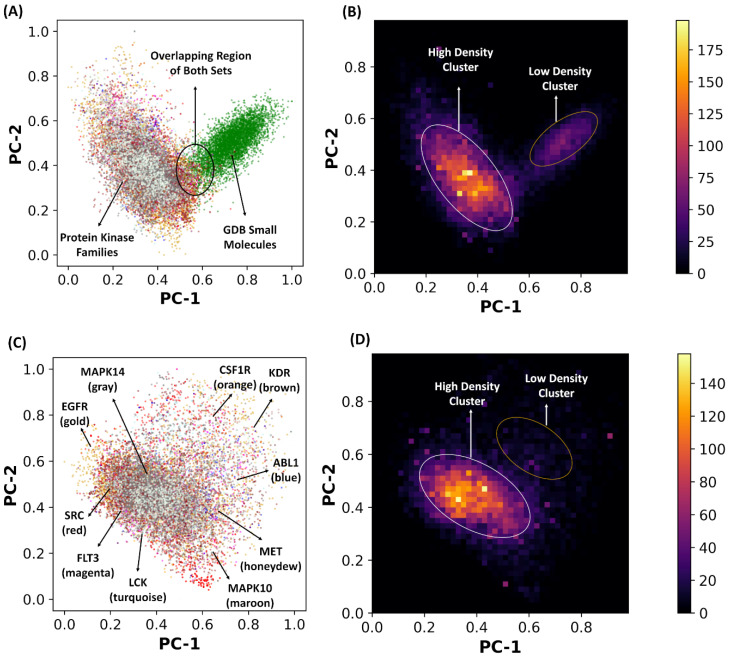
PCA and heatmaps of the latent spaces for small molecules and kinase inhibitors. (**A**) The 2-dimensional latent space representation of kinase molecules and GDB-17 small molecules dataset. Kinase molecules are shown in distinct colors for specific families, whereas GDB-7 small molecules are shown in green. The locations of the latent space for these classes of molecules are indicated by arrows and annotated. The overlapping region is indicated by the circle of the area on the graph. PC-1 accounts for 36.79% of variance and PC-2 accounts for 24.14% of the variance. (**B**) The 2-dimensional heatmap of latent space representation shown in (**A**). The density regions are color-coded with the high-density areas in yellow, whereas low-density regions are in purple. The high-density regions are also circled in white, whereas low-density regions are circled in gold. (**C**) The 2-dimensional latent space representation of the kinase inhibitors. The 10 kinase families in the dataset are SRC (red), ABL1(blue), EGFR (gold), CSF1R (orange), FLT3 (magenta), KDR (brown), LCK (turquoise), MAPK10 (maroon), MAPK14 (gray), MET (honeydew). PC-1 accounts for 44.92% of variance and PC-2 accounts for 33.63% of the variance. (**D**) The 2-dimensional heatmap of latent space representation shown in (**C**).

Using PCA decomposition of the latent space, we analyzed differences in the organization of the latent landscapes for small molecules and the kinase molecules. The two-dimensional latent space plots and heatmaps based on PCA were constructed to visualize and analyze densities of the regions in the latent space (Figure 3). Strikingly, we observed radical differences in the latent space distributions of the GDB-17 generic small molecules and the kinase inhibitors despite their comparable size (fewer than 10 rotatable bonds) and molecular weight (<700 Daltons). Indeed, the kinase inhibitors were found to be concentrated in one area of the latent space, while GDB-17 small molecules predominantly populated a different and non-overlapping region, pointing to significant differences in the respective latent space landscapes (Figure 3A,C). To further investigate the latent space, we constructed and examined the heatmap representations of the latent space that highlighted the distinct concentrations of the encoded molecules within various space regions. The density plots revealed somewhat skewed clusters in the latent space featuring highly concentrated and sparsely populated regions (Figure 3B,D). The presence of a broad high-density region for the kinase molecules suggested that optimal navigation in these populated areas of the latent space may allow for efficient molecule generation.

By visualizing the latent space of kinase inhibitors targeting different families, we also detected signs of the partial overlap in the latent space representation while showing that some regions of their latent space may be more family-specific. One of the focal observations of our experiments was the fact that the SRC kinase inhibitors and ABL1 kinase inhibitors were organized into distinct clusters in the latent space (Figure 3C). This analysis detected only small overlaps between the latent spaces of these kinase inhibitors, with some minor isolated islands seen for the ABL1 kinase inhibitors (Figure 3C). At the same time, a visual inspection of the PCA distributions suggested that the latent space of the SRC inhibitors could be fairly broad, with local clusters occupying different regions and displaying an overlap with the kinase inhibitors targeting different families. 

PCA allowed to reduce the dimensionality of the dataset while still preserving most of the variance by creating a new feature space based on the top eigenvectors. By computing the percentage of variance explained by the individual principal components (PCs), we found that a significant amount of variance in the data samples and the topology of the latent space landscape could be accounted for by the first two PCs (Figure 3). We observed that significant information of the data was accounted for by the principal components. For PCA of the latent space of the kinase molecules and GDB-17 small molecules dataset (Figure 3A), PC-1 accounts for 36.79% of variance and PC-2 accounts for 24.14% of the variance. For PCA of the latent space for the kinase inhibitors, PC-1 accounts for 44.92% of variance and PC-2 accounts for 33.63% of the variance. The high dimensionality of the dataset may be responsible for the observed percentages of variances as higher dimensionality introduces more variability and randomness. Therefore, we performed a statistical analysis of the dataset to assist in the visual analysis of the latent space. In particular, to aid in the analysis of the kinase molecules in the latent space, we calculated statistical measures including the general range, the range of the centroid vector, and the range of the standard deviation vector for each kinase family (Table S1). We found that each kinase family covered a similar minimum and maximum value in the encoded space, with all families occupying the same critical region of the latent space. These observations reinforced the notion that the encoded representations of the kinase molecules shared important chemical and functional characteristics common for molecules binding to kinase targets. The locations of the kinase molecules in the latent space corresponded to representations of specific chemical or molecular properties in those regions. 

The minimum standard deviation values were in the range of 0.65–0.86, while the maximum standard deviation values were within 1.29–1.63 (Table S1). The SRC kinase inhibitors were characterized by the largest minimum and maximum standard deviation values, which was reflected in a large distribution spread of the latent space region populated by these kinase molecules (Figure 3C,D). As a result, it may be assumed that most of the chemical kinase scaffolds encoded in the latent space of the kinase molecules could be potentially present within the latent space region populated by the SRC kinase molecules. In addition, a broad distribution of the latent space for the SRC kinase inhibitors indicated that targeted perturbations in the latent space of the kinase inhibitors targeting other families could transform these molecules into SRC inhibitors.

### 2.3. Latent Space and Chemical Feature-Based Kinase Inhibition Likelihood Classifiers

Using Random Forest (RF) architectures, we developed and compared several different ML classifiers of the kinase inhibition likelihood for the generated molecules. The models were constructed using the training set of kinase molecules from 10 kinase families combined with a set of GDB-17 small molecules. The binary and multiclass kinase inhibition likelihood classifiers were developed using both the latent space and chemical feature-based representations of the molecules. The binary classification models considered all kinase families (except for the target SRC kinase family) together with the dataset of GDB-17 molecules and assigned them a target value of 0, while the SRC kinase inhibitors were assigned a target value of 1. The multiclass classification models assigned each of the considered kinase families a different target variable value, with the goal to identify the differences between family-specific kinase inhibitors.

The binary latent space classification model was trained using the coordinates of each molecule in the latent space, where each molecule was represented by a total of 197 feature variables (196 latent space dimension variables and one classification variable). While there were distinct clusters of molecules within the latent space, a substantial number of features for each molecule, when converted to its latent space coordinates, decreased the overall accuracy (Table S2). The confusion matrix further supported this assessment showing that the binary latent space model displayed a better performance in classifying the non-SRC kinase molecules than the SRC kinase molecules (Table S3). Based on this analysis, we concluded that the overall predictive ability of the latent space-based binary classifier to identify a newly generated molecule as a potential SRC kinase inhibitor was not satisfactory. For the chemical feature-based kinase inhibition classifier, we considered a total of 20 different chemical features for each molecule during training and testing (see Materials and Methods for a detailed description of chemical features). With the exception of QED [15], SAS [16], and logP features [17] that are also an integral part of ChemVAE, an arbitrary selection of other chemical molecular features was utilized to allow for a more robust classification of the kinase inhibitors. The evaluation metrics and the confusion matrix for the binary chemical feature-based model displayed an excellent classification performance (Tables S4 and S5). The sensitivity of the specificity values showed that the chemical feature binary model accurately distinguished the SRC inhibitors from the non-SRC kinase molecules in comparison with the latent space binary model.

The latent space-based and chemical feature multiclass models considered the kinase inhibitors of different protein kinase families as distinct categories by assigning each class of the kinase inhibitors a different target variable value. The analysis of these classifiers indicated that the number of classification variables lowered the accuracy of the models in their predictions of the target values (Tables S6 and S7). Interestingly, it could be seen that both the multiclass RF model in the latent space (Table S6) and the multiclass chemical feature-based RF model (Table S7) were less efficient than the binary chemical feature classifier that was subsequently used in the generative molecular design pipeline (Figure 1) as the measure of the kinase inhibition likelihood guiding sampling of the latent space.

**Figure 4:**
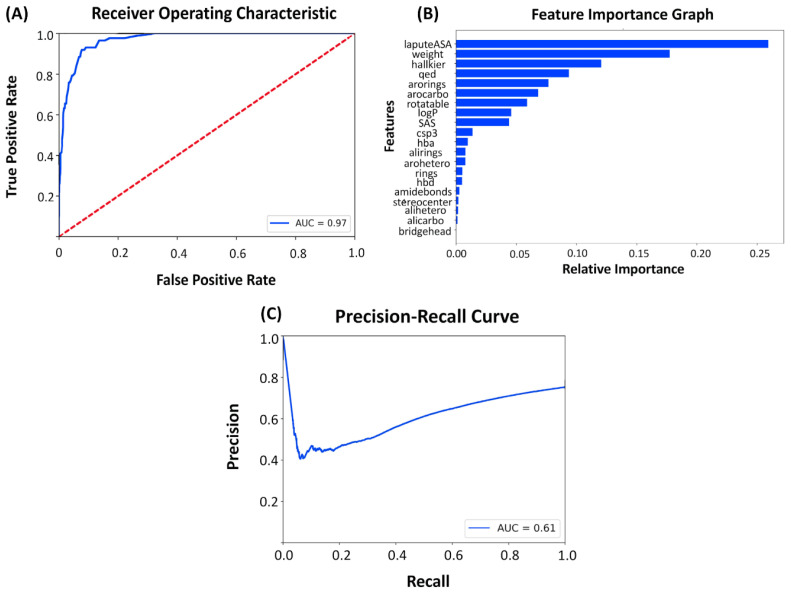
The performance and feature importance analysis of the chemical feature-based kinase inhibition classifier. (**A**) The receiver operating curve (ROC) is a graph where sensitivity is plotted as a function of 1-specificity. The area under the ROC is denoted as AUC. The ROC–AUC graph measures the performance of the classifier in differentiating the kinase inhibitor molecules from GDB-17 small molecules. (**B**) The feature importance analysis of the model. The importance of features is listed in descending order. (**C**) Precision–recall curve of the classification model.

The receiving operating characteristic (ROC) graph was used to evaluate the performance of the classifier that differentiated the kinase inhibitors from small molecules. The AUC of the model showed that the coverage of variation within the dataset was ~97% and that the model could accurately distinguish both classes of the non-SRC molecules and the SRC kinase inhibitors with a high level of certainty (Figure 4A). To identify the primary features in the classification prediction, we also performed the feature importance analysis (Figure 4B). The top 10 features that contributed the most to the predictive power of the model included the accessible surface area (labuteASA), molecular weight, Hall–Kier Alpha descriptor, the number of aromatic rings, aromaticity, the QED score, number of rotatable bonds, the logP score, the SAS score, and the number of hydrogen bond acceptors (Figure 4B). Hall–Kier α belongs to the class of topological descriptors that quantify molecular shape similarity within a set of molecules. Interestingly, among the most informative features of the binary classifier were QED, logP, and SAS scores that are also integral components of the ChemVAE encoder–decoder network. Hence, the results revealed that the SRC kinase molecules can be differentiated from other kinase molecules using a combination of fundamental chemical features (the number of aromatic rings, aromaticity, the number of rotatable bonds, and the number of hydrogen bond acceptors) and drug-like metrics. The precision–recall graph (Figure 4C) highlighted the accuracy of the model, showing that the model starting with a high precision value could eventually trade precision for recall to reach an equilibrium between the two metrics. 

### 2.4. Cluster-Based Perturbation Approach for Targeted Exploration of the Latent Space and Molecular Design

We developed a cluster-based perturbation approach to facilitate targeted sampling of the latent space. In this model, molecules were first clustered into groups in a non-biased manner allowing molecules with comparable properties to assemble into clusters. Accordingly, it was assumed that the molecules clustered within respective clusters would be characterized by specific molecular and chemical properties. To transform these molecules, we then invoked a controllable step of cluster-based perturbation. Using the centroid of each cluster as the representative of the properties, we navigated every data point in the cluster closer to the centroid by optimizing a set of parameters. Using this strategy, a cluster-based perturbation sampling explored the latent space along controllable directions yielding a diverse set of novel molecules featuring various molecular scaffolds. The generated molecules were then evaluated using the kinase inhibition likelihood classifier, and when the output probability was greater than 70%, the respective molecules were designated as SRC kinase-like inhibitors. 

**Figure 5:**
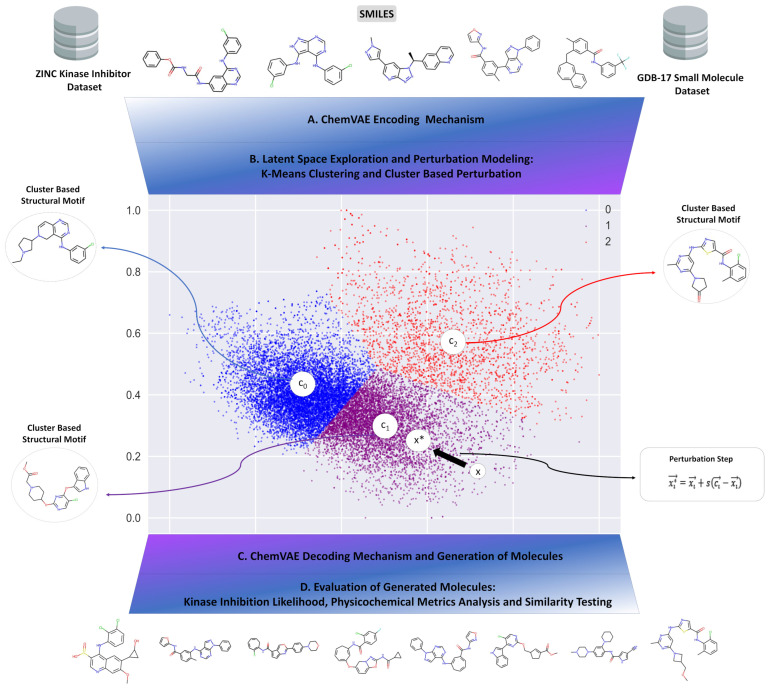
A schematic workflow of the cluster-based perturbation design implementation. K-means clustering is applied in the latent space, where different clusters represent specific molecular characteristics. The 3-cluster split is represented by the graph on the right, where the colors of blue, green, and orange indicate the 3 clusters, respectively. The centroids of each cluster, depicted by the labels of c_0_, c_1_, and c_2_, function as the representative of the structural motifs and molecular properties of that cluster. Utilizing the centroid, we modify our input by employing a cluster-based perturbation, as shown in the perturbation step, where c represents the centroid, x represents the original encoded molecule, and x* represents the molecule after perturbation. This implementation alters the encoded input such that it converges towards the centroid, and, in turn, generates molecules close to the specific motifs of the respective cluster. After the input is modified with the perturbation step, ChemVAE decodes the latent space areas and produces new molecules.

During the cluster-based perturbation stage of the generative design process, 1000 encoded molecules from each of the 10 kinase families were selected and processed through the pipeline to assess the quality of the predicted molecules and obtain the optimal parameters for the perturbation protocol. These parameters included the number of clusters, a scaling factor for the centroid-based targeted perturbation, and a noise level for a perturbation step (Figure 5). Using the results of 1000 independent generative experiments with different values of these parameters, a 3-cluster-based split, a scaling factor of 0.8, and a noise level of 5.0 were identified as an optimal set of parameters that guaranteed a high generation yield of valid and novel compounds.

After the perturbation step was executed, the perturbed vector was decoded using the ChemVAE network. During this stage, the probabilistic neural network performed 500 decoding attempts of the latent variable into a valid SMILES, each time subjecting the resulting molecules to a validity check via chemical property computations [52] including an assessment of the QED, logP, and SAS scores. When the network yielded at least 100 valid molecules out of the 500 decoding attempts, the generated molecular output was subsequently subjected to a comprehensive post-processing analysis in which various metrics were employed to evaluate the novelty and validity of the chemical transformation process. We found that together this generative molecular design pipeline produced a high yield of valid generated molecules while simultaneously morphing the inhibitors of different kinase families into potential SRC kinase molecules.

**Figure 6:**
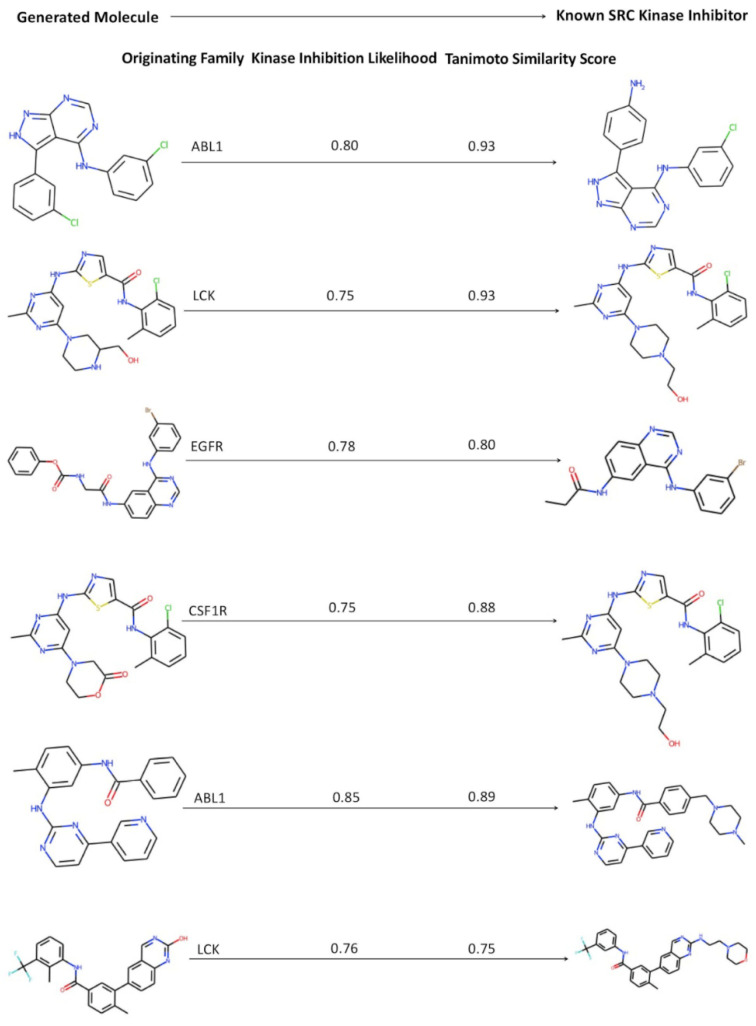
A selected sample of the generated molecules obtained using the perturbation approach and displaying the high kinase inhibition likelihood and considerable similarity to the existing SRC kinase inhibitors. (**Left panel**) The generated molecules along with the indication of the respective originating kinase family. (**Right panel**) The known SRC inhibitor to which the generated molecule has the highest similarity score to. The kinase inhibition likelihood score of the generated molecule and the similarity score between the known SRC inhibitor and the generated molecule are shown.

To illustrate the output of the generative pipeline, we compiled a list of several generated SRC-like kinase molecules that originated from the inhibitors of different kinase families. The presented molecules were characterized by the high kinase inhibition likelihood and a considerable similarity to the existing SRC kinase inhibitors (Figure 6). We noticed that some of the novel valid molecules with the highest similarity to the SRC inhibitors were produced starting from the latent space regions of the ABL1 and LCK kinase inhibitors. It is worth noting that perturbation-based exploration of the latent space was guided by a feature-based classifier and the kinase inhibition likelihood evaluation that did not include any direct measurements of similarity to the SRC molecules. A sample of generated molecules reflected both the diversity of molecular scaffolds and a high degree of synthetic feasibility that were enabled through a cluster-based perturbation approach (Figure 6).

### 2.5. Kinase Inhibition Likelihood and Similarity Analyses of the Generated Molecules

**Figure 7:**
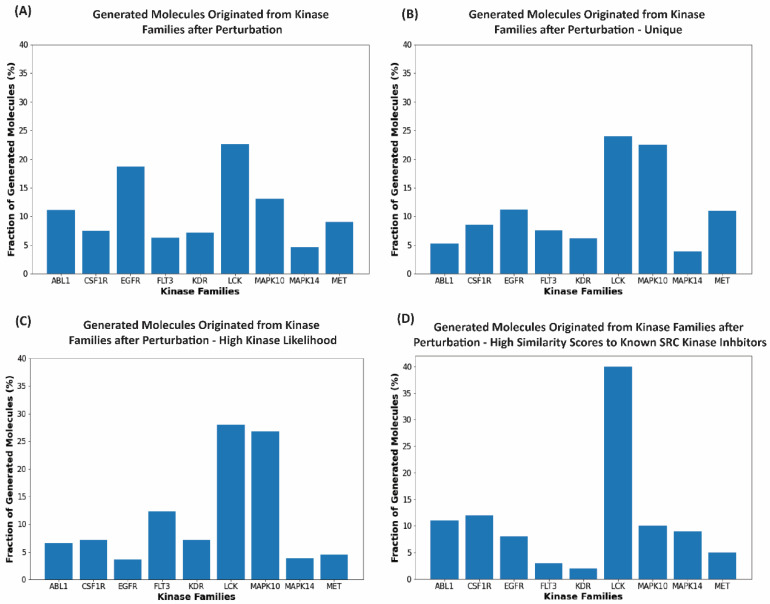
Analyses of the generated molecules. The histograms represent the fraction of the generated valid molecules with respect to the originated kinase inhibitor families. The horizontal axes represent the kinase inhibitor families from where the transformed output originated from. The vertical axes represent the percentage of molecules for each family from the set of molecules generated after each stage of evaluation. (**A**) The percentage of the generated molecules from each family after the initial perturbation experiment. (**B**) The percentage of the generated molecules from each family after removing the duplicate molecules. Due to the stochastic nature of the variational autoencoder, there are some molecules that are produced multiple times. (**C**) The percentage of generated molecules from each family that feature kinase inhibition likelihood probability score > 0.75. (**D**) The percentage of molecules from each family with the high kinase inhibition likelihood probability score and high similarity scores to the known SRC kinase inhibitors. The generated molecules considered in this analysis are in the top one hundred molecules of the high Tanimoto similarity [53] scores (>0.6) to the known SRC kinase inhibitors.

In the post-processing analysis, we examined whether novel SRC-like molecules could be generated by the chemical transformation process initiated from the inhibitors of different kinase families. According to this analysis, the perturbation-based molecular generation process that started from the inhibitors of LCK and EGFR kinases yielded a higher percentage of molecules (~19–23%) as compared to the MAPK14 and FLT3 families, which produced only 5–7% of novel valid molecules (Figure 7A). When only unique and novel generated molecules were considered, it appeared that starting from the LCK and MAPK10 kinase inhibitors, the developed model could produce ~20–25% of the total molecular output (Figure 7B). Due to the presence of highly dense and low populated regions in the latent space, clusters that contained high concentrations of the initial data points eventually resulted in a higher molecular output. When we considered generated molecules that yielded a high kinase inhibition likelihood value (>0.75), the perturbation model favored the LCK and MAPK10 inhibitors as starting data points for successful chemical transformation to the SRC-like molecules, yielding > 25% of the molecules with a high kinase inhibition likelihood (Figure 7C).

To evaluate similarity between the generated molecules and known SRC kinase inhibitors, we also examined the distribution of the generated molecules with the high Tanimoto similarity coefficient [53]. Our analysis revealed that the generated molecules originating from LCK inhibitors formed the largest fraction of novel kinase-like compounds (~40%) with a high similarity to the SRC kinase inhibitors (Figure 7D). The generated molecules initiated from the inhibitors of ABL1, MAPK10, MET, and EGFR kinases also formed a significant fraction of the generated valid molecules with high similarity to the known SRC inhibitors (Figure 7D). The SRC/ABL and SRC/LCK duality of many kinase drugs is well recognized, most notably exemplified by dual SRC/ABL drugs dasatinib and ponatinib. Strikingly, our results reflected these functional observations by revealing the interconnectivity of the latent spaces for these classes of kinase molecules. According to our findings, the perturbation-based exploration of the latent space that optimized sampling along interpretable directions guided by the kinase inhibition likelihood score could facilitate the generation of novel valid molecules in different areas of the latent space. 

To summarize, a combination of the cluster decomposition and perturbation-based exploration of the latent space allowed for the generation of novel molecules with a high similarity to the experimentally known SRC kinase inhibitors via chemical transformation of the kinase inhibitors from different families.

### 2.6. Drug-like Properties’ Assessment of the Generated Molecules

To evaluate the properties of the generated molecules with a high similarity to the known SRC inhibitors, we calculated three different drug-like metrics: QED, logP, and SAS values for the produced molecules, with a specific focus on novel compounds originating from the inhibitors of ABL1, LCK, and EGFR kinases (Table 1). For comparison, we also computed these parameters for the known SRC inhibitors. The analysis showed that the average values of the drug-like parameters for the existing SRC inhibitors and the generated molecules were similar. Hence, the perturbation-based generation approach yielded novel valid compounds with a high kinase inhibition likelihood score, high similarity to known SRC inhibitors, and drug-like properties that were consistent with the corresponding values seen for the existing SRC inhibitors (Table 1).

In addition, we conducted an analysis of the main physicochemical properties of the generated molecules with a high kinase inhibition likelihood (Table S8). The distribution of physicochemical descriptors related to the druglikeness of compounds for targeting protein kinases was recently investigated [54]. According to this study, the number of hydrogen bond acceptors (HBA) in the kinase molecules is within a range of 3 to 10, while the number of hydrogen bond donors (HBD) is in the range of 0 to 4 [54]. The average HBA and HBD numbers for the generated molecules (Table S8) were within the acceptable range for existing kinase inhibitors meeting the guidelines used for the prioritization of kinase inhibitors in the drug-like analysis [54]. Similarly, the generated molecules using our approach also satisfied other criteria for the kinase inhibitors, including the acceptable molecular weight, and the number of aromatic rings and rotatable bonds (Table S8). Hence, the pattern and range of major chemical and drug-like parameters for the generated molecules were consistent with the values featured by the known kinase inhibitors. 

**Figure 8:**
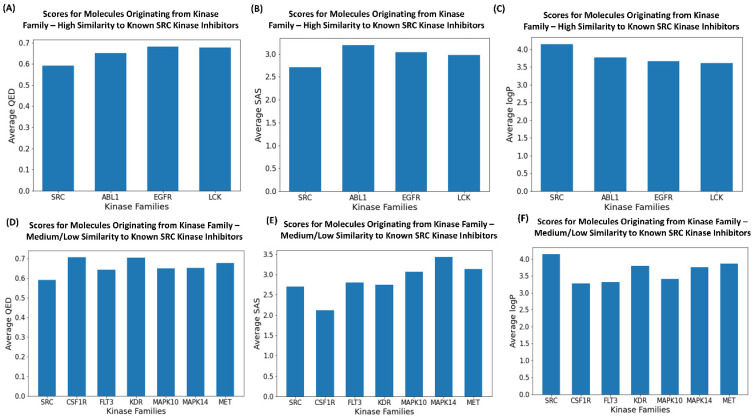
The analysis of drug-like properties for the generated molecules. (**A**) The average QED values for generated molecules originated from inhibitors of the 3 kinase families and comparison with the QED values for SRC kinase inhibitors. The generated molecules produced the highest similarity scores to the known SRC kinase inhibitors. (**B**) The average SAS values for generated molecules originating from inhibitors of the 3 kinase families and comparison with the SAS values for the SRC kinase inhibitors. The generated molecules produced the highest similarity scores to the known SRC kinase inhibitors. (**C**) The average logP values for generated molecules originating from inhibitors of the 3 kinase families and comparison with the logP values for SRC kinase inhibitors. The generated molecules produced the highest similarity scores to the known SRC kinase inhibitors. (**D**) The average QED values for generated molecules originating from inhibitors of the 3 kinase families and comparison with the QED values for SRC kinase inhibitors. The generated molecules produced medium/low similarity scores to the known SRC kinase inhibitors. (**E**) The average SAS values for generated molecules originating from inhibitors of the 3 kinase families and comparison with the SAS values for SRC kinase inhibitors. The generated molecules produced medium/low similarity scores to the known SRC kinase inhibitors. (**F**) The average logP values for generated molecules originating from inhibitors of the 3 kinase families and comparison with the logP values for SRC kinase inhibitors. The generated molecules produced medium/low similarity scores to the known SRC kinase inhibitors.

We also performed a comparative analysis of the drug-like properties averaged over generated molecules including QED, SAS, and LogP for classes of produced molecules that were characterized by a high similarity to the known SRC kinase inhibitors (Figure 8A–C) as well as by a medium similarity to the SRC inhibitors (Figure 8D–F). Interestingly, we observed a consistent range of acceptable drug-like properties for the generated molecules regardless of their similarity to the existing SRC kinase inhibitors. The QED, SAS and LogP parameters for the generated SRC-like molecules were similar to the ones attained by the existing SRC inhibitors. These observations provided further support to the notion that the proposed combination of clustering and perturbation-based exploration of the latent space could allow for robust generation of valid and diverse kinase molecules with drug-like properties.

A more complete description of the generated molecules is presented in the Supplementary Materials Information where the following data can be found: (a) the generated molecules with similarity score, kinase inhibition likelihood score and drug metrics from the initial experiment of perturbation modeling with only SRC inhibition data; (b) the generated molecules with similarity score, kinase inhibition likelihood score, and drug metrics from the molecule transformation perturbation experiments of the kinase inhibitors from different families into SRC kinase-like inhibitors; and (c) the generated molecules from the initial experiments of modifying known SRC kinase inhibitors to design novel SRC molecules. 

All scripts, software, and models used in the development and experiments are available in the GitHub site https://github.com/kassabry/Perturbation_Experiment (accessed on 27 August 2022). The GitHub site provides detailed documentation and a guide of the deposited information and software. The deep learning frameworks were supported by the TensorFlow backend and python tools such as NumPy, SciPy, pandas, and scikitlearn (Supplementary Materials Information).

## 3. Materials and Methods 

### 3.1. Datasets of Protein Kinase Inhibitors and Small Molecules

For the training datasets of small molecules, we employed a fraction of the GDB-17 database version (GDB database release, 9 August 2021, https://zenodo.org/record/7041051#.YypEcbTMI2w (accessed on 6 October 2021)) corresponding to small molecules from a variety of domains, with the following atoms (C, N, O, S, F, Cl, Br, P, I) [50,51]. For the datasets of kinase chemical probes we used (a) ~20,000 known kinase inhibitors representing 10 major kinase families SRC, ABL1, CSF1R, EGFR, FLT3, KDR, LCK, MAPK10, MAPK14, and MET that were obtained from ZINC15 database [48] and (b) a dataset of competitive and allosteric protein kinase inhibitors confirmed by X-ray crystallography that contained a total of 2899 unique inhibitors including 136 allosteric and 2763 orthosteric compounds with a total of 231 protein kinases [49]. The kinase inhibitors set included 1883 unique ABL1 kinase inhibitors and 3477 unique SRC kinase inhibitors. By encoding a set of GDB-17 molecules and a set of kinase inhibitors using ChemVAE framework data, every molecule was transformed into a 196-dimensional vectorial representation in the continuous space. The 196-dimensional vectors representing these molecules were then fed through principal component analysis (PCA) so that we could visualize the continuous space in two dimensions and analyze different regions (Figure 1).

### 3.2. Clustering and Perturbation-Based Exploration of the Latent Space and Variational Autoencoder

The perturbation approach was implemented by using a combination of K-means clustering [55] and perturbation-based exploration of the continuous latent space. Different cluster-based splits of the latent space were assessed (2-cluster-based split, 3-cluster-based split, 4-cluster-based split, and 5-cluster-based split) to determine which cluster split gave the best yield of valid molecules during generation process. Three-cluster-based split of the latent space yielded the best generational output and was subsequently used in the perturbation experiments and generation of novel kinase molecules. After implementing K-means clustering in the latent space, we performed a cluster-based perturbation to each data point in a cluster using an averaging mechanism. We used the cluster centroid as an anchoring point for the initial perturbation and applied perturbation to the respective vector representation of the chemical features and attributes representing the data of the respective cluster. Utilizing the centroid values invoked targeted alteration to the data within each cluster, which allowed for generation of new molecules that retained similar chemical and structural properties to the encoded kinase inhibitors. If each cluster represents a chemical or structural motif, motif-specific perturbations can be made rather than a “one fits all” approach. Assuming that the centroid is $\vec{c_{i}}$ and one of the datapoints in the cluster is $\vec{x_{i}}$ we take the difference between $\vec{x_{i}}$ and $\vec{c_{i}}$, and multiply that vector by a scaling factor s, where 0≤s≤1, giving us our new perturbed vector $\vec{x_{i}^{*}}$. The targeted perturbation step can be formulated as

(1) $$\vec{x_{i}^{*}}=\vec{x_{i}}+s\left(\vec{c_{i}}-\vec{x_{i}}\right)$$

The scaling factor s acts as a parameter to tune the level of perturbation we want to achieve. Given that the lower bound of s=0 corresponds to the original encoding of a given molecule, while s=1 provides us with the centroid of the cluster, this parameter is initially set to be a threshold of 0.5. By performing perturbation steps and evaluating kinase inhibition likelihood probabilities, we find that with the scaling factor s<0.5 the yield of valid molecules decreases, while a scaling factor s=0.8 can perturb the initial molecule gradually towards the centroid of the cluster yielding valid molecules without losing information of the molecular attributes. The scalar values s=0.75−0.8 allow for more efficient sampling of the high density areas and valid decoding, producing novel compounds with the higher success of transformation into the class of potential SRC kinase inhibitors.

To observe how noise would affect the generation yield of novel compounds, we evaluated six distinct levels of noise, from level 5 to 30 in increments of five. We observed that a noise level of 5 contributed to a greater generation yield of valid molecules as compared to noise levels of 10 and above. At a noise level of 10, there was less generation yield than at a lower noise value. When using noise level 15 and above, the generation yield was low. Due to the extensive alteration in the continuous latent space, adding random noise to already perturbed data points could cause additional changes in the chemical and molecular features of the encoded compounds, which results in low density areas of decoding potential and reduced yield of valid novel molecules. After evaluating and optimizing every parameter that is involved in the targeted perturbation protocol, we found that a 3-cluster-based split, a scaling factor of 0.8 for the centroid-based perturbation, and a noise level of 5.0 were the optimal set of parameters to guarantee a sufficiently high generation yield of valid and novel molecules.

After perturbation step was executed, the perturbed vector was decoded using the chemical VAE network. We imposed validity constraints on the created molecules by filtering out any latent locations that had a 0% decode validity. The probabilistic chemical VAE neural network attempted to decode the latent variable into a valid SMILES string 500 times, each time feeding the molecule into RDKit [52] computations to assess validity. If the network could yield at least 100 valid molecules out of the 500 decode attempts, we moved the molecule into the next validity stage. The next validity gate checked the length of the SMILES string to ensure that no molecule made it through if it had a length less than 10. The generated molecular output was then run through a post-processing analysis, where it was evaluated on various metrics to determine validity and success of transformation.

All scripts, software, and models used in the development and experiments are available in the GitHub site https://github.com/kassabry/Perturbation_Experiment (accessed on 27 August 2022). The GitHub repository contains the framework and code for molecular transformations of kinase inhibitors using generative learning methodologies and targeted perturbation modeling. The GitHub site provides detailed documentation and guide of the deposited information and software. The deep learning frameworks are supported by the TensorFlow backend [56] and python tools such as NumPy, SciPy, pandas, and scikitlearn. The developed software used to conduct PCA analysis and visualization of the latent space is contained in the perturbation experiment python notebook. The developed software for the use of the variational autoencoder integrated with the perturbation approach is contained in the perturbation experiment python notebook. More information on these files and how to perform the initial setup of the environment can be found in the GitHub README file.

### 3.3. Kinase Inhibition Likelihood Classifiers

The Random Forest classification method [57] is used to construct multiclass and binary kinase inhibition likelihood classifiers in the latent and chemical spaces of small molecules. The model is initiated with the training set of molecules from all kinase families as well as GDB-17 molecules. Each molecule within the training set is processed through RDKit [52] to calculate chemical features. Binary decision trees are created, and the chemical attributes are used as parameters to determine the most key features in determining the target variable. Each decision tree makes a prediction on the value of the target variable and the predictions are then aggregated and averaged to obtain a value between 0 and 1. If there are more than two classes, the predictions are normalized and then averaged to maintain a predicted value between 0 and 1. This would ensure that the target value would still be between 0 and 1 while allowing for multiple classification variables.

For chemical feature-based classifier, 20 chemical features are considered for each molecule during training and testing: the number of rings, the exact molecular weight, the number of rotatable bonds, the fraction of carbon Sp3 atoms, the Hall–Kier alpha value, the Labute ASA value, the number of aliphatic carbocycles, the number of aliphatic heterocycles, the number of aliphatic rings, the number of amide bonds, the number of aromatic carbocycles, the number of aromatic heterocycles, the number of aromatic rings, the number of stereocenters, the number of bridgehead atoms, the number of H-bond acceptors, the number of H-bond donors, the QED value, the SAS value, and the logP value. The resulting score in the Random Forest model’s output represents the probability or likelihood that a molecule can be deemed a kinase inhibitor. Values closer to 0 indicate that the molecule has a low kinase inhibition likelihood, whereas values closer to 1 indicate that the molecules have a high kinase inhibition likelihood.

To assess the performance of each model, accuracy, recall, precision and F1 scores were calculated to measure the performance of classification models. These parameters are defined as follows

(2) $$\mathrm{Accuracy}=\frac{\mathrm{TP}+\mathrm{TN}}{\mathrm{all}};\;\mathrm{Precision}=\frac{\mathrm{TP}}{\mathrm{TP}+\mathrm{FP}}\;$$

(3) $$\mathrm{Recall}=\frac{\mathrm{TP}}{\mathrm{TP}+\mathrm{FN}};{\;F}_{1}=2\frac{\mathrm{Precision}*\mathrm{Recall}}{\mathrm{Precision}+\mathrm{Recall}}$$

An F-score is a measure of precision and recall and is often used in binary classification problems. Precision is defined as the amount of positive samples the model predicts correctly (true positives) divided by the true positives plus the false positives. Recall is defined as true positives divided by true positives plus false negatives. The model performance is evaluated using the receiver operating characteristic area under the curve. The receiver operating curve (ROC) is a graph where sensitivity is plotted as a function of 1-specificity. The area under the ROC is denoted AUC. A reliable and valid AUC estimate can be interpreted as the probability that the classifier will assign a higher score to a randomly chosen positive example than to a randomly chosen negative example.

## 4. Conclusions

In this work, we proposed an integrated ML approach for controllable molecule manipulation and an efficient perturbation strategy to improve the steerability and interpretability of the generative models. The results of this study showed that the proposed method allowed for an efficient exploration of the latent space along interpretable directions guiding the generation of novel SRC family-specific kinase molecules featuring scaffold diversity and optimized drug-like biochemical properties. A robust chemical feature-based machine learning predictor of the kinase inhibition likelihood was developed to aid in the perturbation-based transformation of small molecules. By combining molecular perturbation design with the kinase inhibition likelihood analysis and similarity assessments, we demonstrated that the proposed strategy allowed for morphing of the kinase inhibitors into novel chemical probes of the SRC kinase that exhibit desirable ranges for all included chemical properties and display a high similarity to the known potent SRC kinase inhibitors. The central finding of this study revealed that the integration of cluster partitioning and perturbation-based exploration of the latent space allows for the generation of novel kinase molecules with a high similarity to the experimentally known SRC kinase inhibitors via chemical transformation of the kinase inhibitors from different families. Furthermore, the generated molecular output originating from LCK and ABL1 kinase inhibitors yielded ~40% of novel and valid kinase compounds with kinase inhibition likelihood probability values (*p* > 0.75) and high similarity values (Tanimoto coefficient > 0.6).

Although the proposed perturbation-based approach was robust and showed promise in the generation of novel valid molecules and the “interpretable transformation” of kinase molecules towards family-specific kinase probes, there is a significant room for improvement in this approach and related models. Our analysis suggested that one of the important lines for the improvement of the interpretable and target-oriented molecular design approaches would be retraining and adapting the variational autoencoder to use the wealth and diversity of the existing kinase inhibitors with different levels of activity, binding affinity, and selectivity. Since the ChemVAE model is not specifically trained on kinase molecules, the retraining of the deep neural network using the vast space of kinase inhibitors could potentially lead to a more accurate and granular latent space representation of the kinase space, thus enabling a more accurate and robust navigation of the kinase latent landscapes.

An important problem associated with generative learning is the “black box” nature of neural networks, which often hinders the interpretability of predictions preventing us from explicitly discerning what specific property or properties are primarily harnessed to generate output. Despite the ability to generate molecules with desired properties, it is often difficult to understand the generation process and the chemical rules that govern the generation process with previous methods. Through assessment of the latent-based and chemical feature-based binary and multiclass classifiers, we developed a robust probabilistic evaluator of kinase inhibition likelihood that is specifically tailored to predict novel SRC kinase inhibitors. The approach leveraged the learned latent space landscapes to achieve controllable generation by accepting/rejecting sampled molecules based on a robust feature-engineered classifier. 

Another potential avenue for future improvement would also include the incorporation of three-dimensional features and diverse experimental data on kinase molecules into VAE models by adding an additional embedding scheme so that the topological and biological information of molecules can also be embedded and contribute to clustering and the subsequent perturbation navigation stage of the design process. While exploring the chemical space with unbiased pretrained autoencoder generative models becomes a dominant theme in many applications of deep molecular design models, the results of our study suggested that task-specific manipulation of a biased latent space may be an important future direction for more effective task-oriented and target-specific autonomous chemical design models.

## Figures and Tables

**Table 1:** Drug-Like properties of the generated molecules originating from kinase inhibitors of different kinase families and exhibiting high similarity with the SRC kinase inhibitors.

Drug-Like Metrics	SRC Inhibitors	ABL1 Inhibitors	LCK Inhibitors	EGFR Inhibitors
Average QED Scores	0.605	0.6742	0.6952	0.6752
Average SAS Score	2.6829	2.9806	2.012	2.8175
Average logP Scores	3.869	3.8989	3.803	3.7687

## Data Availability

Data is fully contained within the article and Supplementary Materials. The data presented in this study are available in the article and Supplementary Materials.

## Supplementary Materials

The supporting information can be downloaded at: https://www.mdpi.com/article/10.3390/ijms231911262/s1.
