# Supplementary material for: Interpretable Machine Learning Models for Molecular Design of Tyrosine Kinase Inhibitors Using Variational Autoencoders and Perturbation-Based Approach of Chemical Space Exploration
Source: Int J Mol Sci. 2022 Sep 24;23(19):11262. doi: 10.3390/ijms231911262 (PMC9569663; doi:10.3390/ijms231911262)
Supplement: Supplementary file 1 [file ijms-23-11262-s001.zip › SUPPLEMENTARY_MATERIALS/Generated_Molecules_With_Similarity/final_similarity_data.pdf]

	src known mol	similarity	mol	smiles	qed	logP	SAS	rings	kinase inhibition likeliness
**0**	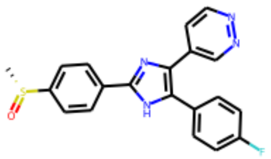	[1.0]	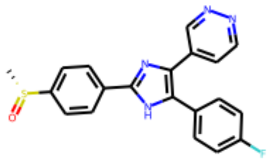	C[S@@](=O)c1ccc(-c2nc(-c3ccnnc3)c(-c3ccc(F)cc3)[nH]2)cc1	0.709527	2.560076	2.763322	4	0.805595
**1**	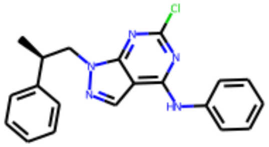	[0.9631067961165048]	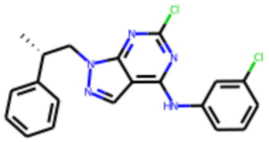	Clc1nc(Nc2cccc(Cl)c2)c2cnn(C[C@@H](C)c3ccccc3)c2n1	0.812670	2.803140	2.415127	4	0.800810
**2**	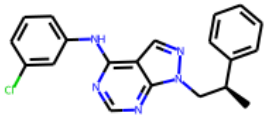	[0.9525316455696202]	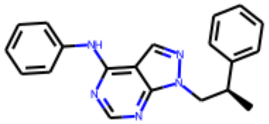	C[C@@H](Cn1ncc2c(Nc3ccccc3)ncnc21)c1ccccc1	0.582066	3.453236	4.511770	4	0.858060
**3**	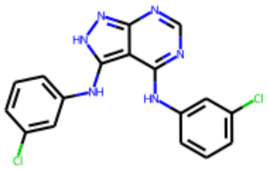	[0.9335142469470827]	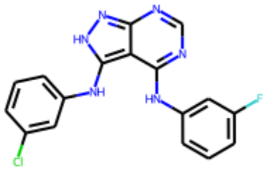	Fc1cccc(Nc2ncnc3n[nH]c(Nc4cccc(Cl)c4)c23)c1	0.648843	2.528773	3.556390	4	0.786036

	src known mol	similarity	mol	smiles	qed	logP	SAS	rings	kinase inhibition likeliness
**4**	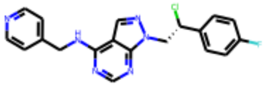	[0.9122098890010091]	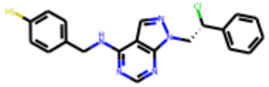	Sc1ccc(CNc2ncnc3c2cnn3C[C@H](Cl)c2ccccc2)cc1	0.773249	2.760402	2.523284	4	0.788643
**5**	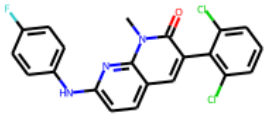	[0.9105760963026656]	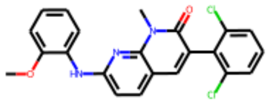	COc1ccccc1Nc1ccc2cc(-c3c(Cl)cccc3Cl)c(=O)n(C)c2n1	0.773249	2.760402	2.523284	4	0.821857
**6**	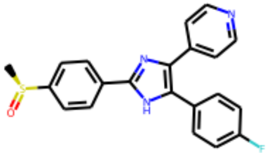	[0.9077901430842608]	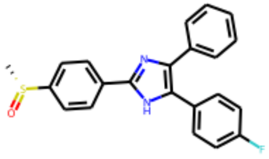	C[S@@](=O)c1ccc(-c2nc(-c3ccccc3)c(-c3ccc(F)cc3)[nH]2)cc1	0.693247	2.498708	3.629723	4	0.800488
**7**	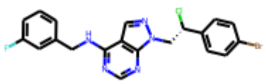	[0.9036742800397219]	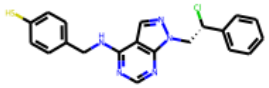	Sc1ccc(CNc2ncnc3c2cnn3C[C@H](Cl)c2ccccc2)cc1	0.773249	2.760402	2.523284	4	0.788643

	src known mol	similarity	mol	smiles	qed	logP	SAS	rings	kinase inhibition likeliness
**8**	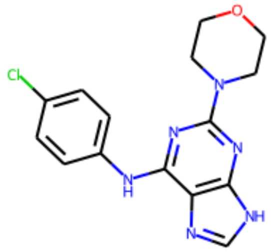	[0.8751418842224744]	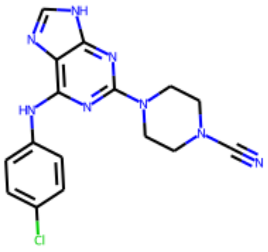	N#CN1CCN(c2nc(Nc3ccc(Cl)cc3)c3nc[nH]c3n2)CC1	0.582066	3.453236	4.511770	4	0.832190
**9**	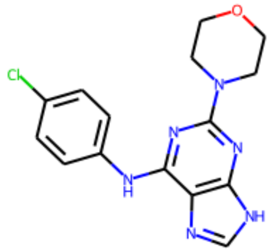	[0.8751418842224744]	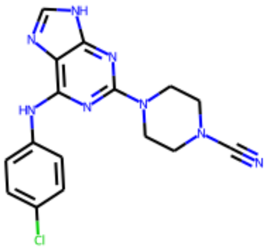	N#CN1CCN(c2nc(Nc3ccc(Cl)cc3)c3nc[nH]c3n2)CC1	0.582066	3.453236	4.511770	4	0.832190
**10**	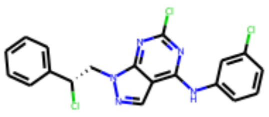	[0.8729729729729729]	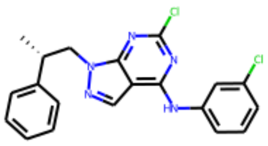	Clc1nc(Nc2cccc(Cl)c2)c2cnn(C[C@@H](C)c3ccccc3)c2n1	0.812670	2.803140	2.415127	4	0.800810
**11**	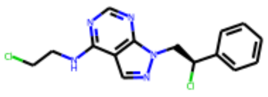	[0.8715313463514902]	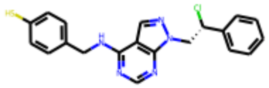	Sc1ccc(CNc2ncnc3c2cnn3C[C@H](Cl)c2ccccc2)cc1	0.773249	2.760402	2.523284	4	0.788643

	src known mol	similarity	mol	smiles	qed	logP	SAS	rings	kinase inhibition likeliness
**12**	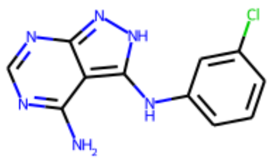	[0.864314789687924]	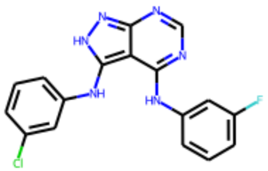	Fc1cccc(Nc2ncnc3n[nH]c(Nc4cccc(Cl)c4)c23)c1	0.648843	2.528773	3.556390	4	0.786036
**13**	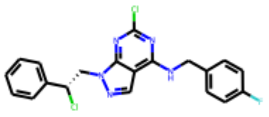	[0.8641975308641975]	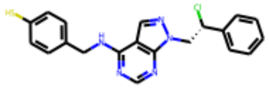	Sc1ccc(CNc2ncnc3c2cnn3C[C@H](Cl)c2ccccc2)cc1	0.773249	2.760402	2.523284	4	0.788643
**14**	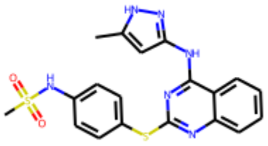	[0.8505747126436781]	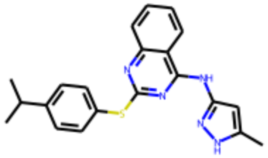	C[C@@H](C)c1ccc(Sc2nc(Nc3cc(C)[nH]n3)c3ccccc3n2)cc1	0.789802	3.119093	1.773532	4	0.800611
**15**	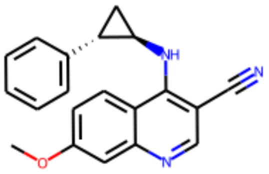	[0.8269230769230769]	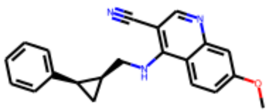	COc1ccc2c(NC[C@H]3C[C@H]3c3ccccc3)c(C#N)cnc2c1	0.709527	2.560076	2.763322	4	0.790969

	src known mol	similarity	mol	smiles	qed	logP	SAS	rings	kinase inhibition likeliness
16	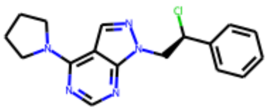	[0.8228460793804453]	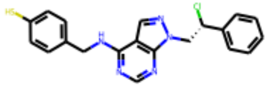	Sc1ccc(CNc2ncnc3c2cnn3C[C@H](Cl)c2ccccc2)cc1	0.773249	2.760402	2.523284	4	0.788643
17	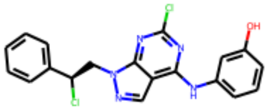	[0.8197725284339458]	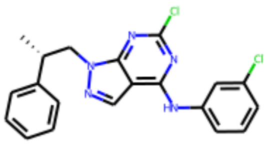	Clc1nc(Nc2cccc(Cl)c2)c2cnn(C[C@@H](C)c3ccccc3)c2n1	0.812670	2.803140	2.415127	4	0.800810
18	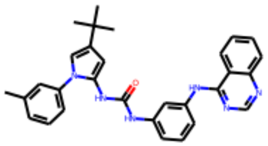	[0.812254516889238]	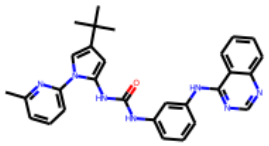	CC(C)(C)c1cc(NC(=O)Nc2cccc(Nc3ncnc4ccccc34)c2)n(-c2cccc(C)n2)c1	0.511693	2.495788	5.669147	5	0.771803
19	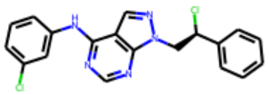	[0.8091743119266055]	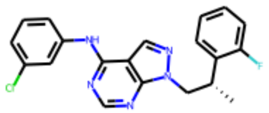	Clc1cccc(Nc2ncnc3c2cnn3C[C@@H](C)c2ccccc2F)c1	0.812670	2.803140	2.415127	4	0.793690

	src known mol	similarity	mol	smiles	qed	logP	SAS	rings	kinase inhibition likeliness
**20**	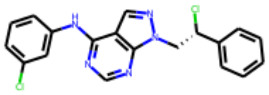	[0.8091743119266055]	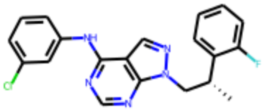	Clc1cccc(Nc2ncnc3c2cnn3C[C@@H](C)c2ccccc2F)c1	0.812670	2.803140	2.415127	4	0.793690
**21**	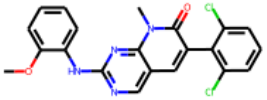	[0.8064257028112449]	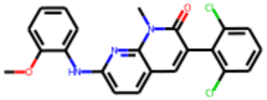	COc1ccccc1Nc1ccc2cc(-c3c(Cl)cccc3Cl)c(=O)n(C)c2n1	0.773249	2.760402	2.523284	4	0.821857
**22**	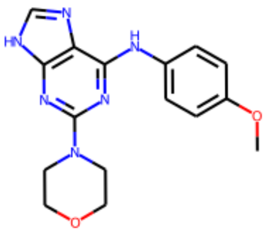	[0.8047464940668824]	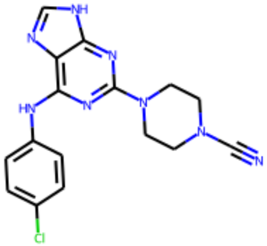	N#CN1CCN(c2nc(Nc3ccc(Cl)cc3)c3nc[nH]c3n2)CC1	0.582066	3.453236	4.511770	4	0.832190
**23**	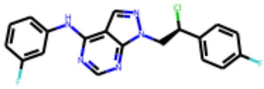	[0.8011527377521613]	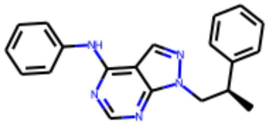	C[C@@H](Cn1ncc2c(Nc3ccccc3)ncnc21)c1ccccc1	0.582066	3.453236	4.511770	4	0.858060

	src known mol	similarity	mol	smiles	qed	logP	SAS	rings	kinase inhibition likeliness
**24**	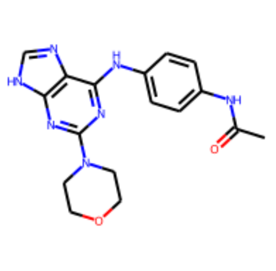	[0.7972251867662753]	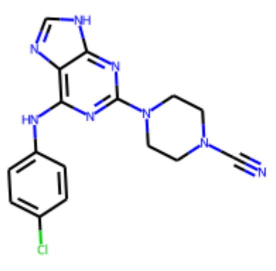	N#CN1CCN(c2nc(Nc3ccc(Cl)cc3)c3nc[nH]c3n2)CC1	0.582066	3.453236	4.511770	4	0.832190
**25**	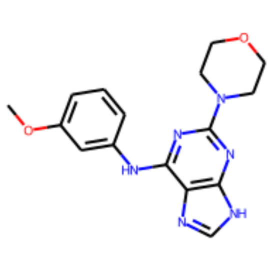	[0.7824267782426778]	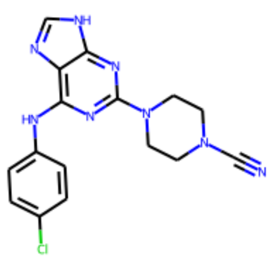	N#CN1CCN(c2nc(Nc3ccc(Cl)cc3)c3nc[nH]c3n2)CC1	0.582066	3.453236	4.511770	4	0.832190
**26**	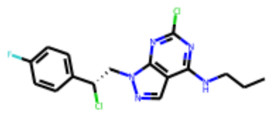	[0.779707495429616]	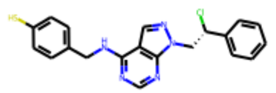	Sc1ccc(CNc2ncnc3c2cnn3C[C@H](Cl)c2ccccc2)cc1	0.773249	2.760402	2.523284	4	0.788643
**27**	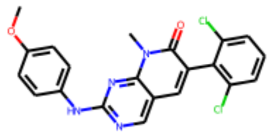	[0.7676282051282052]	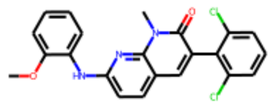	COc1ccccc1Nc1ccc2cc(-c3c(Cl)cccc3Cl)c(=O)n(C)c2n1	0.773249	2.760402	2.523284	4	0.821857

	src known mol	similarity	mol	smiles	qed	logP	SAS	rings	kinase inhibition likeliness
**28**	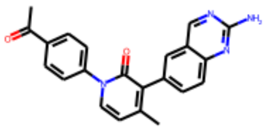	[0.7672493100275989]	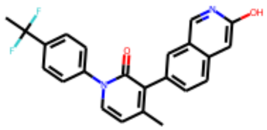	Cc1ccn(-c2ccc(C(C)(F)F)cc2)c(=O)c1-c1ccc2cc(O)ncc2c1	0.773249	2.760402	2.523284	4	0.781500
**29**	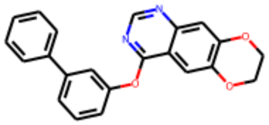	[0.764642082429501]	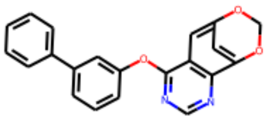	c1ccc(-c2cccc(Oc3ncnc4c3cc(cc34)OCO3)c2)cc1	0.582066	3.453236	4.511770	5	0.847750
**30**	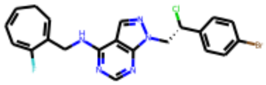	[0.7606382978723404]	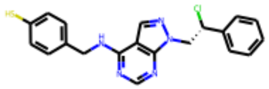	Sc1ccc(CNc2ncnc3c2cnn3C[C@H](Cl)c2ccccc2)cc1	0.773249	2.760402	2.523284	4	0.788643
**31**	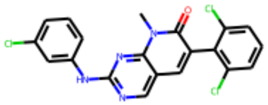	[0.7529880478087649]	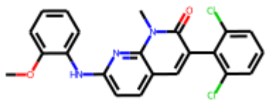	COc1ccccc1Nc1ccc2cc(-c3c(Cl)cccc3Cl)c(=O)n(C)c2n1	0.773249	2.760402	2.523284	4	0.821857

	src known mol	similarity	mol	smiles	qed	logP	SAS	rings	kinase inhibition likeliness
**32**	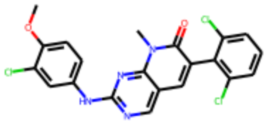	[0.750388802488336]	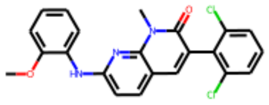	COc1ccccc1Nc1ccc2cc(-c3c(Cl)cccc3Cl)c(=O)n(C)c2n1	0.773249	2.760402	2.523284	4	0.821857
**33**	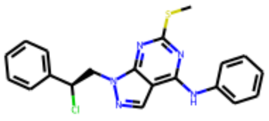	[0.7491007194244604]	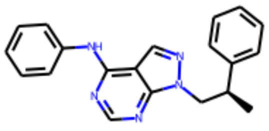	C[C@@H](Cn1ncc2c(Nc3ccccc3)ncnc21)c1ccccc1	0.582066	3.453236	4.511770	4	0.858060
**34**	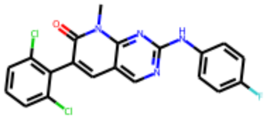	[0.7468253968253968]	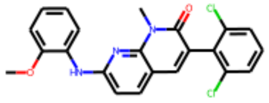	COc1ccccc1Nc1ccc2cc(-c3c(Cl)cccc3Cl)c(=O)n(C)c2n1	0.773249	2.760402	2.523284	4	0.821857
**35**	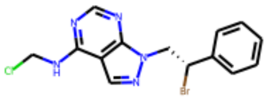	[0.7463414634146341]	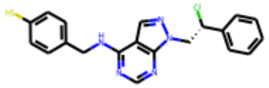	Sc1ccc(CNc2ncnc3c2cnn3C[C@H](Cl)c2ccccc2)cc1	0.773249	2.760402	2.523284	4	0.788643

	src known mol	similarity	mol	smiles	qed	logP	SAS	rings	kinase inhibition likeliness
**36**	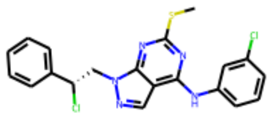	[0.742714404662781]	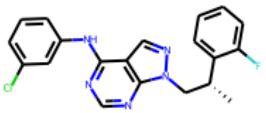	Clc1cccc(Nc2ncnc3c2cnn3C[C@@H](C)c2ccccc2F)c1	0.812670	2.803140	2.415127	4	0.793690
**37**	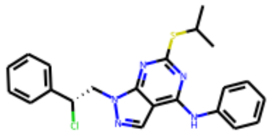	[0.7383259911894273]	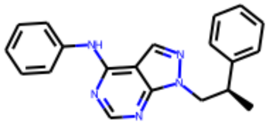	C[C@@H](Cn1ncc2c(Nc3ccccc3)ncnc21)c1ccccc1	0.582066	3.453236	4.511770	4	0.858060
**38**	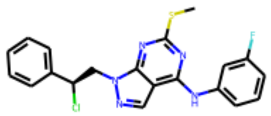	[0.7318777292576419]	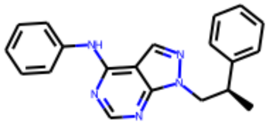	C[C@@H](Cn1ncc2c(Nc3ccccc3)ncnc21)c1ccccc1	0.582066	3.453236	4.511770	4	0.858060
**39**	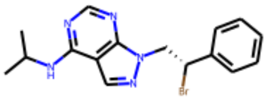	[0.7289272030651341]	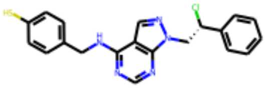	Sc1ccc(CNc2ncnc3c2cnn3C[C@H](Cl)c2ccccc2)cc1	0.773249	2.760402	2.523284	4	0.788643

	src known mol	similarity	mol	smiles	qed	logP	SAS	rings	kinase inhibition likeliness
**40**	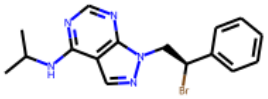	[0.7289272030651341]	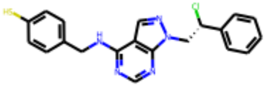	Sc1ccc(CNc2ncnc3c2cnn3C[C@H](Cl)c2ccccc2)cc1	0.773249	2.760402	2.523284	4	0.788643
**41**	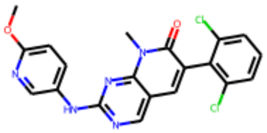	[0.7248475609756098]	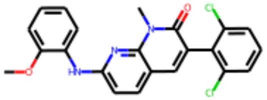	COc1ccccc1Nc1ccc2cc(-c3c(Cl)cccc3Cl)c(=O)n(C)c2n1	0.773249	2.760402	2.523284	4	0.821857
**42**	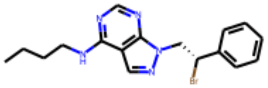	[0.7243346007604563]	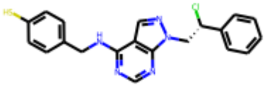	Sc1ccc(CNc2ncnc3c2cnn3C[C@H](Cl)c2ccccc2)cc1	0.773249	2.760402	2.523284	4	0.788643
**43**	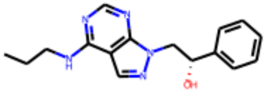	[0.7217235188509874]	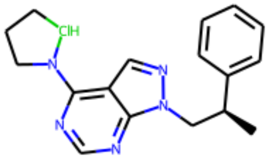	C[C@@H](Cn1ncc2c(N3CCCCl3)ncnc21)c1ccccc1	0.618172	2.645624	3.611880	4	0.778794

	src known mol	similarity	mol	smiles	qed	logP	SAS	rings	kinase inhibition likeliness
**44**	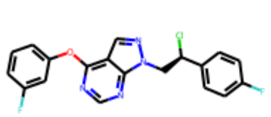	[0.7177280550774526]	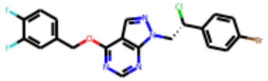	Fc1ccc(COc2ncnc3c2cnn3C[C@H](Cl)c2ccc(Br)cc2)cc1F	0.709527	2.560076	2.763322	4	0.846183
**45**	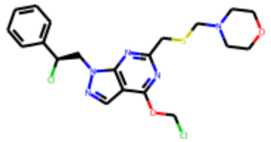	[0.7112387202625102]	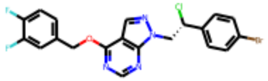	Fc1ccc(COc2ncnc3c2cnn3C[C@H](Cl)c2ccc(Br)cc2)cc1F	0.709527	2.560076	2.763322	4	0.846183
**46**	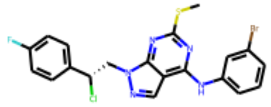	[0.7097872340425532]	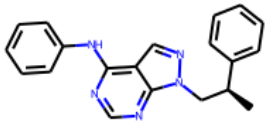	C[C@@H](Cn1ncc2c(Nc3ccccc3)ncnc21)c1ccccc1	0.582066	3.453236	4.511770	4	0.858060
**47**	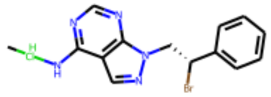	[0.7088607594936709]	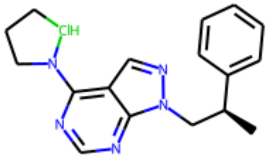	C[C@@H](Cn1ncc2c(N3CCCCl3)ncnc21)c1ccccc1	0.618172	2.645624	3.611880	4	0.778794

	src known mol	similarity	mol	smiles	qed	logP	SAS	rings	kinase inhibition likeliness
**48**	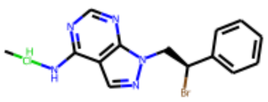	[0.7088607594936709]	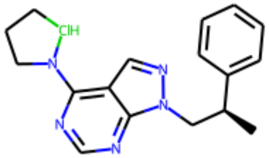	C[C@@H](Cn1ncc2c(N3CCCCl3)ncnc21)c1ccccc1	0.618172	2.645624	3.611880	4	0.778794
**49**	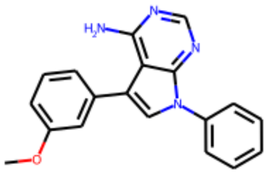	[0.7066436583261432]	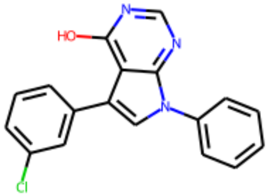	Clc1cccc(-c2cn(-c3ccccc3)c3ncnc(O)c23)c1	0.789759	3.120557	2.191082	4	0.824512
**50**	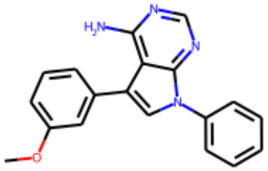	[0.7066436583261432]	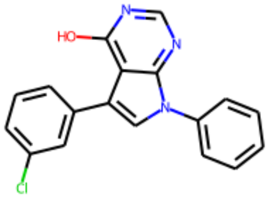	Clc1cccc(-c2cn(-c3ccccc3)c3ncnc(O)c23)c1	0.789759	3.120557	2.191082	4	0.824512
**51**	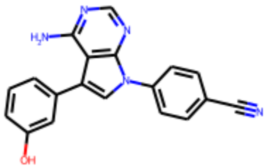	[0.7035775127768313]	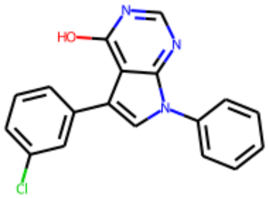	Clc1cccc(-c2cn(-c3ccccc3)c3ncnc(O)c23)c1	0.789759	3.120557	2.191082	4	0.824512

	src known mol	similarity	mol	smiles	qed	logP	SAS	rings	kinase inhibition likeliness
**52**	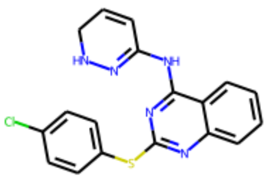	[0.687]	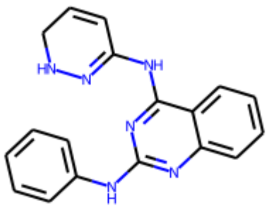	C1ccc(Nc2nc(Nc3ccccc3)nc3ccccc23)n[nH]1	0.618172	2.645624	3.611880	4	0.818496
**53**	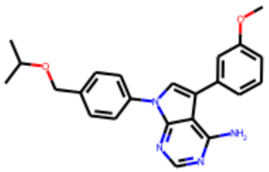	[0.6836065573770492]	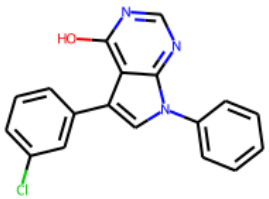	Clc1cccc(-c2cn(-c3ccccc3)c3ncnc(O)c23)c1	0.789759	3.120557	2.191082	4	0.824512
**54**	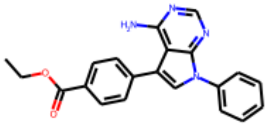	[0.6825795644891123]	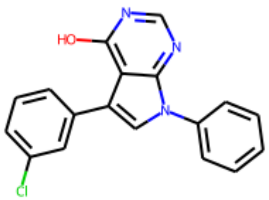	Clc1cccc(-c2cn(-c3ccccc3)c3ncnc(O)c23)c1	0.789759	3.120557	2.191082	4	0.824512
**55**	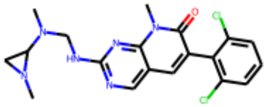	[0.6795162509448224]	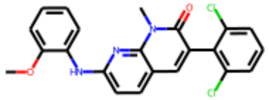	COc1ccccc1Nc1ccc2cc(-c3c(Cl)cccc3Cl)c(=O)n(C)c2n1	0.773249	2.760402	2.523284	4	0.821857

	src known mol	similarity	mol	smiles	qed	logP	SAS	rings	kinase inhibition likeliness
**56**	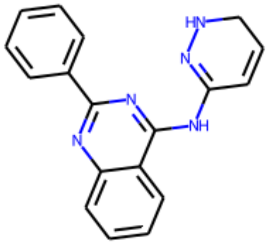	[0.6730575176589304]	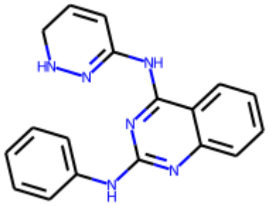	C1ccc(Nc2nc(Nc3ccccc3)nc3ccccc23)n[nH]1	0.618172	2.645624	3.611880	4	0.818496
**57**	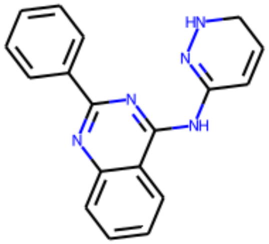	[0.6730575176589304]	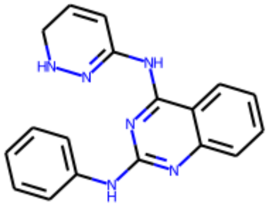	C1ccc(Nc2nc(Nc3ccccc3)nc3ccccc23)n[nH]1	0.618172	2.645624	3.611880	4	0.818496
**58**	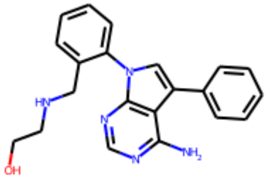	[0.6618819776714514]	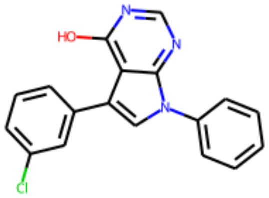	Clc1cccc(-c2cn(-c3ccccc3)c3ncnc(O)c23)c1	0.789759	3.120557	2.191082	4	0.824512
**59**	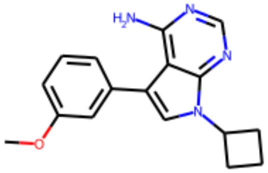	[0.6614420062695925]	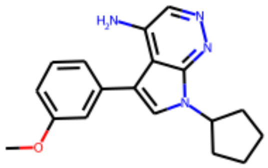	COc1cccc(-c2cn(C3CCCC3)c3nncc(N)c23)c1	0.618172	2.645624	3.611880	4	0.828369

	src known mol	similarity	mol	smiles	qed	logP	SAS	rings	kinase inhibition likeliness
**60**	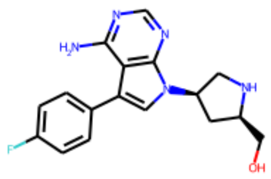	[0.6448345712356516]	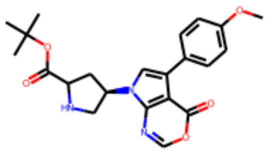	COc1ccc(-c2cn([C@H]3CNC(C(=O)OC(C)(C)C)C3)c3ncoc(=O)c23)cc1	0.709527	2.560076	2.763322	4	0.791833
**61**	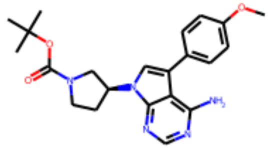	[0.634527687296417]	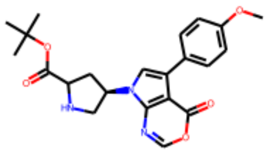	COc1ccc(-c2cn([C@H]3CNC(C(=O)OC(C)(C)C)C3)c3ncoc(=O)c23)cc1	0.709527	2.560076	2.763322	4	0.791833
**62**	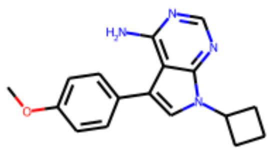	[0.6310452418096724]	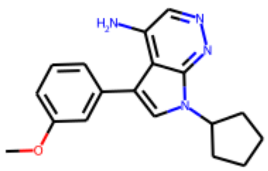	COc1cccc(-c2cn(C3CCCC3)c3nncc(N)c23)c1	0.618172	2.645624	3.611880	4	0.828369
**63**	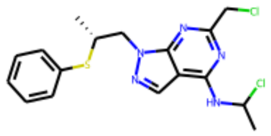	[0.6270523846755277]	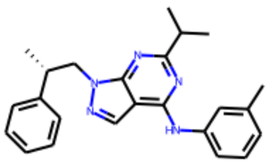	CC(C)c1nc(Nc2cccc(C)c2)c2cnn(C[C@@H](C)c3ccccc3)c2n1	0.582066	3.453236	4.511770	4	0.771524

	src known mol	similarity	mol	smiles	qed	logP	SAS	rings	kinase inhibition likeliness
**64**	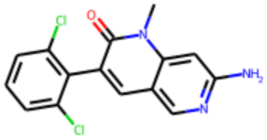	[0.6256938937351308]	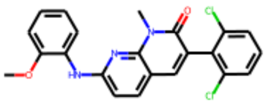	COc1ccccc1Nc1ccc2cc(-c3c(Cl)cccc3Cl)c(=O)n(C)c2n1	0.773249	2.760402	2.523284	4	0.821857
**65**	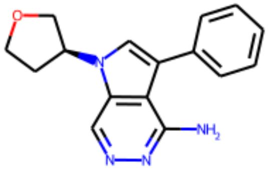	[0.6236559139784946]	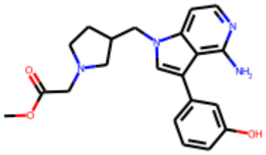	COC(=O)CN1CC(Cn2cc(-c3cccc(O)c3)c3c(N)nccc32)CC1	0.582066	3.453236	4.511770	4	0.822500
**66**	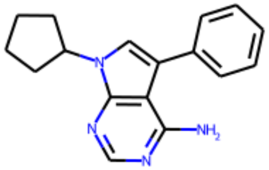	[0.6208791208791209]	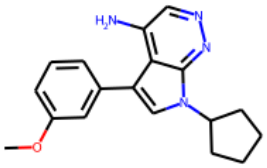	COc1cccc(-c2cn(C3CCCC3)c3nncc(N)c23)c1	0.618172	2.645624	3.611880	4	0.828369
**67**	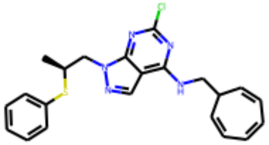	[0.6201427438540841]	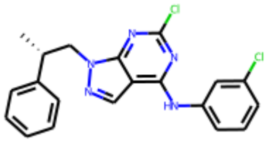	Clc1nc(Nc2cccc(Cl)c2)c2cnn(C[C@@H](C)c3ccccc3)c2n1	0.812670	2.803140	2.415127	4	0.800810

	src known mol	similarity	mol	smiles	qed	logP	SAS	rings	kinase inhibition likeliness
**68**	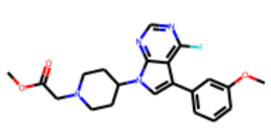	[0.59734219269103]	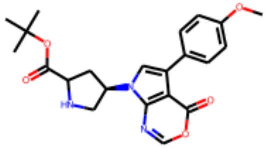	COc1ccc(-c2cn([C@H]3CNC(C(=O)OC(C)(C)C)C3)c3ncoc(=O)c23)cc1	0.709527	2.560076	2.763322	4	0.791833
**69**	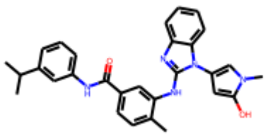	[0.5907894736842105]	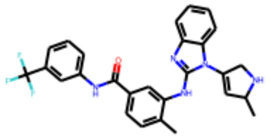	Cc1ccc(C(=O)Nc2cccc(C(F)(F)F)c2)cc1Nc1nc2ccccc2n1-c1CNC(C)c1	0.837081	4.329537	2.430174	5	0.773214
**70**	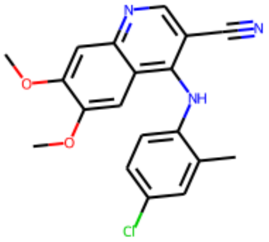	[0.582089552238806]	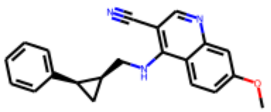	COc1ccc2c(NC[C@H]3C[C@H]3c3ccccc3)c(C#N)cnc2c1	0.709527	2.560076	2.763322	4	0.790969
**71**	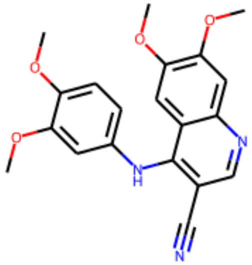	[0.5786993402450519]	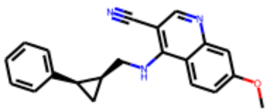	COc1ccc2c(NC[C@H]3C[C@H]3c3ccccc3)c(C#N)cnc2c1	0.709527	2.560076	2.763322	4	0.790969

	src known mol	similarity	mol	smiles	qed	logP	SAS	rings	kinase inhibition likeliness
**72**	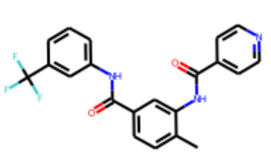	[0.5786314525810324]	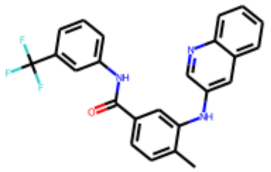	Cc1ccc(C(=O)Nc2cccc(C(F)(F)F)c2)cc1Nc1cc2ccccc2nc1	0.801881	4.739956	1.893492	4	0.736596
**73**	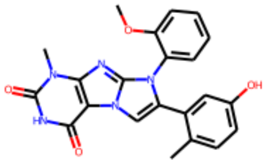	[0.5759625390218522]	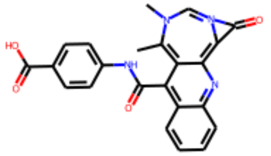	Cn1c(C)c2c(C(=O)Nc3ccc(C(=O)O)cc3)c3ccccc3nc2c2C(=O)n2=C1	0.563628	3.441104	4.745130	5	0.732357
**74**	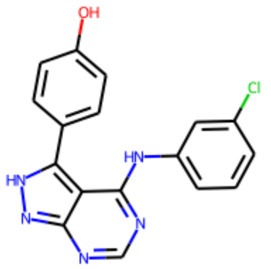	[0.5752636625119847]	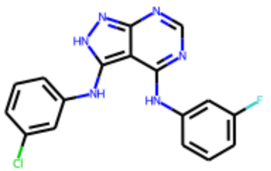	Fc1cccc(Nc2ncnc3n[nH]c(Nc4cccc(Cl)c4)c23)c1	0.648843	2.528773	3.556390	4	0.786036
**75**	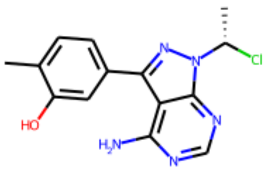	[0.5751724137931035]	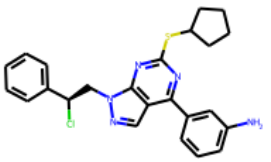	Nc1cccc(-c2nc(SC3CCCC3)nc3c2cnn3C[C@@H](Cl)c2ccccc2)c1	0.693247	2.498708	3.629723	5	0.805611

	src known mol	similarity	mol	smiles	qed	logP	SAS	rings	kinase inhibition likeliness
**76**	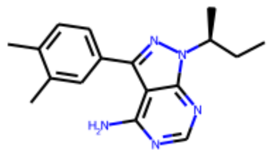	[0.5750174459176552]	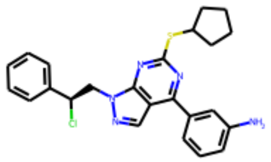	Nc1cccc(-c2nc(SC3CCCC3)nc3c2cnn3C[C@@H](Cl)c2ccccc2)c1	0.693247	2.498708	3.629723	5	0.805611
**77**	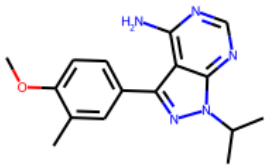	[0.5745276417074877]	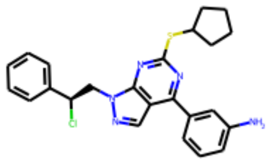	Nc1cccc(-c2nc(SC3CCCC3)nc3c2cnn3C[C@@H](Cl)c2ccccc2)c1	0.693247	2.498708	3.629723	5	0.805611
**78**	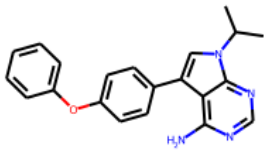	[0.5737451737451738]	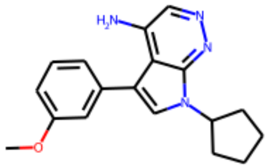	COc1cccc(-c2cn(C3CCCC3)c3nncc(N)c23)c1	0.618172	2.645624	3.611880	4	0.828369
**79**	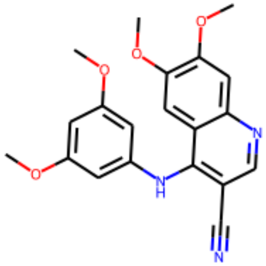	[0.5727611940298507]	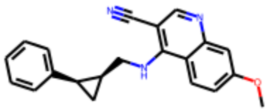	COc1ccc2c(NC[C@H]3C[C@H]3c3ccccc3)c(C#N)cnc2c1	0.709527	2.560076	2.763322	4	0.790969

	src known mol	similarity	mol	smiles	qed	logP	SAS	rings	kinase inhibition likeliness
**80**	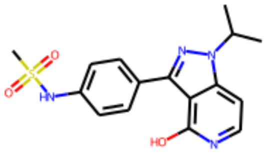	[0.5720620842572062]	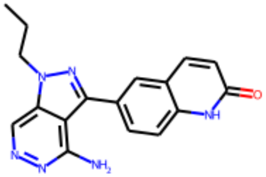	C(CC)n1nc(-c2ccc3[nH]c(=O)ccc3c2)c2c(N)nncc21	0.518739	2.583386	5.711228	4	0.891891
**81**	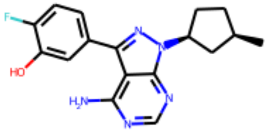	[0.5718270571827058]	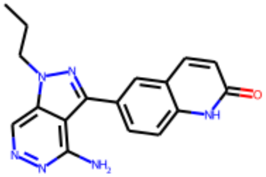	C(CC)n1nc(-c2ccc3[nH]c(=O)ccc3c2)c2c(N)nncc21	0.518739	2.583386	5.711228	4	0.891891
**82**	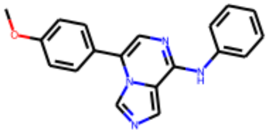	[0.5711592836946278]	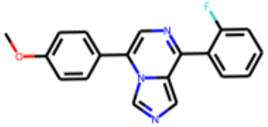	COc1ccc(-c2cnc(-c3ccccc3F)c3cncn23)cc1	0.836239	3.357318	2.011011	4	0.800881
**83**	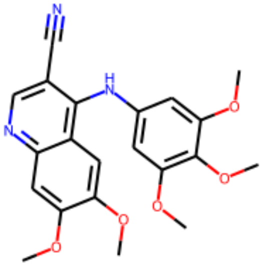	[0.5710332103321033]	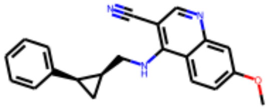	COc1ccc2c(NC[C@H]3C[C@H]3c3ccccc3)c(C#N)cnc2c1	0.709527	2.560076	2.763322	4	0.790969

	src known mol	similarity	mol	smiles	qed	logP	SAS	rings	kinase inhibition likeliness
**84**	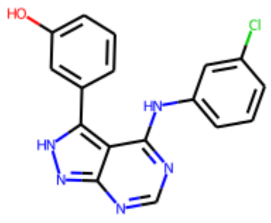	[0.568738229755179]	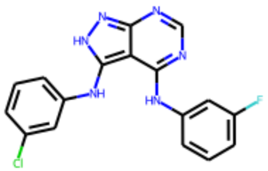	Fc1cccc(Nc2ncnc3n[nH]c(Nc4cccc(Cl)c4)c23)c1	0.648843	2.528773	3.556390	4	0.786036
**85**	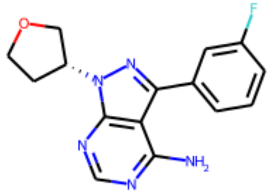	[0.5677731673582296]	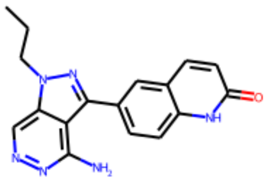	C(CC)n1nc(-c2ccc3[nH]c(=O)ccc3c2)c2c(N)nncc21	0.518739	2.583386	5.711228	4	0.891891
**86**	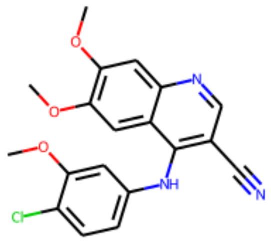	[0.5652173913043478]	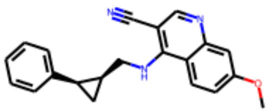	COc1ccc2c(NC[C@H]3C[C@H]3c3ccccc3)c(C#N)cnc2c1	0.709527	2.560076	2.763322	4	0.790969
**87**	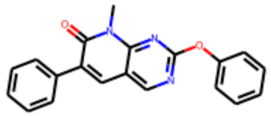	[0.5645896656534954]	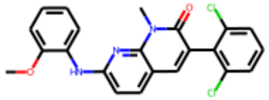	COc1ccccc1Nc1ccc2cc(-c3c(Cl)cccc3Cl)c(=O)n(C)c2n1	0.773249	2.760402	2.523284	4	0.821857

	src known mol	similarity	mol	smiles	qed	logP	SAS	rings	kinase inhibition likeliness
**88**	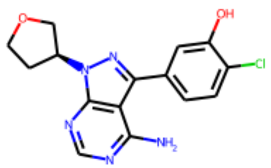	[0.563858695652174]	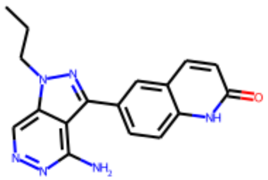	C(CC)n1nc(-c2ccc3[nH]c(=O)ccc3c2)c2c(N)nncc21	0.518739	2.583386	5.711228	4	0.891891
**89**	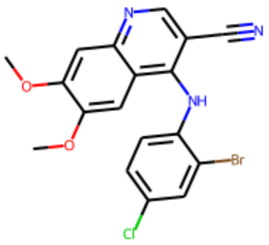	[0.5630550621669627]	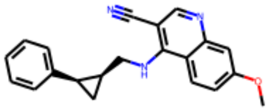	COc1ccc2c(NC[C@H]3C[C@H]3c3ccccc3)c(C#N)cnc2c1	0.709527	2.560076	2.763322	4	0.790969
**90**	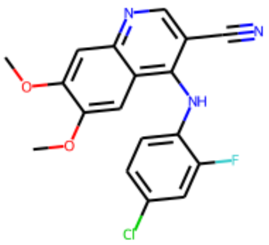	[0.5626666666666666]	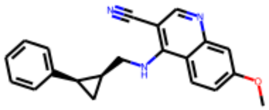	COc1ccc2c(NC[C@H]3C[C@H]3c3ccccc3)c(C#N)cnc2c1	0.709527	2.560076	2.763322	4	0.790969
**91**	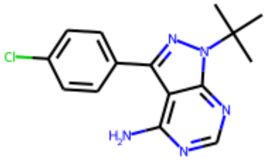	[0.5624548736462094]	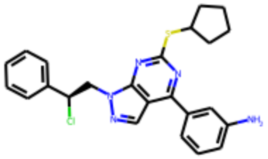	Nc1cccc(-c2nc(SC3CCCC3)nc3c2cnn3C[C@@H](Cl)c2ccccc2)c1	0.693247	2.498708	3.629723	5	0.805611

	src known mol	similarity	mol	smiles	qed	logP	SAS	rings	kinase inhibition likeliness
**92**	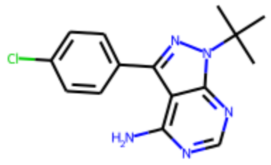	[0.5624548736462094]	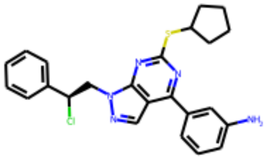	Nc1cccc(-c2nc(SC3CCCC3)nc3c2cnn3C[C@@H](Cl)c2ccccc2)c1	0.693247	2.498708	3.629723	5	0.805611
**93**	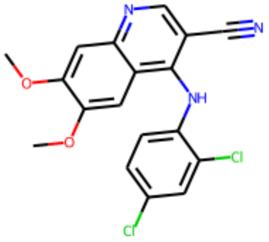	[0.5609756097560976]	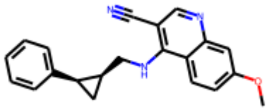	COc1ccc2c(NC[C@H]3C[C@H]3c3ccccc3)c(C#N)cnc2c1	0.709527	2.560076	2.763322	4	0.790969
**94**	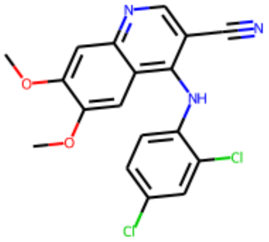	[0.5609756097560976]	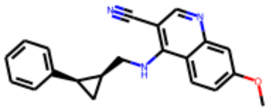	COc1ccc2c(NC[C@H]3C[C@H]3c3ccccc3)c(C#N)cnc2c1	0.709527	2.560076	2.763322	4	0.790969
**95**	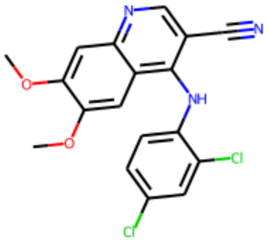	[0.5609756097560976]	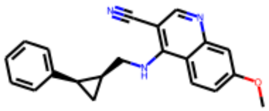	COc1ccc2c(NC[C@H]3C[C@H]3c3ccccc3)c(C#N)cnc2c1	0.709527	2.560076	2.763322	4	0.790969

	src known mol	similarity	mol	smiles	qed	logP	SAS	rings	kinase inhibition likeliness
**96**	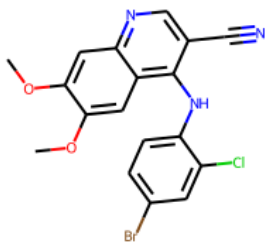	[0.5608888888888889]	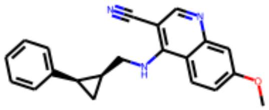	COc1ccc2c(NC[C@H]3C[C@H]3c3ccccc3)c(C#N)cnc2c1	0.709527	2.560076	2.763322	4	0.790969
**97**	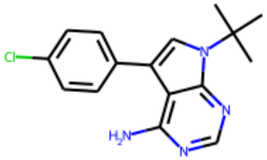	[0.5604311008468053]	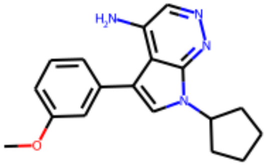	COc1cccc(-c2cn(C3CCCC3)c3nncc(N)c23)c1	0.618172	2.645624	3.611880	4	0.828369
**98**	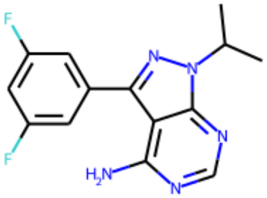	[0.5578571428571428]	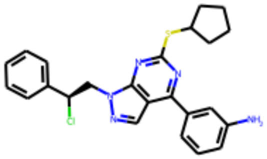	Nc1cccc(-c2nc(SC3CCCC3)nc3c2cnn3C[C@@H](Cl)c2ccccc2)c1	0.693247	2.498708	3.629723	5	0.805611
**99**	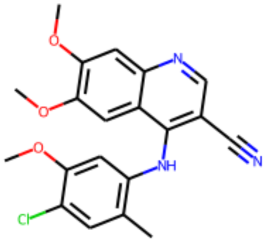	[0.5572052401746724]	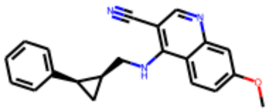	COc1ccc2c(NC[C@H]3C[C@H]3c3ccccc3)c(C#N)cnc2c1	0.709527	2.560076	2.763322	4	0.790969

	src known mol	similarity	mol	smiles	qed	logP	SAS	rings	kinase inhibition likeliness
**100**	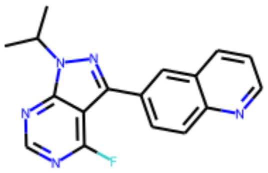	[0.5553956834532374]	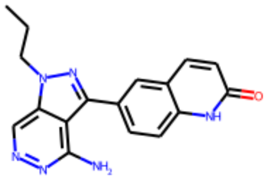	C(CC)n1nc(-c2ccc3[nH]c(=O)ccc3c2)c2c(N)nncc21	0.518739	2.583386	5.711228	4	0.891891
**101**	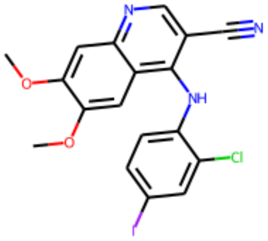	[0.5546666666666666]	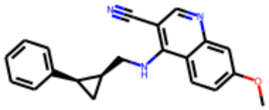	COc1ccc2c(NC[C@H]3C[C@H]3c3ccccc3)c(C#N)cnc2c1	0.709527	2.560076	2.763322	4	0.790969
**102**	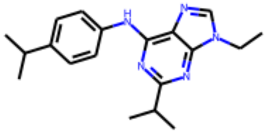	[0.5541158536585366]	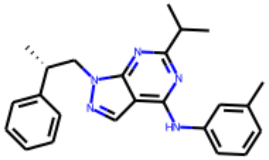	CC(C)c1nc(Nc2cccc(C)c2)c2cnn(C[C@@H](C)c3ccccc3)c2n1	0.582066	3.453236	4.511770	4	0.771524
**103**	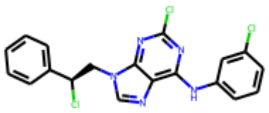	[0.5540641312453393]	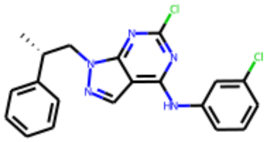	Clc1nc(Nc2cccc(Cl)c2)c2cnn(C[C@@H](C)c3ccccc3)c2n1	0.812670	2.803140	2.415127	4	0.800810

	src known mol	similarity	mol	smiles	qed	logP	SAS	rings	kinase inhibition likeliness
**104**	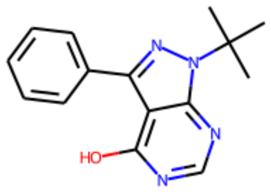	[0.55394641564084]	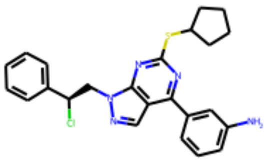	Nc1cccc(-c2nc(SC3CCCC3)nc3c2cnn3C[C@@H](Cl)c2ccccc2)c1	0.693247	2.498708	3.629723	5	0.805611
**105**	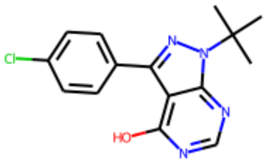	[0.5538132573057734]	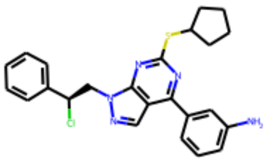	Nc1cccc(-c2nc(SC3CCCC3)nc3c2cnn3C[C@@H](Cl)c2ccccc2)c1	0.693247	2.498708	3.629723	5	0.805611
**106**	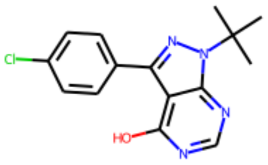	[0.5538132573057734]	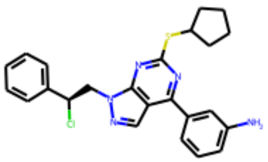	Nc1cccc(-c2nc(SC3CCCC3)nc3c2cnn3C[C@@H](Cl)c2ccccc2)c1	0.693247	2.498708	3.629723	5	0.805611
**107**	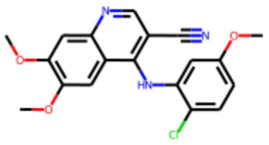	[0.5517241379310345]	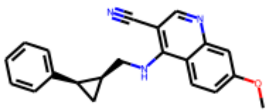	COc1ccc2c(NC[C@H]3C[C@H]3c3ccccc3)c(C#N)cnc2c1	0.709527	2.560076	2.763322	4	0.790969

	src known mol	similarity	mol	smiles	qed	logP	SAS	rings	kinase inhibition likeliness
**108**	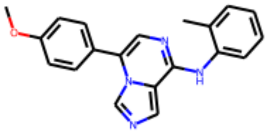	[0.5497335701598579]	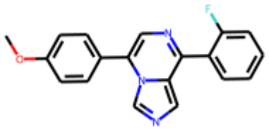	COc1ccc(-c2cnc(-c3ccccc3F)c3cncn23)cc1	0.836239	3.357318	2.011011	4	0.800881
**109**	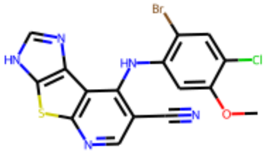	[0.5487012987012987]	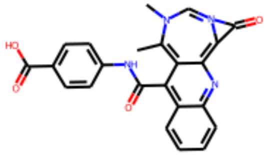	Cn1c(C)c2c(C(=O)Nc3ccc(C(=O)O)cc3)c3ccccc3nc2c2C(=O)n2=C1	0.563628	3.441104	4.745130	5	0.732357
**110**	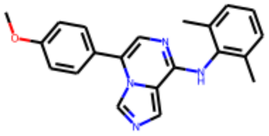	[0.5485008818342152]	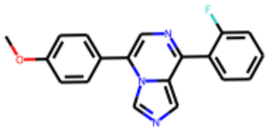	COc1ccc(-c2cnc(-c3ccccc3F)c3cncn23)cc1	0.836239	3.357318	2.011011	4	0.800881
**111**	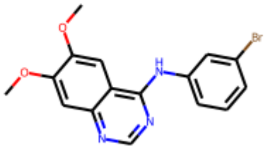	[0.5482587064676617]	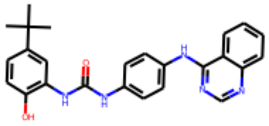	CC(C)(C)c1cc(NC(=O)Nc2ccc(Nc3ncnc4ccccc34)cc2)c(O)cc1	0.718099	4.269790	-0.023583	4	0.763474

	src known mol	similarity	mol	smiles	qed	logP	SAS	rings	kinase inhibition likeliness
**112**	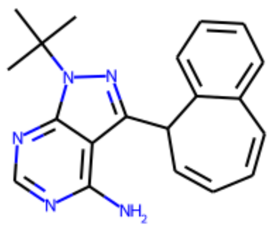	[0.5470588235294118]	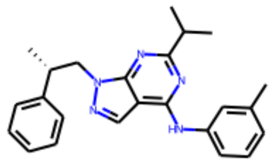	CC(C)c1nc(Nc2cccc(C)c2)c2cnn(C[C@@H](C)c3ccccc3)c2n1	0.582066	3.453236	4.511770	4	0.771524
**113**	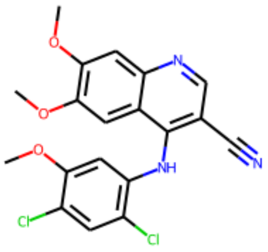	[0.5459227467811159]	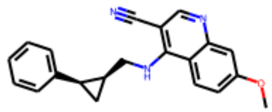	COc1ccc2c(NC[C@H]3C[C@H]3c3ccccc3)c(C#N)cnc2c1	0.709527	2.560076	2.763322	4	0.790969
**114**	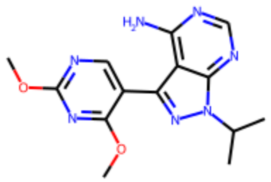	[0.5431773236651285]	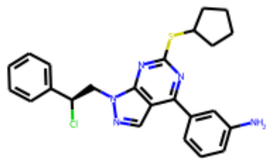	Nc1cccc(-c2nc(SC3CCCC3)nc3c2cnn3C[C@@H](Cl)c2ccccc2)c1	0.693247	2.498708	3.629723	5	0.805611
**115**	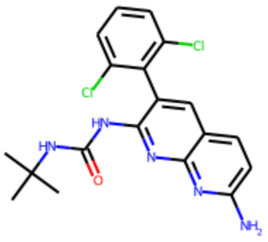	[0.5430711610486891]	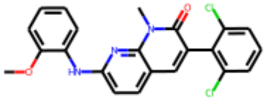	COc1ccccc1Nc1ccc2cc(-c3c(Cl)cccc3Cl)c(=O)n(C)c2n1	0.773249	2.760402	2.523284	4	0.821857

	src known mol	similarity	mol	smiles	qed	logP	SAS	rings	kinase inhibition likeliness
**116**	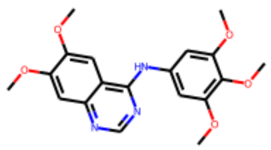	[0.5412221144519883]	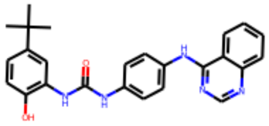	CC(C)(C)c1cc(NC(=O)Nc2ccc(Nc3ncnc4ccccc34)cc2)c(O)cc1	0.718099	4.269790	-0.023583	4	0.763474
**117**	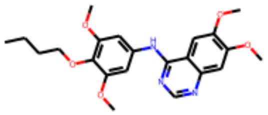	[0.5409219190968956]	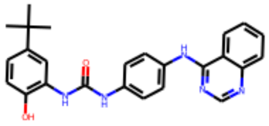	CC(C)(C)c1cc(NC(=O)Nc2ccc(Nc3ncnc4ccccc34)cc2)c(O)cc1	0.718099	4.269790	-0.023583	4	0.763474
**118**	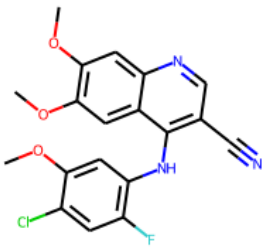	[0.540133779264214]	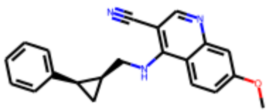	COc1ccc2c(NC[C@H]3C[C@H]3c3ccccc3)c(C#N)cnc2c1	0.709527	2.560076	2.763322	4	0.790969
**119**	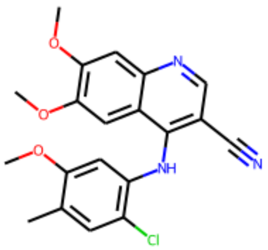	[0.5398981324278438]	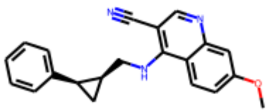	COc1ccc2c(NC[C@H]3C[C@H]3c3ccccc3)c(C#N)cnc2c1	0.709527	2.560076	2.763322	4	0.790969

	src known mol	similarity	mol	smiles	qed	logP	SAS	rings	kinase inhibition likeliness
**120**	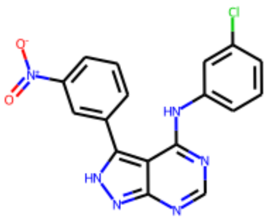	[0.5394853593611357]	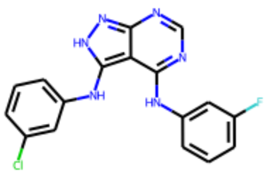	Fc1cccc(Nc2ncnc3n[nH]c(Nc4cccc(Cl)c4)c23)c1	0.648843	2.528773	3.556390	4	0.786036
**121**	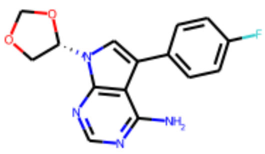	[0.5391737891737892]	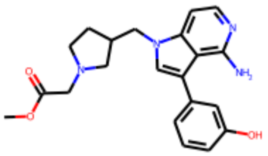	COC(=O)CN1CC(Cn2cc(-c3cccc(O)c3)c3c(N)nccc32)CC1	0.582066	3.453236	4.511770	4	0.822500
**122**	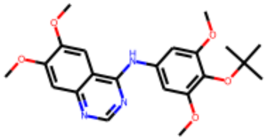	[0.5380997177798683]	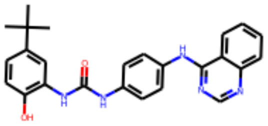	CC(C)(C)c1cc(NC(=O)Nc2ccc(Nc3ncnc4ccccc34)cc2)c(O)cc1	0.718099	4.269790	-0.023583	4	0.763474
**123**	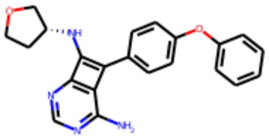	[0.5364751452550033]	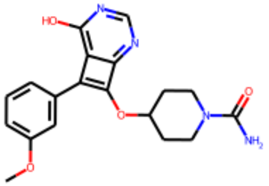	COc1cccc(-c2c(O[C@H]3CCN(C(N)=O)CC3)c3ncnc(O)c32)c1	0.528532	2.861050	5.353921	4	0.767083

	src known mol	similarity	mol	smiles	qed	logP	SAS	rings	kinase inhibition likeliness
**124**	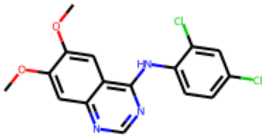	[0.5336538461538461]	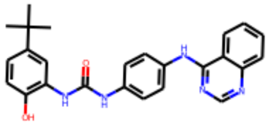	CC(C)(C)c1cc(NC(=O)Nc2ccc(Nc3ncnc4ccccc34)cc2)c(O)cc1	0.718099	4.269790	-0.023583	4	0.763474
**125**	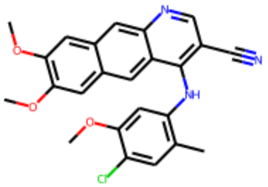	[0.529567519858782]	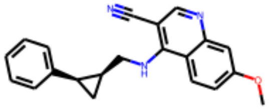	COc1ccc2c(NC[C@H]3C[C@H]3c3ccccc3)c(C#N)cnc2c1	0.709527	2.560076	2.763322	4	0.790969
**126**	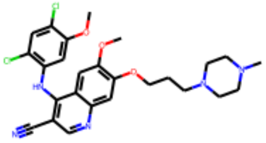	[0.5278900565885206]	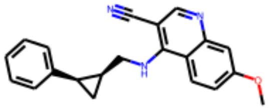	COc1ccc2c(NC[C@H]3C[C@H]3c3ccccc3)c(C#N)cnc2c1	0.709527	2.560076	2.763322	4	0.790969
**127**	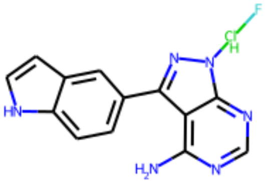	[0.5267341040462428]	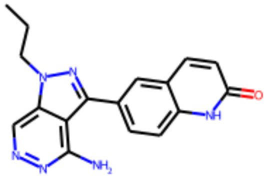	C(CC)n1nc(-c2ccc3[nH]c(=O)ccc3c2)c2c(N)nncc21	0.518739	2.583386	5.711228	4	0.891891

	src known mol	similarity	mol	smiles	qed	logP	SAS	rings	kinase inhibition likeliness
**128**	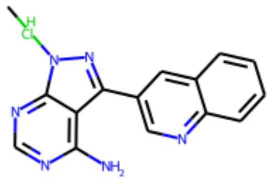	[0.5263157894736842]	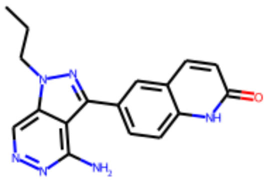	C(CC)n1nc(-c2ccc3[nH]c(=O)ccc3c2)c2c(N)nncc21	0.518739	2.583386	5.711228	4	0.891891
**129**	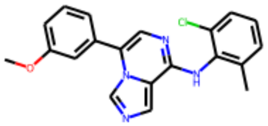	[0.5255354200988468]	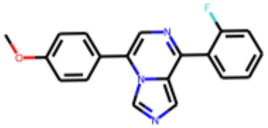	COc1ccc(-c2cnc(-c3ccccc3F)c3cncn23)cc1	0.836239	3.357318	2.011011	4	0.800881
**130**	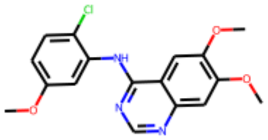	[0.5247895229186156]	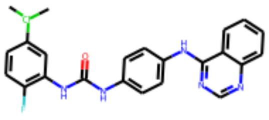	Cl(C)(C)c1cc(NC(=O)Nc2ccc(Nc3ncnc4ccccc34)cc2)c(F)cc1	0.802754	4.453978	1.994952	4	0.757125
**131**	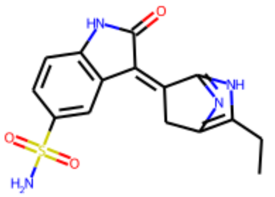	[0.5245641838351822]	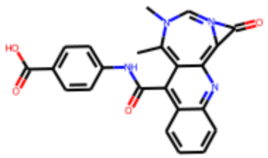	Cn1c(C)c2c(C(=O)Nc3ccc(C(=O)O)cc3)c3ccccc3nc2c2C(=O)n2=C1	0.563628	3.441104	4.745130	5	0.732357

	src known mol	similarity	mol	smiles	qed	logP	SAS	rings	kinase inhibition likeliness
**132**	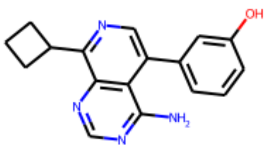	[0.5220913107511046]	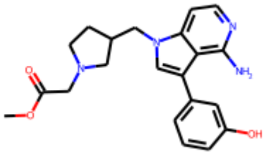	COC(=O)CN1CC(Cn2cc(-c3cccc(O)c3)c3c(N)nccc32)CC1	0.582066	3.453236	4.511770	4	0.822500
**133**	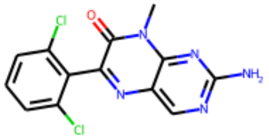	[0.5215243472124206]	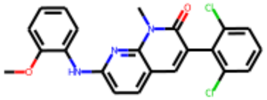	COc1ccccc1Nc1ccc2cc(-c3c(Cl)cccc3Cl)c(=O)n(C)c2n1	0.773249	2.760402	2.523284	4	0.821857
**134**	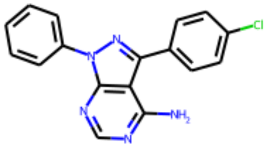	[0.5202808112324493]	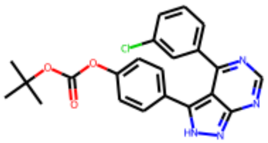	CC(C)(C)OC(=O)Oc1ccc(-c2[nH]nc3ncnc(-c4cccc(Cl)c4)c23)cc1	0.821471	2.730201	2.924359	4	0.823833
**135**	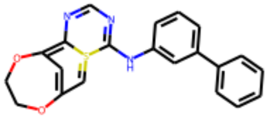	[0.5196078431372549]	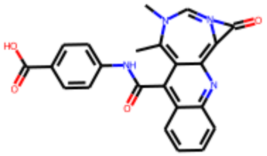	Cn1c(C)c2c(C(=O)Nc3ccc(C(=O)O)cc3)c3ccccc3nc2c2C(=O)n2=C1	0.563628	3.441104	4.745130	5	0.732357

	src known mol	similarity	mol	smiles	qed	logP	SAS	rings	kinase inhibition likeliness
**136**	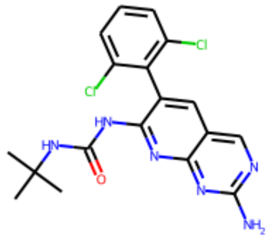	[0.5194805194805194]	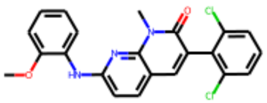	COc1ccccc1Nc1ccc2cc(-c3c(Cl)cccc3Cl)c(=O)n(C)c2n1	0.773249	2.760402	2.523284	4	0.821857
**137**	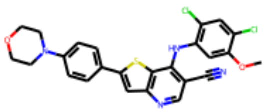	[0.5179240235420011]	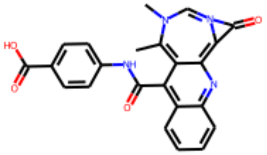	Cn1c(C)c2c(C(=O)Nc3ccc(C(=O)O)cc3)c3ccccc3nc2c2C(=O)n2=C1	0.563628	3.441104	4.745130	5	0.732357
**138**	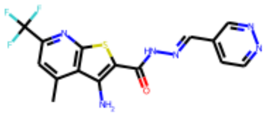	[0.5161821173889194]	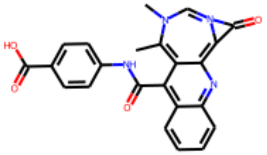	Cn1c(C)c2c(C(=O)Nc3ccc(C(=O)O)cc3)c3ccccc3nc2c2C(=O)n2=C1	0.563628	3.441104	4.745130	5	0.732357
**139**	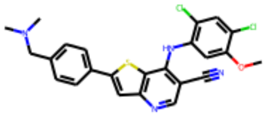	[0.5140237324703344]	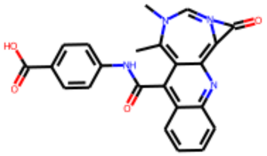	Cn1c(C)c2c(C(=O)Nc3ccc(C(=O)O)cc3)c3ccccc3nc2c2C(=O)n2=C1	0.563628	3.441104	4.745130	5	0.732357

	src known mol	similarity	mol	smiles	qed	logP	SAS	rings	kinase inhibition likeliness
**140**	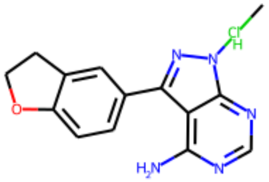	[0.5136157337367625]	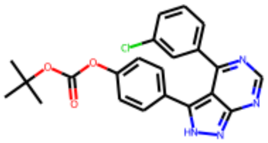	CC(C)(C)OC(=O)Oc1ccc(-c2[nH]nc3ncnc(-c4cccc(Cl)c4)c23)cc1	0.821471	2.730201	2.924359	4	0.823833
**141**	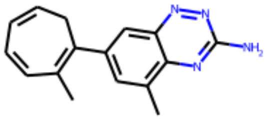	[0.5082508250825083]	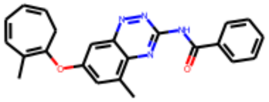	C1ccccc(C)c1Oc1cc(C)c2nc(NC(=O)c3ccccc3)nnc2c1	0.693247	2.498708	3.629723	4	0.794857
**142**	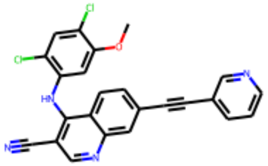	[0.4991624790619765]	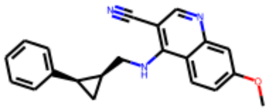	COc1ccc2c(NC[C@H]3C[C@H]3c3ccccc3)c(C#N)cnc2c1	0.709527	2.560076	2.763322	4	0.790969
**143**	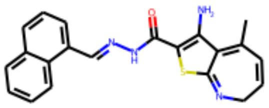	[0.4991587212563096]	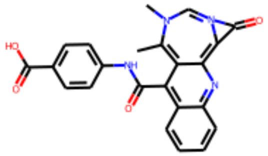	Cn1c(C)c2c(C(=O)Nc3ccc(C(=O)O)cc3)c3ccccc3nc2c2C(=O)n2=C1	0.563628	3.441104	4.745130	5	0.732357

	src known mol	similarity	mol	smiles	qed	logP	SAS	rings	kinase inhibition likeliness
**144**	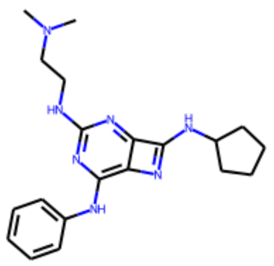	[0.49305169538632576]	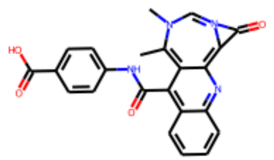	Cn1c(C)c2c(C(=O)Nc3ccc(C(=O)O)cc3)c3ccccc3nc2c2C(=O)n2=C1	0.563628	3.441104	4.745130	5	0.732357
**145**	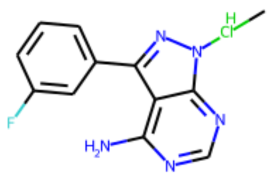	[0.49029126213592233]	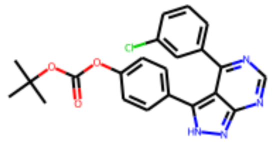	CC(C)(C)OC(=O)Oc1ccc(-c2[nH]nc3ncnc(-c4cccc(Cl)c4)c23)cc1	0.821471	2.730201	2.924359	4	0.823833
**146**	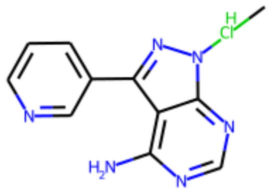	[0.4859887910328263]	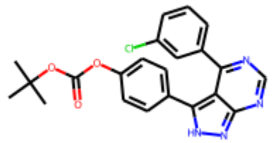	CC(C)(C)OC(=O)Oc1ccc(-c2[nH]nc3ncnc(-c4cccc(Cl)c4)c23)cc1	0.821471	2.730201	2.924359	4	0.823833
**147**	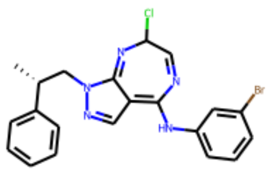	[0.48321408915795266]	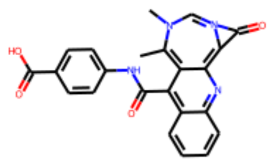	Cn1c(C)c2c(C(=O)Nc3ccc(C(=O)O)cc3)c3ccccc3nc2c2C(=O)n2=C1	0.563628	3.441104	4.745130	5	0.732357

	src known mol	similarity	mol	smiles	qed	logP	SAS	rings	kinase inhibition likeliness
**148**	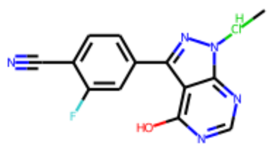	[0.4821981424148607]	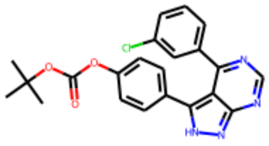	CC(C)(C)OC(=O)Oc1ccc(-c2[nH]nc3ncnc(-c4cccc(Cl)c4)c23)cc1	0.821471	2.730201	2.924359	4	0.823833
**149**	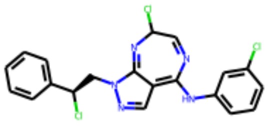	[0.4817880794701987]	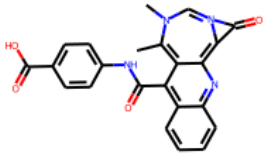	Cn1c(C)c2c(C(=O)Nc3ccc(C(=O)O)cc3)c3ccccc3nc2c2C(=O)n2=C1	0.563628	3.441104	4.745130	5	0.732357
**150**	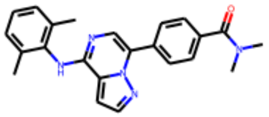	[0.4811946902654867]	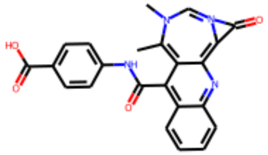	Cn1c(C)c2c(C(=O)Nc3ccc(C(=O)O)cc3)c3ccccc3nc2c2C(=O)n2=C1	0.563628	3.441104	4.745130	5	0.732357
**151**	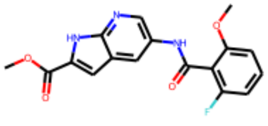	[0.48011363636363635]	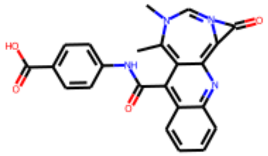	Cn1c(C)c2c(C(=O)Nc3ccc(C(=O)O)cc3)c3ccccc3nc2c2C(=O)n2=C1	0.563628	3.441104	4.745130	5	0.732357

	src known mol	similarity	mol	smiles	qed	logP	SAS	rings	kinase inhibition likeliness
**152**	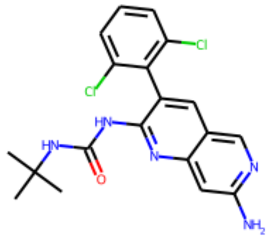	[0.47988505747126436]	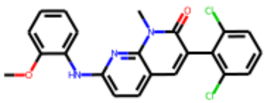	COc1ccccc1Nc1ccc2cc(-c3c(Cl)cccc3Cl)c(=O)n(C)c2n1	0.773249	2.760402	2.523284	4	0.821857
**153**	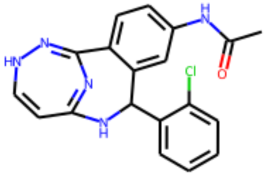	[0.4714285714285714]	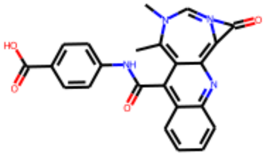	Cn1c(C)c2c(C(=O)Nc3ccc(C(=O)O)cc3)c3ccccc3nc2c2C(=O)n2=C1	0.563628	3.441104	4.745130	5	0.732357
**154**	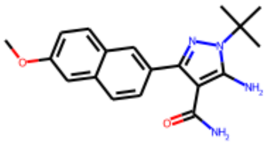	[0.47091800981079185]	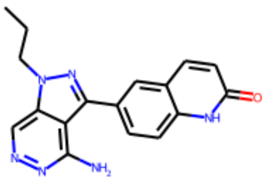	C(CC)n1nc(-c2ccc3[nH]c(=O)ccc3c2)c2c(N)nncc21	0.518739	2.583386	5.711228	4	0.891891
**155**	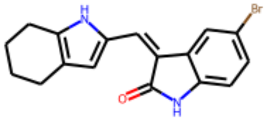	[0.4668141592920354]	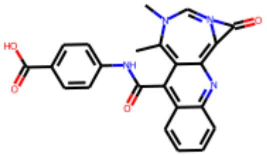	Cn1c(C)c2c(C(=O)Nc3ccc(C(=O)O)cc3)c3ccccc3nc2c2C(=O)n2=C1	0.563628	3.441104	4.745130	5	0.732357

	src known mol	similarity	mol	smiles	qed	logP	SAS	rings	kinase inhibition likeliness
**156**	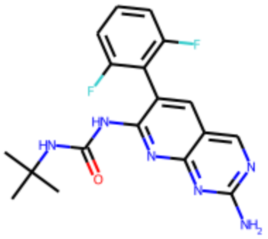	[0.4626038781163435]	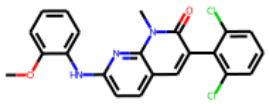	COc1ccccc1Nc1ccc2cc(-c3c(Cl)cccc3Cl)c(=O)n(C)c2n1	0.773249	2.760402	2.523284	4	0.821857
**157**	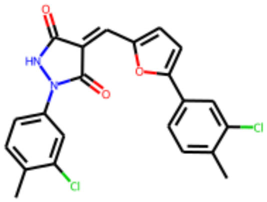	[0.46102819237147596]	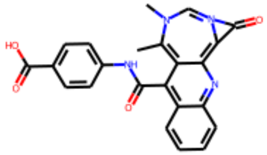	Cn1c(C)c2c(C(=O)Nc3ccc(C(=O)O)cc3)c3ccccc3nc2c2C(=O)n2=C1	0.563628	3.441104	4.745130	5	0.732357
**158**	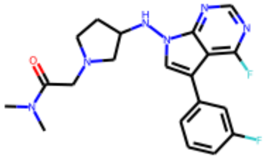	[0.46092307692307694]	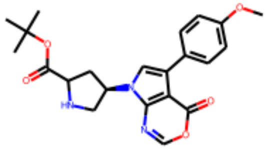	COc1ccc(-c2cn([C@H]3CNC(C(=O)OC(C)(C)C)C3)c3ncoc(=O)c23)cc1	0.709527	2.560076	2.763322	4	0.791833
**159**	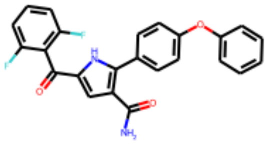	[0.45710627400768244]	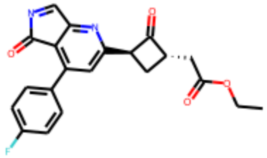	CCOC(=O)C[C@@H]1C[C@@H](c2cc(-c3ccc(F)cc3)c3c(=O)ncc3n2)C1=O	0.814616	2.897929	3.830811	4	0.733397

	src known mol	similarity	mol	smiles	qed	logP	SAS	rings	kinase inhibition likeliness
**160**	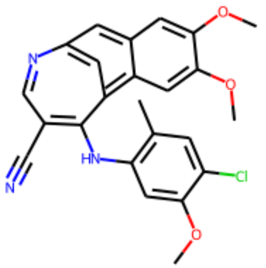	[0.45419211549139366]	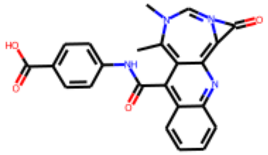	Cn1c(C)c2c(C(=O)Nc3ccc(C(=O)O)cc3)c3ccccc3nc2c2C(=O)n2=C1	0.563628	3.441104	4.745130	5	0.732357
**161**	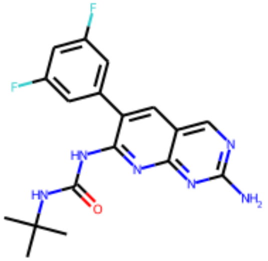	[0.45396600566572237]	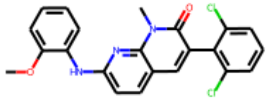	COc1ccccc1Nc1ccc2cc(-c3c(Cl)cccc3Cl)c(=O)n(C)c2n1	0.773249	2.760402	2.523284	4	0.821857
**162**	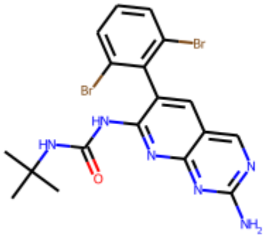	[0.4515905947441217]	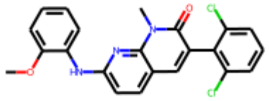	COc1ccccc1Nc1ccc2cc(-c3c(Cl)cccc3Cl)c(=O)n(C)c2n1	0.773249	2.760402	2.523284	4	0.821857
**163**	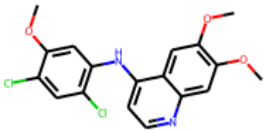	[0.4500998003992016]	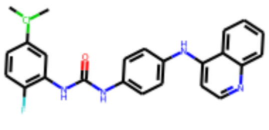	Cl(C)(C)c1cc(NC(=O)Nc2ccc(Nc3ccnc4ccccc34)cc2)c(F)cc1	0.522453	2.355392	5.398832	4	0.845766

	src known mol	similarity	mol	smiles	qed	logP	SAS	rings	kinase inhibition likeliness
**164**	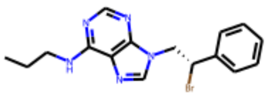	[0.4477498093058734]	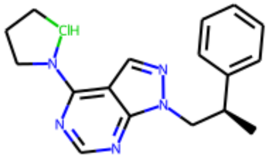	C[C@@H](Cn1ncc2c(N3CCCCl3)ncnc21)c1ccccc1	0.618172	2.645624	3.611880	4	0.778794
**165**	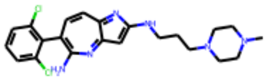	[0.44202389318341534]	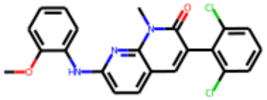	COc1ccccc1Nc1ccc2cc(-c3c(Cl)cccc3Cl)c(=O)n(C)c2n1	0.773249	2.760402	2.523284	4	0.821857
**166**	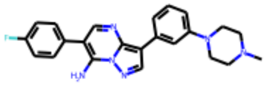	[0.43993231810490696]	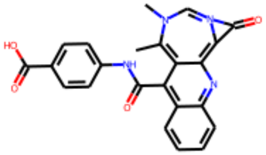	Cn1c(C)c2c(C(=O)Nc3ccc(C(=O)O)cc3)c3ccccc3nc2c2C(=O)n2=C1	0.563628	3.441104	4.745130	5	0.732357
**167**	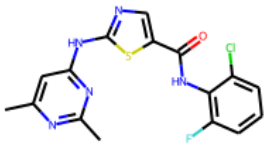	[0.43495475113122173]	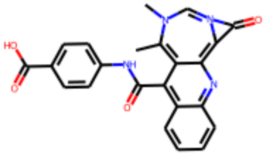	Cn1c(C)c2c(C(=O)Nc3ccc(C(=O)O)cc3)c3ccccc3nc2c2C(=O)n2=C1	0.563628	3.441104	4.745130	5	0.732357

	src known mol	similarity	mol	smiles	qed	logP	SAS	rings	kinase inhibition likeliness
**168**	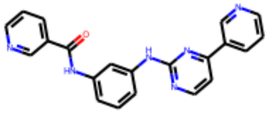	[0.43478260869565216]	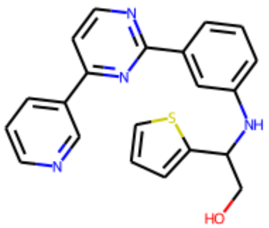	OCC(Nc1cccc(/c2nccc(-c3cccnc3)n2)c1)c1cccs1	0.582066	3.453236	4.511770	4	0.869905
**169**	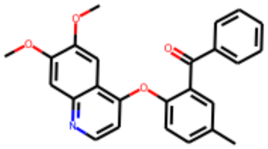	[0.4338807260155575]	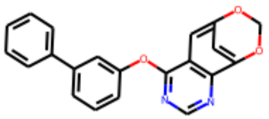	c1ccc(-c2cccc(Oc3ncnc4c3cc(cc34)OCO3)c2)cc1	0.582066	3.453236	4.511770	5	0.847750
**170**	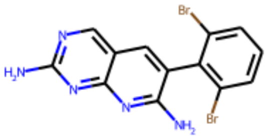	[0.4214939024390244]	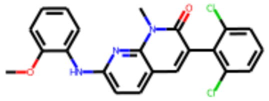	COc1ccccc1Nc1ccc2cc(-c3c(Cl)cccc3Cl)c(=O)n(C)c2n1	0.773249	2.760402	2.523284	4	0.821857
**171**	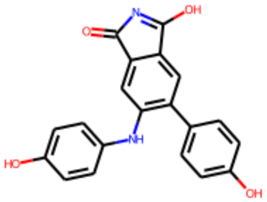	[0.4146825396825397]	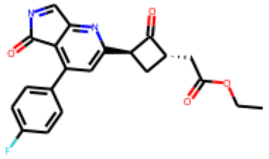	CCOC(=O)C[C@@H]1C[C@@H](c2cc(-c3ccc(F)cc3)c3c(=O)ncc3n2)C1=O	0.814616	2.897929	3.830811	4	0.733397

	src known mol	similarity	mol	smiles	qed	logP	SAS	rings	kinase inhibition likeliness
**172**	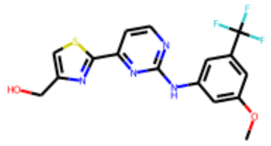	[0.4095052083333333]	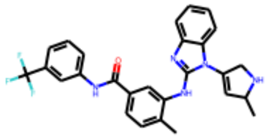	Cc1ccc(C(=O)Nc2cccc(C(F)(F)F)c2)cc1Nc1nc2ccccc2n1-c1CNC(C)c1	0.837081	4.329537	2.430174	5	0.773214
**173**	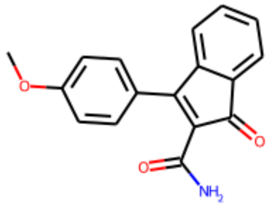	[0.40523338048090524]	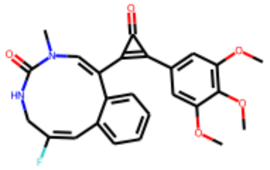	COc1cc(C2=C(c3cn(C(=O)NCc(F)cc4ccccc43)C)C2=O)cc(OC)c1OC	0.618172	2.645624	3.611880	4	0.774782
**174**	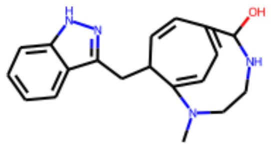	[0.4028011204481793]	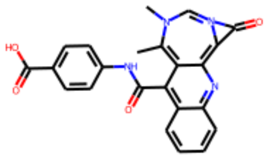	Cn1c(C)c2c(C(=O)Nc3ccc(C(=O)O)cc3)c3ccccc3nc2c2C(=O)n2=C1	0.563628	3.441104	4.745130	5	0.732357
**175**	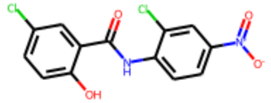	[0.39980059820538383]	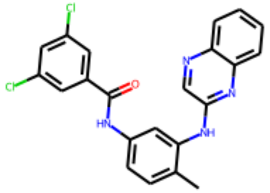	Cc1ccc(NC(=O)c2cc(Cl)cc(Cl)c2)cc1Nc1nc2ccccc2nc1	0.520232	2.229788	3.986566	4	0.818238

	src known mol	similarity	mol	smiles	qed	logP	SAS	rings	kinase inhibition likeliness
**176**	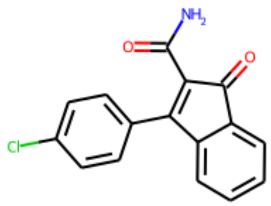	[0.39151943462897526]	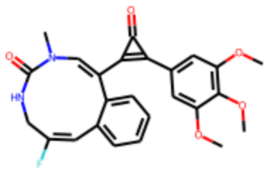	COc1cc(C2=C(c3cn(C(=O)NCc(F)cc4ccccc43)C)C2=O)cc(OC)c1OC	0.618172	2.645624	3.611880	4	0.774782
**177**	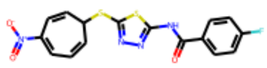	[0.3749271137026239]	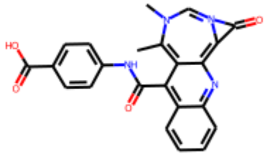	Cn1c(C)c2c(C(=O)Nc3ccc(C(=O)O)cc3)c3ccccc3nc2c2C(=O)n2=C1	0.563628	3.441104	4.745130	5	0.732357
**178**	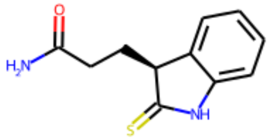	[0.369281045751634]	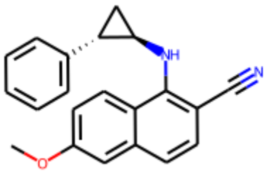	COc1ccc2c(N[C@@H]3C[C@H]3c3ccccc3)c(C#N)ccc2c1	0.693247	2.498708	3.629723	4	0.772905
**179**	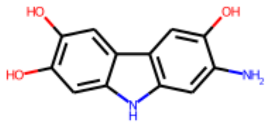	[0.36870310825529475]	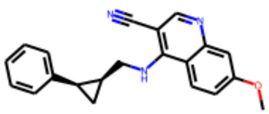	COc1ccc2c(NC[C@H]3C[C@H]3c3ccccc3)c(C#N)cnc2c1	0.709527	2.560076	2.763322	4	0.790969

	src known mol	similarity	mol	smiles	qed	logP	SAS	rings	kinase inhibition likeliness
**180**	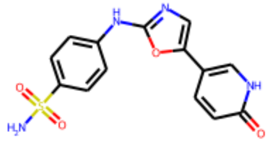	[0.36623748211731044]	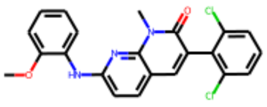	COc1ccccc1Nc1ccc2cc(-c3c(Cl)cccc3Cl)c(=O)n(C)c2n1	0.773249	2.760402	2.523284	4	0.821857
**181**	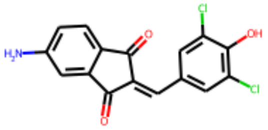	[0.3437738731856379]	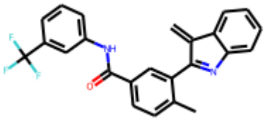	Cc1ccc(C(=O)Nc2cccc(C(F)(F)F)c2)cc1-c1nc2ccccc2c1=C	0.814616	2.897929	3.830811	4	0.746365
**182**	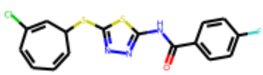	[0.3431603773584906]	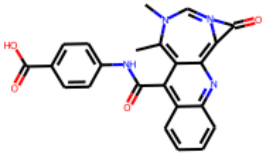	Cn1c(C)c2c(C(=O)Nc3ccc(C(=O)O)cc3)c3ccccc3nc2c2C(=O)n2=C1	0.563628	3.441104	4.745130	5	0.732357
**183**	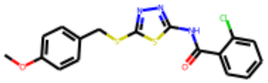	[0.3431516936671576]	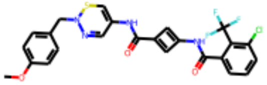	COc1ccc(Cn2ncc(NC(=O)c3cc(NC(=O)c4cccc(Cl)c4C(F)(F)F)c3)cs2)cc1	0.814616	2.897929	3.830811	4	0.744310

	src known mol	similarity	mol	smiles	qed	logP	SAS	rings	kinase inhibition likeliness
**184**	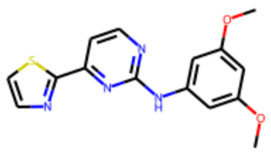	[0.34279475982532753]	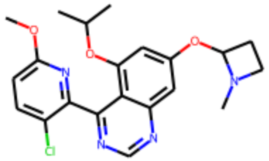	COc1ccc(Cl)c(-c2ncnc3cc(OC(N4C)CC4)cc(OC(C)C)c23)n1	0.710142	2.759610	3.436126	4	0.773833
**185**	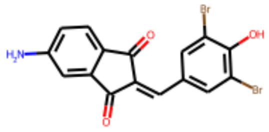	[0.3385345997286296]	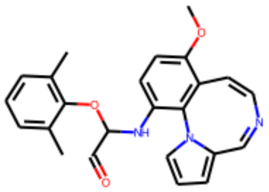	COc1ccc(NC(C=O)Oc2c(C)cccc2C)c2c1ccncc1cccn12	0.563628	3.441104	4.745130	4	0.822733
**186**	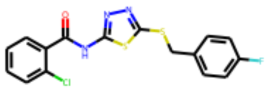	[0.33811475409836067]	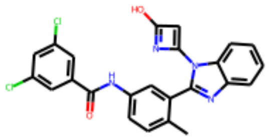	Cc1ccc(NC(=O)c2cc(Cl)cc(Cl)c2)cc1-c1nc2ccccc2n1-c1cc(O)n1	0.821471	2.730201	2.924359	5	0.800890
**187**	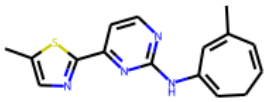	[0.3367756741250717]	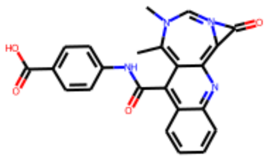	Cn1c(C)c2c(C(=O)Nc3ccc(C(=O)O)cc3)c3ccccc3nc2c2C(=O)n2=C1	0.563628	3.441104	4.745130	5	0.732357

	src known mol	similarity	mol	smiles	qed	logP	SAS	rings	kinase inhibition likeliness
**188**	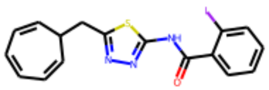	[0.32419465387251545]	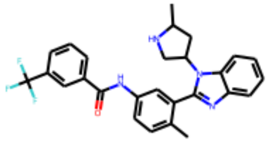	Cc1ccc(NC(=O)c2cccc(C(F)(F)F)c2)cc1-c1nc2ccccc2n1-c1CNC(C)C1	0.814616	2.897929	3.830811	5	0.804286
**189**	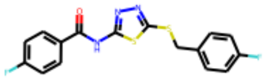	[0.3068340306834031]	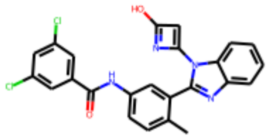	Cc1ccc(NC(=O)c2cc(Cl)cc(Cl)c2)cc1-c1nc2ccccc2n1-c1cc(O)n1	0.821471	2.730201	2.924359	5	0.800890
**190**	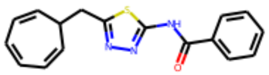	[0.3055555555555556]	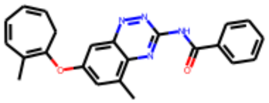	C1ccccc(C)c1Oc1cc(C)c2nc(NC(=O)c3ccccc3)nnc2c1	0.693247	2.498708	3.629723	4	0.794857
