# Supplementary material for: Interpretable Machine Learning Models for Molecular Design of Tyrosine Kinase Inhibitors Using Variational Autoencoders and Perturbation-Based Approach of Chemical Space Exploration
Source: Int J Mol Sci. 2022 Sep 24;23(19):11262. doi: 10.3390/ijms231911262 (PMC9569663; doi:10.3390/ijms231911262)
Supplement: Supplementary file 1 [file ijms-23-11262-s001.zip › SUPPLEMENTARY_MATERIALS/Generated_Molecules_With_Similarity/final_similarity_kinase_family_data (1).pdf]

	src known mol	similarity	smiles	mol	Class	qed	logP	SAS	rings	kinase inhibition likeliness
**0**	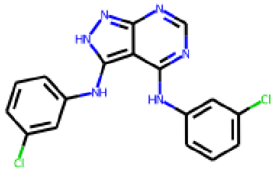	1.000000	Clc1cccc(Nc2ncnc3n[nH]c(Nc4cccc(Cl)c4)c23)c1	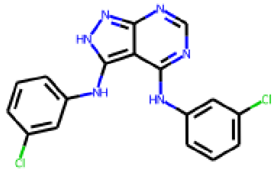	0.0	0.768132	3.608437	2.513170	4	0.771417
**1**	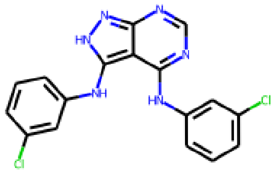	1.000000	Clc1cccc(Nc2ncnc3n[nH]c(Nc4cccc(Cl)c4)c23)c1	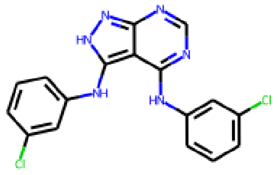	0.0	0.768132	3.608437	2.513170	4	0.771417
**2**	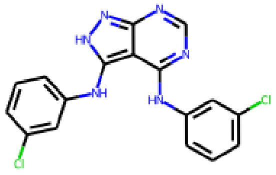	0.982808	Clc1cccc(Nc2ncnc3n[nH]c(Nc4cccc(Cl)c4)c23)c1	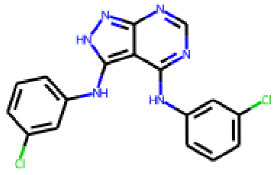	0.0	0.768132	3.608437	2.513170	4	0.771417
**3**	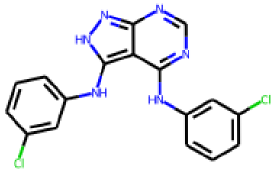	0.954103	Clc1cccc(Nc2ncnc3n[nH]c(Nc4cccc(Cl)c4)c23)c1	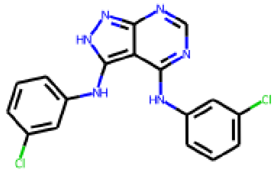	0.0	0.768132	3.608437	2.513170	4	0.771417

	src known mol	similarity	smiles	mol	Class	qed	logP	SAS	rings	kinase inhibition likeliness
**4**	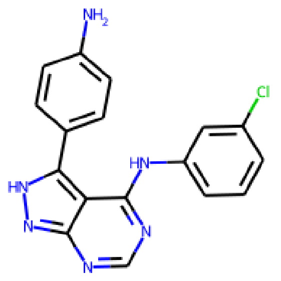	0.933633	c1ccc(Nc2ncnc3n[nH]c(-c4ccccc4)c23)cc1	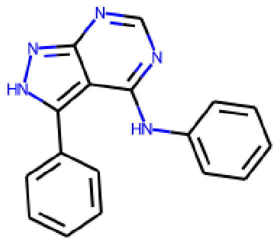	0.0	0.638163	3.995589	2.988471	4	0.800634
**5**	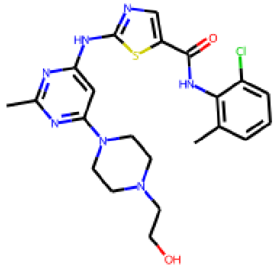	0.926684	Cc1nc(Nc2ncc(C(=O)Nc3c(C)cccc3Cl)s2)cc(N2CCNC(=O)C2)n1	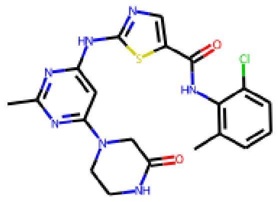	6.0	0.593231	3.250153	2.680275	4	0.755833
**6**	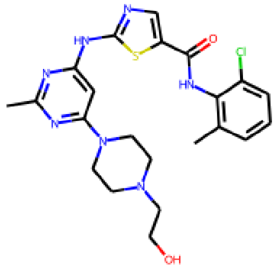	0.926684	Cc1nc(Nc2ncc(C(=O)Nc3c(C)cccc3Cl)s2)cc(N2CCNC(=O)C2)n1	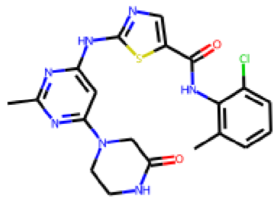	6.0	0.593231	3.250153	2.680275	4	0.755833
**7**	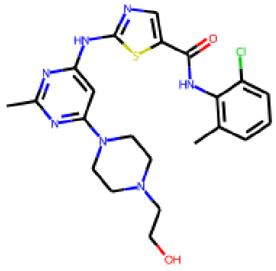	0.926684	Cc1nc(Nc2ncc(C(=O)Nc3c(C)cccc3Cl)s2)cc(N2CCNC(=O)C2)n1	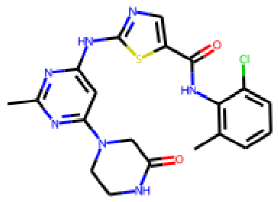	6.0	0.593231	3.250153	2.680275	4	0.755833

	src known mol	similarity	smiles	mol	Class	qed	logP	SAS	rings	kinase inhibition likeliness
**8**	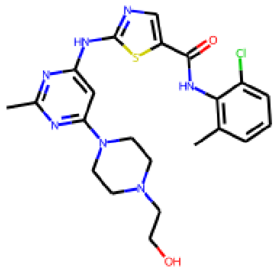	0.926684	Cc1nc(Nc2ncc(C(=O)Nc3c(C)cccc3Cl)s2)cc(N2CCNC(=O)C2)n1	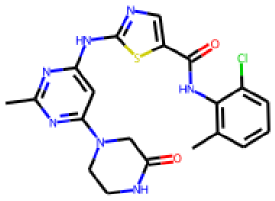	6.0	0.593231	3.250153	2.680275	4	0.755833
**9**	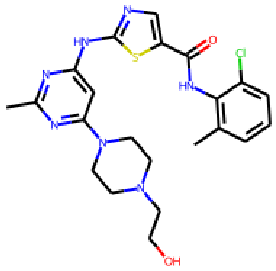	0.926684	Cc1nc(Nc2ncc(C(=O)Nc3c(C)cccc3Cl)s2)cc(N2CCNC(=O)C2)n1	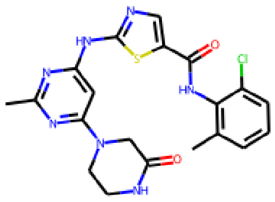	6.0	0.593231	3.250153	2.680275	4	0.755833
**10**	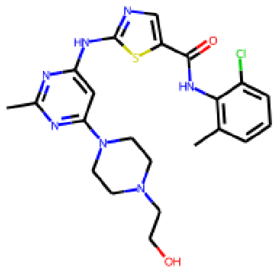	0.926684	Cc1nc(Nc2ncc(C(=O)Nc3c(C)cccc3Cl)s2)cc(N2CCNC(=O)C2)n1	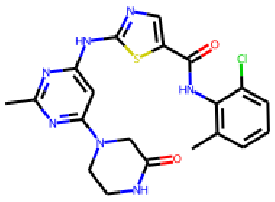	6.0	0.593231	3.250153	2.680275	4	0.755833
**11**	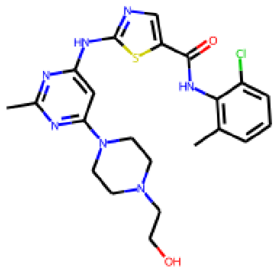	0.926684	Cc1nc(Nc2ncc(C(=O)Nc3c(C)cccc3Cl)s2)cc(N2CCNC(=O)C2)n1	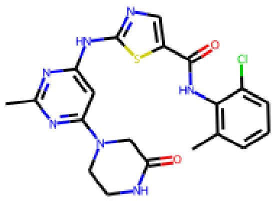	6.0	0.593231	3.250153	2.680275	4	0.755833

	src known mol	similarity	smiles	mol	Class	qed	logP	SAS	rings	kinase inhibition likeliness
**12**	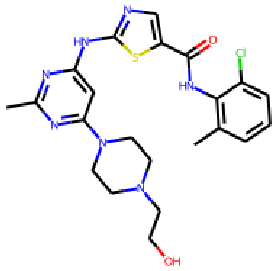	0.926684	Cc1nc(Nc2ncc(C(=O)Nc3c(C)cccc3Cl)s2)cc(N2CCNC(=O)C2)n1	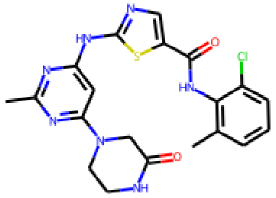	6.0	0.593231	3.250153	2.680275	4	0.755833
**13**	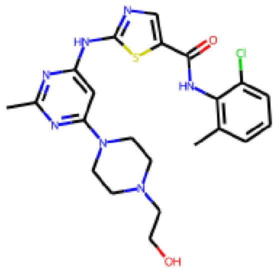	0.926684	Cc1nc(Nc2ncc(C(=O)Nc3c(C)cccc3Cl)s2)cc(N2CCNC(=O)C2)n1	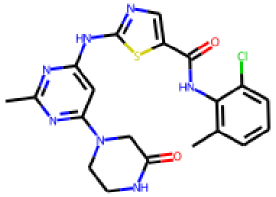	6.0	0.593231	3.250153	2.680275	4	0.755833
**14**	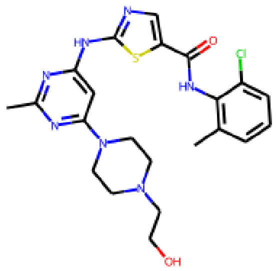	0.926684	Cc1nc(Nc2ncc(C(=O)Nc3c(C)cccc3Cl)s2)cc(N2CCNC(=O)C2)n1	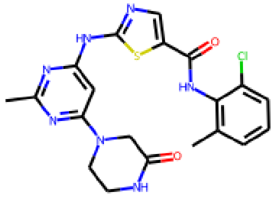	6.0	0.593231	3.250153	2.680275	4	0.755833
**15**	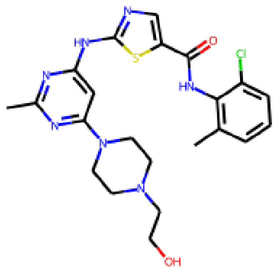	0.926684	Cc1nc(Nc2ncc(C(=O)Nc3c(C)cccc3Cl)s2)cc(N2CCNC(=O)C2)n1	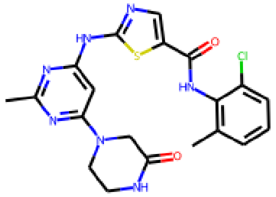	6.0	0.593231	3.250153	2.680275	4	0.755833

	src known mol	similarity	smiles	mol	Class	qed	logP	SAS	rings	kinase inhibition likeliness
**16**	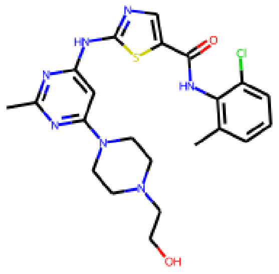	0.926684	Cc1nc(Nc2ncc(C(=O)Nc3c(C)cccc3Cl)s2)cc(N2CCNC(=O)C2)n1	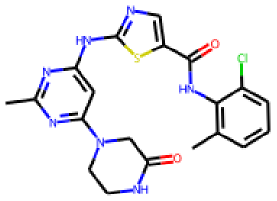	6.0	0.593231	3.250153	2.680275	4	0.755833
**17**	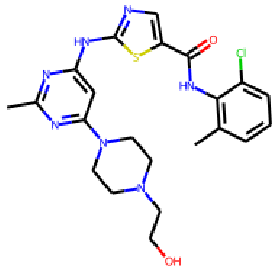	0.926684	Cc1nc(Nc2ncc(C(=O)Nc3c(C)cccc3Cl)s2)cc(N2CCNC(=O)C2)n1	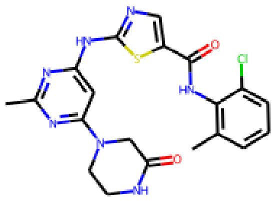	6.0	0.593231	3.250153	2.680275	4	0.755833
**18**	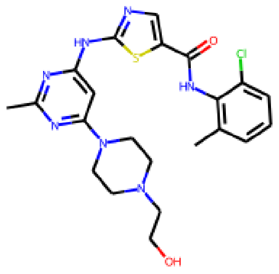	0.926684	Cc1nc(Nc2ncc(C(=O)Nc3c(C)cccc3Cl)s2)cc(N2CCNC(=O)C2)n1	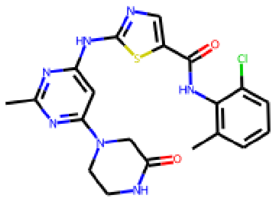	6.0	0.593231	3.250153	2.680275	4	0.755833
**19**	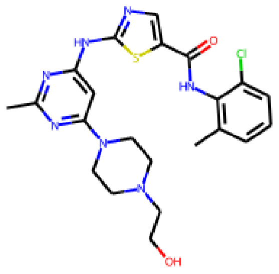	0.926684	Cc1nc(Nc2ncc(C(=O)Nc3c(C)cccc3Cl)s2)cc(N2CCNC(=O)C2)n1	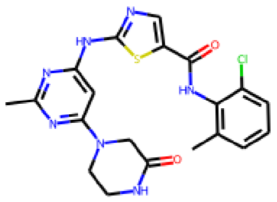	6.0	0.593231	3.250153	2.680275	4	0.755833

	src known mol	similarity	smiles	mol	Class	qed	logP	SAS	rings	kinase inhibition likeliness
**20**	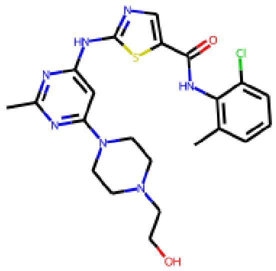	0.926684	Cc1nc(Nc2ncc(C(=O)Nc3c(C)cccc3Cl)s2)cc(N2CCNC(=O)C2)n1	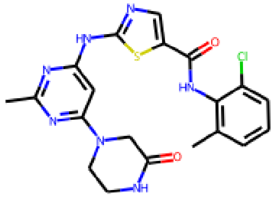	6.0	0.593231	3.250153	2.680275	4	0.755833
**21**	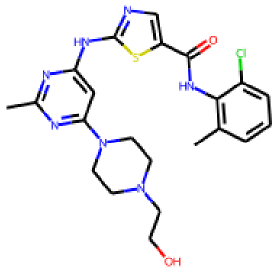	0.926684	Cc1nc(Nc2ncc(C(=O)Nc3c(C)cccc3Cl)s2)cc(N2CCNC(=O)C2)n1	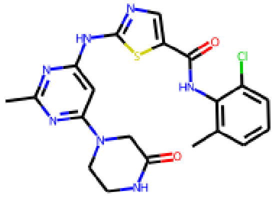	6.0	0.593231	3.250153	2.680275	4	0.755833
**22**	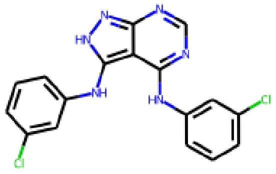	0.925872	Clc1cccc(Nc2ncnc3n[nH]c(Nc4cccc(Cl)c4)c23)c1	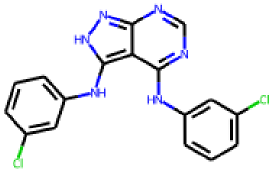	0.0	0.768132	3.608437	2.513170	4	0.771417
**23**	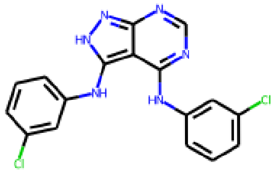	0.925776	Clc1cccc(Nc2ncnc3n[nH]c(Nc4cccc(Cl)c4)c23)c1	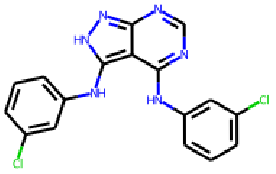	0.0	0.768132	3.608437	2.513170	4	0.771417

	src known mol	similarity	smiles	mol	Class	qed	logP	SAS	rings	kinase inhibition likeliness
**24**	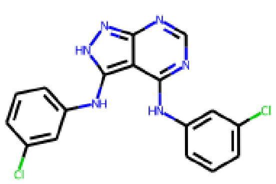	0.923490	Clc1cccc(Nc2ncnc3n[nH]c(Nc4cccc(Cl)c4)c23)c1	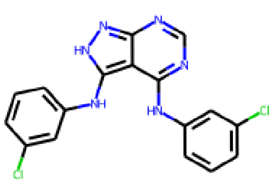	0.0	0.768132	3.608437	2.513170	4	0.771417
**25**	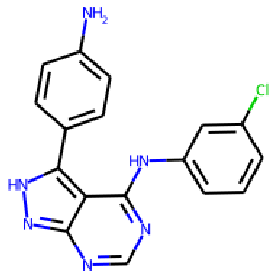	0.916115	c1ccc(Nc2ncnc3n[nH]c(-c4ccccc4)c23)cc1	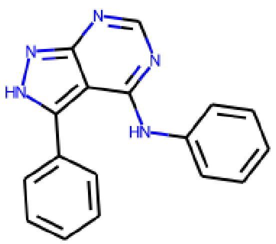	0.0	0.638163	3.995589	2.988471	4	0.800634
**26**	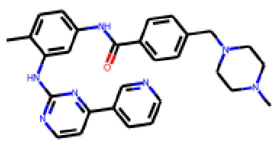	0.899083	Cc1ccc(NC(=O)c2ccccc2)cc1Nc1nccc(-c2cccnc2)n1	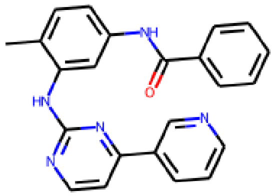	0.0	0.768132	3.608437	2.513170	4	0.854555
**27**	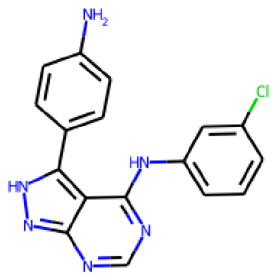	0.893434	c1ccc(Nc2ncnc3n[nH]c(-c4ccccc4)c23)cc1	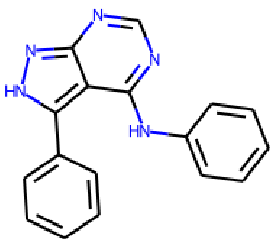	0.0	0.638163	3.995589	2.988471	4	0.800634

	src known mol	similarity	smiles	mol	Class	qed	logP	SAS	rings	kinase inhibition likeliness
**28**	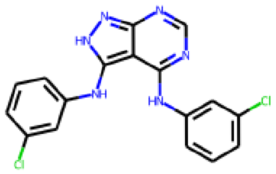	0.888601	Clc1cccc(Nc2ncnc3n[nH]c(Nc4cccc(Cl)c4)c23)c1	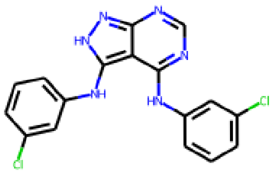	0.0	0.768132	3.608437	2.513170	4	0.771417
**29**	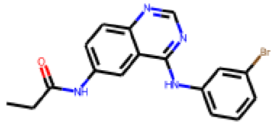	0.850490	O=C(COC(=O)Nc1ccccc1)Nc1ccc2ncnc(Nc3cccc(Br)c3)c2c1	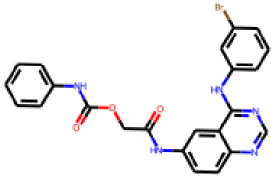	3.0	0.635315	4.542409	3.856266	4	0.784670
**30**	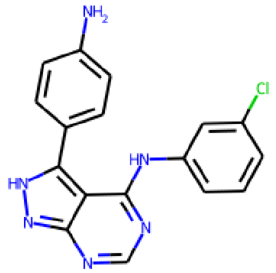	0.847804	c1ccc(Nc2ncnc3n[nH]c(-c4ccccc4)c23)cc1	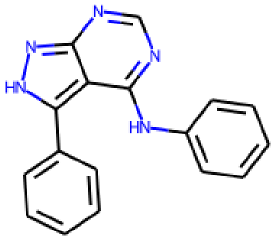	0.0	0.638163	3.995589	2.988471	4	0.800634
**31**	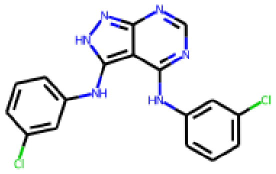	0.847682	Clc1cccc(Nc2ncnc3n[nH]c(Nc4cccc(Cl)c4)c23)c1	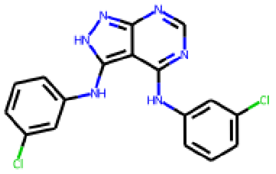	0.0	0.768132	3.608437	2.513170	4	0.771417

	src known mol	similarity	smiles	mol	Class	qed	logP	SAS	rings	kinase inhibition likeliness
**32**	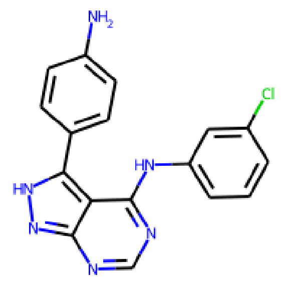	0.831663	c1ccc(Nc2ncnc3n[nH]c(-c4ccccc4)c23)cc1	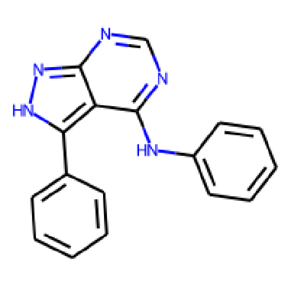	0.0	0.638163	3.995589	2.988471	4	0.800634
**33**	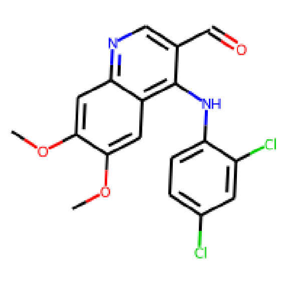	0.827846	COc1cc2ncc(C(=O)O)c(Nc3cccc(Cl)c3Cl)c2cc1OC1C(C)C1	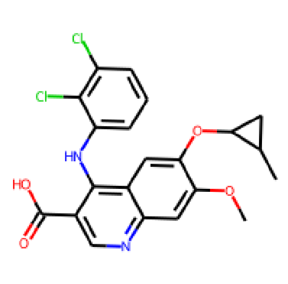	2.0	0.623362	2.525411	2.997881	4	0.774857
**34**	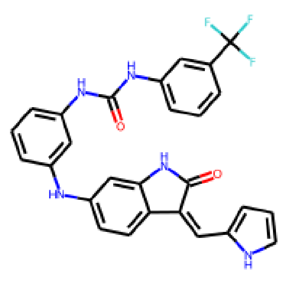	0.827220	COc1ccc(NC(=O)Nc2ccc3c(c2)NC(=O)/C3=C/c2ccc[nH]2)cc1	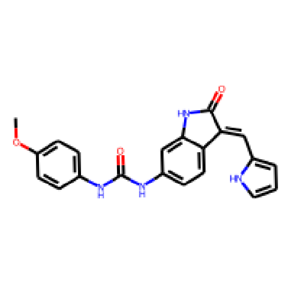	4.0	0.638163	3.995589	2.988471	4	0.768944
**35**	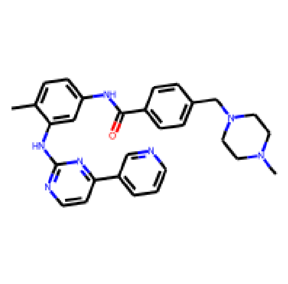	0.817661	Cc1ccc(NC(=O)c2ccccc2)cc1Nc1nccc(-c2cccnc2)n1	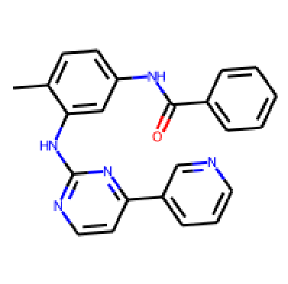	0.0	0.768132	3.608437	2.513170	4	0.854555

	src known mol	similarity	smiles	mol	Class	qed	logP	SAS	rings	kinase inhibition likeliness
**36**	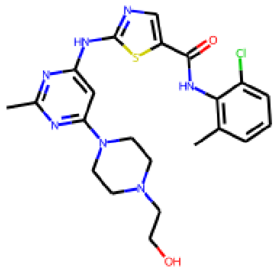	0.809193	Cc1nc(Nc2ncc(C(=O)Nc3c(C)cccc3Cl)s2)cc(N2CC(COC)C2)n1	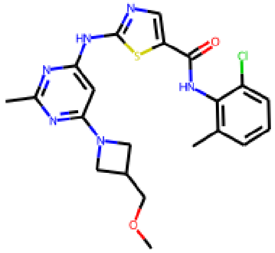	6.0	0.623362	2.525411	2.997881	4	0.837661
**37**	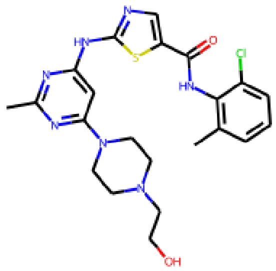	0.809193	Cc1nc(Nc2ncc(C(=O)Nc3c(C)cccc3Cl)s2)cc(N2CC(COC)C2)n1	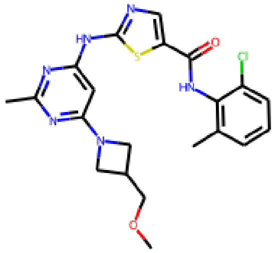	6.0	0.623362	2.525411	2.997881	4	0.837661
**38**	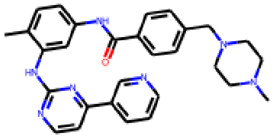	0.789897	Cc1ccc(NC(=O)c2ccccc2)cc1Nc1nccc(-c2cccnc2)n1	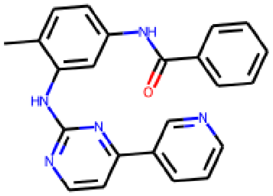	0.0	0.768132	3.608437	2.513170	4	0.854555
**39**	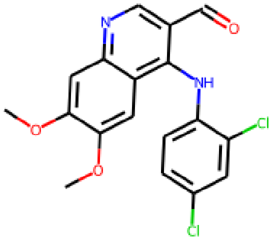	0.782765	COc1cc2ncc(C(=O)O)c(Nc3cccc(Cl)c3Cl)c2cc1OC1C(C)C1	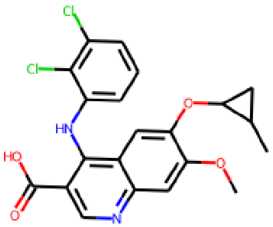	2.0	0.623362	2.525411	2.997881	4	0.774857

	src known mol	similarity	smiles	mol	Class	qed	logP	SAS	rings	kinase inhibition likeliness
**40**	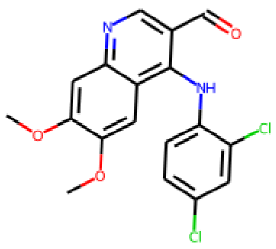	0.779197	COc1cc2ncc(C(=O)O)c(Nc3cccc(Cl)c3Cl)c2cc1OC1C(C)C1	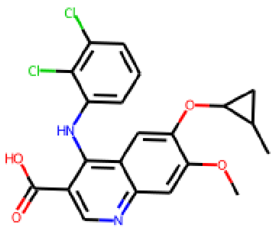	2.0	0.623362	2.525411	2.997881	4	0.774857
**41**	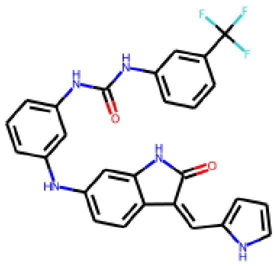	0.772039	COc1ccc(NC(=O)Nc2ccc3c(c2)NC(=O)/C3=C/c2ccc[nH]2)cc1	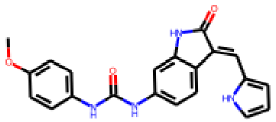	4.0	0.638163	3.995589	2.988471	4	0.768944
**42**	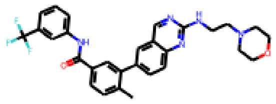	0.771249	Cc1ccc(C(=O)Nc2cccc(C(F)(F)F)c2)cc1-c1ccc2nc(N)ccc2c1	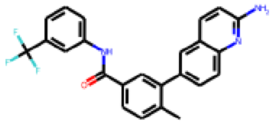	6.0	0.635315	4.542409	3.856266	4	0.833561
**43**	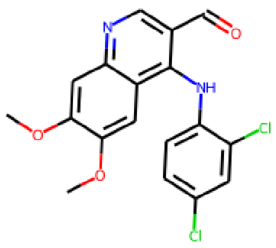	0.767764	COc1cc2ncc(C(=O)O)c(Nc3cccc(Cl)c3Cl)c2cc1OC1C(C)C1	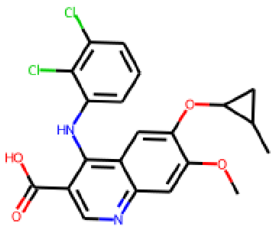	2.0	0.623362	2.525411	2.997881	4	0.774857

	src known mol	similarity	smiles	mol	Class	qed	logP	SAS	rings	kinase inhibition likeliness
**44**	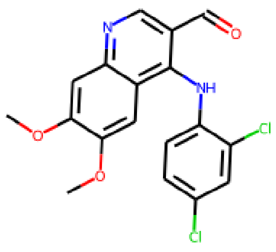	0.767764	COc1cc2ncc(C(=O)O)c(Nc3cccc(Cl)c3Cl)c2cc1OC1C(C)C1	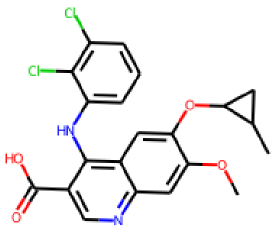	2.0	0.623362	2.525411	2.997881	4	0.774857
**45**	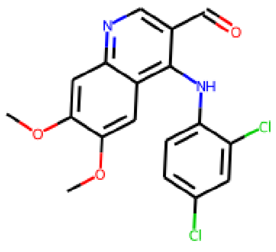	0.765499	COc1cc2ncc(C(=O)O)c(Nc3cccc(Cl)c3Cl)c2cc1OC1C(C)C1	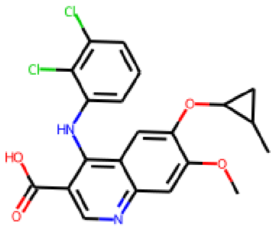	2.0	0.623362	2.525411	2.997881	4	0.774857
**46**	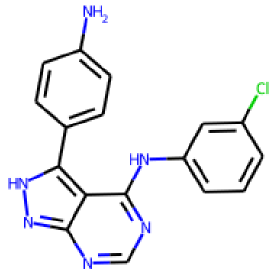	0.760694	c1ccc(Nc2ncnc3n[nH]c(-c4ccccc4)c23)cc1	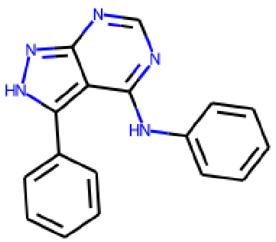	0.0	0.638163	3.995589	2.988471	4	0.800634
**47**	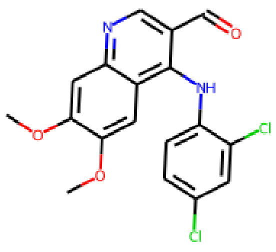	0.759757	COc1cc2ncc(C(=O)O)c(Nc3cccc(Cl)c3Cl)c2cc1OC1C(C)C1	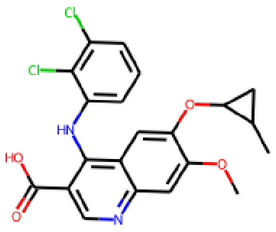	2.0	0.623362	2.525411	2.997881	4	0.774857

	src known mol	similarity	smiles	mol	Class	qed	logP	SAS	rings	kinase inhibition likeliness
**48**	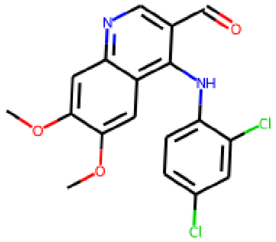	0.759757	COc1cc2ncc(C(=O)O)c(Nc3cccc(Cl)c3Cl)c2cc1OC1C(C)C1	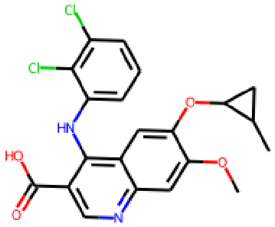	2.0	0.623362	2.525411	2.997881	4	0.774857
**49**	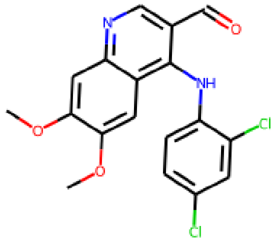	0.759079	COc1cc2ncc(C(=O)O)c(Nc3cccc(Cl)c3Cl)c2cc1OC1C(C)C1	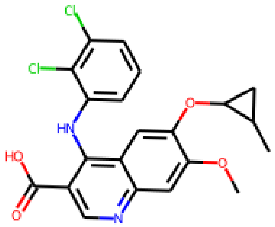	2.0	0.623362	2.525411	2.997881	4	0.774857
**50**	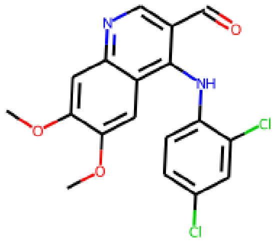	0.756806	COc1cc2ncc(C(=O)O)c(Nc3cccc(Cl)c3Cl)c2cc1OC1C(C)C1	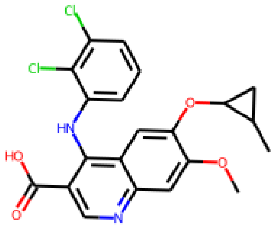	2.0	0.623362	2.525411	2.997881	4	0.774857
**51**	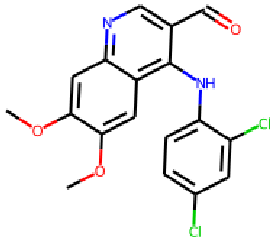	0.756422	COc1cc2ncc(C(=O)O)c(Nc3cccc(Cl)c3Cl)c2cc1OC1C(C)C1	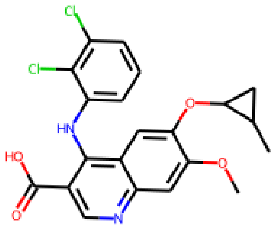	2.0	0.623362	2.525411	2.997881	4	0.774857

	src known mol	similarity	smiles	mol	Class	qed	logP	SAS	rings	kinase inhibition likeliness
**52**	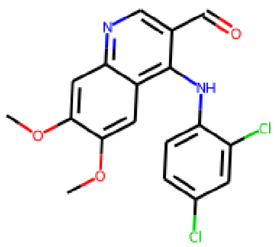	0.755402	COc1cc2ncc(C(=O)O)c(Nc3cccc(Cl)c3Cl)c2cc1OC1C(C)C1	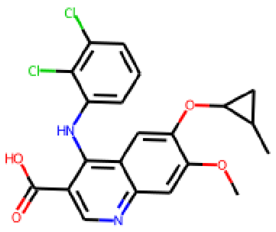	2.0	0.623362	2.525411	2.997881	4	0.774857
**53**	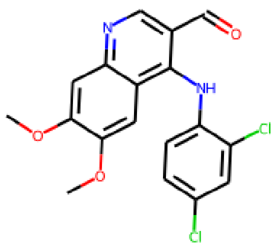	0.755402	COc1cc2ncc(C(=O)O)c(Nc3cccc(Cl)c3Cl)c2cc1OC1C(C)C1	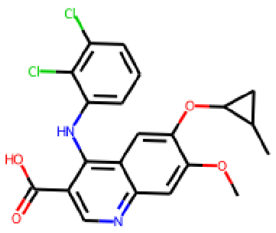	2.0	0.623362	2.525411	2.997881	4	0.774857
**54**	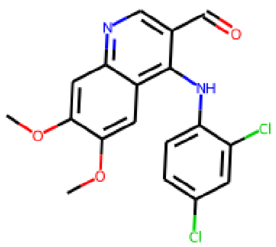	0.755402	COc1cc2ncc(C(=O)O)c(Nc3cccc(Cl)c3Cl)c2cc1OC1C(C)C1	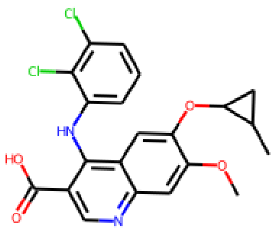	2.0	0.623362	2.525411	2.997881	4	0.774857
**55**	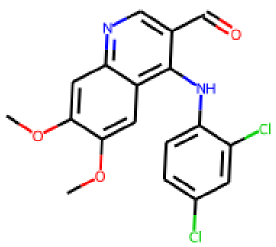	0.754113	COc1cc2ncc(C(=O)O)c(Nc3cccc(Cl)c3Cl)c2cc1OC1C(C)C1	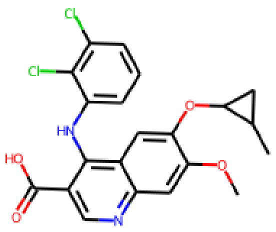	2.0	0.623362	2.525411	2.997881	4	0.774857

	src known mol	similarity	smiles	mol	Class	qed	logP	SAS	rings	kinase inhibition likeliness
**56**	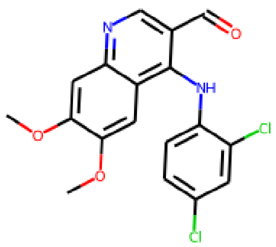	0.752586	COc1cc2ncc(C(=O)O)c(Nc3cccc(Cl)c3Cl)c2cc1OC1C(C)C1	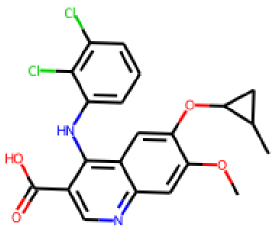	2.0	0.623362	2.525411	2.997881	4	0.774857
**57**	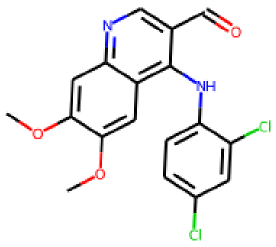	0.752586	COc1cc2ncc(C(=O)O)c(Nc3cccc(Cl)c3Cl)c2cc1OC1C(C)C1	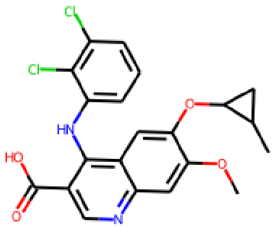	2.0	0.623362	2.525411	2.997881	4	0.774857
**58**	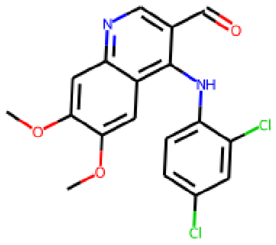	0.750000	COc1cc2ncc(C(=O)O)c(Nc3cccc(Cl)c3Cl)c2cc1OC1C(C)C1	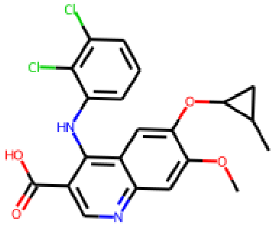	2.0	0.623362	2.525411	2.997881	4	0.774857
**59**	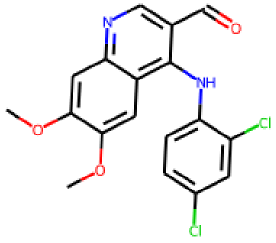	0.750000	COc1cc2ncc(C(=O)O)c(Nc3cccc(Cl)c3Cl)c2cc1OC1C(C)C1	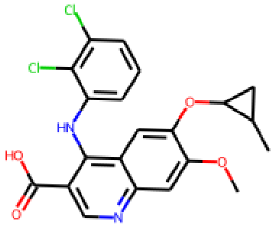	2.0	0.623362	2.525411	2.997881	4	0.774857

	src known mol	similarity	smiles	mol	Class	qed	logP	SAS	rings	kinase inhibition likeliness
**60**	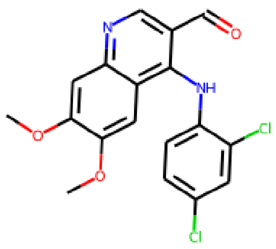	0.750000	COc1cc2ncc(C(=O)O)c(Nc3cccc(Cl)c3Cl)c2cc1OC1C(C)C1	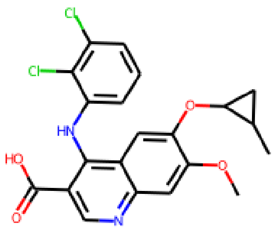	2.0	0.623362	2.525411	2.997881	4	0.774857
**61**	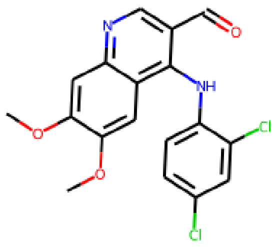	0.748305	COc1cc2ncc(C(=O)O)c(Nc3cccc(Cl)c3Cl)c2cc1OC1C(C)C1	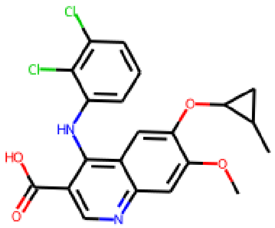	2.0	0.623362	2.525411	2.997881	4	0.774857
**62**	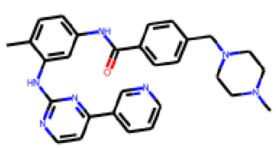	0.746622	Cc1ccc(NC(=O)c2ccccc2)cc1Nc1nccc(-c2cccnc2)n1	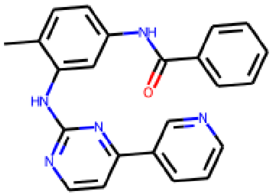	0.0	0.768132	3.608437	2.513170	4	0.854555
**63**	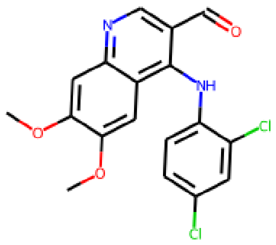	0.745717	COc1cc2ncc(C(=O)O)c(Nc3cccc(Cl)c3Cl)c2cc1OC1C(C)C1	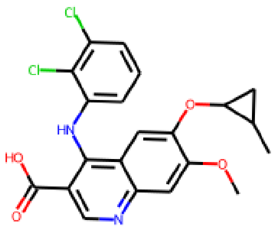	2.0	0.623362	2.525411	2.997881	4	0.774857

	src known mol	similarity	smiles	mol	Class	qed	logP	SAS	rings	kinase inhibition likeliness
**64**	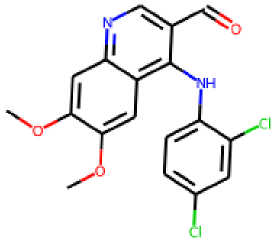	0.745717	COc1cc2ncc(C(=O)O)c(Nc3cccc(Cl)c3Cl)c2cc1OC1C(C)C1	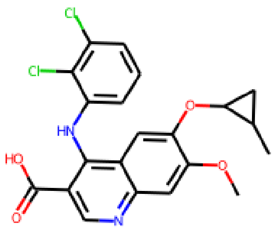	2.0	0.623362	2.525411	2.997881	4	0.774857
**65**	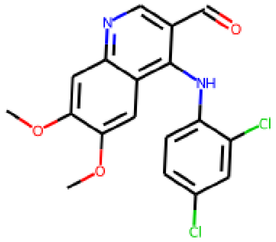	0.745717	COc1cc2ncc(C(=O)O)c(Nc3cccc(Cl)c3Cl)c2cc1OC1C(C)C1	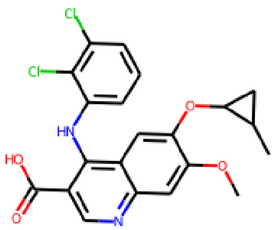	2.0	0.623362	2.525411	2.997881	4	0.774857
**66**	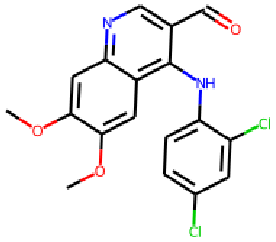	0.745717	COc1cc2ncc(C(=O)O)c(Nc3cccc(Cl)c3Cl)c2cc1OC1C(C)C1	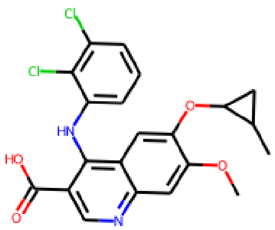	2.0	0.623362	2.525411	2.997881	4	0.774857
**67**	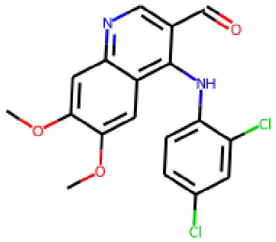	0.745717	COc1cc2ncc(C(=O)O)c(Nc3cccc(Cl)c3Cl)c2cc1OC1C(C)C1	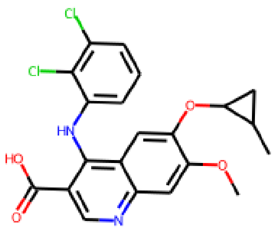	2.0	0.623362	2.525411	2.997881	4	0.774857

	src known mol	similarity	smiles	mol	Class	qed	logP	SAS	rings	kinase inhibition likeliness
**68**	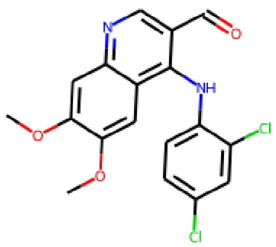	0.745717	COc1cc2ncc(C(=O)O)c(Nc3cccc(Cl)c3Cl)c2cc1OC1C(C)C1	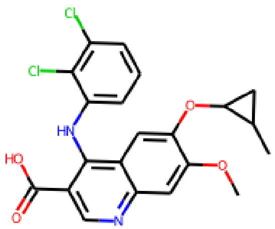	2.0	0.623362	2.525411	2.997881	4	0.774857
**69**	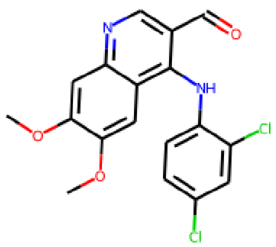	0.745717	COc1cc2ncc(C(=O)O)c(Nc3cccc(Cl)c3Cl)c2cc1OC1C(C)C1	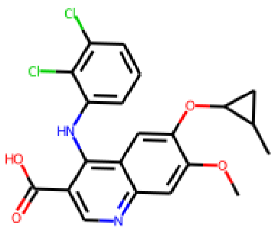	2.0	0.623362	2.525411	2.997881	4	0.774857
**70**	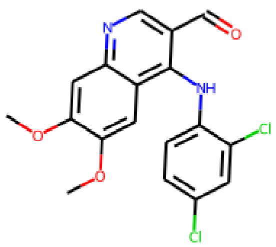	0.745717	COc1cc2ncc(C(=O)O)c(Nc3cccc(Cl)c3Cl)c2cc1OC1C(C)C1	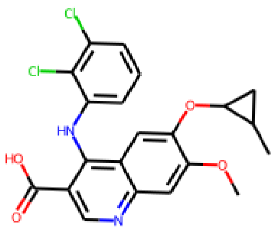	2.0	0.623362	2.525411	2.997881	4	0.774857
**71**	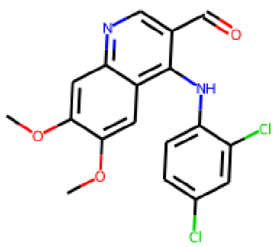	0.745438	COc1cc2ncc(C(=O)O)c(Nc3cccc(Cl)c3Cl)c2cc1OC1C(C)C1	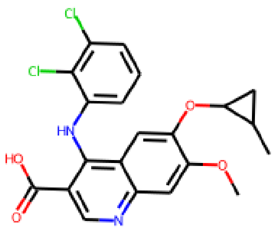	2.0	0.623362	2.525411	2.997881	4	0.774857

	src known mol	similarity	smiles	mol	Class	qed	logP	SAS	rings	kinase inhibition likeliness
**72**	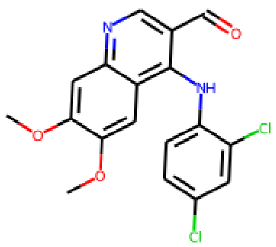	0.743682	COc1cc2ncc(C(=O)O)c(Nc3cccc(Cl)c3Cl)c2cc1OC1C(C)C1	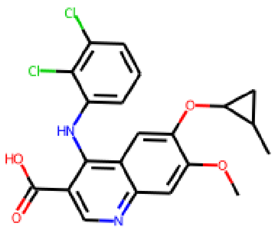	2.0	0.623362	2.525411	2.997881	4	0.774857
**73**	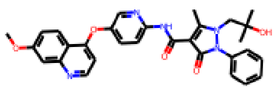	0.742120	COc1ccc2c(=O)cc(CNC(=O)c3c(C)n(C)n(-c4ccccc4)c3=O)nc3cccc3c2n1	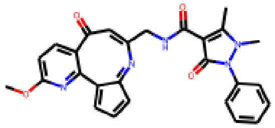	9.0	0.768132	3.608437	2.513170	5	0.823048
**74**	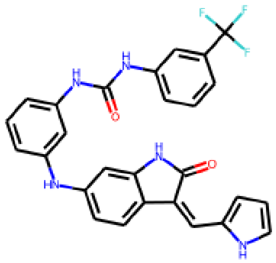	0.741302	COc1ccc(NC(=O)Nc2ccc3c(c2)NC(=O)/C3=C/c2ccc[nH]2)cc1	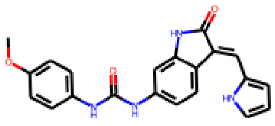	4.0	0.638163	3.995589	2.988471	4	0.768944
**75**	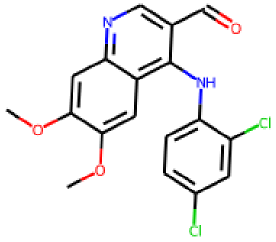	0.739703	COc1cc2ncc(C(=O)O)c(Nc3cccc(Cl)c3Cl)c2cc1OC1C(C)C1	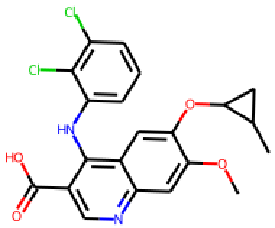	2.0	0.623362	2.525411	2.997881	4	0.774857

	src known mol	similarity	smiles	mol	Class	qed	logP	SAS	rings	kinase inhibition likeliness
**76**	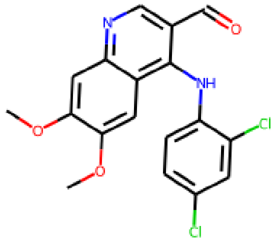	0.738233	COc1cc2ncc(C(=O)O)c(Nc3cccc(Cl)c3Cl)c2cc1OC1C(C)C1	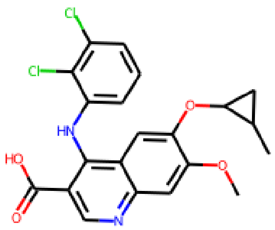	2.0	0.623362	2.525411	2.997881	4	0.774857
**77**	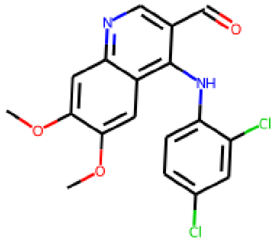	0.738233	COc1cc2ncc(C(=O)O)c(Nc3cccc(Cl)c3Cl)c2cc1OC1C(C)C1	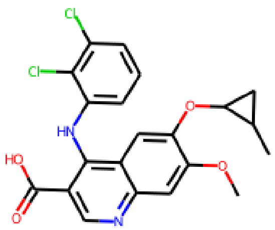	2.0	0.623362	2.525411	2.997881	4	0.774857
**78**	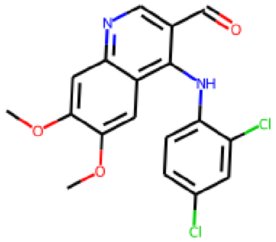	0.737081	COc1cc2ncc(C(=O)O)c(Nc3cccc(Cl)c3Cl)c2cc1OC1C(C)C1	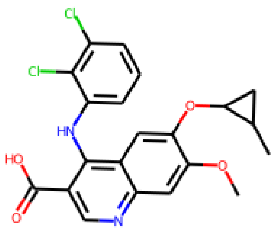	2.0	0.623362	2.525411	2.997881	4	0.774857
**79**	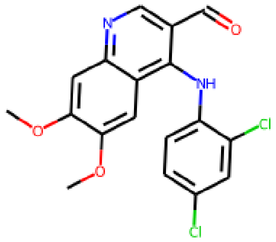	0.734426	COc1cc2ncc(C(=O)O)c(Nc3cccc(Cl)c3Cl)c2cc1OC1C(C)C1	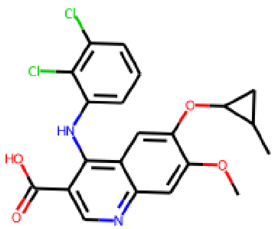	2.0	0.623362	2.525411	2.997881	4	0.774857

	src known mol	similarity	smiles	mol	Class	qed	logP	SAS	rings	kinase inhibition likeliness
**80**	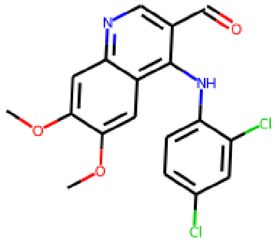	0.733392	COc1cc2ncc(C(=O)O)c(Nc3cccc(Cl)c3Cl)c2cc1OC1C(C)C1	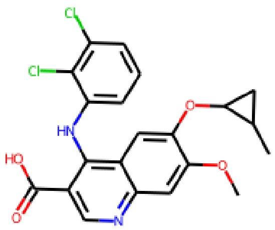	2.0	0.623362	2.525411	2.997881	4	0.774857
**81**	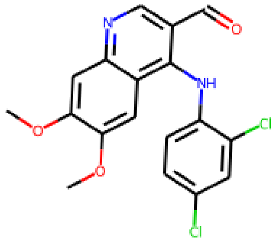	0.732673	COc1cc2ncc(C(=O)O)c(Nc3cccc(Cl)c3Cl)c2cc1OC1C(C)C1	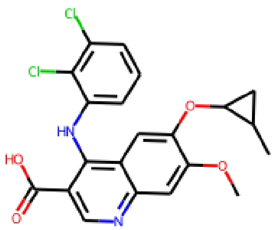	2.0	0.623362	2.525411	2.997881	4	0.774857
**82**	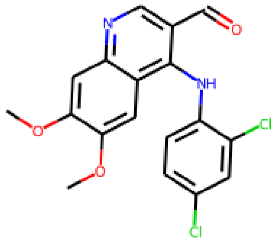	0.732673	COc1cc2ncc(C(=O)O)c(Nc3cccc(Cl)c3Cl)c2cc1OC1C(C)C1	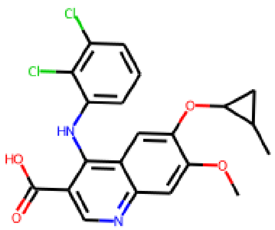	2.0	0.623362	2.525411	2.997881	4	0.774857
**83**	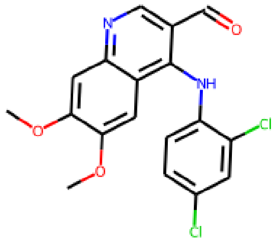	0.732010	COc1cc2ncc(C(=O)O)c(Nc3cccc(Cl)c3Cl)c2cc1OC1C(C)C1	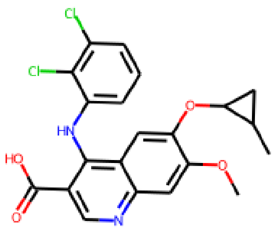	2.0	0.623362	2.525411	2.997881	4	0.774857

	src known mol	similarity	smiles	mol	Class	qed	logP	SAS	rings	kinase inhibition likeliness
**84**	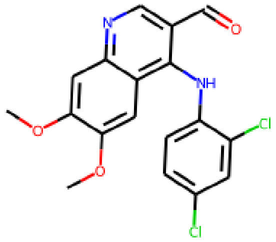	0.732010	COc1cc2ncc(C(=O)O)c(Nc3cccc(Cl)c3Cl)c2cc1OC1C(C)C1	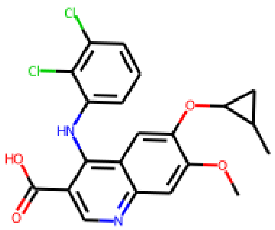	2.0	0.623362	2.525411	2.997881	4	0.774857
**85**	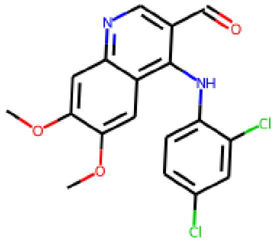	0.731405	COc1cc2ncc(C(=O)O)c(Nc3cccc(Cl)c3Cl)c2cc1OC1C(C)C1	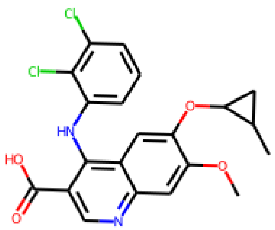	2.0	0.623362	2.525411	2.997881	4	0.774857
**86**	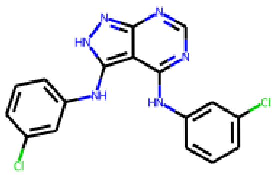	0.730464	Clc1cccc(Nc2ncnc3n[nH]c(Nc4cccc(Cl)c4)c23)c1	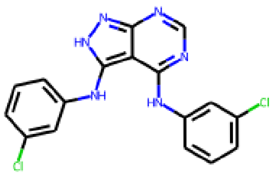	0.0	0.768132	3.608437	2.513170	4	0.771417
**87**	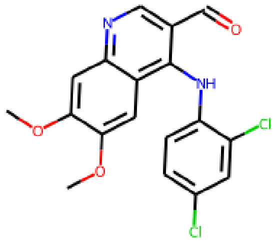	0.730413	COc1cc2ncc(C(=O)O)c(Nc3cccc(Cl)c3Cl)c2cc1OC1C(C)C1	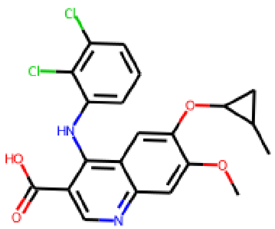	2.0	0.623362	2.525411	2.997881	4	0.774857

	src known mol	similarity	smiles	mol	Class	qed	logP	SAS	rings	kinase inhibition likeliness
**88**	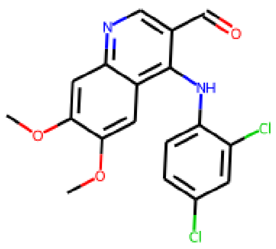	0.730396	COc1cc2ncc(C(=O)O)c(Nc3cccc(Cl)c3Cl)c2cc1OC1C(C)C1	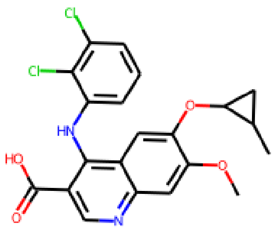	2.0	0.623362	2.525411	2.997881	4	0.774857
**89**	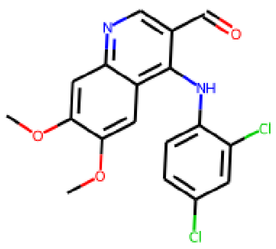	0.729570	COc1cc2ncc(C(=O)O)c(Nc3cccc(Cl)c3Cl)c2cc1OC1C(C)C1	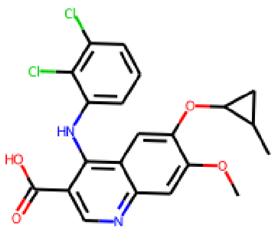	2.0	0.623362	2.525411	2.997881	4	0.774857
**90**	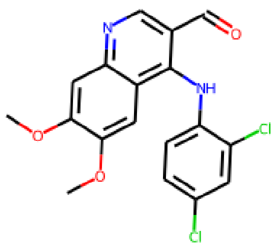	0.729461	COc1cc2ncc(C(=O)O)c(Nc3cccc(Cl)c3Cl)c2cc1OC1C(C)C1	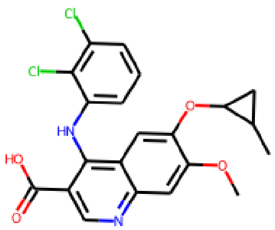	2.0	0.623362	2.525411	2.997881	4	0.774857
**91**	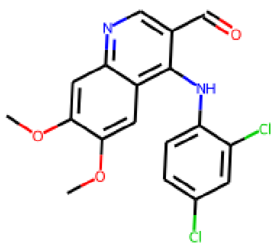	0.728926	COc1cc2ncc(C(=O)O)c(Nc3cccc(Cl)c3Cl)c2cc1OC1C(C)C1	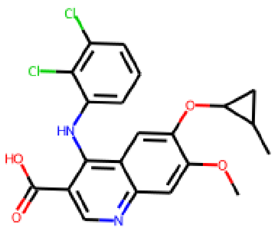	2.0	0.623362	2.525411	2.997881	4	0.774857

	src known mol	similarity	smiles	mol	Class	qed	logP	SAS	rings	kinase inhibition likeliness
**92**	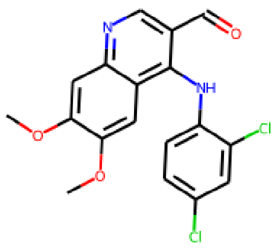	0.727796	COc1cc2ncc(C(=O)O)c(Nc3cccc(Cl)c3Cl)c2cc1OC1C(C)C1	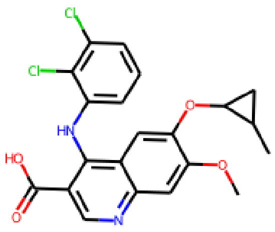	2.0	0.623362	2.525411	2.997881	4	0.774857
**93**	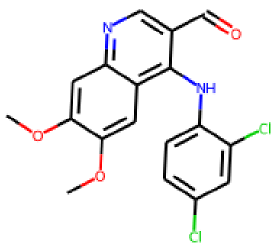	0.727043	COc1cc2ncc(C(=O)O)c(Nc3cccc(Cl)c3Cl)c2cc1OC1C(C)C1	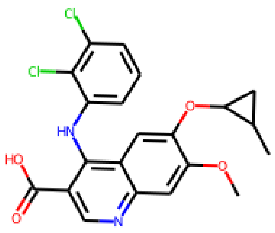	2.0	0.623362	2.525411	2.997881	4	0.774857
**94**	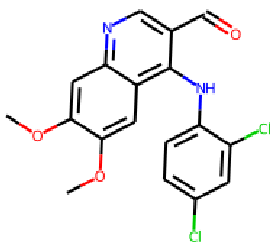	0.726749	COc1cc2ncc(C(=O)O)c(Nc3cccc(Cl)c3Cl)c2cc1OC1C(C)C1	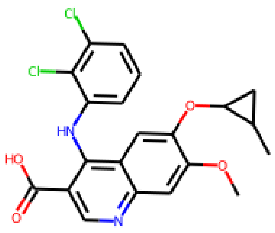	2.0	0.623362	2.525411	2.997881	4	0.774857
**95**	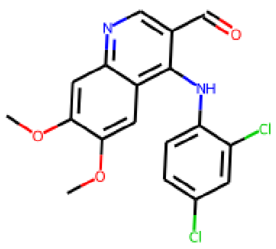	0.726749	COc1cc2ncc(C(=O)O)c(Nc3cccc(Cl)c3Cl)c2cc1OC1C(C)C1	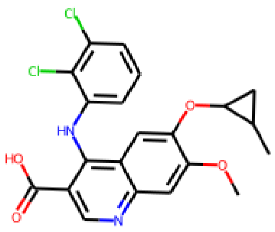	2.0	0.623362	2.525411	2.997881	4	0.774857

	src known mol	similarity	smiles	mol	Class	qed	logP	SAS	rings	kinase inhibition likeliness
**96**	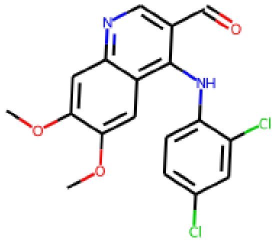	0.726749	COc1cc2ncc(C(=O)O)c(Nc3cccc(Cl)c3Cl)c2cc1OC1C(C)C1	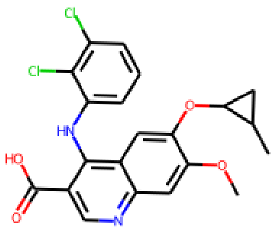	2.0	0.623362	2.525411	2.997881	4	0.774857
**97**	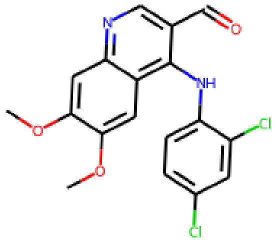	0.726749	COc1cc2ncc(C(=O)O)c(Nc3cccc(Cl)c3Cl)c2cc1OC1C(C)C1	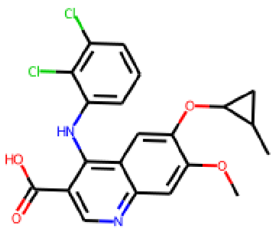	2.0	0.623362	2.525411	2.997881	4	0.774857
**98**	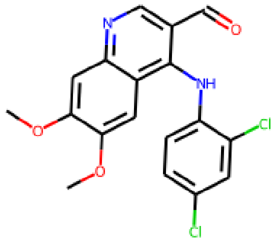	0.726749	COc1cc2ncc(C(=O)O)c(Nc3cccc(Cl)c3Cl)c2cc1OC1C(C)C1	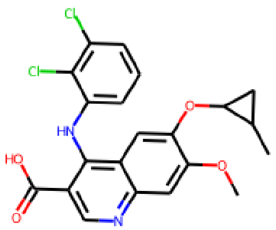	2.0	0.623362	2.525411	2.997881	4	0.774857
**99**	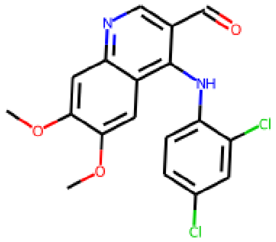	0.726749	COc1cc2ncc(C(=O)O)c(Nc3cccc(Cl)c3Cl)c2cc1OC1C(C)C1	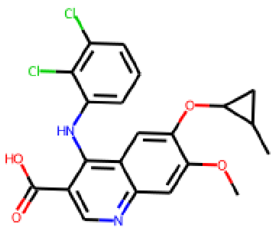	2.0	0.623362	2.525411	2.997881	4	0.774857

	src known mol	similarity	smiles	mol	Class	qed	logP	SAS	rings	kinase inhibition likeliness
**100**	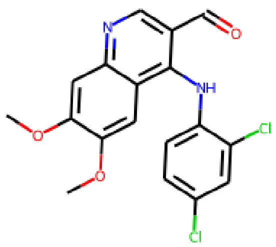	0.726749	COc1cc2ncc(C(=O)O)c(Nc3cccc(Cl)c3Cl)c2cc1OC1C(C)C1	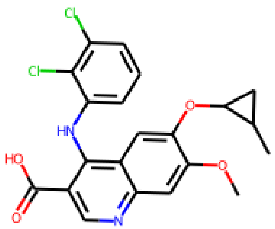	2.0	0.623362	2.525411	2.997881	4	0.774857
**101**	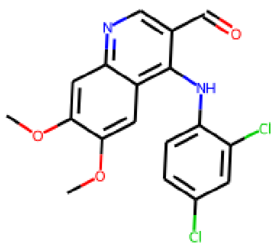	0.726749	COc1cc2ncc(C(=O)O)c(Nc3cccc(Cl)c3Cl)c2cc1OC1C(C)C1	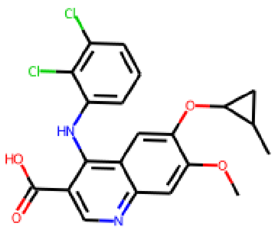	2.0	0.623362	2.525411	2.997881	4	0.774857
**102**	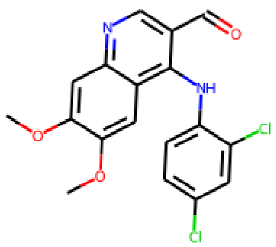	0.726749	COc1cc2ncc(C(=O)O)c(Nc3cccc(Cl)c3Cl)c2cc1OC1C(C)C1	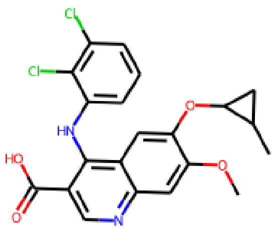	2.0	0.623362	2.525411	2.997881	4	0.774857
**103**	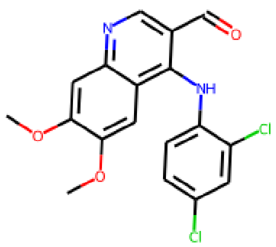	0.726749	COc1cc2ncc(C(=O)O)c(Nc3cccc(Cl)c3Cl)c2cc1OC1C(C)C1	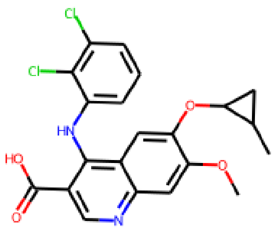	2.0	0.623362	2.525411	2.997881	4	0.774857

	src known mol	similarity	smiles	mol	Class	qed	logP	SAS	rings	kinase inhibition likeliness
**104**	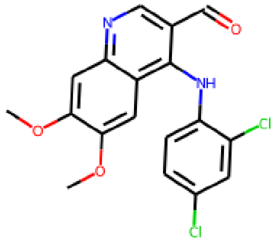	0.726749	COc1cc2ncc(C(=O)O)c(Nc3cccc(Cl)c3Cl)c2cc1OC1C(C)C1	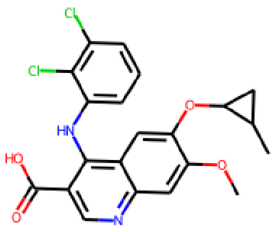	2.0	0.623362	2.525411	2.997881	4	0.774857
**105**	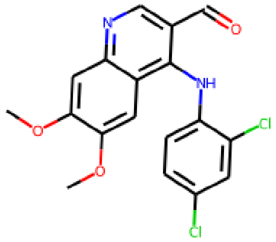	0.726749	COc1cc2ncc(C(=O)O)c(Nc3cccc(Cl)c3Cl)c2cc1OC1C(C)C1	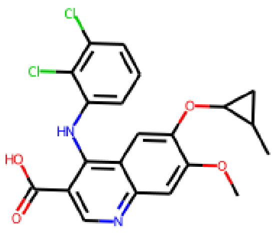	2.0	0.623362	2.525411	2.997881	4	0.774857
**106**	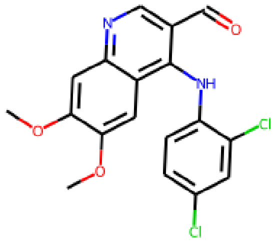	0.726749	COc1cc2ncc(C(=O)O)c(Nc3cccc(Cl)c3Cl)c2cc1OC1C(C)C1	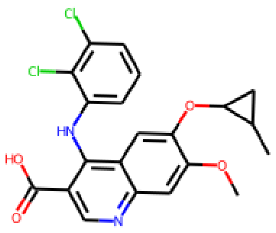	2.0	0.623362	2.525411	2.997881	4	0.774857
**107**	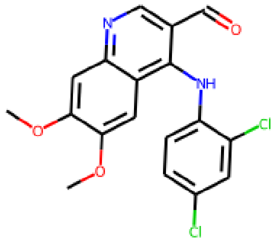	0.726749	COc1cc2ncc(C(=O)O)c(Nc3cccc(Cl)c3Cl)c2cc1OC1C(C)C1	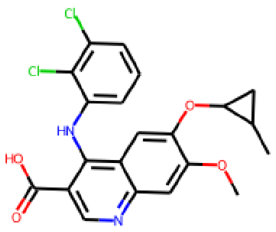	2.0	0.623362	2.525411	2.997881	4	0.774857

	src known mol	similarity	smiles	mol	Class	qed	logP	SAS	rings	kinase inhibition likeliness
**108**	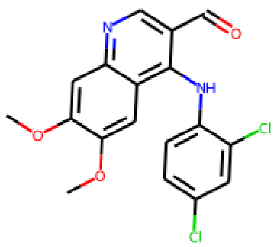	0.726749	COc1cc2ncc(C(=O)O)c(Nc3cccc(Cl)c3Cl)c2cc1OC1C(C)C1	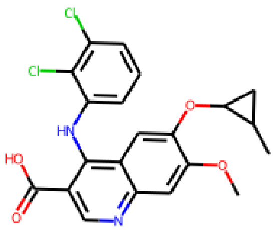	2.0	0.623362	2.525411	2.997881	4	0.774857
**109**	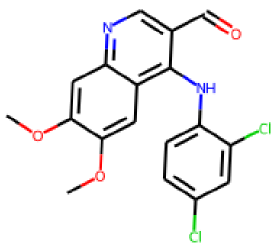	0.726749	COc1cc2ncc(C(=O)O)c(Nc3cccc(Cl)c3Cl)c2cc1OC1C(C)C1	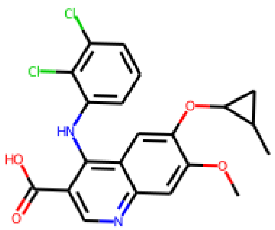	2.0	0.623362	2.525411	2.997881	4	0.774857
**110**	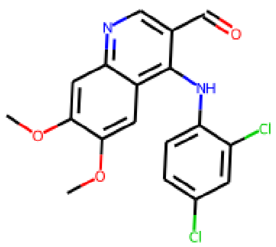	0.726749	COc1cc2ncc(C(=O)O)c(Nc3cccc(Cl)c3Cl)c2cc1OC1C(C)C1	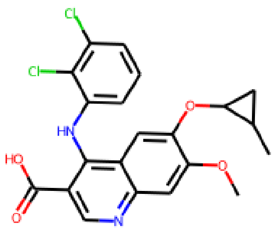	2.0	0.623362	2.525411	2.997881	4	0.774857
**111**	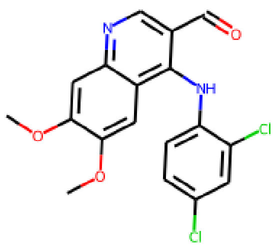	0.726749	COc1cc2ncc(C(=O)O)c(Nc3cccc(Cl)c3Cl)c2cc1OC1C(C)C1	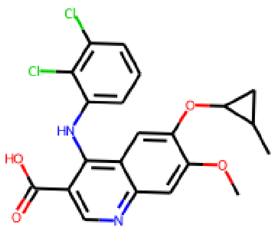	2.0	0.623362	2.525411	2.997881	4	0.774857

	src known mol	similarity	smiles	mol	Class	qed	logP	SAS	rings	kinase inhibition likeliness
**112**	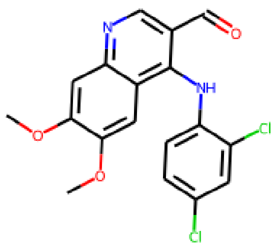	0.726749	COc1cc2ncc(C(=O)O)c(Nc3cccc(Cl)c3Cl)c2cc1OC1C(C)C1	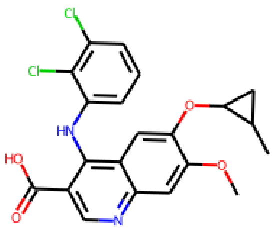	2.0	0.623362	2.525411	2.997881	4	0.774857
**113**	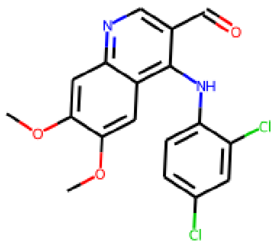	0.726749	COc1cc2ncc(C(=O)O)c(Nc3cccc(Cl)c3Cl)c2cc1OC1C(C)C1	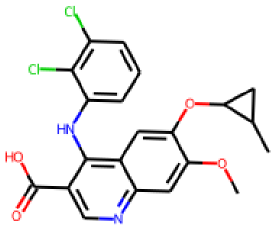	2.0	0.623362	2.525411	2.997881	4	0.774857
**114**	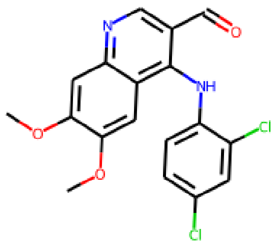	0.726749	COc1cc2ncc(C(=O)O)c(Nc3cccc(Cl)c3Cl)c2cc1OC1C(C)C1	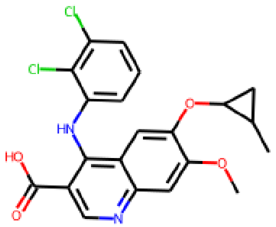	2.0	0.623362	2.525411	2.997881	4	0.774857
**115**	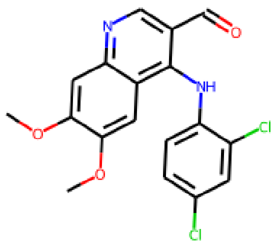	0.726749	COc1cc2ncc(C(=O)O)c(Nc3cccc(Cl)c3Cl)c2cc1OC1C(C)C1	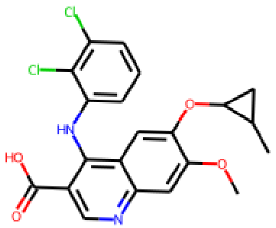	2.0	0.623362	2.525411	2.997881	4	0.774857

	src known mol	similarity	smiles	mol	Class	qed	logP	SAS	rings	kinase inhibition likeliness
**116**	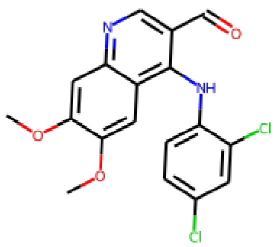	0.726749	COc1cc2ncc(C(=O)O)c(Nc3cccc(Cl)c3Cl)c2cc1OC1C(C)C1	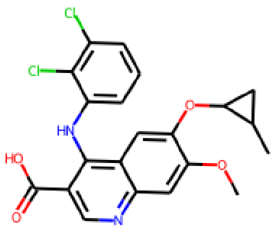	2.0	0.623362	2.525411	2.997881	4	0.774857
**117**	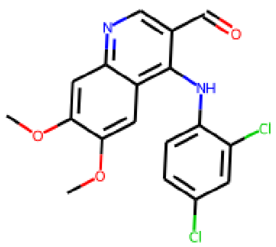	0.726749	COc1cc2ncc(C(=O)O)c(Nc3cccc(Cl)c3Cl)c2cc1OC1C(C)C1	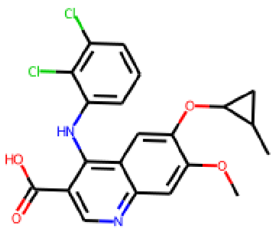	2.0	0.623362	2.525411	2.997881	4	0.774857
**118**	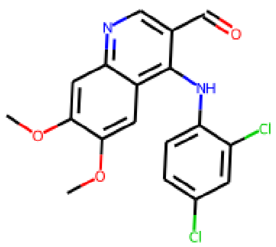	0.726749	COc1cc2ncc(C(=O)O)c(Nc3cccc(Cl)c3Cl)c2cc1OC1C(C)C1	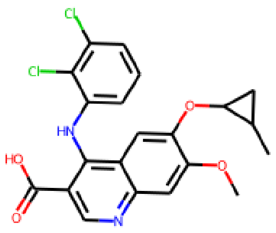	2.0	0.623362	2.525411	2.997881	4	0.774857
**119**	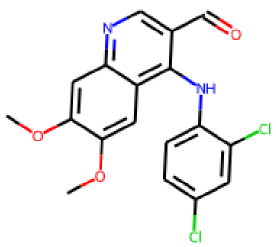	0.726749	COc1cc2ncc(C(=O)O)c(Nc3cccc(Cl)c3Cl)c2cc1OC1C(C)C1	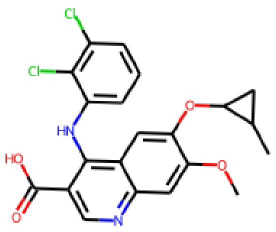	2.0	0.623362	2.525411	2.997881	4	0.774857

	src known mol	similarity	smiles	mol	Class	qed	logP	SAS	rings	kinase inhibition likeliness
**120**	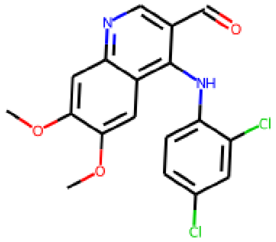	0.726749	COc1cc2ncc(C(=O)O)c(Nc3cccc(Cl)c3Cl)c2cc1OC1C(C)C1	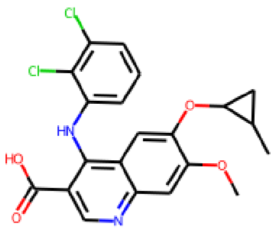	2.0	0.623362	2.525411	2.997881	4	0.774857
**121**	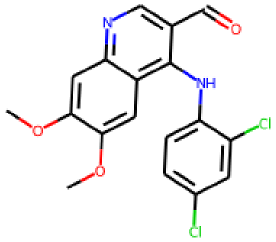	0.726749	COc1cc2ncc(C(=O)O)c(Nc3cccc(Cl)c3Cl)c2cc1OC1C(C)C1	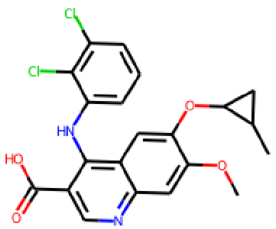	2.0	0.623362	2.525411	2.997881	4	0.774857
**122**	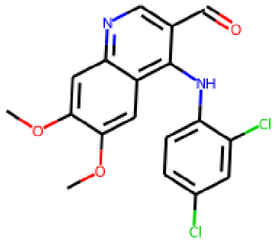	0.726749	COc1cc2ncc(C(=O)O)c(Nc3cccc(Cl)c3Cl)c2cc1OC1C(C)C1	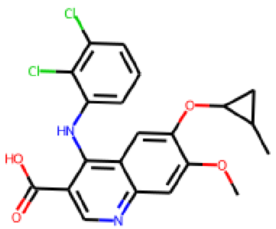	2.0	0.623362	2.525411	2.997881	4	0.774857
**123**	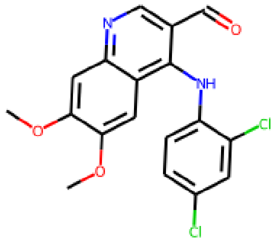	0.726749	COc1cc2ncc(C(=O)O)c(Nc3cccc(Cl)c3Cl)c2cc1OC1C(C)C1	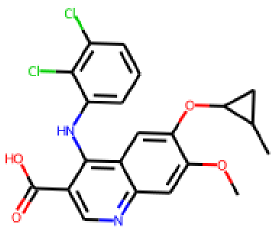	2.0	0.623362	2.525411	2.997881	4	0.774857

	src known mol	similarity	smiles	mol	Class	qed	logP	SAS	rings	kinase inhibition likeliness
**124**	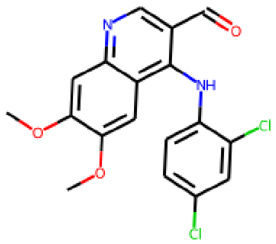	0.726749	COc1cc2ncc(C(=O)O)c(Nc3cccc(Cl)c3Cl)c2cc1OC1C(C)C1	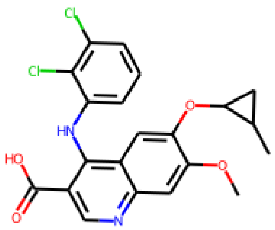	2.0	0.623362	2.525411	2.997881	4	0.774857
**125**	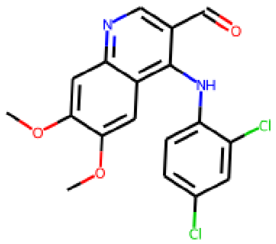	0.726749	COc1cc2ncc(C(=O)O)c(Nc3cccc(Cl)c3Cl)c2cc1OC1C(C)C1	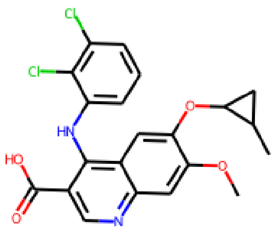	2.0	0.623362	2.525411	2.997881	4	0.774857
**126**	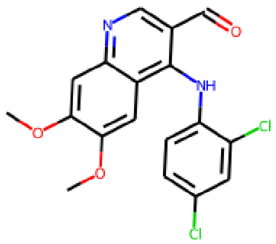	0.726749	COc1cc2ncc(C(=O)O)c(Nc3cccc(Cl)c3Cl)c2cc1OC1C(C)C1	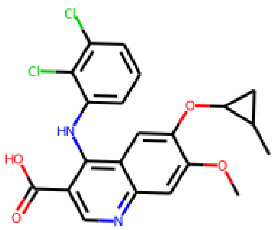	2.0	0.623362	2.525411	2.997881	4	0.774857
**127**	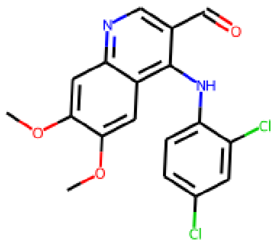	0.726749	COc1cc2ncc(C(=O)O)c(Nc3cccc(Cl)c3Cl)c2cc1OC1C(C)C1	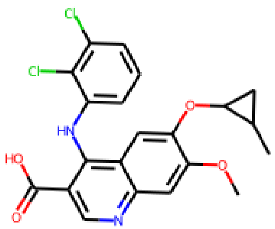	2.0	0.623362	2.525411	2.997881	4	0.774857

	src known mol	similarity	smiles	mol	Class	qed	logP	SAS	rings	kinase inhibition likeliness
**128**	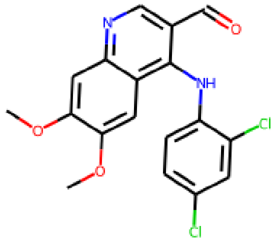	0.726524	COc1cc2ncc(C(=O)O)c(Nc3cccc(Cl)c3Cl)c2cc1OC1C(C)C1	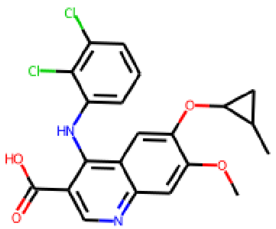	2.0	0.623362	2.525411	2.997881	4	0.774857
**129**	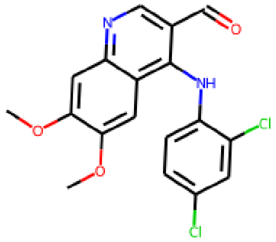	0.726473	COc1cc2ncc(C(=O)O)c(Nc3cccc(Cl)c3Cl)c2cc1OC1C(C)C1	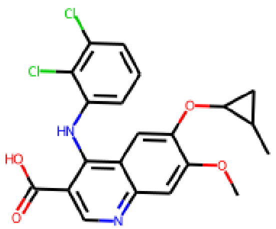	2.0	0.623362	2.525411	2.997881	4	0.774857
**130**	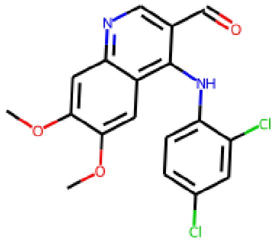	0.725780	COc1cc2ncc(C(=O)O)c(Nc3cccc(Cl)c3Cl)c2cc1OC1C(C)C1	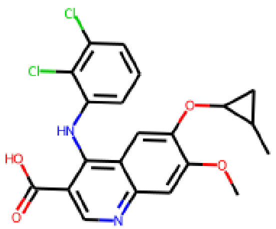	2.0	0.623362	2.525411	2.997881	4	0.774857
**131**	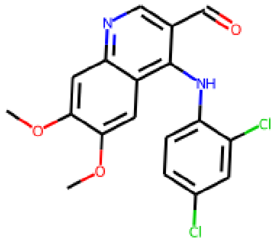	0.724590	COc1cc2ncc(C(=O)O)c(Nc3cccc(Cl)c3Cl)c2cc1OC1C(C)C1	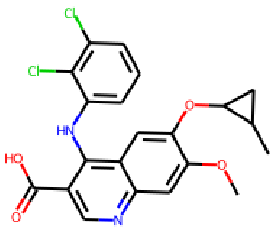	2.0	0.623362	2.525411	2.997881	4	0.774857

	src known mol	similarity	smiles	mol	Class	qed	logP	SAS	rings	kinase inhibition likeliness
**132**	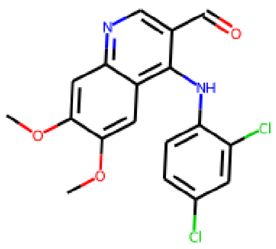	0.724587	COc1cc2ncc(C(=O)O)c(Nc3cccc(Cl)c3Cl)c2cc1OC1C(C)C1	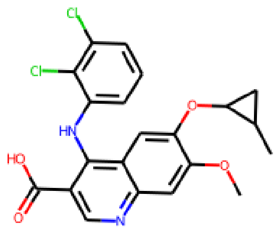	2.0	0.623362	2.525411	2.997881	4	0.774857
**133**	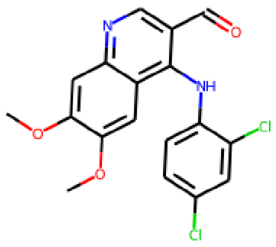	0.724280	COc1cc2ncc(C(=O)O)c(Nc3cccc(Cl)c3Cl)c2cc1OC1C(C)C1	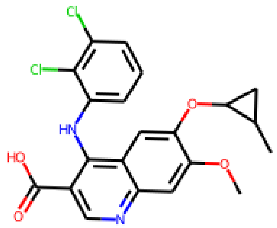	2.0	0.623362	2.525411	2.997881	4	0.774857
**134**	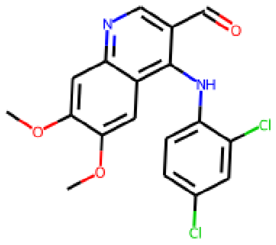	0.724223	COc1cc2ncc(C(=O)O)c(Nc3cccc(Cl)c3Cl)c2cc1OC1C(C)C1	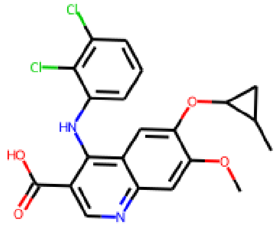	2.0	0.623362	2.525411	2.997881	4	0.774857
**135**	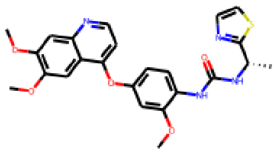	0.724138	COc1ccccc1NC(=O)Nc1ccc(Oc2ccnc3cc(OC)c(OC)cc23)cc1	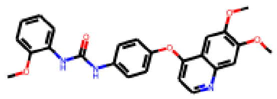	5.0	0.638163	3.995589	2.988471	4	0.789556

	src known mol	similarity	smiles	mol	Class	qed	logP	SAS	rings	kinase inhibition likeliness
**136**	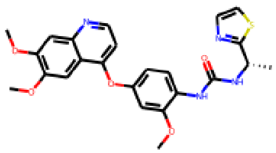	0.724138	COc1ccccc1NC(=O)Nc1ccc(Oc2ccnc3cc(OC)c(OC)cc23)cc1	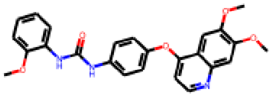	5.0	0.638163	3.995589	2.988471	4	0.789556
**137**	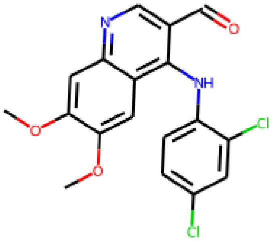	0.723732	COc1cc2ncc(C(=O)O)c(Nc3cccc(Cl)c3Cl)c2cc1OC1C(C)C1	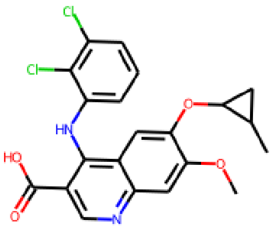	2.0	0.623362	2.525411	2.997881	4	0.774857
**138**	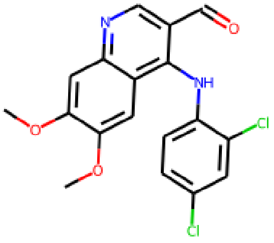	0.723610	COc1cc2ncc(C(=O)O)c(Nc3cccc(Cl)c3Cl)c2cc1OC1C(C)C1	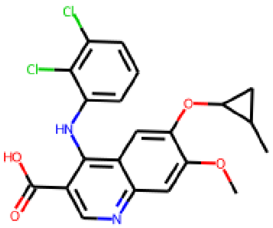	2.0	0.623362	2.525411	2.997881	4	0.774857
**139**	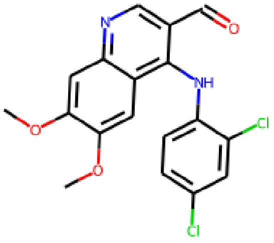	0.723610	COc1cc2ncc(C(=O)O)c(Nc3cccc(Cl)c3Cl)c2cc1OC1C(C)C1	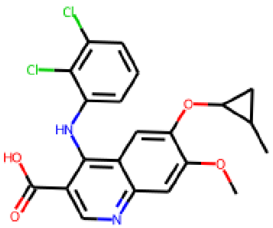	2.0	0.623362	2.525411	2.997881	4	0.774857

	src known mol	similarity	smiles	mol	Class	qed	logP	SAS	rings	kinase inhibition likeliness
**140**	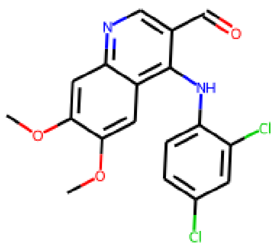	0.722086	COc1cc2ncc(C(=O)O)c(Nc3cccc(Cl)c3Cl)c2cc1OC1C(C)C1	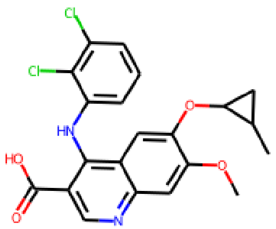	2.0	0.623362	2.525411	2.997881	4	0.774857
**141**	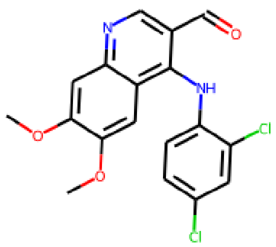	0.721798	COc1cc2ncc(C(=O)O)c(Nc3cccc(Cl)c3Cl)c2cc1OC1C(C)C1	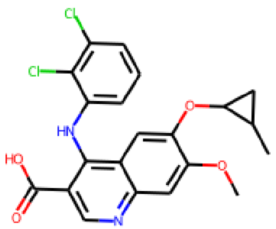	2.0	0.623362	2.525411	2.997881	4	0.774857
**142**	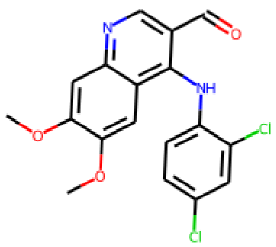	0.721271	COc1cc2ncc(C(=O)O)c(Nc3cccc(Cl)c3Cl)c2cc1OC1C(C)C1	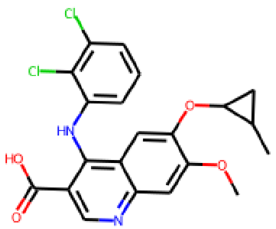	2.0	0.623362	2.525411	2.997881	4	0.774857
**143**	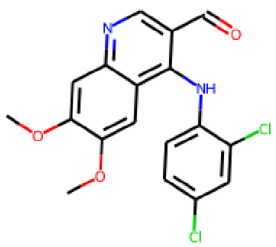	0.721083	COc1cc2ncc(C(=O)O)c(Nc3cccc(Cl)c3Cl)c2cc1OC1C(C)C1	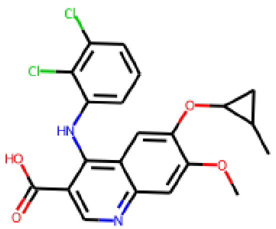	2.0	0.623362	2.525411	2.997881	4	0.774857

	src known mol	similarity	smiles	mol	Class	qed	logP	SAS	rings	kinase inhibition likeliness
**144**	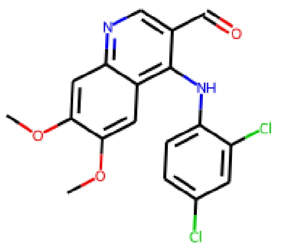	0.720588	COc1cc2ncc(C(=O)O)c(Nc3cccc(Cl)c3Cl)c2cc1OC1C(C)C1	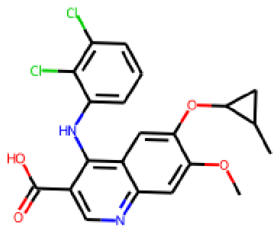	2.0	0.623362	2.525411	2.997881	4	0.774857
**145**	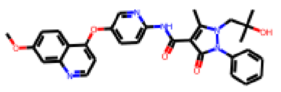	0.720493	COc1ccc2c(=O)cc(CNC(=O)c3c(C)n(C)n(-c4ccccc4)c3=O)nc3cccc3c2n1	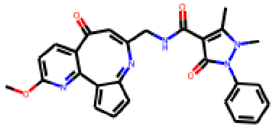	9.0	0.768132	3.608437	2.513170	5	0.823048
**146**	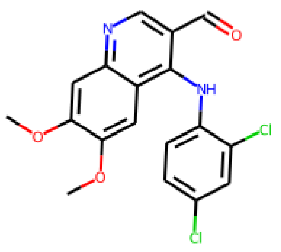	0.717094	COc1cc2ncc(C(=O)O)c(Nc3cccc(Cl)c3Cl)c2cc1OC1C(C)C1	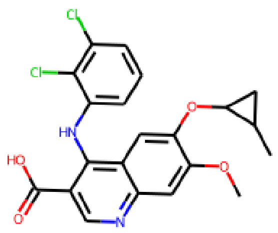	2.0	0.623362	2.525411	2.997881	4	0.774857
**147**	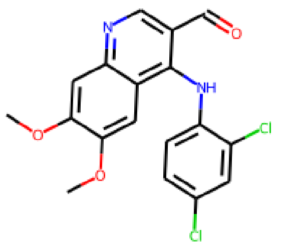	0.717094	COc1cc2ncc(C(=O)O)c(Nc3cccc(Cl)c3Cl)c2cc1OC1C(C)C1	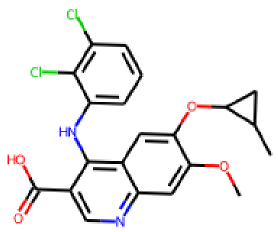	2.0	0.623362	2.525411	2.997881	4	0.774857

	src known mol	similarity	smiles	mol	Class	qed	logP	SAS	rings	kinase inhibition likeliness
**148**	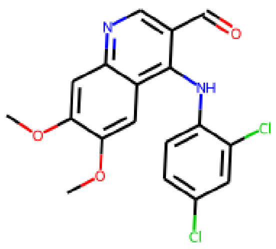	0.716828	COc1cc2ncc(C(=O)O)c(Nc3cccc(Cl)c3Cl)c2cc1OC1C(C)C1	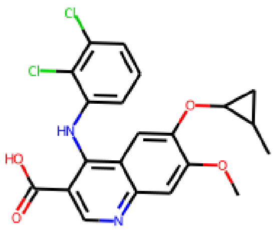	2.0	0.623362	2.525411	2.997881	4	0.774857
**149**	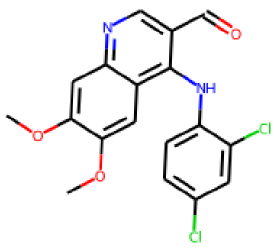	0.716828	COc1cc2ncc(C(=O)O)c(Nc3cccc(Cl)c3Cl)c2cc1OC1C(C)C1	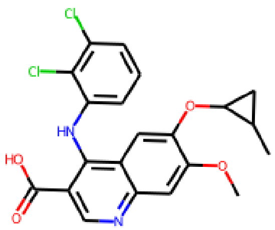	2.0	0.623362	2.525411	2.997881	4	0.774857
**150**	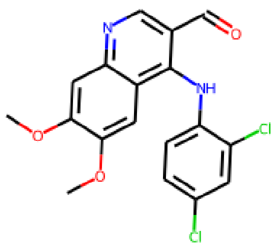	0.716828	COc1cc2ncc(C(=O)O)c(Nc3cccc(Cl)c3Cl)c2cc1OC1C(C)C1	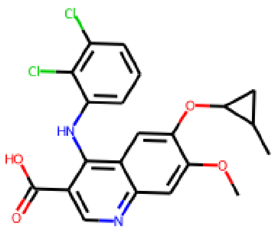	2.0	0.623362	2.525411	2.997881	4	0.774857
**151**	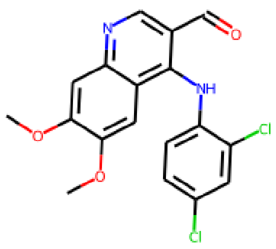	0.711712	COc1cc2ncc(C(=O)O)c(Nc3cccc(Cl)c3Cl)c2cc1OC1C(C)C1	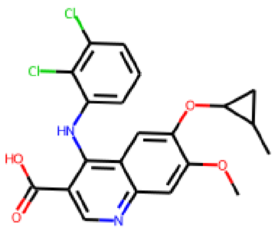	2.0	0.623362	2.525411	2.997881	4	0.774857

	src known mol	similarity	smiles	mol	Class	qed	logP	SAS	rings	kinase inhibition likeliness
**152**	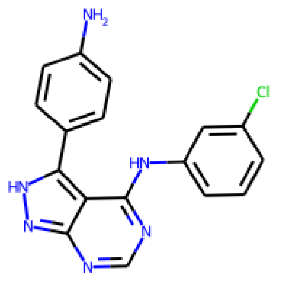	0.711087	c1ccc(Nc2ncnc3n[nH]c(-c4ccccc4)c23)cc1	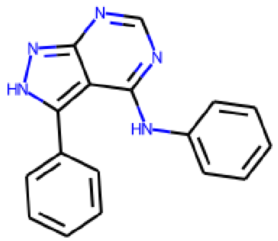	0.0	0.638163	3.995589	2.988471	4	0.800634
**153**	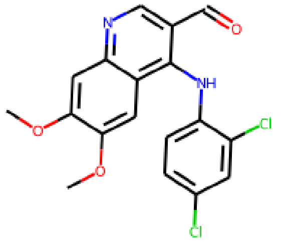	0.706548	COc1cc2ncc(C(=O)O)c(Nc3cccc(Cl)c3Cl)c2cc1OC1C(C)C1	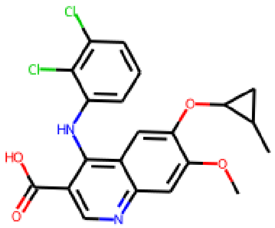	2.0	0.623362	2.525411	2.997881	4	0.774857
**154**	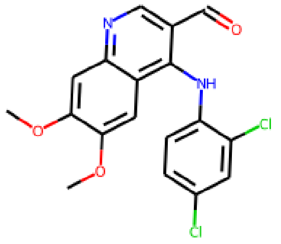	0.705600	COc1cc2ncc(C(=O)O)c(Nc3cccc(Cl)c3Cl)c2cc1OC1C(C)C1	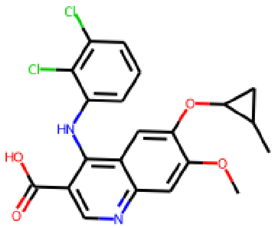	2.0	0.623362	2.525411	2.997881	4	0.774857
**155**	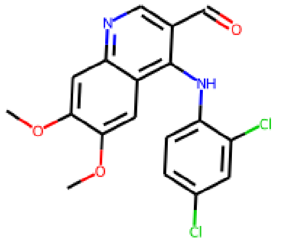	0.705128	COc1cc2ncc(C(=O)O)c(Nc3cccc(Cl)c3Cl)c2cc1OC1C(C)C1	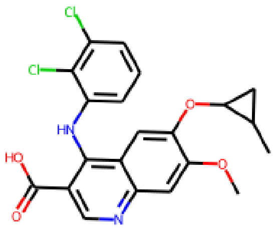	2.0	0.623362	2.525411	2.997881	4	0.774857

	src known mol	similarity	smiles	mol	Class	qed	logP	SAS	rings	kinase inhibition likeliness
**156**	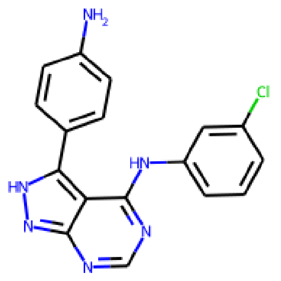	0.704449	c1ccc(Nc2ncnc3n[nH]c(-c4ccccc4)c23)cc1	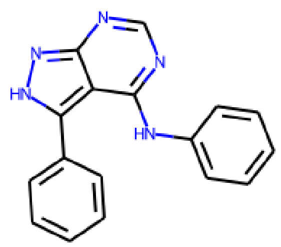	0.0	0.638163	3.995589	2.988471	4	0.800634
**157**	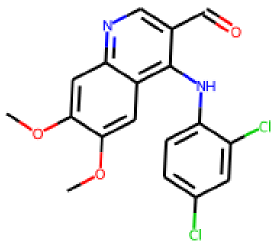	0.704277	COc1cc2ncc(C(=O)O)c(Nc3cccc(Cl)c3Cl)c2cc1OC1C(C)C1	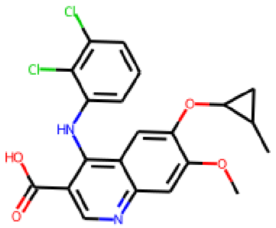	2.0	0.623362	2.525411	2.997881	4	0.774857
**158**	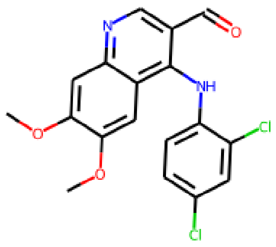	0.702773	COc1cc2ncc(C(=O)O)c(Nc3cccc(Cl)c3Cl)c2cc1OC1C(C)C1	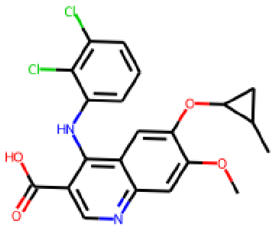	2.0	0.623362	2.525411	2.997881	4	0.774857
**159**	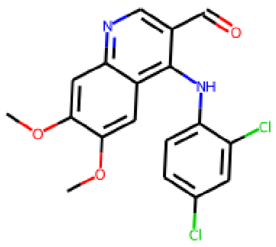	0.702020	COc1cc2ncc(C(=O)O)c(Nc3cccc(Cl)c3Cl)c2cc1OC1C(C)C1	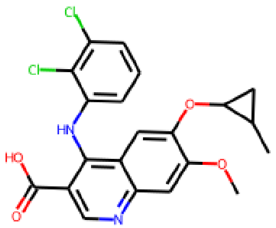	2.0	0.623362	2.525411	2.997881	4	0.774857

	src known mol	similarity	smiles	mol	Class	qed	logP	SAS	rings	kinase inhibition likeliness
**160**	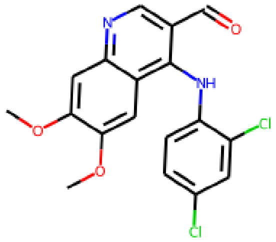	0.701340	COc1cc2ncc(C(=O)O)c(Nc3cccc(Cl)c3Cl)c2cc1OC1C(C)C1	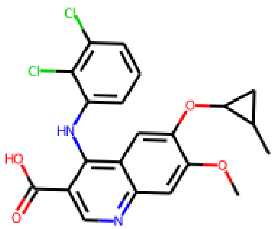	2.0	0.623362	2.525411	2.997881	4	0.774857
**161**	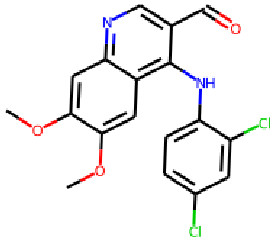	0.701340	COc1cc2ncc(C(=O)O)c(Nc3cccc(Cl)c3Cl)c2cc1OC1C(C)C1	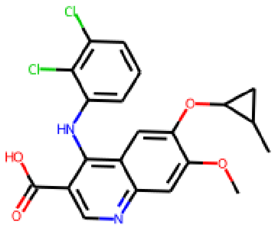	2.0	0.623362	2.525411	2.997881	4	0.774857
**162**	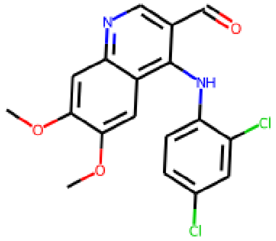	0.699160	COc1cc2ncc(C(=O)O)c(Nc3cccc(Cl)c3Cl)c2cc1OC1C(C)C1	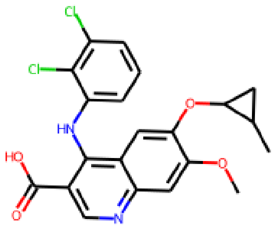	2.0	0.623362	2.525411	2.997881	4	0.774857
**163**	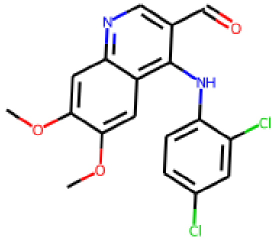	0.699160	COc1cc2ncc(C(=O)O)c(Nc3cccc(Cl)c3Cl)c2cc1OC1C(C)C1	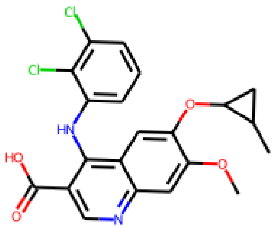	2.0	0.623362	2.525411	2.997881	4	0.774857

	src known mol	similarity	smiles	mol	Class	qed	logP	SAS	rings	kinase inhibition likeliness
**164**	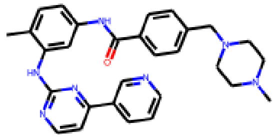	0.698598	Cc1ccc(NC(=O)c2ccccc2)cc1Nc1nccc(-c2cccnc2)n1	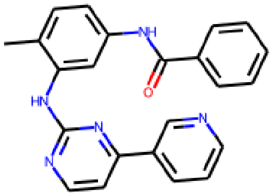	0.0	0.768132	3.608437	2.513170	4	0.854555
**165**	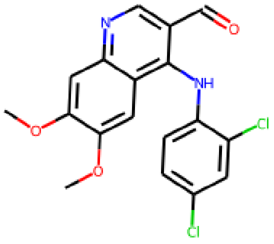	0.698573	COc1cc2ncc(C(=O)O)c(Nc3cccc(Cl)c3Cl)c2cc1OC1C(C)C1	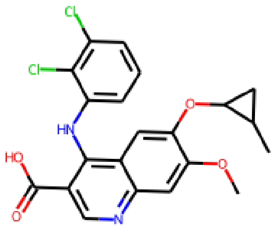	2.0	0.623362	2.525411	2.997881	4	0.774857
**166**	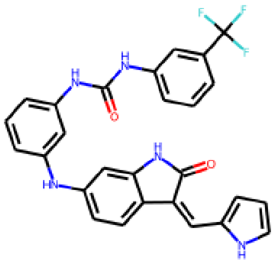	0.698413	COc1ccc(NC(=O)Nc2ccc3c(c2)NC(=O)/C3=C/c2ccc[nH]2)cc1	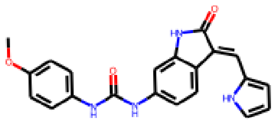	4.0	0.638163	3.995589	2.988471	4	0.768944
**167**	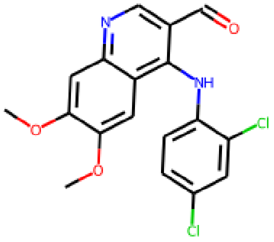	0.694856	COc1cc2ncc(C(=O)O)c(Nc3cccc(Cl)c3Cl)c2cc1OC1C(C)C1	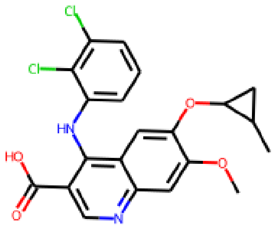	2.0	0.623362	2.525411	2.997881	4	0.774857

	src known mol	similarity	smiles	mol	Class	qed	logP	SAS	rings	kinase inhibition likeliness
**168**	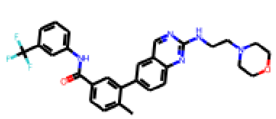	0.692308	Cc1ccc(C(=O)Nc2cccc(C(F)(F)F)c2C)cc1-c1ccc2nc(O)ncc2c1	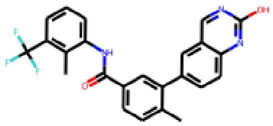	6.0	0.638163	3.995589	2.988471	4	0.808346
**169**	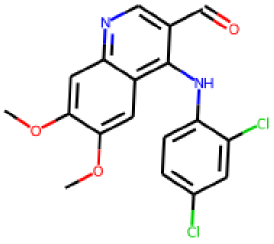	0.689655	COc1cc2ncc(C(=O)O)c(Nc3cccc(Cl)c3Cl)c2cc1OC1C(C)C1	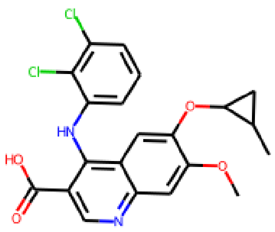	2.0	0.623362	2.525411	2.997881	4	0.774857
**170**	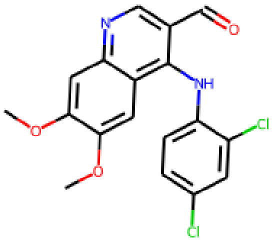	0.688663	COc1cc2ncc(C(=O)O)c(Nc3cccc(Cl)c3Cl)c2cc1OC1C(C)C1	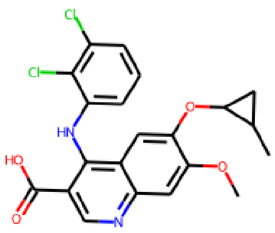	2.0	0.623362	2.525411	2.997881	4	0.774857
**171**	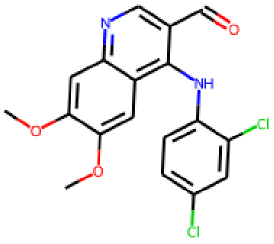	0.688663	COc1cc2ncc(C(=O)O)c(Nc3cccc(Cl)c3Cl)c2cc1OC1C(C)C1	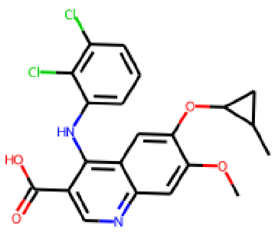	2.0	0.623362	2.525411	2.997881	4	0.774857

	src known mol	similarity	smiles	mol	Class	qed	logP	SAS	rings	kinase inhibition likeliness
**172**	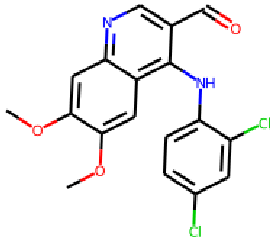	0.687554	COc1cc2ncc(C(=O)O)c(Nc3cccc(Cl)c3Cl)c2cc1OC1C(C)C1	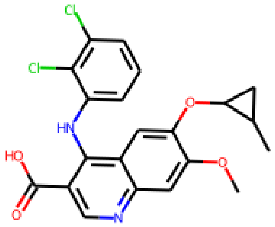	2.0	0.623362	2.525411	2.997881	4	0.774857
**173**	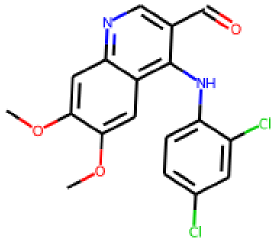	0.686138	COc1cc2ncc(C(=O)O)c(Nc3cccc(Cl)c3Cl)c2cc1OC1C(C)C1	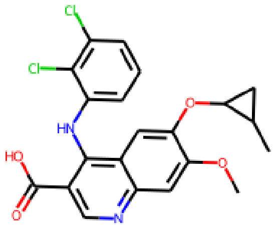	2.0	0.623362	2.525411	2.997881	4	0.774857
**174**	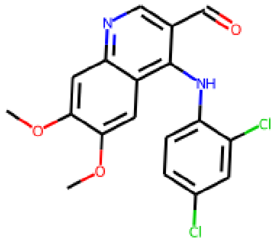	0.684943	COc1cc2ncc(C(=O)O)c(Nc3cccc(Cl)c3Cl)c2cc1OC1C(C)C1	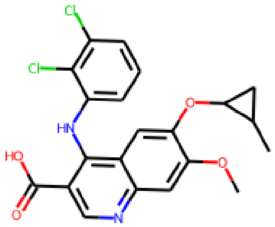	2.0	0.623362	2.525411	2.997881	4	0.774857
**175**	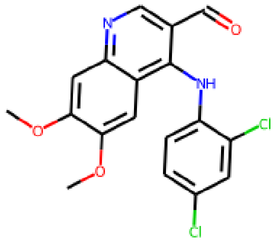	0.684932	COc1cc2ncc(C(=O)O)c(Nc3cccc(Cl)c3Cl)c2cc1OC1C(C)C1	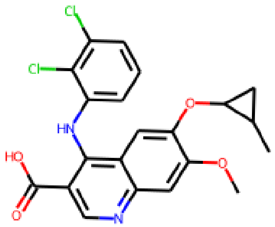	2.0	0.623362	2.525411	2.997881	4	0.774857

	src known mol	similarity	smiles	mol	Class	qed	logP	SAS	rings	kinase inhibition likeliness
**176**	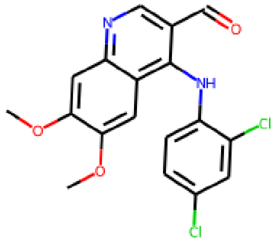	0.684708	COc1cc2ncc(C(=O)O)c(Nc3cccc(Cl)c3Cl)c2cc1OC1C(C)C1	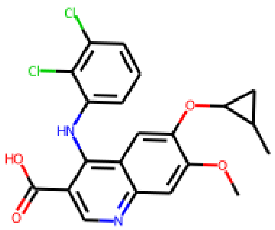	2.0	0.623362	2.525411	2.997881	4	0.774857
**177**	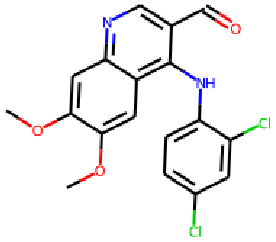	0.684708	COc1cc2ncc(C(=O)O)c(Nc3cccc(Cl)c3Cl)c2cc1OC1C(C)C1	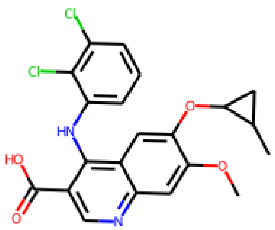	2.0	0.623362	2.525411	2.997881	4	0.774857
**178**	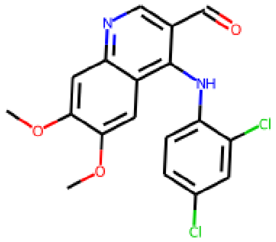	0.682676	COc1cc2ncc(C(=O)O)c(Nc3cccc(Cl)c3Cl)c2cc1OC1C(C)C1	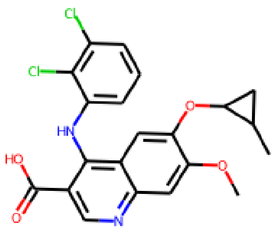	2.0	0.623362	2.525411	2.997881	4	0.774857
**179**	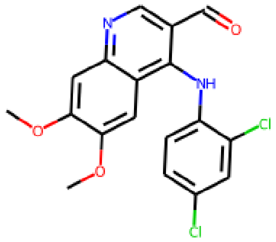	0.682580	COc1cc2ncc(C(=O)O)c(Nc3cccc(Cl)c3Cl)c2cc1OC1C(C)C1	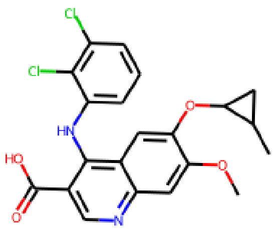	2.0	0.623362	2.525411	2.997881	4	0.774857

	src known mol	similarity	smiles	mol	Class	qed	logP	SAS	rings	kinase inhibition likeliness
**180**	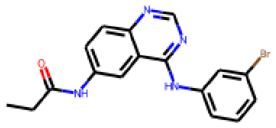	0.681778	O=C(CNC(=O)Oc1ccccc1)Nc1ccc2ncnc(Nc3cccc(Cl)c3)c2c1	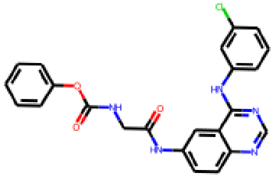	3.0	0.638163	3.995589	2.988471	4	0.793889
**181**	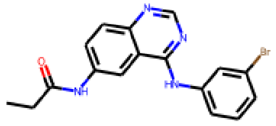	0.681778	O=C(CNC(=O)Oc1ccccc1)Nc1ccc2ncnc(Nc3cccc(Cl)c3)c2c1	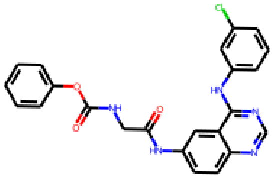	3.0	0.638163	3.995589	2.988471	4	0.793889
**182**	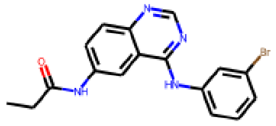	0.681778	O=C(CNC(=O)Oc1ccccc1)Nc1ccc2ncnc(Nc3cccc(Cl)c3)c2c1	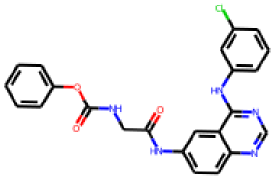	3.0	0.638163	3.995589	2.988471	4	0.793889
**183**	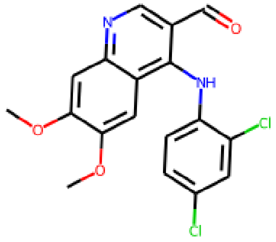	0.681745	COc1cc2ncc(C(=O)O)c(Nc3cccc(Cl)c3Cl)c2cc1OC1C(C)C1	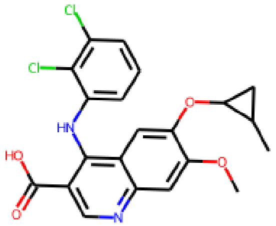	2.0	0.623362	2.525411	2.997881	4	0.774857

	src known mol	similarity	smiles	mol	Class	qed	logP	SAS	rings	kinase inhibition likeliness
**184**	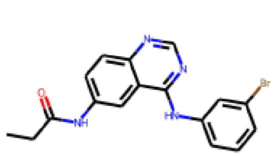	0.680851	O=C(CNC(=O)Oc1ccccc1)Nc1ccc2ncnc(Nc3cccc(Cl)c3)c2c1	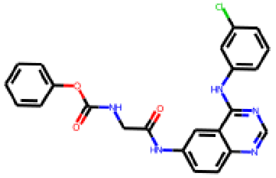	3.0	0.638163	3.995589	2.988471	4	0.793889
**185**	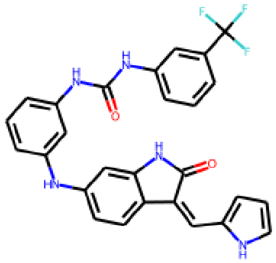	0.680421	COc1ccc(NC(=O)Nc2ccc3c(c2)NC(=O)/C3=C/c2ccc[nH]2)cc1	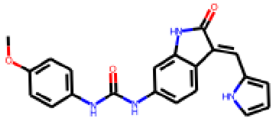	4.0	0.638163	3.995589	2.988471	4	0.768944
**186**	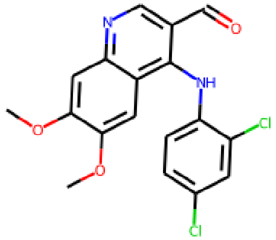	0.679245	COc1cc2ncc(C(=O)O)c(Nc3cccc(Cl)c3Cl)c2cc1OC1C(C)C1	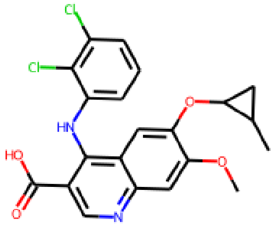	2.0	0.623362	2.525411	2.997881	4	0.774857
**187**	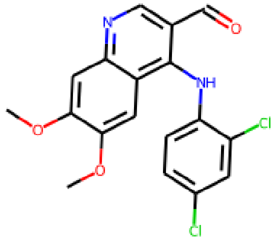	0.678254	COc1cc2ncc(C(=O)O)c(Nc3cccc(Cl)c3Cl)c2cc1OC1C(C)C1	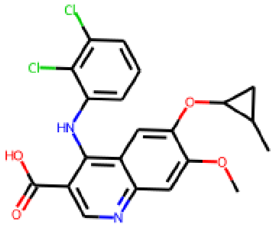	2.0	0.623362	2.525411	2.997881	4	0.774857

	src known mol	similarity	smiles	mol	Class	qed	logP	SAS	rings	kinase inhibition likeliness
**188**	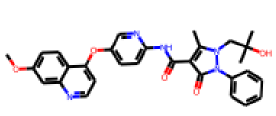	0.676546	COc1ccc2c(=O)cc(CNC(=O)c3c(C)n(C)n(-c4ccccc4)c3=O)nc3cccc2c3c1	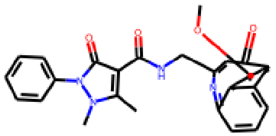	9.0	0.638163	3.995589	2.988471	5	0.774651
**189**	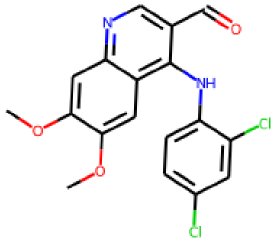	0.674516	COc1cc2ncc(C(=O)O)c(Nc3cccc(Cl)c3Cl)c2cc1OC1C(C)C1	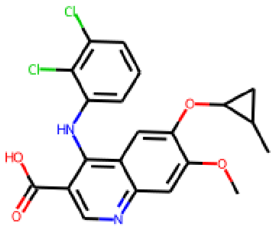	2.0	0.623362	2.525411	2.997881	4	0.774857
**190**	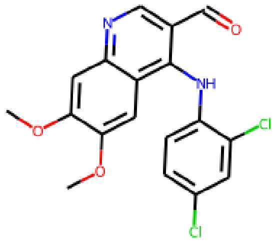	0.674516	COc1cc2ncc(C(=O)O)c(Nc3cccc(Cl)c3Cl)c2cc1OC1C(C)C1	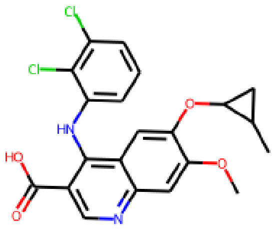	2.0	0.623362	2.525411	2.997881	4	0.774857
**191**	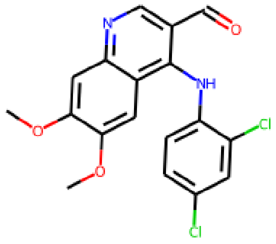	0.674516	COc1cc2ncc(C(=O)O)c(Nc3cccc(Cl)c3Cl)c2cc1OC1C(C)C1	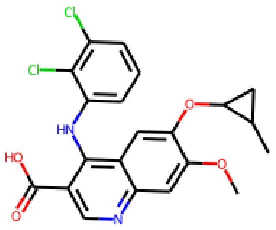	2.0	0.623362	2.525411	2.997881	4	0.774857

	src known mol	similarity	smiles	mol	Class	qed	logP	SAS	rings	kinase inhibition likeliness
**192**	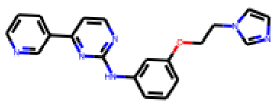	0.673767	Cc1ccc(OC(=O)c2ccccc2)cc1Nc1nccc(-c2cccnc2)n1	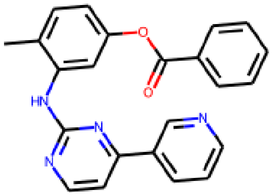	0.0	0.638163	3.995589	2.988471	4	0.775468
**193**	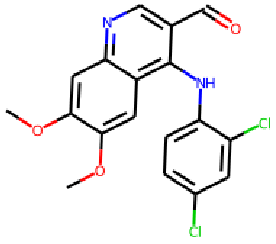	0.672383	COc1cc2ncc(C(=O)O)c(Nc3cccc(Cl)c3Cl)c2cc1OC1C(C)C1	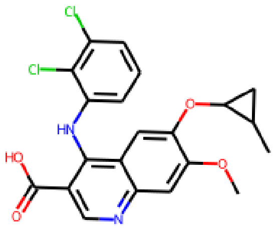	2.0	0.623362	2.525411	2.997881	4	0.774857
**194**	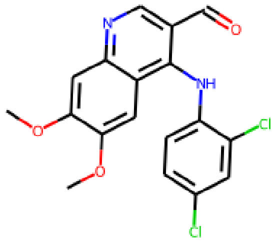	0.670194	COc1cc2ncc(C(=O)O)c(Nc3cccc(Cl)c3Cl)c2cc1OC1C(C)C1	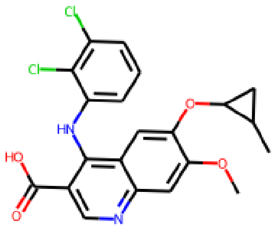	2.0	0.623362	2.525411	2.997881	4	0.774857
**195**	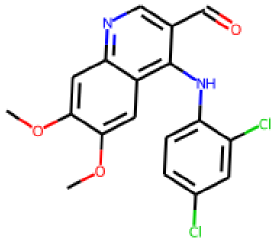	0.669973	COc1cc2ncc(C(=O)O)c(Nc3cccc(Cl)c3Cl)c2cc1OC1C(C)C1	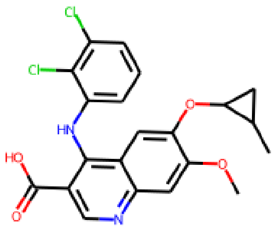	2.0	0.623362	2.525411	2.997881	4	0.774857

	src known mol	similarity	smiles	mol	Class	qed	logP	SAS	rings	kinase inhibition likeliness
**196**	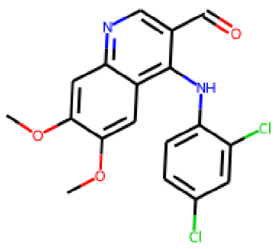	0.669326	COc1cc2ncc(C(=O)O)c(Nc3cccc(Cl)c3Cl)c2cc1OC1C(C)C1	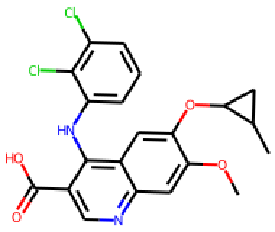	2.0	0.623362	2.525411	2.997881	4	0.774857
**197**	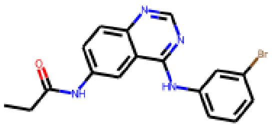	0.666951	O=C(CNC(=O)Oc1ccccc1)Nc1ccc2ncnc(Nc3cccc(Cl)c3)c2c1	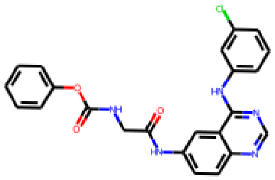	3.0	0.638163	3.995589	2.988471	4	0.793889
**198**	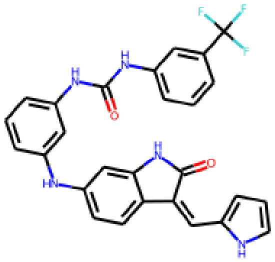	0.666944	COc1ccc(NC(=O)Nc2ccc3c(c2)NC(=O)/C3=C/c2ccc[nH]2)cc1	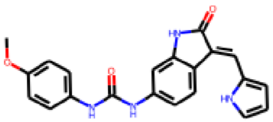	4.0	0.638163	3.995589	2.988471	4	0.768944
**199**	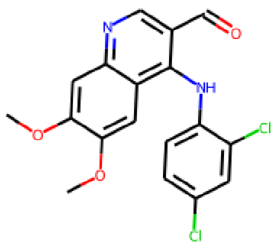	0.666928	COc1cc2ncc(C(=O)O)c(Nc3cccc(Cl)c3Cl)c2cc1OC1C(C)C1	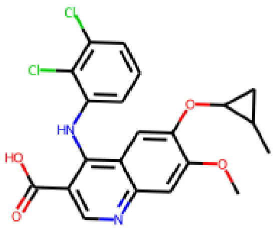	2.0	0.623362	2.525411	2.997881	4	0.774857

	src known mol	similarity	smiles	mol	Class	qed	logP	SAS	rings	kinase inhibition likeliness
**200**	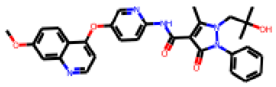	0.666496	COc1ccc2c(=O)cc(CNC(=O)c3c(C)n(C)n(-c4ccccc4)c3=O)nc3cccc2c3c1	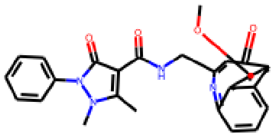	9.0	0.638163	3.995589	2.988471	5	0.774651
**201**	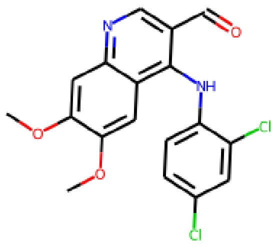	0.666085	COc1cc2ncc(C(=O)O)c(Nc3cccc(Cl)c3Cl)c2cc1OC1C(C)C1	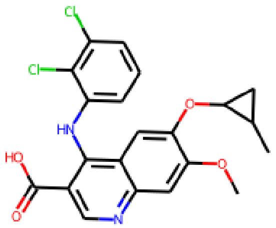	2.0	0.623362	2.525411	2.997881	4	0.774857
**202**	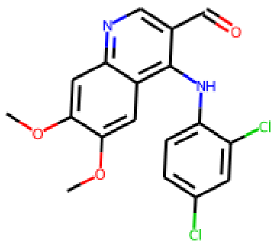	0.666085	COc1cc2ncc(C(=O)O)c(Nc3cccc(Cl)c3Cl)c2cc1OC1C(C)C1	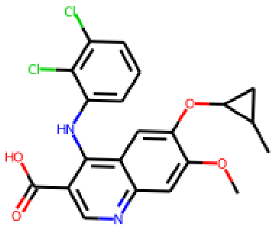	2.0	0.623362	2.525411	2.997881	4	0.774857
**203**	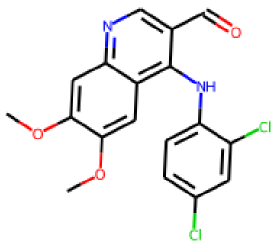	0.666085	COc1cc2ncc(C(=O)O)c(Nc3cccc(Cl)c3Cl)c2cc1OC1C(C)C1	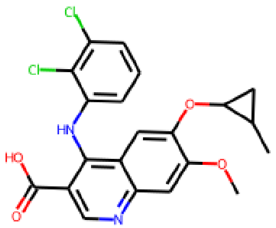	2.0	0.623362	2.525411	2.997881	4	0.774857

	src known mol	similarity	smiles	mol	Class	qed	logP	SAS	rings	kinase inhibition likeliness
**204**	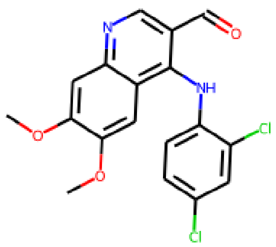	0.666085	COc1cc2ncc(C(=O)O)c(Nc3cccc(Cl)c3Cl)c2cc1OC1C(C)C1	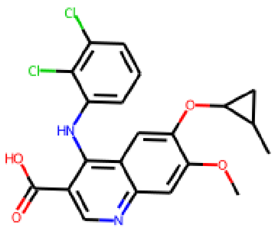	2.0	0.623362	2.525411	2.997881	4	0.774857
**205**	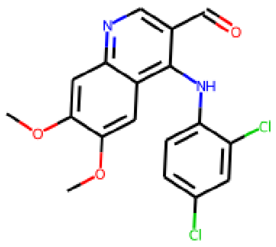	0.666085	COc1cc2ncc(C(=O)O)c(Nc3cccc(Cl)c3Cl)c2cc1OC1C(C)C1	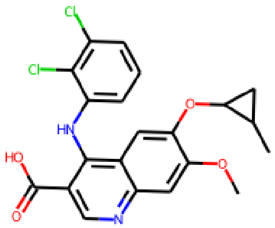	2.0	0.623362	2.525411	2.997881	4	0.774857
**206**	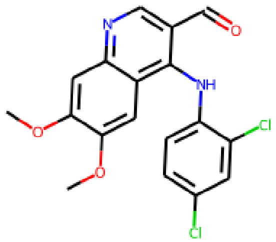	0.665313	COc1cc2ncc(C(=O)O)c(Nc3cccc(Cl)c3Cl)c2cc1OC1C(C)C1	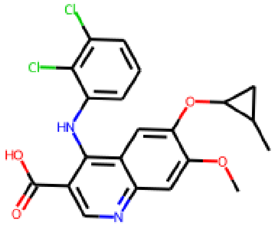	2.0	0.623362	2.525411	2.997881	4	0.774857
**207**	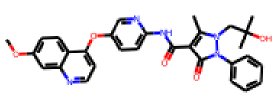	0.664436	COc1ccc2c(=O)cc(CNC(=O)c3c(C)n(C)n(-c4ccccc4)c3=O)nc3cccc2c3c1	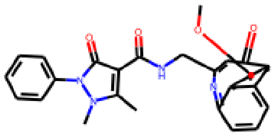	9.0	0.638163	3.995589	2.988471	5	0.774651

	src known mol	similarity	smiles	mol	Class	qed	logP	SAS	rings	kinase inhibition likeliness
**208**	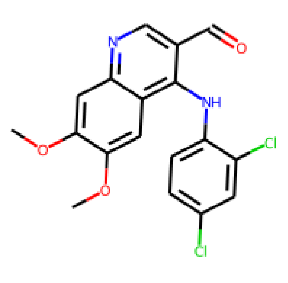	0.661031	COc1cc2ncc(C(=O)O)c(Nc3cccc(Cl)c3Cl)c2cc1OC1C(C)C1	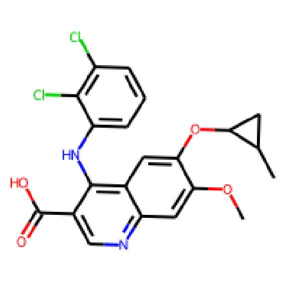	2.0	0.623362	2.525411	2.997881	4	0.774857
**209**	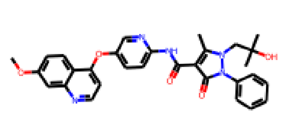	0.660816	COc1ccc2c(=O)cc(CNC(=O)c3c(C)n(C)n(-c4ccccc4)c3=O)nc3cccc2c3c1	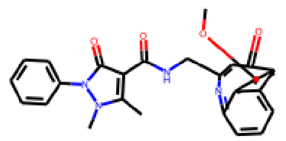	9.0	0.638163	3.995589	2.988471	5	0.774651
**210**	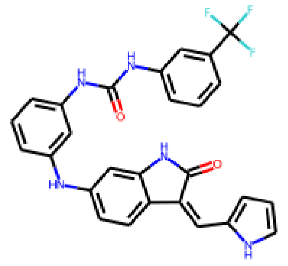	0.659952	COc1ccc(NC(=O)Nc2ccc3c(c2)NC(=O)/C3=C/c2ccc[nH]2)cc1	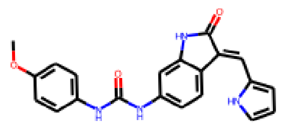	4.0	0.638163	3.995589	2.988471	4	0.768944
**211**	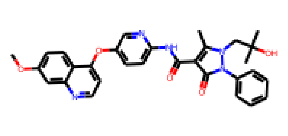	0.659255	COc1ccc2c(=O)cc(CNC(=O)c3c(C)n(C)n(-c4ccccc4)c3=O)nc3cccc2c3c1	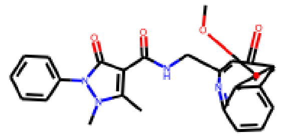	9.0	0.638163	3.995589	2.988471	5	0.774651

	src known mol	similarity	smiles	mol	Class	qed	logP	SAS	rings	kinase inhibition likeliness
**212**	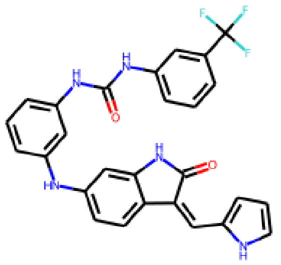	0.659196	COc1ccc(NC(=O)Nc2ccc3c(c2)NC(=O)/C3=C/c2ccc[nH]2)cc1	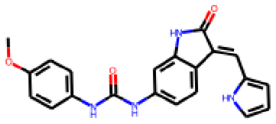	4.0	0.638163	3.995589	2.988471	4	0.768944
**213**	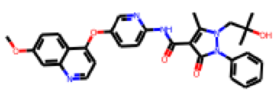	0.658787	COc1ccc2c(=O)cc(CNC(=O)c3c(C)n(C)n(-c4ccccc4)c3=O)nc3cccc2c3c1	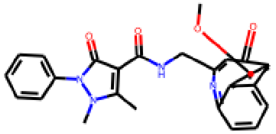	9.0	0.638163	3.995589	2.988471	5	0.774651
**214**	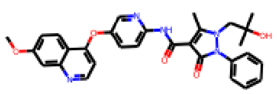	0.658787	COc1ccc2c(=O)cc(CNC(=O)c3c(C)n(C)n(-c4ccccc4)c3=O)nc3cccc2c3c1	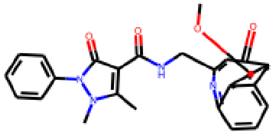	9.0	0.638163	3.995589	2.988471	5	0.774651
**215**	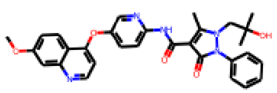	0.658787	COc1ccc2c(=O)cc(CNC(=O)c3c(C)n(C)n(-c4ccccc4)c3=O)nc3cccc2c3c1	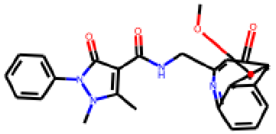	9.0	0.638163	3.995589	2.988471	5	0.774651

	src known mol	similarity	smiles	mol	Class	qed	logP	SAS	rings	kinase inhibition likeliness
**216**	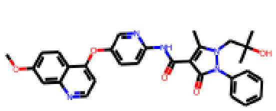	0.658787	COc1ccc2c(=O)cc(CNC(=O)c3c(C)n(C)n(-c4ccccc4)c3=O)nc3cccc2c3c1	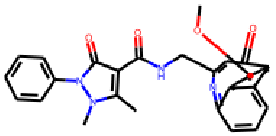	9.0	0.638163	3.995589	2.988471	5	0.774651
**217**	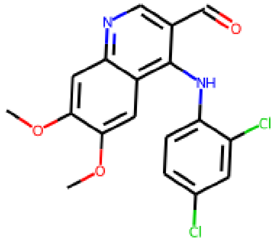	0.658556	COc1cc2ncc(C(=O)O)c(Nc3cccc(Cl)c3Cl)c2cc1OC1C(C)C1	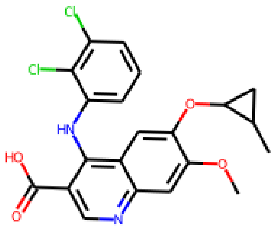	2.0	0.623362	2.525411	2.997881	4	0.774857
**218**	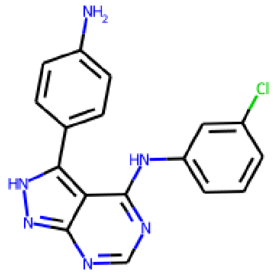	0.658277	c1ccc(Nc2ncnc3n[nH]c(-c4ccccc4)c23)cc1	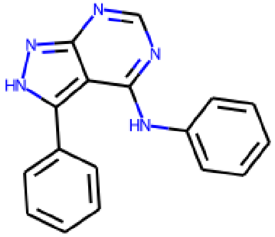	0.0	0.638163	3.995589	2.988471	4	0.800634
**219**	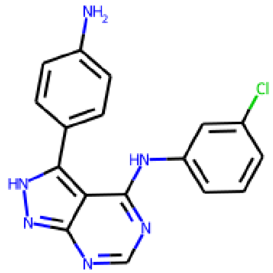	0.658277	c1ccc(Nc2ncnc3n[nH]c(-c4ccccc4)c23)cc1	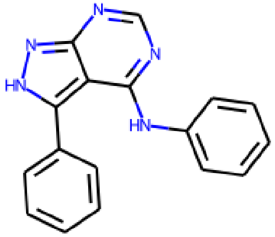	0.0	0.638163	3.995589	2.988471	4	0.800634

	src known mol	similarity	smiles	mol	Class	qed	logP	SAS	rings	kinase inhibition likeliness
**220**	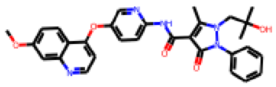	0.657246	COc1ccc2c(=O)cc(CNC(=O)c3c(C)n(C)n(-c4ccccc4)c3=O)nc3cccc2c3c1	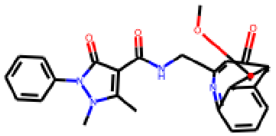	9.0	0.638163	3.995589	2.988471	5	0.774651
**221**	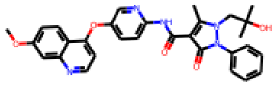	0.656762	COc1ccc2c(=O)cc(CNC(=O)c3c(C)n(C)n(-c4ccccc4)c3=O)nc3cccc2c3c1	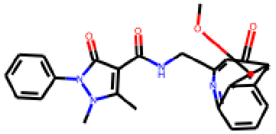	9.0	0.638163	3.995589	2.988471	5	0.774651
**222**	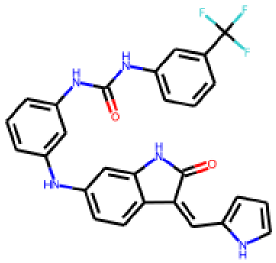	0.655359	COc1ccc(NC(=O)Nc2ccc3c(c2)NC(=O)/C3=C/c2ccc[nH]2)cc1	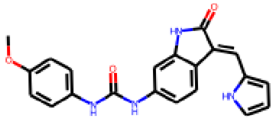	4.0	0.638163	3.995589	2.988471	4	0.768944
**223**	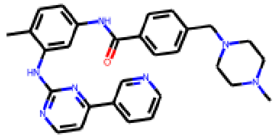	0.652412	Cc1ccc(NC(=O)c2ccccc2)cc1Nc1nccc(-c2cccnc2)n1	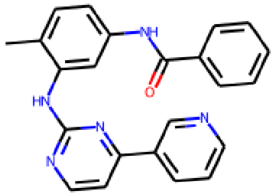	0.0	0.768132	3.608437	2.513170	4	0.854555

	src known mol	similarity	smiles	mol	Class	qed	logP	SAS	rings	kinase inhibition likeliness
**224**	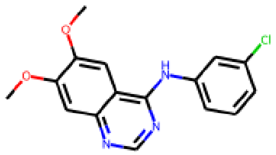	0.651972	Clc1cccc(Nc2ncnc3cc4c[nH]nc4cc23)c1	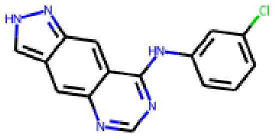	3.0	0.638163	3.995589	2.988471	4	0.781333
**225**	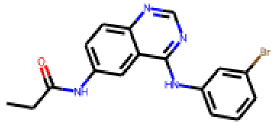	0.651952	O=C(CNC(=O)Oc1ccccc1)Nc1ccc2ncnc(Nc3cccc(Cl)c3)c2c1	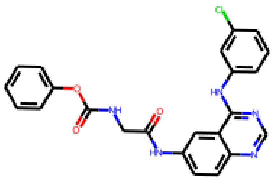	3.0	0.638163	3.995589	2.988471	4	0.793889
**226**	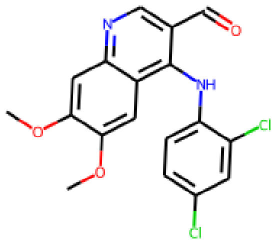	0.651733	COc1cc2ncc(C(=O)O)c(Nc3cccc(Cl)c3Cl)c2cc1OC1C(C)C1	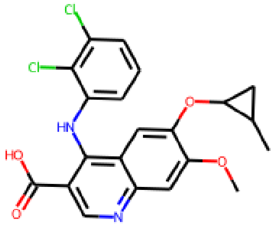	2.0	0.623362	2.525411	2.997881	4	0.774857
**227**	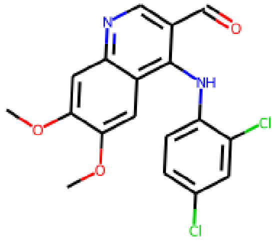	0.651733	COc1cc2ncc(C(=O)O)c(Nc3cccc(Cl)c3Cl)c2cc1OC1C(C)C1	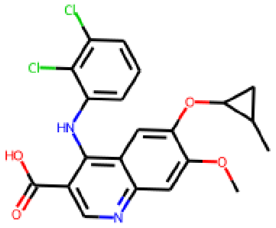	2.0	0.623362	2.525411	2.997881	4	0.774857

	src known mol	similarity	smiles	mol	Class	qed	logP	SAS	rings	kinase inhibition likeliness
**228**	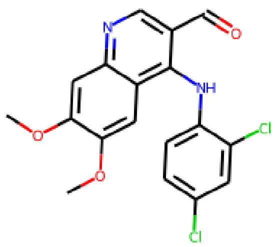	0.651733	COc1cc2ncc(C(=O)O)c(Nc3cccc(Cl)c3Cl)c2cc1OC1C(C)C1	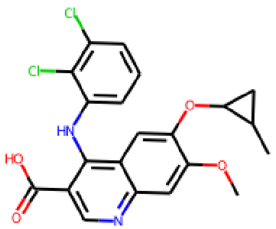	2.0	0.623362	2.525411	2.997881	4	0.774857
**229**	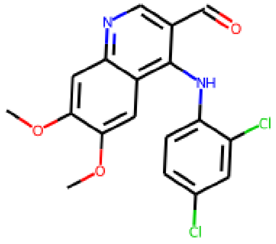	0.651733	COc1cc2ncc(C(=O)O)c(Nc3cccc(Cl)c3Cl)c2cc1OC1C(C)C1	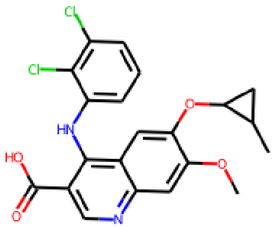	2.0	0.623362	2.525411	2.997881	4	0.774857
**230**	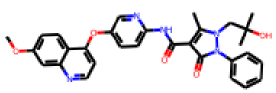	0.650206	COc1ccc2c(=O)cc(CNC(=O)c3c(C)n(C)n(-c4ccccc4)c3=O)nc3cccc2c3c1	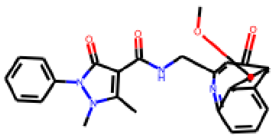	9.0	0.638163	3.995589	2.988471	5	0.774651
**231**	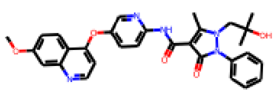	0.650206	COc1ccc2c(=O)cc(CNC(=O)c3c(C)n(C)n(-c4ccccc4)c3=O)nc3cccc2c3c1	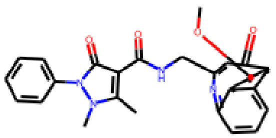	9.0	0.638163	3.995589	2.988471	5	0.774651

	src known mol	similarity	smiles	mol	Class	qed	logP	SAS	rings	kinase inhibition likeliness
**232**	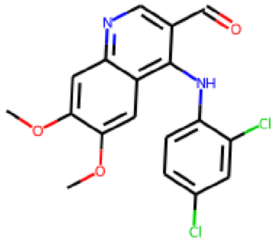	0.649916	COc1cc2ncc(C(=O)O)c(Nc3cccc(Cl)c3Cl)c2cc1OC1C(C)C1	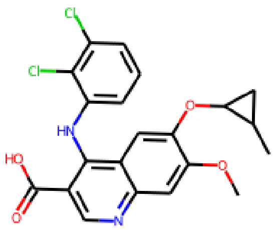	2.0	0.623362	2.525411	2.997881	4	0.774857
**233**	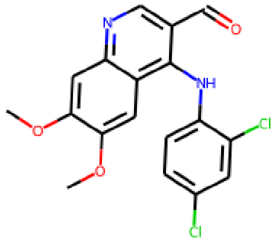	0.648237	COc1cc2ncc(C(=O)O)c(Nc3cccc(Cl)c3Cl)c2cc1OC1C(C)C1	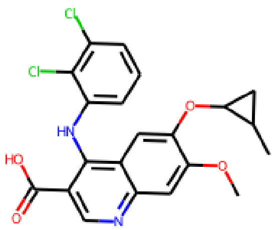	2.0	0.623362	2.525411	2.997881	4	0.774857
**234**	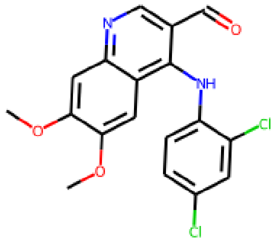	0.648237	COc1cc2ncc(C(=O)O)c(Nc3cccc(Cl)c3Cl)c2cc1OC1C(C)C1	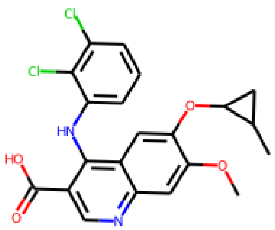	2.0	0.623362	2.525411	2.997881	4	0.774857
**235**	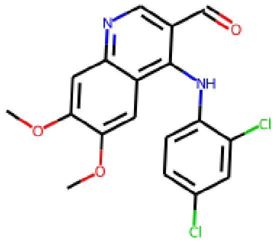	0.648237	COc1cc2ncc(C(=O)O)c(Nc3cccc(Cl)c3Cl)c2cc1OC1C(C)C1	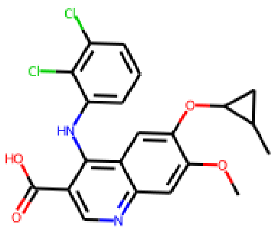	2.0	0.623362	2.525411	2.997881	4	0.774857

	src known mol	similarity	smiles	mol	Class	qed	logP	SAS	rings	kinase inhibition likeliness
**236**	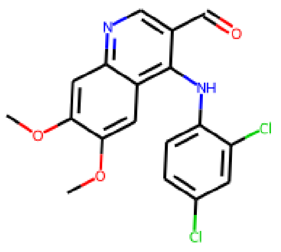	0.648237	COc1cc2ncc(C(=O)O)c(Nc3cccc(Cl)c3Cl)c2cc1OC1C(C)C1	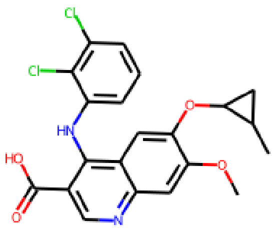	2.0	0.623362	2.525411	2.997881	4	0.774857
**237**	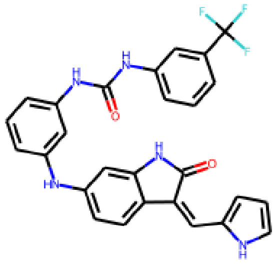	0.648045	COc1ccc(NC(=O)Nc2ccc3c(c2)NC(=O)/C3=C/c2ccc[nH]2)cc1	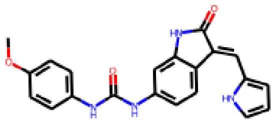	4.0	0.638163	3.995589	2.988471	4	0.768944
**238**	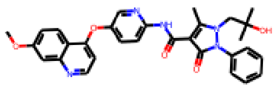	0.647179	COc1ccc2c(=O)cc(CNC(=O)c3c(C)n(C)n(-c4ccccc4)c3=O)nc3cccc2c3c1	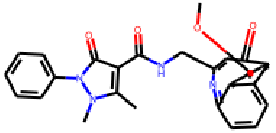	9.0	0.638163	3.995589	2.988471	5	0.774651
**239**	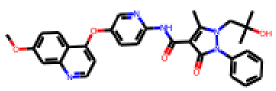	0.647179	COc1ccc2c(=O)cc(CNC(=O)c3c(C)n(C)n(-c4ccccc4)c3=O)nc3cccc2c3c1	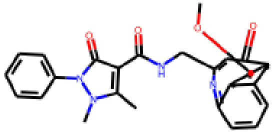	9.0	0.638163	3.995589	2.988471	5	0.774651

	src known mol	similarity	smiles	mol	Class	qed	logP	SAS	rings	kinase inhibition likeliness
**240**	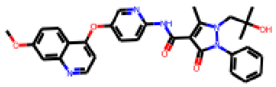	0.645823	COc1ccc2c(=O)cc(CNC(=O)c3c(C)n(C)n(-c4ccccc4)c3=O)nc3cccc2c3c1	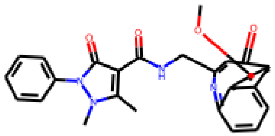	9.0	0.638163	3.995589	2.988471	5	0.774651
**241**	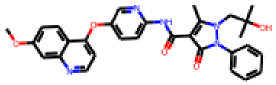	0.645310	COc1ccc2c(=O)cc(CNC(=O)c3c(C)n(C)n(-c4ccccc4)c3=O)nc3cccc2c3c1	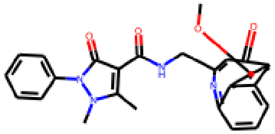	9.0	0.638163	3.995589	2.988471	5	0.774651
**242**	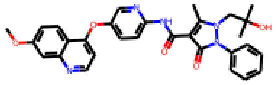	0.645310	COc1ccc2c(=O)cc(CNC(=O)c3c(C)n(C)n(-c4ccccc4)c3=O)nc3cccc2c3c1	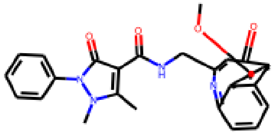	9.0	0.638163	3.995589	2.988471	5	0.774651
**243**	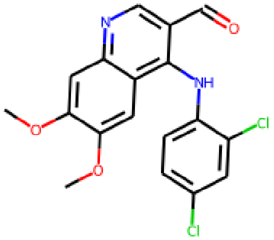	0.644822	COc1cc2ncc(C(=O)O)c(Nc3cccc(Cl)c3Cl)c2cc1OC1C(C)C1	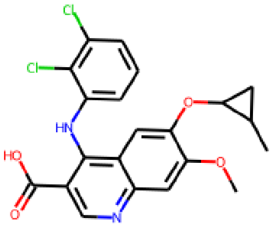	2.0	0.623362	2.525411	2.997881	4	0.774857

	src known mol	similarity	smiles	mol	Class	qed	logP	SAS	rings	kinase inhibition likeliness
**244**	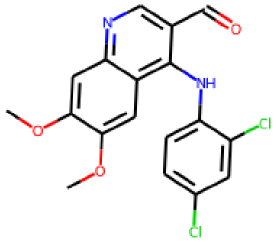	0.643799	COc1cc2ncc(C(=O)O)c(Nc3cccc(Cl)c3Cl)c2cc1OC1C(C)C1	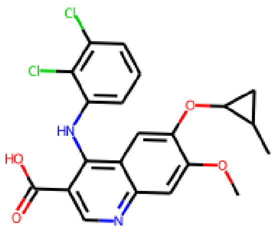	2.0	0.623362	2.525411	2.997881	4	0.774857
**245**	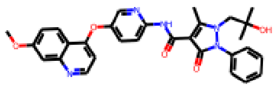	0.643375	COc1ccc2c(=O)cc(CNC(=O)c3c(C)n(C)n(-c4ccccc4)c3=O)nc3cccc2c3c1	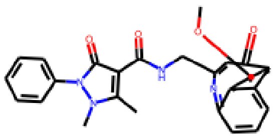	9.0	0.638163	3.995589	2.988471	5	0.774651
**246**	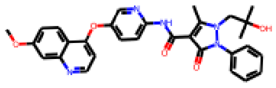	0.643375	COc1ccc2c(=O)cc(CNC(=O)c3c(C)n(C)n(-c4ccccc4)c3=O)nc3cccc2c3c1	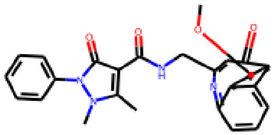	9.0	0.638163	3.995589	2.988471	5	0.774651
**247**	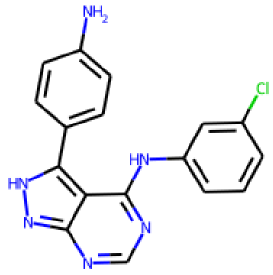	0.642791	c1ccc(Nc2ncnc3n[nH]c(-c4ccccc4)c23)cc1	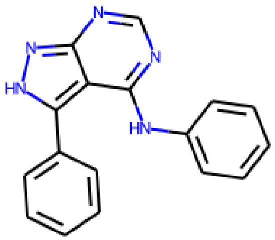	0.0	0.638163	3.995589	2.988471	4	0.800634

	src known mol	similarity	smiles	mol	Class	qed	logP	SAS	rings	kinase inhibition likeliness
**248**	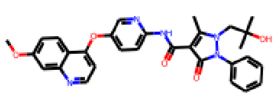	0.642527	COc1ccc2c(=O)cc(CNC(=O)c3c(C)n(C)n(-c4ccccc4)c3=O)nc3cccc2c3c1	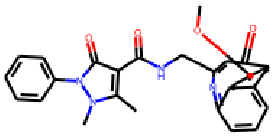	9.0	0.638163	3.995589	2.988471	5	0.774651
**249**	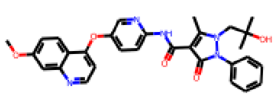	0.641393	COc1ccc2c(=O)cc(CNC(=O)c3c(C)n(C)n(-c4ccccc4)c3=O)nc3cccc2c3c1	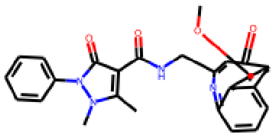	9.0	0.638163	3.995589	2.988471	5	0.774651
**250**	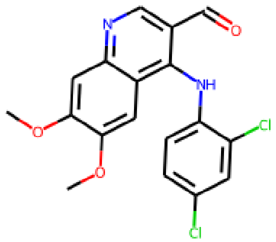	0.641363	COc1cc2ncc(C(=O)O)c(Nc3cccc(Cl)c3Cl)c2cc1OC1C(C)C1	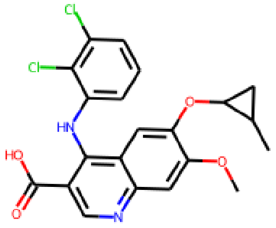	2.0	0.623362	2.525411	2.997881	4	0.774857
**251**	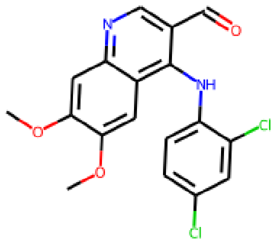	0.641363	COc1cc2ncc(C(=O)O)c(Nc3cccc(Cl)c3Cl)c2cc1OC1C(C)C1	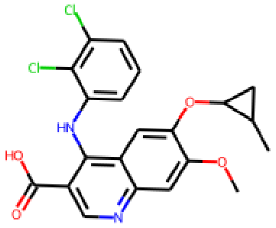	2.0	0.623362	2.525411	2.997881	4	0.774857

	src known mol	similarity	smiles	mol	Class	qed	logP	SAS	rings	kinase inhibition likeliness
**252**	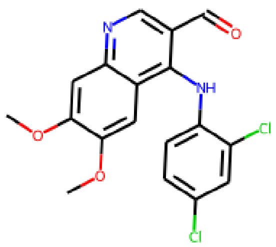	0.640964	COc1cc2ncc(C(=O)O)c(Nc3cccc(Cl)c3Cl)c2cc1OC1C(C)C1	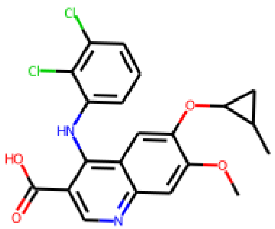	2.0	0.623362	2.525411	2.997881	4	0.774857
**253**	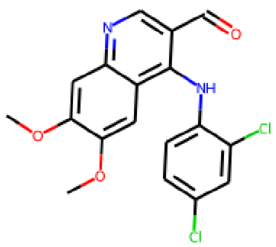	0.640964	COc1cc2ncc(C(=O)O)c(Nc3cccc(Cl)c3Cl)c2cc1OC1C(C)C1	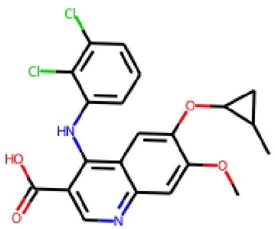	2.0	0.623362	2.525411	2.997881	4	0.774857
**254**	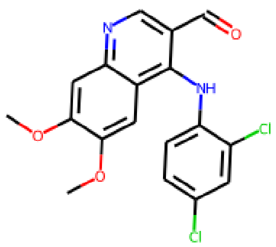	0.640964	COc1cc2ncc(C(=O)O)c(Nc3cccc(Cl)c3Cl)c2cc1OC1C(C)C1	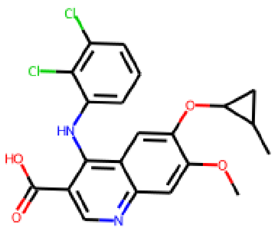	2.0	0.623362	2.525411	2.997881	4	0.774857
**255**	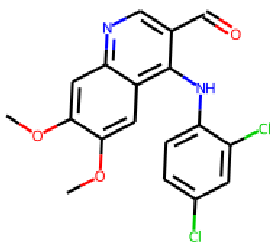	0.640964	COc1cc2ncc(C(=O)O)c(Nc3cccc(Cl)c3Cl)c2cc1OC1C(C)C1	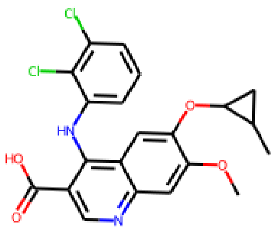	2.0	0.623362	2.525411	2.997881	4	0.774857

	src known mol	similarity	smiles	mol	Class	qed	logP	SAS	rings	kinase inhibition likeliness
**256**	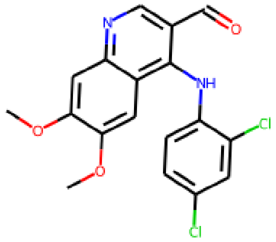	0.640612	COc1cc2ncc(C(=O)O)c(Nc3cccc(Cl)c3Cl)c2cc1OC1C(C)C1	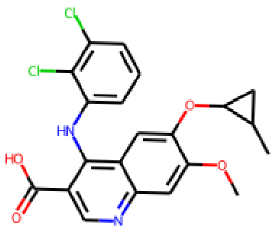	2.0	0.623362	2.525411	2.997881	4	0.774857
**257**	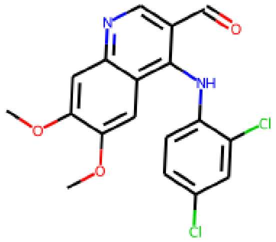	0.640612	COc1cc2ncc(C(=O)O)c(Nc3cccc(Cl)c3Cl)c2cc1OC1C(C)C1	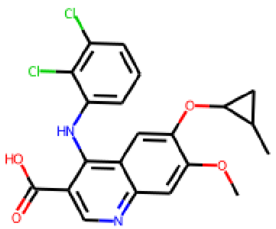	2.0	0.623362	2.525411	2.997881	4	0.774857
**258**	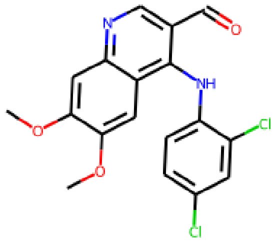	0.640612	COc1cc2ncc(C(=O)O)c(Nc3cccc(Cl)c3Cl)c2cc1OC1C(C)C1	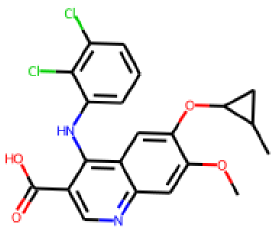	2.0	0.623362	2.525411	2.997881	4	0.774857
**259**	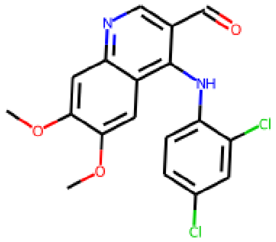	0.640612	COc1cc2ncc(C(=O)O)c(Nc3cccc(Cl)c3Cl)c2cc1OC1C(C)C1	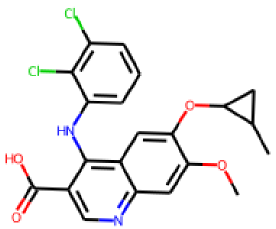	2.0	0.623362	2.525411	2.997881	4	0.774857

	src known mol	similarity	smiles	mol	Class	qed	logP	SAS	rings	kinase inhibition likeliness
**260**	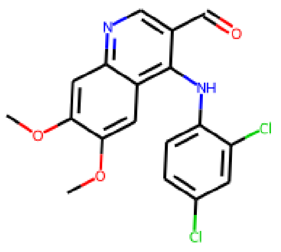	0.640612	COc1cc2ncc(C(=O)O)c(Nc3cccc(Cl)c3Cl)c2cc1OC1C(C)C1	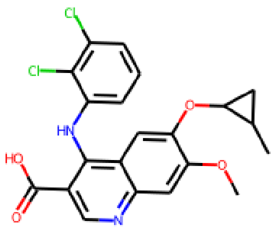	2.0	0.623362	2.525411	2.997881	4	0.774857
**261**	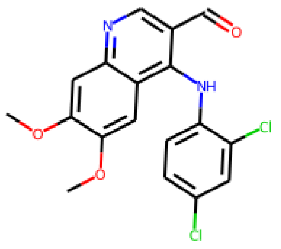	0.640578	COc1cc2ncc(C(=O)O)c(Nc3cccc(Cl)c3Cl)c2cc1OC1C(C)C1	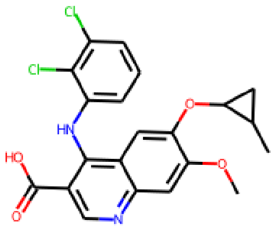	2.0	0.623362	2.525411	2.997881	4	0.774857
**262**	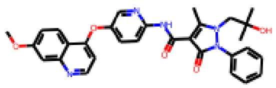	0.639938	COc1ccc2c(=O)cc(CNC(=O)c3c(C)n(C)n(-c4ccccc4)c3=O)nc3cccc2c3c1	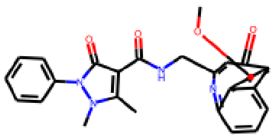	9.0	0.638163	3.995589	2.988471	5	0.774651
**263**	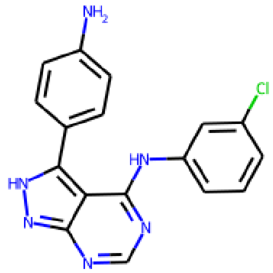	0.639223	c1ccc(Nc2ncnc3n[nH]c(-c4ccccc4)c23)cc1	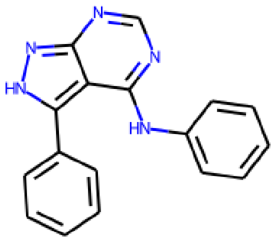	0.0	0.638163	3.995589	2.988471	4	0.800634

	src known mol	similarity	smiles	mol	Class	qed	logP	SAS	rings	kinase inhibition likeliness
**264**	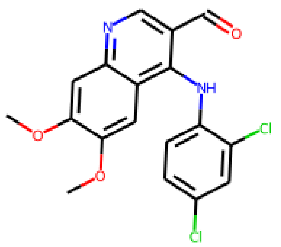	0.639110	COc1cc2ncc(C(=O)O)c(Nc3cccc(Cl)c3Cl)c2cc1OC1C(C)C1	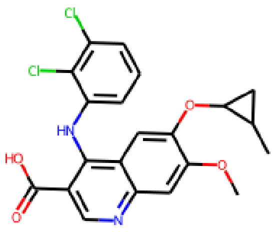	2.0	0.623362	2.525411	2.997881	4	0.774857
**265**	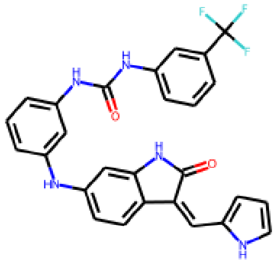	0.637579	COc1ccc(NC(=O)Nc2ccc3c(c2)NC(=O)/C3=C/c2ccc[nH]2)cc1	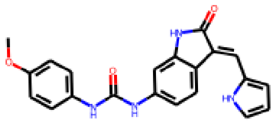	4.0	0.638163	3.995589	2.988471	4	0.768944
**266**	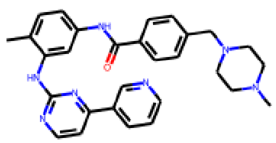	0.637559	Cc1ccc(NC(=O)c2ccccc2)cc1Nc1nccc(-c2cccnc2)n1	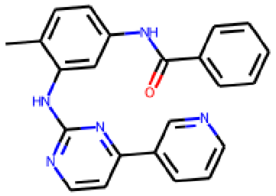	0.0	0.768132	3.608437	2.513170	4	0.854555
**267**	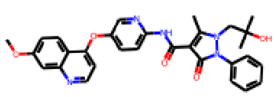	0.636597	COc1ccc2c(=O)cc(CNC(=O)c3c(C)n(C)n(-c4ccccc4)c3=O)nc3cccc2c3c1	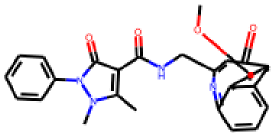	9.0	0.638163	3.995589	2.988471	5	0.774651

	src known mol	similarity	smiles	mol	Class	qed	logP	SAS	rings	kinase inhibition likeliness
**268**	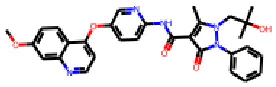	0.636597	COc1ccc2c(=O)cc(CNC(=O)c3c(C)n(C)n(-c4ccccc4)c3=O)nc3cccc2c3c1	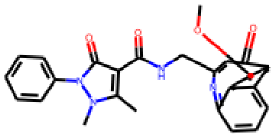	9.0	0.638163	3.995589	2.988471	5	0.774651
**269**	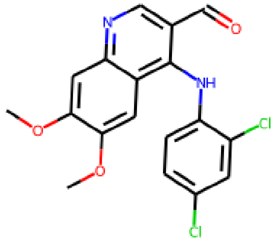	0.636515	COc1cc2ncc(C(=O)O)c(Nc3cccc(Cl)c3Cl)c2cc1OC1C(C)C1	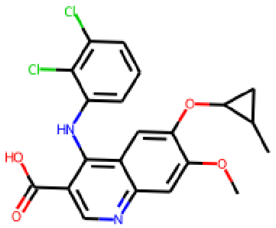	2.0	0.623362	2.525411	2.997881	4	0.774857
**270**	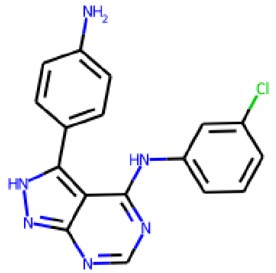	0.635531	c1ccc(Nc2ncnc3n[nH]c(-c4ccccc4)c23)cc1	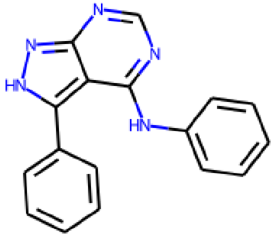	0.0	0.638163	3.995589	2.988471	4	0.800634
**271**	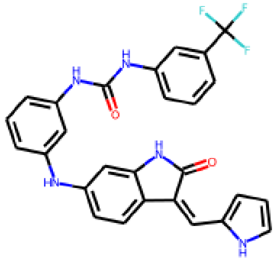	0.634572	COc1ccc(NC(=O)Nc2ccc3c(c2)NC(=O)/C3=C/c2ccc[nH]2)cc1	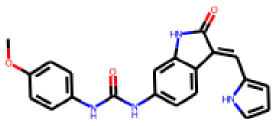	4.0	0.638163	3.995589	2.988471	4	0.768944

	src known mol	similarity	smiles	mol	Class	qed	logP	SAS	rings	kinase inhibition likeliness
**272**	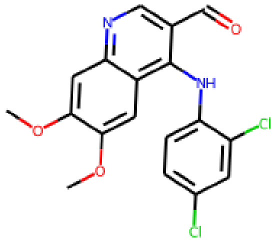	0.634570	COc1cc2ncc(C(=O)O)c(Nc3cccc(Cl)c3Cl)c2cc1OC1C(C)C1	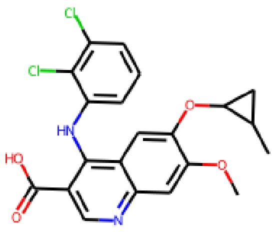	2.0	0.623362	2.525411	2.997881	4	0.774857
**273**	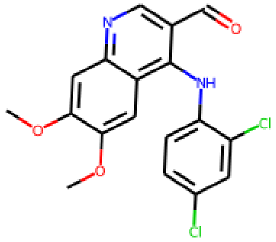	0.632730	COc1cc2ncc(C(=O)O)c(Nc3cccc(Cl)c3Cl)c2cc1OC1C(C)C1	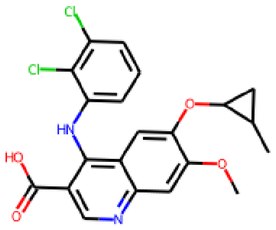	2.0	0.623362	2.525411	2.997881	4	0.774857
**274**	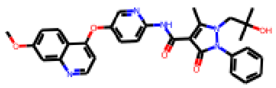	0.631988	COc1ccc2c(=O)cc(CNC(=O)c3c(C)n(C)n(-c4ccccc4)c3=O)nc3cccc2c3c1	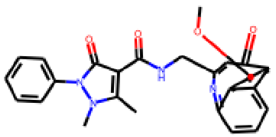	9.0	0.638163	3.995589	2.988471	5	0.774651
**275**	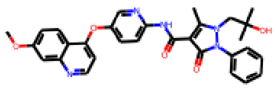	0.631169	COc1ccc2c(=O)cc(CNC(=O)c3c(C)n(C)n(-c4ccccc4)c3=O)nc3cccc2c3c1	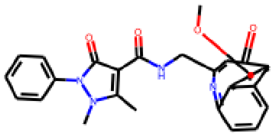	9.0	0.638163	3.995589	2.988471	5	0.774651

	src known mol	similarity	smiles	mol	Class	qed	logP	SAS	rings	kinase inhibition likeliness
**276**	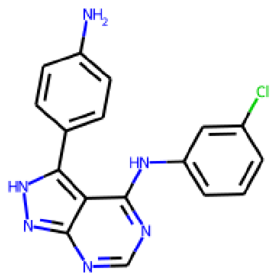	0.630713	c1ccc(Nc2ncnc3n[nH]c(-c4ccccc4)c23)cc1	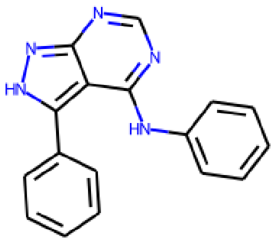	0.0	0.638163	3.995589	2.988471	4	0.800634
**277**	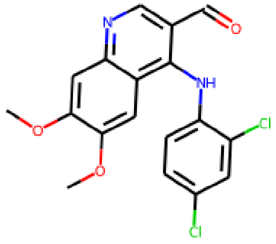	0.630418	COc1cc2ncc(C(=O)O)c(Nc3cccc(Cl)c3Cl)c2cc1OC1C(C)C1	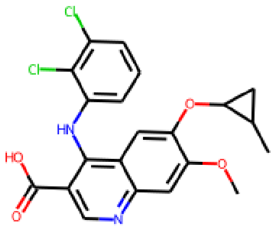	2.0	0.623362	2.525411	2.997881	4	0.774857
**278**	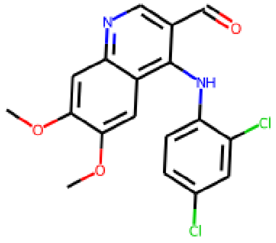	0.630418	COc1cc2ncc(C(=O)O)c(Nc3cccc(Cl)c3Cl)c2cc1OC1C(C)C1	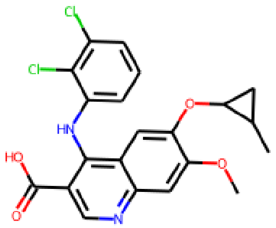	2.0	0.623362	2.525411	2.997881	4	0.774857
**279**	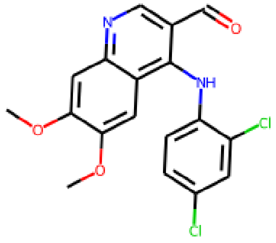	0.630292	COc1cc2ncc(C(=O)O)c(Nc3cccc(Cl)c3Cl)c2cc1OC1C(C)C1	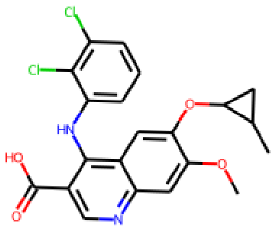	2.0	0.623362	2.525411	2.997881	4	0.774857

	src known mol	similarity	smiles	mol	Class	qed	logP	SAS	rings	kinase inhibition likeliness
**280**	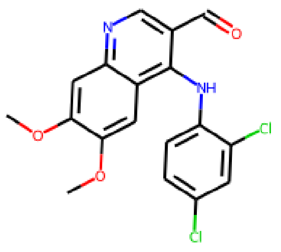	0.630292	COc1cc2ncc(C(=O)O)c(Nc3cccc(Cl)c3Cl)c2cc1OC1C(C)C1	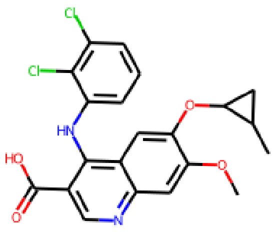	2.0	0.623362	2.525411	2.997881	4	0.774857
**281**	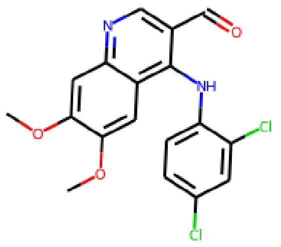	0.630212	COc1cc2ncc(C(=O)O)c(Nc3cccc(Cl)c3Cl)c2cc1OC1C(C)C1	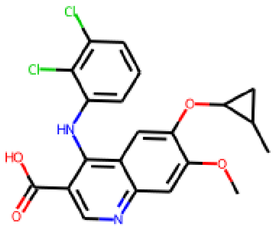	2.0	0.623362	2.525411	2.997881	4	0.774857
**282**	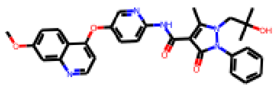	0.629726	COc1ccc2c(=O)cc(CNC(=O)c3c(C)n(C)n(-c4ccccc4)c3=O)nc3cccc2c3c1	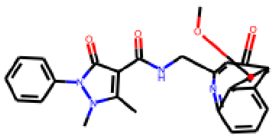	9.0	0.638163	3.995589	2.988471	5	0.774651
**283**	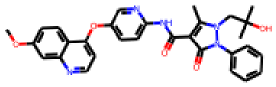	0.629726	COc1ccc2c(=O)cc(CNC(=O)c3c(C)n(C)n(-c4ccccc4)c3=O)nc3cccc2c3c1	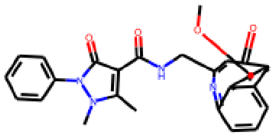	9.0	0.638163	3.995589	2.988471	5	0.774651

	src known mol	similarity	smiles	mol	Class	qed	logP	SAS	rings	kinase inhibition likeliness
**284**	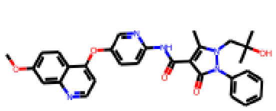	0.629726	COc1ccc2c(=O)cc(CNC(=O)c3c(C)n(C)n(-c4ccccc4)c3=O)nc3cccc2c3c1	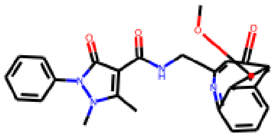	9.0	0.638163	3.995589	2.988471	5	0.774651
**285**	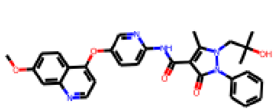	0.629726	COc1ccc2c(=O)cc(CNC(=O)c3c(C)n(C)n(-c4ccccc4)c3=O)nc3cccc2c3c1	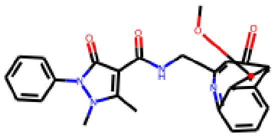	9.0	0.638163	3.995589	2.988471	5	0.774651
**286**	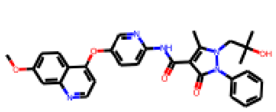	0.629726	COc1ccc2c(=O)cc(CNC(=O)c3c(C)n(C)n(-c4ccccc4)c3=O)nc3cccc2c3c1	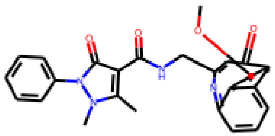	9.0	0.638163	3.995589	2.988471	5	0.774651
**287**	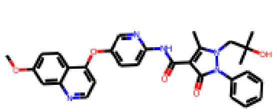	0.629726	COc1ccc2c(=O)cc(CNC(=O)c3c(C)n(C)n(-c4ccccc4)c3=O)nc3cccc2c3c1	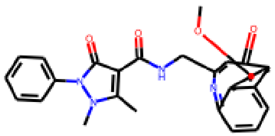	9.0	0.638163	3.995589	2.988471	5	0.774651

	src known mol	similarity	smiles	mol	Class	qed	logP	SAS	rings	kinase inhibition likeliness
**288**	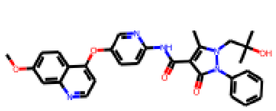	0.629726	COc1ccc2c(=O)cc(CNC(=O)c3c(C)n(C)n(-c4ccccc4)c3=O)nc3cccc2c3c1	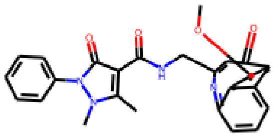	9.0	0.638163	3.995589	2.988471	5	0.774651
**289**	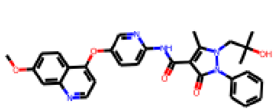	0.629726	COc1ccc2c(=O)cc(CNC(=O)c3c(C)n(C)n(-c4ccccc4)c3=O)nc3cccc2c3c1	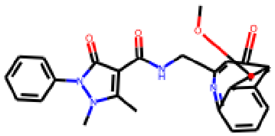	9.0	0.638163	3.995589	2.988471	5	0.774651
**290**	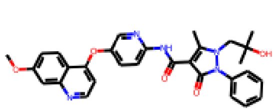	0.629726	COc1ccc2c(=O)cc(CNC(=O)c3c(C)n(C)n(-c4ccccc4)c3=O)nc3cccc2c3c1	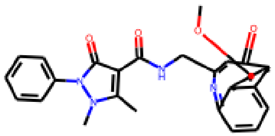	9.0	0.638163	3.995589	2.988471	5	0.774651
**291**	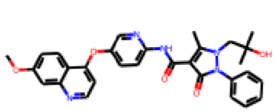	0.629726	COc1ccc2c(=O)cc(CNC(=O)c3c(C)n(C)n(-c4ccccc4)c3=O)nc3cccc2c3c1	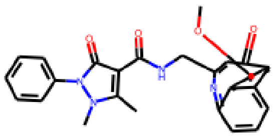	9.0	0.638163	3.995589	2.988471	5	0.774651

	src known mol	similarity	smiles	mol	Class	qed	logP	SAS	rings	kinase inhibition likeliness
**292**	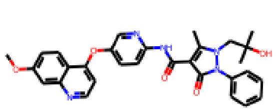	0.629726	COc1ccc2c(=O)cc(CNC(=O)c3c(C)n(C)n(-c4ccccc4)c3=O)nc3cccc2c3c1	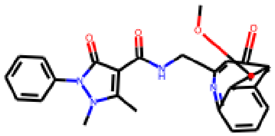	9.0	0.638163	3.995589	2.988471	5	0.774651
**293**	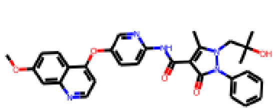	0.629726	COc1ccc2c(=O)cc(CNC(=O)c3c(C)n(C)n(-c4ccccc4)c3=O)nc3cccc2c3c1	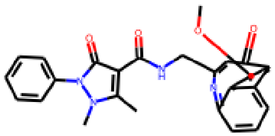	9.0	0.638163	3.995589	2.988471	5	0.774651
**294**	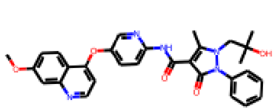	0.629726	COc1ccc2c(=O)cc(CNC(=O)c3c(C)n(C)n(-c4ccccc4)c3=O)nc3cccc2c3c1	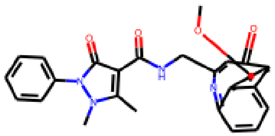	9.0	0.638163	3.995589	2.988471	5	0.774651
**295**	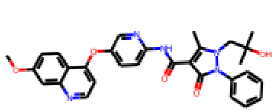	0.629726	COc1ccc2c(=O)cc(CNC(=O)c3c(C)n(C)n(-c4ccccc4)c3=O)nc3cccc2c3c1	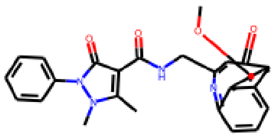	9.0	0.638163	3.995589	2.988471	5	0.774651

	src known mol	similarity	smiles	mol	Class	qed	logP	SAS	rings	kinase inhibition likeliness
**296**	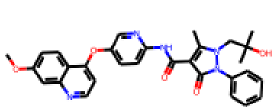	0.629726	COc1ccc2c(=O)cc(CNC(=O)c3c(C)n(C)n(-c4ccccc4)c3=O)nc3cccc2c3c1	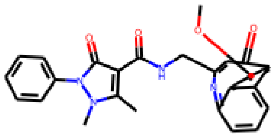	9.0	0.638163	3.995589	2.988471	5	0.774651
**297**	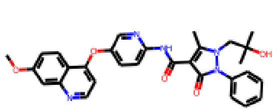	0.629726	COc1ccc2c(=O)cc(CNC(=O)c3c(C)n(C)n(-c4ccccc4)c3=O)nc3cccc2c3c1	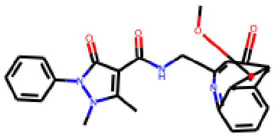	9.0	0.638163	3.995589	2.988471	5	0.774651
**298**	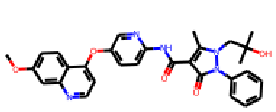	0.629726	COc1ccc2c(=O)cc(CNC(=O)c3c(C)n(C)n(-c4ccccc4)c3=O)nc3cccc2c3c1	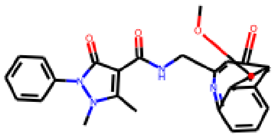	9.0	0.638163	3.995589	2.988471	5	0.774651
**299**	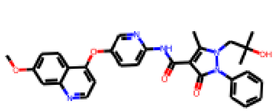	0.629726	COc1ccc2c(=O)cc(CNC(=O)c3c(C)n(C)n(-c4ccccc4)c3=O)nc3cccc2c3c1	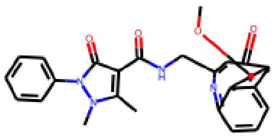	9.0	0.638163	3.995589	2.988471	5	0.774651

	src known mol	similarity	smiles	mol	Class	qed	logP	SAS	rings	kinase inhibition likeliness
**300**	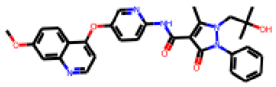	0.629726	COc1ccc2c(=O)cc(CNC(=O)c3c(C)n(C)n(-c4ccccc4)c3=O)nc3cccc2c3c1	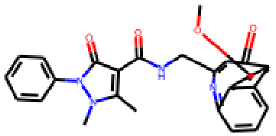	9.0	0.638163	3.995589	2.988471	5	0.774651
**301**	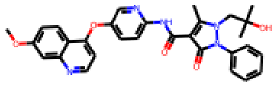	0.629726	COc1ccc2c(=O)cc(CNC(=O)c3c(C)n(C)n(-c4ccccc4)c3=O)nc3cccc2c3c1	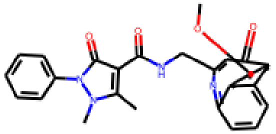	9.0	0.638163	3.995589	2.988471	5	0.774651
**302**	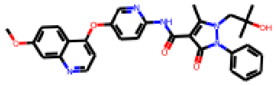	0.629726	COc1ccc2c(=O)cc(CNC(=O)c3c(C)n(C)n(-c4ccccc4)c3=O)nc3cccc2c3c1	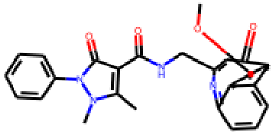	9.0	0.638163	3.995589	2.988471	5	0.774651
**303**	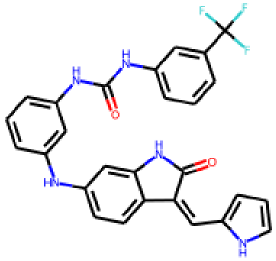	0.627782	COc1ccc(NC(=O)Nc2ccc3c(c2)NC(=O)/C3=C/c2ccc[nH]2)cc1	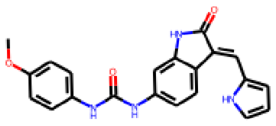	4.0	0.638163	3.995589	2.988471	4	0.768944

	src known mol	similarity	smiles	mol	Class	qed	logP	SAS	rings	kinase inhibition likeliness
**304**	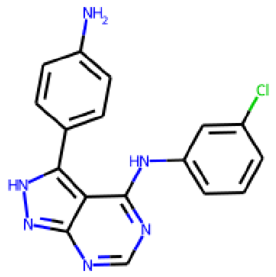	0.627698	c1ccc(Nc2ncnc3n[nH]c(-c4ccccc4)c23)cc1	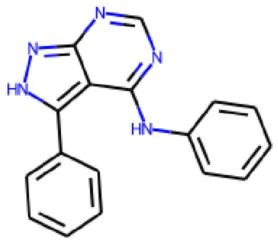	0.0	0.638163	3.995589	2.988471	4	0.800634
**305**	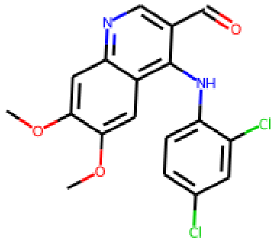	0.626603	COc1cc2ncc(C(=O)O)c(Nc3cccc(Cl)c3Cl)c2cc1OC1C(C)C1	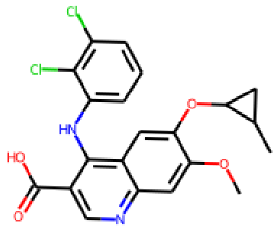	2.0	0.623362	2.525411	2.997881	4	0.774857
**306**	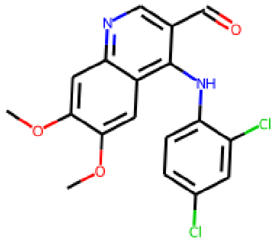	0.626603	COc1cc2ncc(C(=O)O)c(Nc3cccc(Cl)c3Cl)c2cc1OC1C(C)C1	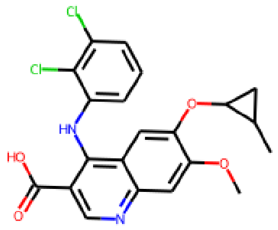	2.0	0.623362	2.525411	2.997881	4	0.774857
**307**	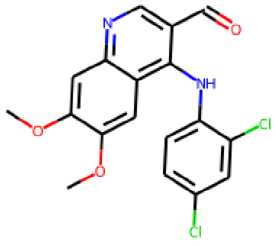	0.626247	COc1cc2ncc(C(=O)O)c(Nc3cccc(Cl)c3Cl)c2cc1OC1C(C)C1	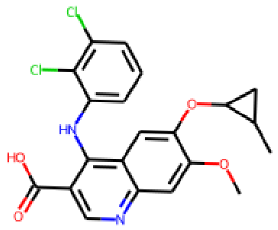	2.0	0.623362	2.525411	2.997881	4	0.774857

	src known mol	similarity	smiles	mol	Class	qed	logP	SAS	rings	kinase inhibition likeliness
**308**	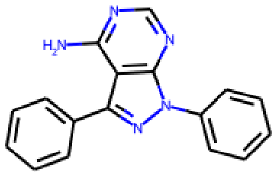	0.625634	Cc1ccc(C(=O)Oc2ccon2)cc1-c1ncnc2c1cnn2-c1ccccc1	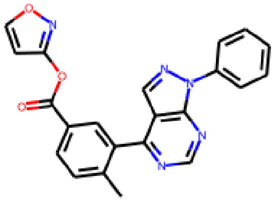	8.0	0.635315	4.542409	3.856266	5	0.760056
**309**	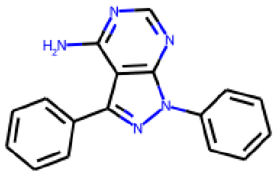	0.625634	Cc1ccc(C(=O)Oc2ccon2)cc1-c1ncnc2c1cnn2-c1ccccc1	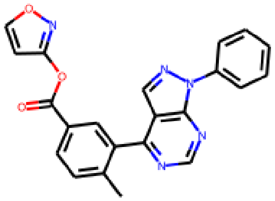	8.0	0.635315	4.542409	3.856266	5	0.760056
**310**	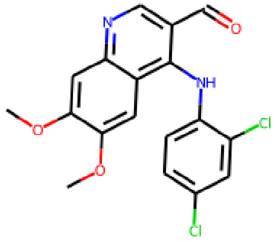	0.625474	COc1cc2ncc(C(=O)O)c(Nc3cccc(Cl)c3Cl)c2cc1OC1C(C)C1	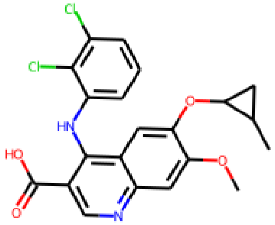	2.0	0.623362	2.525411	2.997881	4	0.774857
**311**	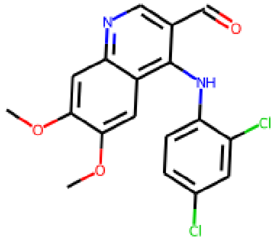	0.625099	COc1cc2ncc(C(=O)O)c(Nc3cccc(Cl)c3Cl)c2cc1OC1C(C)C1	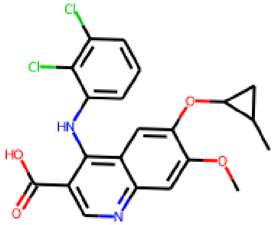	2.0	0.623362	2.525411	2.997881	4	0.774857

	src known mol	similarity	smiles	mol	Class	qed	logP	SAS	rings	kinase inhibition likeliness
**312**	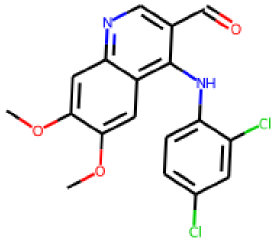	0.625099	COc1cc2ncc(C(=O)O)c(Nc3cccc(Cl)c3Cl)c2cc1OC1C(C)C1	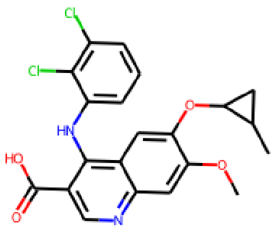	2.0	0.623362	2.525411	2.997881	4	0.774857
**313**	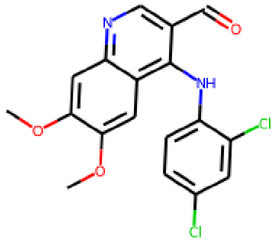	0.625099	COc1cc2ncc(C(=O)O)c(Nc3cccc(Cl)c3Cl)c2cc1OC1C(C)C1	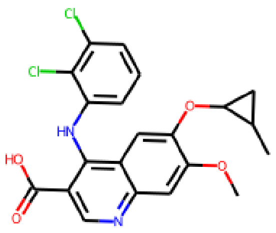	2.0	0.623362	2.525411	2.997881	4	0.774857
**314**	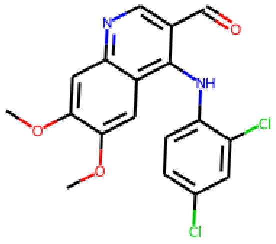	0.625099	COc1cc2ncc(C(=O)O)c(Nc3cccc(Cl)c3Cl)c2cc1OC1C(C)C1	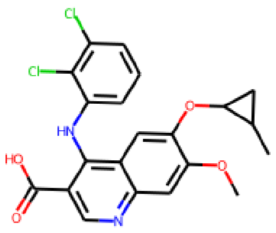	2.0	0.623362	2.525411	2.997881	4	0.774857
**315**	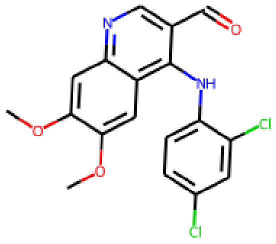	0.625000	COc1cc2ncc(C(=O)O)c(Nc3cccc(Cl)c3Cl)c2cc1OC1C(C)C1	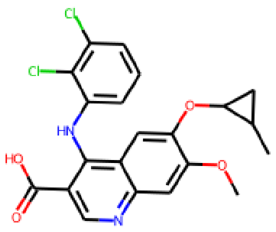	2.0	0.623362	2.525411	2.997881	4	0.774857

	src known mol	similarity	smiles	mol	Class	qed	logP	SAS	rings	kinase inhibition likeliness
**316**	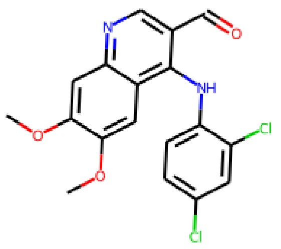	0.625000	COc1cc2ncc(C(=O)O)c(Nc3cccc(Cl)c3Cl)c2cc1OC1C(C)C1	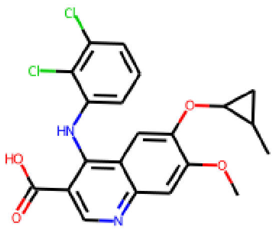	2.0	0.623362	2.525411	2.997881	4	0.774857
**317**	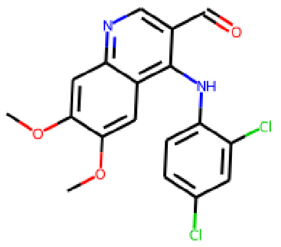	0.625000	COc1cc2ncc(C(=O)O)c(Nc3cccc(Cl)c3Cl)c2cc1OC1C(C)C1	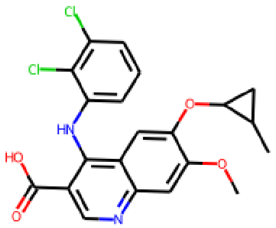	2.0	0.623362	2.525411	2.997881	4	0.774857
**318**	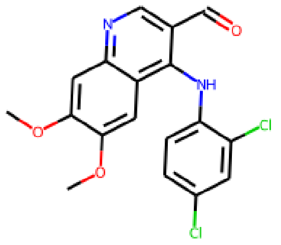	0.625000	COc1cc2ncc(C(=O)O)c(Nc3cccc(Cl)c3Cl)c2cc1OC1C(C)C1	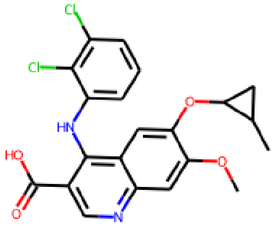	2.0	0.623362	2.525411	2.997881	4	0.774857
**319**	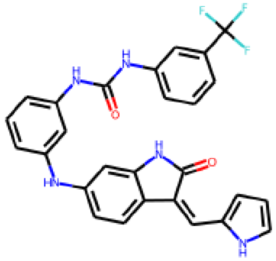	0.624815	COc1ccc(NC(=O)Nc2ccc3c(c2)NC(=O)/C3=C/c2ccc[nH]2)cc1	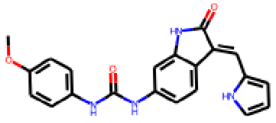	4.0	0.638163	3.995589	2.988471	4	0.768944

	src known mol	similarity	smiles	mol	Class	qed	logP	SAS	rings	kinase inhibition likeliness
**320**	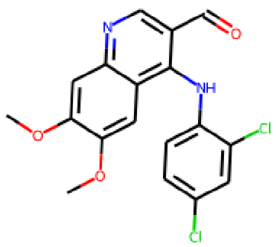	0.624803	COc1cc2ncc(C(=O)O)c(Nc3cccc(Cl)c3Cl)c2cc1OC1C(C)C1	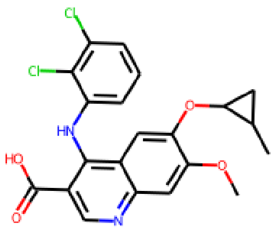	2.0	0.623362	2.525411	2.997881	4	0.774857
**321**	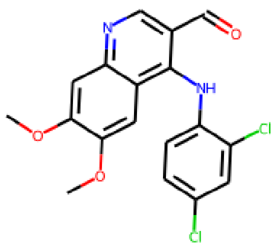	0.624803	COc1cc2ncc(C(=O)O)c(Nc3cccc(Cl)c3Cl)c2cc1OC1C(C)C1	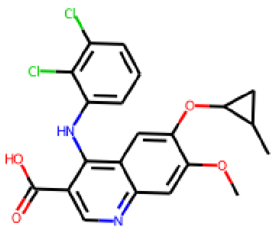	2.0	0.623362	2.525411	2.997881	4	0.774857
**322**	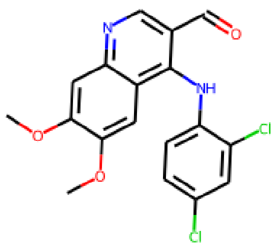	0.624716	COc1cc2ncc(C(=O)O)c(Nc3cccc(Cl)c3Cl)c2cc1OC1C(C)C1	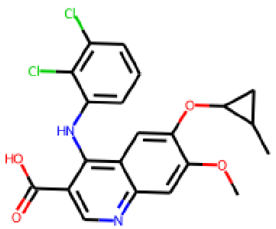	2.0	0.623362	2.525411	2.997881	4	0.774857
**323**	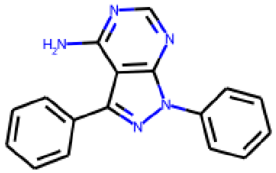	0.624108	Cc1ccc(C(=O)Oc2ccon2)cc1-c1ncnc2c1cnn2-c1ccccc1	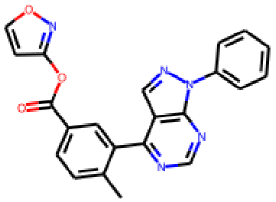	8.0	0.635315	4.542409	3.856266	5	0.760056

	src known mol	similarity	smiles	mol	Class	qed	logP	SAS	rings	kinase inhibition likeliness
**324**	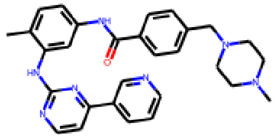	0.623729	Cc1ccc(NC(=O)c2ccccc2)cc1Nc1nccc(-c2cccnc2)n1	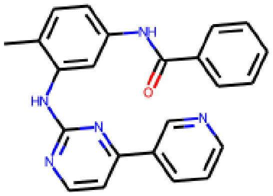	0.0	0.768132	3.608437	2.513170	4	0.854555
**325**	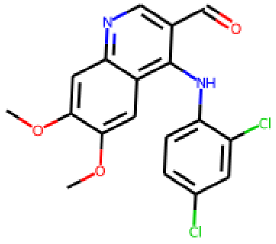	0.623406	COc1cc2ncc(C(=O)O)c(Nc3cccc(Cl)c3Cl)c2cc1OC1C(C)C1	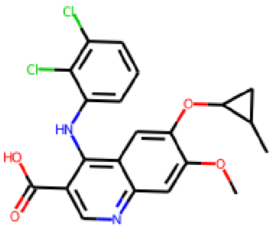	2.0	0.623362	2.525411	2.997881	4	0.774857
**326**	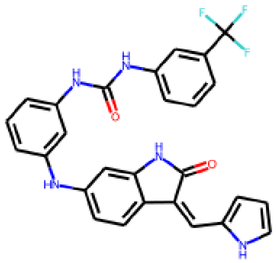	0.623267	COc1ccc(NC(=O)Nc2ccc3c(c2)NC(=O)/C3=C/c2ccc[nH]2)cc1	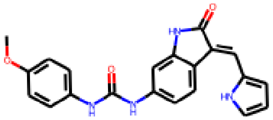	4.0	0.638163	3.995589	2.988471	4	0.768944
**327**	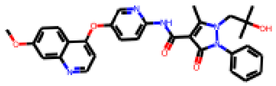	0.623061	COc1ccc2c(=O)cc(CNC(=O)c3c(C)n(C)n(-c4ccccc4)c3=O)nc3cccc2c3c1	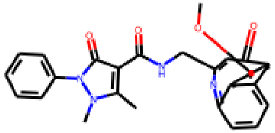	9.0	0.638163	3.995589	2.988471	5	0.774651

	src known mol	similarity	smiles	mol	Class	qed	logP	SAS	rings	kinase inhibition likeliness
**328**	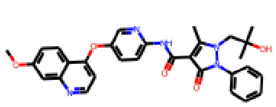	0.623061	COc1ccc2c(=O)cc(CNC(=O)c3c(C)n(C)n(-c4ccccc4)c3=O)nc3cccc2c3c1	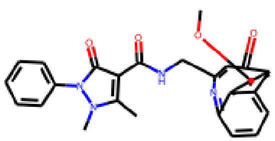	9.0	0.638163	3.995589	2.988471	5	0.774651
**329**	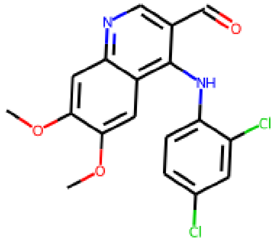	0.622773	COc1cc2ncc(C(=O)O)c(Nc3cccc(Cl)c3Cl)c2cc1OC1C(C)C1	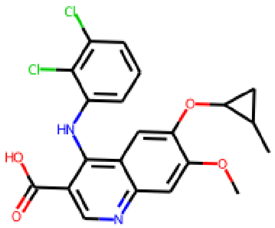	2.0	0.623362	2.525411	2.997881	4	0.774857
**330**	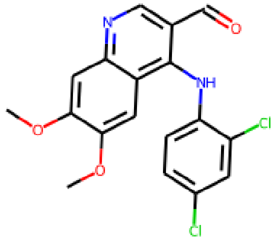	0.622773	COc1cc2ncc(C(=O)O)c(Nc3cccc(Cl)c3Cl)c2cc1OC1C(C)C1	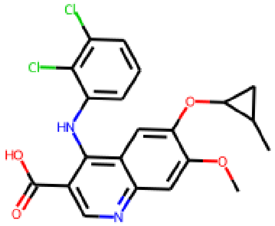	2.0	0.623362	2.525411	2.997881	4	0.774857
**331**	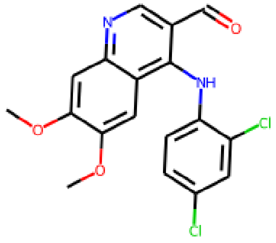	0.622496	COc1cc2ncc(C(=O)O)c(Nc3cccc(Cl)c3Cl)c2cc1OC1C(C)C1	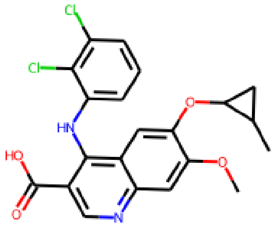	2.0	0.623362	2.525411	2.997881	4	0.774857

	src known mol	similarity	smiles	mol	Class	qed	logP	SAS	rings	kinase inhibition likeliness
**332**	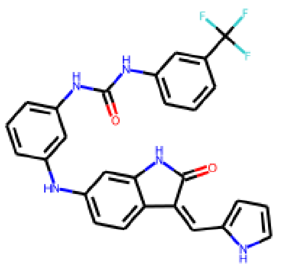	0.622434	COc1ccc(NC(=O)Nc2ccc3c(c2)NC(=O)/C3=C/c2ccc[nH]2)cc1	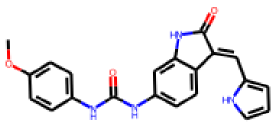	4.0	0.638163	3.995589	2.988471	4	0.768944
**333**	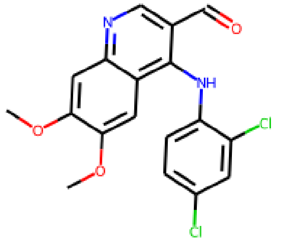	0.622188	COc1cc2ncc(C(=O)O)c(Nc3cccc(Cl)c3Cl)c2cc1OC1C(C)C1	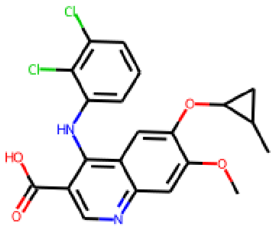	2.0	0.623362	2.525411	2.997881	4	0.774857
**334**	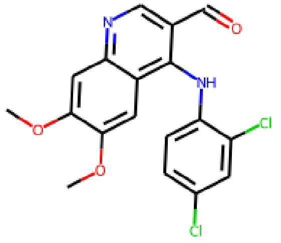	0.620550	COc1cc2ncc(C(=O)O)c(Nc3cccc(Cl)c3Cl)c2cc1OC1C(C)C1	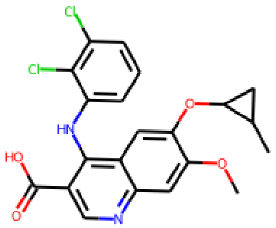	2.0	0.623362	2.525411	2.997881	4	0.774857
**335**	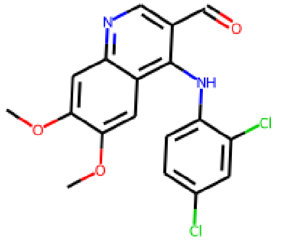	0.620486	COc1cc2ncc(C(=O)O)c(Nc3cccc(Cl)c3Cl)c2cc1OC1C(C)C1	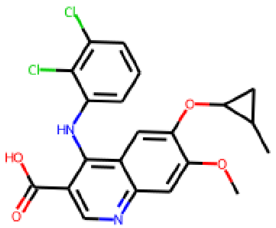	2.0	0.623362	2.525411	2.997881	4	0.774857

	src known mol	similarity	smiles	mol	Class	qed	logP	SAS	rings	kinase inhibition likeliness
**336**	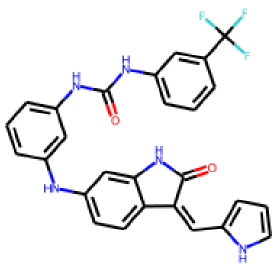	0.620427	COc1ccc(NC(=O)Nc2ccc3c(c2)NC(=O)/C3=C/c2ccc[nH]2)cc1	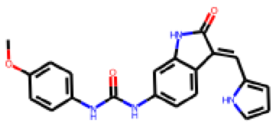	4.0	0.638163	3.995589	2.988471	4	0.768944
**337**	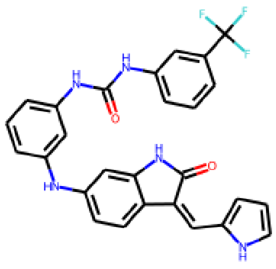	0.620427	COc1ccc(NC(=O)Nc2ccc3c(c2)NC(=O)/C3=C/c2ccc[nH]2)cc1	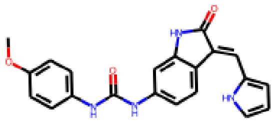	4.0	0.638163	3.995589	2.988471	4	0.768944
**338**	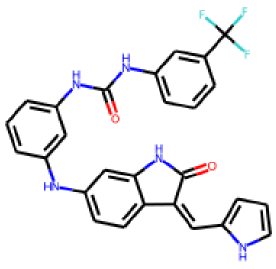	0.620427	COc1ccc(NC(=O)Nc2ccc3c(c2)NC(=O)/C3=C/c2ccc[nH]2)cc1	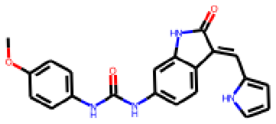	4.0	0.638163	3.995589	2.988471	4	0.768944
**339**	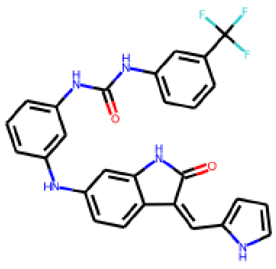	0.620427	COc1ccc(NC(=O)Nc2ccc3c(c2)NC(=O)/C3=C/c2ccc[nH]2)cc1	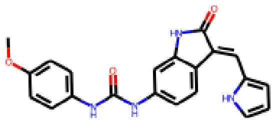	4.0	0.638163	3.995589	2.988471	4	0.768944

	src known mol	similarity	smiles	mol	Class	qed	logP	SAS	rings	kinase inhibition likeliness
**340**	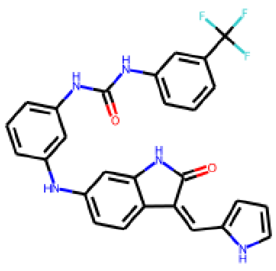	0.620427	COc1ccc(NC(=O)Nc2ccc3c(c2)NC(=O)/C3=C/c2ccc[nH]2)cc1	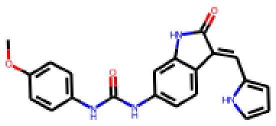	4.0	0.638163	3.995589	2.988471	4	0.768944
**341**	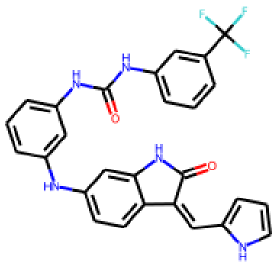	0.620427	COc1ccc(NC(=O)Nc2ccc3c(c2)NC(=O)/C3=C/c2ccc[nH]2)cc1	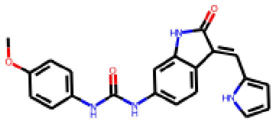	4.0	0.638163	3.995589	2.988471	4	0.768944
**342**	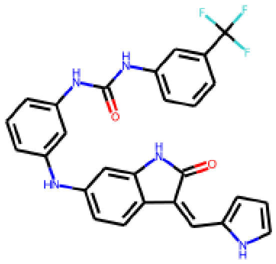	0.620427	COc1ccc(NC(=O)Nc2ccc3c(c2)NC(=O)/C3=C/c2ccc[nH]2)cc1	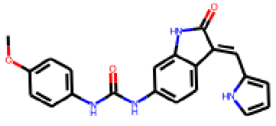	4.0	0.638163	3.995589	2.988471	4	0.768944
**343**	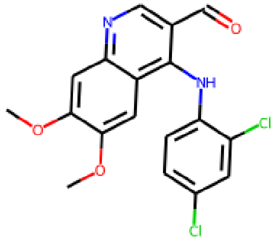	0.620321	COc1cc2ncc(C(=O)O)c(Nc3cccc(Cl)c3Cl)c2cc1OC1C(C)C1	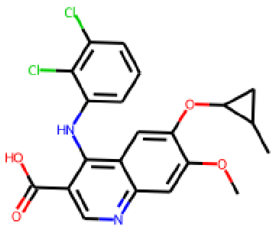	2.0	0.623362	2.525411	2.997881	4	0.774857

	src known mol	similarity	smiles	mol	Class	qed	logP	SAS	rings	kinase inhibition likeliness
**344**	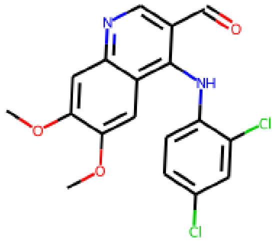	0.620321	COc1cc2ncc(C(=O)O)c(Nc3cccc(Cl)c3Cl)c2cc1OC1C(C)C1	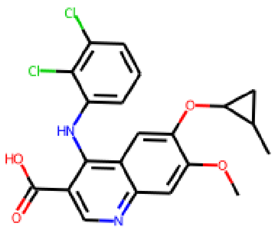	2.0	0.623362	2.525411	2.997881	4	0.774857
**345**	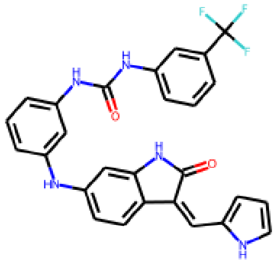	0.620074	COc1ccc(NC(=O)Nc2ccc3c(c2)NC(=O)/C3=C/c2ccc[nH]2)cc1	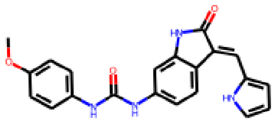	4.0	0.638163	3.995589	2.988471	4	0.768944
**346**	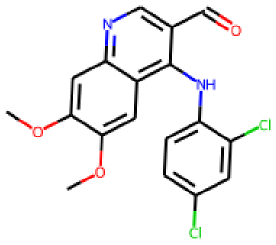	0.619910	COc1cc2ncc(C(=O)O)c(Nc3cccc(Cl)c3Cl)c2cc1OC1C(C)C1	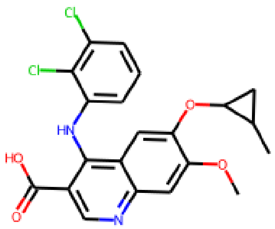	2.0	0.623362	2.525411	2.997881	4	0.774857
**347**	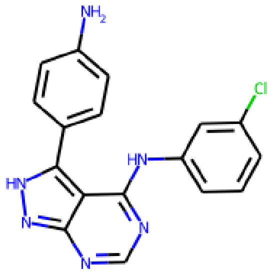	0.619343	c1ccc(Nc2ncnc3n[nH]c(-c4ccccc4)c23)cc1	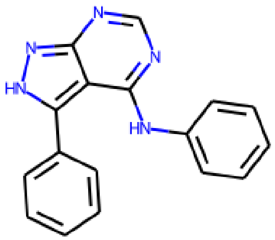	0.0	0.638163	3.995589	2.988471	4	0.800634

	src known mol	similarity	smiles	mol	Class	qed	logP	SAS	rings	kinase inhibition likeliness
**348**	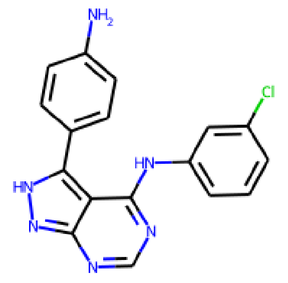	0.618492	c1ccc(Nc2ncnc3n[nH]c(-c4ccccc4)c23)cc1	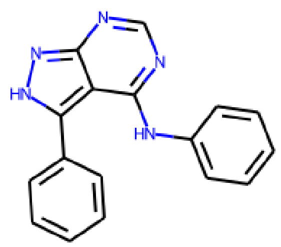	0.0	0.638163	3.995589	2.988471	4	0.800634
**349**	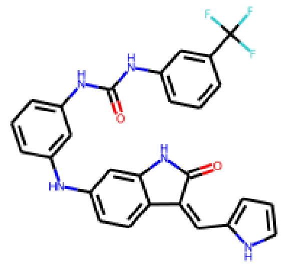	0.618480	COc1ccc(NC(=O)Nc2ccc3c(c2)NC(=O)/C3=C/c2ccc[nH]2)cc1	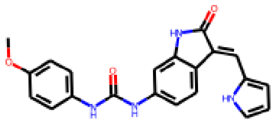	4.0	0.638163	3.995589	2.988471	4	0.768944
**350**	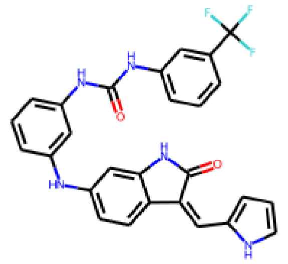	0.618251	COc1ccc(NC(=O)Nc2ccc3c(c2)NC(=O)/C3=C/c2ccc[nH]2)cc1	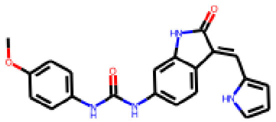	4.0	0.638163	3.995589	2.988471	4	0.768944
**351**	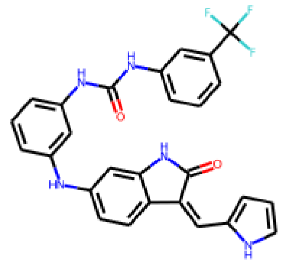	0.617515	COc1ccc(NC(=O)Nc2ccc3c(c2)NC(=O)/C3=C/c2ccc[nH]2)cc1	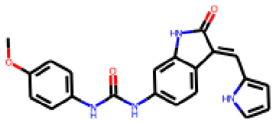	4.0	0.638163	3.995589	2.988471	4	0.768944

	src known mol	similarity	smiles	mol	Class	qed	logP	SAS	rings	kinase inhibition likeliness
**352**	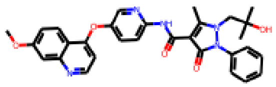	0.617342	COc1ccc2c(=O)cc(CNC(=O)c3c(C)n(C)n(-c4ccccc4)c3=O)nc3cccc2c3c1	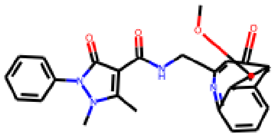	9.0	0.638163	3.995589	2.988471	5	0.774651
**353**	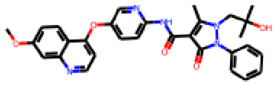	0.617342	COc1ccc2c(=O)cc(CNC(=O)c3c(C)n(C)n(-c4ccccc4)c3=O)nc3cccc2c3c1	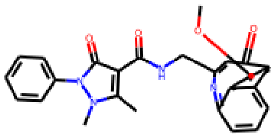	9.0	0.638163	3.995589	2.988471	5	0.774651
**354**	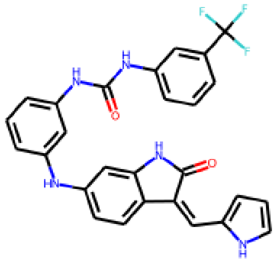	0.617312	COc1ccc(NC(=O)Nc2ccc3c(c2)NC(=O)/C3=C/c2ccc[nH]2)cc1	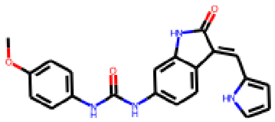	4.0	0.638163	3.995589	2.988471	4	0.768944
**355**	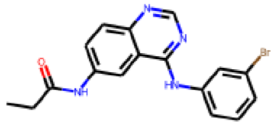	0.616923	O=C(CNC(=O)Oc1ccccc1)Nc1ccc2ncnc(Nc3cccc(Cl)c3)c2c1	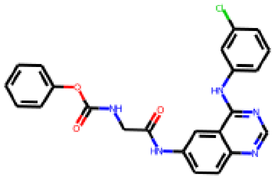	3.0	0.638163	3.995589	2.988471	4	0.793889

	src known mol	similarity	smiles	mol	Class	qed	logP	SAS	rings	kinase inhibition likeliness
**356**	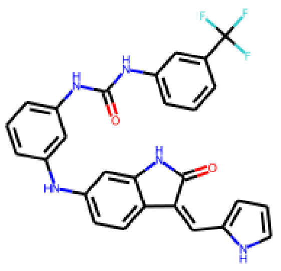	0.616366	COc1ccc(NC(=O)Nc2ccc3c(c2)NC(=O)/C3=C/c2ccc[nH]2)cc1	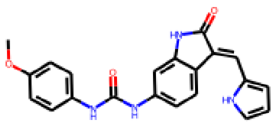	4.0	0.638163	3.995589	2.988471	4	0.768944
**357**	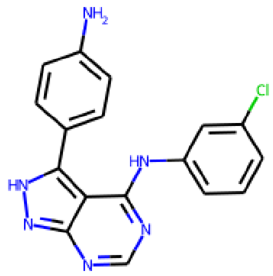	0.615993	c1ccc(Nc2ncnc3n[nH]c(-c4ccccc4)c23)cc1	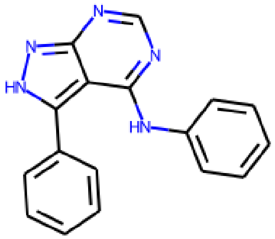	0.0	0.638163	3.995589	2.988471	4	0.800634
**358**	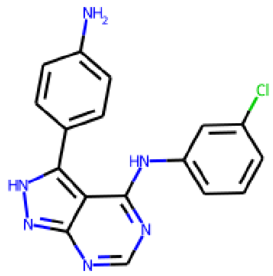	0.615993	c1ccc(Nc2ncnc3n[nH]c(-c4ccccc4)c23)cc1	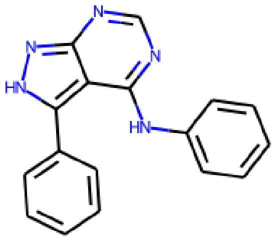	0.0	0.638163	3.995589	2.988471	4	0.800634
**359**	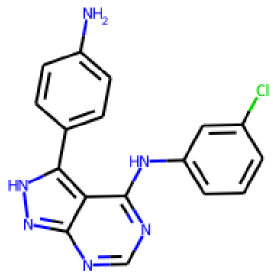	0.615993	c1ccc(Nc2ncnc3n[nH]c(-c4ccccc4)c23)cc1	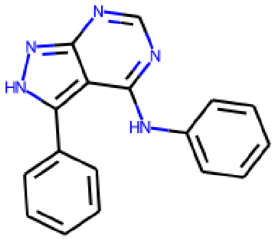	0.0	0.638163	3.995589	2.988471	4	0.800634

	src known mol	similarity	smiles	mol	Class	qed	logP	SAS	rings	kinase inhibition likeliness
**360**	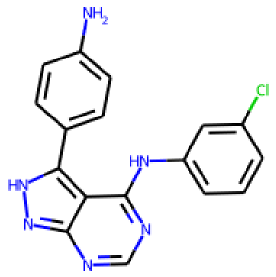	0.615993	c1ccc(Nc2ncnc3n[nH]c(-c4ccccc4)c23)cc1	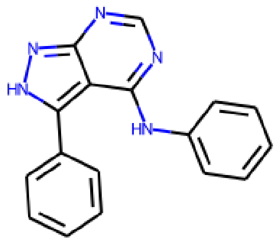	0.0	0.638163	3.995589	2.988471	4	0.800634
**361**	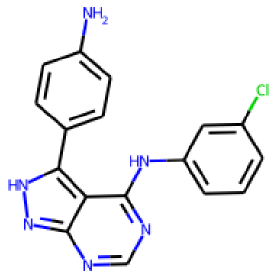	0.615993	c1ccc(Nc2ncnc3n[nH]c(-c4ccccc4)c23)cc1	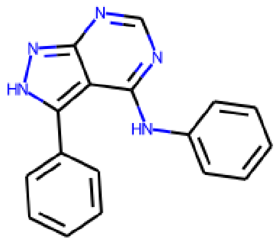	0.0	0.638163	3.995589	2.988471	4	0.800634
**362**	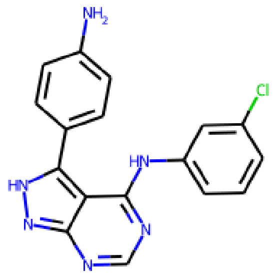	0.615993	c1ccc(Nc2ncnc3n[nH]c(-c4ccccc4)c23)cc1	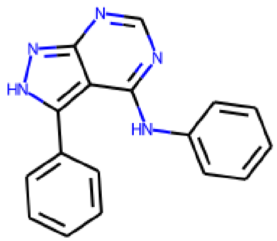	0.0	0.638163	3.995589	2.988471	4	0.800634
**363**	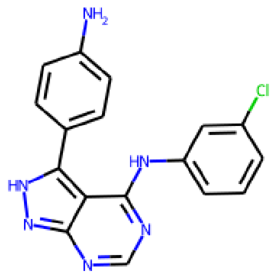	0.615993	c1ccc(Nc2ncnc3n[nH]c(-c4ccccc4)c23)cc1	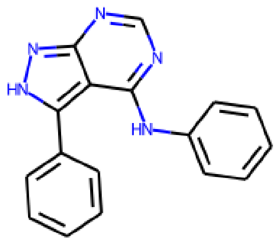	0.0	0.638163	3.995589	2.988471	4	0.800634

	src known mol	similarity	smiles	mol	Class	qed	logP	SAS	rings	kinase inhibition likeliness
**364**	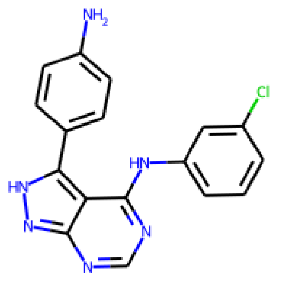	0.615993	c1ccc(Nc2ncnc3n[nH]c(-c4ccccc4)c23)cc1	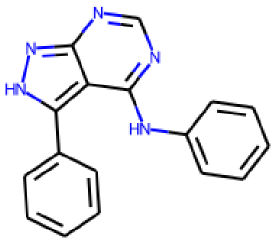	0.0	0.638163	3.995589	2.988471	4	0.800634
**365**	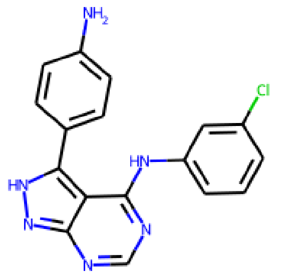	0.615993	c1ccc(Nc2ncnc3n[nH]c(-c4ccccc4)c23)cc1	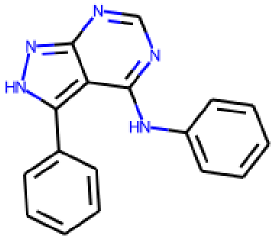	0.0	0.638163	3.995589	2.988471	4	0.800634
**366**	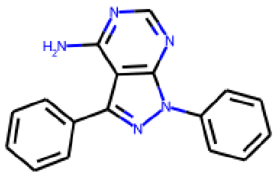	0.615861	Cc1ccc(C(=O)Oc2ccon2)cc1-c1ncnc2c1cnn2-c1ccccc1	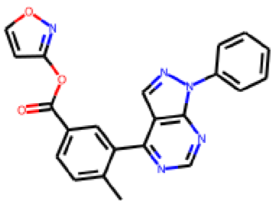	8.0	0.635315	4.542409	3.856266	5	0.760056
**367**	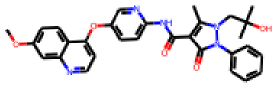	0.615825	COc1ccc2c(=O)cc(CNC(=O)c3c(C)n(C)n(-c4ccccc4)c3=O)nc3cccc2c3c1	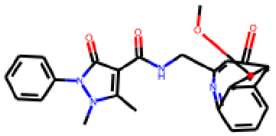	9.0	0.638163	3.995589	2.988471	5	0.774651

	src known mol	similarity	smiles	mol	Class	qed	logP	SAS	rings	kinase inhibition likeliness
**368**	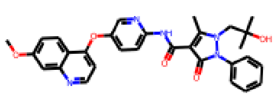	0.615825	COc1ccc2c(=O)cc(CNC(=O)c3c(C)n(C)n(-c4ccccc4)c3=O)nc3cccc2c3c1	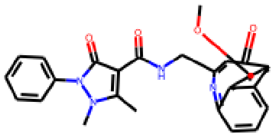	9.0	0.638163	3.995589	2.988471	5	0.774651
**369**	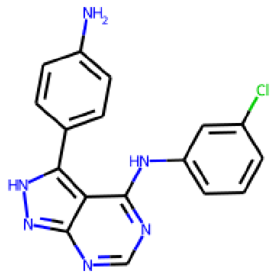	0.615724	c1ccc(Nc2ncnc3n[nH]c(-c4ccccc4)c23)cc1	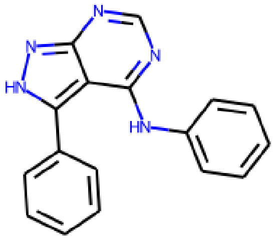	0.0	0.638163	3.995589	2.988471	4	0.800634
**370**	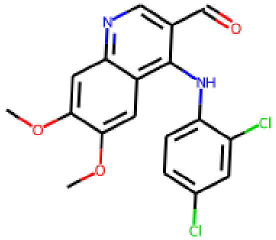	0.615693	COc1cc2ncc(C(=O)O)c(Nc3cccc(Cl)c3Cl)c2cc1OC1C(C)C1	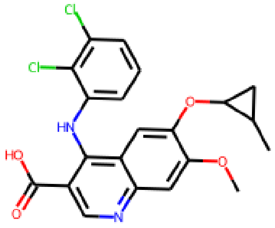	2.0	0.623362	2.525411	2.997881	4	0.774857
**371**	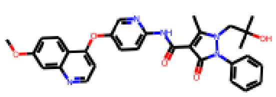	0.615265	COc1ccc2c(=O)cc(CNC(=O)c3c(C)n(C)n(-c4ccccc4)c3=O)nc3cccc2c3c1	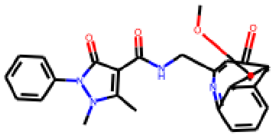	9.0	0.638163	3.995589	2.988471	5	0.774651

	src known mol	similarity	smiles	mol	Class	qed	logP	SAS	rings	kinase inhibition likeliness
**372**	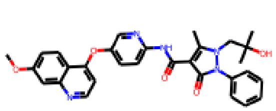	0.615265	COc1ccc2c(=O)cc(CNC(=O)c3c(C)n(C)n(-c4ccccc4)c3=O)nc3cccc2c3c1	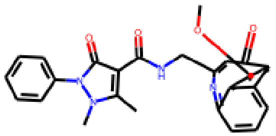	9.0	0.638163	3.995589	2.988471	5	0.774651
**373**	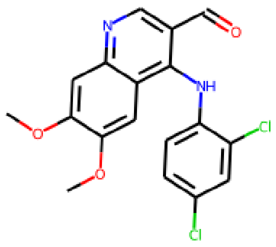	0.615248	COc1cc2ncc(C(=O)O)c(Nc3cccc(Cl)c3Cl)c2cc1OC1C(C)C1	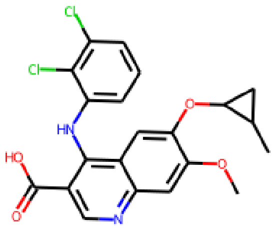	2.0	0.623362	2.525411	2.997881	4	0.774857
**374**	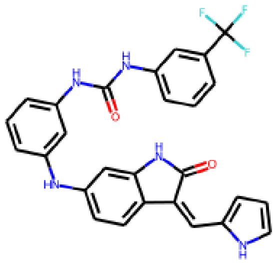	0.615044	COc1ccc(NC(=O)Nc2ccc3c(c2)NC(=O)/C3=C/c2ccc[nH]2)cc1	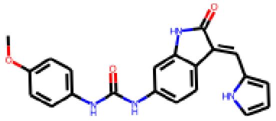	4.0	0.638163	3.995589	2.988471	4	0.768944
**375**	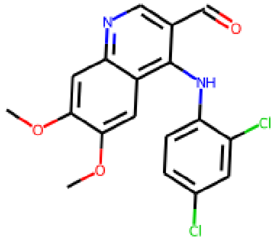	0.615031	COc1cc2ncc(C(=O)O)c(Nc3cccc(Cl)c3Cl)c2cc1OC1C(C)C1	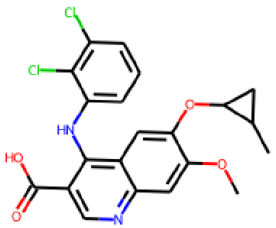	2.0	0.623362	2.525411	2.997881	4	0.774857

	src known mol	similarity	smiles	mol	Class	qed	logP	SAS	rings	kinase inhibition likeliness
**376**	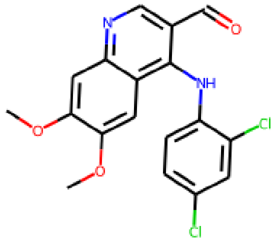	0.615031	COc1cc2ncc(C(=O)O)c(Nc3cccc(Cl)c3Cl)c2cc1OC1C(C)C1	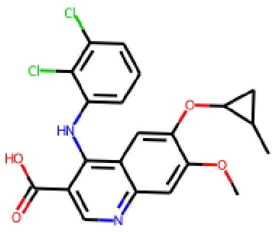	2.0	0.623362	2.525411	2.997881	4	0.774857
**377**	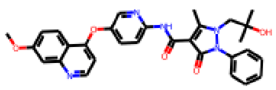	0.614940	COc1ccc2c(=O)cc(CNC(=O)c3c(C)n(C)n(-c4ccccc4)c3=O)nc3cccc2c3c1	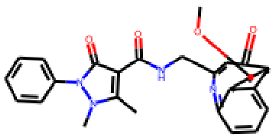	9.0	0.638163	3.995589	2.988471	5	0.774651
**378**	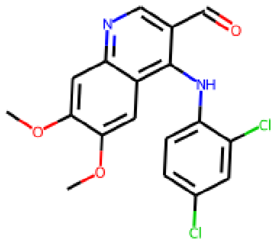	0.614742	COc1cc2ncc(C(=O)O)c(Nc3cccc(Cl)c3Cl)c2cc1OC1C(C)C1	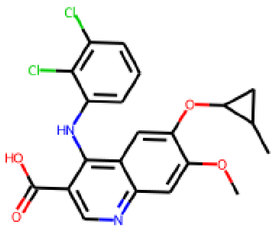	2.0	0.623362	2.525411	2.997881	4	0.774857
**379**	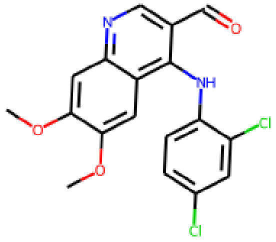	0.614742	COc1cc2ncc(C(=O)O)c(Nc3cccc(Cl)c3Cl)c2cc1OC1C(C)C1	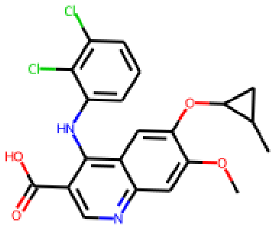	2.0	0.623362	2.525411	2.997881	4	0.774857

	src known mol	similarity	smiles	mol	Class	qed	logP	SAS	rings	kinase inhibition likeliness
**380**	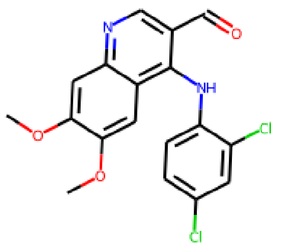	0.614630	COc1cc2ncc(C(=O)O)c(Nc3cccc(Cl)c3Cl)c2cc1OC1C(C)C1	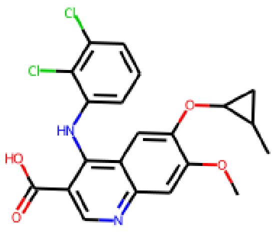	2.0	0.623362	2.525411	2.997881	4	0.774857
**381**	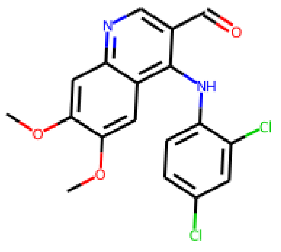	0.614630	COc1cc2ncc(C(=O)O)c(Nc3cccc(Cl)c3Cl)c2cc1OC1C(C)C1	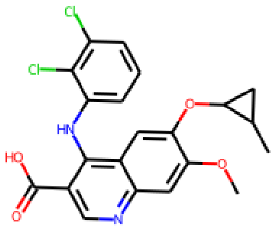	2.0	0.623362	2.525411	2.997881	4	0.774857
**382**	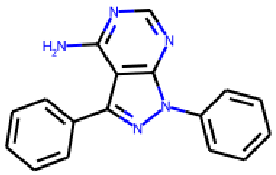	0.614616	Cc1ccc(C(=O)Oc2ccon2)cc1-c1ncnc2c1cnn2-c1ccccc1	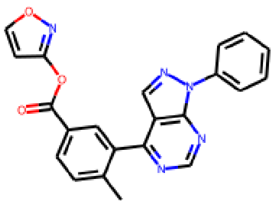	8.0	0.635315	4.542409	3.856266	5	0.760056
**383**	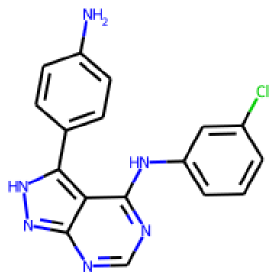	0.614437	c1ccc(Nc2ncnc3n[nH]c(-c4ccccc4)c23)cc1	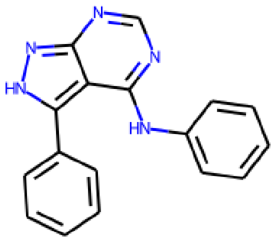	0.0	0.638163	3.995589	2.988471	4	0.800634

	src known mol	similarity	smiles	mol	Class	qed	logP	SAS	rings	kinase inhibition likeliness
**384**	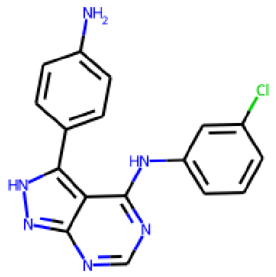	0.614437	c1ccc(Nc2ncnc3n[nH]c(-c4ccccc4)c23)cc1	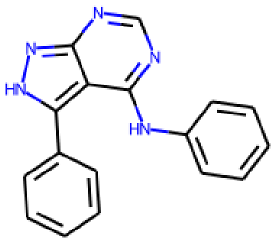	0.0	0.638163	3.995589	2.988471	4	0.800634
**385**	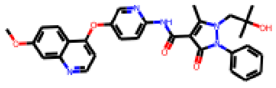	0.614427	COc1ccc2c(=O)cc(CNC(=O)c3c(C)n(C)n(-c4ccccc4)c3=O)nc3cccc2c3c1	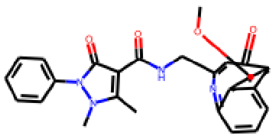	9.0	0.638163	3.995589	2.988471	5	0.774651
**386**	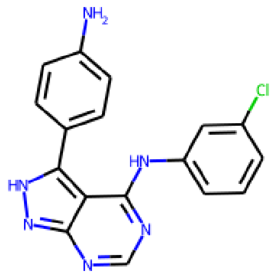	0.614373	c1ccc(Nc2ncnc3n[nH]c(-c4ccccc4)c23)cc1	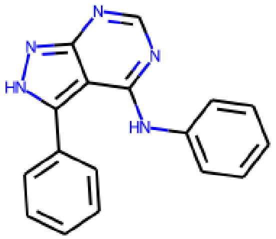	0.0	0.638163	3.995589	2.988471	4	0.800634
**387**	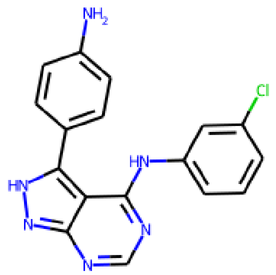	0.614373	c1ccc(Nc2ncnc3n[nH]c(-c4ccccc4)c23)cc1	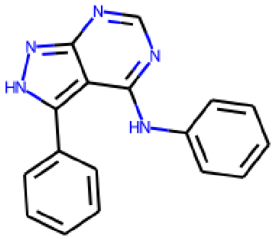	0.0	0.638163	3.995589	2.988471	4	0.800634

	src known mol	similarity	smiles	mol	Class	qed	logP	SAS	rings	kinase inhibition likeliness
**388**	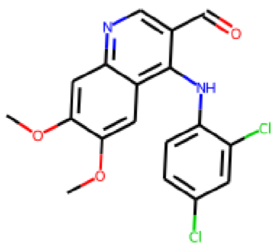	0.614329	COc1cc2ncc(C(=O)O)c(Nc3cccc(Cl)c3Cl)c2cc1OC1C(C)C1	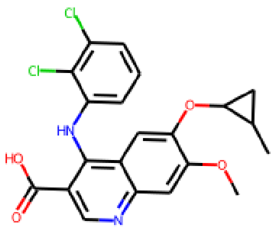	2.0	0.623362	2.525411	2.997881	4	0.774857
**389**	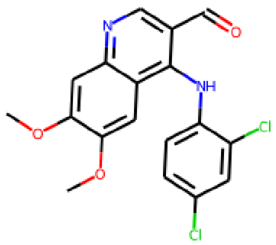	0.614329	COc1cc2ncc(C(=O)O)c(Nc3cccc(Cl)c3Cl)c2cc1OC1C(C)C1	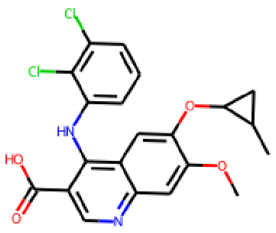	2.0	0.623362	2.525411	2.997881	4	0.774857
**390**	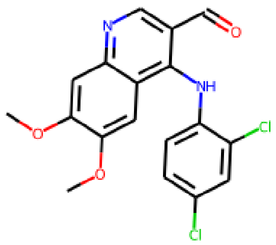	0.614329	COc1cc2ncc(C(=O)O)c(Nc3cccc(Cl)c3Cl)c2cc1OC1C(C)C1	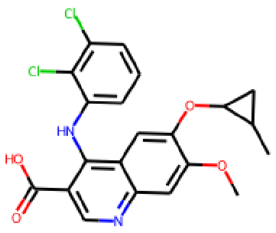	2.0	0.623362	2.525411	2.997881	4	0.774857
**391**	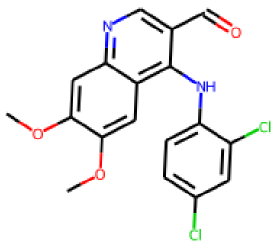	0.614329	COc1cc2ncc(C(=O)O)c(Nc3cccc(Cl)c3Cl)c2cc1OC1C(C)C1	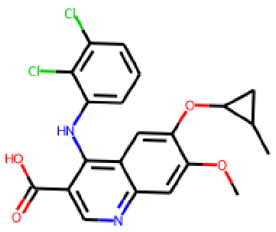	2.0	0.623362	2.525411	2.997881	4	0.774857

	src known mol	similarity	smiles	mol	Class	qed	logP	SAS	rings	kinase inhibition likeliness
**392**	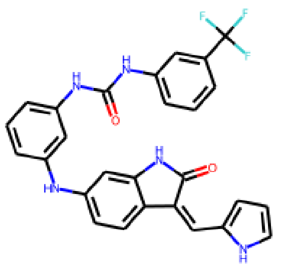	0.614009	COc1ccc(NC(=O)Nc2ccc3c(c2)NC(=O)/C3=C/c2ccc[nH]2)cc1	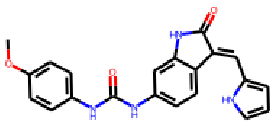	4.0	0.638163	3.995589	2.988471	4	0.768944
**393**	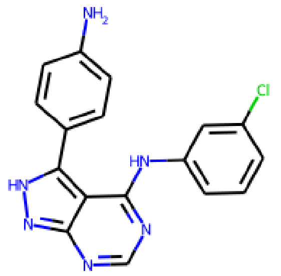	0.613835	c1ccc(Nc2ncnc3n[nH]c(-c4ccccc4)c23)cc1	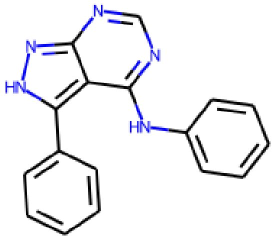	0.0	0.638163	3.995589	2.988471	4	0.800634
**394**	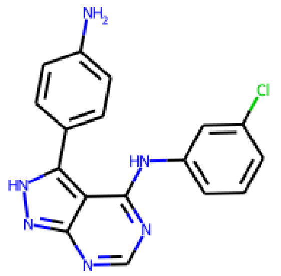	0.613835	c1ccc(Nc2ncnc3n[nH]c(-c4ccccc4)c23)cc1	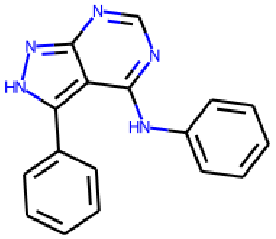	0.0	0.638163	3.995589	2.988471	4	0.800634
**395**	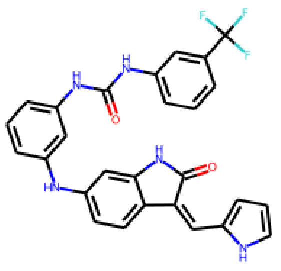	0.613705	COc1ccc(NC(=O)Nc2ccc3c(c2)NC(=O)/C3=C/c2ccc[nH]2)cc1	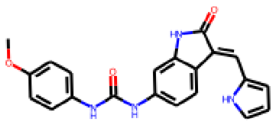	4.0	0.638163	3.995589	2.988471	4	0.768944

	src known mol	similarity	smiles	mol	Class	qed	logP	SAS	rings	kinase inhibition likeliness
**396**	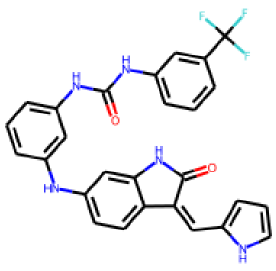	0.613705	COc1ccc(NC(=O)Nc2ccc3c(c2)NC(=O)/C3=C/c2ccc[nH]2)cc1	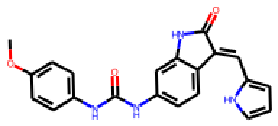	4.0	0.638163	3.995589	2.988471	4	0.768944
**397**	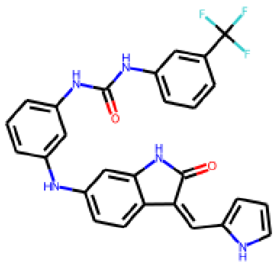	0.613705	COc1ccc(NC(=O)Nc2ccc3c(c2)NC(=O)/C3=C/c2ccc[nH]2)cc1	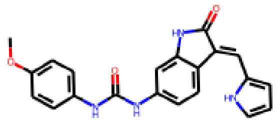	4.0	0.638163	3.995589	2.988471	4	0.768944
**398**	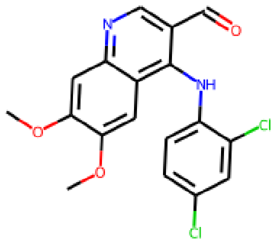	0.613687	COc1cc2ncc(C(=O)O)c(Nc3cccc(Cl)c3Cl)c2cc1OC1C(C)C1	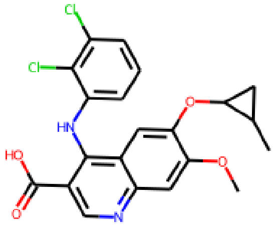	2.0	0.623362	2.525411	2.997881	4	0.774857
**399**	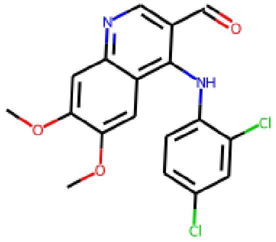	0.613687	COc1cc2ncc(C(=O)O)c(Nc3cccc(Cl)c3Cl)c2cc1OC1C(C)C1	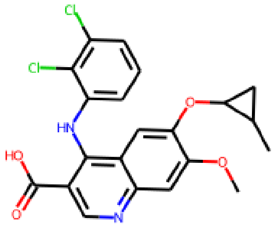	2.0	0.623362	2.525411	2.997881	4	0.774857

	src known mol	similarity	smiles	mol	Class	qed	logP	SAS	rings	kinase inhibition likeliness
**400**	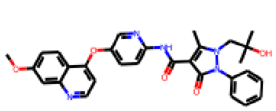	0.613494	COc1ccc2c(=O)cc(CNC(=O)c3c(C)n(C)n(-c4ccccc4)c3=O)nc3cccc2c3c1	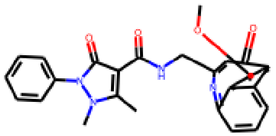	9.0	0.638163	3.995589	2.988471	5	0.774651
**401**	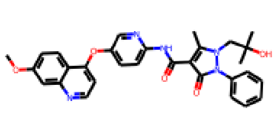	0.613494	COc1ccc2c(=O)cc(CNC(=O)c3c(C)n(C)n(-c4ccccc4)c3=O)nc3cccc2c3c1	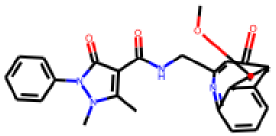	9.0	0.638163	3.995589	2.988471	5	0.774651
**402**	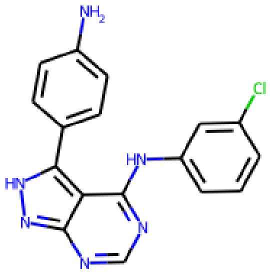	0.613345	c1ccc(Nc2ncnc3n[nH]c(-c4ccccc4)c23)cc1	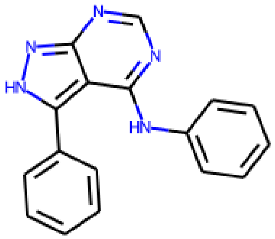	0.0	0.638163	3.995589	2.988471	4	0.800634
**403**	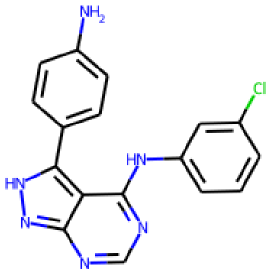	0.613298	c1ccc(Nc2ncnc3n[nH]c(-c4ccccc4)c23)cc1	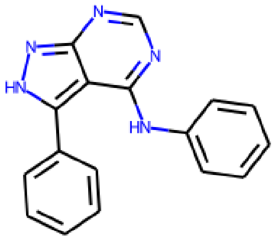	0.0	0.638163	3.995589	2.988471	4	0.800634

	src known mol	similarity	smiles	mol	Class	qed	logP	SAS	rings	kinase inhibition likeliness
**404**	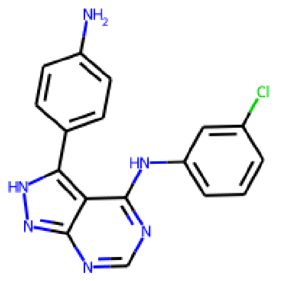	0.613298	c1ccc(Nc2ncnc3n[nH]c(-c4ccccc4)c23)cc1	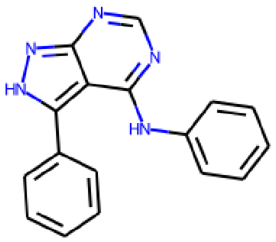	0.0	0.638163	3.995589	2.988471	4	0.800634
**405**	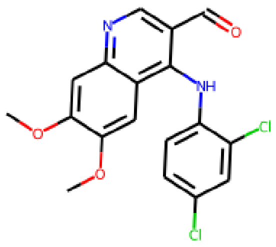	0.613293	COc1cc2ncc(C(=O)O)c(Nc3cccc(Cl)c3Cl)c2cc1OC1C(C)C1	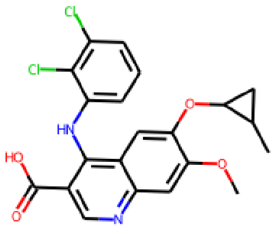	2.0	0.623362	2.525411	2.997881	4	0.774857
**406**	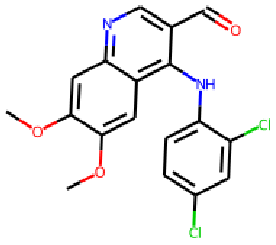	0.613293	COc1cc2ncc(C(=O)O)c(Nc3cccc(Cl)c3Cl)c2cc1OC1C(C)C1	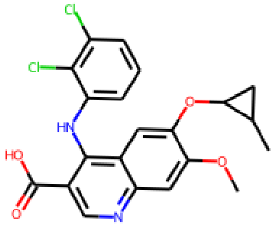	2.0	0.623362	2.525411	2.997881	4	0.774857
**407**	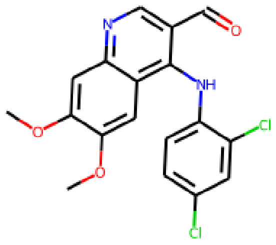	0.613293	COc1cc2ncc(C(=O)O)c(Nc3cccc(Cl)c3Cl)c2cc1OC1C(C)C1	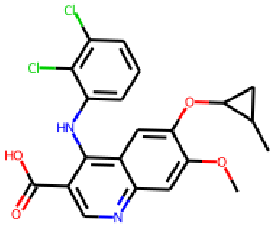	2.0	0.623362	2.525411	2.997881	4	0.774857

	src known mol	similarity	smiles	mol	Class	qed	logP	SAS	rings	kinase inhibition likeliness
**408**	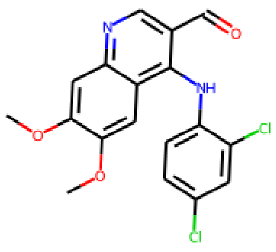	0.613293	COc1cc2ncc(C(=O)O)c(Nc3cccc(Cl)c3Cl)c2cc1OC1C(C)C1	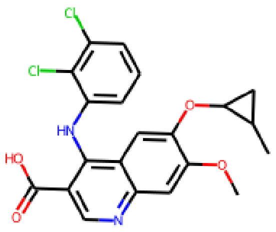	2.0	0.623362	2.525411	2.997881	4	0.774857
**409**	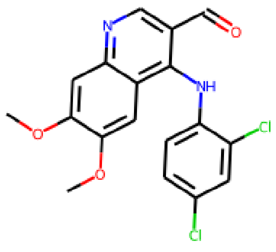	0.613122	COc1cc2ncc(C(=O)O)c(Nc3cccc(Cl)c3Cl)c2cc1OC1C(C)C1	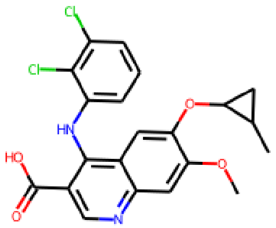	2.0	0.623362	2.525411	2.997881	4	0.774857
**410**	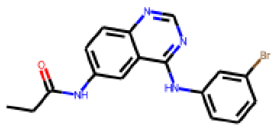	0.612839	O=C(CNC(=O)Oc1ccccc1)Nc1ccc2ncnc(Nc3cccc(Br)c3)c2c1	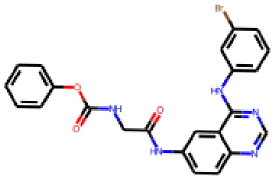	3.0	0.768132	3.608437	2.513170	4	0.763788
**411**	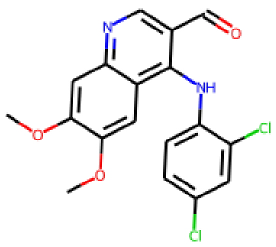	0.612500	COc1cc2ncc(C(=O)O)c(Nc3cccc(Cl)c3Cl)c2cc1OC1C(C)C1	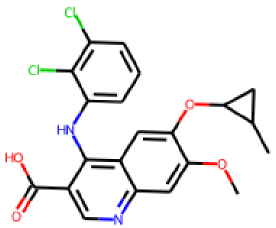	2.0	0.623362	2.525411	2.997881	4	0.774857

	src known mol	similarity	smiles	mol	Class	qed	logP	SAS	rings	kinase inhibition likeliness
**412**	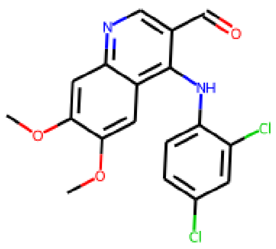	0.612291	COc1cc2ncc(C(=O)O)c(Nc3cccc(Cl)c3Cl)c2cc1OC1C(C)C1	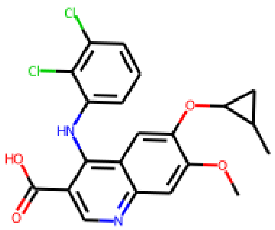	2.0	0.623362	2.525411	2.997881	4	0.774857
**413**	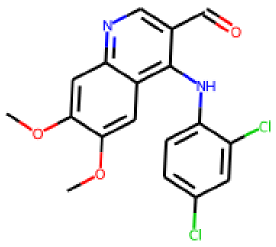	0.612291	COc1cc2ncc(C(=O)O)c(Nc3cccc(Cl)c3Cl)c2cc1OC1C(C)C1	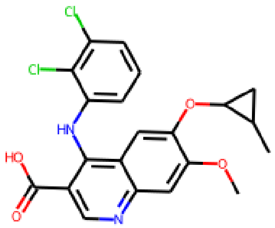	2.0	0.623362	2.525411	2.997881	4	0.774857
**414**	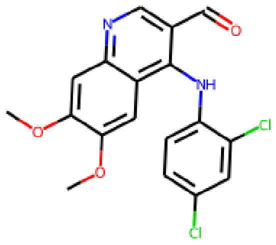	0.612215	COc1cc2ncc(C(=O)O)c(Nc3cccc(Cl)c3Cl)c2cc1OC1C(C)C1	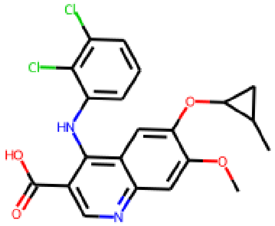	2.0	0.623362	2.525411	2.997881	4	0.774857
**415**	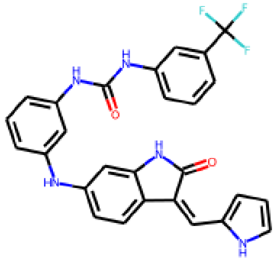	0.611679	COc1ccc(NC(=O)Nc2ccc3c(c2)NC(=O)/C3=C/c2ccc[nH]2)cc1	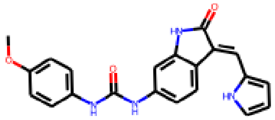	4.0	0.638163	3.995589	2.988471	4	0.768944

	src known mol	similarity	smiles	mol	Class	qed	logP	SAS	rings	kinase inhibition likeliness
**416**	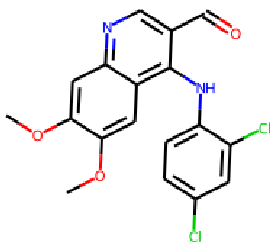	0.611489	COc1cc2ncc(C(=O)O)c(Nc3cccc(Cl)c3Cl)c2cc1OC1C(C)C1	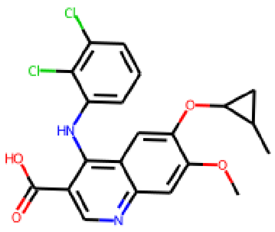	2.0	0.623362	2.525411	2.997881	4	0.774857
**417**	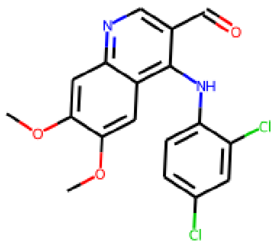	0.611489	COc1cc2ncc(C(=O)O)c(Nc3cccc(Cl)c3Cl)c2cc1OC1C(C)C1	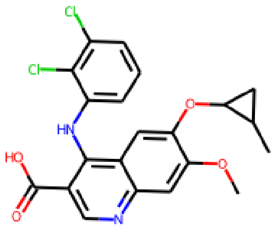	2.0	0.623362	2.525411	2.997881	4	0.774857
**418**	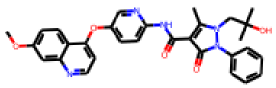	0.611455	COc1ccc2c(=O)cc(CNC(=O)c3c(C)n(C)n(-c4ccccc4)c3=O)nc3cccc2c3c1	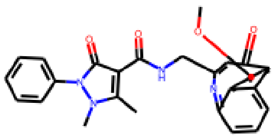	9.0	0.638163	3.995589	2.988471	5	0.774651
**419**	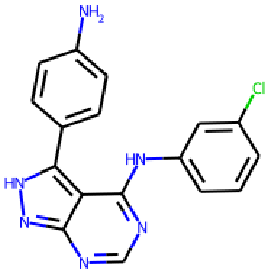	0.611448	c1ccc(Nc2ncnc3n[nH]c(-c4ccccc4)c23)cc1	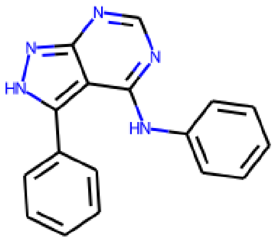	0.0	0.638163	3.995589	2.988471	4	0.800634

	src known mol	similarity	smiles	mol	Class	qed	logP	SAS	rings	kinase inhibition likeliness
**420**	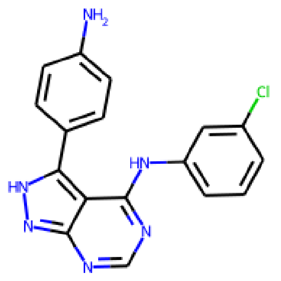	0.611304	c1ccc(Nc2ncnc3n[nH]c(-c4ccccc4)c23)cc1	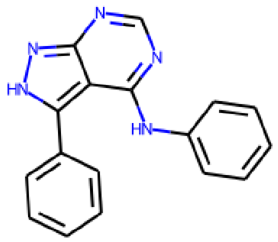	0.0	0.638163	3.995589	2.988471	4	0.800634
**421**	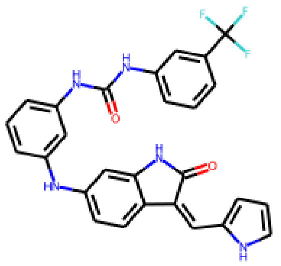	0.610904	COc1ccc(NC(=O)Nc2ccc3c(c2)NC(=O)/C3=C/c2ccc[nH]2)cc1	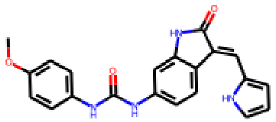	4.0	0.638163	3.995589	2.988471	4	0.768944
**422**	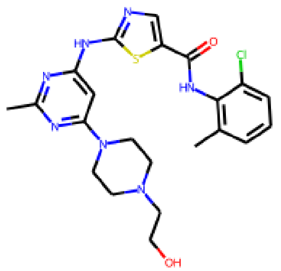	0.610380	Cc1nc(Nc2ncc(C(=O)Nc3c(C)cccc3Cl)s2)cc(N2CCNC(=O)C2)n1	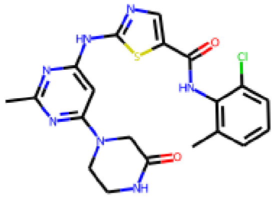	6.0	0.593231	3.250153	2.680275	4	0.755833
**423**	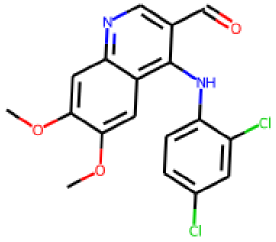	0.610195	COc1cc2ncc(C(=O)O)c(Nc3cccc(Cl)c3Cl)c2cc1OC1C(C)C1	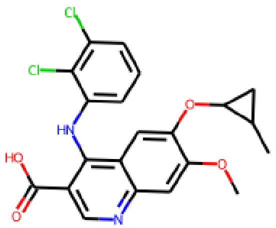	2.0	0.623362	2.525411	2.997881	4	0.774857

	src known mol	similarity	smiles	mol	Class	qed	logP	SAS	rings	kinase inhibition likeliness
**424**	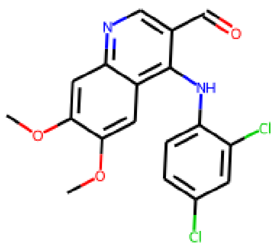	0.610195	COc1cc2ncc(C(=O)O)c(Nc3cccc(Cl)c3Cl)c2cc1OC1C(C)C1	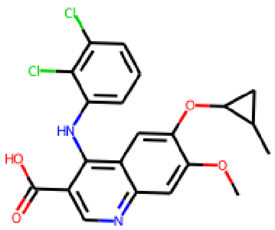	2.0	0.623362	2.525411	2.997881	4	0.774857
**425**	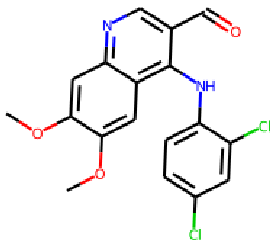	0.610132	COc1cc2ncc(C(=O)O)c(Nc3cccc(Cl)c3Cl)c2cc1OC1C(C)C1	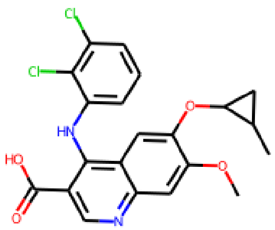	2.0	0.623362	2.525411	2.997881	4	0.774857
**426**	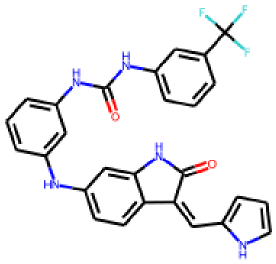	0.610072	COc1ccc(NC(=O)Nc2ccc3c(c2)NC(=O)/C3=C/c2ccc[nH]2)cc1	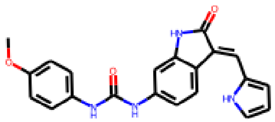	4.0	0.638163	3.995589	2.988471	4	0.768944
**427**	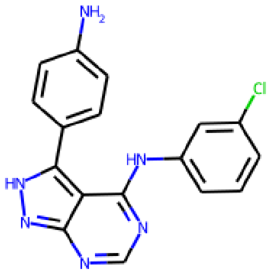	0.609756	c1ccc(Nc2ncnc3n[nH]c(-c4ccccc4)c23)cc1	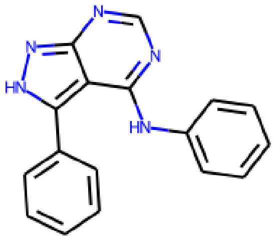	0.0	0.638163	3.995589	2.988471	4	0.800634

	src known mol	similarity	smiles	mol	Class	qed	logP	SAS	rings	kinase inhibition likeliness
**428**	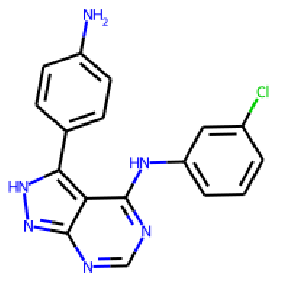	0.609735	c1ccc(Nc2ncnc3n[nH]c(-c4ccccc4)c23)cc1	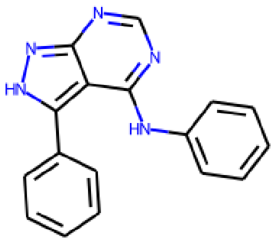	0.0	0.638163	3.995589	2.988471	4	0.800634
**429**	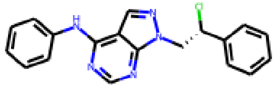	0.609495	Cc1ccc(C(=O)Nc2ccon2)cc1Nc1ncnc2c1cnn2C1cccccc1	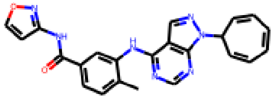	8.0	0.623362	2.525411	2.997881	5	0.770298
**430**	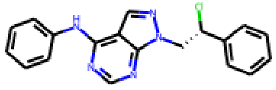	0.609495	Cc1ccc(C(=O)Nc2ccon2)cc1Nc1ncnc2c1cnn2C1cccccc1	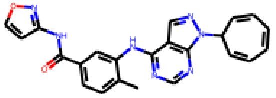	8.0	0.623362	2.525411	2.997881	5	0.770298
**431**	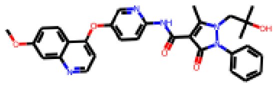	0.609237	COc1ccc2c(=O)cc(CNC(=O)c3c(C)n(C)n(-c4ccccc4)c3=O)nc3cccc2c3c1	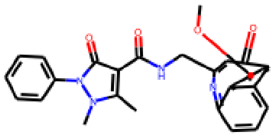	9.0	0.638163	3.995589	2.988471	5	0.774651

	src known mol	similarity	smiles	mol	Class	qed	logP	SAS	rings	kinase inhibition likeliness
**432**	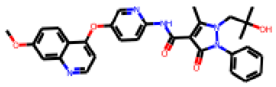	0.608810	COc1ccc2c(=O)cc(CNC(=O)c3c(C)n(C)n(-c4ccccc4)c3=O)nc3cccc2c3c1	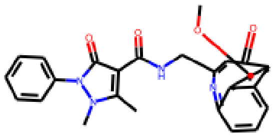	9.0	0.638163	3.995589	2.988471	5	0.774651
**433**	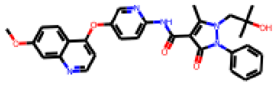	0.608764	COc1ccc2c(=O)cc(CNC(=O)c3c(C)n(C)n(-c4ccccc4)c3=O)nc3cccc2c3c1	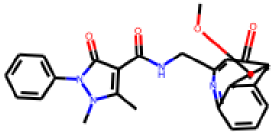	9.0	0.638163	3.995589	2.988471	5	0.774651
**434**	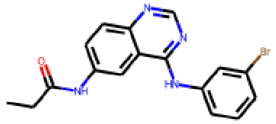	0.608498	O=C(CNC(=O)Oc1ccccc1)Nc1ccc2ncnc(Nc3cccc(Cl)c3)c2c1	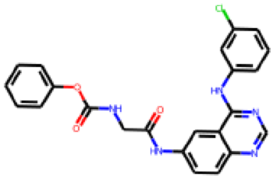	3.0	0.638163	3.995589	2.988471	4	0.793889
**435**	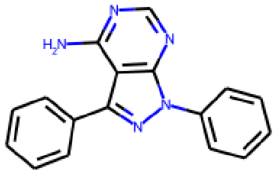	0.608472	Cc1ccc(C(=O)Oc2ccon2)cc1-c1ncnc2c1cnn2-c1ccccc1	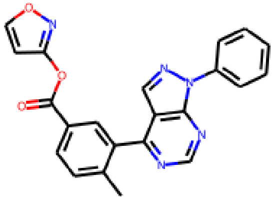	8.0	0.635315	4.542409	3.856266	5	0.760056

	src known mol	similarity	smiles	mol	Class	qed	logP	SAS	rings	kinase inhibition likeliness
**436**	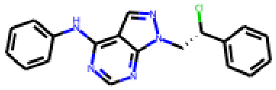	0.608457	Cc1ccc(C(=O)Nc2ccon2)cc1Nc1ncnc2c1cnn2C1cccccc1	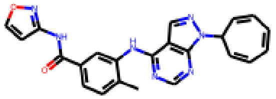	8.0	0.623362	2.525411	2.997881	5	0.770298
**437**	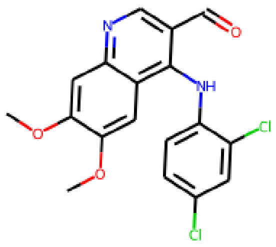	0.608434	COc1cc2ncc(C(=O)O)c(Nc3cccc(Cl)c3Cl)c2cc1OC1C(C)C1	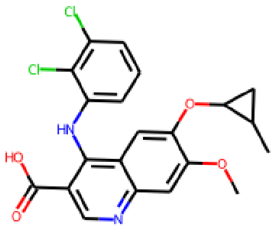	2.0	0.623362	2.525411	2.997881	4	0.774857
**438**	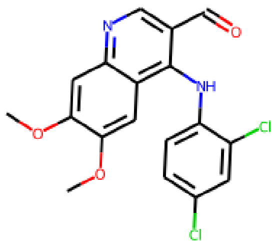	0.608434	COc1cc2ncc(C(=O)O)c(Nc3cccc(Cl)c3Cl)c2cc1OC1C(C)C1	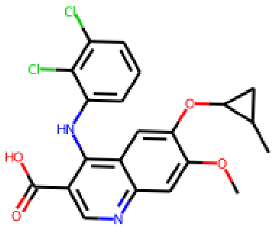	2.0	0.623362	2.525411	2.997881	4	0.774857
**439**	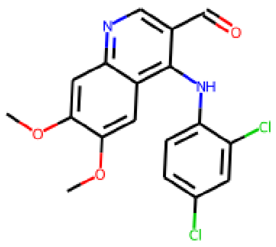	0.607901	COc1cc2ncc(C(=O)O)c(Nc3cccc(Cl)c3Cl)c2cc1OC1C(C)C1	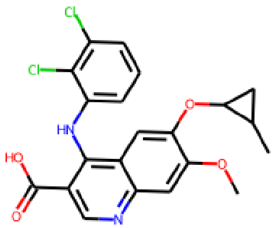	2.0	0.623362	2.525411	2.997881	4	0.774857

	src known mol	similarity	smiles	mol	Class	qed	logP	SAS	rings	kinase inhibition likeliness
**440**	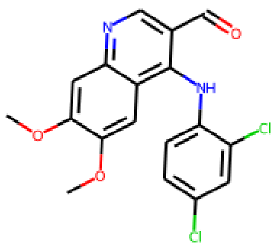	0.607901	COc1cc2ncc(C(=O)O)c(Nc3cccc(Cl)c3Cl)c2cc1OC1C(C)C1	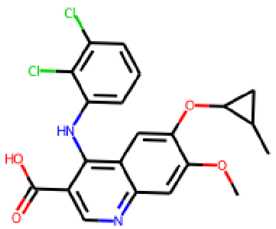	2.0	0.623362	2.525411	2.997881	4	0.774857
**441**	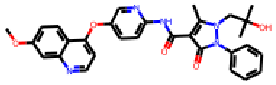	0.607761	COc1ccc2c(=O)cc(CNC(=O)c3c(C)n(C)n(-c4ccccc4)c3=O)nc3cccc2c3c1	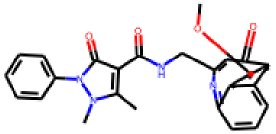	9.0	0.638163	3.995589	2.988471	5	0.774651
**442**	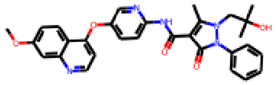	0.607761	COc1ccc2c(=O)cc(CNC(=O)c3c(C)n(C)n(-c4ccccc4)c3=O)nc3cccc2c3c1	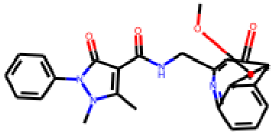	9.0	0.638163	3.995589	2.988471	5	0.774651
**443**	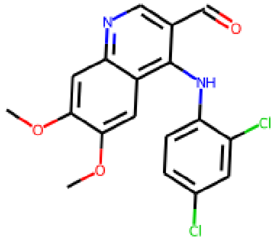	0.607550	COc1cc2ncc(C(=O)O)c(Nc3cccc(Cl)c3Cl)c2cc1OC1C(C)C1	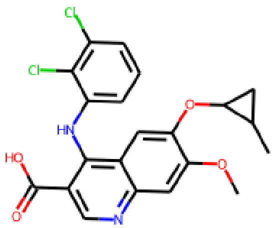	2.0	0.623362	2.525411	2.997881	4	0.774857

	src known mol	similarity	smiles	mol	Class	qed	logP	SAS	rings	kinase inhibition likeliness
**444**	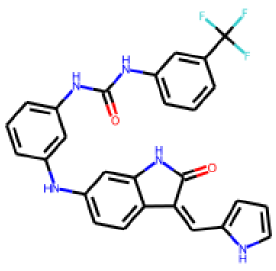	0.607483	COc1ccc(NC(=O)Nc2ccc3c(c2)NC(=O)/C3=C/c2ccc[nH]2)cc1	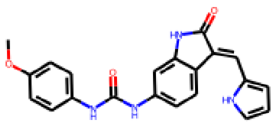	4.0	0.638163	3.995589	2.988471	4	0.768944
**445**	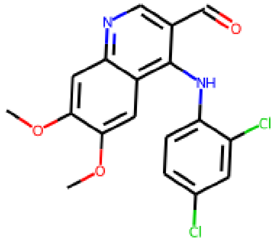	0.607456	COc1cc2ncc(C(=O)O)c(Nc3cccc(Cl)c3Cl)c2cc1OC1C(C)C1	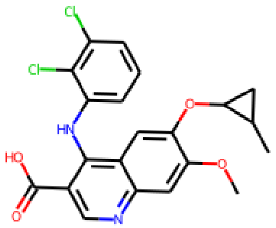	2.0	0.623362	2.525411	2.997881	4	0.774857
**446**	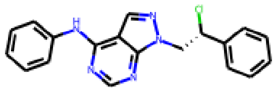	0.607413	Cc1ccc(C(=O)Nc2ccon2)cc1Nc1ncnc2c1cnn2C1cccccc1	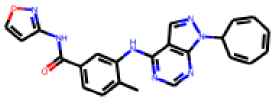	8.0	0.623362	2.525411	2.997881	5	0.770298
**447**	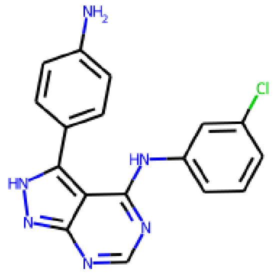	0.607388	c1ccc(Nc2ncnc3n[nH]c(-c4ccccc4)c23)cc1	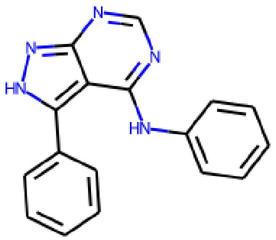	0.0	0.638163	3.995589	2.988471	4	0.800634

	src known mol	similarity	smiles	mol	Class	qed	logP	SAS	rings	kinase inhibition likeliness
**448**	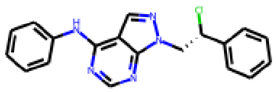	0.607303	Cc1ccc(C(=O)Nc2ccon2)cc1Nc1ncnc2c1cnn2C1cccccc1	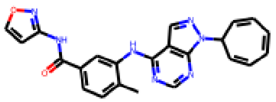	8.0	0.623362	2.525411	2.997881	5	0.770298
**449**	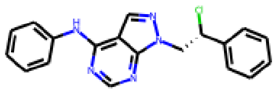	0.607303	Cc1ccc(C(=O)Nc2ccon2)cc1Nc1ncnc2c1cnn2C1cccccc1	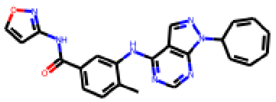	8.0	0.623362	2.525411	2.997881	5	0.770298
**450**	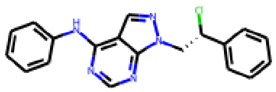	0.607303	Cc1ccc(C(=O)Nc2ccon2)cc1Nc1ncnc2c1cnn2C1cccccc1	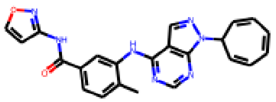	8.0	0.623362	2.525411	2.997881	5	0.770298
**451**	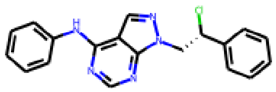	0.607303	Cc1ccc(C(=O)Nc2ccon2)cc1Nc1ncnc2c1cnn2C1cccccc1	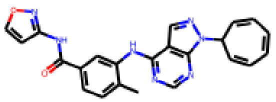	8.0	0.623362	2.525411	2.997881	5	0.770298

	src known mol	similarity	smiles	mol	Class	qed	logP	SAS	rings	kinase inhibition likeliness
**452**	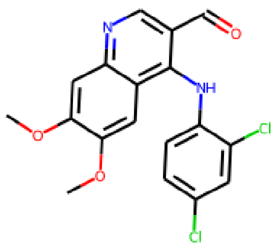	0.607223	COc1cc2ncc(C(=O)O)c(Nc3cccc(Cl)c3Cl)c2cc1OC1C(C)C1	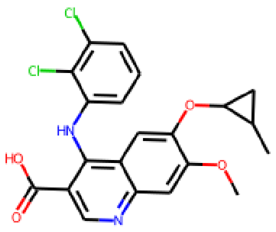	2.0	0.623362	2.525411	2.997881	4	0.774857
**453**	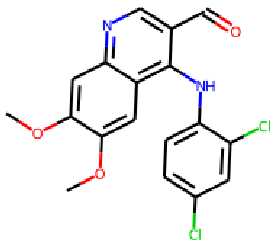	0.607223	COc1cc2ncc(C(=O)O)c(Nc3cccc(Cl)c3Cl)c2cc1OC1C(C)C1	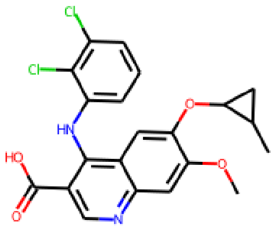	2.0	0.623362	2.525411	2.997881	4	0.774857
**454**	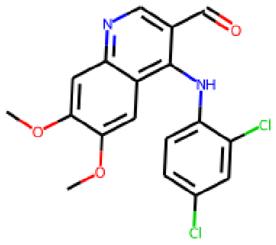	0.607143	COc1cc2ncc(C(=O)O)c(Nc3cccc(Cl)c3Cl)c2cc1OC1C(C)C1	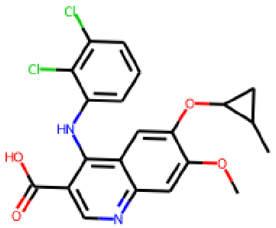	2.0	0.623362	2.525411	2.997881	4	0.774857
**455**	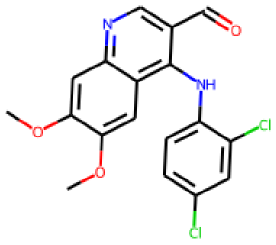	0.607143	COc1cc2ncc(C(=O)O)c(Nc3cccc(Cl)c3Cl)c2cc1OC1C(C)C1	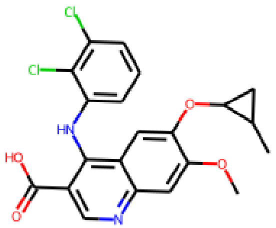	2.0	0.623362	2.525411	2.997881	4	0.774857

	src known mol	similarity	smiles	mol	Class	qed	logP	SAS	rings	kinase inhibition likeliness
**456**	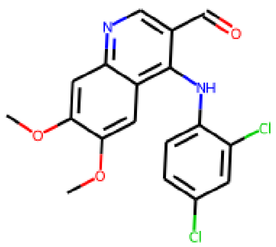	0.607143	COc1cc2ncc(C(=O)O)c(Nc3cccc(Cl)c3Cl)c2cc1OC1C(C)C1	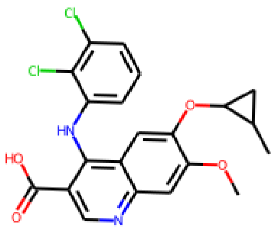	2.0	0.623362	2.525411	2.997881	4	0.774857
**457**	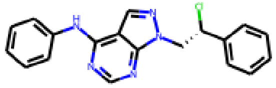	0.606800	Cc1ccc(C(=O)Nc2ccon2)cc1Nc1ncnc2c1cnn2C1cccccc1	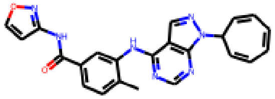	8.0	0.623362	2.525411	2.997881	5	0.770298
**458**	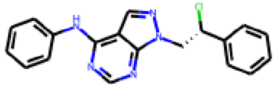	0.606800	Cc1ccc(C(=O)Nc2ccon2)cc1Nc1ncnc2c1cnn2C1cccccc1	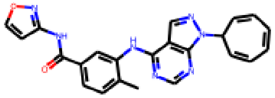	8.0	0.623362	2.525411	2.997881	5	0.770298
**459**	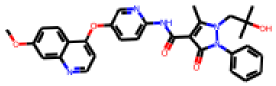	0.606789	COc1ccc2c(=O)cc(CNC(=O)c3c(C)n(C)n(-c4ccccc4)c3=O)nc3cccc2c3c1	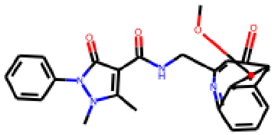	9.0	0.638163	3.995589	2.988471	5	0.774651

	src known mol	similarity	smiles	mol	Class	qed	logP	SAS	rings	kinase inhibition likeliness
**460**	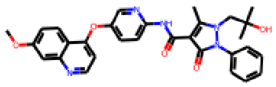	0.606789	COc1ccc2c(=O)cc(CNC(=O)c3c(C)n(C)n(-c4ccccc4)c3=O)nc3cccc2c3c1	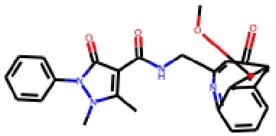	9.0	0.638163	3.995589	2.988471	5	0.774651
**461**	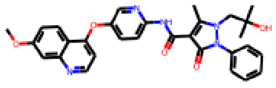	0.606789	COc1ccc2c(=O)cc(CNC(=O)c3c(C)n(C)n(-c4ccccc4)c3=O)nc3cccc2c3c1	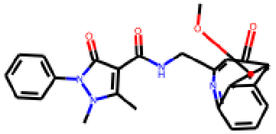	9.0	0.638163	3.995589	2.988471	5	0.774651
**462**	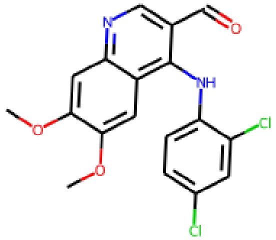	0.606750	COc1cc2ncc(C(=O)O)c(Nc3cccc(Cl)c3Cl)c2cc1OC1C(C)C1	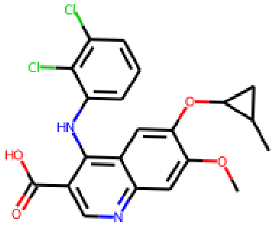	2.0	0.623362	2.525411	2.997881	4	0.774857
**463**	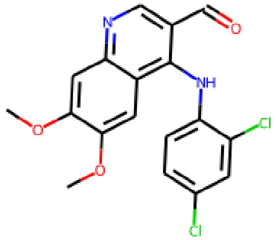	0.606750	COc1cc2ncc(C(=O)O)c(Nc3cccc(Cl)c3Cl)c2cc1OC1C(C)C1	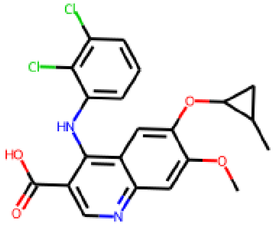	2.0	0.623362	2.525411	2.997881	4	0.774857

	src known mol	similarity	smiles	mol	Class	qed	logP	SAS	rings	kinase inhibition likeliness
**464**	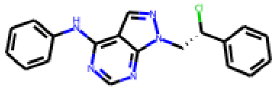	0.606703	Cc1ccc(C(=O)Nc2ccon2)cc1Nc1ncnc2c1cnn2C1cccccc1	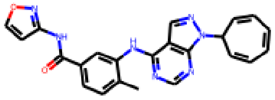	8.0	0.623362	2.525411	2.997881	5	0.770298
**465**	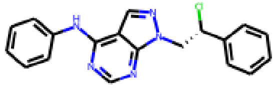	0.606703	Cc1ccc(C(=O)Nc2ccon2)cc1Nc1ncnc2c1cnn2C1cccccc1	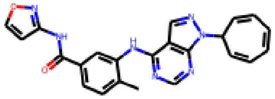	8.0	0.623362	2.525411	2.997881	5	0.770298
**466**	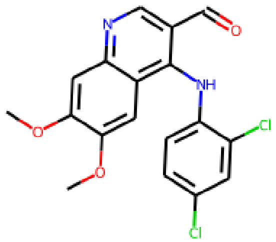	0.606582	COc1cc2ncc(C(=O)O)c(Nc3cccc(Cl)c3Cl)c2cc1OC1C(C)C1	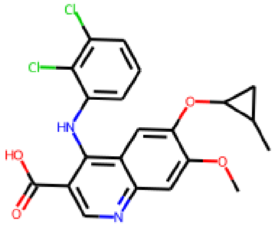	2.0	0.623362	2.525411	2.997881	4	0.774857
**467**	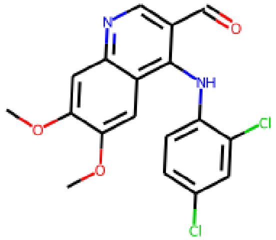	0.606582	COc1cc2ncc(C(=O)O)c(Nc3cccc(Cl)c3Cl)c2cc1OC1C(C)C1	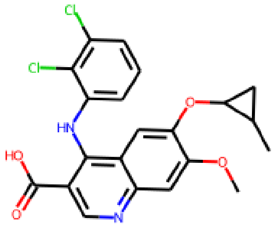	2.0	0.623362	2.525411	2.997881	4	0.774857

	src known mol	similarity	smiles	mol	Class	qed	logP	SAS	rings	kinase inhibition likeliness
**468**	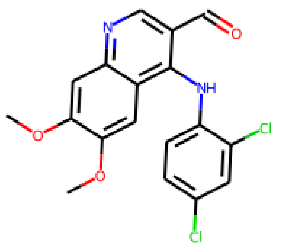	0.606447	COc1cc2ncc(C(=O)O)c(Nc3cccc(Cl)c3Cl)c2cc1OC1C(C)C1	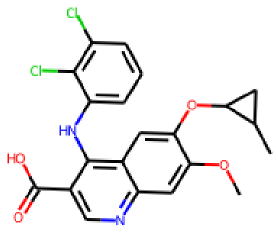	2.0	0.623362	2.525411	2.997881	4	0.774857
**469**	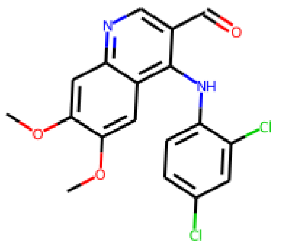	0.606447	COc1cc2ncc(C(=O)O)c(Nc3cccc(Cl)c3Cl)c2cc1OC1C(C)C1	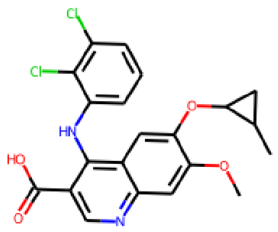	2.0	0.623362	2.525411	2.997881	4	0.774857
**470**	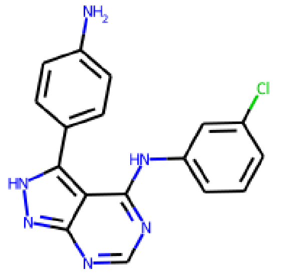	0.606374	c1ccc(Nc2ncnc3n[nH]c(-c4ccccc4)c23)cc1	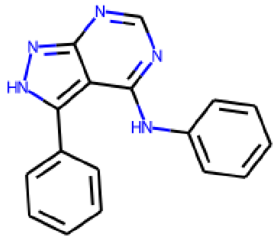	0.0	0.638163	3.995589	2.988471	4	0.800634
**471**	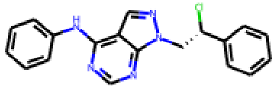	0.606352	Cc1ccc(C(=O)Nc2ccon2)cc1Nc1ncnc2c1cnn2C1cccccc1	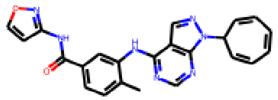	8.0	0.623362	2.525411	2.997881	5	0.770298

	src known mol	similarity	smiles	mol	Class	qed	logP	SAS	rings	kinase inhibition likeliness
**472**	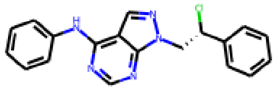	0.606352	Cc1ccc(C(=O)Nc2ccon2)cc1Nc1ncnc2c1cnn2C1cccccc1	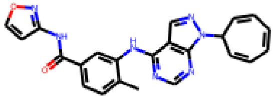	8.0	0.623362	2.525411	2.997881	5	0.770298
**473**	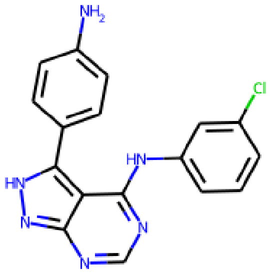	0.606009	c1ccc(Nc2ncnc3n[nH]c(-c4ccccc4)c23)cc1	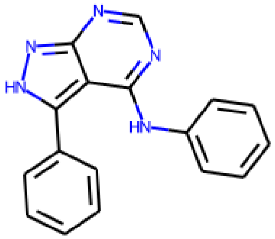	0.0	0.638163	3.995589	2.988471	4	0.800634
**474**	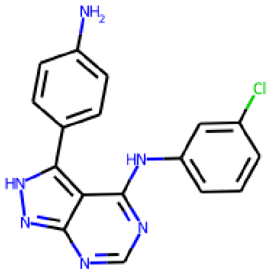	0.606009	c1ccc(Nc2ncnc3n[nH]c(-c4ccccc4)c23)cc1	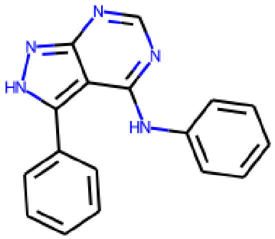	0.0	0.638163	3.995589	2.988471	4	0.800634
**475**	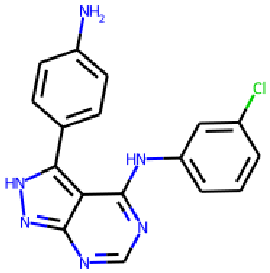	0.606009	c1ccc(Nc2ncnc3n[nH]c(-c4ccccc4)c23)cc1	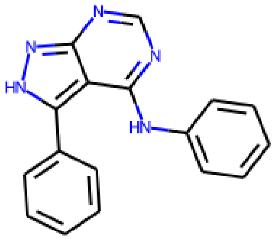	0.0	0.638163	3.995589	2.988471	4	0.800634

	src known mol	similarity	smiles	mol	Class	qed	logP	SAS	rings	kinase inhibition likeliness
**476**	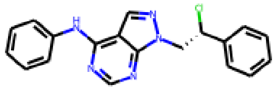	0.605834	Cc1ccc(C(=O)Nc2ccon2)cc1Nc1ncnc2c1cnn2C1cccccc1	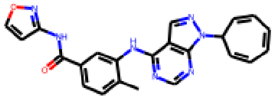	8.0	0.623362	2.525411	2.997881	5	0.770298
**477**	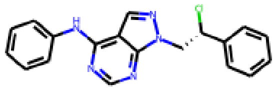	0.605834	Cc1ccc(C(=O)Nc2ccon2)cc1Nc1ncnc2c1cnn2C1cccccc1	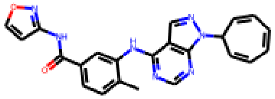	8.0	0.623362	2.525411	2.997881	5	0.770298
**478**	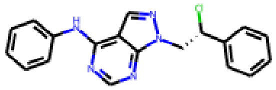	0.605834	Cc1ccc(C(=O)Nc2ccon2)cc1Nc1ncnc2c1cnn2C1cccccc1	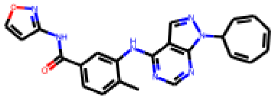	8.0	0.623362	2.525411	2.997881	5	0.770298
**479**	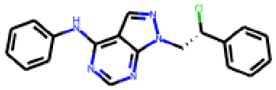	0.605834	Cc1ccc(C(=O)Nc2ccon2)cc1Nc1ncnc2c1cnn2C1cccccc1	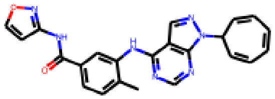	8.0	0.623362	2.525411	2.997881	5	0.770298

	src known mol	similarity	smiles	mol	Class	qed	logP	SAS	rings	kinase inhibition likeliness
**480**	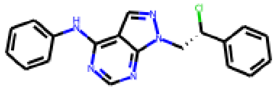	0.605676	Cc1ccc(C(=O)Nc2ccon2)cc1Nc1ncnc2c1cnn2C1cccccc1	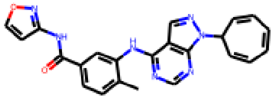	8.0	0.623362	2.525411	2.997881	5	0.770298
**481**	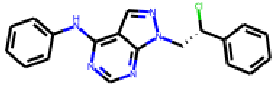	0.605676	Cc1ccc(C(=O)Nc2ccon2)cc1Nc1ncnc2c1cnn2C1cccccc1	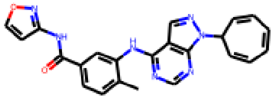	8.0	0.623362	2.525411	2.997881	5	0.770298
**482**	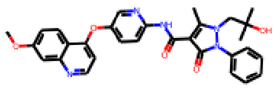	0.605582	COc1ccc2c(=O)cc(CNC(=O)c3c(C)n(C)n(-c4ccccc4)c3=O)nc3cccc2c3c1	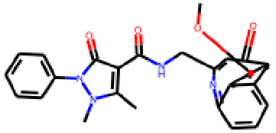	9.0	0.638163	3.995589	2.988471	5	0.774651
**483**	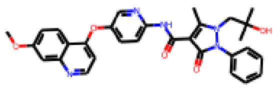	0.605582	COc1ccc2c(=O)cc(CNC(=O)c3c(C)n(C)n(-c4ccccc4)c3=O)nc3cccc2c3c1	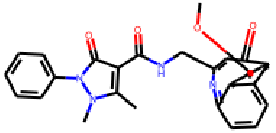	9.0	0.638163	3.995589	2.988471	5	0.774651

	src known mol	similarity	smiles	mol	Class	qed	logP	SAS	rings	kinase inhibition likeliness
**484**	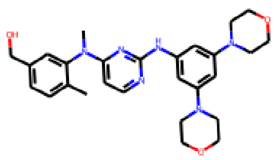	0.605338	NC(=O)c1ccccc1Nc1nc(Nc2ccc(N3CCOCC3)cc2C)ncc1Cl	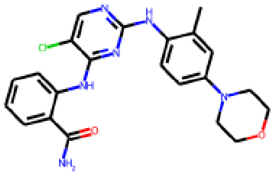	7.0	0.623362	2.525411	2.997881	4	0.783217
**485**	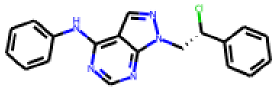	0.604966	Cc1ccc(C(=O)Nc2ccon2)cc1Nc1ncnc2c1cnn2C1cccccc1	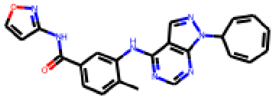	8.0	0.623362	2.525411	2.997881	5	0.770298
**486**	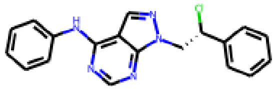	0.604966	Cc1ccc(C(=O)Nc2ccon2)cc1Nc1ncnc2c1cnn2C1cccccc1	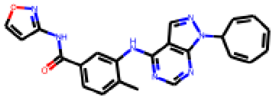	8.0	0.623362	2.525411	2.997881	5	0.770298
**487**	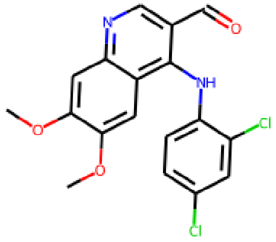	0.604928	COc1cc2ncc(C(=O)O)c(Nc3cccc(Cl)c3Cl)c2cc1OC1C(C)C1	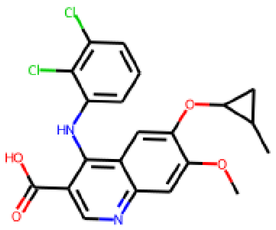	2.0	0.623362	2.525411	2.997881	4	0.774857

	src known mol	similarity	smiles	mol	Class	qed	logP	SAS	rings	kinase inhibition likeliness
**488**	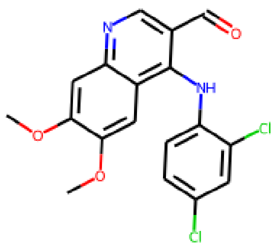	0.604928	COc1cc2ncc(C(=O)O)c(Nc3cccc(Cl)c3Cl)c2cc1OC1C(C)C1	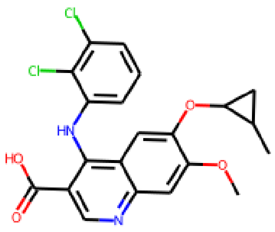	2.0	0.623362	2.525411	2.997881	4	0.774857
**489**	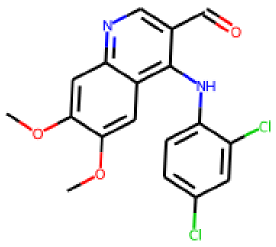	0.604928	COc1cc2ncc(C(=O)O)c(Nc3cccc(Cl)c3Cl)c2cc1OC1C(C)C1	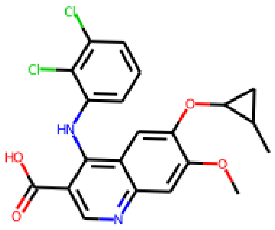	2.0	0.623362	2.525411	2.997881	4	0.774857
**490**	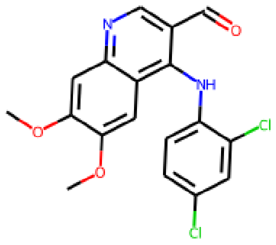	0.604928	COc1cc2ncc(C(=O)O)c(Nc3cccc(Cl)c3Cl)c2cc1OC1C(C)C1	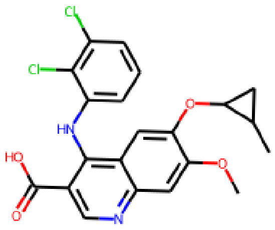	2.0	0.623362	2.525411	2.997881	4	0.774857
**491**	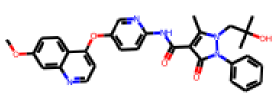	0.604834	COc1ccc2c(=O)cc(CNC(=O)c3c(C)n(C)n(-c4ccccc4)c3=O)nc3cccc2c3c1	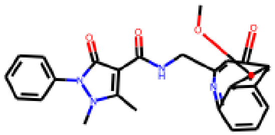	9.0	0.638163	3.995589	2.988471	5	0.774651

	src known mol	similarity	smiles	mol	Class	qed	logP	SAS	rings	kinase inhibition likeliness
**492**	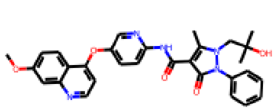	0.604517	COc1ccc2c(=O)cc(CNC(=O)c3c(C)n(C)n(-c4ccccc4)c3=O)nc3cccc2c3c1	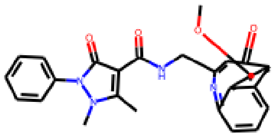	9.0	0.638163	3.995589	2.988471	5	0.774651
**493**	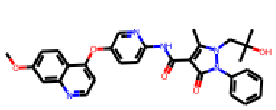	0.604517	COc1ccc2c(=O)cc(CNC(=O)c3c(C)n(C)n(-c4ccccc4)c3=O)nc3cccc2c3c1	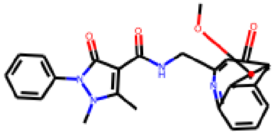	9.0	0.638163	3.995589	2.988471	5	0.774651
**494**	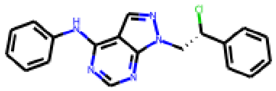	0.604467	Cc1ccc(C(=O)Nc2ccon2)cc1Nc1ncnc2c1cnn2C1cccccc1	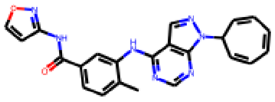	8.0	0.623362	2.525411	2.997881	5	0.770298
**495**	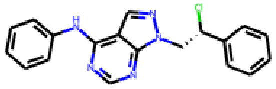	0.604467	Cc1ccc(C(=O)Nc2ccon2)cc1Nc1ncnc2c1cnn2C1cccccc1	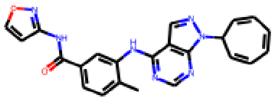	8.0	0.623362	2.525411	2.997881	5	0.770298

	src known mol	similarity	smiles	mol	Class	qed	logP	SAS	rings	kinase inhibition likeliness
**496**	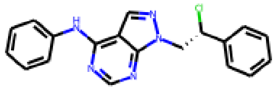	0.604467	Cc1ccc(C(=O)Nc2ccon2)cc1Nc1ncnc2c1cnn2C1cccccc1	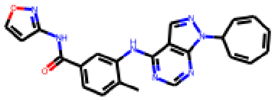	8.0	0.623362	2.525411	2.997881	5	0.770298
**497**	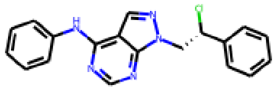	0.604467	Cc1ccc(C(=O)Nc2ccon2)cc1Nc1ncnc2c1cnn2C1cccccc1	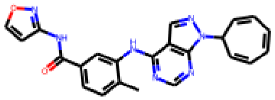	8.0	0.623362	2.525411	2.997881	5	0.770298
**498**	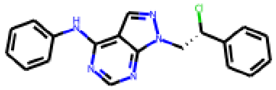	0.604467	Cc1ccc(C(=O)Nc2ccon2)cc1Nc1ncnc2c1cnn2C1cccccc1	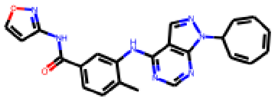	8.0	0.623362	2.525411	2.997881	5	0.770298
**499**	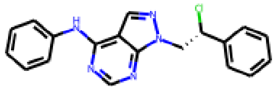	0.604467	Cc1ccc(C(=O)Nc2ccon2)cc1Nc1ncnc2c1cnn2C1cccccc1	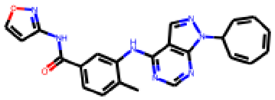	8.0	0.623362	2.525411	2.997881	5	0.770298

	src known mol	similarity	smiles	mol	Class	qed	logP	SAS	rings	kinase inhibition likeliness
**500**	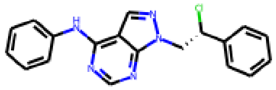	0.604467	Cc1ccc(C(=O)Nc2ccon2)cc1Nc1ncnc2c1cnn2C1cccccc1	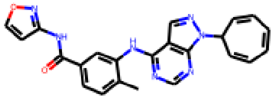	8.0	0.623362	2.525411	2.997881	5	0.770298
**501**	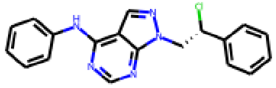	0.604467	Cc1ccc(C(=O)Nc2ccon2)cc1Nc1ncnc2c1cnn2C1cccccc1	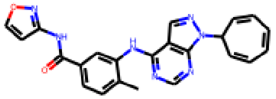	8.0	0.623362	2.525411	2.997881	5	0.770298
**502**	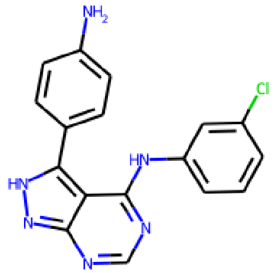	0.604292	c1ccc(Nc2ncnc3n[nH]c(-c4ccccc4)c23)cc1	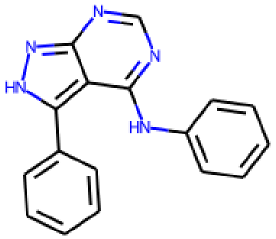	0.0	0.638163	3.995589	2.988471	4	0.800634
**503**	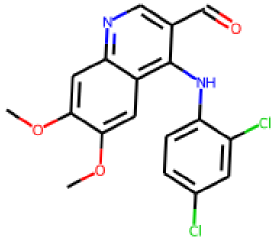	0.604212	COc1cc2ncc(C(=O)O)c(Nc3cccc(Cl)c3Cl)c2cc1OC1C(C)C1	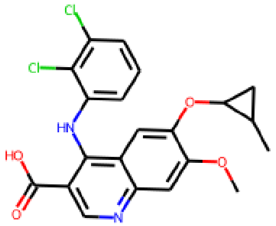	2.0	0.623362	2.525411	2.997881	4	0.774857

	src known mol	similarity	smiles	mol	Class	qed	logP	SAS	rings	kinase inhibition likeliness
**504**	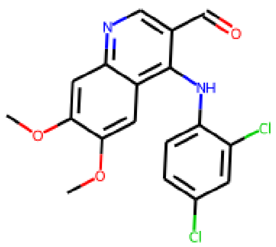	0.604212	COc1cc2ncc(C(=O)O)c(Nc3cccc(Cl)c3Cl)c2cc1OC1C(C)C1	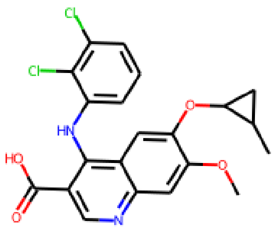	2.0	0.623362	2.525411	2.997881	4	0.774857
**505**	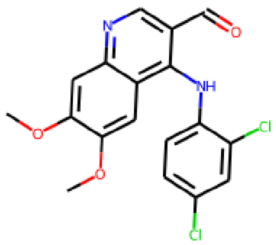	0.604167	COc1cc2ncc(C(=O)O)c(Nc3cccc(Cl)c3Cl)c2cc1OC1C(C)C1	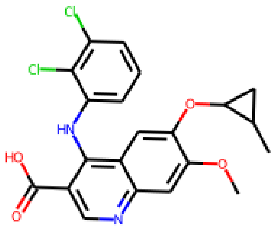	2.0	0.623362	2.525411	2.997881	4	0.774857
**506**	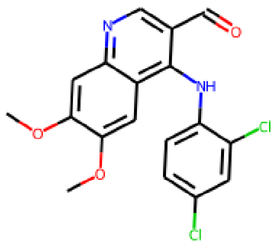	0.604167	COc1cc2ncc(C(=O)O)c(Nc3cccc(Cl)c3Cl)c2cc1OC1C(C)C1	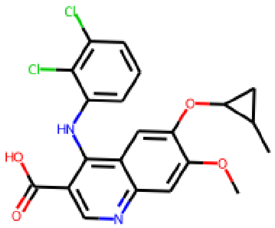	2.0	0.623362	2.525411	2.997881	4	0.774857
**507**	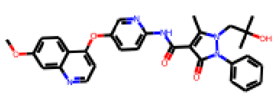	0.604156	COc1ccc2c(=O)cc(CNC(=O)c3c(C)n(C)n(-c4ccccc4)c3=O)nc3cccc2c3c1	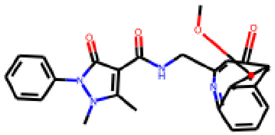	9.0	0.638163	3.995589	2.988471	5	0.774651

	src known mol	similarity	smiles	mol	Class	qed	logP	SAS	rings	kinase inhibition likeliness
**508**	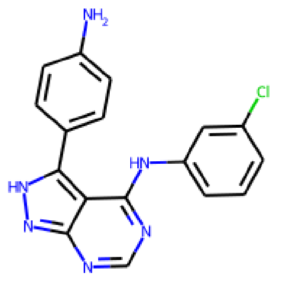	0.603581	c1ccc(Nc2ncnc3n[nH]c(-c4ccccc4)c23)cc1	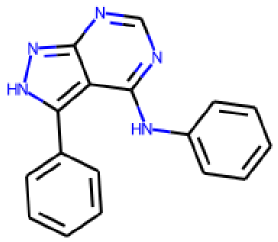	0.0	0.638163	3.995589	2.988471	4	0.800634
**509**	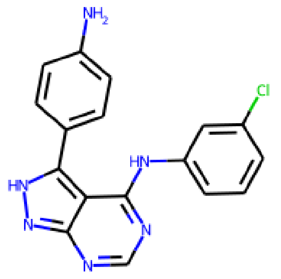	0.603581	c1ccc(Nc2ncnc3n[nH]c(-c4ccccc4)c23)cc1	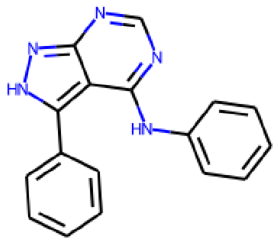	0.0	0.638163	3.995589	2.988471	4	0.800634
**510**	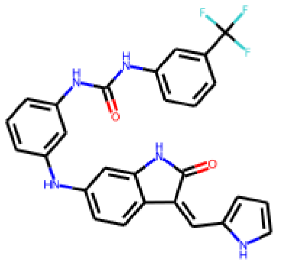	0.603537	COc1ccc(NC(=O)Nc2ccc3c(c2)NC(=O)/C3=C/c2ccc[nH]2)cc1	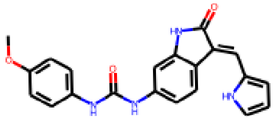	4.0	0.638163	3.995589	2.988471	4	0.768944
**511**	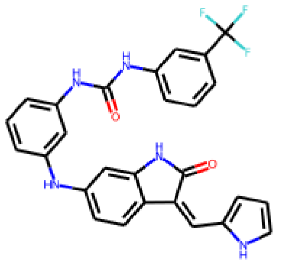	0.603537	COc1ccc(NC(=O)Nc2ccc3c(c2)NC(=O)/C3=C/c2ccc[nH]2)cc1	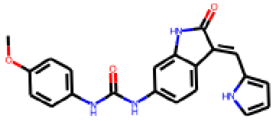	4.0	0.638163	3.995589	2.988471	4	0.768944

	src known mol	similarity	smiles	mol	Class	qed	logP	SAS	rings	kinase inhibition likeliness
**512**	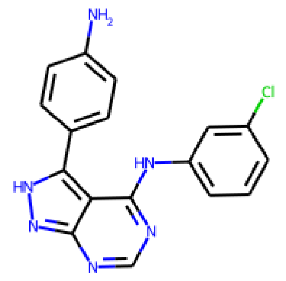	0.603433	c1ccc(Nc2ncnc3n[nH]c(-c4ccccc4)c23)cc1	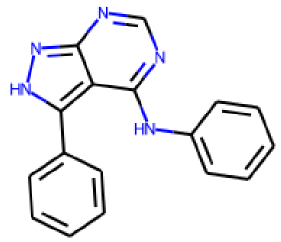	0.0	0.638163	3.995589	2.988471	4	0.800634
**513**	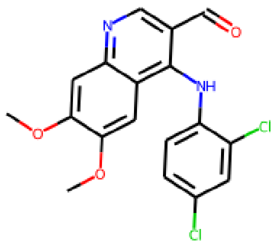	0.603348	COc1cc2ncc(C(=O)O)c(Nc3cccc(Cl)c3Cl)c2cc1OC1C(C)C1	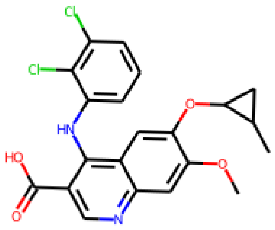	2.0	0.623362	2.525411	2.997881	4	0.774857
**514**	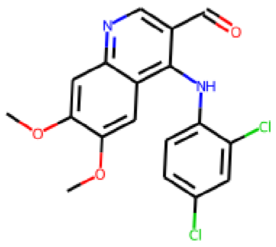	0.603348	COc1cc2ncc(C(=O)O)c(Nc3cccc(Cl)c3Cl)c2cc1OC1C(C)C1	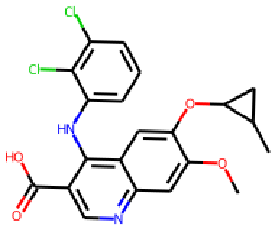	2.0	0.623362	2.525411	2.997881	4	0.774857
**515**	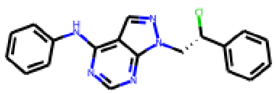	0.603293	Cc1ccc(C(=O)Nc2ccon2)cc1Nc1ncnc2c1cnn2C1cccccc1	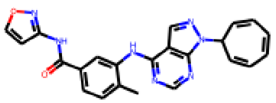	8.0	0.623362	2.525411	2.997881	5	0.770298

	src known mol	similarity	smiles	mol	Class	qed	logP	SAS	rings	kinase inhibition likeliness
**516**	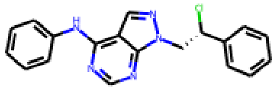	0.603293	Cc1ccc(C(=O)Nc2ccon2)cc1Nc1ncnc2c1cnn2C1cccccc1	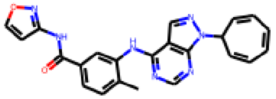	8.0	0.623362	2.525411	2.997881	5	0.770298
**517**	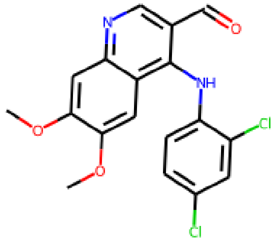	0.603162	COc1cc2ncc(C(=O)O)c(Nc3cccc(Cl)c3Cl)c2cc1OC1C(C)C1	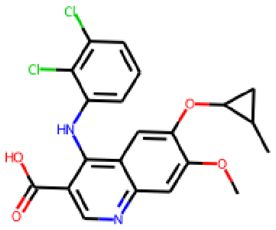	2.0	0.623362	2.525411	2.997881	4	0.774857
**518**	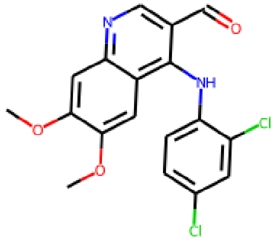	0.603162	COc1cc2ncc(C(=O)O)c(Nc3cccc(Cl)c3Cl)c2cc1OC1C(C)C1	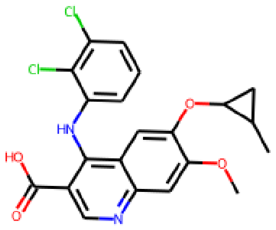	2.0	0.623362	2.525411	2.997881	4	0.774857
**519**	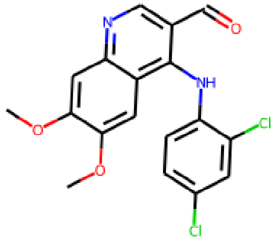	0.603162	COc1cc2ncc(C(=O)O)c(Nc3cccc(Cl)c3Cl)c2cc1OC1C(C)C1	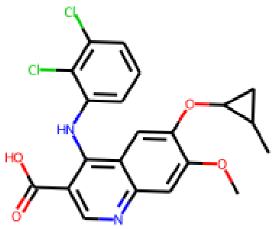	2.0	0.623362	2.525411	2.997881	4	0.774857

	src known mol	similarity	smiles	mol	Class	qed	logP	SAS	rings	kinase inhibition likeliness
**520**	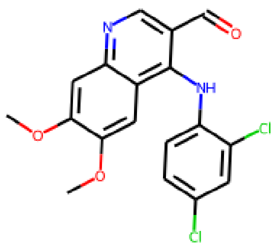	0.603162	COc1cc2ncc(C(=O)O)c(Nc3cccc(Cl)c3Cl)c2cc1OC1C(C)C1	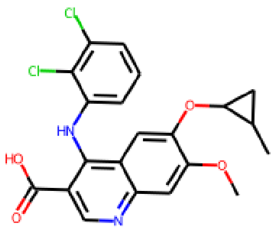	2.0	0.623362	2.525411	2.997881	4	0.774857
**521**	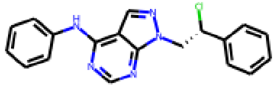	0.603096	Cc1ccc(C(=O)Nc2ccon2)cc1Nc1ncnc2c1cnn2C1cccccc1	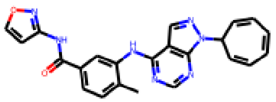	8.0	0.623362	2.525411	2.997881	5	0.770298
**522**	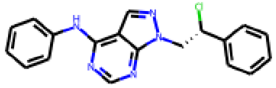	0.603096	Cc1ccc(C(=O)Nc2ccon2)cc1Nc1ncnc2c1cnn2C1cccccc1	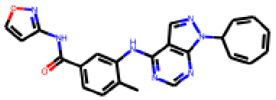	8.0	0.623362	2.525411	2.997881	5	0.770298
**523**	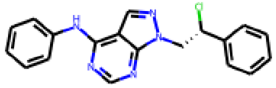	0.602985	Cc1ccc(C(=O)Nc2ccon2)cc1Nc1ncnc2c1cnn2C1cccccc1	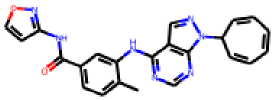	8.0	0.623362	2.525411	2.997881	5	0.770298

	src known mol	similarity	smiles	mol	Class	qed	logP	SAS	rings	kinase inhibition likeliness
**524**	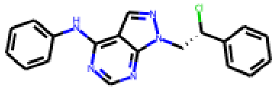	0.602827	Cc1ccc(C(=O)Nc2ccon2)cc1Nc1ncnc2c1cnn2C1cccccc1	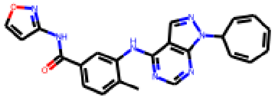	8.0	0.623362	2.525411	2.997881	5	0.770298
**525**	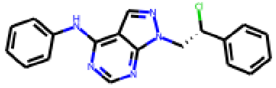	0.602827	Cc1ccc(C(=O)Nc2ccon2)cc1Nc1ncnc2c1cnn2C1cccccc1	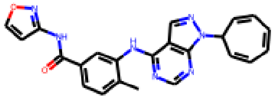	8.0	0.623362	2.525411	2.997881	5	0.770298
**526**	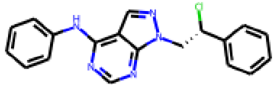	0.602827	Cc1ccc(C(=O)Nc2ccon2)cc1Nc1ncnc2c1cnn2C1cccccc1	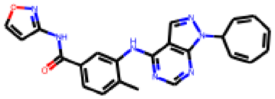	8.0	0.623362	2.525411	2.997881	5	0.770298
**527**	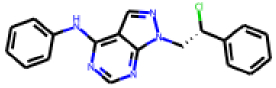	0.602827	Cc1ccc(C(=O)Nc2ccon2)cc1Nc1ncnc2c1cnn2C1cccccc1	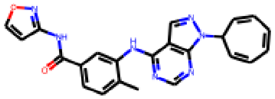	8.0	0.623362	2.525411	2.997881	5	0.770298

	src known mol	similarity	smiles	mol	Class	qed	logP	SAS	rings	kinase inhibition likeliness
**528**	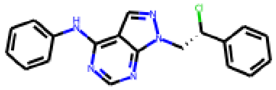	0.602827	Cc1ccc(C(=O)Nc2ccon2)cc1Nc1ncnc2c1cnn2C1cccccc1	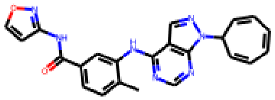	8.0	0.623362	2.525411	2.997881	5	0.770298
**529**	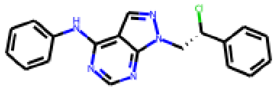	0.602827	Cc1ccc(C(=O)Nc2ccon2)cc1Nc1ncnc2c1cnn2C1cccccc1	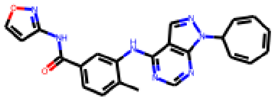	8.0	0.623362	2.525411	2.997881	5	0.770298
**530**	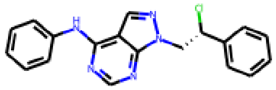	0.602827	Cc1ccc(C(=O)Nc2ccon2)cc1Nc1ncnc2c1cnn2C1cccccc1	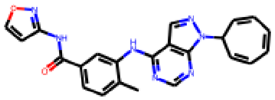	8.0	0.623362	2.525411	2.997881	5	0.770298
**531**	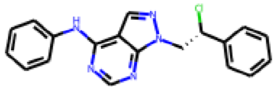	0.602827	Cc1ccc(C(=O)Nc2ccon2)cc1Nc1ncnc2c1cnn2C1cccccc1	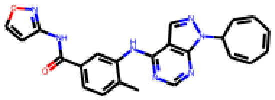	8.0	0.623362	2.525411	2.997881	5	0.770298

	src known mol	similarity	smiles	mol	Class	qed	logP	SAS	rings	kinase inhibition likeliness
**532**	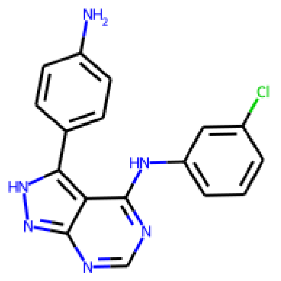	0.602542	c1ccc(Nc2ncnc3n[nH]c(-c4ccccc4)c23)cc1	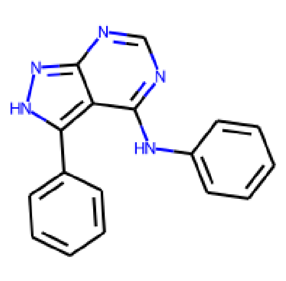	0.0	0.638163	3.995589	2.988471	4	0.800634
**533**	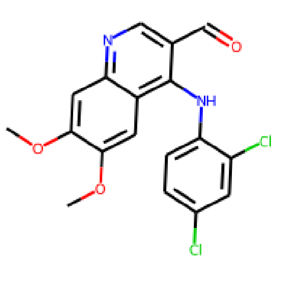	0.602453	COc1cc2ncc(C(=O)O)c(Nc3cccc(Cl)c3Cl)c2cc1OC1C(C)C1	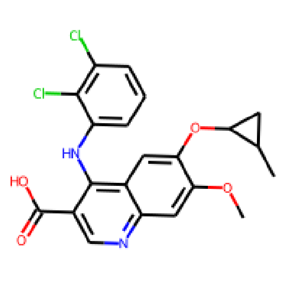	2.0	0.623362	2.525411	2.997881	4	0.774857
**534**	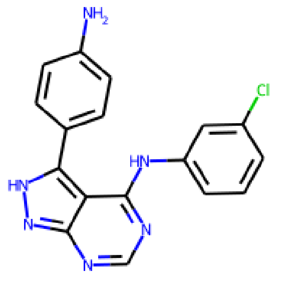	0.602379	c1ccc(Nc2ncnc3n[nH]c(-c4ccccc4)c23)cc1	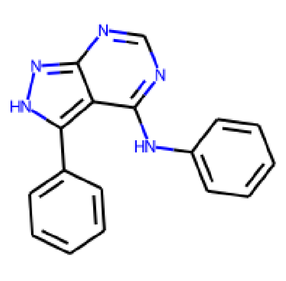	0.0	0.638163	3.995589	2.988471	4	0.800634
**535**	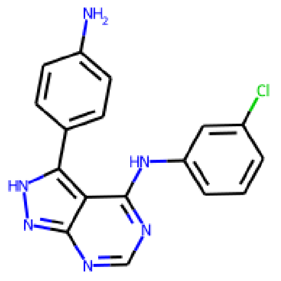	0.602168	c1ccc(Nc2ncnc3n[nH]c(-c4ccccc4)c23)cc1	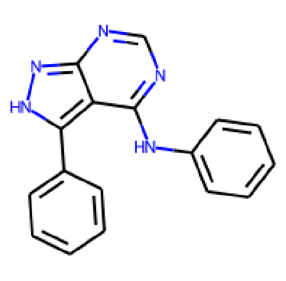	0.0	0.638163	3.995589	2.988471	4	0.800634

	src known mol	similarity	smiles	mol	Class	qed	logP	SAS	rings	kinase inhibition likeliness
**536**	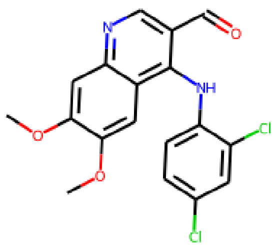	0.602113	COc1cc2ncc(C(=O)O)c(Nc3cccc(Cl)c3Cl)c2cc1OC1C(C)C1	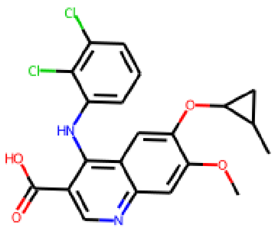	2.0	0.623362	2.525411	2.997881	4	0.774857
**537**	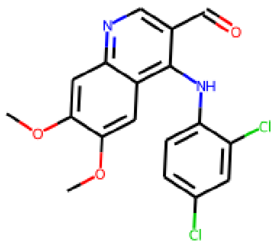	0.602113	COc1cc2ncc(C(=O)O)c(Nc3cccc(Cl)c3Cl)c2cc1OC1C(C)C1	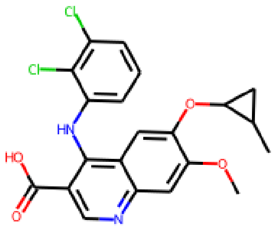	2.0	0.623362	2.525411	2.997881	4	0.774857
**538**	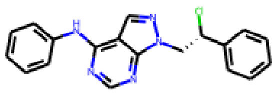	0.602000	Cc1ccc(C(=O)Nc2ccon2)cc1Nc1ncnc2c1cnn2C1cccccc1	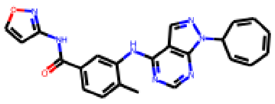	8.0	0.623362	2.525411	2.997881	5	0.770298
**539**	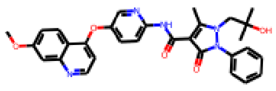	0.601677	COc1ccc2c(=O)cc(CNC(=O)c3c(C)n(C)n(-c4ccccc4)c3=O)nc3cccc2c3c1	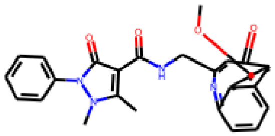	9.0	0.638163	3.995589	2.988471	5	0.774651

	src known mol	similarity	smiles	mol	Class	qed	logP	SAS	rings	kinase inhibition likeliness
**540**	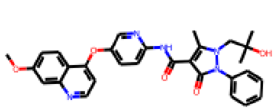	0.601677	COc1ccc2c(=O)cc(CNC(=O)c3c(C)n(C)n(-c4ccccc4)c3=O)nc3cccc2c3c1	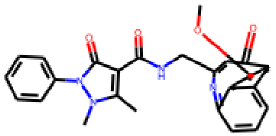	9.0	0.638163	3.995589	2.988471	5	0.774651
**541**	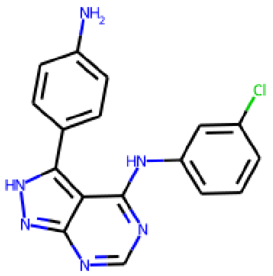	0.601529	c1ccc(Nc2ncnc3n[nH]c(-c4ccccc4)c23)cc1	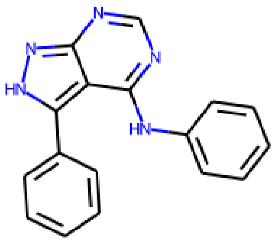	0.0	0.638163	3.995589	2.988471	4	0.800634
**542**	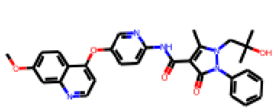	0.601457	COc1ccc2c(=O)cc(CNC(=O)c3c(C)n(C)n(-c4ccccc4)c3=O)nc3cccc2c3c1	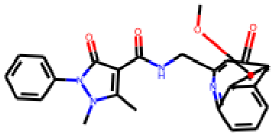	9.0	0.638163	3.995589	2.988471	5	0.774651
**543**	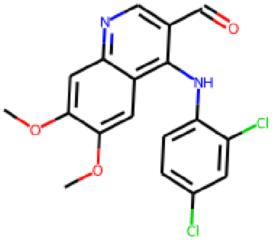	0.601439	COc1cc2ncc(C(=O)O)c(Nc3cccc(Cl)c3Cl)c2cc1OC1C(C)C1	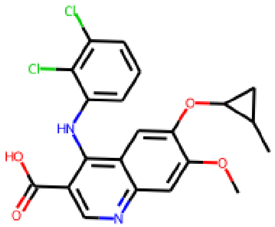	2.0	0.623362	2.525411	2.997881	4	0.774857

	src known mol	similarity	smiles	mol	Class	qed	logP	SAS	rings	kinase inhibition likeliness
**544**	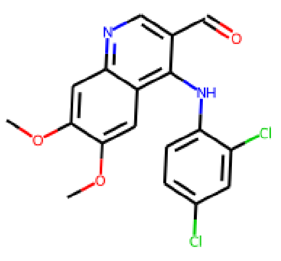	0.601439	COc1cc2ncc(C(=O)O)c(Nc3cccc(Cl)c3Cl)c2cc1OC1C(C)C1	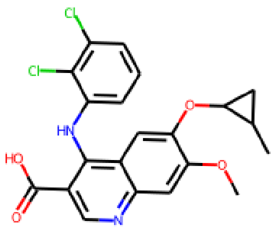	2.0	0.623362	2.525411	2.997881	4	0.774857
**545**	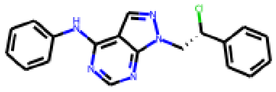	0.601389	Cc1ccc(C(=O)Nc2ccon2)cc1Nc1ncnc2c1cnn2C1cccccc1	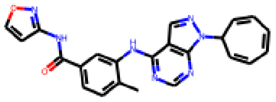	8.0	0.623362	2.525411	2.997881	5	0.770298
**546**	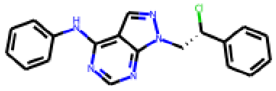	0.601389	Cc1ccc(C(=O)Nc2ccon2)cc1Nc1ncnc2c1cnn2C1cccccc1	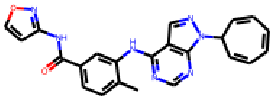	8.0	0.623362	2.525411	2.997881	5	0.770298
**547**	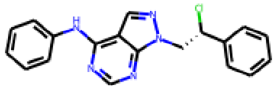	0.601389	Cc1ccc(C(=O)Nc2ccon2)cc1Nc1ncnc2c1cnn2C1cccccc1	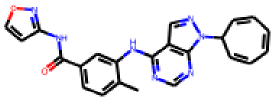	8.0	0.623362	2.525411	2.997881	5	0.770298

	src known mol	similarity	smiles	mol	Class	qed	logP	SAS	rings	kinase inhibition likeliness
**548**	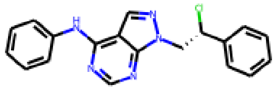	0.601389	Cc1ccc(C(=O)Nc2ccon2)cc1Nc1ncnc2c1cnn2C1cccccc1	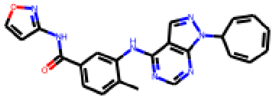	8.0	0.623362	2.525411	2.997881	5	0.770298
**549**	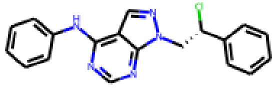	0.601389	Cc1ccc(C(=O)Nc2ccon2)cc1Nc1ncnc2c1cnn2C1cccccc1	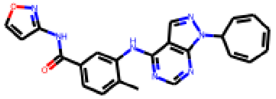	8.0	0.623362	2.525411	2.997881	5	0.770298
**550**	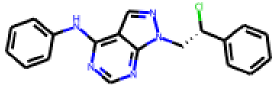	0.601389	Cc1ccc(C(=O)Nc2ccon2)cc1Nc1ncnc2c1cnn2C1cccccc1	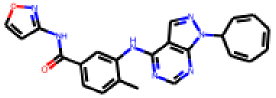	8.0	0.623362	2.525411	2.997881	5	0.770298
**551**	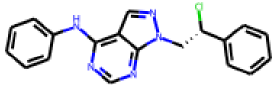	0.601389	Cc1ccc(C(=O)Nc2ccon2)cc1Nc1ncnc2c1cnn2C1cccccc1	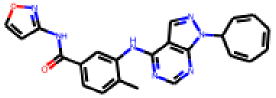	8.0	0.623362	2.525411	2.997881	5	0.770298

	src known mol	similarity	smiles	mol	Class	qed	logP	SAS	rings	kinase inhibition likeliness
**552**	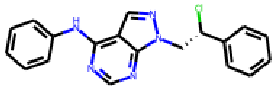	0.601389	Cc1ccc(C(=O)Nc2ccon2)cc1Nc1ncnc2c1cnn2C1cccccc1	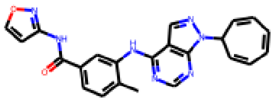	8.0	0.623362	2.525411	2.997881	5	0.770298
**553**	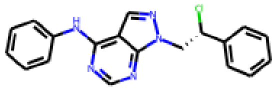	0.601389	Cc1ccc(C(=O)Nc2ccon2)cc1Nc1ncnc2c1cnn2C1cccccc1	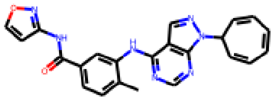	8.0	0.623362	2.525411	2.997881	5	0.770298
**554**	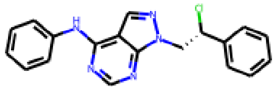	0.601389	Cc1ccc(C(=O)Nc2ccon2)cc1Nc1ncnc2c1cnn2C1cccccc1	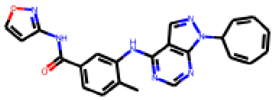	8.0	0.623362	2.525411	2.997881	5	0.770298
**555**	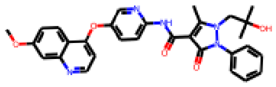	0.601362	COc1ccc2c(=O)cc(CNC(=O)c3c(C)n(C)n(-c4ccccc4)c3=O)nc3cccc2c3c1	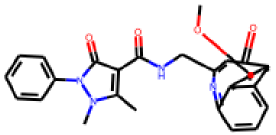	9.0	0.638163	3.995589	2.988471	5	0.774651

	src known mol	similarity	smiles	mol	Class	qed	logP	SAS	rings	kinase inhibition likeliness
**556**	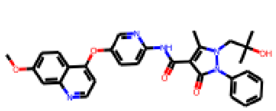	0.601362	COc1ccc2c(=O)cc(CNC(=O)c3c(C)n(C)n(-c4ccccc4)c3=O)nc3cccc2c3c1	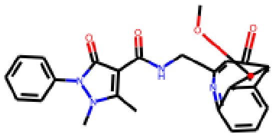	9.0	0.638163	3.995589	2.988471	5	0.774651
**557**	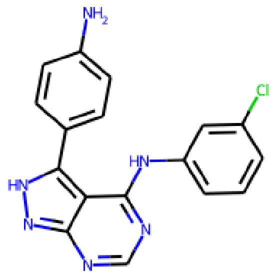	0.601190	c1ccc(Nc2ncnc3n[nH]c(-c4ccccc4)c23)cc1	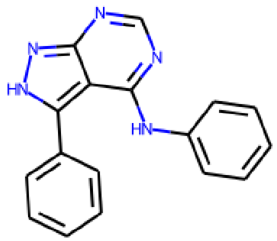	0.0	0.638163	3.995589	2.988471	4	0.800634
**558**	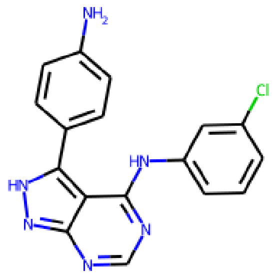	0.601019	c1ccc(Nc2ncnc3n[nH]c(-c4ccccc4)c23)cc1	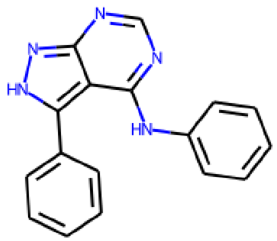	0.0	0.638163	3.995589	2.988471	4	0.800634
**559**	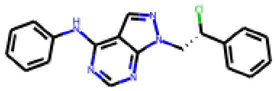	0.600978	Cc1ccc(C(=O)Nc2ccon2)cc1Nc1ncnc2c1cnn2C1cccccc1	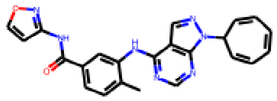	8.0	0.623362	2.525411	2.997881	5	0.770298

	src known mol	similarity	smiles	mol	Class	qed	logP	SAS	rings	kinase inhibition likeliness
**560**	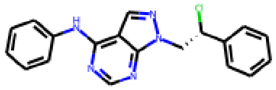	0.600978	Cc1ccc(C(=O)Nc2ccon2)cc1Nc1ncnc2c1cnn2C1cccccc1	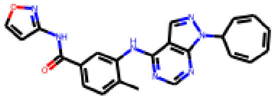	8.0	0.623362	2.525411	2.997881	5	0.770298
**561**	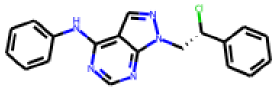	0.600978	Cc1ccc(C(=O)Nc2ccon2)cc1Nc1ncnc2c1cnn2C1cccccc1	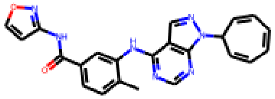	8.0	0.623362	2.525411	2.997881	5	0.770298
**562**	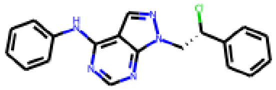	0.600978	Cc1ccc(C(=O)Nc2ccon2)cc1Nc1ncnc2c1cnn2C1cccccc1	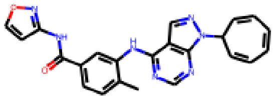	8.0	0.623362	2.525411	2.997881	5	0.770298
**563**	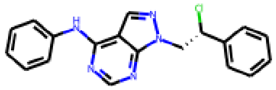	0.600877	Cc1ccc(C(=O)Nc2ccon2)cc1Nc1ncnc2c1cnn2C1cccccc1	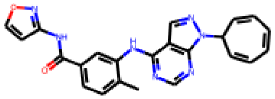	8.0	0.623362	2.525411	2.997881	5	0.770298

	src known mol	similarity	smiles	mol	Class	qed	logP	SAS	rings	kinase inhibition likeliness
**564**	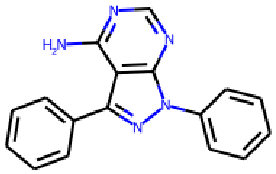	0.600633	Cc1ccc(C(=O)Oc2ccon2)cc1-c1ncnc2c1cnn2-c1ccccc1	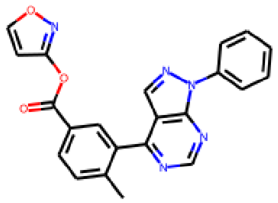	8.0	0.635315	4.542409	3.856266	5	0.760056
**565**	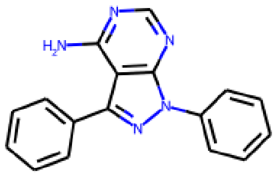	0.600506	Cc1ccc(C(=O)Oc2ccon2)cc1-c1ncnc2c1cnn2-c1ccccc1	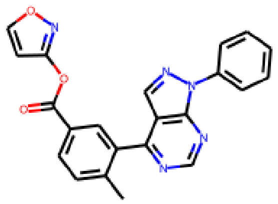	8.0	0.635315	4.542409	3.856266	5	0.760056
**566**	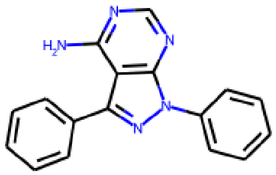	0.600506	Cc1ccc(C(=O)Oc2ccon2)cc1-c1ncnc2c1cnn2-c1ccccc1	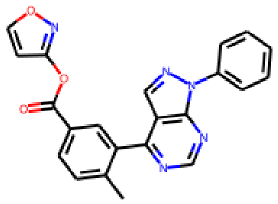	8.0	0.635315	4.542409	3.856266	5	0.760056
**567**	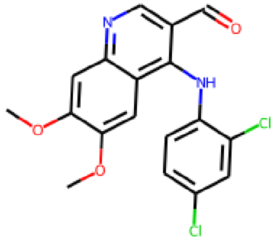	0.600482	COc1cc2ncc(C(=O)O)c(Nc3cccc(Cl)c3Cl)c2cc1OC1C(C)C1	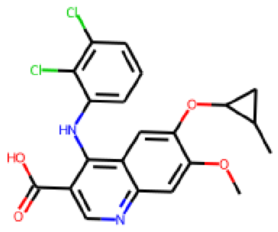	2.0	0.623362	2.525411	2.997881	4	0.774857

	src known mol	similarity	smiles	mol	Class	qed	logP	SAS	rings	kinase inhibition likeliness
**568**	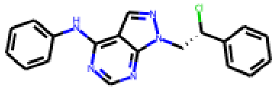	0.600426	Cc1ccc(C(=O)Nc2ccon2)cc1Nc1ncnc2c1cnn2C1cccccc1	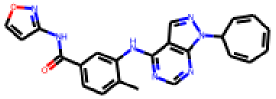	8.0	0.623362	2.525411	2.997881	5	0.770298
**569**	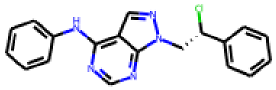	0.600426	Cc1ccc(C(=O)Nc2ccon2)cc1Nc1ncnc2c1cnn2C1cccccc1	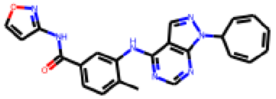	8.0	0.623362	2.525411	2.997881	5	0.770298
**570**	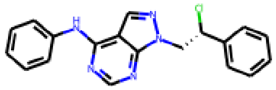	0.600255	Cc1ccc(C(=O)Nc2ccon2)cc1Nc1ncnc2c1cnn2C1cccccc1	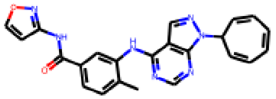	8.0	0.623362	2.525411	2.997881	5	0.770298
**571**	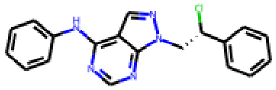	0.600141	Cc1ccc(C(=O)Nc2ccon2)cc1Nc1ncnc2c1cnn2C1cccccc1	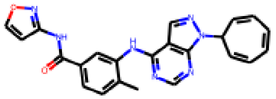	8.0	0.623362	2.525411	2.997881	5	0.770298

	src known mol	similarity	smiles	mol	Class	qed	logP	SAS	rings	kinase inhibition likeliness
**572**	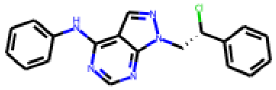	0.600141	Cc1ccc(C(=O)Nc2ccon2)cc1Nc1ncnc2c1cnn2C1cccccc1	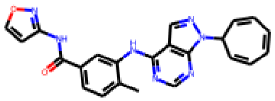	8.0	0.623362	2.525411	2.997881	5	0.770298
**573**	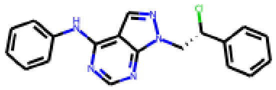	0.600141	Cc1ccc(C(=O)Nc2ccon2)cc1Nc1ncnc2c1cnn2C1cccccc1	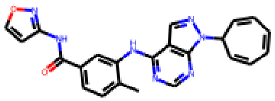	8.0	0.623362	2.525411	2.997881	5	0.770298
**574**	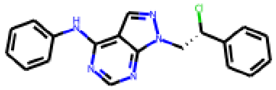	0.600141	Cc1ccc(C(=O)Nc2ccon2)cc1Nc1ncnc2c1cnn2C1cccccc1	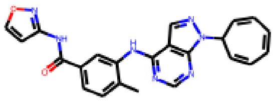	8.0	0.623362	2.525411	2.997881	5	0.770298
**575**	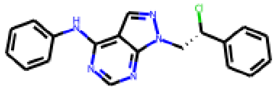	0.600141	Cc1ccc(C(=O)Nc2ccon2)cc1Nc1ncnc2c1cnn2C1cccccc1	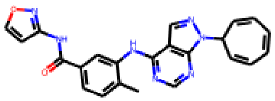	8.0	0.623362	2.525411	2.997881	5	0.770298

	src known mol	similarity	smiles	mol	Class	qed	logP	SAS	rings	kinase inhibition likeliness
**576**	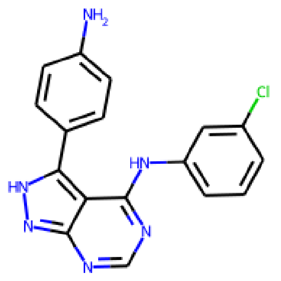	0.600000	c1ccc(Nc2ncnc3n[nH]c(-c4ccccc4)c23)cc1	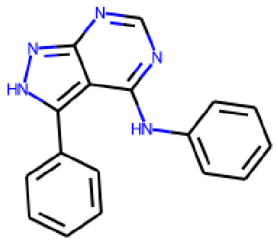	0.0	0.638163	3.995589	2.988471	4	0.800634
**577**	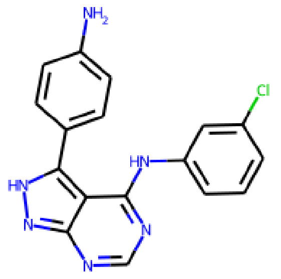	0.600000	c1ccc(Nc2ncnc3n[nH]c(-c4ccccc4)c23)cc1	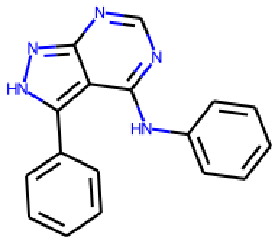	0.0	0.638163	3.995589	2.988471	4	0.800634
**578**	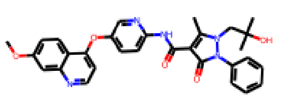	0.599581	COc1ccc2c(=O)cc(CNC(=O)c3c(C)n(C)n(-c4ccccc4)c3=O)nc3cccc2c3c1	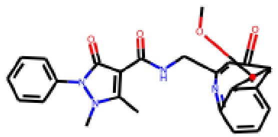	9.0	0.638163	3.995589	2.988471	5	0.774651
**579**	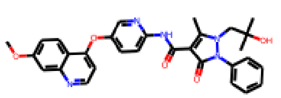	0.599581	COc1ccc2c(=O)cc(CNC(=O)c3c(C)n(C)n(-c4ccccc4)c3=O)nc3cccc2c3c1	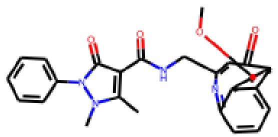	9.0	0.638163	3.995589	2.988471	5	0.774651

	src known mol	similarity	smiles	mol	Class	qed	logP	SAS	rings	kinase inhibition likeliness
**580**	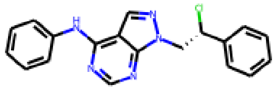	0.599576	Cc1ccc(C(=O)Nc2ccon2)cc1Nc1ncnc2c1cnn2C1cccccc1	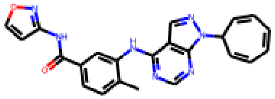	8.0	0.623362	2.525411	2.997881	5	0.770298
**581**	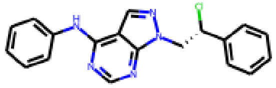	0.599576	Cc1ccc(C(=O)Nc2ccon2)cc1Nc1ncnc2c1cnn2C1cccccc1	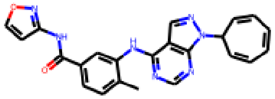	8.0	0.623362	2.525411	2.997881	5	0.770298
**582**	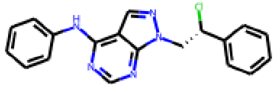	0.599576	Cc1ccc(C(=O)Nc2ccon2)cc1Nc1ncnc2c1cnn2C1cccccc1	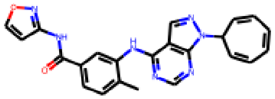	8.0	0.623362	2.525411	2.997881	5	0.770298
**583**	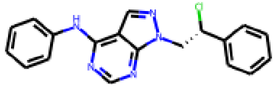	0.599576	Cc1ccc(C(=O)Nc2ccon2)cc1Nc1ncnc2c1cnn2C1cccccc1	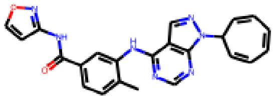	8.0	0.623362	2.525411	2.997881	5	0.770298

	src known mol	similarity	smiles	mol	Class	qed	logP	SAS	rings	kinase inhibition likeliness
**584**	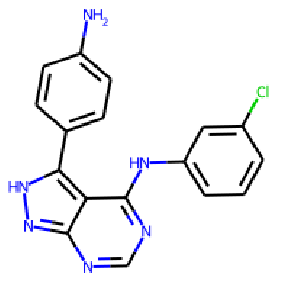	0.599490	c1ccc(Nc2ncnc3n[nH]c(-c4ccccc4)c23)cc1	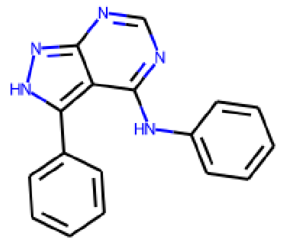	0.0	0.638163	3.995589	2.988471	4	0.800634
**585**	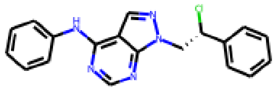	0.599453	Cc1ccc(C(=O)Nc2ccon2)cc1Nc1ncnc2c1cnn2C1cccccc1	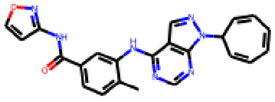	8.0	0.623362	2.525411	2.997881	5	0.770298
**586**	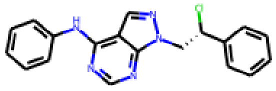	0.599453	Cc1ccc(C(=O)Nc2ccon2)cc1Nc1ncnc2c1cnn2C1cccccc1	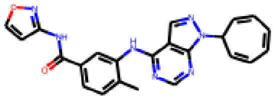	8.0	0.623362	2.525411	2.997881	5	0.770298
**587**	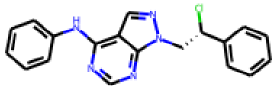	0.599453	Cc1ccc(C(=O)Nc2ccon2)cc1Nc1ncnc2c1cnn2C1cccccc1	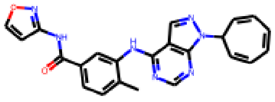	8.0	0.623362	2.525411	2.997881	5	0.770298

	src known mol	similarity	smiles	mol	Class	qed	logP	SAS	rings	kinase inhibition likeliness
**588**	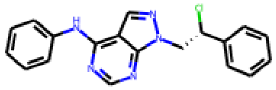	0.599453	Cc1ccc(C(=O)Nc2ccon2)cc1Nc1ncnc2c1cnn2C1cccccc1	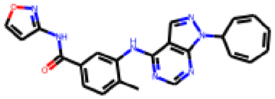	8.0	0.623362	2.525411	2.997881	5	0.770298
**589**	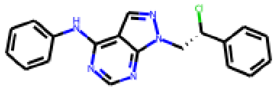	0.599453	Cc1ccc(C(=O)Nc2ccon2)cc1Nc1ncnc2c1cnn2C1cccccc1	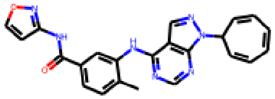	8.0	0.623362	2.525411	2.997881	5	0.770298
**590**	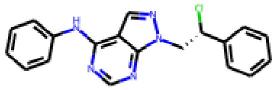	0.599453	Cc1ccc(C(=O)Nc2ccon2)cc1Nc1ncnc2c1cnn2C1cccccc1	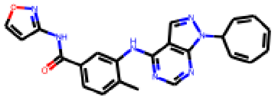	8.0	0.623362	2.525411	2.997881	5	0.770298
**591**	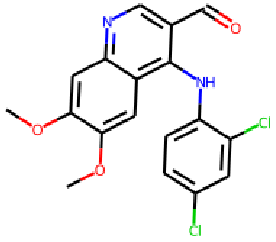	0.599415	COc1cc2ncc(C(=O)O)c(Nc3cccc(Cl)c3Cl)c2cc1OC1C(C)C1	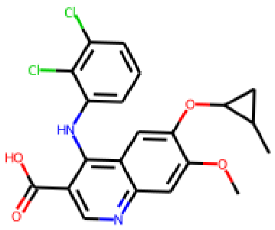	2.0	0.623362	2.525411	2.997881	4	0.774857

	src known mol	similarity	smiles	mol	Class	qed	logP	SAS	rings	kinase inhibition likeliness
**592**	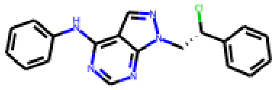	0.599365	Cc1ccc(C(=O)Nc2ccon2)cc1Nc1ncnc2c1cnn2C1cccccc1	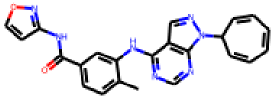	8.0	0.623362	2.525411	2.997881	5	0.770298
**593**	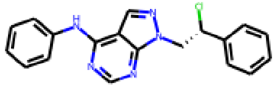	0.599281	Cc1ccc(C(=O)Nc2ccon2)cc1Nc1ncnc2c1cnn2C1cccccc1	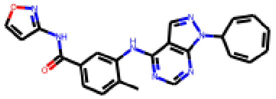	8.0	0.623362	2.525411	2.997881	5	0.770298
**594**	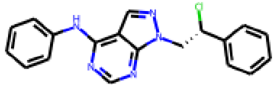	0.599281	Cc1ccc(C(=O)Nc2ccon2)cc1Nc1ncnc2c1cnn2C1cccccc1	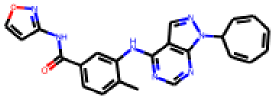	8.0	0.623362	2.525411	2.997881	5	0.770298
**595**	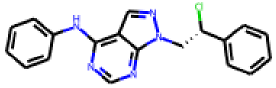	0.599159	Cc1ccc(C(=O)Nc2ccon2)cc1Nc1ncnc2c1cnn2C1cccccc1	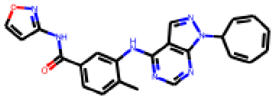	8.0	0.623362	2.525411	2.997881	5	0.770298

	src known mol	similarity	smiles	mol	Class	qed	logP	SAS	rings	kinase inhibition likeliness
**596**	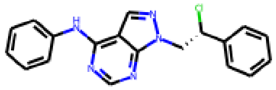	0.599159	Cc1ccc(C(=O)Nc2ccon2)cc1Nc1ncnc2c1cnn2C1cccccc1	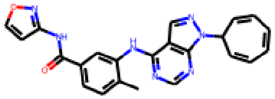	8.0	0.623362	2.525411	2.997881	5	0.770298
**597**	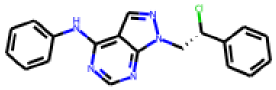	0.599159	Cc1ccc(C(=O)Nc2ccon2)cc1Nc1ncnc2c1cnn2C1cccccc1	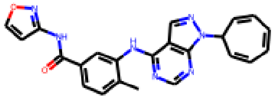	8.0	0.623362	2.525411	2.997881	5	0.770298
**598**	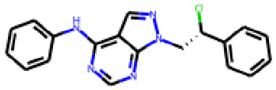	0.599159	Cc1ccc(C(=O)Nc2ccon2)cc1Nc1ncnc2c1cnn2C1cccccc1	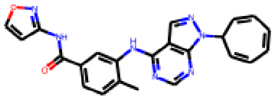	8.0	0.623362	2.525411	2.997881	5	0.770298
**599**	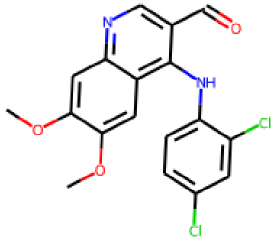	0.599034	COc1cc2ncc(C(=O)O)c(Nc3cccc(Cl)c3Cl)c2cc1OC1C(C)C1	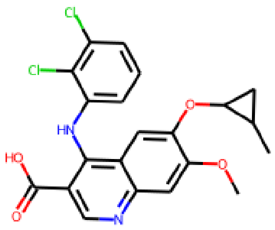	2.0	0.623362	2.525411	2.997881	4	0.774857

	src known mol	similarity	smiles	mol	Class	qed	logP	SAS	rings	kinase inhibition likeliness
**600**	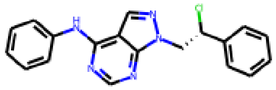	0.598870	Cc1ccc(C(=O)Nc2ccon2)cc1Nc1ncnc2c1cnn2C1cccccc1	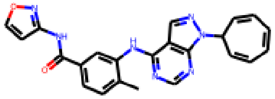	8.0	0.623362	2.525411	2.997881	5	0.770298
**601**	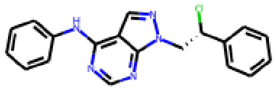	0.598870	Cc1ccc(C(=O)Nc2ccon2)cc1Nc1ncnc2c1cnn2C1cccccc1	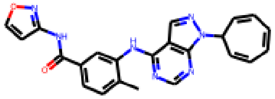	8.0	0.623362	2.525411	2.997881	5	0.770298
**602**	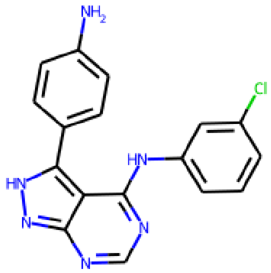	0.598818	c1ccc(Nc2ncnc3n[nH]c(-c4ccccc4)c23)cc1	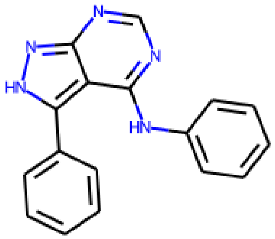	0.0	0.638163	3.995589	2.988471	4	0.800634
**603**	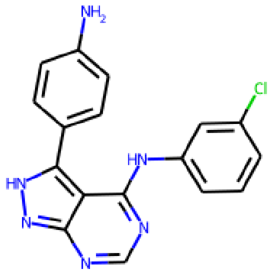	0.598510	c1ccc(Nc2ncnc3n[nH]c(-c4ccccc4)c23)cc1	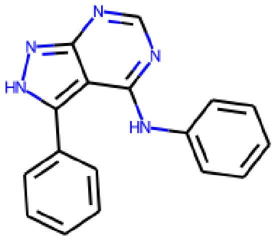	0.0	0.638163	3.995589	2.988471	4	0.800634

	src known mol	similarity	smiles	mol	Class	qed	logP	SAS	rings	kinase inhibition likeliness
**604**	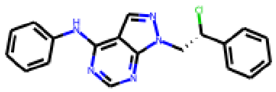	0.598470	Cc1ccc(C(=O)Nc2ccon2)cc1Nc1ncnc2c1cnn2C1cccccc1	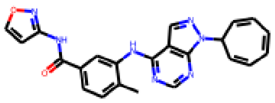	8.0	0.623362	2.525411	2.997881	5	0.770298
**605**	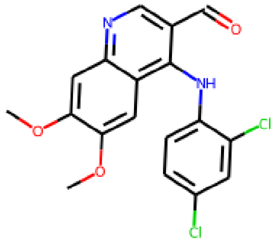	0.598425	COc1cc2ncc(C(=O)O)c(Nc3cccc(Cl)c3Cl)c2cc1OC1C(C)C1	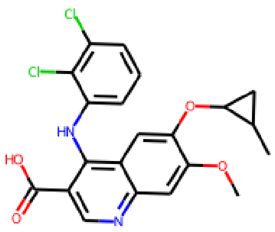	2.0	0.623362	2.525411	2.997881	4	0.774857
**606**	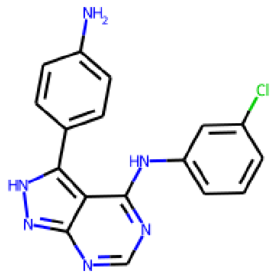	0.598312	c1ccc(Nc2ncnc3n[nH]c(-c4ccccc4)c23)cc1	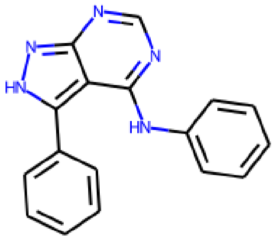	0.0	0.638163	3.995589	2.988471	4	0.800634
**607**	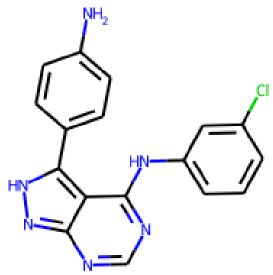	0.598305	c1ccc(Nc2ncnc3n[nH]c(-c4ccccc4)c23)cc1	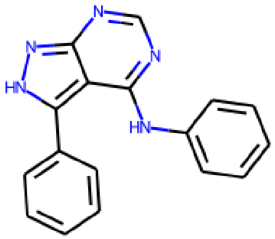	0.0	0.638163	3.995589	2.988471	4	0.800634

	src known mol	similarity	smiles	mol	Class	qed	logP	SAS	rings	kinase inhibition likeliness
**608**	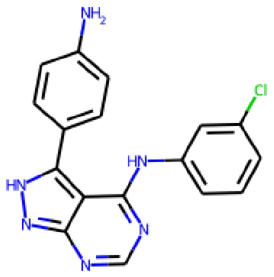	0.598305	c1ccc(Nc2ncnc3n[nH]c(-c4ccccc4)c23)cc1	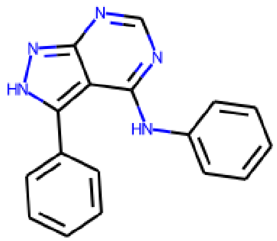	0.0	0.638163	3.995589	2.988471	4	0.800634
**609**	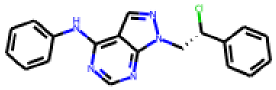	0.598247	Cc1ccc(C(=O)Nc2ccon2)cc1Nc1ncnc2c1cnn2C1cccccc1	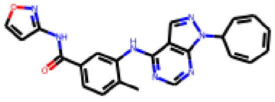	8.0	0.623362	2.525411	2.997881	5	0.770298
**610**	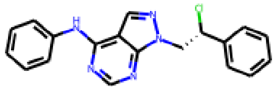	0.598164	Cc1ccc(C(=O)Nc2ccon2)cc1Nc1ncnc2c1cnn2C1cccccc1	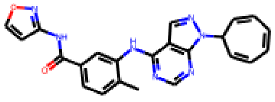	8.0	0.623362	2.525411	2.997881	5	0.770298
**611**	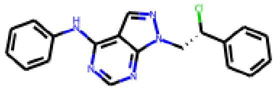	0.598164	Cc1ccc(C(=O)Nc2ccon2)cc1Nc1ncnc2c1cnn2C1cccccc1	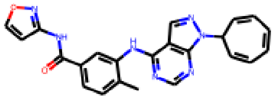	8.0	0.623362	2.525411	2.997881	5	0.770298

	src known mol	similarity	smiles	mol	Class	qed	logP	SAS	rings	kinase inhibition likeliness
**612**	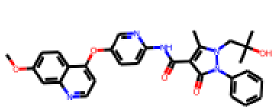	0.598121	COc1ccc2c(=O)cc(CNC(=O)c3c(C)n(C)n(-c4ccccc4)c3=O)nc3cccc2c3c1	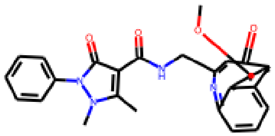	9.0	0.638163	3.995589	2.988471	5	0.774651
**613**	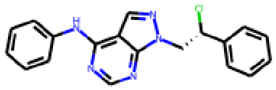	0.598039	Cc1ccc(C(=O)Nc2ccon2)cc1Nc1ncnc2c1cnn2C1cccccc1	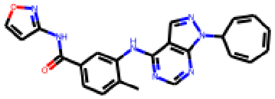	8.0	0.623362	2.525411	2.997881	5	0.770298
**614**	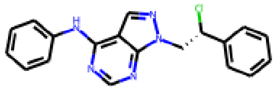	0.598039	Cc1ccc(C(=O)Nc2ccon2)cc1Nc1ncnc2c1cnn2C1cccccc1	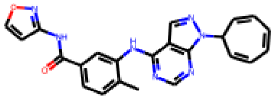	8.0	0.623362	2.525411	2.997881	5	0.770298
**615**	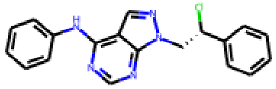	0.597964	Cc1ccc(C(=O)Nc2ccon2)cc1Nc1ncnc2c1cnn2C1cccccc1	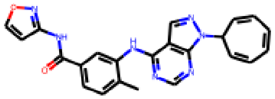	8.0	0.623362	2.525411	2.997881	5	0.770298

	src known mol	similarity	smiles	mol	Class	qed	logP	SAS	rings	kinase inhibition likeliness
**616**	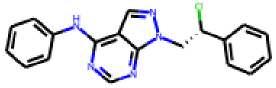	0.597917	Cc1ccc(C(=O)Nc2ccon2)cc1Nc1ncnc2c1cnn2C1cccccc1	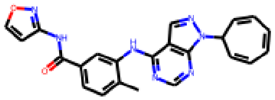	8.0	0.623362	2.525411	2.997881	5	0.770298
**617**	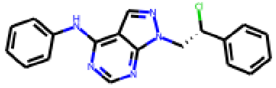	0.597917	Cc1ccc(C(=O)Nc2ccon2)cc1Nc1ncnc2c1cnn2C1cccccc1	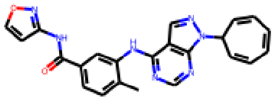	8.0	0.623362	2.525411	2.997881	5	0.770298
**618**	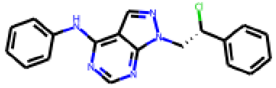	0.597810	Cc1ccc(C(=O)Nc2ccon2)cc1Nc1ncnc2c1cnn2C1cccccc1	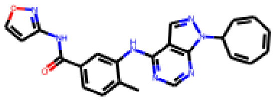	8.0	0.623362	2.525411	2.997881	5	0.770298
**619**	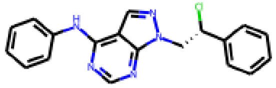	0.597537	Cc1ccc(C(=O)Nc2ccon2)cc1Nc1ncnc2c1cnn2C1cccccc1	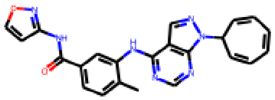	8.0	0.623362	2.525411	2.997881	5	0.770298

	src known mol	similarity	smiles	mol	Class	qed	logP	SAS	rings	kinase inhibition likeliness
**620**	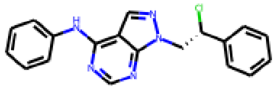	0.597467	Cc1ccc(C(=O)Nc2ccon2)cc1Nc1ncnc2c1cnn2C1cccccc1	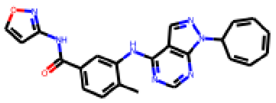	8.0	0.623362	2.525411	2.997881	5	0.770298
**621**	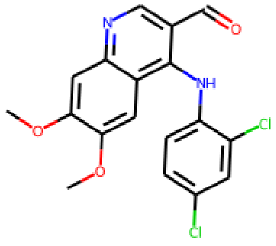	0.597458	COc1cc2ncc(C(=O)O)c(Nc3cccc(Cl)c3Cl)c2cc1OC1C(C)C1	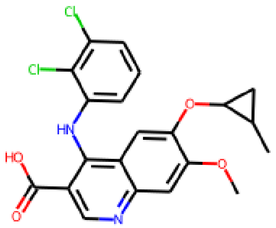	2.0	0.623362	2.525411	2.997881	4	0.774857
**622**	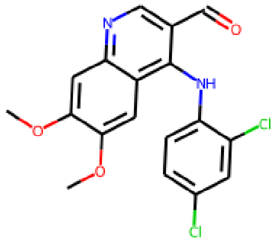	0.597458	COc1cc2ncc(C(=O)O)c(Nc3cccc(Cl)c3Cl)c2cc1OC1C(C)C1	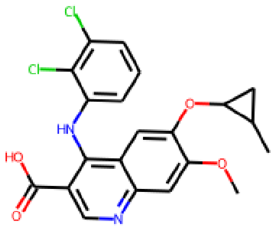	2.0	0.623362	2.525411	2.997881	4	0.774857
**623**	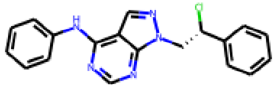	0.597411	Cc1ccc(C(=O)Nc2ccon2)cc1Nc1ncnc2c1cnn2C1cccccc1	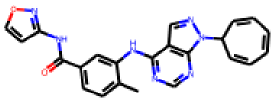	8.0	0.623362	2.525411	2.997881	5	0.770298

	src known mol	similarity	smiles	mol	Class	qed	logP	SAS	rings	kinase inhibition likeliness
**624**	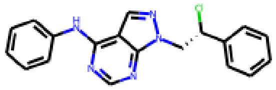	0.597411	Cc1ccc(C(=O)Nc2ccon2)cc1Nc1ncnc2c1cnn2C1cccccc1	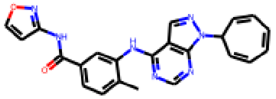	8.0	0.623362	2.525411	2.997881	5	0.770298
**625**	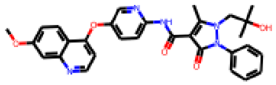	0.597389	COc1ccc2c(=O)cc(CNC(=O)c3c(C)n(C)n(-c4ccccc4)c3=O)nc3cccc2c3c1	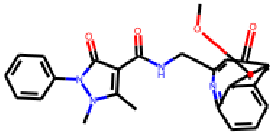	9.0	0.638163	3.995589	2.988471	5	0.774651
**626**	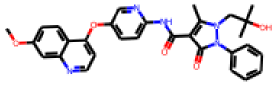	0.597389	COc1ccc2c(=O)cc(CNC(=O)c3c(C)n(C)n(-c4ccccc4)c3=O)nc3cccc2c3c1	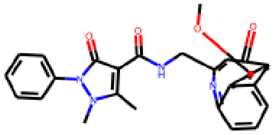	9.0	0.638163	3.995589	2.988471	5	0.774651
**627**	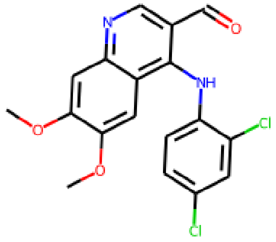	0.597321	COc1cc2ncc(C(=O)O)c(Nc3cccc(Cl)c3Cl)c2cc1OC1C(C)C1	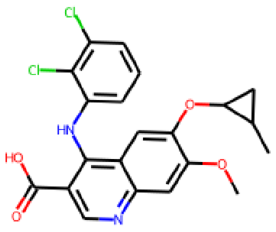	2.0	0.623362	2.525411	2.997881	4	0.774857

	src known mol	similarity	smiles	mol	Class	qed	logP	SAS	rings	kinase inhibition likeliness
**628**	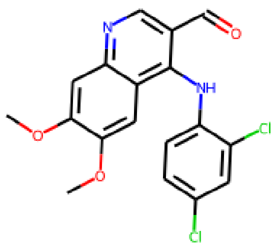	0.597311	COc1cc2ncc(C(=O)O)c(Nc3cccc(Cl)c3Cl)c2cc1OC1C(C)C1	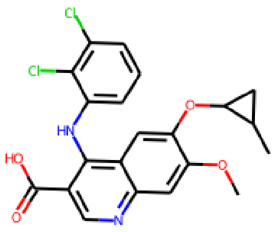	2.0	0.623362	2.525411	2.997881	4	0.774857
**629**	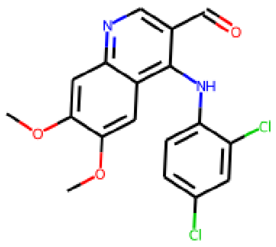	0.597311	COc1cc2ncc(C(=O)O)c(Nc3cccc(Cl)c3Cl)c2cc1OC1C(C)C1	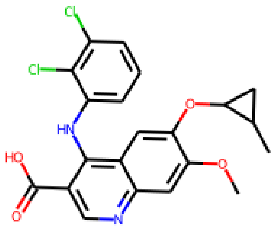	2.0	0.623362	2.525411	2.997881	4	0.774857
**630**	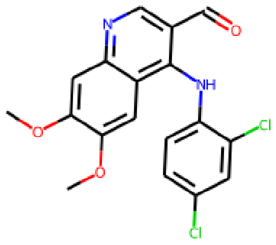	0.597282	COc1cc2ncc(C(=O)O)c(Nc3cccc(Cl)c3Cl)c2cc1OC1C(C)C1	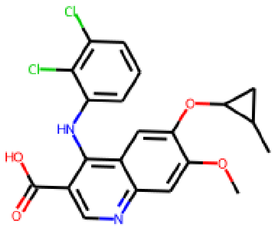	2.0	0.623362	2.525411	2.997881	4	0.774857
**631**	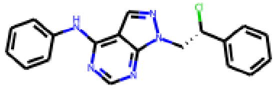	0.597213	Cc1ccc(C(=O)Nc2ccon2)cc1Nc1ncnc2c1cnn2C1cccccc1	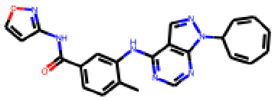	8.0	0.623362	2.525411	2.997881	5	0.770298

	src known mol	similarity	smiles	mol	Class	qed	logP	SAS	rings	kinase inhibition likeliness
**632**	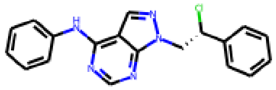	0.597213	Cc1ccc(C(=O)Nc2ccon2)cc1Nc1ncnc2c1cnn2C1cccccc1	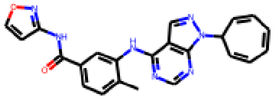	8.0	0.623362	2.525411	2.997881	5	0.770298
**633**	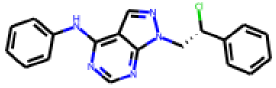	0.597213	Cc1ccc(C(=O)Nc2ccon2)cc1Nc1ncnc2c1cnn2C1cccccc1	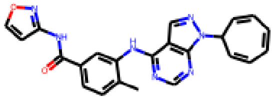	8.0	0.623362	2.525411	2.997881	5	0.770298
**634**	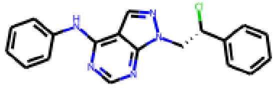	0.597213	Cc1ccc(C(=O)Nc2ccon2)cc1Nc1ncnc2c1cnn2C1cccccc1	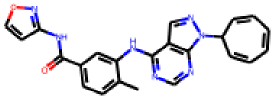	8.0	0.623362	2.525411	2.997881	5	0.770298
**635**	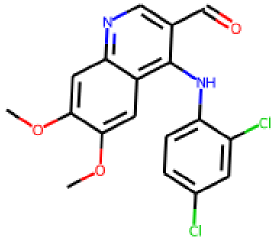	0.597112	COc1cc2ncc(C(=O)O)c(Nc3cccc(Cl)c3Cl)c2cc1OC1C(C)C1	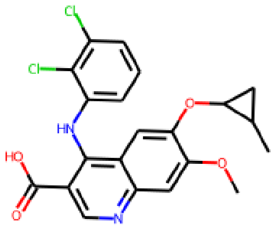	2.0	0.623362	2.525411	2.997881	4	0.774857

	src known mol	similarity	smiles	mol	Class	qed	logP	SAS	rings	kinase inhibition likeliness
**636**	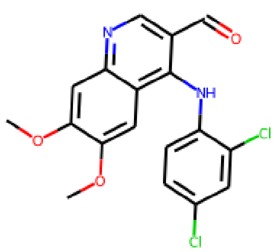	0.597112	COc1cc2ncc(C(=O)O)c(Nc3cccc(Cl)c3Cl)c2cc1OC1C(C)C1	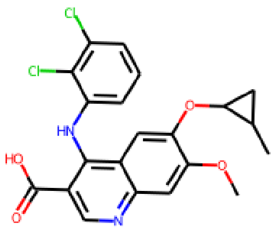	2.0	0.623362	2.525411	2.997881	4	0.774857
**637**	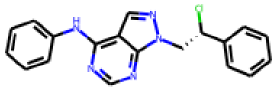	0.596932	Cc1ccc(C(=O)Nc2ccon2)cc1Nc1ncnc2c1cnn2C1cccccc1	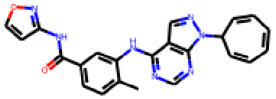	8.0	0.623362	2.525411	2.997881	5	0.770298
**638**	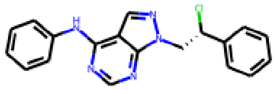	0.596932	Cc1ccc(C(=O)Nc2ccon2)cc1Nc1ncnc2c1cnn2C1cccccc1	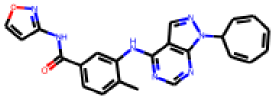	8.0	0.623362	2.525411	2.997881	5	0.770298
**639**	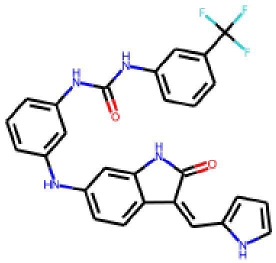	0.596681	COc1ccc(NC(=O)Nc2ccc3c(c2)NC(=O)/C3=C/c2ccc[nH]2)cc1	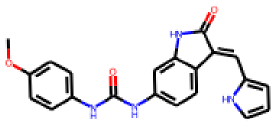	4.0	0.638163	3.995589	2.988471	4	0.768944

	src known mol	similarity	smiles	mol	Class	qed	logP	SAS	rings	kinase inhibition likeliness
**640**	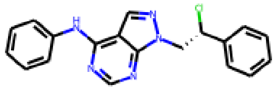	0.596662	Cc1ccc(C(=O)Nc2ccon2)cc1Nc1ncnc2c1cnn2C1cccccc1	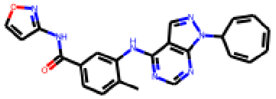	8.0	0.623362	2.525411	2.997881	5	0.770298
**641**	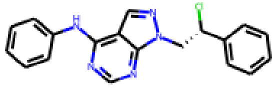	0.596662	Cc1ccc(C(=O)Nc2ccon2)cc1Nc1ncnc2c1cnn2C1cccccc1	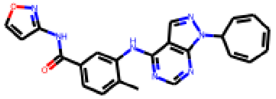	8.0	0.623362	2.525411	2.997881	5	0.770298
**642**	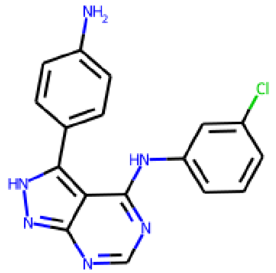	0.596462	c1ccc(Nc2ncnc3n[nH]c(-c4ccccc4)c32)cc1	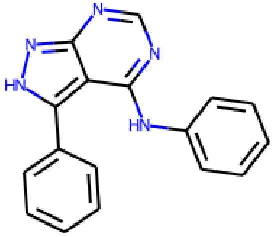	0.0	0.520676	2.336694	2.899742	4	0.831417
**643**	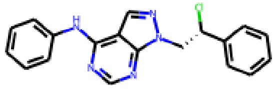	0.596364	Cc1ccc(C(=O)Nc2ccon2)cc1Nc1ncnc2c1cnn2C1cccccc1	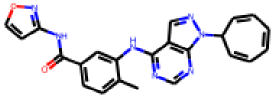	8.0	0.623362	2.525411	2.997881	5	0.770298

	src known mol	similarity	smiles	mol	Class	qed	logP	SAS	rings	kinase inhibition likeliness
**644**	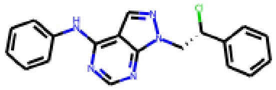	0.596273	Cc1ccc(C(=O)Nc2ccon2)cc1Nc1ncnc2c1cnn2C1cccccc1	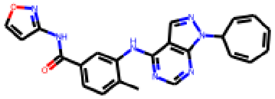	8.0	0.623362	2.525411	2.997881	5	0.770298
**645**	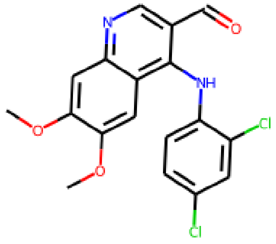	0.596273	COc1cc2ncc(C(=O)O)c(Nc3cccc(Cl)c3Cl)c2cc1OC1C(C)C1	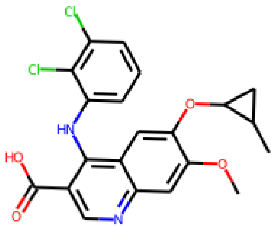	2.0	0.623362	2.525411	2.997881	4	0.774857
**646**	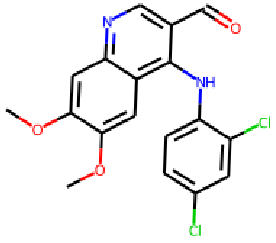	0.596264	COc1cc2ncc(C(=O)O)c(Nc3cccc(Cl)c3Cl)c2cc1OC1C(C)C1	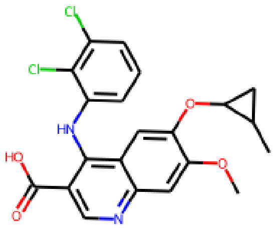	2.0	0.623362	2.525411	2.997881	4	0.774857
**647**	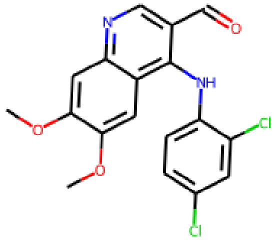	0.596264	COc1cc2ncc(C(=O)O)c(Nc3cccc(Cl)c3Cl)c2cc1OC1C(C)C1	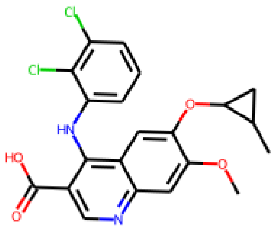	2.0	0.623362	2.525411	2.997881	4	0.774857

	src known mol	similarity	smiles	mol	Class	qed	logP	SAS	rings	kinase inhibition likeliness
**648**	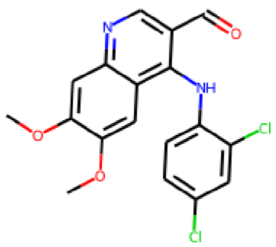	0.596217	COc1cc2ncc(C(=O)O)c(Nc3cccc(Cl)c3Cl)c2cc1OC1C(C)C1	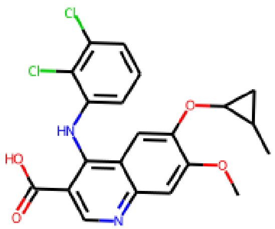	2.0	0.623362	2.525411	2.997881	4	0.774857
**649**	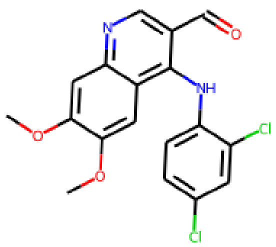	0.596217	COc1cc2ncc(C(=O)O)c(Nc3cccc(Cl)c3Cl)c2cc1OC1C(C)C1	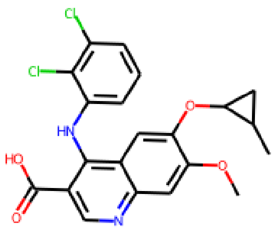	2.0	0.623362	2.525411	2.997881	4	0.774857
**650**	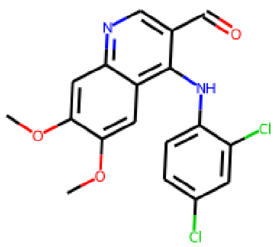	0.596217	COc1cc2ncc(C(=O)O)c(Nc3cccc(Cl)c3Cl)c2cc1OC1C(C)C1	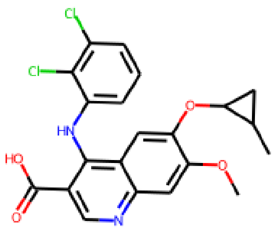	2.0	0.623362	2.525411	2.997881	4	0.774857
**651**	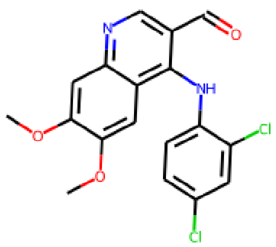	0.596217	COc1cc2ncc(C(=O)O)c(Nc3cccc(Cl)c3Cl)c2cc1OC1C(C)C1	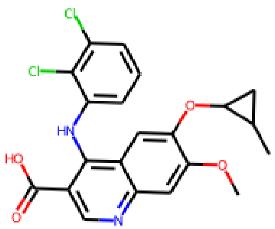	2.0	0.623362	2.525411	2.997881	4	0.774857

	src known mol	similarity	smiles	mol	Class	qed	logP	SAS	rings	kinase inhibition likeliness
**652**	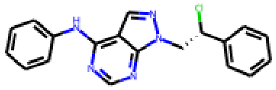	0.596036	Cc1ccc(C(=O)Nc2ccon2)cc1Nc1ncnc2c1cnn2C1cccccc1	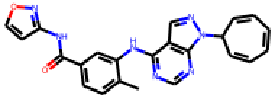	8.0	0.623362	2.525411	2.997881	5	0.770298
**653**	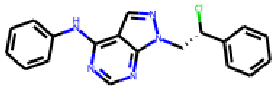	0.596036	Cc1ccc(C(=O)Nc2ccon2)cc1Nc1ncnc2c1cnn2C1cccccc1	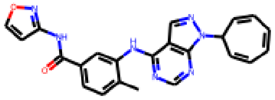	8.0	0.623362	2.525411	2.997881	5	0.770298
**654**	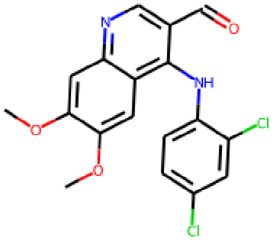	0.595813	COc1cc2ncc(C(=O)O)c(Nc3cccc(Cl)c3Cl)c2cc1OC1C(C)C1	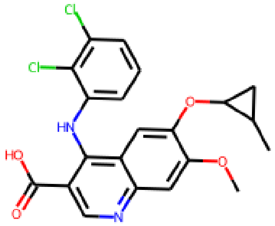	2.0	0.623362	2.525411	2.997881	4	0.774857
**655**	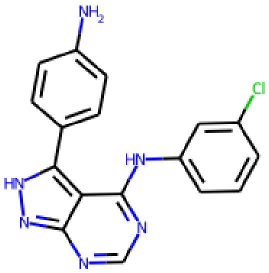	0.595458	c1ccc(Nc2ncnc3n[nH]c(-c4ccccc4)c32)cc1	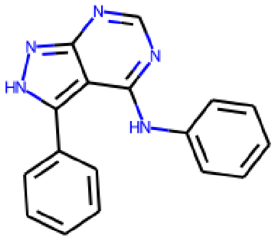	0.0	0.520676	2.336694	2.899742	4	0.831417

	src known mol	similarity	smiles	mol	Class	qed	logP	SAS	rings	kinase inhibition likeliness
**656**	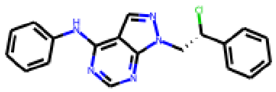	0.595404	Cc1ccc(C(=O)Nc2ccon2)cc1Nc1ncnc2c1cnn2C1cccccc1	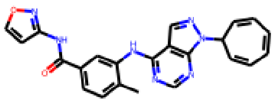	8.0	0.623362	2.525411	2.997881	5	0.770298
**657**	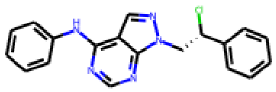	0.595404	Cc1ccc(C(=O)Nc2ccon2)cc1Nc1ncnc2c1cnn2C1cccccc1	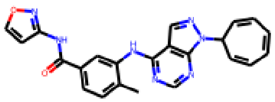	8.0	0.623362	2.525411	2.997881	5	0.770298
**658**	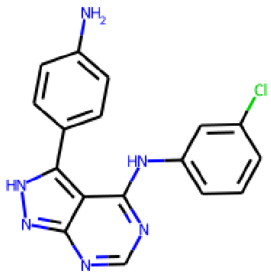	0.595278	c1ccc(Nc2ncnc3n[nH]c(-c4ccccc4)c23)cc1	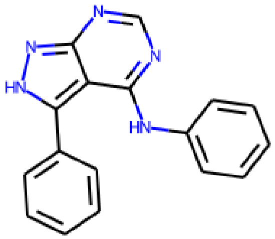	0.0	0.638163	3.995589	2.988471	4	0.800634
**659**	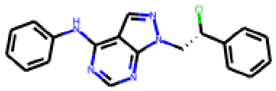	0.595238	Cc1ccc(C(=O)Nc2ccon2)cc1Nc1ncnc2c1cnn2C1cccccc1	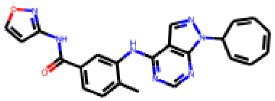	8.0	0.623362	2.525411	2.997881	5	0.770298

	src known mol	similarity	smiles	mol	Class	qed	logP	SAS	rings	kinase inhibition likeliness
**660**	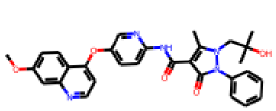	0.595188	COc1ccc2c(=O)cc(CNC(=O)c3c(C)n(C)n(-c4ccccc4)c3=O)nc3cccc2c3c1	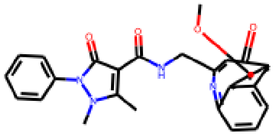	9.0	0.638163	3.995589	2.988471	5	0.774651
**661**	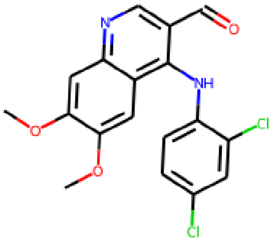	0.595102	COc1cc2ncc(C(=O)O)c(Nc3cccc(Cl)c3Cl)c2cc1OC1C(C)C1	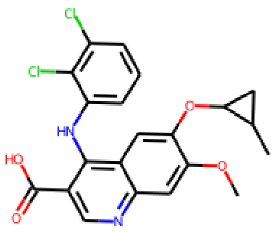	2.0	0.623362	2.525411	2.997881	4	0.774857
**662**	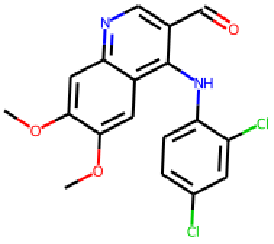	0.595102	COc1cc2ncc(C(=O)O)c(Nc3cccc(Cl)c3Cl)c2cc1OC1C(C)C1	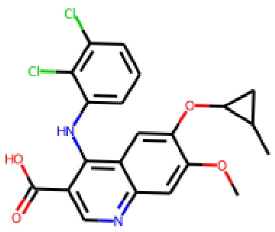	2.0	0.623362	2.525411	2.997881	4	0.774857
**663**	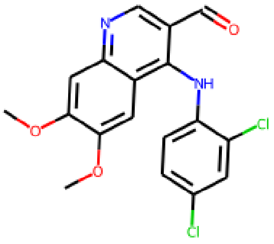	0.595102	COc1cc2ncc(C(=O)O)c(Nc3cccc(Cl)c3Cl)c2cc1OC1C(C)C1	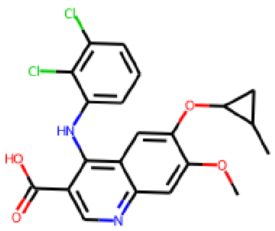	2.0	0.623362	2.525411	2.997881	4	0.774857

	src known mol	similarity	smiles	mol	Class	qed	logP	SAS	rings	kinase inhibition likeliness
**664**	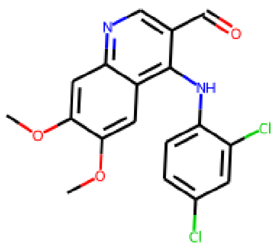	0.595102	COc1cc2ncc(C(=O)O)c(Nc3cccc(Cl)c3Cl)c2cc1OC1C(C)C1	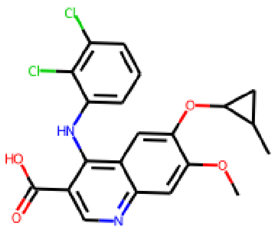	2.0	0.623362	2.525411	2.997881	4	0.774857
**665**	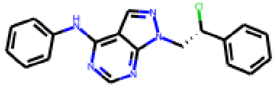	0.595075	Cc1ccc(C(=O)Nc2ccon2)cc1Nc1ncnc2c1cnn2C1cccccc1	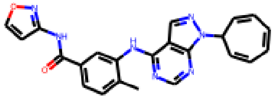	8.0	0.623362	2.525411	2.997881	5	0.770298
**666**	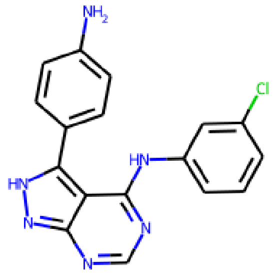	0.594777	c1ccc(Nc2ncnc3n[nH]c(-c4ccccc4)c23)cc1	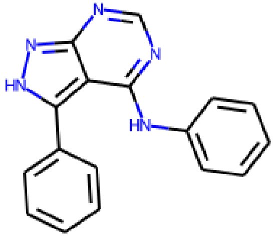	0.0	0.638163	3.995589	2.988471	4	0.800634
**667**	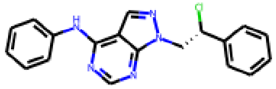	0.594310	Cc1ccc(C(=O)Nc2ccon2)cc1Nc1ncnc2c1cnn2C1cccccc1	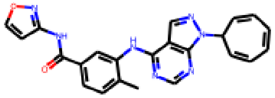	8.0	0.623362	2.525411	2.997881	5	0.770298

	src known mol	similarity	smiles	mol	Class	qed	logP	SAS	rings	kinase inhibition likeliness
**668**	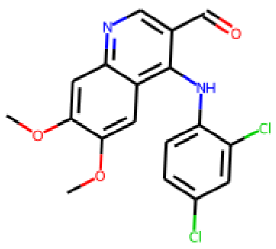	0.594306	COc1cc2ncc(C(=O)O)c(Nc3cccc(Cl)c3Cl)c2cc1OC1C(C)C1	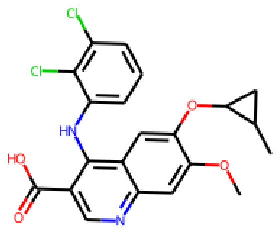	2.0	0.623362	2.525411	2.997881	4	0.774857
**669**	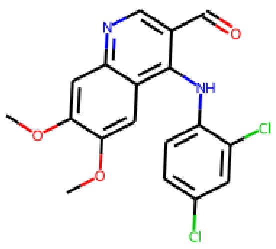	0.594262	COc1cc2ncc(C(=O)O)c(Nc3cccc(Cl)c3Cl)c2cc1OC1C(C)C1	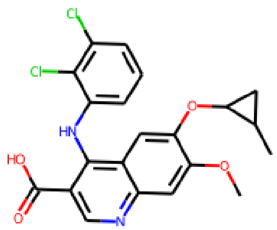	2.0	0.623362	2.525411	2.997881	4	0.774857
**670**	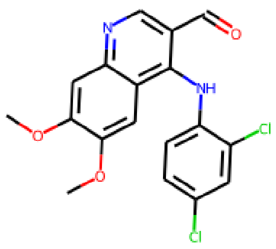	0.594262	COc1cc2ncc(C(=O)O)c(Nc3cccc(Cl)c3Cl)c2cc1OC1C(C)C1	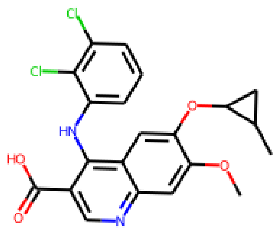	2.0	0.623362	2.525411	2.997881	4	0.774857
**671**	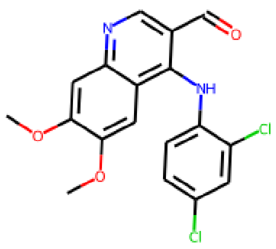	0.594262	COc1cc2ncc(C(=O)O)c(Nc3cccc(Cl)c3Cl)c2cc1OC1C(C)C1	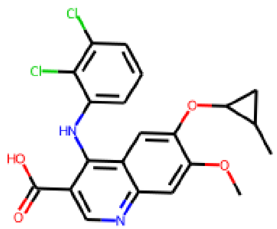	2.0	0.623362	2.525411	2.997881	4	0.774857

	src known mol	similarity	smiles	mol	Class	qed	logP	SAS	rings	kinase inhibition likeliness
**672**	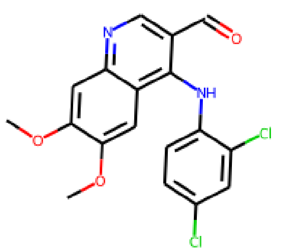	0.594262	COc1cc2ncc(C(=O)O)c(Nc3cccc(Cl)c3Cl)c2cc1OC1C(C)C1	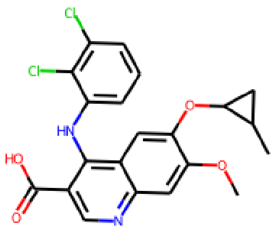	2.0	0.623362	2.525411	2.997881	4	0.774857
**673**	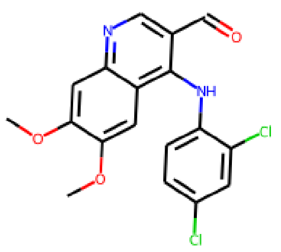	0.594262	COc1cc2ncc(C(=O)O)c(Nc3cccc(Cl)c3Cl)c2cc1OC1C(C)C1	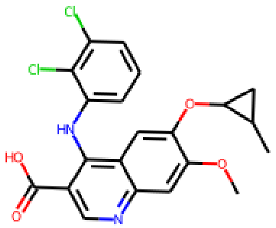	2.0	0.623362	2.525411	2.997881	4	0.774857
**674**	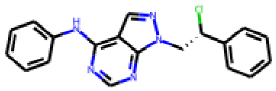	0.594257	Cc1ccc(C(=O)Nc2ccon2)cc1Nc1ncnc2c1cnn2C1cccccc1	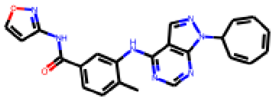	8.0	0.623362	2.525411	2.997881	5	0.770298
**675**	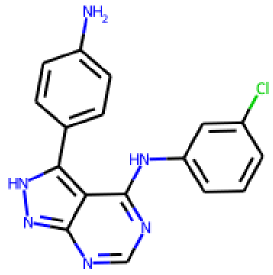	0.594142	c1ccc(Nc2ncnc3n[nH]c(-c4ccccc4)c32)cc1	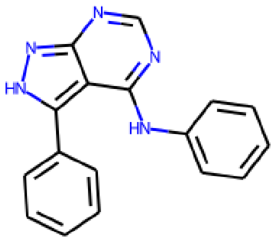	0.0	0.520676	2.336694	2.899742	4	0.831417

	src known mol	similarity	smiles	mol	Class	qed	logP	SAS	rings	kinase inhibition likeliness
**676**	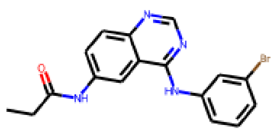	0.594139	O=C(CNC(=O)Oc1ccccc1)Nc1ccc2ncnc(Nc3cccc(Cl)c3)c2c1	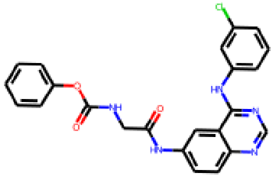	3.0	0.638163	3.995589	2.988471	4	0.793889
**677**	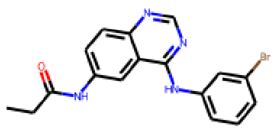	0.594139	O=C(CNC(=O)Oc1ccccc1)Nc1ccc2ncnc(Nc3cccc(Cl)c3)c2c1	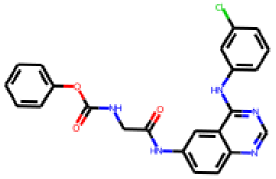	3.0	0.638163	3.995589	2.988471	4	0.793889
**678**	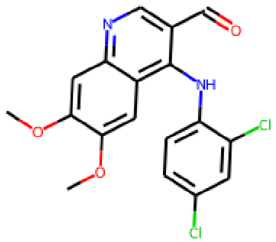	0.593991	COc1cc2ncc(C(=O)O)c(Nc3cccc(Cl)c3Cl)c2cc1OC1C(C)C1	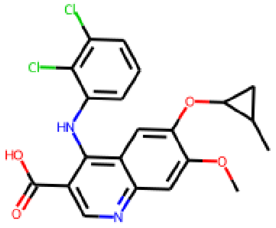	2.0	0.623362	2.525411	2.997881	4	0.774857
**679**	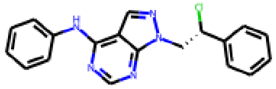	0.593944	Cc1ccc(C(=O)Nc2ccon2)cc1Nc1ncnc2c1cnn2C1cccccc1	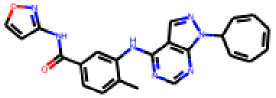	8.0	0.623362	2.525411	2.997881	5	0.770298

	src known mol	similarity	smiles	mol	Class	qed	logP	SAS	rings	kinase inhibition likeliness
**680**	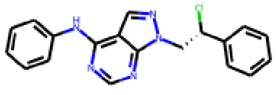	0.593944	Cc1ccc(C(=O)Nc2ccon2)cc1Nc1ncnc2c1cnn2C1cccccc1	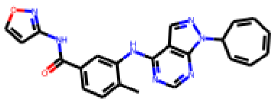	8.0	0.623362	2.525411	2.997881	5	0.770298
**681**	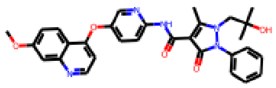	0.593734	COc1ccc2c(=O)cc(CNC(=O)c3c(C)n(C)n(-c4ccccc4)c3=O)nc3cccc2c3c1	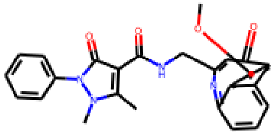	9.0	0.638163	3.995589	2.988471	5	0.774651
**682**	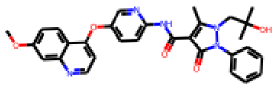	0.593734	COc1ccc2c(=O)cc(CNC(=O)c3c(C)n(C)n(-c4ccccc4)c3=O)nc3cccc2c3c1	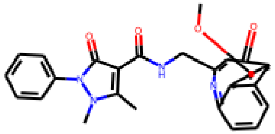	9.0	0.638163	3.995589	2.988471	5	0.774651
**683**	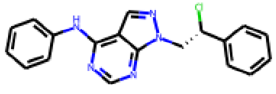	0.593579	Cc1ccc(C(=O)Nc2ccon2)cc1Nc1ncnc2c1cnn2C1cccccc1	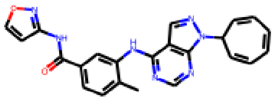	8.0	0.623362	2.525411	2.997881	5	0.770298

	src known mol	similarity	smiles	mol	Class	qed	logP	SAS	rings	kinase inhibition likeliness
**684**	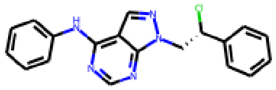	0.593579	Cc1ccc(C(=O)Nc2ccon2)cc1Nc1ncnc2c1cnn2C1cccccc1	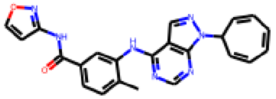	8.0	0.623362	2.525411	2.997881	5	0.770298
**685**	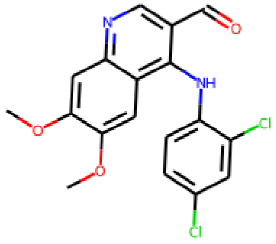	0.593522	COc1cc2ncc(C(=O)O)c(Nc3cccc(Cl)c3Cl)c2cc1OC1C(C)C1	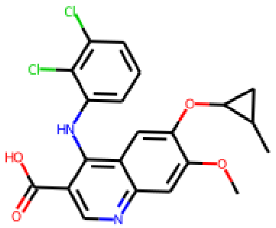	2.0	0.623362	2.525411	2.997881	4	0.774857
**686**	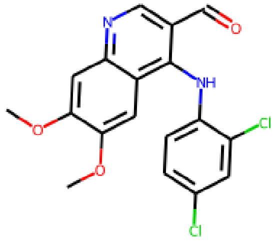	0.593522	COc1cc2ncc(C(=O)O)c(Nc3cccc(Cl)c3Cl)c2cc1OC1C(C)C1	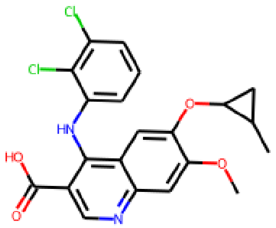	2.0	0.623362	2.525411	2.997881	4	0.774857
**687**	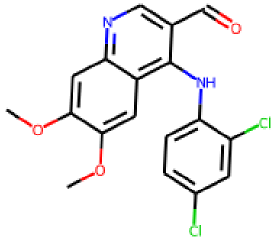	0.593522	COc1cc2ncc(C(=O)O)c(Nc3cccc(Cl)c3Cl)c2cc1OC1C(C)C1	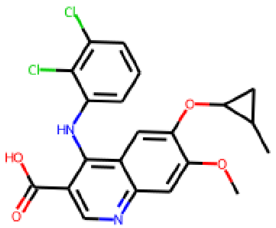	2.0	0.623362	2.525411	2.997881	4	0.774857

	src known mol	similarity	smiles	mol	Class	qed	logP	SAS	rings	kinase inhibition likeliness
**688**	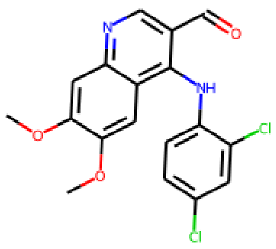	0.593522	COc1cc2ncc(C(=O)O)c(Nc3cccc(Cl)c3Cl)c2cc1OC1C(C)C1	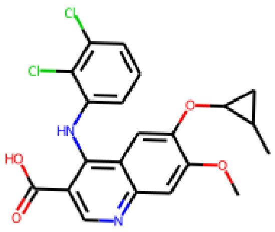	2.0	0.623362	2.525411	2.997881	4	0.774857
**689**	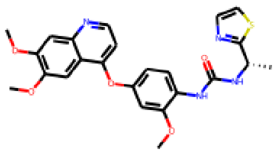	0.593453	COc1ccccc1NC(=O)Nc1ccc(Oc2ccnc3cc(OC)c(OC)cc32)cc1	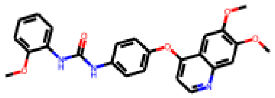	5.0	0.768132	3.608437	2.513170	4	0.790955
**690**	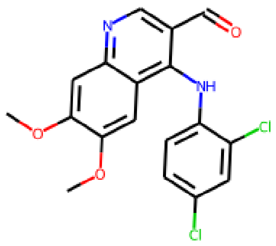	0.593424	COc1cc2ncc(C(=O)O)c(Nc3cccc(Cl)c3Cl)c2cc1OC1C(C)C1	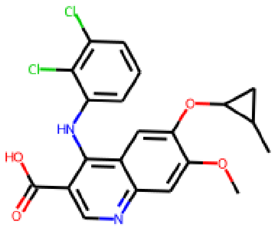	2.0	0.623362	2.525411	2.997881	4	0.774857
**691**	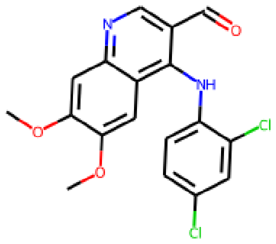	0.593424	COc1cc2ncc(C(=O)O)c(Nc3cccc(Cl)c3Cl)c2cc1OC1C(C)C1	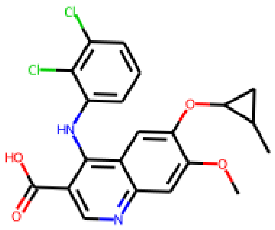	2.0	0.623362	2.525411	2.997881	4	0.774857

	src known mol	similarity	smiles	mol	Class	qed	logP	SAS	rings	kinase inhibition likeliness
**692**	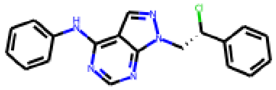	0.593399	Cc1ccc(C(=O)Nc2ccon2)cc1Nc1ncnc2c1cnn2C1cccccc1	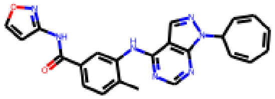	8.0	0.623362	2.525411	2.997881	5	0.770298
**693**	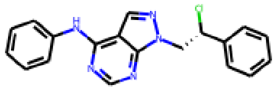	0.593399	Cc1ccc(C(=O)Nc2ccon2)cc1Nc1ncnc2c1cnn2C1cccccc1	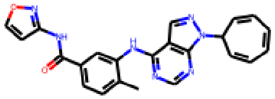	8.0	0.623362	2.525411	2.997881	5	0.770298
**694**	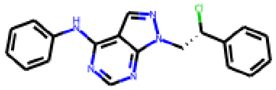	0.593399	Cc1ccc(C(=O)Nc2ccon2)cc1Nc1ncnc2c1cnn2C1cccccc1	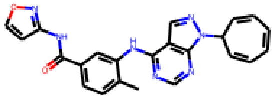	8.0	0.623362	2.525411	2.997881	5	0.770298
**695**	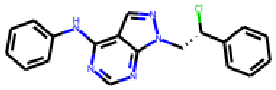	0.593399	Cc1ccc(C(=O)Nc2ccon2)cc1Nc1ncnc2c1cnn2C1cccccc1	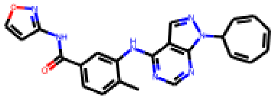	8.0	0.623362	2.525411	2.997881	5	0.770298

	src known mol	similarity	smiles	mol	Class	qed	logP	SAS	rings	kinase inhibition likeliness
**696**	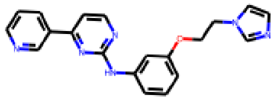	0.593301	Cc1ccc(OC(=O)c2ccccc2)cc1Nc1nccc(-c2cccnc2)n1	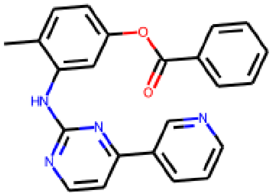	0.0	0.638163	3.995589	2.988471	4	0.775468
**697**	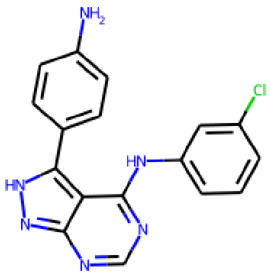	0.593033	c1ccc(Nc2ncnc3n[nH]c(-c4ccccc4)c32)cc1	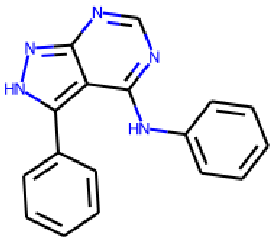	0.0	0.520676	2.336694	2.899742	4	0.831417
**698**	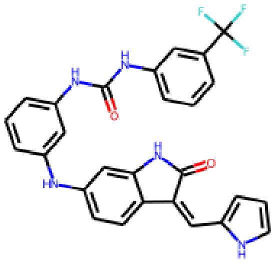	0.592965	COc1ccc(NC(=O)Nc2ccc3c(c2)NC(=O)/C3=C/c2ccc[nH]2)cc1	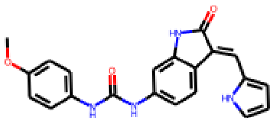	4.0	0.638163	3.995589	2.988471	4	0.768944
**699**	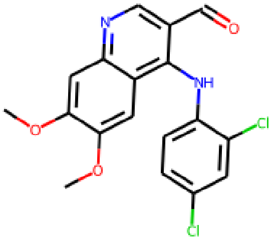	0.592872	COc1cc2ncc(C(=O)O)c(Nc3cccc(Cl)c3Cl)c2cc1OC1C(C)C1	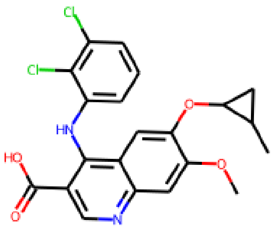	2.0	0.623362	2.525411	2.997881	4	0.774857

	src known mol	similarity	smiles	mol	Class	qed	logP	SAS	rings	kinase inhibition likeliness
**700**	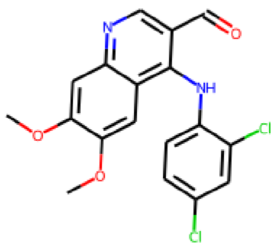	0.592872	COc1cc2ncc(C(=O)O)c(Nc3cccc(Cl)c3Cl)c2cc1OC1C(C)C1	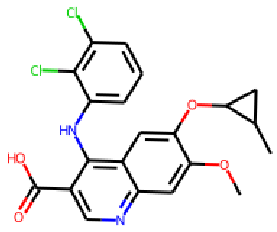	2.0	0.623362	2.525411	2.997881	4	0.774857
**701**	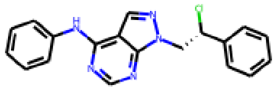	0.592852	Cc1ccc(C(=O)Nc2ccon2)cc1Nc1ncnc2c1cnn2C1cccccc1	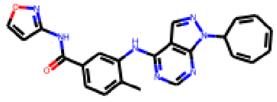	8.0	0.623362	2.525411	2.997881	5	0.770298
**702**	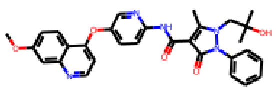	0.592651	COc1ccc2c(=O)cc(CNC(=O)c3c(C)n(C)n(-c4ccccc4)c3=O)nc3cccc2c3c1	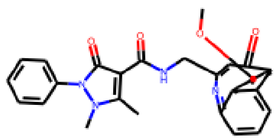	9.0	0.638163	3.995589	2.988471	5	0.774651
**703**	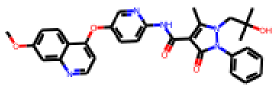	0.592651	COc1ccc2c(=O)cc(CNC(=O)c3c(C)n(C)n(-c4ccccc4)c3=O)nc3cccc2c3c1	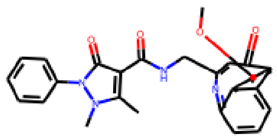	9.0	0.638163	3.995589	2.988471	5	0.774651

	src known mol	similarity	smiles	mol	Class	qed	logP	SAS	rings	kinase inhibition likeliness
**704**	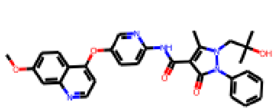	0.592514	COc1ccc2c(=O)cc(CNC(=O)c3c(C)n(C)n(-c4ccccc4)c3=O)nc3cccc2c3c1	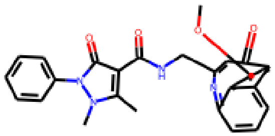	9.0	0.638163	3.995589	2.988471	5	0.774651
**705**	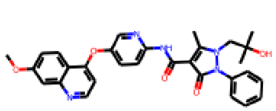	0.592514	COc1ccc2c(=O)cc(CNC(=O)c3c(C)n(C)n(-c4ccccc4)c3=O)nc3cccc2c3c1	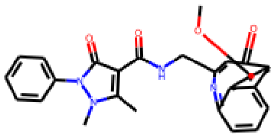	9.0	0.638163	3.995589	2.988471	5	0.774651
**706**	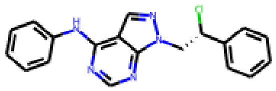	0.592440	Cc1ccc(C(=O)Nc2ccon2)cc1Nc1ncnc2c1cnn2C1cccccc1	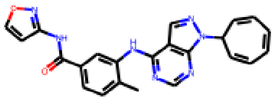	8.0	0.623362	2.525411	2.997881	5	0.770298
**707**	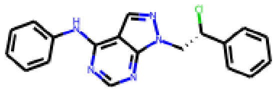	0.592440	Cc1ccc(C(=O)Nc2ccon2)cc1Nc1ncnc2c1cnn2C1cccccc1	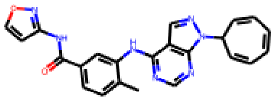	8.0	0.623362	2.525411	2.997881	5	0.770298

	src known mol	similarity	smiles	mol	Class	qed	logP	SAS	rings	kinase inhibition likeliness
**708**	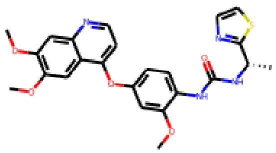	0.592437	COc1ccccc1NC(=O)Nc1ccc(Oc2ccnc3cc(OC)c(OC)cc32)cc1	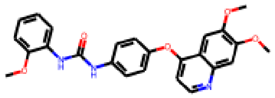	5.0	0.768132	3.608437	2.513170	4	0.790955
**709**	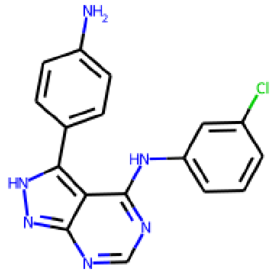	0.592437	c1ccc(Nc2ncnc3n[nH]c(-c4ccccc4)c23)cc1	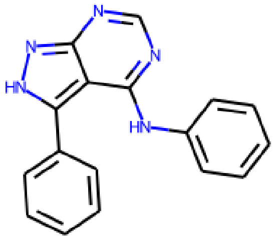	0.0	0.638163	3.995589	2.988471	4	0.800634
**710**	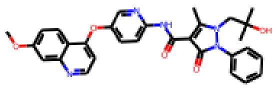	0.592400	COc1ccc2c(=O)cc(CNC(=O)c3c(C)n(C)n(-c4ccccc4)c3=O)nc3cccc2c3c1	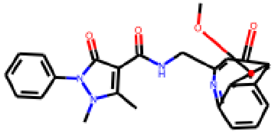	9.0	0.638163	3.995589	2.988471	5	0.774651
**711**	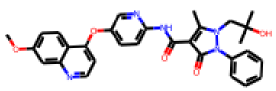	0.592400	COc1ccc2c(=O)cc(CNC(=O)c3c(C)n(C)n(-c4ccccc4)c3=O)nc3cccc2c3c1	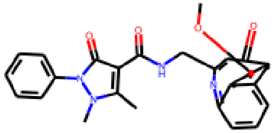	9.0	0.638163	3.995589	2.988471	5	0.774651

	src known mol	similarity	smiles	mol	Class	qed	logP	SAS	rings	kinase inhibition likeliness
**712**	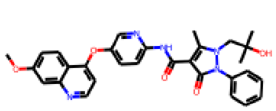	0.592397	COc1ccc2c(=O)cc(CNC(=O)c3c(C)n(C)n(-c4ccccc4)c3=O)nc3cccc2c3c1	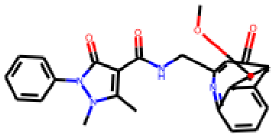	9.0	0.638163	3.995589	2.988471	5	0.774651
**713**	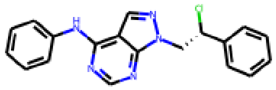	0.592361	Cc1ccc(C(=O)Nc2ccon2)cc1Nc1ncnc2c1cnn2C1cccccc1	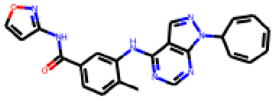	8.0	0.623362	2.525411	2.997881	5	0.770298
**714**	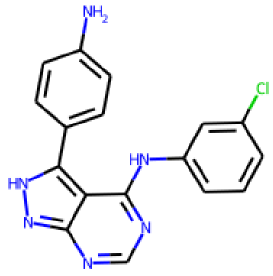	0.592308	c1ccc(Nc2ncnc3n[nH]c(-c4ccccc4)c23)cc1	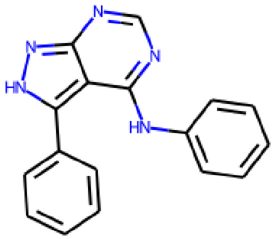	0.0	0.638163	3.995589	2.988471	4	0.800634
**715**	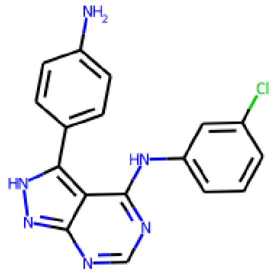	0.592249	c1ccc(Nc2ncnc3n[nH]c(-c4ccccc4)c23)cc1	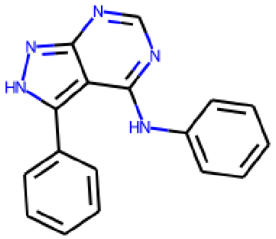	0.0	0.638163	3.995589	2.988471	4	0.800634

	src known mol	similarity	smiles	mol	Class	qed	logP	SAS	rings	kinase inhibition likeliness
**716**	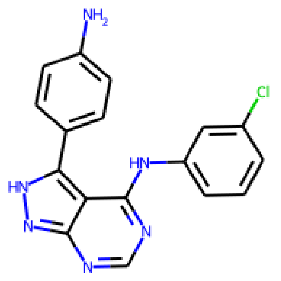	0.592249	c1ccc(Nc2ncnc3n[nH]c(-c4ccccc4)c23)cc1	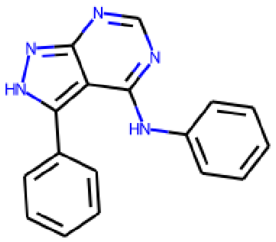	0.0	0.638163	3.995589	2.988471	4	0.800634
**717**	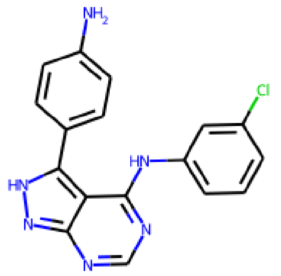	0.592160	c1ccc(Nc2ncnc3n[nH]c(-c4ccccc4)c23)cc1	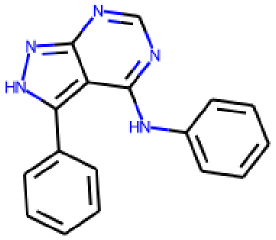	0.0	0.638163	3.995589	2.988471	4	0.800634
**718**	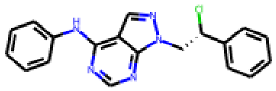	0.592022	Cc1ccc(C(=O)Nc2ccon2)cc1Nc1ncnc2c1cnn2C1cccccc1	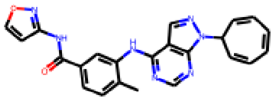	8.0	0.623362	2.525411	2.997881	5	0.770298
**719**	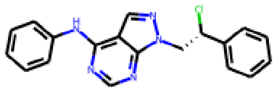	0.592022	Cc1ccc(C(=O)Nc2ccon2)cc1Nc1ncnc2c1cnn2C1cccccc1	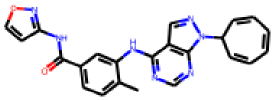	8.0	0.623362	2.525411	2.997881	5	0.770298

	src known mol	similarity	smiles	mol	Class	qed	logP	SAS	rings	kinase inhibition likeliness
**720**	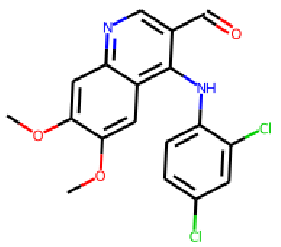	0.591949	COc1cc2ncc(C(=O)O)c(Nc3cccc(Cl)c3Cl)c2cc1OC1C(C)C1	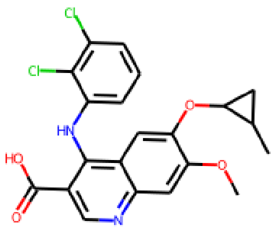	2.0	0.623362	2.525411	2.997881	4	0.774857
**721**	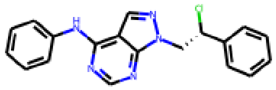	0.591925	Cc1ccc(C(=O)Nc2ccon2)cc1Nc1ncnc2c1cnn2C1cccccc1	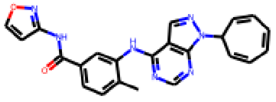	8.0	0.623362	2.525411	2.997881	5	0.770298
**722**	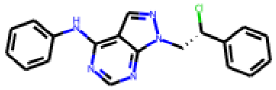	0.591925	Cc1ccc(C(=O)Nc2ccon2)cc1Nc1ncnc2c1cnn2C1cccccc1	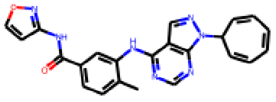	8.0	0.623362	2.525411	2.997881	5	0.770298
**723**	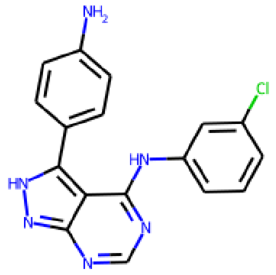	0.591751	c1ccc(Nc2ncnc3n[nH]c(-c4ccccc4)c23)cc1	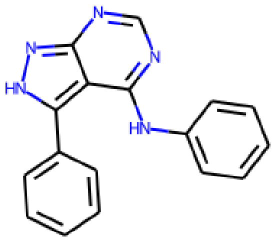	0.0	0.638163	3.995589	2.988471	4	0.800634

	src known mol	similarity	smiles	mol	Class	qed	logP	SAS	rings	kinase inhibition likeliness
**724**	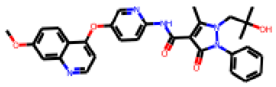	0.591483	COc1ccc2c(=O)cc(CNC(=O)c3c(C)n(C)n(-c4ccccc4)c3=O)nc3cccc2c3c1	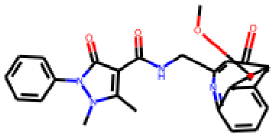	9.0	0.638163	3.995589	2.988471	5	0.774651
**725**	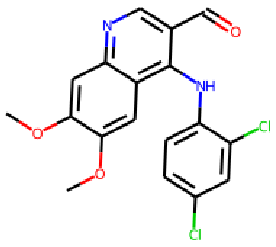	0.591381	COc1cc2ncc(C(=O)O)c(Nc3cccc(Cl)c3Cl)c2cc1OC1C(C)C1	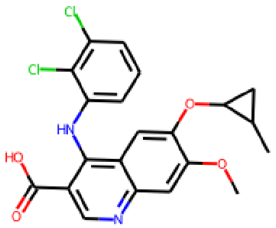	2.0	0.623362	2.525411	2.997881	4	0.774857
**726**	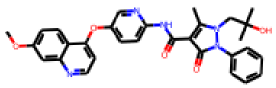	0.591169	COc1ccc2c(=O)cc(CNC(=O)c3c(C)n(C)n(-c4ccccc4)c3=O)nc3cccc2c3c1	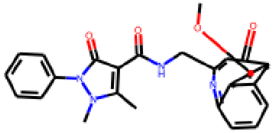	9.0	0.638163	3.995589	2.988471	5	0.774651
**727**	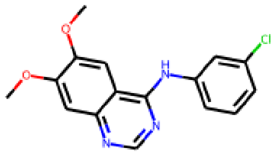	0.591106	Clc1cccc(Nc2ncnc3cc4c[nH]nc4cc23)c1	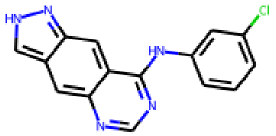	3.0	0.638163	3.995589	2.988471	4	0.781333

	src known mol	similarity	smiles	mol	Class	qed	logP	SAS	rings	kinase inhibition likeliness
**728**	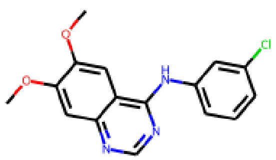	0.591106	Clc1cccc(Nc2ncnc3cc4c[nH]nc4cc23)c1	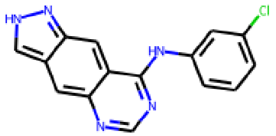	3.0	0.638163	3.995589	2.988471	4	0.781333
**729**	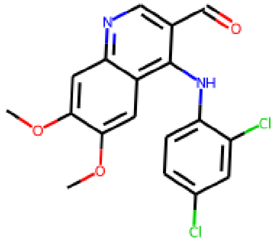	0.591090	COc1cc2ncc(C(=O)O)c(Nc3cccc(Cl)c3Cl)c2cc1OC1C(C)C1	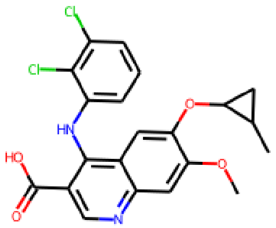	2.0	0.623362	2.525411	2.997881	4	0.774857
**730**	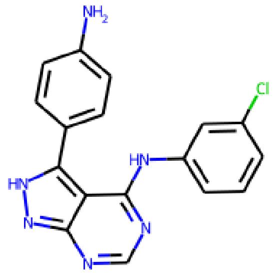	0.590985	c1ccc(Nc2ncnc3n[nH]c(-c4ccccc4)c23)cc1	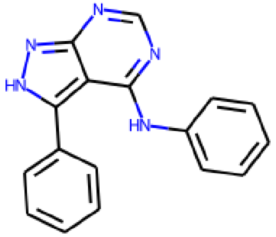	0.0	0.638163	3.995589	2.988471	4	0.800634
**731**	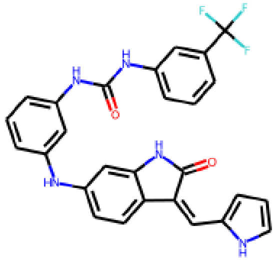	0.590877	COc1ccc(NC(=O)Nc2ccc3c(c2)NC(=O)/C3=C/c2ccc[nH]2)cc1	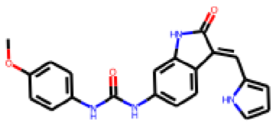	4.0	0.638163	3.995589	2.988471	4	0.768944

	src known mol	similarity	smiles	mol	Class	qed	logP	SAS	rings	kinase inhibition likeliness
**732**	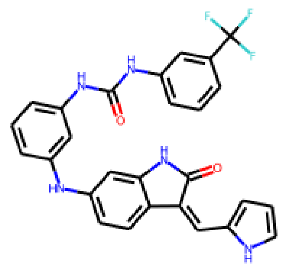	0.590877	COc1ccc(NC(=O)Nc2ccc3c(c2)NC(=O)/C3=C/c2ccc[nH]2)cc1	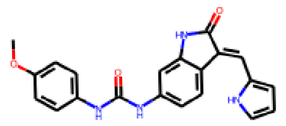	4.0	0.638163	3.995589	2.988471	4	0.768944
**733**	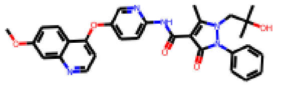	0.590431	COc1ccc2c(=O)cc(CNC(=O)c3c(C)n(C)n(-c4ccccc4)c3=O)nc3cccc2c3c1	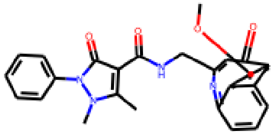	9.0	0.638163	3.995589	2.988471	5	0.774651
**734**	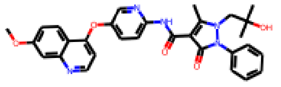	0.590431	COc1ccc2c(=O)cc(CNC(=O)c3c(C)n(C)n(-c4ccccc4)c3=O)nc3cccc2c3c1	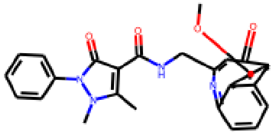	9.0	0.638163	3.995589	2.988471	5	0.774651
**735**	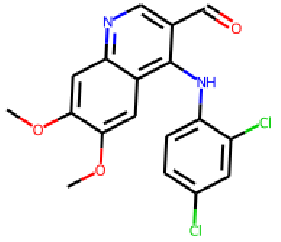	0.590323	COc1cc2ncc(C(=O)O)c(Nc3cccc(Cl)c3Cl)c2cc1OC1C(C)C1	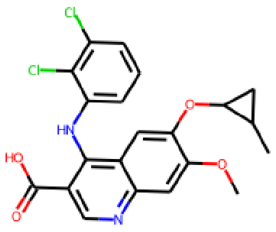	2.0	0.623362	2.525411	2.997881	4	0.774857

	src known mol	similarity	smiles	mol	Class	qed	logP	SAS	rings	kinase inhibition likeliness
**736**	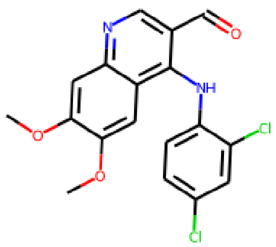	0.590323	COc1cc2ncc(C(=O)O)c(Nc3cccc(Cl)c3Cl)c2cc1OC1C(C)C1	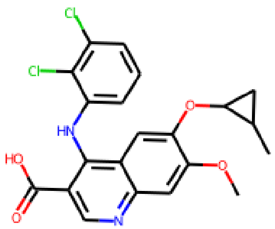	2.0	0.623362	2.525411	2.997881	4	0.774857
**737**	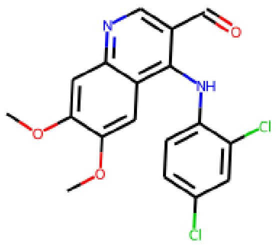	0.590287	COc1cc2ncc(C(=O)O)c(Nc3cccc(Cl)c3Cl)c2cc1OC1C(C)C1	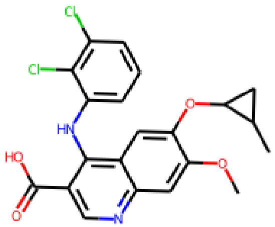	2.0	0.623362	2.525411	2.997881	4	0.774857
**738**	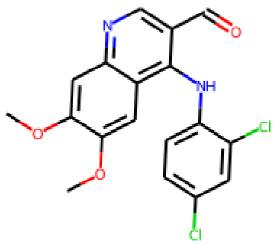	0.590287	COc1cc2ncc(C(=O)O)c(Nc3cccc(Cl)c3Cl)c2cc1OC1C(C)C1	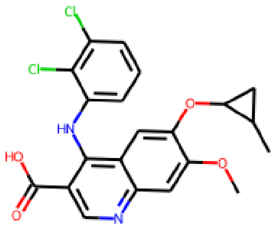	2.0	0.623362	2.525411	2.997881	4	0.774857
**739**	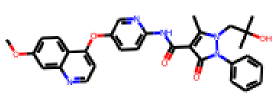	0.590069	COc1ccc2c(=O)cc(CNC(=O)c3c(C)n(C)n(-c4ccccc4)c3=O)nc3cccc2c3c1	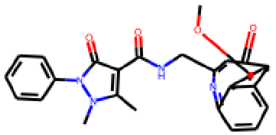	9.0	0.638163	3.995589	2.988471	5	0.774651

	src known mol	similarity	smiles	mol	Class	qed	logP	SAS	rings	kinase inhibition likeliness
**740**	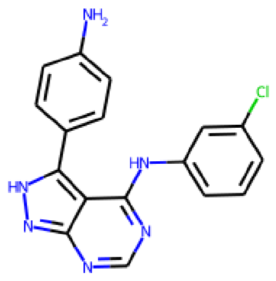	0.590041	c1ccc(Nc2ncnc3n[nH]c(-c4ccccc4)c23)cc1	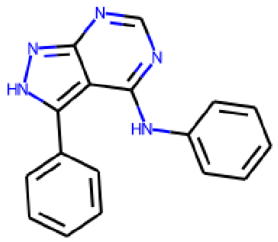	0.0	0.638163	3.995589	2.988471	4	0.800634
**741**	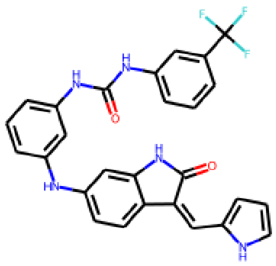	0.589908	COc1ccc(NC(=O)Nc2ccc3c(c2)NC(=O)/C3=C/c2ccc[nH]2)cc1	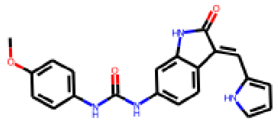	4.0	0.638163	3.995589	2.988471	4	0.768944
**742**	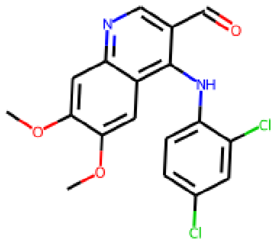	0.589850	COc1cc2ncc(C(=O)O)c(Nc3cccc(Cl)c3Cl)c2cc1OC1C(C)C1	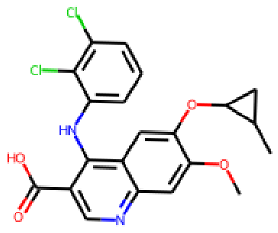	2.0	0.623362	2.525411	2.997881	4	0.774857
**743**	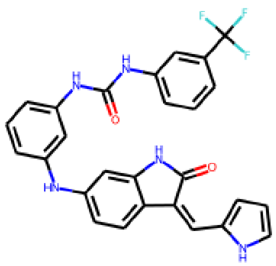	0.589780	COc1ccc(NC(=O)Nc2ccc3c(c2)NC(=O)/C3=C/c2ccc[nH]2)cc1	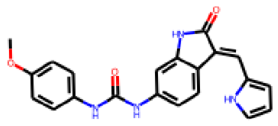	4.0	0.638163	3.995589	2.988471	4	0.768944

	src known mol	similarity	smiles	mol	Class	qed	logP	SAS	rings	kinase inhibition likeliness
**744**	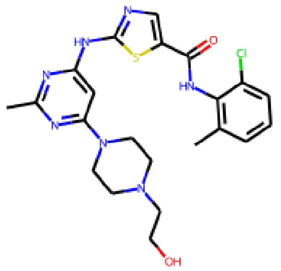	0.589764	Cc1nc(Nc2ncc(C(=O)Nc3c(C)cccc3Cl)s2)cc(N2CCC(CO)C2)n1	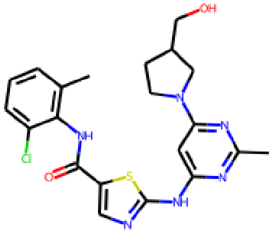	6.0	0.768132	3.608437	2.513170	4	0.818682
**745**	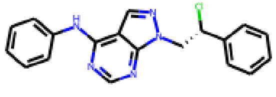	0.589708	Cc1ccc(C(=O)Nc2ccon2)cc1Nc1ncnc2c1cnn2C1cccccc1	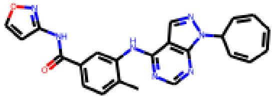	8.0	0.623362	2.525411	2.997881	5	0.770298
**746**	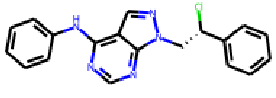	0.589708	Cc1ccc(C(=O)Nc2ccon2)cc1Nc1ncnc2c1cnn2C1cccccc1	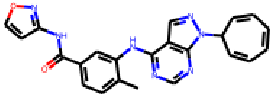	8.0	0.623362	2.525411	2.997881	5	0.770298
**747**	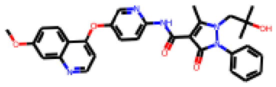	0.589595	COc1ccc2c(=O)cc(CNC(=O)c3c(C)n(C)n(-c4ccccc4)c3=O)nc3cccc2c3c1	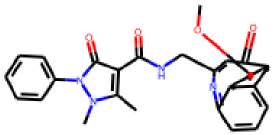	9.0	0.638163	3.995589	2.988471	5	0.774651

	src known mol	similarity	smiles	mol	Class	qed	logP	SAS	rings	kinase inhibition likeliness
**748**	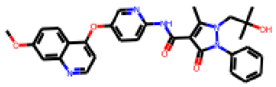	0.589595	COc1ccc2c(=O)cc(CNC(=O)c3c(C)n(C)n(-c4ccccc4)c3=O)nc3cccc2c3c1	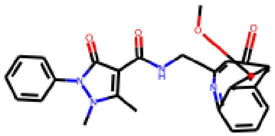	9.0	0.638163	3.995589	2.988471	5	0.774651
**749**	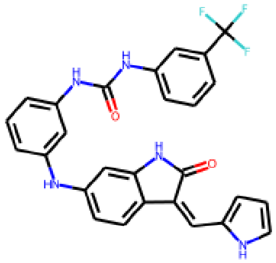	0.589489	COc1ccc(NC(=O)Nc2ccc3c(c2)NC(=O)/C3=C/c2ccc[nH]2)cc1	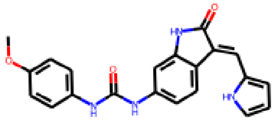	4.0	0.638163	3.995589	2.988471	4	0.768944
**750**	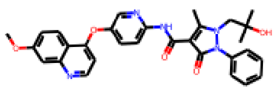	0.589164	COc1ccc2c(=O)cc(CNC(=O)c3c(C)n(C)n(-c4ccccc4)c3=O)nc3cccc2c3c1	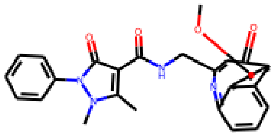	9.0	0.638163	3.995589	2.988471	5	0.774651
**751**	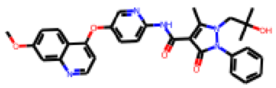	0.588883	COc1ccc2c(=O)cc(CNC(=O)c3c(C)n(C)n(-c4ccccc4)c3=O)nc3cccc2c3c1	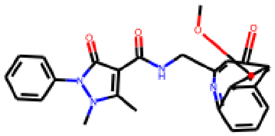	9.0	0.638163	3.995589	2.988471	5	0.774651

	src known mol	similarity	smiles	mol	Class	qed	logP	SAS	rings	kinase inhibition likeliness
**752**	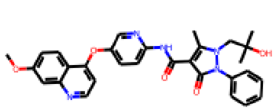	0.588854	COc1ccc2c(=O)cc(CNC(=O)c3c(C)n(C)n(-c4ccccc4)c3=O)nc3cccc2c3c1	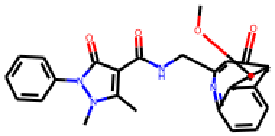	9.0	0.638163	3.995589	2.988471	5	0.774651
**753**	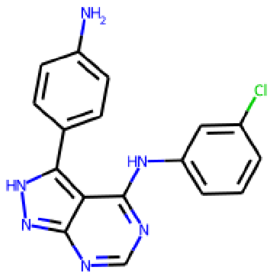	0.588824	c1ccc(Nc2ncnc3n[nH]c(-c4ccccc4)c23)cc1	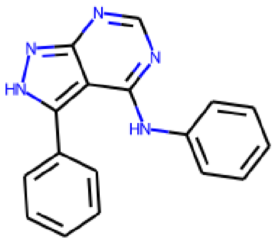	0.0	0.638163	3.995589	2.988471	4	0.800634
**754**	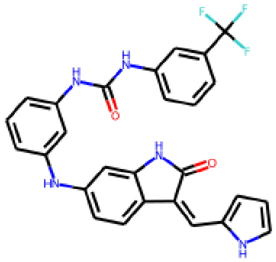	0.588741	COc1ccc(NC(=O)Nc2ccc3c(c2)NC(=O)/C3=C/c2ccc[nH]2)cc1	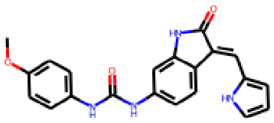	4.0	0.638163	3.995589	2.988471	4	0.768944
**755**	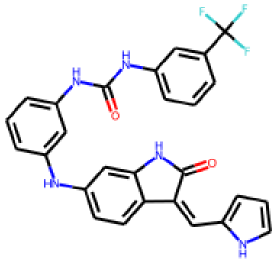	0.588527	COc1ccc(NC(=O)Nc2ccc3c(c2)NC(=O)/C3=C/c2ccc[nH]2)cc1	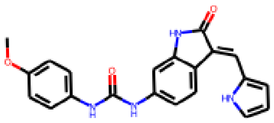	4.0	0.638163	3.995589	2.988471	4	0.768944

	src known mol	similarity	smiles	mol	Class	qed	logP	SAS	rings	kinase inhibition likeliness
**756**	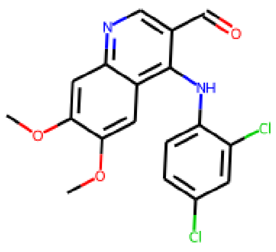	0.588513	COc1cc2ncc(C(=O)O)c(Nc3cccc(Cl)c3Cl)c2cc1OC1C(C)C1	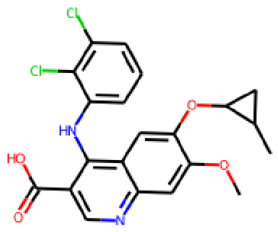	2.0	0.623362	2.525411	2.997881	4	0.774857
**757**	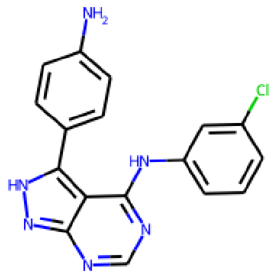	0.588481	c1ccc(Nc2ncnc3n[nH]c(-c4ccccc4)c23)cc1	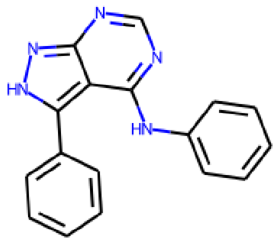	0.0	0.638163	3.995589	2.988471	4	0.800634
**758**	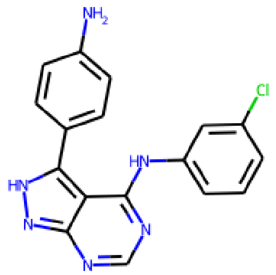	0.588430	c1ccc(Nc2ncnc3n[nH]c(-c4ccccc4)c23)cc1	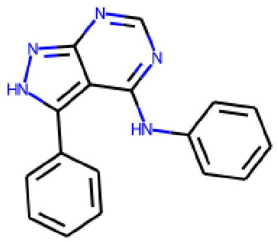	0.0	0.638163	3.995589	2.988471	4	0.800634
**759**	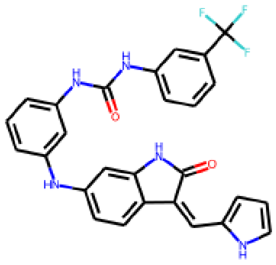	0.588402	COc1ccc(NC(=O)Nc2ccc3c(c2)NC(=O)/C3=C/c2ccc[nH]2)cc1	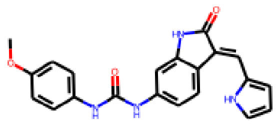	4.0	0.638163	3.995589	2.988471	4	0.768944

	src known mol	similarity	smiles	mol	Class	qed	logP	SAS	rings	kinase inhibition likeliness
**760**	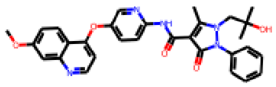	0.588142	COc1ccc2c(=O)cc(CNC(=O)c3c(C)n(C)n(-c4ccccc4)c3=O)nc3cccc2c3c1	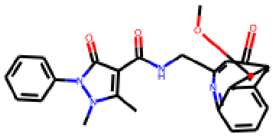	9.0	0.638163	3.995589	2.988471	5	0.774651
**761**	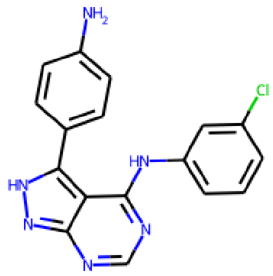	0.588040	c1ccc(Nc2ncnc3n[nH]c(-c4ccccc4)c23)cc1	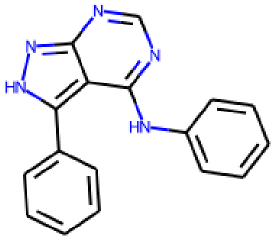	0.0	0.638163	3.995589	2.988471	4	0.800634
**762**	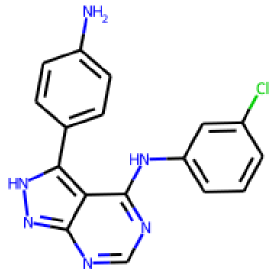	0.587944	c1ccc(Nc2ncnc3n[nH]c(-c4ccccc4)c23)cc1	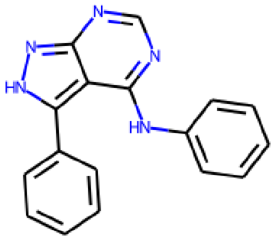	0.0	0.638163	3.995589	2.988471	4	0.800634
**763**	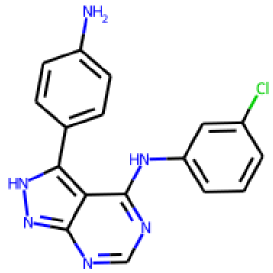	0.587894	c1ccc(Nc2ncnc3n[nH]c(-c4ccccc4)c23)cc1	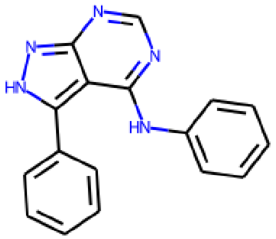	0.0	0.638163	3.995589	2.988471	4	0.800634

	src known mol	similarity	smiles	mol	Class	qed	logP	SAS	rings	kinase inhibition likeliness
**764**	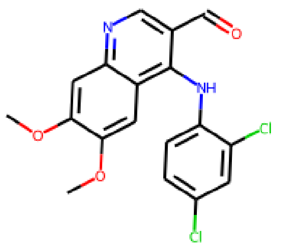	0.587535	COc1cc2ncc(C(=O)O)c(Nc3cccc(Cl)c3Cl)c2cc1OC1C(C)C1	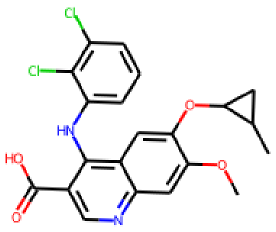	2.0	0.623362	2.525411	2.997881	4	0.774857
**765**	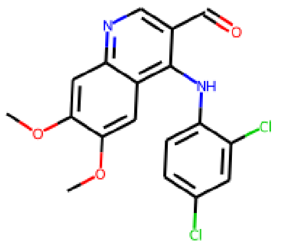	0.587535	COc1cc2ncc(C(=O)O)c(Nc3cccc(Cl)c3Cl)c2cc1OC1C(C)C1	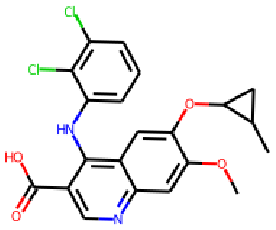	2.0	0.623362	2.525411	2.997881	4	0.774857
**766**	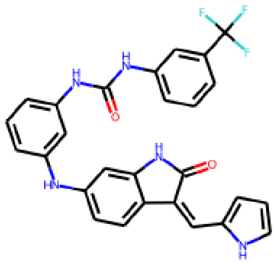	0.587500	COc1ccc(NC(=O)Nc2ccc3c(c2)NC(=O)/C3=C/c2ccc[nH]2)cc1	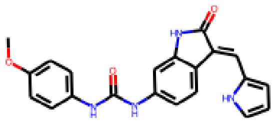	4.0	0.638163	3.995589	2.988471	4	0.768944
**767**	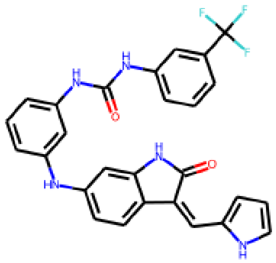	0.587500	COc1ccc(NC(=O)Nc2ccc3c(c2)NC(=O)/C3=C/c2ccc[nH]2)cc1	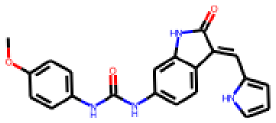	4.0	0.638163	3.995589	2.988471	4	0.768944

	src known mol	similarity	smiles	mol	Class	qed	logP	SAS	rings	kinase inhibition likeliness
**768**	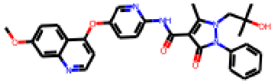	0.587427	COc1ccc2c(=O)cc(CNC(=O)c3c(C)n(C)n(-c4ccccc4)c3=O)nc3cccc2c3c1	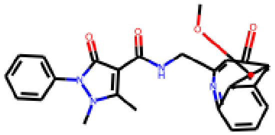	9.0	0.638163	3.995589	2.988471	5	0.774651
**769**	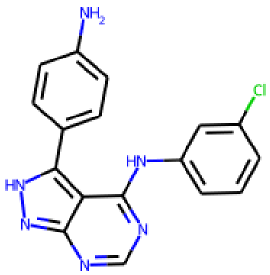	0.587407	c1ccc(Nc2ncnc3n[nH]c(-c4ccccc4)c23)cc1	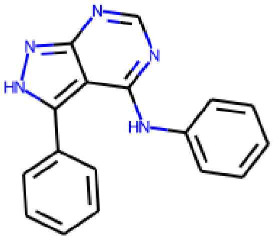	0.0	0.638163	3.995589	2.988471	4	0.800634
**770**	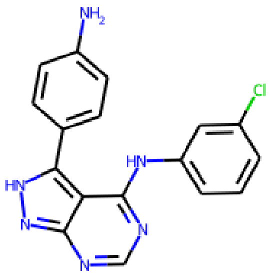	0.587354	c1ccc(Nc2ncnc3n[nH]c(-c4ccccc4)c23)cc1	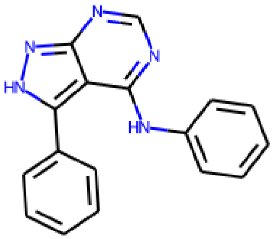	0.0	0.638163	3.995589	2.988471	4	0.800634
**771**	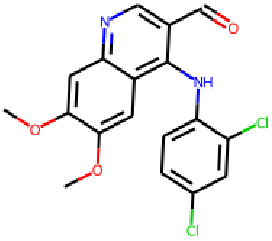	0.587338	COc1cc2ncc(C(=O)O)c(Nc3cccc(Cl)c3Cl)c2cc1OC1C(C)C1	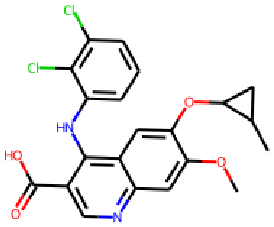	2.0	0.623362	2.525411	2.997881	4	0.774857

	src known mol	similarity	smiles	mol	Class	qed	logP	SAS	rings	kinase inhibition likeliness
**772**	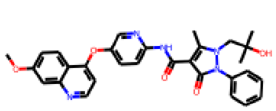	0.587175	COc1ccc2c(=O)cc(CNC(=O)c3c(C)n(C)n(-c4ccccc4)c3=O)nc3cccc2c3c1	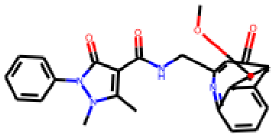	9.0	0.638163	3.995589	2.988471	5	0.774651
**773**	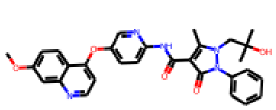	0.587175	COc1ccc2c(=O)cc(CNC(=O)c3c(C)n(C)n(-c4ccccc4)c3=O)nc3cccc2c3c1	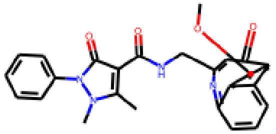	9.0	0.638163	3.995589	2.988471	5	0.774651
**774**	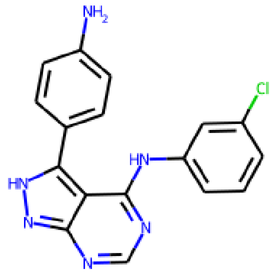	0.587171	c1ccc(Nc2ncnc3n[nH]c(-c4ccccc4)c23)cc1	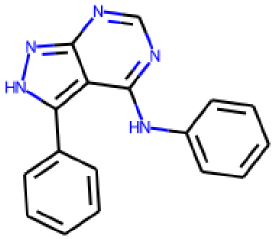	0.0	0.638163	3.995589	2.988471	4	0.800634
**775**	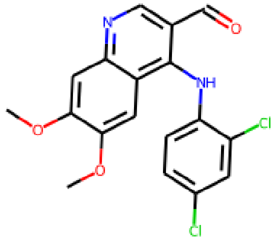	0.587072	COc1cc2ncc(C(=O)O)c(Nc3cccc(Cl)c3Cl)c2cc1OC1C(C)C1	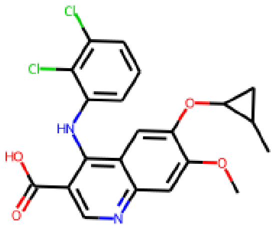	2.0	0.623362	2.525411	2.997881	4	0.774857

	src known mol	similarity	smiles	mol	Class	qed	logP	SAS	rings	kinase inhibition likeliness
**776**	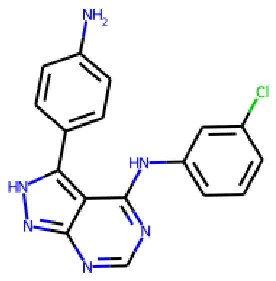	0.587065	c1ccc(Nc2ncnc3n[nH]c(-c4ccccc4)c23)cc1	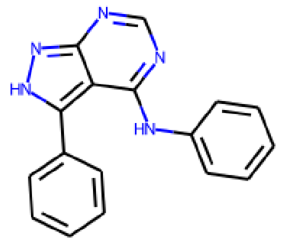	0.0	0.638163	3.995589	2.988471	4	0.800634
**777**	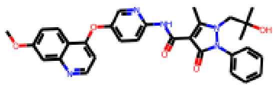	0.587025	COc1ccc2c(=O)cc(CNC(=O)c3c(C)n(C)n(-c4ccccc4)c3=O)nc3cccc2c3c1	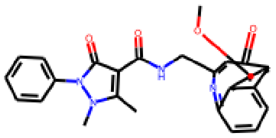	9.0	0.638163	3.995589	2.988471	5	0.774651
**778**	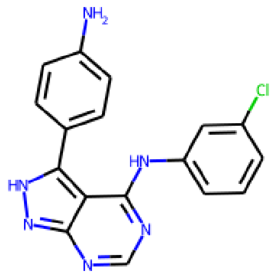	0.586974	c1ccc(Nc2ncnc3n[nH]c(-c4ccccc4)c23)cc1	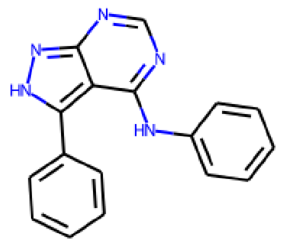	0.0	0.638163	3.995589	2.988471	4	0.800634
**779**	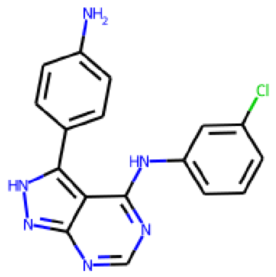	0.586974	c1ccc(Nc2ncnc3n[nH]c(-c4ccccc4)c23)cc1	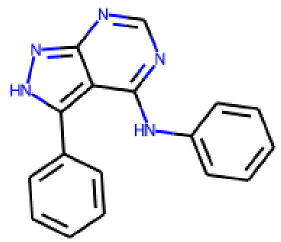	0.0	0.638163	3.995589	2.988471	4	0.800634

	src known mol	similarity	smiles	mol	Class	qed	logP	SAS	rings	kinase inhibition likeliness
**780**	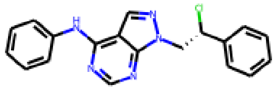	0.586970	Cc1ccc(C(=O)Nc2ccon2)cc1Nc1ncnc2c1cnn2C1cccccc1	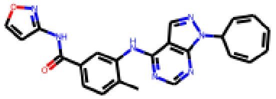	8.0	0.623362	2.525411	2.997881	5	0.770298
**781**	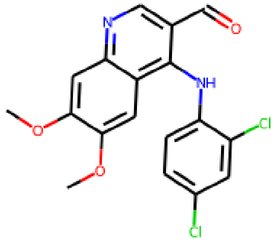	0.586890	COc1cc2ncc(C(=O)O)c(Nc3cccc(Cl)c3Cl)c2cc1OC1C(C)C1	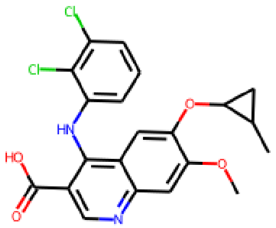	2.0	0.623362	2.525411	2.997881	4	0.774857
**782**	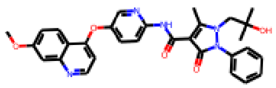	0.586786	COc1ccc2c(=O)cc(CNC(=O)c3c(C)n(C)n(-c4ccccc4)c3=O)nc3cccc2c3c1	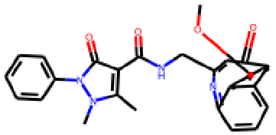	9.0	0.638163	3.995589	2.988471	5	0.774651
**783**	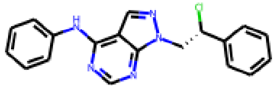	0.586735	Cc1ccc(C(=O)Nc2ccon2)cc1Nc1ncnc2c1cnn2C1cccccc1	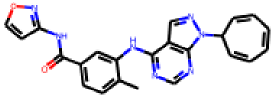	8.0	0.623362	2.525411	2.997881	5	0.770298

	src known mol	similarity	smiles	mol	Class	qed	logP	SAS	rings	kinase inhibition likeliness
**784**	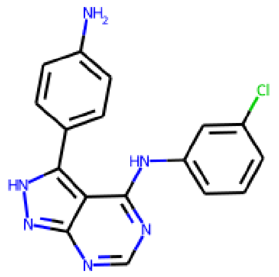	0.586689	c1ccc(Nc2ncnc3n[nH]c(-c4ccccc4)c32)cc1	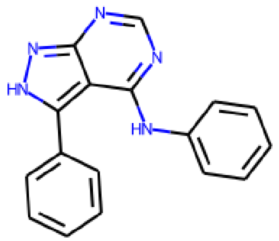	0.0	0.520676	2.336694	2.899742	4	0.831417
**785**	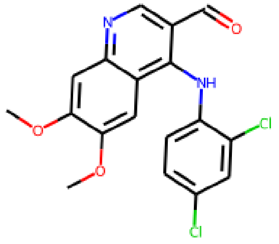	0.586667	COc1cc2ncc(C(=O)O)c(Nc3cccc(Cl)c3Cl)c2cc1OC1C(C)C1	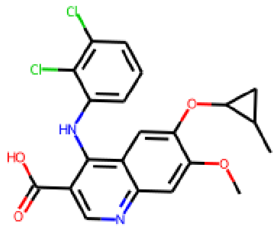	2.0	0.623362	2.525411	2.997881	4	0.774857
**786**	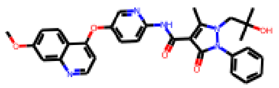	0.586554	COc1ccc2c(=O)cc(CNC(=O)c3c(C)n(C)n(-c4ccccc4)c3=O)nc3cccc2c3c1	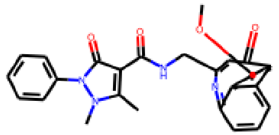	9.0	0.638163	3.995589	2.988471	5	0.774651
**787**	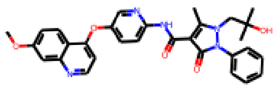	0.586554	COc1ccc2c(=O)cc(CNC(=O)c3c(C)n(C)n(-c4ccccc4)c3=O)nc3cccc2c3c1	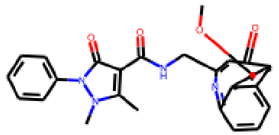	9.0	0.638163	3.995589	2.988471	5	0.774651

	src known mol	similarity	smiles	mol	Class	qed	logP	SAS	rings	kinase inhibition likeliness
**788**	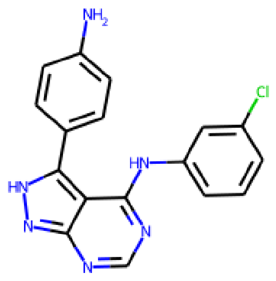	0.586405	c1ccc(Nc2ncnc3n[nH]c(-c4ccccc4)c23)cc1	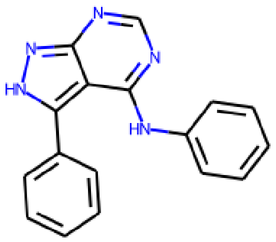	0.0	0.638163	3.995589	2.988471	4	0.800634
**789**	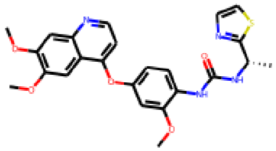	0.586271	COc1ccccc1NC(=O)Nc1ccc(Oc2ccnc3cc(OC)c(OC)cc23)cc1	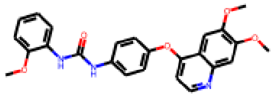	5.0	0.638163	3.995589	2.988471	4	0.789556
**790**	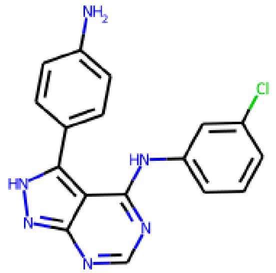	0.586093	c1ccc(Nc2ncnc3n[nH]c(-c4ccccc4)c23)cc1	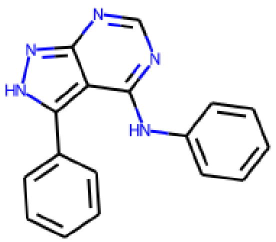	0.0	0.638163	3.995589	2.988471	4	0.800634
**791**	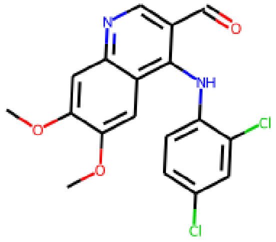	0.586072	COc1cc2ncc(C(=O)O)c(Nc3cccc(Cl)c3Cl)c2cc1OC1C(C)C1	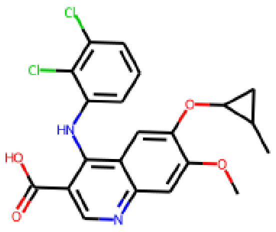	2.0	0.623362	2.525411	2.997881	4	0.774857

	src known mol	similarity	smiles	mol	Class	qed	logP	SAS	rings	kinase inhibition likeliness
**792**	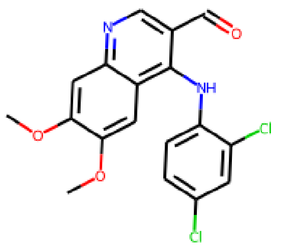	0.585974	COc1cc2ncc(C(=O)O)c(Nc3cccc(Cl)c3Cl)c2cc1OC1C(C)C1	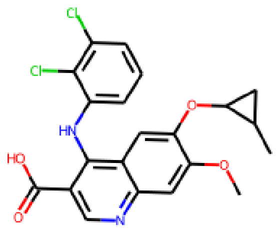	2.0	0.623362	2.525411	2.997881	4	0.774857
**793**	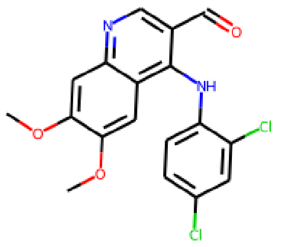	0.585974	COc1cc2ncc(C(=O)O)c(Nc3cccc(Cl)c3Cl)c2cc1OC1C(C)C1	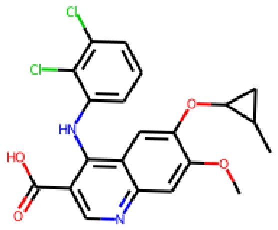	2.0	0.623362	2.525411	2.997881	4	0.774857
**794**	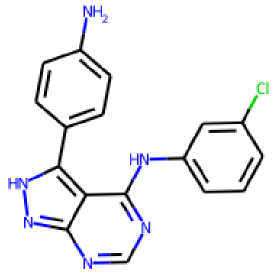	0.585892	c1ccc(Nc2ncnc3n[nH]c(-c4ccccc4)c23)cc1	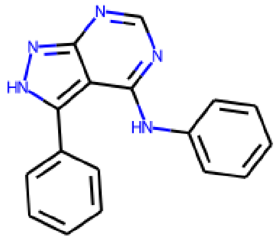	0.0	0.638163	3.995589	2.988471	4	0.800634
**795**	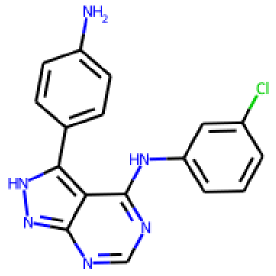	0.585726	c1ccc(Nc2ncnc3n[nH]c(-c4ccccc4)c23)cc1	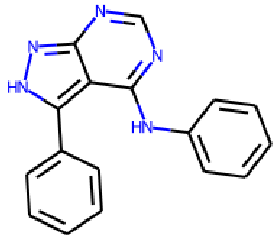	0.0	0.638163	3.995589	2.988471	4	0.800634

	src known mol	similarity	smiles	mol	Class	qed	logP	SAS	rings	kinase inhibition likeliness
**796**	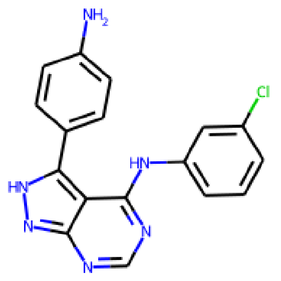	0.585631	c1ccc(Nc2ncnc3n[nH]c(-c4ccccc4)c23)cc1	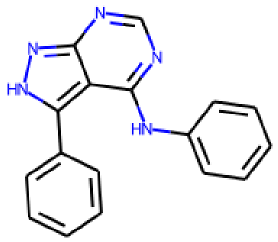	0.0	0.638163	3.995589	2.988471	4	0.800634
**797**	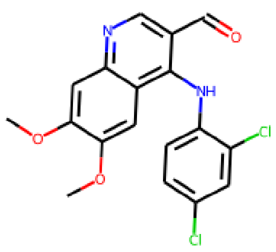	0.585292	COc1cc2ncc(C(=O)O)c(Nc3cccc(Cl)c3Cl)c2cc1OC1C(C)C1	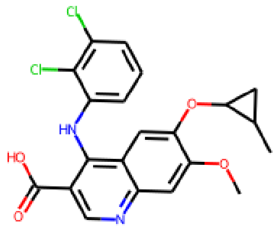	2.0	0.623362	2.525411	2.997881	4	0.774857
**798**	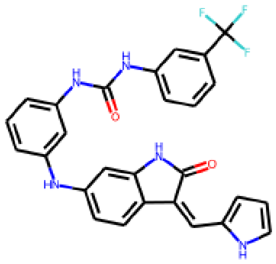	0.584986	COc1ccc(NC(=O)Nc2ccc3c(c2)NC(=O)/C3=C/c2ccc[nH]2)cc1	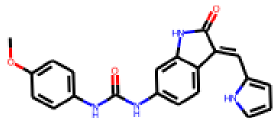	4.0	0.638163	3.995589	2.988471	4	0.768944
**799**	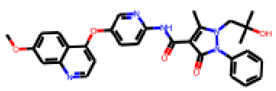	0.584807	COc1ccc2c(=O)cc(CNC(=O)c3c(C)n(C)n(-c4ccccc4)c3=O)nc3cccc2c3c1	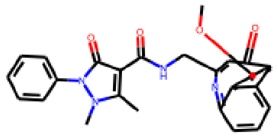	9.0	0.638163	3.995589	2.988471	5	0.774651

	src known mol	similarity	smiles	mol	Class	qed	logP	SAS	rings	kinase inhibition likeliness
**800**	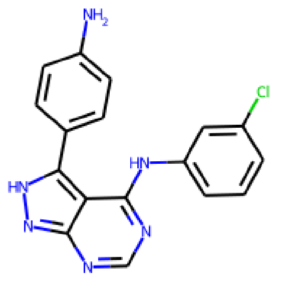	0.584781	c1ccc(Nc2ncnc3n[nH]c(-c4ccccc4)c23)cc1	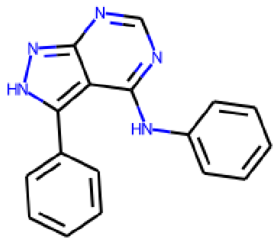	0.0	0.638163	3.995589	2.988471	4	0.800634
**801**	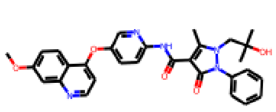	0.584737	COc1ccc2c(=O)cc(CNC(=O)c3c(C)n(C)n(-c4ccccc4)c3=O)nc3cccc2c3c1	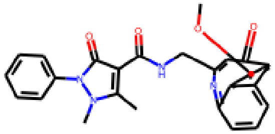	9.0	0.638163	3.995589	2.988471	5	0.774651
**802**	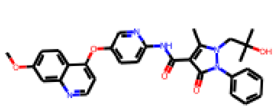	0.584737	COc1ccc2c(=O)cc(CNC(=O)c3c(C)n(C)n(-c4ccccc4)c3=O)nc3cccc2c3c1	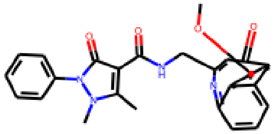	9.0	0.638163	3.995589	2.988471	5	0.774651
**803**	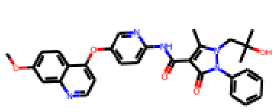	0.584737	COc1ccc2c(=O)cc(CNC(=O)c3c(C)n(C)n(-c4ccccc4)c3=O)nc3cccc2c3c1	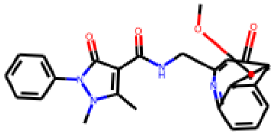	9.0	0.638163	3.995589	2.988471	5	0.774651

	src known mol	similarity	smiles	mol	Class	qed	logP	SAS	rings	kinase inhibition likeliness
**804**	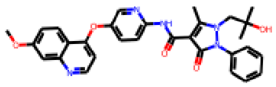	0.584737	COc1ccc2c(=O)cc(CNC(=O)c3c(C)n(C)n(-c4ccccc4)c3=O)nc3cccc2c3c1	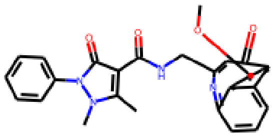	9.0	0.638163	3.995589	2.988471	5	0.774651
**805**	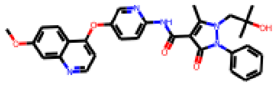	0.584567	COc1ccc2c(=O)cc(CNC(=O)c3c(C)n(C)n(-c4ccccc4)c3=O)nc3cccc2c3c1	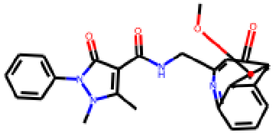	9.0	0.638163	3.995589	2.988471	5	0.774651
**806**	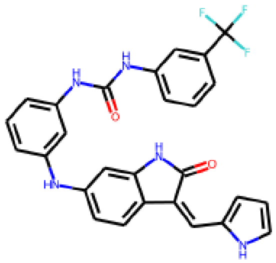	0.584561	COc1ccc(NC(=O)Nc2ccc3c(c2)NC(=O)/C3=C/c2ccc[nH]2)cc1	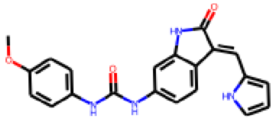	4.0	0.638163	3.995589	2.988471	4	0.768944
**807**	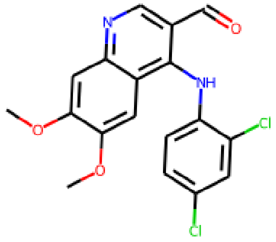	0.584457	COc1cc2ncc(C(=O)O)c(Nc3cccc(Cl)c3Cl)c2cc1OC1C(C)C1	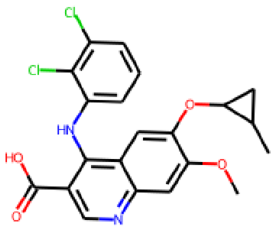	2.0	0.623362	2.525411	2.997881	4	0.774857

	src known mol	similarity	smiles	mol	Class	qed	logP	SAS	rings	kinase inhibition likeliness
**808**	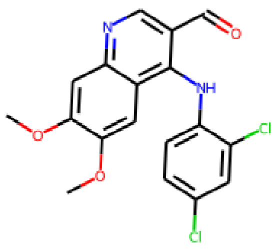	0.584457	COc1cc2ncc(C(=O)O)c(Nc3cccc(Cl)c3Cl)c2cc1OC1C(C)C1	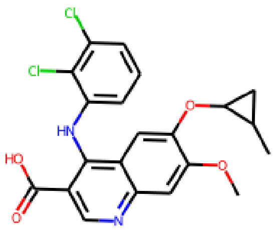	2.0	0.623362	2.525411	2.997881	4	0.774857
**809**	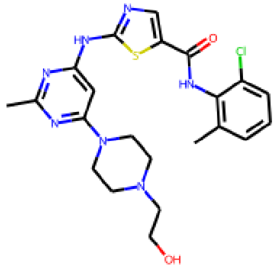	0.584232	Cc1nc(Nc2ncc(C(=O)Nc3c(C)cccc3Cl)s2)cc(N2CCOC(=O)C2)n1	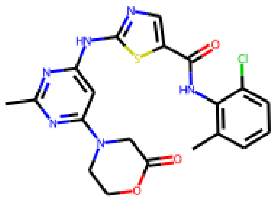	6.0	0.635315	4.542409	3.856266	4	0.801279
**810**	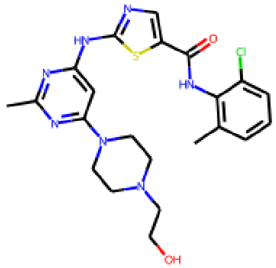	0.584232	Cc1nc(Nc2ncc(C(=O)Nc3c(C)cccc3Cl)s2)cc(N2CCOC(=O)C2)n1	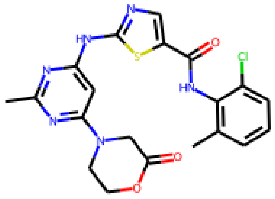	6.0	0.635315	4.542409	3.856266	4	0.801279
**811**	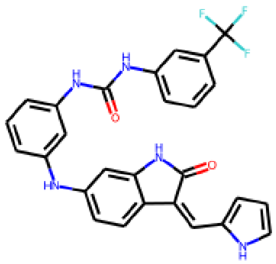	0.584151	COc1ccc(NC(=O)Nc2ccc3c(c2)NC(=O)/C3=C/c2ccc[nH]2)cc1	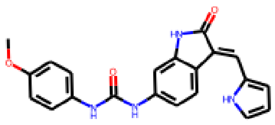	4.0	0.638163	3.995589	2.988471	4	0.768944

	src known mol	similarity	smiles	mol	Class	qed	logP	SAS	rings	kinase inhibition likeliness
**812**	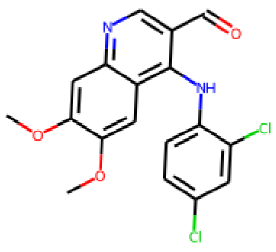	0.584053	COc1cc2ncc(C(=O)O)c(Nc3cccc(Cl)c3Cl)c2cc1OC1C(C)C1	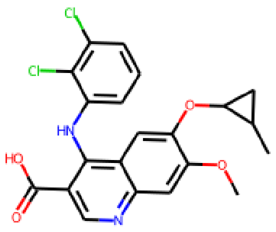	2.0	0.623362	2.525411	2.997881	4	0.774857
**813**	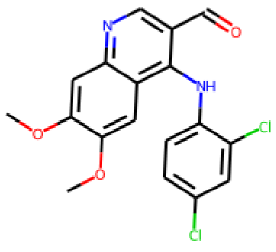	0.584053	COc1cc2ncc(C(=O)O)c(Nc3cccc(Cl)c3Cl)c2cc1OC1C(C)C1	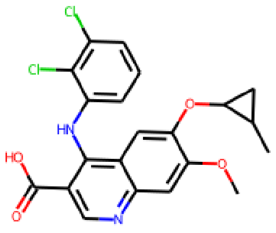	2.0	0.623362	2.525411	2.997881	4	0.774857
**814**	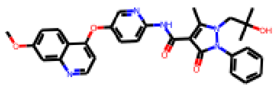	0.583992	COc1ccc2c(=O)cc(CNC(=O)c3c(C)n(C)n(-c4ccccc4)c3=O)nc3cccc2c3c1	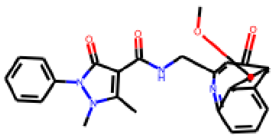	9.0	0.638163	3.995589	2.988471	5	0.774651
**815**	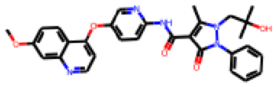	0.583907	COc1ccc2c(=O)cc(CNC(=O)c3c(C)n(C)n(-c4ccccc4)c3=O)nc3cccc2c3c1	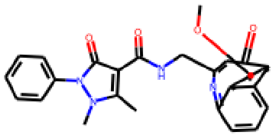	9.0	0.638163	3.995589	2.988471	5	0.774651

	src known mol	similarity	smiles	mol	Class	qed	logP	SAS	rings	kinase inhibition likeliness
**816**	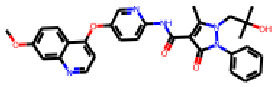	0.583907	COc1ccc2c(=O)cc(CNC(=O)c3c(C)n(C)n(-c4ccccc4)c3=O)nc3cccc2c3c1	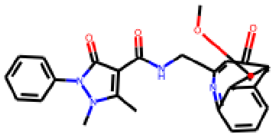	9.0	0.638163	3.995589	2.988471	5	0.774651
**817**	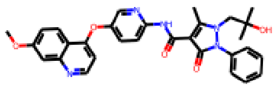	0.583861	COc1ccc2c(=O)cc(CNC(=O)c3c(C)n(C)n(-c4ccccc4)c3=O)nc3cccc2c3c1	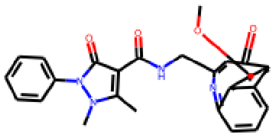	9.0	0.638163	3.995589	2.988471	5	0.774651
**818**	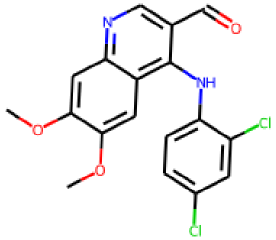	0.583851	COc1cc2ncc(C(=O)O)c(Nc3cccc(Cl)c3Cl)c2cc1OC1C(C)C1	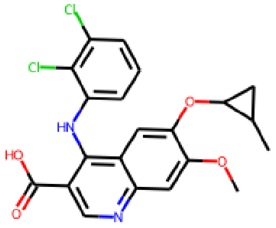	2.0	0.623362	2.525411	2.997881	4	0.774857
**819**	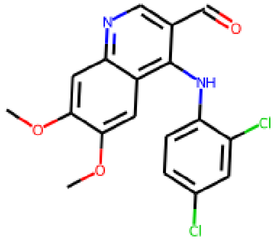	0.583725	COc1cc2ncc(C(=O)O)c(Nc3cccc(Cl)c3Cl)c2cc1OC1C(C)C1	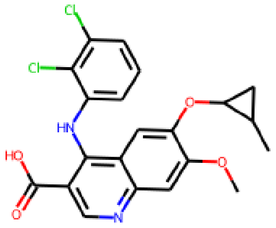	2.0	0.623362	2.525411	2.997881	4	0.774857

	src known mol	similarity	smiles	mol	Class	qed	logP	SAS	rings	kinase inhibition likeliness
**820**	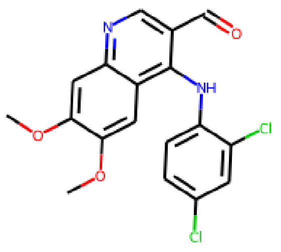	0.583725	COc1cc2ncc(C(=O)O)c(Nc3cccc(Cl)c3Cl)c2cc1OC1C(C)C1	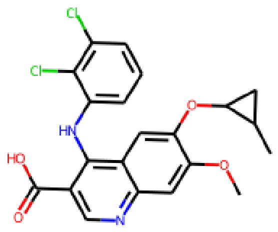	2.0	0.623362	2.525411	2.997881	4	0.774857
**821**	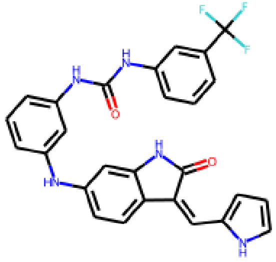	0.583685	COc1ccc(NC(=O)Nc2ccc3c(c2)NC(=O)/C3=C/c2ccc[nH]2)cc1	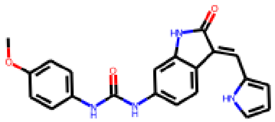	4.0	0.638163	3.995589	2.988471	4	0.768944
**822**	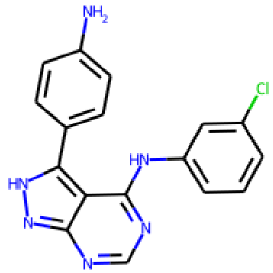	0.583673	c1ccc(Nc2ncnc3n[nH]c(-c4ccccc4)c23)cc1	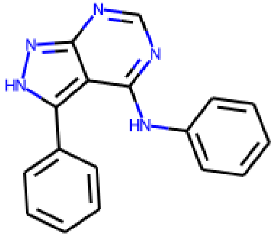	0.0	0.638163	3.995589	2.988471	4	0.800634
**823**	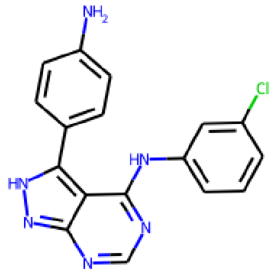	0.583470	c1ccc(Nc2ncnc3n[nH]c(-c4ccccc4)c23)cc1	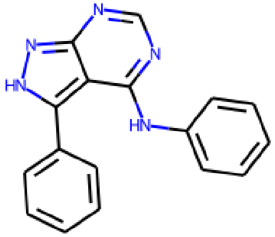	0.0	0.638163	3.995589	2.988471	4	0.800634

	src known mol	similarity	smiles	mol	Class	qed	logP	SAS	rings	kinase inhibition likeliness
**824**	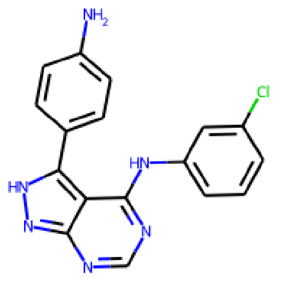	0.583470	c1ccc(Nc2ncnc3n[nH]c(-c4ccccc4)c23)cc1	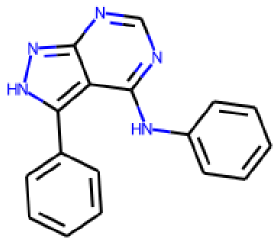	0.0	0.638163	3.995589	2.988471	4	0.800634
**825**	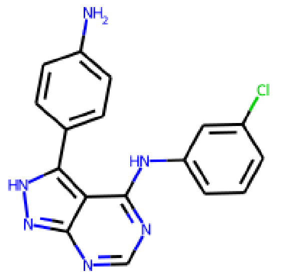	0.583333	c1ccc(Nc2ncnc3n[nH]c(-c4ccccc4)c23)cc1	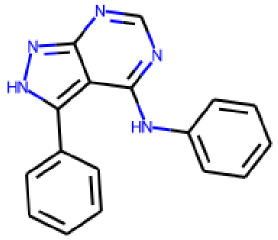	0.0	0.638163	3.995589	2.988471	4	0.800634
**826**	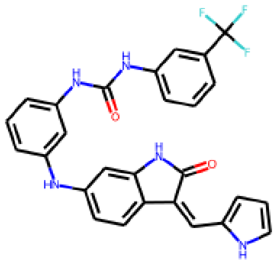	0.583333	COc1ccc(NC(=O)Nc2ccc3c(c2)NC(=O)/C3=C/c2ccc[nH]2)cc1	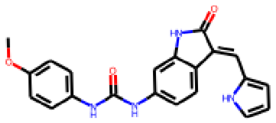	4.0	0.638163	3.995589	2.988471	4	0.768944
**827**	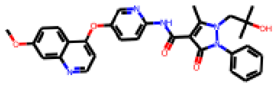	0.583246	COc1ccc2c(=O)cc(CNC(=O)c3c(C)n(C)n(-c4ccccc4)c3=O)nc3cccc2c3c1	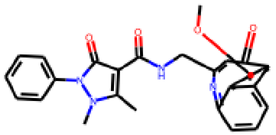	9.0	0.638163	3.995589	2.988471	5	0.774651

	src known mol	similarity	smiles	mol	Class	qed	logP	SAS	rings	kinase inhibition likeliness
**828**	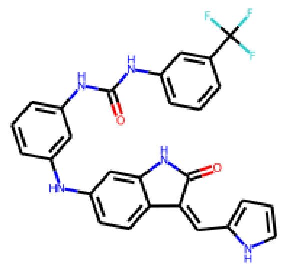	0.583158	COc1ccc(NC(=O)Nc2ccc3c(c2)NC(=O)/C3=C/c2ccc[nH]2)cc1	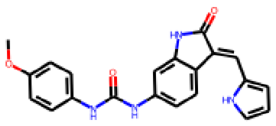	4.0	0.638163	3.995589	2.988471	4	0.768944
**829**	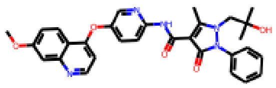	0.583157	COc1ccc2c(=O)cc(CNC(=O)c3c(C)n(C)n(-c4ccccc4)c3=O)nc3cccc2c3c1	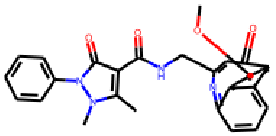	9.0	0.638163	3.995589	2.988471	5	0.774651
**830**	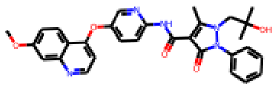	0.583157	COc1ccc2c(=O)cc(CNC(=O)c3c(C)n(C)n(-c4ccccc4)c3=O)nc3cccc2c3c1	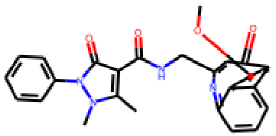	9.0	0.638163	3.995589	2.988471	5	0.774651
**831**	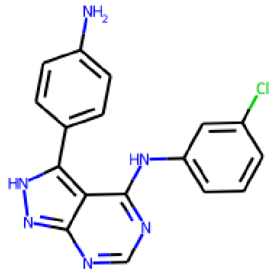	0.583133	c1ccc(Nc2ncnc3n[nH]c(-c4ccccc4)c23)cc1	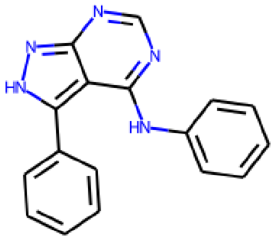	0.0	0.638163	3.995589	2.988471	4	0.800634

	src known mol	similarity	smiles	mol	Class	qed	logP	SAS	rings	kinase inhibition likeliness
**832**	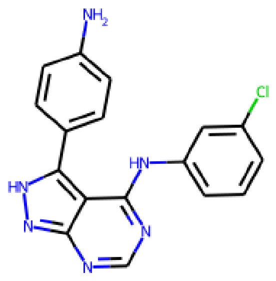	0.583131	c1ccc(Nc2ncnc3n[nH]c(-c4ccccc4)c23)cc1	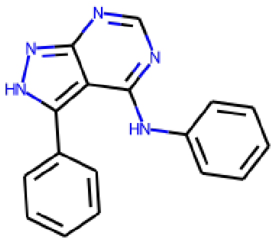	0.0	0.638163	3.995589	2.988471	4	0.800634
**833**	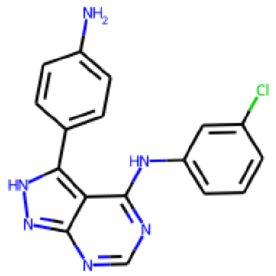	0.583059	c1ccc(Nc2ncnc3n[nH]c(-c4ccccc4)c23)cc1	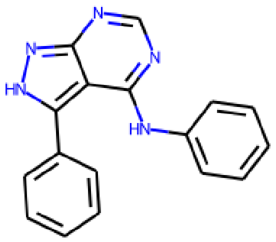	0.0	0.638163	3.995589	2.988471	4	0.800634
**834**	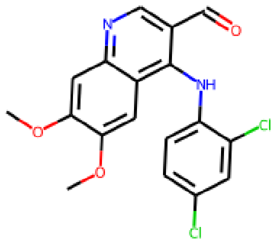	0.583006	COc1cc2ncc(C(=O)O)c(Nc3cccc(Cl)c3Cl)c2cc1OC1C(C)C1	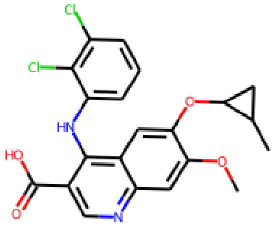	2.0	0.623362	2.525411	2.997881	4	0.774857
**835**	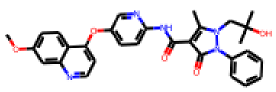	0.582938	COc1ccc2c(=O)cc(CNC(=O)c3c(C)n(C)n(-c4ccccc4)c3=O)nc3cccc2c3c1	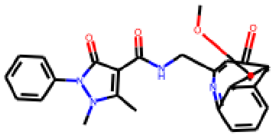	9.0	0.638163	3.995589	2.988471	5	0.774651

	src known mol	similarity	smiles	mol	Class	qed	logP	SAS	rings	kinase inhibition likeliness
**836**	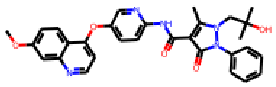	0.582938	COc1ccc2c(=O)cc(CNC(=O)c3c(C)n(C)n(-c4ccccc4)c3=O)nc3cccc2c3c1	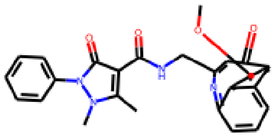	9.0	0.638163	3.995589	2.988471	5	0.774651
**837**	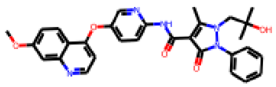	0.582938	COc1ccc2c(=O)cc(CNC(=O)c3c(C)n(C)n(-c4ccccc4)c3=O)nc3cccc2c3c1	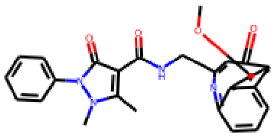	9.0	0.638163	3.995589	2.988471	5	0.774651
**838**	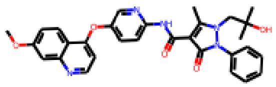	0.582938	COc1ccc2c(=O)cc(CNC(=O)c3c(C)n(C)n(-c4ccccc4)c3=O)nc3cccc2c3c1	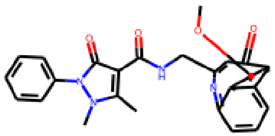	9.0	0.638163	3.995589	2.988471	5	0.774651
**839**	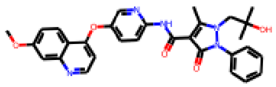	0.582938	COc1ccc2c(=O)cc(CNC(=O)c3c(C)n(C)n(-c4ccccc4)c3=O)nc3cccc2c3c1	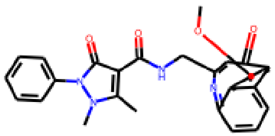	9.0	0.638163	3.995589	2.988471	5	0.774651

	src known mol	similarity	smiles	mol	Class	qed	logP	SAS	rings	kinase inhibition likeliness
**840**	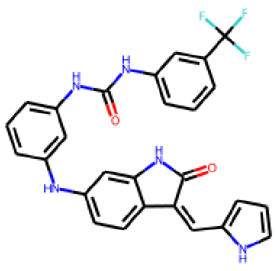	0.582925	COc1ccc(NC(=O)Nc2ccc3c(c2)NC(=O)/C3=C/c2ccc[nH]2)cc1	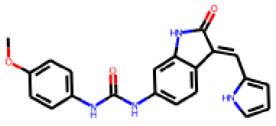	4.0	0.638163	3.995589	2.988471	4	0.768944
**841**	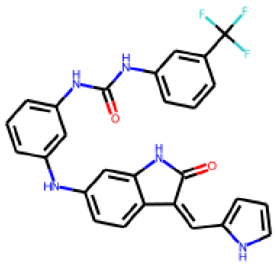	0.582925	COc1ccc(NC(=O)Nc2ccc3c(c2)NC(=O)/C3=C/c2ccc[nH]2)cc1	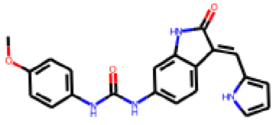	4.0	0.638163	3.995589	2.988471	4	0.768944
**842**	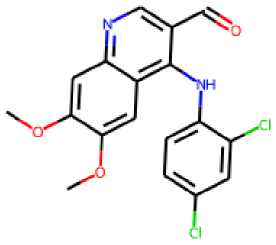	0.582900	COc1cc2ncc(C(=O)O)c(Nc3cccc(Cl)c3Cl)c2cc1OC1C(C)C1	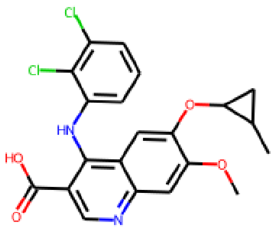	2.0	0.623362	2.525411	2.997881	4	0.774857
**843**	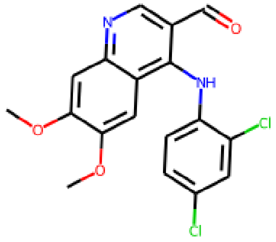	0.582900	COc1cc2ncc(C(=O)O)c(Nc3cccc(Cl)c3Cl)c2cc1OC1C(C)C1	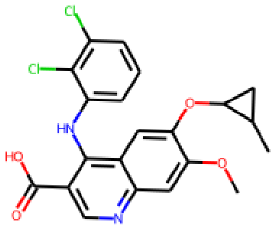	2.0	0.623362	2.525411	2.997881	4	0.774857

	src known mol	similarity	smiles	mol	Class	qed	logP	SAS	rings	kinase inhibition likeliness
**844**	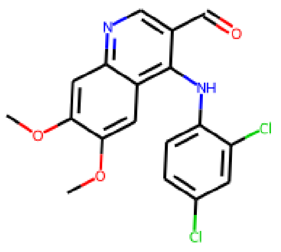	0.582892	COc1cc2ncc(C(=O)O)c(Nc3cccc(Cl)c3Cl)c2cc1OC1C(C)C1	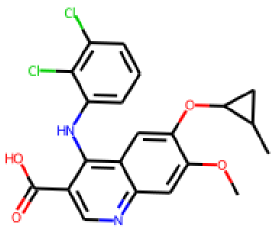	2.0	0.623362	2.525411	2.997881	4	0.774857
**845**	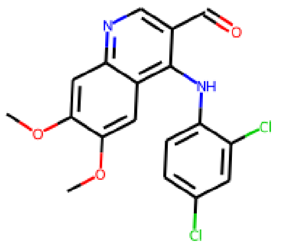	0.582892	COc1cc2ncc(C(=O)O)c(Nc3cccc(Cl)c3Cl)c2cc1OC1C(C)C1	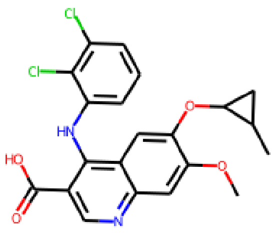	2.0	0.623362	2.525411	2.997881	4	0.774857
**846**	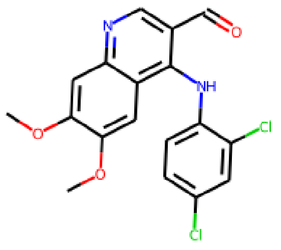	0.582892	COc1cc2ncc(C(=O)O)c(Nc3cccc(Cl)c3Cl)c2cc1OC1C(C)C1	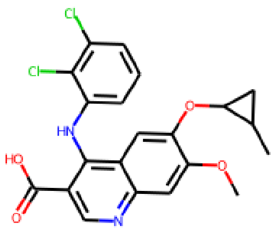	2.0	0.623362	2.525411	2.997881	4	0.774857
**847**	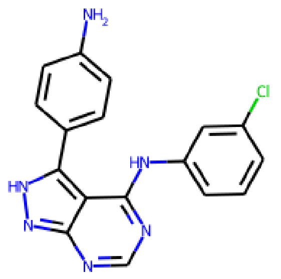	0.582857	c1ccc(Nc2ncnc3n[nH]c(-c4ccccc4)c23)cc1	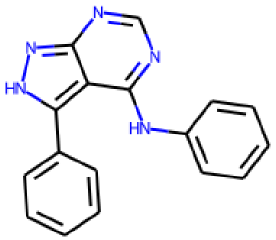	0.0	0.638163	3.995589	2.988471	4	0.800634

	src known mol	similarity	smiles	mol	Class	qed	logP	SAS	rings	kinase inhibition likeliness
**848**	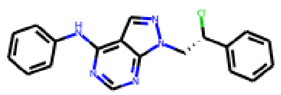	0.582761	Cc1ccc(C(=O)Nc2ccon2)cc1Nc1ncnc2c1cnn2C1cccccc1	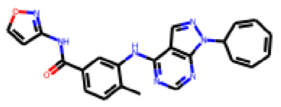	8.0	0.623362	2.525411	2.997881	5	0.770298
**849**	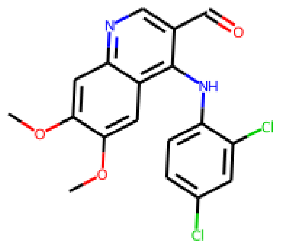	0.582642	COc1cc2ncc(C(=O)O)c(Nc3cccc(Cl)c3Cl)c2cc1OC1C(C)C1	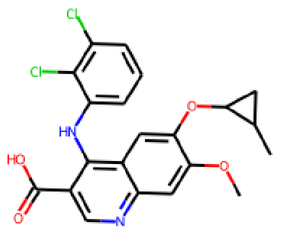	2.0	0.623362	2.525411	2.997881	4	0.774857
**850**	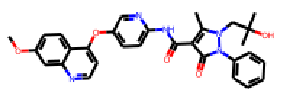	0.582627	COc1ccc2c(=O)cc(CNC(=O)c3c(C)n(C)n(-c4ccccc4)c3=O)nc3cccc2c3c1	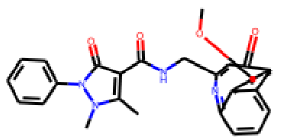	9.0	0.638163	3.995589	2.988471	5	0.774651
**851**	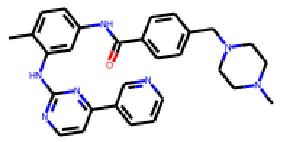	0.582601	Cc1ccc(NC(=O)c2ccccc2)cc1Nc1nccc(-c2cccnc2)n1	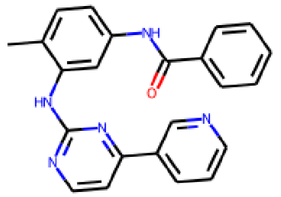	0.0	0.768132	3.608437	2.513170	4	0.854555

	src known mol	similarity	smiles	mol	Class	qed	logP	SAS	rings	kinase inhibition likeliness
**852**	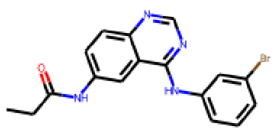	0.582517	O=C(CNC(=O)Oc1ccccc1)Nc1ccc2ncnc(Nc3cccc(Cl)c3)c2c1	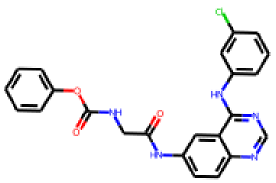	3.0	0.638163	3.995589	2.988471	4	0.793889
**853**	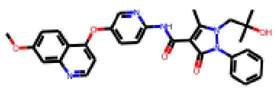	0.582452	COc1ccc2c(=O)cc(CNC(=O)c3c(C)n(C)n(-c4ccccc4)c3=O)nc3cccc2c3c1	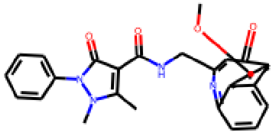	9.0	0.638163	3.995589	2.988471	5	0.774651
**854**	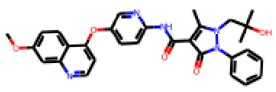	0.582406	COc1ccc2c(=O)cc(CNC(=O)c3c(C)n(C)n(-c4ccccc4)c3=O)nc3cccc2c3c1	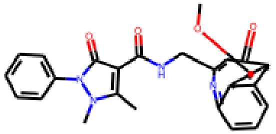	9.0	0.638163	3.995589	2.988471	5	0.774651
**855**	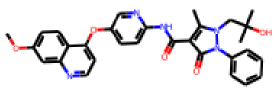	0.582406	COc1ccc2c(=O)cc(CNC(=O)c3c(C)n(C)n(-c4ccccc4)c3=O)nc3cccc2c3c1	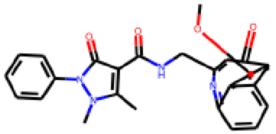	9.0	0.638163	3.995589	2.988471	5	0.774651

	src known mol	similarity	smiles	mol	Class	qed	logP	SAS	rings	kinase inhibition likeliness
**856**	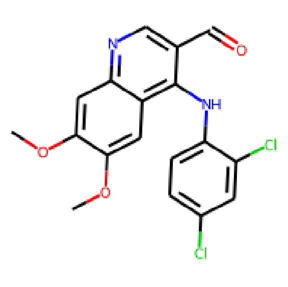	0.582384	COc1cc2ncc(C(=O)O)c(Nc3cccc(Cl)c3Cl)c2cc1OC1C(C)C1	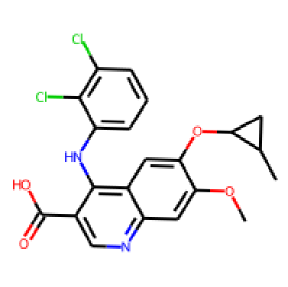	2.0	0.623362	2.525411	2.997881	4	0.774857
**857**	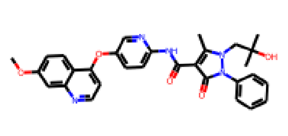	0.582319	COc1ccc2c(=O)cc(CNC(=O)c3c(C)n(C)n(-c4ccccc4)c3=O)nc3cccc2c3c1	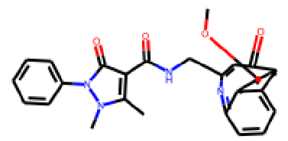	9.0	0.638163	3.995589	2.988471	5	0.774651
**858**	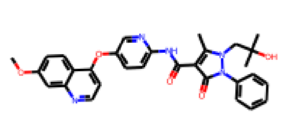	0.582319	COc1ccc2c(=O)cc(CNC(=O)c3c(C)n(C)n(-c4ccccc4)c3=O)nc3cccc2c3c1	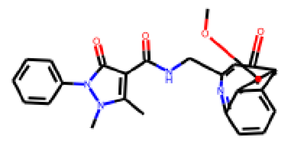	9.0	0.638163	3.995589	2.988471	5	0.774651
**859**	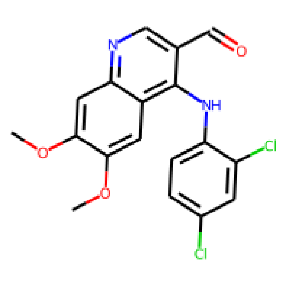	0.582269	COc1cc2ncc(C(=O)O)c(Nc3cccc(Cl)c3Cl)c2cc1OC1C(C)C1	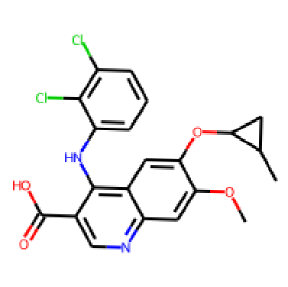	2.0	0.623362	2.525411	2.997881	4	0.774857

	src known mol	similarity	smiles	mol	Class	qed	logP	SAS	rings	kinase inhibition likeliness
**860**	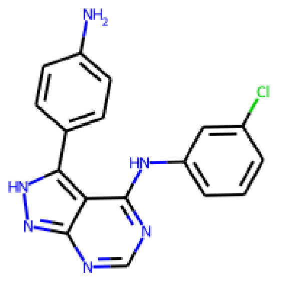	0.582160	c1ccc(Nc2ncnc3n[nH]c(-c4ccccc4)c23)cc1	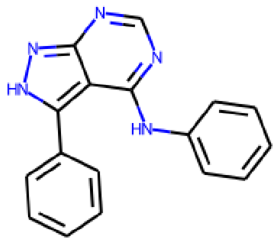	0.0	0.638163	3.995589	2.988471	4	0.800634
**861**	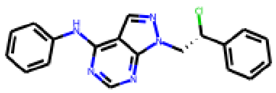	0.581994	Cc1ccc(C(=O)Nc2ccon2)cc1Nc1ncnc2c1cnn2C1cccccc1	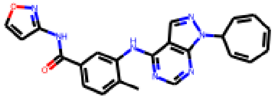	8.0	0.623362	2.525411	2.997881	5	0.770298
**862**	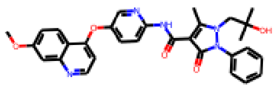	0.581761	COc1ccc2c(=O)cc(CNC(=O)c3c(C)n(C)n(-c4ccccc4)c3=O)nc3cccc2c3c1	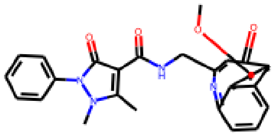	9.0	0.638163	3.995589	2.988471	5	0.774651
**863**	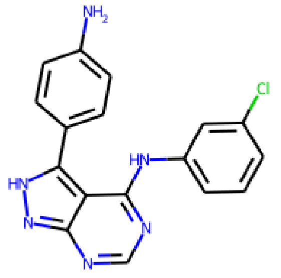	0.581758	c1ccc(Nc2ncnc3n[nH]c(-c4ccccc4)c23)cc1	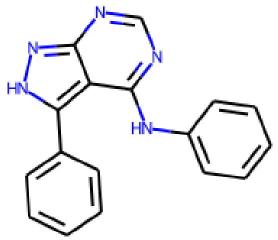	0.0	0.638163	3.995589	2.988471	4	0.800634

	src known mol	similarity	smiles	mol	Class	qed	logP	SAS	rings	kinase inhibition likeliness
**864**	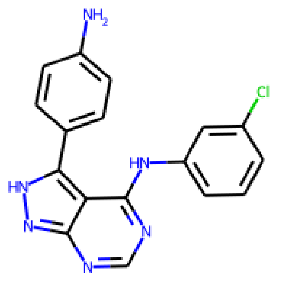	0.581699	c1ccc(Nc2ncnc3n[nH]c(-c4ccccc4)c23)cc1	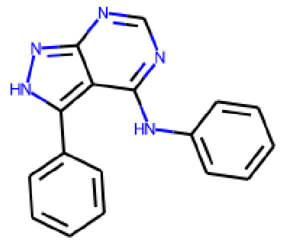	0.0	0.638163	3.995589	2.988471	4	0.800634
**865**	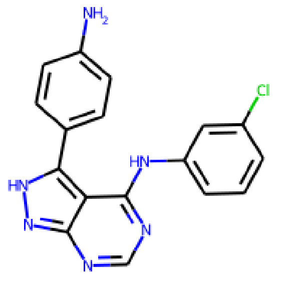	0.581699	c1ccc(Nc2ncnc3n[nH]c(-c4ccccc4)c23)cc1	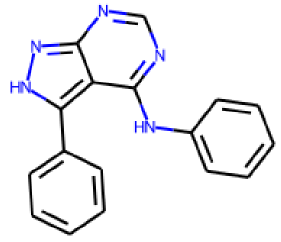	0.0	0.638163	3.995589	2.988471	4	0.800634
**866**	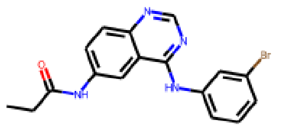	0.581605	O=C(CNC(=O)Oc1ccccc1)Nc1ccc2ncnc(Nc3cccc(Cl)c3)c2c1	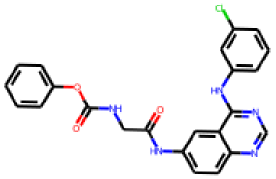	3.0	0.638163	3.995589	2.988471	4	0.793889
**867**	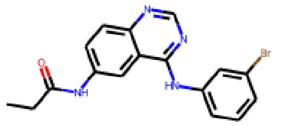	0.581605	O=C(CNC(=O)Oc1ccccc1)Nc1ccc2ncnc(Nc3cccc(Cl)c3)c2c1	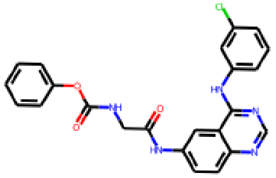	3.0	0.638163	3.995589	2.988471	4	0.793889

	src known mol	similarity	smiles	mol	Class	qed	logP	SAS	rings	kinase inhibition likeliness
**868**	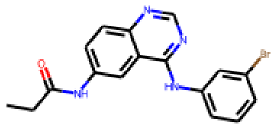	0.581605	O=C(CNC(=O)Oc1ccccc1)Nc1ccc2ncnc(Nc3cccc(Cl)c3)c2c1	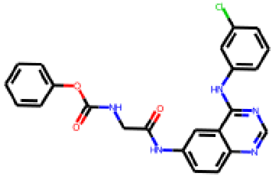	3.0	0.638163	3.995589	2.988471	4	0.793889
**869**	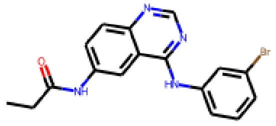	0.581605	O=C(CNC(=O)Oc1ccccc1)Nc1ccc2ncnc(Nc3cccc(Cl)c3)c2c1	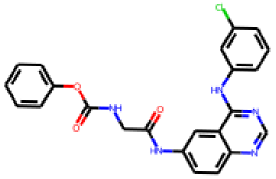	3.0	0.638163	3.995589	2.988471	4	0.793889
**870**	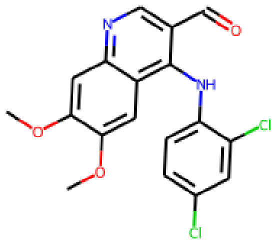	0.581577	COc1cc2ncc(C(=O)O)c(Nc3cccc(Cl)c3Cl)c2cc1OC1C(C)C1	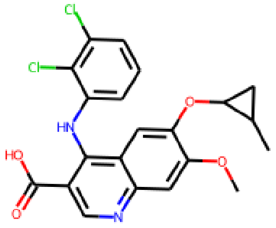	2.0	0.623362	2.525411	2.997881	4	0.774857
**871**	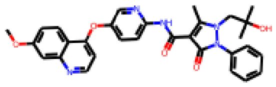	0.581568	COc1ccc2c(=O)cc(CNC(=O)c3c(C)n(C)n(-c4ccccc4)c3=O)nc3cccc2c3c1	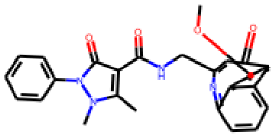	9.0	0.638163	3.995589	2.988471	5	0.774651

	src known mol	similarity	smiles	mol	Class	qed	logP	SAS	rings	kinase inhibition likeliness
**872**	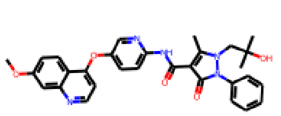	0.581568	COc1ccc2c(=O)cc(CNC(=O)c3c(C)n(C)n(-c4ccccc4)c3=O)nc3cccc2c3c1	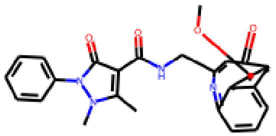	9.0	0.638163	3.995589	2.988471	5	0.774651
**873**	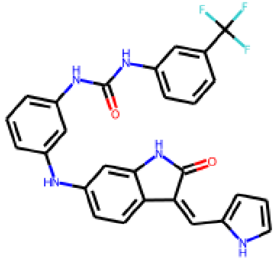	0.581346	COc1ccc(NC(=O)Nc2ccc3c(c2)NC(=O)/C3=C/c2ccc[nH]2)cc1	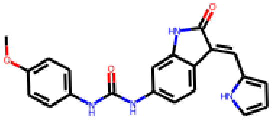	4.0	0.638163	3.995589	2.988471	4	0.768944
**874**	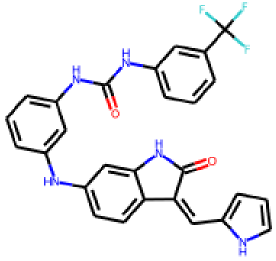	0.581346	COc1ccc(NC(=O)Nc2ccc3c(c2)NC(=O)/C3=C/c2ccc[nH]2)cc1	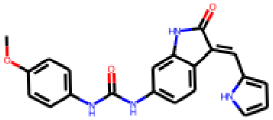	4.0	0.638163	3.995589	2.988471	4	0.768944
**875**	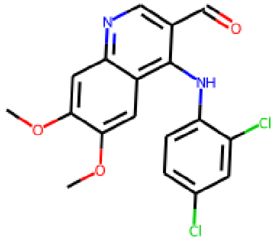	0.581176	COc1cc2ncc(C(=O)O)c(Nc3cccc(Cl)c3Cl)c2cc1OC1C(C)C1	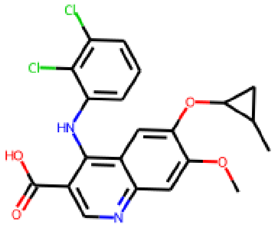	2.0	0.623362	2.525411	2.997881	4	0.774857

	src known mol	similarity	smiles	mol	Class	qed	logP	SAS	rings	kinase inhibition likeliness
**876**	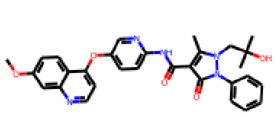	0.581138	COc1ccc2c(=O)cc(CNC(=O)c3c(C)n(C)n(-c4ccccc4)c3=O)nc3cccc2c3c1	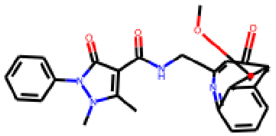	9.0	0.638163	3.995589	2.988471	5	0.774651
**877**	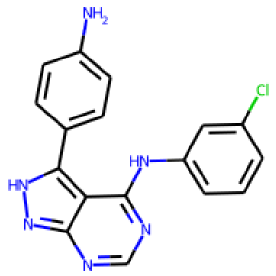	0.580983	c1ccc(Nc2ncnc3n[nH]c(-c4ccccc4)c23)cc1	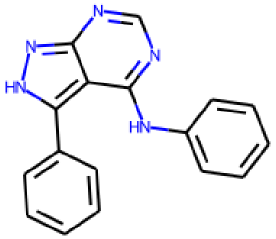	0.0	0.638163	3.995589	2.988471	4	0.800634
**878**	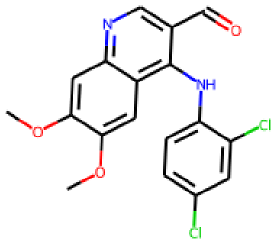	0.580719	COc1cc2ncc(C(=O)O)c(Nc3cccc(Cl)c3Cl)c2cc1OC1C(C)C1	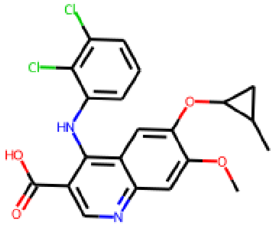	2.0	0.623362	2.525411	2.997881	4	0.774857
**879**	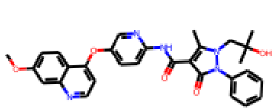	0.580713	COc1ccc2c(=O)cc(CNC(=O)c3c(C)n(C)n(-c4ccccc4)c3=O)nc3cccc2c3c1	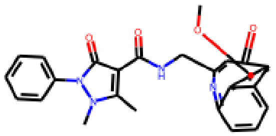	9.0	0.638163	3.995589	2.988471	5	0.774651

	src known mol	similarity	smiles	mol	Class	qed	logP	SAS	rings	kinase inhibition likeliness
**880**	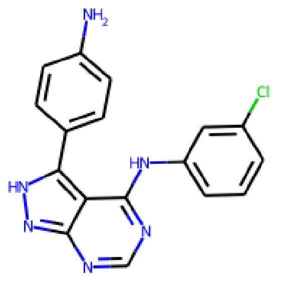	0.580672	c1ccc(Nc2ncnc3n[nH]c(-c4ccccc4)c23)cc1	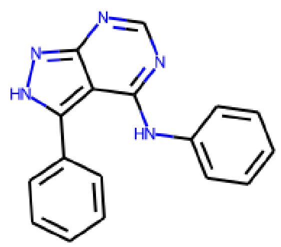	0.0	0.638163	3.995589	2.988471	4	0.800634
**881**	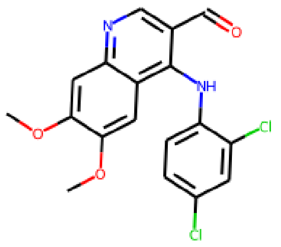	0.580645	COc1cc2ncc(C(=O)O)c(Nc3cccc(Cl)c3Cl)c2cc1OC1C(C)C1	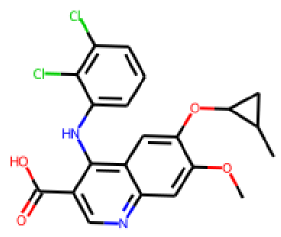	2.0	0.623362	2.525411	2.997881	4	0.774857
**882**	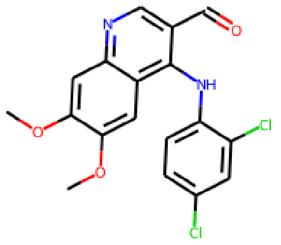	0.580645	COc1cc2ncc(C(=O)O)c(Nc3cccc(Cl)c3Cl)c2cc1OC1C(C)C1	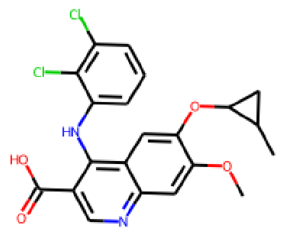	2.0	0.623362	2.525411	2.997881	4	0.774857
**883**	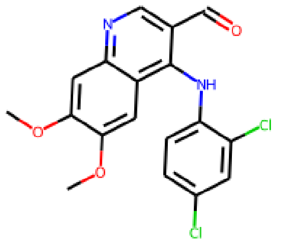	0.580545	COc1cc2ncc(C(=O)O)c(Nc3cccc(Cl)c3Cl)c2cc1OC1C(C)C1	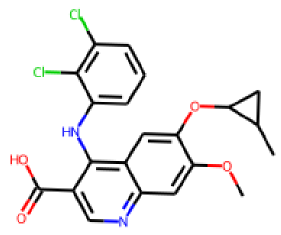	2.0	0.623362	2.525411	2.997881	4	0.774857

	src known mol	similarity	smiles	mol	Class	qed	logP	SAS	rings	kinase inhibition likeliness
**884**	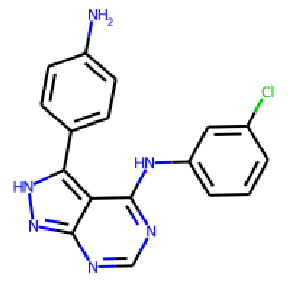	0.580408	c1ccc(Nc2ncnc3n[nH]c(-c4ccccc4)c23)cc1	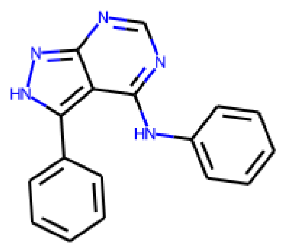	0.0	0.638163	3.995589	2.988471	4	0.800634
**885**	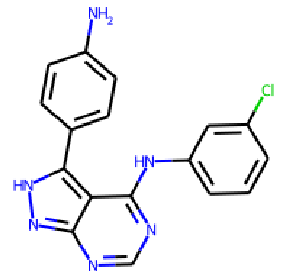	0.580357	c1ccc(Nc2ncnc3n[nH]c(-c4ccccc4)c23)cc1	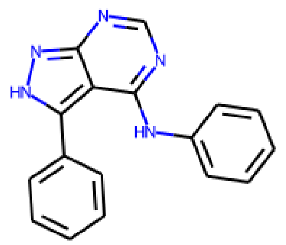	0.0	0.638163	3.995589	2.988471	4	0.800634
**886**	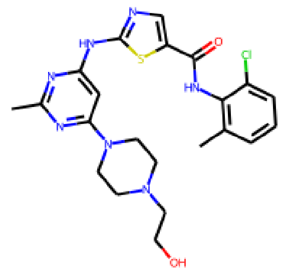	0.580357	Cc1nc(Nc2ncc(C(=O)Nc3c(C)cccc3Cl)s2)cc(N2CCOC(=O)C2)n1	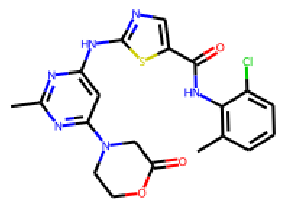	6.0	0.635315	4.542409	3.856266	4	0.801279
**887**	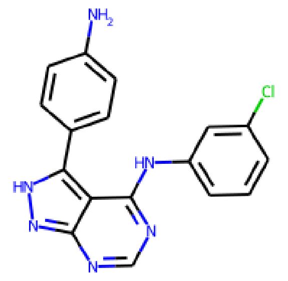	0.580277	c1ccc(Nc2ncnc3n[nH]c(-c4ccccc4)c23)cc1	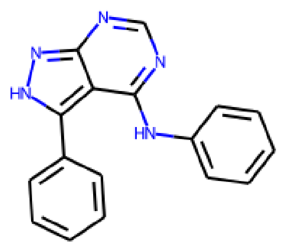	0.0	0.638163	3.995589	2.988471	4	0.800634

	src known mol	similarity	smiles	mol	Class	qed	logP	SAS	rings	kinase inhibition likeliness
**888**	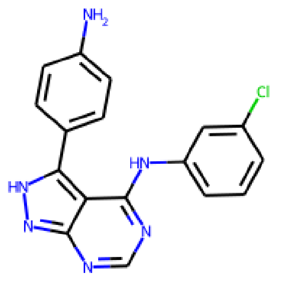	0.580277	c1ccc(Nc2ncnc3n[nH]c(-c4ccccc4)c23)cc1	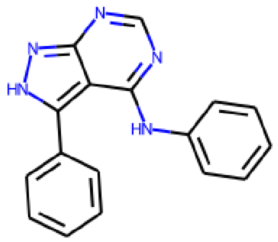	0.0	0.638163	3.995589	2.988471	4	0.800634
**889**	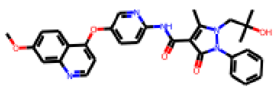	0.580253	COc1ccc2c(=O)cc(CNC(=O)c3c(C)n(C)n(-c4ccccc4)c3=O)nc3cccc2c3c1	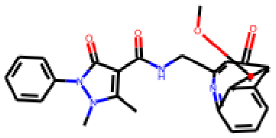	9.0	0.638163	3.995589	2.988471	5	0.774651
**890**	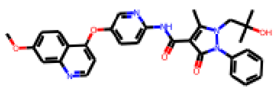	0.579894	COc1ccc2c(=O)cc(CNC(=O)c3c(C)n(C)n(-c4ccccc4)c3=O)nc3cccc2c3c1	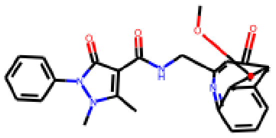	9.0	0.638163	3.995589	2.988471	5	0.774651
**891**	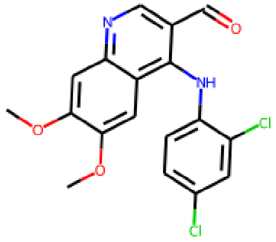	0.579886	COc1cc2ncc(C(=O)O)c(Nc3cccc(Cl)c3Cl)c2cc1OC1C(C)C1	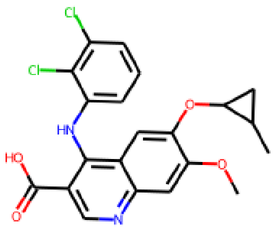	2.0	0.623362	2.525411	2.997881	4	0.774857

	src known mol	similarity	smiles	mol	Class	qed	logP	SAS	rings	kinase inhibition likeliness
**892**	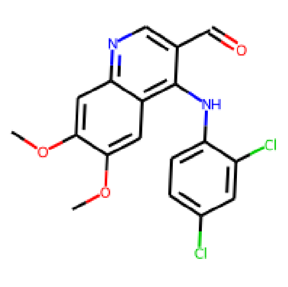	0.579886	COc1cc2ncc(C(=O)O)c(Nc3cccc(Cl)c3Cl)c2cc1OC1C(C)C1	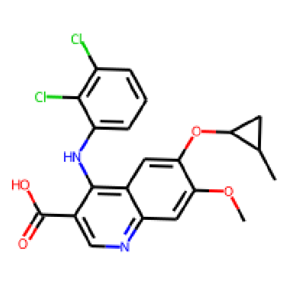	2.0	0.623362	2.525411	2.997881	4	0.774857
**893**	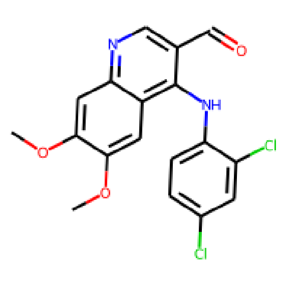	0.579659	COc1cc2ncc(C(=O)O)c(Nc3cccc(Cl)c3Cl)c2cc1OC1C(C)C1	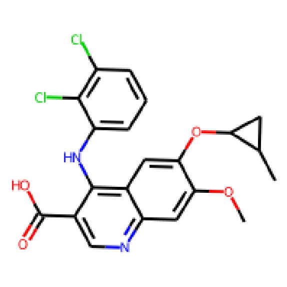	2.0	0.623362	2.525411	2.997881	4	0.774857
**894**	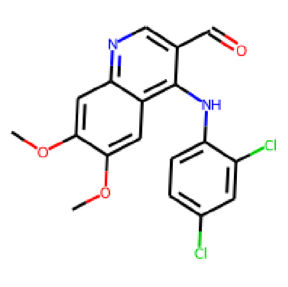	0.579659	COc1cc2ncc(C(=O)O)c(Nc3cccc(Cl)c3Cl)c2cc1OC1C(C)C1	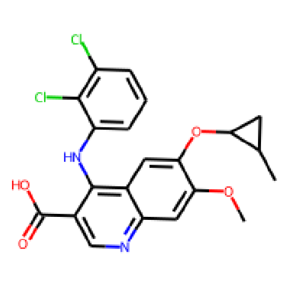	2.0	0.623362	2.525411	2.997881	4	0.774857
**895**	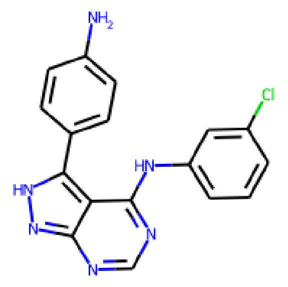	0.579592	c1ccc(Nc2ncnc3n[nH]c(-c4ccccc4)c32)cc1	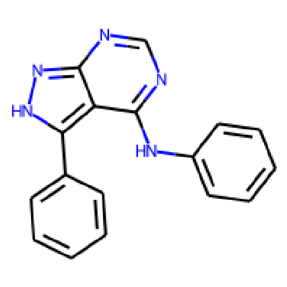	0.0	0.520676	2.336694	2.899742	4	0.831417

	src known mol	similarity	smiles	mol	Class	qed	logP	SAS	rings	kinase inhibition likeliness
**896**	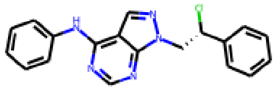	0.579474	Cc1ccc(C(=O)Nc2ccon2)cc1Nc1ncnc2c1cnn2C1cccccc1	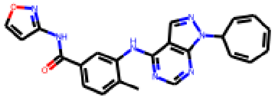	8.0	0.623362	2.525411	2.997881	5	0.770298
**897**	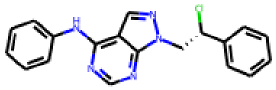	0.579310	Cc1ccc(C(=O)Nc2ccon2)cc1Nc1ncnc2c1cnn2C1cccccc1	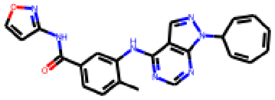	8.0	0.623362	2.525411	2.997881	5	0.770298
**898**	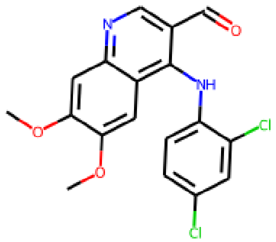	0.579295	COc1cc2ncc(C(=O)O)c(Nc3cccc(Cl)c3Cl)c2cc1OC1C(C)C1	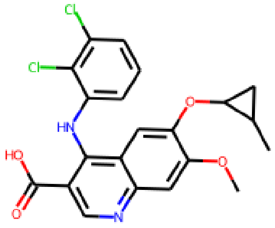	2.0	0.623362	2.525411	2.997881	4	0.774857
**899**	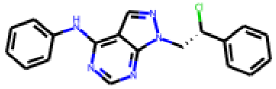	0.578881	Cc1ccc(C(=O)Nc2ccon2)cc1Nc1ncnc2c1cnn2C1cccccc1	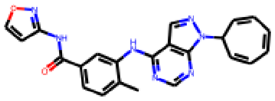	8.0	0.623362	2.525411	2.997881	5	0.770298

	src known mol	similarity	smiles	mol	Class	qed	logP	SAS	rings	kinase inhibition likeliness
**900**	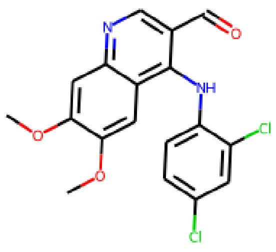	0.578871	COc1cc2ncc(C(=O)O)c(Nc3cccc(Cl)c3Cl)c2cc1OC1C(C)C1	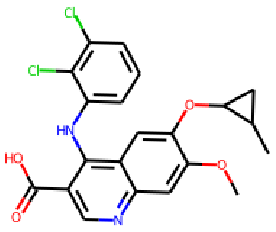	2.0	0.623362	2.525411	2.997881	4	0.774857
**901**	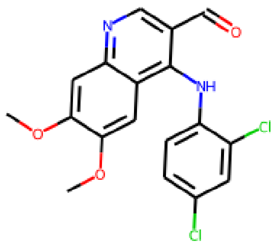	0.578871	COc1cc2ncc(C(=O)O)c(Nc3cccc(Cl)c3Cl)c2cc1OC1C(C)C1	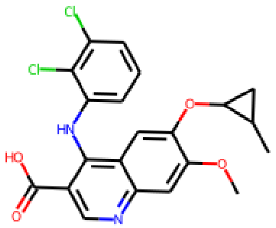	2.0	0.623362	2.525411	2.997881	4	0.774857
**902**	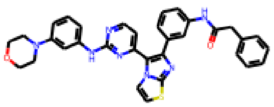	0.578757	ClCNc1nccc(-c2c(-c3ccc(F)cc3)nc3n2[C@H](CO)CC3)n1	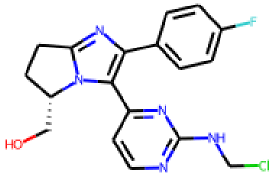	7.0	0.520676	2.336694	2.899742	4	0.812342
**903**	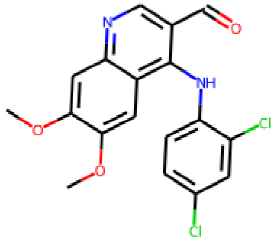	0.578711	COc1cc2ncc(C(=O)O)c(Nc3cccc(Cl)c3Cl)c2cc1OC1C(C)C1	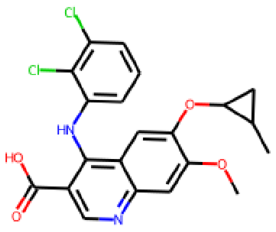	2.0	0.623362	2.525411	2.997881	4	0.774857

	src known mol	similarity	smiles	mol	Class	qed	logP	SAS	rings	kinase inhibition likeliness
**904**	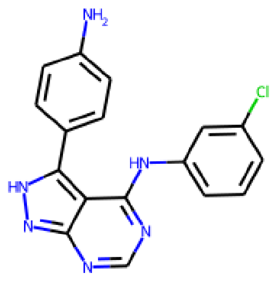	0.578647	c1ccc(Nc2ncnc3n[nH]c(-c4ccccc4)c23)cc1	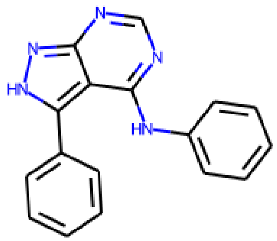	0.0	0.638163	3.995589	2.988471	4	0.800634
**905**	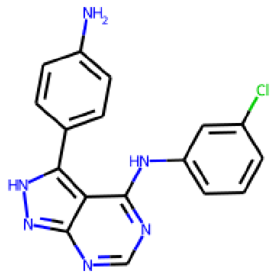	0.578606	c1ccc(Nc2ncnc3n[nH]c(-c4ccccc4)c23)cc1	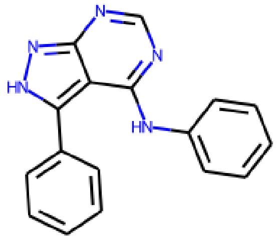	0.0	0.638163	3.995589	2.988471	4	0.800634
**906**	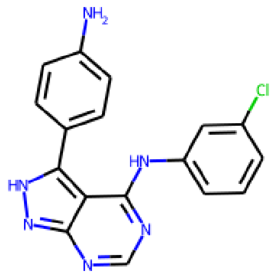	0.578566	c1ccc(Nc2ncnc3n[nH]c(-c4ccccc4)c23)cc1	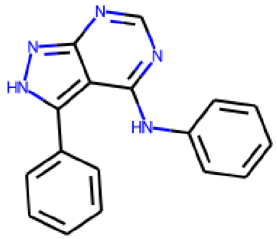	0.0	0.638163	3.995589	2.988471	4	0.800634
**907**	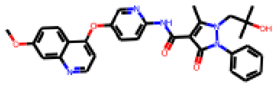	0.578401	COc1ccc2c(=O)cc(CNC(=O)c3c(C)n(C)n(-c4ccccc4)c3=O)nc3cccc2c3c1	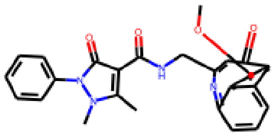	9.0	0.638163	3.995589	2.988471	5	0.774651

	src known mol	similarity	smiles	mol	Class	qed	logP	SAS	rings	kinase inhibition likeliness
**908**	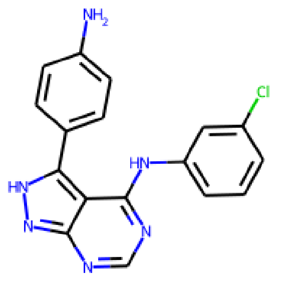	0.578352	c1ccc(Nc2ncnc3n[nH]c(-c4ccccc4)c23)cc1	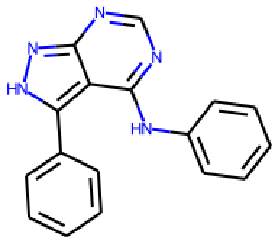	0.0	0.638163	3.995589	2.988471	4	0.800634
**909**	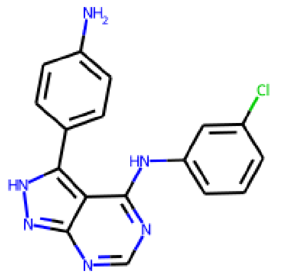	0.577922	c1ccc(Nc2ncnc3n[nH]c(-c4ccccc4)c23)cc1	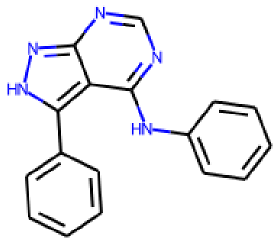	0.0	0.638163	3.995589	2.988471	4	0.800634
**910**	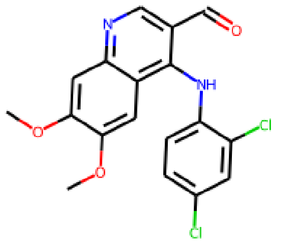	0.577695	COc1cc2ncc(C(=O)O)c(Nc3cccc(Cl)c3Cl)c2cc1OC1C(C)C1	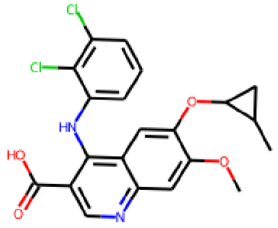	2.0	0.623362	2.525411	2.997881	4	0.774857
**911**	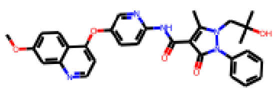	0.577426	COc1ccc2c(=O)cc(CNC(=O)c3c(C)n(C)n(-c4ccccc4)c3=O)nc3cccc3c2n1	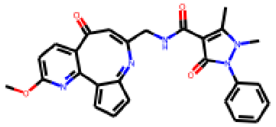	9.0	0.768132	3.608437	2.513170	5	0.823048

	src known mol	similarity	smiles	mol	Class	qed	logP	SAS	rings	kinase inhibition likeliness
**912**	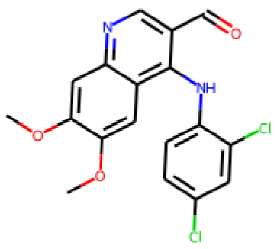	0.577364	COc1cc2ncc(C(=O)O)c(Nc3cccc(Cl)c3Cl)c2cc1OC1C(C)C1	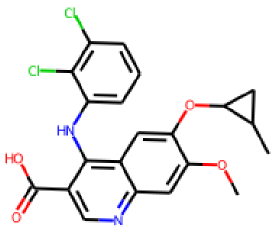	2.0	0.623362	2.525411	2.997881	4	0.774857
**913**	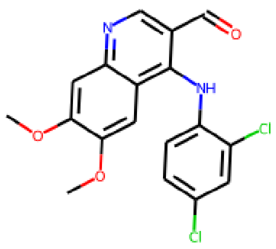	0.577364	COc1cc2ncc(C(=O)O)c(Nc3cccc(Cl)c3Cl)c2cc1OC1C(C)C1	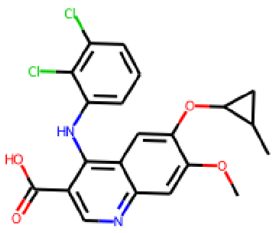	2.0	0.623362	2.525411	2.997881	4	0.774857
**914**	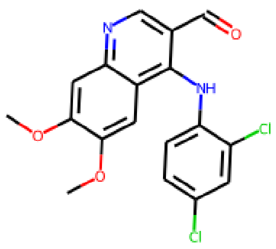	0.577364	COc1cc2ncc(C(=O)O)c(Nc3cccc(Cl)c3Cl)c2cc1OC1C(C)C1	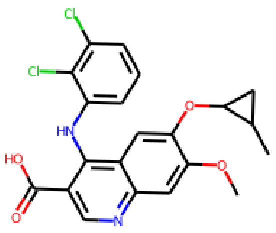	2.0	0.623362	2.525411	2.997881	4	0.774857
**915**	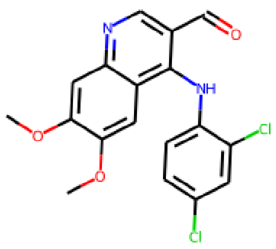	0.577364	COc1cc2ncc(C(=O)O)c(Nc3cccc(Cl)c3Cl)c2cc1OC1C(C)C1	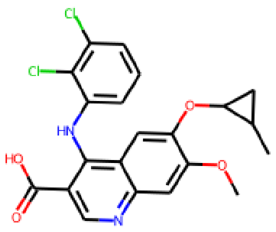	2.0	0.623362	2.525411	2.997881	4	0.774857

	src known mol	similarity	smiles	mol	Class	qed	logP	SAS	rings	kinase inhibition likeliness
**916**	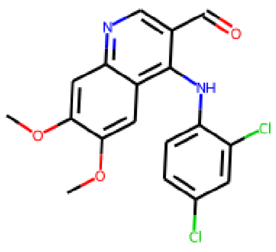	0.577201	COc1cc2ncc(C(=O)O)c(Nc3cccc(Cl)c3Cl)c2cc1OC1C(C)C1	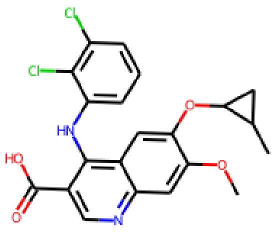	2.0	0.623362	2.525411	2.997881	4	0.774857
**917**	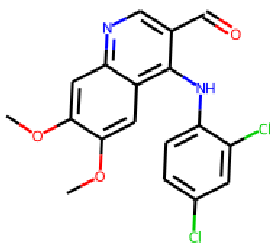	0.577201	COc1cc2ncc(C(=O)O)c(Nc3cccc(Cl)c3Cl)c2cc1OC1C(C)C1	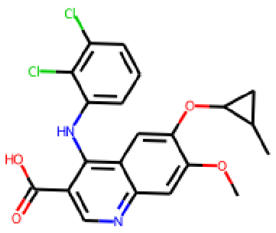	2.0	0.623362	2.525411	2.997881	4	0.774857
**918**	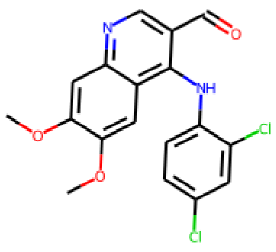	0.577118	COc1cc2ncc(C(=O)O)c(Nc3cccc(Cl)c3Cl)c2cc1OC1C(C)C1	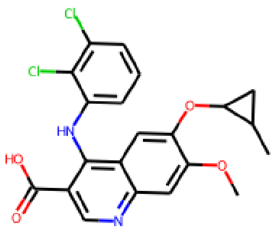	2.0	0.623362	2.525411	2.997881	4	0.774857
**919**	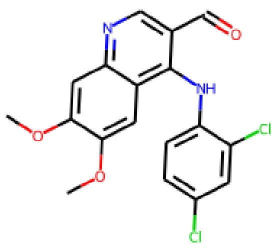	0.577118	COc1cc2ncc(C(=O)O)c(Nc3cccc(Cl)c3Cl)c2cc1OC1C(C)C1	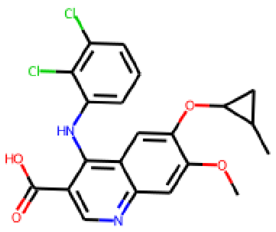	2.0	0.623362	2.525411	2.997881	4	0.774857

	src known mol	similarity	smiles	mol	Class	qed	logP	SAS	rings	kinase inhibition likeliness
**920**	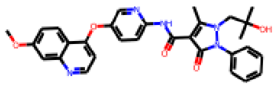	0.576984	COc1ccc2c(=O)cc(CNC(=O)c3c(C)n(C)n(-c4ccccc4)c3=O)nc3cccc2c3c1	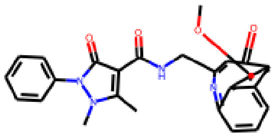	9.0	0.638163	3.995589	2.988471	5	0.774651
**921**	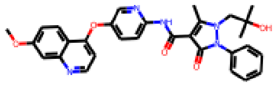	0.576984	COc1ccc2c(=O)cc(CNC(=O)c3c(C)n(C)n(-c4ccccc4)c3=O)nc3cccc2c3c1	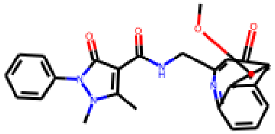	9.0	0.638163	3.995589	2.988471	5	0.774651
**922**	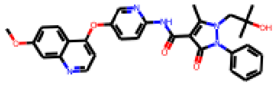	0.576984	COc1ccc2c(=O)cc(CNC(=O)c3c(C)n(C)n(-c4ccccc4)c3=O)nc3cccc2c3c1	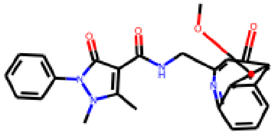	9.0	0.638163	3.995589	2.988471	5	0.774651
**923**	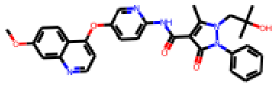	0.576984	COc1ccc2c(=O)cc(CNC(=O)c3c(C)n(C)n(-c4ccccc4)c3=O)nc3cccc2c3c1	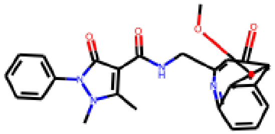	9.0	0.638163	3.995589	2.988471	5	0.774651

	src known mol	similarity	smiles	mol	Class	qed	logP	SAS	rings	kinase inhibition likeliness
**924**	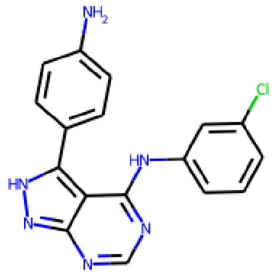	0.576923	c1ccc(Nc2ncnc3n[nH]c(-c4ccccc4)c23)cc1	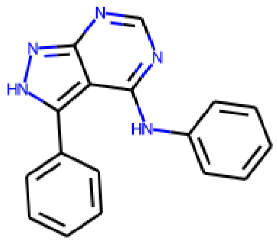	0.0	0.638163	3.995589	2.988471	4	0.800634
**925**	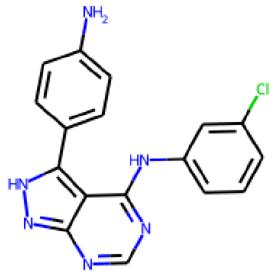	0.576892	c1ccc(Nc2ncnc3n[nH]c(-c4ccccc4)c23)cc1	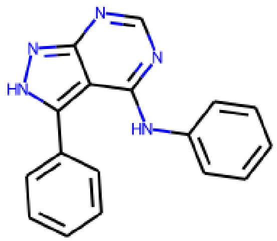	0.0	0.638163	3.995589	2.988471	4	0.800634
**926**	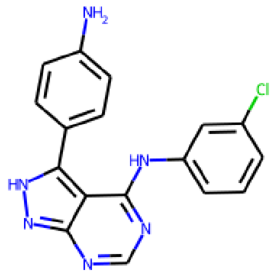	0.576861	c1ccc(Nc2ncnc3n[nH]c(-c4ccccc4)c23)cc1	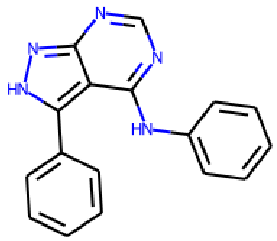	0.0	0.638163	3.995589	2.988471	4	0.800634
**927**	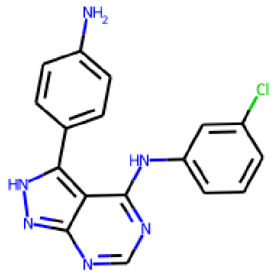	0.576861	c1ccc(Nc2ncnc3n[nH]c(-c4ccccc4)c23)cc1	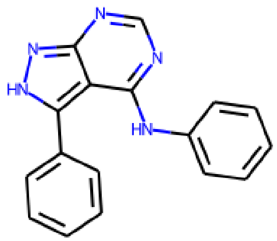	0.0	0.638163	3.995589	2.988471	4	0.800634

	src known mol	similarity	smiles	mol	Class	qed	logP	SAS	rings	kinase inhibition likeliness
**928**	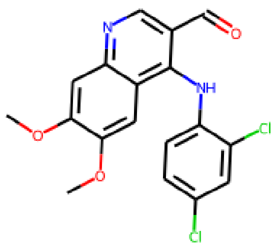	0.576758	COc1cc2ncc(C(=O)O)c(Nc3cccc(Cl)c3Cl)c2cc1OC1C(C)C1	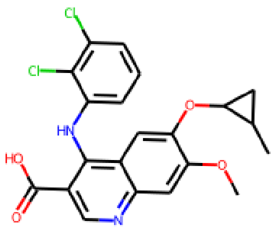	2.0	0.623362	2.525411	2.997881	4	0.774857
**929**	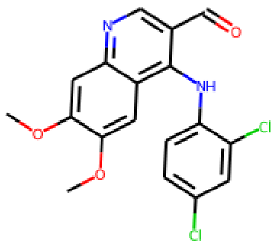	0.576758	COc1cc2ncc(C(=O)O)c(Nc3cccc(Cl)c3Cl)c2cc1OC1C(C)C1	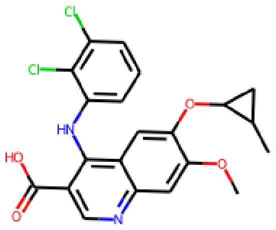	2.0	0.623362	2.525411	2.997881	4	0.774857
**930**	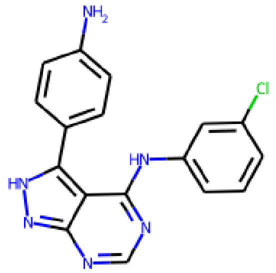	0.576737	c1ccc(Nc2ncnc3n[nH]c(-c4ccccc4)c23)cc1	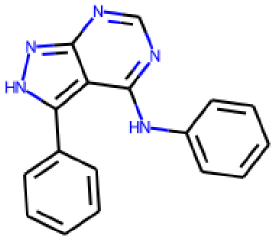	0.0	0.638163	3.995589	2.988471	4	0.800634
**931**	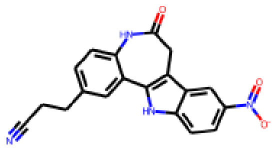	0.576478	CCNC(=O)N1CCC(Nc2n(C(C)c3c(-c4ccccc4)[nH]c4ccccc34)n2)CC1	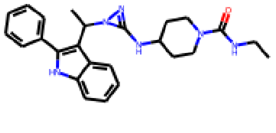	7.0	0.623584	3.603734	4.478009	5	0.804049

	src known mol	similarity	smiles	mol	Class	qed	logP	SAS	rings	kinase inhibition likeliness
**932**	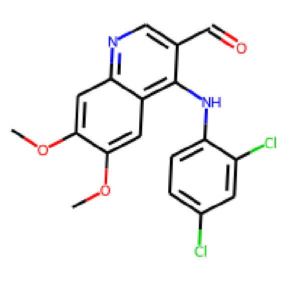	0.576404	COc1cc2ncc(C(=O)O)c(Nc3cccc(Cl)c3Cl)c2cc1OC1C(C)C1	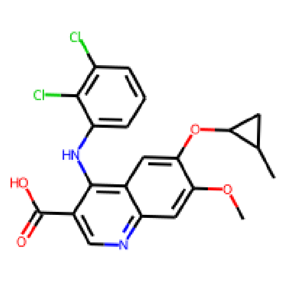	2.0	0.623362	2.525411	2.997881	4	0.774857
**933**	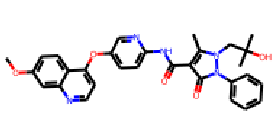	0.576396	COc1ccc2c(=O)cc(CNC(=O)c3c(C)n(C)n(-c4ccccc4)c3=O)nc3cccc2c3c1	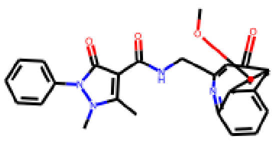	9.0	0.638163	3.995589	2.988471	5	0.774651
**934**	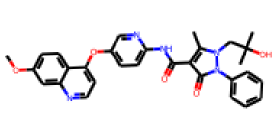	0.576396	COc1ccc2c(=O)cc(CNC(=O)c3c(C)n(C)n(-c4ccccc4)c3=O)nc3cccc2c3c1	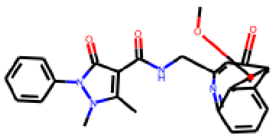	9.0	0.638163	3.995589	2.988471	5	0.774651
**935**	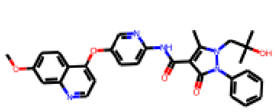	0.576396	COc1ccc2c(=O)cc(CNC(=O)c3c(C)n(C)n(-c4ccccc4)c3=O)nc3cccc2c3c1	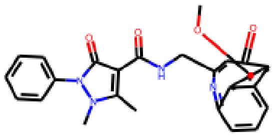	9.0	0.638163	3.995589	2.988471	5	0.774651

	src known mol	similarity	smiles	mol	Class	qed	logP	SAS	rings	kinase inhibition likeliness
**936**	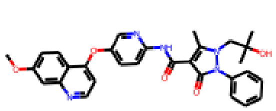	0.576396	COc1ccc2c(=O)cc(CNC(=O)c3c(C)n(C)n(-c4ccccc4)c3=O)nc3cccc2c3c1	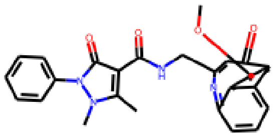	9.0	0.638163	3.995589	2.988471	5	0.774651
**937**	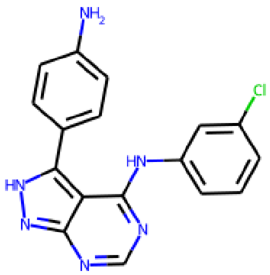	0.576367	c1ccc(Nc2ncnc3n[nH]c(-c4ccccc4)c23)cc1	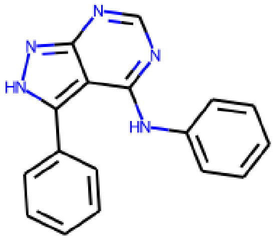	0.0	0.638163	3.995589	2.988471	4	0.800634
**938**	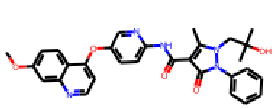	0.576200	COc1ccc2c(=O)cc(CNC(=O)c3c(C)n(C)n(-c4ccccc4)c3=O)nc3cccc2c3c1	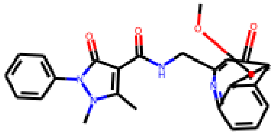	9.0	0.638163	3.995589	2.988471	5	0.774651
**939**	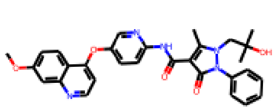	0.576200	COc1ccc2c(=O)cc(CNC(=O)c3c(C)n(C)n(-c4ccccc4)c3=O)nc3cccc2c3c1	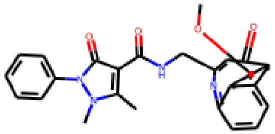	9.0	0.638163	3.995589	2.988471	5	0.774651

	src known mol	similarity	smiles	mol	Class	qed	logP	SAS	rings	kinase inhibition likeliness
**940**	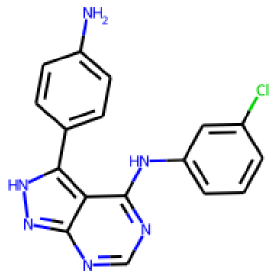	0.576175	c1ccc(Nc2ncnc3n[nH]c(-c4ccccc4)c23)cc1	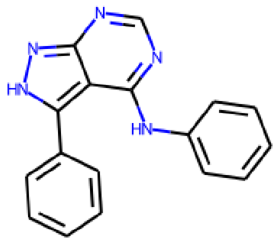	0.0	0.638163	3.995589	2.988471	4	0.800634
**941**	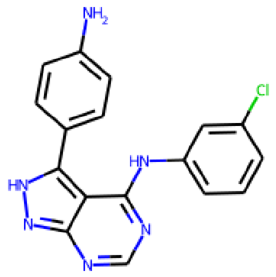	0.576175	c1ccc(Nc2ncnc3n[nH]c(-c4ccccc4)c23)cc1	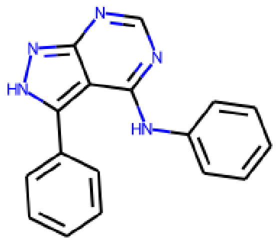	0.0	0.638163	3.995589	2.988471	4	0.800634
**942**	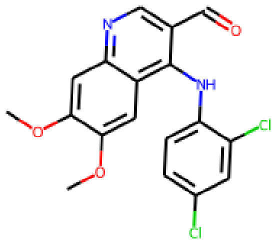	0.576095	COc1cc2ncc(C(=O)O)c(Nc3cccc(Cl)c3Cl)c2cc1OC1C(C)C1	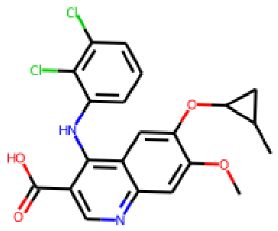	2.0	0.623362	2.525411	2.997881	4	0.774857
**943**	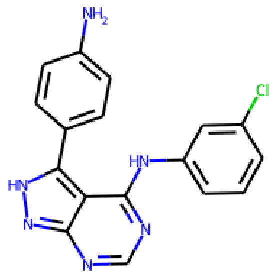	0.575980	c1ccc(Nc2ncnc3n[nH]c(-c4ccccc4)c23)cc1	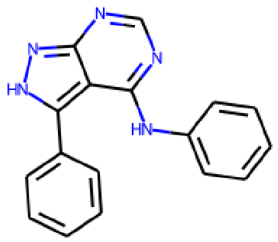	0.0	0.638163	3.995589	2.988471	4	0.800634

	src known mol	similarity	smiles	mol	Class	qed	logP	SAS	rings	kinase inhibition likeliness
**944**	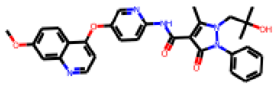	0.575966	COc1ccc2c(=O)cc(CNC(=O)c3c(C)n(C)n(-c4ccccc4)c3=O)nc3cccc2c3c1	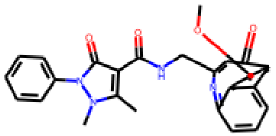	9.0	0.638163	3.995589	2.988471	5	0.774651
**945**	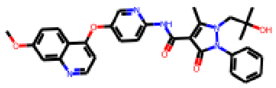	0.575966	COc1ccc2c(=O)cc(CNC(=O)c3c(C)n(C)n(-c4ccccc4)c3=O)nc3cccc2c3c1	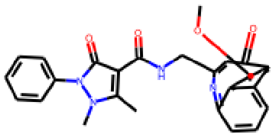	9.0	0.638163	3.995589	2.988471	5	0.774651
**946**	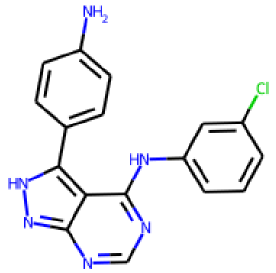	0.575955	c1ccc(Nc2ncnc3n[nH]c(-c4ccccc4)c23)cc1	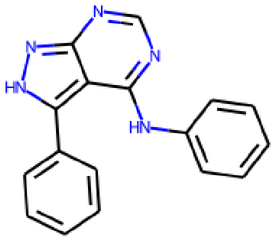	0.0	0.638163	3.995589	2.988471	4	0.800634
**947**	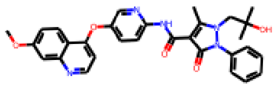	0.575933	COc1ccc2c(=O)cc(CNC(=O)c3c(C)n(C)n(-c4ccccc4)c3=O)nc3cccc2c3c1	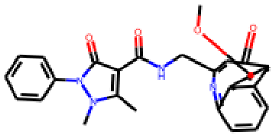	9.0	0.638163	3.995589	2.988471	5	0.774651

	src known mol	similarity	smiles	mol	Class	qed	logP	SAS	rings	kinase inhibition likeliness
**948**	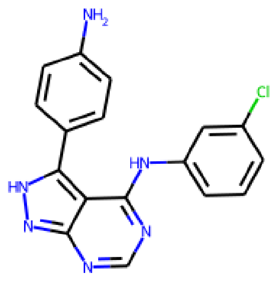	0.575806	c1ccc(Nc2ncnc3n[nH]c(-c4ccccc4)c23)cc1	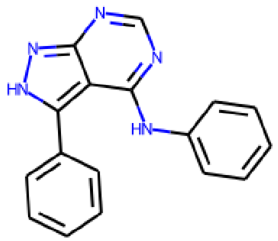	0.0	0.638163	3.995589	2.988471	4	0.800634
**949**	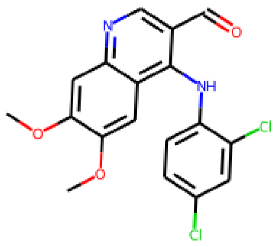	0.575758	COc1cc2ncc(C(=O)O)c(Nc3cccc(Cl)c3Cl)c2cc1OC1C(C)C1	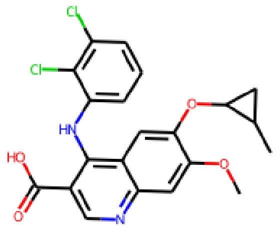	2.0	0.623362	2.525411	2.997881	4	0.774857
**950**	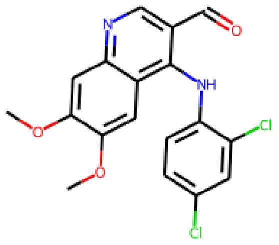	0.575758	COc1cc2ncc(C(=O)O)c(Nc3cccc(Cl)c3Cl)c2cc1OC1C(C)C1	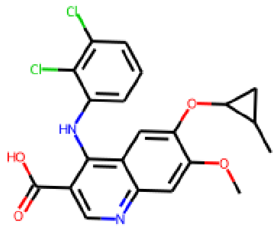	2.0	0.623362	2.525411	2.997881	4	0.774857
**951**	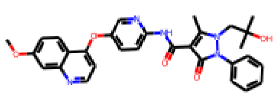	0.575677	COc1ccc2c(=O)cc(CNC(=O)c3c(C)n(C)n(-c4ccccc4)c3=O)nc3cccc2c3c1	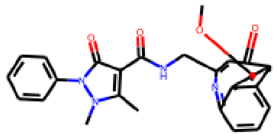	9.0	0.638163	3.995589	2.988471	5	0.774651

	src known mol	similarity	smiles	mol	Class	qed	logP	SAS	rings	kinase inhibition likeliness
**952**	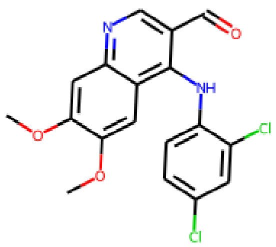	0.575540	COc1cc2ncc(C(=O)O)c(Nc3cccc(Cl)c3Cl)c2cc1OC1C(C)C1	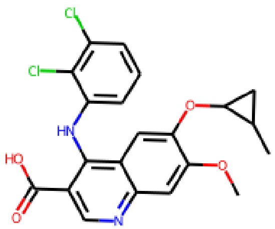	2.0	0.623362	2.525411	2.997881	4	0.774857
**953**	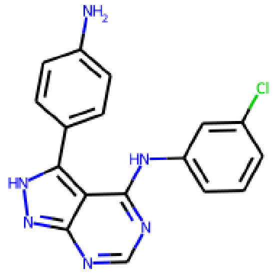	0.575221	c1ccc(Nc2ncnc3n[nH]c(-c4ccccc4)c23)cc1	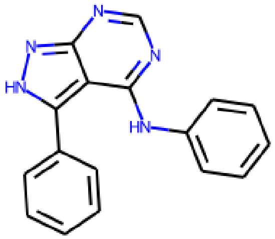	0.0	0.638163	3.995589	2.988471	4	0.800634
**954**	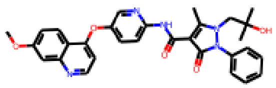	0.575212	COc1ccc2c(=O)cc(CNC(=O)c3c(C)n(C)n(-c4ccccc4)c3=O)nc3cccc2c3c1	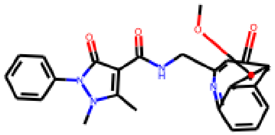	9.0	0.638163	3.995589	2.988471	5	0.774651
**955**	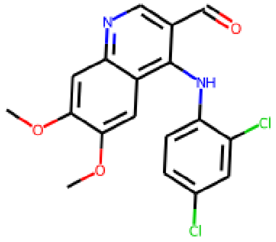	0.575145	COc1cc2ncc(C(=O)O)c(Nc3cccc(Cl)c3Cl)c2cc1OC1C(C)C1	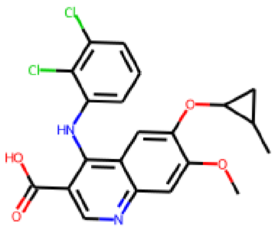	2.0	0.623362	2.525411	2.997881	4	0.774857

	src known mol	similarity	smiles	mol	Class	qed	logP	SAS	rings	kinase inhibition likeliness
**956**	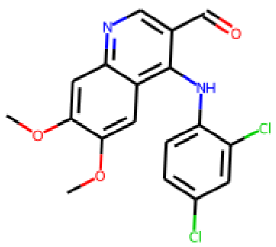	0.575145	COc1cc2ncc(C(=O)O)c(Nc3cccc(Cl)c3Cl)c2cc1OC1C(C)C1	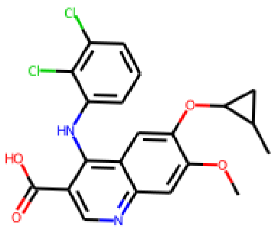	2.0	0.623362	2.525411	2.997881	4	0.774857
**957**	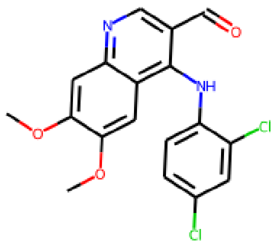	0.575054	COc1cc2ncc(C(=O)O)c(Nc3cccc(Cl)c3Cl)c2cc1OC1C(C)C1	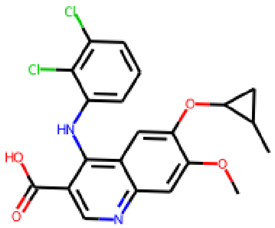	2.0	0.623362	2.525411	2.997881	4	0.774857
**958**	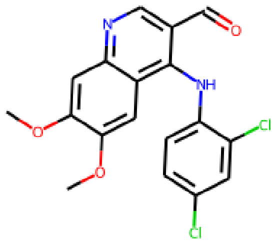	0.575054	COc1cc2ncc(C(=O)O)c(Nc3cccc(Cl)c3Cl)c2cc1OC1C(C)C1	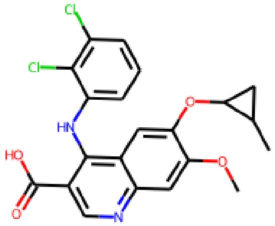	2.0	0.623362	2.525411	2.997881	4	0.774857
**959**	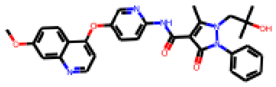	0.575000	COc1ccc2c(=O)cc(CNC(=O)c3c(C)n(C)n(-<br>c4ccccc4)c3=O)nc3cccc2c3c1	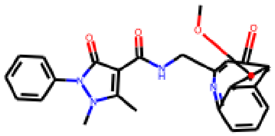	9.0	0.638163	3.995589	2.988471	5	0.774651

	src known mol	similarity	smiles	mol	Class	qed	logP	SAS	rings	kinase inhibition likeliness
**960**	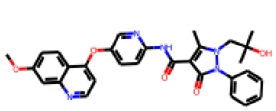	0.575000	COc1ccc2c(=O)cc(CNC(=O)c3c(C)n(C)n(-c4ccccc4)c3=O)nc3cccc2c3c1	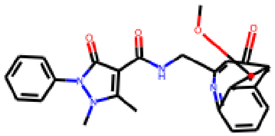	9.0	0.638163	3.995589	2.988471	5	0.774651
**961**	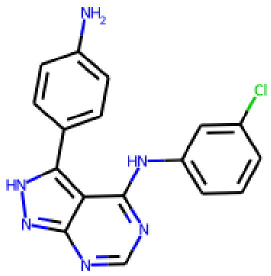	0.574797	c1ccc(Nc2ncnc3n[nH]c(-c4ccccc4)c23)cc1	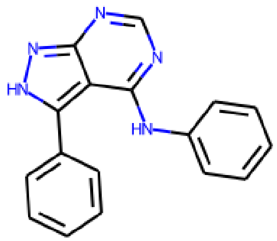	0.0	0.638163	3.995589	2.988471	4	0.800634
**962**	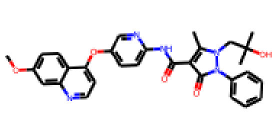	0.574694	COc1ccc2c(=O)cc(CNC(=O)c3c(C)n(C)n(-c4ccccc4)c3=O)nc3cccc2c3c1	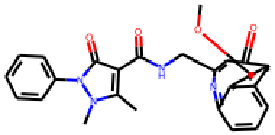	9.0	0.638163	3.995589	2.988471	5	0.774651
**963**	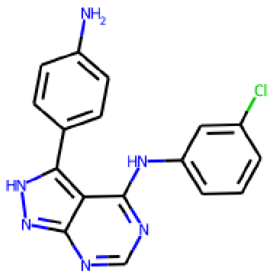	0.574657	c1ccc(Nc2ncnc3n[nH]c(-c4ccccc4)c23)cc1	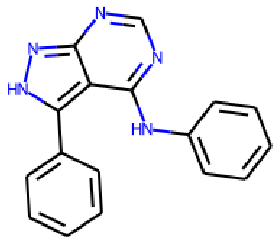	0.0	0.638163	3.995589	2.988471	4	0.800634

	src known mol	similarity	smiles	mol	Class	qed	logP	SAS	rings	kinase inhibition likeliness
**964**	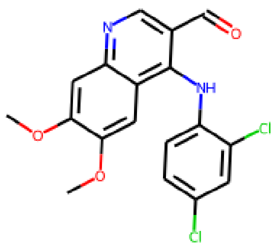	0.574633	COc1cc2ncc(C(=O)O)c(Nc3cccc(Cl)c3Cl)c2cc1OC1C(C)C1	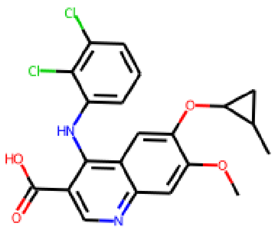	2.0	0.623362	2.525411	2.997881	4	0.774857
**965**	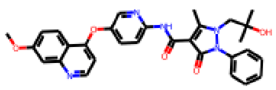	0.574603	COc1ccc2c(=O)cc(CNC(=O)c3c(C)n(C)n(-c4ccccc4)c3=O)nc3cccc2c3c1	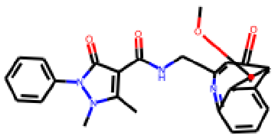	9.0	0.638163	3.995589	2.988471	5	0.774651
**966**	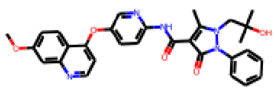	0.574603	COc1ccc2c(=O)cc(CNC(=O)c3c(C)n(C)n(-c4ccccc4)c3=O)nc3cccc2c3c1	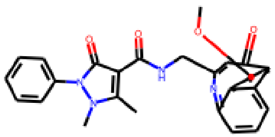	9.0	0.638163	3.995589	2.988471	5	0.774651
**967**	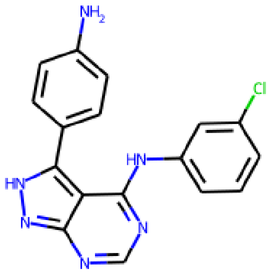	0.574451	c1ccc(Nc2ncnc3n[nH]c(-c4ccccc4)c23)cc1	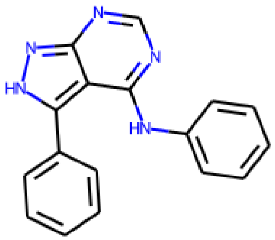	0.0	0.638163	3.995589	2.988471	4	0.800634

	src known mol	similarity	smiles	mol	Class	qed	logP	SAS	rings	kinase inhibition likeliness
**968**	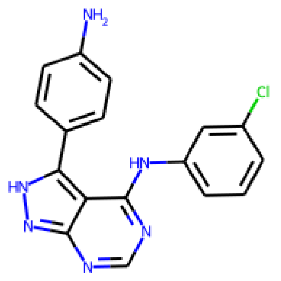	0.574451	c1ccc(Nc2ncnc3n[nH]c(-c4ccccc4)c23)cc1	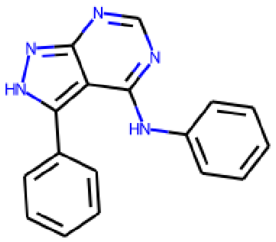	0.0	0.638163	3.995589	2.988471	4	0.800634
**969**	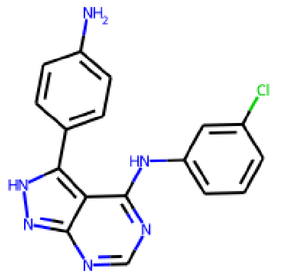	0.574434	c1ccc(Nc2ncnc3n[nH]c(-c4ccccc4)c23)cc1	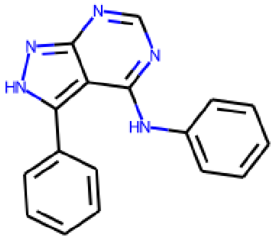	0.0	0.638163	3.995589	2.988471	4	0.800634
**970**	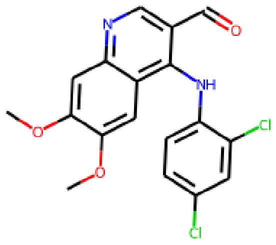	0.574374	COc1cc2ncc(C(=O)O)c(Nc3cccc(Cl)c3Cl)c2cc1OC1C(C)C1	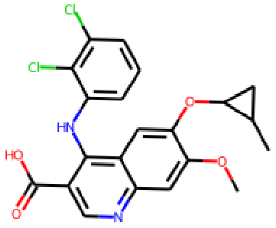	2.0	0.623362	2.525411	2.997881	4	0.774857
**971**	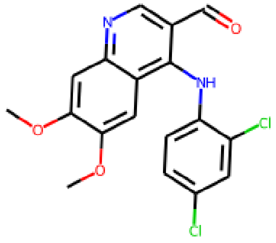	0.574374	COc1cc2ncc(C(=O)O)c(Nc3cccc(Cl)c3Cl)c2cc1OC1C(C)C1	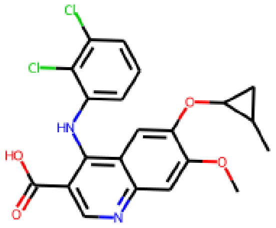	2.0	0.623362	2.525411	2.997881	4	0.774857

	src known mol	similarity	smiles	mol	Class	qed	logP	SAS	rings	kinase inhibition likeliness
**972**	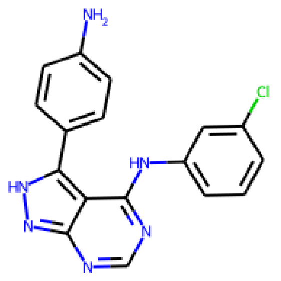	0.574234	c1ccc(Nc2ncnc3n[nH]c(-c4ccccc4)c23)cc1	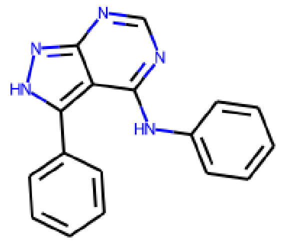	0.0	0.638163	3.995589	2.988471	4	0.800634
**973**	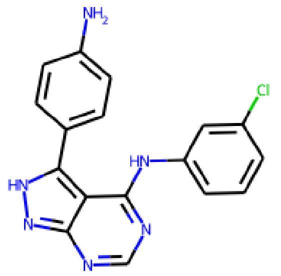	0.574209	c1ccc(Nc2ncnc3n[nH]c(-c4ccccc4)c23)cc1	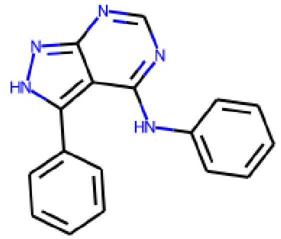	0.0	0.638163	3.995589	2.988471	4	0.800634
**974**	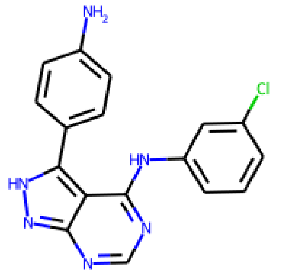	0.574194	c1ccc(Nc2ncnc3n[nH]c(-c4ccccc4)c23)cc1	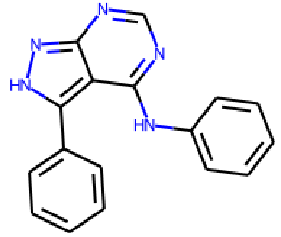	0.0	0.638163	3.995589	2.988471	4	0.800634
**975**	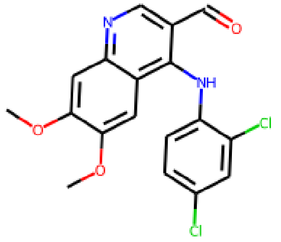	0.574035	COc1cc2ncc(C(=O)O)c(Nc3cccc(Cl)c3Cl)c2cc1OC1C(C)C1	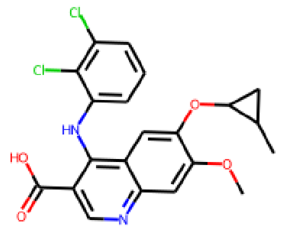	2.0	0.623362	2.525411	2.997881	4	0.774857

	src known mol	similarity	smiles	mol	Class	qed	logP	SAS	rings	kinase inhibition likeliness
**976**	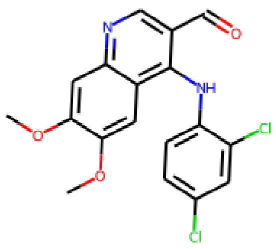	0.574035	COc1cc2ncc(C(=O)O)c(Nc3cccc(Cl)c3Cl)c2cc1OC1C(C)C1	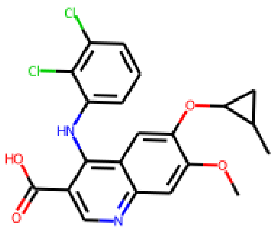	2.0	0.623362	2.525411	2.997881	4	0.774857
**977**	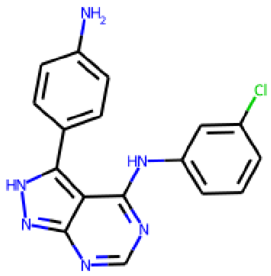	0.573969	c1ccc(Nc2ncnc3n[nH]c(-c4ccccc4)c23)cc1	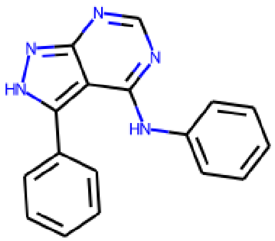	0.0	0.638163	3.995589	2.988471	4	0.800634
**978**	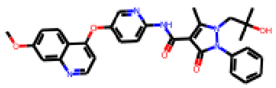	0.573607	COc1ccc2c(=O)cc(CNC(=O)c3c(C)n(C)n(-c4ccccc4)c3=O)nc3cccc2c3c1	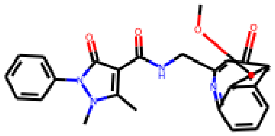	9.0	0.638163	3.995589	2.988471	5	0.774651
**979**	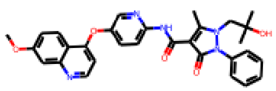	0.573583	COc1ccc2c(=O)cc(CNC(=O)c3c(C)n(C)n(-c4ccccc4)c3=O)nc3cccc2c3c1	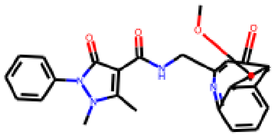	9.0	0.638163	3.995589	2.988471	5	0.774651

	src known mol	similarity	smiles	mol	Class	qed	logP	SAS	rings	kinase inhibition likeliness
**980**	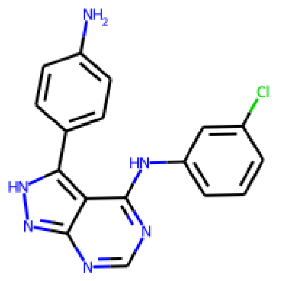	0.573494	c1ccc(Nc2ncnc3n[nH]c(-c4ccccc4)c23)cc1	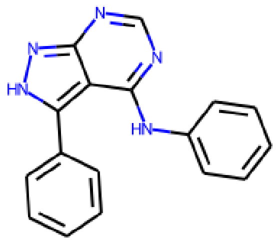	0.0	0.638163	3.995589	2.988471	4	0.800634
**981**	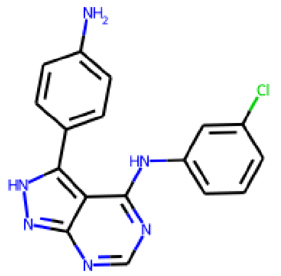	0.573494	c1ccc(Nc2ncnc3n[nH]c(-c4ccccc4)c32)cc1	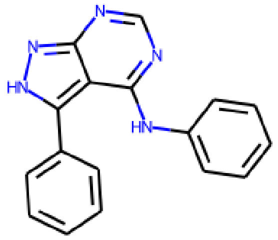	0.0	0.520676	2.336694	2.899742	4	0.831417
**982**	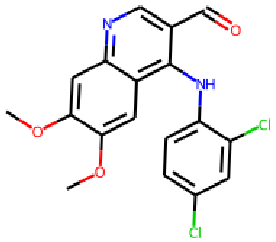	0.573463	COc1cc2ncc(C(=O)O)c(Nc3cccc(Cl)c3Cl)c2cc1OC1C(C)C1	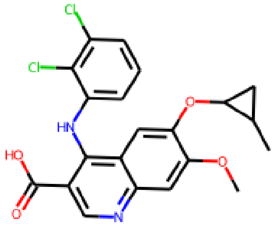	2.0	0.623362	2.525411	2.997881	4	0.774857
**983**	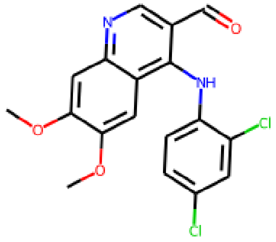	0.573463	COc1cc2ncc(C(=O)O)c(Nc3cccc(Cl)c3Cl)c2cc1OC1C(C)C1	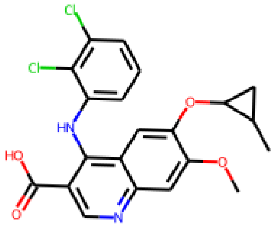	2.0	0.623362	2.525411	2.997881	4	0.774857

	src known mol	similarity	smiles	mol	Class	qed	logP	SAS	rings	kinase inhibition likeliness
**984**	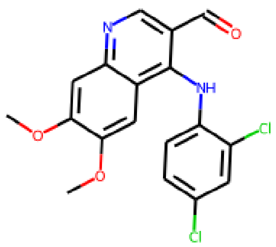	0.573436	COc1cc2ncc(C(=O)O)c(Nc3cccc(Cl)c3Cl)c2cc1OC1C(C)C1	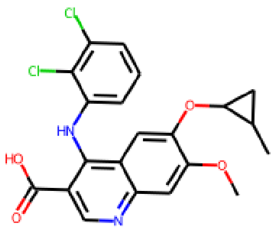	2.0	0.623362	2.525411	2.997881	4	0.774857
**985**	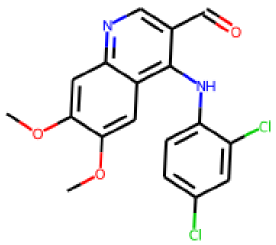	0.573436	COc1cc2ncc(C(=O)O)c(Nc3cccc(Cl)c3Cl)c2cc1OC1C(C)C1	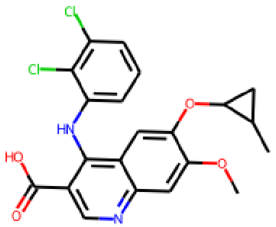	2.0	0.623362	2.525411	2.997881	4	0.774857
**986**	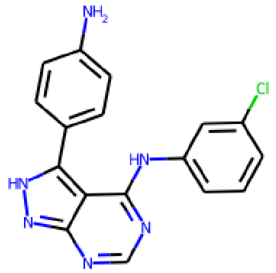	0.573376	c1ccc(Nc2ncnc3n[nH]c(-c4ccccc4)c23)cc1	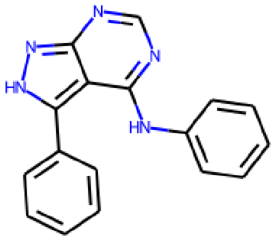	0.0	0.638163	3.995589	2.988471	4	0.800634
**987**	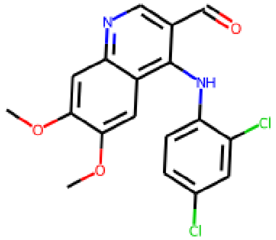	0.572734	COc1cc2ncc(C(=O)O)c(Nc3cccc(Cl)c3Cl)c2cc1OC1C(C)C1	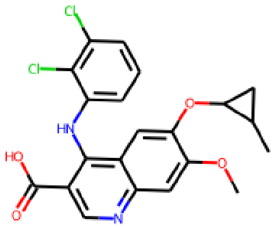	2.0	0.623362	2.525411	2.997881	4	0.774857

	src known mol	similarity	smiles	mol	Class	qed	logP	SAS	rings	kinase inhibition likeliness
**988**	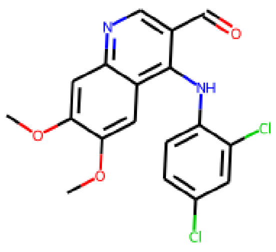	0.572734	COc1cc2ncc(C(=O)O)c(Nc3cccc(Cl)c3Cl)c2cc1OC1C(C)C1	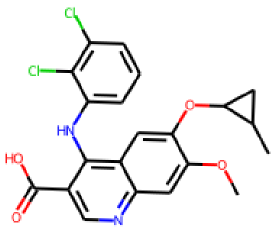	2.0	0.623362	2.525411	2.997881	4	0.774857
**989**	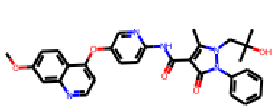	0.572708	COc1ccc2c(=O)cc(CNC(=O)c3c(C)n(C)n(-c4ccccc4)c3=O)nc3cccc2c3c1	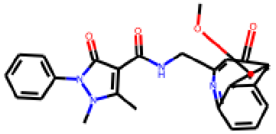	9.0	0.638163	3.995589	2.988471	5	0.774651
**990**	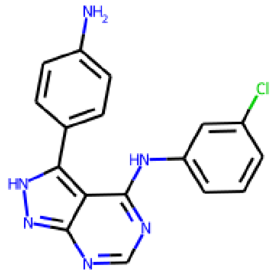	0.572347	c1ccc(Nc2ncnc3n[nH]c(-c4ccccc4)c23)cc1	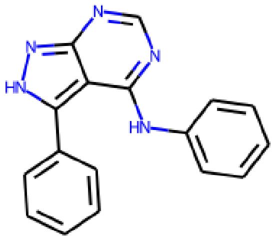	0.0	0.638163	3.995589	2.988471	4	0.800634
**991**	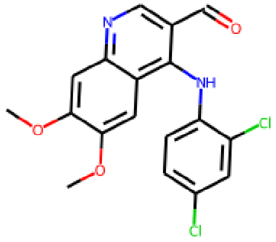	0.572336	COc1cc2ncc(C(=O)O)c(Nc3cccc(Cl)c3Cl)c2cc1OC1C(C)C1	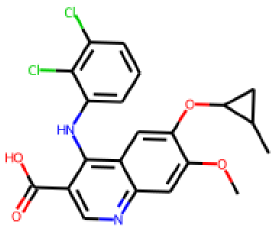	2.0	0.623362	2.525411	2.997881	4	0.774857

	src known mol	similarity	smiles	mol	Class	qed	logP	SAS	rings	kinase inhibition likeliness
**992**	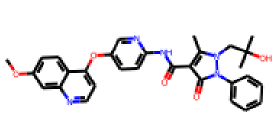	0.572187	COc1ccc2c(=O)cc(CNC(=O)c3c(C)n(C)n(-c4ccccc4)c3=O)nc3cccc2c3c1	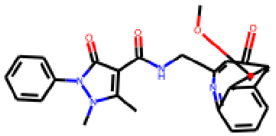	9.0	0.638163	3.995589	2.988471	5	0.774651
**993**	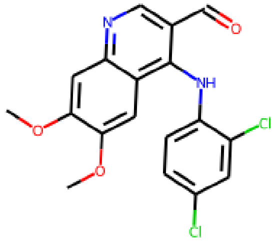	0.572132	COc1cc2ncc(C(=O)O)c(Nc3cccc(Cl)c3Cl)c2cc1OC1C(C)C1	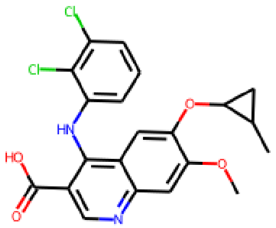	2.0	0.623362	2.525411	2.997881	4	0.774857
**994**	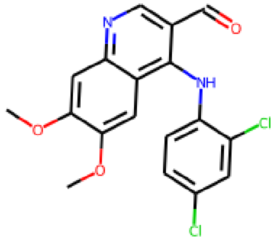	0.571991	COc1cc2ncc(C(=O)O)c(Nc3cccc(Cl)c3Cl)c2cc1OC1C(C)C1	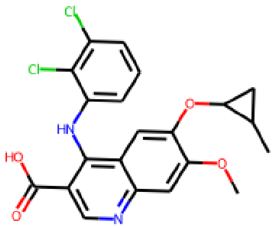	2.0	0.623362	2.525411	2.997881	4	0.774857
**995**	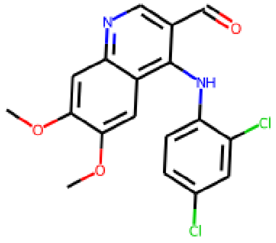	0.571991	COc1cc2ncc(C(=O)O)c(Nc3cccc(Cl)c3Cl)c2cc1OC1C(C)C1	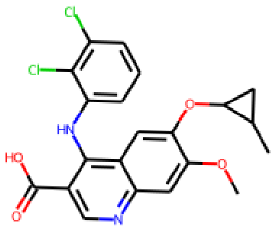	2.0	0.623362	2.525411	2.997881	4	0.774857

	src known mol	similarity	smiles	mol	Class	qed	logP	SAS	rings	kinase inhibition likeliness
**996**	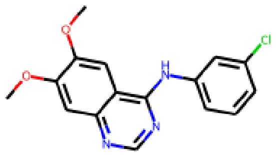	0.571726	Clc1cccc(Nc2ncnc3cc4c[nH]nc4cc23)c1	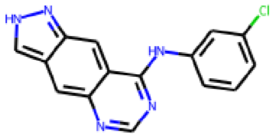	3.0	0.638163	3.995589	2.988471	4	0.781333
**997**	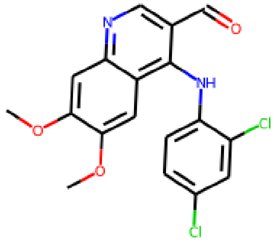	0.571639	COc1cc2ncc(C(=O)O)c(Nc3cccc(Cl)c3Cl)c2cc1OC1C(C)C1	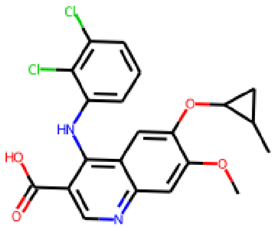	2.0	0.623362	2.525411	2.997881	4	0.774857
**998**	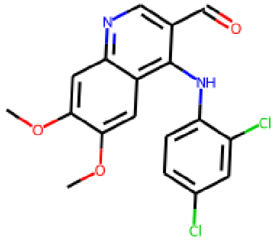	0.571547	COc1cc2ncc(C(=O)O)c(Nc3cccc(Cl)c3Cl)c2cc1OC1C(C)C1	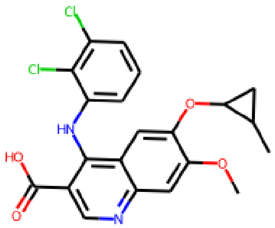	2.0	0.623362	2.525411	2.997881	4	0.774857
**999**	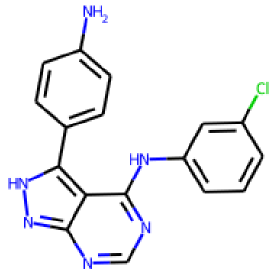	0.571429	c1ccc(Nc2ncnc3n[nH]c(-c4ccccc4)c23)cc1	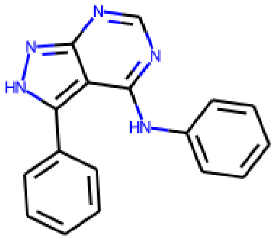	0.0	0.638163	3.995589	2.988471	4	0.800634

	src known mol	similarity	smiles	mol	Class	qed	logP	SAS	rings	kinase inhibition likeliness
**1000**	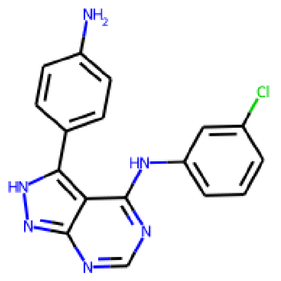	0.571429	c1ccc(Nc2ncnc3n[nH]c(-c4ccccc4)c23)cc1	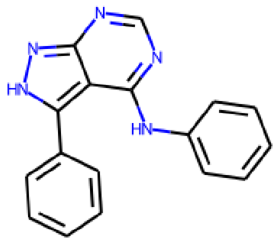	0.0	0.638163	3.995589	2.988471	4	0.800634
**1001**	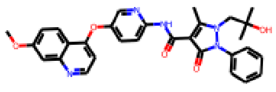	0.571353	COc1ccc2c(=O)cc(CNC(=O)c3c(C)n(C)n(-c4ccccc4)c3=O)nc3cccc2c3c1	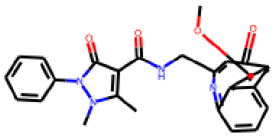	9.0	0.638163	3.995589	2.988471	5	0.774651
**1002**	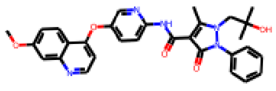	0.571353	COc1ccc2c(=O)cc(CNC(=O)c3c(C)n(C)n(-c4ccccc4)c3=O)nc3cccc2c3c1	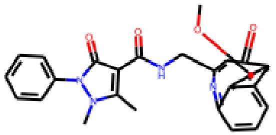	9.0	0.638163	3.995589	2.988471	5	0.774651
**1003**	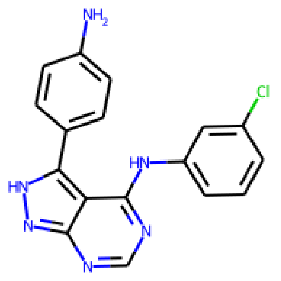	0.571315	c1ccc(Nc2ncnc3n[nH]c(-c4ccccc4)c23)cc1	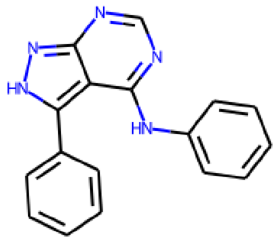	0.0	0.638163	3.995589	2.988471	4	0.800634

	src known mol	similarity	smiles	mol	Class	qed	logP	SAS	rings	kinase inhibition likeliness
**1004**	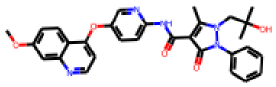	0.571128	COc1ccc2c(=O)cc(CNC(=O)c3c(C)n(C)n(-c4ccccc4)c3=O)nc3cccc2c3c1	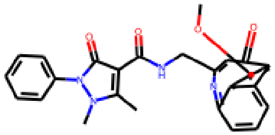	9.0	0.638163	3.995589	2.988471	5	0.774651
**1005**	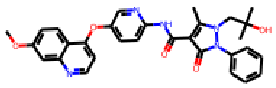	0.571053	COc1ccc2c(=O)cc(CNC(=O)c3c(C)n(C)n(-c4ccccc4)c3=O)nc3cccc2c3c1	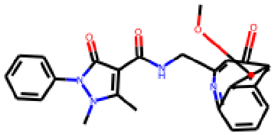	9.0	0.638163	3.995589	2.988471	5	0.774651
**1006**	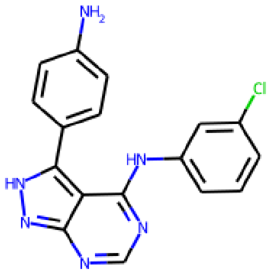	0.570968	c1ccc(Nc2ncnc3n[nH]c(-c4ccccc4)c23)cc1	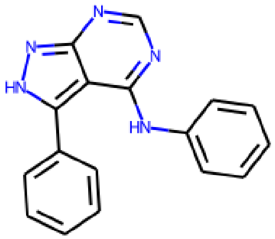	0.0	0.638163	3.995589	2.988471	4	0.800634
**1007**	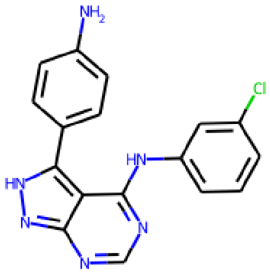	0.570860	c1ccc(Nc2ncnc3n[nH]c(-c4ccccc4)c23)cc1	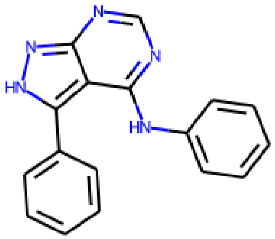	0.0	0.638163	3.995589	2.988471	4	0.800634

	src known mol	similarity	smiles	mol	Class	qed	logP	SAS	rings	kinase inhibition likeliness
**1008**	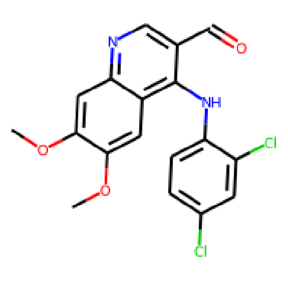	0.570718	COc1cc2ncc(C(=O)O)c(Nc3cccc(Cl)c3Cl)c2cc1OC1C(C)C1	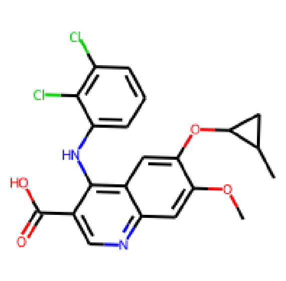	2.0	0.623362	2.525411	2.997881	4	0.774857
**1009**	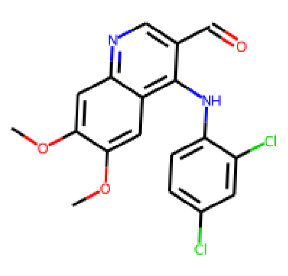	0.570718	COc1cc2ncc(C(=O)O)c(Nc3cccc(Cl)c3Cl)c2cc1OC1C(C)C1	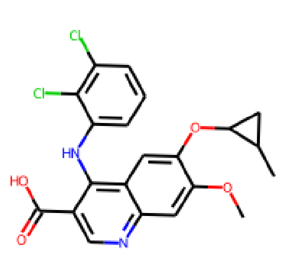	2.0	0.623362	2.525411	2.997881	4	0.774857
**1010**	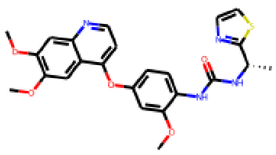	0.570430	COc1ccccc1NC(=O)Nc1ccc(Oc2ccnc3cc(OC)c(OC)cc32)cc1	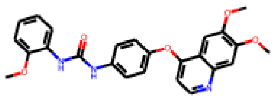	5.0	0.768132	3.608437	2.513170	4	0.790955
**1011**	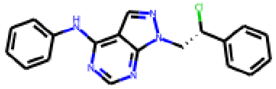	0.570404	Cc1ccc(C(=O)Nc2ccon2)cc1Nc1ncnc2c1cnn2C1cccccc1	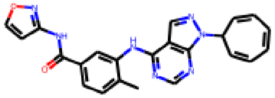	8.0	0.623362	2.525411	2.997881	5	0.770298

	src known mol	similarity	smiles	mol	Class	qed	logP	SAS	rings	kinase inhibition likeliness
**1012**	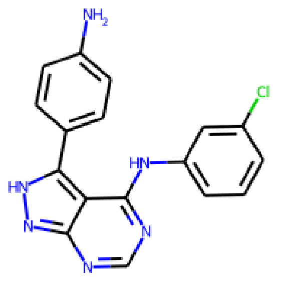	0.570394	c1ccc(Nc2ncnc3n[nH]c(-c4ccccc4)c23)cc1	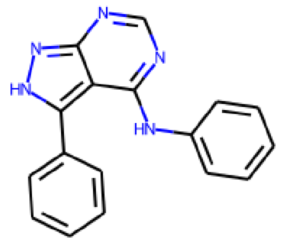	0.0	0.638163	3.995589	2.988471	4	0.800634
**1013**	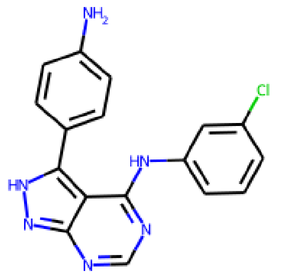	0.570394	c1ccc(Nc2ncnc3n[nH]c(-c4ccccc4)c23)cc1	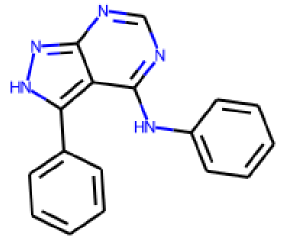	0.0	0.638163	3.995589	2.988471	4	0.800634
**1014**	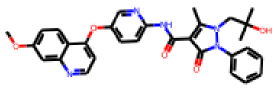	0.570226	COc1ccc2c(=O)cc(CNC(=O)c3c(C)n(C)n(-c4ccccc4)c3=O)nc3cccc2c3c1	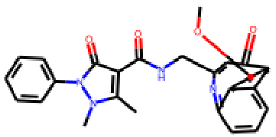	9.0	0.638163	3.995589	2.988471	5	0.774651
**1015**	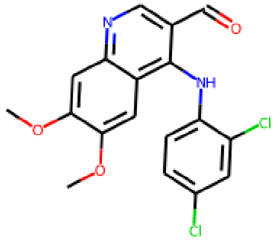	0.570219	COc1cc2ncc(C(=O)O)c(Nc3cccc(Cl)c3Cl)c2cc1OC1C(C)C1	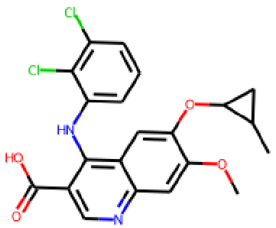	2.0	0.623362	2.525411	2.997881	4	0.774857

	src known mol	similarity	smiles	mol	Class	qed	logP	SAS	rings	kinase inhibition likeliness
**1016**	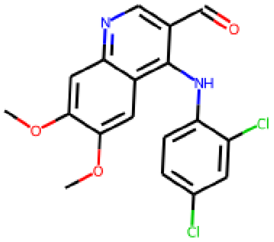	0.570219	COc1cc2ncc(C(=O)O)c(Nc3cccc(Cl)c3Cl)c2cc1OC1C(C)C1	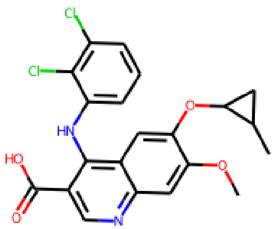	2.0	0.623362	2.525411	2.997881	4	0.774857
**1017**	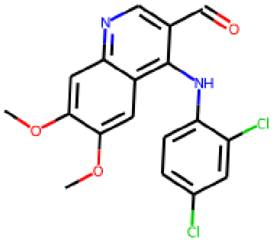	0.570120	COc1cc2ncc(C(=O)O)c(Nc3cccc(Cl)c3Cl)c2cc1OC1C(C)C1	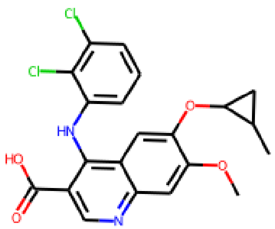	2.0	0.623362	2.525411	2.997881	4	0.774857
**1018**	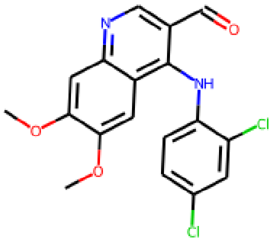	0.570120	COc1cc2ncc(C(=O)O)c(Nc3cccc(Cl)c3Cl)c2cc1OC1C(C)C1	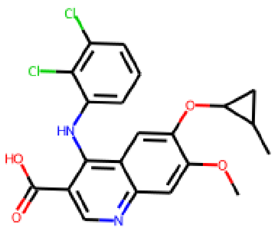	2.0	0.623362	2.525411	2.997881	4	0.774857
**1019**	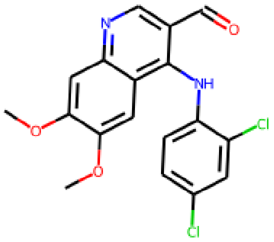	0.570021	COc1cc2ncc(C(=O)O)c(Nc3cccc(Cl)c3Cl)c2cc1OC1C(C)C1	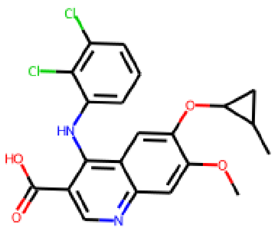	2.0	0.623362	2.525411	2.997881	4	0.774857

	src known mol	similarity	smiles	mol	Class	qed	logP	SAS	rings	kinase inhibition likeliness
**1020**	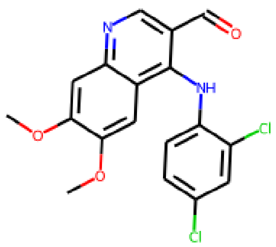	0.570021	COc1cc2ncc(C(=O)O)c(Nc3cccc(Cl)c3Cl)c2cc1OC1C(C)C1	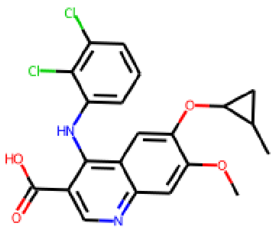	2.0	0.623362	2.525411	2.997881	4	0.774857
**1021**	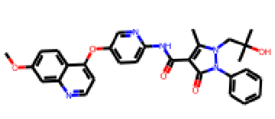	0.569841	COc1ccc2c(=O)cc(CNC(=O)c3c(C)n(C)n(-c4ccccc4)c3=O)nc3cccc2c3c1	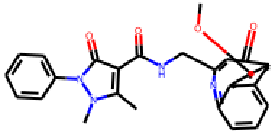	9.0	0.638163	3.995589	2.988471	5	0.774651
**1022**	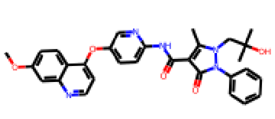	0.569841	COc1ccc2c(=O)cc(CNC(=O)c3c(C)n(C)n(-c4ccccc4)c3=O)nc3cccc2c3c1	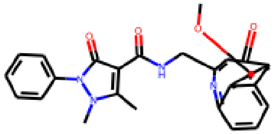	9.0	0.638163	3.995589	2.988471	5	0.774651
**1023**	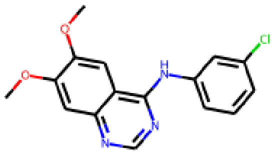	0.569690	Clc1cccc(Nc2ncnc3cc4c[nH]nc4cc23)c1	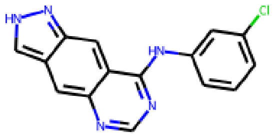	3.0	0.638163	3.995589	2.988471	4	0.781333

	src known mol	similarity	smiles	mol	Class	qed	logP	SAS	rings	kinase inhibition likeliness
**1024**	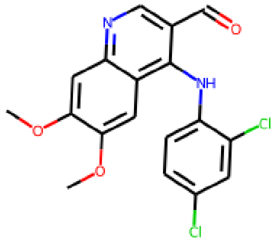	0.569657	COc1cc2ncc(C(=O)O)c(Nc3cccc(Cl)c3Cl)c2cc1OC1C(C)C1	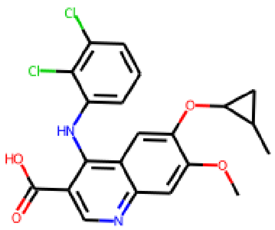	2.0	0.623362	2.525411	2.997881	4	0.774857
**1025**	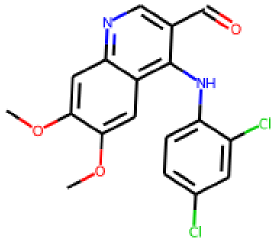	0.569657	COc1cc2ncc(C(=O)O)c(Nc3cccc(Cl)c3Cl)c2cc1OC1C(C)C1	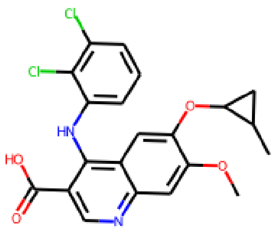	2.0	0.623362	2.525411	2.997881	4	0.774857
**1026**	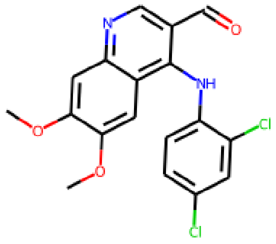	0.569657	COc1cc2ncc(C(=O)O)c(Nc3cccc(Cl)c3Cl)c2cc1OC1C(C)C1	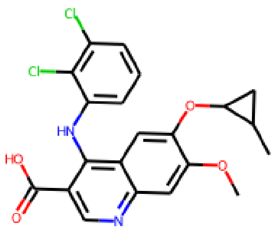	2.0	0.623362	2.525411	2.997881	4	0.774857
**1027**	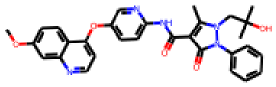	0.569627	COc1ccc2c(=O)cc(CNC(=O)c3c(C)n(C)n(-c4ccccc4)c3=O)nc3cccc2c3c1	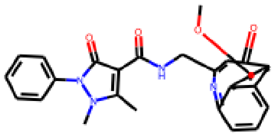	9.0	0.638163	3.995589	2.988471	5	0.774651

	src known mol	similarity	smiles	mol	Class	qed	logP	SAS	rings	kinase inhibition likeliness
**1028**	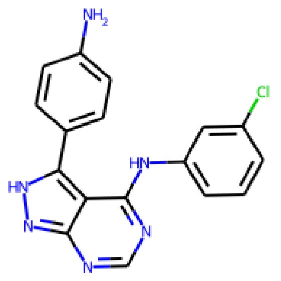	0.569620	c1ccc(Nc2ncnc3n[nH]c(-c4ccccc4)c23)cc1	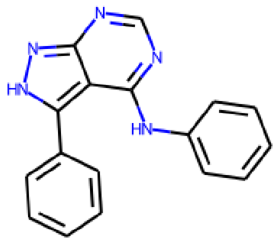	0.0	0.638163	3.995589	2.988471	4	0.800634
**1029**	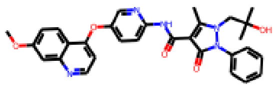	0.569247	COc1ccc2c(=O)cc(CNC(=O)c3c(C)n(C)n(-c4ccccc4)c3=O)nc3cccc2c3c1	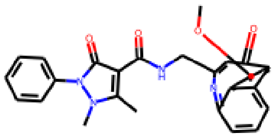	9.0	0.638163	3.995589	2.988471	5	0.774651
**1030**	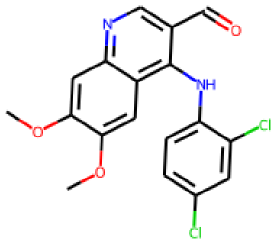	0.569198	COc1cc2ncc(C(=O)O)c(Nc3cccc(Cl)c3Cl)c2cc1OC1C(C)C1	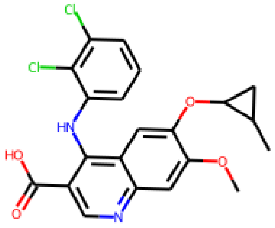	2.0	0.623362	2.525411	2.997881	4	0.774857
**1031**	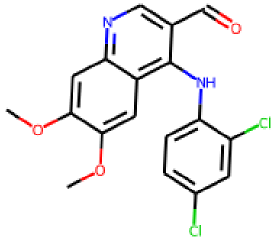	0.569198	COc1cc2ncc(C(=O)O)c(Nc3cccc(Cl)c3Cl)c2cc1OC1C(C)C1	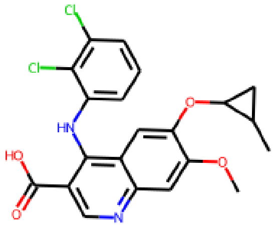	2.0	0.623362	2.525411	2.997881	4	0.774857

	src known mol	similarity	smiles	mol	Class	qed	logP	SAS	rings	kinase inhibition likeliness
**1032**	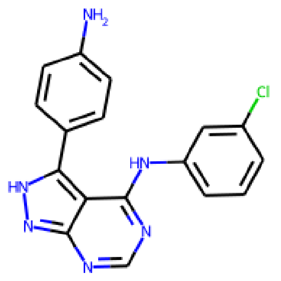	0.569157	c1ccc(Nc2ncnc3n[nH]c(-c4ccccc4)c23)cc1	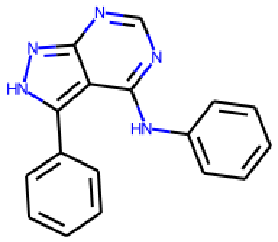	0.0	0.638163	3.995589	2.988471	4	0.800634
**1033**	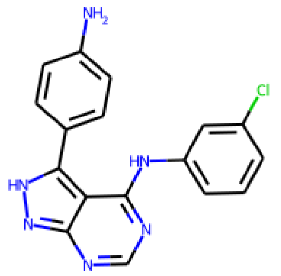	0.569145	c1ccc(Nc2ncnc3n[nH]c(-c4ccccc4)c23)cc1	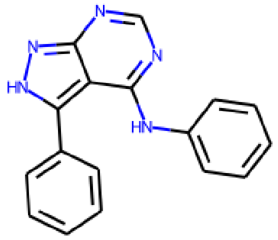	0.0	0.638163	3.995589	2.988471	4	0.800634
**1034**	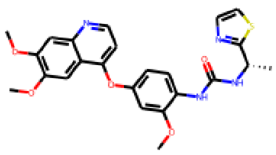	0.569089	COc1ccccc1NC(=O)Nc1ccc(Oc2ccnc3cc(OC)c(OC)cc32)cc1	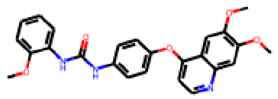	5.0	0.768132	3.608437	2.513170	4	0.790955
**1035**	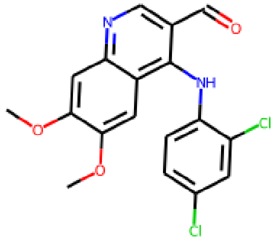	0.569014	COc1cc2ncc(C(=O)O)c(Nc3cccc(Cl)c3Cl)c2cc1OC1C(C)C1	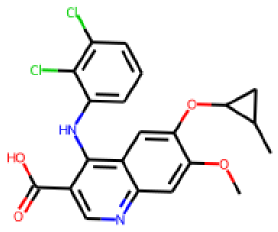	2.0	0.623362	2.525411	2.997881	4	0.774857

	src known mol	similarity	smiles	mol	Class	qed	logP	SAS	rings	kinase inhibition likeliness
**1036**	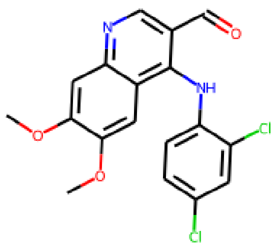	0.569014	COc1cc2ncc(C(=O)O)c(Nc3cccc(Cl)c3Cl)c2cc1OC1C(C)C1	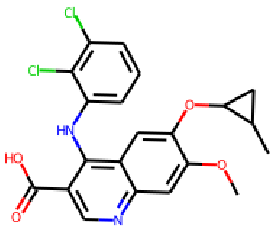	2.0	0.623362	2.525411	2.997881	4	0.774857
**1037**	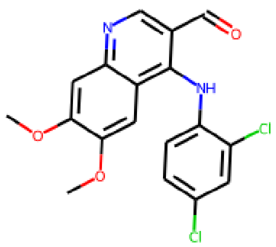	0.569002	COc1cc2ncc(C(=O)O)c(Nc3cccc(Cl)c3Cl)c2cc1OC1C(C)C1	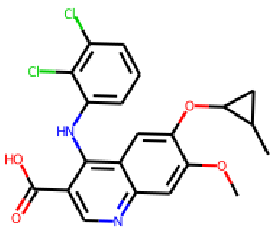	2.0	0.623362	2.525411	2.997881	4	0.774857
**1038**	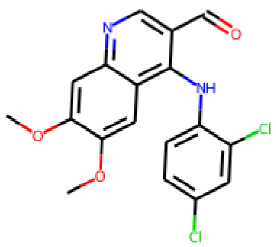	0.569002	COc1cc2ncc(C(=O)O)c(Nc3cccc(Cl)c3Cl)c2cc1OC1C(C)C1	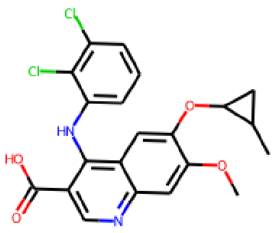	2.0	0.623362	2.525411	2.997881	4	0.774857
**1039**	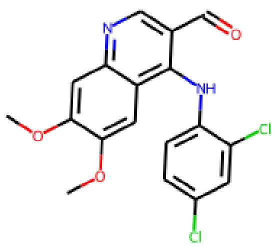	0.568787	COc1cc2ncc(C(=O)O)c(Nc3cccc(Cl)c3Cl)c2cc1OC1C(C)C1	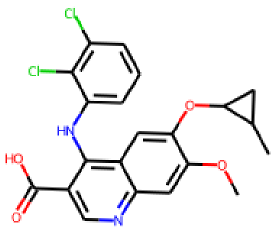	2.0	0.623362	2.525411	2.997881	4	0.774857

	src known mol	similarity	smiles	mol	Class	qed	logP	SAS	rings	kinase inhibition likeliness
**1040**	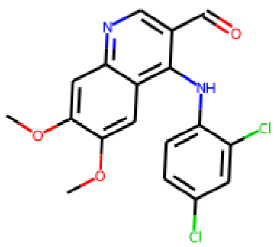	0.568761	COc1cc2ncc(C(=O)O)c(Nc3cccc(Cl)c3Cl)c2cc1OC1C(C)C1	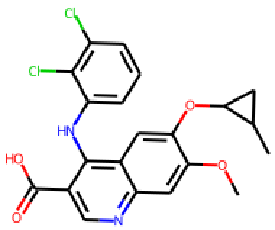	2.0	0.623362	2.525411	2.997881	4	0.774857
**1041**	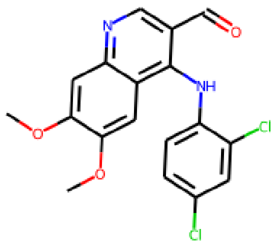	0.568761	COc1cc2ncc(C(=O)O)c(Nc3cccc(Cl)c3Cl)c2cc1OC1C(C)C1	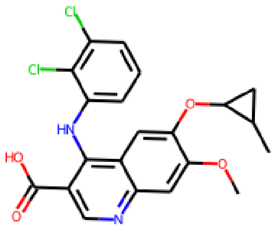	2.0	0.623362	2.525411	2.997881	4	0.774857
**1042**	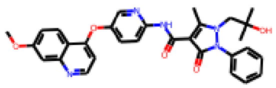	0.568638	COc1ccc2c(=O)cc(CNC(=O)c3c(C)n(C)n(-c4ccccc4)c3=O)nc3cccc2c3c1	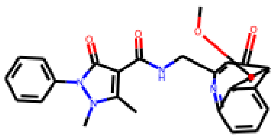	9.0	0.638163	3.995589	2.988471	5	0.774651
**1043**	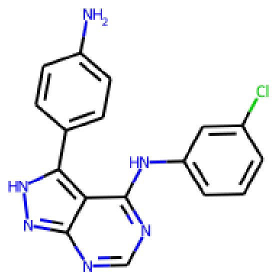	0.568254	c1ccc(Nc2ncnc3n[nH]c(-c4ccccc4)c23)cc1	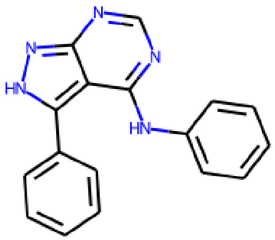	0.0	0.638163	3.995589	2.988471	4	0.800634

	src known mol	similarity	smiles	mol	Class	qed	logP	SAS	rings	kinase inhibition likeliness
**1044**	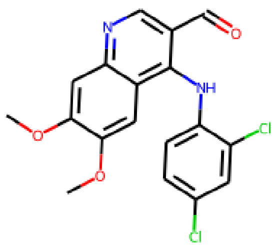	0.568109	COc1cc2ncc(C(=O)O)c(Nc3cccc(Cl)c3Cl)c2cc1OC1C(C)C1	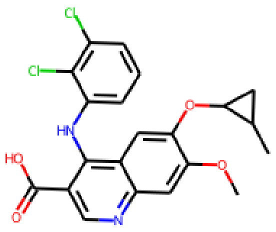	2.0	0.623362	2.525411	2.997881	4	0.774857
**1045**	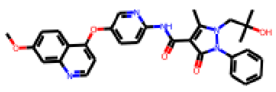	0.567810	COc1ccc2c(=O)cc(CNC(=O)c3c(C)n(C)n(-c4ccccc4)c3=O)nc3cccc2c3c1	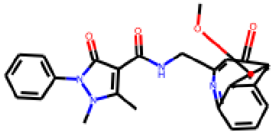	9.0	0.638163	3.995589	2.988471	5	0.774651
**1046**	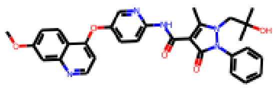	0.567810	COc1ccc2c(=O)cc(CNC(=O)c3c(C)n(C)n(-c4ccccc4)c3=O)nc3cccc2c3c1	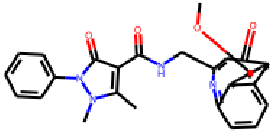	9.0	0.638163	3.995589	2.988471	5	0.774651
**1047**	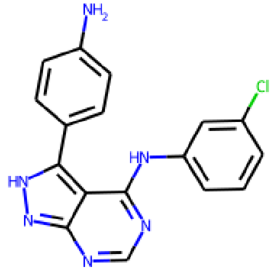	0.567696	c1ccc(Nc2ncnc3n[nH]c(-c4ccccc4)c23)cc1	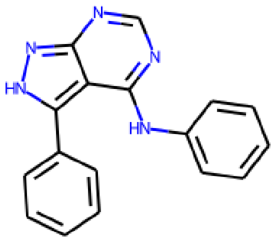	0.0	0.638163	3.995589	2.988471	4	0.800634

	src known mol	similarity	smiles	mol	Class	qed	logP	SAS	rings	kinase inhibition likeliness
**1048**	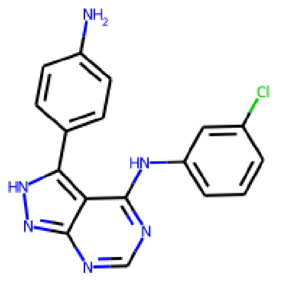	0.567675	c1ccc(Nc2ncnc3n[nH]c(-c4ccccc4)c23)cc1	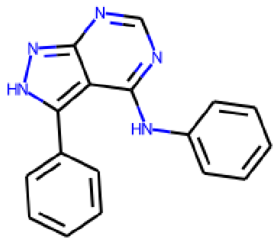	0.0	0.638163	3.995589	2.988471	4	0.800634
**1049**	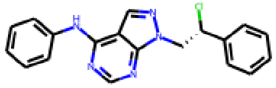	0.567534	Cc1ccc(C(=O)Nc2ccon2)cc1Nc1ncnc2c1cnn2C1cccccc1	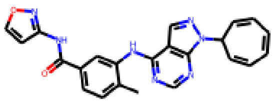	8.0	0.623362	2.525411	2.997881	5	0.770298
**1050**	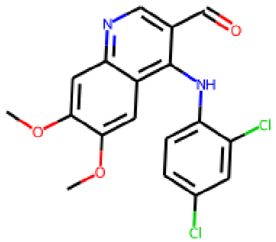	0.567527	COc1cc2ncc(C(=O)O)c(Nc3cccc(Cl)c3Cl)c2cc1OC1C(C)C1	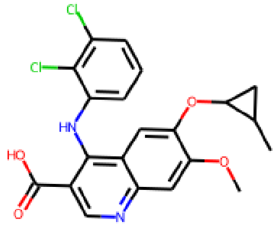	2.0	0.623362	2.525411	2.997881	4	0.774857
**1051**	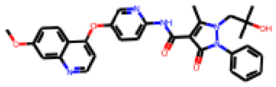	0.567434	COc1ccc2c(=O)cc(CNC(=O)c3c(C)n(C)n(-c4ccccc4)c3=O)nc3cccc3c2n1	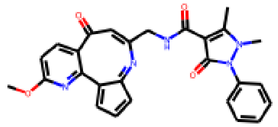	9.0	0.768132	3.608437	2.513170	5	0.823048

	src known mol	similarity	smiles	mol	Class	qed	logP	SAS	rings	kinase inhibition likeliness
**1052**	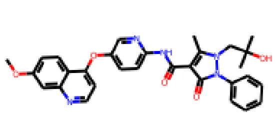	0.567353	COc1ccc2c(=O)cc(CNC(=O)c3c(C)n(C)n(-c4ccccc4)c3=O)nc3cccc2c3c1	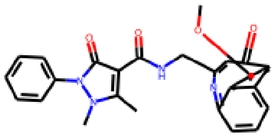	9.0	0.638163	3.995589	2.988471	5	0.774651
**1053**	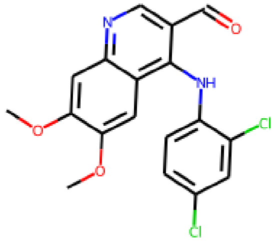	0.567185	COc1cc2ncc(C(=O)O)c(Nc3cccc(Cl)c3Cl)c2cc1OC1C(C)C1	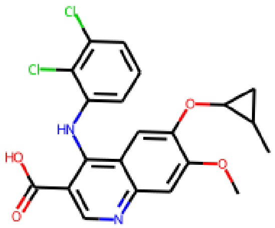	2.0	0.623362	2.525411	2.997881	4	0.774857
**1054**	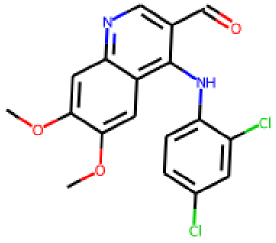	0.567185	COc1cc2ncc(C(=O)O)c(Nc3cccc(Cl)c3Cl)c2cc1OC1C(C)C1	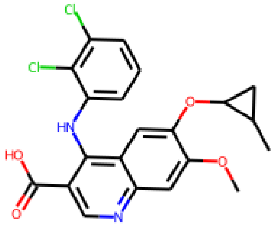	2.0	0.623362	2.525411	2.997881	4	0.774857
**1055**	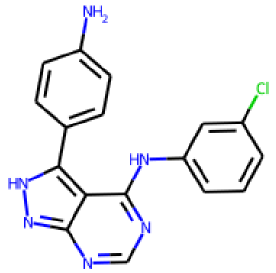	0.567068	c1ccc(Nc2ncnc3n[nH]c(-c4ccccc4)c23)cc1	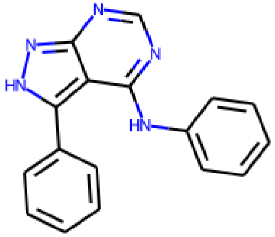	0.0	0.638163	3.995589	2.988471	4	0.800634

	src known mol	similarity	smiles	mol	Class	qed	logP	SAS	rings	kinase inhibition likeliness
**1056**	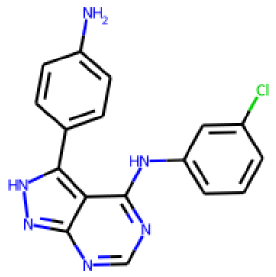	0.567068	c1ccc(Nc2ncnc3n[nH]c(-c4ccccc4)c23)cc1	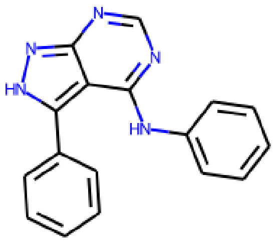	0.0	0.638163	3.995589	2.988471	4	0.800634
**1057**	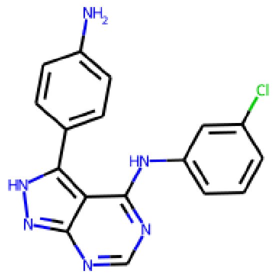	0.567068	c1ccc(Nc2ncnc3n[nH]c(-c4ccccc4)c23)cc1	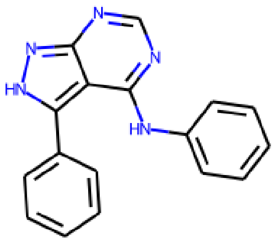	0.0	0.638163	3.995589	2.988471	4	0.800634
**1058**	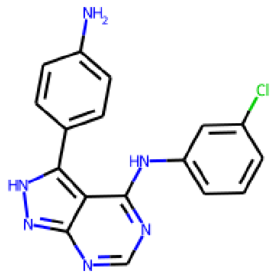	0.566904	c1ccc(Nc2ncnc3n[nH]c(-c4ccccc4)c23)cc1	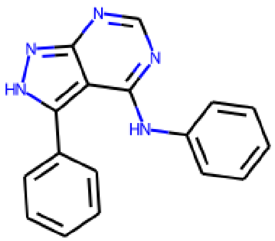	0.0	0.638163	3.995589	2.988471	4	0.800634
**1059**	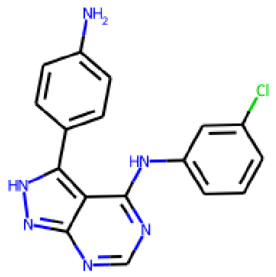	0.566904	c1ccc(Nc2ncnc3n[nH]c(-c4ccccc4)c23)cc1	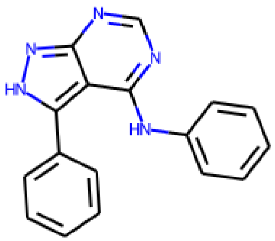	0.0	0.638163	3.995589	2.988471	4	0.800634

	src known mol	similarity	smiles	mol	Class	qed	logP	SAS	rings	kinase inhibition likeliness
**1060**	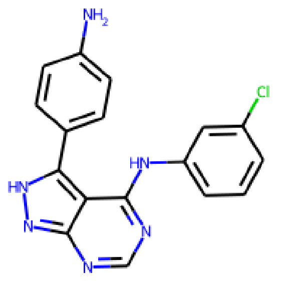	0.566904	c1ccc(Nc2ncnc3n[nH]c(-c4ccccc4)c23)cc1	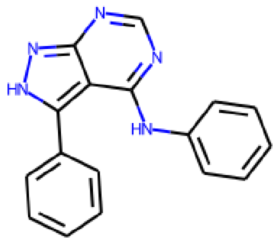	0.0	0.638163	3.995589	2.988471	4	0.800634
**1061**	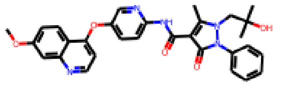	0.566720	COc1ccc2c(=O)cc(CNC(=O)c3c(C)n(C)n(-c4ccccc4)c3=O)nc3cccc2c3c1	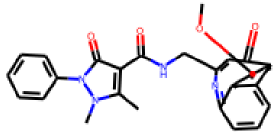	9.0	0.638163	3.995589	2.988471	5	0.774651
**1062**	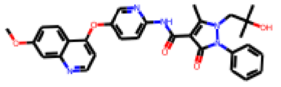	0.566720	COc1ccc2c(=O)cc(CNC(=O)c3c(C)n(C)n(-c4ccccc4)c3=O)nc3cccc2c3c1	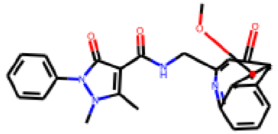	9.0	0.638163	3.995589	2.988471	5	0.774651
**1063**	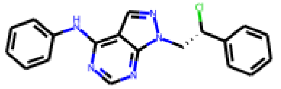	0.566711	Cc1ccc(C(=O)Nc2ccon2)cc1Nc1ncnc2c1cnn2C1cccccc1	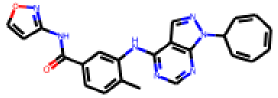	8.0	0.623362	2.525411	2.997881	5	0.770298

	src known mol	similarity	smiles	mol	Class	qed	logP	SAS	rings	kinase inhibition likeliness
**1064**	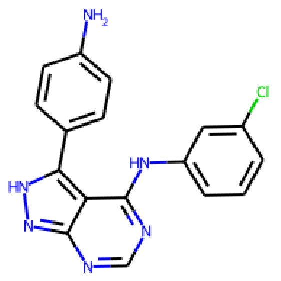	0.566667	c1ccc(Nc2ncnc3n[nH]c(-c4ccccc4)c23)cc1	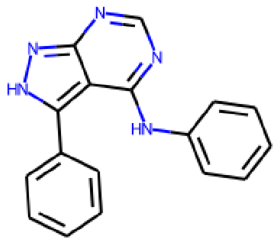	0.0	0.638163	3.995589	2.988471	4	0.800634
**1065**	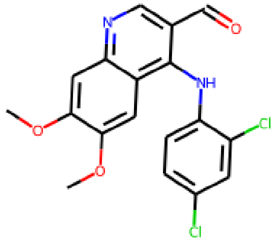	0.566618	COc1cc2ncc(C(=O)O)c(Nc3cccc(Cl)c3Cl)c2cc1OC1C(C)C1	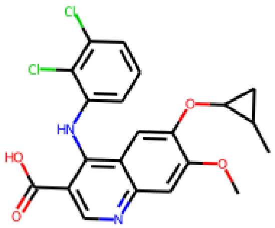	2.0	0.623362	2.525411	2.997881	4	0.774857
**1066**	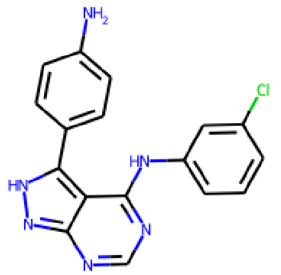	0.566588	c1ccc(Nc2ncnc3n[nH]c(-c4ccccc4)c23)cc1	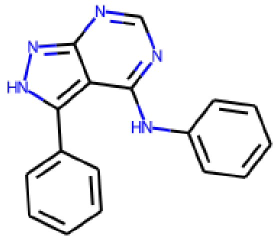	0.0	0.638163	3.995589	2.988471	4	0.800634
**1067**	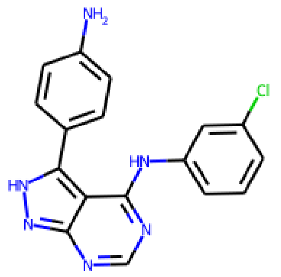	0.566351	c1ccc(Nc2ncnc3n[nH]c(-c4ccccc4)c23)cc1	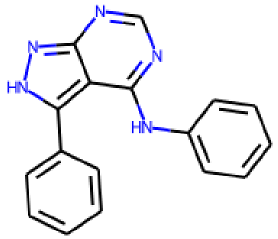	0.0	0.638163	3.995589	2.988471	4	0.800634

	src known mol	similarity	smiles	mol	Class	qed	logP	SAS	rings	kinase inhibition likeliness
**1068**	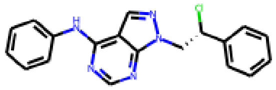	0.566317	Cc1ccc(C(=O)Nc2ccon2)cc1Nc1ncnc2c1cnn2C1cccccc1	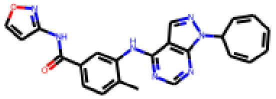	8.0	0.623362	2.525411	2.997881	5	0.770298
**1069**	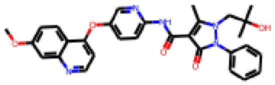	0.566297	COc1ccc2c(=O)cc(CNC(=O)c3c(C)n(C)n(-c4ccccc4)c3=O)nc3cccc2c3c1	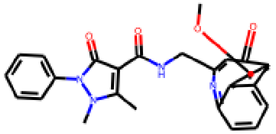	9.0	0.638163	3.995589	2.988471	5	0.774651
**1070**	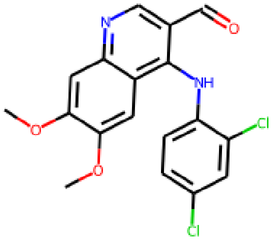	0.566203	COc1cc2ncc(C(=O)O)c(Nc3cccc(Cl)c3Cl)c2cc1OC1C(C)C1	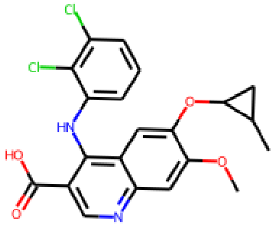	2.0	0.623362	2.525411	2.997881	4	0.774857
**1071**	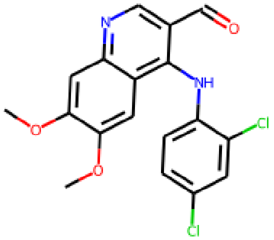	0.566171	COc1cc2ncc(C(=O)O)c(Nc3cccc(Cl)c3Cl)c2cc1OC1C(C)C1	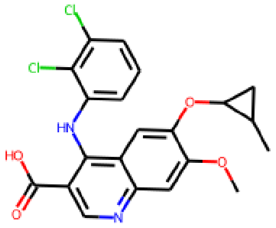	2.0	0.623362	2.525411	2.997881	4	0.774857

	src known mol	similarity	smiles	mol	Class	qed	logP	SAS	rings	kinase inhibition likeliness
**1072**	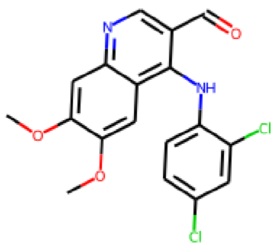	0.566104	COc1cc2ncc(C(=O)O)c(Nc3cccc(Cl)c3Cl)c2cc1OC1C(C)C1	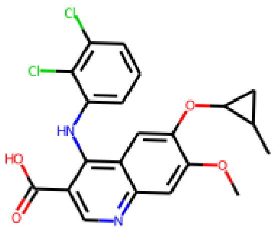	2.0	0.623362	2.525411	2.997881	4	0.774857
**1073**	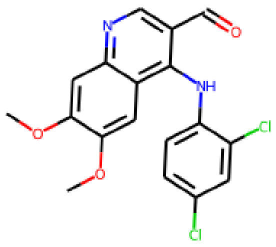	0.566104	COc1cc2ncc(C(=O)O)c(Nc3cccc(Cl)c3Cl)c2cc1OC1C(C)C1	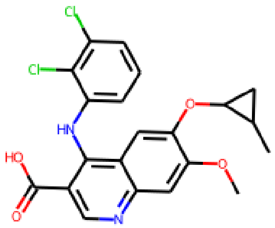	2.0	0.623362	2.525411	2.997881	4	0.774857
**1074**	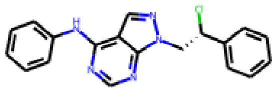	0.565689	Cc1ccc(C(=O)Nc2ccon2)cc1Nc1ncnc2c1cnn2C1cccccc1	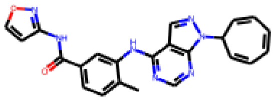	8.0	0.623362	2.525411	2.997881	5	0.770298
**1075**	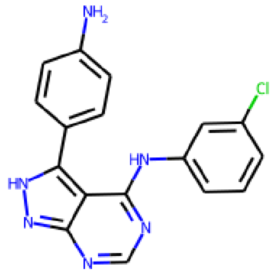	0.565561	c1ccc(Nc2ncnc3n[nH]c(-c4ccccc4)c32)cc1	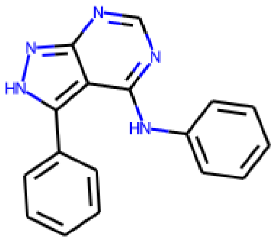	0.0	0.520676	2.336694	2.899742	4	0.831417

	src known mol	similarity	smiles	mol	Class	qed	logP	SAS	rings	kinase inhibition likeliness
**1076**	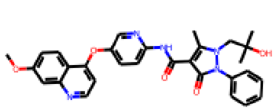	0.565401	COc1ccc2c(=O)cc(CNC(=O)c3c(C)n(C)n(-c4ccccc4)c3=O)nc3cccc2c3c1	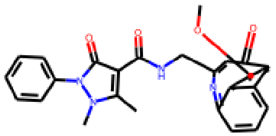	9.0	0.638163	3.995589	2.988471	5	0.774651
**1077**	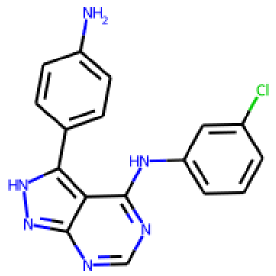	0.565388	c1ccc(Nc2ncnc3n[nH]c(-c4ccccc4)c23)cc1	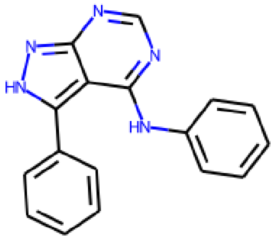	0.0	0.638163	3.995589	2.988471	4	0.800634
**1078**	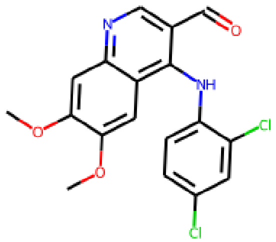	0.565249	COc1cc2ncc(C(=O)O)c(Nc3cccc(Cl)c3Cl)c2cc1OC1C(C)C1	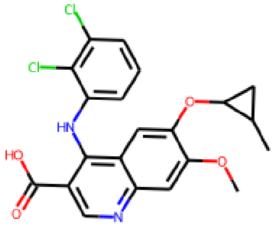	2.0	0.623362	2.525411	2.997881	4	0.774857
**1079**	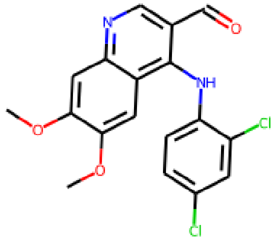	0.565249	COc1cc2ncc(C(=O)O)c(Nc3cccc(Cl)c3Cl)c2cc1OC1C(C)C1	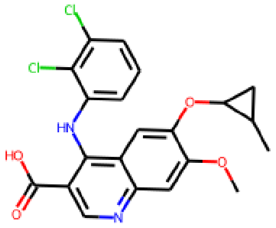	2.0	0.623362	2.525411	2.997881	4	0.774857

	src known mol	similarity	smiles	mol	Class	qed	logP	SAS	rings	kinase inhibition likeliness
**1080**	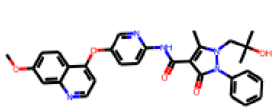	0.564894	COc1ccc2c(=O)cc(CNC(=O)c3c(C)n(C)n(-c4ccccc4)c3=O)nc3cccc2c3c1	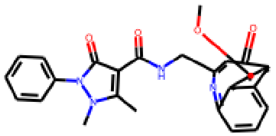	9.0	0.638163	3.995589	2.988471	5	0.774651
**1081**	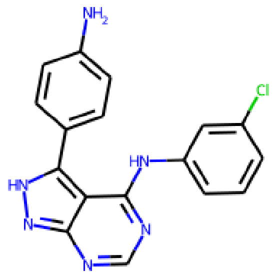	0.564850	c1ccc(Nc2ncnc3n[nH]c(-c4ccccc4)c23)cc1	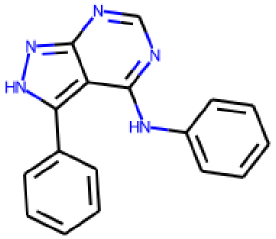	0.0	0.638163	3.995589	2.988471	4	0.800634
**1082**	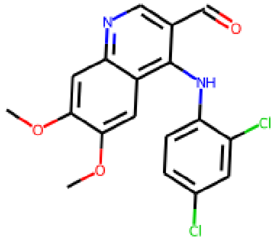	0.564680	COc1cc2ncc(C(=O)O)c(Nc3cccc(Cl)c3Cl)c2cc1OC1C(C)C1	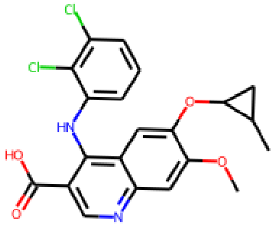	2.0	0.623362	2.525411	2.997881	4	0.774857
**1083**	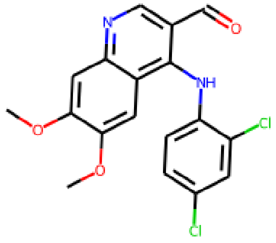	0.564680	COc1cc2ncc(C(=O)O)c(Nc3cccc(Cl)c3Cl)c2cc1OC1C(C)C1	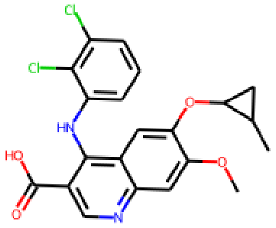	2.0	0.623362	2.525411	2.997881	4	0.774857

	src known mol	similarity	smiles	mol	Class	qed	logP	SAS	rings	kinase inhibition likeliness
**1084**	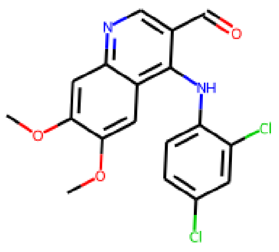	0.564680	COc1cc2ncc(C(=O)O)c(Nc3cccc(Cl)c3Cl)c2cc1OC1C(C)C1	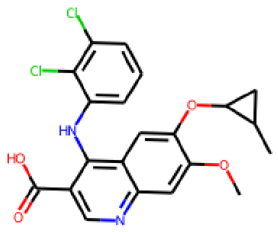	2.0	0.623362	2.525411	2.997881	4	0.774857
**1085**	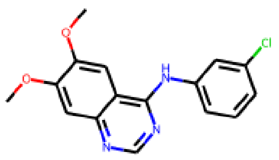	0.564641	Clc1cccc(Nc2ncnc3cc4c[nH]nc4cc23)c1	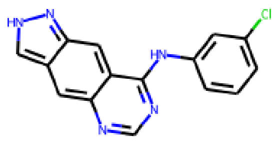	3.0	0.638163	3.995589	2.988471	4	0.781333
**1086**	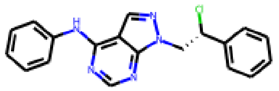	0.564401	Cc1ccc(C(=O)Nc2ccon2)cc1Nc1ncnc2c1cnn2C1cccccc1	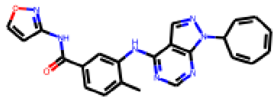	8.0	0.623362	2.525411	2.997881	5	0.770298
**1087**	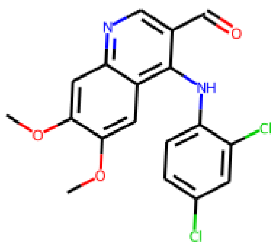	0.564327	COc1cc2ncc(C(=O)O)c(Nc3cccc(Cl)c3Cl)c2cc1OC1C(C)C1	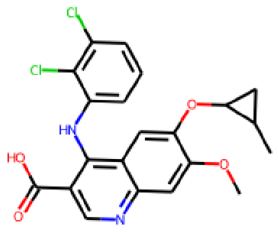	2.0	0.623362	2.525411	2.997881	4	0.774857

	src known mol	similarity	smiles	mol	Class	qed	logP	SAS	rings	kinase inhibition likeliness
**1088**	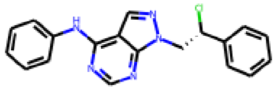	0.564272	Cc1ccc(C(=O)Nc2ccon2)cc1Nc1ncnc2c1cnn2C1cccccc1	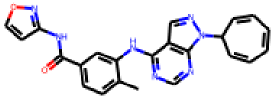	8.0	0.623362	2.525411	2.997881	5	0.770298
**1089**	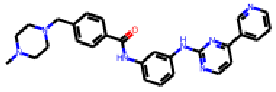	0.564129	Fc1ccc(NC(=O)c2ccccc2)cc1Nc1nccc(-c2cccnc2)n1	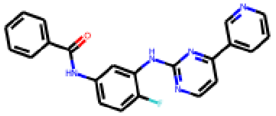	0.0	0.632663	4.308233	4.065316	4	0.777346
**1090**	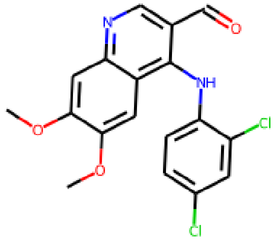	0.563934	COc1cc2ncc(C(=O)O)c(Nc3cccc(Cl)c3Cl)c2cc1OC1C(C)C1	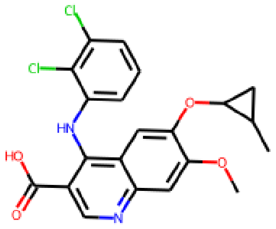	2.0	0.623362	2.525411	2.997881	4	0.774857
**1091**	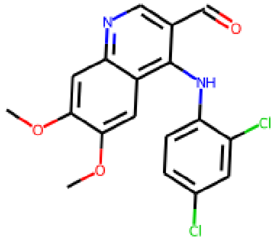	0.563899	COc1cc2ncc(C(=O)O)c(Nc3cccc(Cl)c3Cl)c2cc1OC1C(C)C1	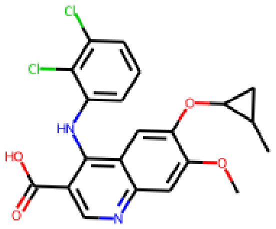	2.0	0.623362	2.525411	2.997881	4	0.774857

	src known mol	similarity	smiles	mol	Class	qed	logP	SAS	rings	kinase inhibition likeliness
**1092**	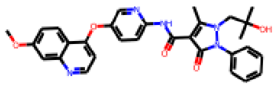	0.563898	COc1ccc2c(=O)cc(CNC(=O)c3c(C)n(C)n(-c4ccccc4)c3=O)nc3cccc2c3c1	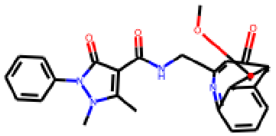	9.0	0.638163	3.995589	2.988471	5	0.774651
**1093**	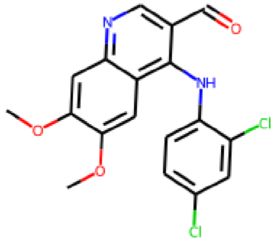	0.563768	COc1cc2ncc(C(=O)O)c(Nc3cccc(Cl)c3Cl)c2cc1OC1C(C)C1	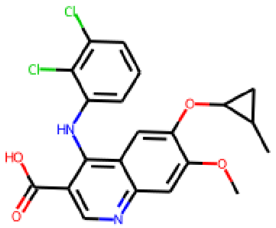	2.0	0.623362	2.525411	2.997881	4	0.774857
**1094**	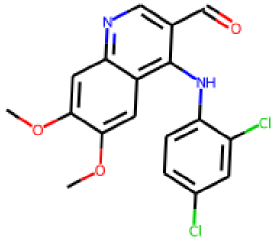	0.563768	COc1cc2ncc(C(=O)O)c(Nc3cccc(Cl)c3Cl)c2cc1OC1C(C)C1	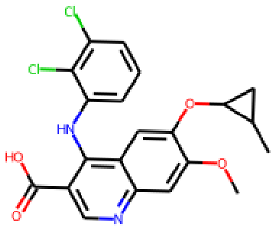	2.0	0.623362	2.525411	2.997881	4	0.774857
**1095**	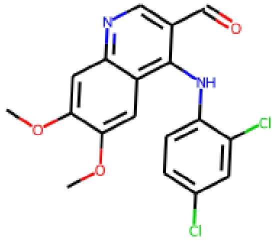	0.563768	COc1cc2ncc(C(=O)O)c(Nc3cccc(Cl)c3Cl)c2cc1OC1C(C)C1	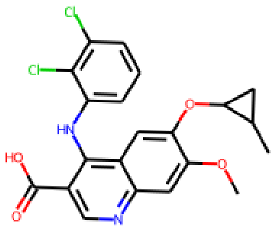	2.0	0.623362	2.525411	2.997881	4	0.774857

	src known mol	similarity	smiles	mol	Class	qed	logP	SAS	rings	kinase inhibition likeliness
**1096**	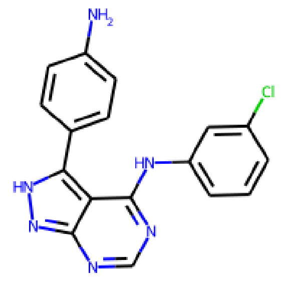	0.563737	c1ccc(Nc2ncnc3n[nH]c(-c4ccccc4)c23)cc1	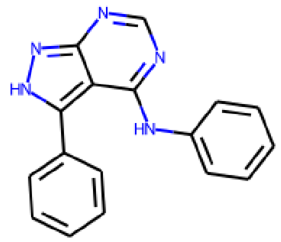	0.0	0.638163	3.995589	2.988471	4	0.800634
**1097**	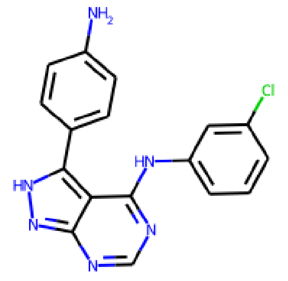	0.563737	c1ccc(Nc2ncnc3n[nH]c(-c4ccccc4)c23)cc1	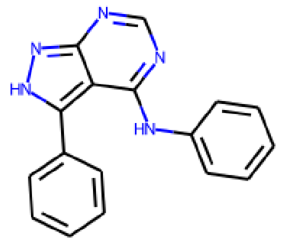	0.0	0.638163	3.995589	2.988471	4	0.800634
**1098**	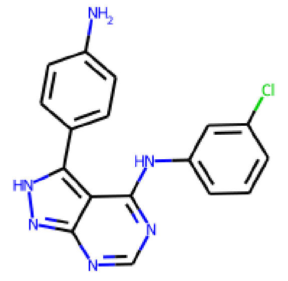	0.563480	c1ccc(Nc2ncnc3n[nH]c(-c4ccccc4)c23)cc1	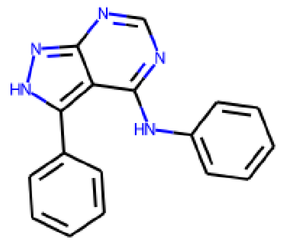	0.0	0.638163	3.995589	2.988471	4	0.800634
**1099**	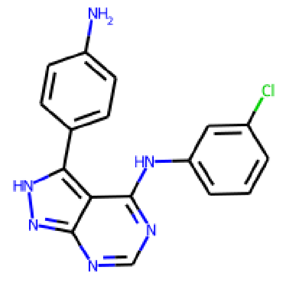	0.563480	c1ccc(Nc2ncnc3n[nH]c(-c4ccccc4)c23)cc1	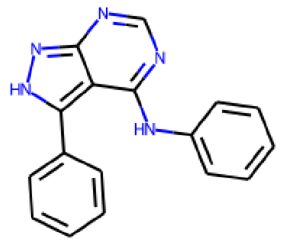	0.0	0.638163	3.995589	2.988471	4	0.800634

	src known mol	similarity	smiles	mol	Class	qed	logP	SAS	rings	kinase inhibition likeliness
**1100**	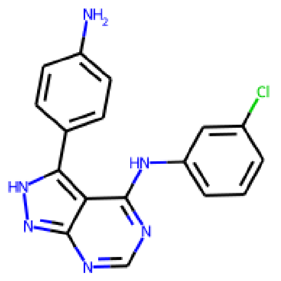	0.563480	c1ccc(Nc2ncnc3n[nH]c(-c4ccccc4)c23)cc1	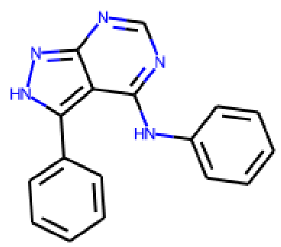	0.0	0.638163	3.995589	2.988471	4	0.800634
**1101**	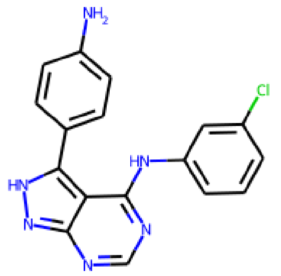	0.563480	c1ccc(Nc2ncnc3n[nH]c(-c4ccccc4)c23)cc1	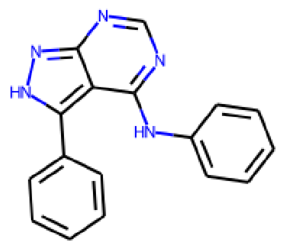	0.0	0.638163	3.995589	2.988471	4	0.800634
**1102**	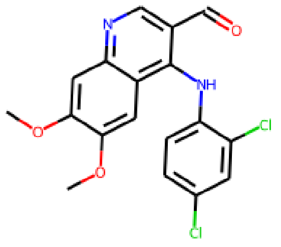	0.563473	COc1cc2ncc(C(=O)O)c(Nc3cccc(Cl)c3Cl)c2cc1OC1C(C)C1	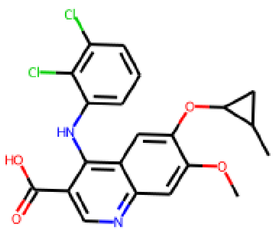	2.0	0.623362	2.525411	2.997881	4	0.774857
**1103**	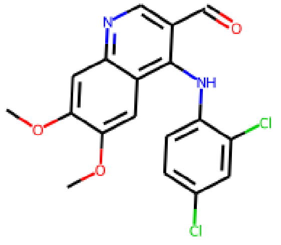	0.563473	COc1cc2ncc(C(=O)O)c(Nc3cccc(Cl)c3Cl)c2cc1OC1C(C)C1	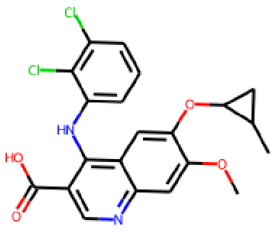	2.0	0.623362	2.525411	2.997881	4	0.774857

	src known mol	similarity	smiles	mol	Class	qed	logP	SAS	rings	kinase inhibition likeliness
**1104**	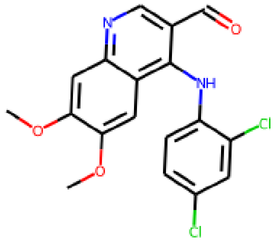	0.563265	COc1cc2ncc(C(=O)O)c(Nc3cccc(Cl)c3Cl)c2cc1OC1C(C)C1	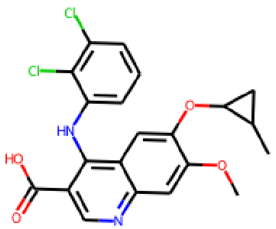	2.0	0.623362	2.525411	2.997881	4	0.774857
**1105**	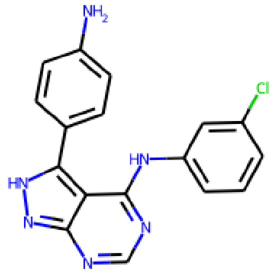	0.563183	c1ccc(Nc2ncnc3n[nH]c(-c4ccccc4)c23)cc1	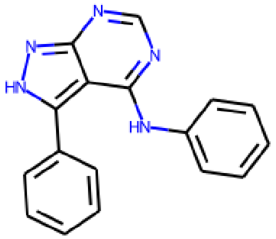	0.0	0.638163	3.995589	2.988471	4	0.800634
**1106**	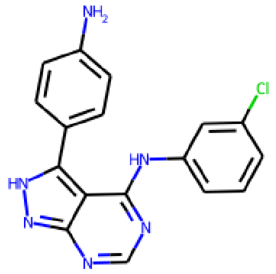	0.563137	c1ccc(Nc2ncnc3n[nH]c(-c4ccccc4)c23)cc1	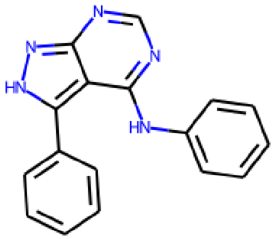	0.0	0.638163	3.995589	2.988471	4	0.800634
**1107**	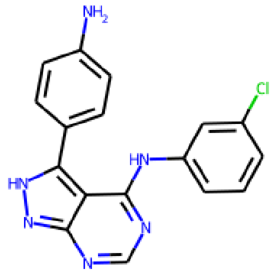	0.562794	c1ccc(Nc2ncnc3n[nH]c(-c4ccccc4)c23)cc1	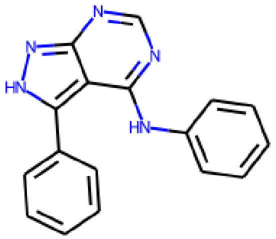	0.0	0.638163	3.995589	2.988471	4	0.800634

	src known mol	similarity	smiles	mol	Class	qed	logP	SAS	rings	kinase inhibition likeliness
**1108**	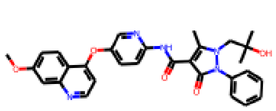	0.562732	COc1ccc2c(=O)cc(CNC(=O)c3c(C)n(C)n(-c4ccccc4)c3=O)nc3cccc2c3c1	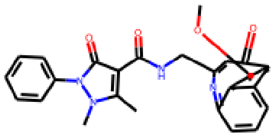	9.0	0.638163	3.995589	2.988471	5	0.774651
**1109**	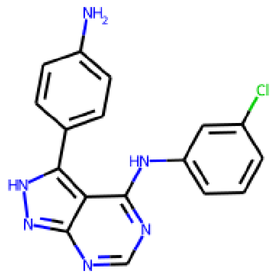	0.562598	c1ccc(Nc2ncnc3n[nH]c(-c4ccccc4)c23)cc1	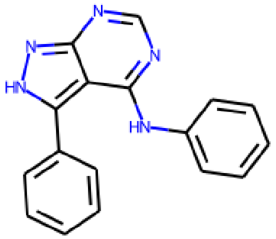	0.0	0.638163	3.995589	2.988471	4	0.800634
**1110**	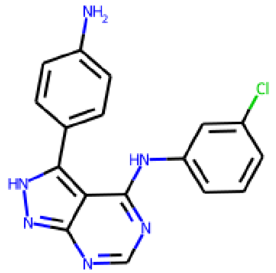	0.562598	c1ccc(Nc2ncnc3n[nH]c(-c4ccccc4)c23)cc1	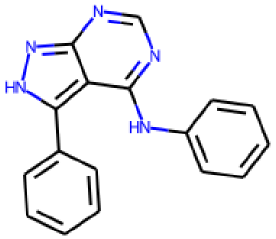	0.0	0.638163	3.995589	2.988471	4	0.800634
**1111**	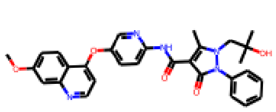	0.562467	COc1ccc2c(=O)cc(CNC(=O)c3c(C)n(C)n(-c4ccccc4)c3=O)nc3cccc2c3c1	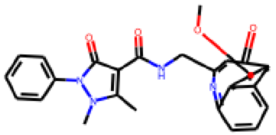	9.0	0.638163	3.995589	2.988471	5	0.774651

	src known mol	similarity	smiles	mol	Class	qed	logP	SAS	rings	kinase inhibition likeliness
**1112**	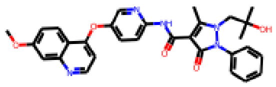	0.562467	COc1ccc2c(=O)cc(CNC(=O)c3c(C)n(C)n(-c4ccccc4)c3=O)nc3cccc2c3c1	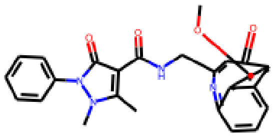	9.0	0.638163	3.995589	2.988471	5	0.774651
**1113**	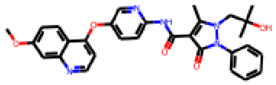	0.562400	COc1ccc2c(=O)cc(CNC(=O)c3c(C)n(C)n(-c4ccccc4)c3=O)nc3cccc2c3c1	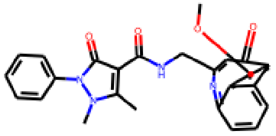	9.0	0.638163	3.995589	2.988471	5	0.774651
**1114**	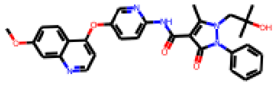	0.562400	COc1ccc2c(=O)cc(CNC(=O)c3c(C)n(C)n(-c4ccccc4)c3=O)nc3cccc2c3c1	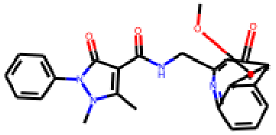	9.0	0.638163	3.995589	2.988471	5	0.774651
**1115**	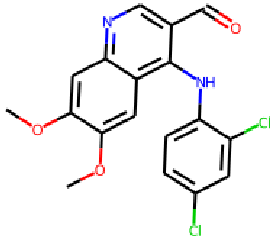	0.562317	COc1cc2ncc(C(=O)O)c(Nc3cccc(Cl)c3Cl)c2cc1OC1C(C)C1	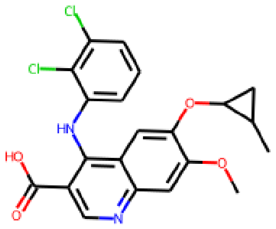	2.0	0.623362	2.525411	2.997881	4	0.774857

	src known mol	similarity	smiles	mol	Class	qed	logP	SAS	rings	kinase inhibition likeliness
**1116**	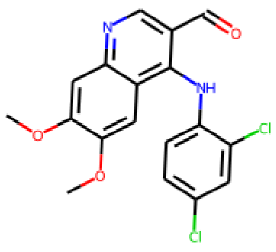	0.562317	COc1cc2ncc(C(=O)O)c(Nc3cccc(Cl)c3Cl)c2cc1OC1C(C)C1	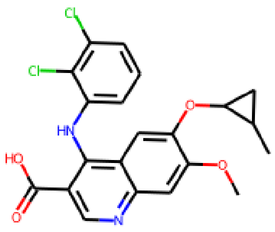	2.0	0.623362	2.525411	2.997881	4	0.774857
**1117**	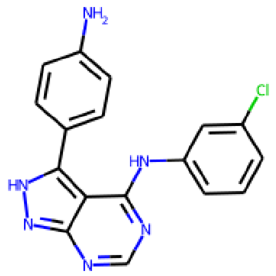	0.562255	c1ccc(Nc2ncnc3n[nH]c(-c4ccccc4)c23)cc1	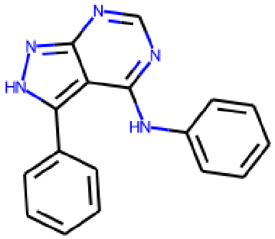	0.0	0.638163	3.995589	2.988471	4	0.800634
**1118**	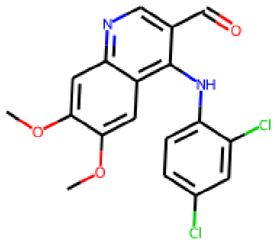	0.562189	COc1cc2ncc(C(=O)O)c(Nc3cccc(Cl)c3Cl)c2cc1OC1C(C)C1	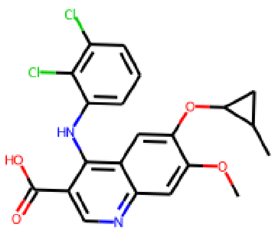	2.0	0.623362	2.525411	2.997881	4	0.774857
**1119**	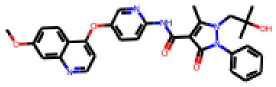	0.561935	COc1ccc2c(=O)cc(CNC(=O)c3c(C)n(C)n(-c4ccccc4)c3=O)nc3cccc2c3c1	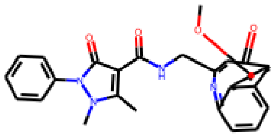	9.0	0.638163	3.995589	2.988471	5	0.774651

	src known mol	similarity	smiles	mol	Class	qed	logP	SAS	rings	kinase inhibition likeliness
**1120**	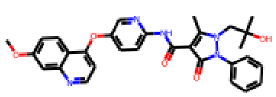	0.561935	COc1ccc2c(=O)cc(CNC(=O)c3c(C)n(C)n(-c4ccccc4)c3=O)nc3cccc2c3c1	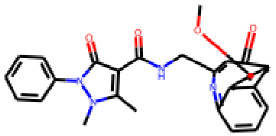	9.0	0.638163	3.995589	2.988471	5	0.774651
**1121**	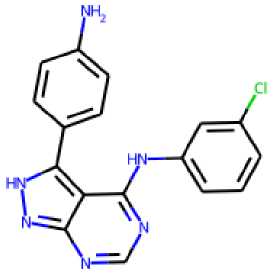	0.561772	c1ccc(Nc2ncnc3n[nH]c(-c4ccccc4)c23)cc1	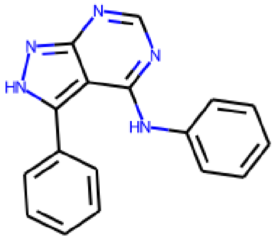	0.0	0.638163	3.995589	2.988471	4	0.800634
**1122**	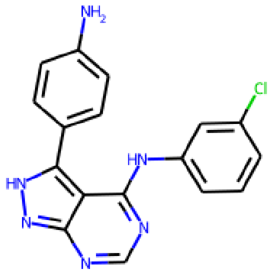	0.561772	c1ccc(Nc2ncnc3n[nH]c(-c4ccccc4)c23)cc1	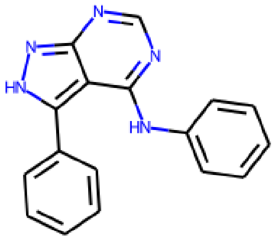	0.0	0.638163	3.995589	2.988471	4	0.800634
**1123**	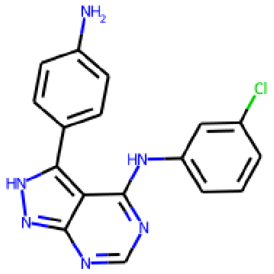	0.561709	c1ccc(Nc2ncnc3n[nH]c(-c4ccccc4)c23)cc1	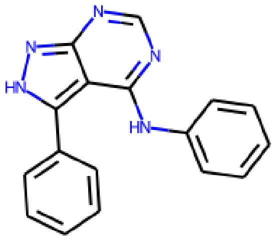	0.0	0.638163	3.995589	2.988471	4	0.800634

	src known mol	similarity	smiles	mol	Class	qed	logP	SAS	rings	kinase inhibition likeliness
**1124**	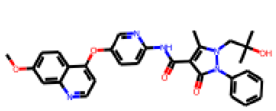	0.561709	COc1ccc2c(=O)cc(CNC(=O)c3c(C)n(C)n(-c4ccccc4)c3=O)nc3cccc2c3c1	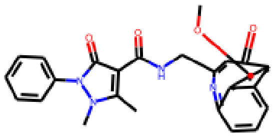	9.0	0.638163	3.995589	2.988471	5	0.774651
**1125**	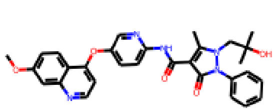	0.561709	COc1ccc2c(=O)cc(CNC(=O)c3c(C)n(C)n(-c4ccccc4)c3=O)nc3cccc2c3c1	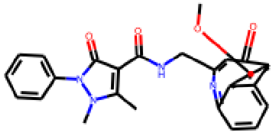	9.0	0.638163	3.995589	2.988471	5	0.774651
**1126**	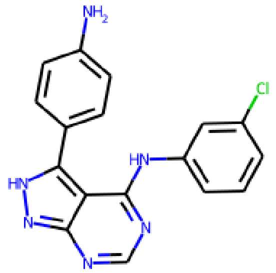	0.561622	c1ccc(Nc2ncnc3n[nH]c(-c4ccccc4)c23)cc1	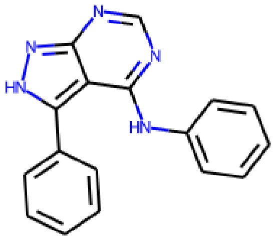	0.0	0.638163	3.995589	2.988471	4	0.800634
**1127**	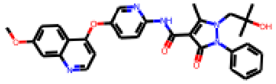	0.561530	COc1ccc2c(=O)cc(CNC(=O)c3c(C)n(C)n(-c4ccccc4)c3=O)nc3cccc3c2n1	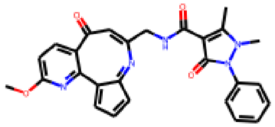	9.0	0.768132	3.608437	2.513170	5	0.823048

	src known mol	similarity	smiles	mol	Class	qed	logP	SAS	rings	kinase inhibition likeliness
**1128**	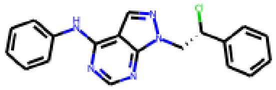	0.561311	Cc1ccc(C(=O)Nc2ccon2)cc1Nc1ncnc2c1cnn2C1cccccc1	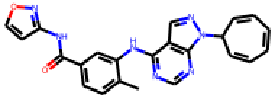	8.0	0.623362	2.525411	2.997881	5	0.770298
**1129**	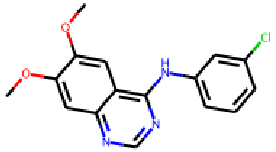	0.561173	Clc1cccc(Nc2ncnc3cc4c[nH]nc4cc23)c1	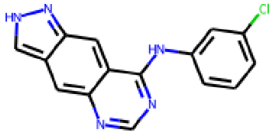	3.0	0.638163	3.995589	2.988471	4	0.781333
**1130**	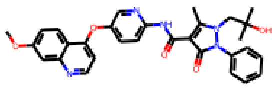	0.560934	COc1ccc2c(=O)cc(CNC(=O)c3c(C)n(C)n(-c4ccccc4)c3=O)nc3cccc3c2n1	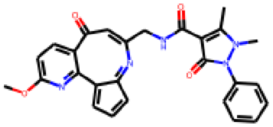	9.0	0.768132	3.608437	2.513170	5	0.823048
**1131**	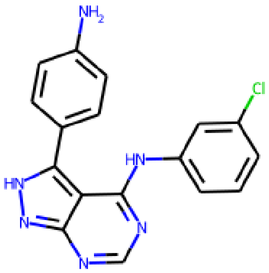	0.560842	c1ccc(Nc2ncnc3n[nH]c(-c4ccccc4)c23)cc1	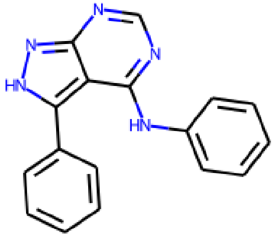	0.0	0.638163	3.995589	2.988471	4	0.800634

	src known mol	similarity	smiles	mol	Class	qed	logP	SAS	rings	kinase inhibition likeliness
**1132**	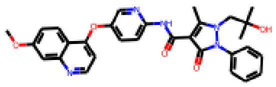	0.560841	COc1ccc2c(=O)cc(CNC(=O)c3c(C)n(C)n(-c4ccccc4)c3=O)nc3cccc3c2n1	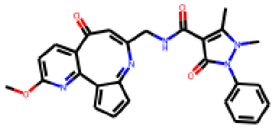	9.0	0.768132	3.608437	2.513170	5	0.823048
**1133**	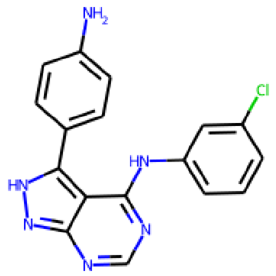	0.560630	c1ccc(Nc2ncnc3n[nH]c(-c4ccccc4)c23)cc1	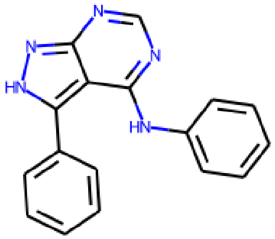	0.0	0.638163	3.995589	2.988471	4	0.800634
**1134**	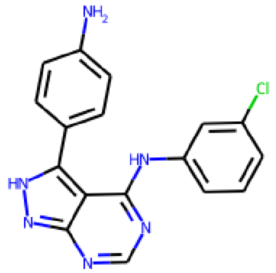	0.560594	c1ccc(Nc2ncnc3n[nH]c(-c4ccccc4)c23)cc1	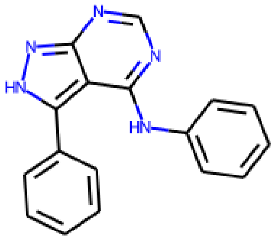	0.0	0.638163	3.995589	2.988471	4	0.800634
**1135**	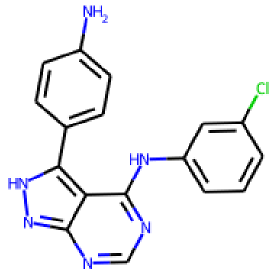	0.560250	c1ccc(Nc2ncnc3n[nH]c(-c4ccccc4)c23)cc1	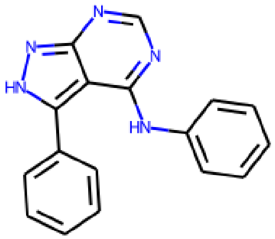	0.0	0.638163	3.995589	2.988471	4	0.800634

	src known mol	similarity	smiles	mol	Class	qed	logP	SAS	rings	kinase inhibition likeliness
**1136**	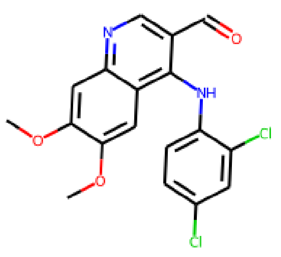	0.560169	COc1cc2ncc(C(=O)O)c(Nc3cccc(Cl)c3Cl)c2cc1OC1C(C)C1	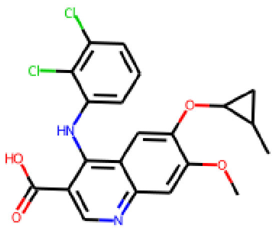	2.0	0.623362	2.525411	2.997881	4	0.774857
**1137**	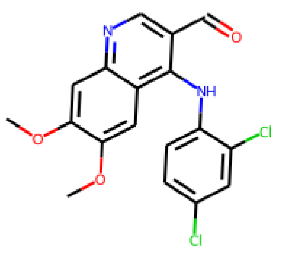	0.560169	COc1cc2ncc(C(=O)O)c(Nc3cccc(Cl)c3Cl)c2cc1OC1C(C)C1	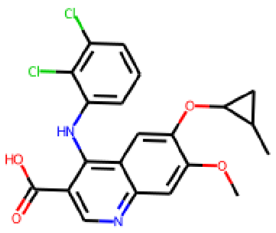	2.0	0.623362	2.525411	2.997881	4	0.774857
**1138**	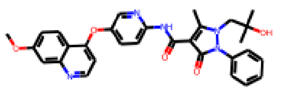	0.560109	COc1ccc2c(=O)cc(CNC(=O)c3c(C)n(C)n(-c4ccccc4)c3=O)nc3cccc2c3c1	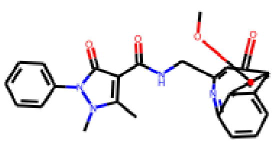	9.0	0.638163	3.995589	2.988471	5	0.774651
**1139**	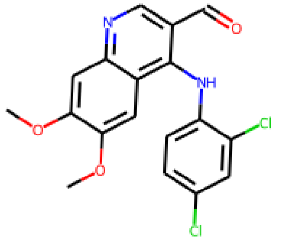	0.560098	COc1cc2ncc(C(=O)O)c(Nc3cccc(Cl)c3Cl)c2cc1OC1C(C)C1	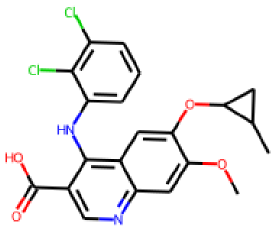	2.0	0.623362	2.525411	2.997881	4	0.774857

	src known mol	similarity	smiles	mol	Class	qed	logP	SAS	rings	kinase inhibition likeliness
**1140**	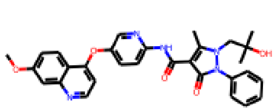	0.560043	COc1ccc2c(=O)cc(CNC(=O)c3c(C)n(C)n(-c4ccccc4)c3=O)nc3cccc2c3c1	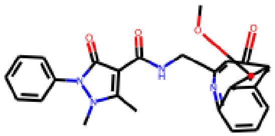	9.0	0.638163	3.995589	2.988471	5	0.774651
**1141**	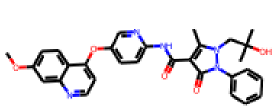	0.559893	COc1ccc2c(=O)cc(CNC(=O)c3c(C)n(C)n(-c4ccccc4)c3=O)nc3cccc2c3c1	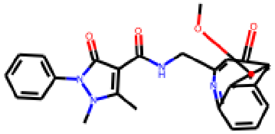	9.0	0.638163	3.995589	2.988471	5	0.774651
**1142**	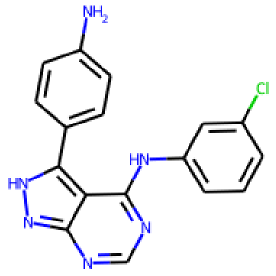	0.559876	c1ccc(Nc2ncnc3n[nH]c(-c4ccccc4)c23)cc1	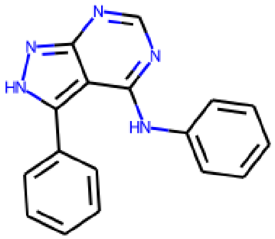	0.0	0.638163	3.995589	2.988471	4	0.800634
**1143**	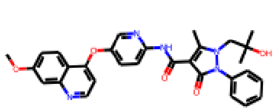	0.559872	COc1ccc2c(=O)cc(CNC(=O)c3c(C)n(C)n(-c4ccccc4)c3=O)nc3cccc2c3c1	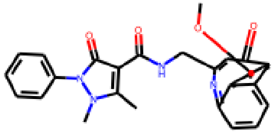	9.0	0.638163	3.995589	2.988471	5	0.774651

	src known mol	similarity	smiles	mol	Class	qed	logP	SAS	rings	kinase inhibition likeliness
**1144**	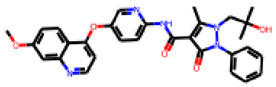	0.559872	COc1ccc2c(=O)cc(CNC(=O)c3c(C)n(C)n(-c4ccccc4)c3=O)nc3cccc2c3c1	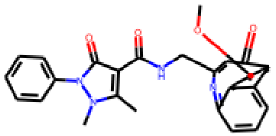	9.0	0.638163	3.995589	2.988471	5	0.774651
**1145**	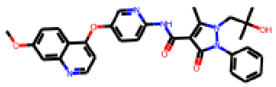	0.559788	COc1ccc2c(=O)cc(CNC(=O)c3c(C)n(C)n(-c4ccccc4)c3=O)nc3cccc2c3c1	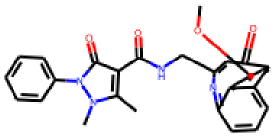	9.0	0.638163	3.995589	2.988471	5	0.774651
**1146**	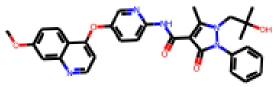	0.559788	COc1ccc2c(=O)cc(CNC(=O)c3c(C)n(C)n(-c4ccccc4)c3=O)nc3cccc2c3c1	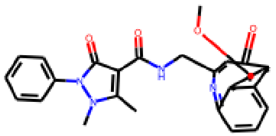	9.0	0.638163	3.995589	2.988471	5	0.774651
**1147**	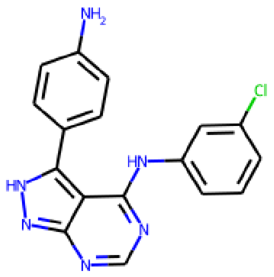	0.559375	c1ccc(Nc2ncnc3n[nH]c(-c4ccccc4)c23)cc1	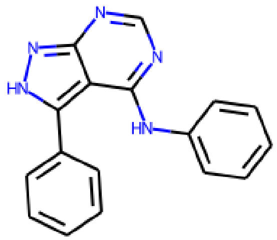	0.0	0.638163	3.995589	2.988471	4	0.800634

	src known mol	similarity	smiles	mol	Class	qed	logP	SAS	rings	kinase inhibition likeliness
**1148**	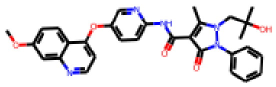	0.559214	COc1ccc2c(=O)cc(CNC(=O)c3c(C)n(C)n(-c4ccccc4)c3=O)nc3cccc2c3c1	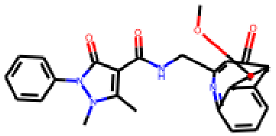	9.0	0.638163	3.995589	2.988471	5	0.774651
**1149**	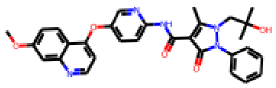	0.559214	COc1ccc2c(=O)cc(CNC(=O)c3c(C)n(C)n(-c4ccccc4)c3=O)nc3cccc2c3c1	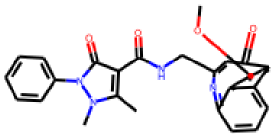	9.0	0.638163	3.995589	2.988471	5	0.774651
**1150**	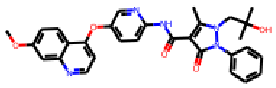	0.559105	COc1ccc2c(=O)cc(CNC(=O)c3c(C)n(C)n(-c4ccccc4)c3=O)nc3cccc2c3c1	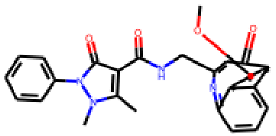	9.0	0.638163	3.995589	2.988471	5	0.774651
**1151**	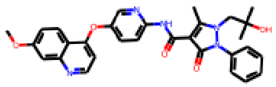	0.559105	COc1ccc2c(=O)cc(CNC(=O)c3c(C)n(C)n(-c4ccccc4)c3=O)nc3cccc2c3c1	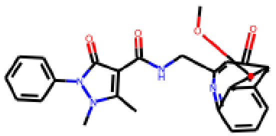	9.0	0.638163	3.995589	2.988471	5	0.774651

	src known mol	similarity	smiles	mol	Class	qed	logP	SAS	rings	kinase inhibition likeliness
**1152**	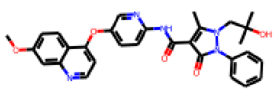	0.559105	COc1ccc2c(=O)cc(CNC(=O)c3c(C)n(C)n(-c4ccccc4)c3=O)nc3cccc2c3c1	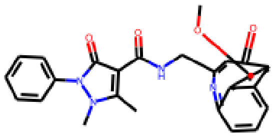	9.0	0.638163	3.995589	2.988471	5	0.774651
**1153**	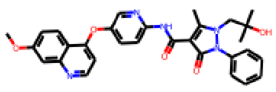	0.559105	COc1ccc2c(=O)cc(CNC(=O)c3c(C)n(C)n(-c4ccccc4)c3=O)nc3cccc2c3c1	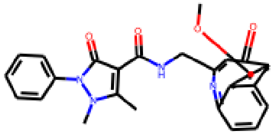	9.0	0.638163	3.995589	2.988471	5	0.774651
**1154**	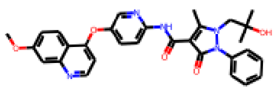	0.559105	COc1ccc2c(=O)cc(CNC(=O)c3c(C)n(C)n(-c4ccccc4)c3=O)nc3cccc2c3c1	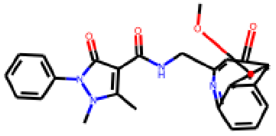	9.0	0.638163	3.995589	2.988471	5	0.774651
**1155**	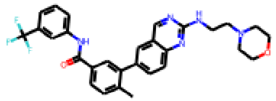	0.559067	Cc1ccc(C(=O)Nc2cccc(C(F)(F)F)c2C)cc1-c1ccc2oc(N)nc2c1	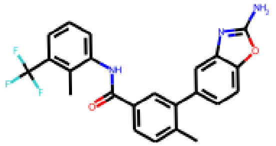	6.0	0.635315	4.542409	3.856266	4	0.806311

	src known mol	similarity	smiles	mol	Class	qed	logP	SAS	rings	kinase inhibition likeliness
**1156**	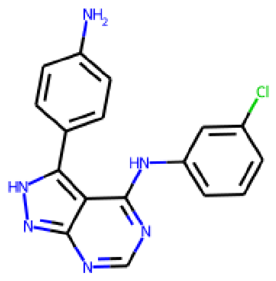	0.558891	c1ccc(Nc2ncnc3n[nH]c(-c4ccccc4)c23)cc1	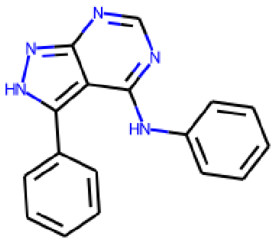	0.0	0.638163	3.995589	2.988471	4	0.800634
**1157**	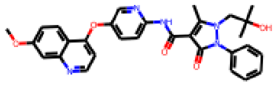	0.558448	COc1ccc2c(=O)cc(CNC(=O)c3c(C)n(C)n(-c4ccccc4)c3=O)nc3cccc2c3c1	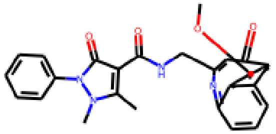	9.0	0.638163	3.995589	2.988471	5	0.774651
**1158**	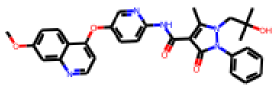	0.558448	COc1ccc2c(=O)cc(CNC(=O)c3c(C)n(C)n(-c4ccccc4)c3=O)nc3cccc2c3c1	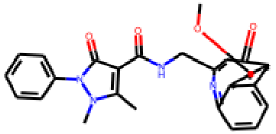	9.0	0.638163	3.995589	2.988471	5	0.774651
**1159**	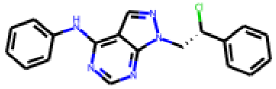	0.558442	Cc1ccc(C(=O)Nc2ccon2)cc1Nc1ncnc2c1cnn2C1cccccc1	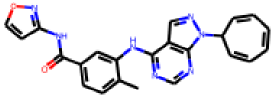	8.0	0.623362	2.525411	2.997881	5	0.770298

	src known mol	similarity	smiles	mol	Class	qed	logP	SAS	rings	kinase inhibition likeliness
**1160**	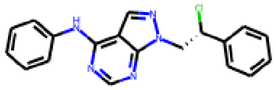	0.558442	Cc1ccc(C(=O)Nc2ccon2)cc1Nc1ncnc2c1cnn2C1cccccc1	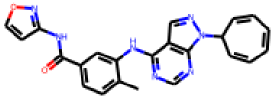	8.0	0.623362	2.525411	2.997881	5	0.770298
**1161**	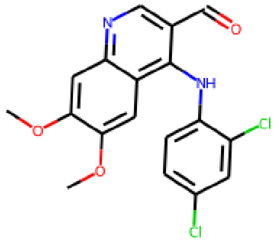	0.558216	COc1cc2ncc(C(=O)O)c(Nc3cccc(Cl)c3Cl)c2cc1OC1C(C)C1	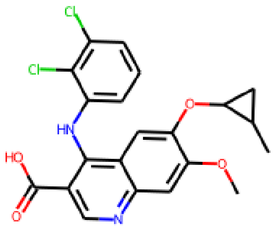	2.0	0.623362	2.525411	2.997881	4	0.774857
**1162**	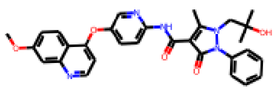	0.558176	COc1ccc2c(=O)cc(CNC(=O)c3c(C)n(C)n(-c4ccccc4)c3=O)nc3cccc2c3c1	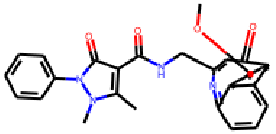	9.0	0.638163	3.995589	2.988471	5	0.774651
**1163**	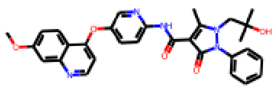	0.558176	COc1ccc2c(=O)cc(CNC(=O)c3c(C)n(C)n(-c4ccccc4)c3=O)nc3cccc2c3c1	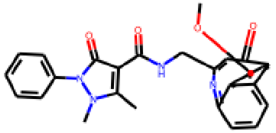	9.0	0.638163	3.995589	2.988471	5	0.774651

	src known mol	similarity	smiles	mol	Class	qed	logP	SAS	rings	kinase inhibition likeliness
**1164**	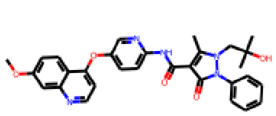	0.558040	COc1ccc2c(=O)cc(CNC(=O)c3c(C)n(C)n(-c4ccccc4)c3=O)nc3cccc2c3c1	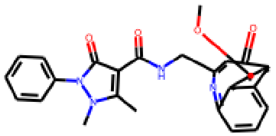	9.0	0.638163	3.995589	2.988471	5	0.774651
**1165**	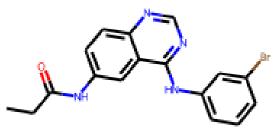	0.558036	O=C(CNC(=O)Oc1ccccc1)Nc1ccc2ncnc(Nc3cccc(Cl)c3)c2c1	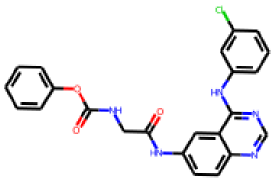	3.0	0.638163	3.995589	2.988471	4	0.793889
**1166**	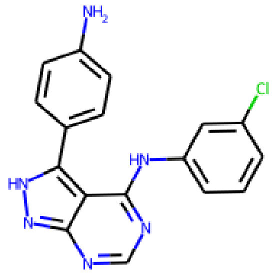	0.557887	c1ccc(Nc2ncnc3n[nH]c(-c4ccccc4)c23)cc1	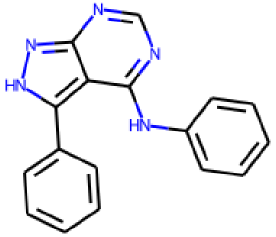	0.0	0.638163	3.995589	2.988471	4	0.800634
**1167**	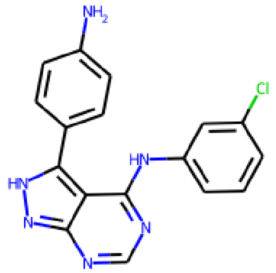	0.557887	c1ccc(Nc2ncnc3n[nH]c(-c4ccccc4)c23)cc1	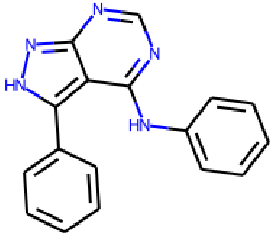	0.0	0.638163	3.995589	2.988471	4	0.800634

	src known mol	similarity	smiles	mol	Class	qed	logP	SAS	rings	kinase inhibition likeliness
**1168**	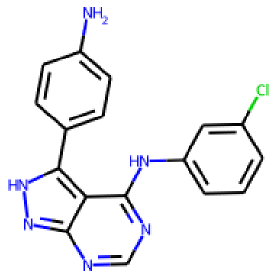	0.557887	c1ccc(Nc2ncnc3n[nH]c(-c4ccccc4)c23)cc1	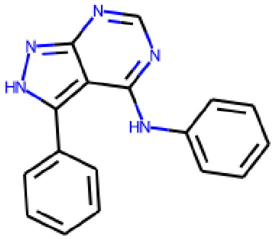	0.0	0.638163	3.995589	2.988471	4	0.800634
**1169**	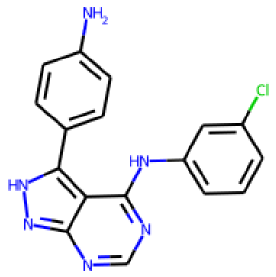	0.557887	c1ccc(Nc2ncnc3n[nH]c(-c4ccccc4)c23)cc1	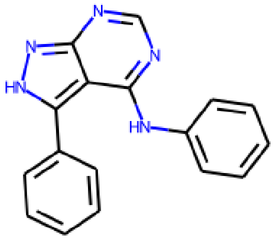	0.0	0.638163	3.995589	2.988471	4	0.800634
**1170**	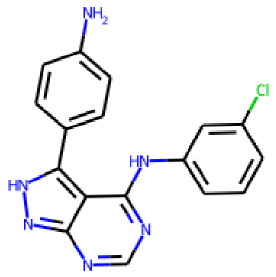	0.557854	c1ccc(Nc2ncnc3n[nH]c(-c4ccccc4)c32)cc1	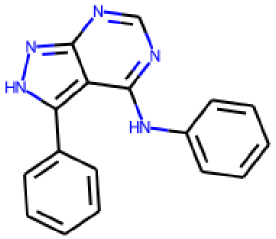	0.0	0.520676	2.336694	2.899742	4	0.831417
**1171**	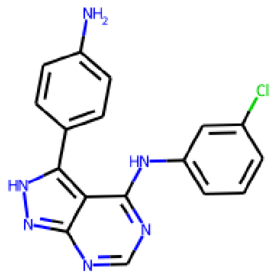	0.557854	c1ccc(Nc2ncnc3n[nH]c(-c4ccccc4)c32)cc1	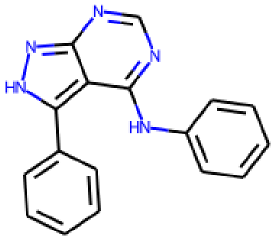	0.0	0.520676	2.336694	2.899742	4	0.831417

	src known mol	similarity	smiles	mol	Class	qed	logP	SAS	rings	kinase inhibition likeliness
**1172**	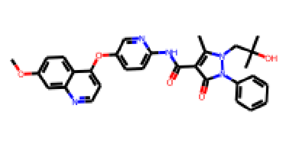	0.557845	COc1ccc2c(=O)cc(CNC(=O)c3c(C)n(C)n(-c4ccccc4)c3=O)nc3cccc2c3c1	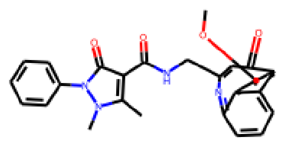	9.0	0.638163	3.995589	2.988471	5	0.774651
**1173**	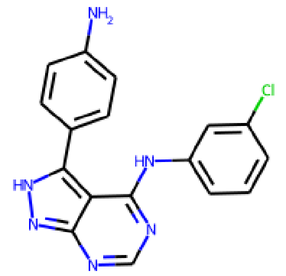	0.557781	c1ccc(Nc2ncnc3n[nH]c(-c4ccccc4)c23)cc1	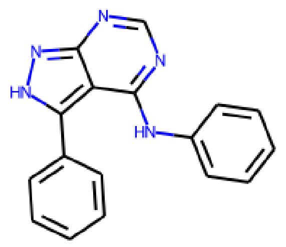	0.0	0.638163	3.995589	2.988471	4	0.800634
**1174**	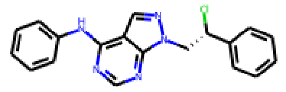	0.557730	Cc1ccc(C(=O)Nc2ccon2)cc1Nc1ncnc2c1cnn2C1cccccc1	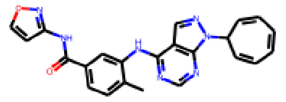	8.0	0.623362	2.525411	2.997881	5	0.770298
**1175**	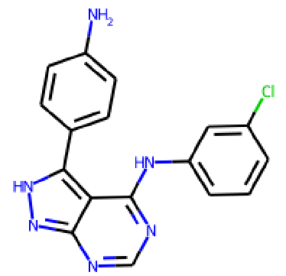	0.557529	c1ccc(Nc2ncnc3n[nH]c(-c4ccccc4)c23)cc1	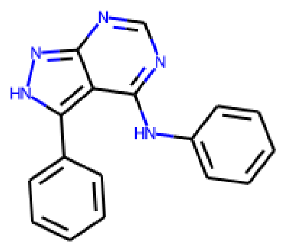	0.0	0.638163	3.995589	2.988471	4	0.800634

	src known mol	similarity	smiles	mol	Class	qed	logP	SAS	rings	kinase inhibition likeliness
**1176**	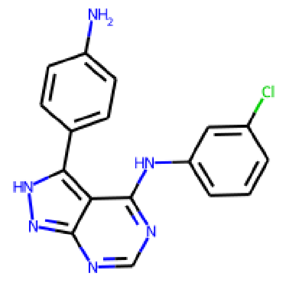	0.557187	c1ccc(Nc2ncnc3n[nH]c(-c4ccccc4)c23)cc1	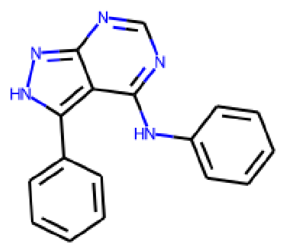	0.0	0.638163	3.995589	2.988471	4	0.800634
**1177**	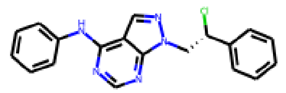	0.557152	Cc1ccc(C(=O)Nc2ccon2)cc1Nc1ncnc2c1cnn2C1cccccc1	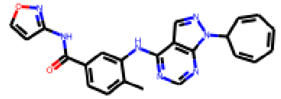	8.0	0.623362	2.525411	2.997881	5	0.770298
**1178**	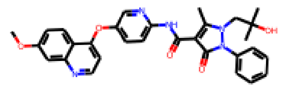	0.556976	COc1ccc2c(=O)cc(CNC(=O)c3c(C)n(C)n(-c4ccccc4)c3=O)nc3cccc2c3c1	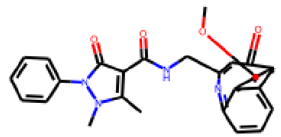	9.0	0.638163	3.995589	2.988471	5	0.774651
**1179**	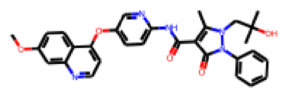	0.556976	COc1ccc2c(=O)cc(CNC(=O)c3c(C)n(C)n(-c4ccccc4)c3=O)nc3cccc2c3c1	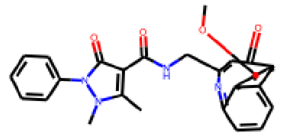	9.0	0.638163	3.995589	2.988471	5	0.774651

	src known mol	similarity	smiles	mol	Class	qed	logP	SAS	rings	kinase inhibition likeliness
**1180**	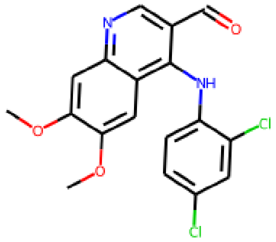	0.556962	COc1cc2ncc(C(=O)O)c(Nc3cccc(Cl)c3Cl)c2cc1OC1C(C)C1	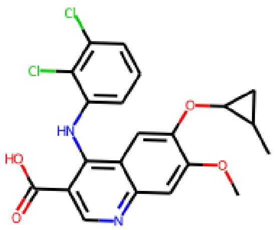	2.0	0.623362	2.525411	2.997881	4	0.774857
**1181**	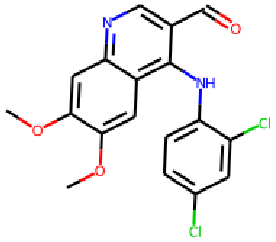	0.556962	COc1cc2ncc(C(=O)O)c(Nc3cccc(Cl)c3Cl)c2cc1OC1C(C)C1	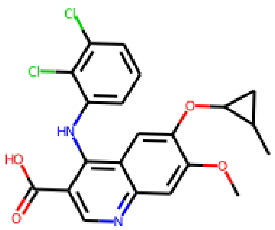	2.0	0.623362	2.525411	2.997881	4	0.774857
**1182**	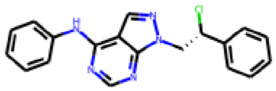	0.556921	Cc1ccc(C(=O)Nc2ccon2)cc1Nc1ncnc2c1cnn2C1cccccc1	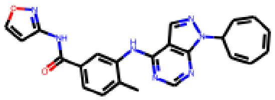	8.0	0.623362	2.525411	2.997881	5	0.770298
**1183**	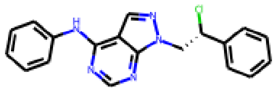	0.556921	Cc1ccc(C(=O)Nc2ccon2)cc1Nc1ncnc2c1cnn2C1cccccc1	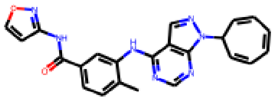	8.0	0.623362	2.525411	2.997881	5	0.770298

	src known mol	similarity	smiles	mol	Class	qed	logP	SAS	rings	kinase inhibition likeliness
1184	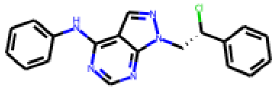	0.556921	Cc1ccc(C(=O)Nc2ccon2)cc1Nc1ncnc2c1cnn2C1cccccc1	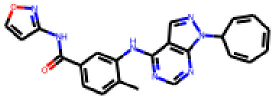	8.0	0.623362	2.525411	2.997881	5	0.770298
1185	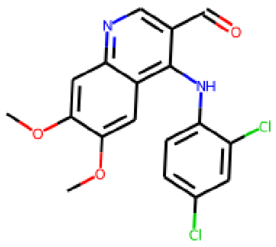	0.556634	COc1cc2ncc(C(=O)O)c(Nc3cccc(Cl)c3Cl)c2cc1OC1C(C)C1	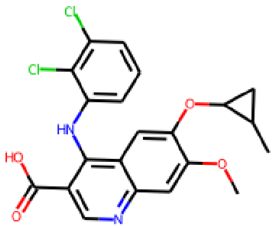	2.0	0.623362	2.525411	2.997881	4	0.774857
1186	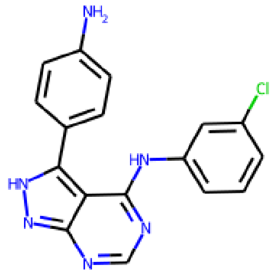	0.556420	c1ccc(Nc2ncnc3n[nH]c(-c4ccccc4)c23)cc1	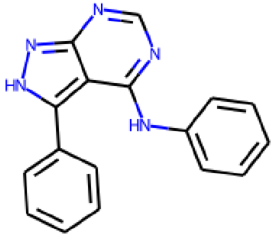	0.0	0.638163	3.995589	2.988471	4	0.800634
1187	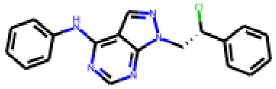	0.556410	Cc1ccc(C(=O)Nc2ccon2)cc1Nc1ncnc2c1cnn2C1cccccc1	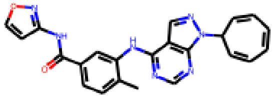	8.0	0.623362	2.525411	2.997881	5	0.770298

	src known mol	similarity	smiles	mol	Class	qed	logP	SAS	rings	kinase inhibition likeliness
**1188**	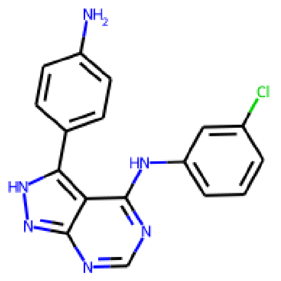	0.556327	c1ccc(Nc2ncnc3n[nH]c(-c4ccccc4)c23)cc1	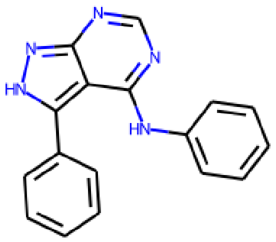	0.0	0.638163	3.995589	2.988471	4	0.800634
**1189**	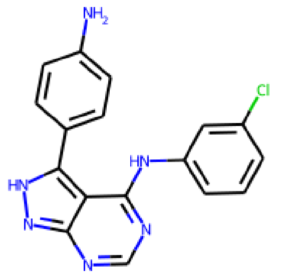	0.556317	c1ccc(Nc2ncnc3n[nH]c(-c4ccccc4)c23)cc1	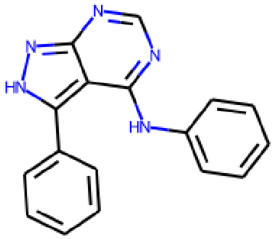	0.0	0.638163	3.995589	2.988471	4	0.800634
**1190**	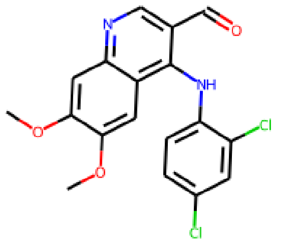	0.556175	COc1cc2ncc(C(=O)O)c(Nc3cccc(Cl)c3Cl)c2cc1OC1C(C)C1	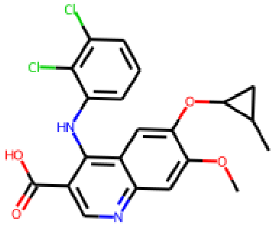	2.0	0.623362	2.525411	2.997881	4	0.774857
**1191**	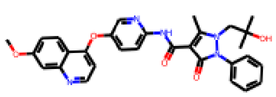	0.556108	COc1ccc2c(=O)cc(CNC(=O)c3c(C)n(C)n(-c4ccccc4)c3=O)nc3cccc3c2n1	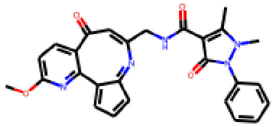	9.0	0.768132	3.608437	2.513170	5	0.823048

	src known mol	similarity	smiles	mol	Class	qed	logP	SAS	rings	kinase inhibition likeliness
**1192**	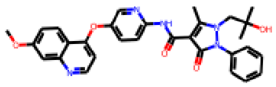	0.556108	COc1ccc2c(=O)cc(CNC(=O)c3c(C)n(C)n(-c4ccccc4)c3=O)nc3cccc3c2n1	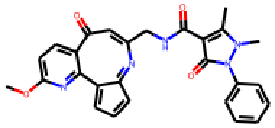	9.0	0.768132	3.608437	2.513170	5	0.823048
**1193**	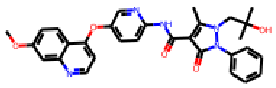	0.556108	COc1ccc2c(=O)cc(CNC(=O)c3c(C)n(C)n(-c4ccccc4)c3=O)nc3cccc3c2n1	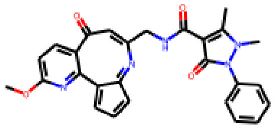	9.0	0.768132	3.608437	2.513170	5	0.823048
**1194**	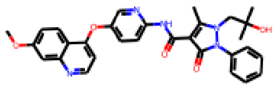	0.556108	COc1ccc2c(=O)cc(CNC(=O)c3c(C)n(C)n(-c4ccccc4)c3=O)nc3cccc3c2n1	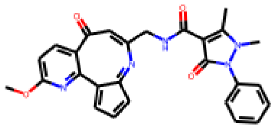	9.0	0.768132	3.608437	2.513170	5	0.823048
**1195**	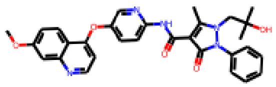	0.556108	COc1ccc2c(=O)cc(CNC(=O)c3c(C)n(C)n(-c4ccccc4)c3=O)nc3cccc3c2n1	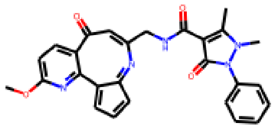	9.0	0.768132	3.608437	2.513170	5	0.823048

	src known mol	similarity	smiles	mol	Class	qed	logP	SAS	rings	kinase inhibition likeliness
**1196**	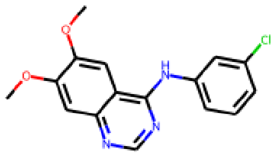	0.555914	Clc1cccc(Nc2ncnc3cc4c[nH]nc4cc23)c1	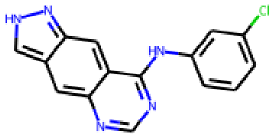	3.0	0.638163	3.995589	2.988471	4	0.781333
**1197**	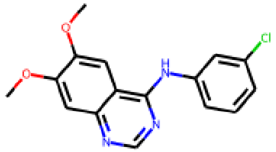	0.555911	Clc1cccc(Nc2ncnc3cc4c[nH]nc4cc23)c1	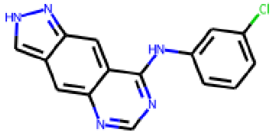	3.0	0.638163	3.995589	2.988471	4	0.781333
**1198**	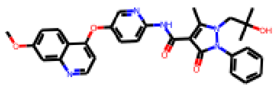	0.555732	COc1ccc2c(=O)cc(CNC(=O)c3c(C)n(C)n(-c4ccccc4)c3=O)nc3cccc2c3c1	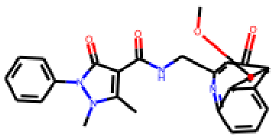	9.0	0.638163	3.995589	2.988471	5	0.774651
**1199**	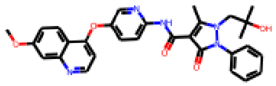	0.555732	COc1ccc2c(=O)cc(CNC(=O)c3c(C)n(C)n(-c4ccccc4)c3=O)nc3cccc2c3c1	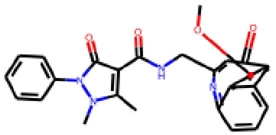	9.0	0.638163	3.995589	2.988471	5	0.774651

	src known mol	similarity	smiles	mol	Class	qed	logP	SAS	rings	kinase inhibition likeliness
**1200**	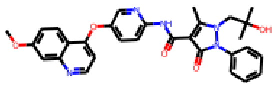	0.555614	COc1ccc2c(=O)cc(CNC(=O)c3c(C)n(C)n(-c4ccccc4)c3=O)nc3cccc2c3c1	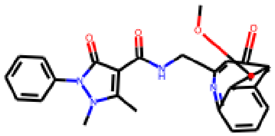	9.0	0.638163	3.995589	2.988471	5	0.774651
**1201**	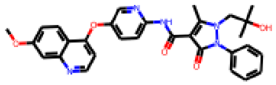	0.555556	COc1ccc2c(=O)cc(CNC(=O)c3c(C)n(C)n(-c4ccccc4)c3=O)nc3cccc2c3c1	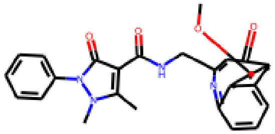	9.0	0.638163	3.995589	2.988471	5	0.774651
**1202**	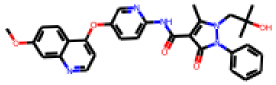	0.555556	COc1ccc2c(=O)cc(CNC(=O)c3c(C)n(C)n(-c4ccccc4)c3=O)nc3cccc2c3c1	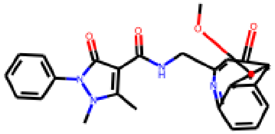	9.0	0.638163	3.995589	2.988471	5	0.774651
**1203**	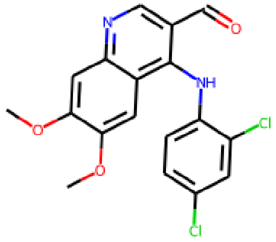	0.555474	COc1cc2ncc(C(=O)O)c(Nc3cccc(Cl)c3Cl)c2cc1OC1C(C)C1	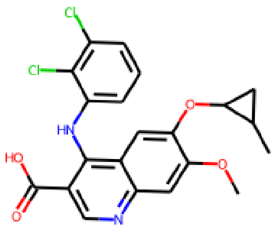	2.0	0.623362	2.525411	2.997881	4	0.774857

	src known mol	similarity	smiles	mol	Class	qed	logP	SAS	rings	kinase inhibition likeliness
**1204**	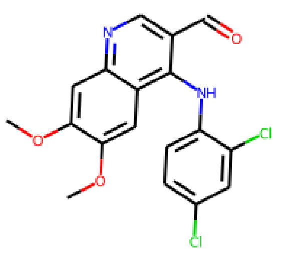	0.555474	COc1cc2ncc(C(=O)O)c(Nc3cccc(Cl)c3Cl)c2cc1OC1C(C)C1	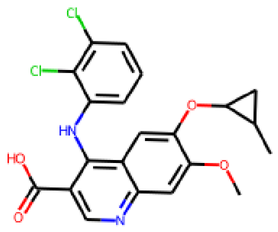	2.0	0.623362	2.525411	2.997881	4	0.774857
**1205**	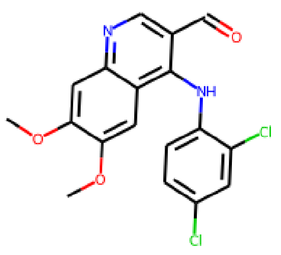	0.555474	COc1cc2ncc(C(=O)O)c(Nc3cccc(Cl)c3Cl)c2cc1OC1C(C)C1	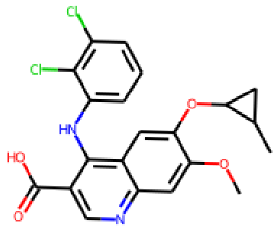	2.0	0.623362	2.525411	2.997881	4	0.774857
**1206**	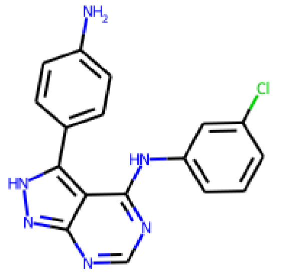	0.555042	c1ccc(Nc2ncnc3n[nH]c(-c4ccccc4)c23)cc1	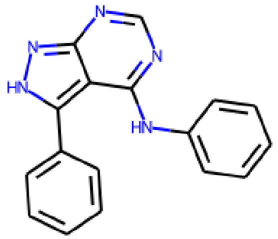	0.0	0.638163	3.995589	2.988471	4	0.800634
**1207**	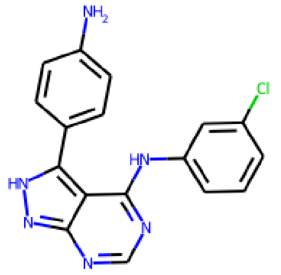	0.554954	c1ccc(Nc2ncnc3n[nH]c(-c4ccccc4)c23)cc1	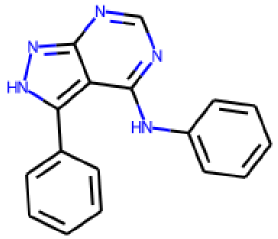	0.0	0.638163	3.995589	2.988471	4	0.800634

	src known mol	similarity	smiles	mol	Class	qed	logP	SAS	rings	kinase inhibition likeliness
**1208**	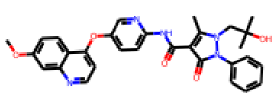	0.554753	COc1ccc2c(=O)cc(CNC(=O)c3c(C)n(C)n(-c4ccccc4)c3=O)nc3cccc3c2n1	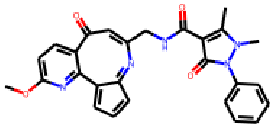	9.0	0.768132	3.608437	2.513170	5	0.823048
**1209**	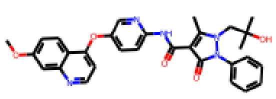	0.554555	COc1ccc2c(=O)cc(CNC(=O)c3c(C)n(C)n(-c4ccccc4)c3=O)nc3cccc2c3c1	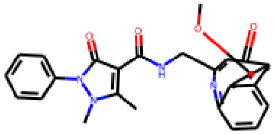	9.0	0.638163	3.995589	2.988471	5	0.774651
**1210**	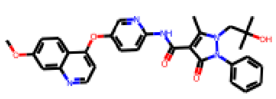	0.554555	COc1ccc2c(=O)cc(CNC(=O)c3c(C)n(C)n(-c4ccccc4)c3=O)nc3cccc2c3c1	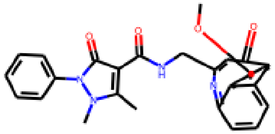	9.0	0.638163	3.995589	2.988471	5	0.774651
**1211**	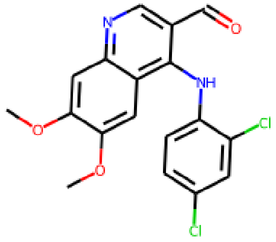	0.554273	COc1cc2ncc(C(=O)O)c(Nc3cccc(Cl)c3Cl)c2cc1OC1C(C)C1	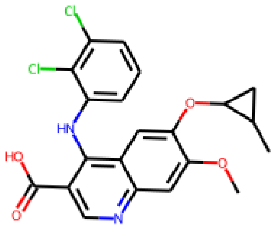	2.0	0.623362	2.525411	2.997881	4	0.774857

	src known mol	similarity	smiles	mol	Class	qed	logP	SAS	rings	kinase inhibition likeliness
**1212**	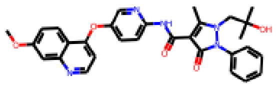	0.554198	COc1ccc2c(=O)cc(CNC(=O)c3c(C)n(C)n(-c4ccccc4)c3=O)nc3cccc2c3c1	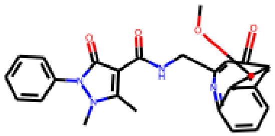	9.0	0.638163	3.995589	2.988471	5	0.774651
**1213**	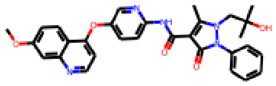	0.554198	COc1ccc2c(=O)cc(CNC(=O)c3c(C)n(C)n(-c4ccccc4)c3=O)nc3cccc2c3c1	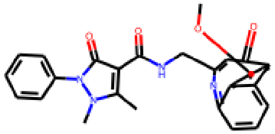	9.0	0.638163	3.995589	2.988471	5	0.774651
**1214**	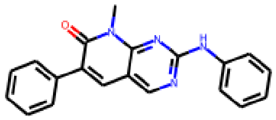	0.554146	Clc1cccc(Nc2ncc3cc(-c3c(Cl)cccc3Cl)c(=O)n(C)c3n2)c1	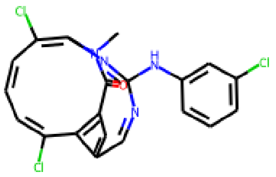	2.0	0.768132	3.608437	2.513170	5	0.784634
**1215**	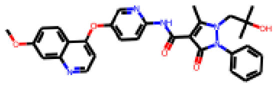	0.554047	COc1ccc2c(=O)cc(CNC(=O)c3c(C)n(C)n(-c4ccccc4)c3=O)nc3cccc3c2n1	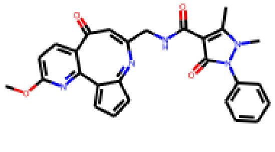	9.0	0.768132	3.608437	2.513170	5	0.823048

	src known mol	similarity	smiles	mol	Class	qed	logP	SAS	rings	kinase inhibition likeliness
**1216**	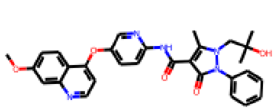	0.554018	COc1ccc2c(=O)cc(CNC(=O)c3c(C)n(C)n(-c4ccccc4)c3=O)nc3cccc2c3c1	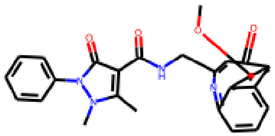	9.0	0.638163	3.995589	2.988471	5	0.774651
**1217**	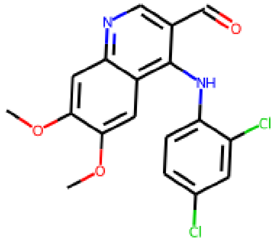	0.553906	COc1cc2ncc(C(=O)O)c(Nc3cccc(Cl)c3Cl)c2cc1OC1C(C)C1	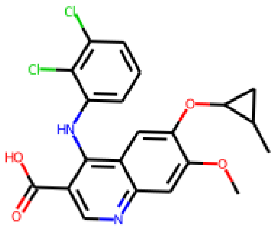	2.0	0.623362	2.525411	2.997881	4	0.774857
**1218**	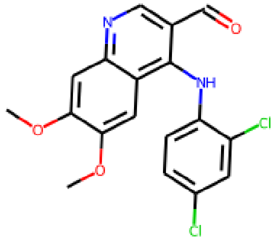	0.553906	COc1cc2ncc(C(=O)O)c(Nc3cccc(Cl)c3Cl)c2cc1OC1C(C)C1	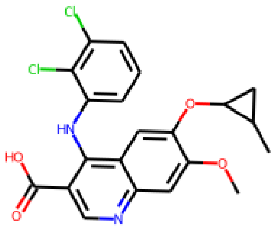	2.0	0.623362	2.525411	2.997881	4	0.774857
**1219**	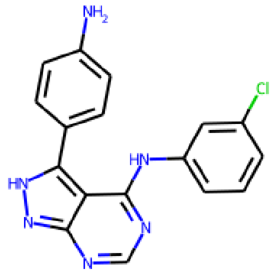	0.553751	c1ccc(Nc2ncnc3n[nH]c(-c4ccccc4)c23)cc1	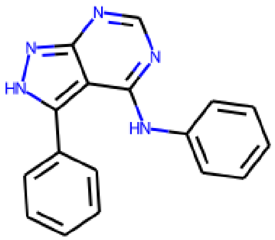	0.0	0.638163	3.995589	2.988471	4	0.800634

	src known mol	similarity	smiles	mol	Class	qed	logP	SAS	rings	kinase inhibition likeliness
**1220**	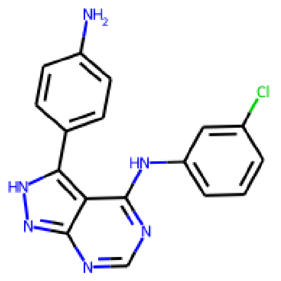	0.553751	c1ccc(Nc2ncnc3n[nH]c(-c4ccccc4)c23)cc1	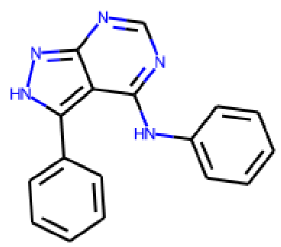	0.0	0.638163	3.995589	2.988471	4	0.800634
**1221**	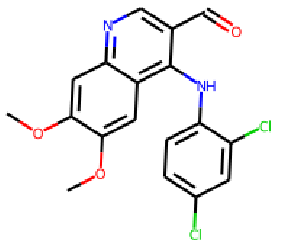	0.553611	COc1cc2ncc(C(=O)O)c(Nc3cccc(Cl)c3Cl)c2cc1OC1C(C)C1	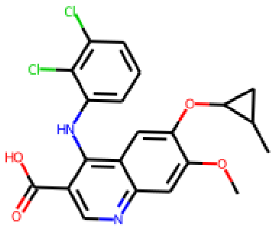	2.0	0.623362	2.525411	2.997881	4	0.774857
**1222**	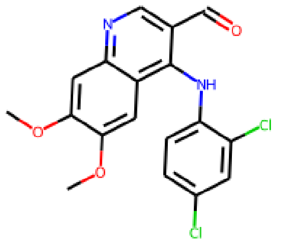	0.553611	COc1cc2ncc(C(=O)O)c(Nc3cccc(Cl)c3Cl)c2cc1OC1C(C)C1	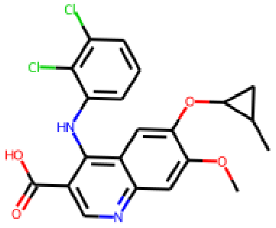	2.0	0.623362	2.525411	2.997881	4	0.774857
**1223**	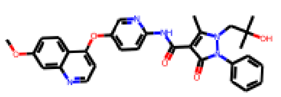	0.553259	COc1ccc2c(=O)cc(CNC(=O)c3c(C)n(C)n(-c4ccccc4)c3=O)nc3cccc2c3c1	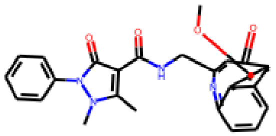	9.0	0.638163	3.995589	2.988471	5	0.774651

	src known mol	similarity	smiles	mol	Class	qed	logP	SAS	rings	kinase inhibition likeliness
**1224**	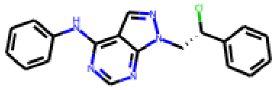	0.553191	Cc1ccc(C(=O)Nc2ccon2)cc1Nc1ncnc2c1cnn2C1cccccc1	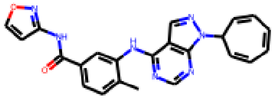	8.0	0.623362	2.525411	2.997881	5	0.770298
**1225**	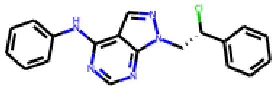	0.553191	Cc1ccc(C(=O)Nc2ccon2)cc1Nc1ncnc2c1cnn2C1cccccc1	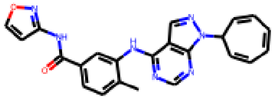	8.0	0.623362	2.525411	2.997881	5	0.770298
**1226**	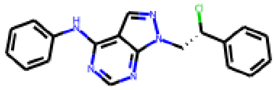	0.553151	Cc1ccc(C(=O)Nc2ccon2)cc1Nc1ncnc2c1cnn2C1cccccc1	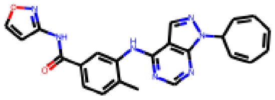	8.0	0.623362	2.525411	2.997881	5	0.770298
**1227**	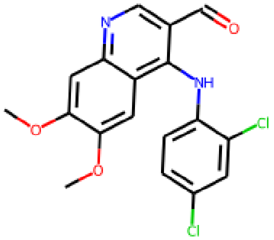	0.552960	COc1cc2ncc(C(=O)O)c(Nc3cccc(Cl)c3Cl)c2cc1OC1C(C)C1	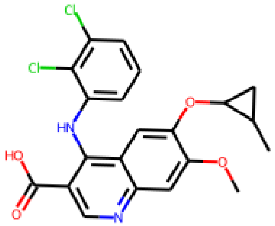	2.0	0.623362	2.525411	2.997881	4	0.774857

	src known mol	similarity	smiles	mol	Class	qed	logP	SAS	rings	kinase inhibition likeliness
**1228**	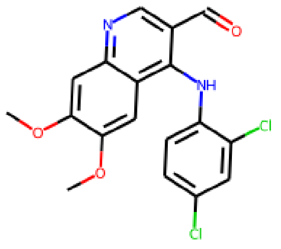	0.552863	COc1cc2ncc(C(=O)O)c(Nc3cccc(Cl)c3Cl)c2cc1OC1C(C)C1	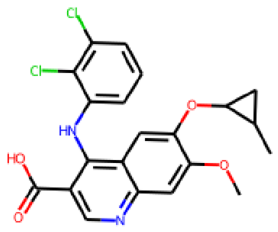	2.0	0.623362	2.525411	2.997881	4	0.774857
**1229**	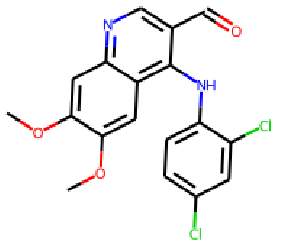	0.552863	COc1cc2ncc(C(=O)O)c(Nc3cccc(Cl)c3Cl)c2cc1OC1C(C)C1	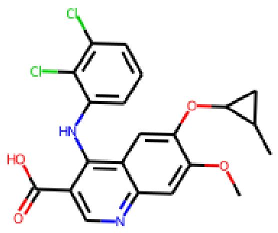	2.0	0.623362	2.525411	2.997881	4	0.774857
**1230**	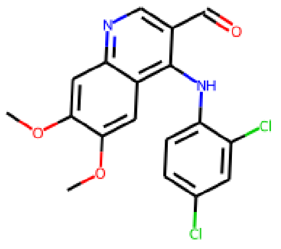	0.552833	COc1cc2ncc(C(=O)O)c(Nc3cccc(Cl)c3Cl)c2cc1OC1C(C)C1	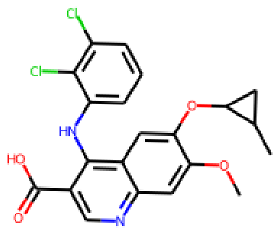	2.0	0.623362	2.525411	2.997881	4	0.774857
**1231**	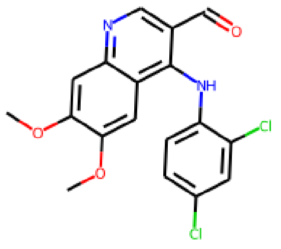	0.552833	COc1cc2ncc(C(=O)O)c(Nc3cccc(Cl)c3Cl)c2cc1OC1C(C)C1	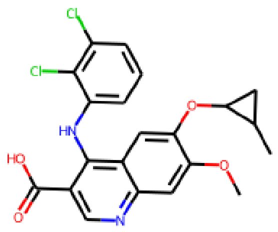	2.0	0.623362	2.525411	2.997881	4	0.774857

	src known mol	similarity	smiles	mol	Class	qed	logP	SAS	rings	kinase inhibition likeliness
**1232**	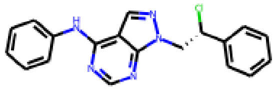	0.552795	Cc1ccc(C(=O)Nc2ccon2)cc1Nc1ncnc2c1cnn2C1cccccc1	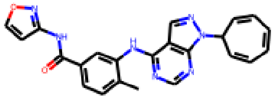	8.0	0.623362	2.525411	2.997881	5	0.770298
**1233**	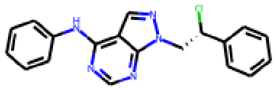	0.552795	Cc1ccc(C(=O)Nc2ccon2)cc1Nc1ncnc2c1cnn2C1cccccc1	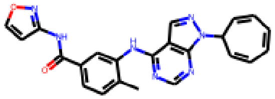	8.0	0.623362	2.525411	2.997881	5	0.770298
**1234**	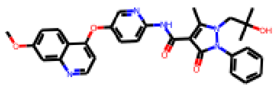	0.552785	COc1ccc2c(=O)cc(CNC(=O)c3c(C)n(C)n(-c4ccccc4)c3=O)nc3cccc2c3c1	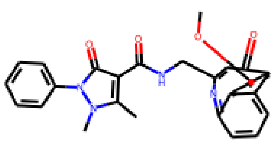	9.0	0.638163	3.995589	2.988471	5	0.774651
**1235**	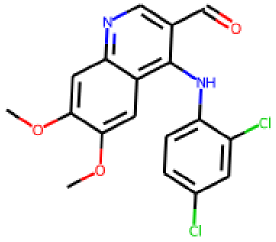	0.552573	COc1cc2ncc(C(=O)O)c(Nc3cccc(Cl)c3Cl)c2cc1OC1C(C)C1	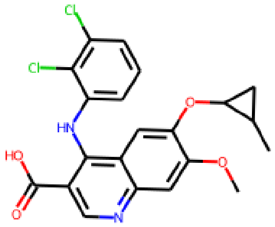	2.0	0.623362	2.525411	2.997881	4	0.774857

	src known mol	similarity	smiles	mol	Class	qed	logP	SAS	rings	kinase inhibition likeliness
**1236**	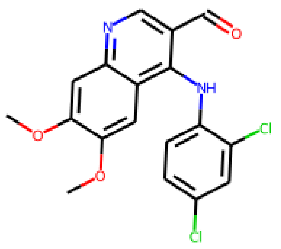	0.552573	COc1cc2ncc(C(=O)O)c(Nc3cccc(Cl)c3Cl)c2cc1OC1C(C)C1	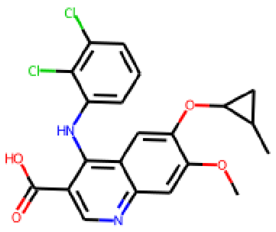	2.0	0.623362	2.525411	2.997881	4	0.774857
**1237**	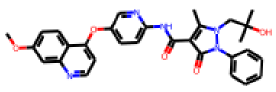	0.552548	COc1ccc2c(=O)cc(CNC(=O)c3c(C)n(C)n(-c4ccccc4)c3=O)nc3cccc2c3c1	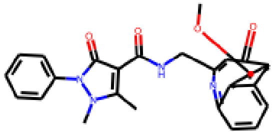	9.0	0.638163	3.995589	2.988471	5	0.774651
**1238**	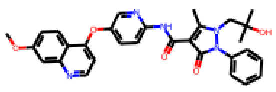	0.552548	COc1ccc2c(=O)cc(CNC(=O)c3c(C)n(C)n(-c4ccccc4)c3=O)nc3cccc2c3c1	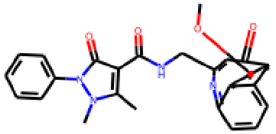	9.0	0.638163	3.995589	2.988471	5	0.774651
**1239**	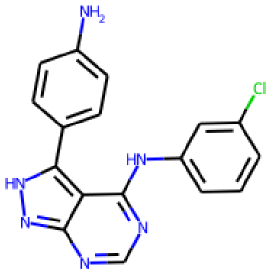	0.552432	c1ccc(Nc2ncnc3n[nH]c(-c4ccccc4)c23)cc1	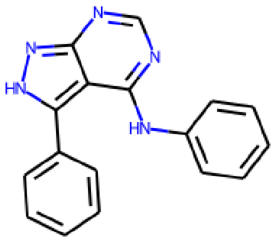	0.0	0.638163	3.995589	2.988471	4	0.800634

	src known mol	similarity	smiles	mol	Class	qed	logP	SAS	rings	kinase inhibition likeliness
**1240**	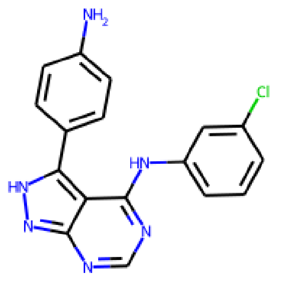	0.552432	c1ccc(Nc2ncnc3n[nH]c(-c4ccccc4)c23)cc1	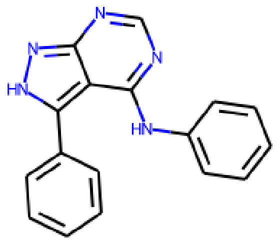	0.0	0.638163	3.995589	2.988471	4	0.800634
**1241**	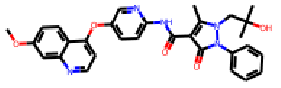	0.552000	COc1ccc2c(=O)cc(CNC(=O)c3c(C)n(C)n(-c4ccccc4)c3=O)nc3cccc2c3c1	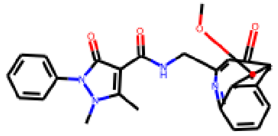	9.0	0.638163	3.995589	2.988471	5	0.774651
**1242**	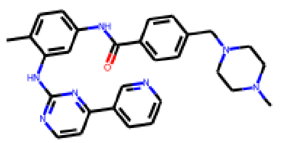	0.551908	Cc1ccc(NC(=O)c2ccccc2)cc1Nc1nccc(-c2cccnc2)n1	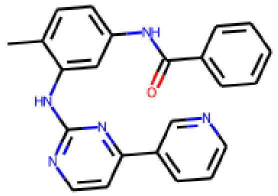	0.0	0.768132	3.608437	2.513170	4	0.854555
**1243**	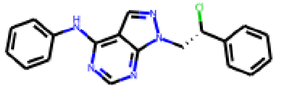	0.551790	Cc1ccc(C(=O)Nc2ccon2)cc1Nc1ncnc2c1cnn2C1cccccc1	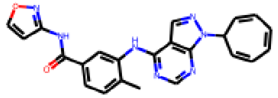	8.0	0.623362	2.525411	2.997881	5	0.770298

	src known mol	similarity	smiles	mol	Class	qed	logP	SAS	rings	kinase inhibition likeliness
**1244**	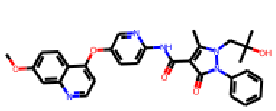	0.551337	COc1ccc2c(=O)cc(CNC(=O)c3c(C)n(C)n(-c4ccccc4)c3=O)nc3cccc2c3c1	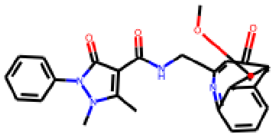	9.0	0.638163	3.995589	2.988471	5	0.774651
**1245**	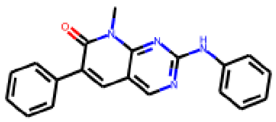	0.551313	Clc1cccc(Nc2ncc3cc(-c3c(Cl)cccc3Cl)c(=O)n(C)c3n2)c1	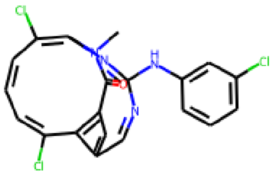	2.0	0.768132	3.608437	2.513170	5	0.784634
**1246**	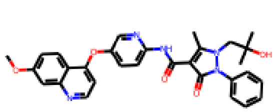	0.551309	COc1ccc2c(=O)cc(CNC(=O)c3c(C)n(C)n(-c4ccccc4)c3=O)nc3cccc2c3c1	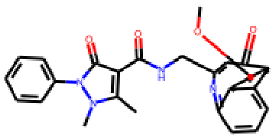	9.0	0.638163	3.995589	2.988471	5	0.774651
**1247**	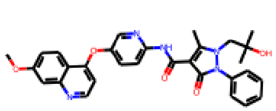	0.551309	COc1ccc2c(=O)cc(CNC(=O)c3c(C)n(C)n(-c4ccccc4)c3=O)nc3cccc2c3c1	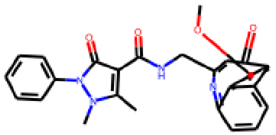	9.0	0.638163	3.995589	2.988471	5	0.774651

	src known mol	similarity	smiles	mol	Class	qed	logP	SAS	rings	kinase inhibition likeliness
**1248**	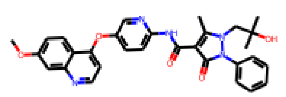	0.551309	COc1ccc2c(=O)cc(CNC(=O)c3c(C)n(C)n(-c4ccccc4)c3=O)nc3cccc2c3c1	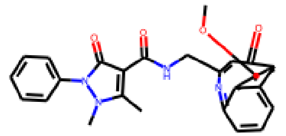	9.0	0.638163	3.995589	2.988471	5	0.774651
**1249**	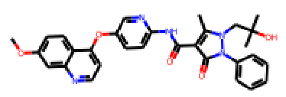	0.551309	COc1ccc2c(=O)cc(CNC(=O)c3c(C)n(C)n(-c4ccccc4)c3=O)nc3cccc2c3c1	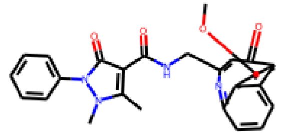	9.0	0.638163	3.995589	2.988471	5	0.774651
**1250**	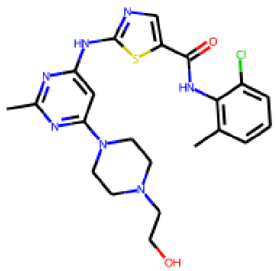	0.551148	Cc1nc(Nc2ncc(C(=O)Nc3c(C)cccc3Cl)s2)cc(N2CCOC(=O)C2)n1	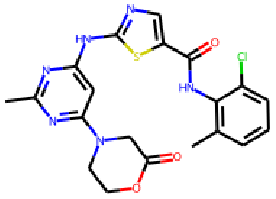	6.0	0.635315	4.542409	3.856266	4	0.801279
**1251**	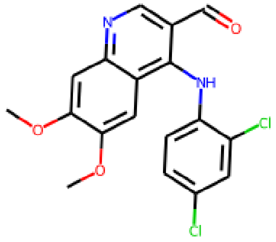	0.550725	COc1cc2ncc(C(=O)O)c(Nc3cccc(Cl)c3Cl)c2cc1OC1C(C)C1	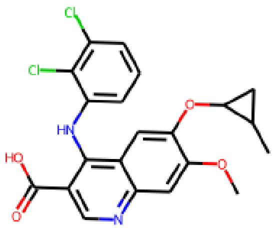	2.0	0.623362	2.525411	2.997881	4	0.774857

	src known mol	similarity	smiles	mol	Class	qed	logP	SAS	rings	kinase inhibition likeliness
**1252**	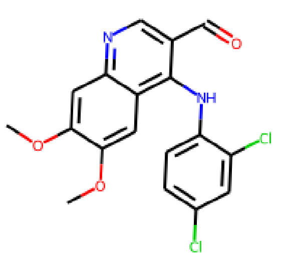	0.550725	COc1cc2ncc(C(=O)O)c(Nc3cccc(Cl)c3Cl)c2cc1OC1C(C)C1	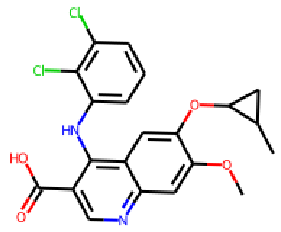	2.0	0.623362	2.525411	2.997881	4	0.774857
**1253**	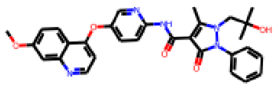	0.550693	COc1ccc2c(=O)cc(CNC(=O)c3c(C)n(C)n(-c4ccccc4)c3=O)nc3cccc3c2n1	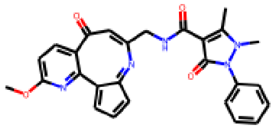	9.0	0.768132	3.608437	2.513170	5	0.823048
**1254**	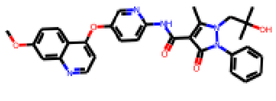	0.550693	COc1ccc2c(=O)cc(CNC(=O)c3c(C)n(C)n(-c4ccccc4)c3=O)nc3cccc3c2n1	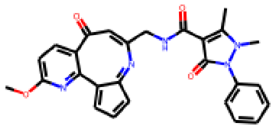	9.0	0.768132	3.608437	2.513170	5	0.823048
**1255**	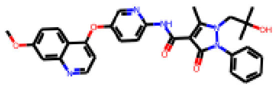	0.550693	COc1ccc2c(=O)cc(CNC(=O)c3c(C)n(C)n(-c4ccccc4)c3=O)nc3cccc3c2n1	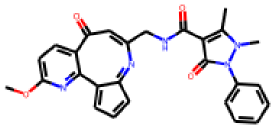	9.0	0.768132	3.608437	2.513170	5	0.823048

	src known mol	similarity	smiles	mol	Class	qed	logP	SAS	rings	kinase inhibition likeliness
**1256**	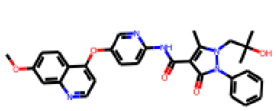	0.550693	COc1ccc2c(=O)cc(CNC(=O)c3c(C)n(C)n(-c4ccccc4)c3=O)nc3cccc3c2n1	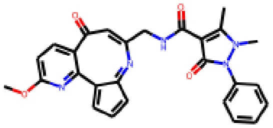	9.0	0.768132	3.608437	2.513170	5	0.823048
**1257**	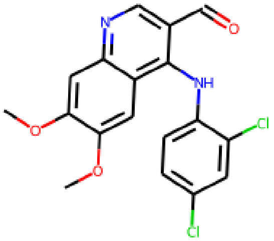	0.550633	COc1cc2ncc(C(=O)O)c(Nc3cccc(Cl)c3Cl)c2cc1OC1C(C)C1	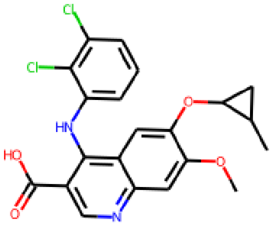	2.0	0.623362	2.525411	2.997881	4	0.774857
**1258**	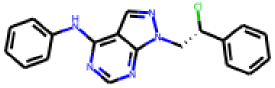	0.550484	Cc1ccc(C(=O)Nc2ccon2)cc1Nc1ncnc2c1cnn2C1cccccc1	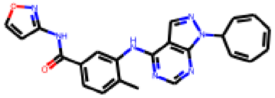	8.0	0.623362	2.525411	2.997881	5	0.770298
**1259**	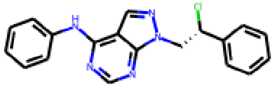	0.550484	Cc1ccc(C(=O)Nc2ccon2)cc1Nc1ncnc2c1cnn2C1cccccc1	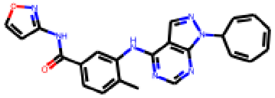	8.0	0.623362	2.525411	2.997881	5	0.770298

	src known mol	similarity	smiles	mol	Class	qed	logP	SAS	rings	kinase inhibition likeliness
**1260**	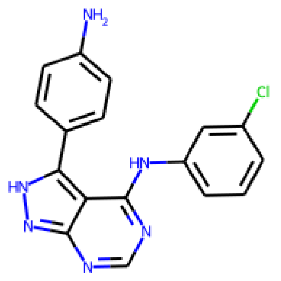	0.550382	c1ccc(Nc2ncnc3n[nH]c(-c4ccccc4)c23)cc1	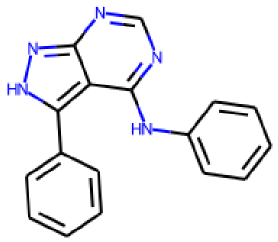	0.0	0.638163	3.995589	2.988471	4	0.800634
**1261**	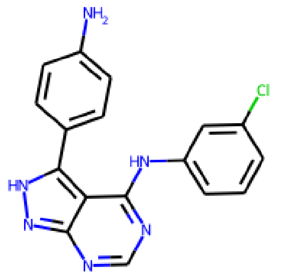	0.550382	c1ccc(Nc2ncnc3n[nH]c(-c4ccccc4)c23)cc1	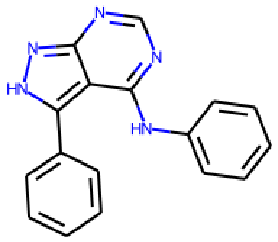	0.0	0.638163	3.995589	2.988471	4	0.800634
**1262**	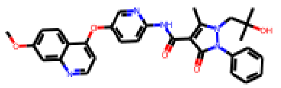	0.550107	COc1ccc2c(=O)cc(CNC(=O)c3c(C)n(C)n(-c4ccccc4)c3=O)nc3cccc2c3c1	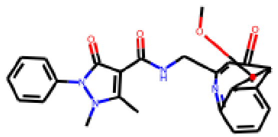	9.0	0.638163	3.995589	2.988471	5	0.774651
**1263**	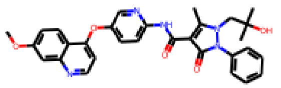	0.550107	COc1ccc2c(=O)cc(CNC(=O)c3c(C)n(C)n(-c4ccccc4)c3=O)nc3cccc2c3c1	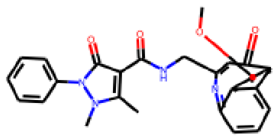	9.0	0.638163	3.995589	2.988471	5	0.774651

	src known mol	similarity	smiles	mol	Class	qed	logP	SAS	rings	kinase inhibition likeliness
**1264**	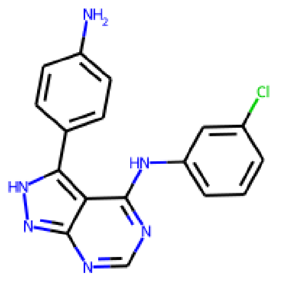	0.550000	c1ccc(Nc2ncnc3n[nH]c(-c4ccccc4)c23)cc1	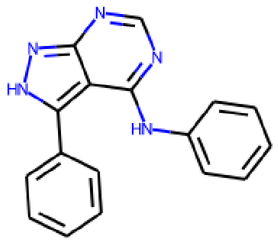	0.0	0.638163	3.995589	2.988471	4	0.800634
**1265**	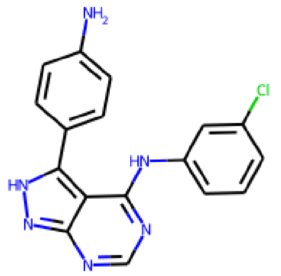	0.550000	c1ccc(Nc2ncnc3n[nH]c(-c4ccccc4)c23)cc1	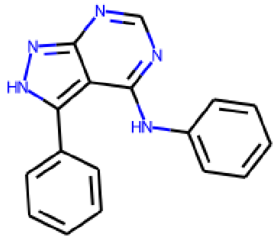	0.0	0.638163	3.995589	2.988471	4	0.800634
**1266**	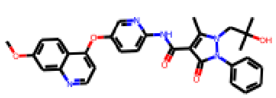	0.549945	COc1ccc2c(=O)cc(CNC(=O)c3c(C)n(C)n(-c4ccccc4)c3=O)nc3cccc3c2n1	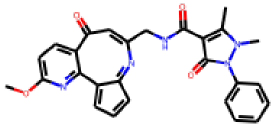	9.0	0.768132	3.608437	2.513170	5	0.823048
**1267**	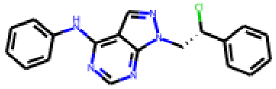	0.549927	Cc1ccc(C(=O)Nc2ccon2)cc1Nc1ncnc2c1cnn2C1cccccc1	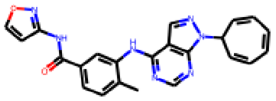	8.0	0.623362	2.525411	2.997881	5	0.770298

	src known mol	similarity	smiles	mol	Class	qed	logP	SAS	rings	kinase inhibition likeliness
**1268**	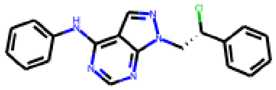	0.549927	Cc1ccc(C(=O)Nc2ccon2)cc1Nc1ncnc2c1cnn2C1cccccc1	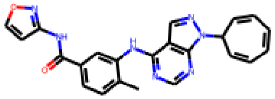	8.0	0.623362	2.525411	2.997881	5	0.770298
**1269**	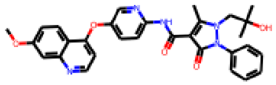	0.549841	COc1ccc2c(=O)cc(CNC(=O)c3c(C)n(C)n(-c4ccccc4)c3=O)nc3cccc2c3c1	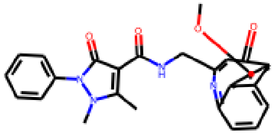	9.0	0.638163	3.995589	2.988471	5	0.774651
**1270**	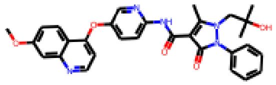	0.549841	COc1ccc2c(=O)cc(CNC(=O)c3c(C)n(C)n(-c4ccccc4)c3=O)nc3cccc2c3c1	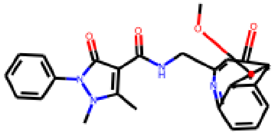	9.0	0.638163	3.995589	2.988471	5	0.774651
**1271**	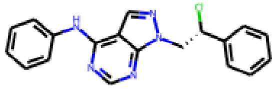	0.549630	Cc1ccc(C(=O)Nc2ccon2)cc1Nc1ncnc2c1cnn2C1cccccc1	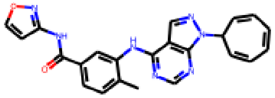	8.0	0.623362	2.525411	2.997881	5	0.770298

	src known mol	similarity	smiles	mol	Class	qed	logP	SAS	rings	kinase inhibition likeliness
**1272**	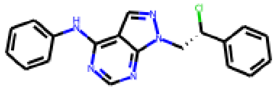	0.549630	Cc1ccc(C(=O)Nc2ccon2)cc1Nc1ncnc2c1cnn2C1cccccc1	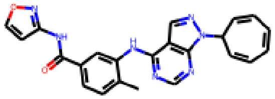	8.0	0.623362	2.525411	2.997881	5	0.770298
**1273**	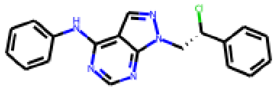	0.549630	Cc1ccc(C(=O)Nc2ccon2)cc1Nc1ncnc2c1cnn2C1cccccc1	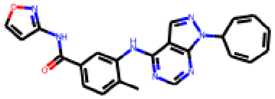	8.0	0.623362	2.525411	2.997881	5	0.770298
**1274**	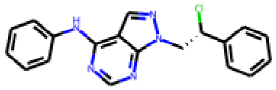	0.549630	Cc1ccc(C(=O)Nc2ccon2)cc1Nc1ncnc2c1cnn2C1cccccc1	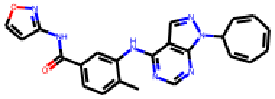	8.0	0.623362	2.525411	2.997881	5	0.770298
**1275**	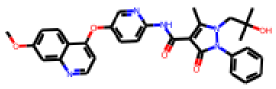	0.549602	COc1ccc2c(=O)cc(CNC(=O)c3c(C)n(C)n(-c4ccccc4)c3=O)nc3cccc2c3c1	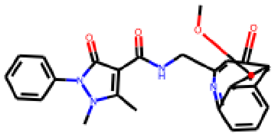	9.0	0.638163	3.995589	2.988471	5	0.774651

	src known mol	similarity	smiles	mol	Class	qed	logP	SAS	rings	kinase inhibition likeliness
**1276**	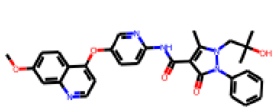	0.549416	COc1ccc2c(=O)cc(CNC(=O)c3c(C)n(C)n(-c4ccccc4)c3=O)nc3cccc2c3c1	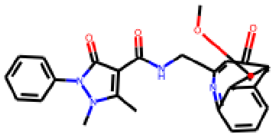	9.0	0.638163	3.995589	2.988471	5	0.774651
**1277**	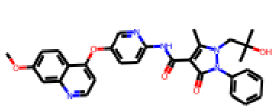	0.549416	COc1ccc2c(=O)cc(CNC(=O)c3c(C)n(C)n(-c4ccccc4)c3=O)nc3cccc2c3c1	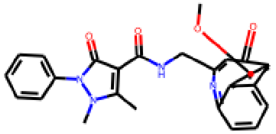	9.0	0.638163	3.995589	2.988471	5	0.774651
**1278**	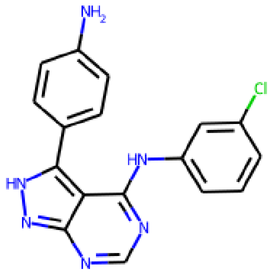	0.549392	c1ccc(Nc2ncnc3n[nH]c(-c4ccccc4)c23)cc1	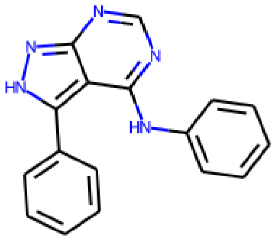	0.0	0.638163	3.995589	2.988471	4	0.800634
**1279**	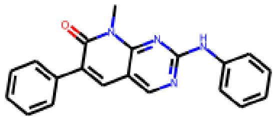	0.549251	Clc1cccc(Nc2ncc3cc(-c3c(Cl)cccc3Cl)c(=O)n(C)c3n2)c1	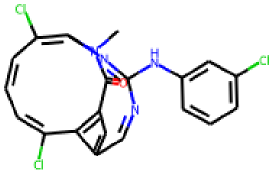	2.0	0.768132	3.608437	2.513170	5	0.784634

	src known mol	similarity	smiles	mol	Class	qed	logP	SAS	rings	kinase inhibition likeliness
**1280**	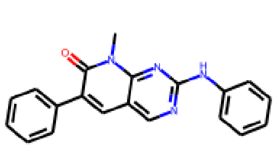	0.549251	Clc1cccc(Nc2ncc3cc(-c3c(Cl)cccc3Cl)c(=O)n(C)c3n2)c1	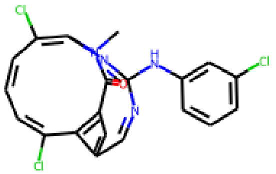	2.0	0.768132	3.608437	2.513170	5	0.784634
**1281**	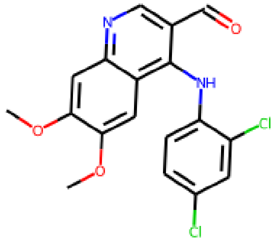	0.549192	COc1cc2ncc(C(=O)O)c(Nc3cccc(Cl)c3Cl)c2cc1OC1C(C)C1	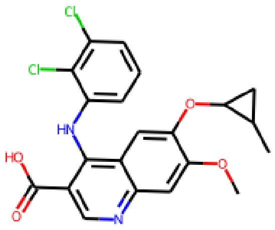	2.0	0.623362	2.525411	2.997881	4	0.774857
**1282**	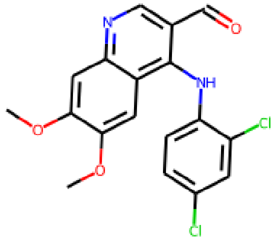	0.549192	COc1cc2ncc(C(=O)O)c(Nc3cccc(Cl)c3Cl)c2cc1OC1C(C)C1	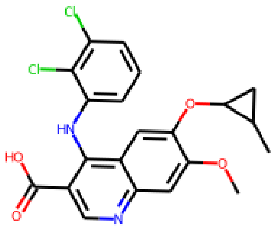	2.0	0.623362	2.525411	2.997881	4	0.774857
**1283**	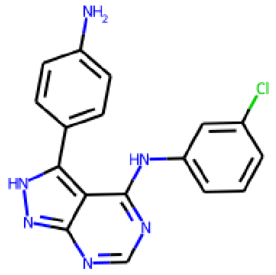	0.549168	c1ccc(Nc2ncnc3n[nH]c(-c4ccccc4)c23)cc1	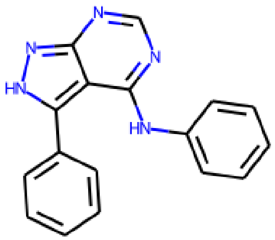	0.0	0.638163	3.995589	2.988471	4	0.800634

	src known mol	similarity	smiles	mol	Class	qed	logP	SAS	rings	kinase inhibition likeliness
**1284**	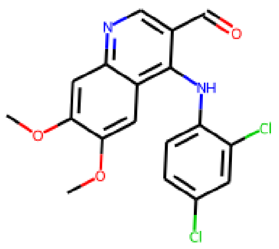	0.549133	COc1cc2ncc(C(=O)O)c(Nc3cccc(Cl)c3Cl)c2cc1OC1C(C)C1	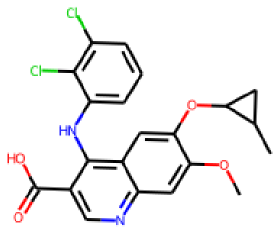	2.0	0.623362	2.525411	2.997881	4	0.774857
**1285**	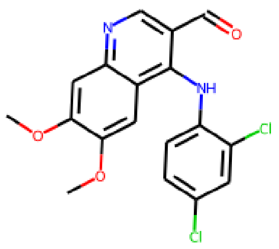	0.549133	COc1cc2ncc(C(=O)O)c(Nc3cccc(Cl)c3Cl)c2cc1OC1C(C)C1	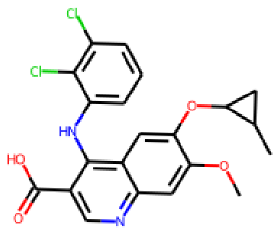	2.0	0.623362	2.525411	2.997881	4	0.774857
**1286**	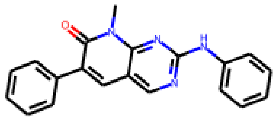	0.549082	Clc1cccc(Nc2ncc3cc(-c3c(Cl)cccc3Cl)c(=O)n(C)c3n2)c1	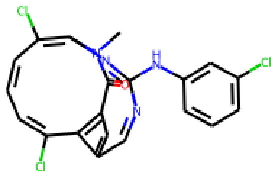	2.0	0.768132	3.608437	2.513170	5	0.784634
**1287**	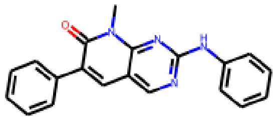	0.549082	Clc1cccc(Nc2ncc3cc(-c3c(Cl)cccc3Cl)c(=O)n(C)c3n2)c1	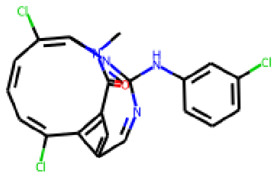	2.0	0.768132	3.608437	2.513170	5	0.784634

	src known mol	similarity	smiles	mol	Class	qed	logP	SAS	rings	kinase inhibition likeliness
**1288**	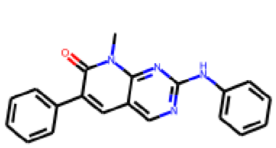	0.549082	Clc1cccc(Nc2ncc3cc(-c3c(Cl)cccc3Cl)c(=O)n(C)c3n2)c1	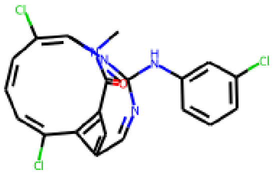	2.0	0.768132	3.608437	2.513170	5	0.784634
**1289**	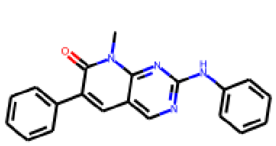	0.549082	Clc1cccc(Nc2ncc3cc(-c3c(Cl)cccc3Cl)c(=O)n(C)c3n2)c1	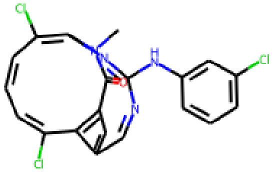	2.0	0.768132	3.608437	2.513170	5	0.784634
**1290**	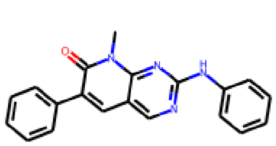	0.549082	Clc1cccc(Nc2ncc3cc(-c3c(Cl)cccc3Cl)c(=O)n(C)c3n2)c1	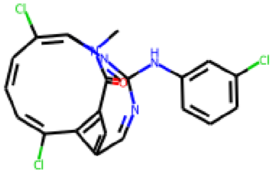	2.0	0.768132	3.608437	2.513170	5	0.784634
**1291**	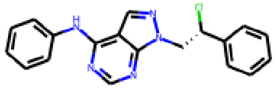	0.548939	Cc1ccc(C(=O)Nc2ccon2)cc1Nc1ncnc2c1cnn2C1cccccc1	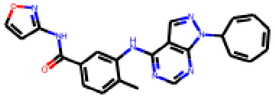	8.0	0.623362	2.525411	2.997881	5	0.770298

	src known mol	similarity	smiles	mol	Class	qed	logP	SAS	rings	kinase inhibition likeliness
**1292**	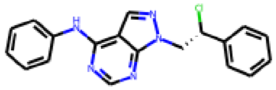	0.548939	Cc1ccc(C(=O)Nc2ccon2)cc1Nc1ncnc2c1cnn2C1cccccc1	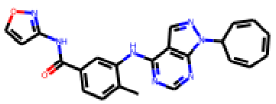	8.0	0.623362	2.525411	2.997881	5	0.770298
**1293**	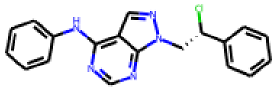	0.548939	Cc1ccc(C(=O)Nc2ccon2)cc1Nc1ncnc2c1cnn2C1cccccc1	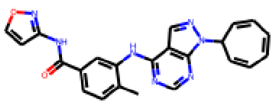	8.0	0.623362	2.525411	2.997881	5	0.770298
**1294**	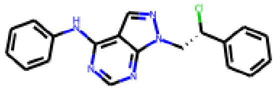	0.548939	Cc1ccc(C(=O)Nc2ccon2)cc1Nc1ncnc2c1cnn2C1cccccc1	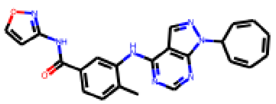	8.0	0.623362	2.525411	2.997881	5	0.770298
**1295**	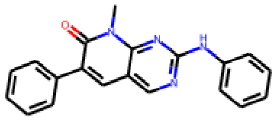	0.548926	Clc1cccc(Nc2ncc3cc(-c3c(Cl)cccc3Cl)c(=O)n(C)c3n2)c1	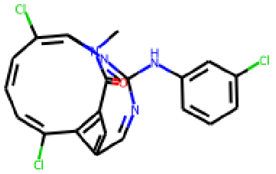	2.0	0.768132	3.608437	2.513170	5	0.784634

	src known mol	similarity	smiles	mol	Class	qed	logP	SAS	rings	kinase inhibition likeliness
**1296**	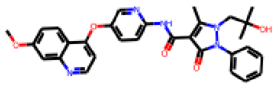	0.548813	COc1ccc2c(=O)cc(CNC(=O)c3c(C)n(C)n(-c4ccccc4)c3=O)nc3cccc2c3c1	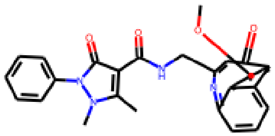	9.0	0.638163	3.995589	2.988471	5	0.774651
**1297**	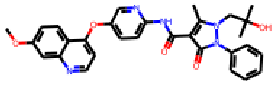	0.548800	COc1ccc2c(=O)cc(CNC(=O)c3c(C)n(C)n(-c4ccccc4)c3=O)nc3cccc2c3c1	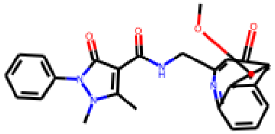	9.0	0.638163	3.995589	2.988471	5	0.774651
**1298**	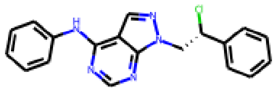	0.548754	Cc1ccc(C(=O)Nc2ccon2)cc1Nc1ncnc2c1cnn2C1cccccc1	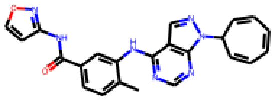	8.0	0.623362	2.525411	2.997881	5	0.770298
**1299**	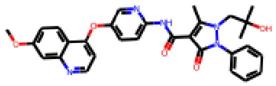	0.548691	COc1ccc2c(=O)cc(CNC(=O)c3c(C)n(C)n(-c4ccccc4)c3=O)nc3cccc2c3c1	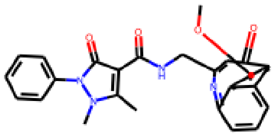	9.0	0.638163	3.995589	2.988471	5	0.774651

	src known mol	similarity	smiles	mol	Class	qed	logP	SAS	rings	kinase inhibition likeliness
**1300**	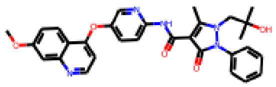	0.548691	COc1ccc2c(=O)cc(CNC(=O)c3c(C)n(C)n(-c4ccccc4)c3=O)nc3cccc2c3c1	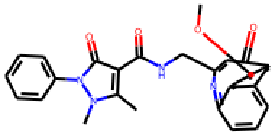	9.0	0.638163	3.995589	2.988471	5	0.774651
**1301**	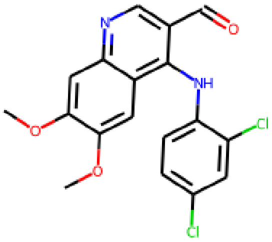	0.548587	COc1cc2ncc(C(=O)O)c(Nc3cccc(Cl)c3Cl)c2cc1OC1C(C)C1	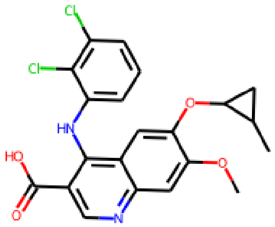	2.0	0.623362	2.525411	2.997881	4	0.774857
**1302**	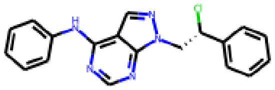	0.548544	Cc1ccc(C(=O)Nc2ccon2)cc1Nc1ncnc2c1cnn2C1cccccc1	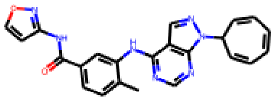	8.0	0.623362	2.525411	2.997881	5	0.770298
**1303**	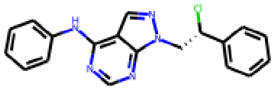	0.548544	Cc1ccc(C(=O)Nc2ccon2)cc1Nc1ncnc2c1cnn2C1cccccc1	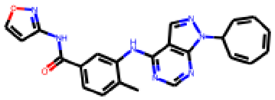	8.0	0.623362	2.525411	2.997881	5	0.770298

	src known mol	similarity	smiles	mol	Class	qed	logP	SAS	rings	kinase inhibition likeliness
**1304**	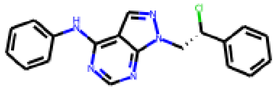	0.548544	Cc1ccc(C(=O)Nc2ccon2)cc1Nc1ncnc2c1cnn2C1cccccc1	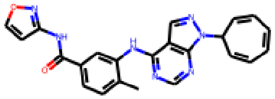	8.0	0.623362	2.525411	2.997881	5	0.770298
**1305**	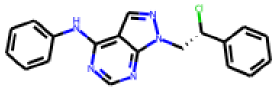	0.548544	Cc1ccc(C(=O)Nc2ccon2)cc1Nc1ncnc2c1cnn2C1cccccc1	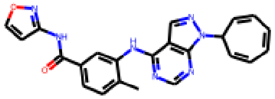	8.0	0.623362	2.525411	2.997881	5	0.770298
**1306**	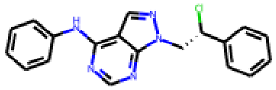	0.548544	Cc1ccc(C(=O)Nc2ccon2)cc1Nc1ncnc2c1cnn2C1cccccc1	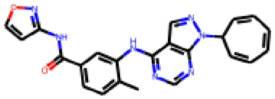	8.0	0.623362	2.525411	2.997881	5	0.770298
**1307**	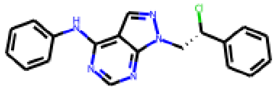	0.548544	Cc1ccc(C(=O)Nc2ccon2)cc1Nc1ncnc2c1cnn2C1cccccc1	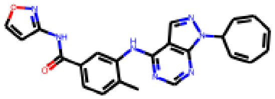	8.0	0.623362	2.525411	2.997881	5	0.770298

	src known mol	similarity	smiles	mol	Class	qed	logP	SAS	rings	kinase inhibition likeliness
**1308**	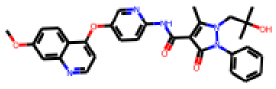	0.548541	COc1ccc2c(=O)cc(CNC(=O)c3c(C)n(C)n(-c4ccccc4)c3=O)nc3cccc2c3c1	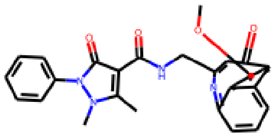	9.0	0.638163	3.995589	2.988471	5	0.774651
**1309**	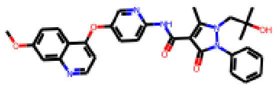	0.548541	COc1ccc2c(=O)cc(CNC(=O)c3c(C)n(C)n(-c4ccccc4)c3=O)nc3cccc2c3c1	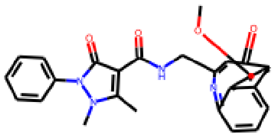	9.0	0.638163	3.995589	2.988471	5	0.774651
**1310**	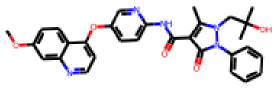	0.548541	COc1ccc2c(=O)cc(CNC(=O)c3c(C)n(C)n(-c4ccccc4)c3=O)nc3cccc2c3c1	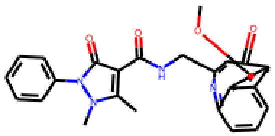	9.0	0.638163	3.995589	2.988471	5	0.774651
**1311**	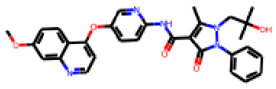	0.548541	COc1ccc2c(=O)cc(CNC(=O)c3c(C)n(C)n(-c4ccccc4)c3=O)nc3cccc2c3c1	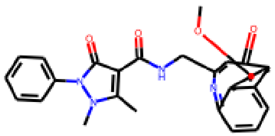	9.0	0.638163	3.995589	2.988471	5	0.774651

	src known mol	similarity	smiles	mol	Class	qed	logP	SAS	rings	kinase inhibition likeliness
**1312**	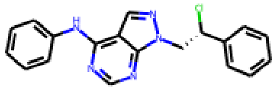	0.548476	Cc1ccc(C(=O)Nc2ccon2)cc1Nc1ncnc2c1cnn2C1cccccc1	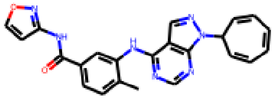	8.0	0.623362	2.525411	2.997881	5	0.770298
**1313**	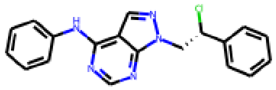	0.548476	Cc1ccc(C(=O)Nc2ccon2)cc1Nc1ncnc2c1cnn2C1cccccc1	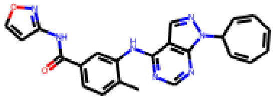	8.0	0.623362	2.525411	2.997881	5	0.770298
**1314**	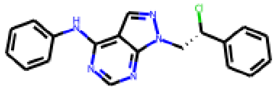	0.548476	Cc1ccc(C(=O)Nc2ccon2)cc1Nc1ncnc2c1cnn2C1cccccc1	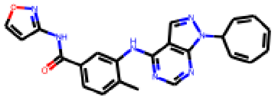	8.0	0.623362	2.525411	2.997881	5	0.770298
**1315**	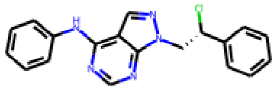	0.548476	Cc1ccc(C(=O)Nc2ccon2)cc1Nc1ncnc2c1cnn2C1cccccc1	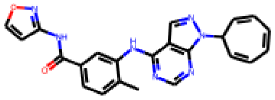	8.0	0.623362	2.525411	2.997881	5	0.770298

	src known mol	similarity	smiles	mol	Class	qed	logP	SAS	rings	kinase inhibition likeliness
**1316**	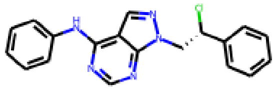	0.548454	Cc1ccc(C(=O)Nc2ccon2)cc1Nc1ncnc2c1cnn2C1cccccc1	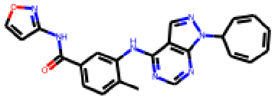	8.0	0.623362	2.525411	2.997881	5	0.770298
**1317**	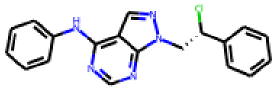	0.548454	Cc1ccc(C(=O)Nc2ccon2)cc1Nc1ncnc2c1cnn2C1cccccc1	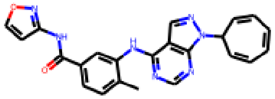	8.0	0.623362	2.525411	2.997881	5	0.770298
**1318**	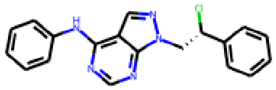	0.548252	Cc1ccc(C(=O)Nc2ccon2)cc1Nc1ncnc2c1cnn2C1cccccc1	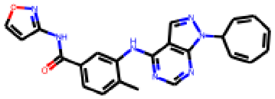	8.0	0.623362	2.525411	2.997881	5	0.770298
**1319**	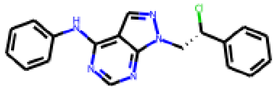	0.548252	Cc1ccc(C(=O)Nc2ccon2)cc1Nc1ncnc2c1cnn2C1cccccc1	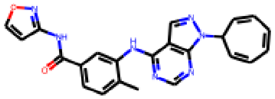	8.0	0.623362	2.525411	2.997881	5	0.770298

	src known mol	similarity	smiles	mol	Class	qed	logP	SAS	rings	kinase inhibition likeliness
**1320**	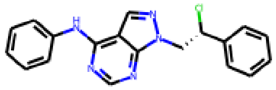	0.548252	Cc1ccc(C(=O)Nc2ccon2)cc1Nc1ncnc2c1cnn2C1cccccc1	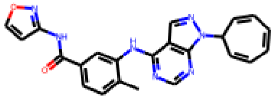	8.0	0.623362	2.525411	2.997881	5	0.770298
**1321**	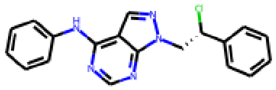	0.548252	Cc1ccc(C(=O)Nc2ccon2)cc1Nc1ncnc2c1cnn2C1cccccc1	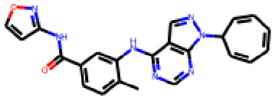	8.0	0.623362	2.525411	2.997881	5	0.770298
**1322**	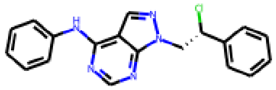	0.548252	Cc1ccc(C(=O)Nc2ccon2)cc1Nc1ncnc2c1cnn2C1cccccc1	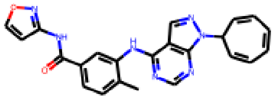	8.0	0.623362	2.525411	2.997881	5	0.770298
**1323**	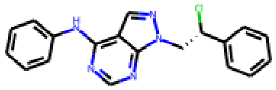	0.548252	Cc1ccc(C(=O)Nc2ccon2)cc1Nc1ncnc2c1cnn2C1cccccc1	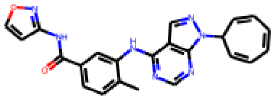	8.0	0.623362	2.525411	2.997881	5	0.770298

	src known mol	similarity	smiles	mol	Class	qed	logP	SAS	rings	kinase inhibition likeliness
**1324**	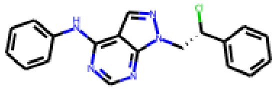	0.548252	Cc1ccc(C(=O)Nc2ccon2)cc1Nc1ncnc2c1cnn2C1cccccc1	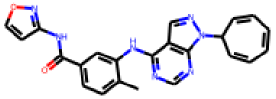	8.0	0.623362	2.525411	2.997881	5	0.770298
**1325**	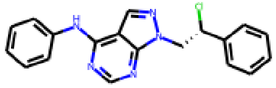	0.548252	Cc1ccc(C(=O)Nc2ccon2)cc1Nc1ncnc2c1cnn2C1cccccc1	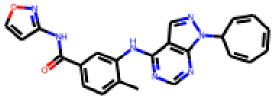	8.0	0.623362	2.525411	2.997881	5	0.770298
**1326**	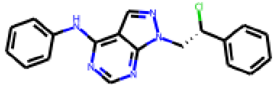	0.548252	Cc1ccc(C(=O)Nc2ccon2)cc1Nc1ncnc2c1cnn2C1cccccc1	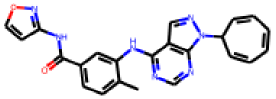	8.0	0.623362	2.525411	2.997881	5	0.770298
**1327**	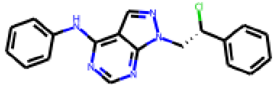	0.548252	Cc1ccc(C(=O)Nc2ccon2)cc1Nc1ncnc2c1cnn2C1cccccc1	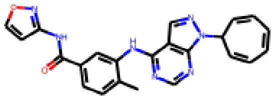	8.0	0.623362	2.525411	2.997881	5	0.770298

	src known mol	similarity	smiles	mol	Class	qed	logP	SAS	rings	kinase inhibition likeliness
**1328**	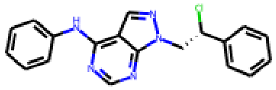	0.548252	Cc1ccc(C(=O)Nc2ccon2)cc1Nc1ncnc2c1cnn2C1cccccc1	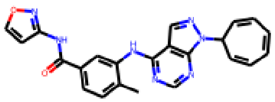	8.0	0.623362	2.525411	2.997881	5	0.770298
**1329**	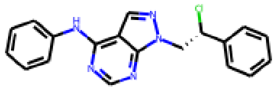	0.548252	Cc1ccc(C(=O)Nc2ccon2)cc1Nc1ncnc2c1cnn2C1cccccc1	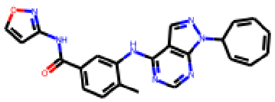	8.0	0.623362	2.525411	2.997881	5	0.770298
**1330**	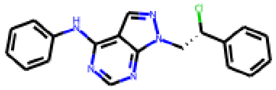	0.548252	Cc1ccc(C(=O)Nc2ccon2)cc1Nc1ncnc2c1cnn2C1cccccc1	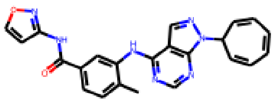	8.0	0.623362	2.525411	2.997881	5	0.770298
**1331**	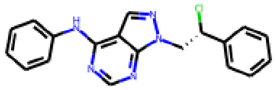	0.548252	Cc1ccc(C(=O)Nc2ccon2)cc1Nc1ncnc2c1cnn2C1cccccc1	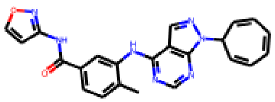	8.0	0.623362	2.525411	2.997881	5	0.770298

	src known mol	similarity	smiles	mol	Class	qed	logP	SAS	rings	kinase inhibition likeliness
**1332**	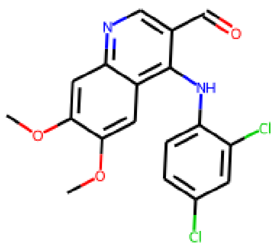	0.548180	COc1cc2ncc(C(=O)O)c(Nc3cccc(Cl)c3Cl)c2cc1OC1C(C)C1	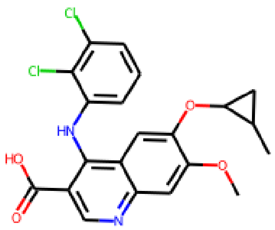	2.0	0.623362	2.525411	2.997881	4	0.774857
**1333**	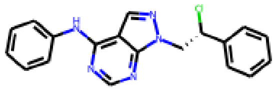	0.548070	Cc1ccc(C(=O)Nc2ccon2)cc1Nc1ncnc2c1cnn2C1cccccc1	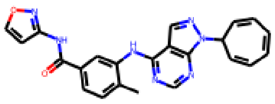	8.0	0.623362	2.525411	2.997881	5	0.770298
**1334**	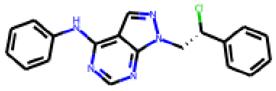	0.548070	Cc1ccc(C(=O)Nc2ccon2)cc1Nc1ncnc2c1cnn2C1cccccc1	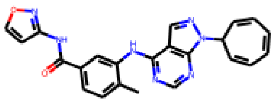	8.0	0.623362	2.525411	2.997881	5	0.770298
**1335**	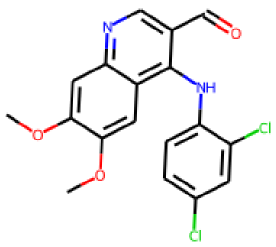	0.548048	COc1cc2ncc(C(=O)O)c(Nc3cccc(Cl)c3Cl)c2cc1OC1C(C)C1	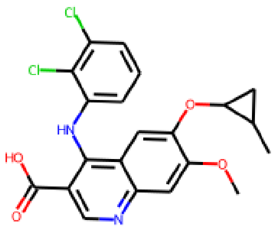	2.0	0.623362	2.525411	2.997881	4	0.774857

	src known mol	similarity	smiles	mol	Class	qed	logP	SAS	rings	kinase inhibition likeliness
**1336**	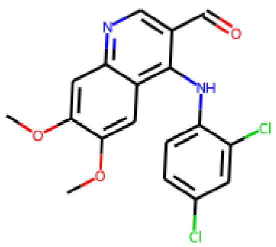	0.547978	COc1cc2ncc(C(=O)O)c(Nc3cccc(Cl)c3Cl)c2cc1OC1C(C)C1	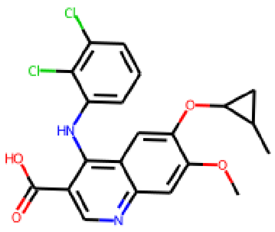	2.0	0.623362	2.525411	2.997881	4	0.774857
**1337**	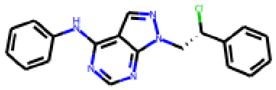	0.547945	Cc1ccc(C(=O)Nc2ccon2)cc1Nc1ncnc2c1cnn2C1cccccc1	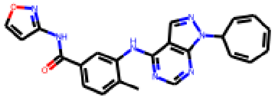	8.0	0.623362	2.525411	2.997881	5	0.770298
**1338**	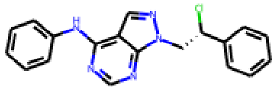	0.547945	Cc1ccc(C(=O)Nc2ccon2)cc1Nc1ncnc2c1cnn2C1cccccc1	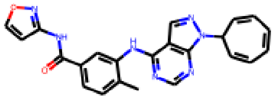	8.0	0.623362	2.525411	2.997881	5	0.770298
**1339**	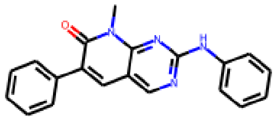	0.547902	Clc1cccc(Nc2ncc3cc(-c3c(Cl)cccc3Cl)c(=O)n(C)c3n2)c1	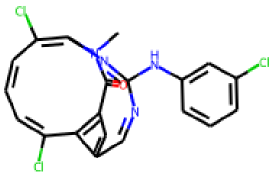	2.0	0.768132	3.608437	2.513170	5	0.784634

	src known mol	similarity	smiles	mol	Class	qed	logP	SAS	rings	kinase inhibition likeliness
**1340**	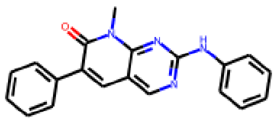	0.547902	Clc1cccc(Nc2ncc3cc(-c3c(Cl)cccc3Cl)c(=O)n(C)c3n2)c1	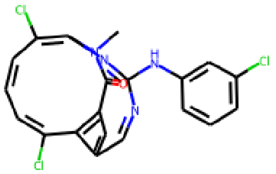	2.0	0.768132	3.608437	2.513170	5	0.784634
**1341**	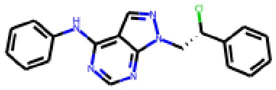	0.547876	Cc1ccc(C(=O)Nc2ccon2)cc1Nc1ncnc2c1cnn2C1cccccc1	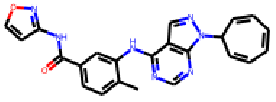	8.0	0.623362	2.525411	2.997881	5	0.770298
**1342**	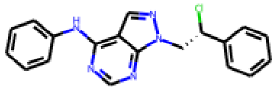	0.547876	Cc1ccc(C(=O)Nc2ccon2)cc1Nc1ncnc2c1cnn2C1cccccc1	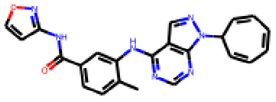	8.0	0.623362	2.525411	2.997881	5	0.770298
**1343**	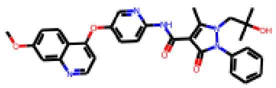	0.547644	COc1ccc2c(=O)cc(CNC(=O)c3c(C)n(C)n(-c4ccccc4)c3=O)nc3cccc2c3c1	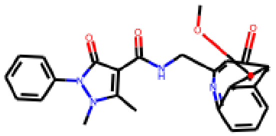	9.0	0.638163	3.995589	2.988471	5	0.774651

	src known mol	similarity	smiles	mol	Class	qed	logP	SAS	rings	kinase inhibition likeliness
**1344**	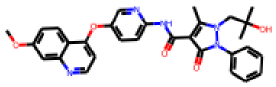	0.547644	COc1ccc2c(=O)cc(CNC(=O)c3c(C)n(C)n(-c4ccccc4)c3=O)nc3cccc2c3c1	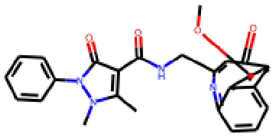	9.0	0.638163	3.995589	2.988471	5	0.774651
**1345**	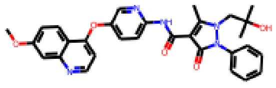	0.547492	COc1ccc2c(=O)cc(CNC(=O)c3c(C)n(C)n(-c4ccccc4)c3=O)nc3cccc2c3c1	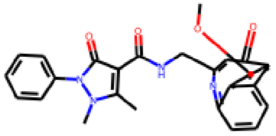	9.0	0.638163	3.995589	2.988471	5	0.774651
**1346**	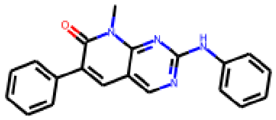	0.547451	Clc1cccc(Nc2ncc3cc(-c3c(Cl)cccc3Cl)c(=O)n(C)c3n2)c1	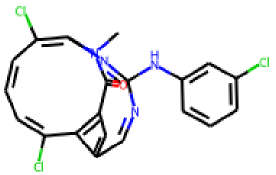	2.0	0.768132	3.608437	2.513170	5	0.784634
**1347**	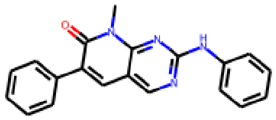	0.547451	Clc1cccc(Nc2ncc3cc(-c3c(Cl)cccc3Cl)c(=O)n(C)c3n2)c1	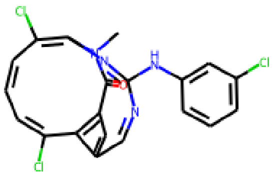	2.0	0.768132	3.608437	2.513170	5	0.784634

	src known mol	similarity	smiles	mol	Class	qed	logP	SAS	rings	kinase inhibition likeliness
**1348**	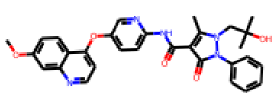	0.547441	COc1ccc2c(=O)cc(CNC(=O)c3c(C)n(C)n(-c4ccccc4)c3=O)nc3cccc2c3c1	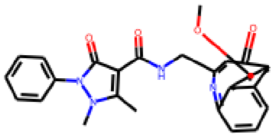	9.0	0.638163	3.995589	2.988471	5	0.774651
**1349**	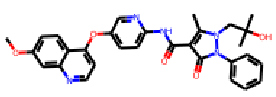	0.547441	COc1ccc2c(=O)cc(CNC(=O)c3c(C)n(C)n(-c4ccccc4)c3=O)nc3cccc2c3c1	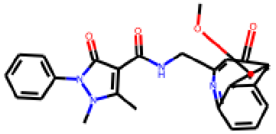	9.0	0.638163	3.995589	2.988471	5	0.774651
**1350**	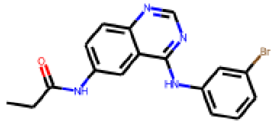	0.547414	O=C(CNC(=O)Oc1ccccc1)Nc1ccc2ncnc(Nc3cccc(Cl)c3)c2c1	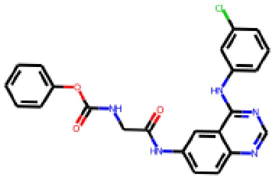	3.0	0.638163	3.995589	2.988471	4	0.793889
**1351**	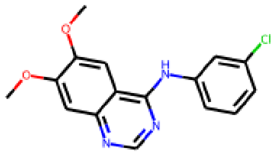	0.547347	Clc1cccc(Nc2ncnc3cc4c[nH]nc4cc23)c1	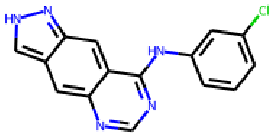	3.0	0.638163	3.995589	2.988471	4	0.781333

	src known mol	similarity	smiles	mol	Class	qed	logP	SAS	rings	kinase inhibition likeliness
**1352**	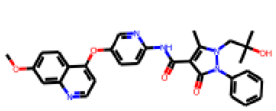	0.547290	COc1ccc2c(=O)cc(CNC(=O)c3c(C)n(C)n(-c4ccccc4)c3=O)nc3cccc2c3c1	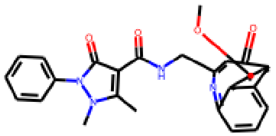	9.0	0.638163	3.995589	2.988471	5	0.774651
**1353**	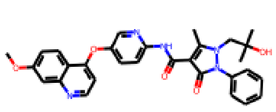	0.547290	COc1ccc2c(=O)cc(CNC(=O)c3c(C)n(C)n(-c4ccccc4)c3=O)nc3cccc2c3c1	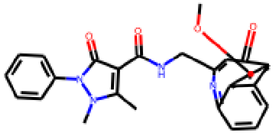	9.0	0.638163	3.995589	2.988471	5	0.774651
**1354**	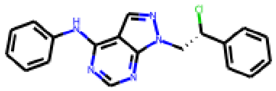	0.547043	Cc1ccc(C(=O)Nc2ccon2)cc1Nc1ncnc2c1cnn2C1cccccc1	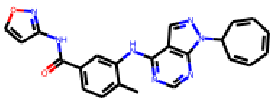	8.0	0.623362	2.525411	2.997881	5	0.770298
**1355**	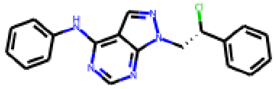	0.547043	Cc1ccc(C(=O)Nc2ccon2)cc1Nc1ncnc2c1cnn2C1cccccc1	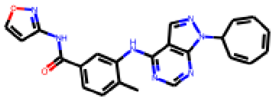	8.0	0.623362	2.525411	2.997881	5	0.770298

	src known mol	similarity	smiles	mol	Class	qed	logP	SAS	rings	kinase inhibition likeliness
**1356**	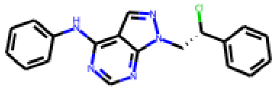	0.546980	Cc1ccc(C(=O)Nc2ccon2)cc1Nc1ncnc2c1cnn2C1cccccc1	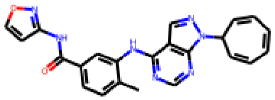	8.0	0.623362	2.525411	2.997881	5	0.770298
**1357**	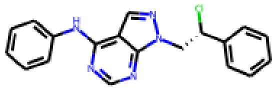	0.546980	Cc1ccc(C(=O)Nc2ccon2)cc1Nc1ncnc2c1cnn2C1cccccc1	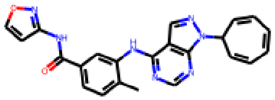	8.0	0.623362	2.525411	2.997881	5	0.770298
**1358**	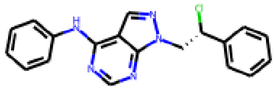	0.546980	Cc1ccc(C(=O)Nc2ccon2)cc1Nc1ncnc2c1cnn2C1cccccc1	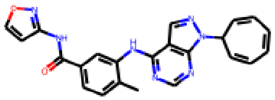	8.0	0.623362	2.525411	2.997881	5	0.770298
**1359**	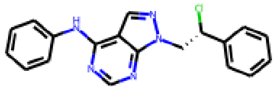	0.546980	Cc1ccc(C(=O)Nc2ccon2)cc1Nc1ncnc2c1cnn2C1cccccc1	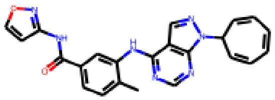	8.0	0.623362	2.525411	2.997881	5	0.770298

	src known mol	similarity	smiles	mol	Class	qed	logP	SAS	rings	kinase inhibition likeliness
**1360**	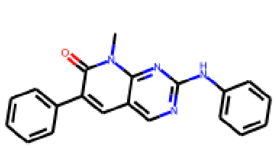	0.546961	Clc1cccc(Nc2ncc3cc(-c3c(Cl)cccc3Cl)c(=O)n(C)c3n2)c1	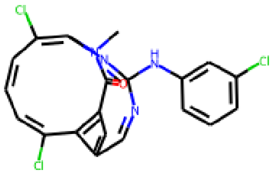	2.0	0.768132	3.608437	2.513170	5	0.784634
**1361**	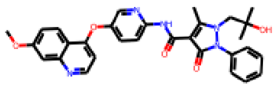	0.546950	COc1ccc2c(=O)cc(CNC(=O)c3c(C)n(C)n(-c4ccccc4)c3=O)nc3cccc2c3c1	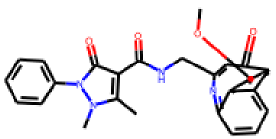	9.0	0.638163	3.995589	2.988471	5	0.774651
**1362**	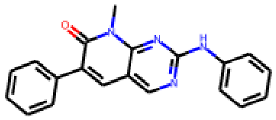	0.546936	Clc1cccc(Nc2ncc3cc(-c3c(Cl)cccc3Cl)c(=O)n(C)c3n2)c1	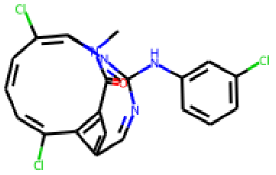	2.0	0.768132	3.608437	2.513170	5	0.784634
**1363**	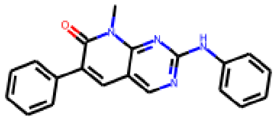	0.546936	Clc1cccc(Nc2ncc3cc(-c3c(Cl)cccc3Cl)c(=O)n(C)c3n2)c1	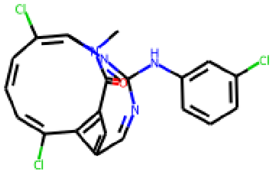	2.0	0.768132	3.608437	2.513170	5	0.784634

	src known mol	similarity	smiles	mol	Class	qed	logP	SAS	rings	kinase inhibition likeliness
**1364**	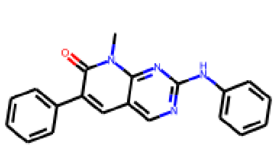	0.546936	Clc1cccc(Nc2ncc3cc(-c3c(Cl)cccc3Cl)c(=O)n(C)c3n2)c1	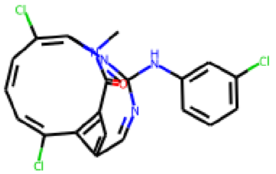	2.0	0.768132	3.608437	2.513170	5	0.784634
**1365**	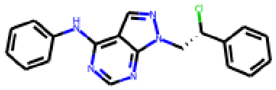	0.546928	Cc1ccc(C(=O)Nc2ccon2)cc1Nc1ncnc2c1cnn2C1cccccc1	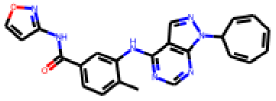	8.0	0.623362	2.525411	2.997881	5	0.770298
**1366**	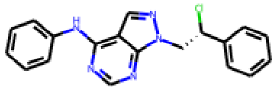	0.546928	Cc1ccc(C(=O)Nc2ccon2)cc1Nc1ncnc2c1cnn2C1cccccc1	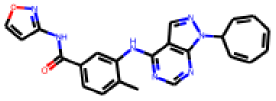	8.0	0.623362	2.525411	2.997881	5	0.770298
**1367**	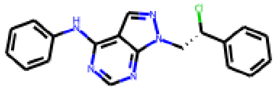	0.546928	Cc1ccc(C(=O)Nc2ccon2)cc1Nc1ncnc2c1cnn2C1cccccc1	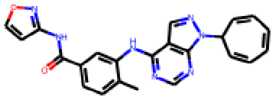	8.0	0.623362	2.525411	2.997881	5	0.770298

	src known mol	similarity	smiles	mol	Class	qed	logP	SAS	rings	kinase inhibition likeliness
**1368**	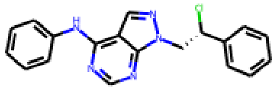	0.546928	Cc1ccc(C(=O)Nc2ccon2)cc1Nc1ncnc2c1cnn2C1cccccc1	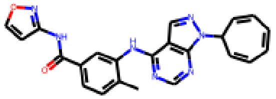	8.0	0.623362	2.525411	2.997881	5	0.770298
**1369**	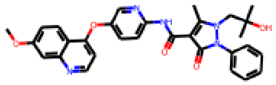	0.546766	COc1ccc2c(=O)cc(CNC(=O)c3c(C)n(C)n(-c4ccccc4)c3=O)nc3cccc2c3c1	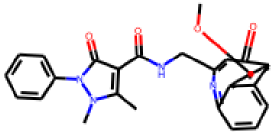	9.0	0.638163	3.995589	2.988471	5	0.774651
**1370**	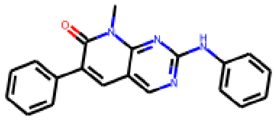	0.546740	Clc1cccc(Nc2ncc3cc(-c3c(Cl)cccc3Cl)c(=O)n(C)c3n2)c1	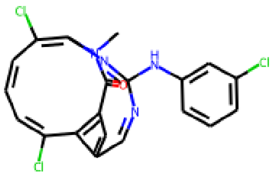	2.0	0.768132	3.608437	2.513170	5	0.784634
**1371**	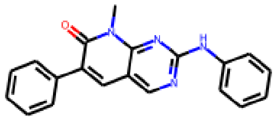	0.546740	Clc1cccc(Nc2ncc3cc(-c3c(Cl)cccc3Cl)c(=O)n(C)c3n2)c1	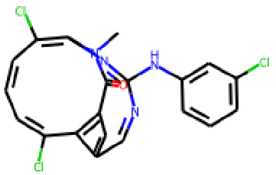	2.0	0.768132	3.608437	2.513170	5	0.784634

	src known mol	similarity	smiles	mol	Class	qed	logP	SAS	rings	kinase inhibition likeliness
**1372**	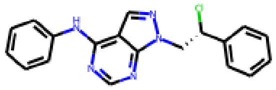	0.546610	Cc1ccc(C(=O)Nc2ccon2)cc1Nc1ncnc2c1cnn2C1cccccc1	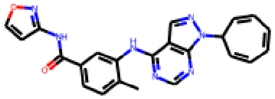	8.0	0.623362	2.525411	2.997881	5	0.770298
**1373**	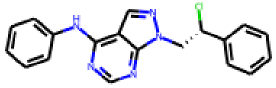	0.546414	Cc1ccc(C(=O)Nc2ccon2)cc1Nc1ncnc2c1cnn2C1cccccc1	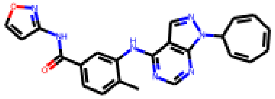	8.0	0.623362	2.525411	2.997881	5	0.770298
**1374**	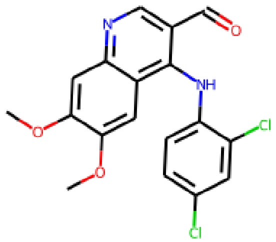	0.546414	COc1cc2ncc(C(=O)O)c(Nc3cccc(Cl)c3Cl)c2cc1OC1C(C)C1	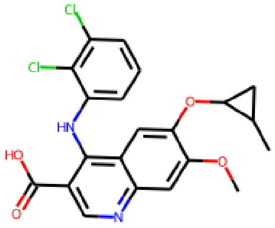	2.0	0.623362	2.525411	2.997881	4	0.774857
**1375**	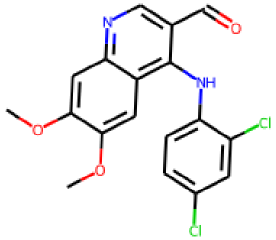	0.546414	COc1cc2ncc(C(=O)O)c(Nc3cccc(Cl)c3Cl)c2cc1OC1C(C)C1	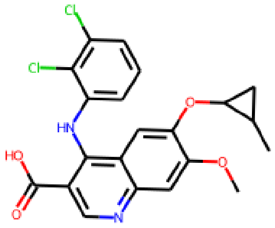	2.0	0.623362	2.525411	2.997881	4	0.774857

	src known mol	similarity	smiles	mol	Class	qed	logP	SAS	rings	kinase inhibition likeliness
**1376**	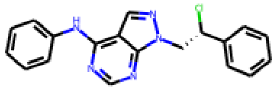	0.546399	Cc1ccc(C(=O)Nc2ccon2)cc1Nc1ncnc2c1cnn2C1cccccc1	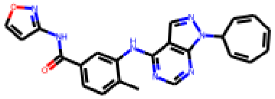	8.0	0.623362	2.525411	2.997881	5	0.770298
**1377**	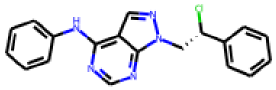	0.546399	Cc1ccc(C(=O)Nc2ccon2)cc1Nc1ncnc2c1cnn2C1cccccc1	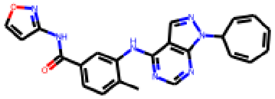	8.0	0.623362	2.525411	2.997881	5	0.770298
**1378**	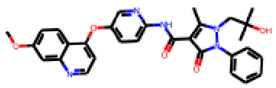	0.546281	COc1ccc2c(=O)cc(CNC(=O)c3c(C)n(C)n(-c4ccccc4)c3=O)nc3cccc2c3c1	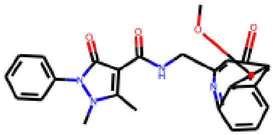	9.0	0.638163	3.995589	2.988471	5	0.774651
**1379**	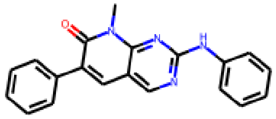	0.546245	Clc1cccc(Nc2ncc3cc(-c3c(Cl)cccc3Cl)c(=O)n(C)c3n2)c1	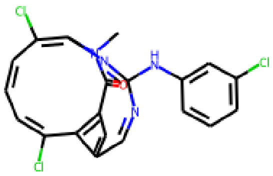	2.0	0.768132	3.608437	2.513170	5	0.784634

	src known mol	similarity	smiles	mol	Class	qed	logP	SAS	rings	kinase inhibition likeliness
**1380**	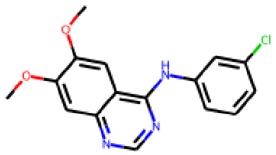	0.546210	Clc1cccc(Nc2ncnc3cc4c[nH]nc4cc23)c1	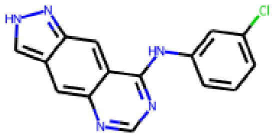	3.0	0.638163	3.995589	2.988471	4	0.781333
**1381**	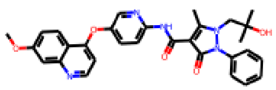	0.546183	COc1ccc2c(=O)cc(CNC(=O)c3c(C)n(C)n(-c4ccccc4)c3=O)nc3cccc2c3c1	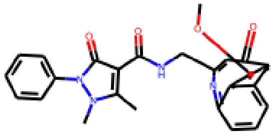	9.0	0.638163	3.995589	2.988471	5	0.774651
**1382**	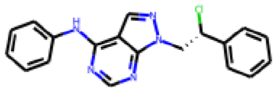	0.546176	Cc1ccc(C(=O)Nc2ccon2)cc1Nc1ncnc2c1cnn2C1cccccc1	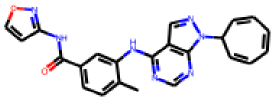	8.0	0.623362	2.525411	2.997881	5	0.770298
**1383**	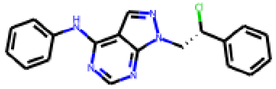	0.546176	Cc1ccc(C(=O)Nc2ccon2)cc1Nc1ncnc2c1cnn2C1cccccc1	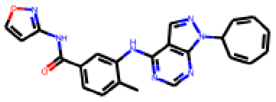	8.0	0.623362	2.525411	2.997881	5	0.770298

	src known mol	similarity	smiles	mol	Class	qed	logP	SAS	rings	kinase inhibition likeliness
**1384**	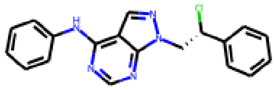	0.546154	Cc1ccc(C(=O)Nc2ccon2)cc1Nc1ncnc2c1cnn2C1cccccc1	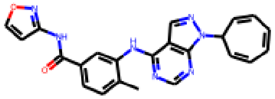	8.0	0.623362	2.525411	2.997881	5	0.770298
**1385**	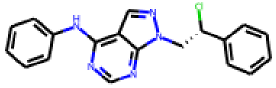	0.545984	Cc1ccc(C(=O)Nc2ccon2)cc1Nc1ncnc2c1cnn2C1cccccc1	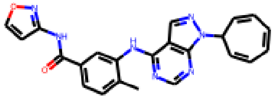	8.0	0.623362	2.525411	2.997881	5	0.770298
**1386**	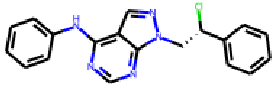	0.545984	Cc1ccc(C(=O)Nc2ccon2)cc1Nc1ncnc2c1cnn2C1cccccc1	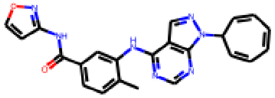	8.0	0.623362	2.525411	2.997881	5	0.770298
**1387**	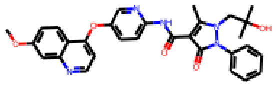	0.545980	COc1ccc2c(=O)cc(CNC(=O)c3c(C)n(C)n(-c4ccccc4)c3=O)nc3cccc2c3c1	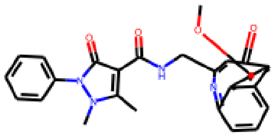	9.0	0.638163	3.995589	2.988471	5	0.774651

	src known mol	similarity	smiles	mol	Class	qed	logP	SAS	rings	kinase inhibition likeliness
**1388**	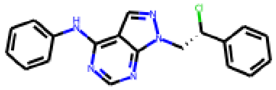	0.545911	Cc1ccc(C(=O)Nc2ccon2)cc1Nc1ncnc2c1cnn2C1cccccc1	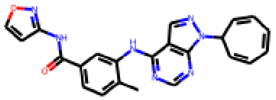	8.0	0.623362	2.525411	2.997881	5	0.770298
**1389**	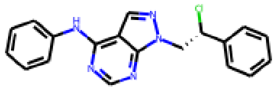	0.545842	Cc1ccc(C(=O)Nc2ccon2)cc1Nc1ncnc2c1cnn2C1cccccc1	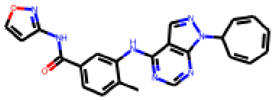	8.0	0.623362	2.525411	2.997881	5	0.770298
**1390**	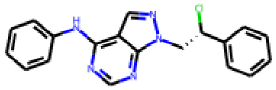	0.545712	Cc1ccc(C(=O)Nc2ccon2)cc1Nc1ncnc2c1cnn2C1cccccc1	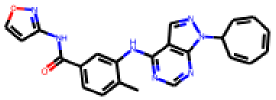	8.0	0.623362	2.525411	2.997881	5	0.770298
**1391**	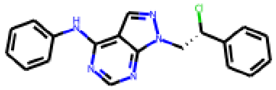	0.545651	Cc1ccc(C(=O)Nc2ccon2)cc1Nc1ncnc2c1cnn2C1cccccc1	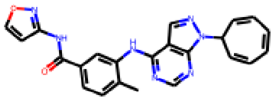	8.0	0.623362	2.525411	2.997881	5	0.770298

	src known mol	similarity	smiles	mol	Class	qed	logP	SAS	rings	kinase inhibition likeliness
**1392**	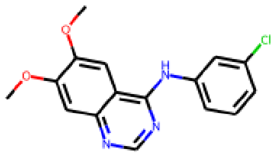	0.545543	Clc1cccc(Nc2ncnc3cc4c[nH]nc4cc23)c1	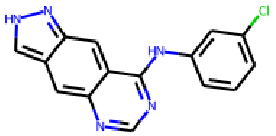	3.0	0.638163	3.995589	2.988471	4	0.781333
**1393**	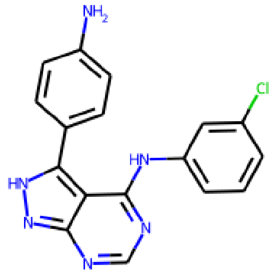	0.545523	c1ccc(Nc2ncnc3n[nH]c(-c4ccccc4)c23)cc1	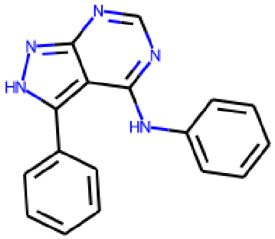	0.0	0.638163	3.995589	2.988471	4	0.800634
**1394**	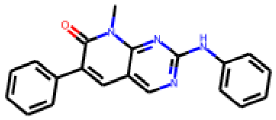	0.545384	Clc1cccc(Nc2ncc3cc(-c3c(Cl)cccc3Cl)c(=O)n(C)c3n2)c1	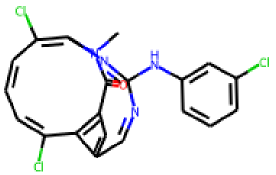	2.0	0.768132	3.608437	2.513170	5	0.784634
**1395**	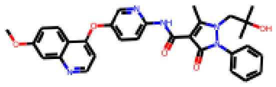	0.545019	COc1ccc2c(=O)cc(CNC(=O)c3c(C)n(C)n(-c4ccccc4)c3=O)nc3cccc2c3c1	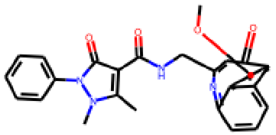	9.0	0.638163	3.995589	2.988471	5	0.774651

	src known mol	similarity	smiles	mol	Class	qed	logP	SAS	rings	kinase inhibition likeliness
**1396**	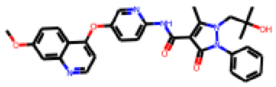	0.545019	COc1ccc2c(=O)cc(CNC(=O)c3c(C)n(C)n(-c4ccccc4)c3=O)nc3cccc2c3c1	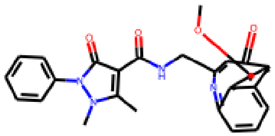	9.0	0.638163	3.995589	2.988471	5	0.774651
**1397**	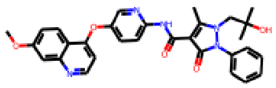	0.545016	COc1ccc2c(=O)cc(CNC(=O)c3c(C)n(C)n(-c4ccccc4)c3=O)nc3cccc2c3c1	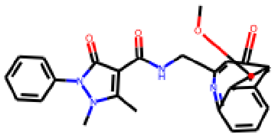	9.0	0.638163	3.995589	2.988471	5	0.774651
**1398**	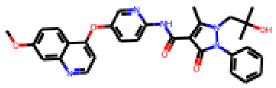	0.545016	COc1ccc2c(=O)cc(CNC(=O)c3c(C)n(C)n(-c4ccccc4)c3=O)nc3cccc2c3c1	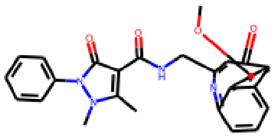	9.0	0.638163	3.995589	2.988471	5	0.774651
**1399**	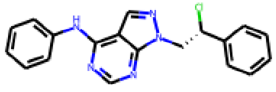	0.544984	Cc1ccc(C(=O)Nc2ccon2)cc1Nc1ncnc2c1cnn2C1cccccc1	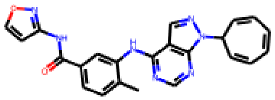	8.0	0.623362	2.525411	2.997881	5	0.770298

	src known mol	similarity	smiles	mol	Class	qed	logP	SAS	rings	kinase inhibition likeliness
**1400**	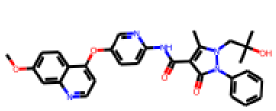	0.544974	COc1ccc2c(=O)cc(CNC(=O)c3c(C)n(C)n(-c4ccccc4)c3=O)nc3cccc2c3c1	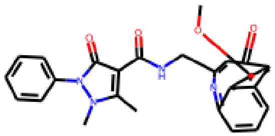	9.0	0.638163	3.995589	2.988471	5	0.774651
**1401**	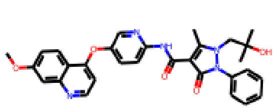	0.544968	COc1ccc2c(=O)cc(CNC(=O)c3c(C)n(C)n(-c4ccccc4)c3=O)nc3cccc2c3c1	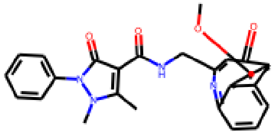	9.0	0.638163	3.995589	2.988471	5	0.774651
**1402**	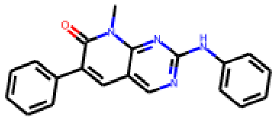	0.544957	Clc1cccc(Nc2ncc3cc(-c3c(Cl)cccc3Cl)c(=O)n(C)c3n2)c1	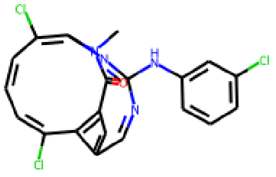	2.0	0.768132	3.608437	2.513170	5	0.784634
**1403**	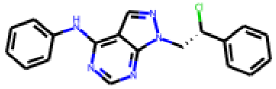	0.544918	Cc1ccc(C(=O)Nc2ccon2)cc1Nc1ncnc2c1cnn2C1cccccc1	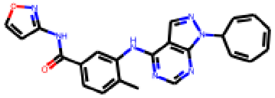	8.0	0.623362	2.525411	2.997881	5	0.770298

	src known mol	similarity	smiles	mol	Class	qed	logP	SAS	rings	kinase inhibition likeliness
**1404**	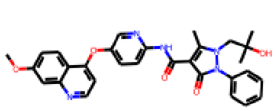	0.544586	COc1ccc2c(=O)cc(CNC(=O)c3c(C)n(C)n(-c4ccccc4)c3=O)nc3cccc2c3c1	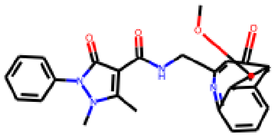	9.0	0.638163	3.995589	2.988471	5	0.774651
**1405**	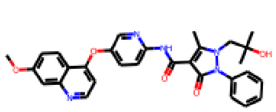	0.544533	COc1ccc2c(=O)cc(CNC(=O)c3c(C)n(C)n(-c4ccccc4)c3=O)nc3cccc2c3c1	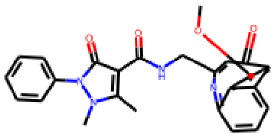	9.0	0.638163	3.995589	2.988471	5	0.774651
**1406**	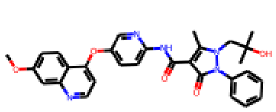	0.544218	COc1ccc2c(=O)cc(CNC(=O)c3c(C)n(C)n(-c4ccccc4)c3=O)nc3cccc2c3c1	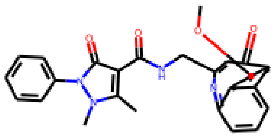	9.0	0.638163	3.995589	2.988471	5	0.774651
**1407**	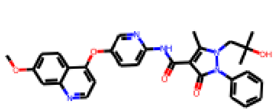	0.544118	COc1ccc2c(=O)cc(CNC(=O)c3c(C)n(C)n(-c4ccccc4)c3=O)nc3cccc2c3c1	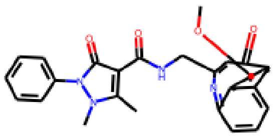	9.0	0.638163	3.995589	2.988471	5	0.774651

	src known mol	similarity	smiles	mol	Class	qed	logP	SAS	rings	kinase inhibition likeliness
**1408**	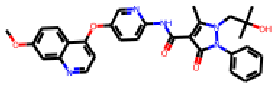	0.544118	COc1ccc2c(=O)cc(CNC(=O)c3c(C)n(C)n(-c4ccccc4)c3=O)nc3cccc2c3c1	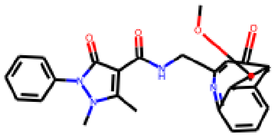	9.0	0.638163	3.995589	2.988471	5	0.774651
**1409**	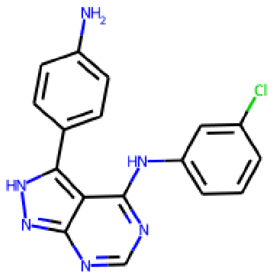	0.544096	c1ccc(Nc2ncnc3n[nH]c(-c4ccccc4)c23)cc1	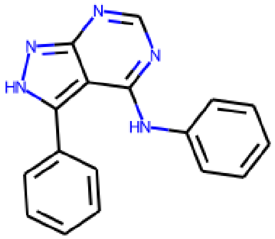	0.0	0.638163	3.995589	2.988471	4	0.800634
**1410**	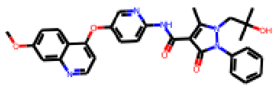	0.544071	COc1ccc2c(=O)cc(CNC(=O)c3c(C)n(C)n(-c4ccccc4)c3=O)nc3cccc2c3c1	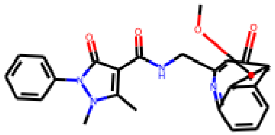	9.0	0.638163	3.995589	2.988471	5	0.774651
**1411**	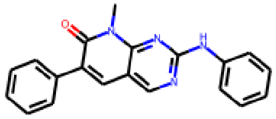	0.544025	Clc1cccc(Nc2ncc3cc(-c3c(Cl)cccc3Cl)c(=O)n(C)c3n2)c1	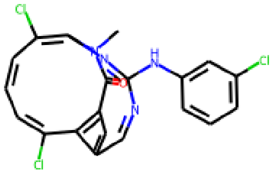	2.0	0.768132	3.608437	2.513170	5	0.784634

	src known mol	similarity	smiles	mol	Class	qed	logP	SAS	rings	kinase inhibition likeliness
**1412**	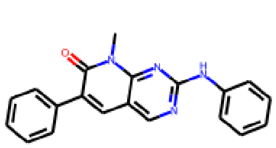	0.544025	Clc1cccc(Nc2ncc3cc(-c3c(Cl)cccc3Cl)c(=O)n(C)c3n2)c1	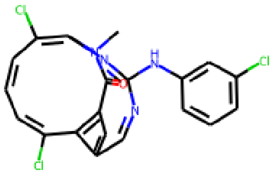	2.0	0.768132	3.608437	2.513170	5	0.784634
**1413**	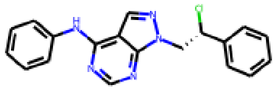	0.544012	Cc1ccc(C(=O)Nc2ccon2)cc1Nc1ncnc2c1cnn2C1cccccc1	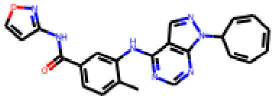	8.0	0.623362	2.525411	2.997881	5	0.770298
**1414**	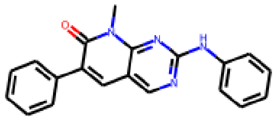	0.543779	Clc1cccc(Nc2ncc3cc(-c3c(Cl)cccc3Cl)c(=O)n(C)c3n2)c1	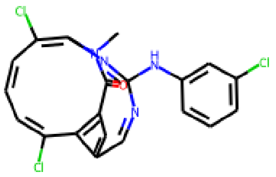	2.0	0.768132	3.608437	2.513170	5	0.784634
**1415**	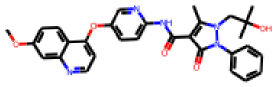	0.543757	COc1ccc2c(=O)cc(CNC(=O)c3c(C)n(C)n(-c4ccccc4)c3=O)nc3cccc2c3c1	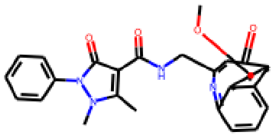	9.0	0.638163	3.995589	2.988471	5	0.774651

	src known mol	similarity	smiles	mol	Class	qed	logP	SAS	rings	kinase inhibition likeliness
**1416**	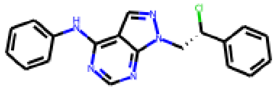	0.543668	Cc1ccc(C(=O)Nc2ccon2)cc1Nc1ncnc2c1cnn2C1cccccc1	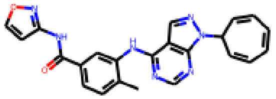	8.0	0.623362	2.525411	2.997881	5	0.770298
**1417**	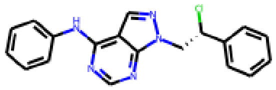	0.543568	Cc1ccc(C(=O)Nc2ccon2)cc1Nc1ncnc2c1cnn2C1cccccc1	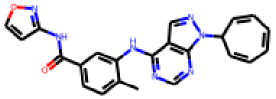	8.0	0.623362	2.525411	2.997881	5	0.770298
**1418**	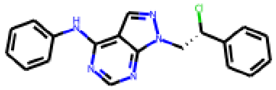	0.543568	Cc1ccc(C(=O)Nc2ccon2)cc1Nc1ncnc2c1cnn2C1cccccc1	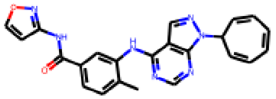	8.0	0.623362	2.525411	2.997881	5	0.770298
**1419**	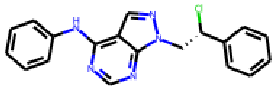	0.543568	Cc1ccc(C(=O)Nc2ccon2)cc1Nc1ncnc2c1cnn2C1cccccc1	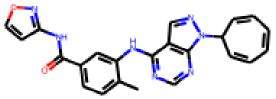	8.0	0.623362	2.525411	2.997881	5	0.770298

	src known mol	similarity	smiles	mol	Class	qed	logP	SAS	rings	kinase inhibition likeliness
**1420**	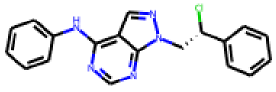	0.543568	Cc1ccc(C(=O)Nc2ccon2)cc1Nc1ncnc2c1cnn2C1cccccc1	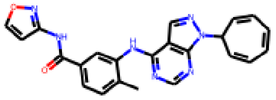	8.0	0.623362	2.525411	2.997881	5	0.770298
**1421**	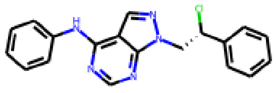	0.543463	Cc1ccc(C(=O)Nc2ccon2)cc1Nc1ncnc2c1cnn2C1cccccc1	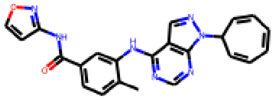	8.0	0.623362	2.525411	2.997881	5	0.770298
**1422**	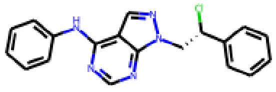	0.543463	Cc1ccc(C(=O)Nc2ccon2)cc1Nc1ncnc2c1cnn2C1cccccc1	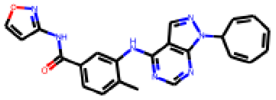	8.0	0.623362	2.525411	2.997881	5	0.770298
**1423**	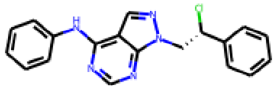	0.543463	Cc1ccc(C(=O)Nc2ccon2)cc1Nc1ncnc2c1cnn2C1cccccc1	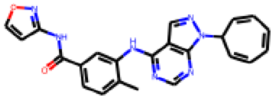	8.0	0.623362	2.525411	2.997881	5	0.770298

	src known mol	similarity	smiles	mol	Class	qed	logP	SAS	rings	kinase inhibition likeliness
**1424**	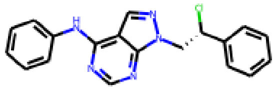	0.543463	Cc1ccc(C(=O)Nc2ccon2)cc1Nc1ncnc2c1cnn2C1cccccc1	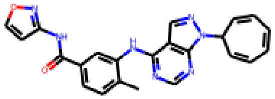	8.0	0.623362	2.525411	2.997881	5	0.770298
**1425**	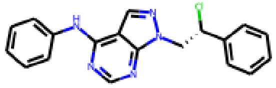	0.543329	Cc1ccc(C(=O)Nc2ccon2)cc1Nc1ncnc2c1cnn2C1cccccc1	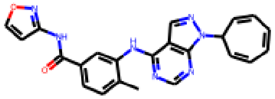	8.0	0.623362	2.525411	2.997881	5	0.770298
**1426**	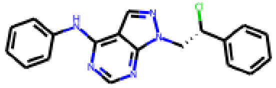	0.543329	Cc1ccc(C(=O)Nc2ccon2)cc1Nc1ncnc2c1cnn2C1cccccc1	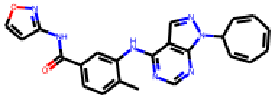	8.0	0.623362	2.525411	2.997881	5	0.770298
**1427**	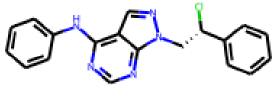	0.543210	Cc1ccc(C(=O)Nc2ccon2)cc1Nc1ncnc2c1cnn2C1cccccc1	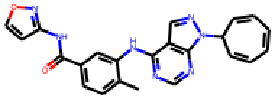	8.0	0.623362	2.525411	2.997881	5	0.770298

	src known mol	similarity	smiles	mol	Class	qed	logP	SAS	rings	kinase inhibition likeliness
**1428**	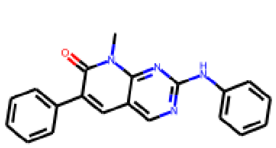	0.542990	Clc1cccc(Nc2ncc3cc(-c3c(Cl)cccc3Cl)c(=O)n(C)c3n2)c1	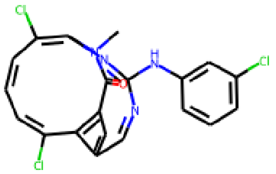	2.0	0.768132	3.608437	2.513170	5	0.784634
**1429**	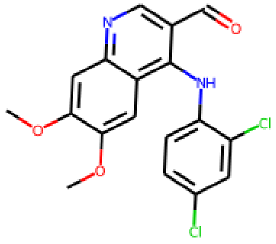	0.542815	COc1cc2ncc(C(=O)O)c(Nc3cccc(Cl)c3Cl)c2cc1OC1C(C)C1	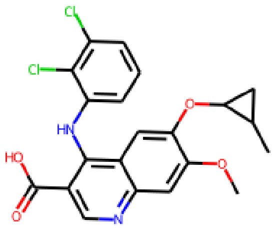	2.0	0.623362	2.525411	2.997881	4	0.774857
**1430**	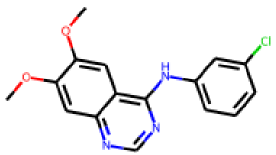	0.542739	Clc1cccc(Nc2ncnc3cc4c[nH]nc4cc23)c1	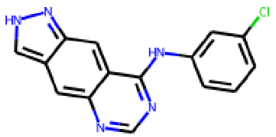	3.0	0.638163	3.995589	2.988471	4	0.781333
**1431**	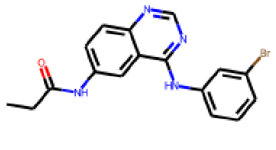	0.542688	O=C(CNC(=O)Oc1ccccc1)Nc1ccc2ncnc(Nc3cccc(Cl)c3)c2c1	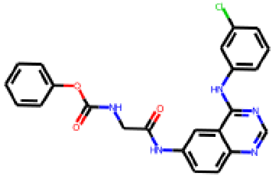	3.0	0.638163	3.995589	2.988471	4	0.793889

	src known mol	similarity	smiles	mol	Class	qed	logP	SAS	rings	kinase inhibition likeliness
**1432**	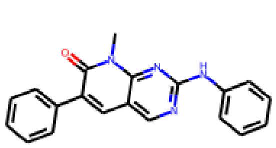	0.542636	Clc1cccc(Nc2ncc3cc(-c3c(Cl)cccc3Cl)c(=O)n(C)c3n2)c1	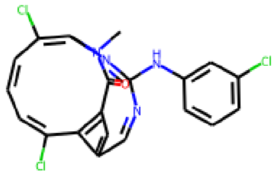	2.0	0.768132	3.608437	2.513170	5	0.784634
**1433**	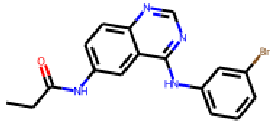	0.542572	O=C(CNC(=O)C1cccccc1)Nc1ccc2ncnc(Nc3cccc(Br)c3)c2c1	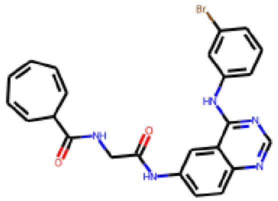	3.0	0.623362	2.525411	2.997881	4	0.772905
**1434**	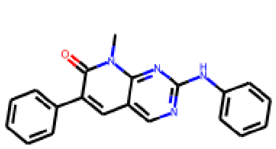	0.542412	Clc1cccc(Nc2ncc3cc(-c3c(Cl)cccc3Cl)c(=O)n(C)c3n2)c1	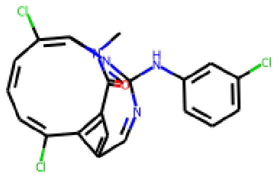	2.0	0.768132	3.608437	2.513170	5	0.784634
**1435**	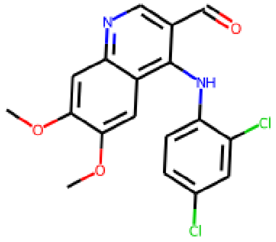	0.542385	COc1cc2ncc(C(=O)O)c(Nc3cccc(Cl)c3Cl)c2cc1OC1C(C)C1	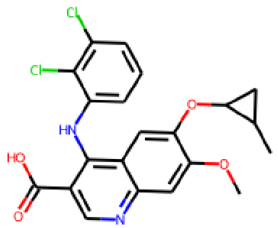	2.0	0.623362	2.525411	2.997881	4	0.774857

	src known mol	similarity	smiles	mol	Class	qed	logP	SAS	rings	kinase inhibition likeliness
**1436**	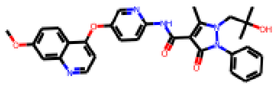	0.542265	COc1ccc2c(=O)cc(CNC(=O)c3c(C)n(C)n(-c4ccccc4)c3=O)nc3cccc2c3c1	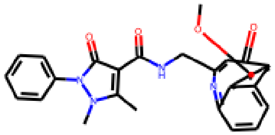	9.0	0.638163	3.995589	2.988471	5	0.774651
**1437**	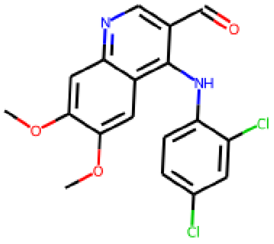	0.542264	COc1cc2ncc(C(=O)O)c(Nc3cccc(Cl)c3Cl)c2cc1OC1C(C)C1	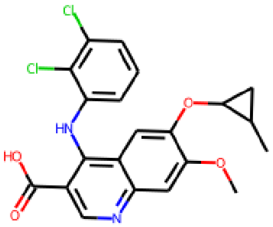	2.0	0.623362	2.525411	2.997881	4	0.774857
**1438**	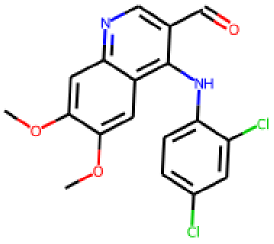	0.542264	COc1cc2ncc(C(=O)O)c(Nc3cccc(Cl)c3Cl)c2cc1OC1C(C)C1	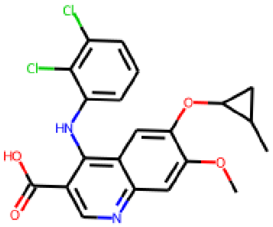	2.0	0.623362	2.525411	2.997881	4	0.774857
**1439**	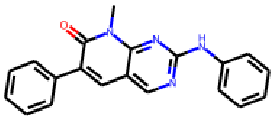	0.542101	Clc1cccc(Nc2ncc3cc(-c3c(Cl)cccc3Cl)c(=O)n(C)c3n2)c1	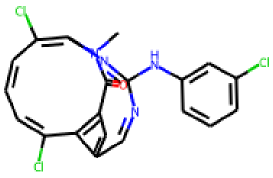	2.0	0.768132	3.608437	2.513170	5	0.784634

	src known mol	similarity	smiles	mol	Class	qed	logP	SAS	rings	kinase inhibition likeliness
**1440**	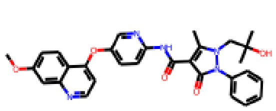	0.542066	COc1ccc2c(=O)cc(CNC(=O)c3c(C)n(C)n(-c4ccccc4)c3=O)nc3cccc2c3c1	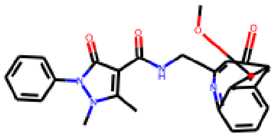	9.0	0.638163	3.995589	2.988471	5	0.774651
**1441**	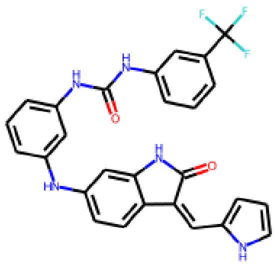	0.542042	COc1ccc(NC(=O)Nc2ccc3c(c2)NC(=O)/C3=C/c2ccc[nH]2)cc1	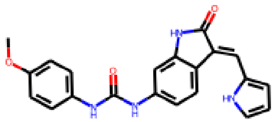	4.0	0.638163	3.995589	2.988471	4	0.768944
**1442**	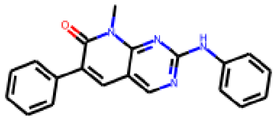	0.541985	Clc1cccc(Nc2ncc3cc(-c3c(Cl)cccc3Cl)c(=O)n(C)c3n2)c1	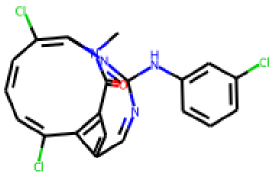	2.0	0.768132	3.608437	2.513170	5	0.784634
**1443**	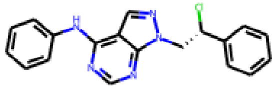	0.541973	Cc1ccc(C(=O)Nc2ccon2)cc1Nc1ncnc2c1cnn2C1cccccc1	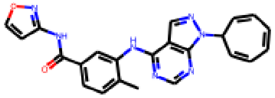	8.0	0.623362	2.525411	2.997881	5	0.770298

	src known mol	similarity	smiles	mol	Class	qed	logP	SAS	rings	kinase inhibition likeliness
**1444**	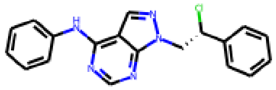	0.541973	Cc1ccc(C(=O)Nc2ccon2)cc1Nc1ncnc2c1cnn2C1cccccc1	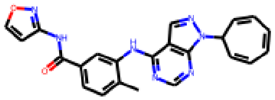	8.0	0.623362	2.525411	2.997881	5	0.770298
**1445**	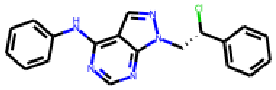	0.541973	Cc1ccc(C(=O)Nc2ccon2)cc1Nc1ncnc2c1cnn2C1cccccc1	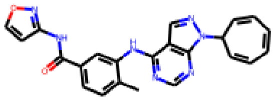	8.0	0.623362	2.525411	2.997881	5	0.770298
**1446**	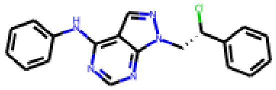	0.541973	Cc1ccc(C(=O)Nc2ccon2)cc1Nc1ncnc2c1cnn2C1cccccc1	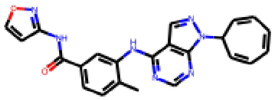	8.0	0.623362	2.525411	2.997881	5	0.770298
**1447**	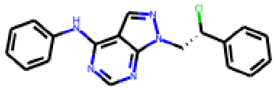	0.541973	Cc1ccc(C(=O)Nc2ccon2)cc1Nc1ncnc2c1cnn2C1cccccc1	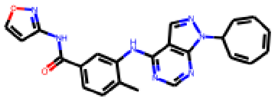	8.0	0.623362	2.525411	2.997881	5	0.770298

	src known mol	similarity	smiles	mol	Class	qed	logP	SAS	rings	kinase inhibition likeliness
**1448**	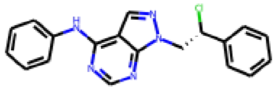	0.541973	Cc1ccc(C(=O)Nc2ccon2)cc1Nc1ncnc2c1cnn2C1cccccc1	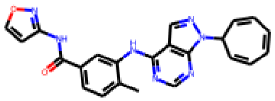	8.0	0.623362	2.525411	2.997881	5	0.770298
**1449**	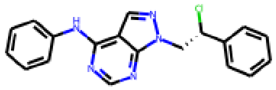	0.541945	Cc1ccc(C(=O)Nc2ccon2)cc1Nc1ncnc2c1cnn2C1cccccc1	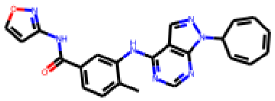	8.0	0.623362	2.525411	2.997881	5	0.770298
**1450**	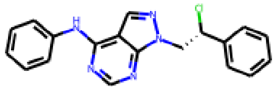	0.541945	Cc1ccc(C(=O)Nc2ccon2)cc1Nc1ncnc2c1cnn2C1cccccc1	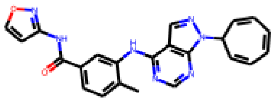	8.0	0.623362	2.525411	2.997881	5	0.770298
**1451**	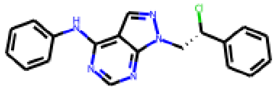	0.541945	Cc1ccc(C(=O)Nc2ccon2)cc1Nc1ncnc2c1cnn2C1cccccc1	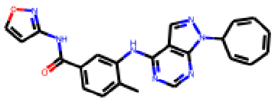	8.0	0.623362	2.525411	2.997881	5	0.770298

	src known mol	similarity	smiles	mol	Class	qed	logP	SAS	rings	kinase inhibition likeliness
**1452**	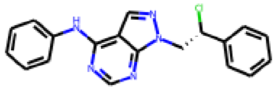	0.541945	Cc1ccc(C(=O)Nc2ccon2)cc1Nc1ncnc2c1cnn2C1cccccc1	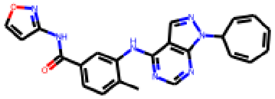	8.0	0.623362	2.525411	2.997881	5	0.770298
**1453**	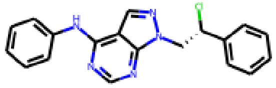	0.541940	Cc1ccc(C(=O)Nc2ccon2)cc1Nc1ncnc2c1cnn2C1cccccc1	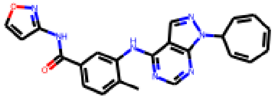	8.0	0.623362	2.525411	2.997881	5	0.770298
**1454**	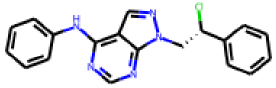	0.541940	Cc1ccc(C(=O)Nc2ccon2)cc1Nc1ncnc2c1cnn2C1cccccc1	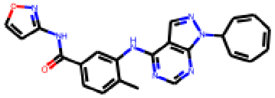	8.0	0.623362	2.525411	2.997881	5	0.770298
**1455**	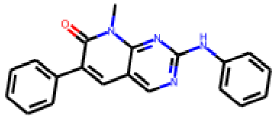	0.541827	Clc1cccc(Nc2ncc3cc(-c3c(Cl)cccc3Cl)c(=O)n(C)c3n2)c1	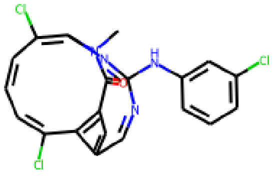	2.0	0.768132	3.608437	2.513170	5	0.784634

	src known mol	similarity	smiles	mol	Class	qed	logP	SAS	rings	kinase inhibition likeliness
**1456**	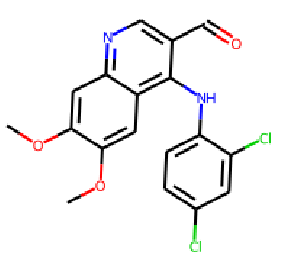	0.541785	COc1cc2ncc(C(=O)O)c(Nc3cccc(Cl)c3Cl)c2cc1OC1C(C)C1	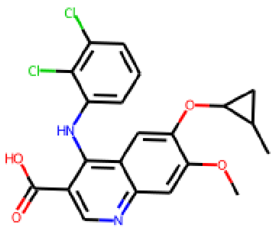	2.0	0.623362	2.525411	2.997881	4	0.774857
**1457**	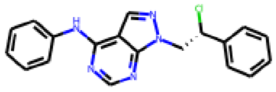	0.541753	Cc1ccc(C(=O)Nc2ccon2)cc1Nc1ncnc2c1cnn2C1cccccc1	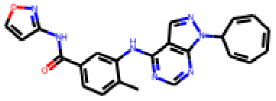	8.0	0.623362	2.525411	2.997881	5	0.770298
**1458**	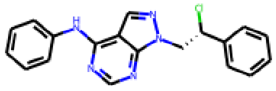	0.541753	Cc1ccc(C(=O)Nc2ccon2)cc1Nc1ncnc2c1cnn2C1cccccc1	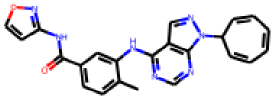	8.0	0.623362	2.525411	2.997881	5	0.770298
**1459**	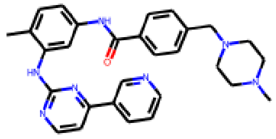	0.541632	Cc1ccc(NC(=O)c2ccccc2)cc1Nc1nccc(-c2cccnc2)n1	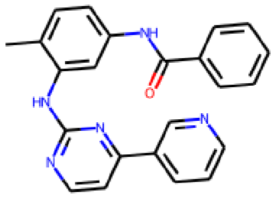	0.0	0.768132	3.608437	2.513170	4	0.854555

	src known mol	similarity	smiles	mol	Class	qed	logP	SAS	rings	kinase inhibition likeliness
**1460**	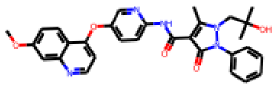	0.541622	COc1ccc2c(=O)cc(CNC(=O)c3c(C)n(C)n(-c4ccccc4)c3=O)nc3cccc2c3c1	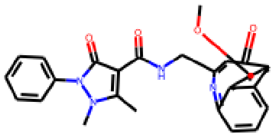	9.0	0.638163	3.995589	2.988471	5	0.774651
**1461**	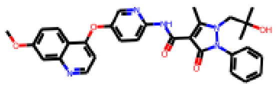	0.541622	COc1ccc2c(=O)cc(CNC(=O)c3c(C)n(C)n(-c4ccccc4)c3=O)nc3cccc2c3c1	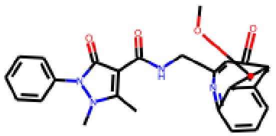	9.0	0.638163	3.995589	2.988471	5	0.774651
**1462**	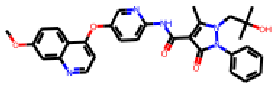	0.541466	COc1ccc2c(=O)cc(CNC(=O)c3c(C)n(C)n(-c4ccccc4)c3=O)nc3cccc2c3c1	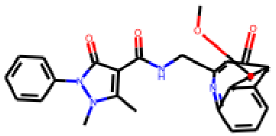	9.0	0.638163	3.995589	2.988471	5	0.774651
**1463**	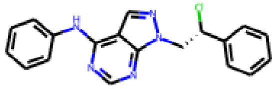	0.541456	Cc1ccc(C(=O)Nc2ccon2)cc1Nc1ncnc2c1cnn2C1cccccc1	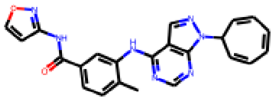	8.0	0.623362	2.525411	2.997881	5	0.770298

	src known mol	similarity	smiles	mol	Class	qed	logP	SAS	rings	kinase inhibition likeliness
**1464**	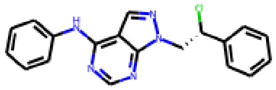	0.541456	Cc1ccc(C(=O)Nc2ccon2)cc1Nc1ncnc2c1cnn2C1cccccc1	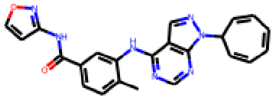	8.0	0.623362	2.525411	2.997881	5	0.770298
**1465**	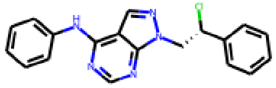	0.541456	Cc1ccc(C(=O)Nc2ccon2)cc1Nc1ncnc2c1cnn2C1cccccc1	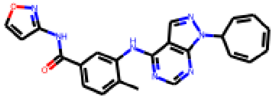	8.0	0.623362	2.525411	2.997881	5	0.770298
**1466**	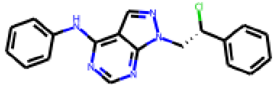	0.541456	Cc1ccc(C(=O)Nc2ccon2)cc1Nc1ncnc2c1cnn2C1cccccc1	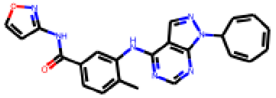	8.0	0.623362	2.525411	2.997881	5	0.770298
**1467**	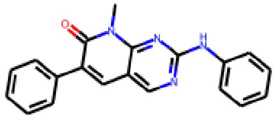	0.541445	Clc1cccc(Nc2ncc3cc(-c3c(Cl)cccc3Cl)c(=O)n(C)c3n2)c1	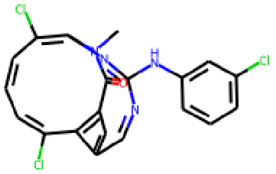	2.0	0.768132	3.608437	2.513170	5	0.784634

	src known mol	similarity	smiles	mol	Class	qed	logP	SAS	rings	kinase inhibition likeliness
**1468**	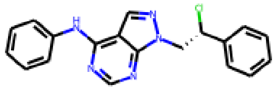	0.541429	Cc1ccc(C(=O)Nc2ccon2)cc1Nc1ncnc2c1cnn2C1cccccc1	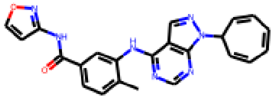	8.0	0.623362	2.525411	2.997881	5	0.770298
**1469**	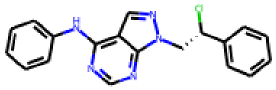	0.541429	Cc1ccc(C(=O)Nc2ccon2)cc1Nc1ncnc2c1cnn2C1cccccc1	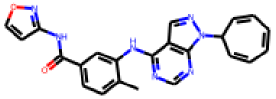	8.0	0.623362	2.525411	2.997881	5	0.770298
**1470**	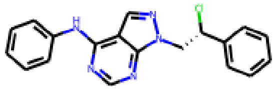	0.541337	Cc1ccc(C(=O)Nc2ccon2)cc1Nc1ncnc2c1cnn2C1cccccc1	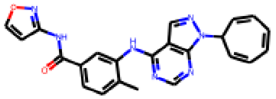	8.0	0.623362	2.525411	2.997881	5	0.770298
**1471**	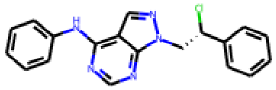	0.541337	Cc1ccc(C(=O)Nc2ccon2)cc1Nc1ncnc2c1cnn2C1cccccc1	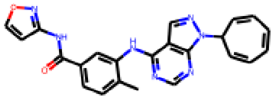	8.0	0.623362	2.525411	2.997881	5	0.770298

	src known mol	similarity	smiles	mol	Class	qed	logP	SAS	rings	kinase inhibition likeliness
**1472**	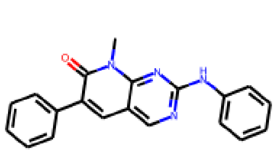	0.541313	Clc1cccc(Nc2ncc3cc(-c3c(Cl)cccc3Cl)c(=O)n(C)c3n2)c1	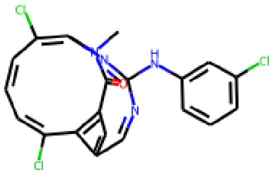	2.0	0.768132	3.608437	2.513170	5	0.784634
**1473**	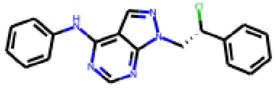	0.541297	Cc1ccc(C(=O)Nc2ccon2)cc1Nc1ncnc2c1cnn2C1cccccc1	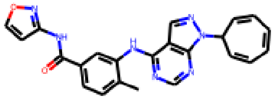	8.0	0.623362	2.525411	2.997881	5	0.770298
**1474**	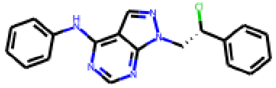	0.541297	Cc1ccc(C(=O)Nc2ccon2)cc1Nc1ncnc2c1cnn2C1cccccc1	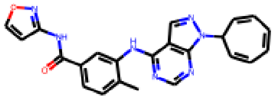	8.0	0.623362	2.525411	2.997881	5	0.770298
**1475**	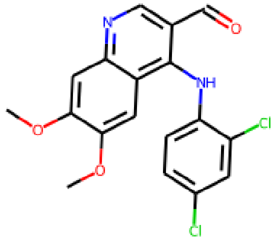	0.540995	COc1cc2ncc(C(=O)O)c(Nc3cccc(Cl)c3Cl)c2cc1OC1C(C)C1	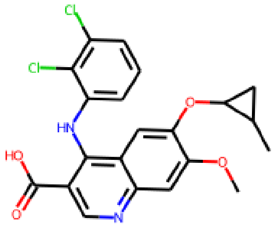	2.0	0.623362	2.525411	2.997881	4	0.774857

	src known mol	similarity	smiles	mol	Class	qed	logP	SAS	rings	kinase inhibition likeliness
**1476**	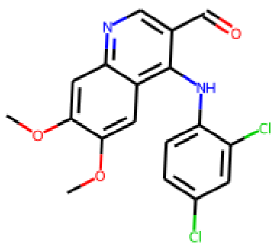	0.540995	COc1cc2ncc(C(=O)O)c(Nc3cccc(Cl)c3Cl)c2cc1OC1C(C)C1	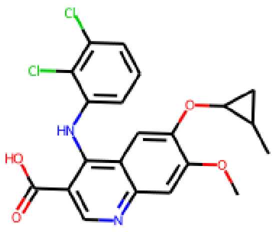	2.0	0.623362	2.525411	2.997881	4	0.774857
**1477**	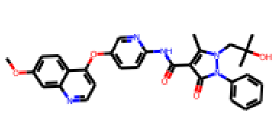	0.540914	COc1ccc2c(=O)cc(CNC(=O)c3c(C)n(C)n(-c4ccccc4)c3=O)nc3cccc2c3c1	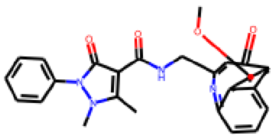	9.0	0.638163	3.995589	2.988471	5	0.774651
**1478**	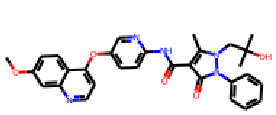	0.540914	COc1ccc2c(=O)cc(CNC(=O)c3c(C)n(C)n(-c4ccccc4)c3=O)nc3cccc2c3c1	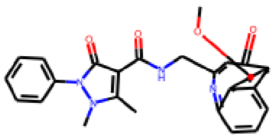	9.0	0.638163	3.995589	2.988471	5	0.774651
**1479**	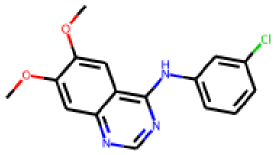	0.540900	Clc1cccc(Nc2ncnc3cc4c[nH]nc4cc23)c1	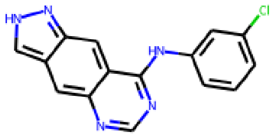	3.0	0.638163	3.995589	2.988471	4	0.781333

	src known mol	similarity	smiles	mol	Class	qed	logP	SAS	rings	kinase inhibition likeliness
**1480**	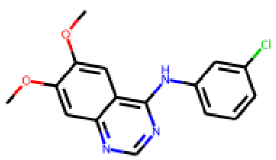	0.540816	Clc1cccc(Nc2ncnc3cc4c[nH]nc4cc23)c1	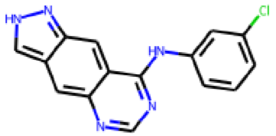	3.0	0.638163	3.995589	2.988471	4	0.781333
**1481**	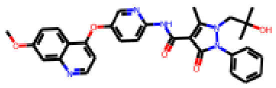	0.540752	COc1ccc2c(=O)cc(CNC(=O)c3c(C)n(C)n(-c4ccccc4)c3=O)nc3cccc2c3c1	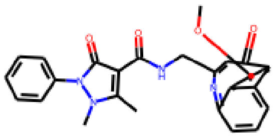	9.0	0.638163	3.995589	2.988471	5	0.774651
**1482**	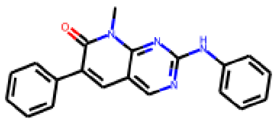	0.540644	Clc1cccc(Nc2ncc3cc(-c3c(Cl)cccc3Cl)c(=O)n(C)c3n2)c1	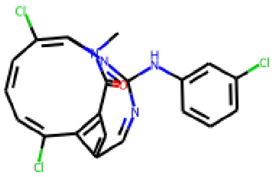	2.0	0.768132	3.608437	2.513170	5	0.784634
**1483**	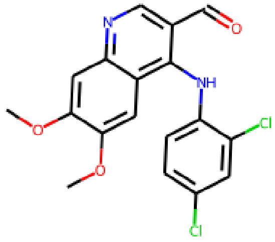	0.540623	COc1cc2ncc(C(=O)O)c(Nc3cccc(Cl)c3Cl)c2cc1OC1C(C)C1	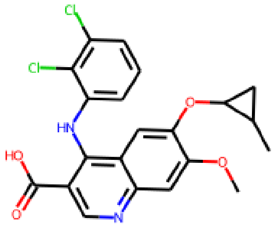	2.0	0.623362	2.525411	2.997881	4	0.774857

	src known mol	similarity	smiles	mol	Class	qed	logP	SAS	rings	kinase inhibition likeliness
**1484**	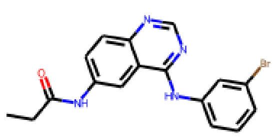	0.540614	O=C(CNC(=O)Oc1ccccc1)Nc1ccc2ncnc(Nc3cccc(Cl)c3)c2c1	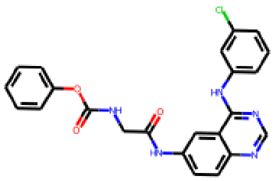	3.0	0.638163	3.995589	2.988471	4	0.793889
**1485**	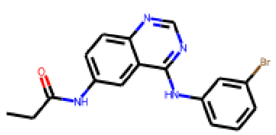	0.540614	O=C(CNC(=O)Oc1ccccc1)Nc1ccc2ncnc(Nc3cccc(Cl)c3)c2c1	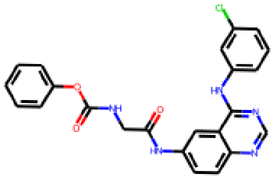	3.0	0.638163	3.995589	2.988471	4	0.793889
**1486**	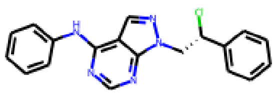	0.540611	Cc1ccc(C(=O)Nc2ccon2)cc1Nc1ncnc2c1cnn2C1cccccc1	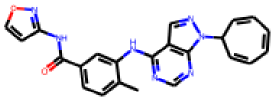	8.0	0.623362	2.525411	2.997881	5	0.770298
**1487**	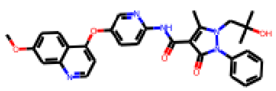	0.540597	COc1ccc2c(=O)cc(CNC(=O)c3c(C)n(C)n(-c4ccccc4)c3=O)nc3cccc2c3c1	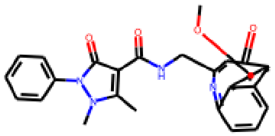	9.0	0.638163	3.995589	2.988471	5	0.774651

	src known mol	similarity	smiles	mol	Class	qed	logP	SAS	rings	kinase inhibition likeliness
**1488**	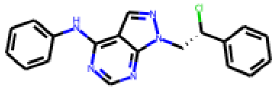	0.540386	Cc1ccc(C(=O)Nc2ccon2)cc1Nc1ncnc2c1cnn2C1cccccc1	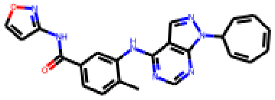	8.0	0.623362	2.525411	2.997881	5	0.770298
**1489**	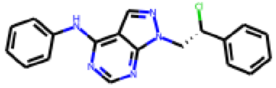	0.540386	Cc1ccc(C(=O)Nc2ccon2)cc1Nc1ncnc2c1cnn2C1cccccc1	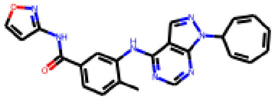	8.0	0.623362	2.525411	2.997881	5	0.770298
**1490**	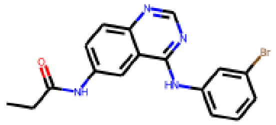	0.540301	O=C(CNC(=O)Oc1ccccc1)Nc1ccc2ncnc(Nc3cccc(Cl)c3)c2c1	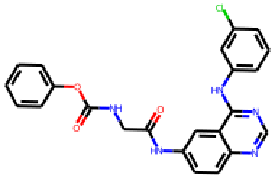	3.0	0.638163	3.995589	2.988471	4	0.793889
**1491**	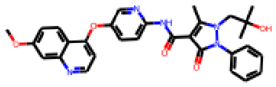	0.540267	COc1ccc2c(=O)cc(CNC(=O)c3c(C)n(C)n(-c4ccccc4)c3=O)nc3cccc2c3c1	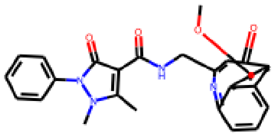	9.0	0.638163	3.995589	2.988471	5	0.774651

	src known mol	similarity	smiles	mol	Class	qed	logP	SAS	rings	kinase inhibition likeliness
**1492**	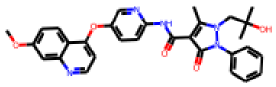	0.540267	COc1ccc2c(=O)cc(CNC(=O)c3c(C)n(C)n(-c4ccccc4)c3=O)nc3cccc2c3c1	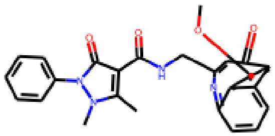	9.0	0.638163	3.995589	2.988471	5	0.774651
**1493**	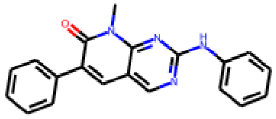	0.540230	Clc1cccc(Nc2ncc3cc(-c3c(Cl)cccc3Cl)c(=O)n(C)c3n2)c1	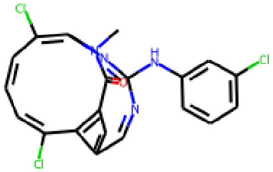	2.0	0.768132	3.608437	2.513170	5	0.784634
**1494**	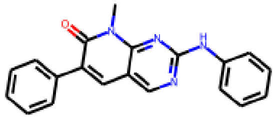	0.540230	Clc1cccc(Nc2ncc3cc(-c3c(Cl)cccc3Cl)c(=O)n(C)c3n2)c1	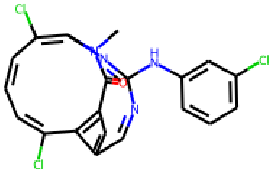	2.0	0.768132	3.608437	2.513170	5	0.784634
**1495**	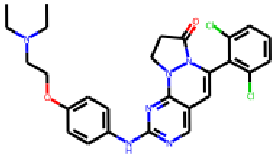	0.540013	COc1cc(Nc2ncc(C)c(-c3ccc4c(n3)NC(=O)C(C)(C)C4)n2)cc(OC)c1OC	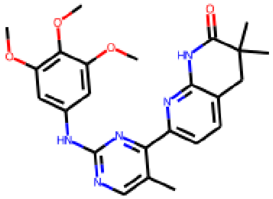	4.0	0.768132	3.608437	2.513170	4	0.757734

	src known mol	similarity	smiles	mol	Class	qed	logP	SAS	rings	kinase inhibition likeliness
**1496**	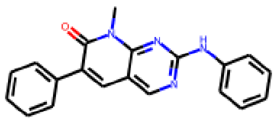	0.540000	Clc1cccc(Nc2ncc3cc(-c3c(Cl)cccc3Cl)c(=O)n(C)c3n2)c1	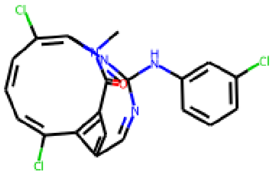	2.0	0.768132	3.608437	2.513170	5	0.784634
**1497**	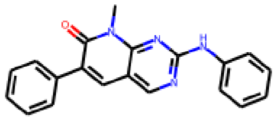	0.540000	Clc1cccc(Nc2ncc3cc(-c3c(Cl)cccc3Cl)c(=O)n(C)c3n2)c1	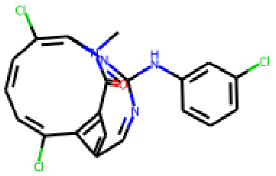	2.0	0.768132	3.608437	2.513170	5	0.784634
**1498**	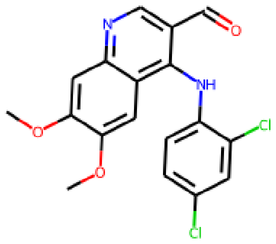	0.539896	COc1cc2ncc(C(=O)O)c(Nc3cccc(Cl)c3Cl)c2cc1OC1C(C)C1	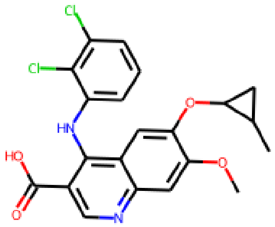	2.0	0.623362	2.525411	2.997881	4	0.774857
**1499**	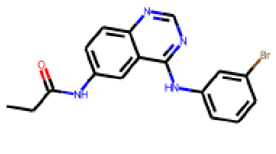	0.539846	O=C(CNC(=O)C1cccccc1)Nc1ccc2ncnc(Nc3cccc(Br)c3)c2c1	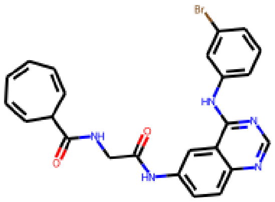	3.0	0.623362	2.525411	2.997881	4	0.772905

	src known mol	similarity	smiles	mol	Class	qed	logP	SAS	rings	kinase inhibition likeliness
**1500**	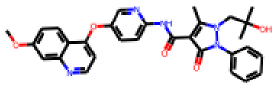	0.539832	COc1ccc2c(=O)cc(CNC(=O)c3c(C)n(C)n(-c4ccccc4)c3=O)nc3cccc2c3c1	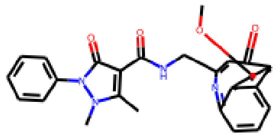	9.0	0.638163	3.995589	2.988471	5	0.774651
**1501**	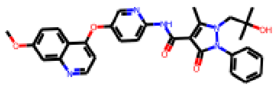	0.539832	COc1ccc2c(=O)cc(CNC(=O)c3c(C)n(C)n(-c4ccccc4)c3=O)nc3cccc2c3c1	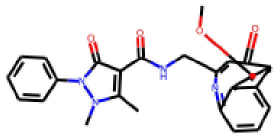	9.0	0.638163	3.995589	2.988471	5	0.774651
**1502**	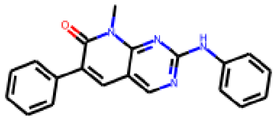	0.539830	Clc1cccc(Nc2ncc3cc(-c3c(Cl)cccc3Cl)c(=O)n(C)c3n2)c1	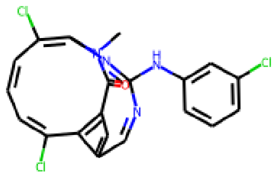	2.0	0.768132	3.608437	2.513170	5	0.784634
**1503**	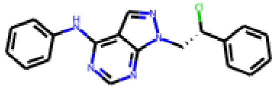	0.539726	Cc1ccc(C(=O)Nc2ccon2)cc1Nc1ncnc2c1cnn2C1cccccc1	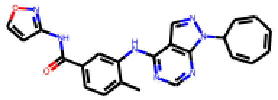	8.0	0.623362	2.525411	2.997881	5	0.770298

	src known mol	similarity	smiles	mol	Class	qed	logP	SAS	rings	kinase inhibition likeliness
**1504**	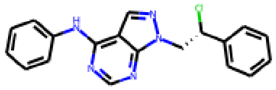	0.539726	Cc1ccc(C(=O)Nc2ccon2)cc1Nc1ncnc2c1cnn2C1cccccc1	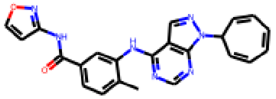	8.0	0.623362	2.525411	2.997881	5	0.770298
**1505**	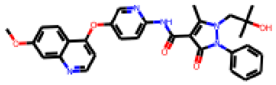	0.539725	COc1ccc2c(=O)cc(CNC(=O)c3c(C)n(C)n(-c4ccccc4)c3=O)nc3cccc2c3c1	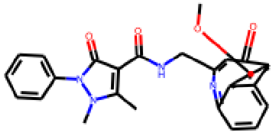	9.0	0.638163	3.995589	2.988471	5	0.774651
**1506**	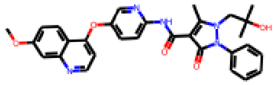	0.539725	COc1ccc2c(=O)cc(CNC(=O)c3c(C)n(C)n(-c4ccccc4)c3=O)nc3cccc2c3c1	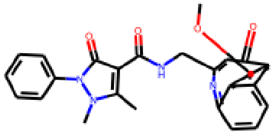	9.0	0.638163	3.995589	2.988471	5	0.774651
**1507**	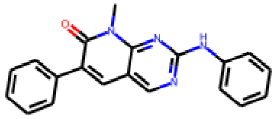	0.539634	Clc1cccc(Nc2ncc3cc(-c3c(Cl)cccc3Cl)c(=O)n(C)c3n2)c1	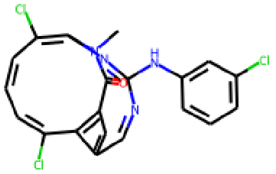	2.0	0.768132	3.608437	2.513170	5	0.784634

	src known mol	similarity	smiles	mol	Class	qed	logP	SAS	rings	kinase inhibition likeliness
**1508**	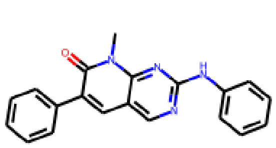	0.539634	Clc1cccc(Nc2ncc3cc(-c3c(Cl)cccc3Cl)c(=O)n(C)c3n2)c1	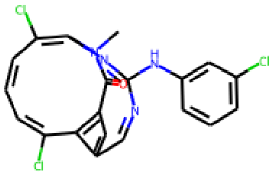	2.0	0.768132	3.608437	2.513170	5	0.784634
**1509**	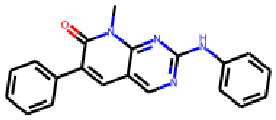	0.539574	Clc1cccc(Nc2ncc3cc(-c3c(Cl)cccc3Cl)c(=O)n(C)c3n2)c1	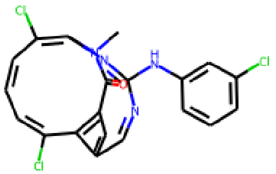	2.0	0.768132	3.608437	2.513170	5	0.784634
**1510**	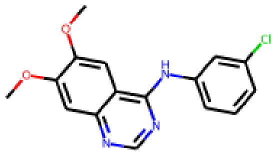	0.539568	Clc1cccc(Nc2ncnc3cc4c[nH]nc4cc23)c1	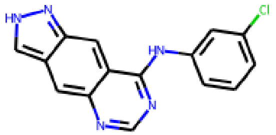	3.0	0.638163	3.995589	2.988471	4	0.781333
**1511**	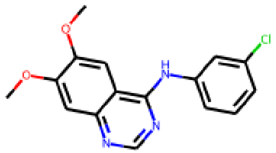	0.539568	Clc1cccc(Nc2ncnc3cc4c[nH]nc4cc23)c1	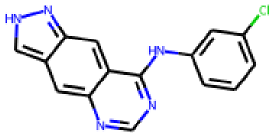	3.0	0.638163	3.995589	2.988471	4	0.781333

	src known mol	similarity	smiles	mol	Class	qed	logP	SAS	rings	kinase inhibition likeliness
**1512**	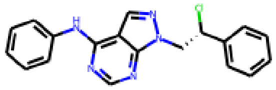	0.539402	Cc1ccc(C(=O)Nc2ccon2)cc1Nc1ncnc2c1cnn2C1cccccc1	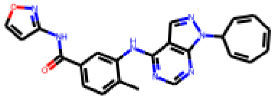	8.0	0.623362	2.525411	2.997881	5	0.770298
**1513**	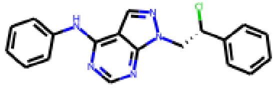	0.539402	Cc1ccc(C(=O)Nc2ccon2)cc1Nc1ncnc2c1cnn2C1cccccc1	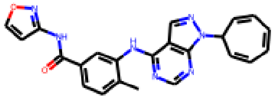	8.0	0.623362	2.525411	2.997881	5	0.770298
**1514**	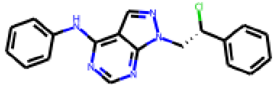	0.539402	Cc1ccc(C(=O)Nc2ccon2)cc1Nc1ncnc2c1cnn2C1cccccc1	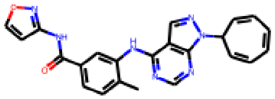	8.0	0.623362	2.525411	2.997881	5	0.770298
**1515**	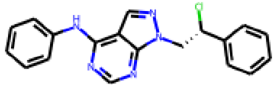	0.539402	Cc1ccc(C(=O)Nc2ccon2)cc1Nc1ncnc2c1cnn2C1cccccc1	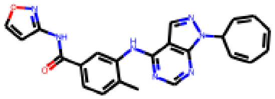	8.0	0.623362	2.525411	2.997881	5	0.770298

	src known mol	similarity	smiles	mol	Class	qed	logP	SAS	rings	kinase inhibition likeliness
**1516**	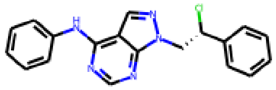	0.539402	Cc1ccc(C(=O)Nc2ccon2)cc1Nc1ncnc2c1cnn2C1cccccc1	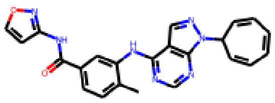	8.0	0.623362	2.525411	2.997881	5	0.770298
**1517**	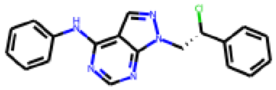	0.539402	Cc1ccc(C(=O)Nc2ccon2)cc1Nc1ncnc2c1cnn2C1cccccc1	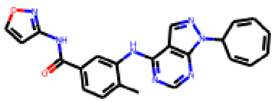	8.0	0.623362	2.525411	2.997881	5	0.770298
**1518**	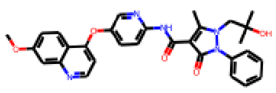	0.539190	COc1ccc2c(=O)cc(CNC(=O)c3c(C)n(C)n(-c4ccccc4)c3=O)nc3cccc2c3c1	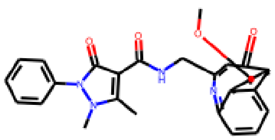	9.0	0.638163	3.995589	2.988471	5	0.774651
**1519**	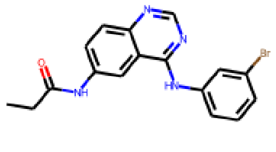	0.539130	O=C(CNC(=O)C1cccccc1)Nc1ccc2ncnc(Nc3cccc(Br)c3)c2c1	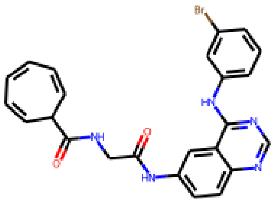	3.0	0.623362	2.525411	2.997881	4	0.772905

	src known mol	similarity	smiles	mol	Class	qed	logP	SAS	rings	kinase inhibition likeliness
**1520**	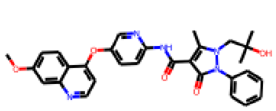	0.539067	COc1ccc2c(=O)cc(CNC(=O)c3c(C)n(C)n(-c4ccccc4)c3=O)nc3cccc2c3c1	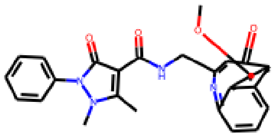	9.0	0.638163	3.995589	2.988471	5	0.774651
**1521**	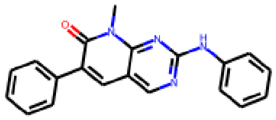	0.538986	Clc1cccc(Nc2ncc3cc(-c3c(Cl)cccc3Cl)c(=O)n(C)c3n2)c1	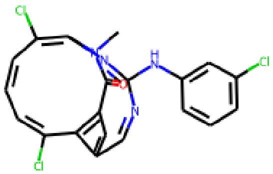	2.0	0.768132	3.608437	2.513170	5	0.784634
**1522**	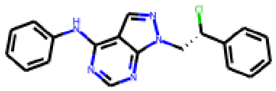	0.538978	Cc1ccc(C(=O)Nc2ccon2)cc1Nc1ncnc2c1cnn2C1cccccc1	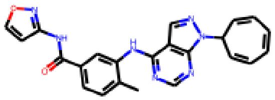	8.0	0.623362	2.525411	2.997881	5	0.770298
**1523**	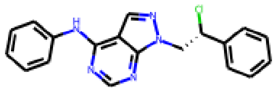	0.538978	Cc1ccc(C(=O)Nc2ccon2)cc1Nc1ncnc2c1cnn2C1cccccc1	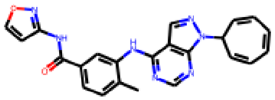	8.0	0.623362	2.525411	2.997881	5	0.770298

	src known mol	similarity	smiles	mol	Class	qed	logP	SAS	rings	kinase inhibition likeliness
**1524**	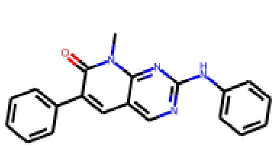	0.538816	Clc1cccc(Nc2ncc3cc(-c3c(Cl)cccc3Cl)c(=O)n(C)c3n2)c1	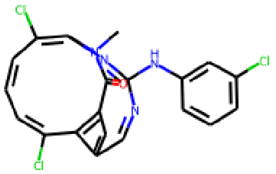	2.0	0.768132	3.608437	2.513170	5	0.784634
**1525**	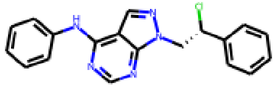	0.538728	Cc1ccc(C(=O)Nc2ccon2)cc1Nc1ncnc2c1cnn2C1cccccc1	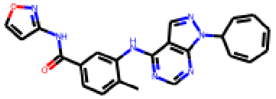	8.0	0.623362	2.525411	2.997881	5	0.770298
**1526**	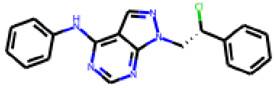	0.538728	Cc1ccc(C(=O)Nc2ccon2)cc1Nc1ncnc2c1cnn2C1cccccc1	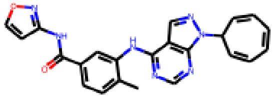	8.0	0.623362	2.525411	2.997881	5	0.770298
**1527**	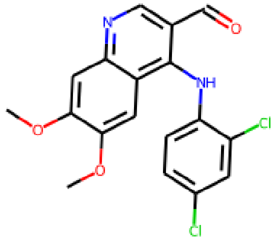	0.538678	COc1cc2ncc(C(=O)O)c(Nc3cccc(Cl)c3Cl)c2cc1OC1C(C)C1	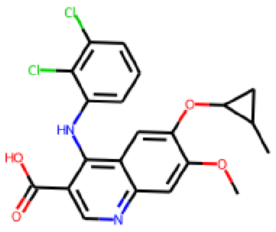	2.0	0.623362	2.525411	2.997881	4	0.774857

	src known mol	similarity	smiles	mol	Class	qed	logP	SAS	rings	kinase inhibition likeliness
**1528**	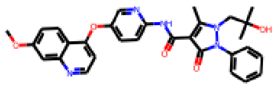	0.538676	COc1ccc2c(=O)cc(CNC(=O)c3c(C)n(C)n(-c4ccccc4)c3=O)nc3cccc3c2n1	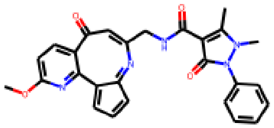	9.0	0.768132	3.608437	2.513170	5	0.823048
**1529**	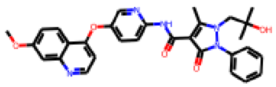	0.538676	COc1ccc2c(=O)cc(CNC(=O)c3c(C)n(C)n(-c4ccccc4)c3=O)nc3cccc3c2n1	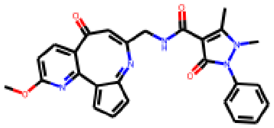	9.0	0.768132	3.608437	2.513170	5	0.823048
**1530**	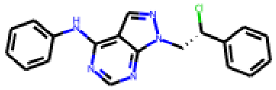	0.538570	Cc1ccc(C(=O)Nc2ccon2)cc1Nc1ncnc2c1cnn2C1cccccc1	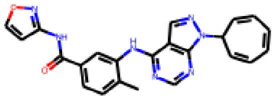	8.0	0.623362	2.525411	2.997881	5	0.770298
**1531**	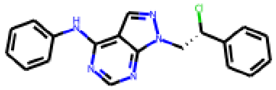	0.538570	Cc1ccc(C(=O)Nc2ccon2)cc1Nc1ncnc2c1cnn2C1cccccc1	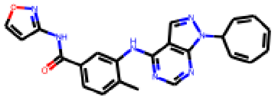	8.0	0.623362	2.525411	2.997881	5	0.770298

	src known mol	similarity	smiles	mol	Class	qed	logP	SAS	rings	kinase inhibition likeliness
**1532**	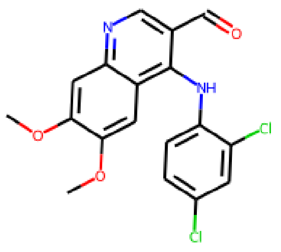	0.538176	COc1cc2ncc(C(=O)O)c(Nc3cccc(Cl)c3Cl)c2cc1OC1C(C)C1	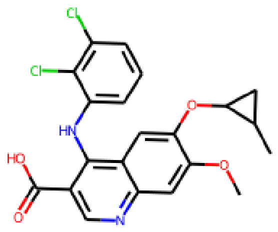	2.0	0.623362	2.525411	2.997881	4	0.774857
**1533**	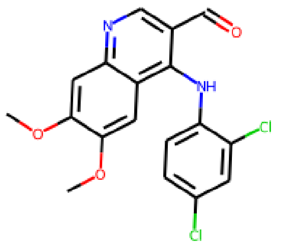	0.538176	COc1cc2ncc(C(=O)O)c(Nc3cccc(Cl)c3Cl)c2cc1OC1C(C)C1	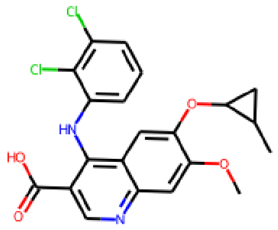	2.0	0.623362	2.525411	2.997881	4	0.774857
**1534**	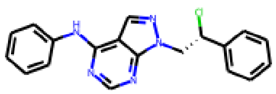	0.538141	Cc1ccc(C(=O)Nc2ccon2)cc1Nc1ncnc2c1cnn2C1cccccc1	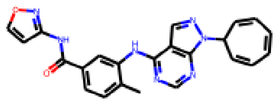	8.0	0.623362	2.525411	2.997881	5	0.770298
**1535**	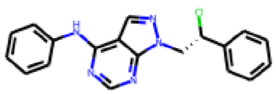	0.538141	Cc1ccc(C(=O)Nc2ccon2)cc1Nc1ncnc2c1cnn2C1cccccc1	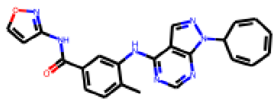	8.0	0.623362	2.525411	2.997881	5	0.770298

	src known mol	similarity	smiles	mol	Class	qed	logP	SAS	rings	kinase inhibition likeliness
**1536**	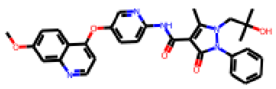	0.538136	COc1ccc2c(=O)cc(CNC(=O)c3c(C)n(C)n(-<br>c4ccccc4)c3=O)nc3cccc2c3c1	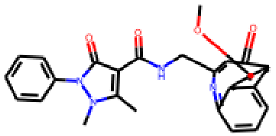	9.0	0.638163	3.995589	2.988471	5	0.774651
**1537**	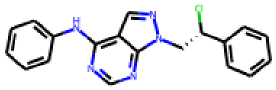	0.538133	Cc1ccc(C(=O)Nc2ccon2)cc1Nc1ncnc2c1cnn2C1cccccc1	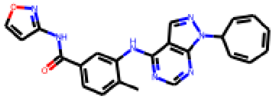	8.0	0.623362	2.525411	2.997881	5	0.770298
**1538**	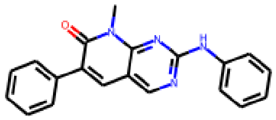	0.538106	Clc1cccc(Nc2ncc3cc(-c3c(Cl)cccc3Cl)c(=O)n(C)c3n2)c1	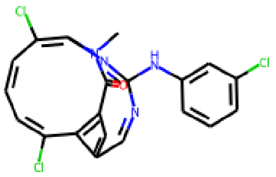	2.0	0.768132	3.608437	2.513170	5	0.784634
**1539**	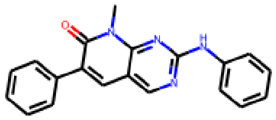	0.538106	Clc1cccc(Nc2ncc3cc(-c3c(Cl)cccc3Cl)c(=O)n(C)c3n2)c1	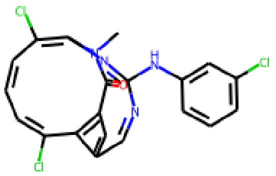	2.0	0.768132	3.608437	2.513170	5	0.784634

	src known mol	similarity	smiles	mol	Class	qed	logP	SAS	rings	kinase inhibition likeliness
**1540**	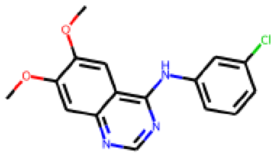	0.537994	Clc1cccc(Nc2ncnc3cc4c[nH]nc4cc23)c1	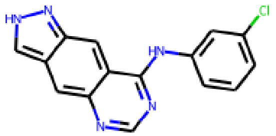	3.0	0.638163	3.995589	2.988471	4	0.781333
**1541**	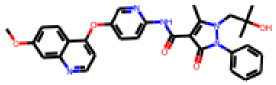	0.537842	COc1ccc2c(=O)cc(CNC(=O)c3c(C)n(C)n(-c4ccccc4)c3=O)nc3cccc2c3c1	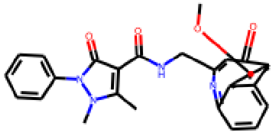	9.0	0.638163	3.995589	2.988471	5	0.774651
**1542**	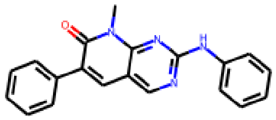	0.537602	Clc1cccc(Nc2ncc3cc(-c3c(Cl)cccc3Cl)c(=O)n(C)c3n2)c1	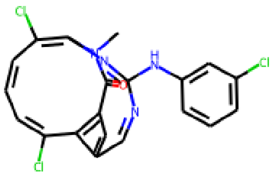	2.0	0.768132	3.608437	2.513170	5	0.784634
**1543**	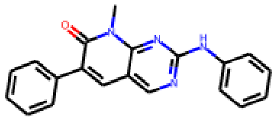	0.537577	Clc1cccc(Nc2ncc3cc(-c3c(Cl)cccc3Cl)c(=O)n(C)c3n2)c1	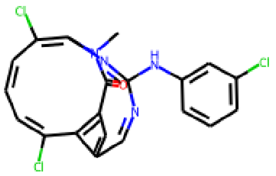	2.0	0.768132	3.608437	2.513170	5	0.784634

	src known mol	similarity	smiles	mol	Class	qed	logP	SAS	rings	kinase inhibition likeliness
**1544**	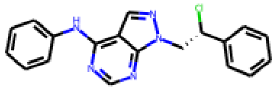	0.537410	Cc1ccc(C(=O)Nc2ccon2)cc1Nc1ncnc2c1cnn2C1cccccc1	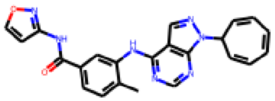	8.0	0.623362	2.525411	2.997881	5	0.770298
**1545**	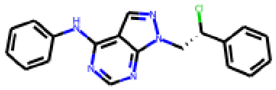	0.537410	Cc1ccc(C(=O)Nc2ccon2)cc1Nc1ncnc2c1cnn2C1cccccc1	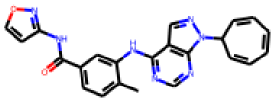	8.0	0.623362	2.525411	2.997881	5	0.770298
**1546**	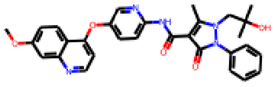	0.537347	COc1ccc2c(=O)cc(CNC(=O)c3c(C)n(C)n(-c4ccccc4)c3=O)nc3cccc3c2n1	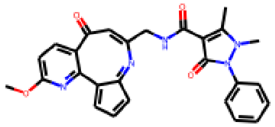	9.0	0.768132	3.608437	2.513170	5	0.823048
**1547**	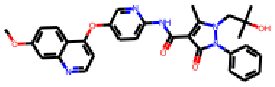	0.537347	COc1ccc2c(=O)cc(CNC(=O)c3c(C)n(C)n(-c4ccccc4)c3=O)nc3cccc3c2n1	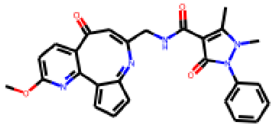	9.0	0.768132	3.608437	2.513170	5	0.823048

	src known mol	similarity	smiles	mol	Class	qed	logP	SAS	rings	kinase inhibition likeliness
**1548**	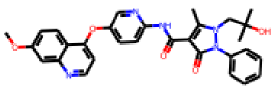	0.537347	COc1ccc2c(=O)cc(CNC(=O)c3c(C)n(C)n(-c4ccccc4)c3=O)nc3cccc3c2n1	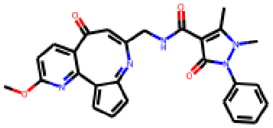	9.0	0.768132	3.608437	2.513170	5	0.823048
**1549**	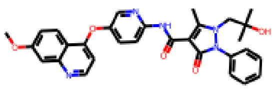	0.537347	COc1ccc2c(=O)cc(CNC(=O)c3c(C)n(C)n(-c4ccccc4)c3=O)nc3cccc3c2n1	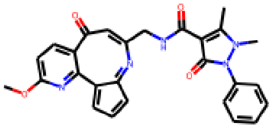	9.0	0.768132	3.608437	2.513170	5	0.823048
**1550**	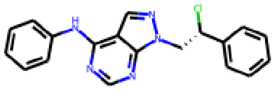	0.537260	Cc1ccc(C(=O)Nc2ccon2)cc1Nc1ncnc2c1cnn2C1cccccc1	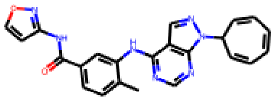	8.0	0.623362	2.525411	2.997881	5	0.770298
**1551**	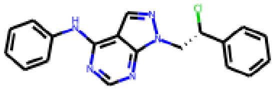	0.537260	Cc1ccc(C(=O)Nc2ccon2)cc1Nc1ncnc2c1cnn2C1cccccc1	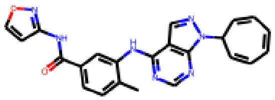	8.0	0.623362	2.525411	2.997881	5	0.770298

	src known mol	similarity	smiles	mol	Class	qed	logP	SAS	rings	kinase inhibition likeliness
**1552**	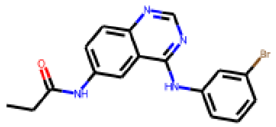	0.537238	O=C(CNC(=O)Oc1ccccc1)Nc1ccc2ncnc(Nc3cccc(Cl)c3)c2c1	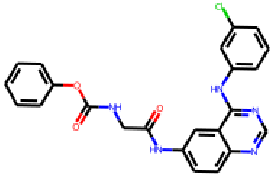	3.0	0.638163	3.995589	2.988471	4	0.793889
**1553**	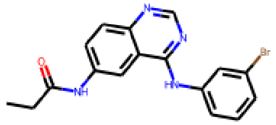	0.537238	O=C(CNC(=O)Oc1ccccc1)Nc1ccc2ncnc(Nc3cccc(Cl)c3)c2c1	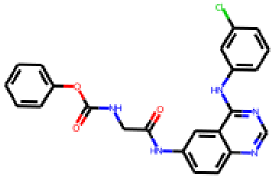	3.0	0.638163	3.995589	2.988471	4	0.793889
**1554**	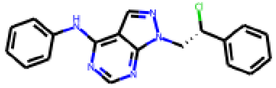	0.537219	Cc1ccc(C(=O)Nc2ccon2)cc1Nc1ncnc2c1cnn2C1cccccc1	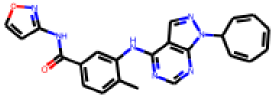	8.0	0.623362	2.525411	2.997881	5	0.770298
**1555**	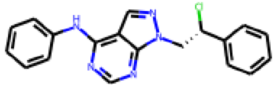	0.537219	Cc1ccc(C(=O)Nc2ccon2)cc1Nc1ncnc2c1cnn2C1cccccc1	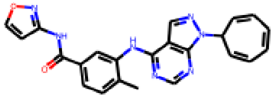	8.0	0.623362	2.525411	2.997881	5	0.770298

	src known mol	similarity	smiles	mol	Class	qed	logP	SAS	rings	kinase inhibition likeliness
**1556**	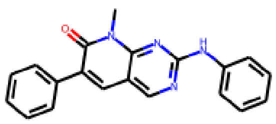	0.537108	Clc1cccc(Nc2ncc3cc(-c3c(Cl)cccc3Cl)c(=O)n(C)c3n2)c1	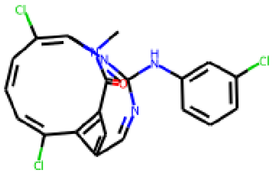	2.0	0.768132	3.608437	2.513170	5	0.784634
**1557**	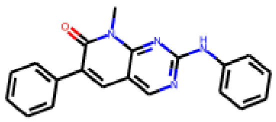	0.536995	Clc1cccc(Nc2ncc3cc(-c3c(Cl)cccc3Cl)c(=O)n(C)c3n2)c1	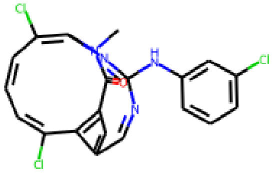	2.0	0.768132	3.608437	2.513170	5	0.784634
**1558**	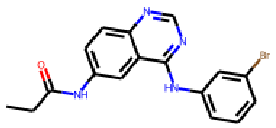	0.536851	O=C(CNC(=O)Oc1ccccc1)Nc1ccc2ncnc(Nc3cccc(Cl)c3)c2c1	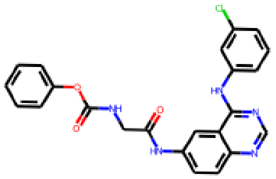	3.0	0.638163	3.995589	2.988471	4	0.793889
**1559**	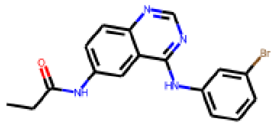	0.536851	O=C(CNC(=O)Oc1ccccc1)Nc1ccc2ncnc(Nc3cccc(Cl)c3)c2c1	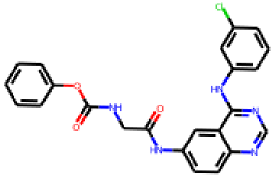	3.0	0.638163	3.995589	2.988471	4	0.793889

	src known mol	similarity	smiles	mol	Class	qed	logP	SAS	rings	kinase inhibition likeliness
**1560**	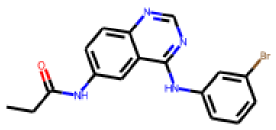	0.536851	O=C(CNC(=O)Oc1ccccc1)Nc1ccc2ncnc(Nc3cccc(Cl)c3)c2c1	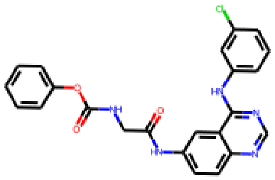	3.0	0.638163	3.995589	2.988471	4	0.793889
**1561**	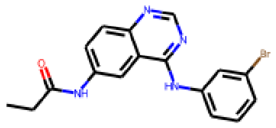	0.536851	O=C(CNC(=O)Oc1ccccc1)Nc1ccc2ncnc(Nc3cccc(Cl)c3)c2c1	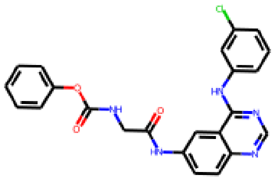	3.0	0.638163	3.995589	2.988471	4	0.793889
**1562**	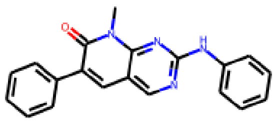	0.536513	Clc1cccc(Nc2ncc3cc(-c3c(Cl)cccc3Cl)c(=O)n(C)c3n2)c1	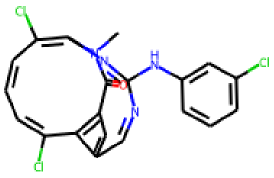	2.0	0.768132	3.608437	2.513170	5	0.784634
**1563**	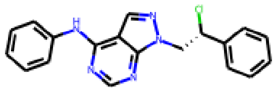	0.536469	Cc1ccc(C(=O)Nc2ccon2)cc1Nc1ncnc2c1cnn2C1cccccc1	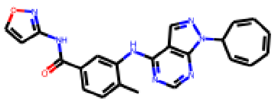	8.0	0.623362	2.525411	2.997881	5	0.770298

	src known mol	similarity	smiles	mol	Class	qed	logP	SAS	rings	kinase inhibition likeliness
**1564**	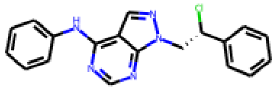	0.536469	Cc1ccc(C(=O)Nc2ccon2)cc1Nc1ncnc2c1cnn2C1cccccc1	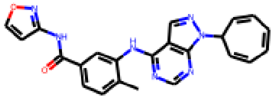	8.0	0.623362	2.525411	2.997881	5	0.770298
**1565**	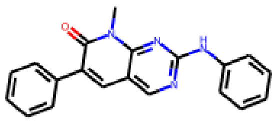	0.536429	Clc1cccc(Nc2ncc3cc(-c3c(Cl)cccc3Cl)c(=O)n(C)c3n2)c1	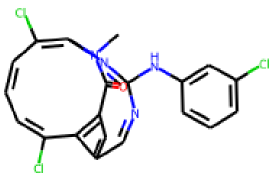	2.0	0.768132	3.608437	2.513170	5	0.784634
**1566**	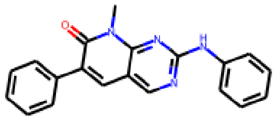	0.536398	Clc1cccc(Nc2ncc3cc(-c3c(Cl)cccc3Cl)c(=O)n(C)c3n2)c1	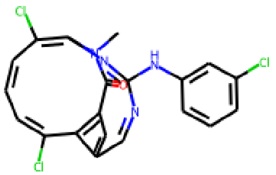	2.0	0.768132	3.608437	2.513170	5	0.784634
**1567**	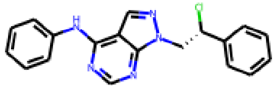	0.536396	Cc1ccc(C(=O)Nc2ccon2)cc1Nc1ncnc2c1cnn2C1cccccc1	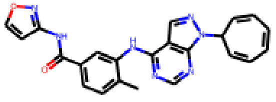	8.0	0.623362	2.525411	2.997881	5	0.770298

	src known mol	similarity	smiles	mol	Class	qed	logP	SAS	rings	kinase inhibition likeliness
**1568**	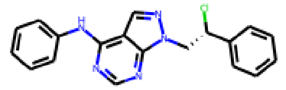	0.536396	Cc1ccc(C(=O)Nc2ccon2)cc1Nc1ncnc2c1cnn2C1cccccc1	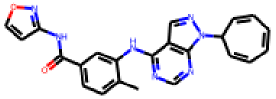	8.0	0.623362	2.525411	2.997881	5	0.770298
**1569**	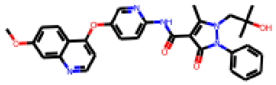	0.536310	COc1ccc2c(=O)cc(CNC(=O)c3c(C)n(C)n(-c4ccccc4)c3=O)nc3cccc2c3c1	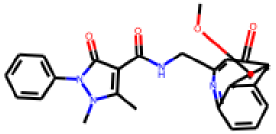	9.0	0.638163	3.995589	2.988471	5	0.774651
**1570**	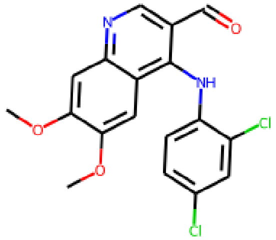	0.536262	COc1cc2ncc(C(=O)O)c(Nc3cccc(Cl)c3Cl)c2cc1OC1C(C)C1	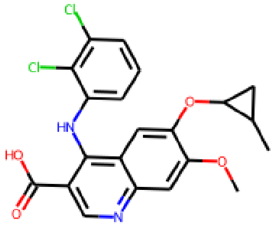	2.0	0.623362	2.525411	2.997881	4	0.774857
**1571**	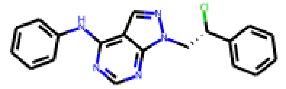	0.536252	Cc1ccc(C(=O)Nc2ccon2)cc1Nc1ncnc2c1cnn2C1cccccc1	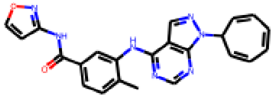	8.0	0.623362	2.525411	2.997881	5	0.770298

	src known mol	similarity	smiles	mol	Class	qed	logP	SAS	rings	kinase inhibition likeliness
**1572**	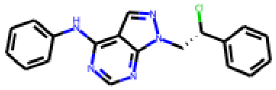	0.536252	Cc1ccc(C(=O)Nc2ccon2)cc1Nc1ncnc2c1cnn2C1cccccc1	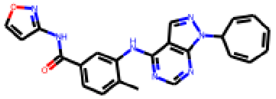	8.0	0.623362	2.525411	2.997881	5	0.770298
**1573**	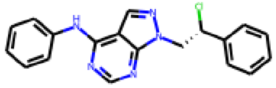	0.536252	Cc1ccc(C(=O)Nc2ccon2)cc1Nc1ncnc2c1cnn2C1cccccc1	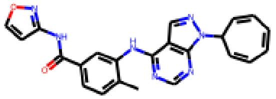	8.0	0.623362	2.525411	2.997881	5	0.770298
**1574**	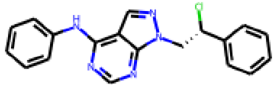	0.536252	Cc1ccc(C(=O)Nc2ccon2)cc1Nc1ncnc2c1cnn2C1cccccc1	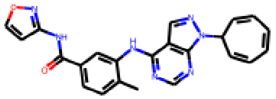	8.0	0.623362	2.525411	2.997881	5	0.770298
**1575**	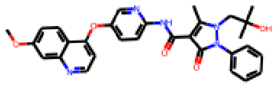	0.536224	COc1ccc2c(=O)cc(CNC(=O)c3c(C)n(C)n(-c4ccccc4)c3=O)nc3cccc2c3c1	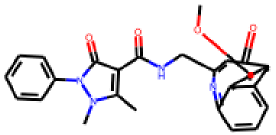	9.0	0.638163	3.995589	2.988471	5	0.774651

	src known mol	similarity	smiles	mol	Class	qed	logP	SAS	rings	kinase inhibition likeliness
**1576**	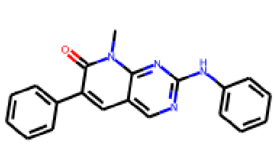	0.536145	Clc1cccc(Nc2ncc3cc(-c3c(Cl)cccc3Cl)c(=O)n(C)c3n2)c1	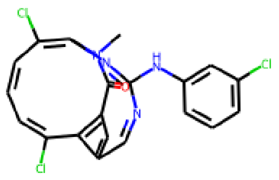	2.0	0.768132	3.608437	2.513170	5	0.784634
**1577**	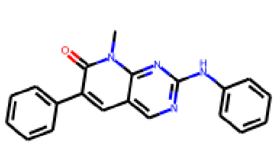	0.536145	Clc1cccc(Nc2ncc3cc(-c3c(Cl)cccc3Cl)c(=O)n(C)c3n2)c1	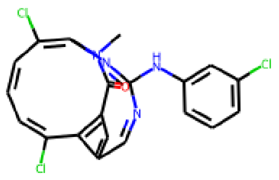	2.0	0.768132	3.608437	2.513170	5	0.784634
**1578**	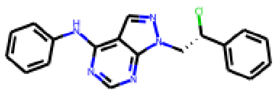	0.536132	Cc1ccc(C(=O)Nc2ccon2)cc1Nc1ncnc2c1cnn2C1cccccc1	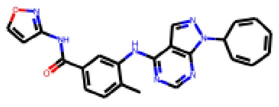	8.0	0.623362	2.525411	2.997881	5	0.770298
**1579**	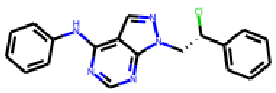	0.536132	Cc1ccc(C(=O)Nc2ccon2)cc1Nc1ncnc2c1cnn2C1cccccc1	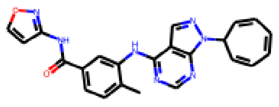	8.0	0.623362	2.525411	2.997881	5	0.770298

	src known mol	similarity	smiles	mol	Class	qed	logP	SAS	rings	kinase inhibition likeliness
**1580**	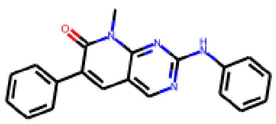	0.535878	Clc1cccc(Nc2ncc3cc(-c3c(Cl)cccc3Cl)c(=O)n(C)c3n2)c1	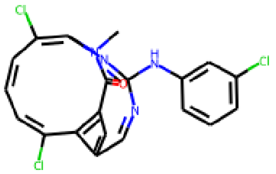	2.0	0.768132	3.608437	2.513170	5	0.784634
**1581**	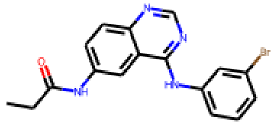	0.535627	O=C(CNC(=O)Oc1ccccc1)Nc1ccc2ncnc(Nc3cccc(Cl)c3)c2c1	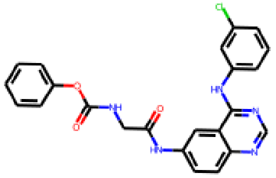	3.0	0.638163	3.995589	2.988471	4	0.793889
**1582**	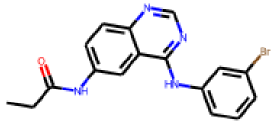	0.535627	O=C(CNC(=O)Oc1ccccc1)Nc1ccc2ncnc(Nc3cccc(Cl)c3)c2c1	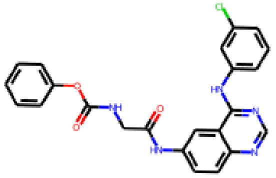	3.0	0.638163	3.995589	2.988471	4	0.793889
**1583**	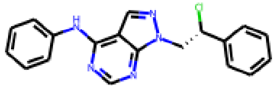	0.535616	Cc1ccc(C(=O)Nc2ccon2)cc1Nc1ncnc2c1cnn2C1cccccc1	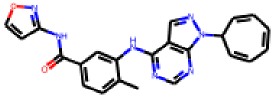	8.0	0.623362	2.525411	2.997881	5	0.770298

	src known mol	similarity	smiles	mol	Class	qed	logP	SAS	rings	kinase inhibition likeliness
**1584**	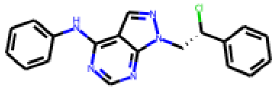	0.535616	Cc1ccc(C(=O)Nc2ccon2)cc1Nc1ncnc2c1cnn2C1cccccc1	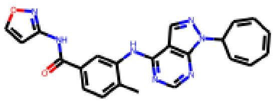	8.0	0.623362	2.525411	2.997881	5	0.770298
**1585**	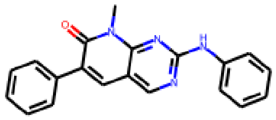	0.535606	Clc1cccc(Nc2ncc3cc(-c3c(Cl)cccc3Cl)c(=O)n(C)c3n2)c1	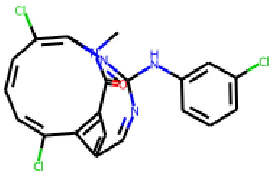	2.0	0.768132	3.608437	2.513170	5	0.784634
**1586**	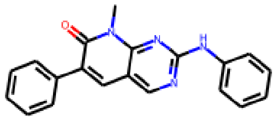	0.535606	Clc1cccc(Nc2ncc3cc(-c3c(Cl)cccc3Cl)c(=O)n(C)c3n2)c1	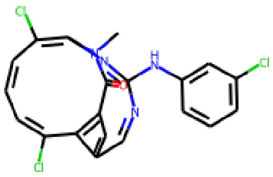	2.0	0.768132	3.608437	2.513170	5	0.784634
**1587**	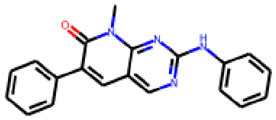	0.535606	Clc1cccc(Nc2ncc3cc(-c3c(Cl)cccc3Cl)c(=O)n(C)c3n2)c1	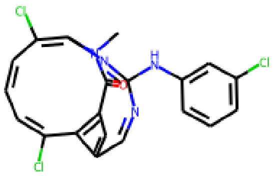	2.0	0.768132	3.608437	2.513170	5	0.784634

	src known mol	similarity	smiles	mol	Class	qed	logP	SAS	rings	kinase inhibition likeliness
**1588**	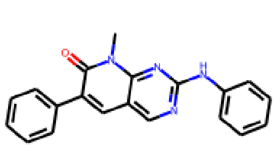	0.535572	Clc1cccc(Nc2ncc3cc(-c3c(Cl)cccc3Cl)c(=O)n(C)c3n2)c1	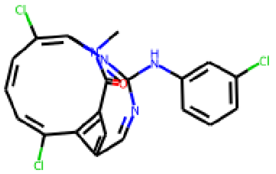	2.0	0.768132	3.608437	2.513170	5	0.784634
**1589**	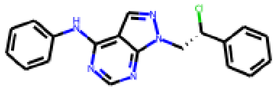	0.535535	Cc1ccc(C(=O)Nc2ccon2)cc1Nc1ncnc2c1cnn2C1cccccc1	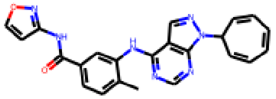	8.0	0.623362	2.525411	2.997881	5	0.770298
**1590**	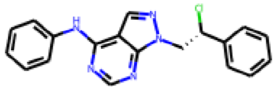	0.535535	Cc1ccc(C(=O)Nc2ccon2)cc1Nc1ncnc2c1cnn2C1cccccc1	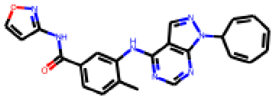	8.0	0.623362	2.525411	2.997881	5	0.770298
**1591**	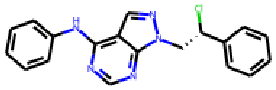	0.535535	Cc1ccc(C(=O)Nc2ccon2)cc1Nc1ncnc2c1cnn2C1cccccc1	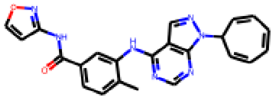	8.0	0.623362	2.525411	2.997881	5	0.770298

	src known mol	similarity	smiles	mol	Class	qed	logP	SAS	rings	kinase inhibition likeliness
**1592**	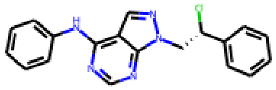	0.535535	Cc1ccc(C(=O)Nc2ccon2)cc1Nc1ncnc2c1cnn2C1cccccc1	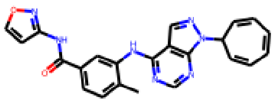	8.0	0.623362	2.525411	2.997881	5	0.770298
**1593**	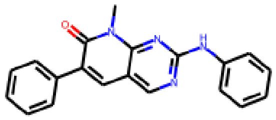	0.535469	Clc1cccc(Nc2ncc3cc(-c3c(Cl)cccc3Cl)c(=O)n(C)c3n2)c1	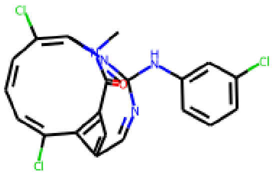	2.0	0.768132	3.608437	2.513170	5	0.784634
**1594**	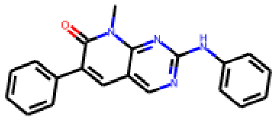	0.535469	Clc1cccc(Nc2ncc3cc(-c3c(Cl)cccc3Cl)c(=O)n(C)c3n2)c1	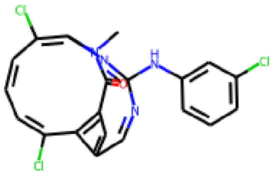	2.0	0.768132	3.608437	2.513170	5	0.784634
**1595**	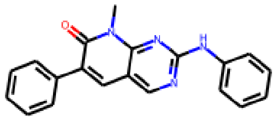	0.535469	Clc1cccc(Nc2ncc3cc(-c3c(Cl)cccc3Cl)c(=O)n(C)c3n2)c1	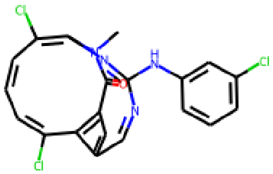	2.0	0.768132	3.608437	2.513170	5	0.784634

	src known mol	similarity	smiles	mol	Class	qed	logP	SAS	rings	kinase inhibition likeliness
**1596**	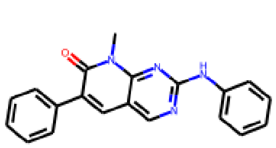	0.535469	Clc1cccc(Nc2ncc3cc(-c3c(Cl)cccc3Cl)c(=O)n(C)c3n2)c1	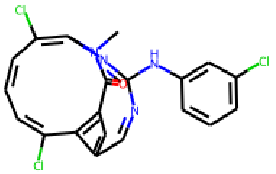	2.0	0.768132	3.608437	2.513170	5	0.784634
**1597**	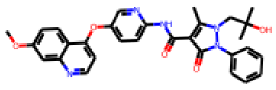	0.535455	COc1ccc2c(=O)cc(CNC(=O)c3c(C)n(C)n(-c4ccccc4)c3=O)nc3cccc3c2n1	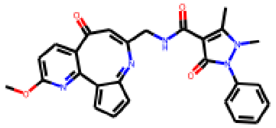	9.0	0.768132	3.608437	2.513170	5	0.823048
**1598**	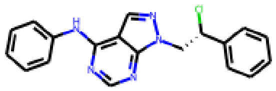	0.535306	Cc1ccc(C(=O)Nc2ccon2)cc1Nc1ncnc2c1cnn2C1cccccc1	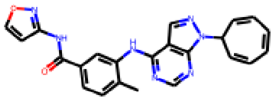	8.0	0.623362	2.525411	2.997881	5	0.770298
**1599**	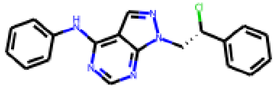	0.535306	Cc1ccc(C(=O)Nc2ccon2)cc1Nc1ncnc2c1cnn2C1cccccc1	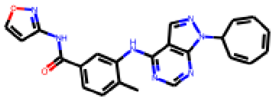	8.0	0.623362	2.525411	2.997881	5	0.770298

	src known mol	similarity	smiles	mol	Class	qed	logP	SAS	rings	kinase inhibition likeliness
**1600**	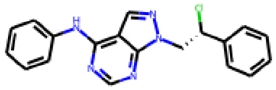	0.535306	Cc1ccc(C(=O)Nc2ccon2)cc1Nc1ncnc2c1cnn2C1cccccc1	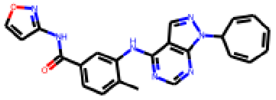	8.0	0.623362	2.525411	2.997881	5	0.770298
**1601**	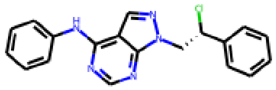	0.535306	Cc1ccc(C(=O)Nc2ccon2)cc1Nc1ncnc2c1cnn2C1cccccc1	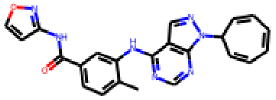	8.0	0.623362	2.525411	2.997881	5	0.770298
**1602**	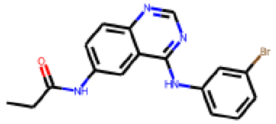	0.535284	O=C(CNC(=O)C1cccccc1)Nc1ccc2ncnc(Nc3cccc(Br)c3)c2c1	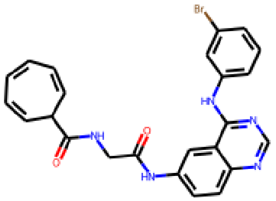	3.0	0.623362	2.525411	2.997881	4	0.772905
**1603**	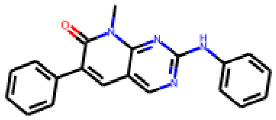	0.535232	Clc1cccc(Nc2ncc3cc(-c3c(Cl)cccc3Cl)c(=O)n(C)c3n2)c1	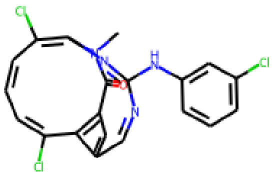	2.0	0.768132	3.608437	2.513170	5	0.784634

	src known mol	similarity	smiles	mol	Class	qed	logP	SAS	rings	kinase inhibition likeliness
**1604**	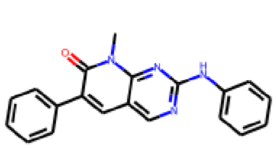	0.535211	Clc1cccc(Nc2ncc3cc(-c3c(Cl)cccc3Cl)c(=O)n(C)c3n2)c1	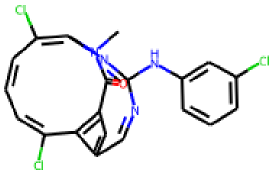	2.0	0.768132	3.608437	2.513170	5	0.784634
**1605**	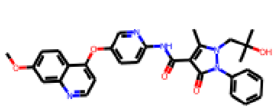	0.535181	COc1ccc2c(=O)cc(CNC(=O)c3c(C)n(C)n(-c4ccccc4)c3=O)nc3cccc2c3c1	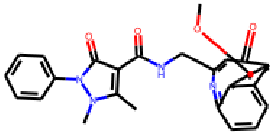	9.0	0.638163	3.995589	2.988471	5	0.774651
**1606**	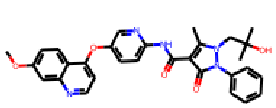	0.535181	COc1ccc2c(=O)cc(CNC(=O)c3c(C)n(C)n(-c4ccccc4)c3=O)nc3cccc2c3c1	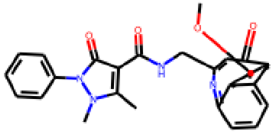	9.0	0.638163	3.995589	2.988471	5	0.774651
**1607**	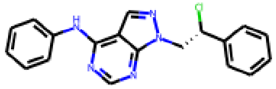	0.535088	Cc1ccc(C(=O)Nc2ccon2)cc1Nc1ncnc2c1cnn2C1cccccc1	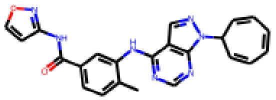	8.0	0.623362	2.525411	2.997881	5	0.770298

	src known mol	similarity	smiles	mol	Class	qed	logP	SAS	rings	kinase inhibition likeliness
**1608**	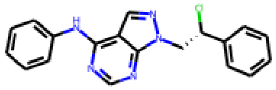	0.535088	Cc1ccc(C(=O)Nc2ccon2)cc1Nc1ncnc2c1cnn2C1cccccc1	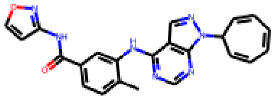	8.0	0.623362	2.525411	2.997881	5	0.770298
**1609**	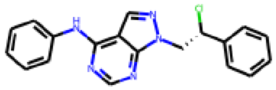	0.535088	Cc1ccc(C(=O)Nc2ccon2)cc1Nc1ncnc2c1cnn2C1cccccc1	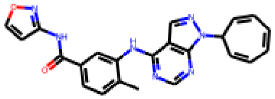	8.0	0.623362	2.525411	2.997881	5	0.770298
**1610**	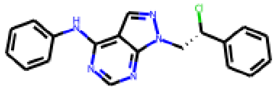	0.535088	Cc1ccc(C(=O)Nc2ccon2)cc1Nc1ncnc2c1cnn2C1cccccc1	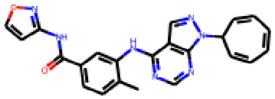	8.0	0.623362	2.525411	2.997881	5	0.770298
**1611**	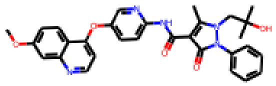	0.535000	COc1ccc2c(=O)cc(CNC(=O)c3c(C)n(C)n(-c4ccccc4)c3=O)nc3cccc3c2n1	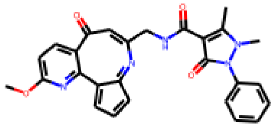	9.0	0.768132	3.608437	2.513170	5	0.823048

	src known mol	similarity	smiles	mol	Class	qed	logP	SAS	rings	kinase inhibition likeliness
**1612**	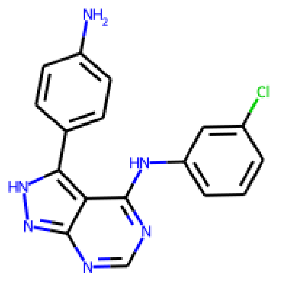	0.534970	c1ccc(Nc2ncnc3n[nH]c(-c4ccccc4)c23)cc1	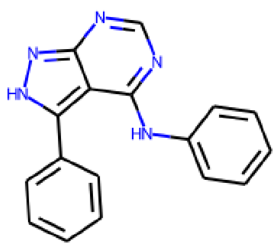	0.0	0.638163	3.995589	2.988471	4	0.800634
**1613**	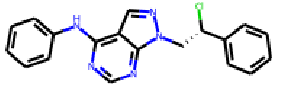	0.534694	Cc1ccc(C(=O)Nc2ccon2)cc1Nc1ncnc2c1cnn2C1cccccc1	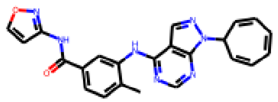	8.0	0.623362	2.525411	2.997881	5	0.770298
**1614**	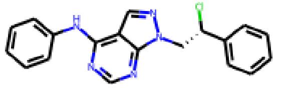	0.534694	Cc1ccc(C(=O)Nc2ccon2)cc1Nc1ncnc2c1cnn2C1cccccc1	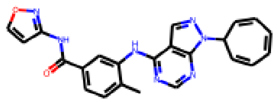	8.0	0.623362	2.525411	2.997881	5	0.770298
**1615**	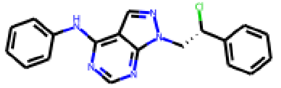	0.534694	Cc1ccc(C(=O)Nc2ccon2)cc1Nc1ncnc2c1cnn2C1cccccc1	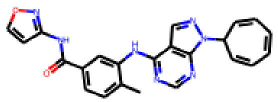	8.0	0.623362	2.525411	2.997881	5	0.770298

	src known mol	similarity	smiles	mol	Class	qed	logP	SAS	rings	kinase inhibition likeliness
**1616**	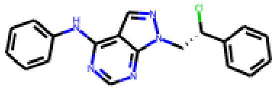	0.534694	Cc1ccc(C(=O)Nc2ccon2)cc1Nc1ncnc2c1cnn2C1cccccc1	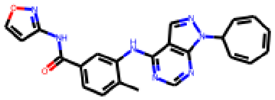	8.0	0.623362	2.525411	2.997881	5	0.770298
**1617**	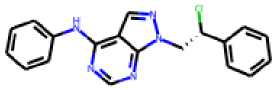	0.534587	Cc1ccc(C(=O)Nc2ccon2)cc1Nc1ncnc2c1cnn2C1cccccc1	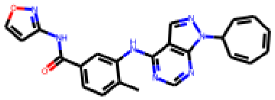	8.0	0.623362	2.525411	2.997881	5	0.770298
**1618**	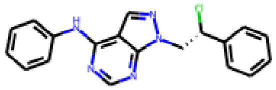	0.534587	Cc1ccc(C(=O)Nc2ccon2)cc1Nc1ncnc2c1cnn2C1cccccc1	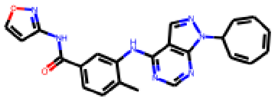	8.0	0.623362	2.525411	2.997881	5	0.770298
**1619**	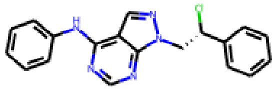	0.534578	Cc1ccc(C(=O)Nc2ccon2)cc1Nc1ncnc2c1cnn2C1cccccc1	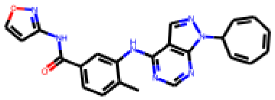	8.0	0.623362	2.525411	2.997881	5	0.770298

	src known mol	similarity	smiles	mol	Class	qed	logP	SAS	rings	kinase inhibition likeliness
**1620**	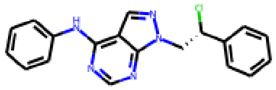	0.534578	Cc1ccc(C(=O)Nc2ccon2)cc1Nc1ncnc2c1cnn2C1cccccc1	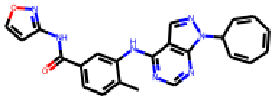	8.0	0.623362	2.525411	2.997881	5	0.770298
**1621**	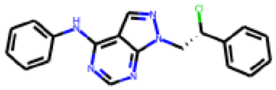	0.534553	Cc1ccc(C(=O)Nc2ccon2)cc1Nc1ncnc2c1cnn2C1cccccc1	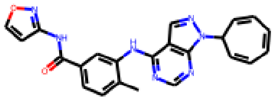	8.0	0.623362	2.525411	2.997881	5	0.770298
**1622**	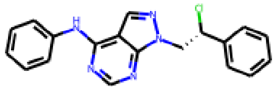	0.534553	Cc1ccc(C(=O)Nc2ccon2)cc1Nc1ncnc2c1cnn2C1cccccc1	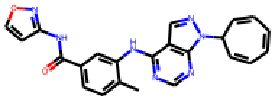	8.0	0.623362	2.525411	2.997881	5	0.770298
**1623**	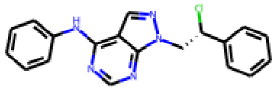	0.534553	Cc1ccc(C(=O)Nc2ccon2)cc1Nc1ncnc2c1cnn2C1cccccc1	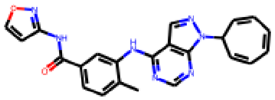	8.0	0.623362	2.525411	2.997881	5	0.770298

	src known mol	similarity	smiles	mol	Class	qed	logP	SAS	rings	kinase inhibition likeliness
**1624**	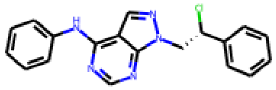	0.534553	Cc1ccc(C(=O)Nc2ccon2)cc1Nc1ncnc2c1cnn2C1cccccc1	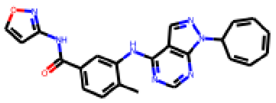	8.0	0.623362	2.525411	2.997881	5	0.770298
**1625**	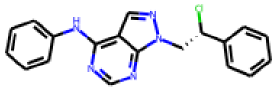	0.534553	Cc1ccc(C(=O)Nc2ccon2)cc1Nc1ncnc2c1cnn2C1cccccc1	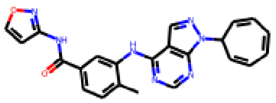	8.0	0.623362	2.525411	2.997881	5	0.770298
**1626**	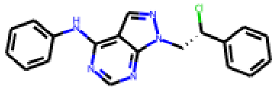	0.534553	Cc1ccc(C(=O)Nc2ccon2)cc1Nc1ncnc2c1cnn2C1cccccc1	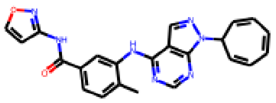	8.0	0.623362	2.525411	2.997881	5	0.770298
**1627**	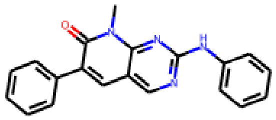	0.534340	Clc1cccc(Nc2ncc3cc(-c3c(Cl)cccc3Cl)c(=O)n(C)c3n2)c1	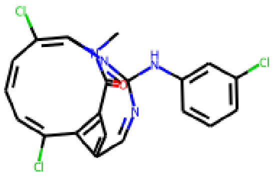	2.0	0.768132	3.608437	2.513170	5	0.784634

	src known mol	similarity	smiles	mol	Class	qed	logP	SAS	rings	kinase inhibition likeliness
**1628**	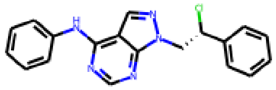	0.534218	Cc1ccc(C(=O)Nc2ccon2)cc1Nc1ncnc2c1cnn2C1cccccc1	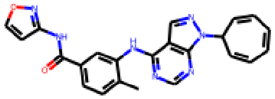	8.0	0.623362	2.525411	2.997881	5	0.770298
**1629**	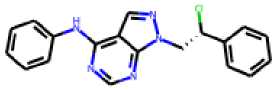	0.534218	Cc1ccc(C(=O)Nc2ccon2)cc1Nc1ncnc2c1cnn2C1cccccc1	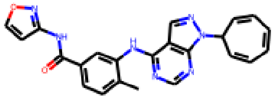	8.0	0.623362	2.525411	2.997881	5	0.770298
**1630**	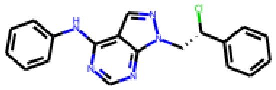	0.534218	Cc1ccc(C(=O)Nc2ccon2)cc1Nc1ncnc2c1cnn2C1cccccc1	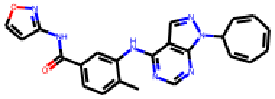	8.0	0.623362	2.525411	2.997881	5	0.770298
**1631**	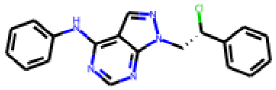	0.534218	Cc1ccc(C(=O)Nc2ccon2)cc1Nc1ncnc2c1cnn2C1cccccc1	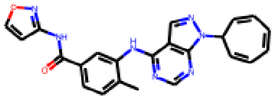	8.0	0.623362	2.525411	2.997881	5	0.770298

	src known mol	similarity	smiles	mol	Class	qed	logP	SAS	rings	kinase inhibition likeliness
**1632**	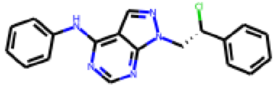	0.534218	Cc1ccc(C(=O)Nc2ccon2)cc1Nc1ncnc2c1cnn2C1cccccc1	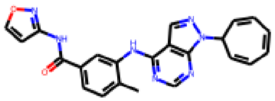	8.0	0.623362	2.525411	2.997881	5	0.770298
**1633**	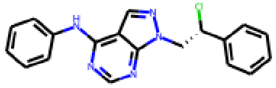	0.534218	Cc1ccc(C(=O)Nc2ccon2)cc1Nc1ncnc2c1cnn2C1cccccc1	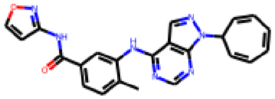	8.0	0.623362	2.525411	2.997881	5	0.770298
**1634**	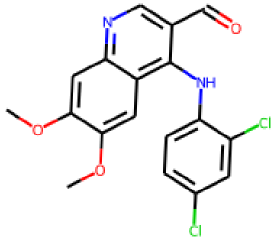	0.534213	COc1cc2ncc(C(=O)O)c(Nc3cccc(Cl)c3Cl)c2cc1OC1C(C)C1	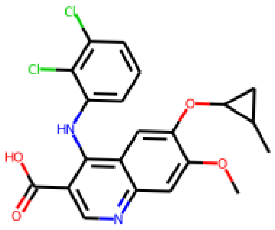	2.0	0.623362	2.525411	2.997881	4	0.774857
**1635**	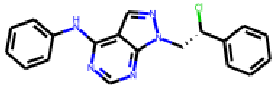	0.534153	Cc1ccc(C(=O)Nc2ccon2)cc1Nc1ncnc2c1cnn2C1cccccc1	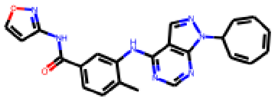	8.0	0.623362	2.525411	2.997881	5	0.770298

	src known mol	similarity	smiles	mol	Class	qed	logP	SAS	rings	kinase inhibition likeliness
**1636**	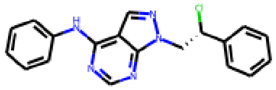	0.534153	Cc1ccc(C(=O)Nc2ccon2)cc1Nc1ncnc2c1cnn2C1cccccc1	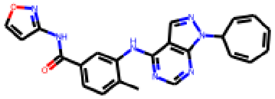	8.0	0.623362	2.525411	2.997881	5	0.770298
**1637**	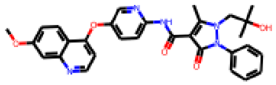	0.534139	COc1ccc2c(=O)cc(CNC(=O)c3c(C)n(C)n(-c4ccccc4)c3=O)nc3cccc2c3c1	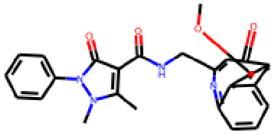	9.0	0.638163	3.995589	2.988471	5	0.774651
**1638**	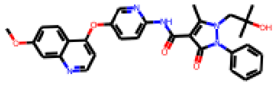	0.534139	COc1ccc2c(=O)cc(CNC(=O)c3c(C)n(C)n(-c4ccccc4)c3=O)nc3cccc2c3c1	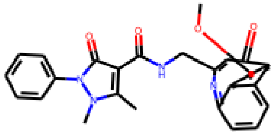	9.0	0.638163	3.995589	2.988471	5	0.774651
**1639**	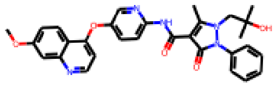	0.534006	COc1ccc2c(=O)cc(CNC(=O)c3c(C)n(C)n(-c4ccccc4)c3=O)nc3cccc2c3c1	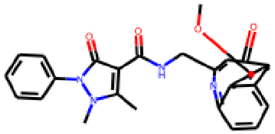	9.0	0.638163	3.995589	2.988471	5	0.774651

	src known mol	similarity	smiles	mol	Class	qed	logP	SAS	rings	kinase inhibition likeliness
**1640**	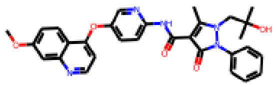	0.534002	COc1ccc2c(=O)cc(CNC(=O)c3c(C)n(C)n(-c4ccccc4)c3=O)nc3cccc3c2n1	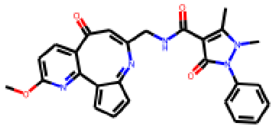	9.0	0.768132	3.608437	2.513170	5	0.823048
**1641**	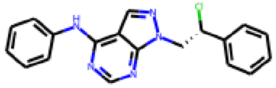	0.533987	Cc1ccc(C(=O)Nc2ccon2)cc1Nc1ncnc2c1cnn2C1cccccc1	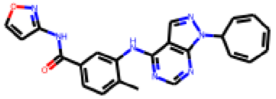	8.0	0.623362	2.525411	2.997881	5	0.770298
**1642**	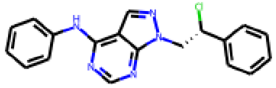	0.533987	Cc1ccc(C(=O)Nc2ccon2)cc1Nc1ncnc2c1cnn2C1cccccc1	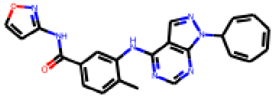	8.0	0.623362	2.525411	2.997881	5	0.770298
**1643**	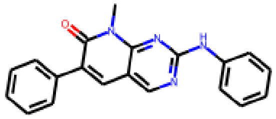	0.533973	Clc1cccc(Nc2ncc3cc(-c3c(Cl)cccc3Cl)c(=O)n(C)c3n2)c1	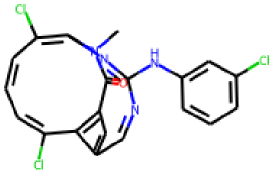	2.0	0.768132	3.608437	2.513170	5	0.784634

	src known mol	similarity	smiles	mol	Class	qed	logP	SAS	rings	kinase inhibition likeliness
**1644**	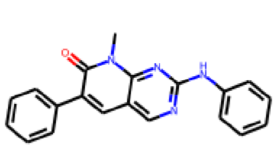	0.533789	Clc1cccc(Nc2ncc3cc(-c3c(Cl)cccc3Cl)c(=O)n(C)c3n2)c1	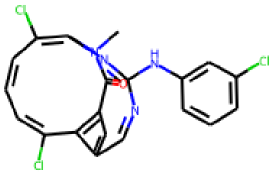	2.0	0.768132	3.608437	2.513170	5	0.784634
**1645**	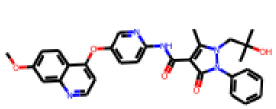	0.533755	COc1ccc2c(=O)cc(CNC(=O)c3c(C)n(C)n(-c4ccccc4)c3=O)nc3cccc2c3c1	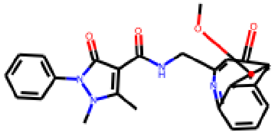	9.0	0.638163	3.995589	2.988471	5	0.774651
**1646**	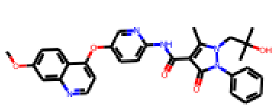	0.533755	COc1ccc2c(=O)cc(CNC(=O)c3c(C)n(C)n(-c4ccccc4)c3=O)nc3cccc2c3c1	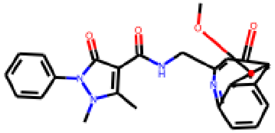	9.0	0.638163	3.995589	2.988471	5	0.774651
**1647**	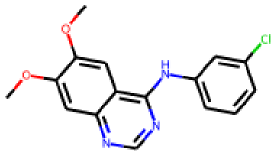	0.533742	Clc1cccc(Nc2ncnc3cc4c[nH]nc4cc23)c1	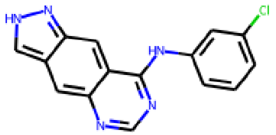	3.0	0.638163	3.995589	2.988471	4	0.781333

	src known mol	similarity	smiles	mol	Class	qed	logP	SAS	rings	kinase inhibition likeliness
**1648**	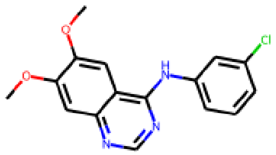	0.533742	Clc1cccc(Nc2ncnc3cc4c[nH]nc4cc23)c1	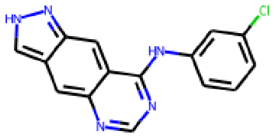	3.0	0.638163	3.995589	2.988471	4	0.781333
**1649**	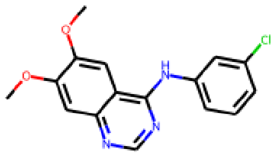	0.533742	Clc1cccc(Nc2ncnc3cc4c[nH]nc4cc23)c1	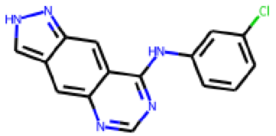	3.0	0.638163	3.995589	2.988471	4	0.781333
**1650**	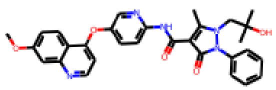	0.533684	COc1ccc2c(=O)cc(CNC(=O)c3c(C)n(C)n(-c4ccccc4)c3=O)nc3cccc2c3c1	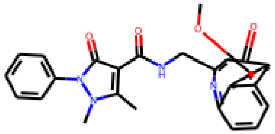	9.0	0.638163	3.995589	2.988471	5	0.774651
**1651**	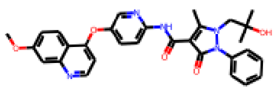	0.533684	COc1ccc2c(=O)cc(CNC(=O)c3c(C)n(C)n(-c4ccccc4)c3=O)nc3cccc2c3c1	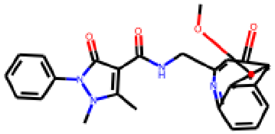	9.0	0.638163	3.995589	2.988471	5	0.774651

	src known mol	similarity	smiles	mol	Class	qed	logP	SAS	rings	kinase inhibition likeliness
**1652**	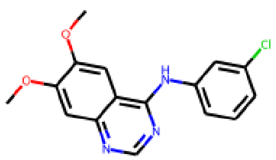	0.533469	Clc1cccc(Nc2ncnc3cc4c[nH]nc4cc23)c1	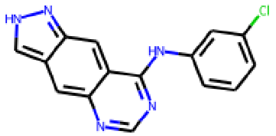	3.0	0.638163	3.995589	2.988471	4	0.781333
**1653**	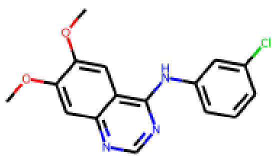	0.533469	Clc1cccc(Nc2ncnc3cc4c[nH]nc4cc23)c1	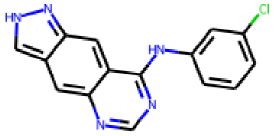	3.0	0.638163	3.995589	2.988471	4	0.781333
**1654**	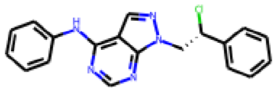	0.533425	Cc1ccc(C(=O)Nc2ccon2)cc1Nc1ncnc2c1cnn2C1cccccc1	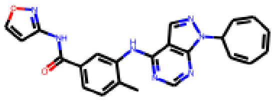	8.0	0.623362	2.525411	2.997881	5	0.770298
**1655**	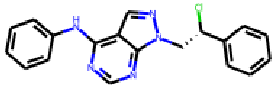	0.533425	Cc1ccc(C(=O)Nc2ccon2)cc1Nc1ncnc2c1cnn2C1cccccc1	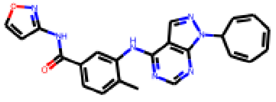	8.0	0.623362	2.525411	2.997881	5	0.770298

	src known mol	similarity	smiles	mol	Class	qed	logP	SAS	rings	kinase inhibition likeliness
**1656**	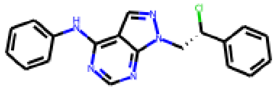	0.533425	Cc1ccc(C(=O)Nc2ccon2)cc1Nc1ncnc2c1cnn2C1cccccc1	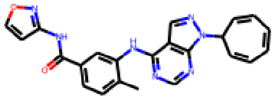	8.0	0.623362	2.525411	2.997881	5	0.770298
**1657**	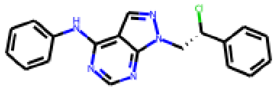	0.533425	Cc1ccc(C(=O)Nc2ccon2)cc1Nc1ncnc2c1cnn2C1cccccc1	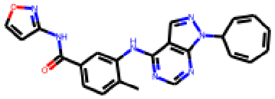	8.0	0.623362	2.525411	2.997881	5	0.770298
**1658**	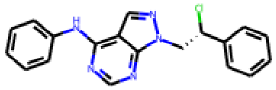	0.533425	Cc1ccc(C(=O)Nc2ccon2)cc1Nc1ncnc2c1cnn2C1cccccc1	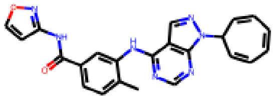	8.0	0.623362	2.525411	2.997881	5	0.770298
**1659**	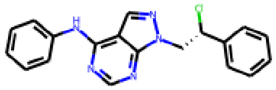	0.533425	Cc1ccc(C(=O)Nc2ccon2)cc1Nc1ncnc2c1cnn2C1cccccc1	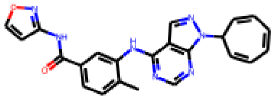	8.0	0.623362	2.525411	2.997881	5	0.770298

	src known mol	similarity	smiles	mol	Class	qed	logP	SAS	rings	kinase inhibition likeliness
**1660**	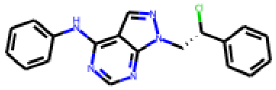	0.533379	Cc1ccc(C(=O)Nc2ccon2)cc1Nc1ncnc2c1cnn2C1cccccc1	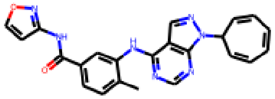	8.0	0.623362	2.525411	2.997881	5	0.770298
**1661**	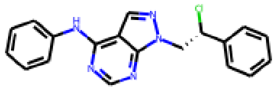	0.533379	Cc1ccc(C(=O)Nc2ccon2)cc1Nc1ncnc2c1cnn2C1cccccc1	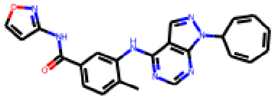	8.0	0.623362	2.525411	2.997881	5	0.770298
**1662**	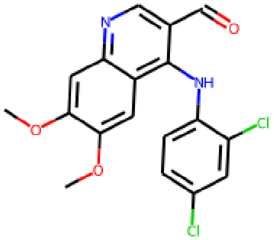	0.533283	COc1cc2ncc(C(=O)O)c(Nc3cccc(Cl)c3Cl)c2cc1OC1C(C)C1	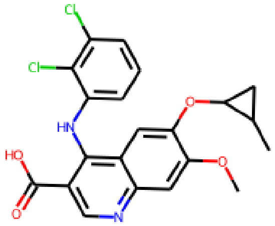	2.0	0.623362	2.525411	2.997881	4	0.774857
**1663**	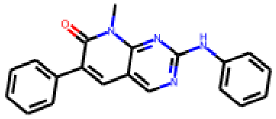	0.533233	Clc1cccc(Nc2ncc3cc(-c3c(Cl)cccc3Cl)c(=O)n(C)c3n2)c1	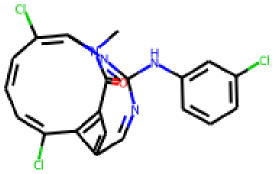	2.0	0.768132	3.608437	2.513170	5	0.784634

	src known mol	similarity	smiles	mol	Class	qed	logP	SAS	rings	kinase inhibition likeliness
**1664**	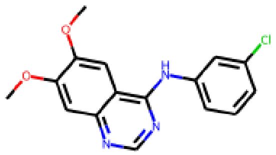	0.533199	Clc1cccc(Nc2ncnc3cc4c[nH]nc4cc23)c1	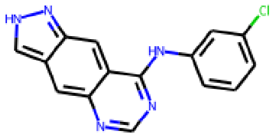	3.0	0.638163	3.995589	2.988471	4	0.781333
**1665**	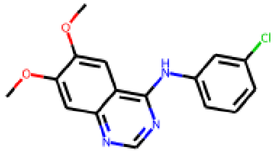	0.533199	Clc1cccc(Nc2ncnc3cc4c[nH]nc4cc23)c1	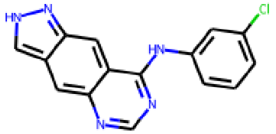	3.0	0.638163	3.995589	2.988471	4	0.781333
**1666**	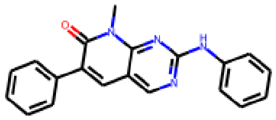	0.533183	Clc1cccc(Nc2ncc3cc(-c3c(Cl)cccc3Cl)c(=O)n(C)c3n2)c1	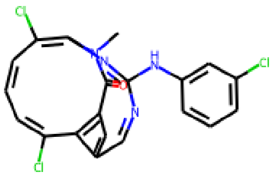	2.0	0.768132	3.608437	2.513170	5	0.784634
**1667**	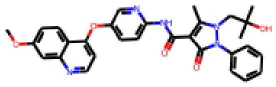	0.533049	COc1ccc2c(=O)cc(CNC(=O)c3c(C)n(C)n(-c4ccccc4)c3=O)nc3cccc2c3c1	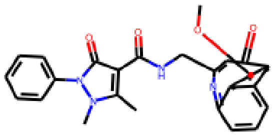	9.0	0.638163	3.995589	2.988471	5	0.774651

	src known mol	similarity	smiles	mol	Class	qed	logP	SAS	rings	kinase inhibition likeliness
**1668**	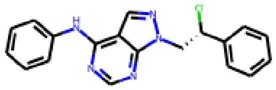	0.532909	Cc1ccc(C(=O)Nc2ccon2)cc1Nc1ncnc2c1cnn2C1cccccc1	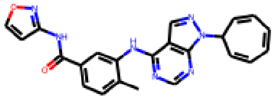	8.0	0.623362	2.525411	2.997881	5	0.770298
**1669**	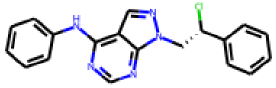	0.532909	Cc1ccc(C(=O)Nc2ccon2)cc1Nc1ncnc2c1cnn2C1cccccc1	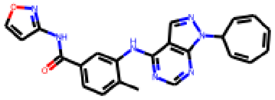	8.0	0.623362	2.525411	2.997881	5	0.770298
**1670**	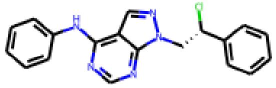	0.532881	Cc1ccc(C(=O)Nc2ccon2)cc1Nc1ncnc2c1cnn2C1cccccc1	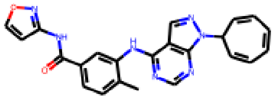	8.0	0.623362	2.525411	2.997881	5	0.770298
**1671**	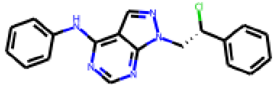	0.532881	Cc1ccc(C(=O)Nc2ccon2)cc1Nc1ncnc2c1cnn2C1cccccc1	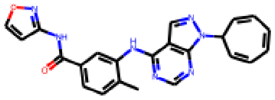	8.0	0.623362	2.525411	2.997881	5	0.770298

	src known mol	similarity	smiles	mol	Class	qed	logP	SAS	rings	kinase inhibition likeliness
**1672**	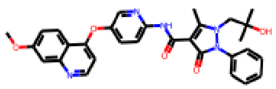	0.532773	COc1ccc2c(=O)cc(CNC(=O)c3c(C)n(C)n(-c4ccccc4)c3=O)nc3cccc3c2n1	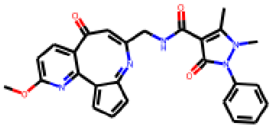	9.0	0.768132	3.608437	2.513170	5	0.823048
**1673**	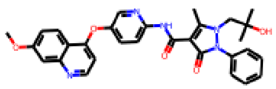	0.532773	COc1ccc2c(=O)cc(CNC(=O)c3c(C)n(C)n(-c4ccccc4)c3=O)nc3cccc3c2n1	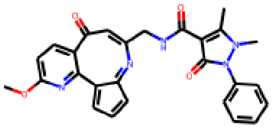	9.0	0.768132	3.608437	2.513170	5	0.823048
**1674**	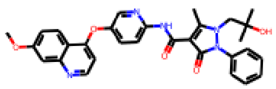	0.532773	COc1ccc2c(=O)cc(CNC(=O)c3c(C)n(C)n(-c4ccccc4)c3=O)nc3cccc3c2n1	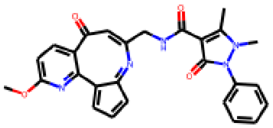	9.0	0.768132	3.608437	2.513170	5	0.823048
**1675**	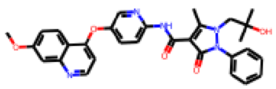	0.532773	COc1ccc2c(=O)cc(CNC(=O)c3c(C)n(C)n(-c4ccccc4)c3=O)nc3cccc3c2n1	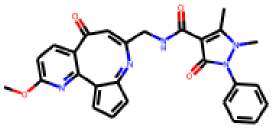	9.0	0.768132	3.608437	2.513170	5	0.823048

	src known mol	similarity	smiles	mol	Class	qed	logP	SAS	rings	kinase inhibition likeliness
**1676**	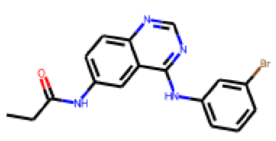	0.532751	O=C(CNC(=O)C1cccccc1)Nc1ccc2ncnc(Nc3cccc(Br)c3)c2c1	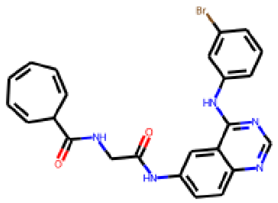	3.0	0.623362	2.525411	2.997881	4	0.772905
**1677**	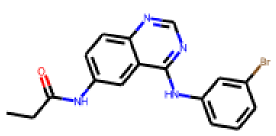	0.532751	O=C(CNC(=O)C1cccccc1)Nc1ccc2ncnc(Nc3cccc(Br)c3)c2c1	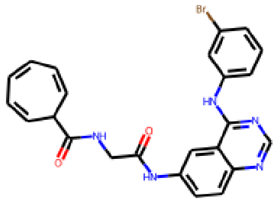	3.0	0.623362	2.525411	2.997881	4	0.772905
**1678**	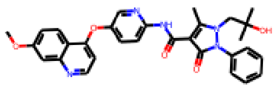	0.532551	COc1ccc2c(=O)cc(CNC(=O)c3c(C)n(C)n(-c4ccccc4)c3=O)nc3cccc2c3c1	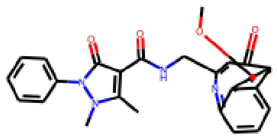	9.0	0.638163	3.995589	2.988471	5	0.774651
**1679**	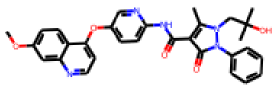	0.532551	COc1ccc2c(=O)cc(CNC(=O)c3c(C)n(C)n(-c4ccccc4)c3=O)nc3cccc2c3c1	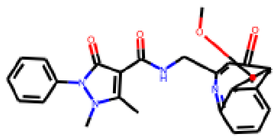	9.0	0.638163	3.995589	2.988471	5	0.774651

	src known mol	similarity	smiles	mol	Class	qed	logP	SAS	rings	kinase inhibition likeliness
**1680**	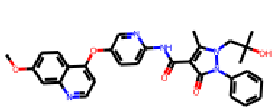	0.532481	COc1ccc2c(=O)cc(CNC(=O)c3c(C)n(C)n(-c4ccccc4)c3=O)nc3cccc2c3c1	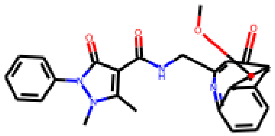	9.0	0.638163	3.995589	2.988471	5	0.774651
**1681**	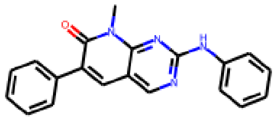	0.532270	Clc1cccc(Nc2ncc3cc(-c3c(Cl)cccc3Cl)c(=O)n(C)c3n2)c1	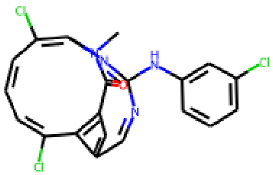	2.0	0.768132	3.608437	2.513170	5	0.784634
**1682**	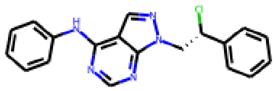	0.532269	Cc1ccc(C(=O)Nc2ccon2)cc1Nc1ncnc2c1cnn2C1cccccc1	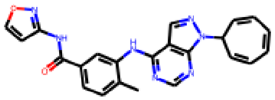	8.0	0.623362	2.525411	2.997881	5	0.770298
**1683**	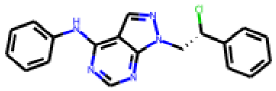	0.532269	Cc1ccc(C(=O)Nc2ccon2)cc1Nc1ncnc2c1cnn2C1cccccc1	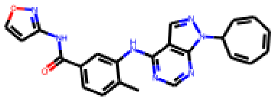	8.0	0.623362	2.525411	2.997881	5	0.770298

	src known mol	similarity	smiles	mol	Class	qed	logP	SAS	rings	kinase inhibition likeliness
**1684**	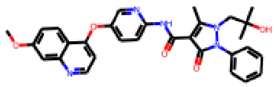	0.532241	COc1ccc2c(=O)cc(CNC(=O)c3c(C)n(C)n(-c4ccccc4)c3=O)nc3cccc2c3c1	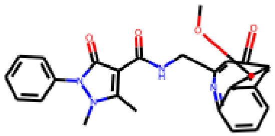	9.0	0.638163	3.995589	2.988471	5	0.774651
**1685**	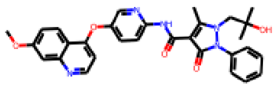	0.532223	COc1ccc2c(=O)cc(CNC(=O)c3c(C)n(C)n(-c4ccccc4)c3=O)nc3cccc2c3c1	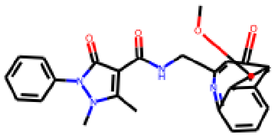	9.0	0.638163	3.995589	2.988471	5	0.774651
**1686**	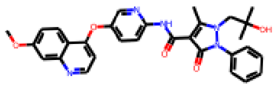	0.532223	COc1ccc2c(=O)cc(CNC(=O)c3c(C)n(C)n(-c4ccccc4)c3=O)nc3cccc2c3c1	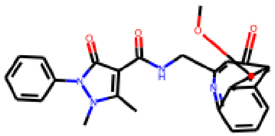	9.0	0.638163	3.995589	2.988471	5	0.774651
**1687**	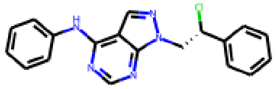	0.532192	Cc1ccc(C(=O)Nc2ccon2)cc1Nc1ncnc2c1cnn2C1cccccc1	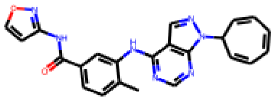	8.0	0.623362	2.525411	2.997881	5	0.770298

	src known mol	similarity	smiles	mol	Class	qed	logP	SAS	rings	kinase inhibition likeliness
**1688**	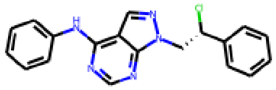	0.532192	Cc1ccc(C(=O)Nc2ccon2)cc1Nc1ncnc2c1cnn2C1cccccc1	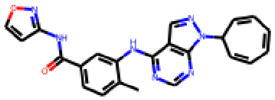	8.0	0.623362	2.525411	2.997881	5	0.770298
**1689**	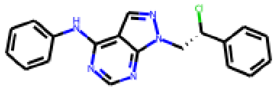	0.532192	Cc1ccc(C(=O)Nc2ccon2)cc1Nc1ncnc2c1cnn2C1cccccc1	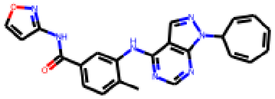	8.0	0.623362	2.525411	2.997881	5	0.770298
**1690**	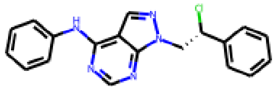	0.532192	Cc1ccc(C(=O)Nc2ccon2)cc1Nc1ncnc2c1cnn2C1cccccc1	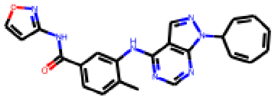	8.0	0.623362	2.525411	2.997881	5	0.770298
**1691**	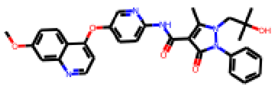	0.532115	COc1ccc2c(=O)cc(CNC(=O)c3c(C)n(C)n(-c4ccccc4)c3=O)nc3cccc3c2n1	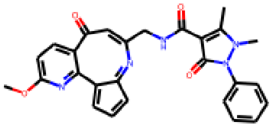	9.0	0.768132	3.608437	2.513170	5	0.823048

	src known mol	similarity	smiles	mol	Class	qed	logP	SAS	rings	kinase inhibition likeliness
**1692**	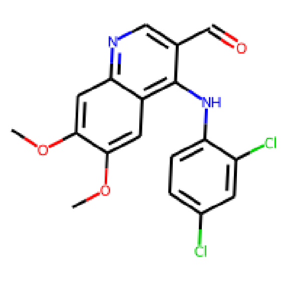	0.532091	COc1cc2ncc(C(=O)O)c(Nc3cccc(Cl)c3Cl)c2cc1OC1C(C)C1	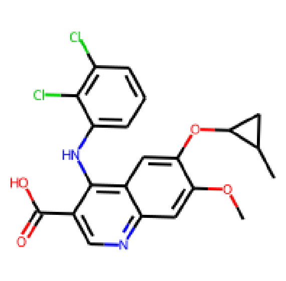	2.0	0.623362	2.525411	2.997881	4	0.774857
**1693**	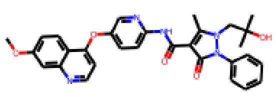	0.532086	COc1ccc2c(=O)cc(CNC(=O)c3c(C)n(C)n(-c4ccccc4)c3=O)nc3cccc2c3c1	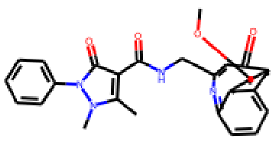	9.0	0.638163	3.995589	2.988471	5	0.774651
**1694**	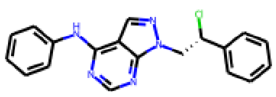	0.532003	Cc1ccc(C(=O)Nc2ccon2)cc1Nc1ncnc2c1cnn2C1cccccc1	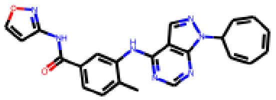	8.0	0.623362	2.525411	2.997881	5	0.770298
**1695**	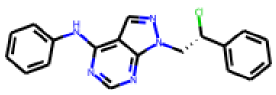	0.532003	Cc1ccc(C(=O)Nc2ccon2)cc1Nc1ncnc2c1cnn2C1cccccc1	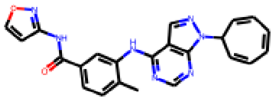	8.0	0.623362	2.525411	2.997881	5	0.770298

	src known mol	similarity	smiles	mol	Class	qed	logP	SAS	rings	kinase inhibition likeliness
**1696**	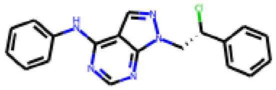	0.531812	Cc1ccc(C(=O)Nc2ccon2)cc1Nc1ncnc2c1cnn2C1cccccc1	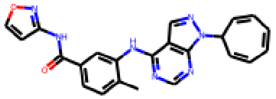	8.0	0.623362	2.525411	2.997881	5	0.770298
**1697**	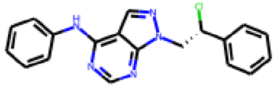	0.531812	Cc1ccc(C(=O)Nc2ccon2)cc1Nc1ncnc2c1cnn2C1cccccc1	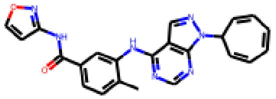	8.0	0.623362	2.525411	2.997881	5	0.770298
**1698**	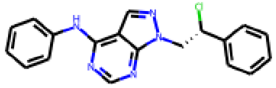	0.531812	Cc1ccc(C(=O)Nc2ccon2)cc1Nc1ncnc2c1cnn2C1cccccc1	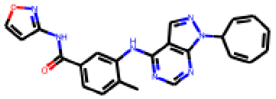	8.0	0.623362	2.525411	2.997881	5	0.770298
**1699**	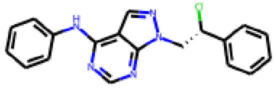	0.531812	Cc1ccc(C(=O)Nc2ccon2)cc1Nc1ncnc2c1cnn2C1cccccc1	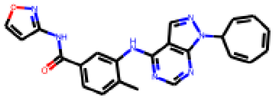	8.0	0.623362	2.525411	2.997881	5	0.770298

	src known mol	similarity	smiles	mol	Class	qed	logP	SAS	rings	kinase inhibition likeliness
**1700**	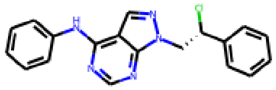	0.531812	Cc1ccc(C(=O)Nc2ccon2)cc1Nc1ncnc2c1cnn2C1cccccc1	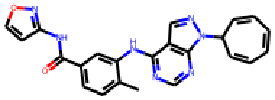	8.0	0.623362	2.525411	2.997881	5	0.770298
**1701**	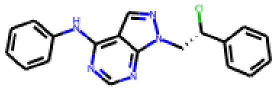	0.531812	Cc1ccc(C(=O)Nc2ccon2)cc1Nc1ncnc2c1cnn2C1cccccc1	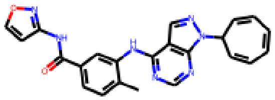	8.0	0.623362	2.525411	2.997881	5	0.770298
**1702**	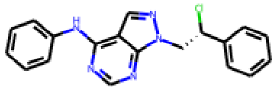	0.531812	Cc1ccc(C(=O)Nc2ccon2)cc1Nc1ncnc2c1cnn2C1cccccc1	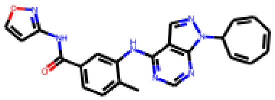	8.0	0.623362	2.525411	2.997881	5	0.770298
**1703**	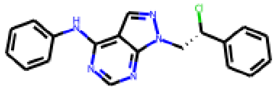	0.531812	Cc1ccc(C(=O)Nc2ccon2)cc1Nc1ncnc2c1cnn2C1cccccc1	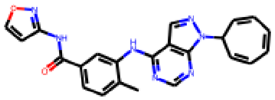	8.0	0.623362	2.525411	2.997881	5	0.770298

	src known mol	similarity	smiles	mol	Class	qed	logP	SAS	rings	kinase inhibition likeliness
**1704**	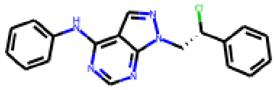	0.531812	Cc1ccc(C(=O)Nc2ccon2)cc1Nc1ncnc2c1cnn2C1cccccc1	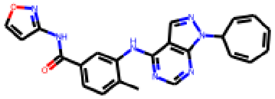	8.0	0.623362	2.525411	2.997881	5	0.770298
**1705**	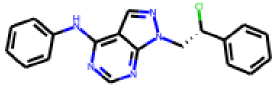	0.531812	Cc1ccc(C(=O)Nc2ccon2)cc1Nc1ncnc2c1cnn2C1cccccc1	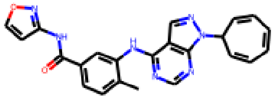	8.0	0.623362	2.525411	2.997881	5	0.770298
**1706**	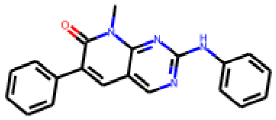	0.531773	Clc1cccc(Nc2ncc3cc(-c3c(Cl)cccc3Cl)c(=O)n(C)c3n2)c1	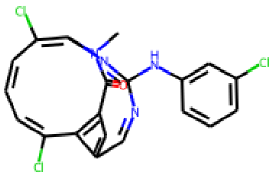	2.0	0.768132	3.608437	2.513170	5	0.784634
**1707**	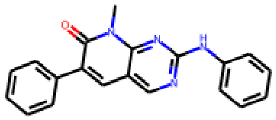	0.531646	Clc1cccc(Nc2ncc3cc(-c3c(Cl)cccc3Cl)c(=O)n(C)c3n2)c1	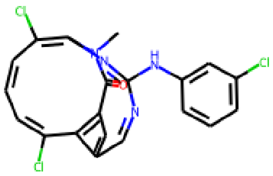	2.0	0.768132	3.608437	2.513170	5	0.784634

	src known mol	similarity	smiles	mol	Class	qed	logP	SAS	rings	kinase inhibition likeliness
**1708**	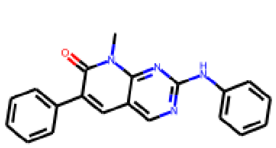	0.531646	Clc1cccc(Nc2ncc3cc(-c3c(Cl)cccc3Cl)c(=O)n(C)c3n2)c1	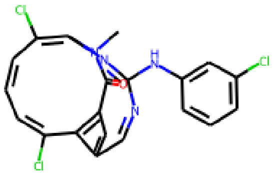	2.0	0.768132	3.608437	2.513170	5	0.784634
**1709**	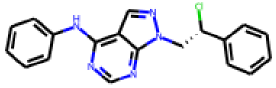	0.531619	Cc1ccc(C(=O)Nc2ccon2)cc1Nc1ncnc2c1cnn2C1cccccc1	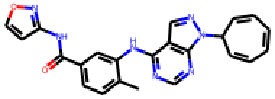	8.0	0.623362	2.525411	2.997881	5	0.770298
**1710**	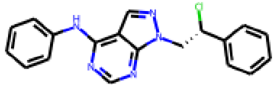	0.531619	Cc1ccc(C(=O)Nc2ccon2)cc1Nc1ncnc2c1cnn2C1cccccc1	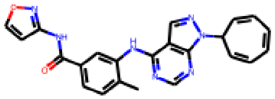	8.0	0.623362	2.525411	2.997881	5	0.770298
**1711**	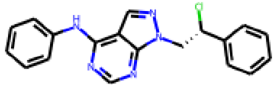	0.531619	Cc1ccc(C(=O)Nc2ccon2)cc1Nc1ncnc2c1cnn2C1cccccc1	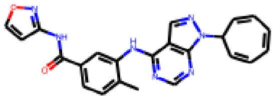	8.0	0.623362	2.525411	2.997881	5	0.770298

	src known mol	similarity	smiles	mol	Class	qed	logP	SAS	rings	kinase inhibition likeliness
**1712**	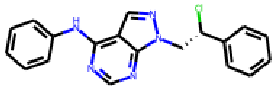	0.531619	Cc1ccc(C(=O)Nc2ccon2)cc1Nc1ncnc2c1cnn2C1cccccc1	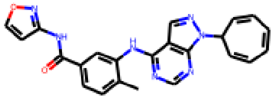	8.0	0.623362	2.525411	2.997881	5	0.770298
**1713**	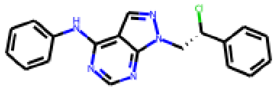	0.531619	Cc1ccc(C(=O)Nc2ccon2)cc1Nc1ncnc2c1cnn2C1cccccc1	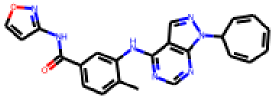	8.0	0.623362	2.525411	2.997881	5	0.770298
**1714**	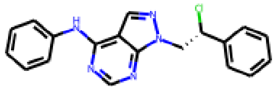	0.531619	Cc1ccc(C(=O)Nc2ccon2)cc1Nc1ncnc2c1cnn2C1cccccc1	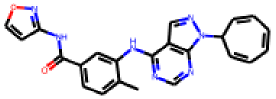	8.0	0.623362	2.525411	2.997881	5	0.770298
**1715**	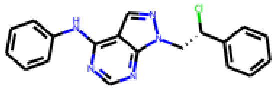	0.531381	Cc1ccc(C(=O)Nc2ccon2)cc1Nc1ncnc2c1cnn2C1cccccc1	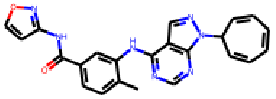	8.0	0.623362	2.525411	2.997881	5	0.770298

	src known mol	similarity	smiles	mol	Class	qed	logP	SAS	rings	kinase inhibition likeliness
**1716**	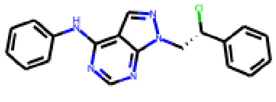	0.531381	Cc1ccc(C(=O)Nc2ccon2)cc1Nc1ncnc2c1cnn2C1cccccc1	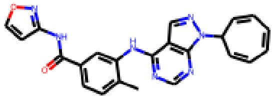	8.0	0.623362	2.525411	2.997881	5	0.770298
**1717**	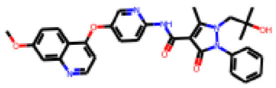	0.531332	COc1ccc2c(=O)cc(CNC(=O)c3c(C)n(C)n(-c4ccccc4)c3=O)nc3cccc2c3c1	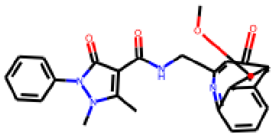	9.0	0.638163	3.995589	2.988471	5	0.774651
**1718**	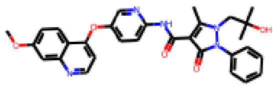	0.531332	COc1ccc2c(=O)cc(CNC(=O)c3c(C)n(C)n(-c4ccccc4)c3=O)nc3cccc2c3c1	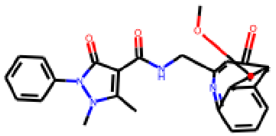	9.0	0.638163	3.995589	2.988471	5	0.774651
**1719**	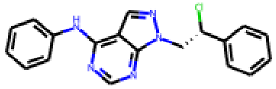	0.531272	Cc1ccc(C(=O)Nc2ccon2)cc1Nc1ncnc2c1cnn2C1cccccc1	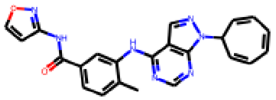	8.0	0.623362	2.525411	2.997881	5	0.770298

	src known mol	similarity	smiles	mol	Class	qed	logP	SAS	rings	kinase inhibition likeliness
**1720**	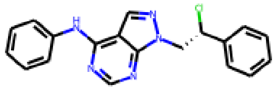	0.531272	Cc1ccc(C(=O)Nc2ccon2)cc1Nc1ncnc2c1cnn2C1cccccc1	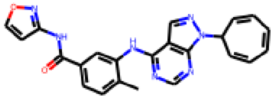	8.0	0.623362	2.525411	2.997881	5	0.770298
**1721**	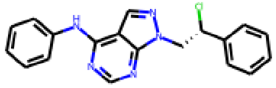	0.531272	Cc1ccc(C(=O)Nc2ccon2)cc1Nc1ncnc2c1cnn2C1cccccc1	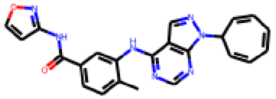	8.0	0.623362	2.525411	2.997881	5	0.770298
**1722**	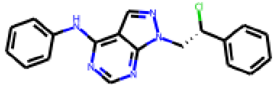	0.531272	Cc1ccc(C(=O)Nc2ccon2)cc1Nc1ncnc2c1cnn2C1cccccc1	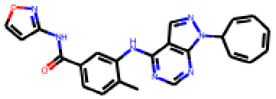	8.0	0.623362	2.525411	2.997881	5	0.770298
**1723**	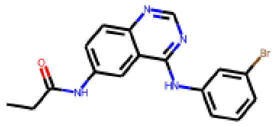	0.531120	O=C(CNC(=O)Oc1ccccc1)Nc1ccc2ncnc(Nc3cccc(Cl)c3)c2c1	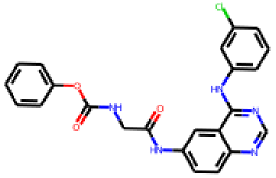	3.0	0.638163	3.995589	2.988471	4	0.793889

	src known mol	similarity	smiles	mol	Class	qed	logP	SAS	rings	kinase inhibition likeliness
**1724**	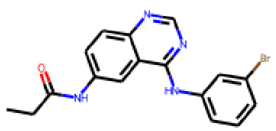	0.531097	O=C(CNC(=O)Oc1ccccc1)Nc1ccc2ncnc(Nc3cccc(Cl)c3)c2c1	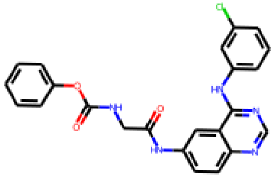	3.0	0.638163	3.995589	2.988471	4	0.793889
**1725**	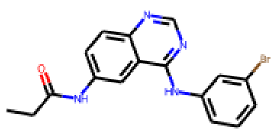	0.531097	O=C(CNC(=O)Oc1ccccc1)Nc1ccc2ncnc(Nc3cccc(Cl)c3)c2c1	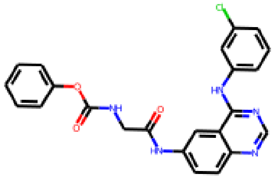	3.0	0.638163	3.995589	2.988471	4	0.793889
**1726**	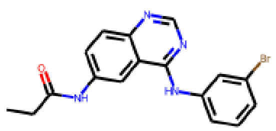	0.531097	O=C(CNC(=O)Oc1ccccc1)Nc1ccc2ncnc(Nc3cccc(Cl)c3)c2c1	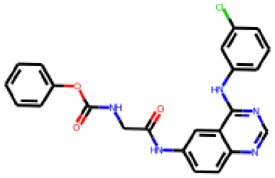	3.0	0.638163	3.995589	2.988471	4	0.793889
**1727**	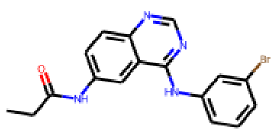	0.531097	O=C(CNC(=O)Oc1ccccc1)Nc1ccc2ncnc(Nc3cccc(Cl)c3)c2c1	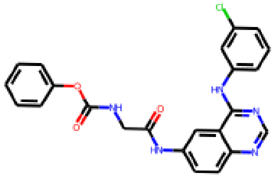	3.0	0.638163	3.995589	2.988471	4	0.793889

	src known mol	similarity	smiles	mol	Class	qed	logP	SAS	rings	kinase inhibition likeliness
**1728**	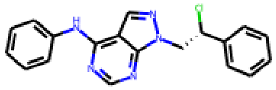	0.530747	Cc1ccc(C(=O)Nc2ccon2)cc1Nc1ncnc2c1cnn2C1cccccc1	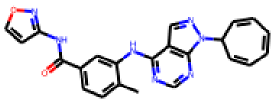	8.0	0.623362	2.525411	2.997881	5	0.770298
**1729**	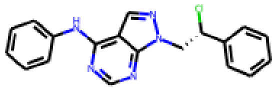	0.530747	Cc1ccc(C(=O)Nc2ccon2)cc1Nc1ncnc2c1cnn2C1cccccc1	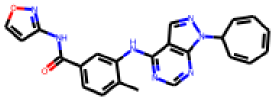	8.0	0.623362	2.525411	2.997881	5	0.770298
**1730**	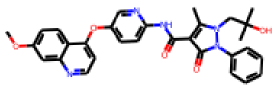	0.530732	COc1ccc2c(=O)cc(CNC(=O)c3c(C)n(C)n(-c4ccccc4)c3=O)nc3cccc2c3c1	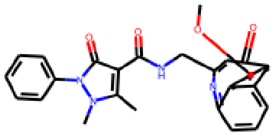	9.0	0.638163	3.995589	2.988471	5	0.774651
**1731**	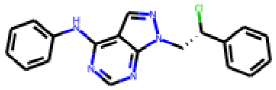	0.530714	Cc1ccc(C(=O)Nc2ccon2)cc1Nc1ncnc2c1cnn2C1cccccc1	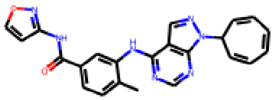	8.0	0.623362	2.525411	2.997881	5	0.770298

	src known mol	similarity	smiles	mol	Class	qed	logP	SAS	rings	kinase inhibition likeliness
**1732**	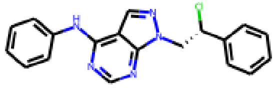	0.530714	Cc1ccc(C(=O)Nc2ccon2)cc1Nc1ncnc2c1cnn2C1cccccc1	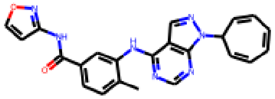	8.0	0.623362	2.525411	2.997881	5	0.770298
**1733**	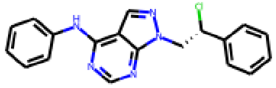	0.530627	Cc1ccc(C(=O)Nc2ccon2)cc1Nc1ncnc2c1cnn2C1cccccc1	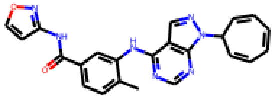	8.0	0.623362	2.525411	2.997881	5	0.770298
**1734**	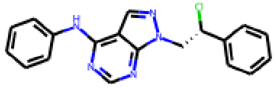	0.530627	Cc1ccc(C(=O)Nc2ccon2)cc1Nc1ncnc2c1cnn2C1cccccc1	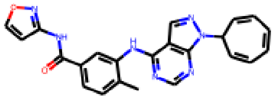	8.0	0.623362	2.525411	2.997881	5	0.770298
**1735**	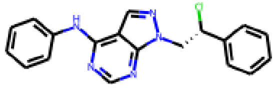	0.530598	Cc1ccc(C(=O)Nc2ccon2)cc1Nc1ncnc2c1cnn2C1cccccc1	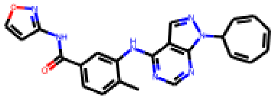	8.0	0.623362	2.525411	2.997881	5	0.770298

	src known mol	similarity	smiles	mol	Class	qed	logP	SAS	rings	kinase inhibition likeliness
**1736**	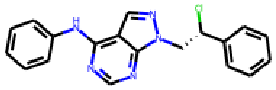	0.530598	Cc1ccc(C(=O)Nc2ccon2)cc1Nc1ncnc2c1cnn2C1cccccc1	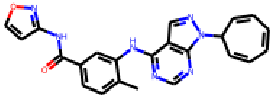	8.0	0.623362	2.525411	2.997881	5	0.770298
**1737**	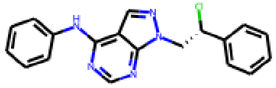	0.530516	Cc1ccc(C(=O)Nc2ccon2)cc1Nc1ncnc2c1cnn2C1cccccc1	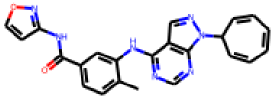	8.0	0.623362	2.525411	2.997881	5	0.770298
**1738**	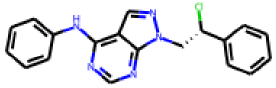	0.530516	Cc1ccc(C(=O)Nc2ccon2)cc1Nc1ncnc2c1cnn2C1cccccc1	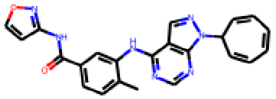	8.0	0.623362	2.525411	2.997881	5	0.770298
**1739**	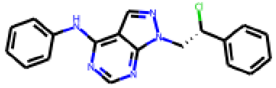	0.530516	Cc1ccc(C(=O)Nc2ccon2)cc1Nc1ncnc2c1cnn2C1cccccc1	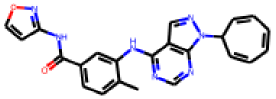	8.0	0.623362	2.525411	2.997881	5	0.770298

	src known mol	similarity	smiles	mol	Class	qed	logP	SAS	rings	kinase inhibition likeliness
**1740**	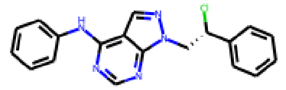	0.530516	Cc1ccc(C(=O)Nc2ccon2)cc1Nc1ncnc2c1cnn2C1cccccc1	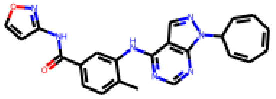	8.0	0.623362	2.525411	2.997881	5	0.770298
**1741**	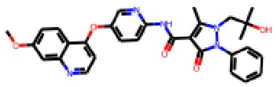	0.530464	COc1ccc2c(=O)cc(CNC(=O)c3c(C)n(C)n(-c4ccccc4)c3=O)nc3cccc3c2n1	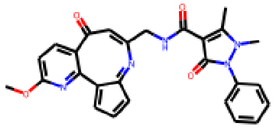	9.0	0.768132	3.608437	2.513170	5	0.823048
**1742**	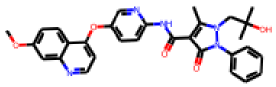	0.530464	COc1ccc2c(=O)cc(CNC(=O)c3c(C)n(C)n(-c4ccccc4)c3=O)nc3cccc3c2n1	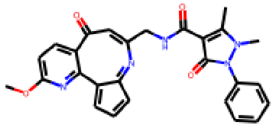	9.0	0.768132	3.608437	2.513170	5	0.823048
**1743**	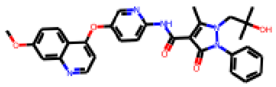	0.530464	COc1ccc2c(=O)cc(CNC(=O)c3c(C)n(C)n(-c4ccccc4)c3=O)nc3cccc3c2n1	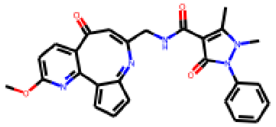	9.0	0.768132	3.608437	2.513170	5	0.823048

	src known mol	similarity	smiles	mol	Class	qed	logP	SAS	rings	kinase inhibition likeliness
**1744**	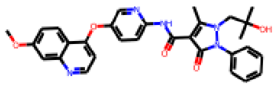	0.530464	COc1ccc2c(=O)cc(CNC(=O)c3c(C)n(C)n(-c4ccccc4)c3=O)nc3cccc3c2n1	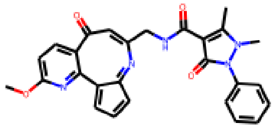	9.0	0.768132	3.608437	2.513170	5	0.823048
**1745**	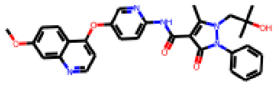	0.530430	COc1ccc2c(=O)cc(CNC(=O)c3c(C)n(C)n(-c4ccccc4)c3=O)nc3cccc2c3c1	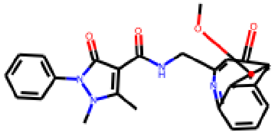	9.0	0.638163	3.995589	2.988471	5	0.774651
**1746**	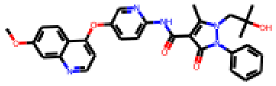	0.530430	COc1ccc2c(=O)cc(CNC(=O)c3c(C)n(C)n(-c4ccccc4)c3=O)nc3cccc2c3c1	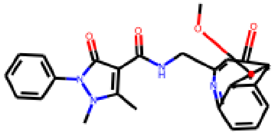	9.0	0.638163	3.995589	2.988471	5	0.774651
**1747**	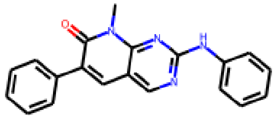	0.530428	Clc1cccc(Nc2ncc3cc(-c3c(Cl)cccc3Cl)c(=O)n(C)c3n2)c1	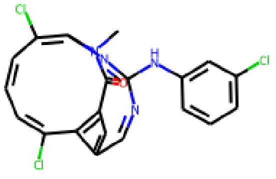	2.0	0.768132	3.608437	2.513170	5	0.784634

	src known mol	similarity	smiles	mol	Class	qed	logP	SAS	rings	kinase inhibition likeliness
**1748**	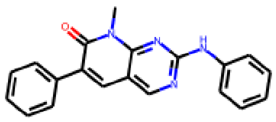	0.530428	Clc1cccc(Nc2ncc3cc(-c3c(Cl)cccc3Cl)c(=O)n(C)c3n2)c1	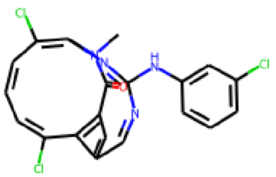	2.0	0.768132	3.608437	2.513170	5	0.784634
**1749**	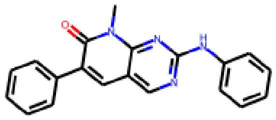	0.530428	Clc1cccc(Nc2ncc3cc(-c3c(Cl)cccc3Cl)c(=O)n(C)c3n2)c1	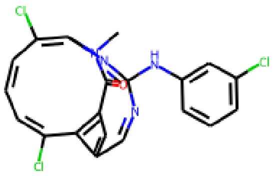	2.0	0.768132	3.608437	2.513170	5	0.784634
**1750**	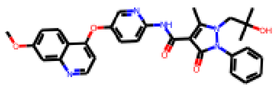	0.530311	COc1ccc2c(=O)cc(CNC(=O)c3c(C)n(C)n(-c4ccccc4)c3=O)nc3cccc2c3c1	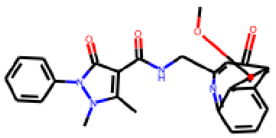	9.0	0.638163	3.995589	2.988471	5	0.774651
**1751**	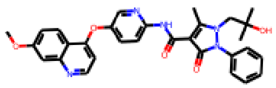	0.530247	COc1ccc2c(=O)cc(CNC(=O)c3c(C)n(C)n(-c4ccccc4)c3=O)nc3cccc2c3c1	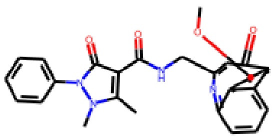	9.0	0.638163	3.995589	2.988471	5	0.774651

	src known mol	similarity	smiles	mol	Class	qed	logP	SAS	rings	kinase inhibition likeliness
**1752**	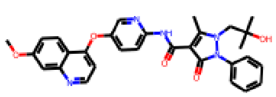	0.530247	COc1ccc2c(=O)cc(CNC(=O)c3c(C)n(C)n(-c4ccccc4)c3=O)nc3cccc2c3c1	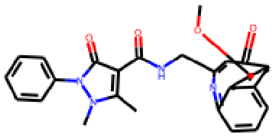	9.0	0.638163	3.995589	2.988471	5	0.774651
**1753**	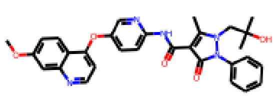	0.530159	COc1ccc2c(=O)cc(CNC(=O)c3c(C)n(C)n(-c4ccccc4)c3=O)nc3cccc2c3c1	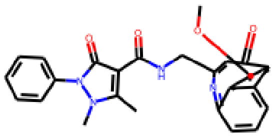	9.0	0.638163	3.995589	2.988471	5	0.774651
**1754**	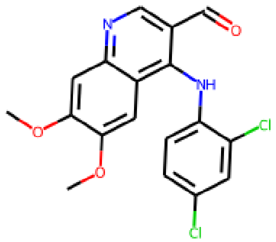	0.530137	COc1cc2ncc(C(=O)O)c(Nc3cccc(Cl)c3Cl)c2cc1OC1C(C)C1	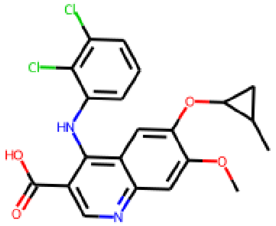	2.0	0.623362	2.525411	2.997881	4	0.774857
**1755**	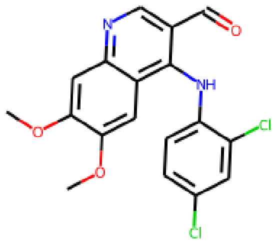	0.530137	COc1cc2ncc(C(=O)O)c(Nc3cccc(Cl)c3Cl)c2cc1OC1C(C)C1	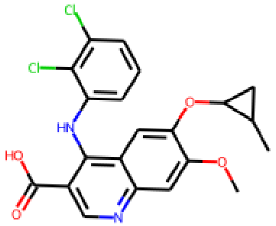	2.0	0.623362	2.525411	2.997881	4	0.774857

	src known mol	similarity	smiles	mol	Class	qed	logP	SAS	rings	kinase inhibition likeliness
**1756**	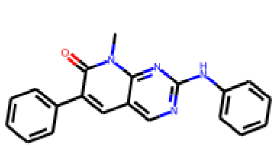	0.530075	Clc1cccc(Nc2ncc3cc(-c3c(Cl)cccc3Cl)c(=O)n(C)c3n2)c1	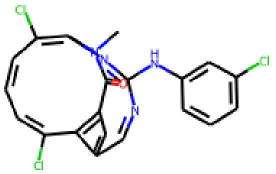	2.0	0.768132	3.608437	2.513170	5	0.784634
**1757**	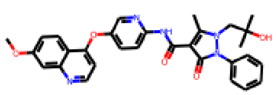	0.530069	COc1ccc2c(=O)cc(CNC(=O)c3c(C)n(C)n(-c4ccccc4)c3=O)nc3cccc2c3c1	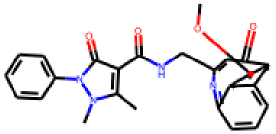	9.0	0.638163	3.995589	2.988471	5	0.774651
**1758**	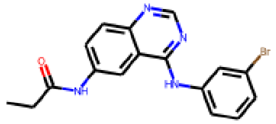	0.530032	O=C(CNC(=O)Oc1ccccc1)Nc1ccc2ncnc(Nc3cccc(Cl)c3)c2c1	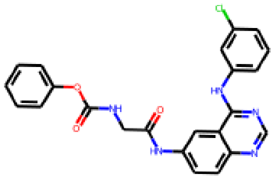	3.0	0.638163	3.995589	2.988471	4	0.793889
**1759**	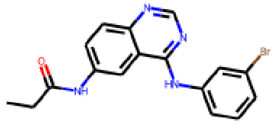	0.530032	O=C(CNC(=O)Oc1ccccc1)Nc1ccc2ncnc(Nc3cccc(Cl)c3)c2c1	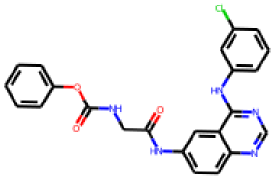	3.0	0.638163	3.995589	2.988471	4	0.793889

	src known mol	similarity	smiles	mol	Class	qed	logP	SAS	rings	kinase inhibition likeliness
**1760**	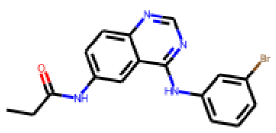	0.530032	O=C(CNC(=O)Oc1ccccc1)Nc1ccc2ncnc(Nc3cccc(Cl)c3)c2c1	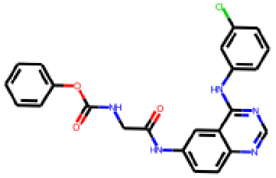	3.0	0.638163	3.995589	2.988471	4	0.793889
**1761**	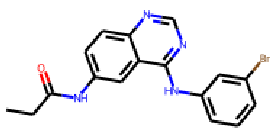	0.530032	O=C(CNC(=O)Oc1ccccc1)Nc1ccc2ncnc(Nc3cccc(Cl)c3)c2c1	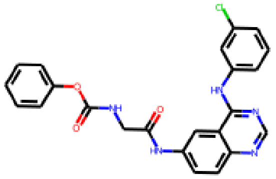	3.0	0.638163	3.995589	2.988471	4	0.793889
**1762**	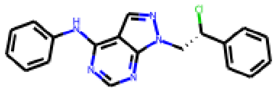	0.530007	Cc1ccc(C(=O)Nc2ccon2)cc1Nc1ncnc2c1cnn2C1cccccc1	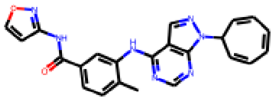	8.0	0.623362	2.525411	2.997881	5	0.770298
**1763**	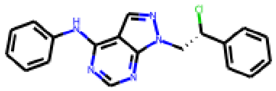	0.530007	Cc1ccc(C(=O)Nc2ccon2)cc1Nc1ncnc2c1cnn2C1cccccc1	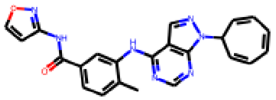	8.0	0.623362	2.525411	2.997881	5	0.770298

	src known mol	similarity	smiles	mol	Class	qed	logP	SAS	rings	kinase inhibition likeliness
**1764**	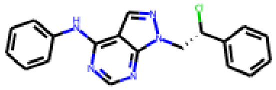	0.530007	Cc1ccc(C(=O)Nc2ccon2)cc1Nc1ncnc2c1cnn2C1cccccc1	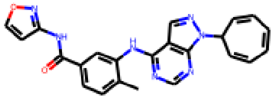	8.0	0.623362	2.525411	2.997881	5	0.770298
**1765**	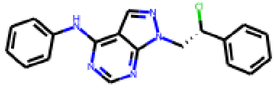	0.530007	Cc1ccc(C(=O)Nc2ccon2)cc1Nc1ncnc2c1cnn2C1cccccc1	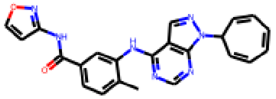	8.0	0.623362	2.525411	2.997881	5	0.770298
**1766**	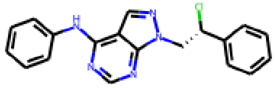	0.529696	Cc1ccc(C(=O)Nc2ccon2)cc1Nc1ncnc2c1cnn2C1cccccc1	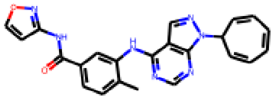	8.0	0.623362	2.525411	2.997881	5	0.770298
**1767**	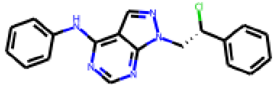	0.529696	Cc1ccc(C(=O)Nc2ccon2)cc1Nc1ncnc2c1cnn2C1cccccc1	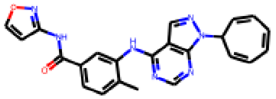	8.0	0.623362	2.525411	2.997881	5	0.770298

	src known mol	similarity	smiles	mol	Class	qed	logP	SAS	rings	kinase inhibition likeliness
**1768**	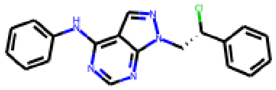	0.529696	Cc1ccc(C(=O)Nc2ccon2)cc1Nc1ncnc2c1cnn2C1cccccc1	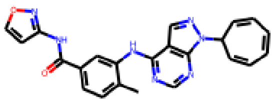	8.0	0.623362	2.525411	2.997881	5	0.770298
**1769**	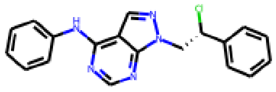	0.529696	Cc1ccc(C(=O)Nc2ccon2)cc1Nc1ncnc2c1cnn2C1cccccc1	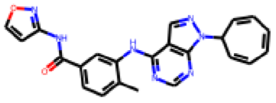	8.0	0.623362	2.525411	2.997881	5	0.770298
**1770**	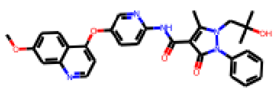	0.529690	COc1ccc2c(=O)cc(CNC(=O)c3c(C)n(C)n(-c4ccccc4)c3=O)nc3cccc2c3c1	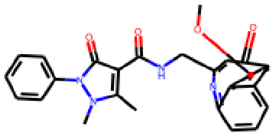	9.0	0.638163	3.995589	2.988471	5	0.774651
**1771**	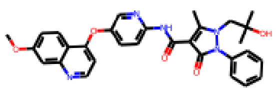	0.529690	COc1ccc2c(=O)cc(CNC(=O)c3c(C)n(C)n(-c4ccccc4)c3=O)nc3cccc2c3c1	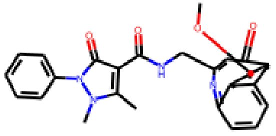	9.0	0.638163	3.995589	2.988471	5	0.774651

	src known mol	similarity	smiles	mol	Class	qed	logP	SAS	rings	kinase inhibition likeliness
**1772**	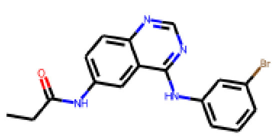	0.529597	O=C(CNC(=O)Oc1ccccc1)Nc1ccc2ncnc(Nc3cccc(Cl)c3)c2c1	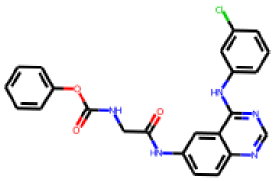	3.0	0.638163	3.995589	2.988471	4	0.793889
**1773**	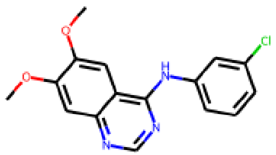	0.529412	Clc1cccc(Nc2ncnc3cc4c[nH]nc4cc23)c1	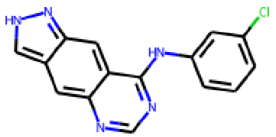	3.0	0.638163	3.995589	2.988471	4	0.781333
**1774**	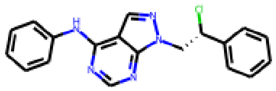	0.529332	Cc1ccc(C(=O)Nc2ccon2)cc1Nc1ncnc2c1cnn2C1cccccc1	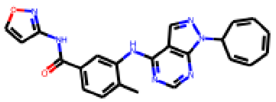	8.0	0.623362	2.525411	2.997881	5	0.770298
**1775**	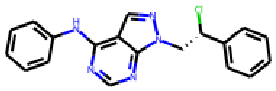	0.529332	Cc1ccc(C(=O)Nc2ccon2)cc1Nc1ncnc2c1cnn2C1cccccc1	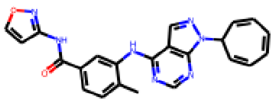	8.0	0.623362	2.525411	2.997881	5	0.770298

	src known mol	similarity	smiles	mol	Class	qed	logP	SAS	rings	kinase inhibition likeliness
**1776**	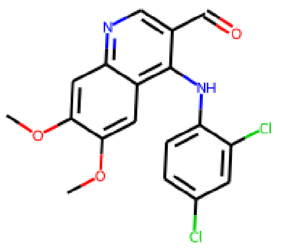	0.529321	COc1cc2ncc(C(=O)O)c(Nc3cccc(Cl)c3Cl)c2cc1OC1C(C)C1	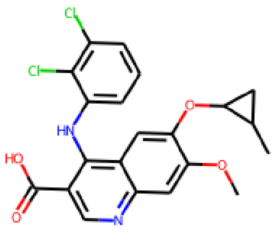	2.0	0.623362	2.525411	2.997881	4	0.774857
**1777**	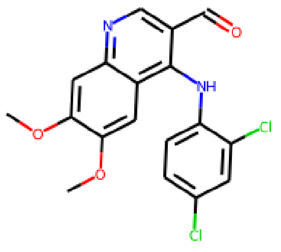	0.529250	COc1cc2ncc(C(=O)O)c(Nc3cccc(Cl)c3Cl)c2cc1OC1C(C)C1	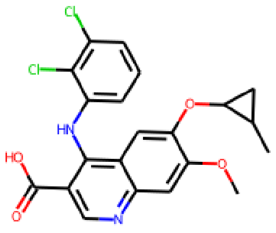	2.0	0.623362	2.525411	2.997881	4	0.774857
**1778**	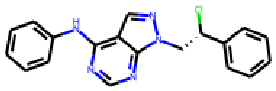	0.529238	Cc1ccc(C(=O)Nc2ccon2)cc1Nc1ncnc2c1cnn2C1cccccc1	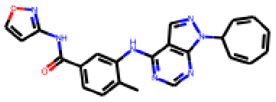	8.0	0.623362	2.525411	2.997881	5	0.770298
**1779**	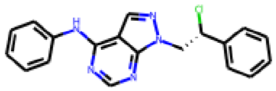	0.529238	Cc1ccc(C(=O)Nc2ccon2)cc1Nc1ncnc2c1cnn2C1cccccc1	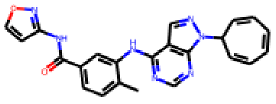	8.0	0.623362	2.525411	2.997881	5	0.770298

	src known mol	similarity	smiles	mol	Class	qed	logP	SAS	rings	kinase inhibition likeliness
**1780**	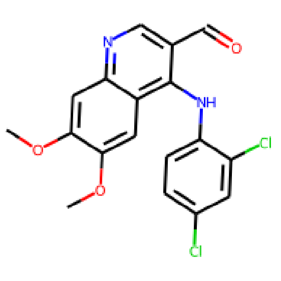	0.529210	COc1cc2ncc(C(=O)O)c(Nc3cccc(Cl)c3Cl)c2cc1OC1C(C)C1	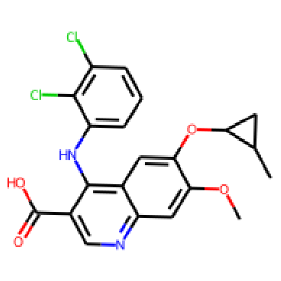	2.0	0.623362	2.525411	2.997881	4	0.774857
**1781**	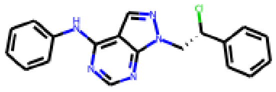	0.529192	Cc1ccc(C(=O)Nc2ccon2)cc1Nc1ncnc2c1cnn2C1cccccc1	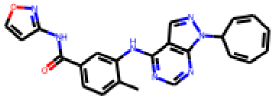	8.0	0.623362	2.525411	2.997881	5	0.770298
**1782**	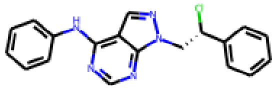	0.529172	Cc1ccc(C(=O)Nc2ccon2)cc1Nc1ncnc2c1cnn2C1cccccc1	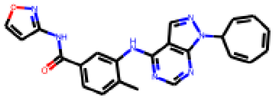	8.0	0.623362	2.525411	2.997881	5	0.770298
**1783**	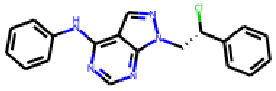	0.529172	Cc1ccc(C(=O)Nc2ccon2)cc1Nc1ncnc2c1cnn2C1cccccc1	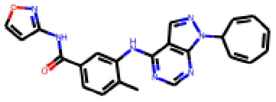	8.0	0.623362	2.525411	2.997881	5	0.770298

	src known mol	similarity	smiles	mol	Class	qed	logP	SAS	rings	kinase inhibition likeliness
**1784**	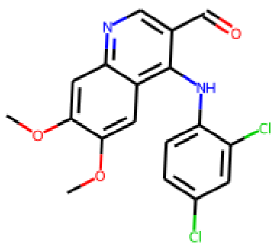	0.529167	COc1cc2ncc(C(=O)O)c(Nc3cccc(Cl)c3Cl)c2cc1OC1C(C)C1	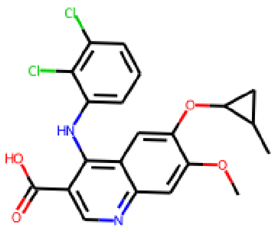	2.0	0.623362	2.525411	2.997881	4	0.774857
**1785**	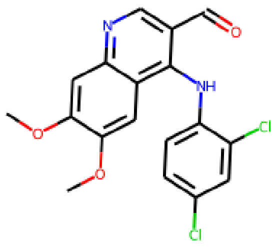	0.529167	COc1cc2ncc(C(=O)O)c(Nc3cccc(Cl)c3Cl)c2cc1OC1C(C)C1	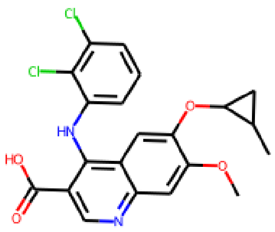	2.0	0.623362	2.525411	2.997881	4	0.774857
**1786**	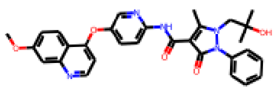	0.529008	COc1ccc2c(=O)cc(CNC(=O)c3c(C)n(C)n(-c4ccccc4)c3=O)nc3cccc2c3c1	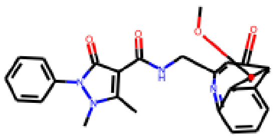	9.0	0.638163	3.995589	2.988471	5	0.774651
**1787**	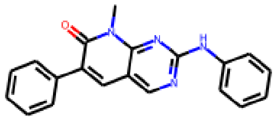	0.528727	Clc1cccc(Nc2ncc3cc(-c3c(Cl)cccc3Cl)c(=O)n(C)c3n2)c1	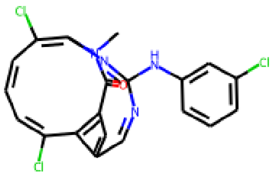	2.0	0.768132	3.608437	2.513170	5	0.784634

	src known mol	similarity	smiles	mol	Class	qed	logP	SAS	rings	kinase inhibition likeliness
**1788**	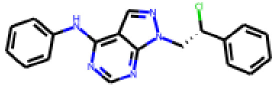	0.528551	Cc1ccc(C(=O)Nc2ccon2)cc1Nc1ncnc2c1cnn2C1cccccc1	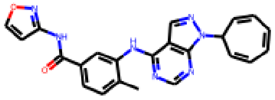	8.0	0.623362	2.525411	2.997881	5	0.770298
**1789**	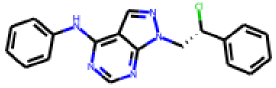	0.528551	Cc1ccc(C(=O)Nc2ccon2)cc1Nc1ncnc2c1cnn2C1cccccc1	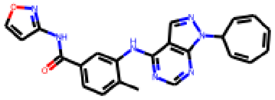	8.0	0.623362	2.525411	2.997881	5	0.770298
**1790**	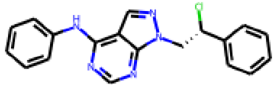	0.528551	Cc1ccc(C(=O)Nc2ccon2)cc1Nc1ncnc2c1cnn2C1cccccc1	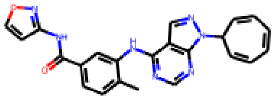	8.0	0.623362	2.525411	2.997881	5	0.770298
**1791**	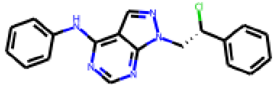	0.528551	Cc1ccc(C(=O)Nc2ccon2)cc1Nc1ncnc2c1cnn2C1cccccc1	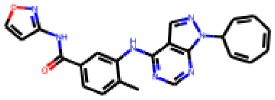	8.0	0.623362	2.525411	2.997881	5	0.770298

	src known mol	similarity	smiles	mol	Class	qed	logP	SAS	rings	kinase inhibition likeliness
**1792**	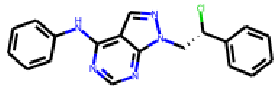	0.528551	Cc1ccc(C(=O)Nc2ccon2)cc1Nc1ncnc2c1cnn2C1cccccc1	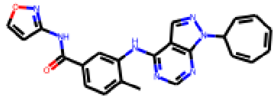	8.0	0.623362	2.525411	2.997881	5	0.770298
**1793**	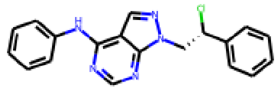	0.528551	Cc1ccc(C(=O)Nc2ccon2)cc1Nc1ncnc2c1cnn2C1cccccc1	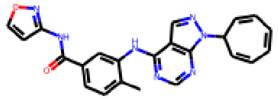	8.0	0.623362	2.525411	2.997881	5	0.770298
**1794**	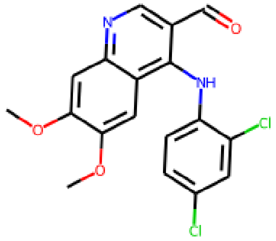	0.528444	COc1cc2ncc(C(=O)O)c(Nc3cccc(Cl)c3Cl)c2cc1OC1C(C)C1	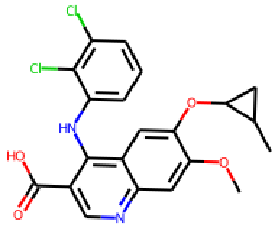	2.0	0.623362	2.525411	2.997881	4	0.774857
**1795**	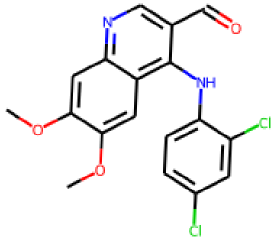	0.528444	COc1cc2ncc(C(=O)O)c(Nc3cccc(Cl)c3Cl)c2cc1OC1C(C)C1	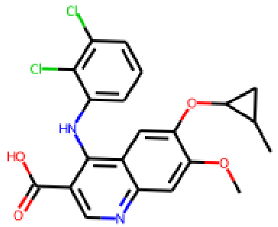	2.0	0.623362	2.525411	2.997881	4	0.774857

	src known mol	similarity	smiles	mol	Class	qed	logP	SAS	rings	kinase inhibition likeliness
**1796**	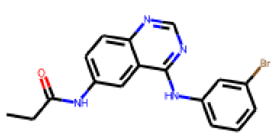	0.528363	O=C(CNC(=O)Oc1ccccc1)Nc1ccc2ncnc(Nc3cccc(Cl)c3)c2c1	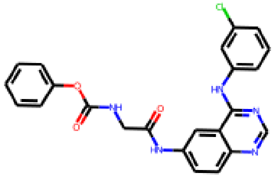	3.0	0.638163	3.995589	2.988471	4	0.793889
**1797**	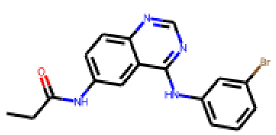	0.528363	O=C(CNC(=O)Oc1ccccc1)Nc1ccc2ncnc(Nc3cccc(Cl)c3)c2c1	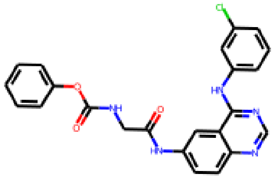	3.0	0.638163	3.995589	2.988471	4	0.793889
**1798**	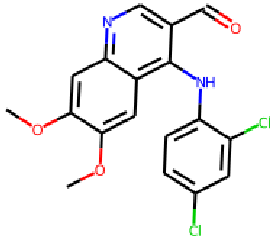	0.528274	COc1cc2ncc(C(=O)O)c(Nc3cccc(Cl)c3Cl)c2cc1OC1C(C)C1	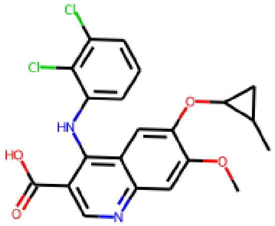	2.0	0.623362	2.525411	2.997881	4	0.774857
**1799**	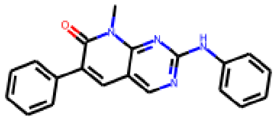	0.528220	Clc1cccc(Nc2ncc3cc(-c3c(Cl)cccc3Cl)c(=O)n(C)c3n2)c1	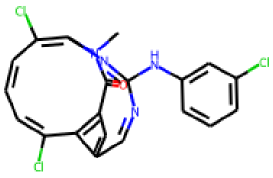	2.0	0.768132	3.608437	2.513170	5	0.784634

	src known mol	similarity	smiles	mol	Class	qed	logP	SAS	rings	kinase inhibition likeliness
**1800**	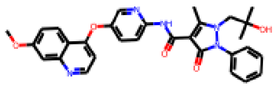	0.528211	COc1ccc2c(=O)cc(CNC(=O)c3c(C)n(C)n(-c4ccccc4)c3=O)nc3cccc2c3c1	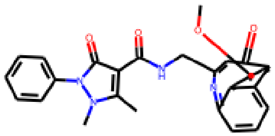	9.0	0.638163	3.995589	2.988471	5	0.774651
**1801**	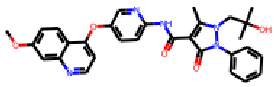	0.528211	COc1ccc2c(=O)cc(CNC(=O)c3c(C)n(C)n(-c4ccccc4)c3=O)nc3cccc2c3c1	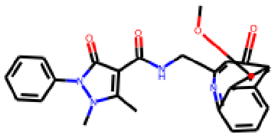	9.0	0.638163	3.995589	2.988471	5	0.774651
**1802**	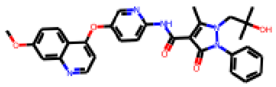	0.528211	COc1ccc2c(=O)cc(CNC(=O)c3c(C)n(C)n(-c4ccccc4)c3=O)nc3cccc2c3c1	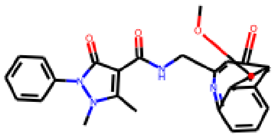	9.0	0.638163	3.995589	2.988471	5	0.774651
**1803**	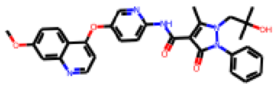	0.528211	COc1ccc2c(=O)cc(CNC(=O)c3c(C)n(C)n(-c4ccccc4)c3=O)nc3cccc2c3c1	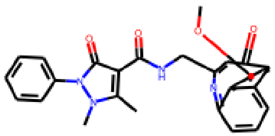	9.0	0.638163	3.995589	2.988471	5	0.774651

	src known mol	similarity	smiles	mol	Class	qed	logP	SAS	rings	kinase inhibition likeliness
**1804**	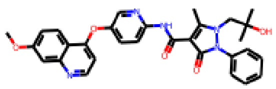	0.528165	COc1ccc2c(=O)cc(CNC(=O)c3c(C)n(C)n(-c4ccccc4)c3=O)nc3cccc3c2n1	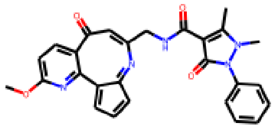	9.0	0.768132	3.608437	2.513170	5	0.823048
**1805**	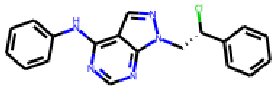	0.528159	Cc1ccc(C(=O)Nc2ccon2)cc1Nc1ncnc2c1cnn2C1cccccc1	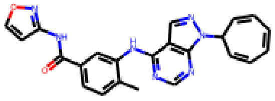	8.0	0.623362	2.525411	2.997881	5	0.770298
**1806**	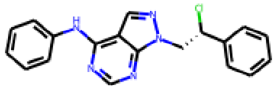	0.528159	Cc1ccc(C(=O)Nc2ccon2)cc1Nc1ncnc2c1cnn2C1cccccc1	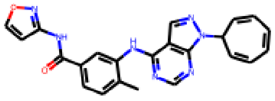	8.0	0.623362	2.525411	2.997881	5	0.770298
**1807**	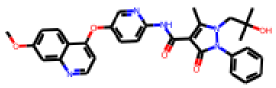	0.528143	COc1ccc2c(=O)cc(CNC(=O)c3c(C)n(C)n(-c4ccccc4)c3=O)nc3cccc2c3c1	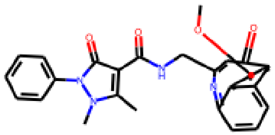	9.0	0.638163	3.995589	2.988471	5	0.774651

	src known mol	similarity	smiles	mol	Class	qed	logP	SAS	rings	kinase inhibition likeliness
**1808**	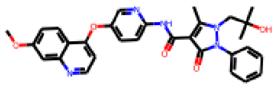	0.528143	COc1ccc2c(=O)cc(CNC(=O)c3c(C)n(C)n(-c4ccccc4)c3=O)nc3cccc2c3c1	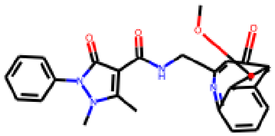	9.0	0.638163	3.995589	2.988471	5	0.774651
**1809**	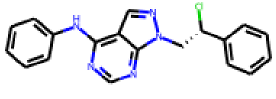	0.528067	Cc1ccc(C(=O)Nc2ccon2)cc1Nc1ncnc2c1cnn2C1cccccc1	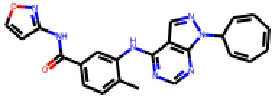	8.0	0.623362	2.525411	2.997881	5	0.770298
**1810**	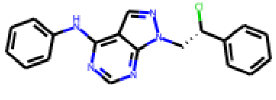	0.528067	Cc1ccc(C(=O)Nc2ccon2)cc1Nc1ncnc2c1cnn2C1cccccc1	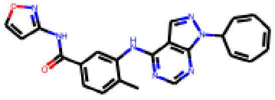	8.0	0.623362	2.525411	2.997881	5	0.770298
**1811**	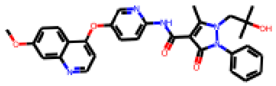	0.528022	COc1ccc2c(=O)cc(CNC(=O)c3c(C)n(C)n(-c4ccccc4)c3=O)nc3cccc3c2n1	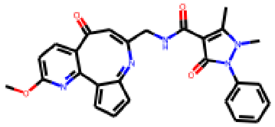	9.0	0.768132	3.608437	2.513170	5	0.823048

	src known mol	similarity	smiles	mol	Class	qed	logP	SAS	rings	kinase inhibition likeliness
**1812**	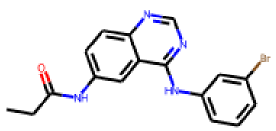	0.528021	O=C(CNC(=O)Oc1ccccc1)Nc1ccc2ncnc(Nc3cccc(Cl)c3)c2c1	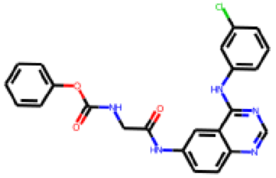	3.0	0.638163	3.995589	2.988471	4	0.793889
**1813**	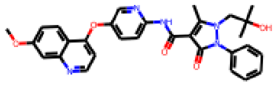	0.527867	COc1ccc2c(=O)cc(CNC(=O)c3c(C)n(C)n(-c4ccccc4)c3=O)nc3cccc2c3c1	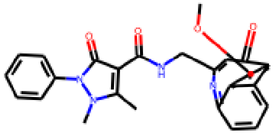	9.0	0.638163	3.995589	2.988471	5	0.774651
**1814**	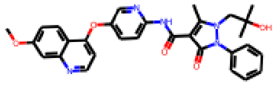	0.527867	COc1ccc2c(=O)cc(CNC(=O)c3c(C)n(C)n(-c4ccccc4)c3=O)nc3cccc2c3c1	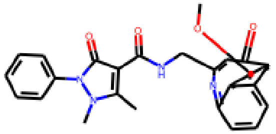	9.0	0.638163	3.995589	2.988471	5	0.774651
**1815**	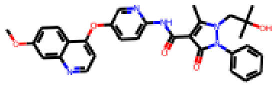	0.527867	COc1ccc2c(=O)cc(CNC(=O)c3c(C)n(C)n(-c4ccccc4)c3=O)nc3cccc2c3c1	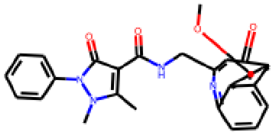	9.0	0.638163	3.995589	2.988471	5	0.774651

	src known mol	similarity	smiles	mol	Class	qed	logP	SAS	rings	kinase inhibition likeliness
**1816**	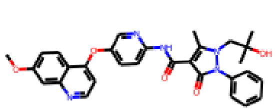	0.527867	COc1ccc2c(=O)cc(CNC(=O)c3c(C)n(C)n(-c4ccccc4)c3=O)nc3cccc2c3c1	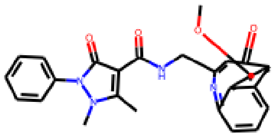	9.0	0.638163	3.995589	2.988471	5	0.774651
**1817**	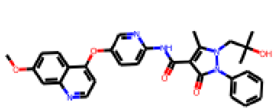	0.527867	COc1ccc2c(=O)cc(CNC(=O)c3c(C)n(C)n(-c4ccccc4)c3=O)nc3cccc2c3c1	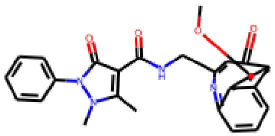	9.0	0.638163	3.995589	2.988471	5	0.774651
**1818**	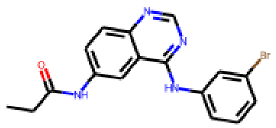	0.527823	O=C(CNC(=O)Oc1ccccc1)Nc1ccc2ncnc(Nc3cccc(Cl)c3)c2c1	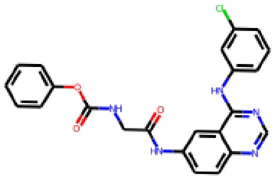	3.0	0.638163	3.995589	2.988471	4	0.793889
**1819**	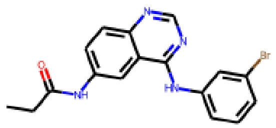	0.527823	O=C(CNC(=O)Oc1ccccc1)Nc1ccc2ncnc(Nc3cccc(Cl)c3)c2c1	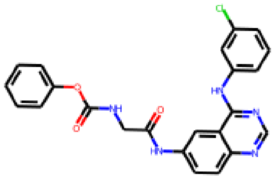	3.0	0.638163	3.995589	2.988471	4	0.793889

	src known mol	similarity	smiles	mol	Class	qed	logP	SAS	rings	kinase inhibition likeliness
**1820**	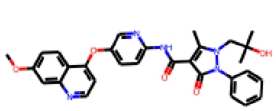	0.527823	COc1ccc2c(=O)cc(CNC(=O)c3c(C)n(C)n(-c4ccccc4)c3=O)nc3cccc2c3c1	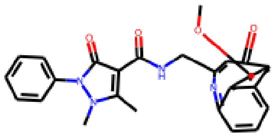	9.0	0.638163	3.995589	2.988471	5	0.774651
**1821**	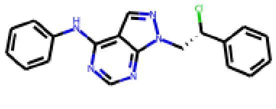	0.527718	Cc1ccc(C(=O)Nc2ccon2)cc1Nc1ncnc2c1cnn2C1cccccc1	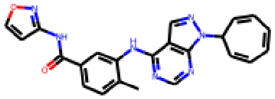	8.0	0.623362	2.525411	2.997881	5	0.770298
**1822**	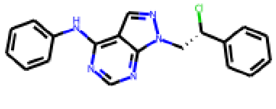	0.527718	Cc1ccc(C(=O)Nc2ccon2)cc1Nc1ncnc2c1cnn2C1cccccc1	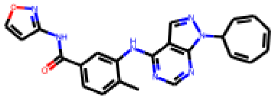	8.0	0.623362	2.525411	2.997881	5	0.770298
**1823**	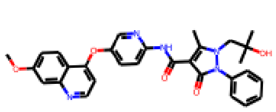	0.527361	COc1ccc2c(=O)cc(CNC(=O)c3c(C)n(C)n(-c4ccccc4)c3=O)nc3cccc2c3c1	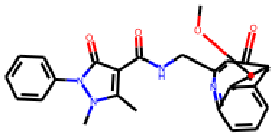	9.0	0.638163	3.995589	2.988471	5	0.774651

	src known mol	similarity	smiles	mol	Class	qed	logP	SAS	rings	kinase inhibition likeliness
**1824**	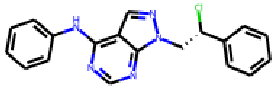	0.527360	Cc1ccc(C(=O)Nc2ccon2)cc1Nc1ncnc2c1cnn2C1cccccc1	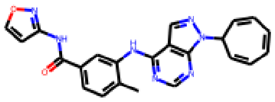	8.0	0.623362	2.525411	2.997881	5	0.770298
**1825**	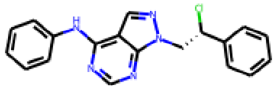	0.527360	Cc1ccc(C(=O)Nc2ccon2)cc1Nc1ncnc2c1cnn2C1cccccc1	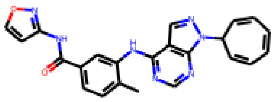	8.0	0.623362	2.525411	2.997881	5	0.770298
**1826**	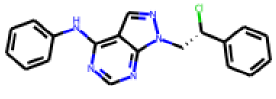	0.527360	Cc1ccc(C(=O)Nc2ccon2)cc1Nc1ncnc2c1cnn2C1cccccc1	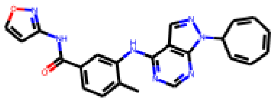	8.0	0.623362	2.525411	2.997881	5	0.770298
**1827**	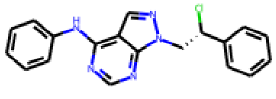	0.527360	Cc1ccc(C(=O)Nc2ccon2)cc1Nc1ncnc2c1cnn2C1cccccc1	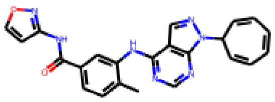	8.0	0.623362	2.525411	2.997881	5	0.770298

	src known mol	similarity	smiles	mol	Class	qed	logP	SAS	rings	kinase inhibition likeliness
**1828**	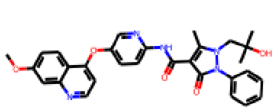	0.527194	COc1ccc2c(=O)cc(CNC(=O)c3c(C)n(C)n(-c4ccccc4)c3=O)nc3cccc2c3c1	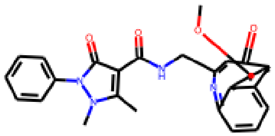	9.0	0.638163	3.995589	2.988471	5	0.774651
**1829**	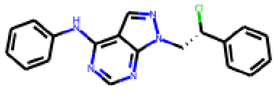	0.527148	Cc1ccc(C(=O)Nc2ccon2)cc1Nc1ncnc2c1cnn2C1cccccc1	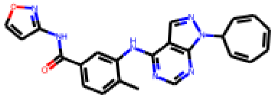	8.0	0.623362	2.525411	2.997881	5	0.770298
**1830**	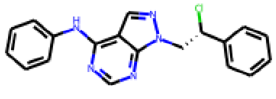	0.527148	Cc1ccc(C(=O)Nc2ccon2)cc1Nc1ncnc2c1cnn2C1cccccc1	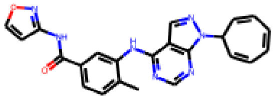	8.0	0.623362	2.525411	2.997881	5	0.770298
**1831**	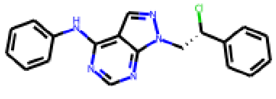	0.527148	Cc1ccc(C(=O)Nc2ccon2)cc1Nc1ncnc2c1cnn2C1cccccc1	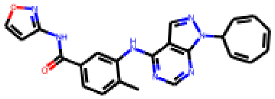	8.0	0.623362	2.525411	2.997881	5	0.770298

	src known mol	similarity	smiles	mol	Class	qed	logP	SAS	rings	kinase inhibition likeliness
**1832**	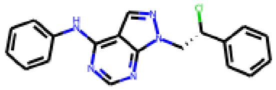	0.527148	Cc1ccc(C(=O)Nc2ccon2)cc1Nc1ncnc2c1cnn2C1cccccc1	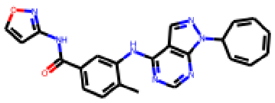	8.0	0.623362	2.525411	2.997881	5	0.770298
**1833**	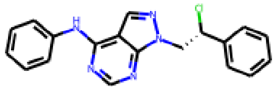	0.527148	Cc1ccc(C(=O)Nc2ccon2)cc1Nc1ncnc2c1cnn2C1cccccc1	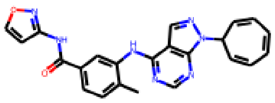	8.0	0.623362	2.525411	2.997881	5	0.770298
**1834**	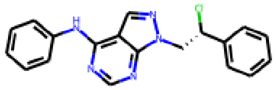	0.527148	Cc1ccc(C(=O)Nc2ccon2)cc1Nc1ncnc2c1cnn2C1cccccc1	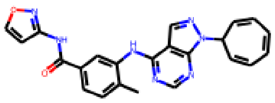	8.0	0.623362	2.525411	2.997881	5	0.770298
**1835**	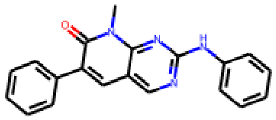	0.527120	Clc1cccc(Nc2ncc3cc(-c3c(Cl)cccc3Cl)c(=O)n(C)c3n2)c1	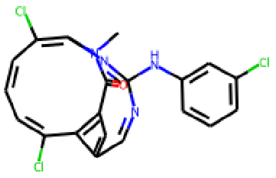	2.0	0.768132	3.608437	2.513170	5	0.784634

	src known mol	similarity	smiles	mol	Class	qed	logP	SAS	rings	kinase inhibition likeliness
**1836**	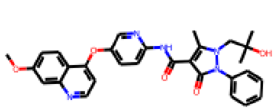	0.526485	COc1ccc2c(=O)cc(CNC(=O)c3c(C)n(C)n(-c4ccccc4)c3=O)nc3cccc2c3c1	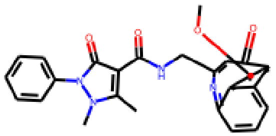	9.0	0.638163	3.995589	2.988471	5	0.774651
**1837**	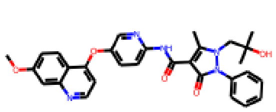	0.526485	COc1ccc2c(=O)cc(CNC(=O)c3c(C)n(C)n(-c4ccccc4)c3=O)nc3cccc2c3c1	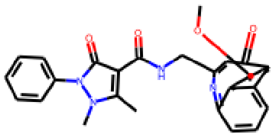	9.0	0.638163	3.995589	2.988471	5	0.774651
**1838**	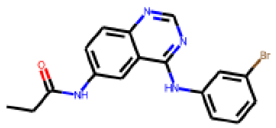	0.526360	O=C(CNC(=O)Oc1ccccc1)Nc1ccc2ncnc(Nc3cccc(Cl)c3)c2c1	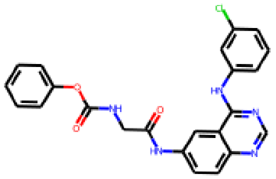	3.0	0.638163	3.995589	2.988471	4	0.793889
**1839**	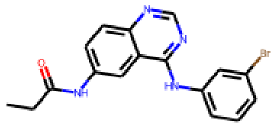	0.526012	O=C(CNC(=O)C1cccccc1)Nc1ccc2ncnc(Nc3cccc(Br)c3)c2c1	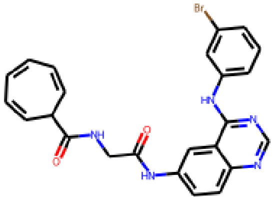	3.0	0.623362	2.525411	2.997881	4	0.772905

	src known mol	similarity	smiles	mol	Class	qed	logP	SAS	rings	kinase inhibition likeliness
**1840**	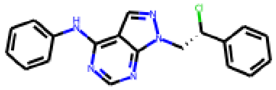	0.525632	Cc1ccc(C(=O)Nc2ccon2)cc1Nc1ncnc2c1cnn2C1cccccc1	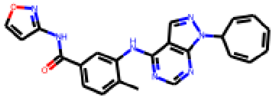	8.0	0.623362	2.525411	2.997881	5	0.770298
**1841**	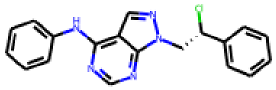	0.525632	Cc1ccc(C(=O)Nc2ccon2)cc1Nc1ncnc2c1cnn2C1cccccc1	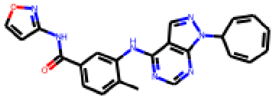	8.0	0.623362	2.525411	2.997881	5	0.770298
**1842**	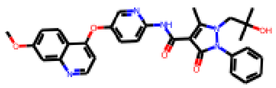	0.525567	COc1ccc2c(=O)cc(CNC(=O)c3c(C)n(C)n(-c4ccccc4)c3=O)nc3cccc2c3c1	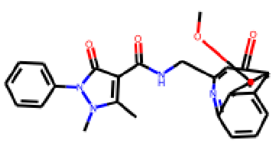	9.0	0.638163	3.995589	2.988471	5	0.774651
**1843**	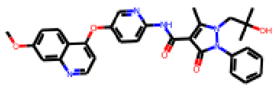	0.525567	COc1ccc2c(=O)cc(CNC(=O)c3c(C)n(C)n(-c4ccccc4)c3=O)nc3cccc2c3c1	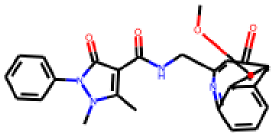	9.0	0.638163	3.995589	2.988471	5	0.774651

	src known mol	similarity	smiles	mol	Class	qed	logP	SAS	rings	kinase inhibition likeliness
**1844**	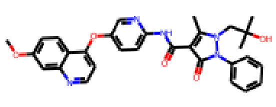	0.525478	COc1ccc2c(=O)cc(CNC(=O)c3c(C)n(C)n(-c4ccccc4)c3=O)nc3cccc2c3c1	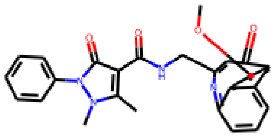	9.0	0.638163	3.995589	2.988471	5	0.774651
**1845**	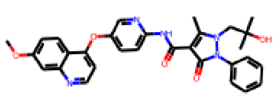	0.525263	COc1ccc2c(=O)cc(CNC(=O)c3c(C)n(C)n(-c4ccccc4)c3=O)nc3cccc2c3c1	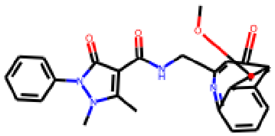	9.0	0.638163	3.995589	2.988471	5	0.774651
**1846**	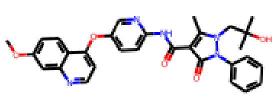	0.525253	COc1ccc2c(=O)cc(CNC(=O)c3c(C)n(C)n(-c4ccccc4)c3=O)nc3cccc3c2n1	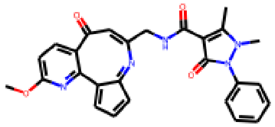	9.0	0.768132	3.608437	2.513170	5	0.823048
**1847**	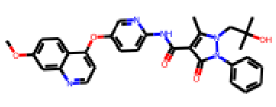	0.525253	COc1ccc2c(=O)cc(CNC(=O)c3c(C)n(C)n(-c4ccccc4)c3=O)nc3cccc3c2n1	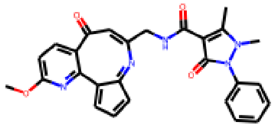	9.0	0.768132	3.608437	2.513170	5	0.823048

	src known mol	similarity	smiles	mol	Class	qed	logP	SAS	rings	kinase inhibition likeliness
**1848**	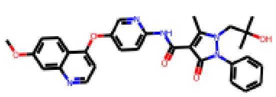	0.525253	COc1ccc2c(=O)cc(CNC(=O)c3c(C)n(C)n(-c4ccccc4)c3=O)nc3cccc3c2n1	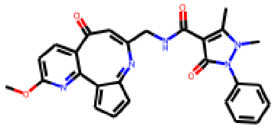	9.0	0.768132	3.608437	2.513170	5	0.823048
**1849**	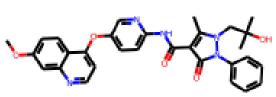	0.525253	COc1ccc2c(=O)cc(CNC(=O)c3c(C)n(C)n(-c4ccccc4)c3=O)nc3cccc3c2n1	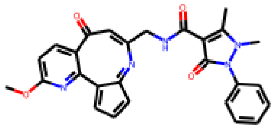	9.0	0.768132	3.608437	2.513170	5	0.823048
**1850**	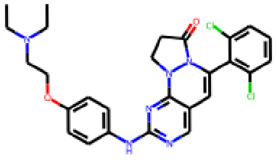	0.524938	COc1cc(Nc2ncc(C)c(-c3ccc4c(n3)NC(=O)C(C)(C)C4)n2)cc(OC)c1OC	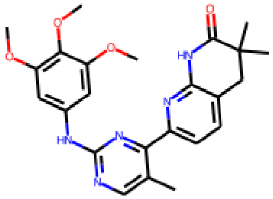	4.0	0.768132	3.608437	2.513170	4	0.757734
**1851**	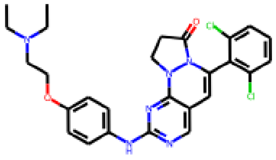	0.524938	COc1cc(Nc2ncc(C)c(-c3ccc4c(n3)NC(=O)C(C)(C)C4)n2)cc(OC)c1OC	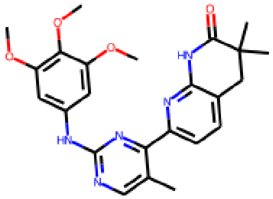	4.0	0.768132	3.608437	2.513170	4	0.757734

	src known mol	similarity	smiles	mol	Class	qed	logP	SAS	rings	kinase inhibition likeliness
**1852**	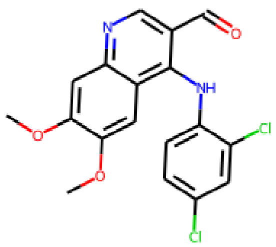	0.524874	COc1cc2ncc(C(=O)O)c(Nc3cccc(Cl)c3Cl)c2cc1OC1C(C)C1	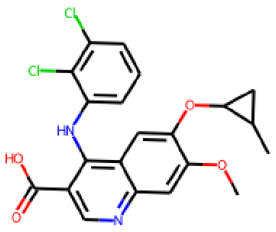	2.0	0.623362	2.525411	2.997881	4	0.774857
**1853**	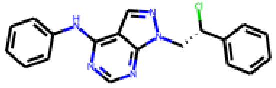	0.524870	Cc1ccc(C(=O)Nc2ccon2)cc1Nc1ncnc2c1cnn2C1cccccc1	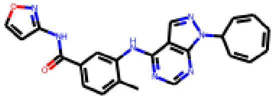	8.0	0.623362	2.525411	2.997881	5	0.770298
**1854**	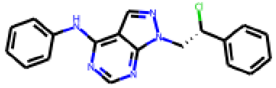	0.524870	Cc1ccc(C(=O)Nc2ccon2)cc1Nc1ncnc2c1cnn2C1cccccc1	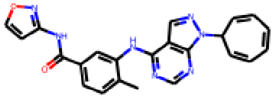	8.0	0.623362	2.525411	2.997881	5	0.770298
**1855**	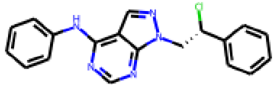	0.524870	Cc1ccc(C(=O)Nc2ccon2)cc1Nc1ncnc2c1cnn2C1cccccc1	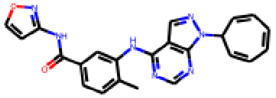	8.0	0.623362	2.525411	2.997881	5	0.770298

	src known mol	similarity	smiles	mol	Class	qed	logP	SAS	rings	kinase inhibition likeliness
**1856**	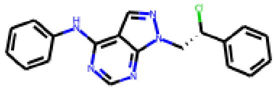	0.524870	Cc1ccc(C(=O)Nc2ccon2)cc1Nc1ncnc2c1cnn2C1cccccc1	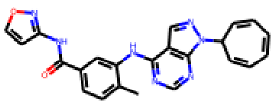	8.0	0.623362	2.525411	2.997881	5	0.770298
**1857**	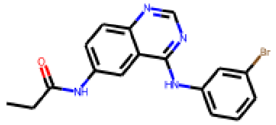	0.524735	O=C(CNC(=O)Oc1ccccc1)Nc1ccc2ncnc(Nc3cccc(Cl)c3)c2c1	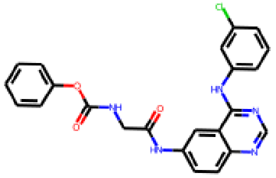	3.0	0.638163	3.995589	2.988471	4	0.793889
**1858**	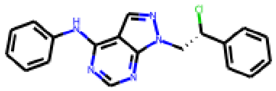	0.524687	Cc1ccc(C(=O)Nc2ccon2)cc1Nc1ncnc2c1cnn2C1cccccc1	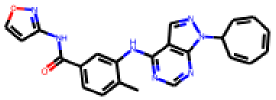	8.0	0.623362	2.525411	2.997881	5	0.770298
**1859**	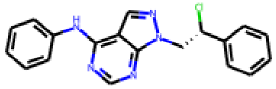	0.524687	Cc1ccc(C(=O)Nc2ccon2)cc1Nc1ncnc2c1cnn2C1cccccc1	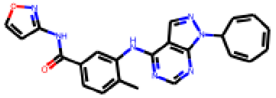	8.0	0.623362	2.525411	2.997881	5	0.770298

	src known mol	similarity	smiles	mol	Class	qed	logP	SAS	rings	kinase inhibition likeliness
**1860**	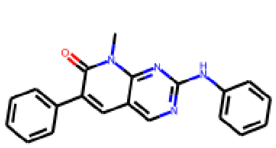	0.524454	Clc1cccc(Nc2ncc3cc(-c3c(Cl)cccc3Cl)c(=O)n(C)c3n2)c1	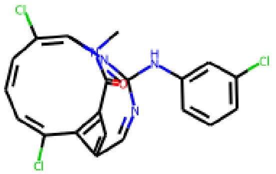	2.0	0.768132	3.608437	2.513170	5	0.784634
**1861**	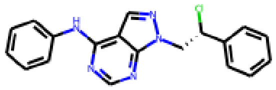	0.524364	Cc1ccc(C(=O)Nc2ccon2)cc1Nc1ncnc2c1cnn2C1cccccc1	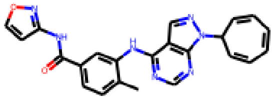	8.0	0.623362	2.525411	2.997881	5	0.770298
**1862**	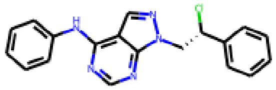	0.524364	Cc1ccc(C(=O)Nc2ccon2)cc1Nc1ncnc2c1cnn2C1cccccc1	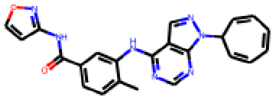	8.0	0.623362	2.525411	2.997881	5	0.770298
**1863**	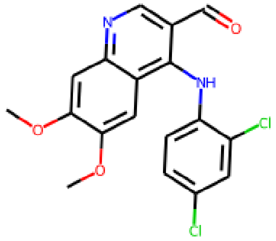	0.524306	COc1cc2ncc(C(=O)O)c(Nc3cccc(Cl)c3Cl)c2cc1OC1C(C)C1	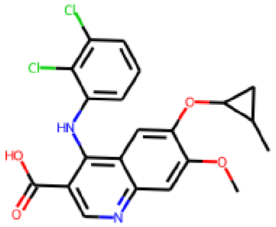	2.0	0.623362	2.525411	2.997881	4	0.774857

	src known mol	similarity	smiles	mol	Class	qed	logP	SAS	rings	kinase inhibition likeliness
**1864**	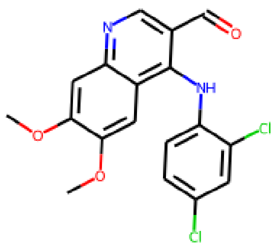	0.524306	COc1cc2ncc(C(=O)O)c(Nc3cccc(Cl)c3Cl)c2cc1OC1C(C)C1	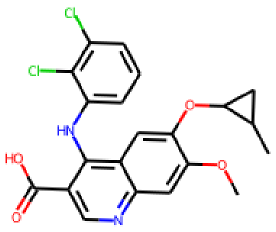	2.0	0.623362	2.525411	2.997881	4	0.774857
**1865**	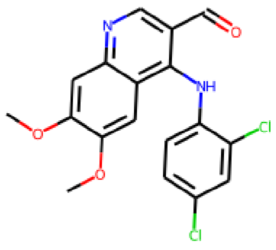	0.524306	COc1cc2ncc(C(=O)O)c(Nc3cccc(Cl)c3Cl)c2cc1OC1C(C)C1	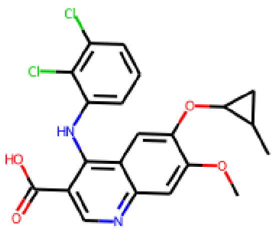	2.0	0.623362	2.525411	2.997881	4	0.774857
**1866**	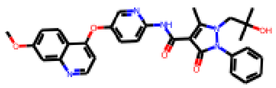	0.524262	COc1ccc2c(=O)cc(CNC(=O)c3c(C)n(C)n(-c4ccccc4)c3=O)nc3cccc2c3c1	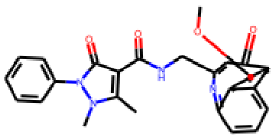	9.0	0.638163	3.995589	2.988471	5	0.774651
**1867**	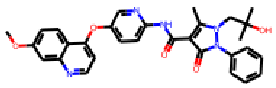	0.524262	COc1ccc2c(=O)cc(CNC(=O)c3c(C)n(C)n(-c4ccccc4)c3=O)nc3cccc2c3c1	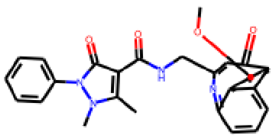	9.0	0.638163	3.995589	2.988471	5	0.774651

	src known mol	similarity	smiles	mol	Class	qed	logP	SAS	rings	kinase inhibition likeliness
**1868**	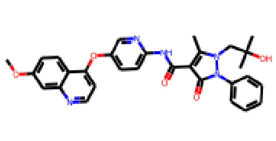	0.524241	COc1ccc2c(=O)cc(CNC(=O)c3c(C)n(C)n(-c4ccccc4)c3=O)nc3cccc2c3c1	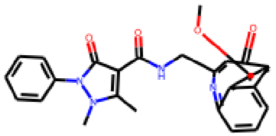	9.0	0.638163	3.995589	2.988471	5	0.774651
**1869**	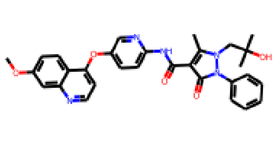	0.524202	COc1ccc2c(=O)cc(CNC(=O)c3c(C)n(C)n(-c4ccccc4)c3=O)nc3cccc3c2n1	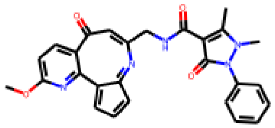	9.0	0.768132	3.608437	2.513170	5	0.823048
**1870**	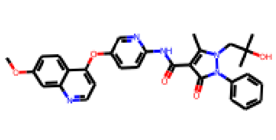	0.524142	COc1ccc2c(=O)cc(CNC(=O)c3c(C)n(C)n(-c4ccccc4)c3=O)nc3cccc2c3c1	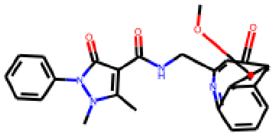	9.0	0.638163	3.995589	2.988471	5	0.774651
**1871**	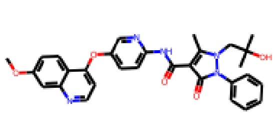	0.524142	COc1ccc2c(=O)cc(CNC(=O)c3c(C)n(C)n(-c4ccccc4)c3=O)nc3cccc2c3c1	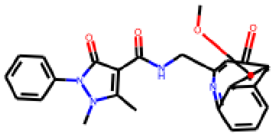	9.0	0.638163	3.995589	2.988471	5	0.774651

	src known mol	similarity	smiles	mol	Class	qed	logP	SAS	rings	kinase inhibition likeliness
**1872**	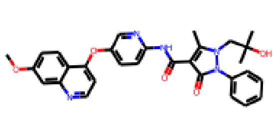	0.524142	COc1ccc2c(=O)cc(CNC(=O)c3c(C)n(C)n(-c4ccccc4)c3=O)nc3cccc2c3c1	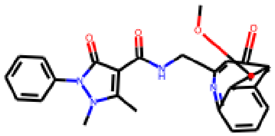	9.0	0.638163	3.995589	2.988471	5	0.774651
**1873**	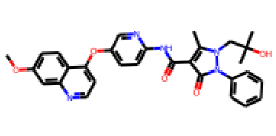	0.524142	COc1ccc2c(=O)cc(CNC(=O)c3c(C)n(C)n(-c4ccccc4)c3=O)nc3cccc2c3c1	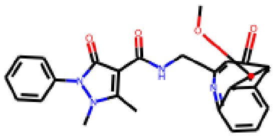	9.0	0.638163	3.995589	2.988471	5	0.774651
**1874**	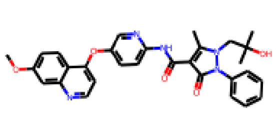	0.523989	COc1ccc2c(=O)cc(CNC(=O)c3c(C)n(C)n(-c4ccccc4)c3=O)nc3cccc2c3c1	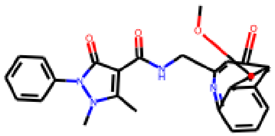	9.0	0.638163	3.995589	2.988471	5	0.774651
**1875**	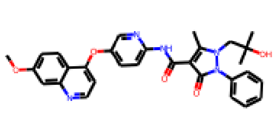	0.523989	COc1ccc2c(=O)cc(CNC(=O)c3c(C)n(C)n(-c4ccccc4)c3=O)nc3cccc2c3c1	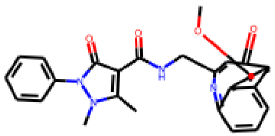	9.0	0.638163	3.995589	2.988471	5	0.774651

	src known mol	similarity	smiles	mol	Class	qed	logP	SAS	rings	kinase inhibition likeliness
**1876**	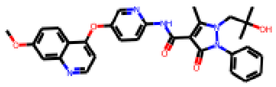	0.523989	COc1ccc2c(=O)cc(CNC(=O)c3c(C)n(C)n(-c4ccccc4)c3=O)nc3cccc2c3c1	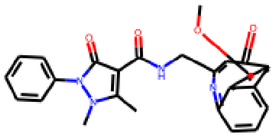	9.0	0.638163	3.995589	2.988471	5	0.774651
**1877**	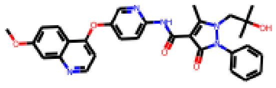	0.523960	COc1ccc2c(=O)cc(CNC(=O)c3c(C)n(C)n(-c4ccccc4)c3=O)nc3cccc2c3c1	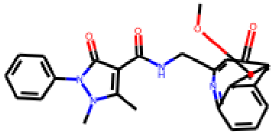	9.0	0.638163	3.995589	2.988471	5	0.774651
**1878**	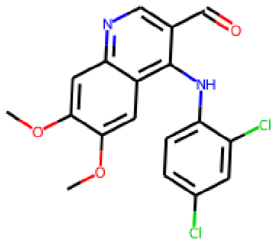	0.523842	COc1cc2ncc(C(=O)O)c(Nc3cccc(Cl)c3Cl)c2cc1OC1C(C)C1	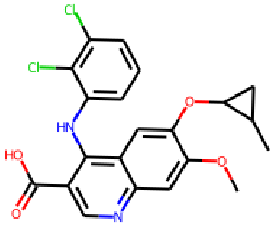	2.0	0.623362	2.525411	2.997881	4	0.774857
**1879**	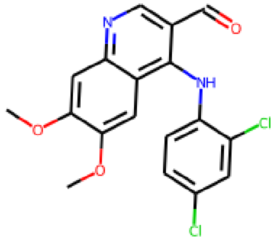	0.523842	COc1cc2ncc(C(=O)O)c(Nc3cccc(Cl)c3Cl)c2cc1OC1C(C)C1	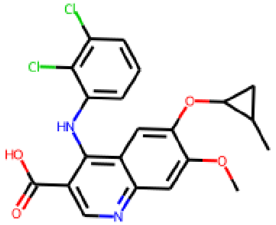	2.0	0.623362	2.525411	2.997881	4	0.774857

	src known mol	similarity	smiles	mol	Class	qed	logP	SAS	rings	kinase inhibition likeliness
**1880**	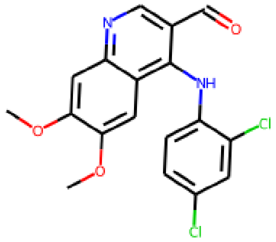	0.523842	COc1cc2ncc(C(=O)O)c(Nc3cccc(Cl)c3Cl)c2cc1OC1C(C)C1	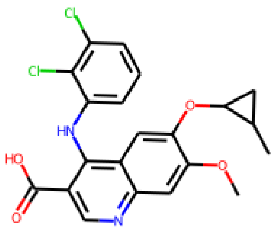	2.0	0.623362	2.525411	2.997881	4	0.774857
**1881**	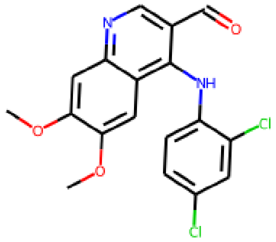	0.523842	COc1cc2ncc(C(=O)O)c(Nc3cccc(Cl)c3Cl)c2cc1OC1C(C)C1	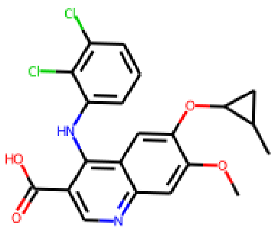	2.0	0.623362	2.525411	2.997881	4	0.774857
**1882**	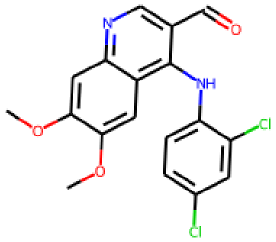	0.523842	COc1cc2ncc(C(=O)O)c(Nc3cccc(Cl)c3Cl)c2cc1OC1C(C)C1	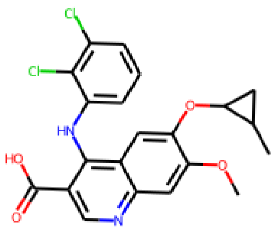	2.0	0.623362	2.525411	2.997881	4	0.774857
**1883**	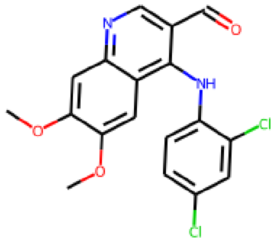	0.523842	COc1cc2ncc(C(=O)O)c(Nc3cccc(Cl)c3Cl)c2cc1OC1C(C)C1	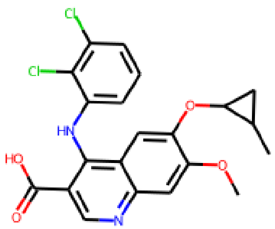	2.0	0.623362	2.525411	2.997881	4	0.774857

	src known mol	similarity	smiles	mol	Class	qed	logP	SAS	rings	kinase inhibition likeliness
**1884**	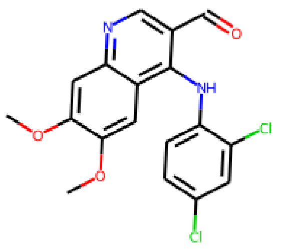	0.523842	COc1cc2ncc(C(=O)O)c(Nc3cccc(Cl)c3Cl)c2cc1OC1C(C)C1	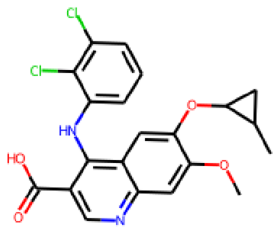	2.0	0.623362	2.525411	2.997881	4	0.774857
**1885**	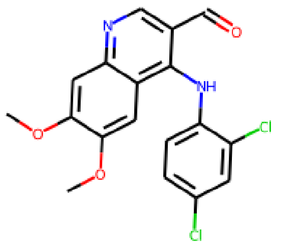	0.523842	COc1cc2ncc(C(=O)O)c(Nc3cccc(Cl)c3Cl)c2cc1OC1C(C)C1	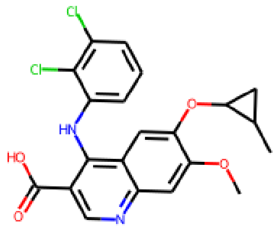	2.0	0.623362	2.525411	2.997881	4	0.774857
**1886**	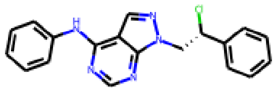	0.523739	Cc1ccc(C(=O)Nc2ccon2)cc1Nc1ncnc2c1cnn2C1cccccc1	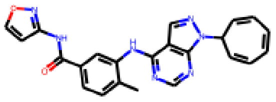	8.0	0.623362	2.525411	2.997881	5	0.770298
**1887**	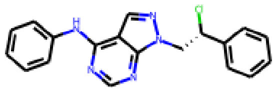	0.523739	Cc1ccc(C(=O)Nc2ccon2)cc1Nc1ncnc2c1cnn2C1cccccc1	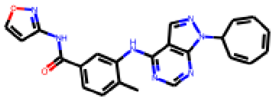	8.0	0.623362	2.525411	2.997881	5	0.770298

	src known mol	similarity	smiles	mol	Class	qed	logP	SAS	rings	kinase inhibition likeliness
**1888**	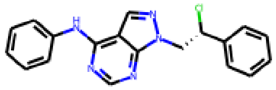	0.523739	Cc1ccc(C(=O)Nc2ccon2)cc1Nc1ncnc2c1cnn2C1cccccc1	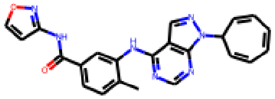	8.0	0.623362	2.525411	2.997881	5	0.770298
**1889**	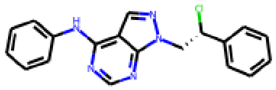	0.523739	Cc1ccc(C(=O)Nc2ccon2)cc1Nc1ncnc2c1cnn2C1cccccc1	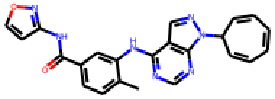	8.0	0.623362	2.525411	2.997881	5	0.770298
**1890**	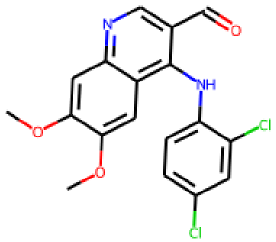	0.523711	COc1cc2ncc(C(=O)O)c(Nc3cccc(Cl)c3Cl)c2cc1OC1C(C)C1	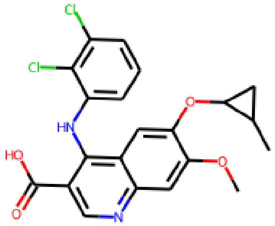	2.0	0.623362	2.525411	2.997881	4	0.774857
**1891**	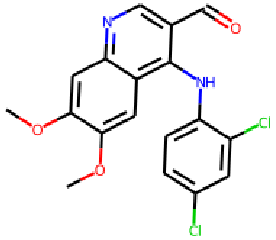	0.523711	COc1cc2ncc(C(=O)O)c(Nc3cccc(Cl)c3Cl)c2cc1OC1C(C)C1	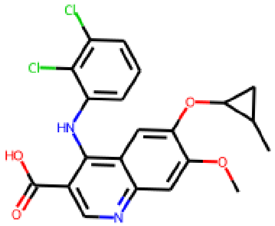	2.0	0.623362	2.525411	2.997881	4	0.774857

	src known mol	similarity	smiles	mol	Class	qed	logP	SAS	rings	kinase inhibition likeliness
**1892**	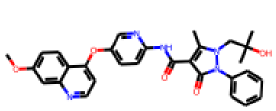	0.523607	COc1ccc2c(=O)cc(CNC(=O)c3c(C)n(C)n(-c4ccccc4)c3=O)nc3cccc2c3c1	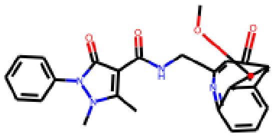	9.0	0.638163	3.995589	2.988471	5	0.774651
**1893**	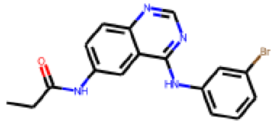	0.523577	O=C(CNC(=O)Oc1ccccc1)Nc1ccc2ncnc(Nc3cccc(Cl)c3)c2c1	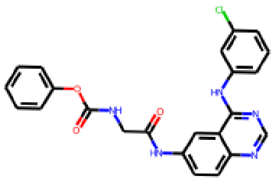	3.0	0.638163	3.995589	2.988471	4	0.793889
**1894**	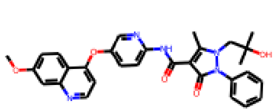	0.523557	COc1ccc2c(=O)cc(CNC(=O)c3c(C)n(C)n(-c4ccccc4)c3=O)nc3cccc2c3c1	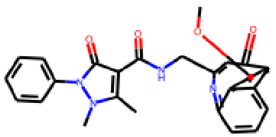	9.0	0.638163	3.995589	2.988471	5	0.774651
**1895**	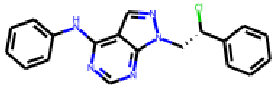	0.523460	Cc1ccc(C(=O)Nc2ccon2)cc1Nc1ncnc2c1cnn2C1cccccc1	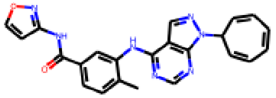	8.0	0.623362	2.525411	2.997881	5	0.770298

	src known mol	similarity	smiles	mol	Class	qed	logP	SAS	rings	kinase inhibition likeliness
**1896**	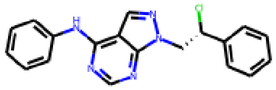	0.523460	Cc1ccc(C(=O)Nc2ccon2)cc1Nc1ncnc2c1cnn2C1cccccc1	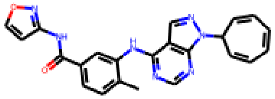	8.0	0.623362	2.525411	2.997881	5	0.770298
**1897**	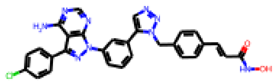	0.523407	Cc1ccc(C(=O)Nc2ccoc2)cc1Nc1ncnc2c1cnn2-c1ccccc1	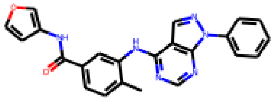	8.0	0.593231	3.250153	2.680275	5	0.871119
**1898**	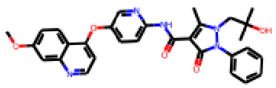	0.523231	COc1ccc2c(=O)cc(CNC(=O)c3c(C)n(C)n(-c4ccccc4)c3=O)nc3cccc2c3c1	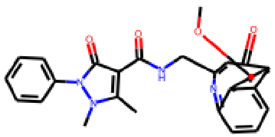	9.0	0.638163	3.995589	2.988471	5	0.774651
**1899**	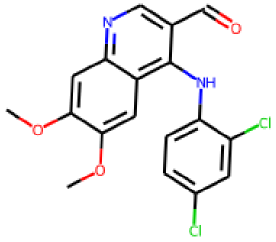	0.523151	COc1cc2ncc(C(=O)O)c(Nc3cccc(Cl)c3Cl)c2cc1OC1C(C)C1	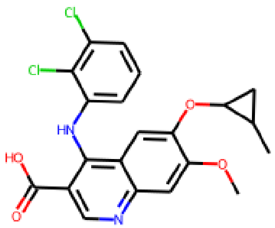	2.0	0.623362	2.525411	2.997881	4	0.774857

	src known mol	similarity	smiles	mol	Class	qed	logP	SAS	rings	kinase inhibition likeliness
**1900**	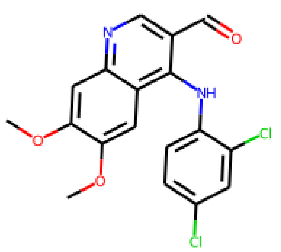	0.523151	COc1cc2ncc(C(=O)O)c(Nc3cccc(Cl)c3Cl)c2cc1OC1C(C)C1	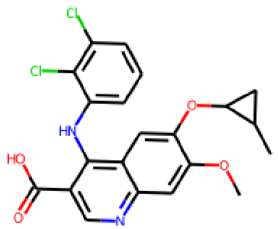	2.0	0.623362	2.525411	2.997881	4	0.774857
**1901**	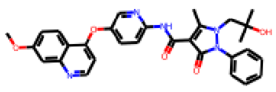	0.523101	COc1ccc2c(=O)cc(CNC(=O)c3c(C)n(C)n(-c4ccccc4)c3=O)nc3cccc2c3c1	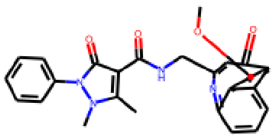	9.0	0.638163	3.995589	2.988471	5	0.774651
**1902**	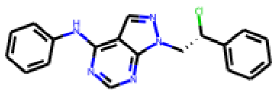	0.522976	Cc1ccc(C(=O)Nc2ccon2)cc1Nc1ncnc2c1cnn2C1cccccc1	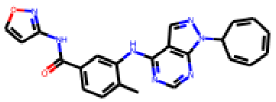	8.0	0.623362	2.525411	2.997881	5	0.770298
**1903**	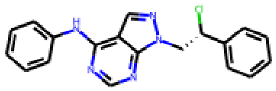	0.522976	Cc1ccc(C(=O)Nc2ccon2)cc1Nc1ncnc2c1cnn2C1cccccc1	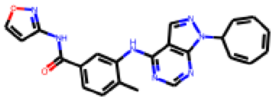	8.0	0.623362	2.525411	2.997881	5	0.770298

	src known mol	similarity	smiles	mol	Class	qed	logP	SAS	rings	kinase inhibition likeliness
**1904**	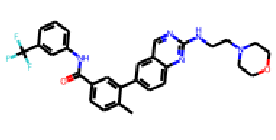	0.522839	Cc1ccc(C(=O)Nc2cccc(C(F)(C)F)c2C)cc1-c1ccc2nc(C)ccccc(F)nc2c1	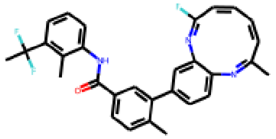	6.0	0.742375	4.384502	0.286234	4	0.779167
**1905**	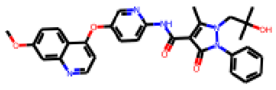	0.522824	COc1ccc2c(=O)cc(CNC(=O)c3c(C)n(C)n(-c4ccccc4)c3=O)nc3cccc2c3c1	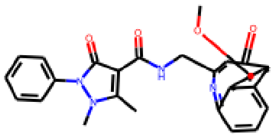	9.0	0.638163	3.995589	2.988471	5	0.774651
**1906**	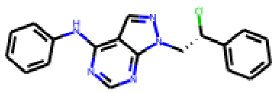	0.522509	Cc1ccc(C(=O)Nc2ccon2)cc1Nc1ncnc2c1cnn2C1cccccc1	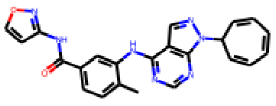	8.0	0.623362	2.525411	2.997881	5	0.770298
**1907**	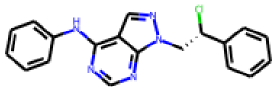	0.522509	Cc1ccc(C(=O)Nc2ccon2)cc1Nc1ncnc2c1cnn2C1cccccc1	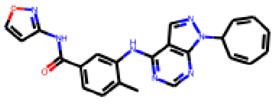	8.0	0.623362	2.525411	2.997881	5	0.770298

	src known mol	similarity	smiles	mol	Class	qed	logP	SAS	rings	kinase inhibition likeliness
**1908**	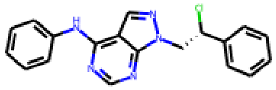	0.522509	Cc1ccc(C(=O)Nc2ccon2)cc1Nc1ncnc2c1cnn2C1cccccc1	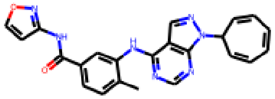	8.0	0.623362	2.525411	2.997881	5	0.770298
**1909**	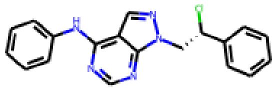	0.522509	Cc1ccc(C(=O)Nc2ccon2)cc1Nc1ncnc2c1cnn2C1cccccc1	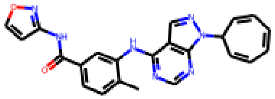	8.0	0.623362	2.525411	2.997881	5	0.770298
**1910**	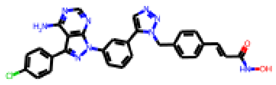	0.522398	Cc1ccc(C(=O)Nc2ccoc2)cc1Nc1ncnc2c1cnn2-c1ccccc1	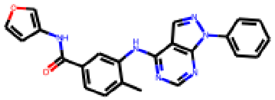	8.0	0.593231	3.250153	2.680275	5	0.871119
**1911**	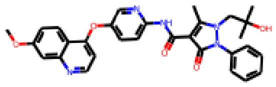	0.522293	COc1ccc2c(=O)cc(CNC(=O)c3c(C)n(C)n(-c4ccccc4)c3=O)nc3cccc2c3c1	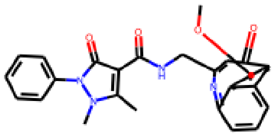	9.0	0.638163	3.995589	2.988471	5	0.774651

	src known mol	similarity	smiles	mol	Class	qed	logP	SAS	rings	kinase inhibition likeliness
**1912**	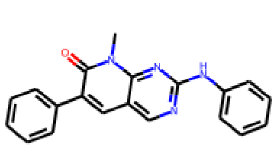	0.522293	Clc1cccc(Nc2ncc3cc(-c3c(Cl)cccc3Cl)c(=O)n(C)c3n2)c1	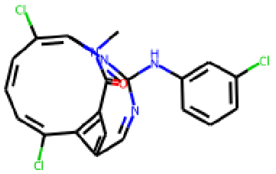	2.0	0.768132	3.608437	2.513170	5	0.784634
**1913**	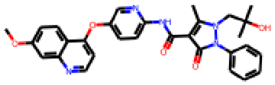	0.522039	COc1ccc2c(=O)cc(CNC(=O)c3c(C)n(C)n(-c4ccccc4)c3=O)nc3cccc2c3c1	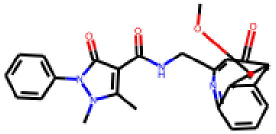	9.0	0.638163	3.995589	2.988471	5	0.774651
**1914**	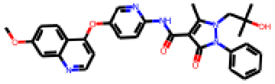	0.521969	COc1ccc2c(=O)cc(CNC(=O)c3c(C)n(C)n(-c4ccccc4)c3=O)nc3cccc2c3c1	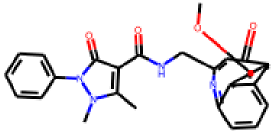	9.0	0.638163	3.995589	2.988471	5	0.774651
**1915**	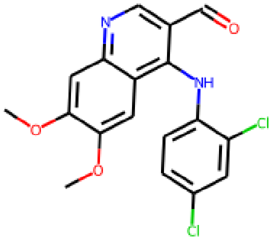	0.521951	COc1cc2ncc(C(=O)O)c(Nc3cccc(Cl)c3Cl)c2cc1OC1C(C)C1	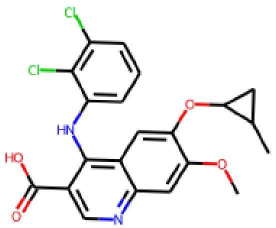	2.0	0.623362	2.525411	2.997881	4	0.774857

	src known mol	similarity	smiles	mol	Class	qed	logP	SAS	rings	kinase inhibition likeliness
**1916**	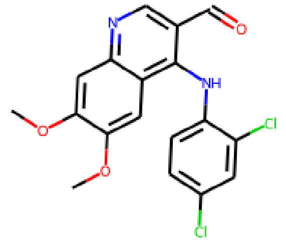	0.521951	COc1cc2ncc(C(=O)O)c(Nc3cccc(Cl)c3Cl)c2cc1OC1C(C)C1	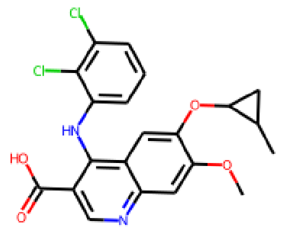	2.0	0.623362	2.525411	2.997881	4	0.774857
**1917**	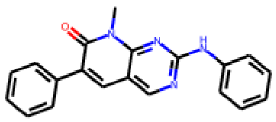	0.521930	Clc1cccc(Nc2ncc3cc(-c3c(Cl)cccc3Cl)c(=O)n(C)c3n2)c1	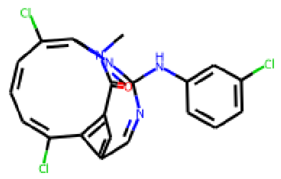	2.0	0.768132	3.608437	2.513170	5	0.784634
**1918**	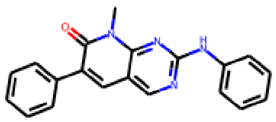	0.521930	Clc1cccc(Nc2ncc3cc(-c3c(Cl)cccc3Cl)c(=O)n(C)c3n2)c1	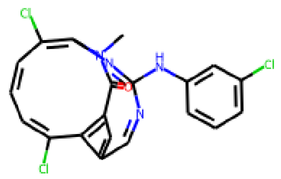	2.0	0.768132	3.608437	2.513170	5	0.784634
**1919**	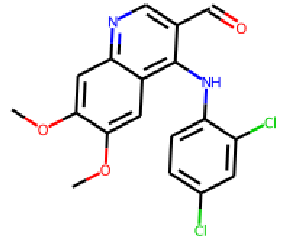	0.521798	COc1cc2ncc(C(=O)O)c(Nc3cccc(Cl)c3Cl)c2cc1OC1C(C)C1	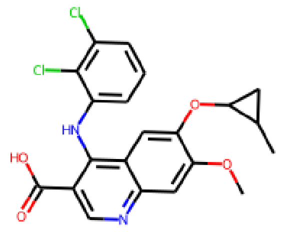	2.0	0.623362	2.525411	2.997881	4	0.774857

	src known mol	similarity	smiles	mol	Class	qed	logP	SAS	rings	kinase inhibition likeliness
**1920**	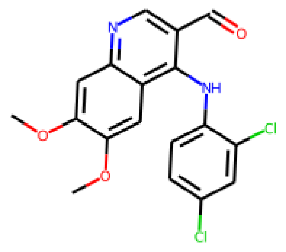	0.521798	COc1cc2ncc(C(=O)O)c(Nc3cccc(Cl)c3Cl)c2cc1OC1C(C)C1	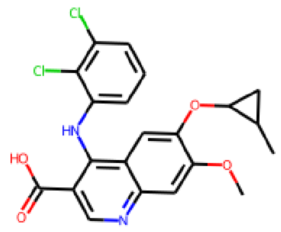	2.0	0.623362	2.525411	2.997881	4	0.774857
**1921**	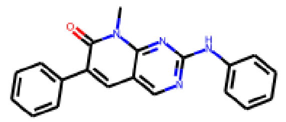	0.521739	Clc1cccc(Nc2ncc3cc(-c3c(Cl)cccc3Cl)c(=O)n(C)c3n2)c1	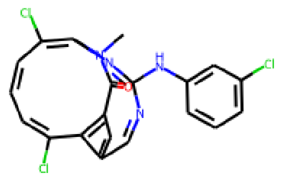	2.0	0.768132	3.608437	2.513170	5	0.784634
**1922**	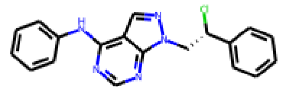	0.521707	Cc1ccc(C(=O)Nc2ccon2)cc1Nc1ncnc2c1cnn2C1cccccc1	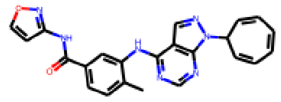	8.0	0.623362	2.525411	2.997881	5	0.770298
**1923**	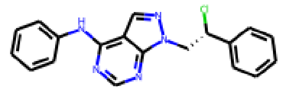	0.521707	Cc1ccc(C(=O)Nc2ccon2)cc1Nc1ncnc2c1cnn2C1cccccc1	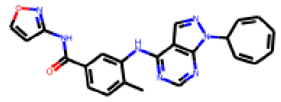	8.0	0.623362	2.525411	2.997881	5	0.770298

	src known mol	similarity	smiles	mol	Class	qed	logP	SAS	rings	kinase inhibition likeliness
**1924**	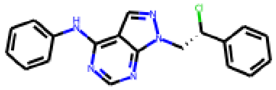	0.521707	Cc1ccc(C(=O)Nc2ccon2)cc1Nc1ncnc2c1cnn2C1cccccc1	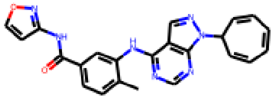	8.0	0.623362	2.525411	2.997881	5	0.770298
**1925**	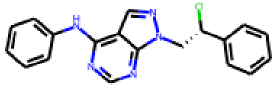	0.521707	Cc1ccc(C(=O)Nc2ccon2)cc1Nc1ncnc2c1cnn2C1cccccc1	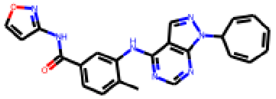	8.0	0.623362	2.525411	2.997881	5	0.770298
**1926**	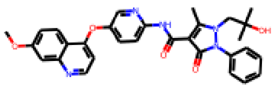	0.521436	COc1ccc2c(=O)cc(CNC(=O)c3c(C)n(C)n(-c4ccccc4)c3=O)nc3cccc2c3c1	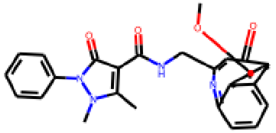	9.0	0.638163	3.995589	2.988471	5	0.774651
**1927**	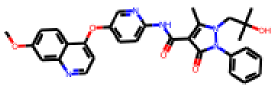	0.521413	COc1ccc2c(=O)cc(CNC(=O)c3c(C)n(C)n(-c4ccccc4)c3=O)nc3cccc2c3c1	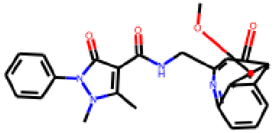	9.0	0.638163	3.995589	2.988471	5	0.774651

	src known mol	similarity	smiles	mol	Class	qed	logP	SAS	rings	kinase inhibition likeliness
**1928**	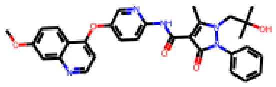	0.521368	COc1ccc2c(=O)cc(CNC(=O)c3c(C)n(C)n(-c4ccccc4)c3=O)nc3cccc2c3c1	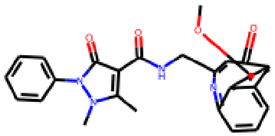	9.0	0.638163	3.995589	2.988471	5	0.774651
**1929**	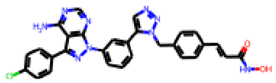	0.521305	Cc1ccc(C(=O)Nc2ccoc2)cc1Nc1ncnc2c1cnn2-c1ccccc1	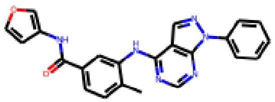	8.0	0.593231	3.250153	2.680275	5	0.871119
**1930**	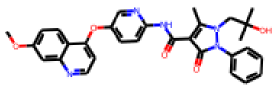	0.521231	COc1ccc2c(=O)cc(CNC(=O)c3c(C)n(C)n(-c4ccccc4)c3=O)nc3cccc2c3c1	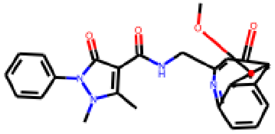	9.0	0.638163	3.995589	2.988471	5	0.774651
**1931**	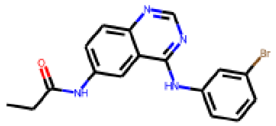	0.521226	OCC(C(N1CC)CC1)Nc1ccc2ncnc(Nc3cccc(Br)c3)c2c1	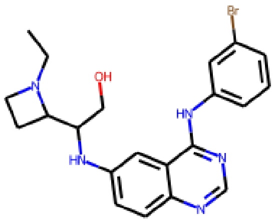	3.0	0.768132	3.608437	2.513170	4	0.789015

	src known mol	similarity	smiles	mol	Class	qed	logP	SAS	rings	kinase inhibition likeliness
**1932**	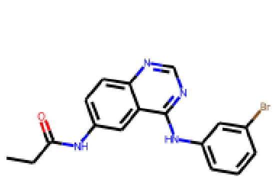	0.521226	OCC(C(N1CC)CC1)Nc1ccc2ncnc(Nc3cccc(Br)c3)c2c1	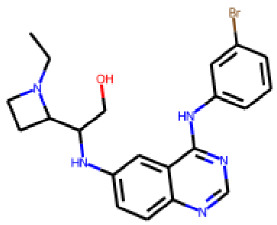	3.0	0.768132	3.608437	2.513170	4	0.789015
**1933**	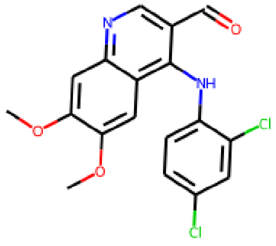	0.521204	COc1cc2ncc(C(=O)O)c(Nc3cccc(Cl)c3Cl)c2cc1OC1C(C)C1	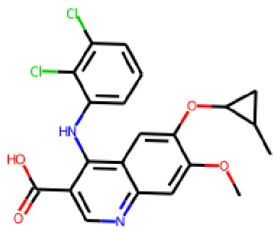	2.0	0.623362	2.525411	2.997881	4	0.774857
**1934**	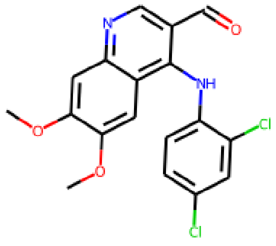	0.521204	COc1cc2ncc(C(=O)O)c(Nc3cccc(Cl)c3Cl)c2cc1OC1C(C)C1	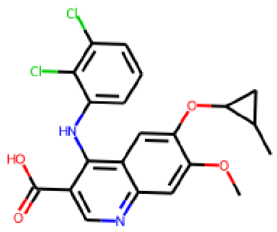	2.0	0.623362	2.525411	2.997881	4	0.774857
**1935**	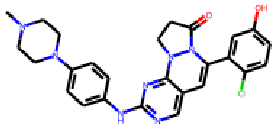	0.521198	O/C(Nc1ccc(Oc2ccccc2)cc1)Oc1ccc2(Cc1)NC(=O)/C(=O)c1ccc(O)n12	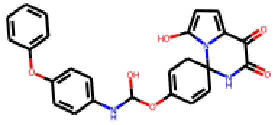	4.0	0.520676	2.336694	2.899742	5	0.820778

	src known mol	similarity	smiles	mol	Class	qed	logP	SAS	rings	kinase inhibition likeliness
**1936**	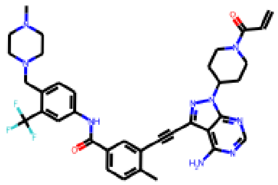	0.520927	Cc1ccc(C(=O)Nc2cccc(C(F)(C)F)c2C)cc1-c1ccc2nc(=O)ccc(C)cn2n1	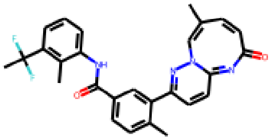	6.0	0.762994	3.476405	2.421118	4	0.764424
**1937**	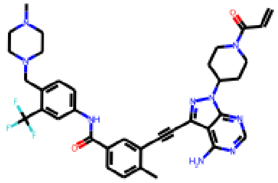	0.520927	Cc1ccc(C(=O)Nc2cccc(C(F)(C)F)c2C)cc1-c1ccc2nc(=O)ccc(C)cn2n1	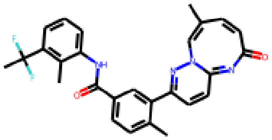	6.0	0.762994	3.476405	2.421118	4	0.764424
**1938**	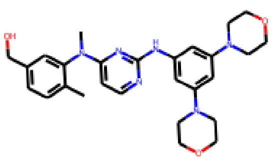	0.520801	NC(=O)c1ccccc1Nc1nc(Nc2ccc(N3CCCC3=O)cc2)ncc1Cl	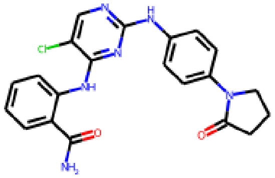	7.0	0.768132	3.608437	2.513170	4	0.773984
**1939**	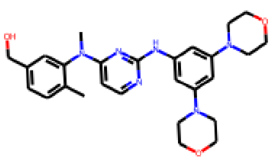	0.520801	NC(=O)c1ccccc1Nc1nc(Nc2ccc(N3CCCC3=O)cc2)ncc1Cl	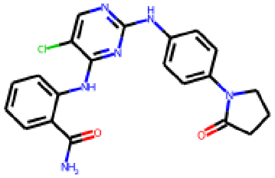	7.0	0.768132	3.608437	2.513170	4	0.773984

	src known mol	similarity	smiles	mol	Class	qed	logP	SAS	rings	kinase inhibition likeliness
**1940**	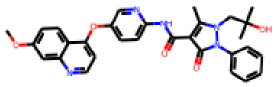	0.520577	COc1ccc2c(=O)cc(CNC(=O)c3c(C)n(C)n(-c4ccccc4)c3=O)nc3cccc2c3c1	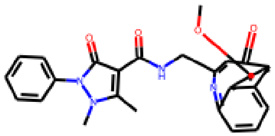	9.0	0.638163	3.995589	2.988471	5	0.774651
**1941**	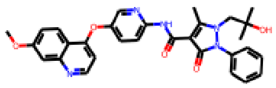	0.520577	COc1ccc2c(=O)cc(CNC(=O)c3c(C)n(C)n(-c4ccccc4)c3=O)nc3cccc2c3c1	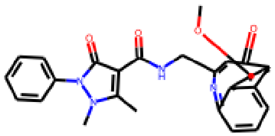	9.0	0.638163	3.995589	2.988471	5	0.774651
**1942**	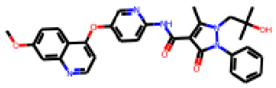	0.520563	COc1ccc2c(=O)cc(CNC(=O)c3c(C)n(C)n(-c4ccccc4)c3=O)nc3cccc2c3c1	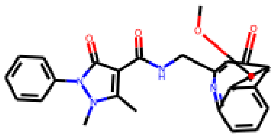	9.0	0.638163	3.995589	2.988471	5	0.774651
**1943**	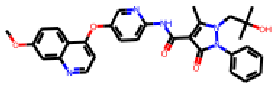	0.520386	COc1ccc2c(=O)cc(CNC(=O)c3c(C)n(C)n(-c4ccccc4)c3=O)nc3cccc2c3c1	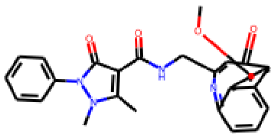	9.0	0.638163	3.995589	2.988471	5	0.774651

	src known mol	similarity	smiles	mol	Class	qed	logP	SAS	rings	kinase inhibition likeliness
**1944**	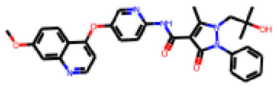	0.520386	COc1ccc2c(=O)cc(CNC(=O)c3c(C)n(C)n(-c4ccccc4)c3=O)nc3cccc2c3c1	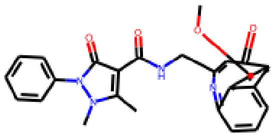	9.0	0.638163	3.995589	2.988471	5	0.774651
**1945**	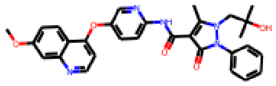	0.520386	COc1ccc2c(=O)cc(CNC(=O)c3c(C)n(C)n(-c4ccccc4)c3=O)nc3cccc2c3c1	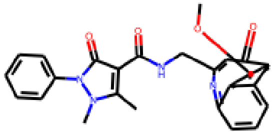	9.0	0.638163	3.995589	2.988471	5	0.774651
**1946**	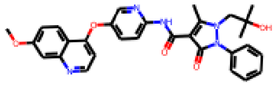	0.520386	COc1ccc2c(=O)cc(CNC(=O)c3c(C)n(C)n(-c4ccccc4)c3=O)nc3cccc2c3c1	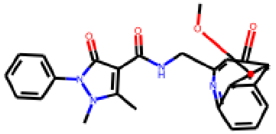	9.0	0.638163	3.995589	2.988471	5	0.774651
**1947**	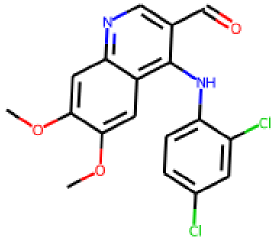	0.520139	COc1cc2ncc(C(=O)O)c(Nc3cccc(Cl)c3Cl)c2cc1OC1C(C)C1	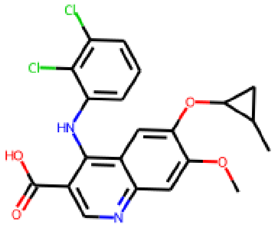	2.0	0.623362	2.525411	2.997881	4	0.774857

	src known mol	similarity	smiles	mol	Class	qed	logP	SAS	rings	kinase inhibition likeliness
**1948**	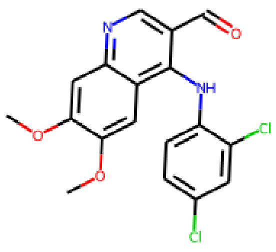	0.520139	COc1cc2ncc(C(=O)O)c(Nc3cccc(Cl)c3Cl)c2cc1OC1C(C)C1	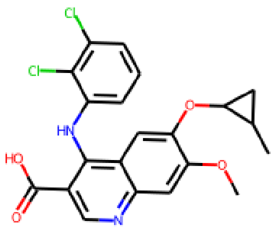	2.0	0.623362	2.525411	2.997881	4	0.774857
**1949**	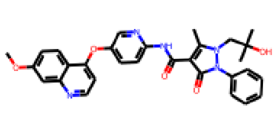	0.520086	COc1ccc2c(=O)cc(CNC(=O)c3c(C)n(C)n(-c4ccccc4)c3=O)nc3cccc2c3c1	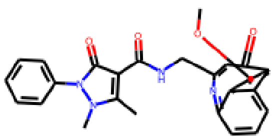	9.0	0.638163	3.995589	2.988471	5	0.774651
**1950**	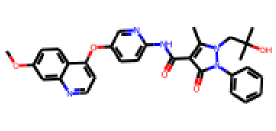	0.520021	COc1ccc2c(=O)cc(CNC(=O)c3c(C)n(C)n(-c4ccccc4)c3=O)nc3cccc2c3c1	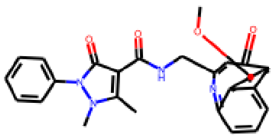	9.0	0.638163	3.995589	2.988471	5	0.774651
**1951**	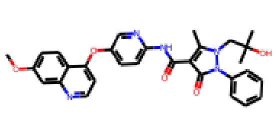	0.519935	COc1ccc2c(=O)cc(CNC(=O)c3c(C)n(C)n(-c4ccccc4)c3=O)nc3cccc2c3c1	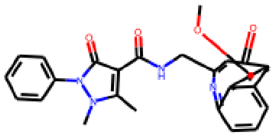	9.0	0.638163	3.995589	2.988471	5	0.774651

	src known mol	similarity	smiles	mol	Class	qed	logP	SAS	rings	kinase inhibition likeliness
**1952**	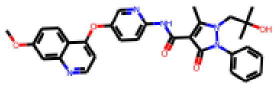	0.519935	COc1ccc2c(=O)cc(CNC(=O)c3c(C)n(C)n(-c4ccccc4)c3=O)nc3cccc2c3c1	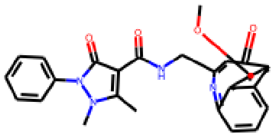	9.0	0.638163	3.995589	2.988471	5	0.774651
**1953**	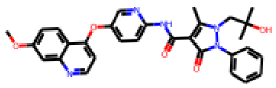	0.519829	COc1ccc2c(=O)cc(CNC(=O)c3c(C)n(C)n(-c4ccccc4)c3=O)nc3cccc2c3c1	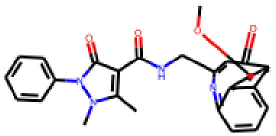	9.0	0.638163	3.995589	2.988471	5	0.774651
**1954**	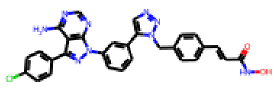	0.519706	Cc1ccc(C(=O)Nc2ccoc2)cc1Nc1ncnc2c1cnn2-c1ccccc1	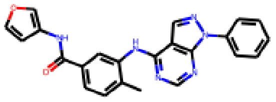	8.0	0.593231	3.250153	2.680275	5	0.871119
**1955**	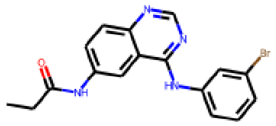	0.519534	O=C(CNC(=O)Oc1ccccc1)Nc1ccc2ncnc(Nc3cccc(Cl)c3)c2c1	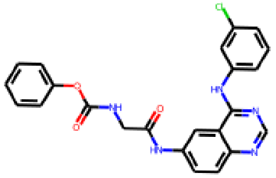	3.0	0.638163	3.995589	2.988471	4	0.793889

	src known mol	similarity	smiles	mol	Class	qed	logP	SAS	rings	kinase inhibition likeliness
**1956**	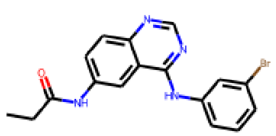	0.519534	O=C(CNC(=O)Oc1ccccc1)Nc1ccc2ncnc(Nc3cccc(Cl)c3)c2c1	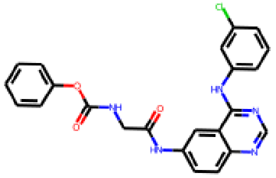	3.0	0.638163	3.995589	2.988471	4	0.793889
**1957**	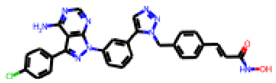	0.519395	Cc1ccc(C(=O)Nc2ccoc2)cc1Nc1ncnc2c1cnn2-c1ccccc1	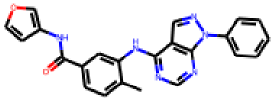	8.0	0.593231	3.250153	2.680275	5	0.871119
**1958**	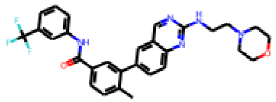	0.519313	Cc1ccc(C(=O)Oc2cccc(C(F)(F)F)c2)cc1-c1ccc2nc(N)ncc2c1	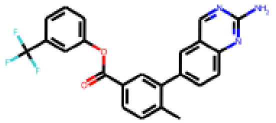	6.0	0.638163	3.995589	2.988471	4	0.789898
**1959**	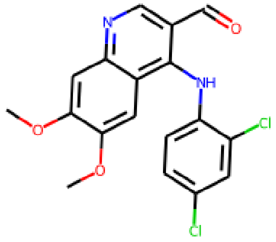	0.519298	COc1cc2ncc(C(=O)O)c(Nc3cccc(Cl)c3Cl)c2cc1OC1C(C)C1	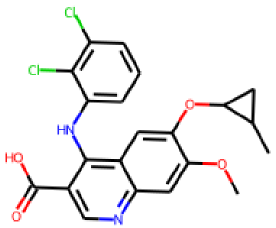	2.0	0.623362	2.525411	2.997881	4	0.774857

	src known mol	similarity	smiles	mol	Class	qed	logP	SAS	rings	kinase inhibition likeliness
**1960**	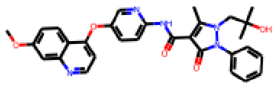	0.518878	COc1ccc2c(=O)cc(CNC(=O)c3c(C)n(C)n(-c4ccccc4)c3=O)nc3cccc2c3c1	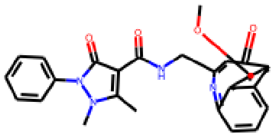	9.0	0.638163	3.995589	2.988471	5	0.774651
**1961**	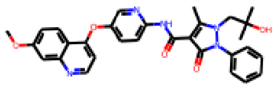	0.518878	COc1ccc2c(=O)cc(CNC(=O)c3c(C)n(C)n(-c4ccccc4)c3=O)nc3cccc2c3c1	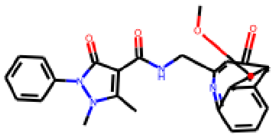	9.0	0.638163	3.995589	2.988471	5	0.774651
**1962**	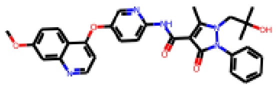	0.518852	COc1ccc2c(=O)cc(CNC(=O)c3c(C)n(C)n(-c4ccccc4)c3=O)nc3cccc3c2n1	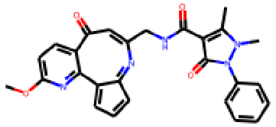	9.0	0.768132	3.608437	2.513170	5	0.823048
**1963**	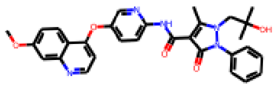	0.518852	COc1ccc2c(=O)cc(CNC(=O)c3c(C)n(C)n(-c4ccccc4)c3=O)nc3cccc3c2n1	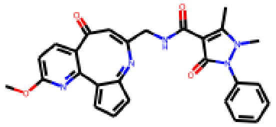	9.0	0.768132	3.608437	2.513170	5	0.823048

	src known mol	similarity	smiles	mol	Class	qed	logP	SAS	rings	kinase inhibition likeliness
**1964**	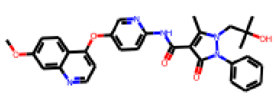	0.518757	COc1ccc2c(=O)cc(CNC(=O)c3c(C)n(C)n(-c4ccccc4)c3=O)nc3cccc2c3c1	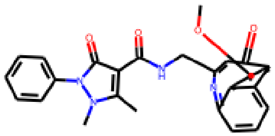	9.0	0.638163	3.995589	2.988471	5	0.774651
**1965**	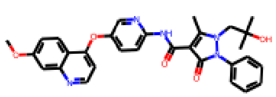	0.518637	COc1ccc2c(=O)cc(CNC(=O)c3c(C)n(C)n(-c4ccccc4)c3=O)nc3cccc2c3c1	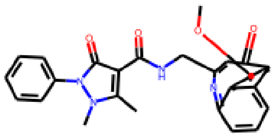	9.0	0.638163	3.995589	2.988471	5	0.774651
**1966**	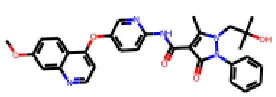	0.518637	COc1ccc2c(=O)cc(CNC(=O)c3c(C)n(C)n(-c4ccccc4)c3=O)nc3cccc2c3c1	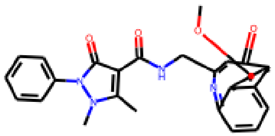	9.0	0.638163	3.995589	2.988471	5	0.774651
**1967**	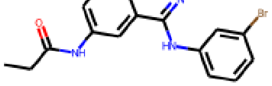	0.518519	O=C(CNC(=O)Oc1ccccc1)Nc1ccc2ncnc(Nc3cccc(Cl)c3)c2c1	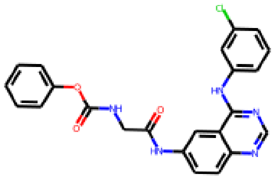	3.0	0.638163	3.995589	2.988471	4	0.793889

	src known mol	similarity	smiles	mol	Class	qed	logP	SAS	rings	kinase inhibition likeliness
**1968**	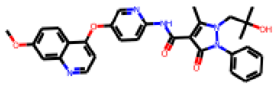	0.518498	COc1ccc2c(=O)cc(CNC(=O)c3c(C)n(C)n(-c4ccccc4)c3=O)nc3cccc3c2n1	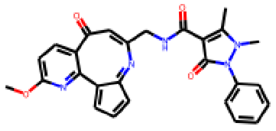	9.0	0.768132	3.608437	2.513170	5	0.823048
**1969**	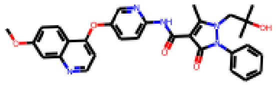	0.518498	COc1ccc2c(=O)cc(CNC(=O)c3c(C)n(C)n(-c4ccccc4)c3=O)nc3cccc3c2n1	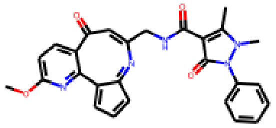	9.0	0.768132	3.608437	2.513170	5	0.823048
**1970**	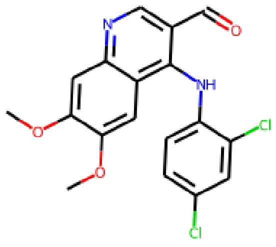	0.518412	COc1cc2ncc(C(=O)O)c(Nc3cccc(Cl)c3Cl)c2cc1OC1C(C)C1	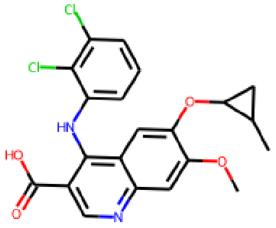	2.0	0.623362	2.525411	2.997881	4	0.774857
**1971**	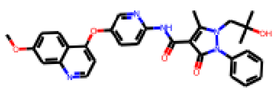	0.518228	COc1ccc2c(=O)cc(CNC(=O)c3c(C)n(C)n(-c4ccccc4)c3=O)nc3cccc3c2n1	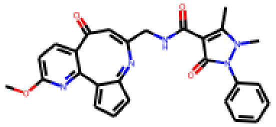	9.0	0.768132	3.608437	2.513170	5	0.823048

	src known mol	similarity	smiles	mol	Class	qed	logP	SAS	rings	kinase inhibition likeliness
**1972**	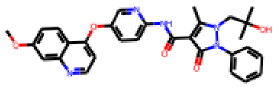	0.518228	COc1ccc2c(=O)cc(CNC(=O)c3c(C)n(C)n(-<br>c4ccccc4)c3=O)nc3cccc3c2n1	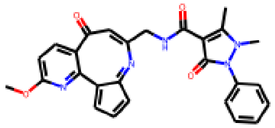	9.0	0.768132	3.608437	2.513170	5	0.823048
**1973**	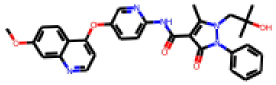	0.518201	COc1ccc2c(=O)cc(CNC(=O)c3c(C)n(C)n(-<br>c4ccccc4)c3=O)nc3cccc2c3c1	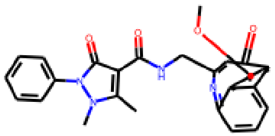	9.0	0.638163	3.995589	2.988471	5	0.774651
**1974**	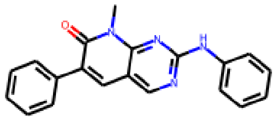	0.518100	Clc1cccc(Nc2ncc3cc(-c3c(Cl)cccc3Cl)c(=O)n(C)c3n2)c1	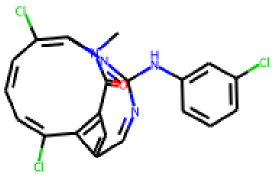	2.0	0.768132	3.608437	2.513170	5	0.784634
**1975**	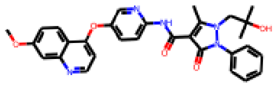	0.518028	COc1ccc2c(=O)cc(CNC(=O)c3c(C)n(C)n(-<br>c4ccccc4)c3=O)nc3cccc2c3c1	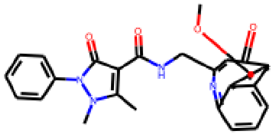	9.0	0.638163	3.995589	2.988471	5	0.774651

	src known mol	similarity	smiles	mol	Class	qed	logP	SAS	rings	kinase inhibition likeliness
**1976**	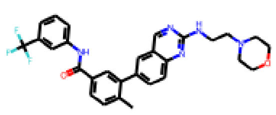	0.517992	Cc1ccc(C(=O)Oc2cccc(C(F)(F)F)c2)cc1-c1ccc2nc(N)ncc2c1	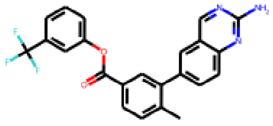	6.0	0.638163	3.995589	2.988471	4	0.789898
**1977**	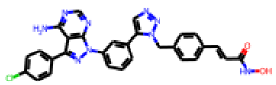	0.517670	Cc1ccc(C(=O)Nc2ccoc2)cc1Nc1ncnc2c1cnn2-c1ccccc1	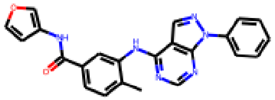	8.0	0.593231	3.250153	2.680275	5	0.871119
**1978**	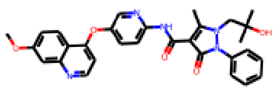	0.517647	COc1ccc2c(=O)cc(CNC(=O)c3c(C)n(C)n(-c4ccccc4)c3=O)nc3cccc2c3c1	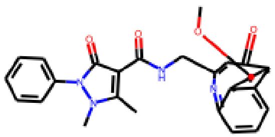	9.0	0.638163	3.995589	2.988471	5	0.774651
**1979**	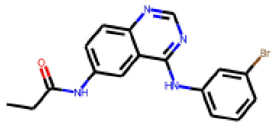	0.517372	OCC(C(N1CC)CC1)Nc1ccc2ncnc(Nc3cccc(Br)c3)c2c1	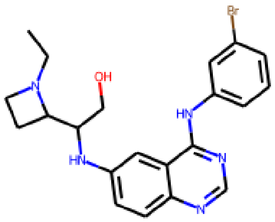	3.0	0.768132	3.608437	2.513170	4	0.789015

	src known mol	similarity	smiles	mol	Class	qed	logP	SAS	rings	kinase inhibition likeliness
**1980**	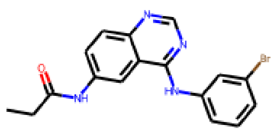	0.517372	OCC(C(N1CC)CC1)Nc1ccc2ncnc(Nc3cccc(Br)c3)c2c1	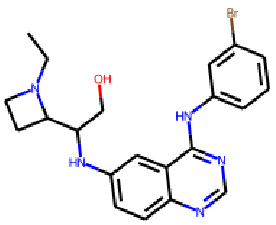	3.0	0.768132	3.608437	2.513170	4	0.789015
**1981**	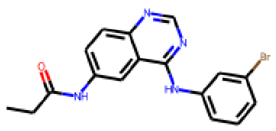	0.517214	OCC(C(N1CC)CC1)Nc1ccc2ncnc(Nc3cccc(Br)c3)c2c1	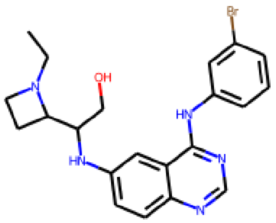	3.0	0.768132	3.608437	2.513170	4	0.789015
**1982**	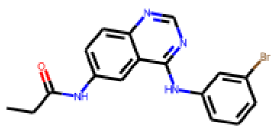	0.517214	OCC(C(N1CC)CC1)Nc1ccc2ncnc(Nc3cccc(Br)c3)c2c1	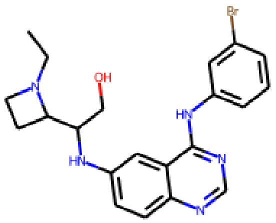	3.0	0.768132	3.608437	2.513170	4	0.789015
**1983**	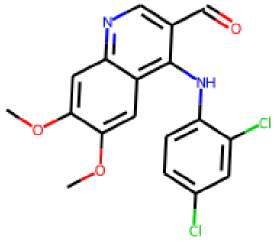	0.517169	COc1cc2ncc(C(=O)O)c(Nc3cccc(Cl)c3Cl)c2cc1OC1C(C)C1	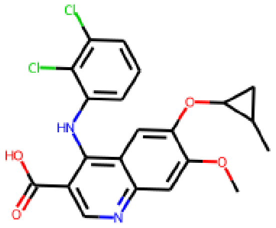	2.0	0.623362	2.525411	2.997881	4	0.774857

	src known mol	similarity	smiles	mol	Class	qed	logP	SAS	rings	kinase inhibition likeliness
**1984**	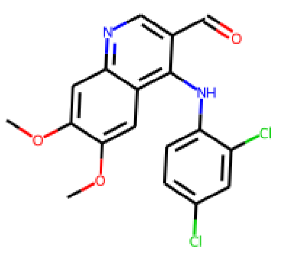	0.517169	COc1cc2ncc(C(=O)O)c(Nc3cccc(Cl)c3Cl)c2cc1OC1C(C)C1	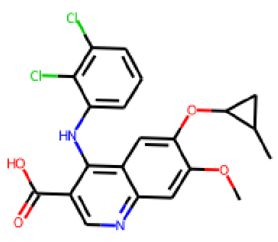	2.0	0.623362	2.525411	2.997881	4	0.774857
**1985**	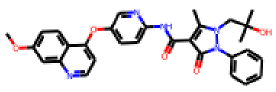	0.517108	COc1ccc2c(=O)cc(CNC(=O)c3c(C)n(C)n(-c4ccccc4)c3=O)nc3cccc3c2n1	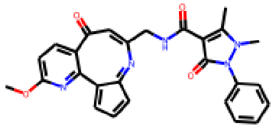	9.0	0.768132	3.608437	2.513170	5	0.823048
**1986**	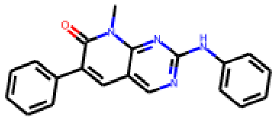	0.517023	Clc1cccc(Nc2ncc3cc(-c3c(Cl)cccc3Cl)c(=O)n(C)c3n2)c1	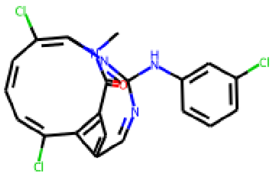	2.0	0.768132	3.608437	2.513170	5	0.784634
**1987**	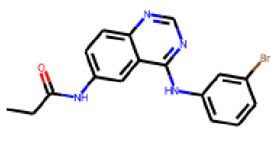	0.516720	O=C(CNC(=O)Oc1ccccc1)Nc1ccc2ncnc(Nc3cccc(Cl)c3)c2c1	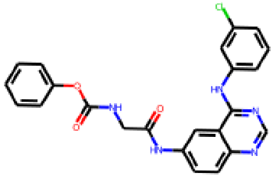	3.0	0.638163	3.995589	2.988471	4	0.793889

	src known mol	similarity	smiles	mol	Class	qed	logP	SAS	rings	kinase inhibition likeliness
**1988**	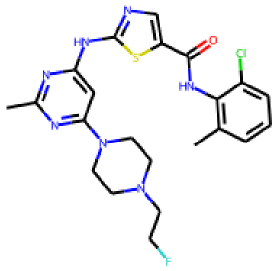	0.516656	Cc1nc(Nc2ncc(C(=O)Nc3c(C)cccc3Cl)s2)cc(N2C(NC(C)C2)O)n1	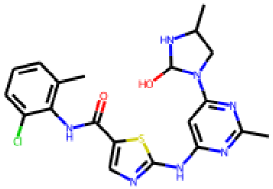	2.0	0.604401	2.952698	2.735638	4	0.781538
**1989**	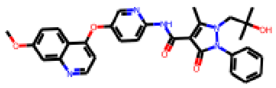	0.516640	COc1ccc2c(=O)cc(CNC(=O)c3c(C)n(C)n(-c4ccccc4)c3=O)nc3cccc2c3c1	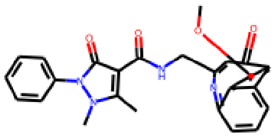	9.0	0.638163	3.995589	2.988471	5	0.774651
**1990**	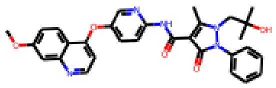	0.516542	COc1ccc2c(=O)cc(CNC(=O)c3c(C)n(C)n(-c4ccccc4)c3=O)nc3cccc2c3c1	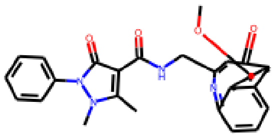	9.0	0.638163	3.995589	2.988471	5	0.774651
**1991**	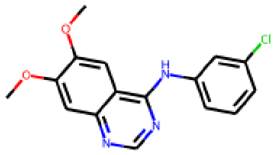	0.516360	Clc1cccc(Nc2ncnc3cc4c[nH]nc4cc23)c1	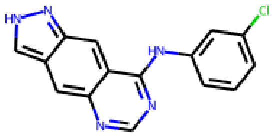	3.0	0.638163	3.995589	2.988471	4	0.781333

	src known mol	similarity	smiles	mol	Class	qed	logP	SAS	rings	kinase inhibition likeliness
**1992**	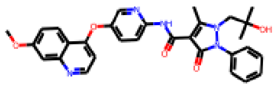	0.516164	COc1ccc2c(=O)cc(CNC(=O)c3c(C)n(C)n(-c4ccccc4)c3=O)nc3cccc2c3c1	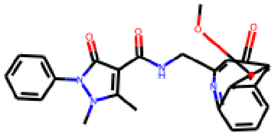	9.0	0.638163	3.995589	2.988471	5	0.774651
**1993**	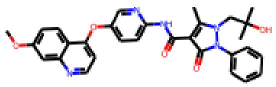	0.516043	COc1ccc2c(=O)cc(CNC(=O)c3c(C)n(C)n(-c4ccccc4)c3=O)nc3cccc2c3c1	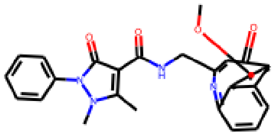	9.0	0.638163	3.995589	2.988471	5	0.774651
**1994**	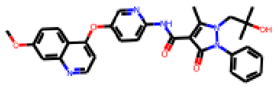	0.515852	COc1ccc2c(=O)cc(CNC(=O)c3c(C)n(C)n(-c4ccccc4)c3=O)nc3cccc2c3c1	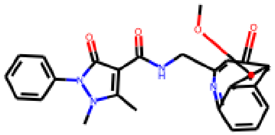	9.0	0.638163	3.995589	2.988471	5	0.774651
**1995**	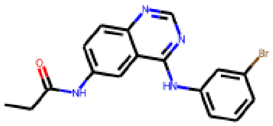	0.515781	OCC(C(N1CC)CC1)Nc1ccc2ncnc(Nc3cccc(Br)c3)c2c1	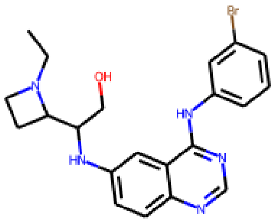	3.0	0.768132	3.608437	2.513170	4	0.789015

	src known mol	similarity	smiles	mol	Class	qed	logP	SAS	rings	kinase inhibition likeliness
**1996**	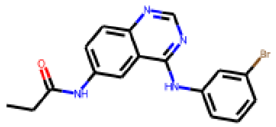	0.515781	OCC(C(N1CC)CC1)Nc1ccc2ncnc(Nc3cccc(Br)c3)c2c1	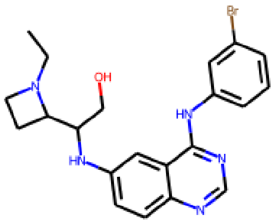	3.0	0.768132	3.608437	2.513170	4	0.789015
**1997**	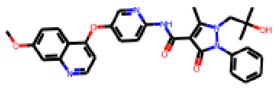	0.515650	COc1ccc2c(=O)cc(CNC(=O)c3c(C)n(C)n(-c4ccccc4)c3=O)nc3cccc2c3c1	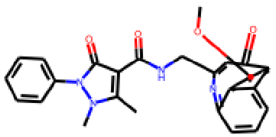	9.0	0.638163	3.995589	2.988471	5	0.774651
**1998**	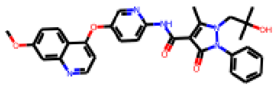	0.515625	COc1ccc2c(=O)cc(CNC(=O)c3c(C)n(C)n(-c4ccccc4)c3=O)nc3cccc3c2n1	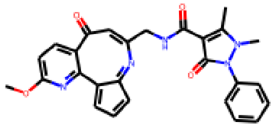	9.0	0.768132	3.608437	2.513170	5	0.823048
**1999**	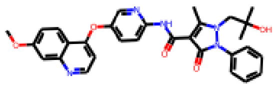	0.515184	COc1ccc2c(=O)cc(CNC(=O)c3c(C)n(C)n(-c4ccccc4)c3=O)nc3cccc2c3c1	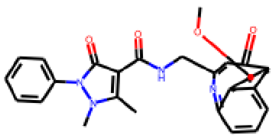	9.0	0.638163	3.995589	2.988471	5	0.774651

	src known mol	similarity	smiles	mol	Class	qed	logP	SAS	rings	kinase inhibition likeliness
**2000**	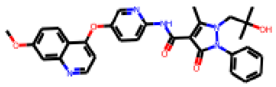	0.515118	COc1ccc2c(=O)cc(CNC(=O)c3c(C)n(C)n(-c4ccccc4)c3=O)nc3cccc3c2n1	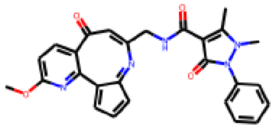	9.0	0.768132	3.608437	2.513170	5	0.823048
**2001**	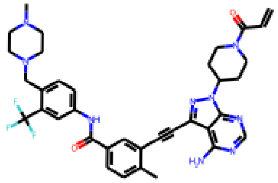	0.515113	Cc1ccc(C(=O)Nc2cccc(C(F)(C)F)c2C)cc1-c1ccc2nc(=O)ccc(C)cn2n1	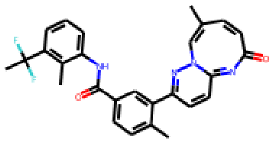	6.0	0.762994	3.476405	2.421118	4	0.764424
**2002**	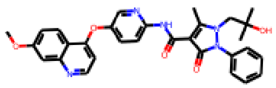	0.515103	COc1ccc2c(=O)cc(CNC(=O)c3c(C)n(C)n(-c4ccccc4)c3=O)nc3cccc2c3c1	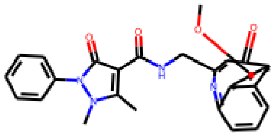	9.0	0.638163	3.995589	2.988471	5	0.774651
**2003**	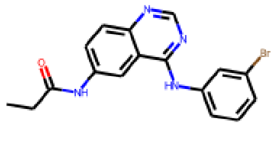	0.514965	O=C(COC(=O)Nc1CCccc1)Nc1ccc2ncnc(Nc3cccc(Br)c3)c2c1	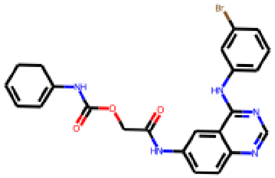	3.0	0.771867	2.951597	1.935530	4	0.775927

	src known mol	similarity	smiles	mol	Class	qed	logP	SAS	rings	kinase inhibition likeliness
**2004**	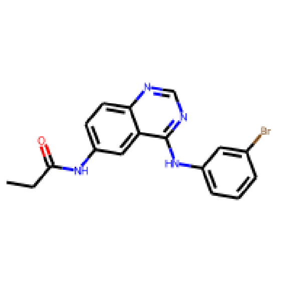	0.514965	O=C(COC(=O)Nc1CCccc1)Nc1ccc2ncnc(Nc3cccc(Br)c3)c2c1	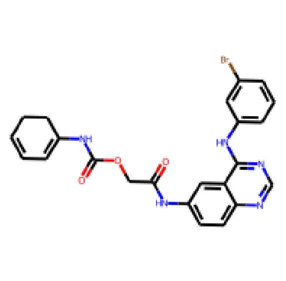	3.0	0.771867	2.951597	1.935530	4	0.775927
**2005**	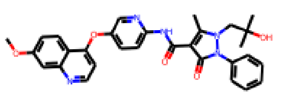	0.514909	COc1ccc2c(=O)cc(CNC(=O)c3c(C)n(C)n(-c4ccccc4)c3=O)nc3cccc2c3c1	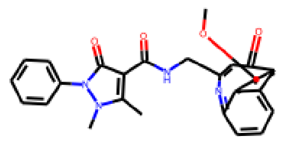	9.0	0.638163	3.995589	2.988471	5	0.774651
**2006**	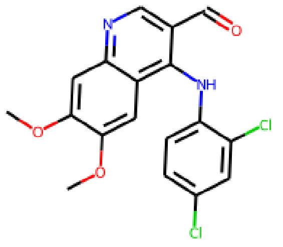	0.514104	COc1cc2ncc(C(=O)O)c(Nc3cccc(Cl)c3Cl)c2cc1OC1C(C)C1	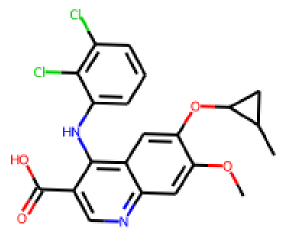	2.0	0.623362	2.525411	2.997881	4	0.774857
**2007**	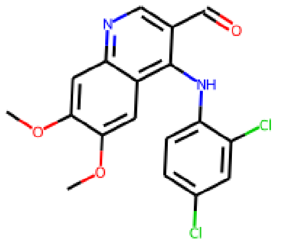	0.514104	COc1cc2ncc(C(=O)O)c(Nc3cccc(Cl)c3Cl)c2cc1OC1C(C)C1	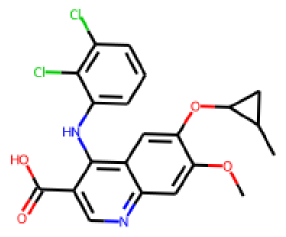	2.0	0.623362	2.525411	2.997881	4	0.774857

	src known mol	similarity	smiles	mol	Class	qed	logP	SAS	rings	kinase inhibition likeliness
**2008**	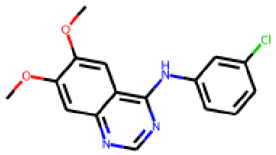	0.513733	Clc1cccc(Nc2ncnc3cc4c[nH]nc4cc23)c1	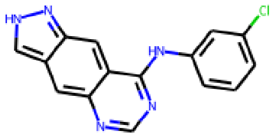	3.0	0.638163	3.995589	2.988471	4	0.781333
**2009**	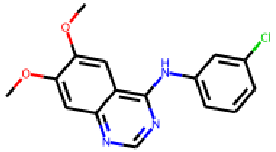	0.513733	Clc1cccc(Nc2ncnc3cc4c[nH]nc4cc23)c1	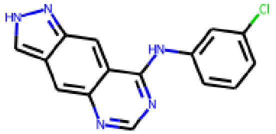	3.0	0.638163	3.995589	2.988471	4	0.781333
**2010**	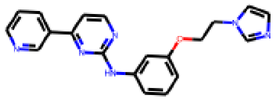	0.513536	Cc1ccc(OC(=O)c2ccccc2)cc1Nc1nccc(-c2cccnc2)n1	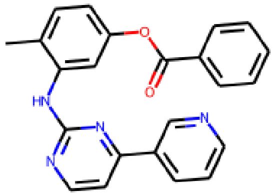	0.0	0.638163	3.995589	2.988471	4	0.775468
**2011**	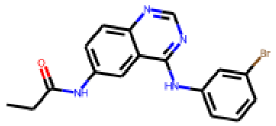	0.513328	O=C(COC(=O)Nc1CCccc1)Nc1ccc2ncnc(Nc3cccc(Br)c3)c2c1	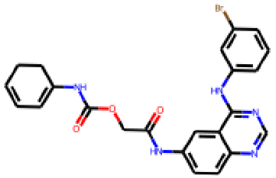	3.0	0.771867	2.951597	1.935530	4	0.775927

	src known mol	similarity	smiles	mol	Class	qed	logP	SAS	rings	kinase inhibition likeliness
**2012**	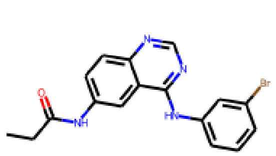	0.513328	O=C(COC(=O)Nc1CCccc1)Nc1ccc2ncnc(Nc3cccc(Br)c3)c2c1	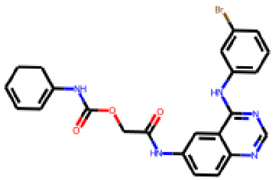	3.0	0.771867	2.951597	1.935530	4	0.775927
**2013**	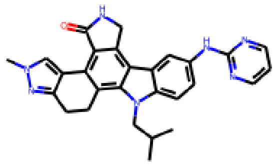	0.513317	C#Cc1ccc(C(=O)OC)cc1-c1C(=O)c-n2nc(-c3ccc(F)cc3F)cc2C1	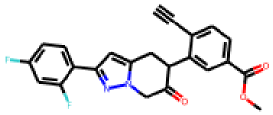	8.0	0.768132	3.608437	2.513170	4	0.763730
**2014**	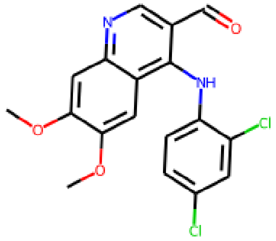	0.513302	COc1cc2ncc(C(=O)O)c(Nc3cccc(Cl)c3Cl)c2cc1OC1C(C)C1	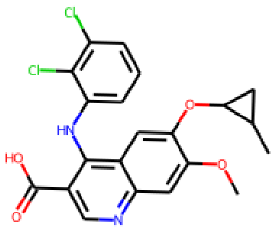	2.0	0.623362	2.525411	2.997881	4	0.774857
**2015**	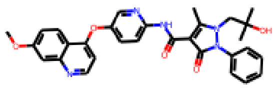	0.513222	COc1ccc2c(=O)cc(CNC(=O)c3c(C)n(C)n(-<br>c4ccccc4)c3=O)nc3cccc2c3c1	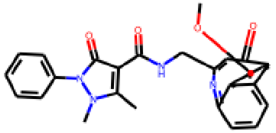	9.0	0.638163	3.995589	2.988471	5	0.774651

	src known mol	similarity	smiles	mol	Class	qed	logP	SAS	rings	kinase inhibition likeliness
**2016**	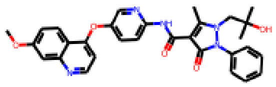	0.513222	COc1ccc2c(=O)cc(CNC(=O)c3c(C)n(C)n(-c4ccccc4)c3=O)nc3cccc2c3c1	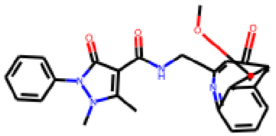	9.0	0.638163	3.995589	2.988471	5	0.774651
**2017**	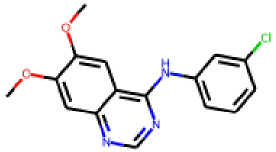	0.513211	Clc1cccc(Nc2ncnc3cc4c[nH]nc4cc23)c1	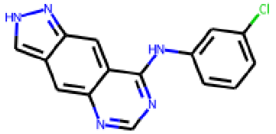	3.0	0.638163	3.995589	2.988471	4	0.781333
**2018**	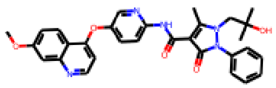	0.513025	COc1ccc2c(=O)cc(CNC(=O)c3c(C)n(C)n(-c4ccccc4)c3=O)nc3cccc2c3c1	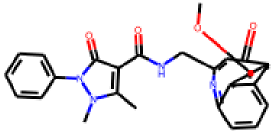	9.0	0.638163	3.995589	2.988471	5	0.774651
**2019**	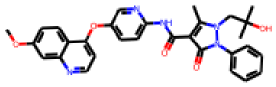	0.512614	COc1ccc2c(=O)cc(CNC(=O)c3c(C)n(C)n(-c4ccccc4)c3=O)nc3cccc2c3c1	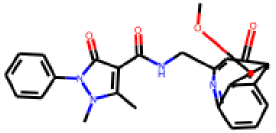	9.0	0.638163	3.995589	2.988471	5	0.774651

	src known mol	similarity	smiles	mol	Class	qed	logP	SAS	rings	kinase inhibition likeliness
**2020**	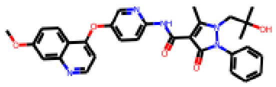	0.512326	COc1ccc2c(=O)cc(CNC(=O)c3c(C)n(C)n(-c4ccccc4)c3=O)nc3cccc2c3c1	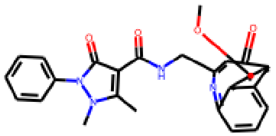	9.0	0.638163	3.995589	2.988471	5	0.774651
**2021**	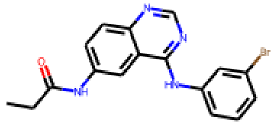	0.512214	OCC(C(N1CC)CC1)Nc1ccc2ncnc(Nc3cccc(Br)c3)c2c1	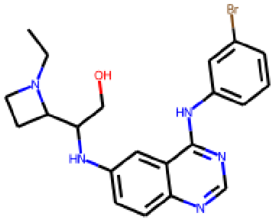	3.0	0.768132	3.608437	2.513170	4	0.789015
**2022**	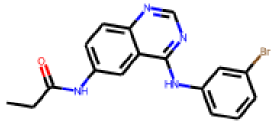	0.512214	OCC(C(N1CC)CC1)Nc1ccc2ncnc(Nc3cccc(Br)c3)c2c1	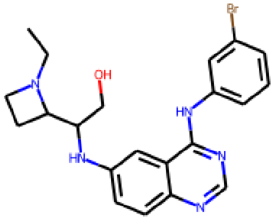	3.0	0.768132	3.608437	2.513170	4	0.789015
**2023**	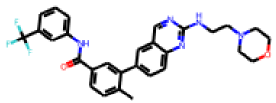	0.511795	Cc1ccc(C(=O)Nc2cccc(C(F)(F)F)c2C)cc1-c1ccc2cc(N)ncc2c1	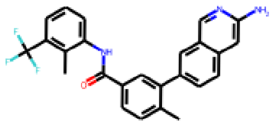	6.0	0.771867	2.951597	1.935530	4	0.754945

	src known mol	similarity	smiles	mol	Class	qed	logP	SAS	rings	kinase inhibition likeliness
**2024**	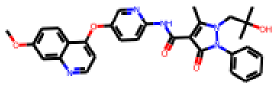	0.511565	COc1ccc2c(=O)cc(CNC(=O)c3c(C)n(C)n(-c4ccccc4)c3=O)nc3cccc2c3c1	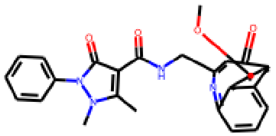	9.0	0.638163	3.995589	2.988471	5	0.774651
**2025**	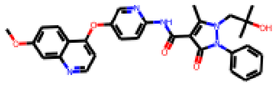	0.511565	COc1ccc2c(=O)cc(CNC(=O)c3c(C)n(C)n(-c4ccccc4)c3=O)nc3cccc2c3c1	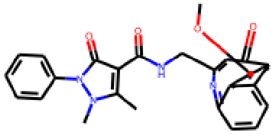	9.0	0.638163	3.995589	2.988471	5	0.774651
**2026**	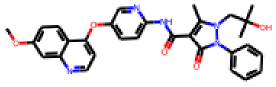	0.510961	COc1ccc2c(=O)cc(CNC(=O)c3c(C)n(C)n(-c4ccccc4)c3=O)nc3cccc3c2n1	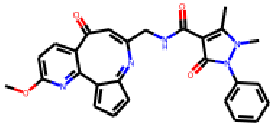	9.0	0.768132	3.608437	2.513170	5	0.823048
**2027**	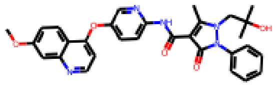	0.510961	COc1ccc2c(=O)cc(CNC(=O)c3c(C)n(C)n(-c4ccccc4)c3=O)nc3cccc3c2n1	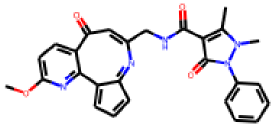	9.0	0.768132	3.608437	2.513170	5	0.823048

	src known mol	similarity	smiles	mol	Class	qed	logP	SAS	rings	kinase inhibition likeliness
**2028**	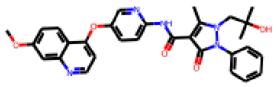	0.510961	COc1ccc2c(=O)cc(CNC(=O)c3c(C)n(C)n(-c4ccccc4)c3=O)nc3cccc3c2n1	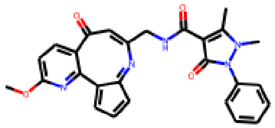	9.0	0.768132	3.608437	2.513170	5	0.823048
**2029**	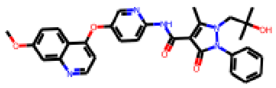	0.510961	COc1ccc2c(=O)cc(CNC(=O)c3c(C)n(C)n(-c4ccccc4)c3=O)nc3cccc3c2n1	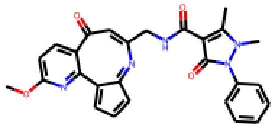	9.0	0.768132	3.608437	2.513170	5	0.823048
**2030**	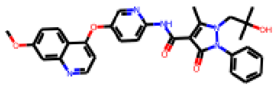	0.510957	COc1ccc2c(=O)cc(CNC(=O)c3c(C)n(C)n(-c4ccccc4)c3=O)nc3cccc2c3c1	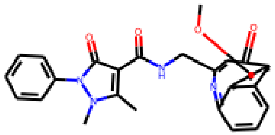	9.0	0.638163	3.995589	2.988471	5	0.774651
**2031**	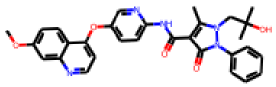	0.510945	COc1ccc2c(=O)cc(CNC(=O)c3c(C)n(C)n(-c4ccccc4)c3=O)nc3cccc2c3c1	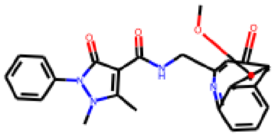	9.0	0.638163	3.995589	2.988471	5	0.774651

	src known mol	similarity	smiles	mol	Class	qed	logP	SAS	rings	kinase inhibition likeliness
**2032**	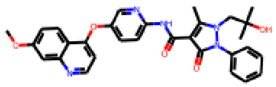	0.510945	COc1ccc2c(=O)cc(CNC(=O)c3c(C)n(C)n(-c4ccccc4)c3=O)nc3cccc2c3c1	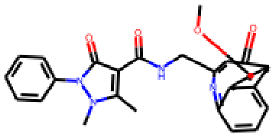	9.0	0.638163	3.995589	2.988471	5	0.774651
**2033**	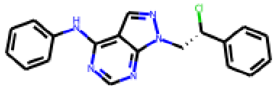	0.510911	Cc1ccc(C(=O)Nc2ccon2)cc1Nc1ncnc2c1cnn2C1cccccc1	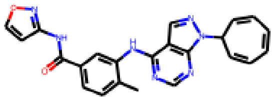	8.0	0.623362	2.525411	2.997881	5	0.770298
**2034**	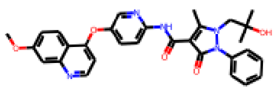	0.510910	COc1ccc2c(=O)cc(CNC(=O)c3c(C)n(C)n(-c4ccccc4)c3=O)nc3cccc2c3c1	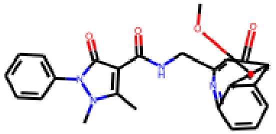	9.0	0.638163	3.995589	2.988471	5	0.774651
**2035**	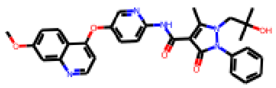	0.510638	COc1ccc2c(=O)cc(CNC(=O)c3c(C)n(C)n(-c4ccccc4)c3=O)nc3cccc2c3c1	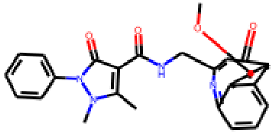	9.0	0.638163	3.995589	2.988471	5	0.774651

	src known mol	similarity	smiles	mol	Class	qed	logP	SAS	rings	kinase inhibition likeliness
**2036**	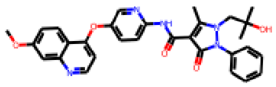	0.510556	COc1ccc2c(=O)cc(CNC(=O)c3c(C)n(C)n(-c4ccccc4)c3=O)nc3cccc3c2n1	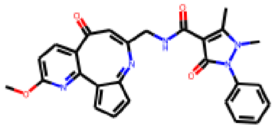	9.0	0.768132	3.608437	2.513170	5	0.823048
**2037**	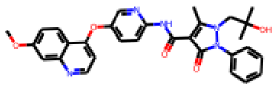	0.510182	COc1ccc2c(=O)cc(CNC(=O)c3c(C)n(C)n(-c4ccccc4)c3=O)nc3cccc2c3c1	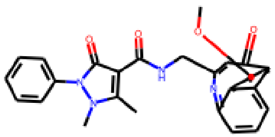	9.0	0.638163	3.995589	2.988471	5	0.774651
**2038**	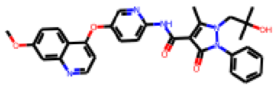	0.510124	COc1ccc2c(=O)cc(CNC(=O)c3c(C)n(C)n(-c4ccccc4)c3=O)nc3cccc3c2n1	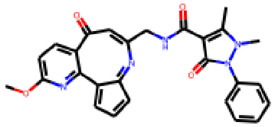	9.0	0.768132	3.608437	2.513170	5	0.823048
**2039**	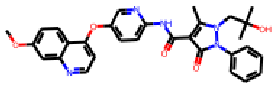	0.510124	COc1ccc2c(=O)cc(CNC(=O)c3c(C)n(C)n(-c4ccccc4)c3=O)nc3cccc3c2n1	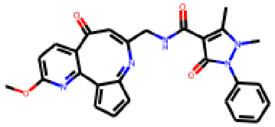	9.0	0.768132	3.608437	2.513170	5	0.823048

	src known mol	similarity	smiles	mol	Class	qed	logP	SAS	rings	kinase inhibition likeliness
**2040**	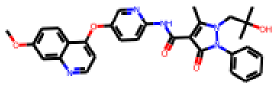	0.510124	COc1ccc2c(=O)cc(CNC(=O)c3c(C)n(C)n(-c4ccccc4)c3=O)nc3cccc3c2n1	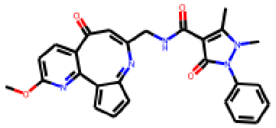	9.0	0.768132	3.608437	2.513170	5	0.823048
**2041**	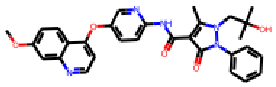	0.510124	COc1ccc2c(=O)cc(CNC(=O)c3c(C)n(C)n(-c4ccccc4)c3=O)nc3cccc3c2n1	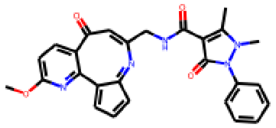	9.0	0.768132	3.608437	2.513170	5	0.823048
**2042**	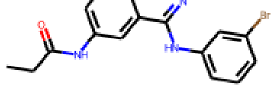	0.510062	O=C(CNC(=O)Oc1ccccc1)Nc1ccc2ncnc(Nc3cccc(Cl)c3)c2c1	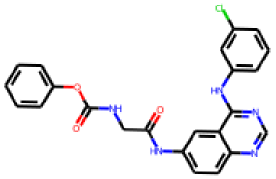	3.0	0.638163	3.995589	2.988471	4	0.793889
**2043**	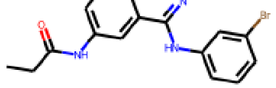	0.510062	O=C(CNC(=O)Oc1ccccc1)Nc1ccc2ncnc(Nc3cccc(Cl)c3)c2c1	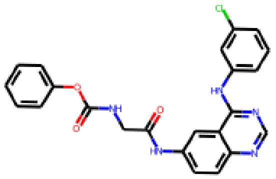	3.0	0.638163	3.995589	2.988471	4	0.793889

	src known mol	similarity	smiles	mol	Class	qed	logP	SAS	rings	kinase inhibition likeliness
**2044**	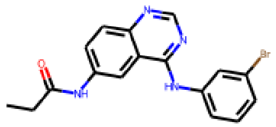	0.509745	OCC(C(N1CC)CC1)Nc1ccc2ncnc(Nc3cccc(Br)c3)c2c1	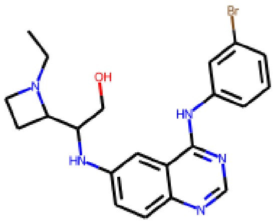	3.0	0.768132	3.608437	2.513170	4	0.789015
**2045**	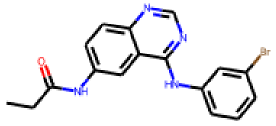	0.509745	OCC(C(N1CC)CC1)Nc1ccc2ncnc(Nc3cccc(Br)c3)c2c1	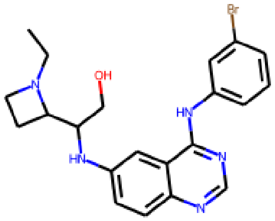	3.0	0.768132	3.608437	2.513170	4	0.789015
**2046**	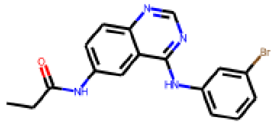	0.509363	OCC(C(N1CC)CC1)Nc1ccc2ncnc(Nc3cccc(Br)c3)c2c1	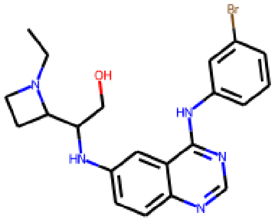	3.0	0.768132	3.608437	2.513170	4	0.789015
**2047**	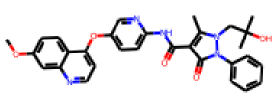	0.509323	COc1ccc2c(=O)cc(CNC(=O)c3c(C)n(C)n(-c4ccccc4)c3=O)nc3cccc2c3c1	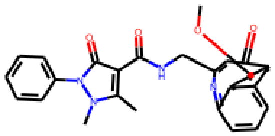	9.0	0.638163	3.995589	2.988471	5	0.774651

	src known mol	similarity	smiles	mol	Class	qed	logP	SAS	rings	kinase inhibition likeliness
**2048**	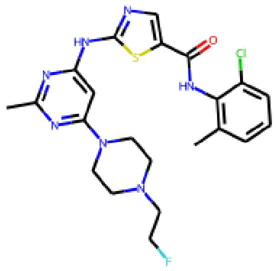	0.509066	Cc1nc(Nc2ncc(C(=O)Nc3c(C)cccc3Cl)s2)cc(N2C(NC(C)C2)O)n1	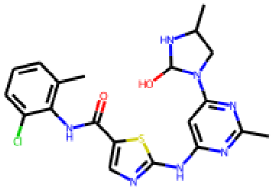	2.0	0.604401	2.952698	2.735638	4	0.781538
**2049**	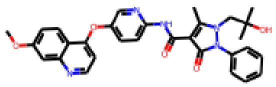	0.508885	COc1ccc2c(=O)cc(CNC(=O)c3c(C)n(C)n(-c4ccccc4)c3=O)nc3cccc2c3c1	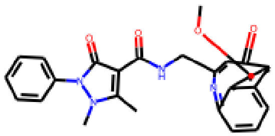	9.0	0.638163	3.995589	2.988471	5	0.774651
**2050**	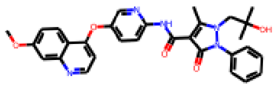	0.508885	COc1ccc2c(=O)cc(CNC(=O)c3c(C)n(C)n(-c4ccccc4)c3=O)nc3cccc2c3c1	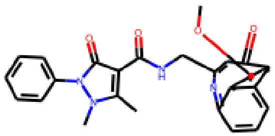	9.0	0.638163	3.995589	2.988471	5	0.774651
**2051**	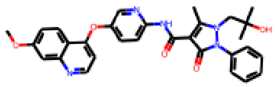	0.508838	COc1ccc2c(=O)cc(CNC(=O)c3c(C)n(C)n(-c4ccccc4)c3=O)nc3cccc2c3c1	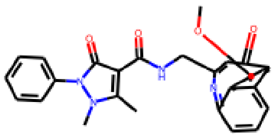	9.0	0.638163	3.995589	2.988471	5	0.774651

	src known mol	similarity	smiles	mol	Class	qed	logP	SAS	rings	kinase inhibition likeliness
**2052**	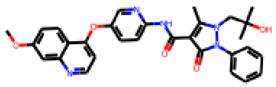	0.508593	COc1ccc2c(=O)cc(CNC(=O)c3c(C)n(C)n(-c4ccccc4)c3=O)nc3cccc2c3c1	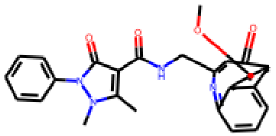	9.0	0.638163	3.995589	2.988471	5	0.774651
**2053**	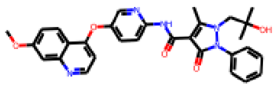	0.508593	COc1ccc2c(=O)cc(CNC(=O)c3c(C)n(C)n(-c4ccccc4)c3=O)nc3cccc2c3c1	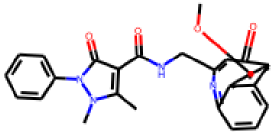	9.0	0.638163	3.995589	2.988471	5	0.774651
**2054**	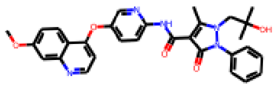	0.508574	COc1ccc2c(=O)cc(CNC(=O)c3c(C)n(C)n(-c4ccccc4)c3=O)nc3cccc2c3c1	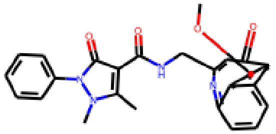	9.0	0.638163	3.995589	2.988471	5	0.774651
**2055**	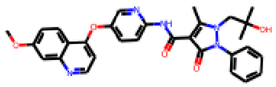	0.507865	COc1ccc2c(=O)cc(CNC(=O)c3c(C)n(C)n(-c4ccccc4)c3=O)nc3cccc3c2n1	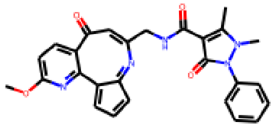	9.0	0.768132	3.608437	2.513170	5	0.823048

	src known mol	similarity	smiles	mol	Class	qed	logP	SAS	rings	kinase inhibition likeliness
**2056**	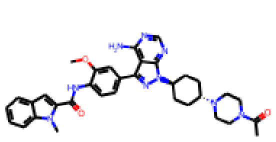	0.507778	Cc1ccc(C(=O)Nc2cccc(C(F)(F)F)c2C)cc1-c1ccc2oc(=O)cccc(F)c2n1	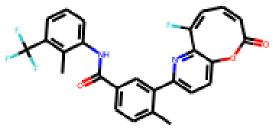	6.0	0.790896	3.985943	2.115897	4	0.783675
**2057**	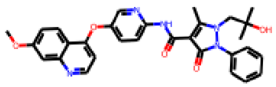	0.507463	COc1ccc2c(=O)cc(CNC(=O)c3c(C)n(C)n(-c4ccccc4)c3=O)nc3cccc2c3c1	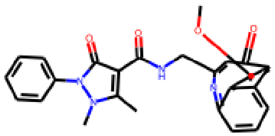	9.0	0.638163	3.995589	2.988471	5	0.774651
**2058**	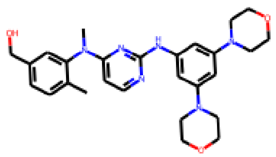	0.507014	NC(=O)c1ccccc1Nc1nc(Nc2ccc(N3CCOCC3)cc2)ncc1Cl	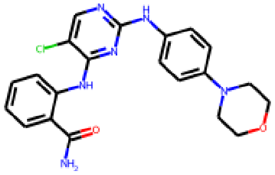	7.0	0.768132	3.608437	2.513170	4	0.755040
**2059**	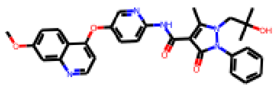	0.506878	COc1ccc2c(=O)cc(CNC(=O)c3c(C)n(C)n(-c4ccccc4)c3=O)nc3cccc2c3c1	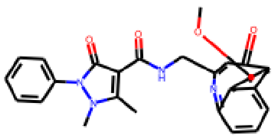	9.0	0.638163	3.995589	2.988471	5	0.774651

	src known mol	similarity	smiles	mol	Class	qed	logP	SAS	rings	kinase inhibition likeliness
**2060**	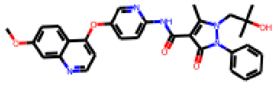	0.506746	COc1ccc2c(=O)cc(CNC(=O)c3c(C)n(C)n(-c4ccccc4)c3=O)nc3cccc2c3c1	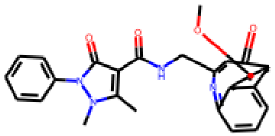	9.0	0.638163	3.995589	2.988471	5	0.774651
**2061**	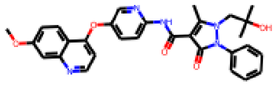	0.506746	COc1ccc2c(=O)cc(CNC(=O)c3c(C)n(C)n(-c4ccccc4)c3=O)nc3cccc2c3c1	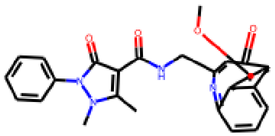	9.0	0.638163	3.995589	2.988471	5	0.774651
**2062**	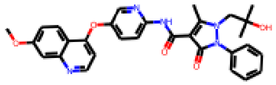	0.506681	COc1ccc2c(=O)cc(CNC(=O)c3c(C)n(C)n(-c4ccccc4)c3=O)nc3cccc2c3c1	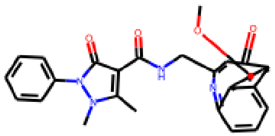	9.0	0.638163	3.995589	2.988471	5	0.774651
**2063**	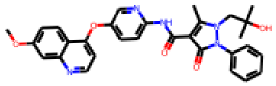	0.506652	COc1ccc2c(=O)cc(CNC(=O)c3c(C)n(C)n(-c4ccccc4)c3=O)nc3cccc2c3c1	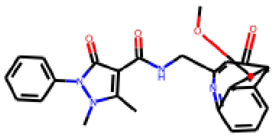	9.0	0.638163	3.995589	2.988471	5	0.774651

	src known mol	similarity	smiles	mol	Class	qed	logP	SAS	rings	kinase inhibition likeliness
**2064**	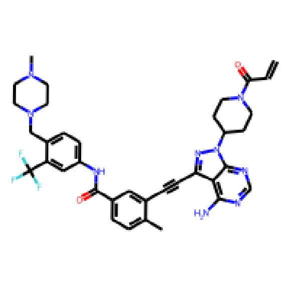	0.506462	Cc1ccc(C(=O)Nc2cccc(C(F)(C)F)c2C)cc1-c1ccc2nc(=O)ccc(C)cn2n1	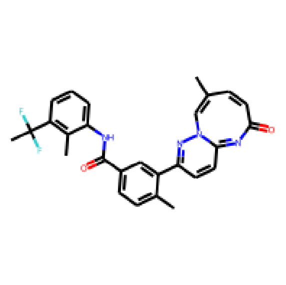	6.0	0.762994	3.476405	2.421118	4	0.764424
**2065**	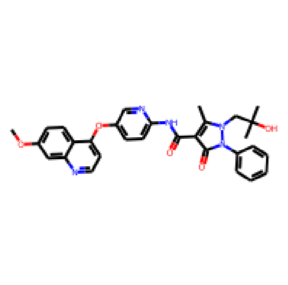	0.506459	COc1ccc2c(=O)cc(CNC(=O)c3c(C)n(C)n(-c4ccccc4)c3=O)nc3cccc2c3c1	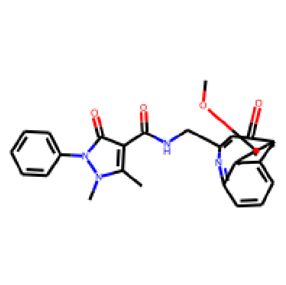	9.0	0.638163	3.995589	2.988471	5	0.774651
**2066**	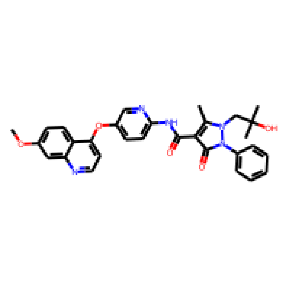	0.506363	COc1ccc2c(=O)cc(CNC(=O)c3c(C)n(C)n(-c4ccccc4)c3=O)nc3cccc2c3c1	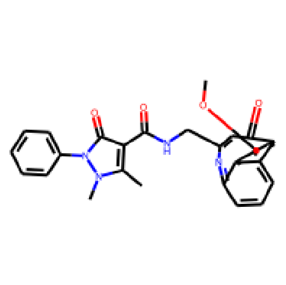	9.0	0.638163	3.995589	2.988471	5	0.774651
**2067**	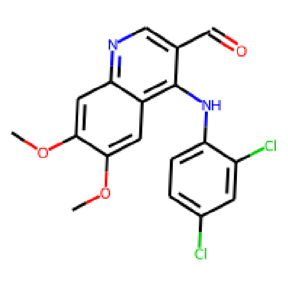	0.506173	COc1cc2ncc(C(=O)O)c(Nc3cccc(Cl)c3Cl)c2cc1OC1C(C)C1	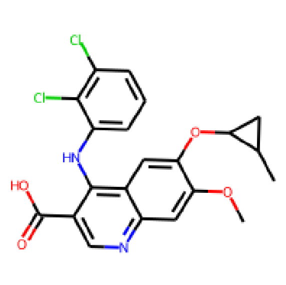	2.0	0.623362	2.525411	2.997881	4	0.774857

	src known mol	similarity	smiles	mol	Class	qed	logP	SAS	rings	kinase inhibition likeliness
**2068**	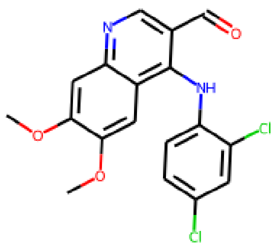	0.506173	COc1cc2ncc(C(=O)O)c(Nc3cccc(Cl)c3Cl)c2cc1OC1C(C)C1	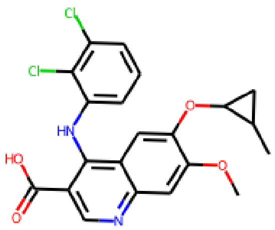	2.0	0.623362	2.525411	2.997881	4	0.774857
**2069**	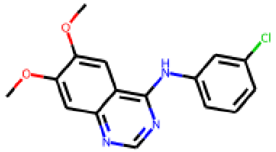	0.506098	Clc1cccc(Nc2ncnc3cc4c[nH]nc4cc23)c1	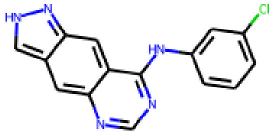	3.0	0.638163	3.995589	2.988471	4	0.781333
**2070**	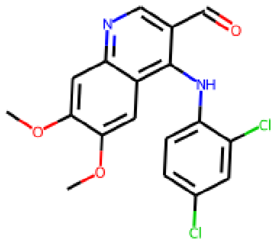	0.505495	COc1cc2ncc(C(=O)O)c(Nc3cccc(Cl)c3Cl)c2cc1OC1C(C)C1	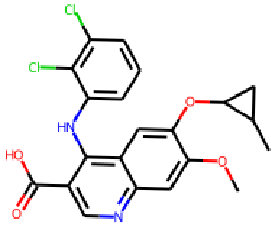	2.0	0.623362	2.525411	2.997881	4	0.774857
**2071**	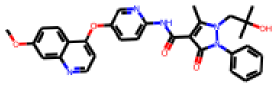	0.505371	COc1ccc2c(=O)cc(CNC(=O)c3c(C)n(C)n(-c4ccccc4)c3=O)nc3cccc2c3c1	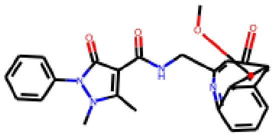	9.0	0.638163	3.995589	2.988471	5	0.774651

	src known mol	similarity	smiles	mol	Class	qed	logP	SAS	rings	kinase inhibition likeliness
**2072**	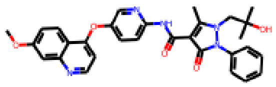	0.505328	COc1ccc2c(=O)cc(CNC(=O)c3c(C)n(C)n(-c4ccccc4)c3=O)nc3cccc3c2n1	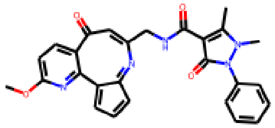	9.0	0.768132	3.608437	2.513170	5	0.823048
**2073**	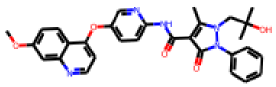	0.505308	COc1ccc2c(=O)cc(CNC(=O)c3c(C)n(C)n(-c4ccccc4)c3=O)nc3cccc2c3c1	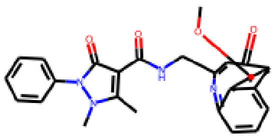	9.0	0.638163	3.995589	2.988471	5	0.774651
**2074**	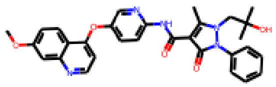	0.505308	COc1ccc2c(=O)cc(CNC(=O)c3c(C)n(C)n(-c4ccccc4)c3=O)nc3cccc2c3c1	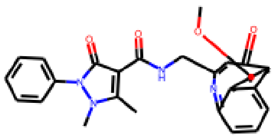	9.0	0.638163	3.995589	2.988471	5	0.774651
**2075**	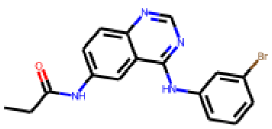	0.505271	O=C(COC(=O)Nc1CCccc1)Nc1ccc2ncnc(Nc3cccc(Br)c3)c2c1	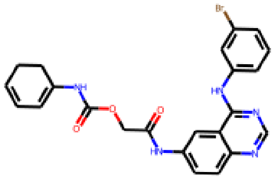	3.0	0.771867	2.951597	1.935530	4	0.775927

	src known mol	similarity	smiles	mol	Class	qed	logP	SAS	rings	kinase inhibition likeliness
**2076**	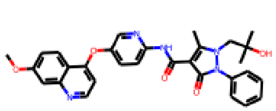	0.504762	COc1ccc2c(=O)cc(CNC(=O)c3c(C)n(C)n(-c4ccccc4)c3=O)nc3cccc2c3c1	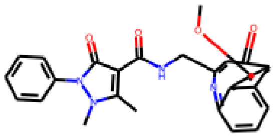	9.0	0.638163	3.995589	2.988471	5	0.774651
**2077**	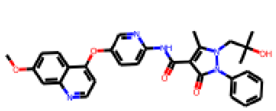	0.504039	COc1ccc2c(=O)cc(CNC(=O)c3c(C)n(C)n(-c4ccccc4)c3=O)nc3cccc2c3c1	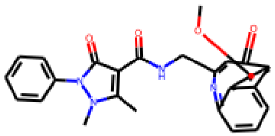	9.0	0.638163	3.995589	2.988471	5	0.774651
**2078**	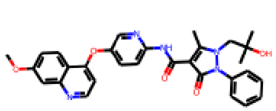	0.503983	COc1ccc2c(=O)cc(CNC(=O)c3c(C)n(C)n(-c4ccccc4)c3=O)nc3cccc2c3c1	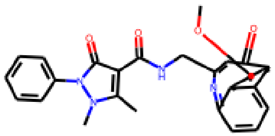	9.0	0.638163	3.995589	2.988471	5	0.774651
**2079**	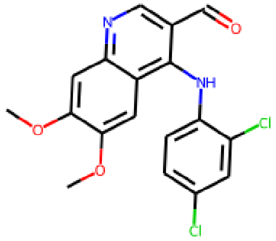	0.503524	COc1cc2ncc(C(=O)O)c(Nc3cccc(Cl)c3Cl)c2cc1OC1C(C)C1	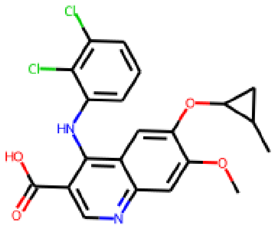	2.0	0.623362	2.525411	2.997881	4	0.774857

	src known mol	similarity	smiles	mol	Class	qed	logP	SAS	rings	kinase inhibition likeliness
**2080**	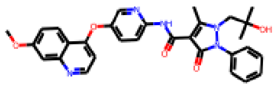	0.503493	COc1ccc2c(=O)cc(CNC(=O)c3c(C)n(C)n(-c4ccccc4)c3=O)nc3cccc2c3c1	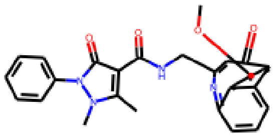	9.0	0.638163	3.995589	2.988471	5	0.774651
**2081**	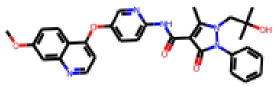	0.503229	COc1ccc2c(=O)cc(CNC(=O)c3c(C)n(C)n(-c4ccccc4)c3=O)nc3cccc2c3c1	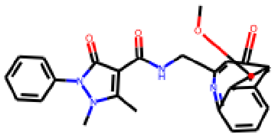	9.0	0.638163	3.995589	2.988471	5	0.774651
**2082**	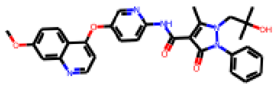	0.503229	COc1ccc2c(=O)cc(CNC(=O)c3c(C)n(C)n(-c4ccccc4)c3=O)nc3cccc2c3c1	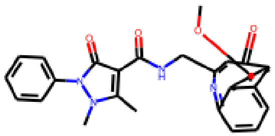	9.0	0.638163	3.995589	2.988471	5	0.774651
**2083**	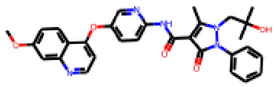	0.502940	COc1ccc2c(=O)cc(CNC(=O)c3c(C)n(C)n(-c4ccccc4)c3=O)nc3cccc2c3c1	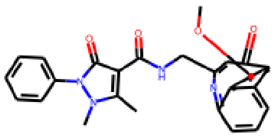	9.0	0.638163	3.995589	2.988471	5	0.774651

	src known mol	similarity	smiles	mol	Class	qed	logP	SAS	rings	kinase inhibition likeliness
**2084**	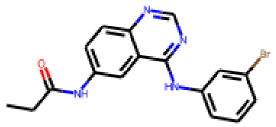	0.502674	O=C(COC(=O)Nc1CCccc1)Nc1ccc2ncnc(Nc3cccc(Br)c3)c2c1	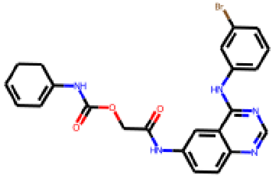	3.0	0.771867	2.951597	1.935530	4	0.775927
**2085**	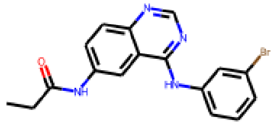	0.502674	O=C(COC(=O)Nc1CCccc1)Nc1ccc2ncnc(Nc3cccc(Br)c3)c2c1	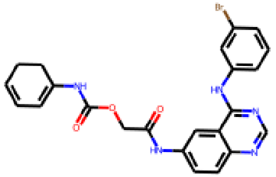	3.0	0.771867	2.951597	1.935530	4	0.775927
**2086**	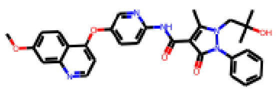	0.502410	COc1ccc2c(=O)cc(CNC(=O)c3c(C)n(C)n(-c4ccccc4)c3=O)nc3cccc2c3c1	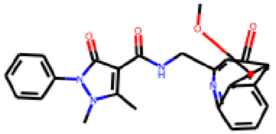	9.0	0.638163	3.995589	2.988471	5	0.774651
**2087**	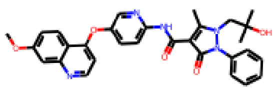	0.502410	COc1ccc2c(=O)cc(CNC(=O)c3c(C)n(C)n(-c4ccccc4)c3=O)nc3cccc2c3c1	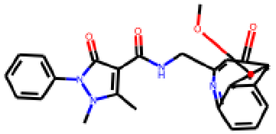	9.0	0.638163	3.995589	2.988471	5	0.774651

	src known mol	similarity	smiles	mol	Class	qed	logP	SAS	rings	kinase inhibition likeliness
**2088**	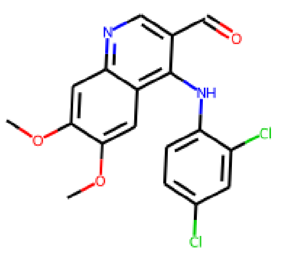	0.502203	COc1cc2ncc(C(=O)O)c(Nc3cccc(Cl)c3Cl)c2cc1OC1C(C)C1	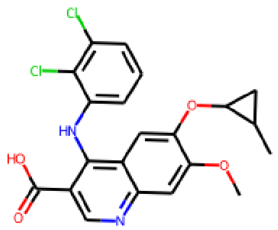	2.0	0.623362	2.525411	2.997881	4	0.774857
**2089**	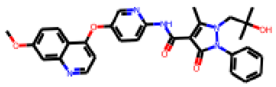	0.502121	COc1ccc2c(=O)cc(CNC(=O)c3c(C)n(C)n(-c4ccccc4)c3=O)nc3cccc2c3c1	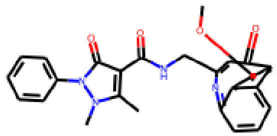	9.0	0.638163	3.995589	2.988471	5	0.774651
**2090**	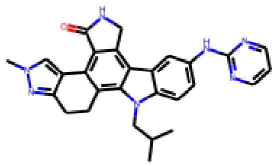	0.502120	Cc1ccc(C(=O)Nc2cccc(C(F)(C)F)c2C)cc1-c1ccc2nc(=O)cccc(F)nc2n1	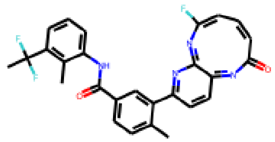	6.0	0.820294	3.038428	1.698172	4	0.833140
**2091**	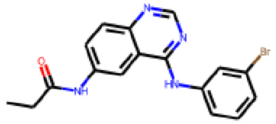	0.502031	O=C(CNC(=O)Oc1ccccc1)Nc1ccc2ncnc(Nc3cccc(Cl)c3)c2c1	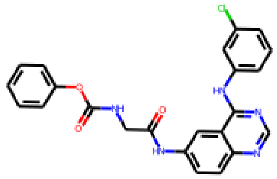	3.0	0.638163	3.995589	2.988471	4	0.793889

	src known mol	similarity	smiles	mol	Class	qed	logP	SAS	rings	kinase inhibition likeliness
**2092**	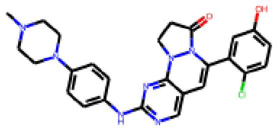	0.501961	O/C(Nc1ccc(Oc2ccccc2)cc1)Oc1ccc2(Cc1)NC(=O)/C(=O)c1ccc(O)n12	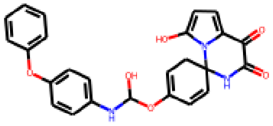	4.0	0.520676	2.336694	2.899742	5	0.820778
**2093**	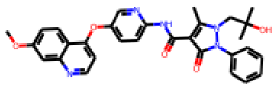	0.501883	COc1ccc2c(=O)cc(CNC(=O)c3c(C)n(C)n(-c4ccccc4)c3=O)nc3cccc2c3c1	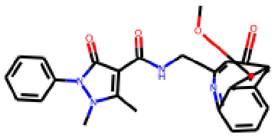	9.0	0.638163	3.995589	2.988471	5	0.774651
**2094**	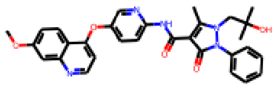	0.501587	COc1ccc2c(=O)cc(CNC(=O)c3c(C)n(C)n(-c4ccccc4)c3=O)nc3cccc2c3c1	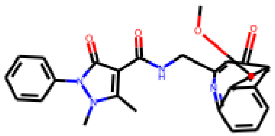	9.0	0.638163	3.995589	2.988471	5	0.774651
**2095**	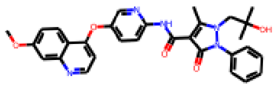	0.501587	COc1ccc2c(=O)cc(CNC(=O)c3c(C)n(C)n(-c4ccccc4)c3=O)nc3cccc2c3c1	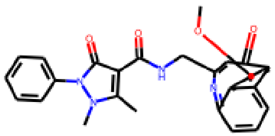	9.0	0.638163	3.995589	2.988471	5	0.774651

	src known mol	similarity	smiles	mol	Class	qed	logP	SAS	rings	kinase inhibition likeliness
**2096**	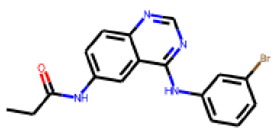	0.501520	OCC(C(N1CC)CC1)Nc1ccc2ncnc(Nc3cccc(Br)c3)c2c1	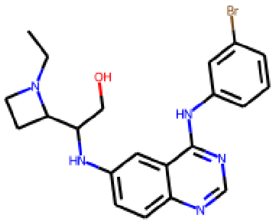	3.0	0.768132	3.608437	2.513170	4	0.789015
**2097**	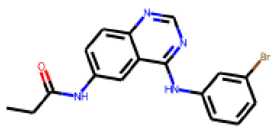	0.501520	OCC(C(N1CC)CC1)Nc1ccc2ncnc(Nc3cccc(Br)c3)c2c1	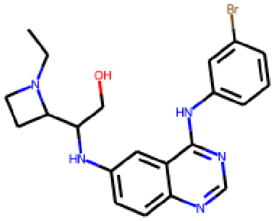	3.0	0.768132	3.608437	2.513170	4	0.789015
**2098**	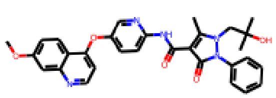	0.501072	COc1ccc2c(=O)cc(CNC(=O)c3c(C)n(C)n(-c4ccccc4)c3=O)nc3cccc2c3c1	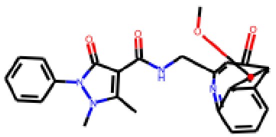	9.0	0.638163	3.995589	2.988471	5	0.774651
**2099**	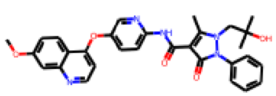	0.501072	COc1ccc2c(=O)cc(CNC(=O)c3c(C)n(C)n(-c4ccccc4)c3=O)nc3cccc2c3c1	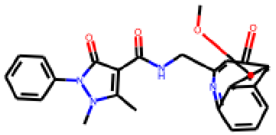	9.0	0.638163	3.995589	2.988471	5	0.774651

	src known mol	similarity	smiles	mol	Class	qed	logP	SAS	rings	kinase inhibition likeliness
**2100**	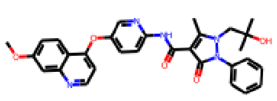	0.500283	COc1ccc2c(=O)cc(CNC(=O)c3c(C)n(C)n(-c4ccccc4)c3=O)nc3cccc3c2n1	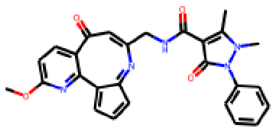	9.0	0.768132	3.608437	2.513170	5	0.823048
**2101**	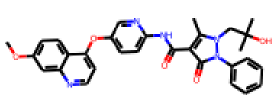	0.500283	COc1ccc2c(=O)cc(CNC(=O)c3c(C)n(C)n(-c4ccccc4)c3=O)nc3cccc3c2n1	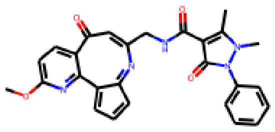	9.0	0.768132	3.608437	2.513170	5	0.823048
**2102**	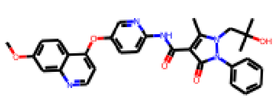	0.500283	COc1ccc2c(=O)cc(CNC(=O)c3c(C)n(C)n(-c4ccccc4)c3=O)nc3cccc3c2n1	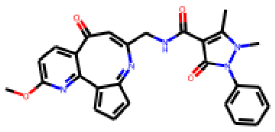	9.0	0.768132	3.608437	2.513170	5	0.823048
**2103**	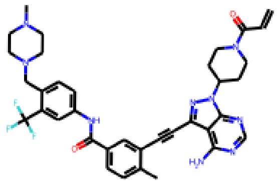	0.500000	Cc1ccc(C(=O)Nc2cccc(C(F)(C)F)c2C)cc1-c1ccc2nc(=O)ccc(C)cn2n1	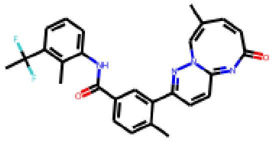	6.0	0.762994	3.476405	2.421118	4	0.764424

	src known mol	similarity	smiles	mol	Class	qed	logP	SAS	rings	kinase inhibition likeliness
**2104**	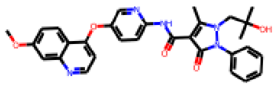	0.499731	COc1ccc2c(=O)cc(CNC(=O)c3c(C)n(C)n(-c4ccccc4)c3=O)nc3cccc2c3c1	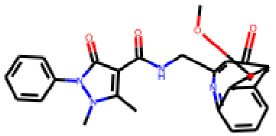	9.0	0.638163	3.995589	2.988471	5	0.774651
**2105**	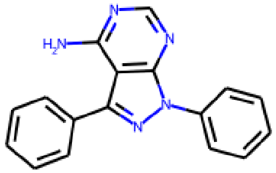	0.499675	Cc1ccc(C(=O)Oc2ccon2)cc1-c1ncnc2c1cnn2-c1ccccc1	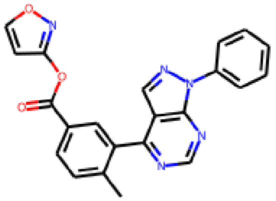	8.0	0.635315	4.542409	3.856266	5	0.760056
**2106**	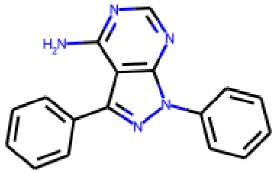	0.499339	Cc1ccc(C(=O)Oc2ccon2)cc1-c1ncnc2c1cnn2-c1ccccc1	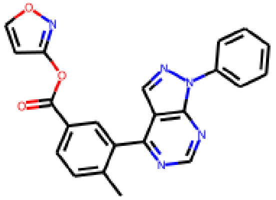	8.0	0.635315	4.542409	3.856266	5	0.760056
**2107**	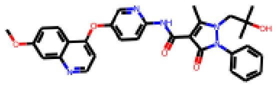	0.499155	COc1ccc2c(=O)cc(CNC(=O)c3c(C)n(C)n(-c4ccccc4)c3=O)nc3cccc3c2n1	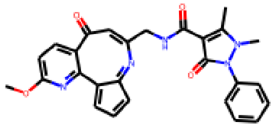	9.0	0.768132	3.608437	2.513170	5	0.823048

	src known mol	similarity	smiles	mol	Class	qed	logP	SAS	rings	kinase inhibition likeliness
**2108**	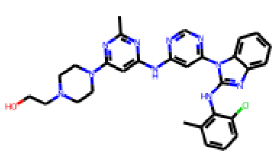	0.499056	Cc1nc(Nc2ccc(C(=O)Oc3c(C)cccc3Cl)s2)cc(N2CCNC(=O)C2=O)n1	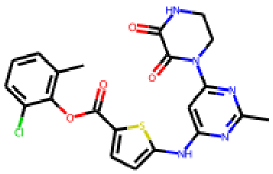	2.0	0.584673	2.312750	2.571988	4	0.770333
**2109**	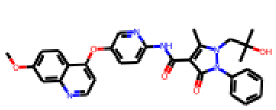	0.498922	COc1ccc2c(=O)cc(CNC(=O)c3c(C)n(C)n(-c4ccccc4)c3=O)nc3cccc2c3c1	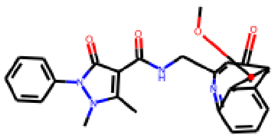	9.0	0.638163	3.995589	2.988471	5	0.774651
**2110**	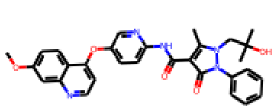	0.498922	COc1ccc2c(=O)cc(CNC(=O)c3c(C)n(C)n(-c4ccccc4)c3=O)nc3cccc2c3c1	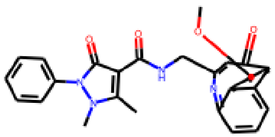	9.0	0.638163	3.995589	2.988471	5	0.774651
**2111**	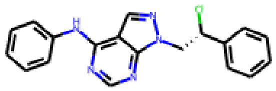	0.498062	Cc1ccc(C(=O)Nc2ccon2)cc1Nc1ncnc2c1cnn2C1cccccc1	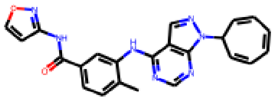	8.0	0.623362	2.525411	2.997881	5	0.770298

	src known mol	similarity	smiles	mol	Class	qed	logP	SAS	rings	kinase inhibition likeliness
**2112**	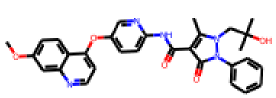	0.498030	COc1ccc2c(=O)cc(CNC(=O)c3c(C)n(C)n(-c4ccccc4)c3=O)nc3cccc3c2n1	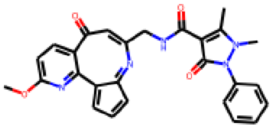	9.0	0.768132	3.608437	2.513170	5	0.823048
**2113**	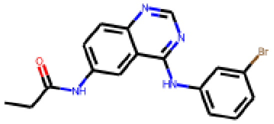	0.497901	O=C(CNC(=O)Oc1ccccc1)Nc1ccc2ncnc(Nc3cccc(Cl)c3)c2c1	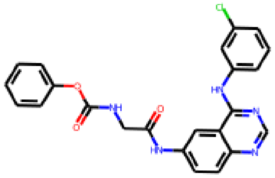	3.0	0.638163	3.995589	2.988471	4	0.793889
**2114**	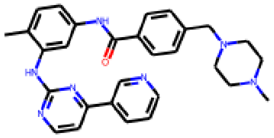	0.497861	Cc1ccc(NC(=O)c2ccccc2)cc1Nc1nccc(-c2cccnc2)n1	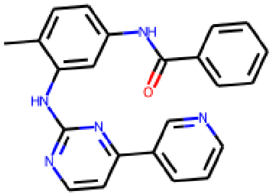	0.0	0.768132	3.608437	2.513170	4	0.854555
**2115**	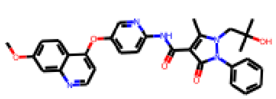	0.497856	COc1ccc2c(=O)cc(CNC(=O)c3c(C)n(C)n(-c4ccccc4)c3=O)nc3cccc2c3c1	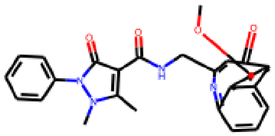	9.0	0.638163	3.995589	2.988471	5	0.774651

	src known mol	similarity	smiles	mol	Class	qed	logP	SAS	rings	kinase inhibition likeliness
**2116**	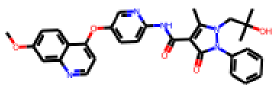	0.497856	COc1ccc2c(=O)cc(CNC(=O)c3c(C)n(C)n(-c4ccccc4)c3=O)nc3cccc2c3c1	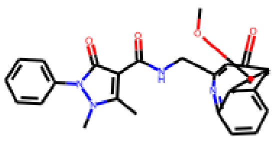	9.0	0.638163	3.995589	2.988471	5	0.774651
**2117**	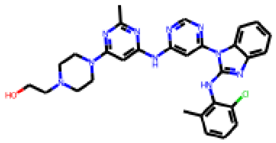	0.497769	Cc1nc(Nc2ccc(C(=O)Oc3c(C)cccc3Cl)s2)cc(N2CCNC(=O)C2=O)n1	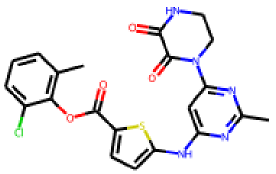	2.0	0.584673	2.312750	2.571988	4	0.770333
**2118**	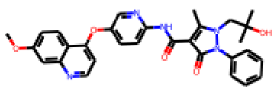	0.497750	COc1ccc2c(=O)cc(CNC(=O)c3c(C)n(C)n(-c4ccccc4)c3=O)nc3cccc3c2n1	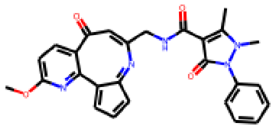	9.0	0.768132	3.608437	2.513170	5	0.823048
**2119**	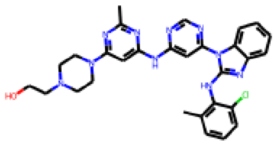	0.497726	Cc1nc(Nc2ccc(C(=O)Oc3c(C)cccc3Cl)s2)cc(N2CCNC(=O)C2=O)n1	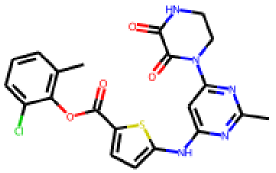	2.0	0.584673	2.312750	2.571988	4	0.770333

	src known mol	similarity	smiles	mol	Class	qed	logP	SAS	rings	kinase inhibition likeliness
**2120**	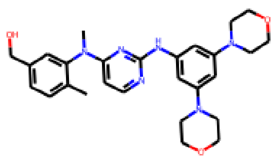	0.497545	NC(=O)c1ccccc1Nc1nc(Nc2ccc(N3CCCC3=O)cc2)ncc1Cl	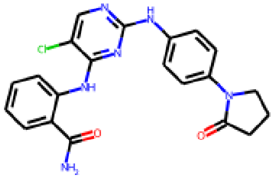	7.0	0.768132	3.608437	2.513170	4	0.773984
**2121**	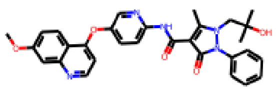	0.497091	COc1ccc2c(=O)cc(CNC(=O)c3c(C)n(C)n(-c4ccccc4)c3=O)nc3cccc2c3c1	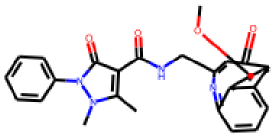	9.0	0.638163	3.995589	2.988471	5	0.774651
**2122**	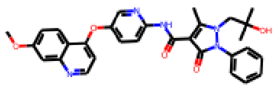	0.497079	COc1ccc2c(=O)cc(CNC(=O)c3c(C)n(C)n(-c4ccccc4)c3=O)nc3cccc2c3c1	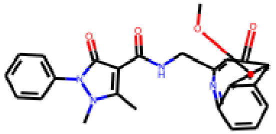	9.0	0.638163	3.995589	2.988471	5	0.774651
**2123**	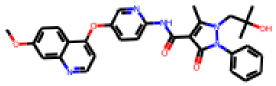	0.497076	COc1ccc2c(=O)cc(CNC(=O)c3c(C)n(C)n(-c4ccccc4)c3=O)nc3cccc3c2n1	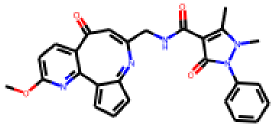	9.0	0.768132	3.608437	2.513170	5	0.823048

	src known mol	similarity	smiles	mol	Class	qed	logP	SAS	rings	kinase inhibition likeliness
**2124**	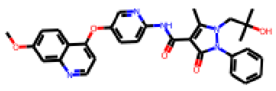	0.496929	COc1ccc2c(=O)cc(CNC(=O)c3c(C)n(C)n(-c4ccccc4)c3=O)nc3cccc3c2n1	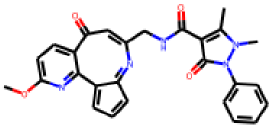	9.0	0.768132	3.608437	2.513170	5	0.823048
**2125**	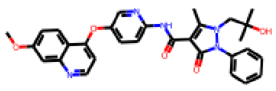	0.496901	COc1ccc2c(=O)cc(CNC(=O)c3c(C)n(C)n(-c4ccccc4)c3=O)nc3cccc3c2n1	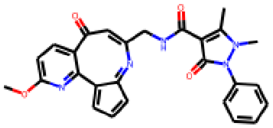	9.0	0.768132	3.608437	2.513170	5	0.823048
**2126**	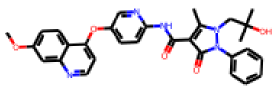	0.496901	COc1ccc2c(=O)cc(CNC(=O)c3c(C)n(C)n(-c4ccccc4)c3=O)nc3cccc3c2n1	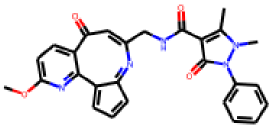	9.0	0.768132	3.608437	2.513170	5	0.823048
**2127**	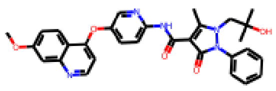	0.496901	COc1ccc2c(=O)cc(CNC(=O)c3c(C)n(C)n(-c4ccccc4)c3=O)nc3cccc3c2n1	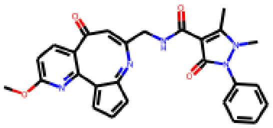	9.0	0.768132	3.608437	2.513170	5	0.823048

	src known mol	similarity	smiles	mol	Class	qed	logP	SAS	rings	kinase inhibition likeliness
**2128**	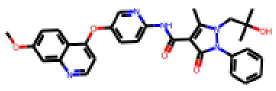	0.496901	COc1ccc2c(=O)cc(CNC(=O)c3c(C)n(C)n(-c4ccccc4)c3=O)nc3cccc3c2n1	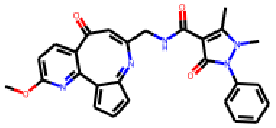	9.0	0.768132	3.608437	2.513170	5	0.823048
**2129**	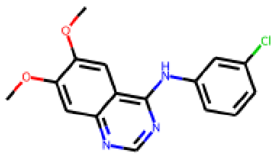	0.496676	Clc1cccc(Nc2ncnc3cc4c[nH]nc4cc23)c1	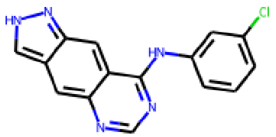	3.0	0.638163	3.995589	2.988471	4	0.781333
**2130**	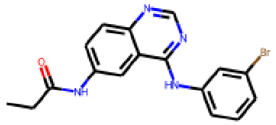	0.496634	OCC(C(N1CC)CC1)Nc1ccc2ncnc(Nc3cccc(Br)c3)c2c1	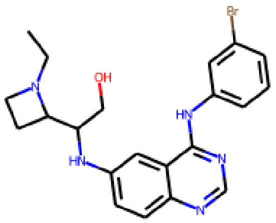	3.0	0.768132	3.608437	2.513170	4	0.789015
**2131**	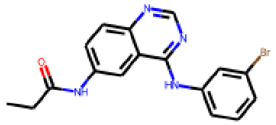	0.496634	OCC(C(N1CC)CC1)Nc1ccc2ncnc(Nc3cccc(Br)c3)c2c1	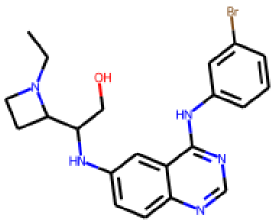	3.0	0.768132	3.608437	2.513170	4	0.789015

	src known mol	similarity	smiles	mol	Class	qed	logP	SAS	rings	kinase inhibition likeliness
**2132**	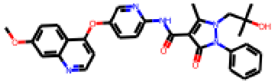	0.496245	COc1ccc2c(=O)cc(CNC(=O)c3c(C)n(C)n(-c4ccccc4)c3=O)nc3cccc2c3c1	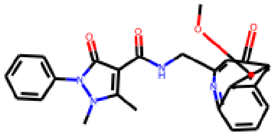	9.0	0.638163	3.995589	2.988471	5	0.774651
**2133**	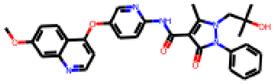	0.496245	COc1ccc2c(=O)cc(CNC(=O)c3c(C)n(C)n(-c4ccccc4)c3=O)nc3cccc2c3c1	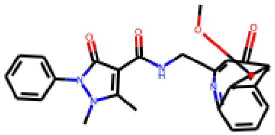	9.0	0.638163	3.995589	2.988471	5	0.774651
**2134**	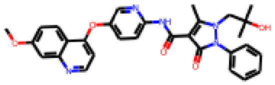	0.496059	COc1ccc2c(=O)cc(CNC(=O)c3c(C)n(C)n(-c4ccccc4)c3=O)nc3cccc3c2n1	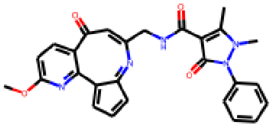	9.0	0.768132	3.608437	2.513170	5	0.823048
**2135**	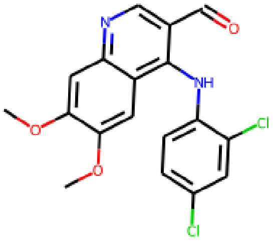	0.495805	COc1cc2ncc(C(=O)O)c(Nc3cccc(Cl)c3Cl)c2cc1OC1C(C)C1	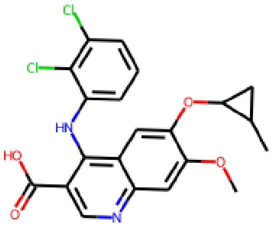	2.0	0.623362	2.525411	2.997881	4	0.774857

	src known mol	similarity	smiles	mol	Class	qed	logP	SAS	rings	kinase inhibition likeliness
**2136**	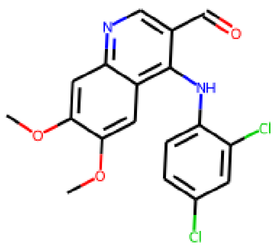	0.495633	COc1cc2ncc(C(=O)O)c(Nc3cccc(Cl)c3Cl)c2cc1OC1C(C)C1	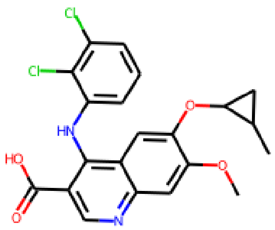	2.0	0.623362	2.525411	2.997881	4	0.774857
**2137**	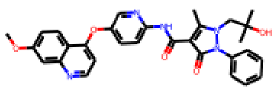	0.495480	COc1ccc2c(=O)cc(CNC(=O)c3c(C)n(C)n(-c4ccccc4)c3=O)nc3cccc3c2n1	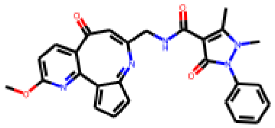	9.0	0.768132	3.608437	2.513170	5	0.823048
**2138**	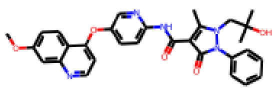	0.495480	COc1ccc2c(=O)cc(CNC(=O)c3c(C)n(C)n(-c4ccccc4)c3=O)nc3cccc3c2n1	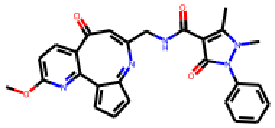	9.0	0.768132	3.608437	2.513170	5	0.823048
**2139**	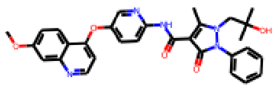	0.495182	COc1ccc2c(=O)cc(CNC(=O)c3c(C)n(C)n(-c4ccccc4)c3=O)nc3cccc2c3c1	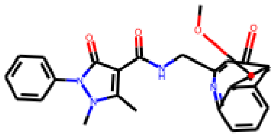	9.0	0.638163	3.995589	2.988471	5	0.774651

	src known mol	similarity	smiles	mol	Class	qed	logP	SAS	rings	kinase inhibition likeliness
**2140**	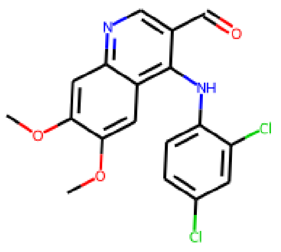	0.495116	COc1cc2ncc(C(=O)O)c(Nc3cccc(Cl)c3Cl)c2cc1OC1C(C)C1	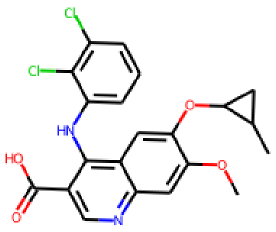	2.0	0.623362	2.525411	2.997881	4	0.774857
**2141**	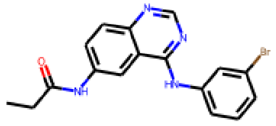	0.495042	OCC(C(N1CC)CC1)Nc1ccc2ncnc(Nc3cccc(Br)c3)c2c1	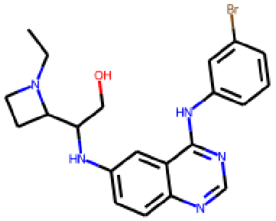	3.0	0.768132	3.608437	2.513170	4	0.789015
**2142**	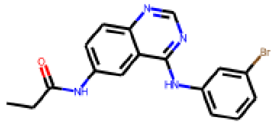	0.495042	OCC(C(N1CC)CC1)Nc1ccc2ncnc(Nc3cccc(Br)c3)c2c1	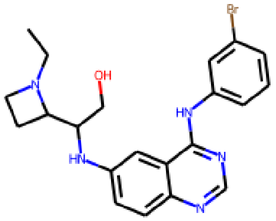	3.0	0.768132	3.608437	2.513170	4	0.789015
**2143**	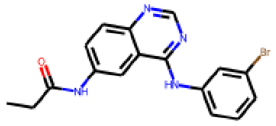	0.494845	O=C(CNC(=O)Oc1ccccc1)Nc1ccc2ncnc(Nc3cccc(Cl)c3)c2c1	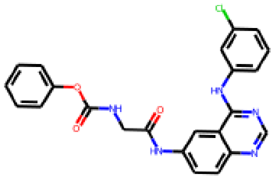	3.0	0.638163	3.995589	2.988471	4	0.793889

	src known mol	similarity	smiles	mol	Class	qed	logP	SAS	rings	kinase inhibition likeliness
**2144**	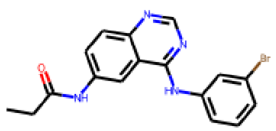	0.494681	OCC(C(N1CC)CC1)Nc1ccc2ncnc(Nc3cccc(Br)c3)c2c1	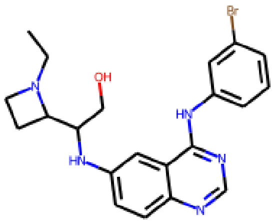	3.0	0.768132	3.608437	2.513170	4	0.789015
**2145**	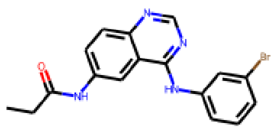	0.494681	OCC(C(N1CC)CC1)Nc1ccc2ncnc(Nc3cccc(Br)c3)c2c1	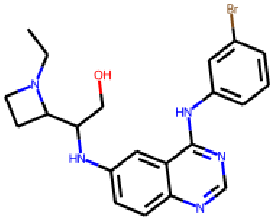	3.0	0.768132	3.608437	2.513170	4	0.789015
**2146**	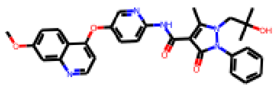	0.494647	COc1ccc2c(=O)cc(CNC(=O)c3c(C)n(C)n(-c4ccccc4)c3=O)nc3cccc2c3c1	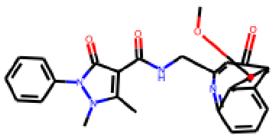	9.0	0.638163	3.995589	2.988471	5	0.774651
**2147**	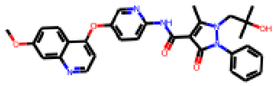	0.494647	COc1ccc2c(=O)cc(CNC(=O)c3c(C)n(C)n(-c4ccccc4)c3=O)nc3cccc2c3c1	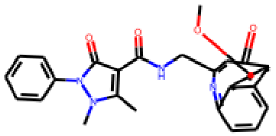	9.0	0.638163	3.995589	2.988471	5	0.774651

	src known mol	similarity	smiles	mol	Class	qed	logP	SAS	rings	kinase inhibition likeliness
**2148**	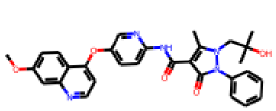	0.494321	COc1ccc2c(=O)cc(CNC(=O)c3c(C)n(C)n(-c4ccccc4)c3=O)nc3cccc2c3c1	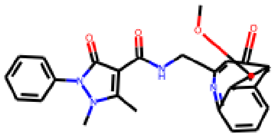	9.0	0.638163	3.995589	2.988471	5	0.774651
**2149**	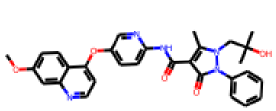	0.494321	COc1ccc2c(=O)cc(CNC(=O)c3c(C)n(C)n(-c4ccccc4)c3=O)nc3cccc2c3c1	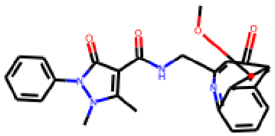	9.0	0.638163	3.995589	2.988471	5	0.774651
**2150**	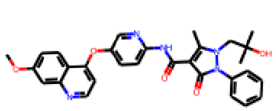	0.494321	COc1ccc2c(=O)cc(CNC(=O)c3c(C)n(C)n(-c4ccccc4)c3=O)nc3cccc2c3c1	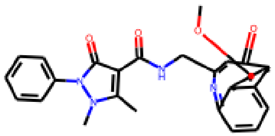	9.0	0.638163	3.995589	2.988471	5	0.774651
**2151**	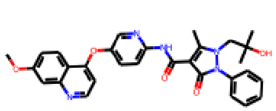	0.494321	COc1ccc2c(=O)cc(CNC(=O)c3c(C)n(C)n(-c4ccccc4)c3=O)nc3cccc2c3c1	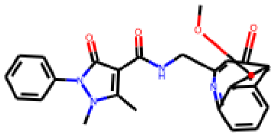	9.0	0.638163	3.995589	2.988471	5	0.774651

	src known mol	similarity	smiles	mol	Class	qed	logP	SAS	rings	kinase inhibition likeliness
**2152**	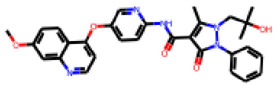	0.494292	COc1ccc2c(=O)cc(CNC(=O)c3c(C)n(C)n(-c4ccccc4)c3=O)nc3cccc3c2n1	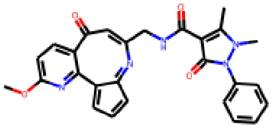	9.0	0.768132	3.608437	2.513170	5	0.823048
**2153**	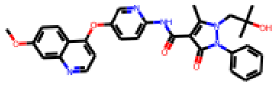	0.494111	COc1ccc2c(=O)cc(CNC(=O)c3c(C)n(C)n(-c4ccccc4)c3=O)nc3cccc3c2n1	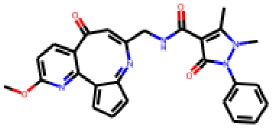	9.0	0.768132	3.608437	2.513170	5	0.823048
**2154**	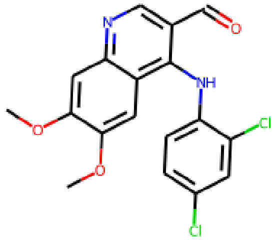	0.493958	COc1cc2ncc(C(=O)O)c(Nc3cccc(Cl)c3Cl)c2cc1OC1C(C)C1	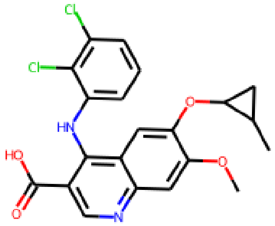	2.0	0.623362	2.525411	2.997881	4	0.774857
**2155**	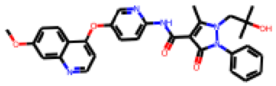	0.493801	COc1ccc2c(=O)cc(CNC(=O)c3c(C)n(C)n(-c4ccccc4)c3=O)nc3cccc2c3c1	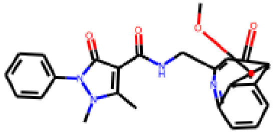	9.0	0.638163	3.995589	2.988471	5	0.774651

	src known mol	similarity	smiles	mol	Class	qed	logP	SAS	rings	kinase inhibition likeliness
**2156**	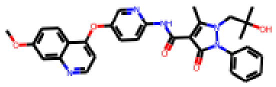	0.493780	COc1ccc2c(=O)cc(CNC(=O)c3c(C)n(C)n(-c4ccccc4)c3=O)nc3cccc2c3c1	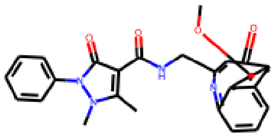	9.0	0.638163	3.995589	2.988471	5	0.774651
**2157**	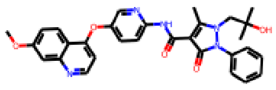	0.493778	COc1ccc2c(=O)cc(CNC(=O)c3c(C)n(C)n(-c4ccccc4)c3=O)nc3cccc3c2n1	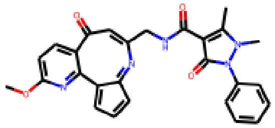	9.0	0.768132	3.608437	2.513170	5	0.823048
**2158**	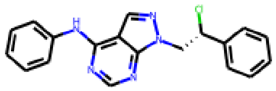	0.493652	Cc1ccc(C(=O)Nc2ccon2)cc1Nc1ncnc2c1cnn2C1cccccc1	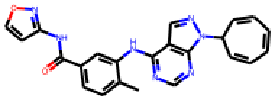	8.0	0.623362	2.525411	2.997881	5	0.770298
**2159**	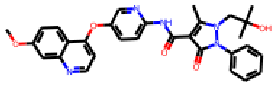	0.493333	COc1ccc2c(=O)cc(CNC(=O)c3c(C)n(C)n(-c4ccccc4)c3=O)nc3cccc2c3c1	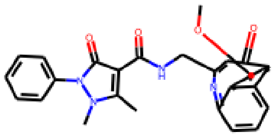	9.0	0.638163	3.995589	2.988471	5	0.774651

	src known mol	similarity	smiles	mol	Class	qed	logP	SAS	rings	kinase inhibition likeliness
**2160**	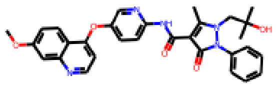	0.493333	COc1ccc2c(=O)cc(CNC(=O)c3c(C)n(C)n(-c4ccccc4)c3=O)nc3cccc2c3c1	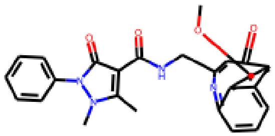	9.0	0.638163	3.995589	2.988471	5	0.774651
**2161**	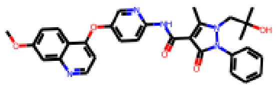	0.493333	COc1ccc2c(=O)cc(CNC(=O)c3c(C)n(C)n(-c4ccccc4)c3=O)nc3cccc2c3c1	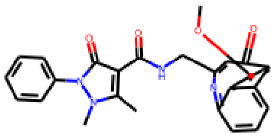	9.0	0.638163	3.995589	2.988471	5	0.774651
**2162**	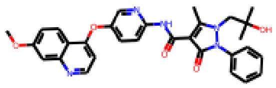	0.493269	COc1ccc2c(=O)cc(CNC(=O)c3c(C)n(C)n(-c4ccccc4)c3=O)nc3cccc2c3c1	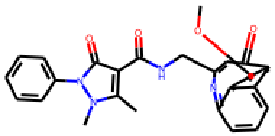	9.0	0.638163	3.995589	2.988471	5	0.774651
**2163**	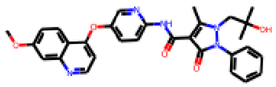	0.493269	COc1ccc2c(=O)cc(CNC(=O)c3c(C)n(C)n(-c4ccccc4)c3=O)nc3cccc2c3c1	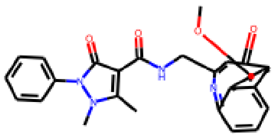	9.0	0.638163	3.995589	2.988471	5	0.774651

	src known mol	similarity	smiles	mol	Class	qed	logP	SAS	rings	kinase inhibition likeliness
**2164**	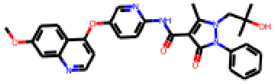	0.493236	COc1ccc2c(=O)cc(CNC(=O)c3c(C)n(C)n(-c4ccccc4)c3=O)nc3cccc3c2n1	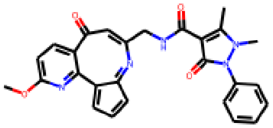	9.0	0.768132	3.608437	2.513170	5	0.823048
**2165**	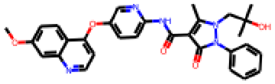	0.493236	COc1ccc2c(=O)cc(CNC(=O)c3c(C)n(C)n(-c4ccccc4)c3=O)nc3cccc3c2n1	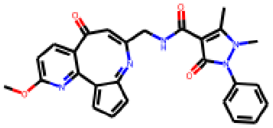	9.0	0.768132	3.608437	2.513170	5	0.823048
**2166**	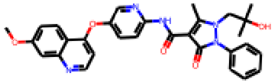	0.493220	COc1ccc2c(=O)cc(CNC(=O)c3c(C)n(C)n(-c4ccccc4)c3=O)nc3cccc3c2n1	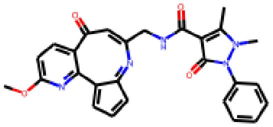	9.0	0.768132	3.608437	2.513170	5	0.823048
**2167**	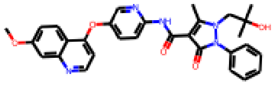	0.493220	COc1ccc2c(=O)cc(CNC(=O)c3c(C)n(C)n(-c4ccccc4)c3=O)nc3cccc3c2n1	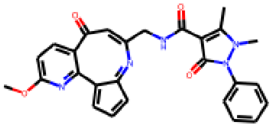	9.0	0.768132	3.608437	2.513170	5	0.823048

	src known mol	similarity	smiles	mol	Class	qed	logP	SAS	rings	kinase inhibition likeliness
**2168**	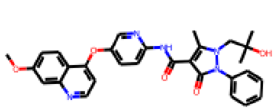	0.493070	COc1ccc2c(=O)cc(CNC(=O)c3c(C)n(C)n(-c4ccccc4)c3=O)nc3cccc2c3c1	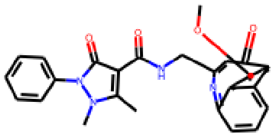	9.0	0.638163	3.995589	2.988471	5	0.774651
**2169**	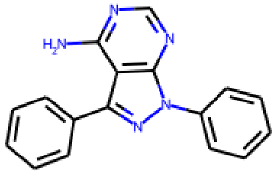	0.492881	Cc1ccc(C(=O)Oc2ccon2)cc1-c1ncnc2c1cnn2-c1ccccc1	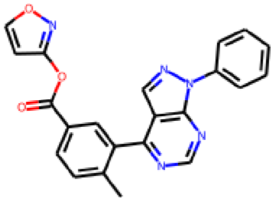	8.0	0.635315	4.542409	3.856266	5	0.760056
**2170**	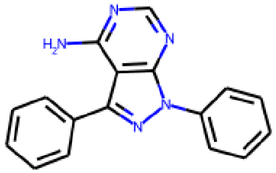	0.492881	Cc1ccc(C(=O)Oc2ccon2)cc1-c1ncnc2c1cnn2-c1ccccc1	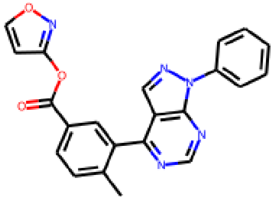	8.0	0.635315	4.542409	3.856266	5	0.760056
**2171**	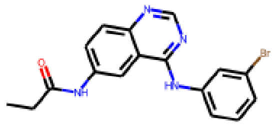	0.492731	OCC(C(N1CC)CC1)Nc1ccc2ncnc(Nc3cccc(Br)c3)c2c1	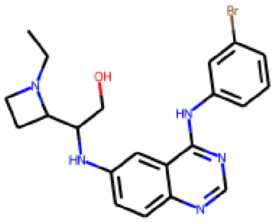	3.0	0.768132	3.608437	2.513170	4	0.789015

	src known mol	similarity	smiles	mol	Class	qed	logP	SAS	rings	kinase inhibition likeliness
**2172**	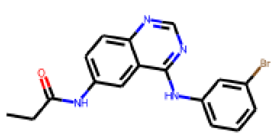	0.492731	OCC(C(N1CC)CC1)Nc1ccc2ncnc(Nc3cccc(Br)c3)c2c1	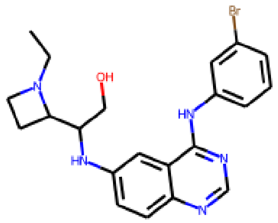	3.0	0.768132	3.608437	2.513170	4	0.789015
**2173**	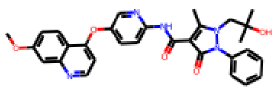	0.492441	COc1ccc2c(=O)cc(CNC(=O)c3c(C)n(C)n(-c4ccccc4)c3=O)nc3cccc2c3c1	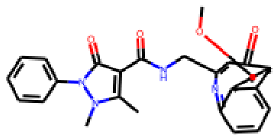	9.0	0.638163	3.995589	2.988471	5	0.774651
**2174**	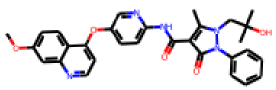	0.492441	COc1ccc2c(=O)cc(CNC(=O)c3c(C)n(C)n(-c4ccccc4)c3=O)nc3cccc2c3c1	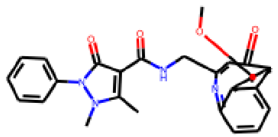	9.0	0.638163	3.995589	2.988471	5	0.774651
**2175**	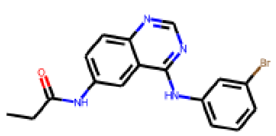	0.492063	OCC(C(N1CC)CC1)Nc1ccc2ncnc(Nc3cccc(Br)c3)c2c1	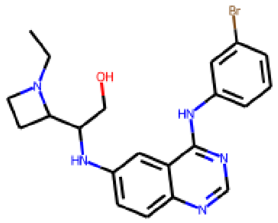	3.0	0.768132	3.608437	2.513170	4	0.789015

	src known mol	similarity	smiles	mol	Class	qed	logP	SAS	rings	kinase inhibition likeliness
**2176**	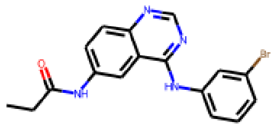	0.492063	OCC(C(N1CC)CC1)Nc1ccc2ncnc(Nc3cccc(Br)c3)c2c1	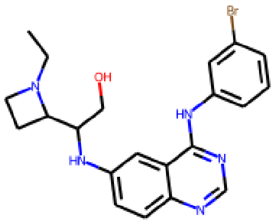	3.0	0.768132	3.608437	2.513170	4	0.789015
**2177**	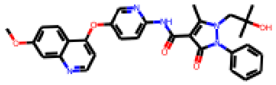	0.492054	COc1ccc2c(=O)cc(CNC(=O)c3c(C)n(C)n(-c4ccccc4)c3=O)nc3cccc3c2n1	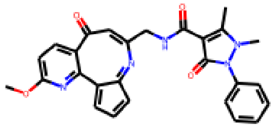	9.0	0.768132	3.608437	2.513170	5	0.823048
**2178**	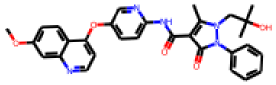	0.492054	COc1ccc2c(=O)cc(CNC(=O)c3c(C)n(C)n(-c4ccccc4)c3=O)nc3cccc3c2n1	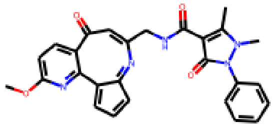	9.0	0.768132	3.608437	2.513170	5	0.823048
**2179**	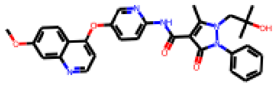	0.492054	COc1ccc2c(=O)cc(CNC(=O)c3c(C)n(C)n(-c4ccccc4)c3=O)nc3cccc3c2n1	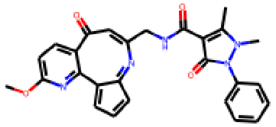	9.0	0.768132	3.608437	2.513170	5	0.823048

	src known mol	similarity	smiles	mol	Class	qed	logP	SAS	rings	kinase inhibition likeliness
**2180**	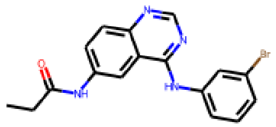	0.492015	OCC(C(N1CC)CC1)Nc1ccc2ncnc(Nc3cccc(Br)c3)c2c1	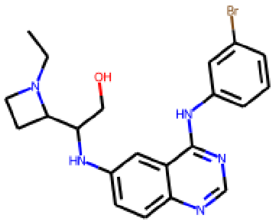	3.0	0.768132	3.608437	2.513170	4	0.789015
**2181**	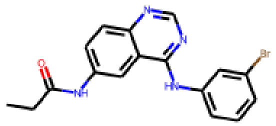	0.492015	OCC(C(N1CC)CC1)Nc1ccc2ncnc(Nc3cccc(Br)c3)c2c1	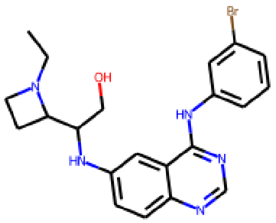	3.0	0.768132	3.608437	2.513170	4	0.789015
**2182**	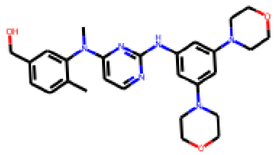	0.491539	NC(=O)c1ccccc1Nc1nc(Nc2ccc(N3CCCC3=O)cc2)ncc1Cl	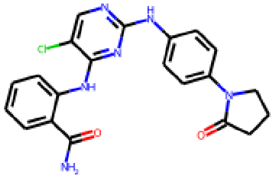	7.0	0.768132	3.608437	2.513170	4	0.773984
**2183**	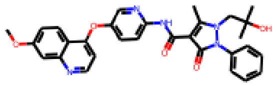	0.491317	COc1ccc2c(=O)cc(CNC(=O)c3c(C)n(C)n(-c4ccccc4)c3=O)nc3cccc3c2n1	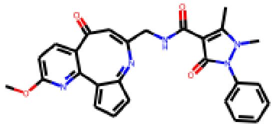	9.0	0.768132	3.608437	2.513170	5	0.823048

	src known mol	similarity	smiles	mol	Class	qed	logP	SAS	rings	kinase inhibition likeliness
**2184**	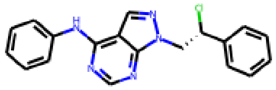	0.491228	Cc1ccc(C(=O)Nc2ccon2)cc1Nc1ncnc2c1cnn2C1cccccc1	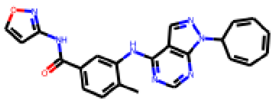	8.0	0.623362	2.525411	2.997881	5	0.770298
**2185**	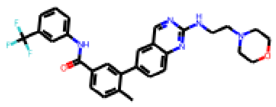	0.491150	Cc1ccc(C(=O)Nc2cccc(C(F)(F)F)c2)cc1-c1ccc2cc(N)ncc2c1	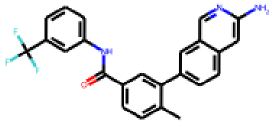	6.0	0.761557	4.954058	3.852993	4	0.750321
**2186**	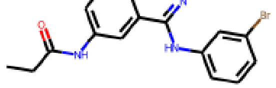	0.491085	OCC(C(N1CC)CC1)Nc1ccc2ncnc(Nc3cccc(Br)c3)c2c1	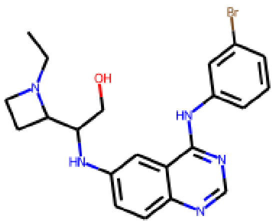	3.0	0.768132	3.608437	2.513170	4	0.789015
**2187**	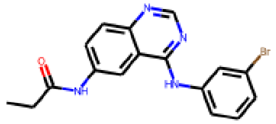	0.491085	OCC(C(N1CC)CC1)Nc1ccc2ncnc(Nc3cccc(Br)c3)c2c1	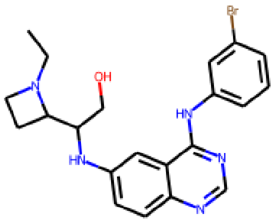	3.0	0.768132	3.608437	2.513170	4	0.789015

	src known mol	similarity	smiles	mol	Class	qed	logP	SAS	rings	kinase inhibition likeliness
**2188**	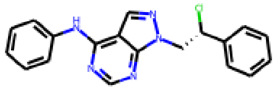	0.491010	Cc1ccc(C(=O)Nc2ccon2)cc1Nc1ncnc2c1cnn2C1cccccc1	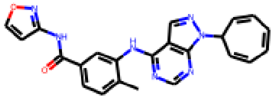	8.0	0.623362	2.525411	2.997881	5	0.770298
**2189**	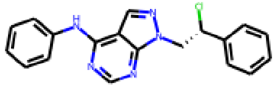	0.491010	Cc1ccc(C(=O)Nc2ccon2)cc1Nc1ncnc2c1cnn2C1cccccc1	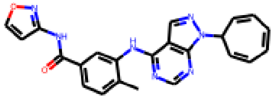	8.0	0.623362	2.525411	2.997881	5	0.770298
**2190**	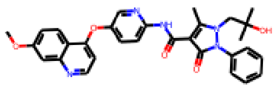	0.490928	COc1ccc2c(=O)cc(CNC(=O)c3c(C)n(C)n(-c4ccccc4)c3=O)nc3cccc2c3c1	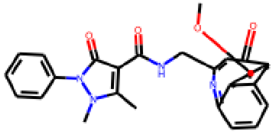	9.0	0.638163	3.995589	2.988471	5	0.774651
**2191**	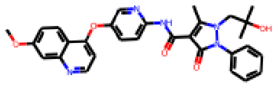	0.490860	COc1ccc2c(=O)cc(CNC(=O)c3c(C)n(C)n(-c4ccccc4)c3=O)nc3cccc2c3c1	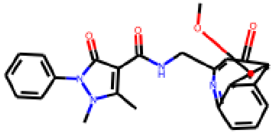	9.0	0.638163	3.995589	2.988471	5	0.774651

	src known mol	similarity	smiles	mol	Class	qed	logP	SAS	rings	kinase inhibition likeliness
**2192**	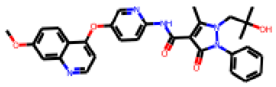	0.490673	COc1ccc2c(=O)cc(CNC(=O)c3c(C)n(C)n(-c4ccccc4)c3=O)nc3cccc3c2n1	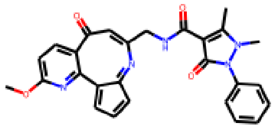	9.0	0.768132	3.608437	2.513170	5	0.823048
**2193**	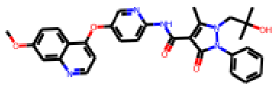	0.490673	COc1ccc2c(=O)cc(CNC(=O)c3c(C)n(C)n(-c4ccccc4)c3=O)nc3cccc3c2n1	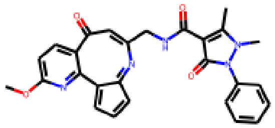	9.0	0.768132	3.608437	2.513170	5	0.823048
**2194**	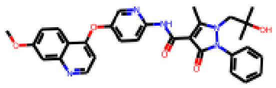	0.490673	COc1ccc2c(=O)cc(CNC(=O)c3c(C)n(C)n(-c4ccccc4)c3=O)nc3cccc3c2n1	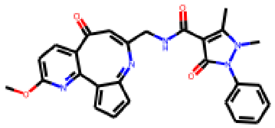	9.0	0.768132	3.608437	2.513170	5	0.823048
**2195**	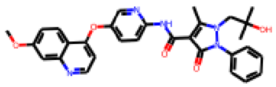	0.490673	COc1ccc2c(=O)cc(CNC(=O)c3c(C)n(C)n(-c4ccccc4)c3=O)nc3cccc3c2n1	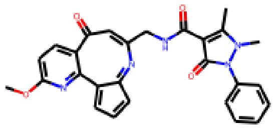	9.0	0.768132	3.608437	2.513170	5	0.823048

	src known mol	similarity	smiles	mol	Class	qed	logP	SAS	rings	kinase inhibition likeliness
**2196**	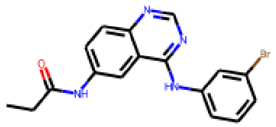	0.490651	OCC(C(N1CC)CC1)Nc1ccc2ncnc(Nc3cccc(Br)c3)c2c1	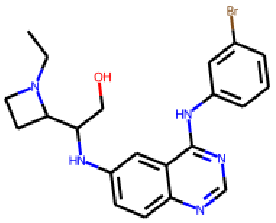	3.0	0.768132	3.608437	2.513170	4	0.789015
**2197**	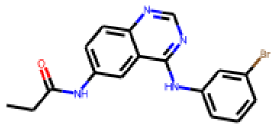	0.490651	OCC(C(N1CC)CC1)Nc1ccc2ncnc(Nc3cccc(Br)c3)c2c1	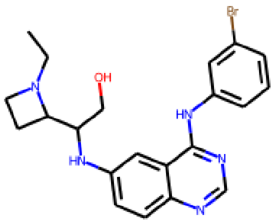	3.0	0.768132	3.608437	2.513170	4	0.789015
**2198**	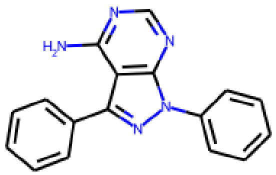	0.490617	Cc1ccc(C(=O)Oc2ccon2)cc1-c1ncnc2c1cnn2-c1ccccc1	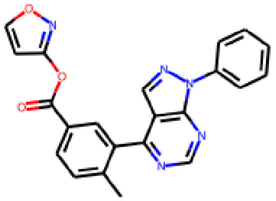	8.0	0.635315	4.542409	3.856266	5	0.760056
**2199**	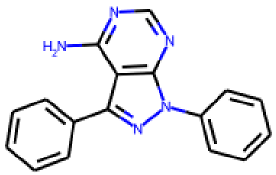	0.490617	Cc1ccc(C(=O)Oc2ccon2)cc1-c1ncnc2c1cnn2-c1ccccc1	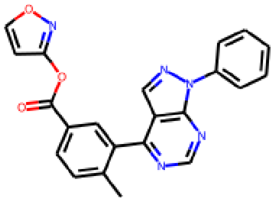	8.0	0.635315	4.542409	3.856266	5	0.760056

	src known mol	similarity	smiles	mol	Class	qed	logP	SAS	rings	kinase inhibition likeliness
**2200**	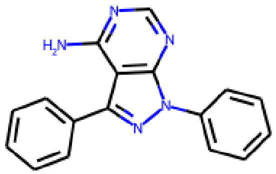	0.490617	Cc1ccc(C(=O)Oc2ccon2)cc1-c1ncnc2c1cnn2-c1ccccc1	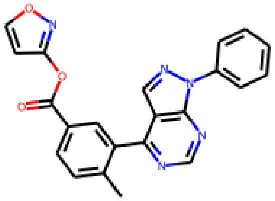	8.0	0.635315	4.542409	3.856266	5	0.760056
**2201**	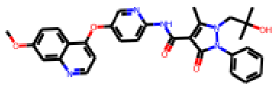	0.490556	COc1ccc2c(=O)cc(CNC(=O)c3c(C)n(C)n(-c4ccccc4)c3=O)nc3cccc2c3c1	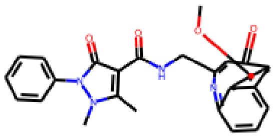	9.0	0.638163	3.995589	2.988471	5	0.774651
**2202**	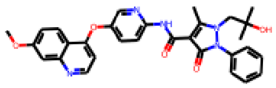	0.490556	COc1ccc2c(=O)cc(CNC(=O)c3c(C)n(C)n(-c4ccccc4)c3=O)nc3cccc2c3c1	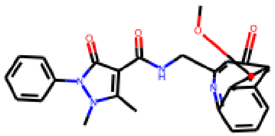	9.0	0.638163	3.995589	2.988471	5	0.774651
**2203**	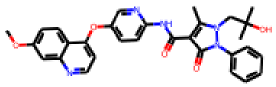	0.490556	COc1ccc2c(=O)cc(CNC(=O)c3c(C)n(C)n(-c4ccccc4)c3=O)nc3cccc2c3c1	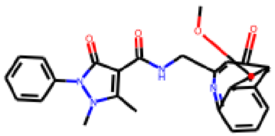	9.0	0.638163	3.995589	2.988471	5	0.774651

	src known mol	similarity	smiles	mol	Class	qed	logP	SAS	rings	kinase inhibition likeliness
**2204**	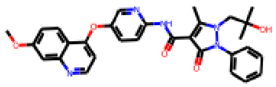	0.490556	COc1ccc2c(=O)cc(CNC(=O)c3c(C)n(C)n(-c4ccccc4)c3=O)nc3cccc2c3c1	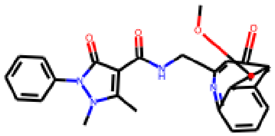	9.0	0.638163	3.995589	2.988471	5	0.774651
**2205**	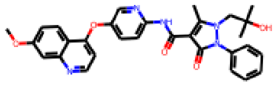	0.490152	COc1ccc2c(=O)cc(CNC(=O)c3c(C)n(C)n(-c4ccccc4)c3=O)nc3cccc3c2n1	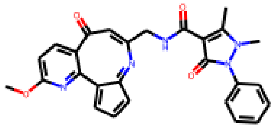	9.0	0.768132	3.608437	2.513170	5	0.823048
**2206**	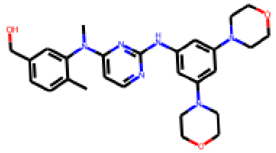	0.490103	NC(=O)c1ccccc1Nc1nc(Nc2ccc(N3CCCC3=O)cc2)ncc1Cl	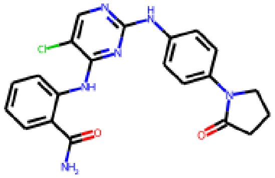	7.0	0.768132	3.608437	2.513170	4	0.773984
**2207**	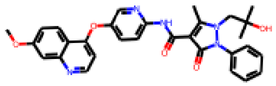	0.490096	COc1ccc2c(=O)cc(CNC(=O)c3c(C)n(C)n(-c4ccccc4)c3=O)nc3cccc3c2n1	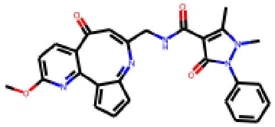	9.0	0.768132	3.608437	2.513170	5	0.823048

	src known mol	similarity	smiles	mol	Class	qed	logP	SAS	rings	kinase inhibition likeliness
**2208**	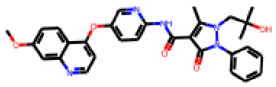	0.490096	COc1ccc2c(=O)cc(CNC(=O)c3c(C)n(C)n(-c4ccccc4)c3=O)nc3cccc3c2n1	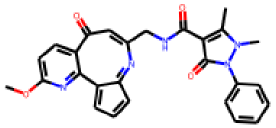	9.0	0.768132	3.608437	2.513170	5	0.823048
**2209**	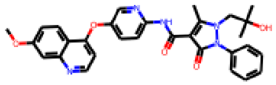	0.490096	COc1ccc2c(=O)cc(CNC(=O)c3c(C)n(C)n(-c4ccccc4)c3=O)nc3cccc3c2n1	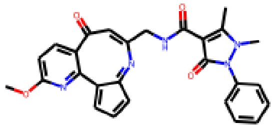	9.0	0.768132	3.608437	2.513170	5	0.823048
**2210**	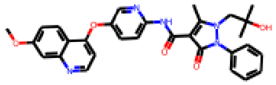	0.490070	COc1ccc2c(=O)cc(CNC(=O)c3c(C)n(C)n(-c4ccccc4)c3=O)nc3cccc2c3c1	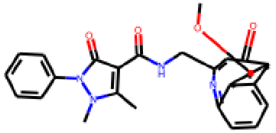	9.0	0.638163	3.995589	2.988471	5	0.774651
**2211**	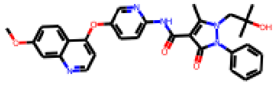	0.489984	COc1ccc2c(=O)cc(CNC(=O)c3c(C)n(C)n(-c4ccccc4)c3=O)nc3cccc2c3c1	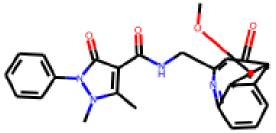	9.0	0.638163	3.995589	2.988471	5	0.774651

	src known mol	similarity	smiles	mol	Class	qed	logP	SAS	rings	kinase inhibition likeliness
**2212**	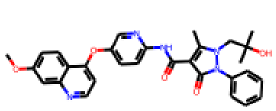	0.489984	COc1ccc2c(=O)cc(CNC(=O)c3c(C)n(C)n(-c4ccccc4)c3=O)nc3cccc2c3c1	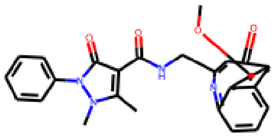	9.0	0.638163	3.995589	2.988471	5	0.774651
**2213**	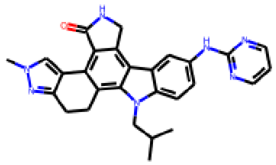	0.489707	C#Cc1ccc(C(=O)OC)cc1-c1C(=O)c-n2nc(-c3ccc(F)cc3F)cc2C1	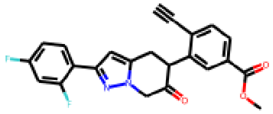	8.0	0.768132	3.608437	2.513170	4	0.763730
**2214**	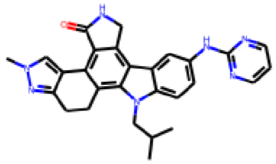	0.489707	C#Cc1ccc(C(=O)OC)cc1-c1C(=O)c-n2nc(-c3ccc(F)cc3F)cc2C1	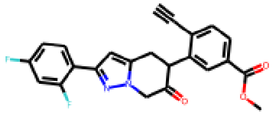	8.0	0.768132	3.608437	2.513170	4	0.763730
**2215**	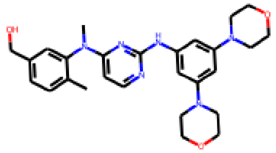	0.489617	NC(=O)c1ccccc1Nc1nc(Nc2ccc(N3CCCC3=O)cc2)ncc1Cl	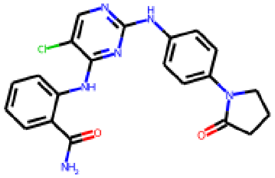	7.0	0.768132	3.608437	2.513170	4	0.773984

	src known mol	similarity	smiles	mol	Class	qed	logP	SAS	rings	kinase inhibition likeliness
**2216**	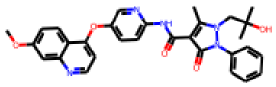	0.489542	COc1ccc2c(=O)cc(CNC(=O)c3c(C)n(C)n(-c4ccccc4)c3=O)nc3cccc3c2n1	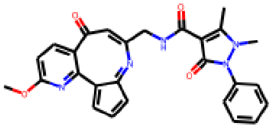	9.0	0.768132	3.608437	2.513170	5	0.823048
**2217**	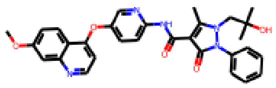	0.489542	COc1ccc2c(=O)cc(CNC(=O)c3c(C)n(C)n(-c4ccccc4)c3=O)nc3cccc3c2n1	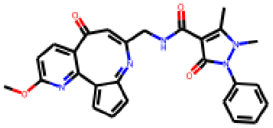	9.0	0.768132	3.608437	2.513170	5	0.823048
**2218**	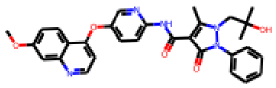	0.489542	COc1ccc2c(=O)cc(CNC(=O)c3c(C)n(C)n(-c4ccccc4)c3=O)nc3cccc3c2n1	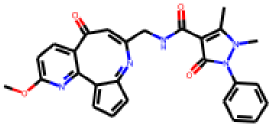	9.0	0.768132	3.608437	2.513170	5	0.823048
**2219**	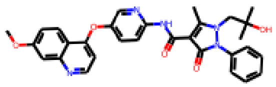	0.489407	COc1ccc2c(=O)cc(CNC(=O)c3c(C)n(C)n(-c4ccccc4)c3=O)nc3cccc2c3c1	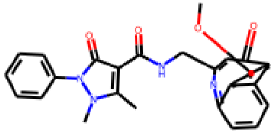	9.0	0.638163	3.995589	2.988471	5	0.774651

	src known mol	similarity	smiles	mol	Class	qed	logP	SAS	rings	kinase inhibition likeliness
**2220**	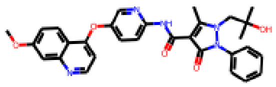	0.489290	COc1ccc2c(=O)cc(CNC(=O)c3c(C)n(C)n(-c4ccccc4)c3=O)nc3cccc3c2n1	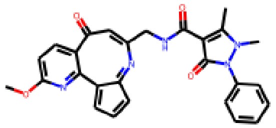	9.0	0.768132	3.608437	2.513170	5	0.823048
**2221**	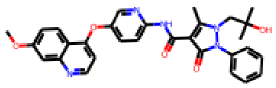	0.488952	COc1ccc2c(=O)cc(CNC(=O)c3c(C)n(C)n(-c4ccccc4)c3=O)nc3cccc3c2n1	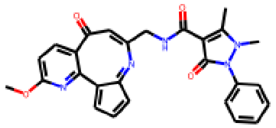	9.0	0.768132	3.608437	2.513170	5	0.823048
**2222**	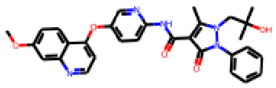	0.488952	COc1ccc2c(=O)cc(CNC(=O)c3c(C)n(C)n(-c4ccccc4)c3=O)nc3cccc3c2n1	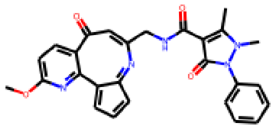	9.0	0.768132	3.608437	2.513170	5	0.823048
**2223**	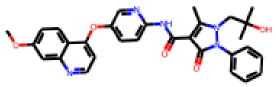	0.488925	COc1ccc2c(=O)cc(CNC(=O)c3c(C)n(C)n(-c4ccccc4)c3=O)nc3cccc2c3c1	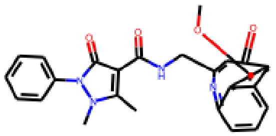	9.0	0.638163	3.995589	2.988471	5	0.774651

	src known mol	similarity	smiles	mol	Class	qed	logP	SAS	rings	kinase inhibition likeliness
**2224**	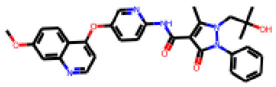	0.488925	COc1ccc2c(=O)cc(CNC(=O)c3c(C)n(C)n(-c4ccccc4)c3=O)nc3cccc2c3c1	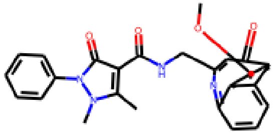	9.0	0.638163	3.995589	2.988471	5	0.774651
**2225**	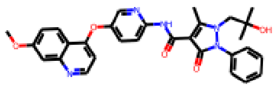	0.488698	COc1ccc2c(=O)cc(CNC(=O)c3c(C)n(C)n(-c4ccccc4)c3=O)nc3cccc2c3c1	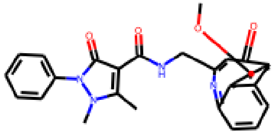	9.0	0.638163	3.995589	2.988471	5	0.774651
**2226**	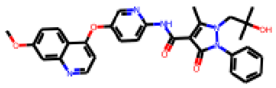	0.488587	COc1ccc2c(=O)cc(CNC(=O)c3c(C)n(C)n(-c4ccccc4)c3=O)nc3cccc2c3c1	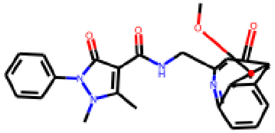	9.0	0.638163	3.995589	2.988471	5	0.774651
**2227**	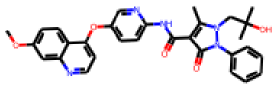	0.488347	COc1ccc2c(=O)cc(CNC(=O)c3c(C)n(C)n(-c4ccccc4)c3=O)nc3cccc2c3c1	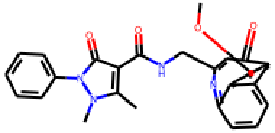	9.0	0.638163	3.995589	2.988471	5	0.774651

	src known mol	similarity	smiles	mol	Class	qed	logP	SAS	rings	kinase inhibition likeliness
**2228**	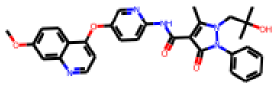	0.488013	COc1ccc2c(=O)cc(CNC(=O)c3c(C)n(C)n(-c4ccccc4)c3=O)nc3cccc2c3c1	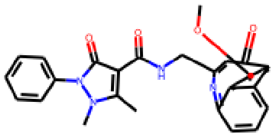	9.0	0.638163	3.995589	2.988471	5	0.774651
**2229**	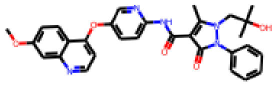	0.487737	COc1ccc2c(=O)cc(CNC(=O)c3c(C)n(C)n(-c4ccccc4)c3=O)nc3cccc3c2n1	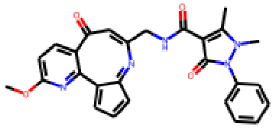	9.0	0.768132	3.608437	2.513170	5	0.823048
**2230**	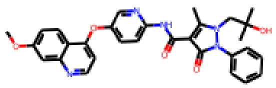	0.487599	COc1ccc2c(=O)cc(CNC(=O)c3c(C)n(C)n(-c4ccccc4)c3=O)nc3cccc2c3c1	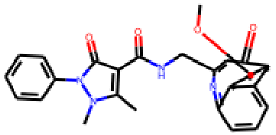	9.0	0.638163	3.995589	2.988471	5	0.774651
**2231**	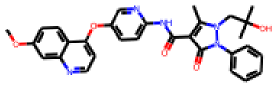	0.487599	COc1ccc2c(=O)cc(CNC(=O)c3c(C)n(C)n(-c4ccccc4)c3=O)nc3cccc3c2n1	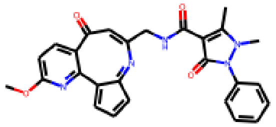	9.0	0.768132	3.608437	2.513170	5	0.823048

	src known mol	similarity	smiles	mol	Class	qed	logP	SAS	rings	kinase inhibition likeliness
**2232**	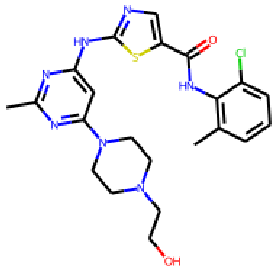	0.487491	Cc1nc(Nc2ccc(C(=O)Oc3c(C)cccc3Cl)s2)cc(N2CCNC(C)c2C)n1	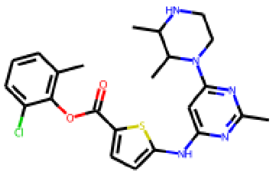	2.0	0.776263	3.686558	0.949744	4	0.784833
**2233**	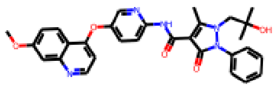	0.487453	COc1ccc2c(=O)cc(CNC(=O)c3c(C)n(C)n(-c4ccccc4)c3=O)nc3cccc2c3c1	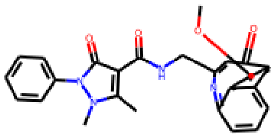	9.0	0.638163	3.995589	2.988471	5	0.774651
**2234**	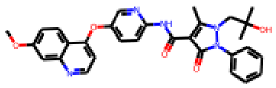	0.487288	COc1ccc2c(=O)cc(CNC(=O)c3c(C)n(C)n(-c4ccccc4)c3=O)nc3cccc2c3c1	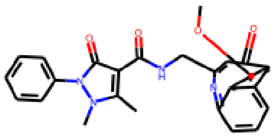	9.0	0.638163	3.995589	2.988471	5	0.774651
**2235**	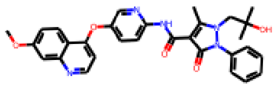	0.487288	COc1ccc2c(=O)cc(CNC(=O)c3c(C)n(C)n(-c4ccccc4)c3=O)nc3cccc2c3c1	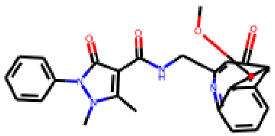	9.0	0.638163	3.995589	2.988471	5	0.774651

	src known mol	similarity	smiles	mol	Class	qed	logP	SAS	rings	kinase inhibition likeliness
**2236**	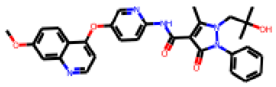	0.487281	COc1ccc2c(=O)cc(CNC(=O)c3c(C)n(C)n(-c4ccccc4)c3=O)nc3cccc3c2n1	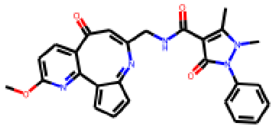	9.0	0.768132	3.608437	2.513170	5	0.823048
**2237**	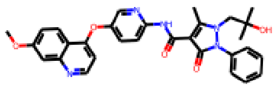	0.487281	COc1ccc2c(=O)cc(CNC(=O)c3c(C)n(C)n(-c4ccccc4)c3=O)nc3cccc3c2n1	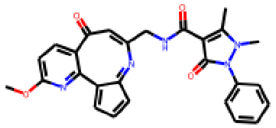	9.0	0.768132	3.608437	2.513170	5	0.823048
**2238**	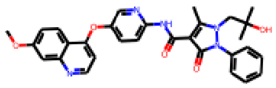	0.487252	COc1ccc2c(=O)cc(CNC(=O)c3c(C)n(C)n(-c4ccccc4)c3=O)nc3cccc3c2n1	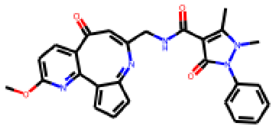	9.0	0.768132	3.608437	2.513170	5	0.823048
**2239**	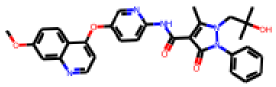	0.487252	COc1ccc2c(=O)cc(CNC(=O)c3c(C)n(C)n(-c4ccccc4)c3=O)nc3cccc3c2n1	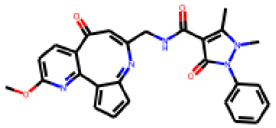	9.0	0.768132	3.608437	2.513170	5	0.823048

	src known mol	similarity	smiles	mol	Class	qed	logP	SAS	rings	kinase inhibition likeliness
**2240**	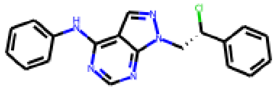	0.487162	Cc1ccc(C(=O)Nc2ccon2)cc1Nc1ncnc2c1cnn2C1cccccc1	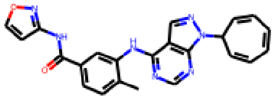	8.0	0.623362	2.525411	2.997881	5	0.770298
**2241**	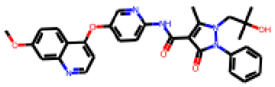	0.486961	COc1ccc2c(=O)cc(CNC(=O)c3c(C)n(C)n(-c4ccccc4)c3=O)nc3cccc3c2n1	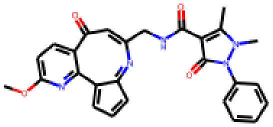	9.0	0.768132	3.608437	2.513170	5	0.823048
**2242**	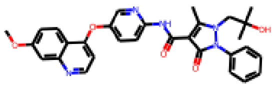	0.486961	COc1ccc2c(=O)cc(CNC(=O)c3c(C)n(C)n(-c4ccccc4)c3=O)nc3cccc3c2n1	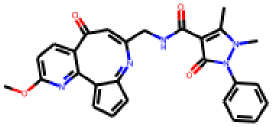	9.0	0.768132	3.608437	2.513170	5	0.823048
**2243**	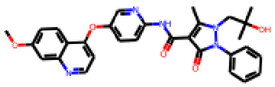	0.486961	COc1ccc2c(=O)cc(CNC(=O)c3c(C)n(C)n(-c4ccccc4)c3=O)nc3cccc3c2n1	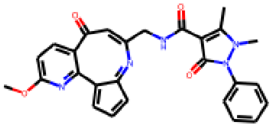	9.0	0.768132	3.608437	2.513170	5	0.823048

	src known mol	similarity	smiles	mol	Class	qed	logP	SAS	rings	kinase inhibition likeliness
**2244**	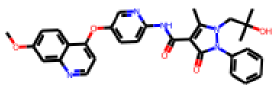	0.486902	COc1ccc2c(=O)cc(CNC(=O)c3c(C)n(C)n(-c4ccccc4)c3=O)nc3cccc3c2n1	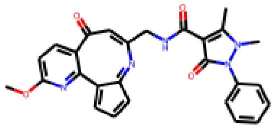	9.0	0.768132	3.608437	2.513170	5	0.823048
**2245**	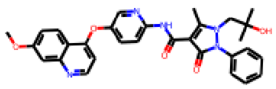	0.486716	COc1ccc2c(=O)cc(CNC(=O)c3c(C)n(C)n(-c4ccccc4)c3=O)nc3cccc3c2n1	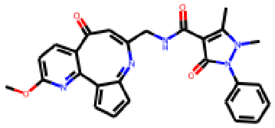	9.0	0.768132	3.608437	2.513170	5	0.823048
**2246**	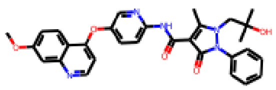	0.486670	COc1ccc2c(=O)cc(CNC(=O)c3c(C)n(C)n(-c4ccccc4)c3=O)nc3cccc3c2n1	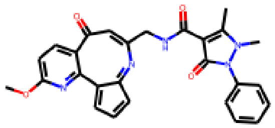	9.0	0.768132	3.608437	2.513170	5	0.823048
**2247**	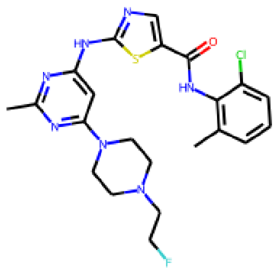	0.486649	Cc1nc(Nc2ncc(C(=O)Nc3c(C)cccc3Cl)s2)cc(N2C(NC(C)C2)O)n1	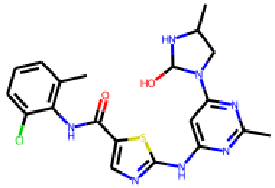	2.0	0.604401	2.952698	2.735638	4	0.781538

	src known mol	similarity	smiles	mol	Class	qed	logP	SAS	rings	kinase inhibition likeliness
**2248**	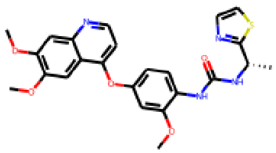	0.486600	COc1ccccc1NC(=O)Nc1ccc(Oc2ccnc3cc(OC)c(OC)cc23)cc1	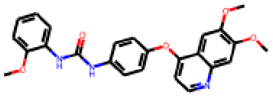	5.0	0.638163	3.995589	2.988471	4	0.789556
**2249**	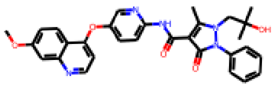	0.486564	COc1ccc2c(=O)cc(CNC(=O)c3c(C)n(C)n(-c4ccccc4)c3=O)nc3cccc3c2n1	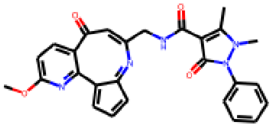	9.0	0.768132	3.608437	2.513170	5	0.823048
**2250**	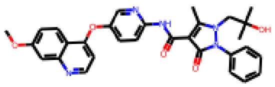	0.486559	COc1ccc2c(=O)cc(CNC(=O)c3c(C)n(C)n(-c4ccccc4)c3=O)nc3cccc2c3c1	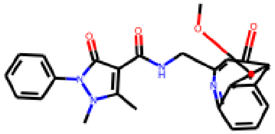	9.0	0.638163	3.995589	2.988471	5	0.774651
**2251**	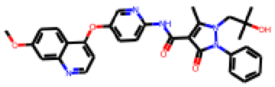	0.486559	COc1ccc2c(=O)cc(CNC(=O)c3c(C)n(C)n(-c4ccccc4)c3=O)nc3cccc2c3c1	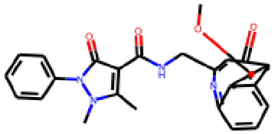	9.0	0.638163	3.995589	2.988471	5	0.774651

	src known mol	similarity	smiles	mol	Class	qed	logP	SAS	rings	kinase inhibition likeliness
**2252**	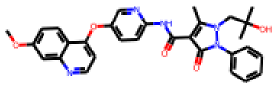	0.486559	COc1ccc2c(=O)cc(CNC(=O)c3c(C)n(C)n(-c4ccccc4)c3=O)nc3cccc2c3c1	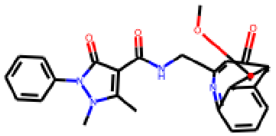	9.0	0.638163	3.995589	2.988471	5	0.774651
**2253**	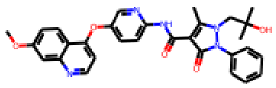	0.486559	COc1ccc2c(=O)cc(CNC(=O)c3c(C)n(C)n(-c4ccccc4)c3=O)nc3cccc2c3c1	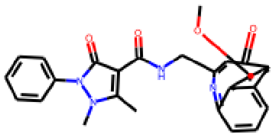	9.0	0.638163	3.995589	2.988471	5	0.774651
**2254**	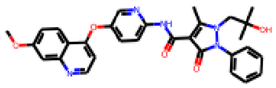	0.486081	COc1ccc2c(=O)cc(CNC(=O)c3c(C)n(C)n(-c4ccccc4)c3=O)nc3cccc2c3c1	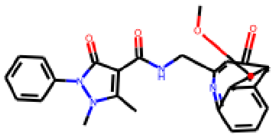	9.0	0.638163	3.995589	2.988471	5	0.774651
**2255**	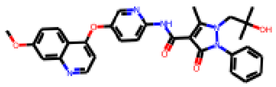	0.485730	COc1ccc2c(=O)cc(CNC(=O)c3c(C)n(C)n(-c4ccccc4)c3=O)nc3cccc2c3c1	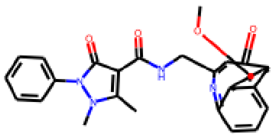	9.0	0.638163	3.995589	2.988471	5	0.774651

	src known mol	similarity	smiles	mol	Class	qed	logP	SAS	rings	kinase inhibition likeliness
**2256**	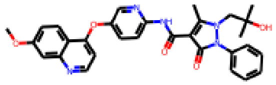	0.485618	COc1ccc2c(=O)cc(CNC(=O)c3c(C)n(C)n(-c4ccccc4)c3=O)nc3cccc3c2n1	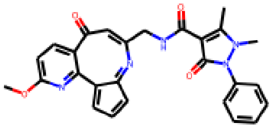	9.0	0.768132	3.608437	2.513170	5	0.823048
**2257**	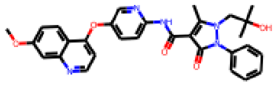	0.485585	COc1ccc2c(=O)cc(CNC(=O)c3c(C)n(C)n(-c4ccccc4)c3=O)nc3cccc3c2n1	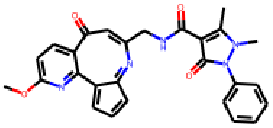	9.0	0.768132	3.608437	2.513170	5	0.823048
**2258**	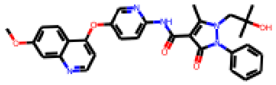	0.485585	COc1ccc2c(=O)cc(CNC(=O)c3c(C)n(C)n(-c4ccccc4)c3=O)nc3cccc3c2n1	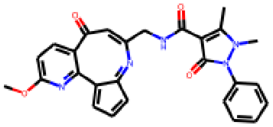	9.0	0.768132	3.608437	2.513170	5	0.823048
**2259**	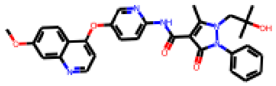	0.485585	COc1ccc2c(=O)cc(CNC(=O)c3c(C)n(C)n(-c4ccccc4)c3=O)nc3cccc3c2n1	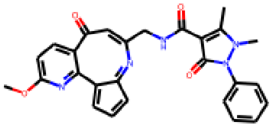	9.0	0.768132	3.608437	2.513170	5	0.823048

	src known mol	similarity	smiles	mol	Class	qed	logP	SAS	rings	kinase inhibition likeliness
**2260**	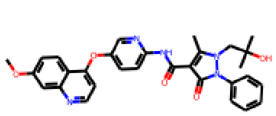	0.485561	COc1ccc2c(=O)cc(CNC(=O)c3c(C)n(C)n(-c4ccccc4)c3=O)nc3cccc2c3c1	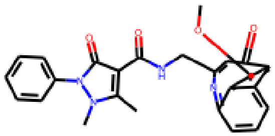	9.0	0.638163	3.995589	2.988471	5	0.774651
**2261**	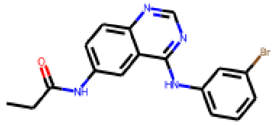	0.485327	O=C(COC(=O)Nc1CCccc1)Nc1ccc2ncnc(Nc3cccc(Br)c3)c2c1	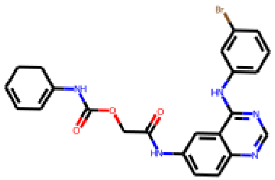	3.0	0.771867	2.951597	1.935530	4	0.775927
**2262**	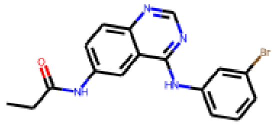	0.485327	O=C(COC(=O)Nc1CCccc1)Nc1ccc2ncnc(Nc3cccc(Br)c3)c2c1	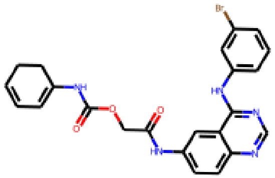	3.0	0.771867	2.951597	1.935530	4	0.775927
**2263**	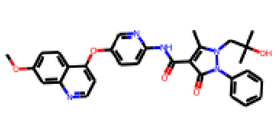	0.484832	COc1ccc2c(=O)cc(CNC(=O)c3c(C)n(C)n(-c4ccccc4)c3=O)nc3cccc2c3c1	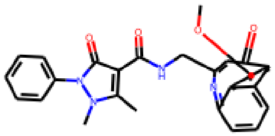	9.0	0.638163	3.995589	2.988471	5	0.774651

	src known mol	similarity	smiles	mol	Class	qed	logP	SAS	rings	kinase inhibition likeliness
**2264**	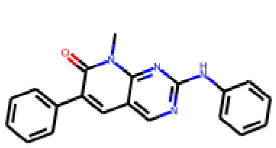	0.484824	Clc1cccc(Nc2ncc3cc(-c3c(Cl)cccc3Cl)c(=O)n(C)c3n2)c1	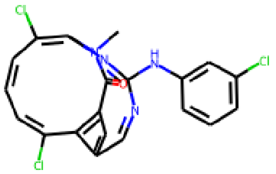	2.0	0.768132	3.608437	2.513170	5	0.784634
**2265**	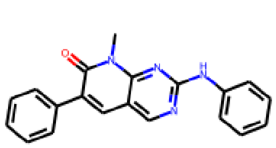	0.484824	Clc1cccc(Nc2ncc3cc(-c3c(Cl)cccc3Cl)c(=O)n(C)c3n2)c1	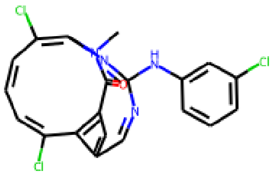	2.0	0.768132	3.608437	2.513170	5	0.784634
**2266**	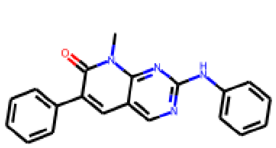	0.484824	Clc1cccc(Nc2ncc3cc(-c3c(Cl)cccc3Cl)c(=O)n(C)c3n2)c1	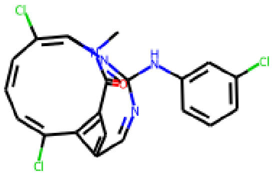	2.0	0.768132	3.608437	2.513170	5	0.784634
**2267**	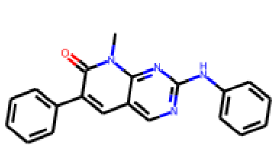	0.484824	Clc1cccc(Nc2ncc3cc(-c3c(Cl)cccc3Cl)c(=O)n(C)c3n2)c1	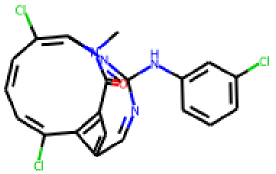	2.0	0.768132	3.608437	2.513170	5	0.784634

	src known mol	similarity	smiles	mol	Class	qed	logP	SAS	rings	kinase inhibition likeliness
**2268**	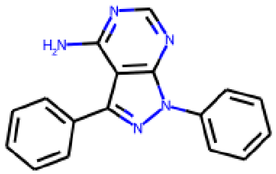	0.484790	Cc1ccc(C(=O)Oc2ccon2)cc1-c1ncnc2c1cnn2-c1ccccc1	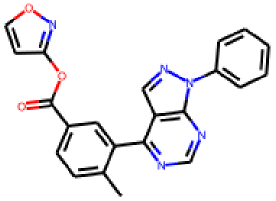	8.0	0.635315	4.542409	3.856266	5	0.760056
**2269**	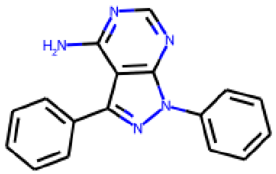	0.484790	Cc1ccc(C(=O)Oc2ccon2)cc1-c1ncnc2c1cnn2-c1ccccc1	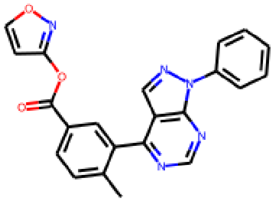	8.0	0.635315	4.542409	3.856266	5	0.760056
**2270**	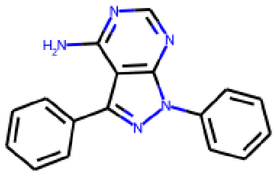	0.484790	Cc1ccc(C(=O)Oc2ccon2)cc1-c1ncnc2c1cnn2-c1ccccc1	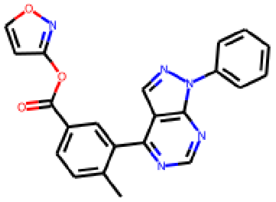	8.0	0.635315	4.542409	3.856266	5	0.760056
**2271**	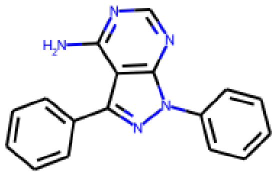	0.484790	Cc1ccc(C(=O)Oc2ccon2)cc1-c1ncnc2c1cnn2-c1ccccc1	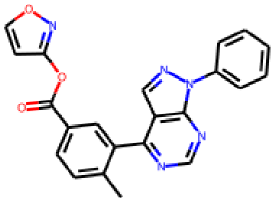	8.0	0.635315	4.542409	3.856266	5	0.760056

	src known mol	similarity	smiles	mol	Class	qed	logP	SAS	rings	kinase inhibition likeliness
**2272**	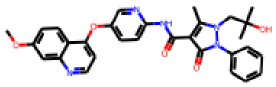	0.484763	COc1ccc2c(=O)cc(CNC(=O)c3c(C)n(C)n(-c4ccccc4)c3=O)nc3cccc3c2n1	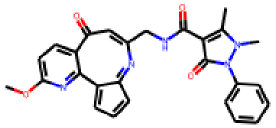	9.0	0.768132	3.608437	2.513170	5	0.823048
**2273**	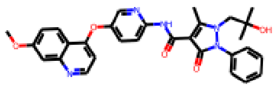	0.484763	COc1ccc2c(=O)cc(CNC(=O)c3c(C)n(C)n(-c4ccccc4)c3=O)nc3cccc3c2n1	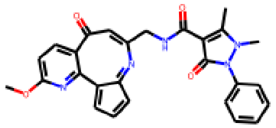	9.0	0.768132	3.608437	2.513170	5	0.823048
**2274**	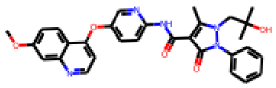	0.484763	COc1ccc2c(=O)cc(CNC(=O)c3c(C)n(C)n(-c4ccccc4)c3=O)nc3cccc3c2n1	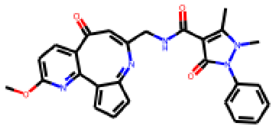	9.0	0.768132	3.608437	2.513170	5	0.823048
**2275**	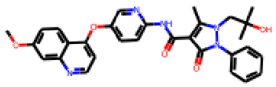	0.484402	COc1ccc2c(=O)cc(CNC(=O)c3c(C)n(C)n(-c4ccccc4)c3=O)nc3cccc3c2n1	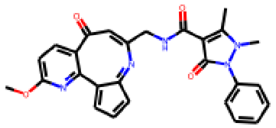	9.0	0.768132	3.608437	2.513170	5	0.823048

	src known mol	similarity	smiles	mol	Class	qed	logP	SAS	rings	kinase inhibition likeliness
**2276**	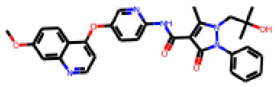	0.484402	COc1ccc2c(=O)cc(CNC(=O)c3c(C)n(C)n(-c4ccccc4)c3=O)nc3cccc3c2n1	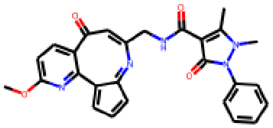	9.0	0.768132	3.608437	2.513170	5	0.823048
**2277**	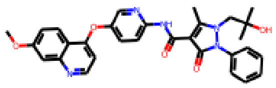	0.484402	COc1ccc2c(=O)cc(CNC(=O)c3c(C)n(C)n(-c4ccccc4)c3=O)nc3cccc3c2n1	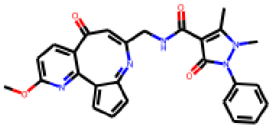	9.0	0.768132	3.608437	2.513170	5	0.823048
**2278**	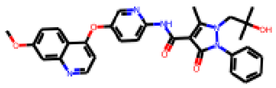	0.484402	COc1ccc2c(=O)cc(CNC(=O)c3c(C)n(C)n(-c4ccccc4)c3=O)nc3cccc3c2n1	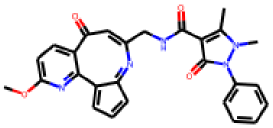	9.0	0.768132	3.608437	2.513170	5	0.823048
**2279**	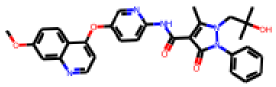	0.484402	COc1ccc2c(=O)cc(CNC(=O)c3c(C)n(C)n(-c4ccccc4)c3=O)nc3cccc3c2n1	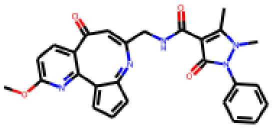	9.0	0.768132	3.608437	2.513170	5	0.823048

	src known mol	similarity	smiles	mol	Class	qed	logP	SAS	rings	kinase inhibition likeliness
**2280**	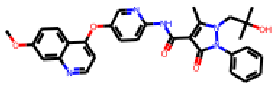	0.484290	COc1ccc2c(=O)cc(CNC(=O)c3c(C)n(C)n(-c4ccccc4)c3=O)nc3cccc2c3c1	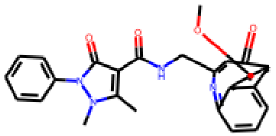	9.0	0.638163	3.995589	2.988471	5	0.774651
**2281**	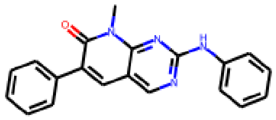	0.484277	Clc1cccc(Nc2ncc3cc(-c3c(Cl)cccc3Cl)c(=O)n(C)c3n2)c1	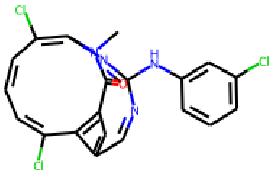	2.0	0.768132	3.608437	2.513170	5	0.784634
**2282**	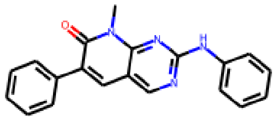	0.484277	Clc1cccc(Nc2ncc3cc(-c3c(Cl)cccc3Cl)c(=O)n(C)c3n2)c1	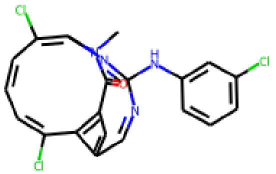	2.0	0.768132	3.608437	2.513170	5	0.784634
**2283**	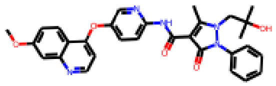	0.484127	COc1ccc2c(=O)cc(CNC(=O)c3c(C)n(C)n(-c4ccccc4)c3=O)nc3cccc3c2n1	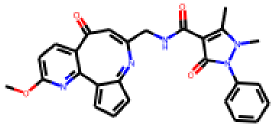	9.0	0.768132	3.608437	2.513170	5	0.823048

	src known mol	similarity	smiles	mol	Class	qed	logP	SAS	rings	kinase inhibition likeliness
**2284**	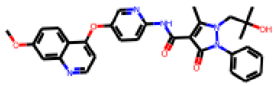	0.484127	COc1ccc2c(=O)cc(CNC(=O)c3c(C)n(C)n(-c4ccccc4)c3=O)nc3cccc3c2n1	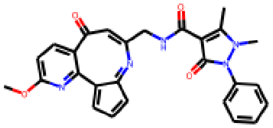	9.0	0.768132	3.608437	2.513170	5	0.823048
**2285**	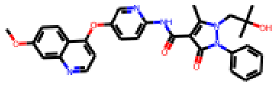	0.484080	COc1ccc2c(=O)cc(CNC(=O)c3c(C)n(C)n(-c4ccccc4)c3=O)nc3cccc2c3c1	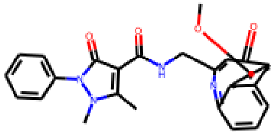	9.0	0.638163	3.995589	2.988471	5	0.774651
**2286**	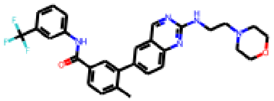	0.483971	Cc1ccc(NC(=O)c2cccc(C(F)(F)F)c2)cc1-c1ccc2nc(O)ncc2c1	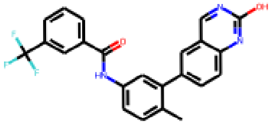	6.0	0.638163	3.995589	2.988471	4	0.818430
**2287**	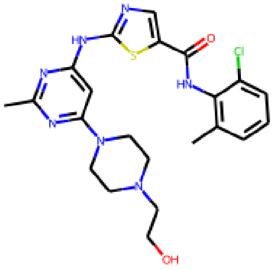	0.483894	Cc1nc(Nc2ccc(C(=O)Nc3c(C)cccc3Cl)s2)cc(N2CCNC(O)CC2)n1	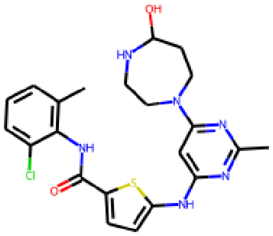	2.0	0.700685	3.487358	0.062602	4	0.770048

	src known mol	similarity	smiles	mol	Class	qed	logP	SAS	rings	kinase inhibition likeliness
**2288**	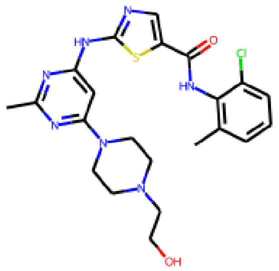	0.483894	Cc1nc(Nc2ccc(C(=O)Nc3c(C)cccc3Cl)s2)cc(N2CCNC(O)CC2)n1	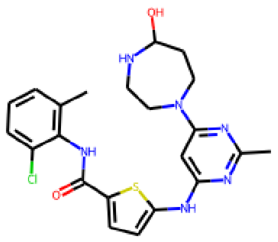	2.0	0.700685	3.487358	0.062602	4	0.770048
**2289**	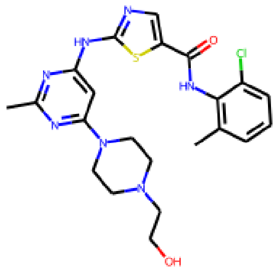	0.483894	Cc1nc(Nc2ccc(C(=O)Nc3c(C)cccc3Cl)s2)cc(N2CCNC(O)CC2)n1	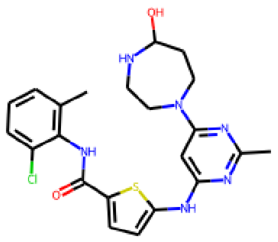	2.0	0.700685	3.487358	0.062602	4	0.770048
**2290**	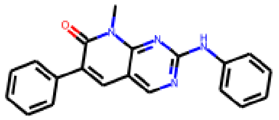	0.483846	Clc1cccc(Nc2ncc3cc(-c3c(Cl)cccc3Cl)c(=O)n(C)c3n2)c1	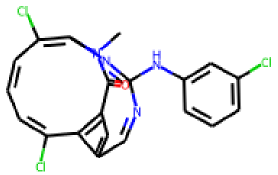	2.0	0.768132	3.608437	2.513170	5	0.784634
**2291**	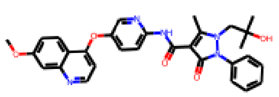	0.483819	COc1ccc2c(=O)cc(CNC(=O)c3c(C)n(C)n(-c4ccccc4)c3=O)nc3cccc2c3c1	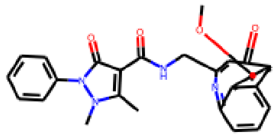	9.0	0.638163	3.995589	2.988471	5	0.774651

	src known mol	similarity	smiles	mol	Class	qed	logP	SAS	rings	kinase inhibition likeliness
**2292**	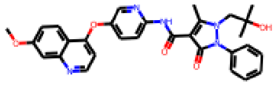	0.483816	COc1ccc2c(=O)cc(CNC(=O)c3c(C)n(C)n(-c4ccccc4)c3=O)nc3cccc3c2n1	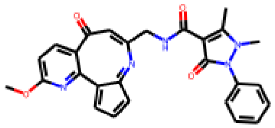	9.0	0.768132	3.608437	2.513170	5	0.823048
**2293**	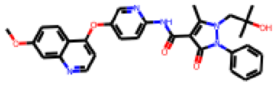	0.483816	COc1ccc2c(=O)cc(CNC(=O)c3c(C)n(C)n(-c4ccccc4)c3=O)nc3cccc3c2n1	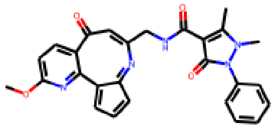	9.0	0.768132	3.608437	2.513170	5	0.823048
**2294**	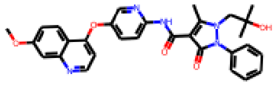	0.483801	COc1ccc2c(=O)cc(CNC(=O)c3c(C)n(C)n(-c4ccccc4)c3=O)nc3cccc2c3c1	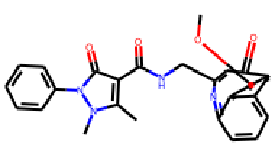	9.0	0.638163	3.995589	2.988471	5	0.774651
**2295**	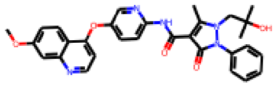	0.483801	COc1ccc2c(=O)cc(CNC(=O)c3c(C)n(C)n(-c4ccccc4)c3=O)nc3cccc2c3c1	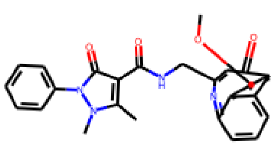	9.0	0.638163	3.995589	2.988471	5	0.774651

	src known mol	similarity	smiles	mol	Class	qed	logP	SAS	rings	kinase inhibition likeliness
**2296**	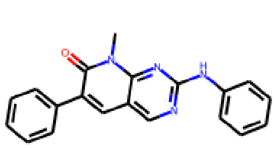	0.483796	Clc1cccc(Nc2ncc3cc(-c3c(Cl)cccc3Cl)c(=O)n(C)c3n2)c1	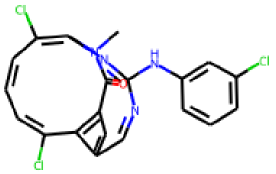	2.0	0.768132	3.608437	2.513170	5	0.784634
**2297**	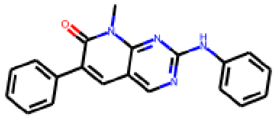	0.483645	Clc1cccc(Nc2ncc3cc(-c3c(Cl)cccc3Cl)c(=O)n(C)c3n2)c1	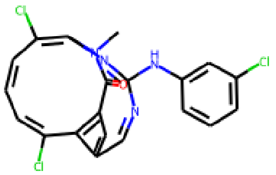	2.0	0.768132	3.608437	2.513170	5	0.784634
**2298**	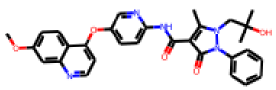	0.483504	COc1ccc2c(=O)cc(CNC(=O)c3c(C)n(C)n(-c4ccccc4)c3=O)nc3cccc3c2n1	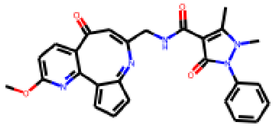	9.0	0.768132	3.608437	2.513170	5	0.823048
**2299**	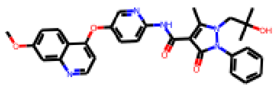	0.483504	COc1ccc2c(=O)cc(CNC(=O)c3c(C)n(C)n(-c4ccccc4)c3=O)nc3cccc3c2n1	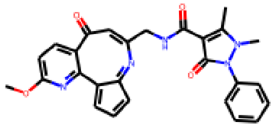	9.0	0.768132	3.608437	2.513170	5	0.823048

	src known mol	similarity	smiles	mol	Class	qed	logP	SAS	rings	kinase inhibition likeliness
**2300**	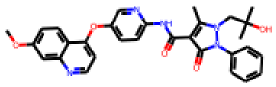	0.483504	COc1ccc2c(=O)cc(CNC(=O)c3c(C)n(C)n(-c4ccccc4)c3=O)nc3cccc3c2n1	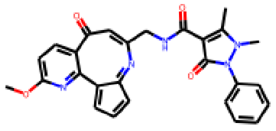	9.0	0.768132	3.608437	2.513170	5	0.823048
**2301**	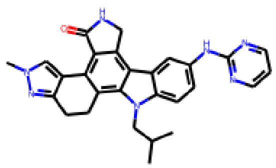	0.483251	Cc1ccc(C(=O)Nc2cccc(C(F)(C)F)c2C)cc1-c1ccc2nc(=O)cccc(F)nc2n1	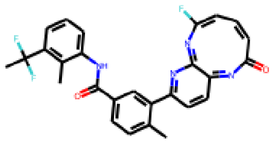	6.0	0.820294	3.038428	1.698172	4	0.833140
**2302**	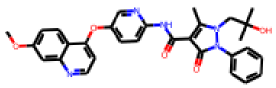	0.483032	COc1ccc2c(=O)cc(CNC(=O)c3c(C)n(C)n(-c4ccccc4)c3=O)nc3cccc3c2n1	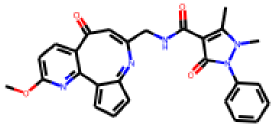	9.0	0.768132	3.608437	2.513170	5	0.823048
**2303**	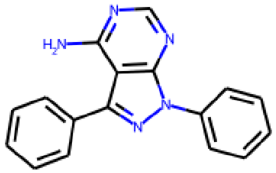	0.482984	Cc1ccc(C(=O)Oc2ccon2)cc1-c1ncnc2c1cnn2-c1ccccc1	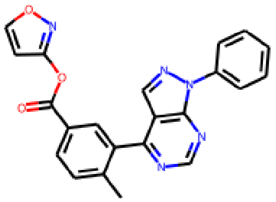	8.0	0.635315	4.542409	3.856266	5	0.760056

	src known mol	similarity	smiles	mol	Class	qed	logP	SAS	rings	kinase inhibition likeliness
**2304**	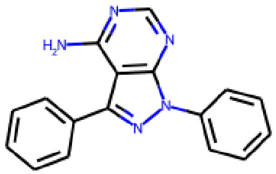	0.482984	Cc1ccc(C(=O)Oc2ccon2)cc1-c1ncnc2c1cnn2-c1ccccc1	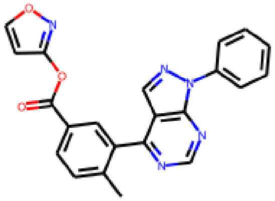	8.0	0.635315	4.542409	3.856266	5	0.760056
**2305**	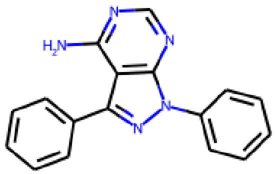	0.482984	Cc1ccc(C(=O)Oc2ccon2)cc1-c1ncnc2c1cnn2-c1ccccc1	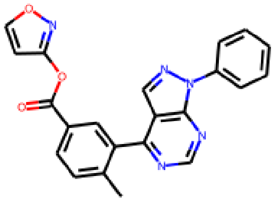	8.0	0.635315	4.542409	3.856266	5	0.760056
**2306**	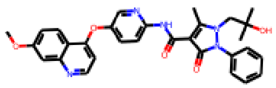	0.482852	COc1ccc2c(=O)cc(CNC(=O)c3c(C)n(C)n(-c4ccccc4)c3=O)nc3cccc2c3c1	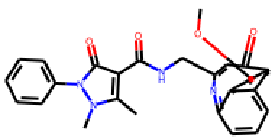	9.0	0.638163	3.995589	2.988471	5	0.774651
**2307**	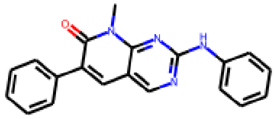	0.482838	Clc1cccc(Nc2ncc3cc(-c3c(Cl)cccc3Cl)c(=O)n(C)c3n2)c1	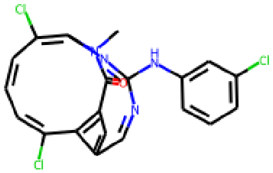	2.0	0.768132	3.608437	2.513170	5	0.784634

	src known mol	similarity	smiles	mol	Class	qed	logP	SAS	rings	kinase inhibition likeliness
**2308**	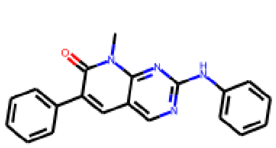	0.482838	Clc1cccc(Nc2ncc3cc(-c3c(Cl)cccc3Cl)c(=O)n(C)c3n2)c1	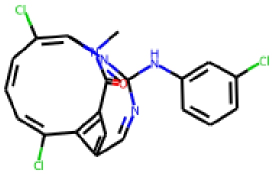	2.0	0.768132	3.608437	2.513170	5	0.784634
**2309**	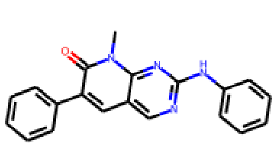	0.482838	Clc1cccc(Nc2ncc3cc(-c3c(Cl)cccc3Cl)c(=O)n(C)c3n2)c1	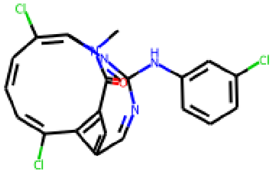	2.0	0.768132	3.608437	2.513170	5	0.784634
**2310**	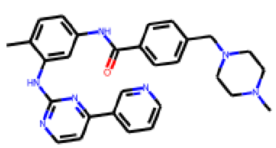	0.482818	Cc1ccc(NC(=O)c2ccccc2)cc1Nc1nccc(-c2cccnc2)n1	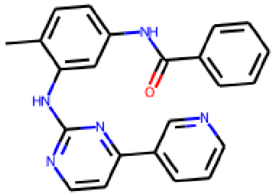	0.0	0.768132	3.608437	2.513170	4	0.854555
**2311**	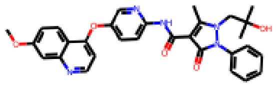	0.482759	COc1ccc2c(=O)cc(CNC(=O)c3c(C)n(C)n(-c4ccccc4)c3=O)nc3cccc2c3c1	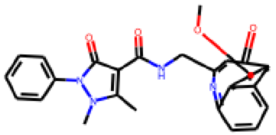	9.0	0.638163	3.995589	2.988471	5	0.774651

	src known mol	similarity	smiles	mol	Class	qed	logP	SAS	rings	kinase inhibition likeliness
**2312**	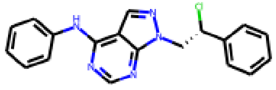	0.482517	Cc1ccc(C(=O)Nc2ccon2)cc1Nc1ncnc2c1cnn2C1cccccc1	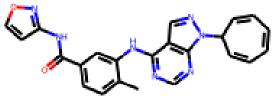	8.0	0.623362	2.525411	2.997881	5	0.770298
**2313**	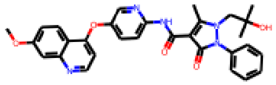	0.482486	COc1ccc2c(=O)cc(CNC(=O)c3c(C)n(C)n(-c4ccccc4)c3=O)nc3cccc3c2n1	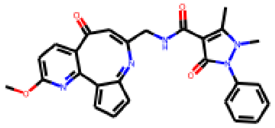	9.0	0.768132	3.608437	2.513170	5	0.823048
**2314**	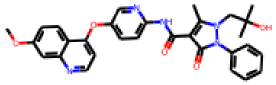	0.482486	COc1ccc2c(=O)cc(CNC(=O)c3c(C)n(C)n(-c4ccccc4)c3=O)nc3cccc3c2n1	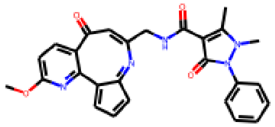	9.0	0.768132	3.608437	2.513170	5	0.823048
**2315**	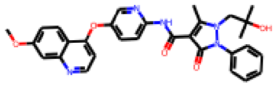	0.482480	COc1ccc2c(=O)cc(CNC(=O)c3c(C)n(C)n(-c4ccccc4)c3=O)nc3cccc2c3c1	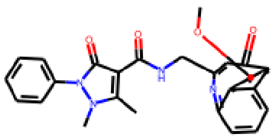	9.0	0.638163	3.995589	2.988471	5	0.774651

	src known mol	similarity	smiles	mol	Class	qed	logP	SAS	rings	kinase inhibition likeliness
**2316**	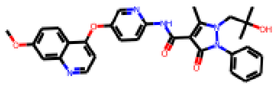	0.482461	COc1ccc2c(=O)cc(CNC(=O)c3c(C)n(C)n(-c4ccccc4)c3=O)nc3cccc2c3c1	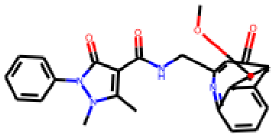	9.0	0.638163	3.995589	2.988471	5	0.774651
**2317**	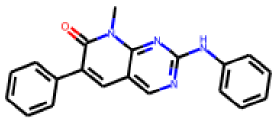	0.482264	Clc1cccc(Nc2ncc3cc(-c3c(Cl)cccc3Cl)c(=O)n(C)c3n2)c1	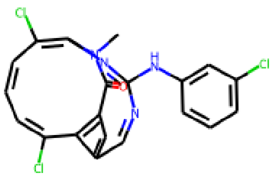	2.0	0.768132	3.608437	2.513170	5	0.784634
**2318**	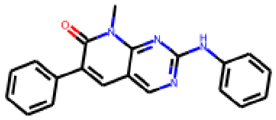	0.482264	Clc1cccc(Nc2ncc3cc(-c3c(Cl)cccc3Cl)c(=O)n(C)c3n2)c1	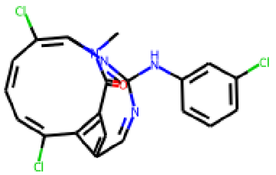	2.0	0.768132	3.608437	2.513170	5	0.784634
**2319**	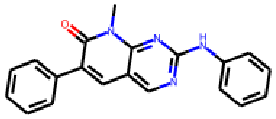	0.482198	Clc1cccc(Nc2ncc3cc(-c3c(Cl)cccc3Cl)c(=O)n(C)c3n2)c1	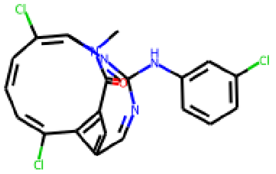	2.0	0.768132	3.608437	2.513170	5	0.784634

	src known mol	similarity	smiles	mol	Class	qed	logP	SAS	rings	kinase inhibition likeliness
**2320**	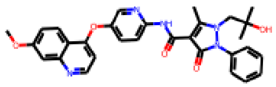	0.482063	COc1ccc2c(=O)cc(CNC(=O)c3c(C)n(C)n(-c4ccccc4)c3=O)nc3cccc3c2n1	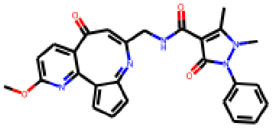	9.0	0.768132	3.608437	2.513170	5	0.823048
**2321**	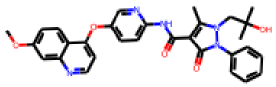	0.482063	COc1ccc2c(=O)cc(CNC(=O)c3c(C)n(C)n(-c4ccccc4)c3=O)nc3cccc3c2n1	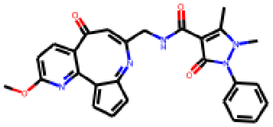	9.0	0.768132	3.608437	2.513170	5	0.823048
**2322**	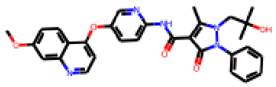	0.482051	COc1ccc2c(=O)cc(CNC(=O)c3c(C)n(C)n(-c4ccccc4)c3=O)nc3cccc3c2n1	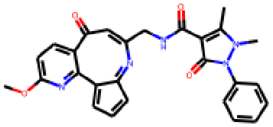	9.0	0.768132	3.608437	2.513170	5	0.823048
**2323**	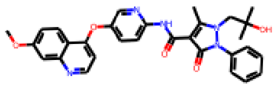	0.482051	COc1ccc2c(=O)cc(CNC(=O)c3c(C)n(C)n(-c4ccccc4)c3=O)nc3cccc3c2n1	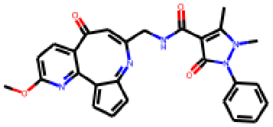	9.0	0.768132	3.608437	2.513170	5	0.823048

	src known mol	similarity	smiles	mol	Class	qed	logP	SAS	rings	kinase inhibition likeliness
**2324**	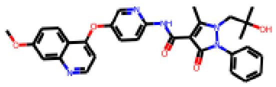	0.481962	COc1ccc2c(=O)cc(CNC(=O)c3c(C)n(C)n(-c4ccccc4)c3=O)nc3cccc3c2n1	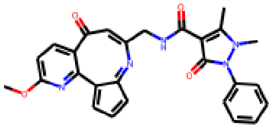	9.0	0.768132	3.608437	2.513170	5	0.823048
**2325**	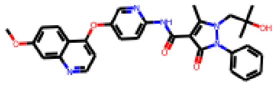	0.481880	COc1ccc2c(=O)cc(CNC(=O)c3c(C)n(C)n(-c4ccccc4)c3=O)nc3cccc3c2n1	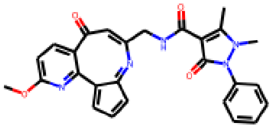	9.0	0.768132	3.608437	2.513170	5	0.823048
**2326**	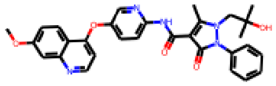	0.481740	COc1ccc2c(=O)cc(CNC(=O)c3c(C)n(C)n(-c4ccccc4)c3=O)nc3cccc2c3c1	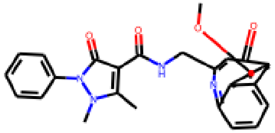	9.0	0.638163	3.995589	2.988471	5	0.774651
**2327**	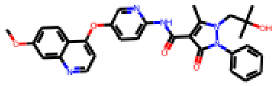	0.481711	COc1ccc2c(=O)cc(CNC(=O)c3c(C)n(C)n(-c4ccccc4)c3=O)nc3cccc3c2n1	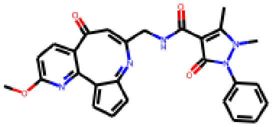	9.0	0.768132	3.608437	2.513170	5	0.823048

	src known mol	similarity	smiles	mol	Class	qed	logP	SAS	rings	kinase inhibition likeliness
**2328**	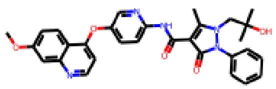	0.481641	COc1ccc2c(=O)cc(CNC(=O)c3c(C)n(C)n(-<br>c4ccccc4)c3=O)nc3cccc2c3c1	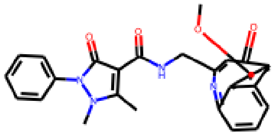	9.0	0.638163	3.995589	2.988471	5	0.774651
**2329**	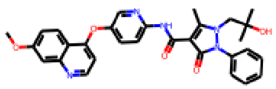	0.481561	COc1ccc2c(=O)cc(CNC(=O)c3c(C)n(C)n(-<br>c4ccccc4)c3=O)nc3cccc2c3c1	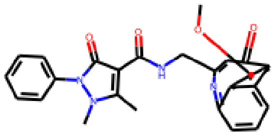	9.0	0.638163	3.995589	2.988471	5	0.774651
**2330**	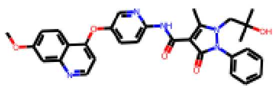	0.481460	COc1ccc2c(=O)cc(CNC(=O)c3c(C)n(C)n(-<br>c4ccccc4)c3=O)nc3cccc3c2n1	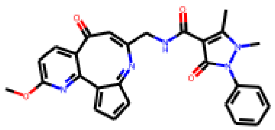	9.0	0.768132	3.608437	2.513170	5	0.823048
**2331**	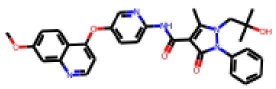	0.481460	COc1ccc2c(=O)cc(CNC(=O)c3c(C)n(C)n(-<br>c4ccccc4)c3=O)nc3cccc3c2n1	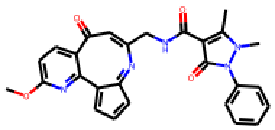	9.0	0.768132	3.608437	2.513170	5	0.823048

	src known mol	similarity	smiles	mol	Class	qed	logP	SAS	rings	kinase inhibition likeliness
**2332**	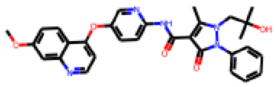	0.481229	COc1ccc2c(=O)cc(CNC(=O)c3c(C)n(C)n(-c4ccccc4)c3=O)nc3cccc3c2n1	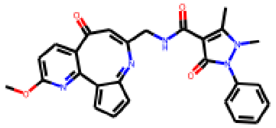	9.0	0.768132	3.608437	2.513170	5	0.823048
**2333**	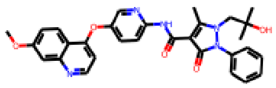	0.481229	COc1ccc2c(=O)cc(CNC(=O)c3c(C)n(C)n(-c4ccccc4)c3=O)nc3cccc3c2n1	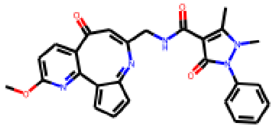	9.0	0.768132	3.608437	2.513170	5	0.823048
**2334**	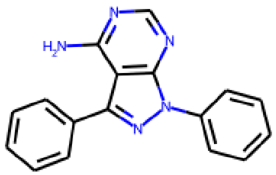	0.481070	Cc1ccc(C(=O)Oc2ccon2)cc1-c1ncnc2c1cnn2-c1ccccc1	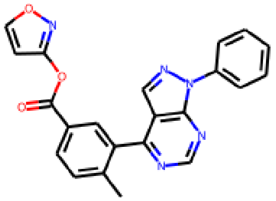	8.0	0.635315	4.542409	3.856266	5	0.760056
**2335**	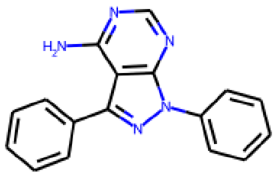	0.481070	Cc1ccc(C(=O)Oc2ccon2)cc1-c1ncnc2c1cnn2-c1ccccc1	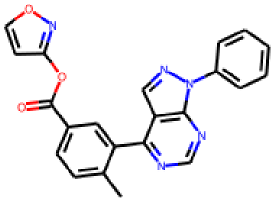	8.0	0.635315	4.542409	3.856266	5	0.760056

	src known mol	similarity	smiles	mol	Class	qed	logP	SAS	rings	kinase inhibition likeliness
**2336**	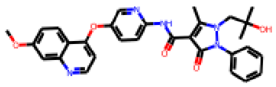	0.481041	COc1ccc2c(=O)cc(CNC(=O)c3c(C)n(C)n(-c4ccccc4)c3=O)nc3cccc3c2n1	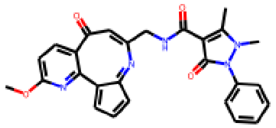	9.0	0.768132	3.608437	2.513170	5	0.823048
**2337**	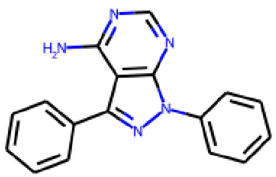	0.480996	Cc1ccc(C(=O)Oc2ccon2)cc1-c1ncnc2c1cnn2-c1ccccc1	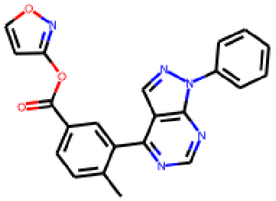	8.0	0.635315	4.542409	3.856266	5	0.760056
**2338**	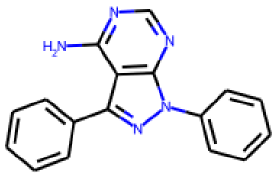	0.480996	Cc1ccc(C(=O)Oc2ccon2)cc1-c1ncnc2c1cnn2-c1ccccc1	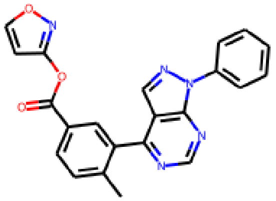	8.0	0.635315	4.542409	3.856266	5	0.760056
**2339**	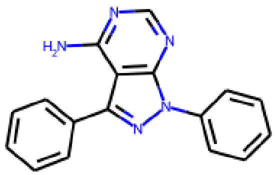	0.480996	Cc1ccc(C(=O)Oc2ccon2)cc1-c1ncnc2c1cnn2-c1ccccc1	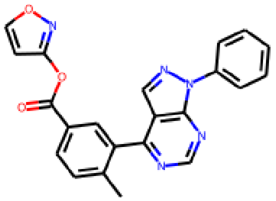	8.0	0.635315	4.542409	3.856266	5	0.760056

	src known mol	similarity	smiles	mol	Class	qed	logP	SAS	rings	kinase inhibition likeliness
**2340**	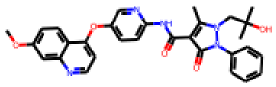	0.480977	COc1ccc2c(=O)cc(CNC(=O)c3c(C)n(C)n(-c4ccccc4)c3=O)nc3cccc3c2n1	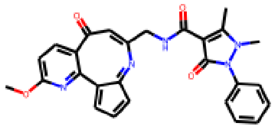	9.0	0.768132	3.608437	2.513170	5	0.823048
**2341**	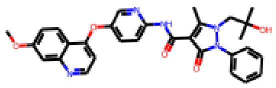	0.480977	COc1ccc2c(=O)cc(CNC(=O)c3c(C)n(C)n(-c4ccccc4)c3=O)nc3cccc3c2n1	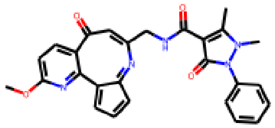	9.0	0.768132	3.608437	2.513170	5	0.823048
**2342**	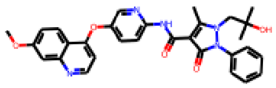	0.480977	COc1ccc2c(=O)cc(CNC(=O)c3c(C)n(C)n(-c4ccccc4)c3=O)nc3cccc3c2n1	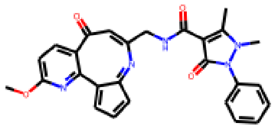	9.0	0.768132	3.608437	2.513170	5	0.823048
**2343**	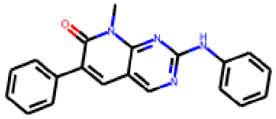	0.480956	Clc1cccc(Nc2ncc3cc(-c3c(Cl)cccc3Cl)c(=O)n(C)c3n2)c1	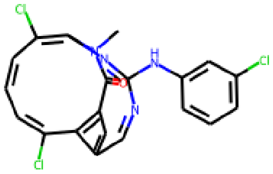	2.0	0.768132	3.608437	2.513170	5	0.784634

	src known mol	similarity	smiles	mol	Class	qed	logP	SAS	rings	kinase inhibition likeliness
**2344**	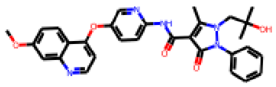	0.480955	COc1ccc2c(=O)cc(CNC(=O)c3c(C)n(C)n(-c4ccccc4)c3=O)nc3cccc3c2n1	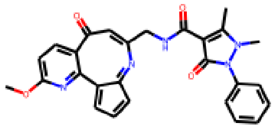	9.0	0.768132	3.608437	2.513170	5	0.823048
**2345**	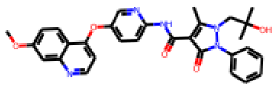	0.480933	COc1ccc2c(=O)cc(CNC(=O)c3c(C)n(C)n(-c4ccccc4)c3=O)nc3cccc3c2n1	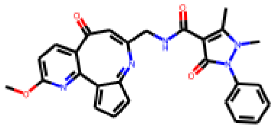	9.0	0.768132	3.608437	2.513170	5	0.823048
**2346**	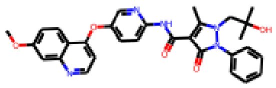	0.480933	COc1ccc2c(=O)cc(CNC(=O)c3c(C)n(C)n(-c4ccccc4)c3=O)nc3cccc3c2n1	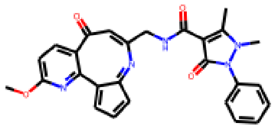	9.0	0.768132	3.608437	2.513170	5	0.823048
**2347**	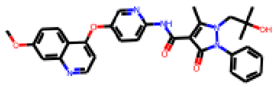	0.480933	COc1ccc2c(=O)cc(CNC(=O)c3c(C)n(C)n(-c4ccccc4)c3=O)nc3cccc3c2n1	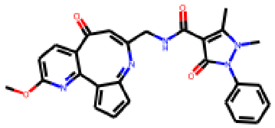	9.0	0.768132	3.608437	2.513170	5	0.823048

	src known mol	similarity	smiles	mol	Class	qed	logP	SAS	rings	kinase inhibition likeliness
**2348**	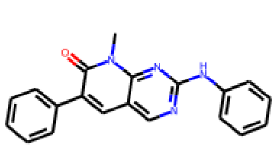	0.480755	Clc1cccc(Nc2ncc3cc(-c3c(Cl)cccc3Cl)c(=O)n(C)c3n2)c1	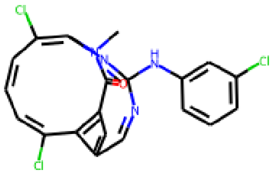	2.0	0.768132	3.608437	2.513170	5	0.784634
**2349**	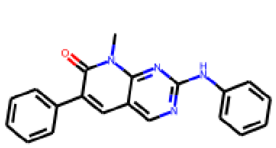	0.480755	Clc1cccc(Nc2ncc3cc(-c3c(Cl)cccc3Cl)c(=O)n(C)c3n2)c1	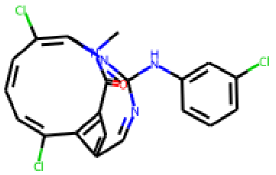	2.0	0.768132	3.608437	2.513170	5	0.784634
**2350**	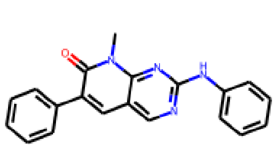	0.480755	Clc1cccc(Nc2ncc3cc(-c3c(Cl)cccc3Cl)c(=O)n(C)c3n2)c1	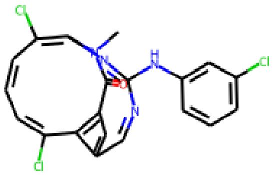	2.0	0.768132	3.608437	2.513170	5	0.784634
**2351**	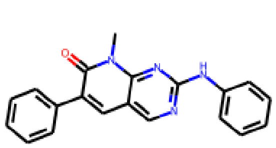	0.480680	Clc1cccc(Nc2ncc3cc(-c3c(Cl)cccc3Cl)c(=O)n(C)c3n2)c1	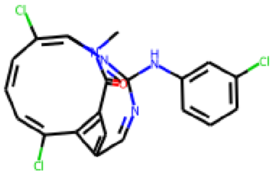	2.0	0.768132	3.608437	2.513170	5	0.784634

	src known mol	similarity	smiles	mol	Class	qed	logP	SAS	rings	kinase inhibition likeliness
**2352**	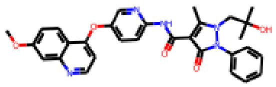	0.480645	COc1ccc2c(=O)cc(CNC(=O)c3c(C)n(C)n(-c4ccccc4)c3=O)nc3cccc2c3c1	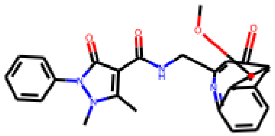	9.0	0.638163	3.995589	2.988471	5	0.774651
**2353**	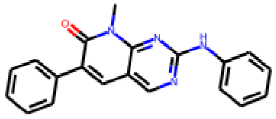	0.480422	Clc1cccc(Nc2ncc3cc(-c3c(Cl)cccc3Cl)c(=O)n(C)c3n2)c1	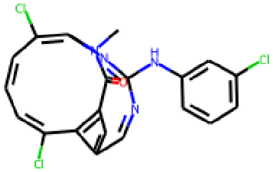	2.0	0.768132	3.608437	2.513170	5	0.784634
**2354**	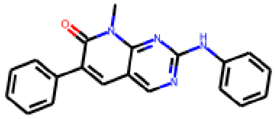	0.480422	Clc1cccc(Nc2ncc3cc(-c3c(Cl)cccc3Cl)c(=O)n(C)c3n2)c1	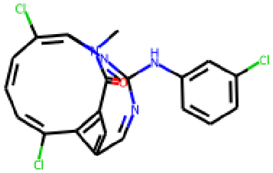	2.0	0.768132	3.608437	2.513170	5	0.784634
**2355**	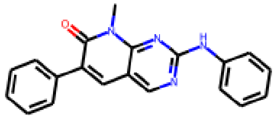	0.480422	Clc1cccc(Nc2ncc3cc(-c3c(Cl)cccc3Cl)c(=O)n(C)c3n2)c1	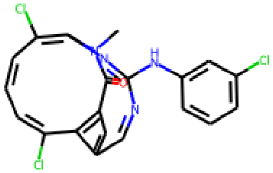	2.0	0.768132	3.608437	2.513170	5	0.784634

	src known mol	similarity	smiles	mol	Class	qed	logP	SAS	rings	kinase inhibition likeliness
**2356**	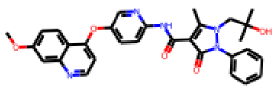	0.480409	COc1ccc2c(=O)cc(CNC(=O)c3c(C)n(C)n(-c4ccccc4)c3=O)nc3cccc3c2n1	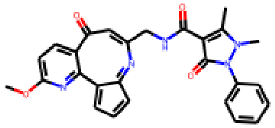	9.0	0.768132	3.608437	2.513170	5	0.823048
**2357**	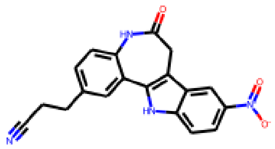	0.480346	CCNC(=O)N1CCC(Nc2n(C(C)c3c(-c4ccccc4)[nH]c4ccccc34)n2)CC1	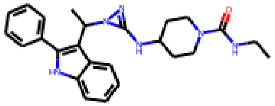	7.0	0.623584	3.603734	4.478009	5	0.804049
**2358**	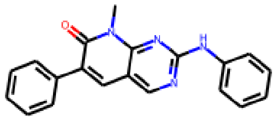	0.480030	Clc1cccc(Nc2ncc3cc(-c3c(Cl)cccc3Cl)c(=O)n(C)c3n2)c1	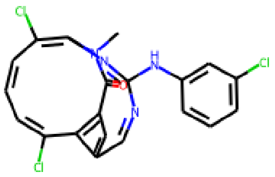	2.0	0.768132	3.608437	2.513170	5	0.784634
**2359**	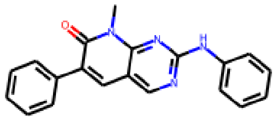	0.480030	Clc1cccc(Nc2ncc3cc(-c3c(Cl)cccc3Cl)c(=O)n(C)c3n2)c1	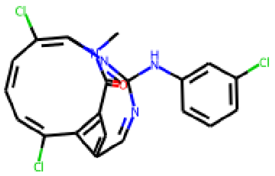	2.0	0.768132	3.608437	2.513170	5	0.784634

	src known mol	similarity	smiles	mol	Class	qed	logP	SAS	rings	kinase inhibition likeliness
**2360**	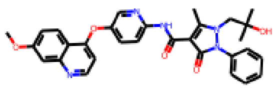	0.479935	COc1ccc2c(=O)cc(CNC(=O)c3c(C)n(C)n(-c4ccccc4)c3=O)nc3cccc2c3c1	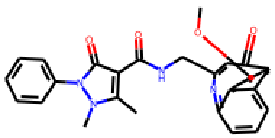	9.0	0.638163	3.995589	2.988471	5	0.774651
**2361**	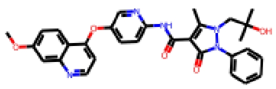	0.479935	COc1ccc2c(=O)cc(CNC(=O)c3c(C)n(C)n(-c4ccccc4)c3=O)nc3cccc2c3c1	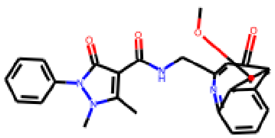	9.0	0.638163	3.995589	2.988471	5	0.774651
**2362**	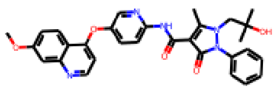	0.479935	COc1ccc2c(=O)cc(CNC(=O)c3c(C)n(C)n(-c4ccccc4)c3=O)nc3cccc2c3c1	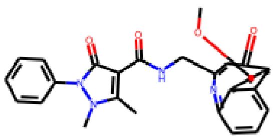	9.0	0.638163	3.995589	2.988471	5	0.774651
**2363**	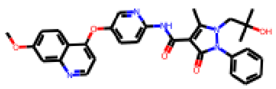	0.479935	COc1ccc2c(=O)cc(CNC(=O)c3c(C)n(C)n(-c4ccccc4)c3=O)nc3cccc2c3c1	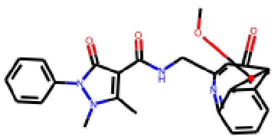	9.0	0.638163	3.995589	2.988471	5	0.774651

	src known mol	similarity	smiles	mol	Class	qed	logP	SAS	rings	kinase inhibition likeliness
**2364**	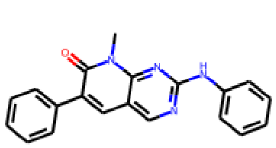	0.479848	Clc1cccc(Nc2ncc3cc(-c3c(Cl)cccc3Cl)c(=O)n(C)c3n2)c1	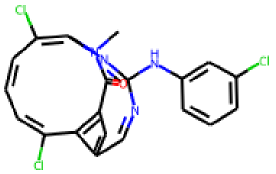	2.0	0.768132	3.608437	2.513170	5	0.784634
**2365**	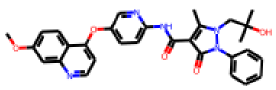	0.479818	COc1ccc2c(=O)cc(CNC(=O)c3c(C)n(C)n(-c4ccccc4)c3=O)nc3cccc3c2n1	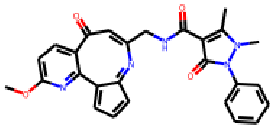	9.0	0.768132	3.608437	2.513170	5	0.823048
**2366**	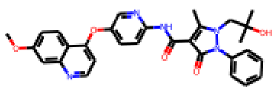	0.479818	COc1ccc2c(=O)cc(CNC(=O)c3c(C)n(C)n(-c4ccccc4)c3=O)nc3cccc3c2n1	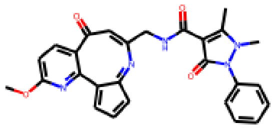	9.0	0.768132	3.608437	2.513170	5	0.823048
**2367**	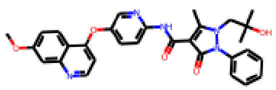	0.479569	COc1ccc2c(=O)cc(CNC(=O)c3c(C)n(C)n(-c4ccccc4)c3=O)nc3cccc3c2n1	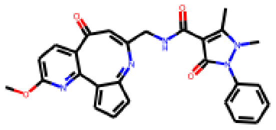	9.0	0.768132	3.608437	2.513170	5	0.823048

	src known mol	similarity	smiles	mol	Class	qed	logP	SAS	rings	kinase inhibition likeliness
**2368**	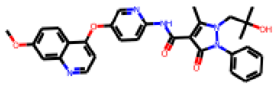	0.479569	COc1ccc2c(=O)cc(CNC(=O)c3c(C)n(C)n(-c4ccccc4)c3=O)nc3cccc3c2n1	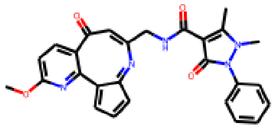	9.0	0.768132	3.608437	2.513170	5	0.823048
**2369**	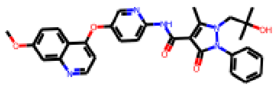	0.479569	COc1ccc2c(=O)cc(CNC(=O)c3c(C)n(C)n(-c4ccccc4)c3=O)nc3cccc3c2n1	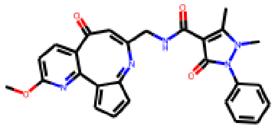	9.0	0.768132	3.608437	2.513170	5	0.823048
**2370**	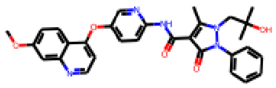	0.479545	COc1ccc2c(=O)cc(CNC(=O)c3c(C)n(C)n(-c4ccccc4)c3=O)nc3cccc3c2n1	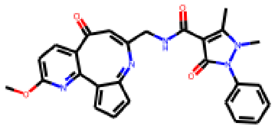	9.0	0.768132	3.608437	2.513170	5	0.823048
**2371**	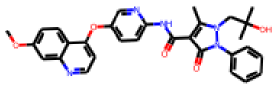	0.479545	COc1ccc2c(=O)cc(CNC(=O)c3c(C)n(C)n(-c4ccccc4)c3=O)nc3cccc3c2n1	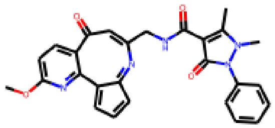	9.0	0.768132	3.608437	2.513170	5	0.823048

	src known mol	similarity	smiles	mol	Class	qed	logP	SAS	rings	kinase inhibition likeliness
**2372**	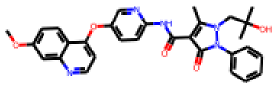	0.479545	COc1ccc2c(=O)cc(CNC(=O)c3c(C)n(C)n(-c4ccccc4)c3=O)nc3cccc3c2n1	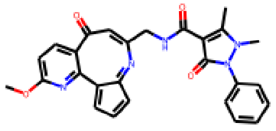	9.0	0.768132	3.608437	2.513170	5	0.823048
**2373**	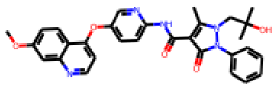	0.479545	COc1ccc2c(=O)cc(CNC(=O)c3c(C)n(C)n(-c4ccccc4)c3=O)nc3cccc3c2n1	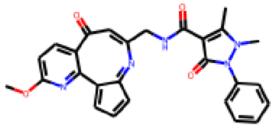	9.0	0.768132	3.608437	2.513170	5	0.823048
**2374**	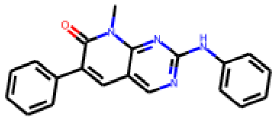	0.479514	Clc1cccc(Nc2ncc3cc(-c3c(Cl)cccc3Cl)c(=O)n(C)c3n2)c1	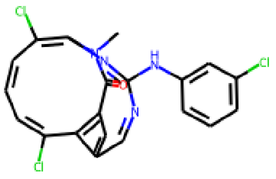	2.0	0.768132	3.608437	2.513170	5	0.784634
**2375**	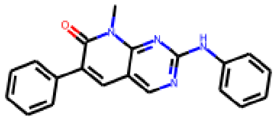	0.479493	Clc1cccc(Nc2ncc3cc(-c3c(Cl)cccc3Cl)c(=O)n(C)c3n2)c1	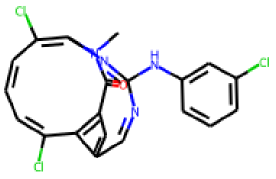	2.0	0.768132	3.608437	2.513170	5	0.784634

	src known mol	similarity	smiles	mol	Class	qed	logP	SAS	rings	kinase inhibition likeliness
**2376**	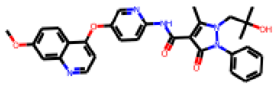	0.479025	COc1ccc2c(=O)cc(CNC(=O)c3c(C)n(C)n(-c4ccccc4)c3=O)nc3cccc3c2n1	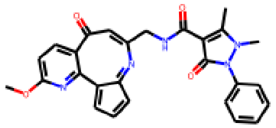	9.0	0.768132	3.608437	2.513170	5	0.823048
**2377**	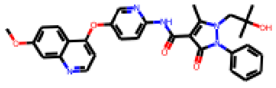	0.479025	COc1ccc2c(=O)cc(CNC(=O)c3c(C)n(C)n(-c4ccccc4)c3=O)nc3cccc3c2n1	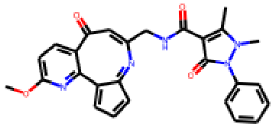	9.0	0.768132	3.608437	2.513170	5	0.823048
**2378**	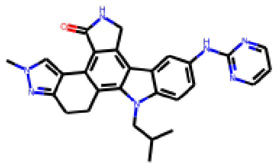	0.478986	Cc1ccc(C(=O)Nc2cccc(C(F)(C)F)c2C)cc1-c1ccc2nc(=O)cccc(F)nc2n1	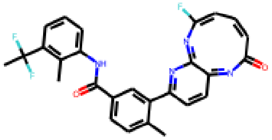	6.0	0.820294	3.038428	1.698172	4	0.833140
**2379**	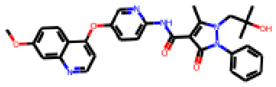	0.478953	COc1ccc2c(=O)cc(CNC(=O)c3c(C)n(C)n(-c4ccccc4)c3=O)nc3cccc3c2n1	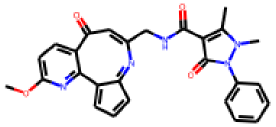	9.0	0.768132	3.608437	2.513170	5	0.823048

	src known mol	similarity	smiles	mol	Class	qed	logP	SAS	rings	kinase inhibition likeliness
**2380**	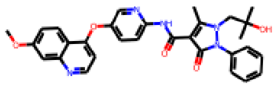	0.478953	COc1ccc2c(=O)cc(CNC(=O)c3c(C)n(C)n(-c4ccccc4)c3=O)nc3cccc3c2n1	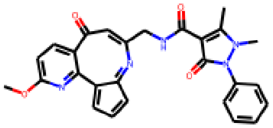	9.0	0.768132	3.608437	2.513170	5	0.823048
**2381**	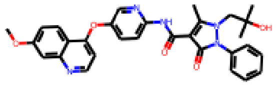	0.478942	COc1ccc2c(=O)cc(CNC(=O)c3c(C)n(C)n(-c4ccccc4)c3=O)nc3cccc2c3c1	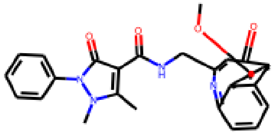	9.0	0.638163	3.995589	2.988471	5	0.774651
**2382**	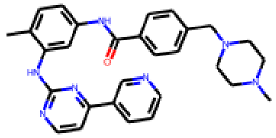	0.478849	Cc1ccc(NC(=O)c2ccccc2)cc1Nc1nccc(-c2cccnc2)n1	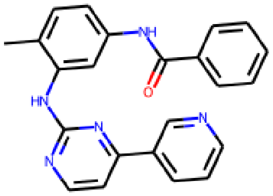	0.0	0.768132	3.608437	2.513170	4	0.854555
**2383**	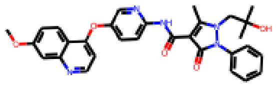	0.478754	COc1ccc2c(=O)cc(CNC(=O)c3c(C)n(C)n(-c4ccccc4)c3=O)nc3cccc3c2n1	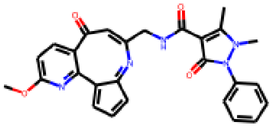	9.0	0.768132	3.608437	2.513170	5	0.823048

	src known mol	similarity	smiles	mol	Class	qed	logP	SAS	rings	kinase inhibition likeliness
**2384**	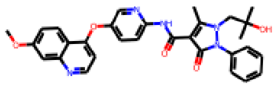	0.478706	COc1ccc2c(=O)cc(CNC(=O)c3c(C)n(C)n(-c4ccccc4)c3=O)nc3cccc2c3c1	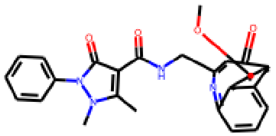	9.0	0.638163	3.995589	2.988471	5	0.774651
**2385**	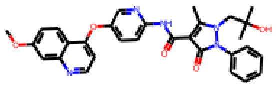	0.478705	COc1ccc2c(=O)cc(CNC(=O)c3c(C)n(C)n(-c4ccccc4)c3=O)nc3cccc3c2n1	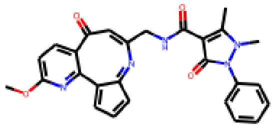	9.0	0.768132	3.608437	2.513170	5	0.823048
**2386**	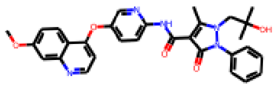	0.478705	COc1ccc2c(=O)cc(CNC(=O)c3c(C)n(C)n(-c4ccccc4)c3=O)nc3cccc3c2n1	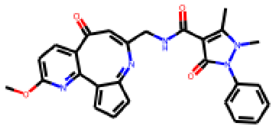	9.0	0.768132	3.608437	2.513170	5	0.823048
**2387**	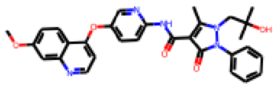	0.478463	COc1ccc2c(=O)cc(CNC(=O)c3c(C)n(C)n(-c4ccccc4)c3=O)nc3cccc3c2n1	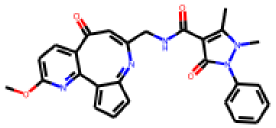	9.0	0.768132	3.608437	2.513170	5	0.823048

	src known mol	similarity	smiles	mol	Class	qed	logP	SAS	rings	kinase inhibition likeliness
**2388**	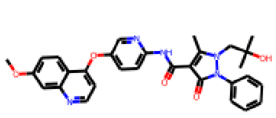	0.478451	COc1ccc2c(=O)cc(CNC(=O)c3c(C)n(C)n(-c4ccccc4)c3=O)nc3cccc2c3c1	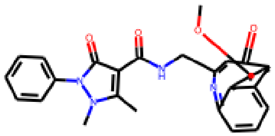	9.0	0.638163	3.995589	2.988471	5	0.774651
**2389**	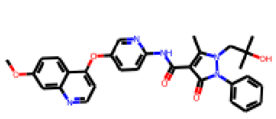	0.478451	COc1ccc2c(=O)cc(CNC(=O)c3c(C)n(C)n(-c4ccccc4)c3=O)nc3cccc2c3c1	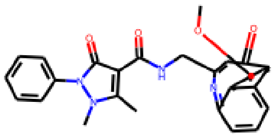	9.0	0.638163	3.995589	2.988471	5	0.774651
**2390**	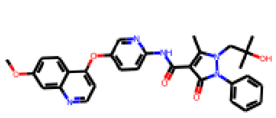	0.478451	COc1ccc2c(=O)cc(CNC(=O)c3c(C)n(C)n(-c4ccccc4)c3=O)nc3cccc2c3c1	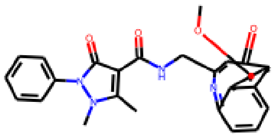	9.0	0.638163	3.995589	2.988471	5	0.774651
**2391**	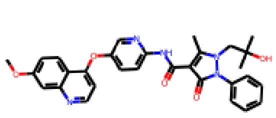	0.478354	COc1ccc2c(=O)cc(CNC(=O)c3c(C)n(C)n(-c4ccccc4)c3=O)nc3cccc2c3c1	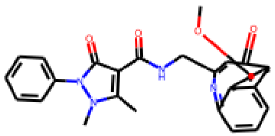	9.0	0.638163	3.995589	2.988471	5	0.774651

	src known mol	similarity	smiles	mol	Class	qed	logP	SAS	rings	kinase inhibition likeliness
**2392**	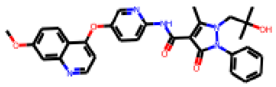	0.478235	COc1ccc2c(=O)cc(CNC(=O)c3c(C)n(C)n(-c4ccccc4)c3=O)nc3cccc3c2n1	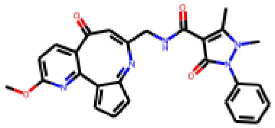	9.0	0.768132	3.608437	2.513170	5	0.823048
**2393**	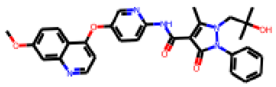	0.478144	COc1ccc2c(=O)cc(CNC(=O)c3c(C)n(C)n(-c4ccccc4)c3=O)nc3cccc2c3c1	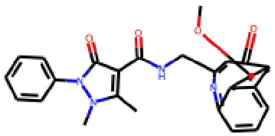	9.0	0.638163	3.995589	2.988471	5	0.774651
**2394**	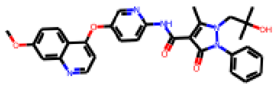	0.478144	COc1ccc2c(=O)cc(CNC(=O)c3c(C)n(C)n(-c4ccccc4)c3=O)nc3cccc2c3c1	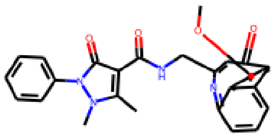	9.0	0.638163	3.995589	2.988471	5	0.774651
**2395**	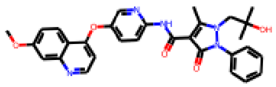	0.478144	COc1ccc2c(=O)cc(CNC(=O)c3c(C)n(C)n(-c4ccccc4)c3=O)nc3cccc2c3c1	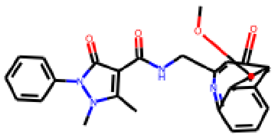	9.0	0.638163	3.995589	2.988471	5	0.774651

	src known mol	similarity	smiles	mol	Class	qed	logP	SAS	rings	kinase inhibition likeliness
**2396**	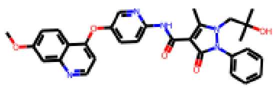	0.478144	COc1ccc2c(=O)cc(CNC(=O)c3c(C)n(C)n(-c4ccccc4)c3=O)nc3cccc2c3c1	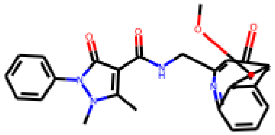	9.0	0.638163	3.995589	2.988471	5	0.774651
**2397**	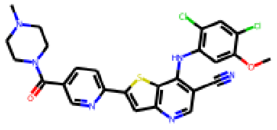	0.478008	CNC(=O)c1ccccc1Nc1cnc(-c2sc(N3CCOCC3)cc2C#N)cc1Cl	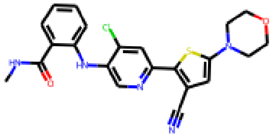	7.0	0.635315	4.542409	3.856266	4	0.763112
**2398**	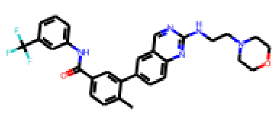	0.477978	Cc1ccc(C(=O)Nc2cccc(C(F)(C)F)c2C)cc1-c1ccc2nc(C)ccccc(F)nc2c1	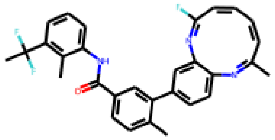	6.0	0.742375	4.384502	0.286234	4	0.779167
**2399**	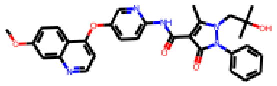	0.477891	COc1ccc2c(=O)cc(CNC(=O)c3c(C)n(C)n(-c4ccccc4)c3=O)nc3cccc3c2n1	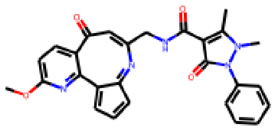	9.0	0.768132	3.608437	2.513170	5	0.823048

	src known mol	similarity	smiles	mol	Class	qed	logP	SAS	rings	kinase inhibition likeliness
**2400**	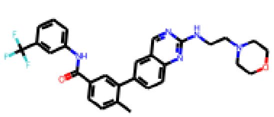	0.477818	Cc1ccc(C(=O)Nc2cccc(C(F)(C)F)c2C)cc1-c1ccc2nc(C)ccccc(F)nc2c1	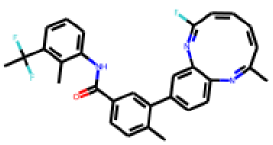	6.0	0.742375	4.384502	0.286234	4	0.779167
**2401**	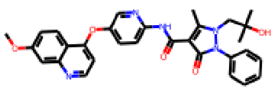	0.477707	COc1ccc2c(=O)cc(CNC(=O)c3c(C)n(C)n(-c4ccccc4)c3=O)nc3cccc3c2n1	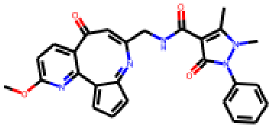	9.0	0.768132	3.608437	2.513170	5	0.823048
**2402**	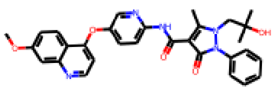	0.477646	COc1ccc2c(=O)cc(CNC(=O)c3c(C)n(C)n(-c4ccccc4)c3=O)nc3cccc3c2n1	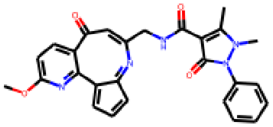	9.0	0.768132	3.608437	2.513170	5	0.823048
**2403**	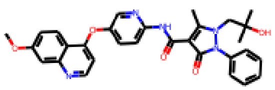	0.477604	COc1ccc2c(=O)cc(CNC(=O)c3c(C)n(C)n(-c4ccccc4)c3=O)nc3cccc2c3c1	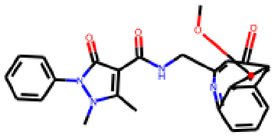	9.0	0.638163	3.995589	2.988471	5	0.774651

	src known mol	similarity	smiles	mol	Class	qed	logP	SAS	rings	kinase inhibition likeliness
**2404**	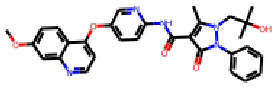	0.477587	COc1ccc2c(=O)cc(CNC(=O)c3c(C)n(C)n(-c4ccccc4)c3=O)nc3cccc2c3c1	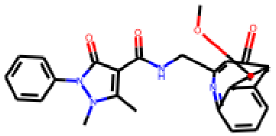	9.0	0.638163	3.995589	2.988471	5	0.774651
**2405**	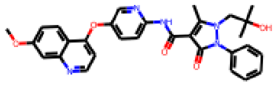	0.477580	COc1ccc2c(=O)cc(CNC(=O)c3c(C)n(C)n(-c4ccccc4)c3=O)nc3cccc2c3c1	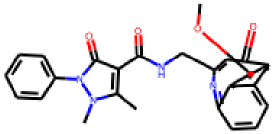	9.0	0.638163	3.995589	2.988471	5	0.774651
**2406**	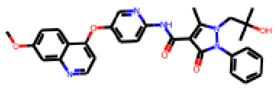	0.477185	COc1ccc2c(=O)cc(CNC(=O)c3c(C)n(C)n(-c4ccccc4)c3=O)nc3cccc2c3c1	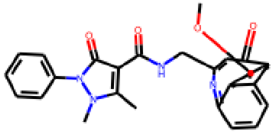	9.0	0.638163	3.995589	2.988471	5	0.774651
**2407**	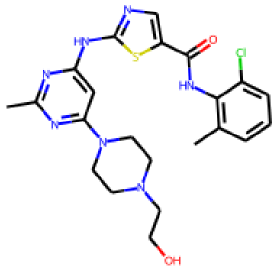	0.477170	Cc1nc(Nc2ncc(C(=O)Oc3c(C)cccc3Cl)s2)cc(N2CCNC(=O)C2=O)n1	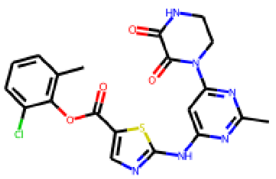	2.0	0.771867	2.951597	1.935530	4	0.787643

	src known mol	similarity	smiles	mol	Class	qed	logP	SAS	rings	kinase inhibition likeliness
**2408**	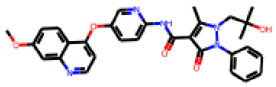	0.476949	COc1ccc2c(=O)cc(CNC(=O)c3c(C)n(C)n(-c4ccccc4)c3=O)nc3cccc3c2n1	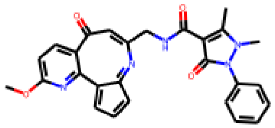	9.0	0.768132	3.608437	2.513170	5	0.823048
**2409**	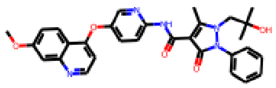	0.476949	COc1ccc2c(=O)cc(CNC(=O)c3c(C)n(C)n(-c4ccccc4)c3=O)nc3cccc3c2n1	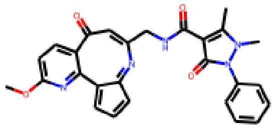	9.0	0.768132	3.608437	2.513170	5	0.823048
**2410**	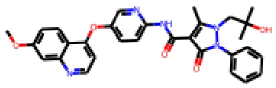	0.476949	COc1ccc2c(=O)cc(CNC(=O)c3c(C)n(C)n(-c4ccccc4)c3=O)nc3cccc3c2n1	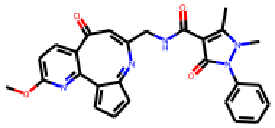	9.0	0.768132	3.608437	2.513170	5	0.823048
**2411**	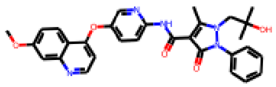	0.476949	COc1ccc2c(=O)cc(CNC(=O)c3c(C)n(C)n(-c4ccccc4)c3=O)nc3cccc3c2n1	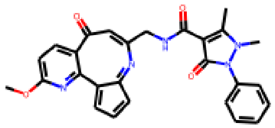	9.0	0.768132	3.608437	2.513170	5	0.823048

	src known mol	similarity	smiles	mol	Class	qed	logP	SAS	rings	kinase inhibition likeliness
**2412**	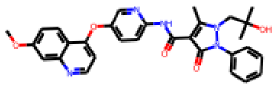	0.476757	COc1ccc2c(=O)cc(CNC(=O)c3c(C)n(C)n(-c4ccccc4)c3=O)nc3cccc3c2n1	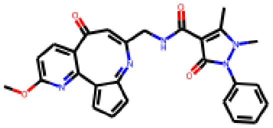	9.0	0.768132	3.608437	2.513170	5	0.823048
**2413**	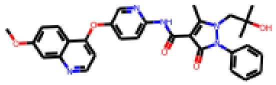	0.476757	COc1ccc2c(=O)cc(CNC(=O)c3c(C)n(C)n(-c4ccccc4)c3=O)nc3cccc3c2n1	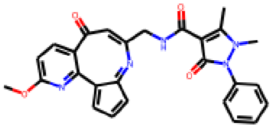	9.0	0.768132	3.608437	2.513170	5	0.823048
**2414**	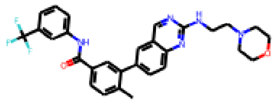	0.476556	Cc1ccc(C(=O)Nc2cccc(C(F)(C)F)c2C)cc1-c1ccc2nc(C)ccccc(F)nc2c1	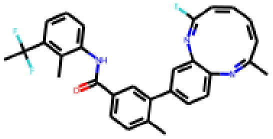	6.0	0.742375	4.384502	0.286234	4	0.779167
**2415**	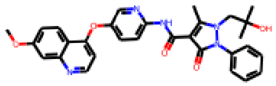	0.476514	COc1ccc2c(=O)cc(CNC(=O)c3c(C)n(C)n(-c4ccccc4)c3=O)nc3cccc3c2n1	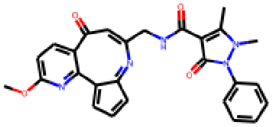	9.0	0.768132	3.608437	2.513170	5	0.823048

	src known mol	similarity	smiles	mol	Class	qed	logP	SAS	rings	kinase inhibition likeliness
**2416**	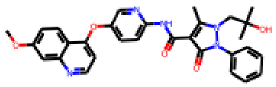	0.476395	COc1ccc2c(=O)cc(CNC(=O)c3c(C)n(C)n(-c4ccccc4)c3=O)nc3cccc2c3c1	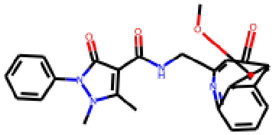	9.0	0.638163	3.995589	2.988471	5	0.774651
**2417**	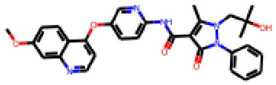	0.476319	COc1ccc2c(=O)cc(CNC(=O)c3c(C)n(C)n(-c4ccccc4)c3=O)nc3cccc2c3c1	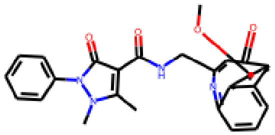	9.0	0.638163	3.995589	2.988471	5	0.774651
**2418**	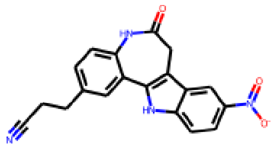	0.476160	CCNC(=O)N1CCC(Nc2n(C(C)c3c(-c4ccccc4)[nH]c4ccccc34)n2)CC1	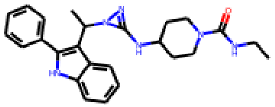	7.0	0.623584	3.603734	4.478009	5	0.804049
**2419**	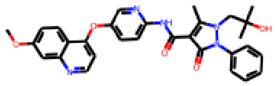	0.475959	COc1ccc2c(=O)cc(CNC(=O)c3c(C)n(C)n(-c4ccccc4)c3=O)nc3cccc2c3c1	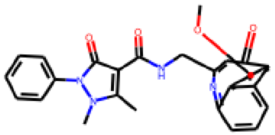	9.0	0.638163	3.995589	2.988471	5	0.774651

	src known mol	similarity	smiles	mol	Class	qed	logP	SAS	rings	kinase inhibition likeliness
**2420**	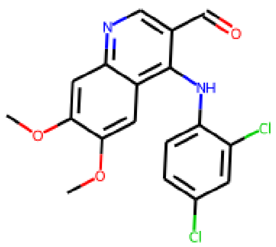	0.475921	COc1cc2ncc(C(=O)O)c(Nc3cccc(Cl)c3Cl)c2cc1OC1C(C)C1	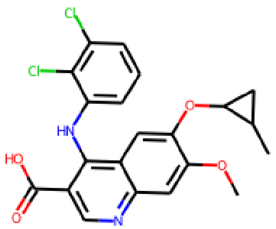	2.0	0.623362	2.525411	2.997881	4	0.774857
**2421**	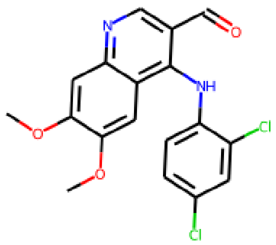	0.475921	COc1cc2ncc(C(=O)O)c(Nc3cccc(Cl)c3Cl)c2cc1OC1C(C)C1	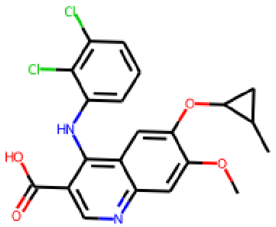	2.0	0.623362	2.525411	2.997881	4	0.774857
**2422**	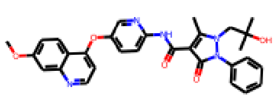	0.475811	COc1ccc2c(=O)cc(CNC(=O)c3c(C)n(C)n(-c4ccccc4)c3=O)nc3cccc3c2n1	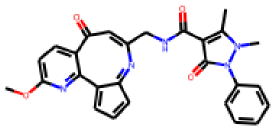	9.0	0.768132	3.608437	2.513170	5	0.823048
**2423**	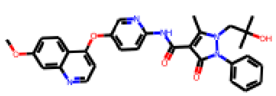	0.475780	COc1ccc2c(=O)cc(CNC(=O)c3c(C)n(C)n(-c4ccccc4)c3=O)nc3cccc2c3c1	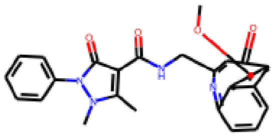	9.0	0.638163	3.995589	2.988471	5	0.774651

	src known mol	similarity	smiles	mol	Class	qed	logP	SAS	rings	kinase inhibition likeliness
**2424**	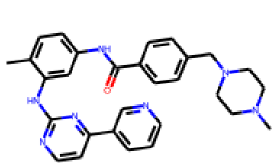	0.475755	Cc1ccc(NC(=O)c2ccccc2)cc1Nc1nccc(-c2cccnc2)n1	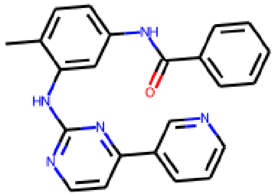	0.0	0.768132	3.608437	2.513170	4	0.854555
**2425**	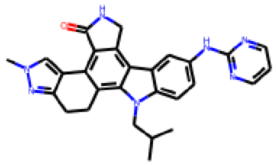	0.475564	C#Cc1ccc(C(=O)OC)cc1-c1C(=O)c-n2nc(-c3ccc(F)cc3F)cc2C1	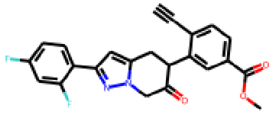	8.0	0.768132	3.608437	2.513170	4	0.763730
**2426**	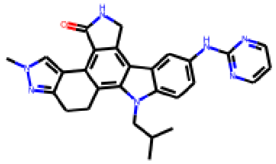	0.475564	C#Cc1ccc(C(=O)OC)cc1-c1C(=O)c-n2nc(-c3ccc(F)cc3F)cc2C1	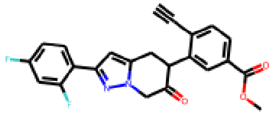	8.0	0.768132	3.608437	2.513170	4	0.763730
**2427**	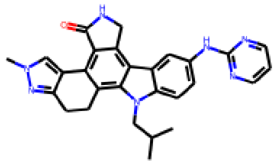	0.475564	C#Cc1ccc(C(=O)OC)cc1-c1C(=O)c-n2nc(-c3ccc(F)cc3F)cc2C1	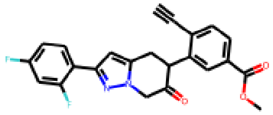	8.0	0.768132	3.608437	2.513170	4	0.763730

	src known mol	similarity	smiles	mol	Class	qed	logP	SAS	rings	kinase inhibition likeliness
**2428**	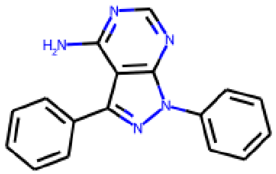	0.475474	Cc1ccc(C(=O)Oc2ccon2)cc1-c1ncnc2c1cnn2-c1ccccc1	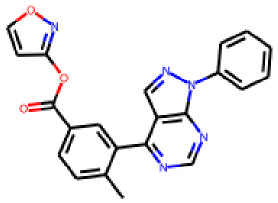	8.0	0.635315	4.542409	3.856266	5	0.760056
**2429**	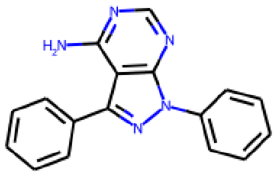	0.475474	Cc1ccc(C(=O)Oc2ccon2)cc1-c1ncnc2c1cnn2-c1ccccc1	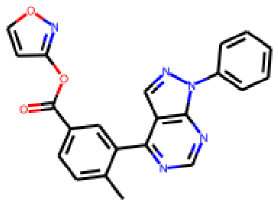	8.0	0.635315	4.542409	3.856266	5	0.760056
**2430**	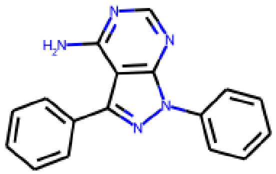	0.475474	Cc1ccc(C(=O)Oc2ccon2)cc1-c1ncnc2c1cnn2-c1ccccc1	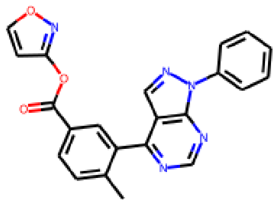	8.0	0.635315	4.542409	3.856266	5	0.760056
**2431**	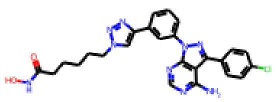	0.475321	C1cccc(C(=O)Nc2cscn2)cc1Nc1ncnc2c1cnn2-c1ccccc1	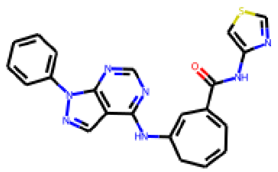	8.0	0.591949	3.050396	3.414230	5	0.770627

	src known mol	similarity	smiles	mol	Class	qed	logP	SAS	rings	kinase inhibition likeliness
**2432**	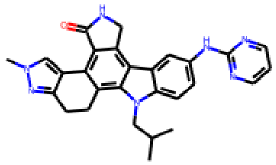	0.475248	Cc1ccc(C(=O)Nc2cccc(C(F)(C)F)c2C)cc1-c1ccc2nc(=O)cccc(F)nc2n1	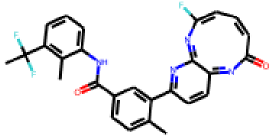	6.0	0.820294	3.038428	1.698172	4	0.833140
**2433**	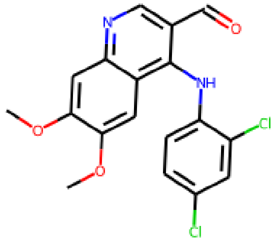	0.475212	COc1cc2ncc(C(=O)O)c(Nc3cccc(Cl)c3Cl)c2cc1OC1C(C)C1	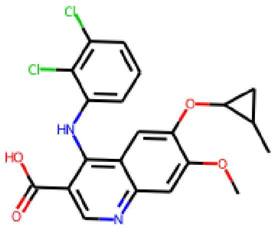	2.0	0.623362	2.525411	2.997881	4	0.774857
**2434**	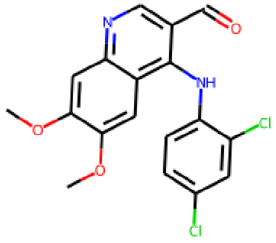	0.475212	COc1cc2ncc(C(=O)O)c(Nc3cccc(Cl)c3Cl)c2cc1OC1C(C)C1	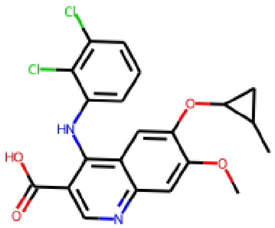	2.0	0.623362	2.525411	2.997881	4	0.774857
**2435**	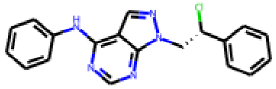	0.475081	Cc1ccc(C(=O)Nc2ccon2)cc1Nc1ncnc2c1cnn2C1cccccc1	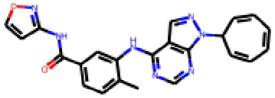	8.0	0.623362	2.525411	2.997881	5	0.770298

	src known mol	similarity	smiles	mol	Class	qed	logP	SAS	rings	kinase inhibition likeliness
**2436**	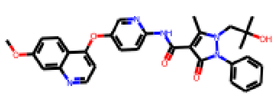	0.475027	COc1ccc2c(=O)cc(CNC(=O)c3c(C)n(C)n(-c4ccccc4)c3=O)nc3cccc2c3c1	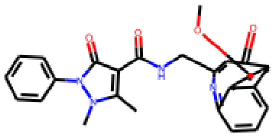	9.0	0.638163	3.995589	2.988471	5	0.774651
**2437**	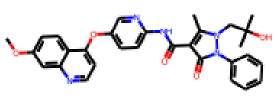	0.474933	COc1ccc2c(=O)cc(CNC(=O)c3c(C)n(C)n(-c4ccccc4)c3=O)nc3cccc2c3c1	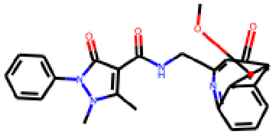	9.0	0.638163	3.995589	2.988471	5	0.774651
**2438**	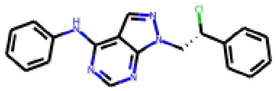	0.474777	Cc1ccc(C(=O)Nc2ccon2)cc1Nc1ncnc2c1cnn2C1cccccc1	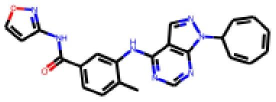	8.0	0.623362	2.525411	2.997881	5	0.770298
**2439**	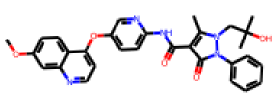	0.474540	COc1ccc2c(=O)cc(CNC(=O)c3c(C)n(C)n(-c4ccccc4)c3=O)nc3cccc2c3c1	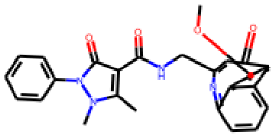	9.0	0.638163	3.995589	2.988471	5	0.774651

	src known mol	similarity	smiles	mol	Class	qed	logP	SAS	rings	kinase inhibition likeliness
**2440**	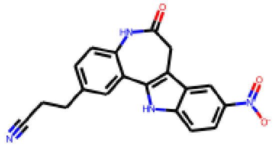	0.474476	CCNC(=O)N1CCC(Nc2n(C(C)c3c(-c4ccccc4)[nH]c4ccccc34)n2)CC1	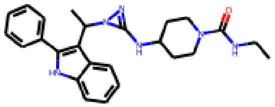	7.0	0.623584	3.603734	4.478009	5	0.804049
**2441**	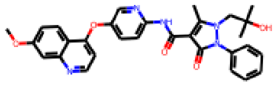	0.474337	COc1ccc2c(=O)cc(CNC(=O)c3c(C)n(C)n(-c4ccccc4)c3=O)nc3cccc3c2n1	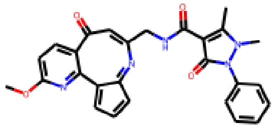	9.0	0.768132	3.608437	2.513170	5	0.823048
**2442**	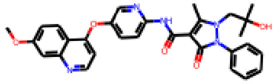	0.474199	COc1ccc2c(=O)cc(CNC(=O)c3c(C)n(C)n(-c4ccccc4)c3=O)nc3cccc2c3c1	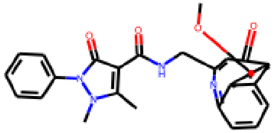	9.0	0.638163	3.995589	2.988471	5	0.774651
**2443**	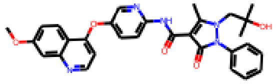	0.474199	COc1ccc2c(=O)cc(CNC(=O)c3c(C)n(C)n(-c4ccccc4)c3=O)nc3cccc2c3c1	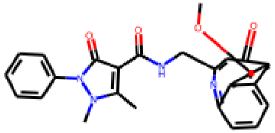	9.0	0.638163	3.995589	2.988471	5	0.774651

	src known mol	similarity	smiles	mol	Class	qed	logP	SAS	rings	kinase inhibition likeliness
**2444**	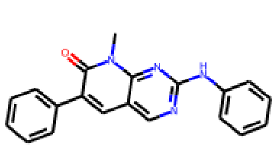	0.474164	Clc1cccc(Nc2ncc3cc(-c3c(Cl)cccc3Cl)c(=O)n(C)c3n2)c1	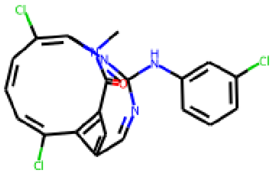	2.0	0.768132	3.608437	2.513170	5	0.784634
**2445**	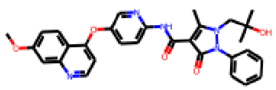	0.473864	COc1ccc2c(=O)cc(CNC(=O)c3c(C)n(C)n(-c4ccccc4)c3=O)nc3cccc3c2n1	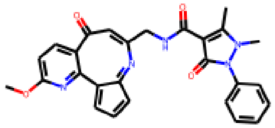	9.0	0.768132	3.608437	2.513170	5	0.823048
**2446**	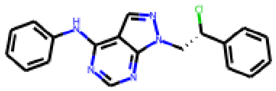	0.473684	Cc1ccc(C(=O)Nc2ccon2)cc1Nc1ncnc2c1cnn2C1cccccc1	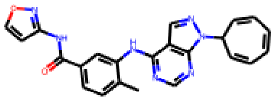	8.0	0.623362	2.525411	2.997881	5	0.770298
**2447**	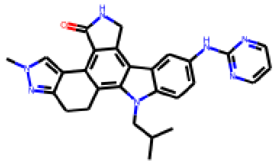	0.473684	Cc1ccc(C(=O)Nc2cccc(C(F)(F)C)c2C)cc1-c1ccc2nc(=O)cccc(F)nc2n1	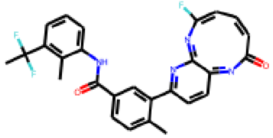	6.0	0.695894	2.840371	2.841045	4	0.823833

	src known mol	similarity	smiles	mol	Class	qed	logP	SAS	rings	kinase inhibition likeliness
**2448**	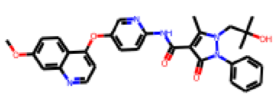	0.473656	COc1ccc2c(=O)cc(CNC(=O)c3c(C)n(C)n(-c4ccccc4)c3=O)nc3cccc2c3c1	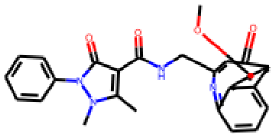	9.0	0.638163	3.995589	2.988471	5	0.774651
**2449**	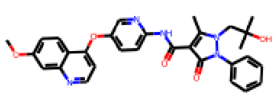	0.473656	COc1ccc2c(=O)cc(CNC(=O)c3c(C)n(C)n(-c4ccccc4)c3=O)nc3cccc2c3c1	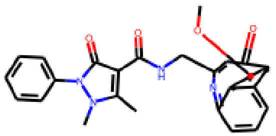	9.0	0.638163	3.995589	2.988471	5	0.774651
**2450**	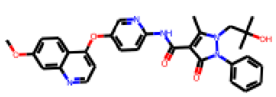	0.473174	COc1ccc2c(=O)cc(CNC(=O)c3c(C)n(C)n(-c4ccccc4)c3=O)nc3cccc3c2n1	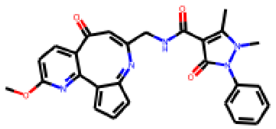	9.0	0.768132	3.608437	2.513170	5	0.823048
**2451**	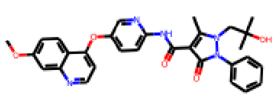	0.473174	COc1ccc2c(=O)cc(CNC(=O)c3c(C)n(C)n(-c4ccccc4)c3=O)nc3cccc3c2n1	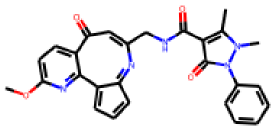	9.0	0.768132	3.608437	2.513170	5	0.823048

	src known mol	similarity	smiles	mol	Class	qed	logP	SAS	rings	kinase inhibition likeliness
**2452**	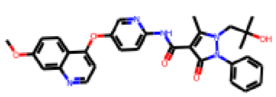	0.473174	COc1ccc2c(=O)cc(CNC(=O)c3c(C)n(C)n(-c4ccccc4)c3=O)nc3cccc3c2n1	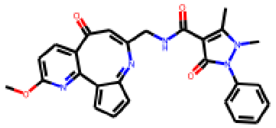	9.0	0.768132	3.608437	2.513170	5	0.823048
**2453**	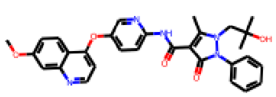	0.473174	COc1ccc2c(=O)cc(CNC(=O)c3c(C)n(C)n(-c4ccccc4)c3=O)nc3cccc3c2n1	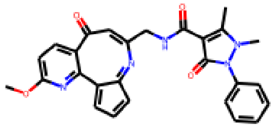	9.0	0.768132	3.608437	2.513170	5	0.823048
**2454**	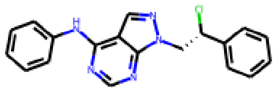	0.472991	Cc1ccc(C(=O)Nc2ccon2)cc1Nc1ncnc2c1cnn2C1cccccc1	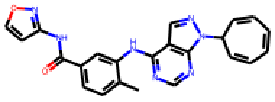	8.0	0.623362	2.525411	2.997881	5	0.770298
**2455**	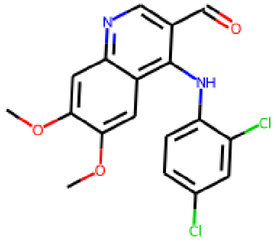	0.472727	COc1cc2ncc(C(=O)O)c(Nc3cccc(Cl)c3Cl)c2cc1OC1C(C)C1	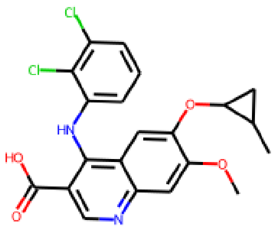	2.0	0.623362	2.525411	2.997881	4	0.774857

	src known mol	similarity	smiles	mol	Class	qed	logP	SAS	rings	kinase inhibition likeliness
**2456**	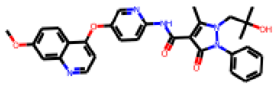	0.472658	COc1ccc2c(=O)cc(CNC(=O)c3c(C)n(C)n(-c4ccccc4)c3=O)nc3cccc2c3c1	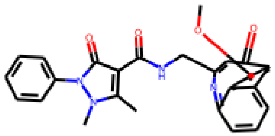	9.0	0.638163	3.995589	2.988471	5	0.774651
**2457**	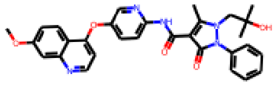	0.472552	COc1ccc2c(=O)cc(CNC(=O)c3c(C)n(C)n(-c4ccccc4)c3=O)nc3cccc3c2n1	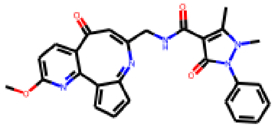	9.0	0.768132	3.608437	2.513170	5	0.823048
**2458**	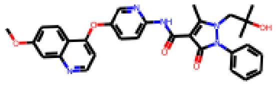	0.472552	COc1ccc2c(=O)cc(CNC(=O)c3c(C)n(C)n(-c4ccccc4)c3=O)nc3cccc3c2n1	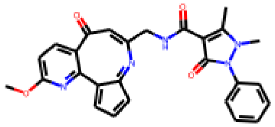	9.0	0.768132	3.608437	2.513170	5	0.823048
**2459**	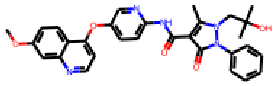	0.472539	COc1ccc2c(=O)cc(CNC(=O)c3c(C)n(C)n(-c4ccccc4)c3=O)nc3cccc2c3c1	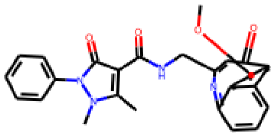	9.0	0.638163	3.995589	2.988471	5	0.774651

	src known mol	similarity	smiles	mol	Class	qed	logP	SAS	rings	kinase inhibition likeliness
**2460**	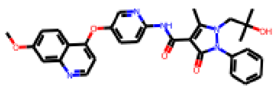	0.472428	COc1ccc2c(=O)cc(CNC(=O)c3c(C)n(C)n(-c4ccccc4)c3=O)nc3cccc3c2n1	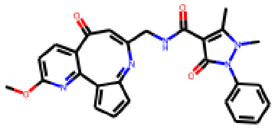	9.0	0.768132	3.608437	2.513170	5	0.823048
**2461**	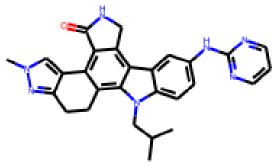	0.472222	Cc1ccc(C(=O)Nc2cccc(C(F)(F)C)c2C)cc1-c1ccc2nc(=O)cccc(F)nc2n1	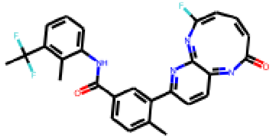	6.0	0.695894	2.840371	2.841045	4	0.823833
**2462**	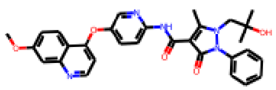	0.471831	COc1ccc2c(=O)cc(CNC(=O)c3c(C)n(C)n(-c4ccccc4)c3=O)nc3cccc2c3c1	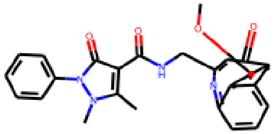	9.0	0.638163	3.995589	2.988471	5	0.774651
**2463**	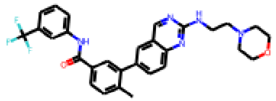	0.471338	Cc1ccc(C(=O)Nc2cccc(C(F)(C)F)c2C)cc1-c1ccc2nc(C)ccccc(F)nc2c1	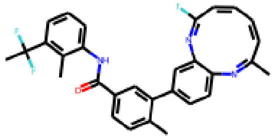	6.0	0.742375	4.384502	0.286234	4	0.779167

	src known mol	similarity	smiles	mol	Class	qed	logP	SAS	rings	kinase inhibition likeliness
**2464**	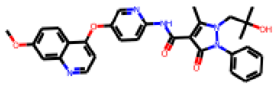	0.471258	COc1ccc2c(=O)cc(CNC(=O)c3c(C)n(C)n(-c4ccccc4)c3=O)nc3cccc2c3c1	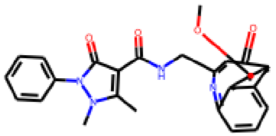	9.0	0.638163	3.995589	2.988471	5	0.774651
**2465**	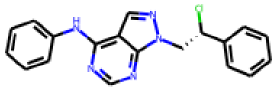	0.471019	Cc1ccc(C(=O)Nc2ccon2)cc1Nc1ncnc2c1cnn2C1cccccc1	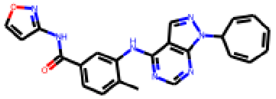	8.0	0.623362	2.525411	2.997881	5	0.770298
**2466**	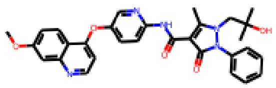	0.470957	COc1ccc2c(=O)cc(CNC(=O)c3c(C)n(C)n(-c4ccccc4)c3=O)nc3cccc3c2n1	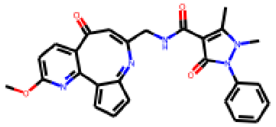	9.0	0.768132	3.608437	2.513170	5	0.823048
**2467**	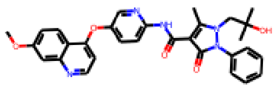	0.470957	COc1ccc2c(=O)cc(CNC(=O)c3c(C)n(C)n(-c4ccccc4)c3=O)nc3cccc3c2n1	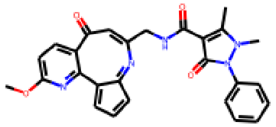	9.0	0.768132	3.608437	2.513170	5	0.823048

	src known mol	similarity	smiles	mol	Class	qed	logP	SAS	rings	kinase inhibition likeliness
**2468**	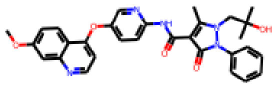	0.470888	COc1ccc2c(=O)cc(CNC(=O)c3c(C)n(C)n(-c4ccccc4)c3=O)nc3cccc3c2n1	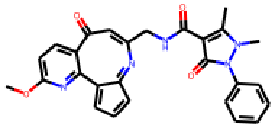	9.0	0.768132	3.608437	2.513170	5	0.823048
**2469**	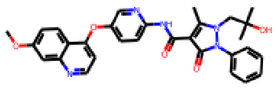	0.470888	COc1ccc2c(=O)cc(CNC(=O)c3c(C)n(C)n(-c4ccccc4)c3=O)nc3cccc3c2n1	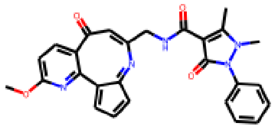	9.0	0.768132	3.608437	2.513170	5	0.823048
**2470**	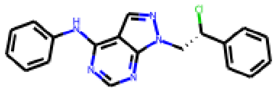	0.470668	Cc1ccc(C(=O)Nc2ccon2)cc1Nc1ncnc2c1cnn2C1cccccc1	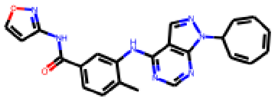	8.0	0.623362	2.525411	2.997881	5	0.770298
**2471**	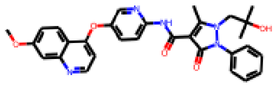	0.470621	COc1ccc2c(=O)cc(CNC(=O)c3c(C)n(C)n(-c4ccccc4)c3=O)nc3cccc3c2n1	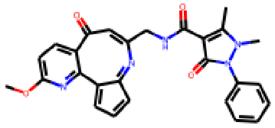	9.0	0.768132	3.608437	2.513170	5	0.823048

	src known mol	similarity	smiles	mol	Class	qed	logP	SAS	rings	kinase inhibition likeliness
**2472**	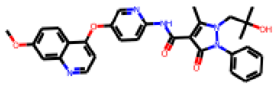	0.470621	COc1ccc2c(=O)cc(CNC(=O)c3c(C)n(C)n(-c4ccccc4)c3=O)nc3cccc3c2n1	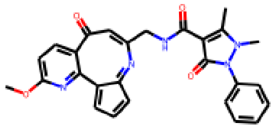	9.0	0.768132	3.608437	2.513170	5	0.823048
**2473**	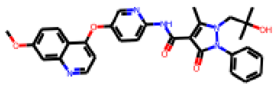	0.470620	COc1ccc2c(=O)cc(CNC(=O)c3c(C)n(C)n(-c4ccccc4)c3=O)nc3cccc2c3c1	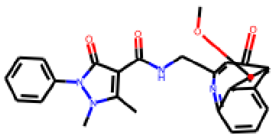	9.0	0.638163	3.995589	2.988471	5	0.774651
**2474**	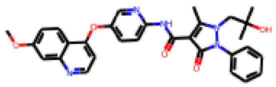	0.470387	COc1ccc2c(=O)cc(CNC(=O)c3c(C)n(C)n(-c4ccccc4)c3=O)nc3cccc3c2n1	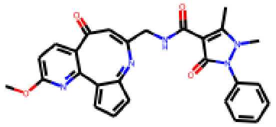	9.0	0.768132	3.608437	2.513170	5	0.823048
**2475**	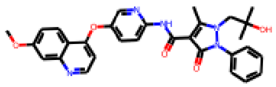	0.470387	COc1ccc2c(=O)cc(CNC(=O)c3c(C)n(C)n(-c4ccccc4)c3=O)nc3cccc3c2n1	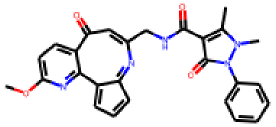	9.0	0.768132	3.608437	2.513170	5	0.823048

	src known mol	similarity	smiles	mol	Class	qed	logP	SAS	rings	kinase inhibition likeliness
**2476**	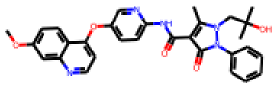	0.470387	COc1ccc2c(=O)cc(CNC(=O)c3c(C)n(C)n(-c4ccccc4)c3=O)nc3cccc3c2n1	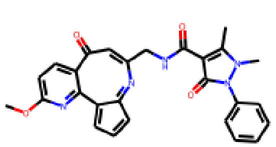	9.0	0.768132	3.608437	2.513170	5	0.823048
**2477**	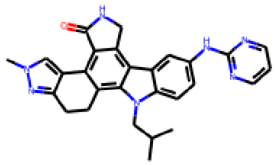	0.470331	C#Cc1ccc(C(=O)OC)cc1-c1C(=O)c-n2nc(-c3ccc(F)cc3F)cc2C1	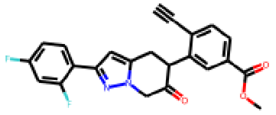	8.0	0.768132	3.608437	2.513170	4	0.763730
**2478**	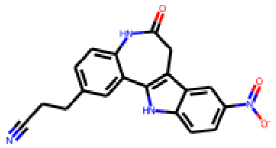	0.469746	CCNC(=O)N1CCC(Nc2n(C(C)c3c(-c4ccccc4)[nH]c4ccccc34)n2)CC1	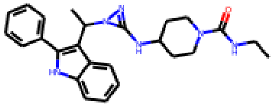	7.0	0.623584	3.603734	4.478009	5	0.804049
**2479**	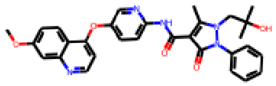	0.469649	COc1ccc2c(=O)cc(CNC(=O)c3c(C)n(C)n(-c4ccccc4)c3=O)nc3cccc2c3c1	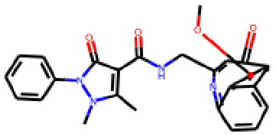	9.0	0.638163	3.995589	2.988471	5	0.774651

	src known mol	similarity	smiles	mol	Class	qed	logP	SAS	rings	kinase inhibition likeliness
**2480**	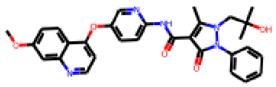	0.469560	COc1ccc2c(=O)cc(CNC(=O)c3c(C)n(C)n(-c4ccccc4)c3=O)nc3cccc3c2n1	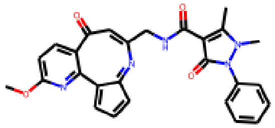	9.0	0.768132	3.608437	2.513170	5	0.823048
**2481**	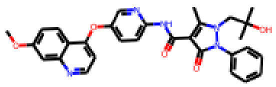	0.469516	COc1ccc2c(=O)cc(CNC(=O)c3c(C)n(C)n(-c4ccccc4)c3=O)nc3cccc3c2n1	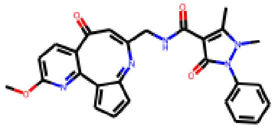	9.0	0.768132	3.608437	2.513170	5	0.823048
**2482**	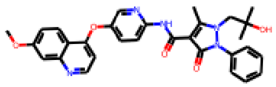	0.469516	COc1ccc2c(=O)cc(CNC(=O)c3c(C)n(C)n(-c4ccccc4)c3=O)nc3cccc3c2n1	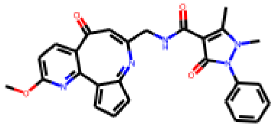	9.0	0.768132	3.608437	2.513170	5	0.823048
**2483**	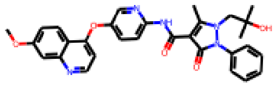	0.469466	COc1ccc2c(=O)cc(CNC(=O)c3c(C)n(C)n(-c4ccccc4)c3=O)nc3cccc2c3c1	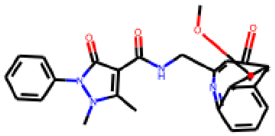	9.0	0.638163	3.995589	2.988471	5	0.774651

	src known mol	similarity	smiles	mol	Class	qed	logP	SAS	rings	kinase inhibition likeliness
**2484**	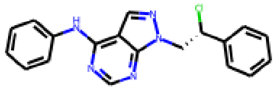	0.469375	Cc1ccc(C(=O)Nc2ccon2)cc1Nc1ncnc2c1cnn2C1cccccc1	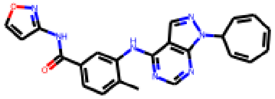	8.0	0.623362	2.525411	2.997881	5	0.770298
**2485**	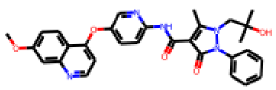	0.469298	COc1ccc2c(=O)cc(CNC(=O)c3c(C)n(C)n(-c4ccccc4)c3=O)nc3cccc2c3c1	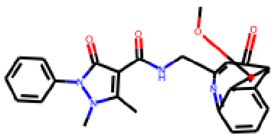	9.0	0.638163	3.995589	2.988471	5	0.774651
**2486**	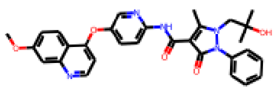	0.469298	COc1ccc2c(=O)cc(CNC(=O)c3c(C)n(C)n(-c4ccccc4)c3=O)nc3cccc2c3c1	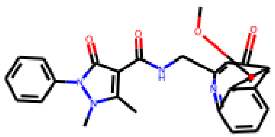	9.0	0.638163	3.995589	2.988471	5	0.774651
**2487**	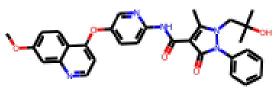	0.469210	COc1ccc2c(=O)cc(CNC(=O)c3c(C)n(C)n(-c4ccccc4)c3=O)nc3cccc2c3c1	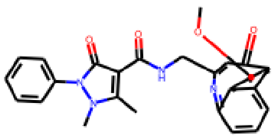	9.0	0.638163	3.995589	2.988471	5	0.774651

	src known mol	similarity	smiles	mol	Class	qed	logP	SAS	rings	kinase inhibition likeliness
**2488**	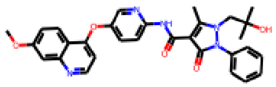	0.469210	COc1ccc2c(=O)cc(CNC(=O)c3c(C)n(C)n(-c4ccccc4)c3=O)nc3cccc2c3c1	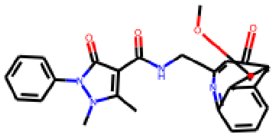	9.0	0.638163	3.995589	2.988471	5	0.774651
**2489**	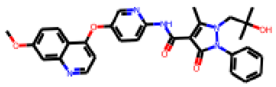	0.469210	COc1ccc2c(=O)cc(CNC(=O)c3c(C)n(C)n(-c4ccccc4)c3=O)nc3cccc2c3c1	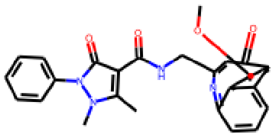	9.0	0.638163	3.995589	2.988471	5	0.774651
**2490**	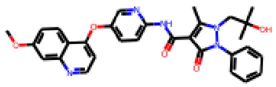	0.469210	COc1ccc2c(=O)cc(CNC(=O)c3c(C)n(C)n(-c4ccccc4)c3=O)nc3cccc2c3c1	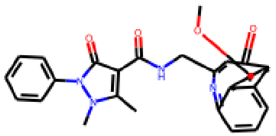	9.0	0.638163	3.995589	2.988471	5	0.774651
**2491**	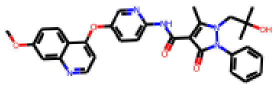	0.469210	COc1ccc2c(=O)cc(CNC(=O)c3c(C)n(C)n(-c4ccccc4)c3=O)nc3cccc2c3c1	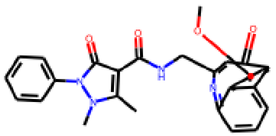	9.0	0.638163	3.995589	2.988471	5	0.774651

	src known mol	similarity	smiles	mol	Class	qed	logP	SAS	rings	kinase inhibition likeliness
**2492**	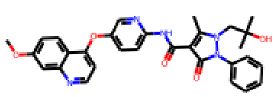	0.469210	COc1ccc2c(=O)cc(CNC(=O)c3c(C)n(C)n(-c4ccccc4)c3=O)nc3cccc2c3c1	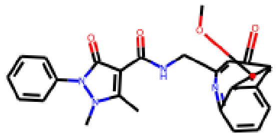	9.0	0.638163	3.995589	2.988471	5	0.774651
**2493**	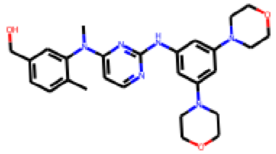	0.469136	NC(=O)c1ccccc1Nc1nc(Nc2ccc(N3CCOCC3)cc2)ncc1Cl	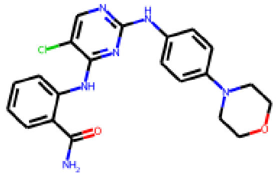	7.0	0.768132	3.608437	2.513170	4	0.755040
**2494**	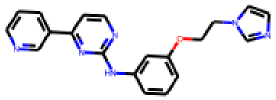	0.469091	Cc1ccc(OC(=O)c2ccccc2)cc1Nc1nccc(-c2cccnc2)n1	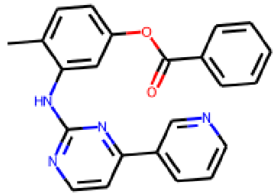	0.0	0.638163	3.995589	2.988471	4	0.775468
**2495**	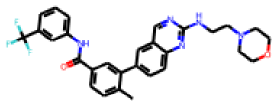	0.469042	Cc1ccc(C(=O)Oc2cccc(C(F)(F)F)c2)cc1-c1ccc2nc(N)ncc2c1	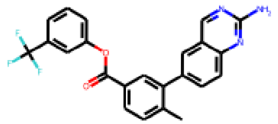	6.0	0.638163	3.995589	2.988471	4	0.789898

	src known mol	similarity	smiles	mol	Class	qed	logP	SAS	rings	kinase inhibition likeliness
**2496**	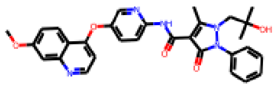	0.468997	COc1ccc2c(=O)cc(CNC(=O)c3c(C)n(C)n(-c4ccccc4)c3=O)nc3cccc3c2n1	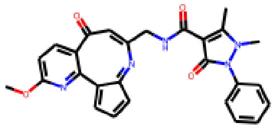	9.0	0.768132	3.608437	2.513170	5	0.823048
**2497**	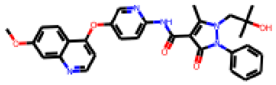	0.468902	COc1ccc2c(=O)cc(CNC(=O)c3c(C)n(C)n(-c4ccccc4)c3=O)nc3cccc2c3c1	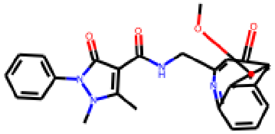	9.0	0.638163	3.995589	2.988471	5	0.774651
**2498**	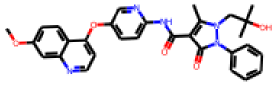	0.468902	COc1ccc2c(=O)cc(CNC(=O)c3c(C)n(C)n(-c4ccccc4)c3=O)nc3cccc2c3c1	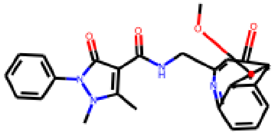	9.0	0.638163	3.995589	2.988471	5	0.774651
**2499**	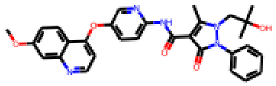	0.468902	COc1ccc2c(=O)cc(CNC(=O)c3c(C)n(C)n(-c4ccccc4)c3=O)nc3cccc2c3c1	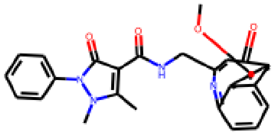	9.0	0.638163	3.995589	2.988471	5	0.774651

	src known mol	similarity	smiles	mol	Class	qed	logP	SAS	rings	kinase inhibition likeliness
**2500**	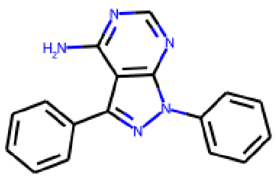	0.468896	Cc1ccc(C(=O)Oc2ccon2)cc1-c1ncnc2c1cnn2-c1ccccc1	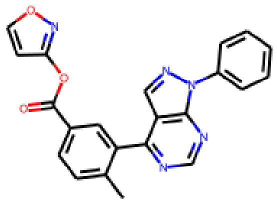	8.0	0.635315	4.542409	3.856266	5	0.760056
**2501**	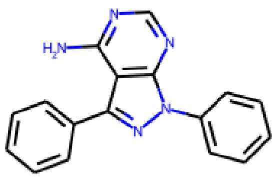	0.468896	Cc1ccc(C(=O)Oc2ccon2)cc1-c1ncnc2c1cnn2-c1ccccc1	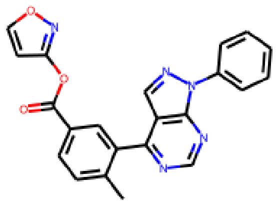	8.0	0.635315	4.542409	3.856266	5	0.760056
**2502**	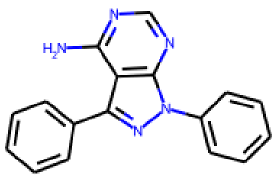	0.468896	Cc1ccc(C(=O)Oc2ccon2)cc1-c1ncnc2c1cnn2-c1ccccc1	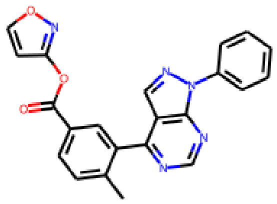	8.0	0.635315	4.542409	3.856266	5	0.760056
**2503**	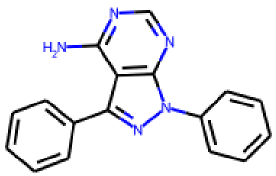	0.468893	Cc1ccc(C(=O)Oc2ccon2)cc1-c1ncnc2c1cnn2-c1ccccc1	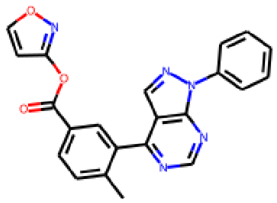	8.0	0.635315	4.542409	3.856266	5	0.760056

	src known mol	similarity	smiles	mol	Class	qed	logP	SAS	rings	kinase inhibition likeliness
**2504**	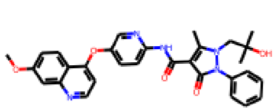	0.468512	COc1ccc2c(=O)cc(CNC(=O)c3c(C)n(C)n(-c4ccccc4)c3=O)nc3cccc2c3c1	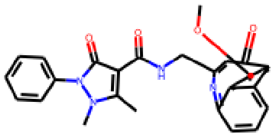	9.0	0.638163	3.995589	2.988471	5	0.774651
**2505**	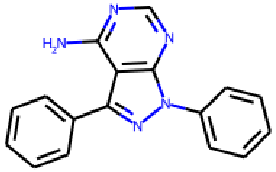	0.468475	Cc1ccc(C(=O)Oc2ccon2)cc1-c1ncnc2c1cnn2-c1ccccc1	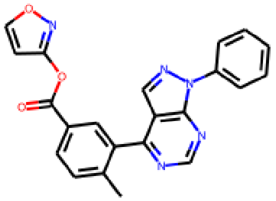	8.0	0.635315	4.542409	3.856266	5	0.760056
**2506**	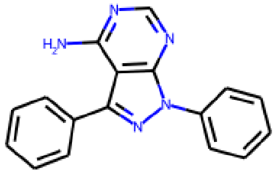	0.468475	Cc1ccc(C(=O)Oc2ccon2)cc1-c1ncnc2c1cnn2-c1ccccc1	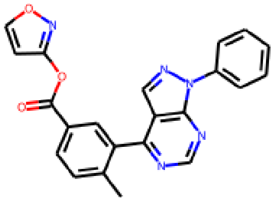	8.0	0.635315	4.542409	3.856266	5	0.760056
**2507**	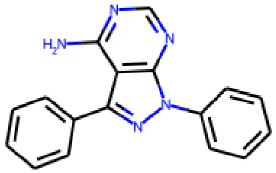	0.468475	Cc1ccc(C(=O)Oc2ccon2)cc1-c1ncnc2c1cnn2-c1ccccc1	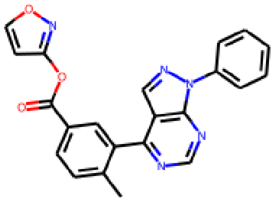	8.0	0.635315	4.542409	3.856266	5	0.760056

	src known mol	similarity	smiles	mol	Class	qed	logP	SAS	rings	kinase inhibition likeliness
**2508**	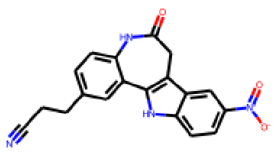	0.468379	CCNC(=O)N1CCC(Nc2n(C(C)c3c(-c4ccccc4)[nH]c4ccccc34)n2)CC1	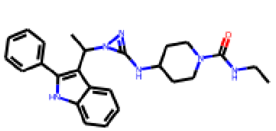	7.0	0.623584	3.603734	4.478009	5	0.804049
**2509**	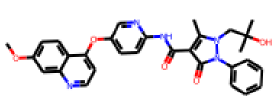	0.468109	COc1ccc2c(=O)cc(CNC(=O)c3c(C)n(C)n(-c4ccccc4)c3=O)nc3cccc3c2n1	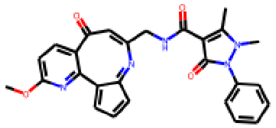	9.0	0.768132	3.608437	2.513170	5	0.823048
**2510**	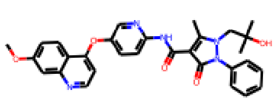	0.468109	COc1ccc2c(=O)cc(CNC(=O)c3c(C)n(C)n(-c4ccccc4)c3=O)nc3cccc3c2n1	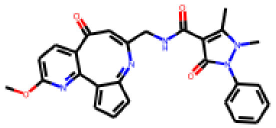	9.0	0.768132	3.608437	2.513170	5	0.823048
**2511**	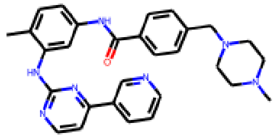	0.467875	Cc1ccc(NC(=O)c2ccccc2)cc1Nc1nccc(-c2cccnc2)n1	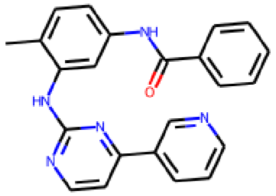	0.0	0.768132	3.608437	2.513170	4	0.854555

	src known mol	similarity	smiles	mol	Class	qed	logP	SAS	rings	kinase inhibition likeliness
**2512**	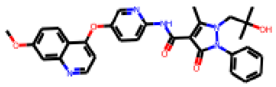	0.467820	COc1ccc2c(=O)cc(CNC(=O)c3c(C)n(C)n(-c4ccccc4)c3=O)nc3cccc2c3c1	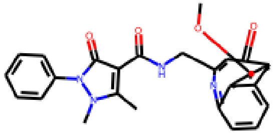	9.0	0.638163	3.995589	2.988471	5	0.774651
**2513**	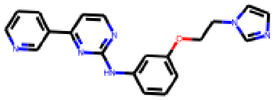	0.467797	Cc1ccc(OC(=O)c2ccccc2)cc1Nc1nccc(-c2cccnc2)n1	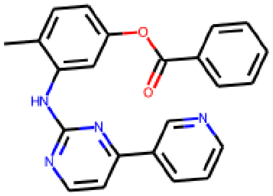	0.0	0.638163	3.995589	2.988471	4	0.775468
**2514**	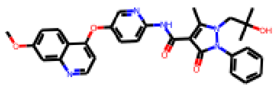	0.467687	COc1ccc2c(=O)cc(CNC(=O)c3c(C)n(C)n(-c4ccccc4)c3=O)nc3cccc3c2n1	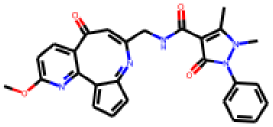	9.0	0.768132	3.608437	2.513170	5	0.823048
**2515**	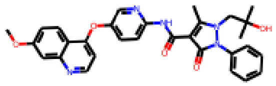	0.467681	COc1ccc2c(=O)cc(CNC(=O)c3c(C)n(C)n(-c4ccccc4)c3=O)nc3cccc2c3c1	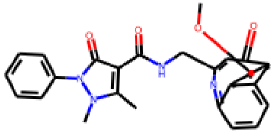	9.0	0.638163	3.995589	2.988471	5	0.774651

	src known mol	similarity	smiles	mol	Class	qed	logP	SAS	rings	kinase inhibition likeliness
**2516**	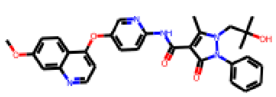	0.467614	COc1ccc2c(=O)cc(CNC(=O)c3c(C)n(C)n(-c4ccccc4)c3=O)nc3cccc3c2n1	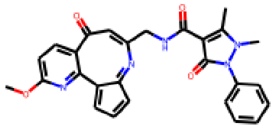	9.0	0.768132	3.608437	2.513170	5	0.823048
**2517**	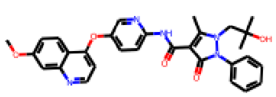	0.467577	COc1ccc2c(=O)cc(CNC(=O)c3c(C)n(C)n(-c4ccccc4)c3=O)nc3cccc3c2n1	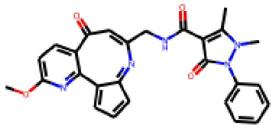	9.0	0.768132	3.608437	2.513170	5	0.823048
**2518**	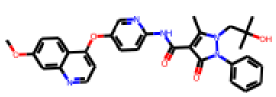	0.467577	COc1ccc2c(=O)cc(CNC(=O)c3c(C)n(C)n(-c4ccccc4)c3=O)nc3cccc3c2n1	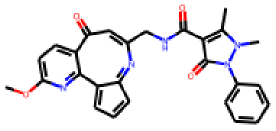	9.0	0.768132	3.608437	2.513170	5	0.823048
**2519**	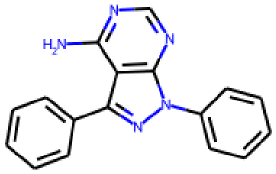	0.467456	Cc1ccc(C(=O)Oc2ccon2)cc1-c1ncnc2c1cnn2-c1ccccc1	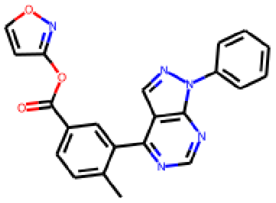	8.0	0.635315	4.542409	3.856266	5	0.760056

	src known mol	similarity	smiles	mol	Class	qed	logP	SAS	rings	kinase inhibition likeliness
**2520**	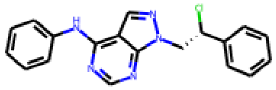	0.467398	Cc1ccc(C(=O)Nc2ccon2)cc1Nc1ncnc2c1cnn2C1cccccc1	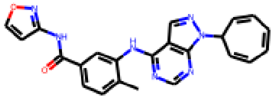	8.0	0.623362	2.525411	2.997881	5	0.770298
**2521**	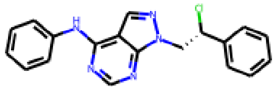	0.467398	Cc1ccc(C(=O)Nc2ccon2)cc1Nc1ncnc2c1cnn2C1cccccc1	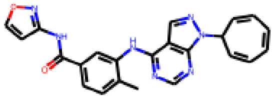	8.0	0.623362	2.525411	2.997881	5	0.770298
**2522**	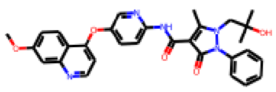	0.467385	COc1ccc2c(=O)cc(CNC(=O)c3c(C)n(C)n(-c4ccccc4)c3=O)nc3cccc3c2n1	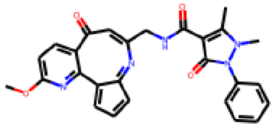	9.0	0.768132	3.608437	2.513170	5	0.823048
**2523**	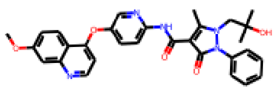	0.467385	COc1ccc2c(=O)cc(CNC(=O)c3c(C)n(C)n(-c4ccccc4)c3=O)nc3cccc3c2n1	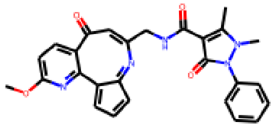	9.0	0.768132	3.608437	2.513170	5	0.823048

	src known mol	similarity	smiles	mol	Class	qed	logP	SAS	rings	kinase inhibition likeliness
**2524**	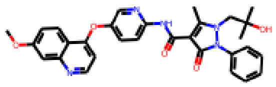	0.467385	COc1ccc2c(=O)cc(CNC(=O)c3c(C)n(C)n(-c4ccccc4)c3=O)nc3cccc3c2n1	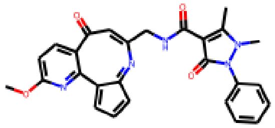	9.0	0.768132	3.608437	2.513170	5	0.823048
**2525**	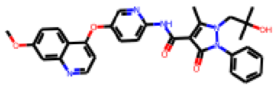	0.467385	COc1ccc2c(=O)cc(CNC(=O)c3c(C)n(C)n(-c4ccccc4)c3=O)nc3cccc3c2n1	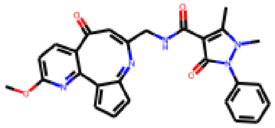	9.0	0.768132	3.608437	2.513170	5	0.823048
**2526**	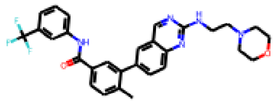	0.467382	Cc1ccc(C(=O)Oc2cccc(C(F)(F)F)c2)cc1-c1ccc2nc(N)ncc2c1	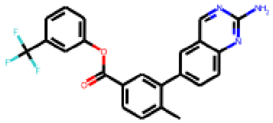	6.0	0.638163	3.995589	2.988471	4	0.789898
**2527**	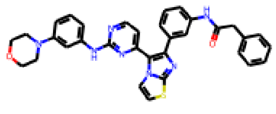	0.467290	ClCNc1nccc(-c2c(-c3ccc(F)cc3)nc3n2[C@H](CO)CC3)n1	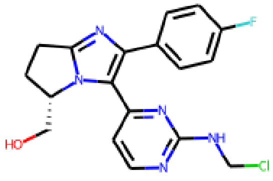	7.0	0.520676	2.336694	2.899742	4	0.812342

	src known mol	similarity	smiles	mol	Class	qed	logP	SAS	rings	kinase inhibition likeliness
**2528**	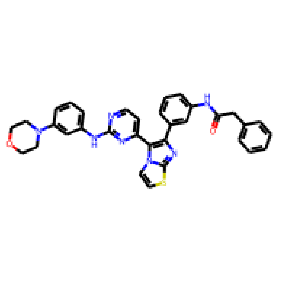	0.467290	ClCNc1nccc(-c2c(-c3ccc(F)cc3)nc3n2[C@H](CO)CC3)n1	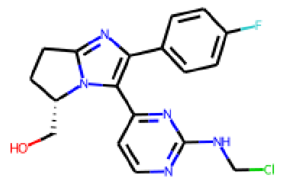	7.0	0.520676	2.336694	2.899742	4	0.812342
**2529**	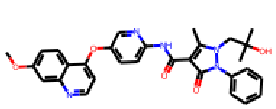	0.467134	COc1ccc2c(=O)cc(CNC(=O)c3c(C)n(C)n(-c4ccccc4)c3=O)nc3cccc2c3c1	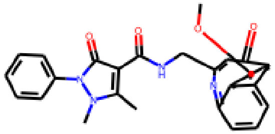	9.0	0.638163	3.995589	2.988471	5	0.774651
**2530**	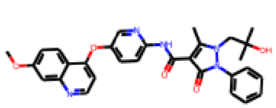	0.467030	COc1ccc2c(=O)cc(CNC(=O)c3c(C)n(C)n(-c4ccccc4)c3=O)nc3cccc2c3c1	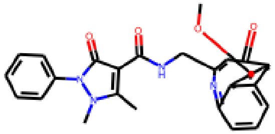	9.0	0.638163	3.995589	2.988471	5	0.774651
**2531**	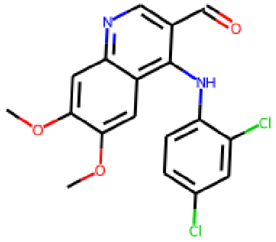	0.466906	COc1cc2ncc(C(=O)O)c(Nc3cccc(Cl)c3Cl)c2cc1OC1C(C)C1	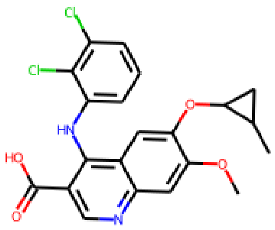	2.0	0.623362	2.525411	2.997881	4	0.774857

	src known mol	similarity	smiles	mol	Class	qed	logP	SAS	rings	kinase inhibition likeliness
**2532**	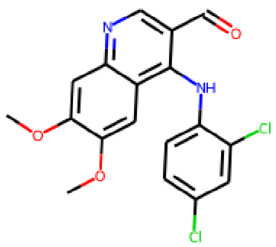	0.466906	COc1cc2ncc(C(=O)O)c(Nc3cccc(Cl)c3Cl)c2cc1OC1C(C)C1	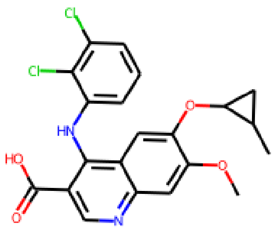	2.0	0.623362	2.525411	2.997881	4	0.774857
**2533**	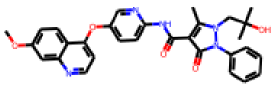	0.466591	COc1ccc2c(=O)cc(CNC(=O)c3c(C)n(C)n(-c4ccccc4)c3=O)nc3cccc3c2n1	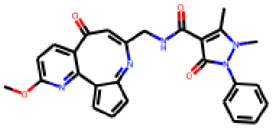	9.0	0.768132	3.608437	2.513170	5	0.823048
**2534**	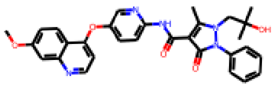	0.466591	COc1ccc2c(=O)cc(CNC(=O)c3c(C)n(C)n(-c4ccccc4)c3=O)nc3cccc3c2n1	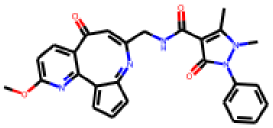	9.0	0.768132	3.608437	2.513170	5	0.823048
**2535**	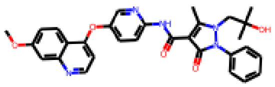	0.466486	COc1ccc2c(=O)cc(CNC(=O)c3c(C)n(C)n(-c4ccccc4)c3=O)nc3cccc2c3c1	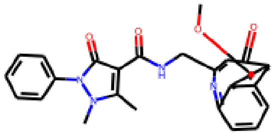	9.0	0.638163	3.995589	2.988471	5	0.774651

	src known mol	similarity	smiles	mol	Class	qed	logP	SAS	rings	kinase inhibition likeliness
**2536**	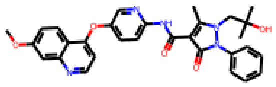	0.466405	COc1ccc2c(=O)cc(CNC(=O)c3c(C)n(C)n(-c4ccccc4)c3=O)nc3cccc3c2n1	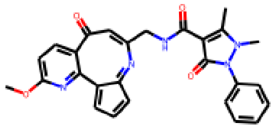	9.0	0.768132	3.608437	2.513170	5	0.823048
**2537**	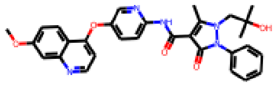	0.465957	COc1ccc2c(=O)cc(CNC(=O)c3c(C)n(C)n(-c4ccccc4)c3=O)nc3cccc2c3c1	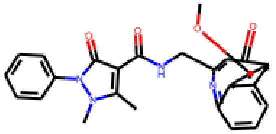	9.0	0.638163	3.995589	2.988471	5	0.774651
**2538**	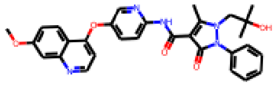	0.465957	COc1ccc2c(=O)cc(CNC(=O)c3c(C)n(C)n(-c4ccccc4)c3=O)nc3cccc2c3c1	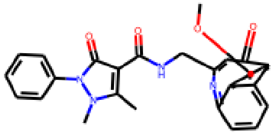	9.0	0.638163	3.995589	2.988471	5	0.774651
**2539**	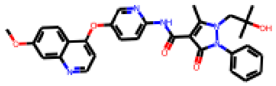	0.465636	COc1ccc2c(=O)cc(CNC(=O)c3c(C)n(C)n(-c4ccccc4)c3=O)nc3cccc3c2n1	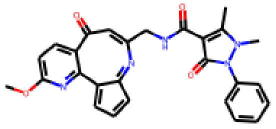	9.0	0.768132	3.608437	2.513170	5	0.823048

	src known mol	similarity	smiles	mol	Class	qed	logP	SAS	rings	kinase inhibition likeliness
**2540**	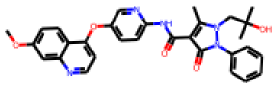	0.465636	COc1ccc2c(=O)cc(CNC(=O)c3c(C)n(C)n(-c4ccccc4)c3=O)nc3cccc3c2n1	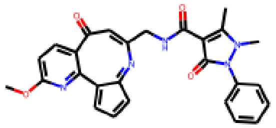	9.0	0.768132	3.608437	2.513170	5	0.823048
**2541**	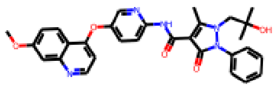	0.465627	COc1ccc2c(=O)cc(CNC(=O)c3c(C)n(C)n(-c4ccccc4)c3=O)nc3cccc3c2n1	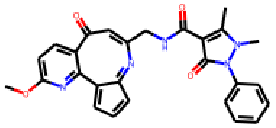	9.0	0.768132	3.608437	2.513170	5	0.823048
**2542**	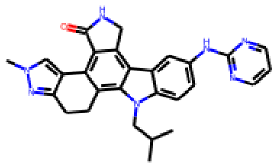	0.465539	C#Cc1ccc(C(=O)OC)cc1-c1C(=O)c-n2nc(-c3ccc(F)cc3F)cc2C1	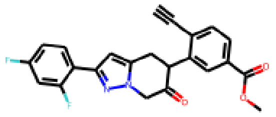	8.0	0.768132	3.608437	2.513170	4	0.763730
**2543**	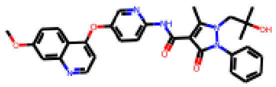	0.465527	COc1ccc2c(=O)cc(CNC(=O)c3c(C)n(C)n(-c4ccccc4)c3=O)nc3cccc3c2n1	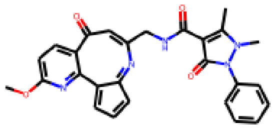	9.0	0.768132	3.608437	2.513170	5	0.823048

	src known mol	similarity	smiles	mol	Class	qed	logP	SAS	rings	kinase inhibition likeliness
**2544**	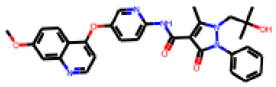	0.465527	COc1ccc2c(=O)cc(CNC(=O)c3c(C)n(C)n(-c4ccccc4)c3=O)nc3cccc3c2n1	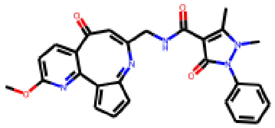	9.0	0.768132	3.608437	2.513170	5	0.823048
**2545**	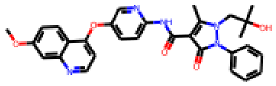	0.465488	COc1ccc2c(=O)cc(CNC(=O)c3c(C)n(C)n(-c4ccccc4)c3=O)nc3cccc3c2n1	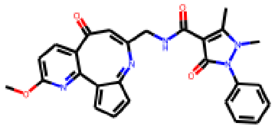	9.0	0.768132	3.608437	2.513170	5	0.823048
**2546**	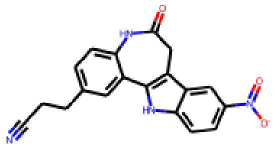	0.465392	CCNC(=O)N1CCC(Nc2n(C(C)c3c(-c4ccccc4)[nH]c4ccccc34)n2)CC1	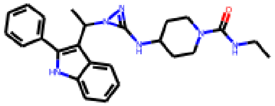	7.0	0.623584	3.603734	4.478009	5	0.804049
**2547**	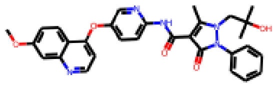	0.465274	COc1ccc2c(=O)cc(CNC(=O)c3c(C)n(C)n(-c4ccccc4)c3=O)nc3cccc3c2n1	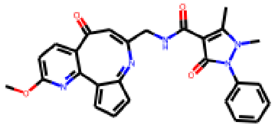	9.0	0.768132	3.608437	2.513170	5	0.823048

	src known mol	similarity	smiles	mol	Class	qed	logP	SAS	rings	kinase inhibition likeliness
**2548**	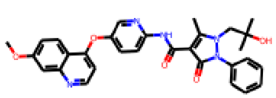	0.465195	COc1ccc2c(=O)cc(CNC(=O)c3c(C)n(C)n(-c4ccccc4)c3=O)nc3cccc3c2n1	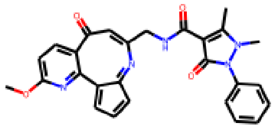	9.0	0.768132	3.608437	2.513170	5	0.823048
**2549**	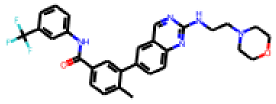	0.465170	Cc1ccc(C(=O)Nc2cccc(C(F)(F)F)c2C)cc1-c1ccc2nc(O)ncc2c1	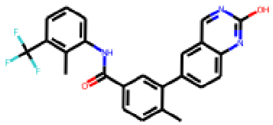	6.0	0.638163	3.995589	2.988471	4	0.808346
**2550**	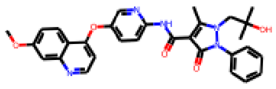	0.465079	COc1ccc2c(=O)cc(CNC(=O)c3c(C)n(C)n(-c4ccccc4)c3=O)nc3cccc2c3c1	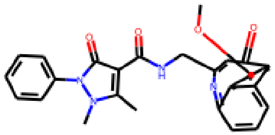	9.0	0.638163	3.995589	2.988471	5	0.774651
**2551**	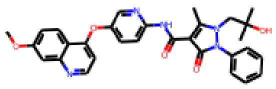	0.464917	COc1ccc2c(=O)cc(CNC(=O)c3c(C)n(C)n(-c4ccccc4)c3=O)nc3cccc3c2n1	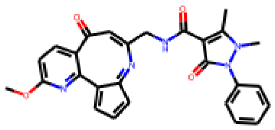	9.0	0.768132	3.608437	2.513170	5	0.823048

	src known mol	similarity	smiles	mol	Class	qed	logP	SAS	rings	kinase inhibition likeliness
**2552**	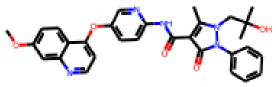	0.464917	COc1ccc2c(=O)cc(CNC(=O)c3c(C)n(C)n(-c4ccccc4)c3=O)nc3cccc3c2n1	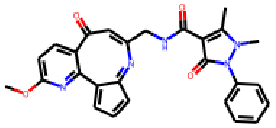	9.0	0.768132	3.608437	2.513170	5	0.823048
**2553**	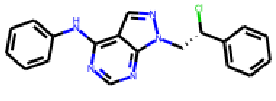	0.464854	Cc1ccc(C(=O)Nc2ccon2)cc1Nc1ncnc2c1cnn2C1cccccc1	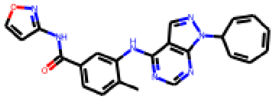	8.0	0.623362	2.525411	2.997881	5	0.770298
**2554**	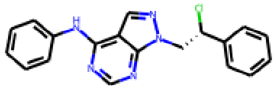	0.464826	Cc1ccc(C(=O)Nc2ccon2)cc1Nc1ncnc2c1cnn2C1cccccc1	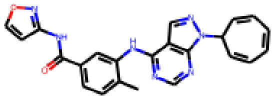	8.0	0.623362	2.525411	2.997881	5	0.770298
**2555**	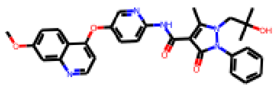	0.464751	COc1ccc2c(=O)cc(CNC(=O)c3c(C)n(C)n(-c4ccccc4)c3=O)nc3cccc2c3c1	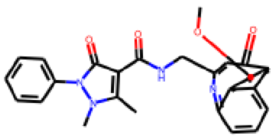	9.0	0.638163	3.995589	2.988471	5	0.774651

	src known mol	similarity	smiles	mol	Class	qed	logP	SAS	rings	kinase inhibition likeliness
**2556**	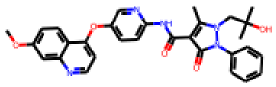	0.464674	COc1ccc2c(=O)cc(CNC(=O)c3c(C)n(C)n(-c4ccccc4)c3=O)nc3cccc2c3c1	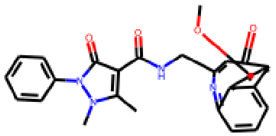	9.0	0.638163	3.995589	2.988471	5	0.774651
**2557**	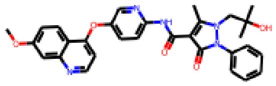	0.464674	COc1ccc2c(=O)cc(CNC(=O)c3c(C)n(C)n(-c4ccccc4)c3=O)nc3cccc2c3c1	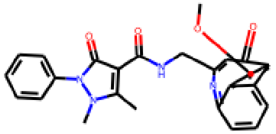	9.0	0.638163	3.995589	2.988471	5	0.774651
**2558**	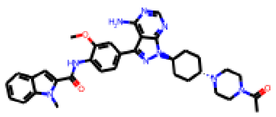	0.464672	Cc1ccc(C(=O)Nc2cccc(C(F)(F)F)c2C)cc1-c1ccc2oc(=O)cccc(F)c2n1	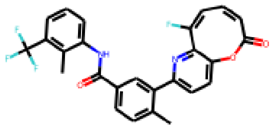	6.0	0.790896	3.985943	2.115897	4	0.783675
**2559**	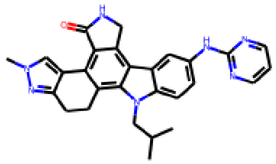	0.464512	C#Cc1ccc(C(=O)OC)cc1-c1C(=O)c-n2nc(-c3ccc(F)cc3F)cc2C1	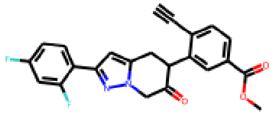	8.0	0.768132	3.608437	2.513170	4	0.763730

	src known mol	similarity	smiles	mol	Class	qed	logP	SAS	rings	kinase inhibition likeliness
**2560**	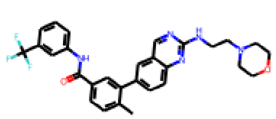	0.464422	Cc1ccc(C(=O)Nc2cccc(C(F)(F)F)c2C)cc1-c1ccc2nc(O)ncc2c1	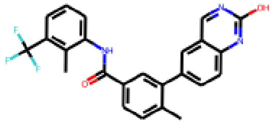	6.0	0.638163	3.995589	2.988471	4	0.808346
**2561**	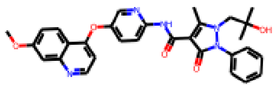	0.464112	COc1ccc2c(=O)cc(CNC(=O)c3c(C)n(C)n(-c4ccccc4)c3=O)nc3cccc2c3c1	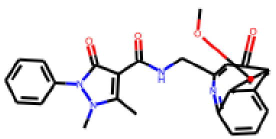	9.0	0.638163	3.995589	2.988471	5	0.774651
**2562**	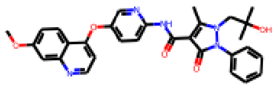	0.464101	COc1ccc2c(=O)cc(CNC(=O)c3c(C)n(C)n(-c4ccccc4)c3=O)nc3cccc3c2n1	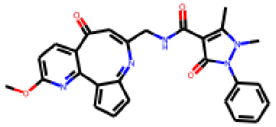	9.0	0.768132	3.608437	2.513170	5	0.823048
**2563**	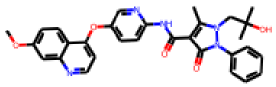	0.464101	COc1ccc2c(=O)cc(CNC(=O)c3c(C)n(C)n(-c4ccccc4)c3=O)nc3cccc3c2n1	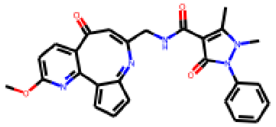	9.0	0.768132	3.608437	2.513170	5	0.823048

	src known mol	similarity	smiles	mol	Class	qed	logP	SAS	rings	kinase inhibition likeliness
**2564**	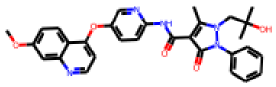	0.464101	COc1ccc2c(=O)cc(CNC(=O)c3c(C)n(C)n(-c4ccccc4)c3=O)nc3cccc3c2n1	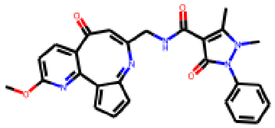	9.0	0.768132	3.608437	2.513170	5	0.823048
**2565**	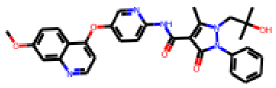	0.463878	COc1ccc2c(=O)cc(CNC(=O)c3c(C)n(C)n(-c4ccccc4)c3=O)nc3cccc2c3c1	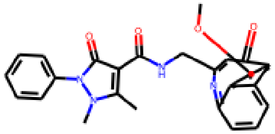	9.0	0.638163	3.995589	2.988471	5	0.774651
**2566**	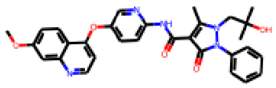	0.463876	COc1ccc2c(=O)cc(CNC(=O)c3c(C)n(C)n(-c4ccccc4)c3=O)nc3cccc3c2n1	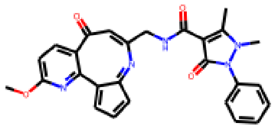	9.0	0.768132	3.608437	2.513170	5	0.823048
**2567**	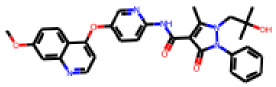	0.463876	COc1ccc2c(=O)cc(CNC(=O)c3c(C)n(C)n(-c4ccccc4)c3=O)nc3cccc3c2n1	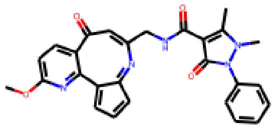	9.0	0.768132	3.608437	2.513170	5	0.823048

	src known mol	similarity	smiles	mol	Class	qed	logP	SAS	rings	kinase inhibition likeliness
**2568**	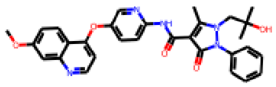	0.463876	COc1ccc2c(=O)cc(CNC(=O)c3c(C)n(C)n(-c4ccccc4)c3=O)nc3cccc3c2n1	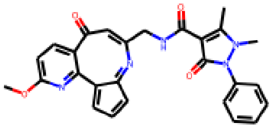	9.0	0.768132	3.608437	2.513170	5	0.823048
**2569**	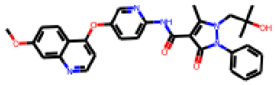	0.463876	COc1ccc2c(=O)cc(CNC(=O)c3c(C)n(C)n(-c4ccccc4)c3=O)nc3cccc3c2n1	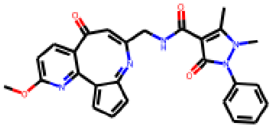	9.0	0.768132	3.608437	2.513170	5	0.823048
**2570**	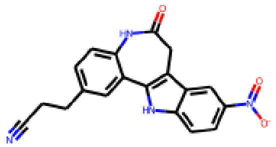	0.463816	CCNC(=O)N1CCC(Nc2n(C(C)c3c(-c4ccccc4)[nH]c4ccccc34)n2)CC1	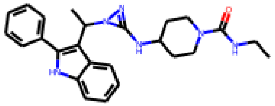	7.0	0.623584	3.603734	4.478009	5	0.804049
**2571**	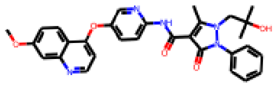	0.463776	COc1ccc2c(=O)cc(CNC(=O)c3c(C)n(C)n(-c4ccccc4)c3=O)nc3cccc3c2n1	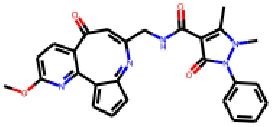	9.0	0.768132	3.608437	2.513170	5	0.823048

	src known mol	similarity	smiles	mol	Class	qed	logP	SAS	rings	kinase inhibition likeliness
**2572**	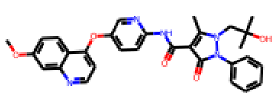	0.463636	COc1ccc2c(=O)cc(CNC(=O)c3c(C)n(C)n(-c4ccccc4)c3=O)nc3cccc3c2n1	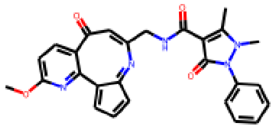	9.0	0.768132	3.608437	2.513170	5	0.823048
**2573**	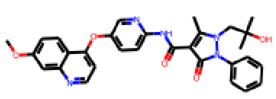	0.463494	COc1ccc2c(=O)cc(CNC(=O)c3c(C)n(C)n(-c4ccccc4)c3=O)nc3cccc2c3c1	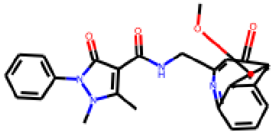	9.0	0.638163	3.995589	2.988471	5	0.774651
**2574**	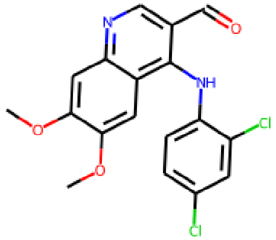	0.463467	COc1cc2ncc(C(=O)O)c(Nc3cccc(Cl)c3Cl)c2cc1OC1C(C)C1	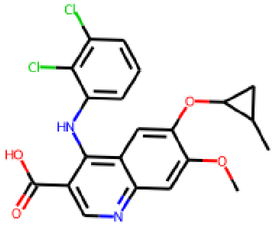	2.0	0.623362	2.525411	2.997881	4	0.774857
**2575**	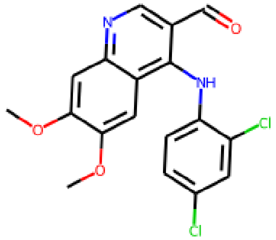	0.463467	COc1cc2ncc(C(=O)O)c(Nc3cccc(Cl)c3Cl)c2cc1OC1C(C)C1	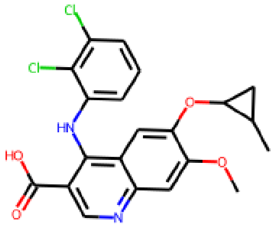	2.0	0.623362	2.525411	2.997881	4	0.774857

	src known mol	similarity	smiles	mol	Class	qed	logP	SAS	rings	kinase inhibition likeliness
**2576**	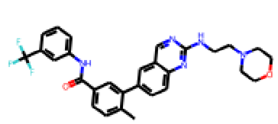	0.463309	Cc1ccc(C(=O)Nc2cccc(C(F)(C)F)c2C)cc1-c1ccc2nc(C)ccccc(F)nc2c1	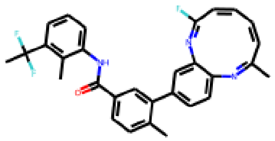	6.0	0.742375	4.384502	0.286234	4	0.779167
**2577**	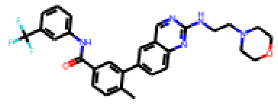	0.463309	Cc1ccc(C(=O)Nc2cccc(C(F)(C)F)c2C)cc1-c1ccc2nc(C)ccccc(F)nc2c1	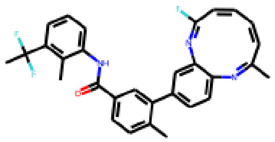	6.0	0.742375	4.384502	0.286234	4	0.779167
**2578**	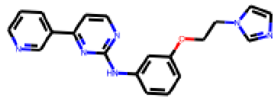	0.463307	Cc1ccc(OC(=O)c2ccccc2)cc1Nc1nccc(-c2cccnc2)n1	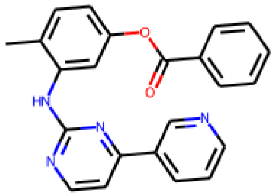	0.0	0.638163	3.995589	2.988471	4	0.775468
**2579**	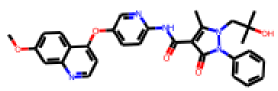	0.463003	COc1ccc2c(=O)cc(CNC(=O)c3c(C)n(C)n(-c4ccccc4)c3=O)nc3cccc2c3c1	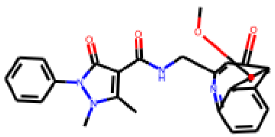	9.0	0.638163	3.995589	2.988471	5	0.774651

	src known mol	similarity	smiles	mol	Class	qed	logP	SAS	rings	kinase inhibition likeliness
**2580**	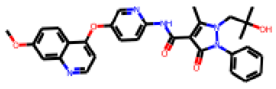	0.462995	COc1ccc2c(=O)cc(CNC(=O)c3c(C)n(C)n(-c4ccccc4)c3=O)nc3cccc3c2n1	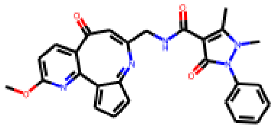	9.0	0.768132	3.608437	2.513170	5	0.823048
**2581**	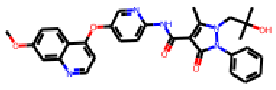	0.462995	COc1ccc2c(=O)cc(CNC(=O)c3c(C)n(C)n(-c4ccccc4)c3=O)nc3cccc3c2n1	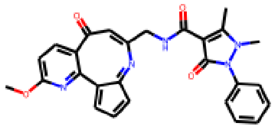	9.0	0.768132	3.608437	2.513170	5	0.823048
**2582**	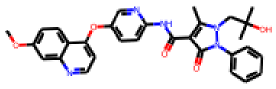	0.462900	COc1ccc2c(=O)cc(CNC(=O)c3c(C)n(C)n(-c4ccccc4)c3=O)nc3cccc3c2n1	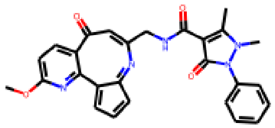	9.0	0.768132	3.608437	2.513170	5	0.823048
**2583**	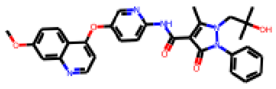	0.462882	COc1ccc2c(=O)cc(CNC(=O)c3c(C)n(C)n(-c4ccccc4)c3=O)nc3cccc2c3c1	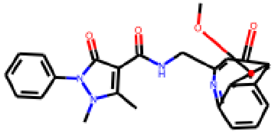	9.0	0.638163	3.995589	2.988471	5	0.774651

	src known mol	similarity	smiles	mol	Class	qed	logP	SAS	rings	kinase inhibition likeliness
**2584**	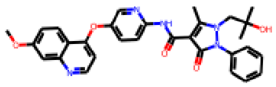	0.462687	COc1ccc2c(=O)cc(CNC(=O)c3c(C)n(C)n(-c4ccccc4)c3=O)nc3cccc3c2n1	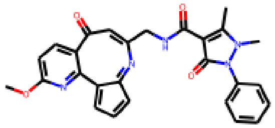	9.0	0.768132	3.608437	2.513170	5	0.823048
**2585**	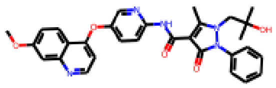	0.462635	COc1ccc2c(=O)cc(CNC(=O)c3c(C)n(C)n(-c4ccccc4)c3=O)nc3cccc3c2n1	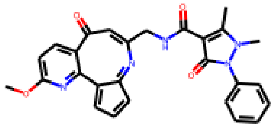	9.0	0.768132	3.608437	2.513170	5	0.823048
**2586**	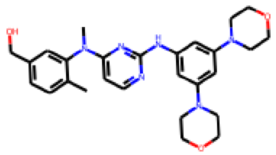	0.462597	NC(=O)c1ccccc1Nc1nc(Nc2ccc(N3CCCC3=O)cc2)ncc1Cl	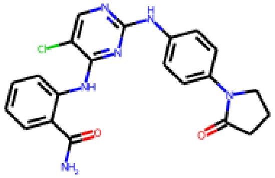	7.0	0.768132	3.608437	2.513170	4	0.773984
**2587**	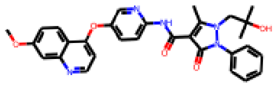	0.462329	COc1ccc2c(=O)cc(CNC(=O)c3c(C)n(C)n(-c4ccccc4)c3=O)nc3cccc3c2n1	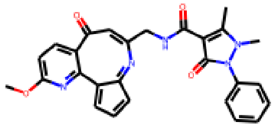	9.0	0.768132	3.608437	2.513170	5	0.823048

	src known mol	similarity	smiles	mol	Class	qed	logP	SAS	rings	kinase inhibition likeliness
**2588**	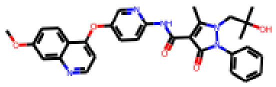	0.462329	COc1ccc2c(=O)cc(CNC(=O)c3c(C)n(C)n(-c4ccccc4)c3=O)nc3cccc3c2n1	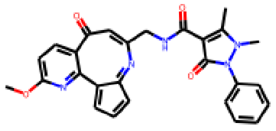	9.0	0.768132	3.608437	2.513170	5	0.823048
**2589**	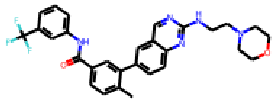	0.462192	Cc1ccc(C(=O)Oc2cccc(C(F)(F)F)c2)cc1-c1ccc2nc(N)ncc2c1	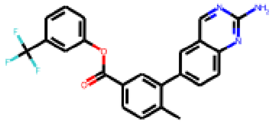	6.0	0.638163	3.995589	2.988471	4	0.789898
**2590**	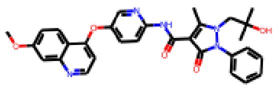	0.462125	COc1ccc2c(=O)cc(CNC(=O)c3c(C)n(C)n(-c4ccccc4)c3=O)nc3cccc2c3c1	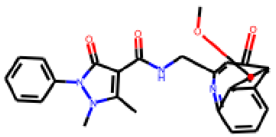	9.0	0.638163	3.995589	2.988471	5	0.774651
**2591**	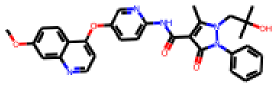	0.462108	COc1ccc2c(=O)cc(CNC(=O)c3c(C)n(C)n(-c4ccccc4)c3=O)nc3cccc3c2n1	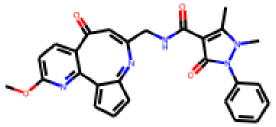	9.0	0.768132	3.608437	2.513170	5	0.823048

	src known mol	similarity	smiles	mol	Class	qed	logP	SAS	rings	kinase inhibition likeliness
**2592**	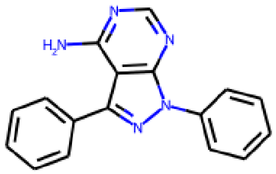	0.462000	Cc1ccc(C(=O)Oc2ccon2)cc1-c1ncnc2c1cnn2-c1ccccc1	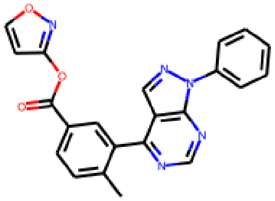	8.0	0.635315	4.542409	3.856266	5	0.760056
**2593**	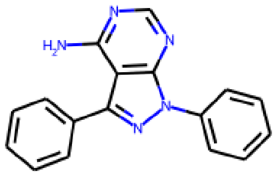	0.462000	Cc1ccc(C(=O)Oc2ccon2)cc1-c1ncnc2c1cnn2-c1ccccc1	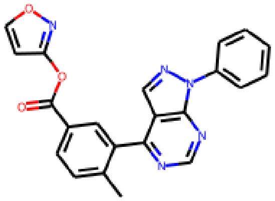	8.0	0.635315	4.542409	3.856266	5	0.760056
**2594**	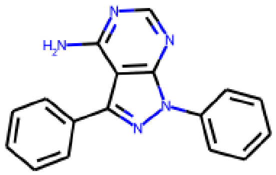	0.462000	Cc1ccc(C(=O)Oc2ccon2)cc1-c1ncnc2c1cnn2-c1ccccc1	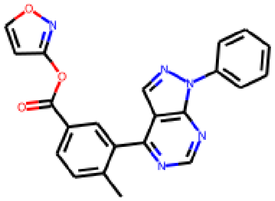	8.0	0.635315	4.542409	3.856266	5	0.760056
**2595**	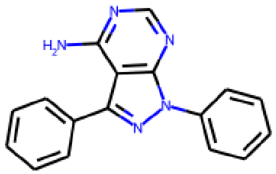	0.462000	Cc1ccc(C(=O)Oc2ccon2)cc1-c1ncnc2c1cnn2-c1ccccc1	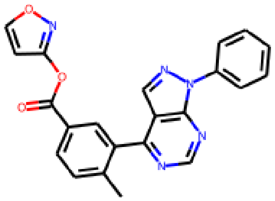	8.0	0.635315	4.542409	3.856266	5	0.760056

	src known mol	similarity	smiles	mol	Class	qed	logP	SAS	rings	kinase inhibition likeliness
**2596**	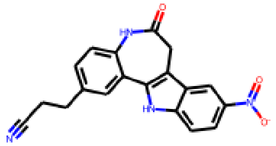	0.461996	CCNC(=O)N1CCC(Nc2n(C(C)c3c(-c4ccccc4)[nH]c4ccccc34)n2)CC1	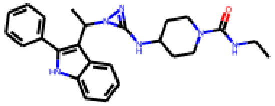	7.0	0.623584	3.603734	4.478009	5	0.804049
**2597**	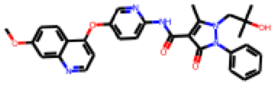	0.461874	COc1ccc2c(=O)cc(CNC(=O)c3c(C)n(C)n(-c4ccccc4)c3=O)nc3cccc2c3c1	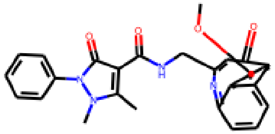	9.0	0.638163	3.995589	2.988471	5	0.774651
**2598**	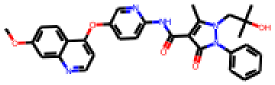	0.461758	COc1ccc2c(=O)cc(CNC(=O)c3c(C)n(C)n(-c4ccccc4)c3=O)nc3cccc3c2n1	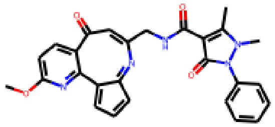	9.0	0.768132	3.608437	2.513170	5	0.823048
**2599**	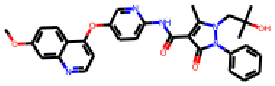	0.461670	COc1ccc2c(=O)cc(CNC(=O)c3c(C)n(C)n(-c4ccccc4)c3=O)nc3cccc3c2n1	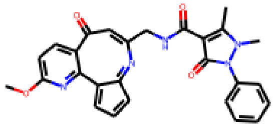	9.0	0.768132	3.608437	2.513170	5	0.823048

	src known mol	similarity	smiles	mol	Class	qed	logP	SAS	rings	kinase inhibition likeliness
**2600**	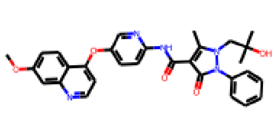	0.461664	COc1ccc2c(=O)cc(CNC(=O)c3c(C)n(C)n(-c4ccccc4)c3=O)nc3cccc2c3c1	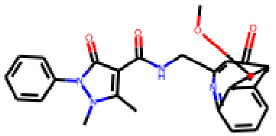	9.0	0.638163	3.995589	2.988471	5	0.774651
**2601**	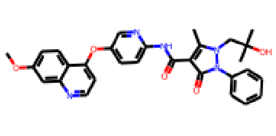	0.461538	COc1ccc2c(=O)cc(CNC(=O)c3c(C)n(C)n(-c4ccccc4)c3=O)nc3cccc3c2n1	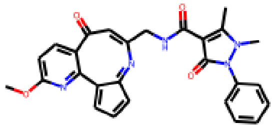	9.0	0.768132	3.608437	2.513170	5	0.823048
**2602**	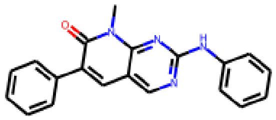	0.461355	Clc1cccc(Nc2ncc3cc(-c3c(Cl)cccc3Cl)c(=O)n(C)c3n2)c1	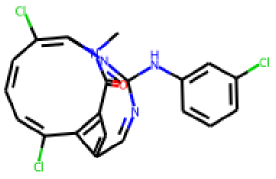	2.0	0.768132	3.608437	2.513170	5	0.784634
**2603**	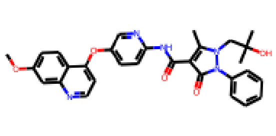	0.461231	COc1ccc2c(=O)cc(CNC(=O)c3c(C)n(C)n(-c4ccccc4)c3=O)nc3cccc3c2n1	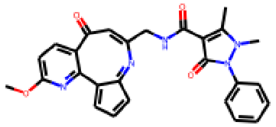	9.0	0.768132	3.608437	2.513170	5	0.823048

	src known mol	similarity	smiles	mol	Class	qed	logP	SAS	rings	kinase inhibition likeliness
**2604**	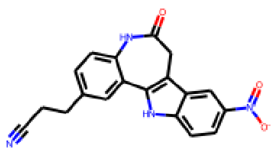	0.460969	CCNC(=O)N1CCC(Nc2n(C(C)c3c(-c4ccccc4)[nH]c4ccccc34)n2)CC1	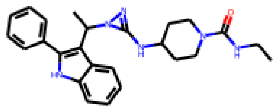	7.0	0.623584	3.603734	4.478009	5	0.804049
**2605**	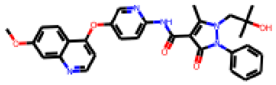	0.460960	COc1ccc2c(=O)cc(CNC(=O)c3c(C)n(C)n(-<br>c4ccccc4)c3=O)nc3cccc3c2n1	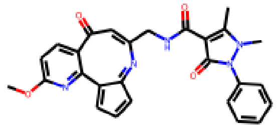	9.0	0.768132	3.608437	2.513170	5	0.823048
**2606**	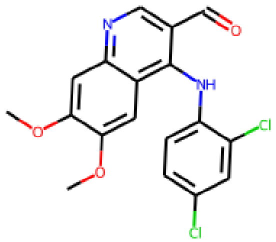	0.460902	COc1cc2ncc(C(=O)O)c(Nc3cccc(Cl)c3Cl)c2cc1OC1C(C)C1	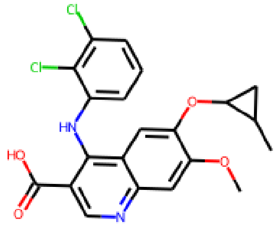	2.0	0.623362	2.525411	2.997881	4	0.774857
**2607**	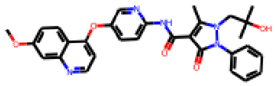	0.460884	COc1ccc2c(=O)cc(CNC(=O)c3c(C)n(C)n(-<br>c4ccccc4)c3=O)nc3cccc3c2n1	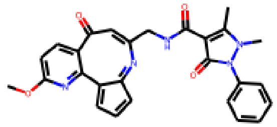	9.0	0.768132	3.608437	2.513170	5	0.823048

	src known mol	similarity	smiles	mol	Class	qed	logP	SAS	rings	kinase inhibition likeliness
**2608**	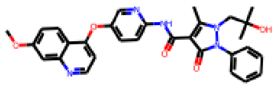	0.460884	COc1ccc2c(=O)cc(CNC(=O)c3c(C)n(C)n(-c4ccccc4)c3=O)nc3cccc3c2n1	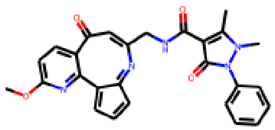	9.0	0.768132	3.608437	2.513170	5	0.823048
**2609**	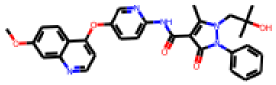	0.460668	COc1ccc2c(=O)cc(CNC(=O)c3c(C)n(C)n(-c4ccccc4)c3=O)nc3cccc3c2n1	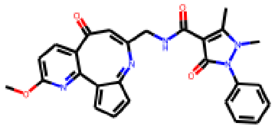	9.0	0.768132	3.608437	2.513170	5	0.823048
**2610**	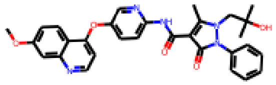	0.460576	COc1ccc2c(=O)cc(CNC(=O)c3c(C)n(C)n(-c4ccccc4)c3=O)nc3cccc2c3c1	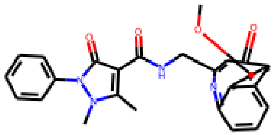	9.0	0.638163	3.995589	2.988471	5	0.774651
**2611**	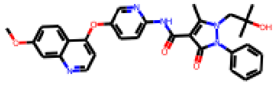	0.460526	COc1ccc2c(=O)cc(CNC(=O)c3c(C)n(C)n(-c4ccccc4)c3=O)nc3cccc3c2n1	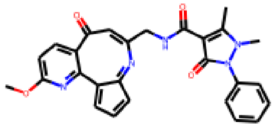	9.0	0.768132	3.608437	2.513170	5	0.823048

	src known mol	similarity	smiles	mol	Class	qed	logP	SAS	rings	kinase inhibition likeliness
**2612**	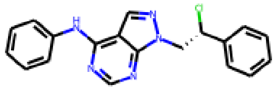	0.460518	Cc1ccc(C(=O)Nc2ccon2)cc1Nc1ncnc2c1cnn2C1cccccc1	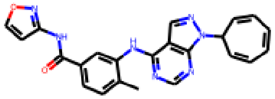	8.0	0.623362	2.525411	2.997881	5	0.770298
**2613**	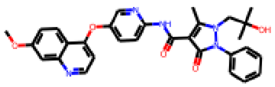	0.460490	COc1ccc2c(=O)cc(CNC(=O)c3c(C)n(C)n(-c4ccccc4)c3=O)nc3cccc2c3c1	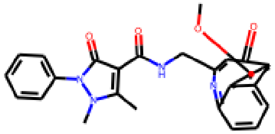	9.0	0.638163	3.995589	2.988471	5	0.774651
**2614**	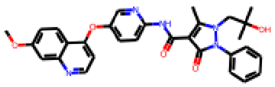	0.460447	COc1ccc2c(=O)cc(CNC(=O)c3c(C)n(C)n(-c4ccccc4)c3=O)nc3cccc2c3c1	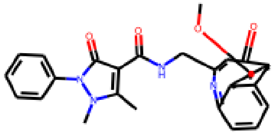	9.0	0.638163	3.995589	2.988471	5	0.774651
**2615**	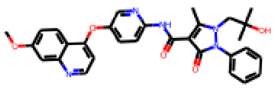	0.460399	COc1ccc2c(=O)cc(CNC(=O)c3c(C)n(C)n(-c4ccccc4)c3=O)nc3cccc3c2n1	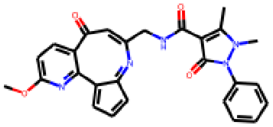	9.0	0.768132	3.608437	2.513170	5	0.823048

	src known mol	similarity	smiles	mol	Class	qed	logP	SAS	rings	kinase inhibition likeliness
**2616**	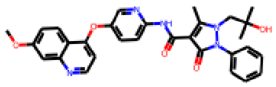	0.460399	COc1ccc2c(=O)cc(CNC(=O)c3c(C)n(C)n(-c4ccccc4)c3=O)nc3cccc3c2n1	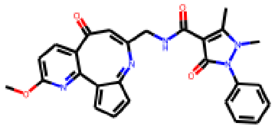	9.0	0.768132	3.608437	2.513170	5	0.823048
**2617**	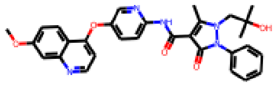	0.460399	COc1ccc2c(=O)cc(CNC(=O)c3c(C)n(C)n(-c4ccccc4)c3=O)nc3cccc3c2n1	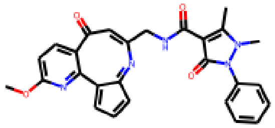	9.0	0.768132	3.608437	2.513170	5	0.823048
**2618**	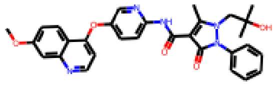	0.460369	COc1ccc2c(=O)cc(CNC(=O)c3c(C)n(C)n(-c4ccccc4)c3=O)nc3cccc2c3c1	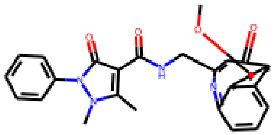	9.0	0.638163	3.995589	2.988471	5	0.774651
**2619**	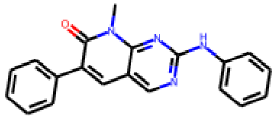	0.460265	Clc1cccc(Nc2ncc3cc(-c3c(Cl)cccc3Cl)c(=O)n(C)c3n2)c1	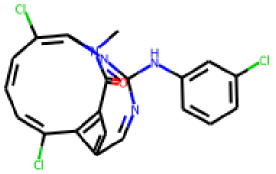	2.0	0.768132	3.608437	2.513170	5	0.784634

	src known mol	similarity	smiles	mol	Class	qed	logP	SAS	rings	kinase inhibition likeliness
**2620**	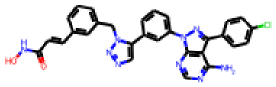	0.460123	Cc1ccc(C(=O)Nc2cscn2)cc1Nc1ncnc2c1cnn2-c1ccccc1	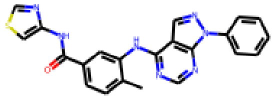	8.0	0.632663	4.308233	4.065316	5	0.804786
**2621**	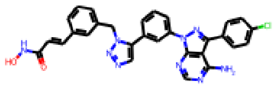	0.460123	Cc1ccc(C(=O)Nc2cscn2)cc1Nc1ncnc2c1cnn2-c1ccccc1	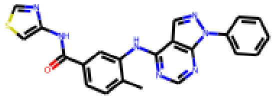	8.0	0.632663	4.308233	4.065316	5	0.804786
**2622**	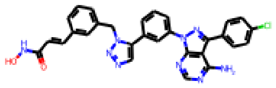	0.460123	Cc1ccc(C(=O)Nc2cscn2)cc1Nc1ncnc2c1cnn2-c1ccccc1	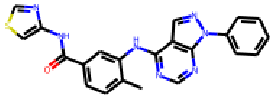	8.0	0.632663	4.308233	4.065316	5	0.804786
**2623**	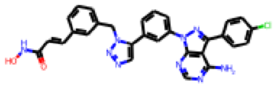	0.460123	Cc1ccc(C(=O)Nc2cscn2)cc1Nc1ncnc2c1cnn2-c1ccccc1	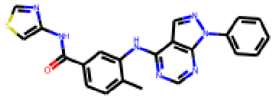	8.0	0.632663	4.308233	4.065316	5	0.804786

	src known mol	similarity	smiles	mol	Class	qed	logP	SAS	rings	kinase inhibition likeliness
**2624**	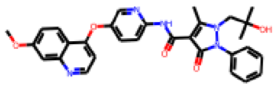	0.459875	COc1ccc2c(=O)cc(CNC(=O)c3c(C)n(C)n(-c4ccccc4)c3=O)nc3cccc3c2n1	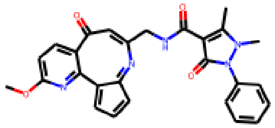	9.0	0.768132	3.608437	2.513170	5	0.823048
**2625**	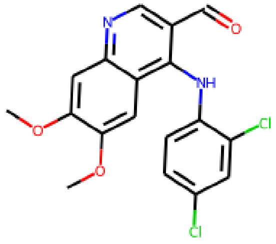	0.459854	COc1cc2ncc(C(=O)O)c(Nc3cccc(Cl)c3Cl)c2cc1OC1C(C)C1	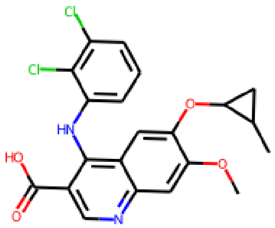	2.0	0.623362	2.525411	2.997881	4	0.774857
**2626**	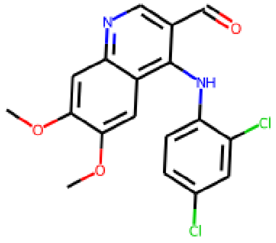	0.459854	COc1cc2ncc(C(=O)O)c(Nc3cccc(Cl)c3Cl)c2cc1OC1C(C)C1	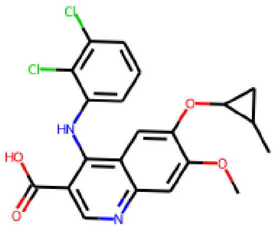	2.0	0.623362	2.525411	2.997881	4	0.774857
**2627**	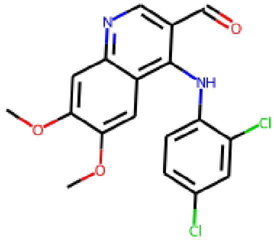	0.459854	COc1cc2ncc(C(=O)O)c(Nc3cccc(Cl)c3Cl)c2cc1OC1C(C)C1	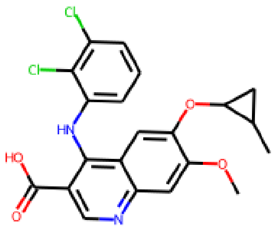	2.0	0.623362	2.525411	2.997881	4	0.774857

	src known mol	similarity	smiles	mol	Class	qed	logP	SAS	rings	kinase inhibition likeliness
**2628**	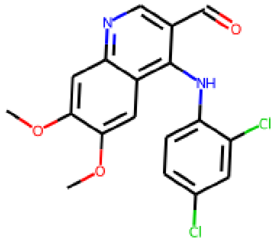	0.459854	COc1cc2ncc(C(=O)O)c(Nc3cccc(Cl)c3Cl)c2cc1OC1C(C)C1	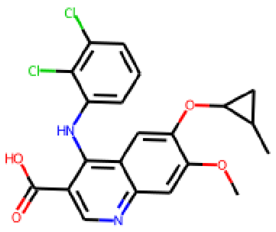	2.0	0.623362	2.525411	2.997881	4	0.774857
**2629**	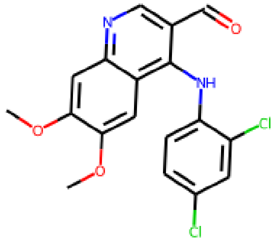	0.459854	COc1cc2ncc(C(=O)O)c(Nc3cccc(Cl)c3Cl)c2cc1OC1C(C)C1	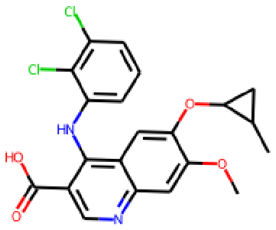	2.0	0.623362	2.525411	2.997881	4	0.774857
**2630**	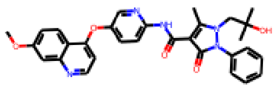	0.459664	COc1ccc2c(=O)cc(CNC(=O)c3c(C)n(C)n(-c4ccccc4)c3=O)nc3cccc2c3c1	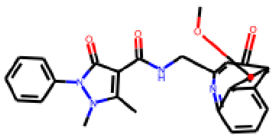	9.0	0.638163	3.995589	2.988471	5	0.774651
**2631**	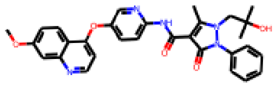	0.459645	COc1ccc2c(=O)cc(CNC(=O)c3c(C)n(C)n(-c4ccccc4)c3=O)nc3cccc3c2n1	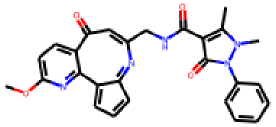	9.0	0.768132	3.608437	2.513170	5	0.823048

	src known mol	similarity	smiles	mol	Class	qed	logP	SAS	rings	kinase inhibition likeliness
**2632**	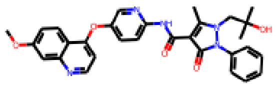	0.459527	COc1cc2c(OC)ccc(NC(=O)c3nn(-c4ccccc4C(F)(C)C)o3)c2C1	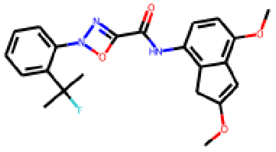	9.0	0.642592	3.893046	3.717919	4	0.765810
**2633**	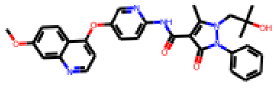	0.459416	COc1ccc2c(=O)cc(CNC(=O)c3c(C)n(C)n(-c4ccccc4)c3=O)nc3cccc2c3c1	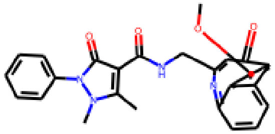	9.0	0.638163	3.995589	2.988471	5	0.774651
**2634**	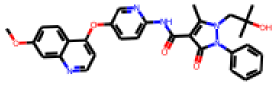	0.459398	COc1ccc2c(=O)cc(CNC(=O)c3c(C)n(C)n(-c4ccccc4)c3=O)nc3cccc3c2n1	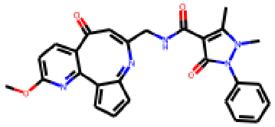	9.0	0.768132	3.608437	2.513170	5	0.823048
**2635**	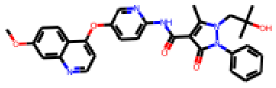	0.459398	COc1ccc2c(=O)cc(CNC(=O)c3c(C)n(C)n(-c4ccccc4)c3=O)nc3cccc3c2n1	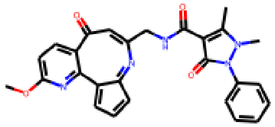	9.0	0.768132	3.608437	2.513170	5	0.823048

	src known mol	similarity	smiles	mol	Class	qed	logP	SAS	rings	kinase inhibition likeliness
**2636**	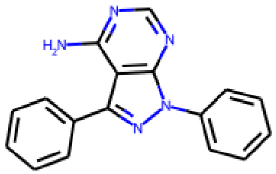	0.459369	Cc1ccc(C(=O)Oc2ccon2)cc1-c1ncnc2c1cnn2-c1ccccc1	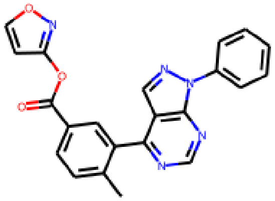	8.0	0.635315	4.542409	3.856266	5	0.760056
**2637**	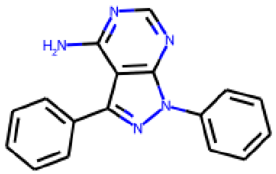	0.459369	Cc1ccc(C(=O)Oc2ccon2)cc1-c1ncnc2c1cnn2-c1ccccc1	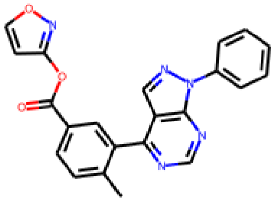	8.0	0.635315	4.542409	3.856266	5	0.760056
**2638**	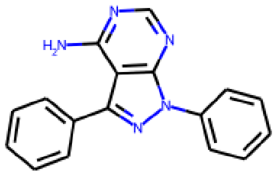	0.459369	Cc1ccc(C(=O)Oc2ccon2)cc1-c1ncnc2c1cnn2-c1ccccc1	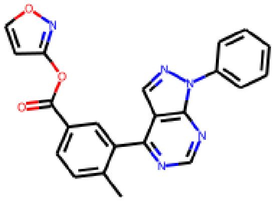	8.0	0.635315	4.542409	3.856266	5	0.760056
**2639**	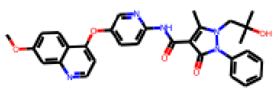	0.459259	COc1ccc2c(=O)cc(CNC(=O)c3c(C)n(C)n(-c4ccccc4)c3=O)nc3cccc3c2n1	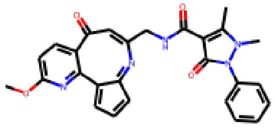	9.0	0.768132	3.608437	2.513170	5	0.823048

	src known mol	similarity	smiles	mol	Class	qed	logP	SAS	rings	kinase inhibition likeliness
**2640**	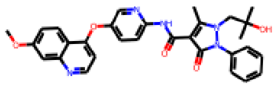	0.459213	COc1ccc2c(=O)cc(CNC(=O)c3c(C)n(C)n(-c4ccccc4)c3=O)nc3cccc3c2n1	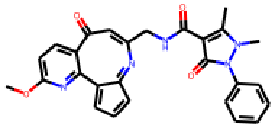	9.0	0.768132	3.608437	2.513170	5	0.823048
**2641**	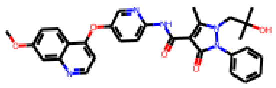	0.458904	COc1ccc2c(=O)cc(CNC(=O)c3c(C)n(C)n(-c4ccccc4)c3=O)nc3cccc3c2n1	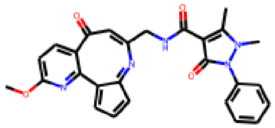	9.0	0.768132	3.608437	2.513170	5	0.823048
**2642**	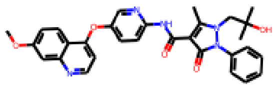	0.458857	COc1ccc2c(=O)cc(CNC(=O)c3c(C)n(C)n(-c4ccccc4)c3=O)nc3cccc3c2n1	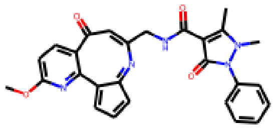	9.0	0.768132	3.608437	2.513170	5	0.823048
**2643**	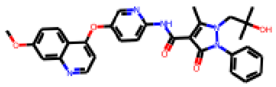	0.458857	COc1ccc2c(=O)cc(CNC(=O)c3c(C)n(C)n(-c4ccccc4)c3=O)nc3cccc3c2n1	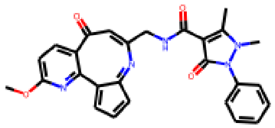	9.0	0.768132	3.608437	2.513170	5	0.823048

	src known mol	similarity	smiles	mol	Class	qed	logP	SAS	rings	kinase inhibition likeliness
**2644**	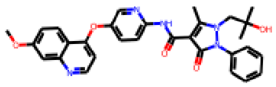	0.458844	COc1ccc2c(=O)cc(CNC(=O)c3c(C)n(C)n(-c4ccccc4)c3=O)nc3cccc3c2n1	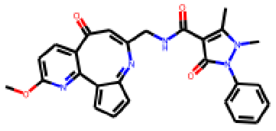	9.0	0.768132	3.608437	2.513170	5	0.823048
**2645**	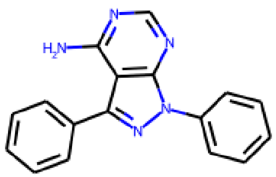	0.458642	Cc1ccc(C(=O)Oc2ccon2)cc1-c1ncnc2c1cnn2-c1ccccc1	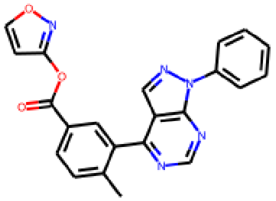	8.0	0.635315	4.542409	3.856266	5	0.760056
**2646**	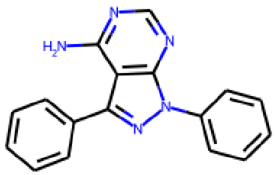	0.458642	Cc1ccc(C(=O)Oc2ccon2)cc1-c1ncnc2c1cnn2-c1ccccc1	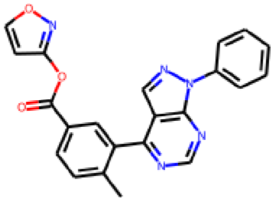	8.0	0.635315	4.542409	3.856266	5	0.760056
**2647**	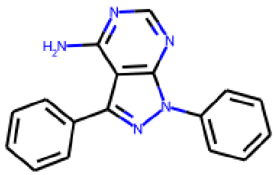	0.458642	Cc1ccc(C(=O)Oc2ccon2)cc1-c1ncnc2c1cnn2-c1ccccc1	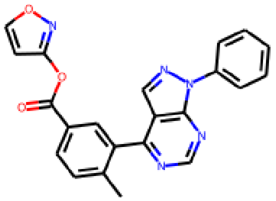	8.0	0.635315	4.542409	3.856266	5	0.760056

	src known mol	similarity	smiles	mol	Class	qed	logP	SAS	rings	kinase inhibition likeliness
**2648**	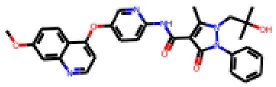	0.458515	COc1ccc2c(=O)cc(CNC(=O)c3c(C)n(C)n(-c4ccccc4)c3=O)nc3cccc2c3c1	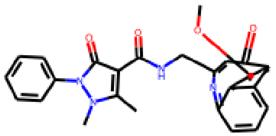	9.0	0.638163	3.995589	2.988471	5	0.774651
**2649**	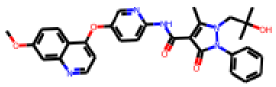	0.458492	COc1ccc2c(=O)cc(CNC(=O)c3c(C)n(C)n(-c4ccccc4)c3=O)nc3cccc2c3c1	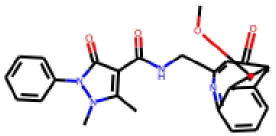	9.0	0.638163	3.995589	2.988471	5	0.774651
**2650**	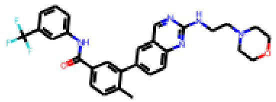	0.458484	Cc1ccc(NC(=O)c2cccc(C(F)(F)F)c2)cc1-c1ccc2nc(O)ncc2c1	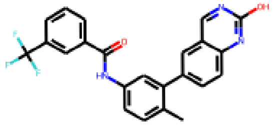	6.0	0.638163	3.995589	2.988471	4	0.818430
**2651**	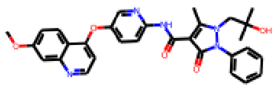	0.458020	COc1ccc2c(=O)cc(CNC(=O)c3c(C)n(C)n(-c4ccccc4)c3=O)nc3cccc3c2n1	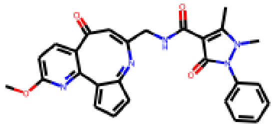	9.0	0.768132	3.608437	2.513170	5	0.823048

	src known mol	similarity	smiles	mol	Class	qed	logP	SAS	rings	kinase inhibition likeliness
**2652**	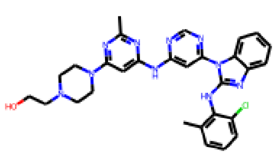	0.457862	Cc1nc(Nc2ccc(C(=O)Oc3c(C)cccc3Cl)s2)cc(N2CCNC(=O)C2=O)n1	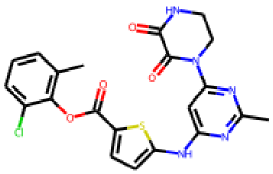	2.0	0.584673	2.312750	2.571988	4	0.770333
**2653**	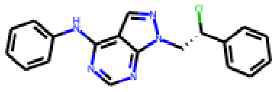	0.457728	Cc1ccc(C(=O)Nc2ccon2)cc1Nc1ncnc2c1cnn2C1cccccc1	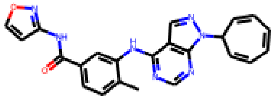	8.0	0.623362	2.525411	2.997881	5	0.770298
**2654**	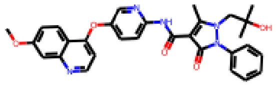	0.457706	COc1ccc2c(=O)cc(CNC(=O)c3c(C)n(C)n(-c4ccccc4)c3=O)nc3cccc3c2n1	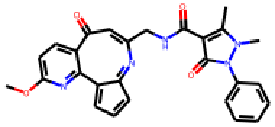	9.0	0.768132	3.608437	2.513170	5	0.823048
**2655**	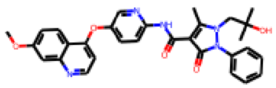	0.457646	COc1ccc2c(=O)cc(CNC(=O)c3c(C)n(C)n(-c4ccccc4)c3=O)nc3cccc3c2n1	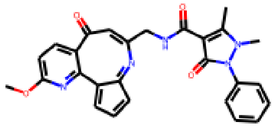	9.0	0.768132	3.608437	2.513170	5	0.823048

	src known mol	similarity	smiles	mol	Class	qed	logP	SAS	rings	kinase inhibition likeliness
**2656**	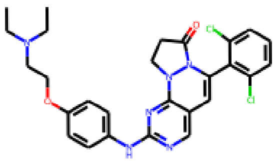	0.457498	COc1cc(Nc2ncc(C)c(-c3ccc4c(n3)NC(=O)C(C)(C)C4)n2)cc(OC)c1OC	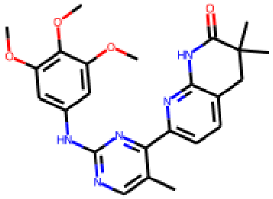	4.0	0.768132	3.608437	2.513170	4	0.757734
**2657**	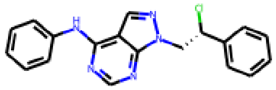	0.457440	Cc1ccc(C(=O)Nc2ccon2)cc1Nc1ncnc2c1cnn2C1cccccc1	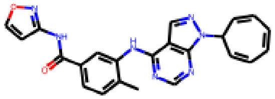	8.0	0.623362	2.525411	2.997881	5	0.770298
**2658**	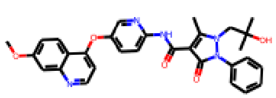	0.457355	COc1ccc2c(=O)cc(CNC(=O)c3c(C)n(C)n(-c4ccccc4)c3=O)nc3cccc3c2n1	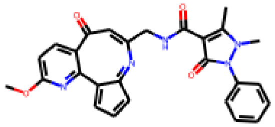	9.0	0.768132	3.608437	2.513170	5	0.823048
**2659**	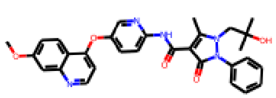	0.457221	COc1ccc2c(=O)cc(CNC(=O)c3c(C)n(C)n(-c4ccccc4)c3=O)nc3cccc2c3c1	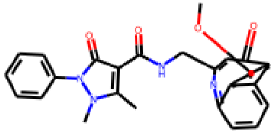	9.0	0.638163	3.995589	2.988471	5	0.774651

	src known mol	similarity	smiles	mol	Class	qed	logP	SAS	rings	kinase inhibition likeliness
**2660**	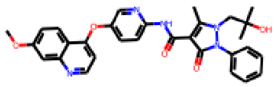	0.457221	COc1ccc2c(=O)cc(CNC(=O)c3c(C)n(C)n(-c4ccccc4)c3=O)nc3cccc2c3c1	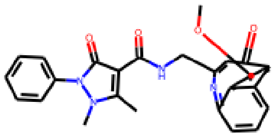	9.0	0.638163	3.995589	2.988471	5	0.774651
**2661**	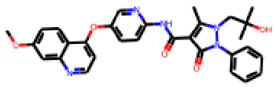	0.456995	COc1ccc2c(=O)cc(CNC(=O)c3c(C)n(C)n(-c4ccccc4)c3=O)nc3cccc3c2n1	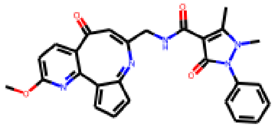	9.0	0.768132	3.608437	2.513170	5	0.823048
**2662**	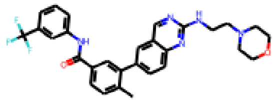	0.456969	Cc1ccc(C(=O)Nc2cccc(C(F)(C)F)c2C)cc1-c1ccc2nc(C)ccccc(F)nc2c1	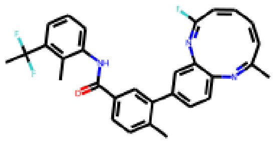	6.0	0.742375	4.384502	0.286234	4	0.779167
**2663**	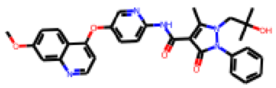	0.456679	COc1ccc2c(=O)cc(CNC(=O)c3c(C)n(C)n(-c4ccccc4)c3=O)nc3cccc3c2n1	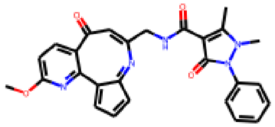	9.0	0.768132	3.608437	2.513170	5	0.823048

	src known mol	similarity	smiles	mol	Class	qed	logP	SAS	rings	kinase inhibition likeliness
**2664**	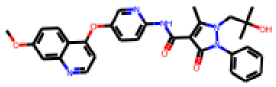	0.456472	COc1ccc2c(=O)cc(CNC(=O)c3c(C)n(C)n(-c4ccccc4)c3=O)nc3cccc3c2n1	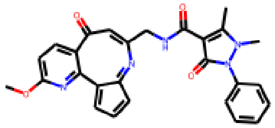	9.0	0.768132	3.608437	2.513170	5	0.823048
**2665**	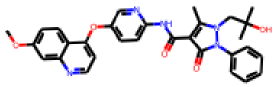	0.456472	COc1ccc2c(=O)cc(CNC(=O)c3c(C)n(C)n(-c4ccccc4)c3=O)nc3cccc3c2n1	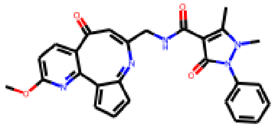	9.0	0.768132	3.608437	2.513170	5	0.823048
**2666**	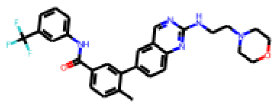	0.456454	Cc1ccc(C(=O)Nc2cccc(C(F)(F)F)c2C)cc1-c1ccc2nc(O)ncc2c1	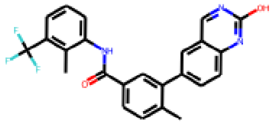	6.0	0.638163	3.995589	2.988471	4	0.808346
**2667**	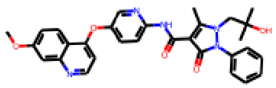	0.456361	COc1ccc2c(=O)cc(CNC(=O)c3c(C)n(C)n(-c4ccccc4)c3=O)nc3cccc3c2n1	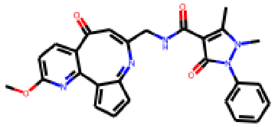	9.0	0.768132	3.608437	2.513170	5	0.823048

	src known mol	similarity	smiles	mol	Class	qed	logP	SAS	rings	kinase inhibition likeliness
**2668**	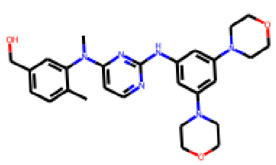	0.456359	NC(=O)c1ccccc1Nc1nc(Nc2ccc(N3CCCC3=O)cc2)ncc1Cl	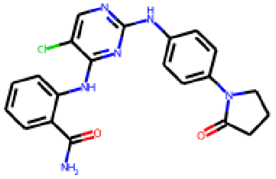	7.0	0.768132	3.608437	2.513170	4	0.773984
**2669**	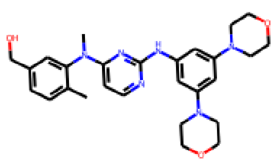	0.456229	NC(=O)c1ccccc1Nc1nc(Nc2ccc(N3CCCC3=O)cc2)ncc1Cl	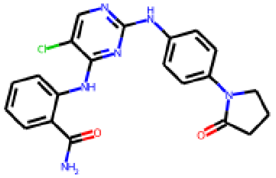	7.0	0.768132	3.608437	2.513170	4	0.773984
**2670**	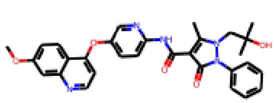	0.456150	COc1ccc2c(=O)cc(CNC(=O)c3c(C)n(C)n(-c4ccccc4)c3=O)nc3cccc3c2n1	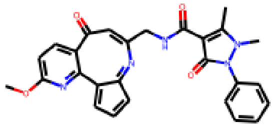	9.0	0.768132	3.608437	2.513170	5	0.823048
**2671**	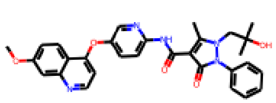	0.455950	COc1ccc2c(=O)cc(CNC(=O)c3c(C)n(C)n(-c4ccccc4)c3=O)nc3cccc3c2n1	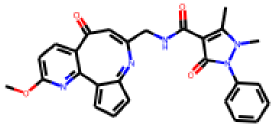	9.0	0.768132	3.608437	2.513170	5	0.823048

	src known mol	similarity	smiles	mol	Class	qed	logP	SAS	rings	kinase inhibition likeliness
**2672**	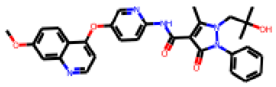	0.455950	COc1ccc2c(=O)cc(CNC(=O)c3c(C)n(C)n(-c4ccccc4)c3=O)nc3cccc3c2n1	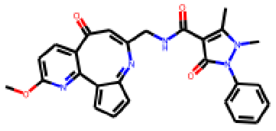	9.0	0.768132	3.608437	2.513170	5	0.823048
**2673**	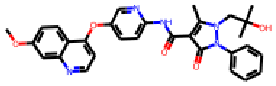	0.455950	COc1ccc2c(=O)cc(CNC(=O)c3c(C)n(C)n(-c4ccccc4)c3=O)nc3cccc3c2n1	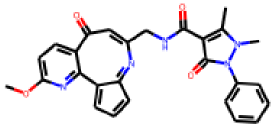	9.0	0.768132	3.608437	2.513170	5	0.823048
**2674**	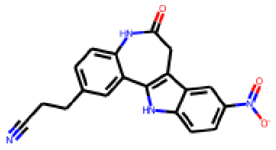	0.455911	CCNC(=O)N1CCC(Nc2n(C(C)c3c(-c4ccccc4)[nH]c4ccccc34)n2)CC1	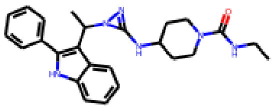	7.0	0.623584	3.603734	4.478009	5	0.804049
**2675**	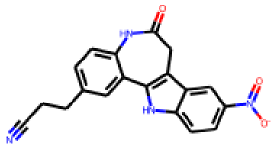	0.455911	CCNC(=O)N1CCC(Nc2n(C(C)c3c(-c4ccccc4)[nH]c4ccccc34)n2)CC1	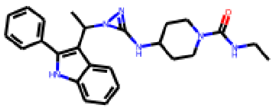	7.0	0.623584	3.603734	4.478009	5	0.804049

	src known mol	similarity	smiles	mol	Class	qed	logP	SAS	rings	kinase inhibition likeliness
**2676**	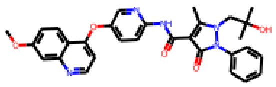	0.455891	COc1ccc2c(=O)cc(CNC(=O)c3c(C)n(C)n(-c4ccccc4)c3=O)nc3cccc3c2n1	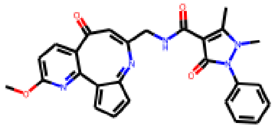	9.0	0.768132	3.608437	2.513170	5	0.823048
**2677**	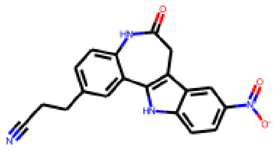	0.455688	CCNC(=O)N1CCC(Nc2n(C(C)c3c(-c4ccccc4)[nH]c4ccccc34)n2)CC1	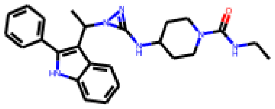	7.0	0.623584	3.603734	4.478009	5	0.804049
**2678**	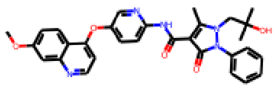	0.455682	COc1ccc2c(=O)cc(CNC(=O)c3c(C)n(C)n(-c4ccccc4)c3=O)nc3cccc2c3c1	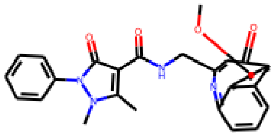	9.0	0.638163	3.995589	2.988471	5	0.774651
**2679**	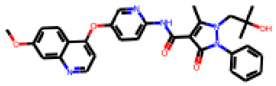	0.455378	COc1ccc2c(=O)cc(CNC(=O)c3c(C)n(C)n(-c4ccccc4)c3=O)nc3cccc3c2n1	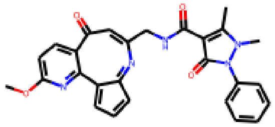	9.0	0.768132	3.608437	2.513170	5	0.823048

	src known mol	similarity	smiles	mol	Class	qed	logP	SAS	rings	kinase inhibition likeliness
**2680**	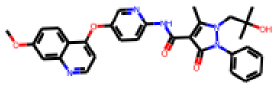	0.455378	COc1ccc2c(=O)cc(CNC(=O)c3c(C)n(C)n(-c4ccccc4)c3=O)nc3cccc3c2n1	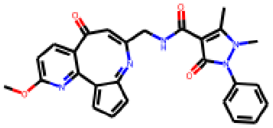	9.0	0.768132	3.608437	2.513170	5	0.823048
**2681**	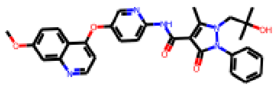	0.455378	COc1ccc2c(=O)cc(CNC(=O)c3c(C)n(C)n(-c4ccccc4)c3=O)nc3cccc3c2n1	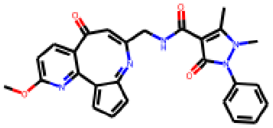	9.0	0.768132	3.608437	2.513170	5	0.823048
**2682**	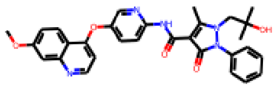	0.455378	COc1ccc2c(=O)cc(CNC(=O)c3c(C)n(C)n(-c4ccccc4)c3=O)nc3cccc3c2n1	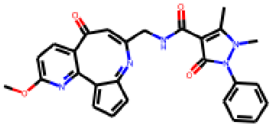	9.0	0.768132	3.608437	2.513170	5	0.823048
**2683**	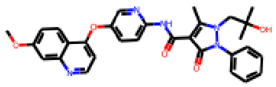	0.455378	COc1ccc2c(=O)cc(CNC(=O)c3c(C)n(C)n(-c4ccccc4)c3=O)nc3cccc3c2n1	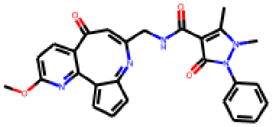	9.0	0.768132	3.608437	2.513170	5	0.823048

	src known mol	similarity	smiles	mol	Class	qed	logP	SAS	rings	kinase inhibition likeliness
**2684**	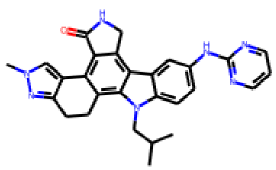	0.455290	C#Cc1ccc(C(=O)OC)cc1-c1C(=O)c-n2nc(-c3ccc(F)cc3F)cc2C1	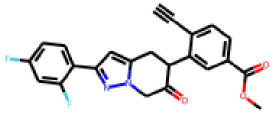	8.0	0.768132	3.608437	2.513170	4	0.763730
**2685**	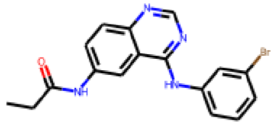	0.455272	O=C(CNC(=O)Oc1ccccc1)Nc1ccc2ncnc(Nc3cccc(Cl)c3)c2c1	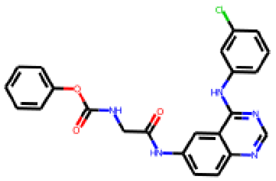	3.0	0.638163	3.995589	2.988471	4	0.793889
**2686**	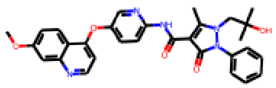	0.455041	COc1ccc2c(=O)cc(CNC(=O)c3c(C)n(C)n(-c4ccccc4)c3=O)nc3cccc2c3c1	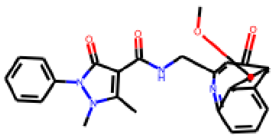	9.0	0.638163	3.995589	2.988471	5	0.774651
**2687**	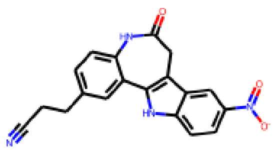	0.455020	CCNC(=O)N1CCC(Nc2n(C(C)c3c(-c4ccccc4)[nH]c4ccccc34)n2)CC1	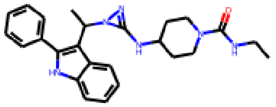	7.0	0.623584	3.603734	4.478009	5	0.804049

	src known mol	similarity	smiles	mol	Class	qed	logP	SAS	rings	kinase inhibition likeliness
**2688**	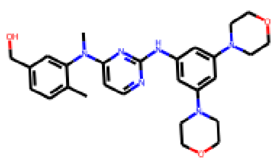	0.454699	NC(=O)c1ccccc1Nc1nc(Nc2ccc(N3CCCC3=O)cc2)ncc1Cl	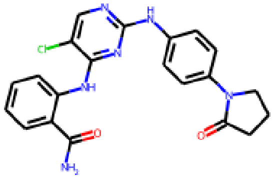	7.0	0.768132	3.608437	2.513170	4	0.773984
**2689**	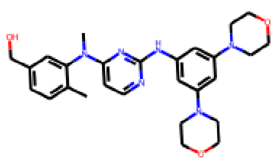	0.454545	NC(=O)c1ccccc1Nc1nc(Nc2ccc(N3CCCC3=O)cc2)ncc1Cl	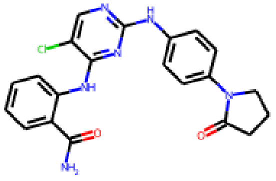	7.0	0.768132	3.608437	2.513170	4	0.773984
**2690**	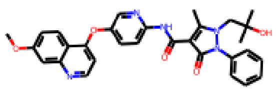	0.454493	COc1ccc2c(=O)cc(CNC(=O)c3c(C)n(C)n(-c4ccccc4)c3=O)nc3cccc3c2n1	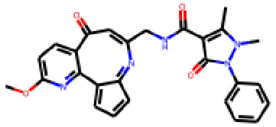	9.0	0.768132	3.608437	2.513170	5	0.823048
**2691**	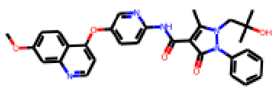	0.454287	COc1ccc2c(=O)cc(CNC(=O)c3c(C)n(C)n(-c4ccccc4)c3=O)nc3cccc3c2n1	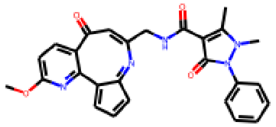	9.0	0.768132	3.608437	2.513170	5	0.823048

	src known mol	similarity	smiles	mol	Class	qed	logP	SAS	rings	kinase inhibition likeliness
**2692**	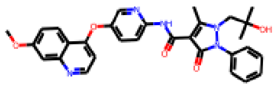	0.454201	COc1ccc2c(=O)cc(CNC(=O)c3c(C)n(C)n(-c4ccccc4)c3=O)nc3cccc2c3c1	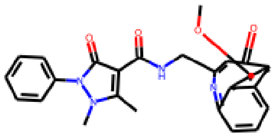	9.0	0.638163	3.995589	2.988471	5	0.774651
**2693**	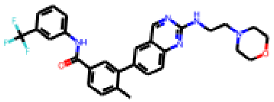	0.454132	Cc1ccc(C(=O)Nc2cccc(C(F)(C)F)c2C)cc1-c1ccc2nc(C)ccccc(F)nc2c1	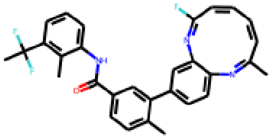	6.0	0.742375	4.384502	0.286234	4	0.779167
**2694**	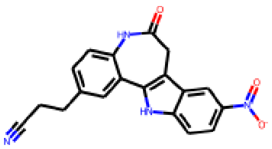	0.454131	CCNC(=O)N1CCC(Nc2n(C(C)c3c(-c4ccccc4)[nH]c4ccccc34)n2)CC1	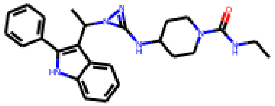	7.0	0.623584	3.603734	4.478009	5	0.804049
**2695**	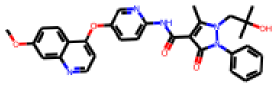	0.453905	COc1ccc2c(=O)cc(CNC(=O)c3c(C)n(C)n(-c4ccccc4)c3=O)nc3cccc2c3c1	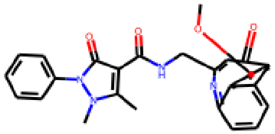	9.0	0.638163	3.995589	2.988471	5	0.774651

	src known mol	similarity	smiles	mol	Class	qed	logP	SAS	rings	kinase inhibition likeliness
**2696**	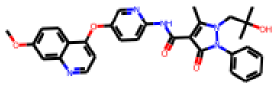	0.453824	COc1ccc2c(=O)cc(CNC(=O)c3c(C)n(C)n(-c4ccccc4)c3=O)nc3cccc3c2n1	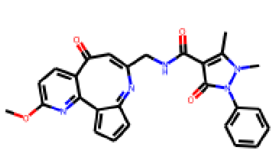	9.0	0.768132	3.608437	2.513170	5	0.823048
**2697**	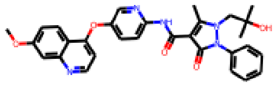	0.453714	COc1ccc2c(=O)cc(CNC(=O)c3c(C)n(C)n(-c4ccccc4)c3=O)nc3cccc3c2n1	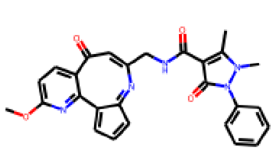	9.0	0.768132	3.608437	2.513170	5	0.823048
**2698**	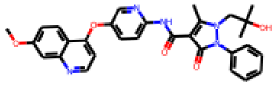	0.453704	COc1ccc2c(=O)cc(CNC(=O)c3c(C)n(C)n(-c4ccccc4)c3=O)nc3ccccc2c3c1	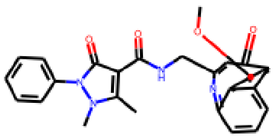	9.0	0.638163	3.995589	2.988471	5	0.774651
**2699**	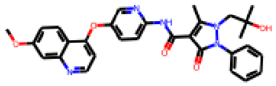	0.453555	COc1ccc2c(=O)cc(CNC(=O)c3c(C)n(C)n(-c4ccccc4)c3=O)nc3cccc3c2n1	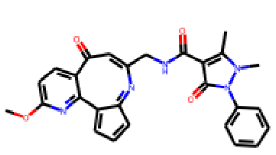	9.0	0.768132	3.608437	2.513170	5	0.823048

	src known mol	similarity	smiles	mol	Class	qed	logP	SAS	rings	kinase inhibition likeliness
**2700**	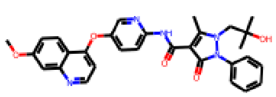	0.453542	COc1ccc2c(=O)cc(CNC(=O)c3c(C)n(C)n(-c4ccccc4)c3=O)nc3cccc3c2n1	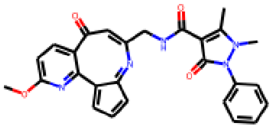	9.0	0.768132	3.608437	2.513170	5	0.823048
**2701**	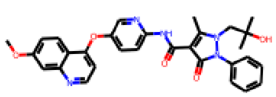	0.453349	COc1ccc2c(=O)cc(CNC(=O)c3c(C)n(C)n(-c4ccccc4)c3=O)nc3cccc3c2n1	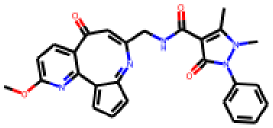	9.0	0.768132	3.608437	2.513170	5	0.823048
**2702**	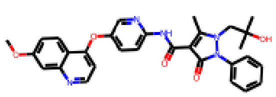	0.453089	COc1ccc2c(=O)cc(CNC(=O)c3c(C)n(C)n(-c4ccccc4)c3=O)nc3cccc3c2n1	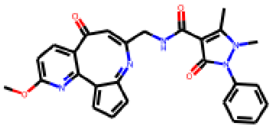	9.0	0.768132	3.608437	2.513170	5	0.823048
**2703**	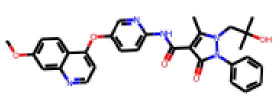	0.453089	COc1ccc2c(=O)cc(CNC(=O)c3c(C)n(C)n(-c4ccccc4)c3=O)nc3cccc3c2n1	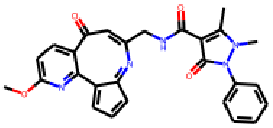	9.0	0.768132	3.608437	2.513170	5	0.823048

	src known mol	similarity	smiles	mol	Class	qed	logP	SAS	rings	kinase inhibition likeliness
**2704**	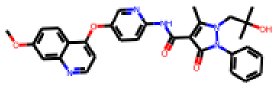	0.453089	COc1ccc2c(=O)cc(CNC(=O)c3c(C)n(C)n(-c4ccccc4)c3=O)nc3cccc3c2n1	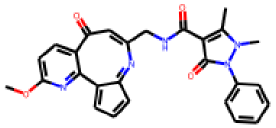	9.0	0.768132	3.608437	2.513170	5	0.823048
**2705**	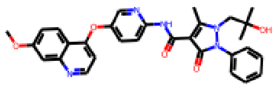	0.453089	COc1ccc2c(=O)cc(CNC(=O)c3c(C)n(C)n(-c4ccccc4)c3=O)nc3cccc3c2n1	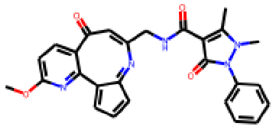	9.0	0.768132	3.608437	2.513170	5	0.823048
**2706**	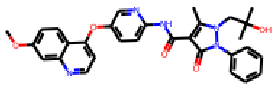	0.453089	COc1ccc2c(=O)cc(CNC(=O)c3c(C)n(C)n(-c4ccccc4)c3=O)nc3cccc3c2n1	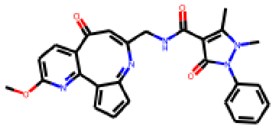	9.0	0.768132	3.608437	2.513170	5	0.823048
**2707**	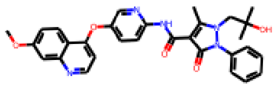	0.453089	COc1ccc2c(=O)cc(CNC(=O)c3c(C)n(C)n(-c4ccccc4)c3=O)nc3cccc3c2n1	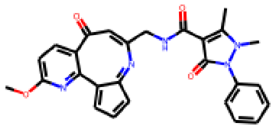	9.0	0.768132	3.608437	2.513170	5	0.823048

	src known mol	similarity	smiles	mol	Class	qed	logP	SAS	rings	kinase inhibition likeliness
**2708**	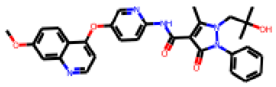	0.453089	COc1ccc2c(=O)cc(CNC(=O)c3c(C)n(C)n(-c4ccccc4)c3=O)nc3cccc3c2n1	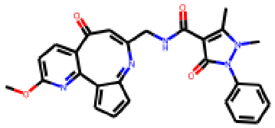	9.0	0.768132	3.608437	2.513170	5	0.823048
**2709**	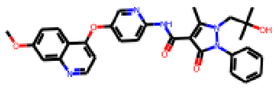	0.453089	COc1ccc2c(=O)cc(CNC(=O)c3c(C)n(C)n(-c4ccccc4)c3=O)nc3cccc3c2n1	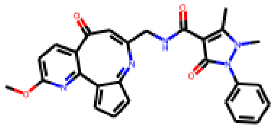	9.0	0.768132	3.608437	2.513170	5	0.823048
**2710**	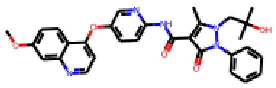	0.452830	COc1ccc2c(=O)cc(CNC(=O)c3c(C)n(C)n(-c4ccccc4)c3=O)nc3cccc3c2n1	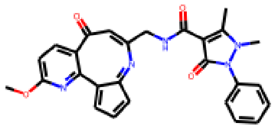	9.0	0.768132	3.608437	2.513170	5	0.823048
**2711**	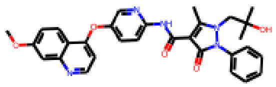	0.452830	COc1ccc2c(=O)cc(CNC(=O)c3c(C)n(C)n(-c4ccccc4)c3=O)nc3cccc3c2n1	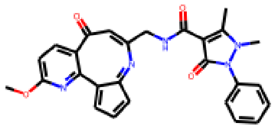	9.0	0.768132	3.608437	2.513170	5	0.823048

	src known mol	similarity	smiles	mol	Class	qed	logP	SAS	rings	kinase inhibition likeliness
**2712**	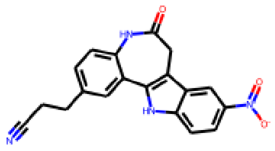	0.452805	CCNC(=O)N1CCC(Nc2n(C(C)c3c(-c4ccccc4)[nH]c4ccccc34)n2)CC1	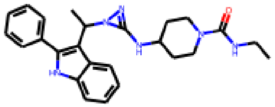	7.0	0.623584	3.603734	4.478009	5	0.804049
**2713**	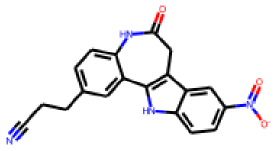	0.452805	CCNC(=O)N1CCC(Nc2n(C(C)c3c(-c4ccccc4)[nH]c4ccccc34)n2)CC1	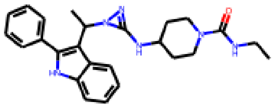	7.0	0.623584	3.603734	4.478009	5	0.804049
**2714**	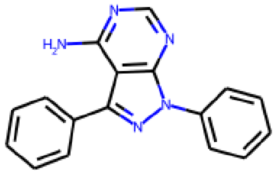	0.452689	Cc1ccc(C(=O)Oc2ccon2)cc1-c1ncnc2c1cnn2-c1ccccc1	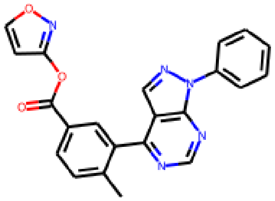	8.0	0.635315	4.542409	3.856266	5	0.760056
**2715**	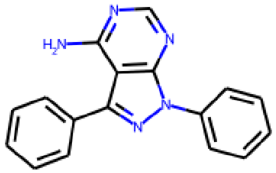	0.452689	Cc1ccc(C(=O)Oc2ccon2)cc1-c1ncnc2c1cnn2-c1ccccc1	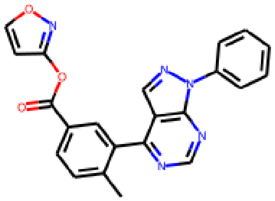	8.0	0.635315	4.542409	3.856266	5	0.760056

	src known mol	similarity	smiles	mol	Class	qed	logP	SAS	rings	kinase inhibition likeliness
**2716**	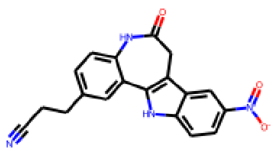	0.452521	CCNC(=O)N1CCC(Nc2n(C(C)c3c(-c4ccccc4)[nH]c4ccccc34)n2)CC1	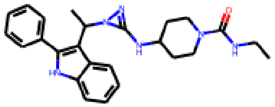	7.0	0.623584	3.603734	4.478009	5	0.804049
**2717**	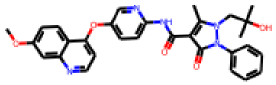	0.452408	COc1ccc2c(=O)cc(CNC(=O)c3c(C)n(C)n(-c4ccccc4)c3=O)nc3cccc3c2n1	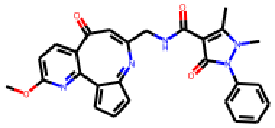	9.0	0.768132	3.608437	2.513170	5	0.823048
**2718**	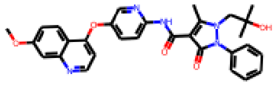	0.452408	COc1ccc2c(=O)cc(CNC(=O)c3c(C)n(C)n(-c4ccccc4)c3=O)nc3cccc3c2n1	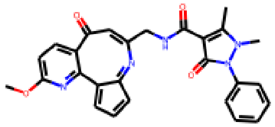	9.0	0.768132	3.608437	2.513170	5	0.823048
**2719**	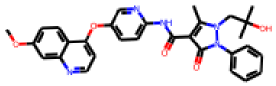	0.452408	COc1ccc2c(=O)cc(CNC(=O)c3c(C)n(C)n(-c4ccccc4)c3=O)nc3cccc3c2n1	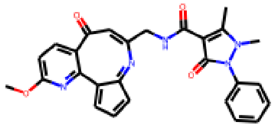	9.0	0.768132	3.608437	2.513170	5	0.823048

	src known mol	similarity	smiles	mol	Class	qed	logP	SAS	rings	kinase inhibition likeliness
**2720**	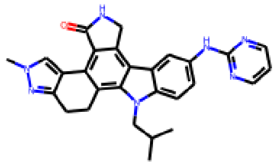	0.452306	C#Cc1ccc(C(=O)OC)cc1-c1C(=O)c-n2nc(-c3ccc(F)cc3F)cc2C1	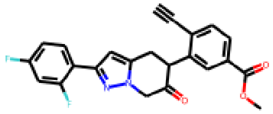	8.0	0.768132	3.608437	2.513170	4	0.763730
**2721**	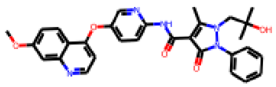	0.452303	COc1ccc2c(=O)cc(CNC(=O)c3c(C)n(C)n(-c4ccccc4)c3=O)nc3cccc2c3c1	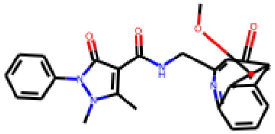	9.0	0.638163	3.995589	2.988471	5	0.774651
**2722**	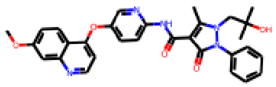	0.452226	COc1ccc2c(=O)cc(CNC(=O)c3c(C)n(C)n(-c4ccccc4)c3=O)nc3cccc2c3c1	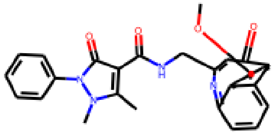	9.0	0.638163	3.995589	2.988471	5	0.774651
**2723**	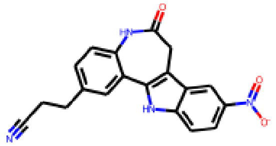	0.452203	CCNC(=O)N1CCC(Nc2n(C(C)c3c(-c4ccccc4)[nH]c4ccccc34)n2)CC1	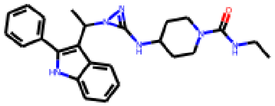	7.0	0.623584	3.603734	4.478009	5	0.804049

	src known mol	similarity	smiles	mol	Class	qed	logP	SAS	rings	kinase inhibition likeliness
**2724**	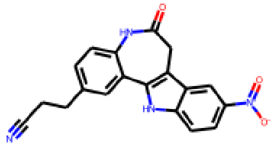	0.452203	CCNC(=O)N1CCC(Nc2n(C(C)c3c(-c4ccccc4)[nH]c4ccccc34)n2)CC1	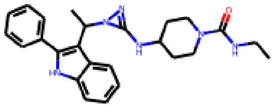	7.0	0.623584	3.603734	4.478009	5	0.804049
**2725**	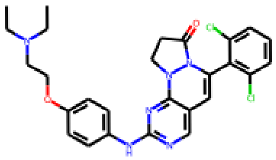	0.452055	COc1cc(Nc2ncc(C)c(-c3ccc4c(n3)NC(=O)C(C)(C)C4)n2)cc(OC)c1OC	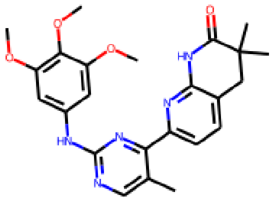	4.0	0.768132	3.608437	2.513170	4	0.757734
**2726**	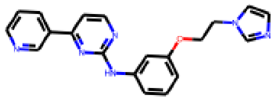	0.452043	Cc1ccc(OC(=O)c2ccccc2)cc1Nc1nccc(-c2cccnc2)n1	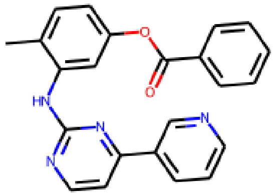	0.0	0.638163	3.995589	2.988471	4	0.775468
**2727**	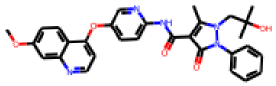	0.451980	COc1ccc2c(=O)cc(CNC(=O)c3c(C)n(C)n(-c4ccccc4)c3=O)nc3cccc2c3c1	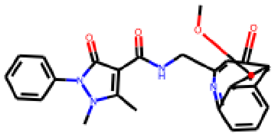	9.0	0.638163	3.995589	2.988471	5	0.774651

	src known mol	similarity	smiles	mol	Class	qed	logP	SAS	rings	kinase inhibition likeliness
**2728**	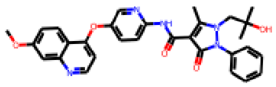	0.451949	COc1ccc2c(=O)cc(CNC(=O)c3c(C)n(C)n(-c4ccccc4)c3=O)nc3cccc2c3c1	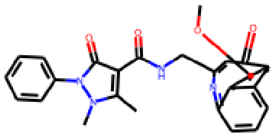	9.0	0.638163	3.995589	2.988471	5	0.774651
**2729**	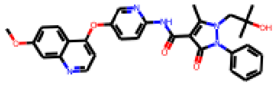	0.451890	COc1ccc2c(=O)cc(CNC(=O)c3c(C)n(C)n(-c4ccccc4)c3=O)nc3cccc3c2n1	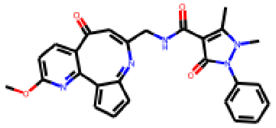	9.0	0.768132	3.608437	2.513170	5	0.823048
**2730**	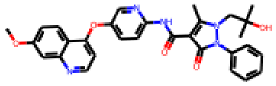	0.451818	COc1ccc2c(=O)cc(CNC(=O)c3c(C)n(C)n(-c4ccccc4)c3=O)nc3cccc3c2n1	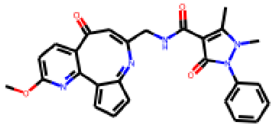	9.0	0.768132	3.608437	2.513170	5	0.823048
**2731**	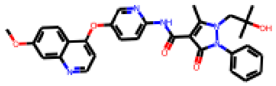	0.451669	COc1ccc2c(=O)cc(CNC(=O)c3c(C)n(C)n(-c4ccccc4)c3=O)nc3cccc3c2n1	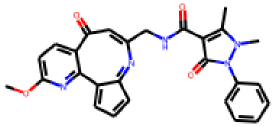	9.0	0.768132	3.608437	2.513170	5	0.823048

	src known mol	similarity	smiles	mol	Class	qed	logP	SAS	rings	kinase inhibition likeliness
**2732**	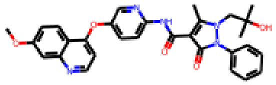	0.451435	COc1ccc2c(=O)cc(CNC(=O)c3c(C)n(C)n(-c4ccccc4)c3=O)nc3cccc2c3c1	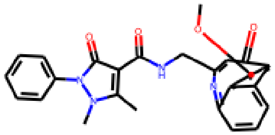	9.0	0.638163	3.995589	2.988471	5	0.774651
**2733**	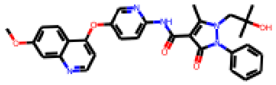	0.451226	COc1ccc2c(=O)cc(CNC(=O)c3c(C)n(C)n(-c4ccccc4)c3=O)nc3cccc2c3c1	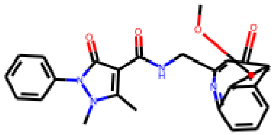	9.0	0.638163	3.995589	2.988471	5	0.774651
**2734**	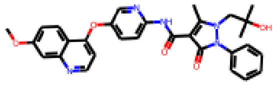	0.451226	COc1ccc2c(=O)cc(CNC(=O)c3c(C)n(C)n(-c4ccccc4)c3=O)nc3cccc2c3c1	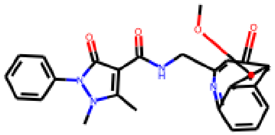	9.0	0.638163	3.995589	2.988471	5	0.774651
**2735**	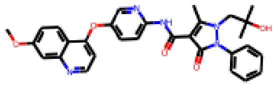	0.451136	COc1ccc2c(=O)cc(CNC(=O)c3c(C)n(C)n(-c4ccccc4)c3=O)nc3cccc3c2n1	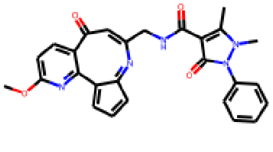	9.0	0.768132	3.608437	2.513170	5	0.823048

	src known mol	similarity	smiles	mol	Class	qed	logP	SAS	rings	kinase inhibition likeliness
**2736**	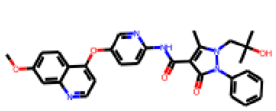	0.450820	COc1ccc2c(=O)cc(CNC(=O)c3c(C)n(C)n(-c4ccccc4)c3=O)nc3cccc2c3c1	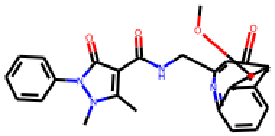	9.0	0.638163	3.995589	2.988471	5	0.774651
**2737**	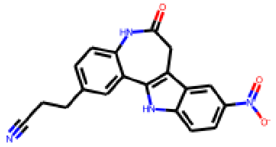	0.450814	CCNC(=O)N1CCC(Nc2n(C(C)c3c(-c4ccccc4)[nH]c4ccccc34)n2)CC1	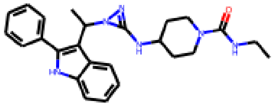	7.0	0.623584	3.603734	4.478009	5	0.804049
**2738**	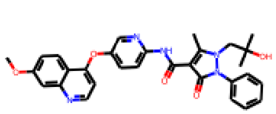	0.450788	COc1ccc2c(=O)cc(CNC(=O)c3c(C)n(C)n(-c4ccccc4)c3=O)nc3cccc2c3c1	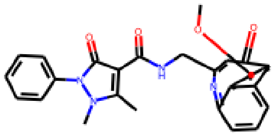	9.0	0.638163	3.995589	2.988471	5	0.774651
**2739**	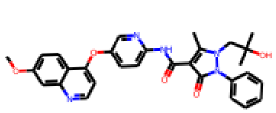	0.450768	COc1ccc2c(=O)cc(CNC(=O)c3c(C)n(C)n(-c4ccccc4)c3=O)nc3cccc3c2n1	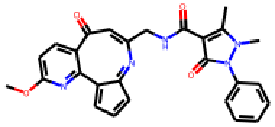	9.0	0.768132	3.608437	2.513170	5	0.823048

	src known mol	similarity	smiles	mol	Class	qed	logP	SAS	rings	kinase inhibition likeliness
**2740**	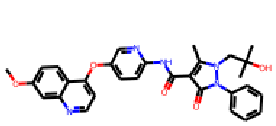	0.450768	COc1ccc2c(=O)cc(CNC(=O)c3c(C)n(C)n(-c4ccccc4)c3=O)nc3cccc3c2n1	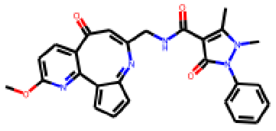	9.0	0.768132	3.608437	2.513170	5	0.823048
**2741**	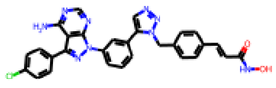	0.450752	Cc1ccc(C(=O)Nc2ccco2)cc1Nc1ncnc2c1cnn2-c1ccccc1	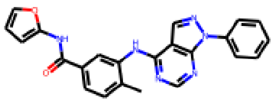	8.0	0.761557	4.954058	3.852993	5	0.826102
**2742**	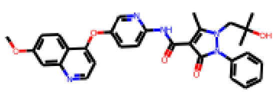	0.450549	COc1ccc2c(=O)cc(CNC(=O)c3c(C)n(C)n(-c4ccccc4)c3=O)nc3cccc2c3c1	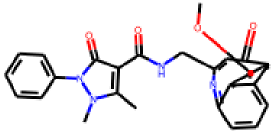	9.0	0.638163	3.995589	2.988471	5	0.774651
**2743**	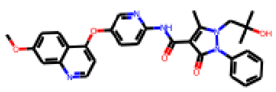	0.450357	COc1ccc2c(=O)cc(CNC(=O)c3c(C)n(C)n(-c4ccccc4)c3=O)nc3cccc2c3c1	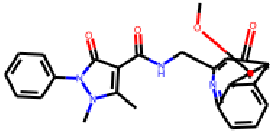	9.0	0.638163	3.995589	2.988471	5	0.774651

	src known mol	similarity	smiles	mol	Class	qed	logP	SAS	rings	kinase inhibition likeliness
**2744**	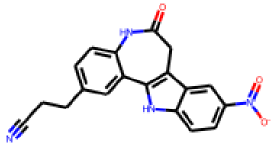	0.450327	CCNC(=O)N1CCC(Nc2n(C(C)c3c(-c4ccccc4)[nH]c4ccccc34)n2)CC1	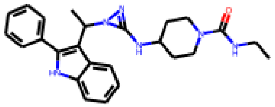	7.0	0.623584	3.603734	4.478009	5	0.804049
**2745**	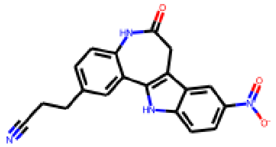	0.450327	CCNC(=O)N1CCC(Nc2n(C(C)c3c(-c4ccccc4)[nH]c4ccccc34)n2)CC1	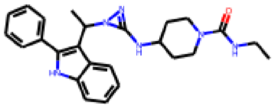	7.0	0.623584	3.603734	4.478009	5	0.804049
**2746**	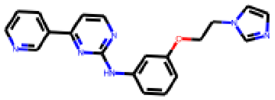	0.450303	Cc1ccc(OC(=O)c2ccccc2)cc1Nc1nccc(-c2cccnc2)n1	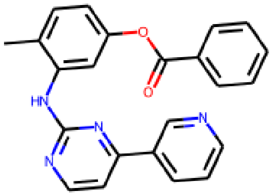	0.0	0.638163	3.995589	2.988471	4	0.775468
**2747**	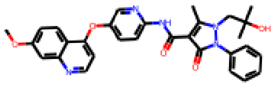	0.450286	COc1ccc2c(=O)cc(CNC(=O)c3c(C)n(C)n(-c4ccccc4)c3=O)nc3cccc3c2n1	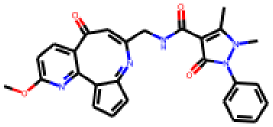	9.0	0.768132	3.608437	2.513170	5	0.823048

	src known mol	similarity	smiles	mol	Class	qed	logP	SAS	rings	kinase inhibition likeliness
**2748**	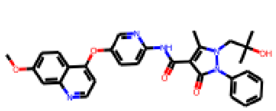	0.450286	COc1ccc2c(=O)cc(CNC(=O)c3c(C)n(C)n(-c4ccccc4)c3=O)nc3cccc3c2n1	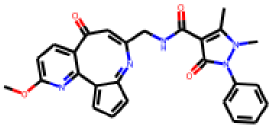	9.0	0.768132	3.608437	2.513170	5	0.823048
**2749**	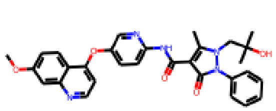	0.450204	COc1ccc2c(=O)cc(CNC(=O)c3c(C)n(C)n(-c4ccccc4)c3=O)nc3cccc3c2n1	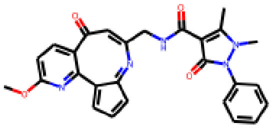	9.0	0.768132	3.608437	2.513170	5	0.823048
**2750**	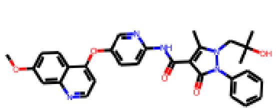	0.450197	COc1ccc2c(=O)cc(CNC(=O)c3c(C)n(C)n(-c4ccccc4)c3=O)nc3cccc3c2n1	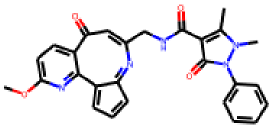	9.0	0.768132	3.608437	2.513170	5	0.823048
**2751**	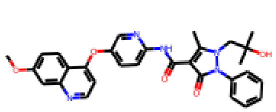	0.450057	COc1ccc2c(=O)cc(CNC(=O)c3c(C)n(C)n(-c4ccccc4)c3=O)nc3cccc3c2n1	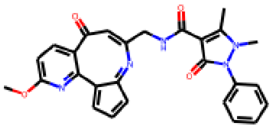	9.0	0.768132	3.608437	2.513170	5	0.823048

	src known mol	similarity	smiles	mol	Class	qed	logP	SAS	rings	kinase inhibition likeliness
**2752**	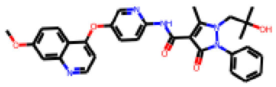	0.450057	COc1ccc2c(=O)cc(CNC(=O)c3c(C)n(C)n(-c4ccccc4)c3=O)nc3cccc3c2n1	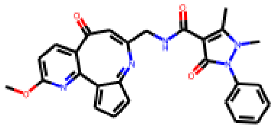	9.0	0.768132	3.608437	2.513170	5	0.823048
**2753**	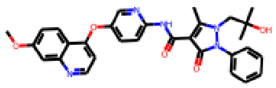	0.450000	COc1ccc2c(=O)cc(CNC(=O)c3c(C)n(C)n(-c4ccccc4)c3=O)nc3cccc3c2n1	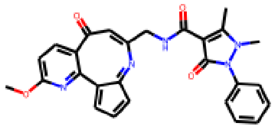	9.0	0.768132	3.608437	2.513170	5	0.823048
**2754**	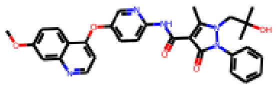	0.449971	COc1ccc2c(=O)cc(CNC(=O)c3c(C)n(C)n(-c4ccccc4)c3=O)nc3cccc3c2n1	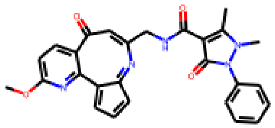	9.0	0.768132	3.608437	2.513170	5	0.823048
**2755**	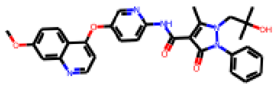	0.449971	COc1ccc2c(=O)cc(CNC(=O)c3c(C)n(C)n(-c4ccccc4)c3=O)nc3cccc3c2n1	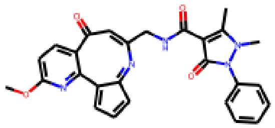	9.0	0.768132	3.608437	2.513170	5	0.823048

	src known mol	similarity	smiles	mol	Class	qed	logP	SAS	rings	kinase inhibition likeliness
**2756**	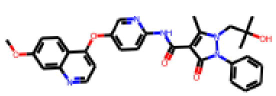	0.449971	COc1ccc2c(=O)cc(CNC(=O)c3c(C)n(C)n(-c4ccccc4)c3=O)nc3cccc3c2n1	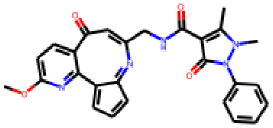	9.0	0.768132	3.608437	2.513170	5	0.823048
**2757**	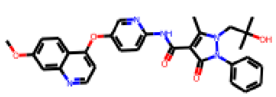	0.449914	COc1ccc2c(=O)cc(CNC(=O)c3c(C)n(C)n(-c4ccccc4)c3=O)nc3cccc3c2n1	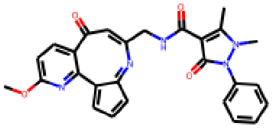	9.0	0.768132	3.608437	2.513170	5	0.823048
**2758**	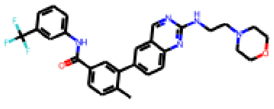	0.449885	Cc1ccc(C(=O)Nc2cccc(C(F)(C)F)c2C)cc1-c1ccc2nc(C)ccccc(F)nc2c1	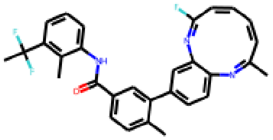	6.0	0.742375	4.384502	0.286234	4	0.779167
**2759**	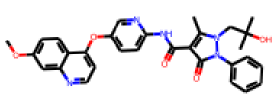	0.449885	COc1ccc2c(=O)cc(CNC(=O)c3c(C)n(C)n(-c4ccccc4)c3=O)nc3cccc3c2n1	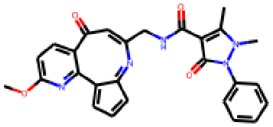	9.0	0.768132	3.608437	2.513170	5	0.823048

	src known mol	similarity	smiles	mol	Class	qed	logP	SAS	rings	kinase inhibition likeliness
**2760**	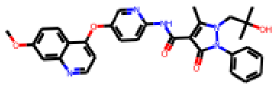	0.449515	COc1ccc2c(=O)cc(CNC(=O)c3c(C)n(C)n(-c4ccccc4)c3=O)nc3cccc3c2n1	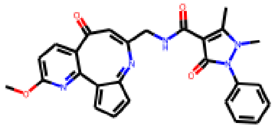	9.0	0.768132	3.608437	2.513170	5	0.823048
**2761**	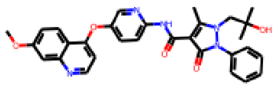	0.449515	COc1ccc2c(=O)cc(CNC(=O)c3c(C)n(C)n(-c4ccccc4)c3=O)nc3cccc3c2n1	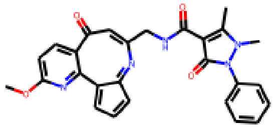	9.0	0.768132	3.608437	2.513170	5	0.823048
**2762**	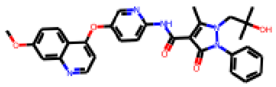	0.449483	COc1ccc2c(=O)cc(CNC(=O)c3c(C)n(C)n(-c4ccccc4)c3=O)nc3cccc3c2n1	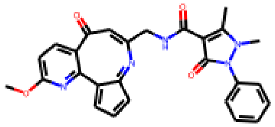	9.0	0.768132	3.608437	2.513170	5	0.823048
**2763**	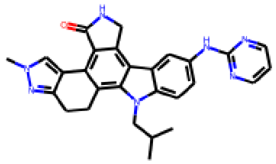	0.449466	Cc1ccc(C(=O)Nc2cccc(C(F)(C)F)c2C)cc1-c1ccc2nc(=O)cccc(F)nc2n1	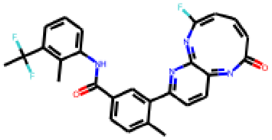	6.0	0.820294	3.038428	1.698172	4	0.833140

	src known mol	similarity	smiles	mol	Class	qed	logP	SAS	rings	kinase inhibition likeliness
**2764**	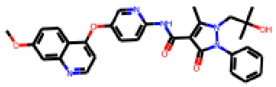	0.449236	COc1ccc2c(=O)cc(CNC(=O)c3c(C)n(C)n(-c4ccccc4)c3=O)nc3cccc2c3c1	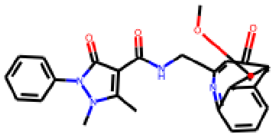	9.0	0.638163	3.995589	2.988471	5	0.774651
**2765**	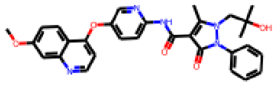	0.449182	COc1ccc2c(=O)cc(CNC(=O)c3c(C)n(C)n(-c4ccccc4)c3=O)nc3cccc3c2n1	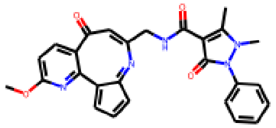	9.0	0.768132	3.608437	2.513170	5	0.823048
**2766**	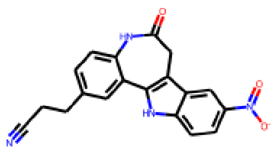	0.448966	CCNC(=O)N1CCC(Nc2n(C(C)c3c(-c4ccccc4)[nH]c4ccccc34)n2)CC1	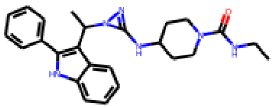	7.0	0.623584	3.603734	4.478009	5	0.804049
**2767**	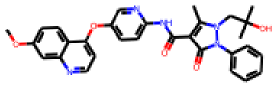	0.448501	COc1ccc2c(=O)cc(CNC(=O)c3c(C)n(C)n(-c4ccccc4)c3=O)nc3cccc2c3c1	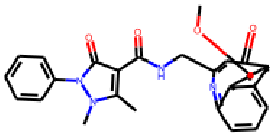	9.0	0.638163	3.995589	2.988471	5	0.774651

	src known mol	similarity	smiles	mol	Class	qed	logP	SAS	rings	kinase inhibition likeliness
**2768**	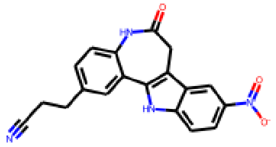	0.448298	CCNC(=O)N1CCC(Nc2n(C(C)c3c(-c4ccccc4)[nH]c4ccccc34)n2)CC1	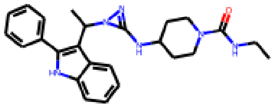	7.0	0.623584	3.603734	4.478009	5	0.804049
**2769**	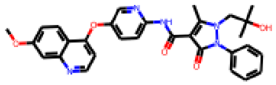	0.448066	COc1ccc2c(=O)cc(CNC(=O)c3c(C)n(C)n(-c4ccccc4)c3=O)nc3cccc2c3c1	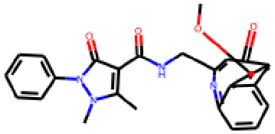	9.0	0.638163	3.995589	2.988471	5	0.774651
**2770**	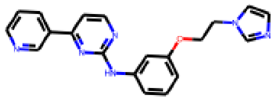	0.447987	Cc1ccc(OC(=O)c2ccccc2)cc1Nc1nccc(-c2cccnc2)n1	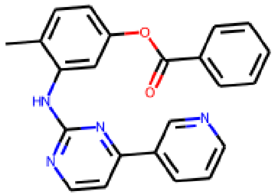	0.0	0.638163	3.995589	2.988471	4	0.775468
**2771**	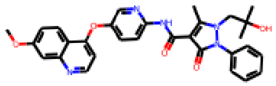	0.447974	COc1ccc2c(=O)cc(CNC(=O)c3c(C)n(C)n(-c4ccccc4)c3=O)nc3cccc2c3c1	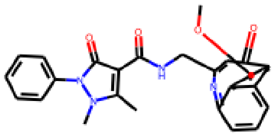	9.0	0.638163	3.995589	2.988471	5	0.774651

	src known mol	similarity	smiles	mol	Class	qed	logP	SAS	rings	kinase inhibition likeliness
**2772**	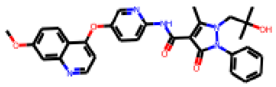	0.447974	COc1ccc2c(=O)cc(CNC(=O)c3c(C)n(C)n(-c4ccccc4)c3=O)nc3cccc2c3c1	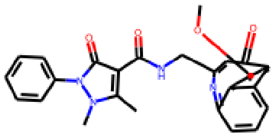	9.0	0.638163	3.995589	2.988471	5	0.774651
**2773**	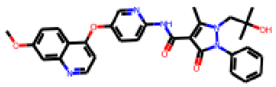	0.447899	COc1ccc2c(=O)cc(CNC(=O)c3c(C)n(C)n(-c4ccccc4)c3=O)nc3cccc3c2n1	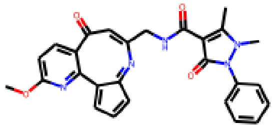	9.0	0.768132	3.608437	2.513170	5	0.823048
**2774**	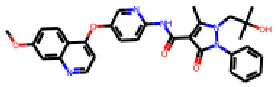	0.447899	COc1ccc2c(=O)cc(CNC(=O)c3c(C)n(C)n(-c4ccccc4)c3=O)nc3cccc3c2n1	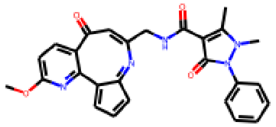	9.0	0.768132	3.608437	2.513170	5	0.823048
**2775**	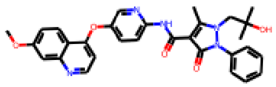	0.447899	COc1ccc2c(=O)cc(CNC(=O)c3c(C)n(C)n(-c4ccccc4)c3=O)nc3cccc3c2n1	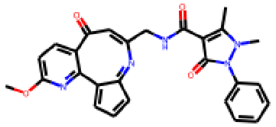	9.0	0.768132	3.608437	2.513170	5	0.823048

	src known mol	similarity	smiles	mol	Class	qed	logP	SAS	rings	kinase inhibition likeliness
**2776**	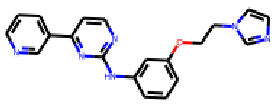	0.447863	Cc1ccc(OC(=O)c2ccccc2)cc1Nc1nccc(-c2cccnc2)n1	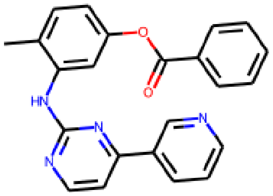	0.0	0.638163	3.995589	2.988471	4	0.775468
**2777**	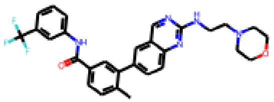	0.447804	Cc1ccc(C(=O)Nc2cccc(C(F)(C)F)c2C)cc1-c1ccc2nc(C)ccccc(F)nc2c1	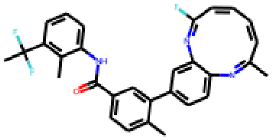	6.0	0.742375	4.384502	0.286234	4	0.779167
**2778**	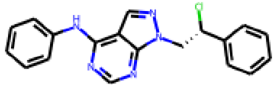	0.447761	Cc1ccc(C(=O)Nc2ccon2)cc1Nc1ncnc2c1cnn2C1cccccc1	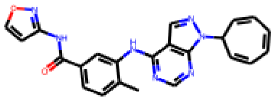	8.0	0.623362	2.525411	2.997881	5	0.770298
**2779**	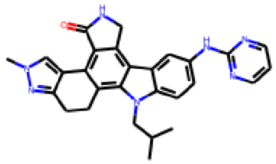	0.447752	C#Cc1ccc(C(=O)OC)cc1-c1C(=O)c-n2nc(-c3ccc(F)cc3F)cc2C1	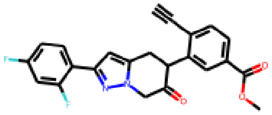	8.0	0.768132	3.608437	2.513170	4	0.763730

	src known mol	similarity	smiles	mol	Class	qed	logP	SAS	rings	kinase inhibition likeliness
**2780**	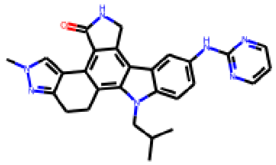	0.447752	C#Cc1ccc(C(=O)OC)cc1-c1C(=O)c-n2nc(-c3ccc(F)cc3F)cc2C1	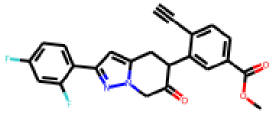	8.0	0.768132	3.608437	2.513170	4	0.763730
**2781**	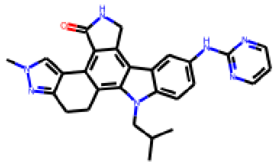	0.447752	C#Cc1ccc(C(=O)OC)cc1-c1C(=O)c-n2nc(-c3ccc(F)cc3F)cc2C1	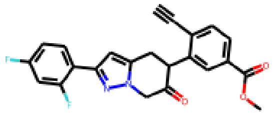	8.0	0.768132	3.608437	2.513170	4	0.763730
**2782**	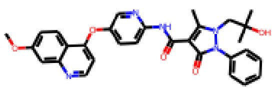	0.447581	COc1ccc2c(=O)cc(CNC(=O)c3c(C)n(C)n(-c4ccccc4)c3=O)nc3cccc3c2n1	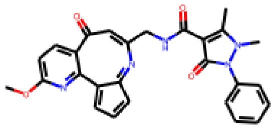	9.0	0.768132	3.608437	2.513170	5	0.823048
**2783**	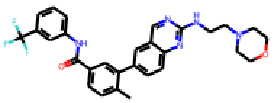	0.447435	Cc1ccc(C(=O)Nc2cccc(C(F)(F)F)c2)cc1-c1ccc2nc(N)ccc2c1	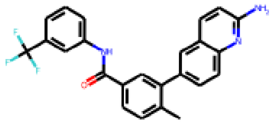	6.0	0.635315	4.542409	3.856266	4	0.833561

	src known mol	similarity	smiles	mol	Class	qed	logP	SAS	rings	kinase inhibition likeliness
**2784**	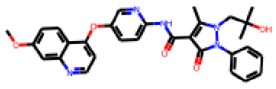	0.447426	COc1ccc2c(=O)cc(CNC(=O)c3c(C)n(C)n(-c4ccccc4)c3=O)nc3cccc2c3c1	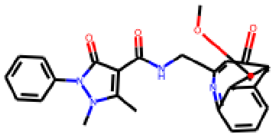	9.0	0.638163	3.995589	2.988471	5	0.774651
**2785**	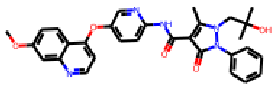	0.447426	COc1ccc2c(=O)cc(CNC(=O)c3c(C)n(C)n(-c4ccccc4)c3=O)nc3cccc2c3c1	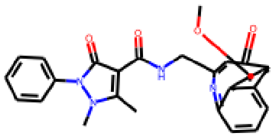	9.0	0.638163	3.995589	2.988471	5	0.774651
**2786**	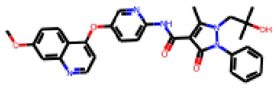	0.447368	COc1ccc2c(=O)cc(CNC(=O)c3c(C)n(C)n(-c4ccccc4)c3=O)nc3cccc3c2n1	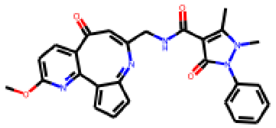	9.0	0.768132	3.608437	2.513170	5	0.823048
**2787**	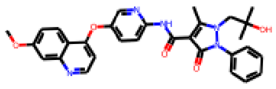	0.447353	COc1ccc2c(=O)cc(CNC(=O)c3c(C)n(C)n(-c4ccccc4)c3=O)nc3cccc3c2n1	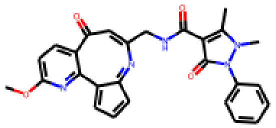	9.0	0.768132	3.608437	2.513170	5	0.823048

	src known mol	similarity	smiles	mol	Class	qed	logP	SAS	rings	kinase inhibition likeliness
**2788**	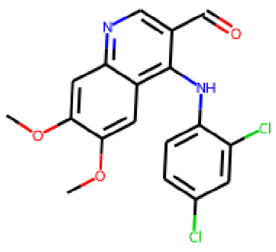	0.447248	COc1cc2ncc(C(=O)O)c(Nc3cccc(Cl)c3Cl)c2cc1OC1C(C)C1	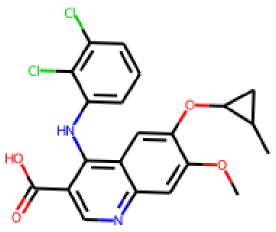	2.0	0.623362	2.525411	2.997881	4	0.774857
**2789**	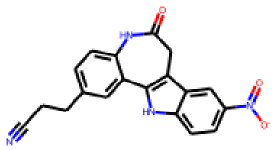	0.447005	CCNC(=O)N1CCC(Nc2n(C(C)c3c(-c4ccccc4)[nH]c4ccccc34)n2)CC1	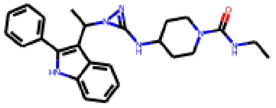	7.0	0.623584	3.603734	4.478009	5	0.804049
**2790**	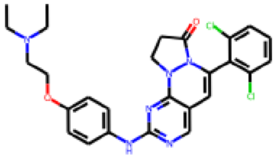	0.446935	COc1cc(Nc2ncc(C)c(-c3ccc4c(n3)NC(=O)C(C)(C)C4)n2)cc(OC)c1OC	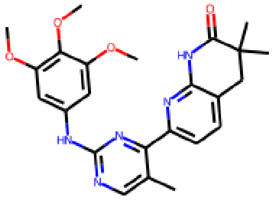	4.0	0.768132	3.608437	2.513170	4	0.757734
**2791**	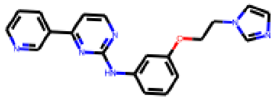	0.446691	Cc1ccc(OC(=O)c2ccccc2)cc1Nc1nccc(-c2cccnc2)n1	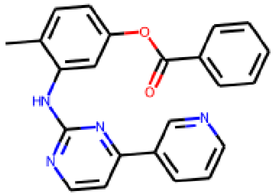	0.0	0.638163	3.995589	2.988471	4	0.775468

	src known mol	similarity	smiles	mol	Class	qed	logP	SAS	rings	kinase inhibition likeliness
**2792**	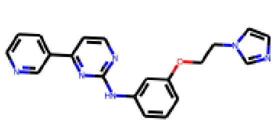	0.446621	Cc1ccc(OC(=O)c2ccccc2)cc1Nc1nccc(-c2cccnc2)n1	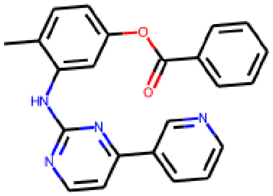	0.0	0.638163	3.995589	2.988471	4	0.775468
**2793**	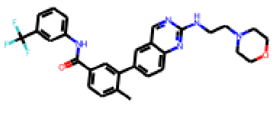	0.446508	Cc1ccc(C(=O)Nc2cccc(C(F)(C)F)c2C)cc1-c1ccc2nc(C)ccccc(F)nc2c1	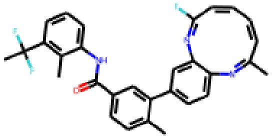	6.0	0.742375	4.384502	0.286234	4	0.779167
**2794**	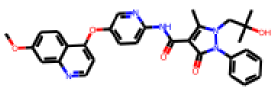	0.446458	COc1ccc2c(=O)cc(CNC(=O)c3c(C)n(C)n(-c4ccccc4)c3=O)nc3cccc2c3c1	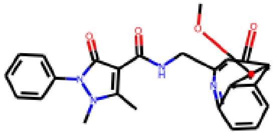	9.0	0.638163	3.995589	2.988471	5	0.774651
**2795**	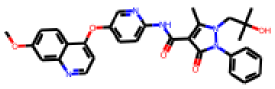	0.446458	COc1ccc2c(=O)cc(CNC(=O)c3c(C)n(C)n(-c4ccccc4)c3=O)nc3cccc2c3c1	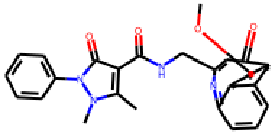	9.0	0.638163	3.995589	2.988471	5	0.774651

	src known mol	similarity	smiles	mol	Class	qed	logP	SAS	rings	kinase inhibition likeliness
**2796**	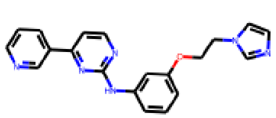	0.446295	Cc1ccc(OC(=O)c2ccccc2)cc1Nc1nccc(-c2cccnc2)n1	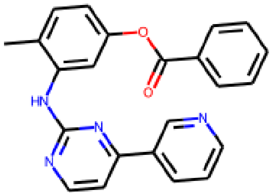	0.0	0.638163	3.995589	2.988471	4	0.775468
**2797**	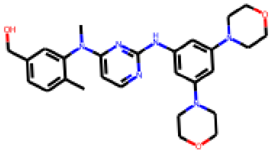	0.446218	CC(=O)c1ccccc1Nc1nc(Nc2ccc(N3CCOCC3)cc2F)ncc1Cl	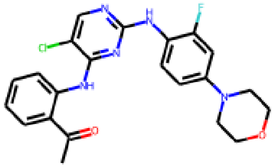	7.0	0.638163	3.995589	2.988471	4	0.772587
**2798**	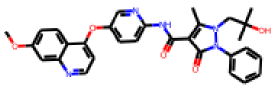	0.446121	COc1ccc2c(=O)cc(CNC(=O)c3c(C)n(C)n(-c4ccccc4)c3=O)nc3cccc2c3c1	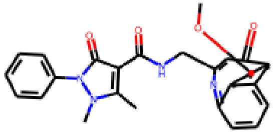	9.0	0.638163	3.995589	2.988471	5	0.774651
**2799**	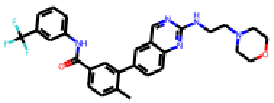	0.446075	Cc1ccc(C(=O)Nc2cccc(C(F)(C)F)c2C)cc1-c1ccc2nc(C)ccccc(F)nc2c1	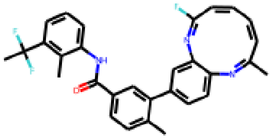	6.0	0.742375	4.384502	0.286234	4	0.779167

	src known mol	similarity	smiles	mol	Class	qed	logP	SAS	rings	kinase inhibition likeliness
**2800**	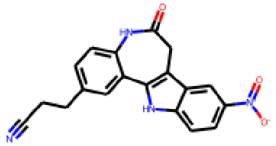	0.446053	CCNC(=O)N1CCC(Nc2n(C(C)c3c(-c4ccccc4)[nH]c4ccccc34)n2)CC1	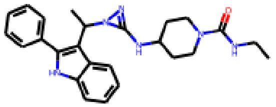	7.0	0.623584	3.603734	4.478009	5	0.804049
**2801**	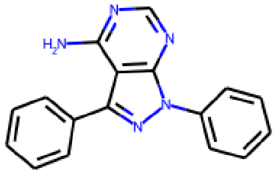	0.445974	Cc1ccc(C(=O)Oc2ccon2)cc1-c1ncnc2c1cnn2-c1ccccc1	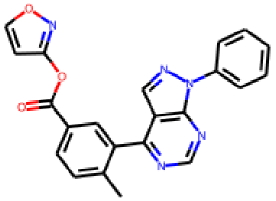	8.0	0.635315	4.542409	3.856266	5	0.760056
**2802**	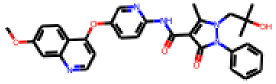	0.445961	COc1ccc2c(=O)cc(CNC(=O)c3c(C)n(C)n(-c4ccccc4)c3=O)nc3cccc3c2n1	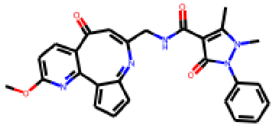	9.0	0.768132	3.608437	2.513170	5	0.823048
**2803**	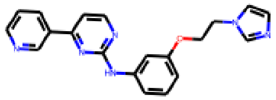	0.445813	Cc1ccc(OC(=O)c2ccccc2)cc1Nc1nccc(-c2cccnc2)n1	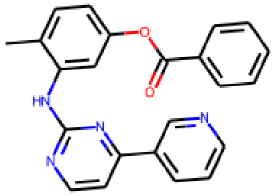	0.0	0.638163	3.995589	2.988471	4	0.775468

	src known mol	similarity	smiles	mol	Class	qed	logP	SAS	rings	kinase inhibition likeliness
**2804**	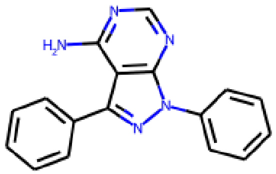	0.445498	Cc1ccc(C(=O)Oc2ccon2)cc1-c1ncnc2c1cnn2-c1ccccc1	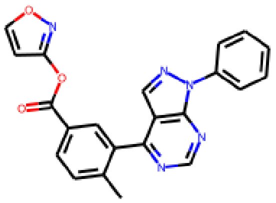	8.0	0.635315	4.542409	3.856266	5	0.760056
**2805**	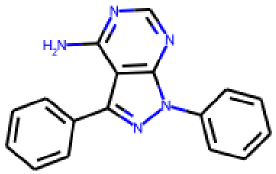	0.445498	Cc1ccc(C(=O)Oc2ccon2)cc1-c1ncnc2c1cnn2-c1ccccc1	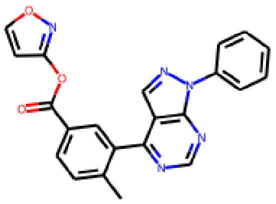	8.0	0.635315	4.542409	3.856266	5	0.760056
**2806**	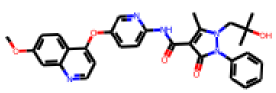	0.445407	COc1ccc2c(=O)cc(CNC(=O)c3c(C)n(C)n(-c4ccccc4)c3=O)nc3cccc3c2n1	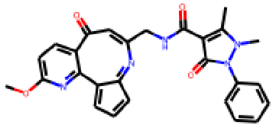	9.0	0.768132	3.608437	2.513170	5	0.823048
**2807**	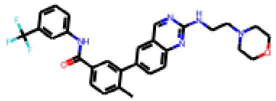	0.445261	Cc1ccc(C(=O)Nc2cccc(C(F)(C)F)c2C)cc1-c1ccc2nc(C)ccccc(F)nc2c1	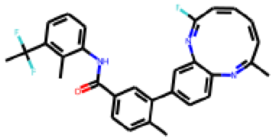	6.0	0.742375	4.384502	0.286234	4	0.779167

	src known mol	similarity	smiles	mol	Class	qed	logP	SAS	rings	kinase inhibition likeliness
**2808**	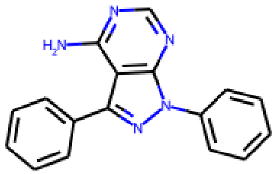	0.445051	Cc1ccc(C(=O)Oc2ccon2)cc1-c1ncnc2c1cnn2-c1ccccc1	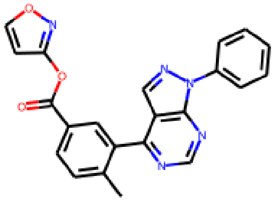	8.0	0.635315	4.542409	3.856266	5	0.760056
**2809**	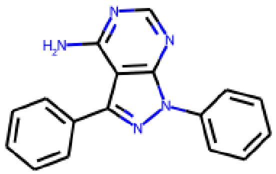	0.445051	Cc1ccc(C(=O)Oc2ccon2)cc1-c1ncnc2c1cnn2-c1ccccc1	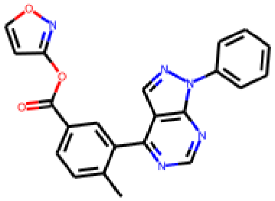	8.0	0.635315	4.542409	3.856266	5	0.760056
**2810**	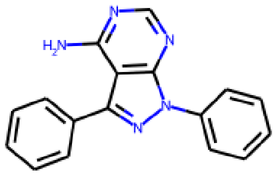	0.445051	Cc1ccc(C(=O)Oc2ccon2)cc1-c1ncnc2c1cnn2-c1ccccc1	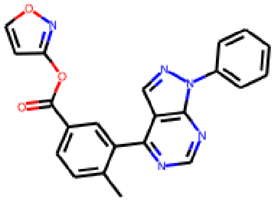	8.0	0.635315	4.542409	3.856266	5	0.760056
**2811**	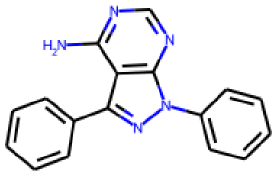	0.445051	Cc1ccc(C(=O)Oc2ccon2)cc1-c1ncnc2c1cnn2-c1ccccc1	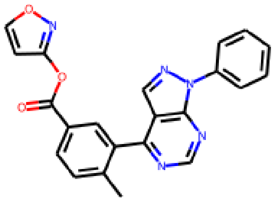	8.0	0.635315	4.542409	3.856266	5	0.760056

	src known mol	similarity	smiles	mol	Class	qed	logP	SAS	rings	kinase inhibition likeliness
**2812**	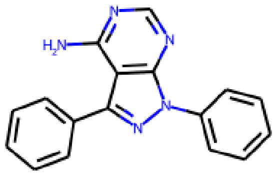	0.445051	Cc1ccc(C(=O)Oc2ccon2)cc1-c1ncnc2c1cnn2-c1ccccc1	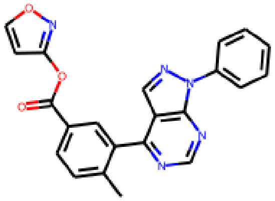	8.0	0.635315	4.542409	3.856266	5	0.760056
**2813**	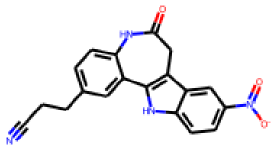	0.444909	CCNC(=O)N1CCC(Nc2n(C(C)c3c(-c4ccccc4)[nH]c4ccccc34)n2)CC1	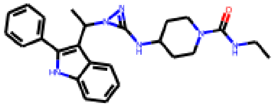	7.0	0.623584	3.603734	4.478009	5	0.804049
**2814**	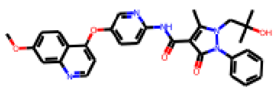	0.444628	COc1ccc2c(=O)cc(CNC(=O)c3c(C)n(C)n(-c4ccccc4)c3=O)nc3cccc2c3c1	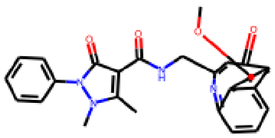	9.0	0.638163	3.995589	2.988471	5	0.774651
**2815**	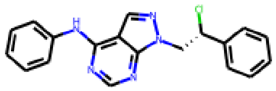	0.444444	Cc1ccc(C(=O)Nc2ccon2)cc1Nc1ncnc2c1cnn2C1cccccc1	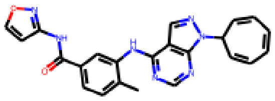	8.0	0.623362	2.525411	2.997881	5	0.770298

	src known mol	similarity	smiles	mol	Class	qed	logP	SAS	rings	kinase inhibition likeliness
**2816**	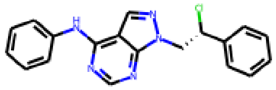	0.444444	Cc1ccc(C(=O)Nc2ccon2)cc1Nc1ncnc2c1cnn2C1cccccc1	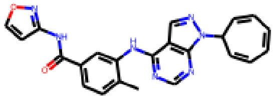	8.0	0.623362	2.525411	2.997881	5	0.770298
**2817**	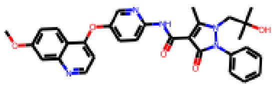	0.444317	COc1ccc2c(=O)cc(CNC(=O)c3c(C)n(C)n(-c4ccccc4)c3=O)nc3cccc3c2n1	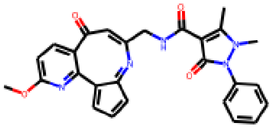	9.0	0.768132	3.608437	2.513170	5	0.823048
**2818**	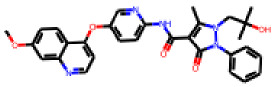	0.444317	COc1ccc2c(=O)cc(CNC(=O)c3c(C)n(C)n(-c4ccccc4)c3=O)nc3cccc3c2n1	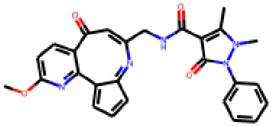	9.0	0.768132	3.608437	2.513170	5	0.823048
**2819**	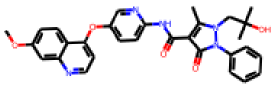	0.444317	COc1ccc2c(=O)cc(CNC(=O)c3c(C)n(C)n(-c4ccccc4)c3=O)nc3cccc3c2n1	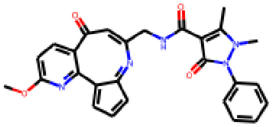	9.0	0.768132	3.608437	2.513170	5	0.823048

	src known mol	similarity	smiles	mol	Class	qed	logP	SAS	rings	kinase inhibition likeliness
2820	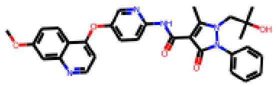	0.444316	COc1ccc2c(=O)cc(CNC(=O)c3c(C)n(C)n(-c4ccccc4)c3=O)nc3cccc3c2n1	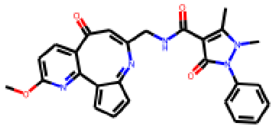	9.0	0.768132	3.608437	2.513170	5	0.823048
2821	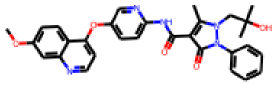	0.443958	COc1ccc2c(=O)cc(CNC(=O)c3c(C)n(C)n(-c4ccccc4)c3=O)nc3cccc2c3c1	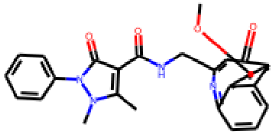	9.0	0.638163	3.995589	2.988471	5	0.774651
2822	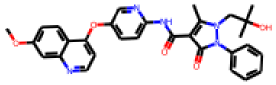	0.443958	COc1ccc2c(=O)cc(CNC(=O)c3c(C)n(C)n(-c4ccccc4)c3=O)nc3cccc2c3c1	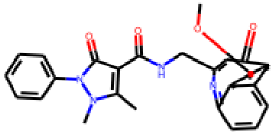	9.0	0.638163	3.995589	2.988471	5	0.774651
2823	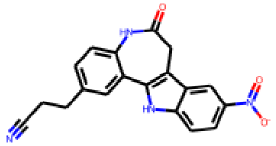	0.443934	CCNC(=O)N1CCC(Nc2n(C(C)c3c(-c4ccccc4)[nH]c4ccccc34)n2)CC1	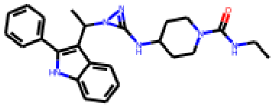	7.0	0.623584	3.603734	4.478009	5	0.804049

	src known mol	similarity	smiles	mol	Class	qed	logP	SAS	rings	kinase inhibition likeliness
**2824**	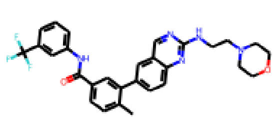	0.443815	Cc1ccc(NC(=O)c2cccc(C(F)(F)F)c2)cc1-c1ccc2nc(O)ncc2c1	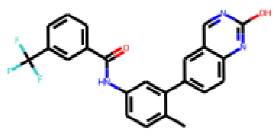	6.0	0.638163	3.995589	2.988471	4	0.818430
**2825**	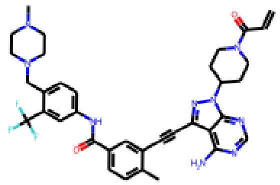	0.443580	Cc1ccc(C(=O)Nc2cccc(C(F)(C)F)c2C)cc1-c1ccc2nc(=O)ccc(C)cn2n1	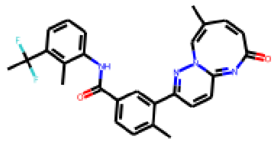	6.0	0.762994	3.476405	2.421118	4	0.764424
**2826**	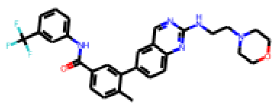	0.443548	Cc1ccc(C(=O)Nc2cccc(C(F)(C)F)c2C)cc1-c1ccc2nc(C)ccccc(F)nc2c1	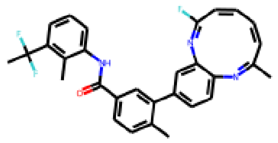	6.0	0.742375	4.384502	0.286234	4	0.779167
**2827**	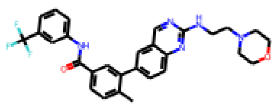	0.443548	Cc1ccc(C(=O)Nc2cccc(C(F)(C)F)c2C)cc1-c1ccc2nc(C)ccccc(F)nc2c1	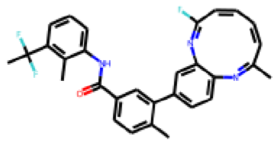	6.0	0.742375	4.384502	0.286234	4	0.779167

	src known mol	similarity	smiles	mol	Class	qed	logP	SAS	rings	kinase inhibition likeliness
**2828**	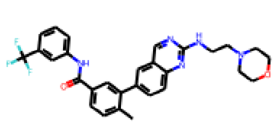	0.443548	Cc1ccc(C(=O)Nc2cccc(C(F)(C)F)c2C)cc1-c1ccc2nc(C)ccccc(F)nc2c1	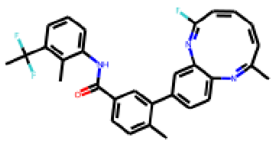	6.0	0.742375	4.384502	0.286234	4	0.779167
**2829**	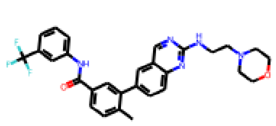	0.443548	Cc1ccc(C(=O)Nc2cccc(C(F)(C)F)c2C)cc1-c1ccc2nc(C)ccccc(F)nc2c1	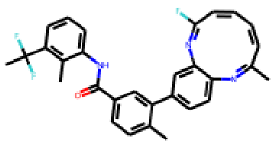	6.0	0.742375	4.384502	0.286234	4	0.779167
**2830**	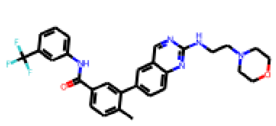	0.443548	Cc1ccc(C(=O)Nc2cccc(C(F)(C)F)c2C)cc1-c1ccc2nc(C)ccccc(F)nc2c1	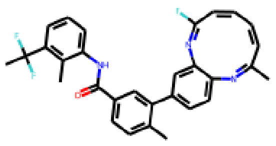	6.0	0.742375	4.384502	0.286234	4	0.779167
**2831**	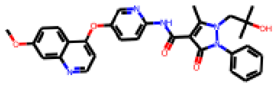	0.443423	COc1ccc2c(=O)cc(CNC(=O)c3c(C)n(C)n(-c4ccccc4)c3=O)nc3cccc3c2n1	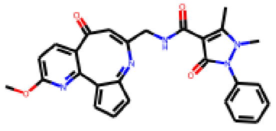	9.0	0.768132	3.608437	2.513170	5	0.823048

	src known mol	similarity	smiles	mol	Class	qed	logP	SAS	rings	kinase inhibition likeliness
**2832**	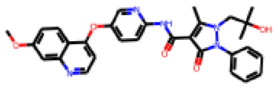	0.442849	COc1ccc2c(=O)cc(CNC(=O)c3c(C)n(C)n(-c4ccccc4)c3=O)nc3cccc3c2n1	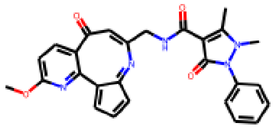	9.0	0.768132	3.608437	2.513170	5	0.823048
**2833**	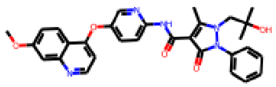	0.442849	COc1ccc2c(=O)cc(CNC(=O)c3c(C)n(C)n(-c4ccccc4)c3=O)nc3cccc3c2n1	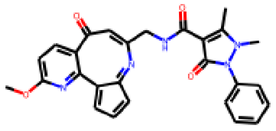	9.0	0.768132	3.608437	2.513170	5	0.823048
**2834**	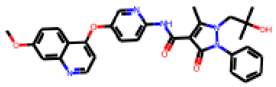	0.442849	COc1ccc2c(=O)cc(CNC(=O)c3c(C)n(C)n(-c4ccccc4)c3=O)nc3cccc3c2n1	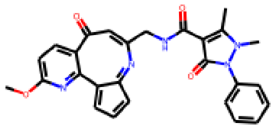	9.0	0.768132	3.608437	2.513170	5	0.823048
**2835**	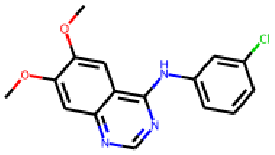	0.442714	Clc1cccc(Nc2ncnc3cc4c[nH]nc4cc23)c1	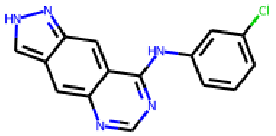	3.0	0.638163	3.995589	2.988471	4	0.781333

	src known mol	similarity	smiles	mol	Class	qed	logP	SAS	rings	kinase inhibition likeliness
**2836**	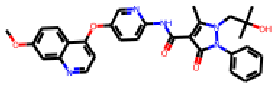	0.442519	COc1ccc2c(=O)cc(CNC(=O)c3c(C)n(C)n(-c4ccccc4)c3=O)nc3cccc3c2n1	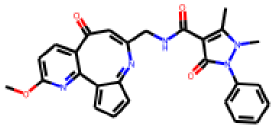	9.0	0.768132	3.608437	2.513170	5	0.823048
**2837**	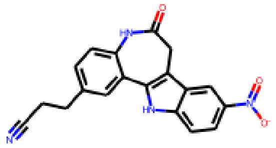	0.442181	CCNC(=O)N1CCC(Nc2n(C(C)c3c(-c4ccccc4)[nH]c4ccccc34)n2)CC1	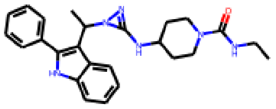	7.0	0.623584	3.603734	4.478009	5	0.804049
**2838**	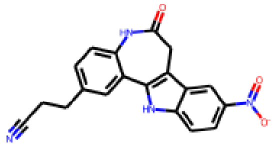	0.442181	CCNC(=O)N1CCC(Nc2n(C(C)c3c(-c4ccccc4)[nH]c4ccccc34)n2)CC1	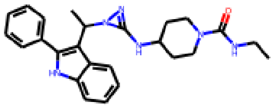	7.0	0.623584	3.603734	4.478009	5	0.804049
**2839**	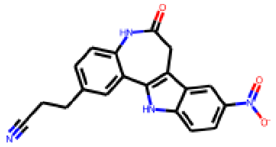	0.442181	CCNC(=O)N1CCC(Nc2n(C(C)c3c(-c4ccccc4)[nH]c4ccccc34)n2)CC1	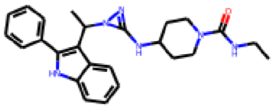	7.0	0.623584	3.603734	4.478009	5	0.804049

	src known mol	similarity	smiles	mol	Class	qed	logP	SAS	rings	kinase inhibition likeliness
**2840**	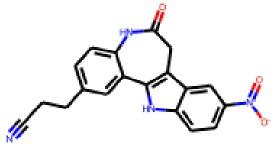	0.442181	CCNC(=O)N1CCC(Nc2n(C(C)c3c(-c4ccccc4)[nH]c4ccccc34)n2)CC1	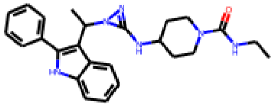	7.0	0.623584	3.603734	4.478009	5	0.804049
**2841**	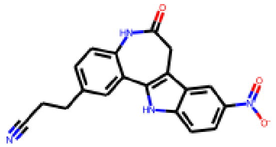	0.442181	CCNC(=O)N1CCC(Nc2n(C(C)c3c(-c4ccccc4)[nH]c4ccccc34)n2)CC1	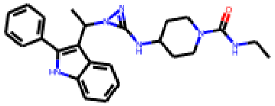	7.0	0.623584	3.603734	4.478009	5	0.804049
**2842**	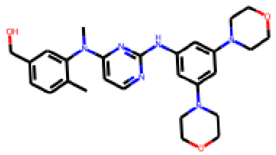	0.442142	NC(=O)c1ccccc1Nc1nc(Nc2ccc(N3CCOCC3)cc2C)ncc1Cl	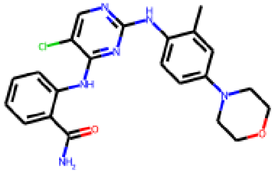	7.0	0.623362	2.525411	2.997881	4	0.783217
**2843**	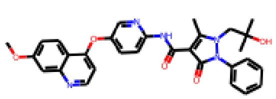	0.442099	COc1ccc2c(=O)cc(CNC(=O)c3c(C)n(C)n(-c4ccccc4)c3=O)nc3cccc3c2n1	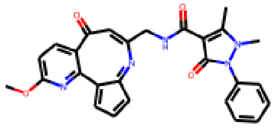	9.0	0.768132	3.608437	2.513170	5	0.823048

	src known mol	similarity	smiles	mol	Class	qed	logP	SAS	rings	kinase inhibition likeliness
**2844**	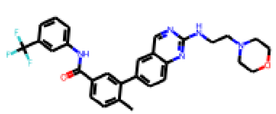	0.441895	Cc1ccc(C(=O)Nc2cccc(C(F)(C)F)c2C)cc1-c1ccc2nc(C)ccccc(F)nc2c1	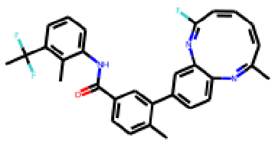	6.0	0.742375	4.384502	0.286234	4	0.779167
**2845**	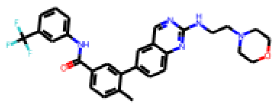	0.441895	Cc1ccc(C(=O)Nc2cccc(C(F)(C)F)c2C)cc1-c1ccc2nc(C)ccccc(F)nc2c1	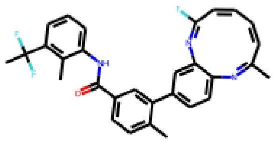	6.0	0.742375	4.384502	0.286234	4	0.779167
**2846**	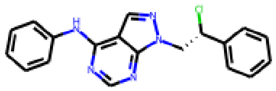	0.441603	Cc1ccc(C(=O)Nc2ccon2)cc1Nc1ncnc2c1cnn2C1cccccc1	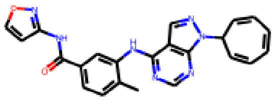	8.0	0.623362	2.525411	2.997881	5	0.770298
**2847**	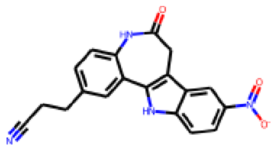	0.441524	CCNC(=O)N1CCC(Nc2n(C(C)c3c(-c4ccccc4)[nH]c4ccccc34)n2)CC1	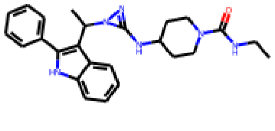	7.0	0.623584	3.603734	4.478009	5	0.804049

	src known mol	similarity	smiles	mol	Class	qed	logP	SAS	rings	kinase inhibition likeliness
**2848**	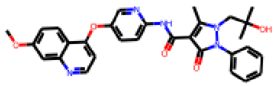	0.441431	COc1ccc2c(=O)cc(CNC(=O)c3c(C)n(C)n(-c4ccccc4)c3=O)nc3cccc3c2n1	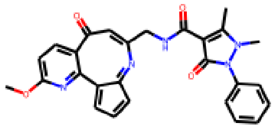	9.0	0.768132	3.608437	2.513170	5	0.823048
**2849**	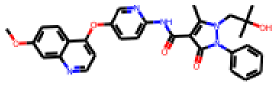	0.441431	COc1ccc2c(=O)cc(CNC(=O)c3c(C)n(C)n(-c4ccccc4)c3=O)nc3cccc3c2n1	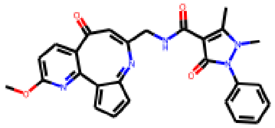	9.0	0.768132	3.608437	2.513170	5	0.823048
**2850**	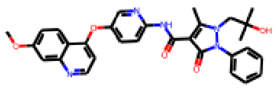	0.441210	COc1ccc2c(=O)cc(CNC(=O)c3c(C)n(C)n(-c4ccccc4)c3=O)nc3cccc3c2n1	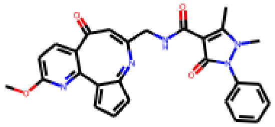	9.0	0.768132	3.608437	2.513170	5	0.823048
**2851**	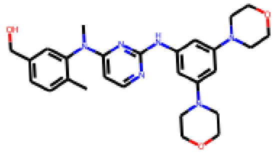	0.441200	CC(=O)c1ccccc1Nc1nc(Nc2ccc(N3CCOCC3)cc2F)ncc1Cl	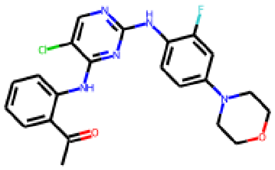	7.0	0.638163	3.995589	2.988471	4	0.772587

	src known mol	similarity	smiles	mol	Class	qed	logP	SAS	rings	kinase inhibition likeliness
**2852**	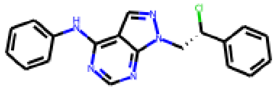	0.441042	Cc1ccc(C(=O)Nc2ccon2)cc1Nc1ncnc2c1cnn2C1cccccc1	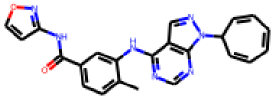	8.0	0.623362	2.525411	2.997881	5	0.770298
**2853**	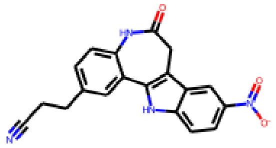	0.440846	CCNC(=O)N1CCC(Nc2n(C(C)c3c(-c4ccccc4)[nH]c4ccccc34)n2)CC1	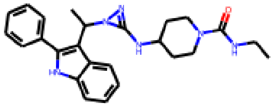	7.0	0.623584	3.603734	4.478009	5	0.804049
**2854**	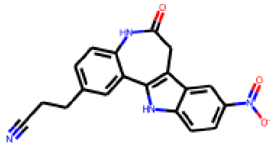	0.440846	CCNC(=O)N1CCC(Nc2n(C(C)c3c(-c4ccccc4)[nH]c4ccccc34)n2)CC1	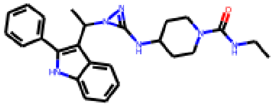	7.0	0.623584	3.603734	4.478009	5	0.804049
**2855**	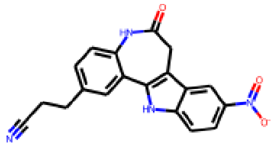	0.440846	CCNC(=O)N1CCC(Nc2n(C(C)c3c(-c4ccccc4)[nH]c4ccccc34)n2)CC1	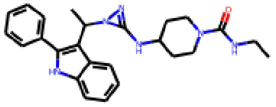	7.0	0.623584	3.603734	4.478009	5	0.804049

	src known mol	similarity	smiles	mol	Class	qed	logP	SAS	rings	kinase inhibition likeliness
**2856**	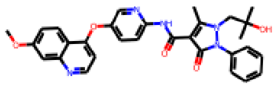	0.440805	COc1ccc2c(=O)cc(CNC(=O)c3c(C)n(C)n(-c4ccccc4)c3=O)nc3cccc3c2n1	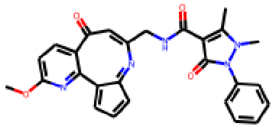	9.0	0.768132	3.608437	2.513170	5	0.823048
**2857**	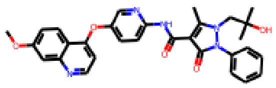	0.440717	COc1ccc2c(=O)cc(CNC(=O)c3c(C)n(C)n(-c4ccccc4)c3=O)nc3cccc3c2n1	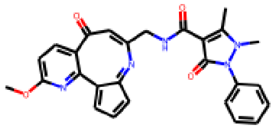	9.0	0.768132	3.608437	2.513170	5	0.823048
**2858**	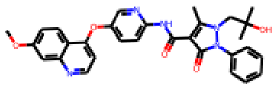	0.440717	COc1ccc2c(=O)cc(CNC(=O)c3c(C)n(C)n(-c4ccccc4)c3=O)nc3cccc3c2n1	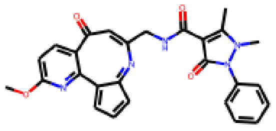	9.0	0.768132	3.608437	2.513170	5	0.823048
**2859**	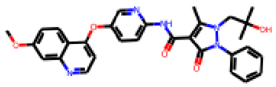	0.440717	COc1ccc2c(=O)cc(CNC(=O)c3c(C)n(C)n(-c4ccccc4)c3=O)nc3cccc3c2n1	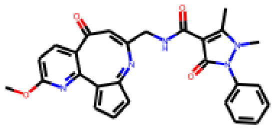	9.0	0.768132	3.608437	2.513170	5	0.823048

	src known mol	similarity	smiles	mol	Class	qed	logP	SAS	rings	kinase inhibition likeliness
**2860**	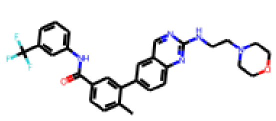	0.440641	Cc1ccc(C(=O)Nc2cccc(C(F)(C)F)c2C)cc1-c1ccc2nc(C)ccccc(F)nc2c1	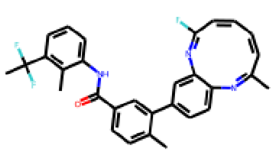	6.0	0.742375	4.384502	0.286234	4	0.779167
**2861**	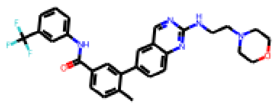	0.440641	Cc1ccc(C(=O)Nc2cccc(C(F)(C)F)c2C)cc1-c1ccc2nc(C)ccccc(F)nc2c1	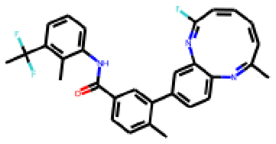	6.0	0.742375	4.384502	0.286234	4	0.779167
**2862**	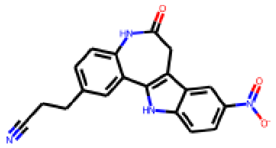	0.440436	CCNC(=O)N1CCC(Nc2n(C(C)c3c(-c4ccccc4)[nH]c4ccccc34)n2)CC1	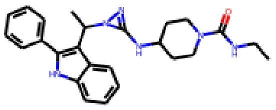	7.0	0.623584	3.603734	4.478009	5	0.804049
**2863**	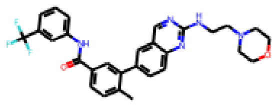	0.440407	Cc1ccc(C(=O)Nc2cccc(C(F)(C)F)c2C)cc1-c1ccc2nc(C)ccccc(F)nc2c1	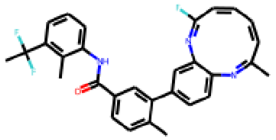	6.0	0.742375	4.384502	0.286234	4	0.779167

	src known mol	similarity	smiles	mol	Class	qed	logP	SAS	rings	kinase inhibition likeliness
**2864**	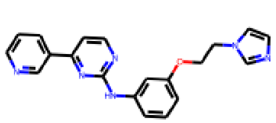	0.440349	Cc1ccc(OC(=O)c2ccccc2)cc1Nc1nccc(-c2cccnc2)n1	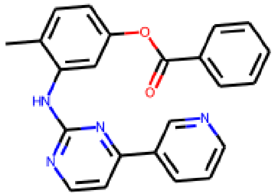	0.0	0.638163	3.995589	2.988471	4	0.775468
**2865**	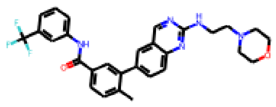	0.440204	Cc1ccc(C(=O)Oc2cccc(C(F)(F)F)c2)cc1-c1ccc2nc(N)ncc2c1	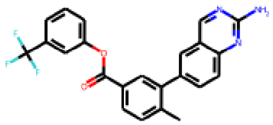	6.0	0.638163	3.995589	2.988471	4	0.789898
**2866**	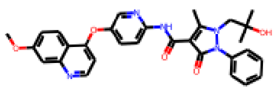	0.440197	COc1ccc2c(=O)cc(CNC(=O)c3c(C)n(C)n(-c4ccccc4)c3=O)nc3cccc2c3c1	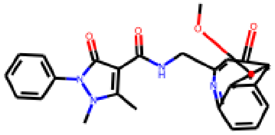	9.0	0.638163	3.995589	2.988471	5	0.774651
**2867**	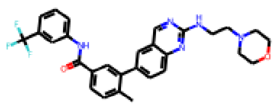	0.439852	Cc1ccc(C(=O)Nc2cccc(C(F)(C)F)c2C)cc1-c1ccc2nc(C)ccccc(F)nc2c1	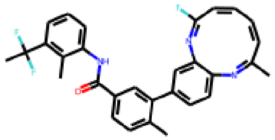	6.0	0.742375	4.384502	0.286234	4	0.779167

	src known mol	similarity	smiles	mol	Class	qed	logP	SAS	rings	kinase inhibition likeliness
**2868**	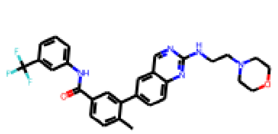	0.439722	Cc1ccc(C(=O)Oc2cccc(C(F)(F)F)c2)cc1-c1ccc2nc(N)ncc2c1	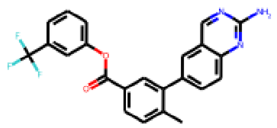	6.0	0.638163	3.995589	2.988471	4	0.789898
**2869**	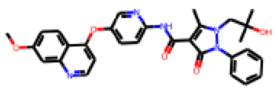	0.439669	COc1ccc2c(=O)cc(CNC(=O)c3c(C)n(C)n(-c4ccccc4)c3=O)nc3cccc2c3c1	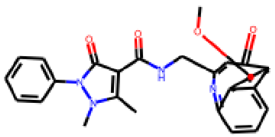	9.0	0.638163	3.995589	2.988471	5	0.774651
**2870**	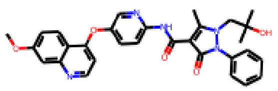	0.439159	COc1ccc2c(=O)cc(CNC(=O)c3c(C)n(C)n(-c4ccccc4)c3=O)nc3cccc2c3c1	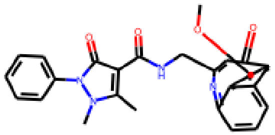	9.0	0.638163	3.995589	2.988471	5	0.774651
**2871**	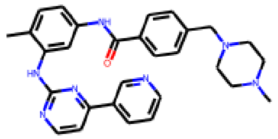	0.439065	Cc1ccc(NC(=O)c2ccccc2)cc1Nc1nccc(-c2cccnc2)n1	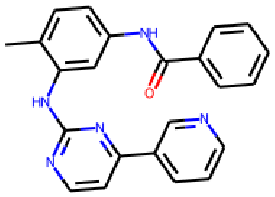	0.0	0.768132	3.608437	2.513170	4	0.854555

	src known mol	similarity	smiles	mol	Class	qed	logP	SAS	rings	kinase inhibition likeliness
**2872**	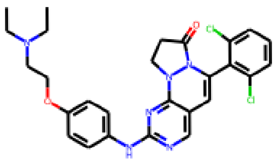	0.438679	COc1cc(Nc2ncc(C)c(-c3ccc4c(n3)NC(=O)C(C)(C)C4)n2)cc(OC)c1OC	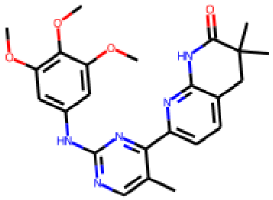	4.0	0.768132	3.608437	2.513170	4	0.757734
**2873**	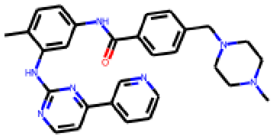	0.438671	Cc1ccc(NC(=O)c2ccccc2)cc1Nc1nccc(-c2cccnc2)n1	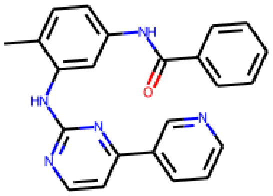	0.0	0.768132	3.608437	2.513170	4	0.854555
**2874**	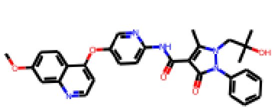	0.438596	COc1ccc2c(=O)cc(CNC(=O)c3c(C)n(C)n(-c4ccccc4)c3=O)nc3cccc2c3c1	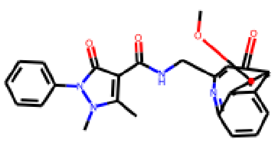	9.0	0.638163	3.995589	2.988471	5	0.774651
**2875**	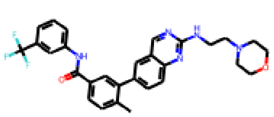	0.437988	Cc1ccc(C(=O)Oc2cccc(C(F)(F)F)c2)cc1-c1ccc2nc(N)ncc2c1	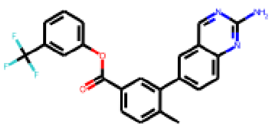	6.0	0.638163	3.995589	2.988471	4	0.789898

	src known mol	similarity	smiles	mol	Class	qed	logP	SAS	rings	kinase inhibition likeliness
**2876**	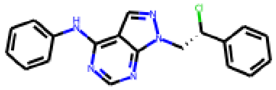	0.437807	Cc1ccc(C(=O)Nc2ccon2)cc1Nc1ncnc2c1cnn2C1cccccc1	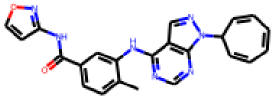	8.0	0.623362	2.525411	2.997881	5	0.770298
**2877**	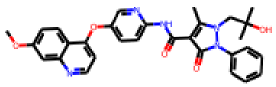	0.437716	COc1ccc2c(=O)cc(CNC(=O)c3c(C)n(C)n(-c4ccccc4)c3=O)nc3cccc3c2n1	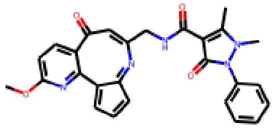	9.0	0.768132	3.608437	2.513170	5	0.823048
**2878**	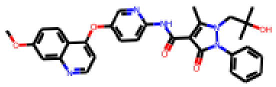	0.437716	COc1ccc2c(=O)cc(CNC(=O)c3c(C)n(C)n(-c4ccccc4)c3=O)nc3cccc3c2n1	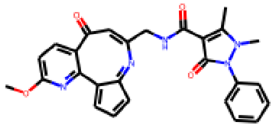	9.0	0.768132	3.608437	2.513170	5	0.823048
**2879**	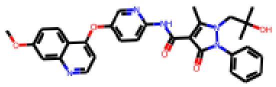	0.437716	COc1ccc2c(=O)cc(CNC(=O)c3c(C)n(C)n(-c4ccccc4)c3=O)nc3cccc3c2n1	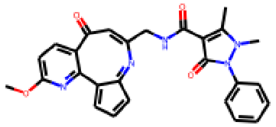	9.0	0.768132	3.608437	2.513170	5	0.823048

	src known mol	similarity	smiles	mol	Class	qed	logP	SAS	rings	kinase inhibition likeliness
**2880**	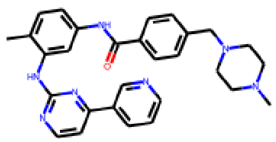	0.437500	Cc1ccc(NC(=O)c2ccccc2)cc1Nc1nccc(-c2ccncc2)n1	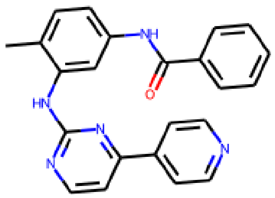	0.0	0.593231	3.250153	2.680275	4	0.858012
**2881**	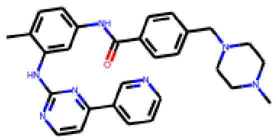	0.437131	Cc1ccc(NC(=O)c2ccccc2)cc1Nc1nccc(-c2cccnc2)n1	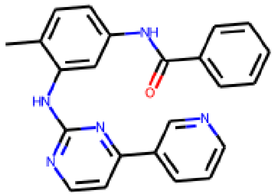	0.0	0.768132	3.608437	2.513170	4	0.854555
**2882**	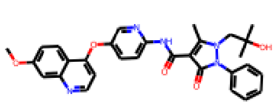	0.436960	COc1ccc2c(=O)cc(CNC(=O)c3c(C)n(C)n(-c4ccccc4)c3=O)nc3cccc3c2n1	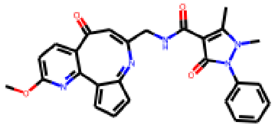	9.0	0.768132	3.608437	2.513170	5	0.823048
**2883**	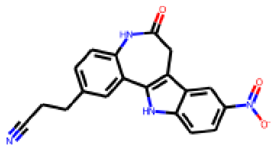	0.436828	CCNC(=O)N1CCC(Nc2n(C(C)c3c(-c4ccccc4)[nH]c4ccccc34)n2)CC1	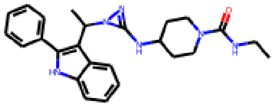	7.0	0.623584	3.603734	4.478009	5	0.804049

	src known mol	similarity	smiles	mol	Class	qed	logP	SAS	rings	kinase inhibition likeliness
**2884**	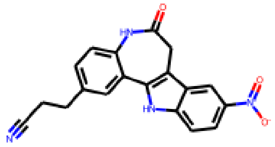	0.436828	CCNC(=O)N1CCC(Nc2n(C(C)c3c(-c4ccccc4)[nH]c4ccccc34)n2)CC1	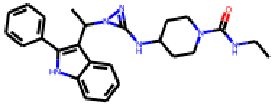	7.0	0.623584	3.603734	4.478009	5	0.804049
**2885**	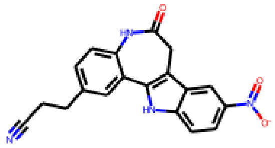	0.436828	CCNC(=O)N1CCC(Nc2n(C(C)c3c(-c4ccccc4)[nH]c4ccccc34)n2)CC1	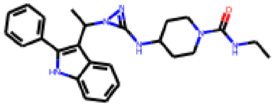	7.0	0.623584	3.603734	4.478009	5	0.804049
**2886**	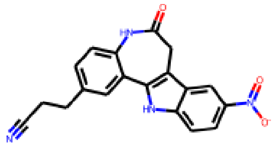	0.436828	CCNC(=O)N1CCC(Nc2n(C(C)c3c(-c4ccccc4)[nH]c4ccccc34)n2)CC1	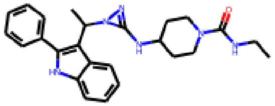	7.0	0.623584	3.603734	4.478009	5	0.804049
**2887**	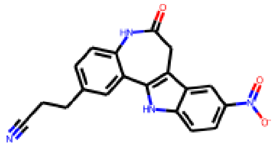	0.436828	CCNC(=O)N1CCC(Nc2n(C(C)c3c(-c4ccccc4)[nH]c4ccccc34)n2)CC1	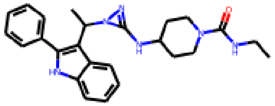	7.0	0.623584	3.603734	4.478009	5	0.804049

	src known mol	similarity	smiles	mol	Class	qed	logP	SAS	rings	kinase inhibition likeliness
**2888**	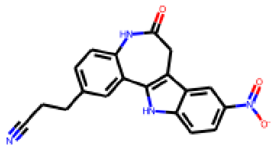	0.436828	CCNC(=O)N1CCC(Nc2n(C(C)c3c(-c4ccccc4)[nH]c4ccccc34)n2)CC1	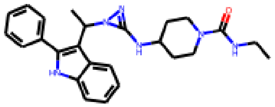	7.0	0.623584	3.603734	4.478009	5	0.804049
**2889**	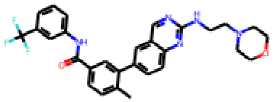	0.436801	Cc1ccc(C(=O)Oc2cccc(C(F)(F)F)c2)cc1-c1ccc2nc(N)ncc2c1	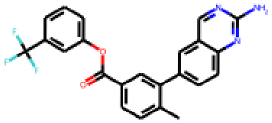	6.0	0.638163	3.995589	2.988471	4	0.789898
**2890**	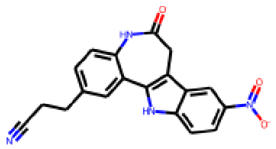	0.436582	CCNC(=O)N1CCC(Nc2n(C(C)c3c(-c4ccccc4)[nH]c4ccccc34)n2)CC1	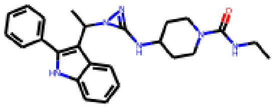	7.0	0.623584	3.603734	4.478009	5	0.804049
**2891**	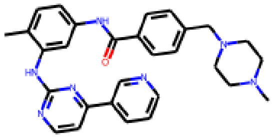	0.436455	Cc1ccc(NC(=O)c2ccccc2)cc1Nc1nccc(-c2cccnc2)n1	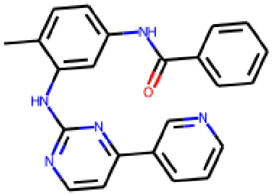	0.0	0.768132	3.608437	2.513170	4	0.854555

	src known mol	similarity	smiles	mol	Class	qed	logP	SAS	rings	kinase inhibition likeliness
**2892**	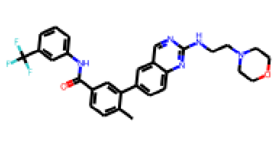	0.436426	Cc1ccc(C(=O)Oc2cccc(C(F)(F)F)c2)cc1-c1ccc2nc(N)ncc2c1	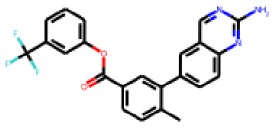	6.0	0.638163	3.995589	2.988471	4	0.789898
**2893**	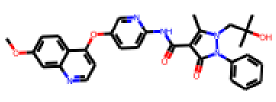	0.436289	COc1ccc2c(=O)cc(CNC(=O)c3c(C)n(C)n(-c4ccccc4)c3=O)nc3cccc3c2n1	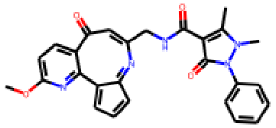	9.0	0.768132	3.608437	2.513170	5	0.823048
**2894**	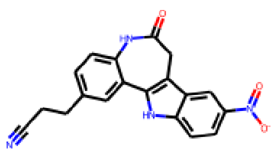	0.436117	CCNC(=O)N1CCC(Nc2n(C(C)c3c(-c4ccccc4)[nH]c4ccccc34)n2)CC1	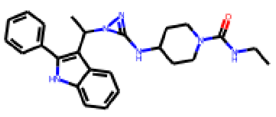	7.0	0.623584	3.603734	4.478009	5	0.804049
**2895**	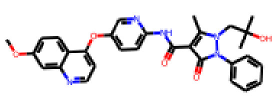	0.435986	COc1ccc2c(=O)cc(CNC(=O)c3c(C)n(C)n(-c4ccccc4)c3=O)nc3cccc3c2n1	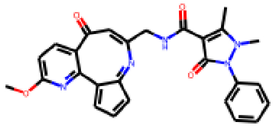	9.0	0.768132	3.608437	2.513170	5	0.823048

	src known mol	similarity	smiles	mol	Class	qed	logP	SAS	rings	kinase inhibition likeliness
**2896**	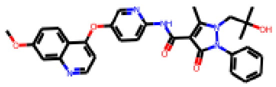	0.435986	COc1ccc2c(=O)cc(CNC(=O)c3c(C)n(C)n(-c4ccccc4)c3=O)nc3cccc3c2n1	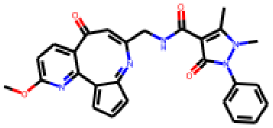	9.0	0.768132	3.608437	2.513170	5	0.823048
**2897**	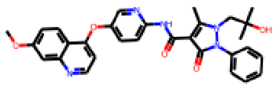	0.435986	COc1ccc2c(=O)cc(CNC(=O)c3c(C)n(C)n(-c4ccccc4)c3=O)nc3cccc3c2n1	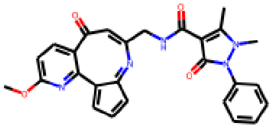	9.0	0.768132	3.608437	2.513170	5	0.823048
**2898**	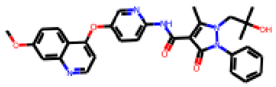	0.435986	COc1ccc2c(=O)cc(CNC(=O)c3c(C)n(C)n(-c4ccccc4)c3=O)nc3cccc3c2n1	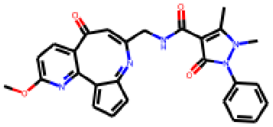	9.0	0.768132	3.608437	2.513170	5	0.823048
**2899**	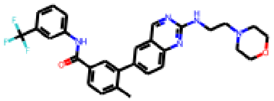	0.435957	Cc1ccc(C(=O)Nc2cccc(C(F)(C)F)c2C)cc1-c1ccc2nc(C)ccccc(F)nc2c1	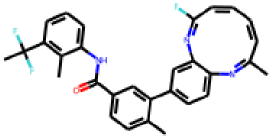	6.0	0.742375	4.384502	0.286234	4	0.779167

	src known mol	similarity	smiles	mol	Class	qed	logP	SAS	rings	kinase inhibition likeliness
**2900**	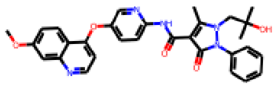	0.435883	COc1ccc2c(=O)cc(CNC(=O)c3c(C)n(C)n(-c4ccccc4)c3=O)nc3cccc2c3c1	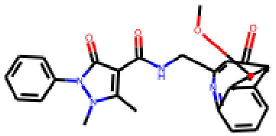	9.0	0.638163	3.995589	2.988471	5	0.774651
**2901**	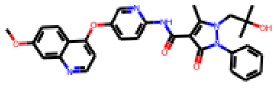	0.435868	COc1ccc2c(=O)cc(CNC(=O)c3c(C)n(C)n(-c4ccccc4)c3=O)nc3cccc3c2n1	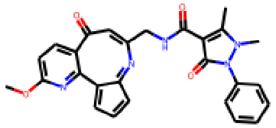	9.0	0.768132	3.608437	2.513170	5	0.823048
**2902**	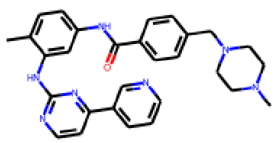	0.435678	Cc1ccc(NC(=O)c2ccccc2)cc1Nc1nccc(-c2cccnc2)n1	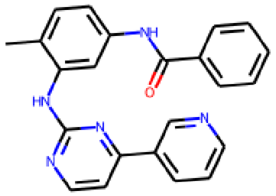	0.0	0.768132	3.608437	2.513170	4	0.854555
**2903**	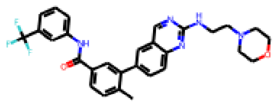	0.435414	Cc1ccc(C(=O)Nc2cccc(C(F)(C)F)c2C)cc1-c1ccc2nc(C)ccccc(F)nc2c1	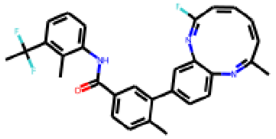	6.0	0.742375	4.384502	0.286234	4	0.779167

	src known mol	similarity	smiles	mol	Class	qed	logP	SAS	rings	kinase inhibition likeliness
**2904**	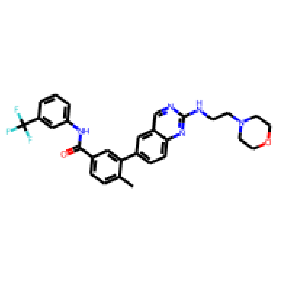	0.435252	Cc1ccc(NC(=O)c2cccc(C(F)(F)F)c2)cc1-c1ccc2nc(O)ncc2c1	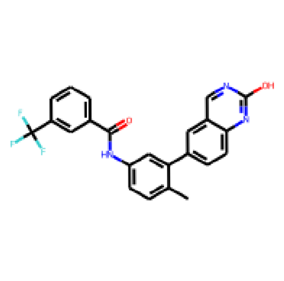	6.0	0.638163	3.995589	2.988471	4	0.818430
**2905**	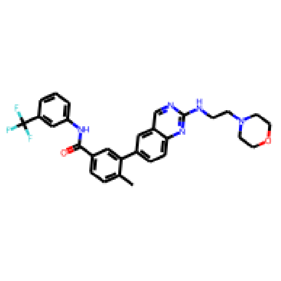	0.435185	Cc1ccc(C(=O)Nc2cccc(C(F)(C)F)c2C)cc1-c1ccc2nc(C)ccccc(F)nc2c1	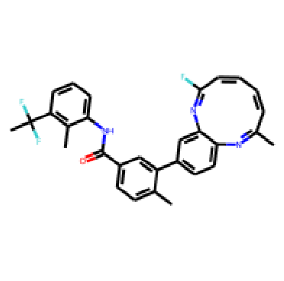	6.0	0.742375	4.384502	0.286234	4	0.779167
**2906**	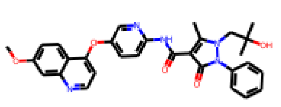	0.435035	COc1ccc2c(=O)cc(CNC(=O)c3c(C)n(C)n(-c4ccccc4)c3=O)nc3cccc3c2n1	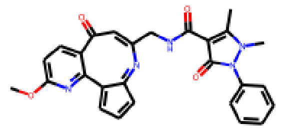	9.0	0.768132	3.608437	2.513170	5	0.823048
**2907**	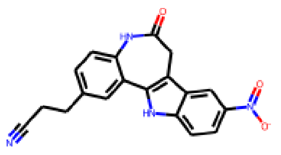	0.434932	CCNC(=O)N1CCC(Nc2n(C(C)c3c(-c4ccccc4)[nH]c4ccccc34)n2)CC1	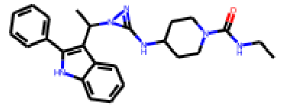	7.0	0.623584	3.603734	4.478009	5	0.804049

	src known mol	similarity	smiles	mol	Class	qed	logP	SAS	rings	kinase inhibition likeliness
**2908**	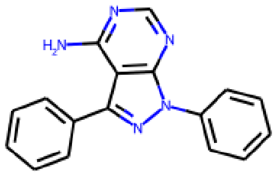	0.434636	Cc1ccc(C(=O)Oc2ccon2)cc1-c1ncnc2c1cnn2-c1ccccc1	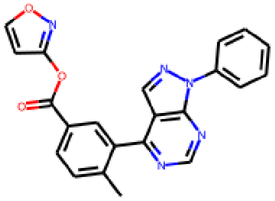	8.0	0.635315	4.542409	3.856266	5	0.760056
**2909**	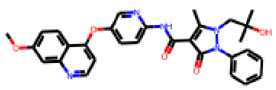	0.434582	COc1ccc2c(=O)cc(CNC(=O)c3c(C)n(C)n(-c4ccccc4)c3=O)nc3cccc3c2n1	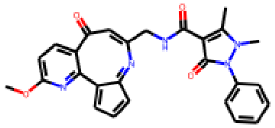	9.0	0.768132	3.608437	2.513170	5	0.823048
**2910**	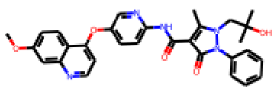	0.434582	COc1ccc2c(=O)cc(CNC(=O)c3c(C)n(C)n(-c4ccccc4)c3=O)nc3cccc3c2n1	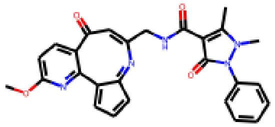	9.0	0.768132	3.608437	2.513170	5	0.823048
**2911**	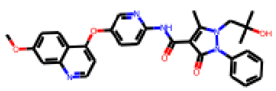	0.434582	COc1ccc2c(=O)cc(CNC(=O)c3c(C)n(C)n(-c4ccccc4)c3=O)nc3cccc3c2n1	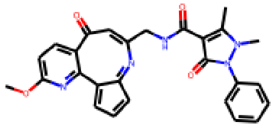	9.0	0.768132	3.608437	2.513170	5	0.823048

	src known mol	similarity	smiles	mol	Class	qed	logP	SAS	rings	kinase inhibition likeliness
**2912**	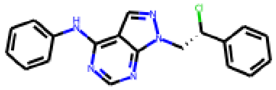	0.434487	Cc1ccc(C(=O)Nc2ccon2)cc1Nc1ncnc2c1cnn2C1cccccc1	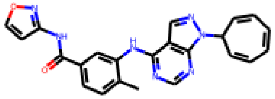	8.0	0.623362	2.525411	2.997881	5	0.770298
**2913**	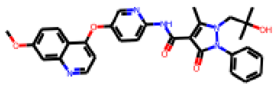	0.434211	COc1ccc2c(=O)cc(CNC(=O)c3c(C)n(C)n(-c4ccccc4)c3=O)nc3cccc2c3c1	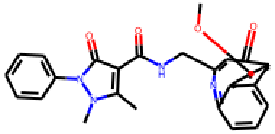	9.0	0.638163	3.995589	2.988471	5	0.774651
**2914**	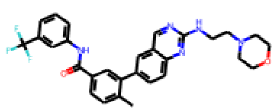	0.433881	Cc1ccc(NC(=O)c2cccc(C(F)(F)F)c2)cc1-c1ccc2nc(O)ncc2c1	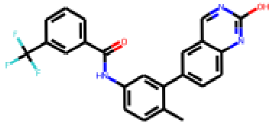	6.0	0.638163	3.995589	2.988471	4	0.818430
**2915**	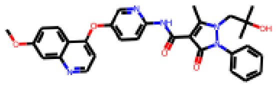	0.433516	COc1ccc2c(=O)cc(CNC(=O)c3c(C)n(C)n(-c4ccccc4)c3=O)nc3cccc2c3c1	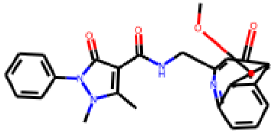	9.0	0.638163	3.995589	2.988471	5	0.774651

	src known mol	similarity	smiles	mol	Class	qed	logP	SAS	rings	kinase inhibition likeliness
**2916**	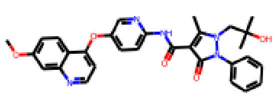	0.433516	COc1ccc2c(=O)cc(CNC(=O)c3c(C)n(C)n(-c4ccccc4)c3=O)nc3cccc2c3c1	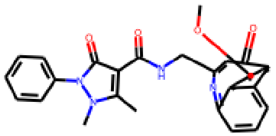	9.0	0.638163	3.995589	2.988471	5	0.774651
**2917**	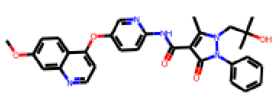	0.433516	COc1ccc2c(=O)cc(CNC(=O)c3c(C)n(C)n(-c4ccccc4)c3=O)nc3cccc2c3c1	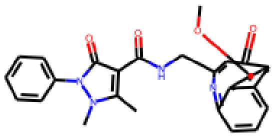	9.0	0.638163	3.995589	2.988471	5	0.774651
**2918**	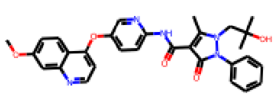	0.433516	COc1ccc2c(=O)cc(CNC(=O)c3c(C)n(C)n(-c4ccccc4)c3=O)nc3cccc2c3c1	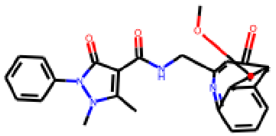	9.0	0.638163	3.995589	2.988471	5	0.774651
**2919**	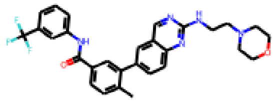	0.433509	Cc1ccc(C(=O)Nc2cccc(C(F)(C)F)c2C)cc1-c1ccc2nc(C)ccccc(F)nc2c1	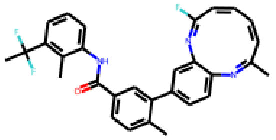	6.0	0.742375	4.384502	0.286234	4	0.779167

	src known mol	similarity	smiles	mol	Class	qed	logP	SAS	rings	kinase inhibition likeliness
**2920**	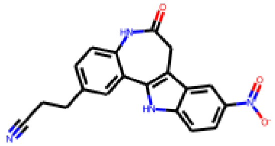	0.432906	CCNC(=O)N1CCC(Nc2n(C(C)c3c(-c4ccccc4)[nH]c4ccccc34)n2)CC1	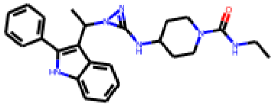	7.0	0.623584	3.603734	4.478009	5	0.804049
**2921**	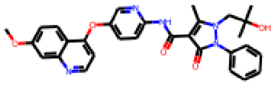	0.432640	COc1ccc2c(=O)cc(CNC(=O)c3c(C)n(C)n(-c4ccccc4)c3=O)nc3cccc2c3c1	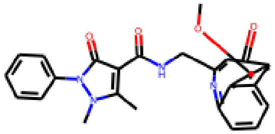	9.0	0.638163	3.995589	2.988471	5	0.774651
**2922**	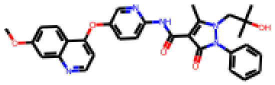	0.432640	COc1ccc2c(=O)cc(CNC(=O)c3c(C)n(C)n(-c4ccccc4)c3=O)nc3cccc2c3c1	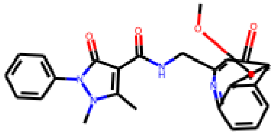	9.0	0.638163	3.995589	2.988471	5	0.774651
**2923**	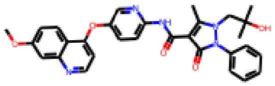	0.432640	COc1ccc2c(=O)cc(CNC(=O)c3c(C)n(C)n(-c4ccccc4)c3=O)nc3cccc2c3c1	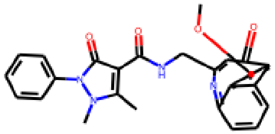	9.0	0.638163	3.995589	2.988471	5	0.774651

	src known mol	similarity	smiles	mol	Class	qed	logP	SAS	rings	kinase inhibition likeliness
**2924**	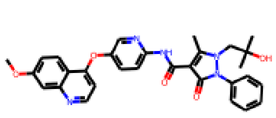	0.432566	COc1ccc2c(=O)cc(CNC(=O)c3c(C)n(C)n(-c4ccccc4)c3=O)nc3cccc2c3c1	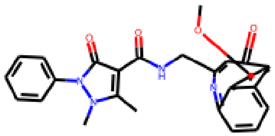	9.0	0.638163	3.995589	2.988471	5	0.774651
**2925**	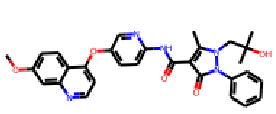	0.432507	COc1ccc2c(=O)cc(CNC(=O)c3c(C)n(C)n(-c4ccccc4)c3=O)nc3cccc2c3c1	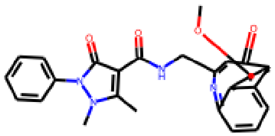	9.0	0.638163	3.995589	2.988471	5	0.774651
**2926**	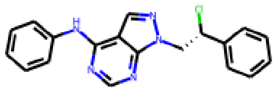	0.432336	Cc1ccc(C(=O)Nc2ccon2)cc1Nc1ncnc2c1cnn2C1cccccc1	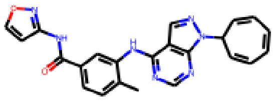	8.0	0.623362	2.525411	2.997881	5	0.770298
**2927**	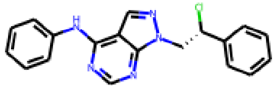	0.432074	Cc1ccc(C(=O)Nc2ccon2)cc1Nc1ncnc2c1cnn2C1cccccc1	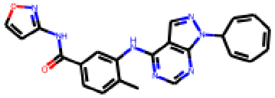	8.0	0.623362	2.525411	2.997881	5	0.770298

	src known mol	similarity	smiles	mol	Class	qed	logP	SAS	rings	kinase inhibition likeliness
**2928**	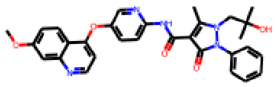	0.431949	COc1ccc2c(=O)cc(CNC(=O)c3c(C)n(C)n(-c4ccccc4)c3=O)nc3cccc3c2n1	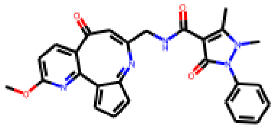	9.0	0.768132	3.608437	2.513170	5	0.823048
**2929**	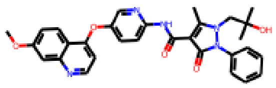	0.431949	COc1ccc2c(=O)cc(CNC(=O)c3c(C)n(C)n(-c4ccccc4)c3=O)nc3cccc3c2n1	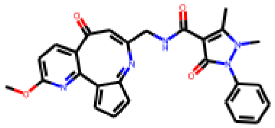	9.0	0.768132	3.608437	2.513170	5	0.823048
**2930**	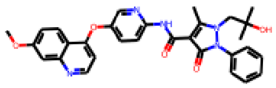	0.431726	COc1ccc2c(=O)cc(CNC(=O)c3c(C)n(C)n(-c4ccccc4)c3=O)nc3cccc3c2n1	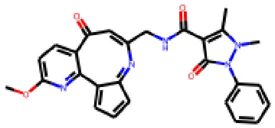	9.0	0.768132	3.608437	2.513170	5	0.823048
**2931**	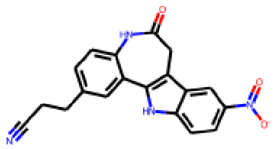	0.431664	CCNC(=O)N1CCC(Nc2n(C(C)c3c(-c4ccccc4)[nH]c4ccccc34)n2)CC1	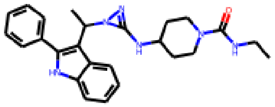	7.0	0.623584	3.603734	4.478009	5	0.804049

	src known mol	similarity	smiles	mol	Class	qed	logP	SAS	rings	kinase inhibition likeliness
**2932**	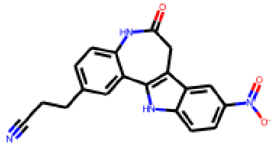	0.431664	CCNC(=O)N1CCC(Nc2n(C(C)c3c(-c4ccccc4)[nH]c4ccccc34)n2)CC1	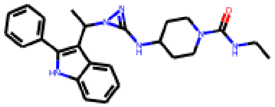	7.0	0.623584	3.603734	4.478009	5	0.804049
**2933**	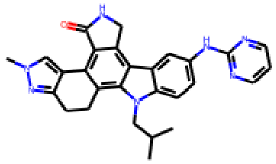	0.431013	Cc1ccc(C(=O)Nc2cccc(C(F)(C)F)c2C)cc1-c1ccc2nc(=O)cccc(F)nc2n1	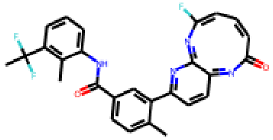	6.0	0.820294	3.038428	1.698172	4	0.833140
**2934**	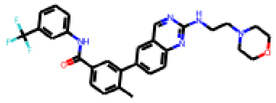	0.431004	Cc1ccc(NC(=O)c2cccc(C(F)(F)F)c2)cc1-c1ccc2nc(O)ncc2c1	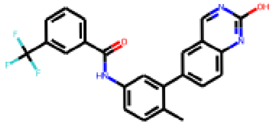	6.0	0.638163	3.995589	2.988471	4	0.818430
**2935**	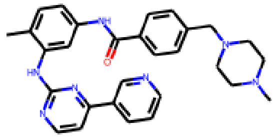	0.430851	Cc1ccc(NC(=O)c2ccccc2)cc1Nc1nccc(-c2ccccc2)n1	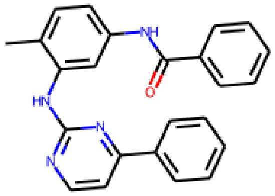	0.0	0.635315	4.542409	3.856266	4	0.812405

	src known mol	similarity	smiles	mol	Class	qed	logP	SAS	rings	kinase inhibition likeliness
**2936**	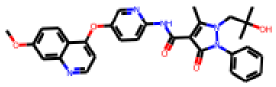	0.430814	COc1ccc2c(=O)cc(CNC(=O)c3c(C)n(C)n(-c4ccccc4)c3=O)nc3cccc3c2n1	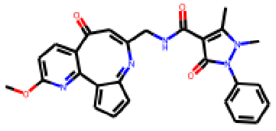	9.0	0.768132	3.608437	2.513170	5	0.823048
**2937**	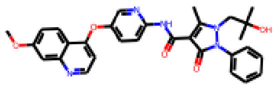	0.430805	COc1ccc2c(=O)cc(CNC(=O)c3c(C)n(C)n(-c4ccccc4)c3=O)nc3cccc3c2n1	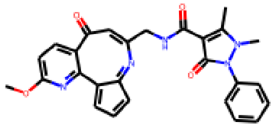	9.0	0.768132	3.608437	2.513170	5	0.823048
**2938**	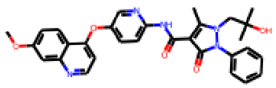	0.430716	COc1ccc2c(=O)cc(CNC(=O)c3c(C)n(C)n(-c4ccccc4)c3=O)nc3cccc3c2n1	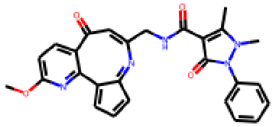	9.0	0.768132	3.608437	2.513170	5	0.823048
**2939**	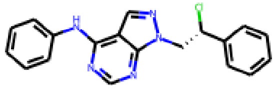	0.430565	Cc1ccc(C(=O)Nc2ccon2)cc1Nc1ncnc2c1cnn2C1cccccc1	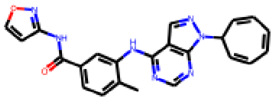	8.0	0.623362	2.525411	2.997881	5	0.770298

	src known mol	similarity	smiles	mol	Class	qed	logP	SAS	rings	kinase inhibition likeliness
**2940**	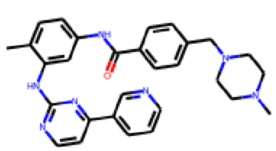	0.430126	Cc1ccc(NC(=O)c2ccccc2)cc1Nc1nccc(-c2cccnc2)n1	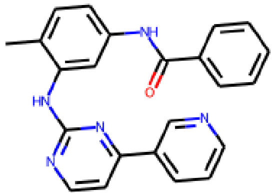	0.0	0.768132	3.608437	2.513170	4	0.854555
**2941**	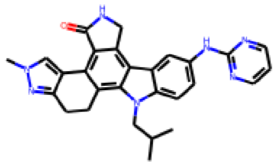	0.429942	Cc1ccc(C(=O)Nc2cccc(C(F)(C)F)c2C)cc1-c1ccc2nc(=O)cccc(F)nc2n1	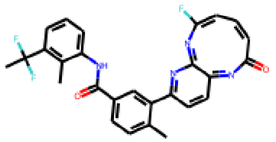	6.0	0.820294	3.038428	1.698172	4	0.833140
**2942**	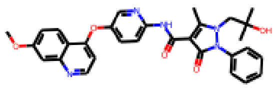	0.429398	COc1ccc2c(=O)cc(CNC(=O)c3c(C)n(C)n(-c4ccccc4)c3=O)nc3cccc3c2n1	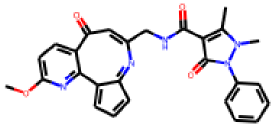	9.0	0.768132	3.608437	2.513170	5	0.823048
**2943**	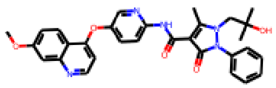	0.429398	COc1ccc2c(=O)cc(CNC(=O)c3c(C)n(C)n(-c4ccccc4)c3=O)nc3cccc3c2n1	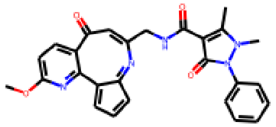	9.0	0.768132	3.608437	2.513170	5	0.823048

	src known mol	similarity	smiles	mol	Class	qed	logP	SAS	rings	kinase inhibition likeliness
**2944**	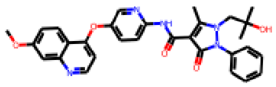	0.428819	COc1ccc2c(=O)cc(CNC(=O)c3c(C)n(C)n(-c4ccccc4)c3=O)nc3cccc3c2n1	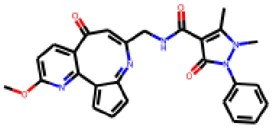	9.0	0.768132	3.608437	2.513170	5	0.823048
**2945**	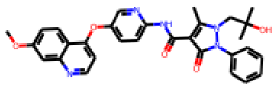	0.428819	COc1ccc2c(=O)cc(CNC(=O)c3c(C)n(C)n(-c4ccccc4)c3=O)nc3cccc3c2n1	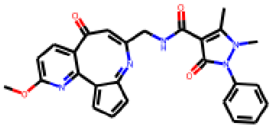	9.0	0.768132	3.608437	2.513170	5	0.823048
**2946**	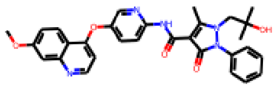	0.428819	COc1ccc2c(=O)cc(CNC(=O)c3c(C)n(C)n(-c4ccccc4)c3=O)nc3cccc3c2n1	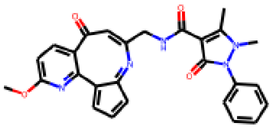	9.0	0.768132	3.608437	2.513170	5	0.823048
**2947**	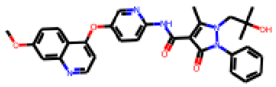	0.428817	COc1ccc2c(=O)cc(CNC(=O)c3c(C)n(C)n(-c4ccccc4)c3=O)nc3cccc3c2n1	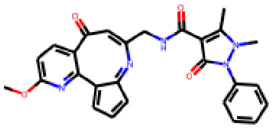	9.0	0.768132	3.608437	2.513170	5	0.823048

	src known mol	similarity	smiles	mol	Class	qed	logP	SAS	rings	kinase inhibition likeliness
**2948**	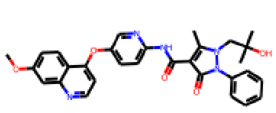	0.428493	COc1ccc2c(=O)cc(CNC(=O)c3c(C)n(C)n(-c4ccccc4)c3=O)nc3cccc2c3c1	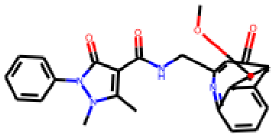	9.0	0.638163	3.995589	2.988471	5	0.774651
**2949**	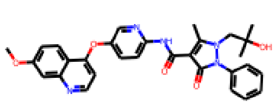	0.427570	COc1ccc2c(=O)cc(CNC(=O)c3c(C)n(C)n(-c4ccccc4)c3=O)nc3cccc3c2n1	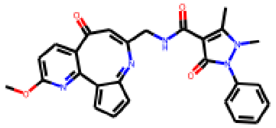	9.0	0.768132	3.608437	2.513170	5	0.823048
**2950**	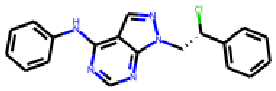	0.427333	Cc1ccc(C(=O)Nc2ccon2)cc1Nc1ncnc2c1cnn2C1cccccc1	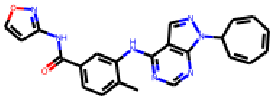	8.0	0.623362	2.525411	2.997881	5	0.770298
**2951**	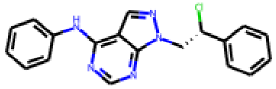	0.427027	Cc1ccc(C(=O)Nc2ccon2)cc1Nc1ncnc2c1cnn2C1cccccc1	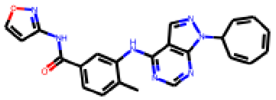	8.0	0.623362	2.525411	2.997881	5	0.770298

	src known mol	similarity	smiles	mol	Class	qed	logP	SAS	rings	kinase inhibition likeliness
**2952**	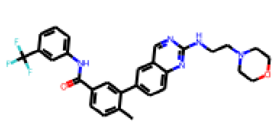	0.426579	Cc1ccc(C(=O)Nc2cccc(C(F)(F)F)c2C)cc1-c1ccc2nc(O)ccccc(F)cc2c1	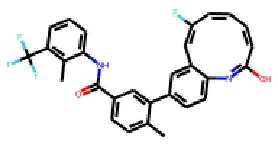	6.0	0.771505	2.481548	2.110899	4	0.808477
**2953**	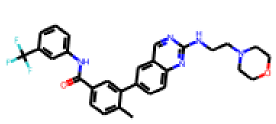	0.426579	Cc1ccc(C(=O)Nc2cccc(C(F)(F)F)c2C)cc1-c1ccc2nc(O)ccccc(F)cc2c1	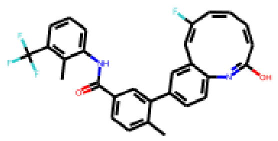	6.0	0.771505	2.481548	2.110899	4	0.808477
**2954**	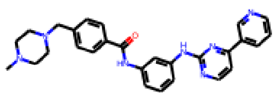	0.426174	Fc1ccc(NC(=O)c2ccccc2)cc1Nc1nccc(-c2cccnc2)n1	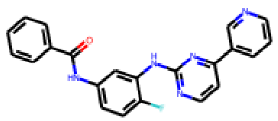	0.0	0.632663	4.308233	4.065316	4	0.777346
**2955**	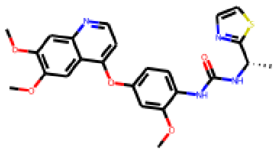	0.426047	COc1ccccc1NC(=O)Nc1ccc(Oc2ccnc3cc(OC)c(OC)cc23)cc1	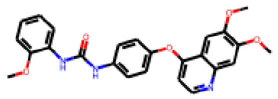	5.0	0.638163	3.995589	2.988471	4	0.789556

	src known mol	similarity	smiles	mol	Class	qed	logP	SAS	rings	kinase inhibition likeliness
**2956**	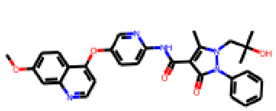	0.425594	COc1ccc2c(=O)cc(CNC(=O)c3c(C)n(C)n(-c4ccccc4)c3=O)nc3cccc3c2n1	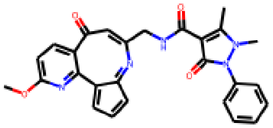	9.0	0.768132	3.608437	2.513170	5	0.823048
**2957**	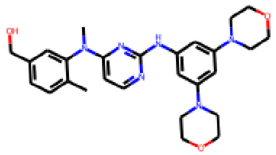	0.425477	NC(=O)c1ccccc1Nc1nc(Nc2ccc(N3CCOCC3)cc2)ncc1Cl	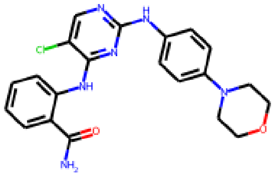	7.0	0.768132	3.608437	2.513170	4	0.755040
**2958**	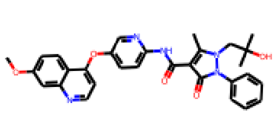	0.425041	COc1ccc2c(=O)cc(CNC(=O)c3c(C)n(C)n(-c4ccccc4)c3=O)nc3cccc2c3c1	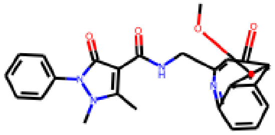	9.0	0.638163	3.995589	2.988471	5	0.774651
**2959**	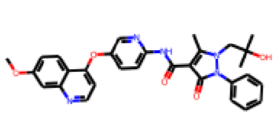	0.425014	COc1ccc2c(=O)cc(CNC(=O)c3c(C)n(C)n(-c4ccccc4)c3=O)nc3cccc2c3c1	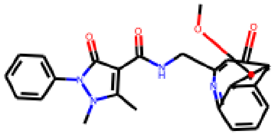	9.0	0.638163	3.995589	2.988471	5	0.774651

	src known mol	similarity	smiles	mol	Class	qed	logP	SAS	rings	kinase inhibition likeliness
**2960**	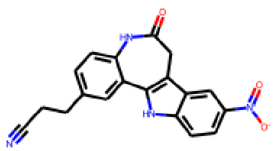	0.424484	CCNC(=O)N1CCC(Nc2n(C(C)c3c(-c4ccccc4)[nH]c4ccccc34)n2)CC1	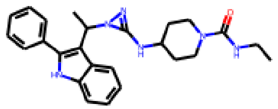	7.0	0.623584	3.603734	4.478009	5	0.804049
**2961**	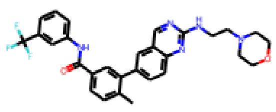	0.424403	Cc1ccc(C(=O)Oc2cccc(C(F)(F)F)c2)cc1-c1ccc2nc(N)ncc2c1	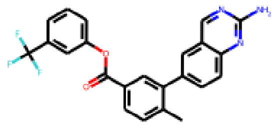	6.0	0.638163	3.995589	2.988471	4	0.789898
**2962**	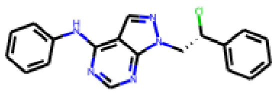	0.424364	Cc1ccc(C(=O)Nc2ccon2)cc1Nc1ncnc2c1cnn2C1cccccc1	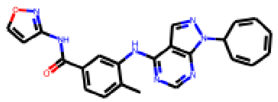	8.0	0.623362	2.525411	2.997881	5	0.770298
**2963**	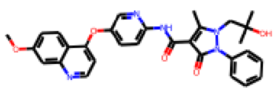	0.424226	COc1ccc2c(=O)cc(CNC(=O)c3c(C)n(C)n(-c4ccccc4)c3=O)nc3cccc2c3c1	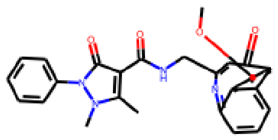	9.0	0.638163	3.995589	2.988471	5	0.774651

	src known mol	similarity	smiles	mol	Class	qed	logP	SAS	rings	kinase inhibition likeliness
**2964**	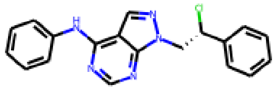	0.423898	Cc1ccc(C(=O)Nc2ccon2)cc1Nc1ncnc2c1cnn2C1cccccc1	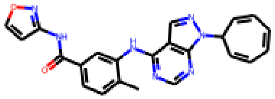	8.0	0.623362	2.525411	2.997881	5	0.770298
**2965**	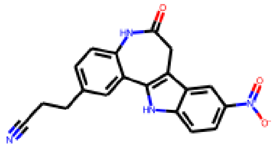	0.423752	CCNC(=O)N1CCC(Nc2n(C(C)c3c(-c4ccccc4)[nH]c4ccccc34)n2)CC1	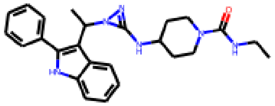	7.0	0.623584	3.603734	4.478009	5	0.804049
**2966**	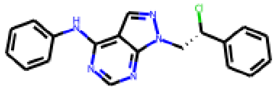	0.423284	Cc1ccc(C(=O)Nc2ccon2)cc1Nc1ncnc2c1cnn2C1cccccc1	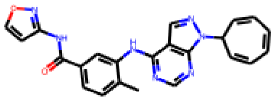	8.0	0.623362	2.525411	2.997881	5	0.770298
**2967**	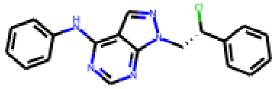	0.423186	Cc1ccc(C(=O)Nc2ccon2)cc1Nc1ncnc2c1cnn2C1cccccc1	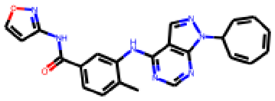	8.0	0.623362	2.525411	2.997881	5	0.770298

	src known mol	similarity	smiles	mol	Class	qed	logP	SAS	rings	kinase inhibition likeliness
**2968**	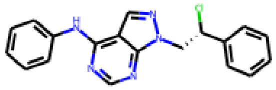	0.423181	Cc1ccc(C(=O)Nc2ccon2)cc1Nc1ncnc2c1cnn2C1cccccc1	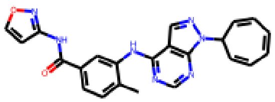	8.0	0.623362	2.525411	2.997881	5	0.770298
**2969**	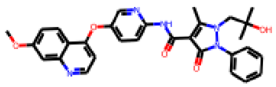	0.422782	COc1ccc2c(=O)cc(CNC(=O)c3c(C)n(C)n(-c4ccccc4)c3=O)nc3cccc2c3c1	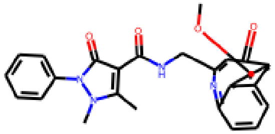	9.0	0.638163	3.995589	2.988471	5	0.774651
**2970**	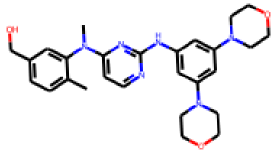	0.422731	NC(=O)c1ccccc1Nc1nc(Nc2ccc(N3CCCC3=O)cc2)ncc1Cl	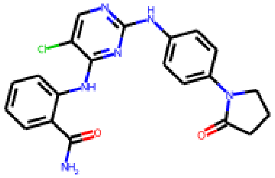	7.0	0.768132	3.608437	2.513170	4	0.773984
**2971**	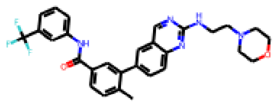	0.422680	Cc1ccc(C(=O)Nc2cccc(C(F)(F)F)c2)cc1-c1ccc2nc(N)ccc2c1	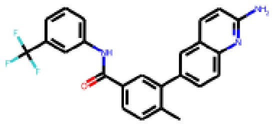	6.0	0.635315	4.542409	3.856266	4	0.833561

	src known mol	similarity	smiles	mol	Class	qed	logP	SAS	rings	kinase inhibition likeliness
**2972**	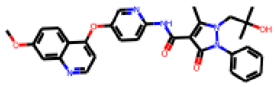	0.422560	COc1ccc2c(=O)cc(CNC(=O)c3c(C)n(C)n(-c4ccccc4)c3=O)nc3cccc3c2n1	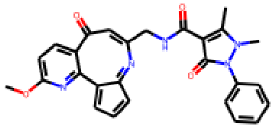	9.0	0.768132	3.608437	2.513170	5	0.823048
**2973**	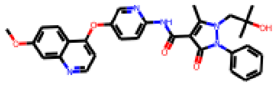	0.422543	COc1ccc2c(=O)cc(CNC(=O)c3c(C)n(C)n(-c4ccccc4)c3=O)nc3cccc3c2n1	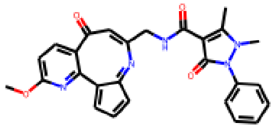	9.0	0.768132	3.608437	2.513170	5	0.823048
**2974**	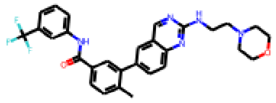	0.422487	Cc1ccc(C(=O)Nc2cccc(C(F)(F)F)c2)cc1-c1ccc2nc(N)ccc2c1	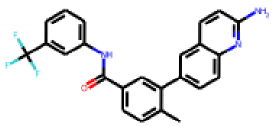	6.0	0.635315	4.542409	3.856266	4	0.833561
**2975**	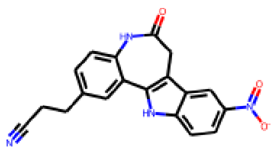	0.422438	CCNC(=O)N1CCC(Nc2n(C(C)c3c(-c4ccccc4)[nH]c4ccccc34)n2)CC1	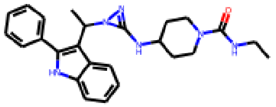	7.0	0.623584	3.603734	4.478009	5	0.804049

	src known mol	similarity	smiles	mol	Class	qed	logP	SAS	rings	kinase inhibition likeliness
**2976**	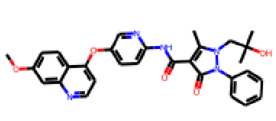	0.422414	COc1ccc2c(=O)cc(CNC(=O)c3c(C)n(C)n(-c4ccccc4)c3=O)nc3cccc3c2n1	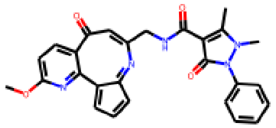	9.0	0.768132	3.608437	2.513170	5	0.823048
**2977**	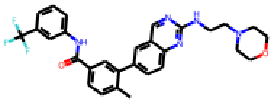	0.421834	Cc1ccc(C(=O)Nc2cccc(C(F)(F)F)c2)cc1-c1ccc2nc(N)ccc2c1	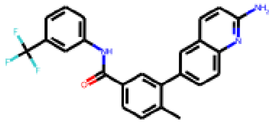	6.0	0.635315	4.542409	3.856266	4	0.833561
**2978**	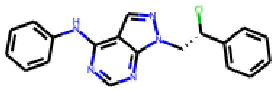	0.421695	Cc1ccc(C(=O)Nc2ccon2)cc1Nc1ncnc2c1cnn2C1cccccc1	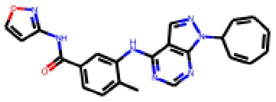	8.0	0.623362	2.525411	2.997881	5	0.770298
**2979**	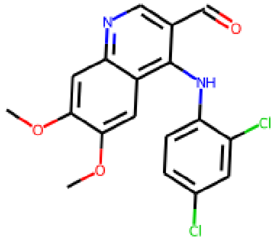	0.421374	COc1cc2ncc(C(=O)O)c(Nc3cccc(Cl)c3Cl)c2cc1OC1C(C)C1	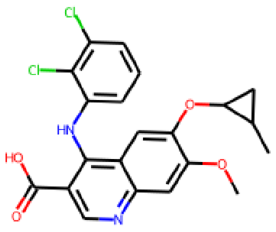	2.0	0.623362	2.525411	2.997881	4	0.774857

	src known mol	similarity	smiles	mol	Class	qed	logP	SAS	rings	kinase inhibition likeliness
**2980**	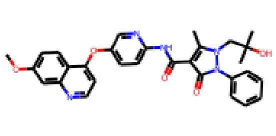	0.420849	COc1ccc2c(=O)cc(CNC(=O)c3c(C)n(C)n(-c4ccccc4)c3=O)nc3cccc2c3c1	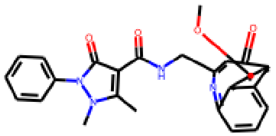	9.0	0.638163	3.995589	2.988471	5	0.774651
**2981**	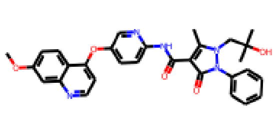	0.420653	COc1ccc2c(=O)cc(CNC(=O)c3c(C)n(C)n(-c4ccccc4)c3=O)nc3cccc3c2n1	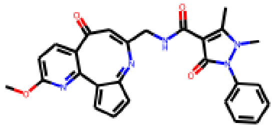	9.0	0.768132	3.608437	2.513170	5	0.823048
**2982**	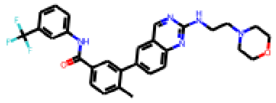	0.420608	Cc1ccc(C(=O)Nc2cccc(C(F)(F)F)c2)cc1-c1ccc2nc(N)ccc2c1	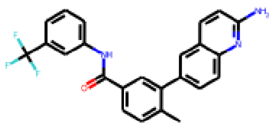	6.0	0.635315	4.542409	3.856266	4	0.833561
**2983**	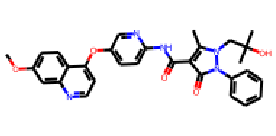	0.420587	COc1ccc2c(=O)cc(CNC(=O)c3c(C)n(C)n(-c4ccccc4)c3=O)nc3cccc2c3c1	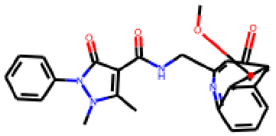	9.0	0.638163	3.995589	2.988471	5	0.774651

	src known mol	similarity	smiles	mol	Class	qed	logP	SAS	rings	kinase inhibition likeliness
**2984**	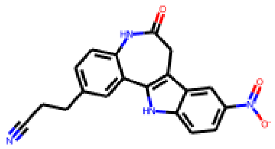	0.420502	CCNC(=O)N1CCC(Nc2n(C(C)c3c(-c4ccccc4)[nH]c4ccccc34)n2)CC1	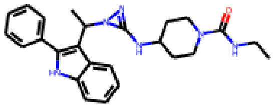	7.0	0.623584	3.603734	4.478009	5	0.804049
**2985**	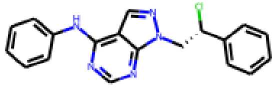	0.420339	Cc1ccc(C(=O)Nc2ccon2)cc1Nc1ncnc2c1cnn2C1cccccc1	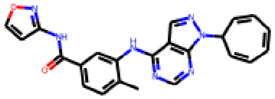	8.0	0.623362	2.525411	2.997881	5	0.770298
**2986**	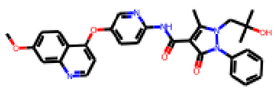	0.420298	COc1ccc2c(=O)cc(CNC(=O)c3c(C)n(C)n(-c4ccccc4)c3=O)nc3cccc2c3c1	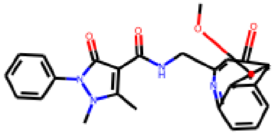	9.0	0.638163	3.995589	2.988471	5	0.774651
**2987**	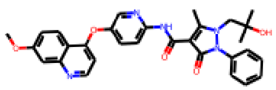	0.420298	COc1ccc2c(=O)cc(CNC(=O)c3c(C)n(C)n(-c4ccccc4)c3=O)nc3cccc2c3c1	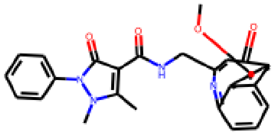	9.0	0.638163	3.995589	2.988471	5	0.774651

	src known mol	similarity	smiles	mol	Class	qed	logP	SAS	rings	kinase inhibition likeliness
**2988**	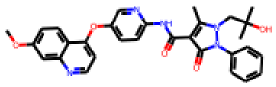	0.420298	COc1ccc2c(=O)cc(CNC(=O)c3c(C)n(C)n(-c4ccccc4)c3=O)nc3cccc2c3c1	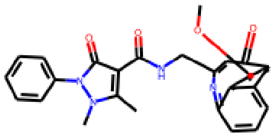	9.0	0.638163	3.995589	2.988471	5	0.774651
**2989**	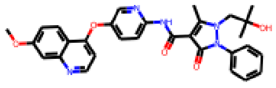	0.420298	COc1ccc2c(=O)cc(CNC(=O)c3c(C)n(C)n(-c4ccccc4)c3=O)nc3cccc2c3c1	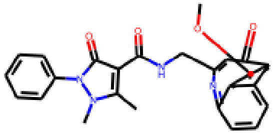	9.0	0.638163	3.995589	2.988471	5	0.774651
**2990**	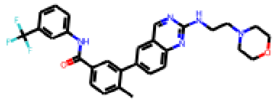	0.420175	Cc1ccc(C(=O)Nc2cccc(C(F)(F)F)c2)cc1-c1ccc2nc(N)ccc2c1	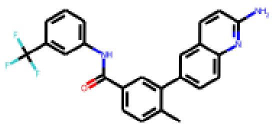	6.0	0.635315	4.542409	3.856266	4	0.833561
**2991**	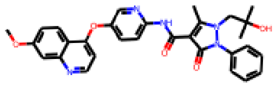	0.419738	COc1ccc2c(=O)cc(CNC(=O)c3c(C)n(C)n(-c4ccccc4)c3=O)nc3cccc3c2n1	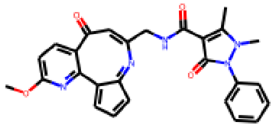	9.0	0.768132	3.608437	2.513170	5	0.823048

	src known mol	similarity	smiles	mol	Class	qed	logP	SAS	rings	kinase inhibition likeliness
**2992**	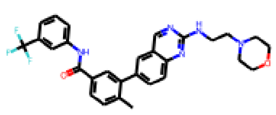	0.419718	Cc1ccc(C(=O)Nc2cccc(C(F)(F)F)c2)cc1-c1ccc2nc(N)ccc2c1	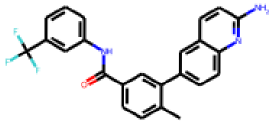	6.0	0.635315	4.542409	3.856266	4	0.833561
**2993**	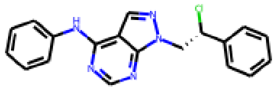	0.419463	Cc1ccc(C(=O)Nc2ccon2)cc1Nc1ncnc2c1cnn2C1cccccc1	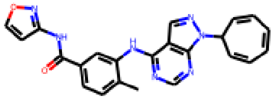	8.0	0.623362	2.525411	2.997881	5	0.770298
**2994**	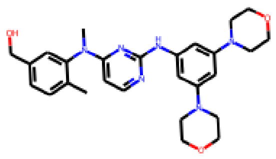	0.419014	NC(=O)c1ccccc1Nc1nc(Nc2ccc(N3CCOCC3)cc2)ncc1Cl	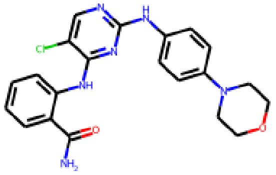	7.0	0.768132	3.608437	2.513170	4	0.755040
**2995**	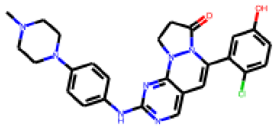	0.418824	O/C(Nc1ccc(Oc2ccccc2)cc1)Oc1ccc2(Cc1)NC(=O)/C(=O)c1ccc(O)n12	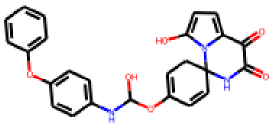	4.0	0.520676	2.336694	2.899742	5	0.820778

	src known mol	similarity	smiles	mol	Class	qed	logP	SAS	rings	kinase inhibition likeliness
**2996**	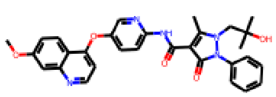	0.418695	COc1ccc2c(=O)cc(CNC(=O)c3c(C)n(C)n(-c4ccccc4)c3=O)nc3cccc2c3c1	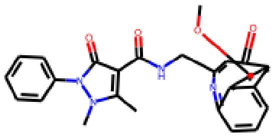	9.0	0.638163	3.995589	2.988471	5	0.774651
**2997**	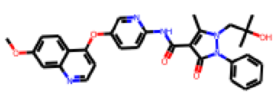	0.418553	COc1ccc2c(=O)cc(CNC(=O)c3c(C)n(C)n(-c4ccccc4)c3=O)nc3cccc2c3c1	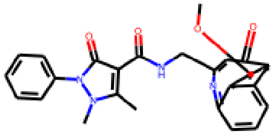	9.0	0.638163	3.995589	2.988471	5	0.774651
**2998**	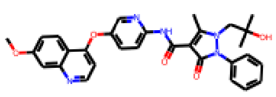	0.418553	COc1ccc2c(=O)cc(CNC(=O)c3c(C)n(C)n(-c4ccccc4)c3=O)nc3cccc2c3c1	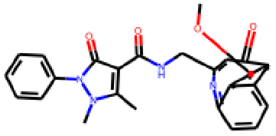	9.0	0.638163	3.995589	2.988471	5	0.774651
**2999**	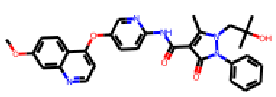	0.418523	COc1ccc2c(=O)cc(CNC(=O)c3c(C)n(C)n(-c4ccccc4)c3=O)nc3cccc3c2n1	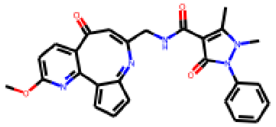	9.0	0.768132	3.608437	2.513170	5	0.823048

	src known mol	similarity	smiles	mol	Class	qed	logP	SAS	rings	kinase inhibition likeliness
**3000**	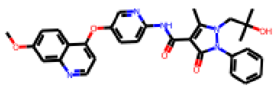	0.418510	COc1ccc2c(=O)cc(CNC(=O)c3c(C)n(C)n(-c4ccccc4)c3=O)nc3cccc3c2n1	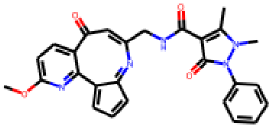	9.0	0.768132	3.608437	2.513170	5	0.823048
**3001**	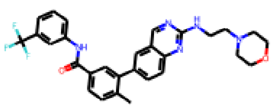	0.417951	Cc1ccc(C(=O)Nc2cccc(C(F)(F)F)c2)cc1-c1ccc2nc(N)ccc2c1	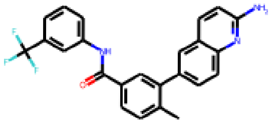	6.0	0.635315	4.542409	3.856266	4	0.833561
**3002**	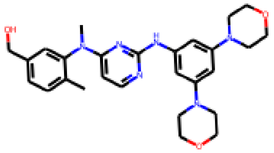	0.417690	NC(=O)c1ccccc1Nc1nc(Nc2ccc(N3CCCC3=O)cc2)ncc1Cl	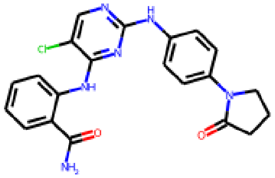	7.0	0.768132	3.608437	2.513170	4	0.773984
**3003**	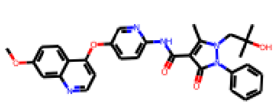	0.417685	COc1ccc2c(=O)cc(CNC(=O)c3c(C)n(C)n(-c4ccccc4)c3=O)nc3cccc3c2n1	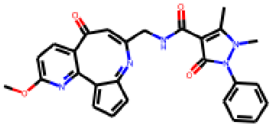	9.0	0.768132	3.608437	2.513170	5	0.823048

	src known mol	similarity	smiles	mol	Class	qed	logP	SAS	rings	kinase inhibition likeliness
**3004**	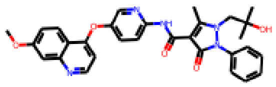	0.417499	COc1ccc2c(=O)cc(CNC(=O)c3c(C)n(C)n(-c4ccccc4)c3=O)nc3cccc3c2n1	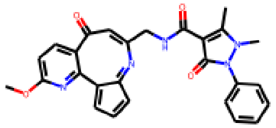	9.0	0.768132	3.608437	2.513170	5	0.823048
**3005**	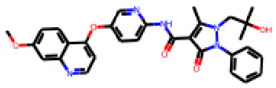	0.417499	COc1ccc2c(=O)cc(CNC(=O)c3c(C)n(C)n(-c4ccccc4)c3=O)nc3cccc3c2n1	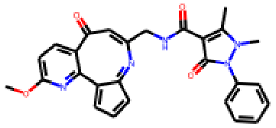	9.0	0.768132	3.608437	2.513170	5	0.823048
**3006**	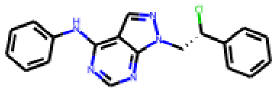	0.417010	Cc1ccc(C(=O)Nc2ccon2)cc1Nc1ncnc2c1cnn2C1cccccc1	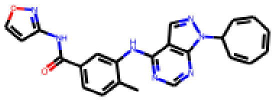	8.0	0.623362	2.525411	2.997881	5	0.770298
**3007**	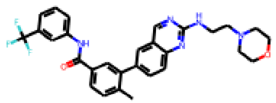	0.416981	Cc1ccc(C(=O)Nc2cccc(C(F)(F)F)c2)cc1-c1ccc2nc(N)ccc2c1	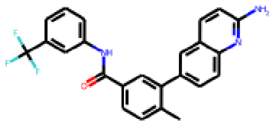	6.0	0.635315	4.542409	3.856266	4	0.833561

	src known mol	similarity	smiles	mol	Class	qed	logP	SAS	rings	kinase inhibition likeliness
**3008**	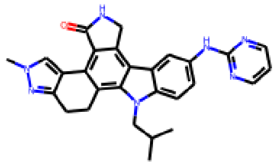	0.416826	C#Cc1ccc(C(=O)OC)cc1-c1C(=O)c-n2nc(-c3ccc(F)cc3F)cc2C1	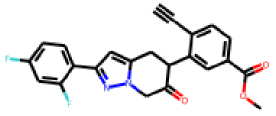	8.0	0.768132	3.608437	2.513170	4	0.763730
**3009**	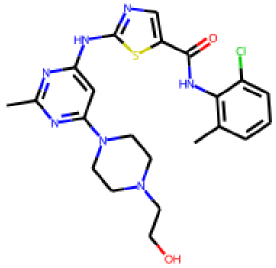	0.416781	Cc1nc(Nc2ccc(C(=O)Oc3c(C)cccc3Cl)s2)cc(N2CCNC(C)c2C)n1	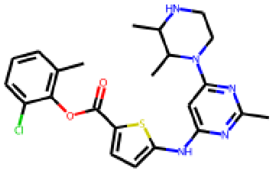	2.0	0.776263	3.686558	0.949744	4	0.784833
**3010**	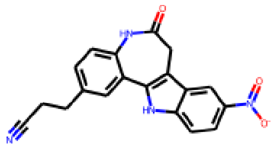	0.416609	CCNC(=O)N1CCC(Nc2n(C(C)c3c(-c4ccccc4)[nH]c4ccccc34)n2)CC1	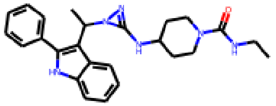	7.0	0.623584	3.603734	4.478009	5	0.804049
**3011**	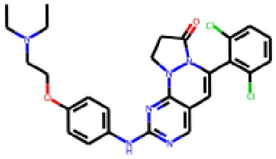	0.416502	COc1cc(Nc2ncc(C)c(-c3ccc4c(n3)NC(=O)C(C)(C)C4)n2)cc(OC)c1OC	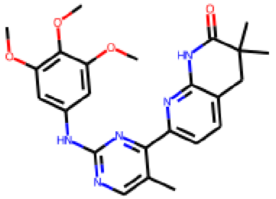	4.0	0.768132	3.608437	2.513170	4	0.757734

	src known mol	similarity	smiles	mol	Class	qed	logP	SAS	rings	kinase inhibition likeliness
**3012**	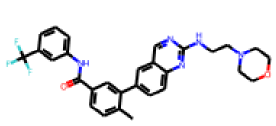	0.416463	Cc1ccc(C(=O)Nc2cccc(C(F)(C)F)c2C)cc1-c1ccc2nc(C)ccccc(F)nc2c1	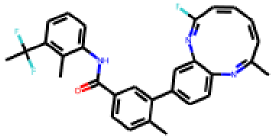	6.0	0.742375	4.384502	0.286234	4	0.779167
**3013**	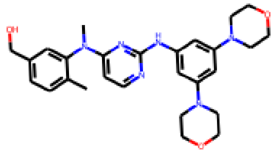	0.416120	NC(=O)c1ccccc1Nc1nc(Nc2ccc(N3CCOCC3)cc2)ncc1Cl	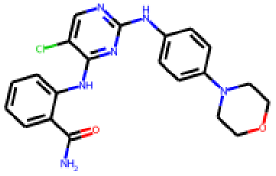	7.0	0.768132	3.608437	2.513170	4	0.755040
**3014**	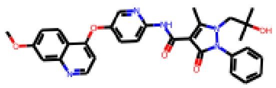	0.416026	COc1ccc2c(=O)cc(CNC(=O)c3c(C)n(C)n(-c4ccccc4)c3=O)nc3cccc2c3c1	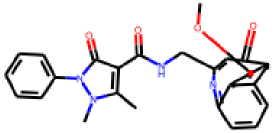	9.0	0.638163	3.995589	2.988471	5	0.774651
**3015**	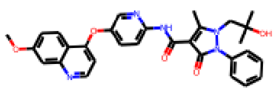	0.415769	COc1ccc2c(=O)cc(CNC(=O)c3c(C)n(C)n(-c4ccccc4)c3=O)nc3cccc3c2n1	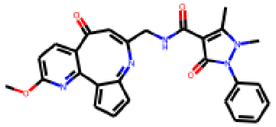	9.0	0.768132	3.608437	2.513170	5	0.823048

	src known mol	similarity	smiles	mol	Class	qed	logP	SAS	rings	kinase inhibition likeliness
**3016**	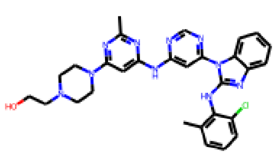	0.415708	Cc1nc(Nc2ccc(C(=O)Oc3c(C)cccc3Cl)s2)cc(N2CCNC(=O)C2=O)n1	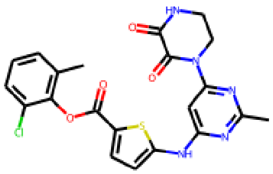	2.0	0.584673	2.312750	2.571988	4	0.770333
**3017**	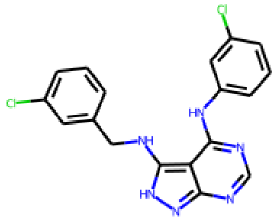	0.415683	Clc1cccc(Nc2ncnc3n[nH]c(N4ccccc(Cl)c4)c23)c1	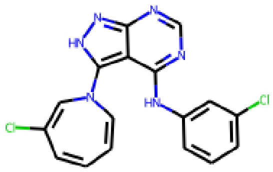	0.0	0.566600	4.200036	5.049812	4	0.795377
**3018**	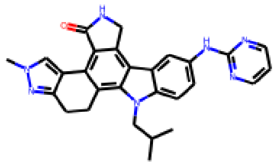	0.414953	C#Cc1ccc(C(=O)OC)cc1-c1C(=O)c-n2nc(-c3ccc(F)cc3F)cc2C1	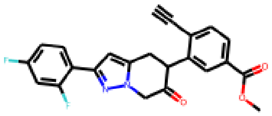	8.0	0.768132	3.608437	2.513170	4	0.763730
**3019**	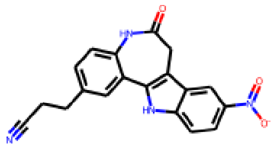	0.414938	CCNC(=O)N1CCC(Nc2n(C(C)c3c(-c4ccccc4)[nH]c4ccccc34)n2)CC1	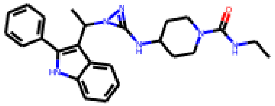	7.0	0.623584	3.603734	4.478009	5	0.804049

	src known mol	similarity	smiles	mol	Class	qed	logP	SAS	rings	kinase inhibition likeliness
**3020**	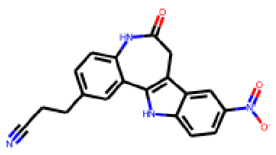	0.414856	CCNC(=O)N1CCC(Nc2n(C(C)c3c(-c4ccccc4)[nH]c4ccccc34)n2)CC1	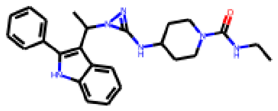	7.0	0.623584	3.603734	4.478009	5	0.804049
**3021**	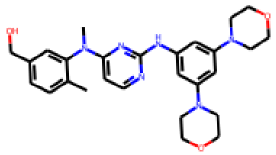	0.414656	NC(=O)c1ccccc1Nc1nc(Nc2ccc(N3CCOCC3)cc2)ncc1Cl	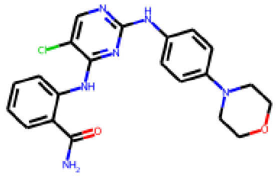	7.0	0.768132	3.608437	2.513170	4	0.755040
**3022**	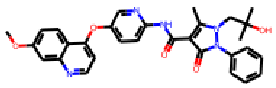	0.414634	COc1ccc2c(=O)cc(CNC(=O)c3c(C)n(C)n(-c4ccccc4)c3=O)nc3cccc3c2n1	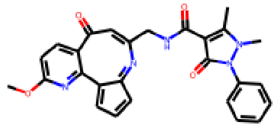	9.0	0.768132	3.608437	2.513170	5	0.823048
**3023**	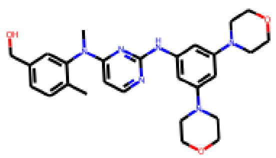	0.414512	NC(=O)c1ccccc1Nc1nc(Nc2ccc(N3CCCC3=O)cc2)ncc1Cl	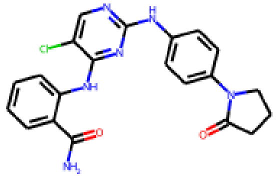	7.0	0.768132	3.608437	2.513170	4	0.773984

	src known mol	similarity	smiles	mol	Class	qed	logP	SAS	rings	kinase inhibition likeliness
**3024**	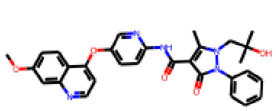	0.414219	COc1ccc2c(=O)cc(CNC(=O)c3c(C)n(C)n(-c4ccccc4)c3=O)nc3cccc3c2n1	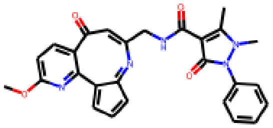	9.0	0.768132	3.608437	2.513170	5	0.823048
**3025**	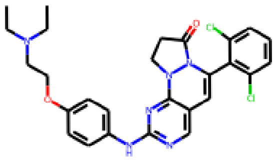	0.413982	COc1cc(Nc2ncc(C)c(-c3ccc4c(n3)NC(=O)C(C)(C)C4)n2)cc(OC)c1OC	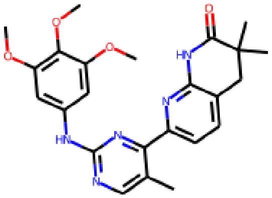	4.0	0.768132	3.608437	2.513170	4	0.757734
**3026**	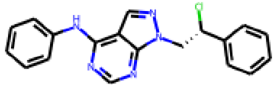	0.413864	Cc1ccc(C(=O)Nc2ccon2)cc1Nc1ncnc2c1cnn2C1cccccc1	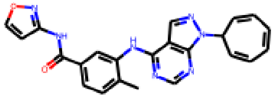	8.0	0.623362	2.525411	2.997881	5	0.770298
**3027**	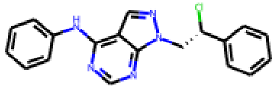	0.413652	Cc1ccc(C(=O)Nc2ccon2)cc1Nc1ncnc2c1cnn2C1cccccc1	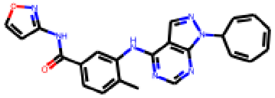	8.0	0.623362	2.525411	2.997881	5	0.770298

	src known mol	similarity	smiles	mol	Class	qed	logP	SAS	rings	kinase inhibition likeliness
**3028**	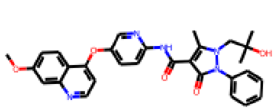	0.413473	COc1ccc2c(=O)cc(CNC(=O)c3c(C)n(C)n(-c4ccccc4)c3=O)nc3cccc3c2n1	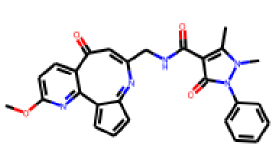	9.0	0.768132	3.608437	2.513170	5	0.823048
**3029**	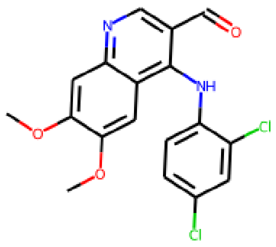	0.413093	COc1cc2ncc(C(=O)O)c(Nc3cccc(Cl)c3Cl)c2cc1OC1C(C)C1	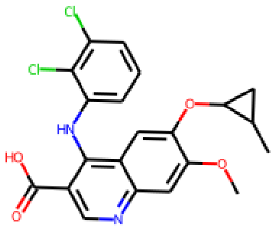	2.0	0.623362	2.525411	2.997881	4	0.774857
**3030**	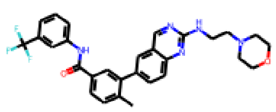	0.412651	Cc1ccc(C(=O)Nc2cccc(C(F)(C)F)c2C)cc1-c1ccc2nc(C)ccccc(F)nc2c1	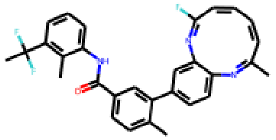	6.0	0.742375	4.384502	0.286234	4	0.779167
**3031**	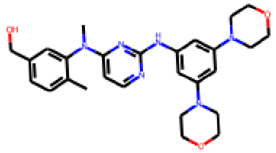	0.412281	NC(=O)c1ccccc1Nc1nc(Nc2ccc(N3CCOCC3)cc2)ncc1Cl	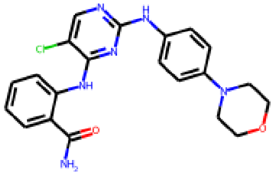	7.0	0.768132	3.608437	2.513170	4	0.755040

	src known mol	similarity	smiles	mol	Class	qed	logP	SAS	rings	kinase inhibition likeliness
**3032**	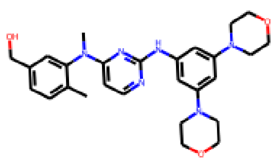	0.412157	NC(=O)c1ccccc1Nc1nc(Nc2ccc(N3CCCC3=O)cc2)ncc1Cl	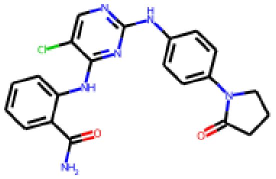	7.0	0.768132	3.608437	2.513170	4	0.773984
**3033**	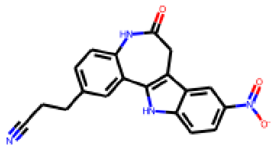	0.411806	CCNC(=O)N1CCC(Nc2n(C(C)c3c(-c4ccccc4)[nH]c4ccccc34)n2)CC1	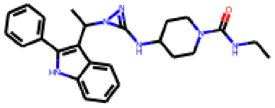	7.0	0.623584	3.603734	4.478009	5	0.804049
**3034**	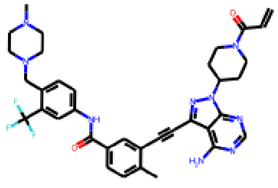	0.411650	Cc1ccc(C(=O)Nc2cccc(C(F)(C)F)c2C)cc1-c1ccc2nc(=O)ccc(C)cn2n1	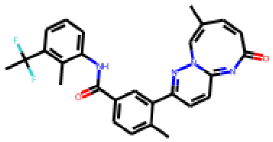	6.0	0.762994	3.476405	2.421118	4	0.764424
**3035**	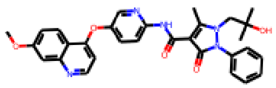	0.411308	COc1ccc2c(=O)cc(CNC(=O)c3c(C)n(C)n(-c4ccccc4)c3=O)nc3cccc2c3c1	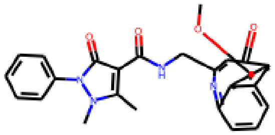	9.0	0.638163	3.995589	2.988471	5	0.774651

	src known mol	similarity	smiles	mol	Class	qed	logP	SAS	rings	kinase inhibition likeliness
**3036**	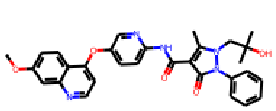	0.411308	COc1ccc2c(=O)cc(CNC(=O)c3c(C)n(C)n(-c4ccccc4)c3=O)nc3cccc2c3c1	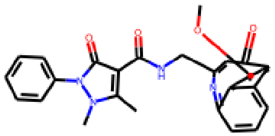	9.0	0.638163	3.995589	2.988471	5	0.774651
**3037**	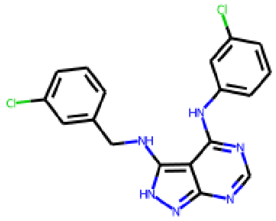	0.411290	Clc1cccc(Nc2ncnc3n[nH]c(N4ccccc(Cl)c4)c23)c1	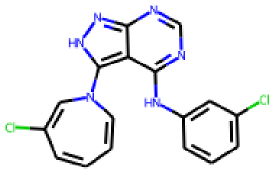	0.0	0.566600	4.200036	5.049812	4	0.795377
**3038**	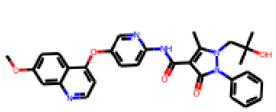	0.411019	COc1ccc2c(=O)cc(CNC(=O)c3c(C)n(C)n(-c4ccccc4)c3=O)nc3cccc2c3c1	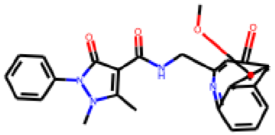	9.0	0.638163	3.995589	2.988471	5	0.774651
**3039**	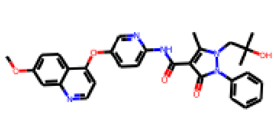	0.410839	COc1ccc2c(=O)cc(CNC(=O)c3c(C)n(C)n(-c4ccccc4)c3=O)nc3cccc3c2n1	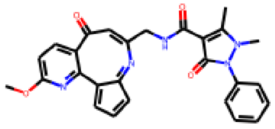	9.0	0.768132	3.608437	2.513170	5	0.823048

	src known mol	similarity	smiles	mol	Class	qed	logP	SAS	rings	kinase inhibition likeliness
**3040**	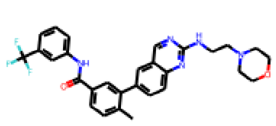	0.410619	Cc1ccc(C(=O)Nc2cccc(C(F)(F)F)c2)cc1-c1ccc2nc(N)ccc2c1	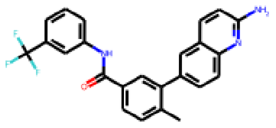	6.0	0.635315	4.542409	3.856266	4	0.833561
**3041**	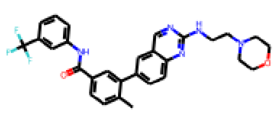	0.410304	Cc1ccc(NC(=O)c2cccc(C(F)(F)F)c2)cc1-c1ccc2nc(O)ncc2c1	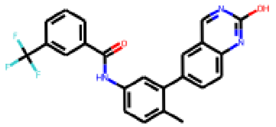	6.0	0.638163	3.995589	2.988471	4	0.818430
**3042**	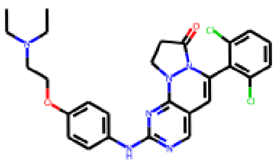	0.410082	COc1cc(Nc2ncc(C)c(-c3ccc4c(n3)NC(=O)C(C)(C)C4)n2)cc(OC)c1OC	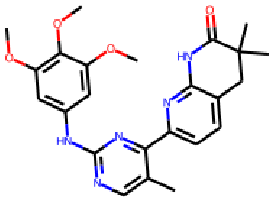	4.0	0.768132	3.608437	2.513170	4	0.757734
**3043**	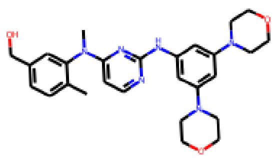	0.409677	CC(=O)c1ccccc1Nc1nc(Nc2ccc(N3CCOCC3)cc2F)ncc1Cl	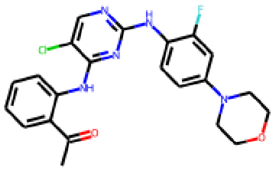	7.0	0.638163	3.995589	2.988471	4	0.772587

	src known mol	similarity	smiles	mol	Class	qed	logP	SAS	rings	kinase inhibition likeliness
**3044**	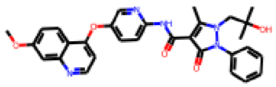	0.409539	COc1ccc2c(=O)cc(CNC(=O)c3c(C)n(C)n(-c4ccccc4)c3=O)nc3cccc2c3c1	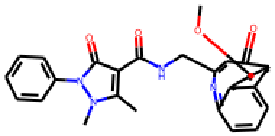	9.0	0.638163	3.995589	2.988471	5	0.774651
**3045**	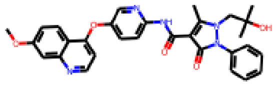	0.409458	COc1ccc2c(=O)cc(CNC(=O)c3c(C)n(C)n(-c4ccccc4)c3=O)nc3cccc3c2n1	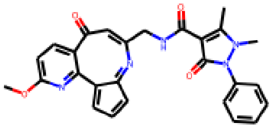	9.0	0.768132	3.608437	2.513170	5	0.823048
**3046**	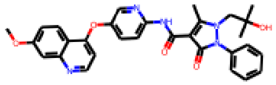	0.409458	COc1ccc2c(=O)cc(CNC(=O)c3c(C)n(C)n(-c4ccccc4)c3=O)nc3cccc3c2n1	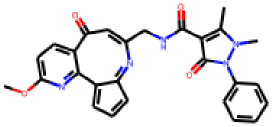	9.0	0.768132	3.608437	2.513170	5	0.823048
**3047**	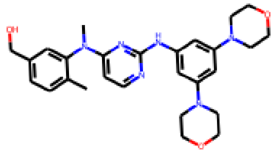	0.409436	NC(=O)c1ccccc1Nc1nc(Nc2ccc(N3CCOCC3)cc2C)ncc1Cl	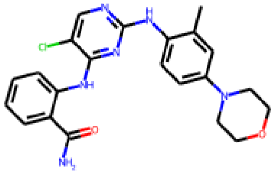	7.0	0.623362	2.525411	2.997881	4	0.783217

	src known mol	similarity	smiles	mol	Class	qed	logP	SAS	rings	kinase inhibition likeliness
**3048**	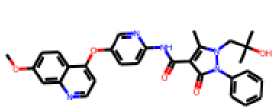	0.409116	COc1ccc2c(=O)cc(CNC(=O)c3c(C)n(C)n(-c4ccccc4)c3=O)nc3cccc2c3c1	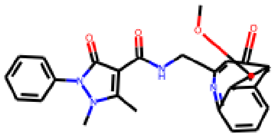	9.0	0.638163	3.995589	2.988471	5	0.774651
**3049**	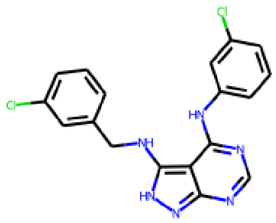	0.409055	Clc1cccc(Nc2ncnc3n[nH]c(N4ccccc(Cl)c4)c23)c1	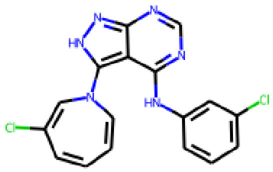	0.0	0.566600	4.200036	5.049812	4	0.795377
**3050**	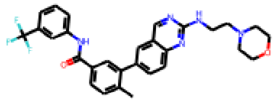	0.408663	Cc1ccc(C(=O)Nc2cccc(C(F)(F)F)c2)cc1-c1ccc2nc(N)ccc2c1	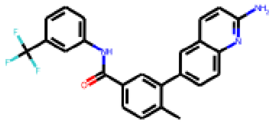	6.0	0.635315	4.542409	3.856266	4	0.833561
**3051**	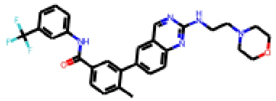	0.408438	Cc1ccc(C(=O)Nc2cccc(C(F)(F)F)c2)cc1-c1ccc2nc(N)ccc2c1	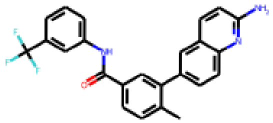	6.0	0.635315	4.542409	3.856266	4	0.833561

	src known mol	similarity	smiles	mol	Class	qed	logP	SAS	rings	kinase inhibition likeliness
**3052**	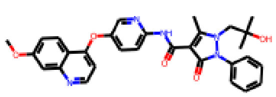	0.408435	COc1ccc2c(=O)cc(CNC(=O)c3c(C)n(C)n(-c4ccccc4)c3=O)nc3cccc2c3c1	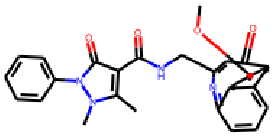	9.0	0.638163	3.995589	2.988471	5	0.774651
**3053**	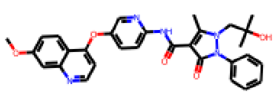	0.408435	COc1ccc2c(=O)cc(CNC(=O)c3c(C)n(C)n(-c4ccccc4)c3=O)nc3cccc2c3c1	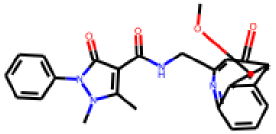	9.0	0.638163	3.995589	2.988471	5	0.774651
**3054**	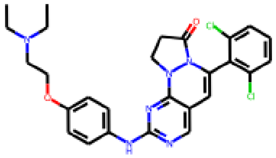	0.408300	COc1cc(Nc2ncc(C)c(-c3ccc4c(n3)NC(=O)C(C)(C)C4)n2)cc(OC)c1OC	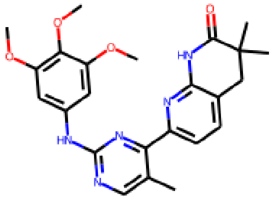	4.0	0.768132	3.608437	2.513170	4	0.757734
**3055**	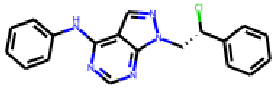	0.408276	Cc1ccc(C(=O)Nc2ccon2)cc1Nc1ncnc2c1cnn2C1cccccc1	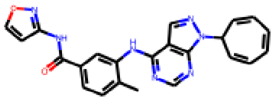	8.0	0.623362	2.525411	2.997881	5	0.770298

	src known mol	similarity	smiles	mol	Class	qed	logP	SAS	rings	kinase inhibition likeliness
**3056**	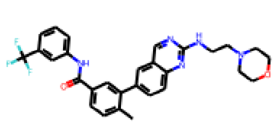	0.408106	Cc1ccc(C(=O)Oc2cccc(C(F)(F)F)c2)cc1-c1ccc2nc(N)ncc2c1	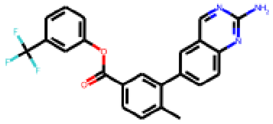	6.0	0.638163	3.995589	2.988471	4	0.789898
**3057**	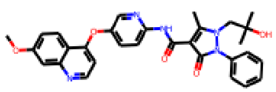	0.408004	COc1ccc2c(=O)cc(CNC(=O)c3c(C)n(C)n(-c4ccccc4)c3=O)nc3cccc2c3c1	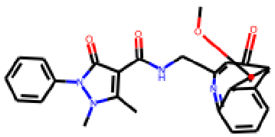	9.0	0.638163	3.995589	2.988471	5	0.774651
**3058**	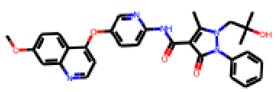	0.408004	COc1ccc2c(=O)cc(CNC(=O)c3c(C)n(C)n(-c4ccccc4)c3=O)nc3cccc2c3c1	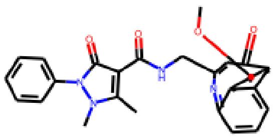	9.0	0.638163	3.995589	2.988471	5	0.774651
**3059**	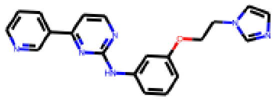	0.407341	Cc1ccc(OC(=O)c2ccccc2)cc1Nc1nccc(-c2cccnc2)n1	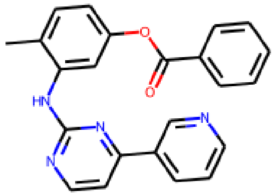	0.0	0.638163	3.995589	2.988471	4	0.775468

	src known mol	similarity	smiles	mol	Class	qed	logP	SAS	rings	kinase inhibition likeliness
**3060**	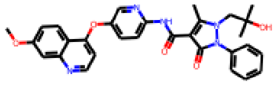	0.406323	COc1ccc2c(=O)cc(CNC(=O)c3c(C)n(C)n(-c4ccccc4)c3=O)nc3cccc3c2n1	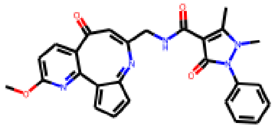	9.0	0.768132	3.608437	2.513170	5	0.823048
**3061**	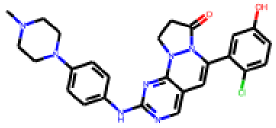	0.406287	O/C(Nc1ccc(Oc2ccccc2)cc1)Oc1ccc2(Cc1)NC(=O)/C(=O)c1ccc(O)n12	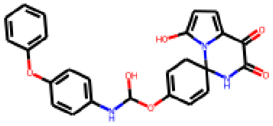	4.0	0.520676	2.336694	2.899742	5	0.820778
**3062**	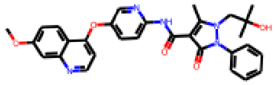	0.406233	COc1ccc2c(=O)cc(CNC(=O)c3c(C)n(C)n(-c4ccccc4)c3=O)nc3cccc2c3c1	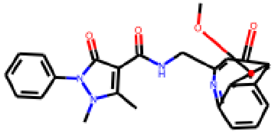	9.0	0.638163	3.995589	2.988471	5	0.774651
**3063**	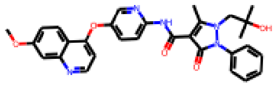	0.405973	COc1ccc2c(=O)cc(CNC(=O)c3c(C)n(C)n(-c4ccccc4)c3=O)nc3cccc2c3c1	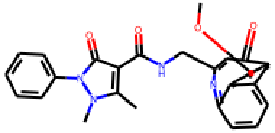	9.0	0.638163	3.995589	2.988471	5	0.774651

	src known mol	similarity	smiles	mol	Class	qed	logP	SAS	rings	kinase inhibition likeliness
**3064**	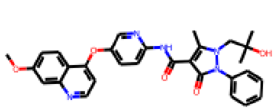	0.405973	COc1ccc2c(=O)cc(CNC(=O)c3c(C)n(C)n(-c4ccccc4)c3=O)nc3cccc2c3c1	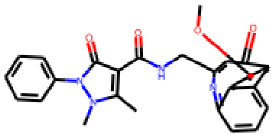	9.0	0.638163	3.995589	2.988471	5	0.774651
**3065**	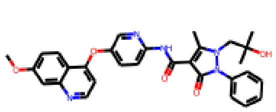	0.405973	COc1ccc2c(=O)cc(CNC(=O)c3c(C)n(C)n(-c4ccccc4)c3=O)nc3cccc2c3c1	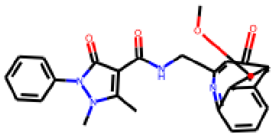	9.0	0.638163	3.995589	2.988471	5	0.774651
**3066**	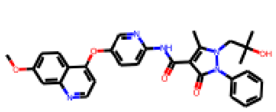	0.405973	COc1ccc2c(=O)cc(CNC(=O)c3c(C)n(C)n(-c4ccccc4)c3=O)nc3cccc2c3c1	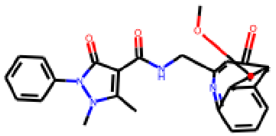	9.0	0.638163	3.995589	2.988471	5	0.774651
**3067**	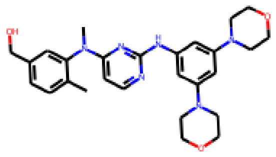	0.405724	NC(=O)c1ccccc1Nc1nc(Nc2ccc(N3CCCC3=O)cc2)ncc1Cl	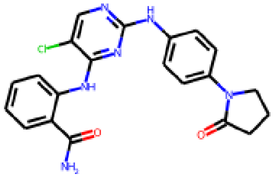	7.0	0.768132	3.608437	2.513170	4	0.773984

	src known mol	similarity	smiles	mol	Class	qed	logP	SAS	rings	kinase inhibition likeliness
**3068**	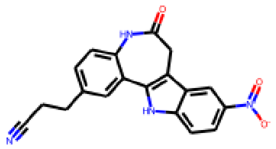	0.405634	CCNC(=O)N1CCC(Nc2n(C(C)c3c(-c4ccccc4)[nH]c4ccccc34)n2)CC1	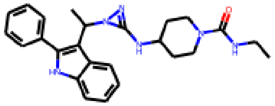	7.0	0.623584	3.603734	4.478009	5	0.804049
**3069**	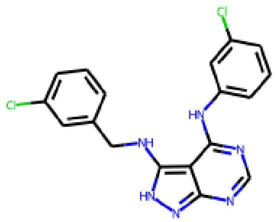	0.405622	Clc1cccc(Nc2ncnc3n[nH]c(N4ccccc(Cl)c4)c23)c1	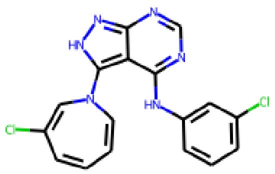	0.0	0.566600	4.200036	5.049812	4	0.795377
**3070**	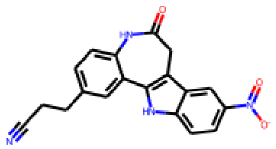	0.404947	CCNC(=O)N1CCC(Nc2n(C(C)c3c(-c4ccccc4)[nH]c4ccccc34)n2)CC1	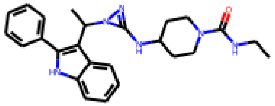	7.0	0.623584	3.603734	4.478009	5	0.804049
**3071**	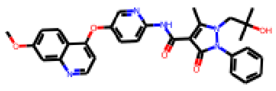	0.404563	COc1ccc2c(=O)cc(CNC(=O)c3c(C)n(C)n(-c4ccccc4)c3=O)nc3cccc2c3c1	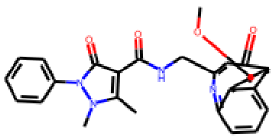	9.0	0.638163	3.995589	2.988471	5	0.774651

	src known mol	similarity	smiles	mol	Class	qed	logP	SAS	rings	kinase inhibition likeliness
**3072**	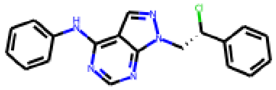	0.404432	Cc1ccc(C(=O)Nc2ccon2)cc1Nc1ncnc2c1cnn2C1cccccc1	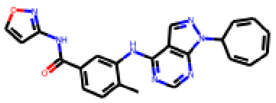	8.0	0.623362	2.525411	2.997881	5	0.770298
**3073**	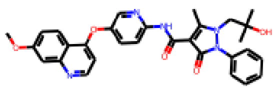	0.403756	COc1ccc2c(=O)cc(CNC(=O)c3c(C)n(C)n(-c4ccccc4)c3=O)nc3cccc3c2n1	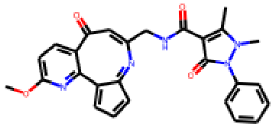	9.0	0.768132	3.608437	2.513170	5	0.823048
**3074**	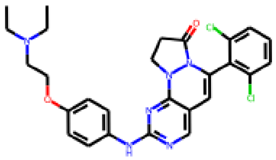	0.403534	COc1cc(Nc2ncc(C)c(-c3ccc4c(n3)NC(=O)C(C)(C)C4)n2)cc(OC)c1OC	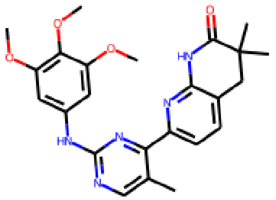	4.0	0.768132	3.608437	2.513170	4	0.757734
**3075**	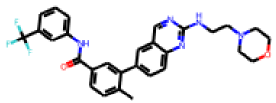	0.403292	Cc1ccc(C(=O)Oc2cccc(C(F)(F)F)c2)cc1-c1ccc2nc(N)ncc2c1	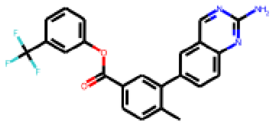	6.0	0.638163	3.995589	2.988471	4	0.789898

	src known mol	similarity	smiles	mol	Class	qed	logP	SAS	rings	kinase inhibition likeliness
**3076**	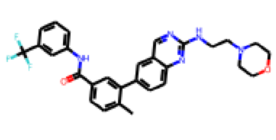	0.401806	Cc1ccc(C(=O)Nc2cccc(C(F)(C)F)c2C)cc1-c1ccc2nc(C)ccccc(F)nc2c1	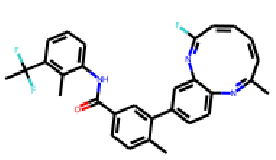	6.0	0.742375	4.384502	0.286234	4	0.779167
**3077**	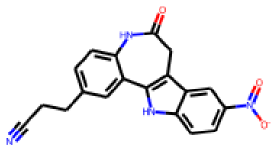	0.401542	CCNC(=O)N1CCC(Nc2n(C(C)c3c(-c4ccccc4)[nH]c4ccccc34)n2)CC1	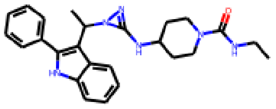	7.0	0.623584	3.603734	4.478009	5	0.804049
**3078**	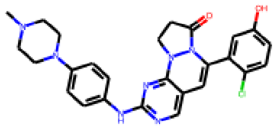	0.401434	O/C(Nc1ccc(Oc2ccccc2)cc1)Oc1ccc2(Cc1)NC(=O)/C(=O)c1ccc(O)n12	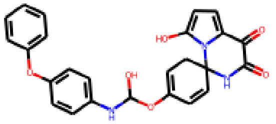	4.0	0.520676	2.336694	2.899742	5	0.820778
**3079**	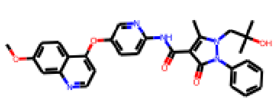	0.401396	COc1ccc2c(=O)cc(CNC(=O)c3c(C)n(C)n(-c4ccccc4)c3=O)nc3cccc3c2n1	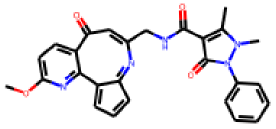	9.0	0.768132	3.608437	2.513170	5	0.823048

	src known mol	similarity	smiles	mol	Class	qed	logP	SAS	rings	kinase inhibition likeliness
**3080**	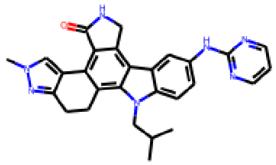	0.401386	C#Cc1ccc(C(=O)OC)cc1-c1C(=O)c-n2nc(-c3ccc(F)cc3F)cc2C1	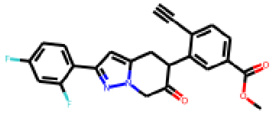	8.0	0.768132	3.608437	2.513170	4	0.763730
**3081**	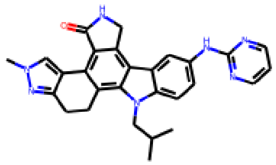	0.401386	C#Cc1ccc(C(=O)OC)cc1-c1C(=O)c-n2nc(-c3ccc(F)cc3F)cc2C1	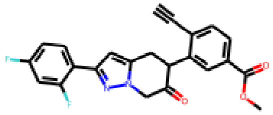	8.0	0.768132	3.608437	2.513170	4	0.763730
**3082**	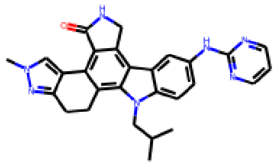	0.401386	C#Cc1ccc(C(=O)OC)cc1-c1C(=O)c-n2nc(-c3ccc(F)cc3F)cc2C1	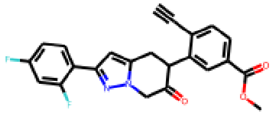	8.0	0.768132	3.608437	2.513170	4	0.763730
**3083**	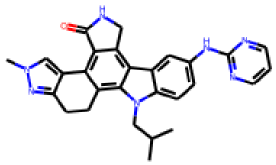	0.401386	C#Cc1ccc(C(=O)OC)cc1-c1C(=O)c-n2nc(-c3ccc(F)cc3F)cc2C1	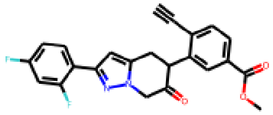	8.0	0.768132	3.608437	2.513170	4	0.763730

	src known mol	similarity	smiles	mol	Class	qed	logP	SAS	rings	kinase inhibition likeliness
**3084**	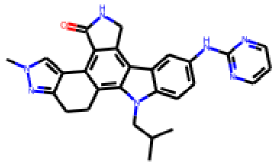	0.401386	C#Cc1ccc(C(=O)OC)cc1-c1C(=O)c-n2nc(-c3ccc(F)cc3F)cc2C1	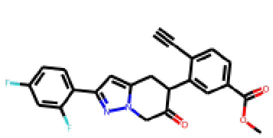	8.0	0.768132	3.608437	2.513170	4	0.763730
**3085**	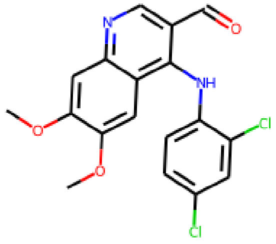	0.401053	COc1cc2ncc(C(=O)O)c(Nc3cccc(Cl)c3Cl)c2cc1OC1C(C)C1	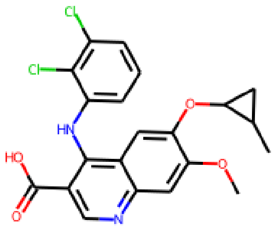	2.0	0.623362	2.525411	2.997881	4	0.774857
**3086**	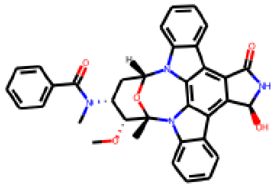	0.400768	COc1nccc(-c2cs/cc2-c2c(CC(=O)Nc3cccc(C(C)(C)C)c3)cc2F)n1	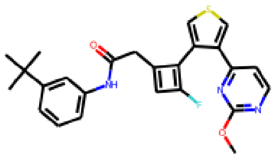	6.0	0.638163	3.995589	2.988471	4	0.831350
**3087**	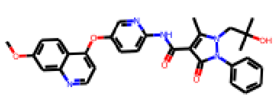	0.400705	COc1ccc2c(=O)cc(CNC(=O)c3c(C)n(C)n(-c4ccccc4)c3=O)nc3cccc3c2n1	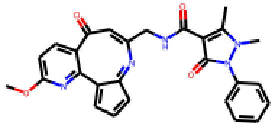	9.0	0.768132	3.608437	2.513170	5	0.823048

	src known mol	similarity	smiles	mol	Class	qed	logP	SAS	rings	kinase inhibition likeliness
**3088**	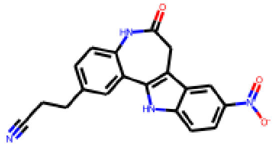	0.400566	CCNC(=O)N1CCC(Nc2n(C(C)c3c(-c4ccccc4)[nH]c4ccccc34)n2)CC1	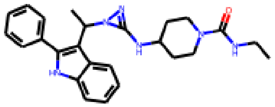	7.0	0.623584	3.603734	4.478009	5	0.804049
**3089**	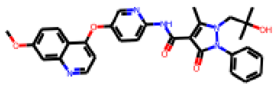	0.400471	COc1ccc2c(=O)cc(CNC(=O)c3c(C)n(C)n(-c4ccccc4)c3=O)nc3cccc3c2n1	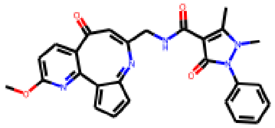	9.0	0.768132	3.608437	2.513170	5	0.823048
**3090**	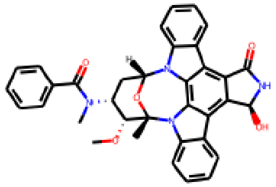	0.400126	COc1nccc(-c2cs/cc2-c2c(CC(=O)Nc3cccc(C(C)(C)C)c3)cc2F)n1	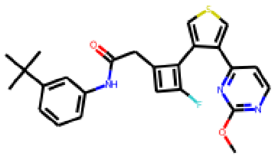	6.0	0.638163	3.995589	2.988471	4	0.831350
**3091**	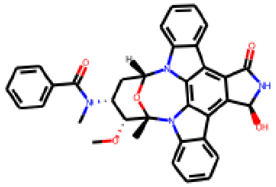	0.400126	COc1nccc(-c2cs/cc2-c2c(CC(=O)Nc3cccc(C(C)(C)C)c3)cc2F)n1	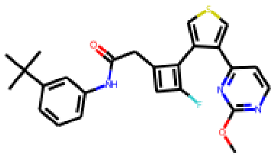	6.0	0.638163	3.995589	2.988471	4	0.831350

	src known mol	similarity	smiles	mol	Class	qed	logP	SAS	rings	kinase inhibition likeliness
**3092**	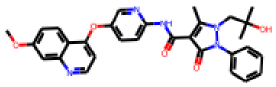	0.399889	COc1ccc2c(=O)cc(CNC(=O)c3c(C)n(C)n(-c4ccccc4)c3=O)nc3cccc2c3c1	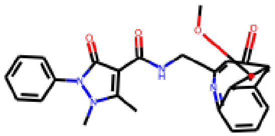	9.0	0.638163	3.995589	2.988471	5	0.774651
**3093**	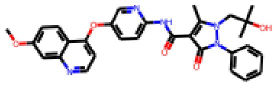	0.399555	COc1ccc2c(=O)cc(CNC(=O)c3c(C)n(C)n(-c4ccccc4)c3=O)nc3cccc2c3c1	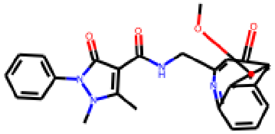	9.0	0.638163	3.995589	2.988471	5	0.774651
**3094**	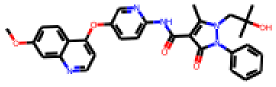	0.399223	COc1ccc2c(=O)cc(CNC(=O)c3c(C)n(C)n(-c4ccccc4)c3=O)nc3cccc2c3c1	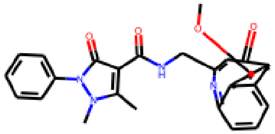	9.0	0.638163	3.995589	2.988471	5	0.774651
**3095**	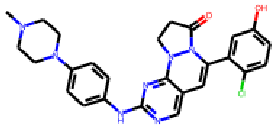	0.399053	O/C(Nc1ccc(Oc2ccccc2)cc1)Oc1ccc2(Cc1)NC(=O)/C(=O)c1ccc(O)n12	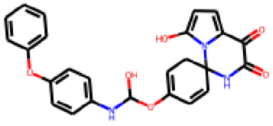	4.0	0.520676	2.336694	2.899742	5	0.820778

	src known mol	similarity	smiles	mol	Class	qed	logP	SAS	rings	kinase inhibition likeliness
**3096**	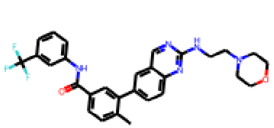	0.398944	Cc1ccc(C(=O)Nc2cccc(C(F)(C)F)c2C)cc1-c1ccc2nc(C)ccccc(F)nc2c1	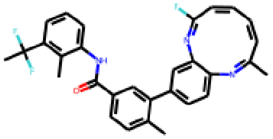	6.0	0.742375	4.384502	0.286234	4	0.779167
**3097**	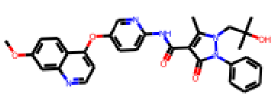	0.398923	COc1ccc2c(=O)cc(CNC(=O)c3c(C)n(C)n(-c4ccccc4)c3=O)nc3cccc3c2n1	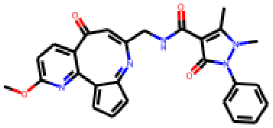	9.0	0.768132	3.608437	2.513170	5	0.823048
**3098**	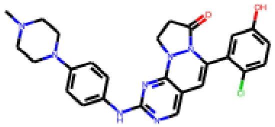	0.398810	O/C(Nc1ccc(Oc2ccccc2)cc1)Oc1ccc2(Cc1)NC(=O)/C(=O)c1ccc(O)n12	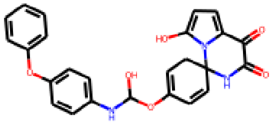	4.0	0.520676	2.336694	2.899742	5	0.820778
**3099**	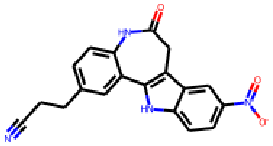	0.398725	CCNC(=O)N1CCC(Nc2n(C(C)c3c(-c4ccccc4)[nH]c4ccccc34)n2)CC1	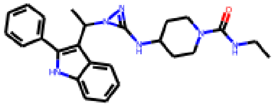	7.0	0.623584	3.603734	4.478009	5	0.804049

	src known mol	similarity	smiles	mol	Class	qed	logP	SAS	rings	kinase inhibition likeliness
**3100**	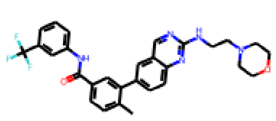	0.398687	Cc1ccc(C(=O)Nc2cccc(C(F)(F)F)c2)cc1-c1ccc2nc(N)ccc2c1	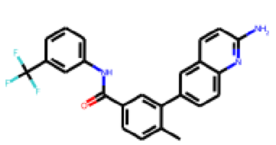	6.0	0.635315	4.542409	3.856266	4	0.833561
**3101**	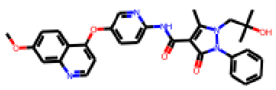	0.397772	COc1ccc2c(=O)cc(CNC(=O)c3c(C)n(C)n(-c4ccccc4)c3=O)nc3cccc2c3c1	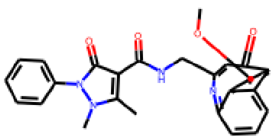	9.0	0.638163	3.995589	2.988471	5	0.774651
**3102**	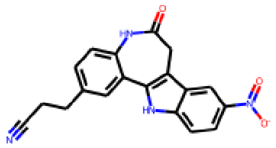	0.397719	CCNC(=O)N1CCC(Nc2n(C(C)c3c(-c4ccccc4)[nH]c4ccccc34)n2)CC1	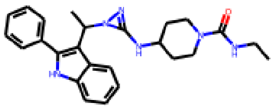	7.0	0.623584	3.603734	4.478009	5	0.804049
**3103**	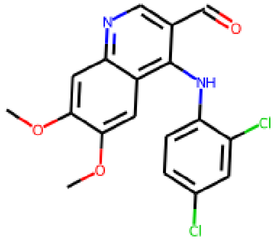	0.397502	COc1cc2ncc(C(=O)O)c(Nc3cccc(Cl)c3Cl)c2cc1OC1C(C)C1	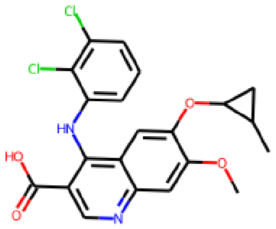	2.0	0.623362	2.525411	2.997881	4	0.774857

	src known mol	similarity	smiles	mol	Class	qed	logP	SAS	rings	kinase inhibition likeliness
**3104**	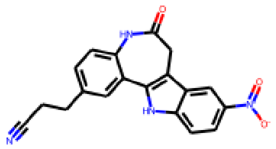	0.396904	CCNC(=O)N1CCC(Nc2n(C(C)c3c(-c4ccccc4)[nH]c4ccccc34)n2)CC1	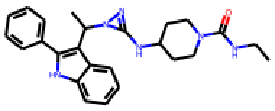	7.0	0.623584	3.603734	4.478009	5	0.804049
**3105**	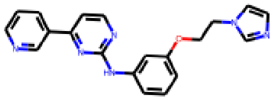	0.396679	Cc1ccc(OC(=O)c2ccccc2)cc1Nc1nccc(-c2cccnc2)n1	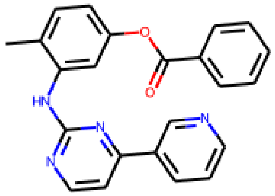	0.0	0.638163	3.995589	2.988471	4	0.775468
**3106**	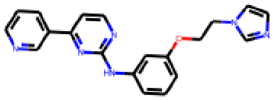	0.396484	Cc1ccc(OC(=O)c2ccccc2)cc1Nc1nccc(-c2cccnc2)n1	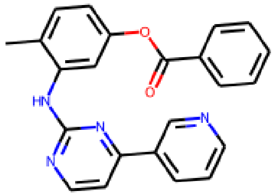	0.0	0.638163	3.995589	2.988471	4	0.775468
**3107**	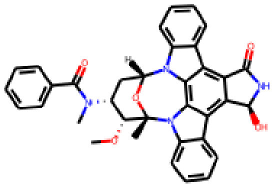	0.396457	COc1cc2c(=O)cc(CNC(=O)c3nn(-c4ccccc4C(C)(F)F)o3)c2o1	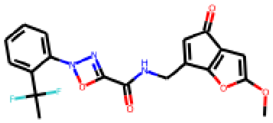	9.0	0.576217	4.086599	3.569904	4	0.783532

	src known mol	similarity	smiles	mol	Class	qed	logP	SAS	rings	kinase inhibition likeliness
**3108**	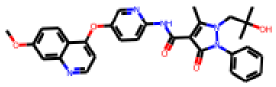	0.396331	COc1ccc2c(=O)cc(CNC(=O)c3c(C)n(C)n(-c4ccccc4)c3=O)nc3cccc2c3c1	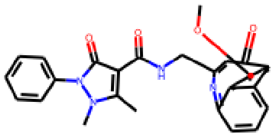	9.0	0.638163	3.995589	2.988471	5	0.774651
**3109**	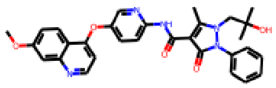	0.396029	COc1ccc2c(=O)cc(CNC(=O)c3c(C)n(C)n(-c4ccccc4)c3=O)nc3cccc2c3c1	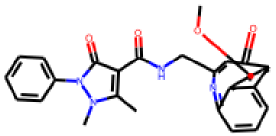	9.0	0.638163	3.995589	2.988471	5	0.774651
**3110**	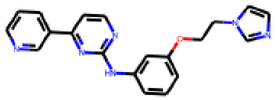	0.395934	Cc1ccc(OC(=O)c2ccccc2)cc1Nc1nccc(-c2cccnc2)n1	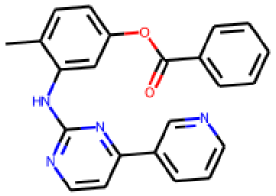	0.0	0.638163	3.995589	2.988471	4	0.775468
**3111**	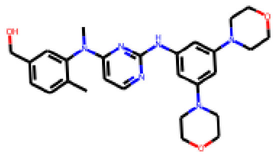	0.395853	NC(=O)c1ccccc1Nc1nc(Nc2ccc(N3CCCC3=O)cc2)ncc1Cl	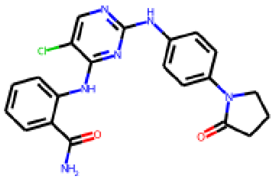	7.0	0.768132	3.608437	2.513170	4	0.773984

	src known mol	similarity	smiles	mol	Class	qed	logP	SAS	rings	kinase inhibition likeliness
**3112**	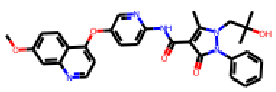	0.394781	COc1ccc2c(=O)cc(CNC(=O)c3c(C)n(C)n(-c4ccccc4)c3=O)nc3cccc2c3c1	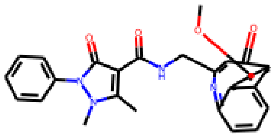	9.0	0.638163	3.995589	2.988471	5	0.774651
**3113**	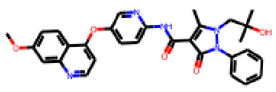	0.394781	COc1ccc2c(=O)cc(CNC(=O)c3c(C)n(C)n(-c4ccccc4)c3=O)nc3cccc2c3c1	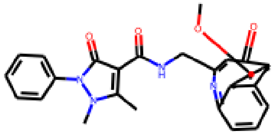	9.0	0.638163	3.995589	2.988471	5	0.774651
**3114**	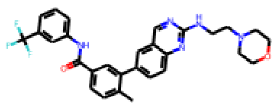	0.394654	Cc1ccc(C(=O)Nc2cccc(C(F)(C)F)c2C)cc1-c1ccc2nc(C)ccccc(F)nc2c1	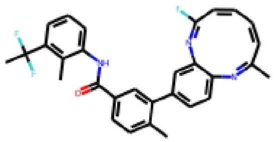	6.0	0.742375	4.384502	0.286234	4	0.779167
**3115**	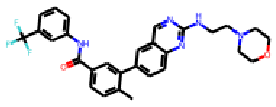	0.394528	Cc1ccc(C(=O)Oc2cccc(C(F)(F)F)c2)cc1-c1ccc2nc(N)ncc2c1	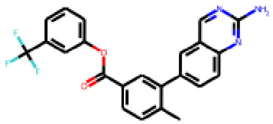	6.0	0.638163	3.995589	2.988471	4	0.789898

	src known mol	similarity	smiles	mol	Class	qed	logP	SAS	rings	kinase inhibition likeliness
**3116**	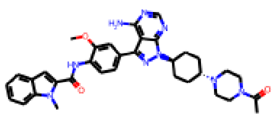	0.394298	Cc1ccc(C(=O)Nc2cccc(C(F)(F)F)c2C)cc1-c1ccc2oc(=O)cccc(F)c2n1	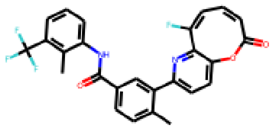	6.0	0.790896	3.985943	2.115897	4	0.783675
**3117**	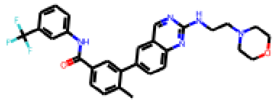	0.394167	Cc1ccc(C(=O)Nc2cccc(C(F)(F)F)c2)cc1-c1ccc2cc(N)ncc2c1	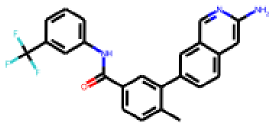	6.0	0.761557	4.954058	3.852993	4	0.750321
**3118**	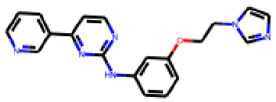	0.393967	Cc1ccc(OC(=O)c2ccccc2)cc1Nc1nccc(-c2cccnc2)n1	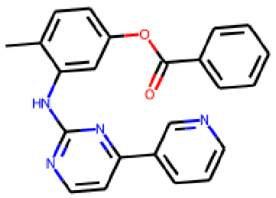	0.0	0.638163	3.995589	2.988471	4	0.775468
**3119**	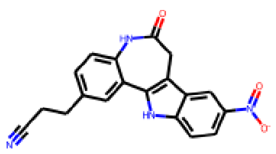	0.393879	CCNC(=O)N1CCC(Nc2n(C(C)c3c(-c4ccccc4)[nH]c4ccccc34)n2)CC1	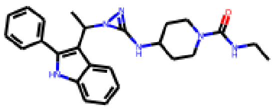	7.0	0.623584	3.603734	4.478009	5	0.804049

	src known mol	similarity	smiles	mol	Class	qed	logP	SAS	rings	kinase inhibition likeliness
**3120**	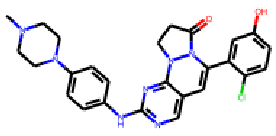	0.393868	O/C(Nc1ccc(Oc2ccccc2)cc1)Oc1ccc2(Cc1)NC(=O)/C(=O)c1ccc(O)n12	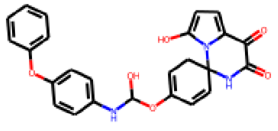	4.0	0.520676	2.336694	2.899742	5	0.820778
**3121**	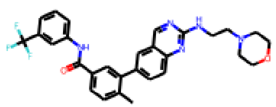	0.392573	Cc1ccc(C(=O)Nc2cccc(C(F)(F)F)c2)cc1-c1ccc2cc(N)ncc2c1	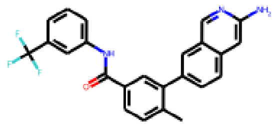	6.0	0.761557	4.954058	3.852993	4	0.750321
**3122**	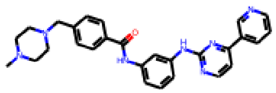	0.392483	Fc1ccc(NC(=O)c2ccccc2)cc1Nc1nccc(-c2cccnc2)n1	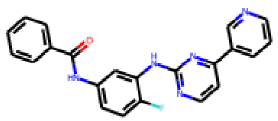	0.0	0.632663	4.308233	4.065316	4	0.777346
**3123**	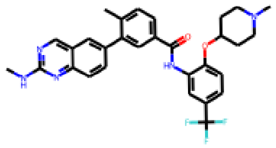	0.391993	Cc1ccc(C(=O)Nc2cccc(C(C)(C)F)c2C)cc1-c1ccc2oc(O)ccccc(F)c2c1	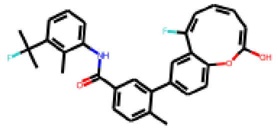	6.0	0.600657	4.044744	5.260394	4	0.788333

	src known mol	similarity	smiles	mol	Class	qed	logP	SAS	rings	kinase inhibition likeliness
**3124**	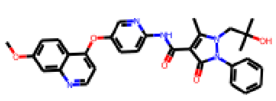	0.391939	COc1ccc2c(=O)cc(CNC(=O)c3c(C)n(C)n(-c4ccccc4)c3=O)nc3cccc3c2n1	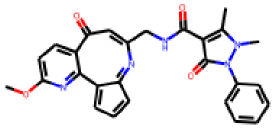	9.0	0.768132	3.608437	2.513170	5	0.823048
**3125**	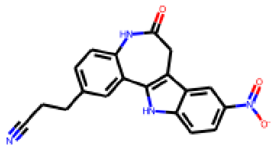	0.391892	CCNC(=O)N1CCC(Nc2n(C(C)c3c(-c4ccccc4)[nH]c4ccccc34)n2)CC1	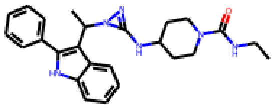	7.0	0.623584	3.603734	4.478009	5	0.804049
**3126**	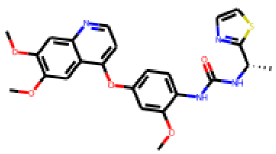	0.391546	COc1ccccc1NC(=O)Nc1ccc(Oc2ccnc3cc(OC)c(OC)cc23)cc1	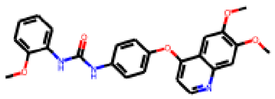	5.0	0.638163	3.995589	2.988471	4	0.789556
**3127**	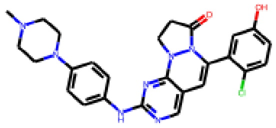	0.391538	O/C(Nc1ccc(Oc2ccccc2)cc1)Oc1ccc2(Cc1)NC(=O)/C(=O)c1ccc(O)n12	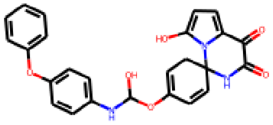	4.0	0.520676	2.336694	2.899742	5	0.820778

	src known mol	similarity	smiles	mol	Class	qed	logP	SAS	rings	kinase inhibition likeliness
**3128**	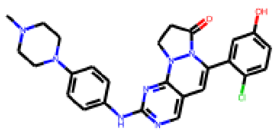	0.391538	O/C(Nc1ccc(Oc2ccccc2)cc1)Oc1ccc2(Cc1)NC(=O)/C(=O)c1ccc(O)n12	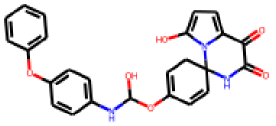	4.0	0.520676	2.336694	2.899742	5	0.820778
**3129**	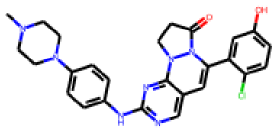	0.391538	O/C(Nc1ccc(Oc2ccccc2)cc1)Oc1ccc2(Cc1)NC(=O)/C(=O)c1ccc(O)n12	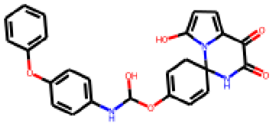	4.0	0.520676	2.336694	2.899742	5	0.820778
**3130**	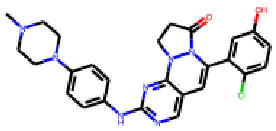	0.391538	O/C(Nc1ccc(Oc2ccccc2)cc1)Oc1ccc2(Cc1)NC(=O)/C(=O)c1ccc(O)n12	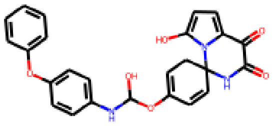	4.0	0.520676	2.336694	2.899742	5	0.820778
**3131**	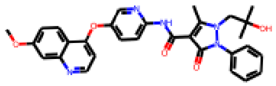	0.390258	COc1ccc2c(=O)cc(CNC(=O)c3c(C)n(C)n(-c4ccccc4)c3=O)nc3cccc2c3c1	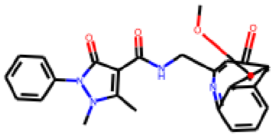	9.0	0.638163	3.995589	2.988471	5	0.774651

	src known mol	similarity	smiles	mol	Class	qed	logP	SAS	rings	kinase inhibition likeliness
**3132**	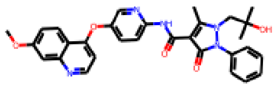	0.390258	COc1ccc2c(=O)cc(CNC(=O)c3c(C)n(C)n(-c4ccccc4)c3=O)nc3cccc2c3c1	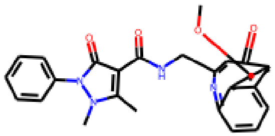	9.0	0.638163	3.995589	2.988471	5	0.774651
**3133**	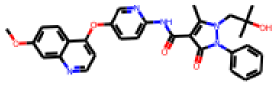	0.390158	COc1ccc2c(=O)cc(CNC(=O)c3c(C)n(C)n(-c4ccccc4)c3=O)nc3cccc3c2n1	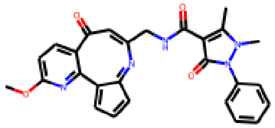	9.0	0.768132	3.608437	2.513170	5	0.823048
**3134**	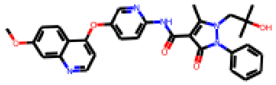	0.389945	COc1ccc2c(=O)cc(CNC(=O)c3c(C)n(C)n(-c4ccccc4)c3=O)nc3cccc3c2n1	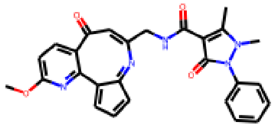	9.0	0.768132	3.608437	2.513170	5	0.823048
**3135**	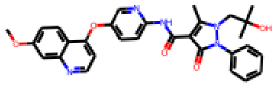	0.389538	COc1ccc2c(=O)cc(CNC(=O)c3c(C)n(C)n(-c4ccccc4)c3=O)nc3cccc2c3c1	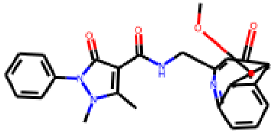	9.0	0.638163	3.995589	2.988471	5	0.774651

	src known mol	similarity	smiles	mol	Class	qed	logP	SAS	rings	kinase inhibition likeliness
**3136**	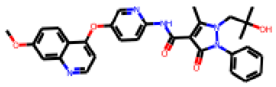	0.389538	COc1ccc2c(=O)cc(CNC(=O)c3c(C)n(C)n(-c4ccccc4)c3=O)nc3cccc2c3c1	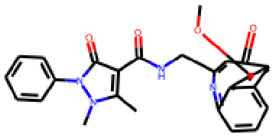	9.0	0.638163	3.995589	2.988471	5	0.774651
**3137**	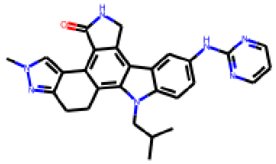	0.389454	C#Cc1ccc(C(=O)OC)cc1-c1C(=O)c-n2nc(-c3ccc(F)cc3F)cc2C1	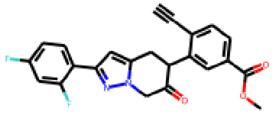	8.0	0.768132	3.608437	2.513170	4	0.763730
**3138**	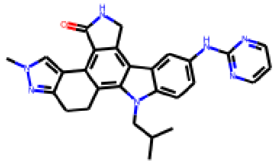	0.389454	C#Cc1ccc(C(=O)OC)cc1-c1C(=O)c-n2nc(-c3ccc(F)cc3F)cc2C1	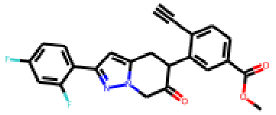	8.0	0.768132	3.608437	2.513170	4	0.763730
**3139**	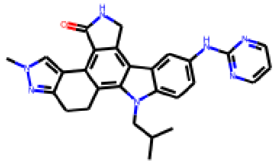	0.389454	C#Cc1ccc(C(=O)OC)cc1-c1C(=O)c-n2nc(-c3ccc(F)cc3F)cc2C1	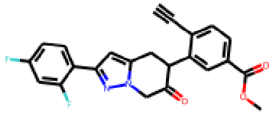	8.0	0.768132	3.608437	2.513170	4	0.763730

	src known mol	similarity	smiles	mol	Class	qed	logP	SAS	rings	kinase inhibition likeliness
**3140**	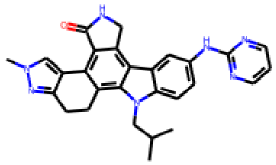	0.389454	C#Cc1ccc(C(=O)OC)cc1-c1C(=O)c-n2nc(-c3ccc(F)cc3F)cc2C1	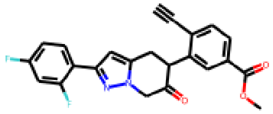	8.0	0.768132	3.608437	2.513170	4	0.763730
**3141**	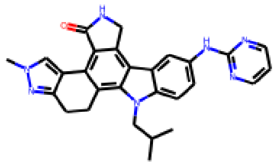	0.389454	C#Cc1ccc(C(=O)OC)cc1-c1C(=O)c-n2nc(-c3ccc(F)cc3F)cc2C1	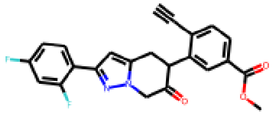	8.0	0.768132	3.608437	2.513170	4	0.763730
**3142**	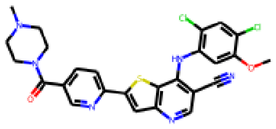	0.389293	CNC(=O)c1ccccc1Nc1cnc(-c2sc(N3CCOCC3)cc2C#N)cc1Cl	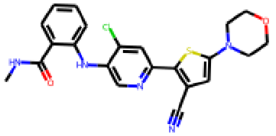	7.0	0.635315	4.542409	3.856266	4	0.763112
**3143**	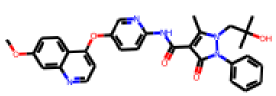	0.389077	COc1ccc2c(=O)cc(CNC(=O)c3c(C)n(C)n(-c4ccccc4)c3=O)nc3cccc2c3c1	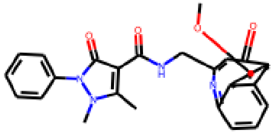	9.0	0.638163	3.995589	2.988471	5	0.774651

	src known mol	similarity	smiles	mol	Class	qed	logP	SAS	rings	kinase inhibition likeliness
**3144**	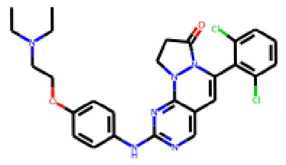	0.388968	COc1cc(Nc2ncc(C)c(-c3ccc4c(n3)NC(=O)C(C)(C)C4)n2)cc(OC)c1OC	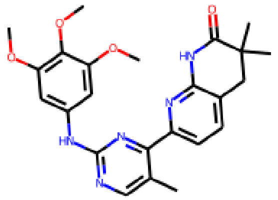	4.0	0.768132	3.608437	2.513170	4	0.757734
**3145**	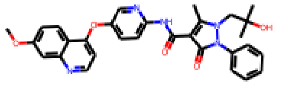	0.388889	COc1ccc2c(=O)cc(CNC(=O)c3c(C)n(C)n(-c4ccccc4)c3=O)nc3cccc2c3c1	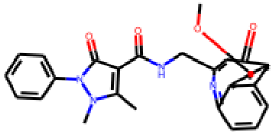	9.0	0.638163	3.995589	2.988471	5	0.774651
**3146**	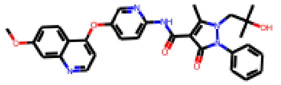	0.388393	COc1ccc2c(=O)cc(CNC(=O)c3c(C)n(C)n(-c4ccccc4)c3=O)nc3cccc2c3c1	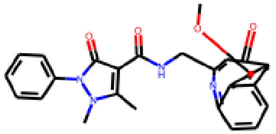	9.0	0.638163	3.995589	2.988471	5	0.774651
**3147**	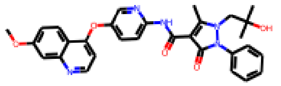	0.388393	COc1ccc2c(=O)cc(CNC(=O)c3c(C)n(C)n(-c4ccccc4)c3=O)nc3cccc2c3c1	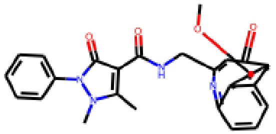	9.0	0.638163	3.995589	2.988471	5	0.774651

	src known mol	similarity	smiles	mol	Class	qed	logP	SAS	rings	kinase inhibition likeliness
**3148**	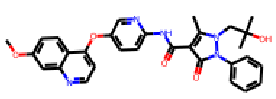	0.388393	COc1ccc2c(=O)cc(CNC(=O)c3c(C)n(C)n(-c4ccccc4)c3=O)nc3cccc2c3c1	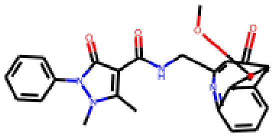	9.0	0.638163	3.995589	2.988471	5	0.774651
**3149**	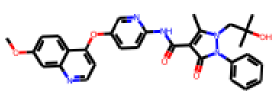	0.388393	COc1ccc2c(=O)cc(CNC(=O)c3c(C)n(C)n(-c4ccccc4)c3=O)nc3cccc2c3c1	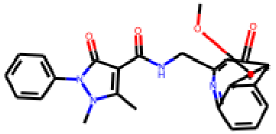	9.0	0.638163	3.995589	2.988471	5	0.774651
**3150**	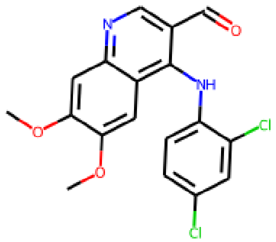	0.388060	COc1cc2ncc(C(=O)O)c(Nc3cccc(Cl)c3Cl)c2cc1OC1C(C)C1	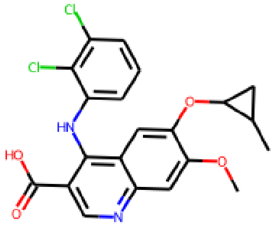	2.0	0.623362	2.525411	2.997881	4	0.774857
**3151**	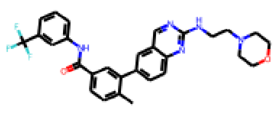	0.387774	Cc1ccc(C(=O)Nc2cccc(C(F)(F)F)c2C)cc1-c1ccc2cc(N)ncc2c1	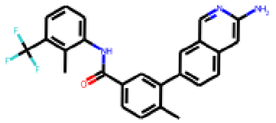	6.0	0.771867	2.951597	1.935530	4	0.754945

	src known mol	similarity	smiles	mol	Class	qed	logP	SAS	rings	kinase inhibition likeliness
**3152**	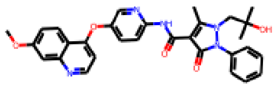	0.386882	COc1ccc2c(=O)cc(CNC(=O)c3c(C)n(C)n(-c4ccccc4)c3=O)nc3cccc2c3c1	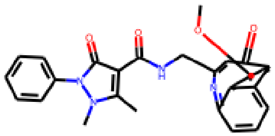	9.0	0.638163	3.995589	2.988471	5	0.774651
**3153**	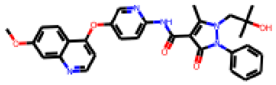	0.386565	COc1ccc2c(=O)cc(CNC(=O)c3c(C)n(C)n(-c4ccccc4)c3=O)nc3cccc3c2n1	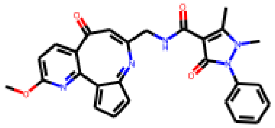	9.0	0.768132	3.608437	2.513170	5	0.823048
**3154**	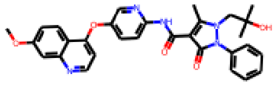	0.386162	COc1ccc2c(=O)cc(CNC(=O)c3c(C)n(C)n(-c4ccccc4)c3=O)nc3cccc3c2n1	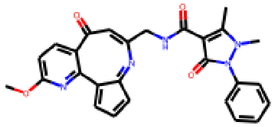	9.0	0.768132	3.608437	2.513170	5	0.823048
**3155**	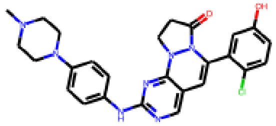	0.386121	O/C(Nc1ccc(Oc2ccccc2)cc1)Oc1ccc2(Cc1)NC(=O)/C(=O)c1ccc(O)n12	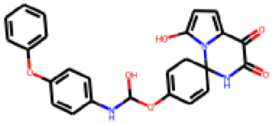	4.0	0.520676	2.336694	2.899742	5	0.820778

	src known mol	similarity	smiles	mol	Class	qed	logP	SAS	rings	kinase inhibition likeliness
**3156**	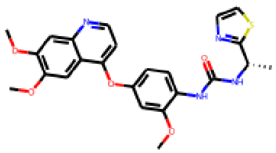	0.386119	COc1ccccc1NC(=O)Nc1ccc(Oc2ccnc3cc(OC)c(OC)cc23)cc1	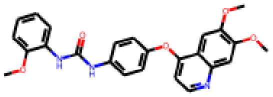	5.0	0.638163	3.995589	2.988471	4	0.789556
**3157**	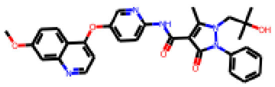	0.385831	COc1ccc2c(=O)cc(CNC(=O)c3c(C)n(C)n(-c4ccccc4)c3=O)nc3cccc3c2n1	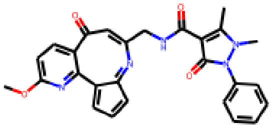	9.0	0.768132	3.608437	2.513170	5	0.823048
**3158**	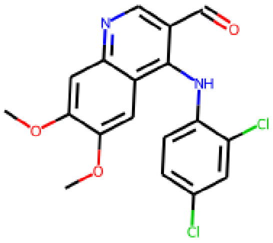	0.385640	COc1cc2ncc(C(=O)O)c(Nc3cccc(Cl)c3Cl)c2cc1OC1C(C)C1	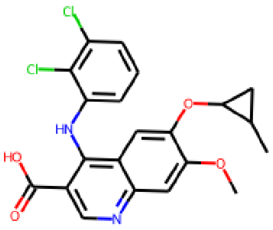	2.0	0.623362	2.525411	2.997881	4	0.774857
**3159**	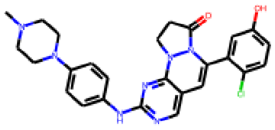	0.385629	O/C(Nc1ccc(Oc2ccccc2)cc1)Oc1ccc2(Cc1)NC(=O)/C(=O)c1ccc(O)n12	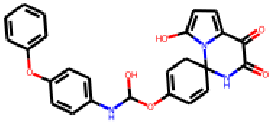	4.0	0.520676	2.336694	2.899742	5	0.820778

	src known mol	similarity	smiles	mol	Class	qed	logP	SAS	rings	kinase inhibition likeliness
**3160**	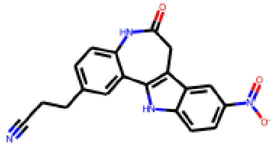	0.385445	CCNC(=O)N1CCC(Nc2n(C(C)c3c(-c4ccccc4)[nH]c4ccccc34)n2)CC1	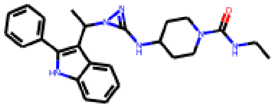	7.0	0.623584	3.603734	4.478009	5	0.804049
**3161**	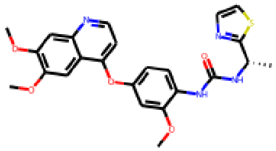	0.384685	COc1ccccc1NC(=O)Nc1ccc(Oc2ccnc3cc(OC)c(OC)cc23)cc1	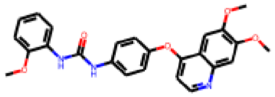	5.0	0.638163	3.995589	2.988471	4	0.789556
**3162**	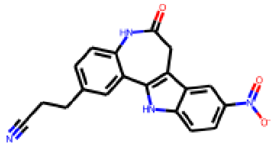	0.383609	CCNC(=O)N1CCC(Nc2n(C(C)c3c(-c4ccccc4)[nH]c4ccccc34)n2)CC1	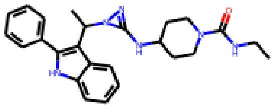	7.0	0.623584	3.603734	4.478009	5	0.804049
**3163**	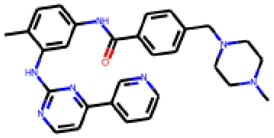	0.383577	Cc1ccc(NC(=O)c2ccccc2)cc1Nc1nccc(-c2ccncc2)n1	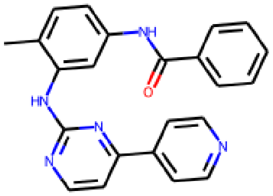	0.0	0.593231	3.250153	2.680275	4	0.858012

	src known mol	similarity	smiles	mol	Class	qed	logP	SAS	rings	kinase inhibition likeliness
**3164**	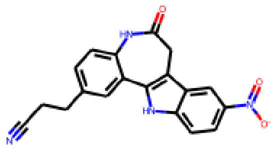	0.382950	CCNC(=O)N1CCC(Nc2n(C(C)c3c(-c4ccccc4)[nH]c4ccccc34)n2)CC1	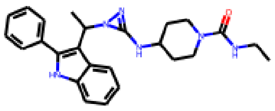	7.0	0.623584	3.603734	4.478009	5	0.804049
**3165**	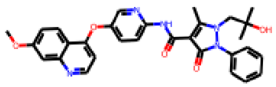	0.382550	COc1ccc2c(=O)cc(CNC(=O)c3c(C)n(C)n(-c4ccccc4)c3=O)nc3cccc2c3c1	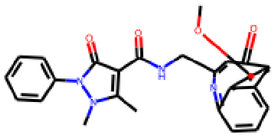	9.0	0.638163	3.995589	2.988471	5	0.774651
**3166**	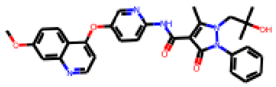	0.382123	COc1ccc2c(=O)cc(CNC(=O)c3c(C)n(C)n(-c4ccccc4)c3=O)nc3cccc2c3c1	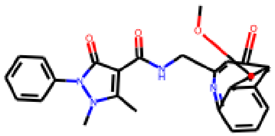	9.0	0.638163	3.995589	2.988471	5	0.774651
**3167**	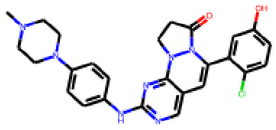	0.381123	O/C(Nc1ccc(Oc2ccccc2)cc1)Oc1ccc2(Cc1)NC(=O)/C(=O)c1ccc(O)n12	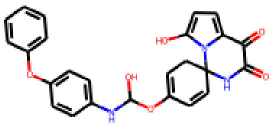	4.0	0.520676	2.336694	2.899742	5	0.820778

	src known mol	similarity	smiles	mol	Class	qed	logP	SAS	rings	kinase inhibition likeliness
**3168**	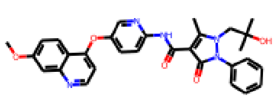	0.381092	COc1ccc2c(=O)cc(CNC(=O)c3c(C)n(C)n(-c4ccccc4)c3=O)nc3cccc3c2n1	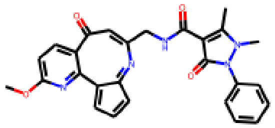	9.0	0.768132	3.608437	2.513170	5	0.823048
**3169**	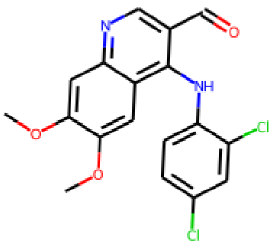	0.380631	COc1cc2ncc(C(=O)O)c(Nc3cccc(Cl)c3Cl)c2cc1OC1C(C)C1	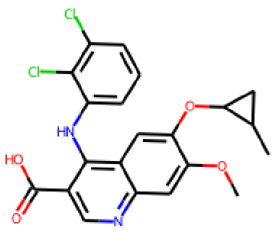	2.0	0.623362	2.525411	2.997881	4	0.774857
**3170**	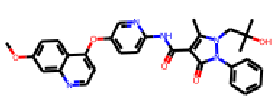	0.380557	COc1ccc2c(=O)cc(CNC(=O)c3c(C)n(C)n(-c4ccccc4)c3=O)nc3cccc3c2n1	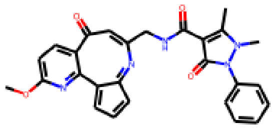	9.0	0.768132	3.608437	2.513170	5	0.823048
**3171**	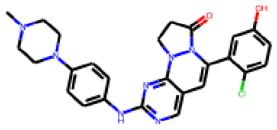	0.380096	O/C(Nc1ccc(Oc2ccccc2)cc1)Oc1ccc2(Cc1)NC(=O)/C(=O)c1ccc(O)n12	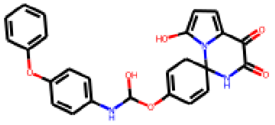	4.0	0.520676	2.336694	2.899742	5	0.820778

	src known mol	similarity	smiles	mol	Class	qed	logP	SAS	rings	kinase inhibition likeliness
**3172**	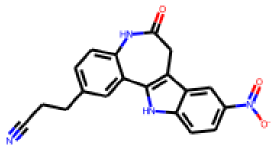	0.379260	CCNC(=O)N1CCC(Nc2n(C(C)c3c(-c4ccccc4)[nH]c4ccccc34)n2)CC1	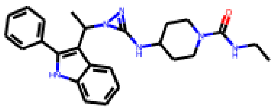	7.0	0.623584	3.603734	4.478009	5	0.804049
**3173**	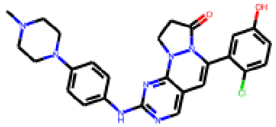	0.379249	O/C(Nc1ccc(Oc2ccccc2)cc1)Oc1ccc2(Cc1)NC(=O)/C(=O)c1ccc(O)n12	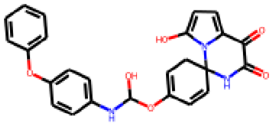	4.0	0.520676	2.336694	2.899742	5	0.820778
**3174**	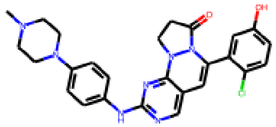	0.379249	O/C(Nc1ccc(Oc2ccccc2)cc1)Oc1ccc2(Cc1)NC(=O)/C(=O)c1ccc(O)n12	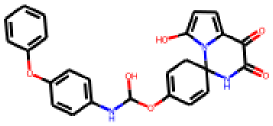	4.0	0.520676	2.336694	2.899742	5	0.820778
**3175**	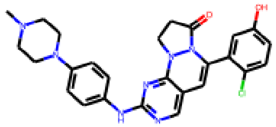	0.379249	O/C(Nc1ccc(Oc2ccccc2)cc1)Oc1ccc2(Cc1)NC(=O)/C(=O)c1ccc(O)n12	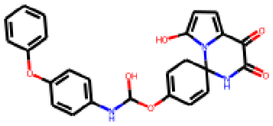	4.0	0.520676	2.336694	2.899742	5	0.820778

	src known mol	similarity	smiles	mol	Class	qed	logP	SAS	rings	kinase inhibition likeliness
**3176**	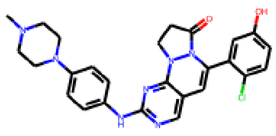	0.379249	O/C(Nc1ccc(Oc2ccccc2)cc1)Oc1ccc2(Cc1)NC(=O)/C(=O)c1ccc(O)n12	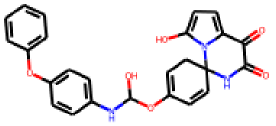	4.0	0.520676	2.336694	2.899742	5	0.820778
**3177**	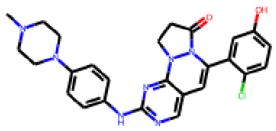	0.379249	O/C(Nc1ccc(Oc2ccccc2)cc1)Oc1ccc2(Cc1)NC(=O)/C(=O)c1ccc(O)n12	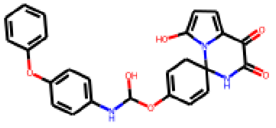	4.0	0.520676	2.336694	2.899742	5	0.820778
**3178**	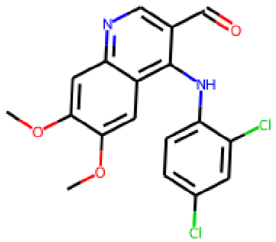	0.378834	COc1cc2ncc(C(=O)O)c(Nc3cccc(Cl)c3Cl)c2cc1OC1C(C)C1	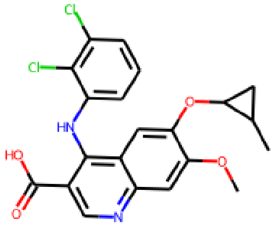	2.0	0.623362	2.525411	2.997881	4	0.774857
**3179**	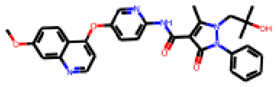	0.378788	COc1ccc2c(=O)cc(CNC(=O)c3c(C)n(C)n(-c4ccccc4)c3=O)nc3cccc2c3c1	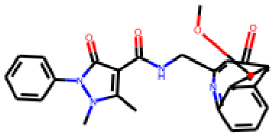	9.0	0.638163	3.995589	2.988471	5	0.774651

	src known mol	similarity	smiles	mol	Class	qed	logP	SAS	rings	kinase inhibition likeliness
**3180**	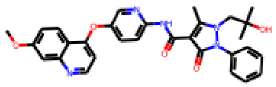	0.378156	COc1ccc2c(=O)cc(CNC(=O)c3c(C)n(C)n(-c4ccccc4)c3=O)nc3cccc3c2n1	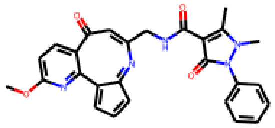	9.0	0.768132	3.608437	2.513170	5	0.823048
**3181**	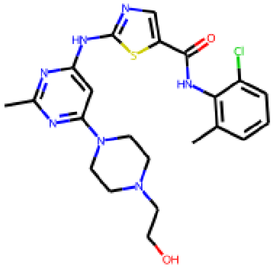	0.377627	Cc1nc(Nc2ncc(C(=O)Oc3c(C)cccc3Cl)s2)cc(N2CCNC(O)C2C)n1	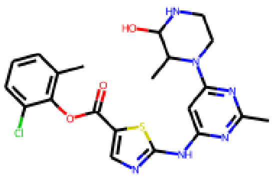	6.0	0.520676	2.336694	2.899742	4	0.820374
**3182**	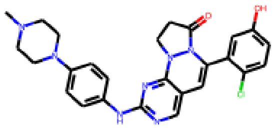	0.376725	O/C(Nc1ccc(Oc2ccccc2)cc1)Oc1ccc2(Cc1)NC(=O)/C(=O)c1ccc(O)n12	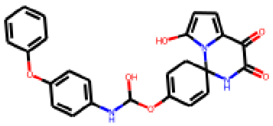	4.0	0.520676	2.336694	2.899742	5	0.820778
**3183**	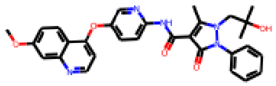	0.375487	COc1ccc2c(=O)cc(CNC(=O)c3c(C)n(C)n(-c4ccccc4)c3=O)nc3cccc2c3c1	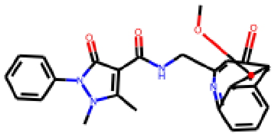	9.0	0.638163	3.995589	2.988471	5	0.774651

	src known mol	similarity	smiles	mol	Class	qed	logP	SAS	rings	kinase inhibition likeliness
**3184**	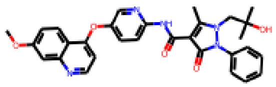	0.374560	COc1ccc2c(=O)cc(CNC(=O)c3c(C)n(C)n(-c4ccccc4)c3=O)nc3cccc3c2n1	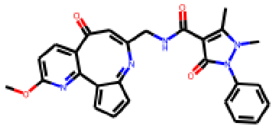	9.0	0.768132	3.608437	2.513170	5	0.823048
**3185**	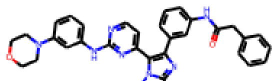	0.374356	ClCNc1nccc(-c2c(-c3ccc(F)cc3)nc3n2[C@H](CO)CC3)n1	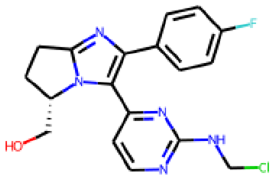	7.0	0.520676	2.336694	2.899742	4	0.812342
**3186**	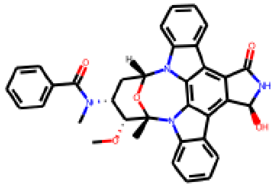	0.374280	COc1nccc(-c2cs/cc2-c2c(CC(=O)Nc3cccc(C(C)(C)C)c3)cc2F)n1	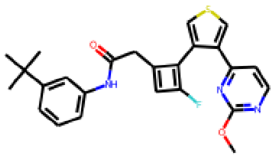	6.0	0.638163	3.995589	2.988471	4	0.831350
**3187**	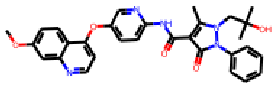	0.373892	COc1ccc2c(=O)cc(CNC(=O)c3c(C)n(C)n(-c4ccccc4)c3=O)nc3cccc3c2n1	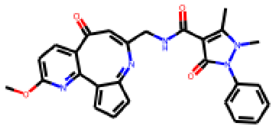	9.0	0.768132	3.608437	2.513170	5	0.823048

	src known mol	similarity	smiles	mol	Class	qed	logP	SAS	rings	kinase inhibition likeliness
**3188**	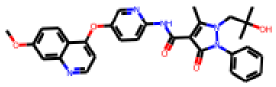	0.373602	COc1ccc2c(=O)cc(CNC(=O)c3c(C)n(C)n(-c4ccccc4)c3=O)nc3cccc2c3c1	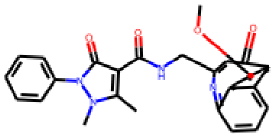	9.0	0.638163	3.995589	2.988471	5	0.774651
**3189**	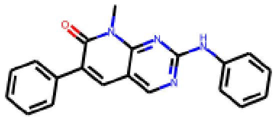	0.372697	Clc1cccc(Nc2ncc3cc(-c3c(Cl)cccc3Cl)c(=O)n(C)c3n2)c1	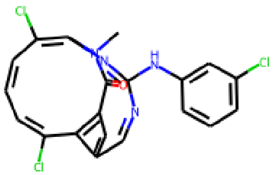	2.0	0.768132	3.608437	2.513170	5	0.784634
**3190**	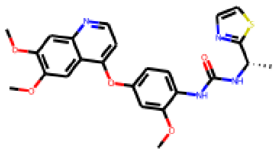	0.372139	COc1ccccc1NC(=O)Nc1ccc(Oc2ccnc3cc(OC)c(OC)cc32)cc1	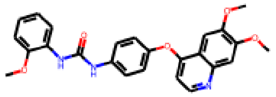	5.0	0.768132	3.608437	2.513170	4	0.790955
**3191**	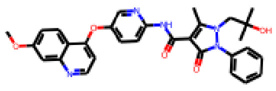	0.370977	COc1ccc2c(=O)cc(CNC(=O)c3c(C)n(C)n(-c4ccccc4)c3=O)nc3cccc2c3c1	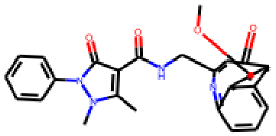	9.0	0.638163	3.995589	2.988471	5	0.774651

	src known mol	similarity	smiles	mol	Class	qed	logP	SAS	rings	kinase inhibition likeliness
**3192**	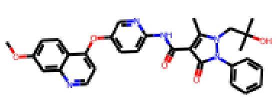	0.370977	COc1ccc2c(=O)cc(CNC(=O)c3c(C)n(C)n(-c4ccccc4)c3=O)nc3cccc2c3c1	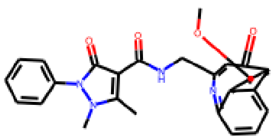	9.0	0.638163	3.995589	2.988471	5	0.774651
**3193**	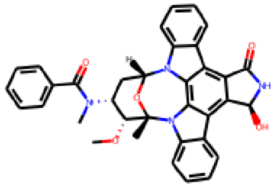	0.370918	COc1cc2c(=O)cc(CNC(=O)c3nn(-c4ccccc4C(C)(F)F)o3)c2o1	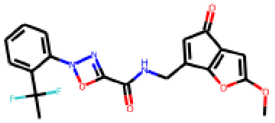	9.0	0.576217	4.086599	3.569904	4	0.783532
**3194**	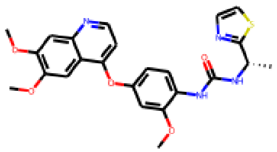	0.370732	COc1ccccc1NC(=O)Nc1ccc(Oc2ccnc3cc(OC)c(OC)cc23)cc1	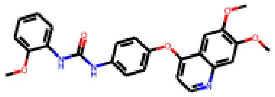	5.0	0.638163	3.995589	2.988471	4	0.789556
**3195**	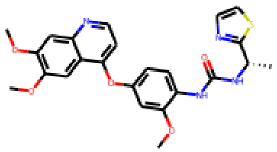	0.370526	COc1ccccc1NC(=O)Nc1ccc(Oc2ccnc3cc(OC)c(OC)cc23)cc1	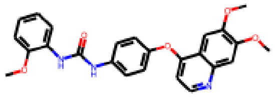	5.0	0.638163	3.995589	2.988471	4	0.789556

	src known mol	similarity	smiles	mol	Class	qed	logP	SAS	rings	kinase inhibition likeliness
**3196**	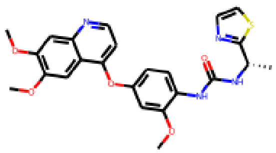	0.370225	COc1ccccc1NC(=O)Nc1ccc(Oc2ccnc3cc(OC)c(OC)cc32)cc1	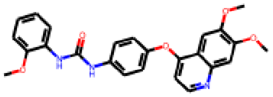	5.0	0.768132	3.608437	2.513170	4	0.790955
**3197**	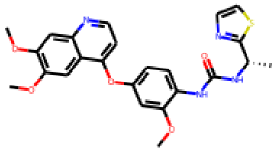	0.369610	COc1ccccc1NC(=O)Nc1ccc(Oc2ccnc3cc(OC)c(OC)cc23)cc1	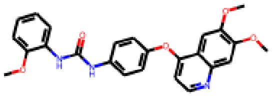	5.0	0.638163	3.995589	2.988471	4	0.789556
**3198**	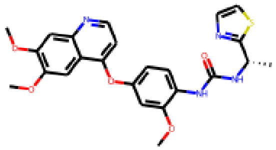	0.369171	COc1ccccc1NC(=O)Nc1ccc(Oc2ccnc3cc(OC)c(OC)cc23)cc1	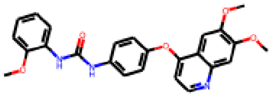	5.0	0.638163	3.995589	2.988471	4	0.789556
**3199**	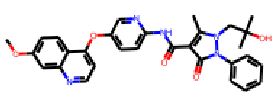	0.368732	COc1ccc2c(=O)cc(CNC(=O)c3c(C)n(C)n(-c4ccccc4)c3=O)nc3cccc3c2n1	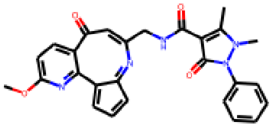	9.0	0.768132	3.608437	2.513170	5	0.823048

	src known mol	similarity	smiles	mol	Class	qed	logP	SAS	rings	kinase inhibition likeliness
**3200**	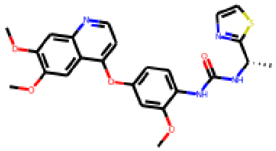	0.368477	COc1ccccc1NC(=O)Nc1ccc(Oc2ccnc3cc(OC)c(OC)cc32)cc1	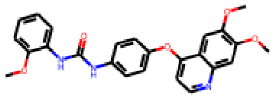	5.0	0.768132	3.608437	2.513170	4	0.790955
**3201**	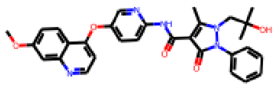	0.368243	COc1ccc2c(=O)cc(CNC(=O)c3c(C)n(C)n(-c4ccccc4)c3=O)nc3cccc2c3c1	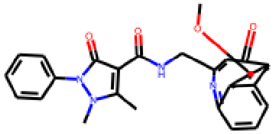	9.0	0.638163	3.995589	2.988471	5	0.774651
**3202**	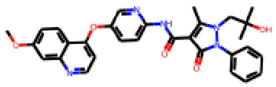	0.367359	COc1ccc2c(=O)cc(CNC(=O)c3c(C)n(C)n(-c4ccccc4)c3=O)nc3cccc3c2n1	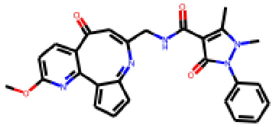	9.0	0.768132	3.608437	2.513170	5	0.823048
**3203**	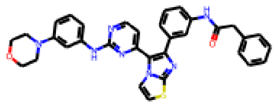	0.367116	ClCNc1nccc(-c2c(-c3ccc(F)cc3)nc3n2[C@H](CO)CC3)n1	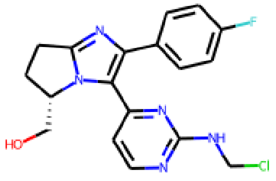	7.0	0.520676	2.336694	2.899742	4	0.812342

	src known mol	similarity	smiles	mol	Class	qed	logP	SAS	rings	kinase inhibition likeliness
**3204**	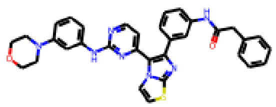	0.367116	ClCNc1nccc(-c2c(-c3ccc(F)cc3)nc3n2[C@H](CO)CC3)n1	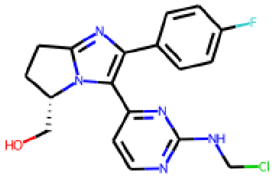	7.0	0.520676	2.336694	2.899742	4	0.812342
**3205**	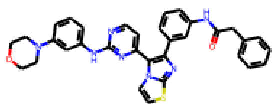	0.367116	ClCNc1nccc(-c2c(-c3ccc(F)cc3)nc3n2[C@H](CO)CC3)n1	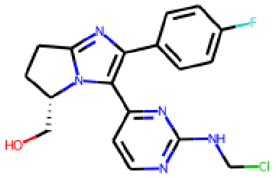	7.0	0.520676	2.336694	2.899742	4	0.812342
**3206**	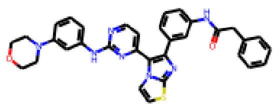	0.367116	ClCNc1nccc(-c2c(-c3ccc(F)cc3)nc3n2[C@H](CO)CC3)n1	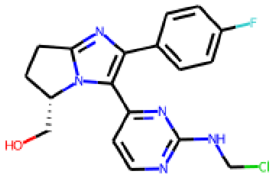	7.0	0.520676	2.336694	2.899742	4	0.812342
**3207**	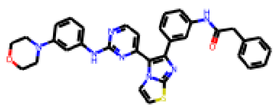	0.367116	ClCNc1nccc(-c2c(-c3ccc(F)cc3)nc3n2[C@H](CO)CC3)n1	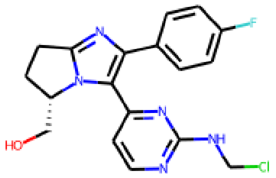	7.0	0.520676	2.336694	2.899742	4	0.812342

	src known mol	similarity	smiles	mol	Class	qed	logP	SAS	rings	kinase inhibition likeliness
**3208**	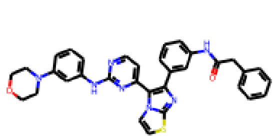	0.367116	ClCNc1nccc(-c2c(-c3ccc(F)cc3)nc3n2[C@H](CO)CC3)n1	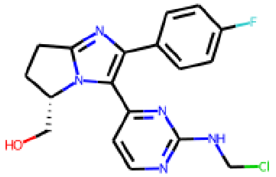	7.0	0.520676	2.336694	2.899742	4	0.812342
**3209**	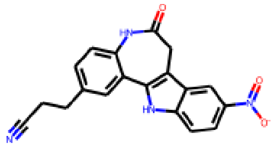	0.366531	CCNC(=O)N1CCC(Nc2n(C(C)c3c(-c4ccccc4)[nH]c4ccccc34)n2)CC1	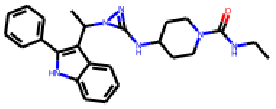	7.0	0.623584	3.603734	4.478009	5	0.804049
**3210**	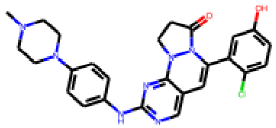	0.366449	O/C(Nc1ccc(Oc2ccccc2)cc1)Oc1ccc2(Cc1)NC(=O)/C(=O)c1ccc(O)n12	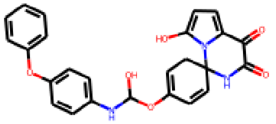	4.0	0.520676	2.336694	2.899742	5	0.820778
**3211**	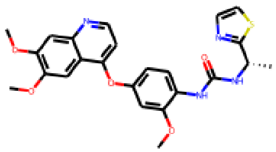	0.366438	COc1ccccc1NC(=O)Nc1ccc(Oc2ccnc3cc(OC)c(OC)cc23)cc1	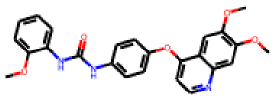	5.0	0.638163	3.995589	2.988471	4	0.789556

	src known mol	similarity	smiles	mol	Class	qed	logP	SAS	rings	kinase inhibition likeliness
**3212**	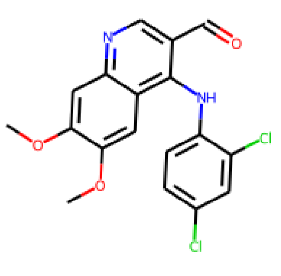	0.366359	COc1cc2ncc(C(=O)O)c(Nc3cccc(Cl)c3Cl)c2cc1OC1C(C)C1	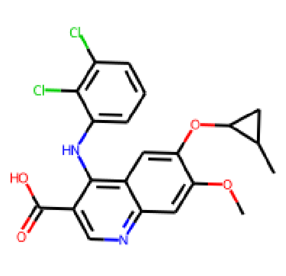	2.0	0.623362	2.525411	2.997881	4	0.774857
**3213**	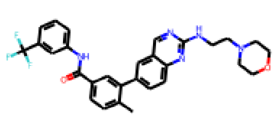	0.365854	Cc1ccc(C(=O)Nc2cccc(C(F)(C)F)c2C)cc1-c1ccc2nc(C)ccccc(F)nc2c1	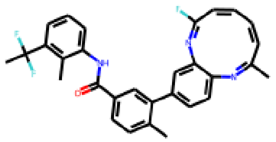	6.0	0.742375	4.384502	0.286234	4	0.779167
**3214**	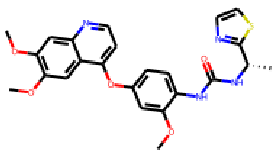	0.365807	COc1ccccc1NC(=O)Nc1ccc(Oc2ccnc3cc(OC)c(OC)cc23)cc1	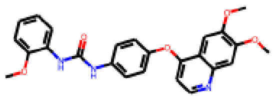	5.0	0.638163	3.995589	2.988471	4	0.789556
**3215**	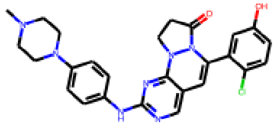	0.365165	O/C(Nc1ccc(Oc2ccccc2)cc1)Oc1ccc2(Cc1)NC(=O)/C(=O)c1ccc(O)n12	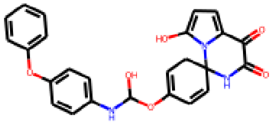	4.0	0.520676	2.336694	2.899742	5	0.820778

	src known mol	similarity	smiles	mol	Class	qed	logP	SAS	rings	kinase inhibition likeliness
**3216**	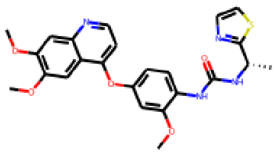	0.364504	COc1ccccc1NC(=O)Nc1ccc(Oc2ccnc3cc(OC)c(OC)cc32)cc1	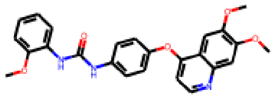	5.0	0.768132	3.608437	2.513170	4	0.790955
**3217**	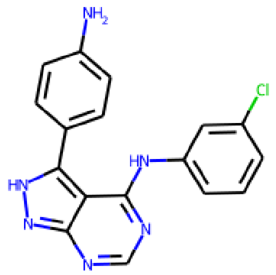	0.364177	c1ccc(Nc2ncnc3n[nH]c(-c4ccccc4)c23)cc1	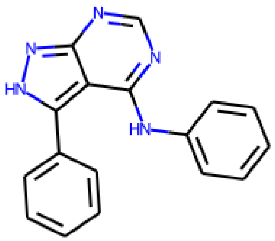	0.0	0.638163	3.995589	2.988471	4	0.800634
**3218**	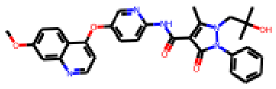	0.363739	COc1ccc2c(=O)cc(CNC(=O)c3c(C)n(C)n(-c4ccccc4)c3=O)nc3cccc2c3c1	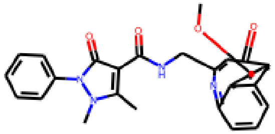	9.0	0.638163	3.995589	2.988471	5	0.774651
**3219**	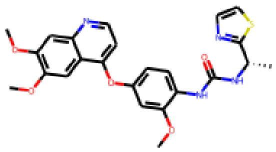	0.363636	COc1ccccc1NC(=O)Nc1ccc(Oc2ccnc3cc(OC)c(OC)cc23)cc1	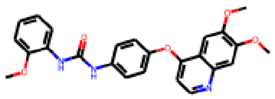	5.0	0.638163	3.995589	2.988471	4	0.789556

	src known mol	similarity	smiles	mol	Class	qed	logP	SAS	rings	kinase inhibition likeliness
**3220**	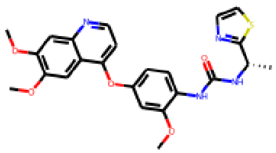	0.362069	COc1ccccc1NC(=O)Nc1ccc(Oc2ccnc3cc(OC)c(OC)cc32)cc1	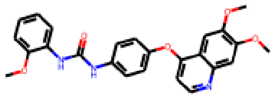	5.0	0.768132	3.608437	2.513170	4	0.790955
**3221**	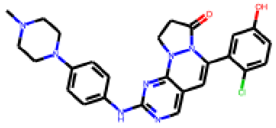	0.361715	O/C(Nc1ccc(Oc2ccccc2)cc1)Oc1ccc2(Cc1)NC(=O)/C(=O)c1ccc(O)n12	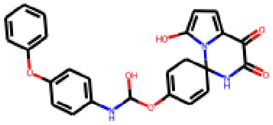	4.0	0.520676	2.336694	2.899742	5	0.820778
**3222**	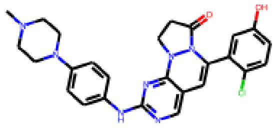	0.361460	O/C(Nc1ccc(Oc2ccccc2)cc1)Oc1ccc2(Cc1)NC(=O)/C(=O)c1ccc(O)n12	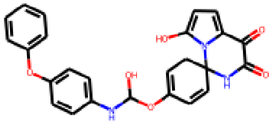	4.0	0.520676	2.336694	2.899742	5	0.820778
**3223**	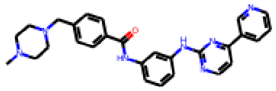	0.358706	Fc1ccc(NC(=O)c2ccccc2)cc1Nc1nccc(-c2cccnc2)n1	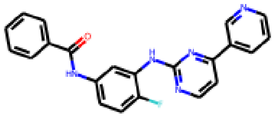	0.0	0.632663	4.308233	4.065316	4	0.777346

	src known mol	similarity	smiles	mol	Class	qed	logP	SAS	rings	kinase inhibition likeliness
**3224**	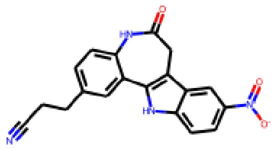	0.358516	CCNC(=O)N1CCC(Nc2n(C(C)c3c(-c4ccccc4)[nH]c4ccccc34)n2)CC1	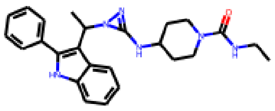	7.0	0.623584	3.603734	4.478009	5	0.804049
**3225**	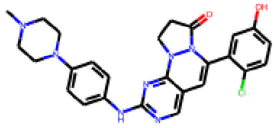	0.358434	O/C(Nc1ccc(Oc2ccccc2)cc1)Oc1ccc2(Cc1)NC(=O)/C(=O)c1ccc(O)n12	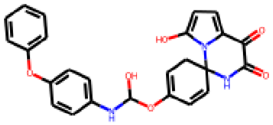	4.0	0.520676	2.336694	2.899742	5	0.820778
**3226**	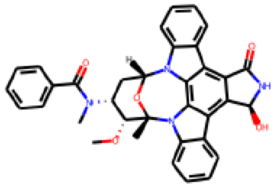	0.357189	COc1nccc(-c2cs/cc2-c2c(CC(=O)Nc3cccc(C(C)(C)C)c3)cc2F)n1	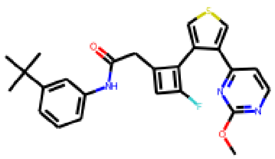	6.0	0.638163	3.995589	2.988471	4	0.831350
**3227**	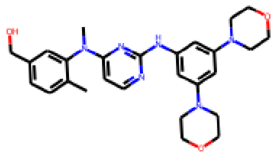	0.357022	Cc1cc(Nc2ncc(Cl)c(Nc3ccccc3C(N)=O)n2)ccc1N1CCOC1	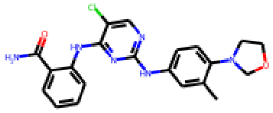	7.0	0.520676	2.336694	2.899742	4	0.767177

	src known mol	similarity	smiles	mol	Class	qed	logP	SAS	rings	kinase inhibition likeliness
**3228**	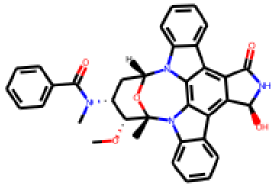	0.356258	COc1cc2c(=O)cc(CNC(=O)c3nn(-c4ccccc4C(C)(F)F)o3)c2o1	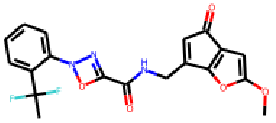	9.0	0.576217	4.086599	3.569904	4	0.783532
**3229**	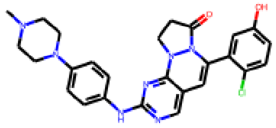	0.356115	O/C(Nc1ccc(Oc2ccccc2)cc1)Oc1ccc2(Cc1)NC(=O)/C(=O)c1ccc(O)n12	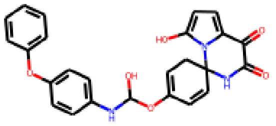	4.0	0.520676	2.336694	2.899742	5	0.820778
**3230**	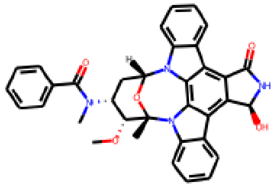	0.355995	COc1cc2c(=O)cc(CNC(=O)c3nn(-c4ccccc4C(C)(F)F)o3)c2o1	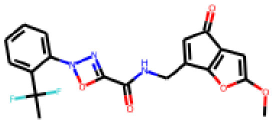	9.0	0.576217	4.086599	3.569904	4	0.783532
**3231**	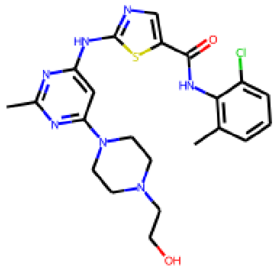	0.355841	Cc1nc(Nc2ncc(C(=O)Oc3c(C)cccc3Cl)s2)cc(N2CCNC(=O)C2=O)n1	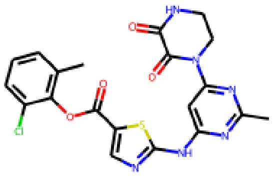	2.0	0.771867	2.951597	1.935530	4	0.787643

	src known mol	similarity	smiles	mol	Class	qed	logP	SAS	rings	kinase inhibition likeliness
**3232**	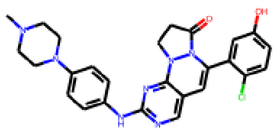	0.355556	O/C(Nc1ccc(Oc2ccccc2)cc1)Oc1ccc2(Cc1)NC(=O)/C(=O)c1ccc(O)n12	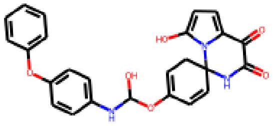	4.0	0.520676	2.336694	2.899742	5	0.820778
**3233**	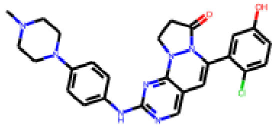	0.355556	O/C(Nc1ccc(Oc2ccccc2)cc1)Oc1ccc2(Cc1)NC(=O)/C(=O)c1ccc(O)n12	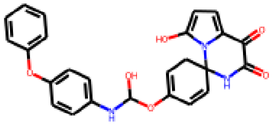	4.0	0.520676	2.336694	2.899742	5	0.820778
**3234**	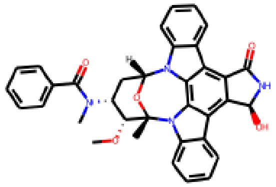	0.354540	COc1cc2c(=O)cc(CNC(=O)c3nn(-c4ccccc4C(C)(F)F)o3)c2o1	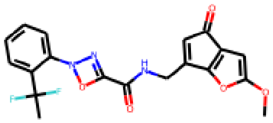	9.0	0.576217	4.086599	3.569904	4	0.783532
**3235**	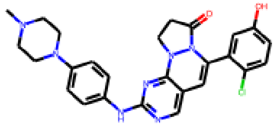	0.353940	Cc1nc(-c2ccc(C(=O)Oc3c(C)cccc3Cl)o2)cc(N2CCNC(=O)C2)n1	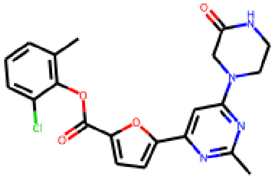	6.0	0.705736	3.500327	0.134263	4	0.764425

	src known mol	similarity	smiles	mol	Class	qed	logP	SAS	rings	kinase inhibition likeliness
**3236**	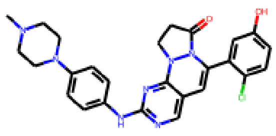	0.353940	Cc1nc(-c2ccc(C(=O)Oc3c(C)cccc3Cl)o2)cc(N2CCNC(=O)C2)n1	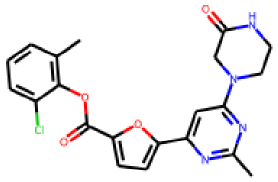	6.0	0.705736	3.500327	0.134263	4	0.764425
**3237**	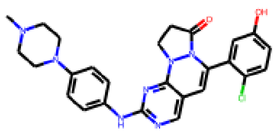	0.353940	Cc1nc(-c2ccc(C(=O)Oc3c(C)cccc3Cl)o2)cc(N2CCNC(=O)C2)n1	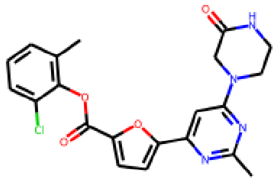	6.0	0.705736	3.500327	0.134263	4	0.764425
**3238**	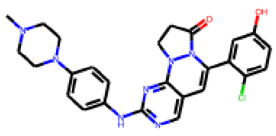	0.353940	Cc1nc(-c2ccc(C(=O)Oc3c(C)cccc3Cl)o2)cc(N2CCNC(=O)C2)n1	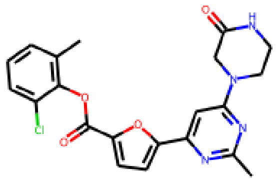	6.0	0.705736	3.500327	0.134263	4	0.764425
**3239**	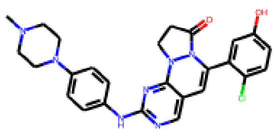	0.353940	Cc1nc(-c2ccc(C(=O)Oc3c(C)cccc3Cl)o2)cc(N2CCNC(=O)C2)n1	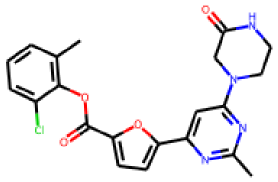	6.0	0.705736	3.500327	0.134263	4	0.764425

	src known mol	similarity	smiles	mol	Class	qed	logP	SAS	rings	kinase inhibition likeliness
**3240**	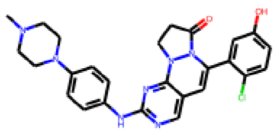	0.353940	Cc1nc(-c2ccc(C(=O)Oc3c(C)cccc3Cl)o2)cc(N2CCNC(=O)C2)n1	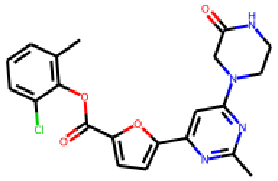	6.0	0.705736	3.500327	0.134263	4	0.764425
**3241**	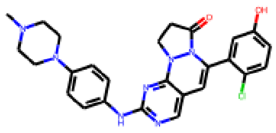	0.353717	O/C(Nc1ccc(Oc2ccccc2)cc1)Oc1ccc2(Cc1)NC(=O)/C(=O)c1ccc(O)n12	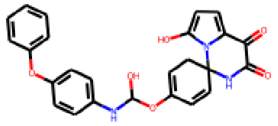	4.0	0.520676	2.336694	2.899742	5	0.820778
**3242**	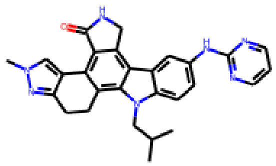	0.353602	Cc1ccc(C(=O)Nc2cccc(C(F)(C)F)c2C)cc1-c1ccc2nc(=O)cccc(F)nc2n1	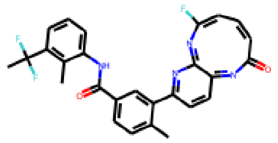	6.0	0.820294	3.038428	1.698172	4	0.833140
**3243**	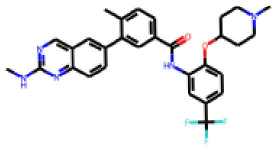	0.353372	Cc1ccc(C(=O)Nc2cccc(C(F)(C)F)c2C)cc1-c1ccc2oc(=O)cccc(F)c2c1	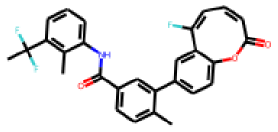	6.0	0.812693	3.141472	1.494671	4	0.774667

	src known mol	similarity	smiles	mol	Class	qed	logP	SAS	rings	kinase inhibition likeliness
**3244**	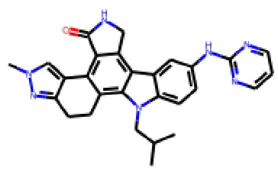	0.353369	C#Cc1ccc(C(=O)OC)cc1-c1C(=O)c-n2nc(-c3ccc(F)cc3F)cc2C1	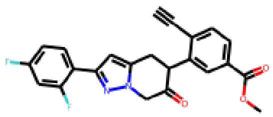	8.0	0.768132	3.608437	2.513170	4	0.763730
**3245**	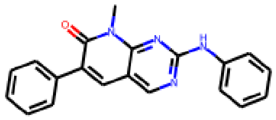	0.352893	Clc1cccc(Nc2ncc3cc(-c3c(Cl)cccc3Cl)c(=O)n(C)c3n2)c1	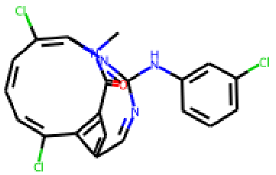	2.0	0.768132	3.608437	2.513170	5	0.784634
**3246**	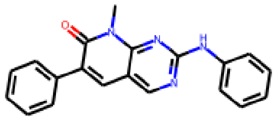	0.352893	Clc1cccc(Nc2ncc3cc(-c3c(Cl)cccc3Cl)c(=O)n(C)c3n2)c1	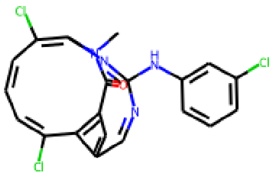	2.0	0.768132	3.608437	2.513170	5	0.784634
**3247**	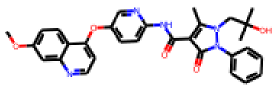	0.352608	COc1ccc2c(=O)cc(CNC(=O)c3c(C)n(C)n(-c4ccccc4)c3=O)nc3cccc2c3c1	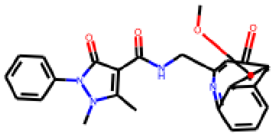	9.0	0.638163	3.995589	2.988471	5	0.774651

	src known mol	similarity	smiles	mol	Class	qed	logP	SAS	rings	kinase inhibition likeliness
**3248**	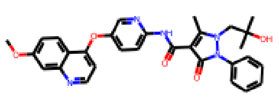	0.351611	COc1ccc2c(=O)cc(CNC(=O)c3c(C)n(C)n(-c4ccccc4)c3=O)nc3cccc2c3c1	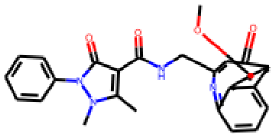	9.0	0.638163	3.995589	2.988471	5	0.774651
**3249**	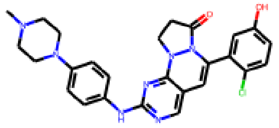	0.351384	O/C(Nc1ccc(Oc2ccccc2)cc1)Oc1ccc2(Cc1)NC(=O)/C(=O)c1ccc(O)n12	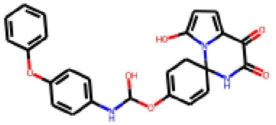	4.0	0.520676	2.336694	2.899742	5	0.820778
**3250**	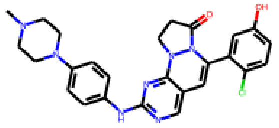	0.350853	Cc1nc(-c2ccc(C(=O)Oc3c(C)cccc3Cl)o2)cc(N2CCNC(=O)C2)n1	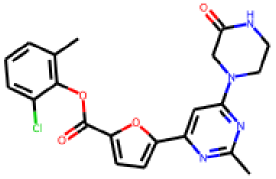	6.0	0.705736	3.500327	0.134263	4	0.764425
**3251**	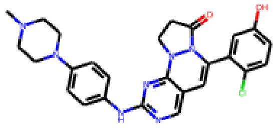	0.350853	Cc1nc(-c2ccc(C(=O)Oc3c(C)cccc3Cl)o2)cc(N2CCNC(=O)C2)n1	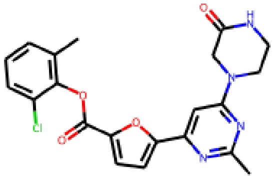	6.0	0.705736	3.500327	0.134263	4	0.764425

	src known mol	similarity	smiles	mol	Class	qed	logP	SAS	rings	kinase inhibition likeliness
**3252**	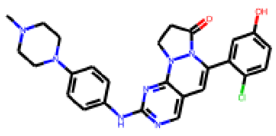	0.350853	Cc1nc(-c2ccc(C(=O)Oc3c(C)cccc3Cl)o2)cc(N2CCNC(=O)C2)n1	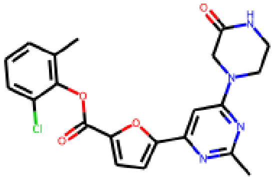	6.0	0.705736	3.500327	0.134263	4	0.764425
**3253**	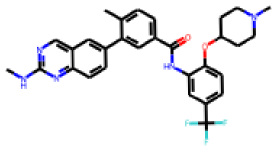	0.350810	Cc1ccc(C(=O)Nc2cccc(C(F)(C)F)c2C)cc1-c1ccc2oc(=O)cccc(F)c2c1	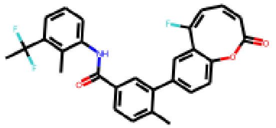	6.0	0.812693	3.141472	1.494671	4	0.774667
**3254**	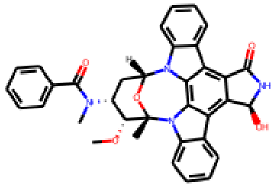	0.349805	COc1nccc(-c2cs/cc2-c2c(CC(=O)Nc3cccc(C(C)(C)C)c3)cc2F)n1	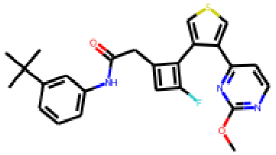	6.0	0.638163	3.995589	2.988471	4	0.831350
**3255**	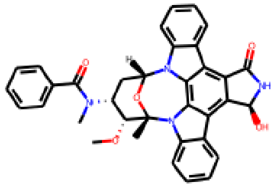	0.349805	COc1nccc(-c2cs/cc2-c2c(CC(=O)Nc3cccc(C(C)(C)C)c3)cc2F)n1	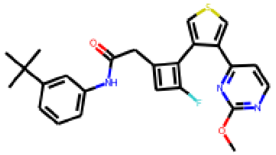	6.0	0.638163	3.995589	2.988471	4	0.831350

	src known mol	similarity	smiles	mol	Class	qed	logP	SAS	rings	kinase inhibition likeliness
**3256**	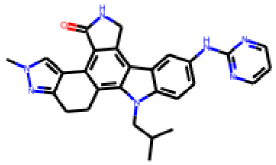	0.348344	Cc1ccc(C(=O)Nc2cccc(C(F)(C)F)c2C)cc1-c1ccc2nc(=O)cccc(F)nc2n1	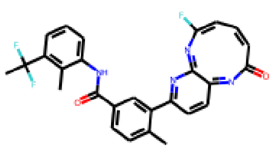	6.0	0.820294	3.038428	1.698172	4	0.833140
**3257**	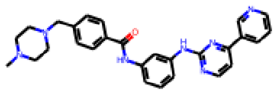	0.348276	Fc1ccc(NC(=O)c2ccccc2)cc1Nc1nccc(-c2cccnc2)n1	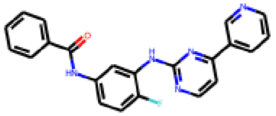	0.0	0.632663	4.308233	4.065316	4	0.777346
**3258**	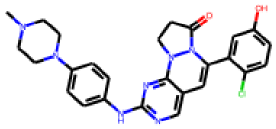	0.347458	O/C(Nc1ccc(Oc2ccccc2)cc1)Oc1ccc2(Cc1)NC(=O)/C(=O)c1ccc(O)n12	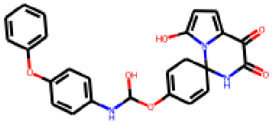	4.0	0.520676	2.336694	2.899742	5	0.820778
**3259**	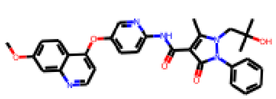	0.347412	COc1ccc2c(=O)cc(CNC(=O)c3c(C)n(C)n(-c4ccccc4)c3=O)nc3cccc3c2n1	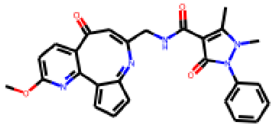	9.0	0.768132	3.608437	2.513170	5	0.823048

	src known mol	similarity	smiles	mol	Class	qed	logP	SAS	rings	kinase inhibition likeliness
**3260**	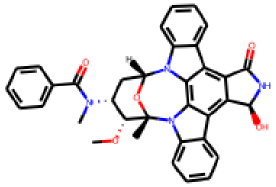	0.346684	COc1nccc(-c2cs/cc2-c2c(CC(=O)Nc3cccc(C(C)(C)C)c3)cc2F)n1	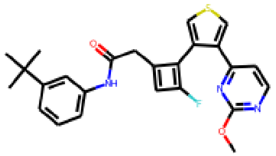	6.0	0.638163	3.995589	2.988471	4	0.831350
**3261**	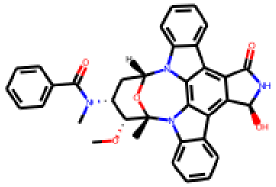	0.346684	COc1nccc(-c2cs/cc2-c2c(CC(=O)Nc3cccc(C(C)(C)C)c3)cc2F)n1	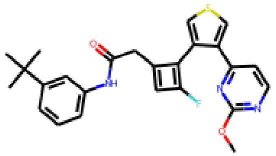	6.0	0.638163	3.995589	2.988471	4	0.831350
**3262**	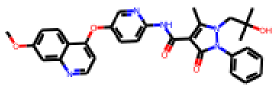	0.346132	COc1ccc2c(=O)cc(CNC(=O)c3c(C)n(C)n(-c4ccccc4)c3=O)nc3cccc2c3c1	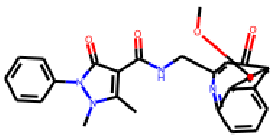	9.0	0.638163	3.995589	2.988471	5	0.774651
**3263**	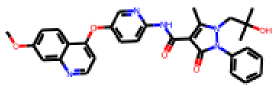	0.345178	COc1ccc2c(=O)cc(CNC(=O)c3c(C)n(C)n(-c4ccccc4)c3=O)nc3cccc2c3c1	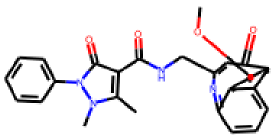	9.0	0.638163	3.995589	2.988471	5	0.774651

	src known mol	similarity	smiles	mol	Class	qed	logP	SAS	rings	kinase inhibition likeliness
**3264**	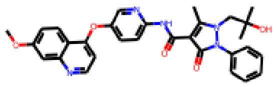	0.345178	COc1ccc2c(=O)cc(CNC(=O)c3c(C)n(C)n(-c4ccccc4)c3=O)nc3cccc2c3c1	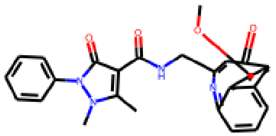	9.0	0.638163	3.995589	2.988471	5	0.774651
**3265**	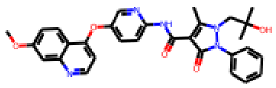	0.345178	COc1ccc2c(=O)cc(CNC(=O)c3c(C)n(C)n(-c4ccccc4)c3=O)nc3cccc2c3c1	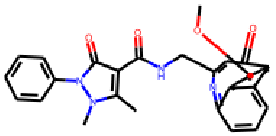	9.0	0.638163	3.995589	2.988471	5	0.774651
**3266**	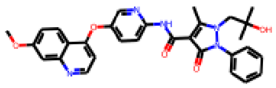	0.344514	COc1ccc2c(=O)cc(CNC(=O)c3c(C)n(C)n(-c4ccccc4)c3=O)nc3cccc2c3c1	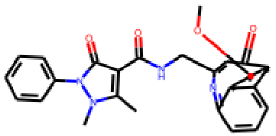	9.0	0.638163	3.995589	2.988471	5	0.774651
**3267**	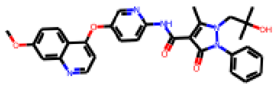	0.343489	COc1ccc2c(=O)cc(CNC(=O)c3c(C)n(C)n(-c4ccccc4)c3=O)nc3cccc3c2n1	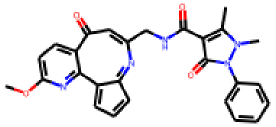	9.0	0.768132	3.608437	2.513170	5	0.823048

	src known mol	similarity	smiles	mol	Class	qed	logP	SAS	rings	kinase inhibition likeliness
**3268**	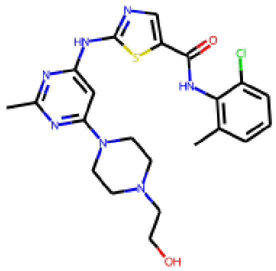	0.341118	Cc1nc(Nc2ncc(C(=O)Nc3c(C)cccc3Cl)s2)cc(N2CCNC(=O)C2)n1	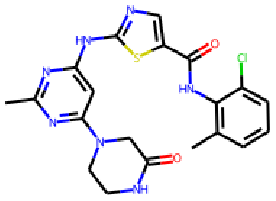	6.0	0.593231	3.250153	2.680275	4	0.755833
**3269**	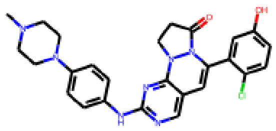	0.340786	Cc1nc(-c2ccc(C(=O)Oc3c(C)cccc3Cl)o2)cc(N2CCNC(=O)C2)n1	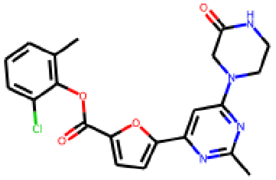	6.0	0.705736	3.500327	0.134263	4	0.764425
**3270**	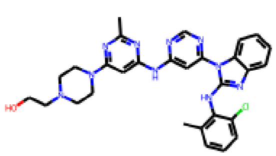	0.340690	Cc1nc(Nc2ccc(C(=O)Oc3c(C)cccc3Cl)s2)cc(N2CCNC(=O)C2=O)n1	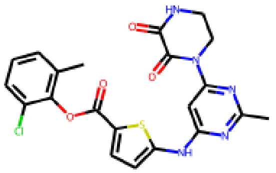	2.0	0.584673	2.312750	2.571988	4	0.770333
**3271**	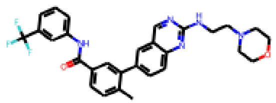	0.340206	Cc1ccc(C(=O)Oc2cccc(C(F)(F)F)c2)cc1-c1ccc2nc(N)ncc2c1	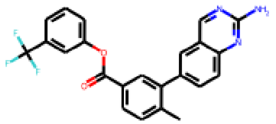	6.0	0.638163	3.995589	2.988471	4	0.789898

	src known mol	similarity	smiles	mol	Class	qed	logP	SAS	rings	kinase inhibition likeliness
**3272**	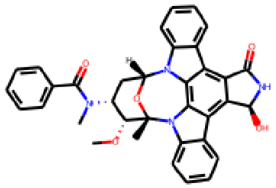	0.339386	COc1cc2c(=O)cc(CNC(=O)c3nn(-c4ccccc4C(C)(F)F)o3)c2o1	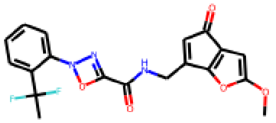	9.0	0.576217	4.086599	3.569904	4	0.783532
**3273**	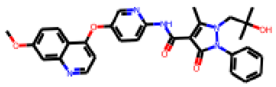	0.339367	COc1ccc2c(=O)cc(CNC(=O)c3c(C)n(C)n(-c4ccccc4)c3=O)nc3cccc2c3c1	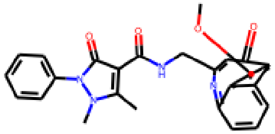	9.0	0.638163	3.995589	2.988471	5	0.774651
**3274**	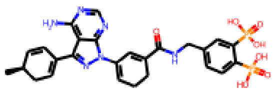	0.339364	C(N1[nH]c(C)c1CCN(c1cc(-c3nn(C)cc3-c3ccc(F)cc3)cc2)nc2C)n1	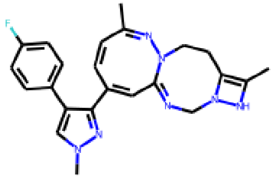	7.0	0.768132	3.608437	2.513170	5	0.754838
**3275**	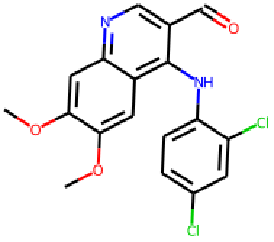	0.337971	COc1cc2ncc(C(=O)O)c(Nc3cccc(Cl)c3Cl)c2cc1OC1C(C)C1	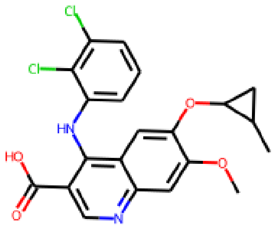	2.0	0.623362	2.525411	2.997881	4	0.774857

	src known mol	similarity	smiles	mol	Class	qed	logP	SAS	rings	kinase inhibition likeliness
**3276**	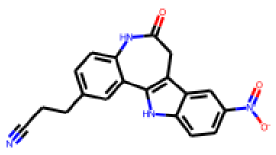	0.337896	CCNC(=O)N1CCC(Nc2n(C(C)c3c(-c4ccccc4)[nH]c4ccccc34)n2)CC1	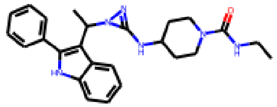	7.0	0.623584	3.603734	4.478009	5	0.804049
**3277**	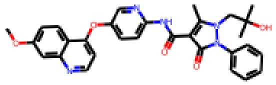	0.337515	COc1ccc2c(=O)cc(CNC(=O)c3c(C)n(C)n(-c4ccccc4)c3=O)nc3cccc3c2n1	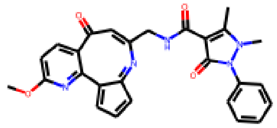	9.0	0.768132	3.608437	2.513170	5	0.823048
**3278**	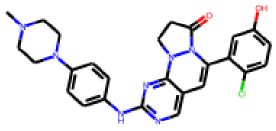	0.337483	Cc1nc(-c2ccc(C(=O)Oc3c(C)cccc3Cl)o2)cc(N2CCNC(=O)C2)n1	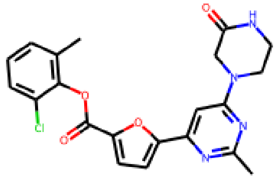	6.0	0.705736	3.500327	0.134263	4	0.764425
**3279**	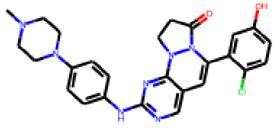	0.337483	Cc1nc(-c2ccc(C(=O)Oc3c(C)cccc3Cl)o2)cc(N2CCNC(=O)C2)n1	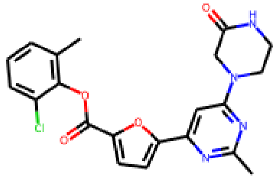	6.0	0.705736	3.500327	0.134263	4	0.764425

	src known mol	similarity	smiles	mol	Class	qed	logP	SAS	rings	kinase inhibition likeliness
**3280**	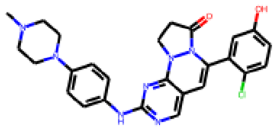	0.337483	Cc1nc(-c2ccc(C(=O)Oc3c(C)cccc3Cl)o2)cc(N2CCNC(=O)C2)n1	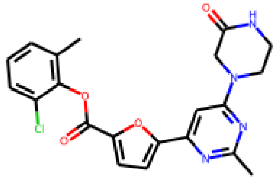	6.0	0.705736	3.500327	0.134263	4	0.764425
**3281**	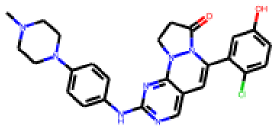	0.337483	Cc1nc(-c2ccc(C(=O)Oc3c(C)cccc3Cl)o2)cc(N2CCNC(=O)C2)n1	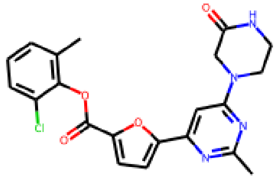	6.0	0.705736	3.500327	0.134263	4	0.764425
**3282**	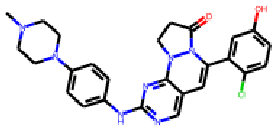	0.337483	Cc1nc(-c2ccc(C(=O)Oc3c(C)cccc3Cl)o2)cc(N2CCNC(=O)C2)n1	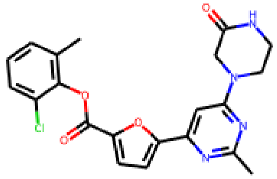	6.0	0.705736	3.500327	0.134263	4	0.764425
**3283**	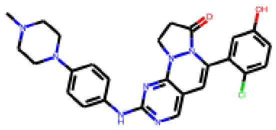	0.337483	Cc1nc(-c2ccc(C(=O)Oc3c(C)cccc3Cl)o2)cc(N2CCNC(=O)C2)n1	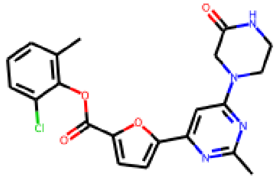	6.0	0.705736	3.500327	0.134263	4	0.764425

	src known mol	similarity	smiles	mol	Class	qed	logP	SAS	rings	kinase inhibition likeliness
**3284**	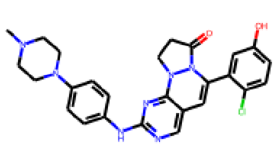	0.337483	Cc1nc(-c2ccc(C(=O)Oc3c(C)cccc3Cl)o2)cc(N2CCNC(=O)C2)n1	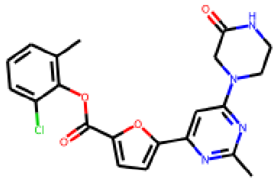	6.0	0.705736	3.500327	0.134263	4	0.764425
**3285**	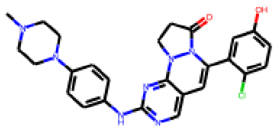	0.337483	Cc1nc(-c2ccc(C(=O)Oc3c(C)cccc3Cl)o2)cc(N2CCNC(=O)C2)n1	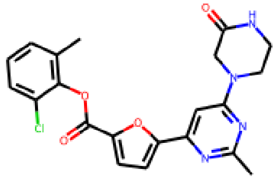	6.0	0.705736	3.500327	0.134263	4	0.764425
**3286**	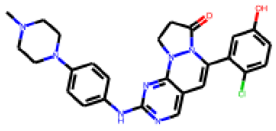	0.337483	Cc1nc(-c2ccc(C(=O)Oc3c(C)cccc3Cl)o2)cc(N2CCNC(=O)C2)n1	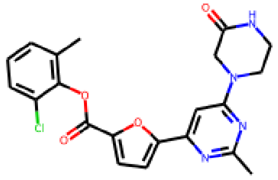	6.0	0.705736	3.500327	0.134263	4	0.764425
**3287**	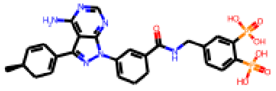	0.337079	C(N1[nH]c(C)c1CCN(c1cc(-c3nn(C)cc3-c3ccc(F)cc3)cc2)nc2C)n1	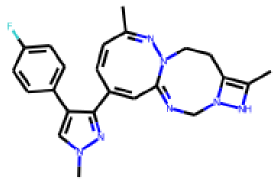	7.0	0.768132	3.608437	2.513170	5	0.754838

	src known mol	similarity	smiles	mol	Class	qed	logP	SAS	rings	kinase inhibition likeliness
**3288**	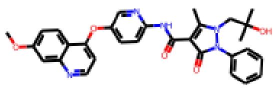	0.335221	COc1ccc2c(=O)cc(CNC(=O)c3c(C)n(C)n(-c4ccccc4)c3=O)nc3cccc2c3c1	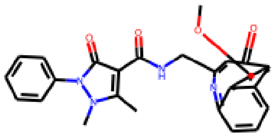	9.0	0.638163	3.995589	2.988471	5	0.774651
**3289**	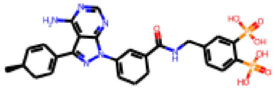	0.335206	C(N1[nH]c(C)c1CCN(c1cc(-c3nn(C)cc3-c3ccc(F)cc3)cc2)nc2C)n1	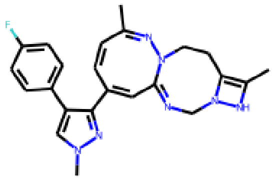	7.0	0.768132	3.608437	2.513170	5	0.754838
**3290**	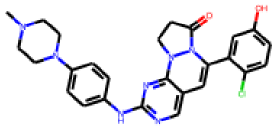	0.335155	O/C(Nc1ccc(Oc2ccccc2)cc1)Oc1ccc2(Cc1)NC(=O)/C(=O)c1ccc(O)n12	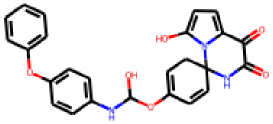	4.0	0.520676	2.336694	2.899742	5	0.820778
**3291**	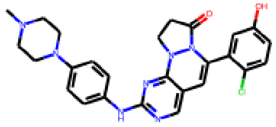	0.335155	O/C(Nc1ccc(Oc2ccccc2)cc1)Oc1ccc2(Cc1)NC(=O)/C(=O)c1ccc(O)n12	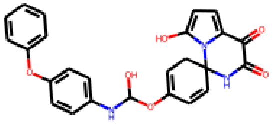	4.0	0.520676	2.336694	2.899742	5	0.820778

	src known mol	similarity	smiles	mol	Class	qed	logP	SAS	rings	kinase inhibition likeliness
**3292**	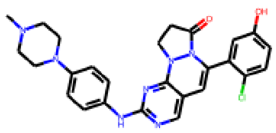	0.335155	O/C(Nc1ccc(Oc2ccccc2)cc1)Oc1ccc2(Cc1)NC(=O)/C(=O)c1ccc(O)n12	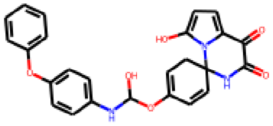	4.0	0.520676	2.336694	2.899742	5	0.820778
**3293**	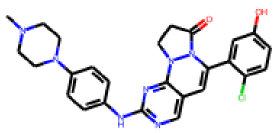	0.335155	O/C(Nc1ccc(Oc2ccccc2)cc1)Oc1ccc2(Cc1)NC(=O)/C(=O)c1ccc(O)n12	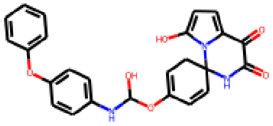	4.0	0.520676	2.336694	2.899742	5	0.820778
**3294**	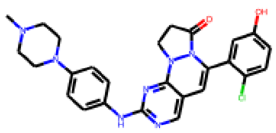	0.335155	O/C(Nc1ccc(Oc2ccccc2)cc1)Oc1ccc2(Cc1)NC(=O)/C(=O)c1ccc(O)n12	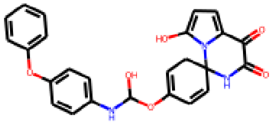	4.0	0.520676	2.336694	2.899742	5	0.820778
**3295**	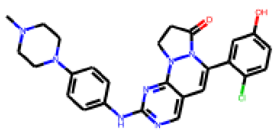	0.334139	O/C(Nc1ccc(Oc2ccccc2)cc1)Oc1ccc2(Cc1)NC(=O)/C(=O)c1ccc(O)n12	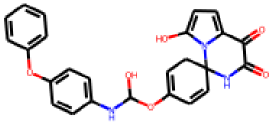	4.0	0.520676	2.336694	2.899742	5	0.820778

	src known mol	similarity	smiles	mol	Class	qed	logP	SAS	rings	kinase inhibition likeliness
**3296**	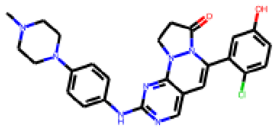	0.334139	O/C(Nc1ccc(Oc2ccccc2)cc1)Oc1ccc2(Cc1)NC(=O)/C(=O)c1ccc(O)n12	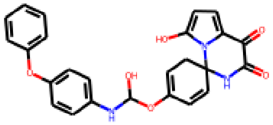	4.0	0.520676	2.336694	2.899742	5	0.820778
**3297**	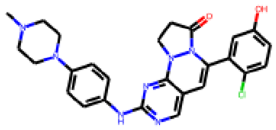	0.334139	O/C(Nc1ccc(Oc2ccccc2)cc1)Oc1ccc2(Cc1)NC(=O)/C(=O)c1ccc(O)n12	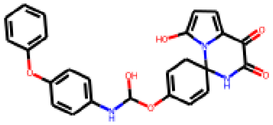	4.0	0.520676	2.336694	2.899742	5	0.820778
**3298**	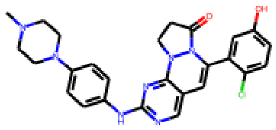	0.334139	O/C(Nc1ccc(Oc2ccccc2)cc1)Oc1ccc2(Cc1)NC(=O)/C(=O)c1ccc(O)n12	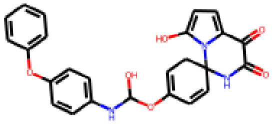	4.0	0.520676	2.336694	2.899742	5	0.820778
**3299**	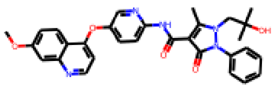	0.332385	COc1ccc2c(=O)cc(CNC(=O)c3c(C)n(C)n(-c4ccccc4)c3=O)nc3cccc2c3c1	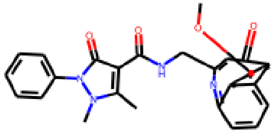	9.0	0.638163	3.995589	2.988471	5	0.774651

	src known mol	similarity	smiles	mol	Class	qed	logP	SAS	rings	kinase inhibition likeliness
**3300**	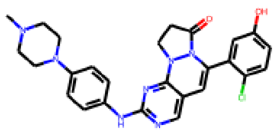	0.331246	Cc1nc(-c2ccc(C(=O)Oc3c(C)cccc3Cl)o2)cc(N2CCNC(=O)C2)n1	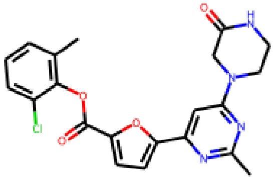	6.0	0.705736	3.500327	0.134263	4	0.764425
**3301**	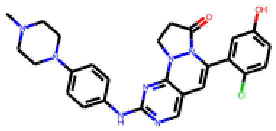	0.330699	O/C(Nc1ccc(Oc2ccccc2)cc1)Oc1ccc2(Cc1)NC(=O)/C(=O)c1ccc(O)n12	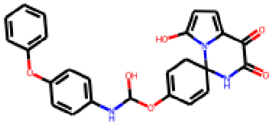	4.0	0.520676	2.336694	2.899742	5	0.820778
**3302**	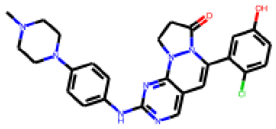	0.330699	O/C(Nc1ccc(Oc2ccccc2)cc1)Oc1ccc2(Cc1)NC(=O)/C(=O)c1ccc(O)n12	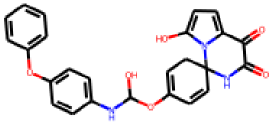	4.0	0.520676	2.336694	2.899742	5	0.820778
**3303**	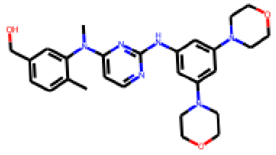	0.330097	Cc1cc(Nc2ncc(Cl)c(Nc3ccccc3C(N)=O)n2)ccc1N1CCOC1	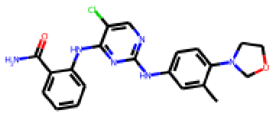	7.0	0.520676	2.336694	2.899742	4	0.767177

	src known mol	similarity	smiles	mol	Class	qed	logP	SAS	rings	kinase inhibition likeliness
**3304**	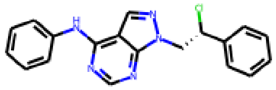	0.330035	Cc1ccc(C(=O)Nc2ccon2)cc1Nc1ncnc2c1cnn2C1cccccc1	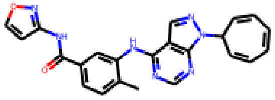	8.0	0.623362	2.525411	2.997881	5	0.770298
**3305**	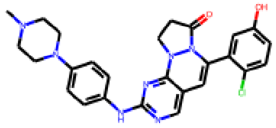	0.329891	O/C(Nc1ccc(Oc2ccccc2)cc1)Oc1ccc2(Cc1)NC(=O)/C(=O)c1ccc(O)n12	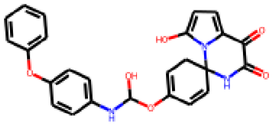	4.0	0.520676	2.336694	2.899742	5	0.820778
**3306**	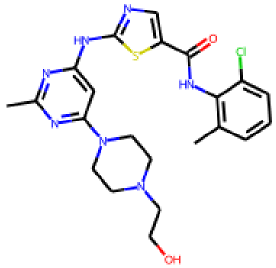	0.329480	Cc1nc(Nc2ncc(C(=O)Nc3c(C)cccc3Cl)s2)cc(N2CCC(CO)C2)n1	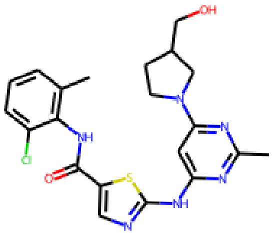	6.0	0.768132	3.608437	2.513170	4	0.818682
**3307**	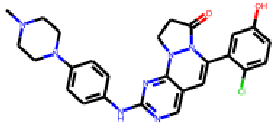	0.329276	O/C(Nc1ccc(Oc2ccccc2)cc1)Oc1ccc2(Cc1)NC(=O)/C(=O)c1ccc(O)n12	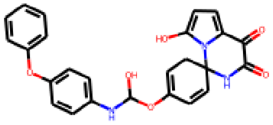	4.0	0.520676	2.336694	2.899742	5	0.820778

	src known mol	similarity	smiles	mol	Class	qed	logP	SAS	rings	kinase inhibition likeliness
**3308**	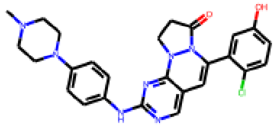	0.329276	O/C(Nc1ccc(Oc2ccccc2)cc1)Oc1ccc2(Cc1)NC(=O)/C(=O)c1ccc(O)n12	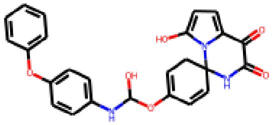	4.0	0.520676	2.336694	2.899742	5	0.820778
**3309**	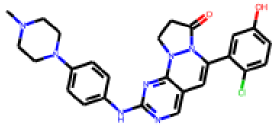	0.329268	O/C(Nc1ccc(Oc2ccccc2)cc1)Oc1ccc2(Cc1)NC(=O)/C(=O)c1ccc(O)n12	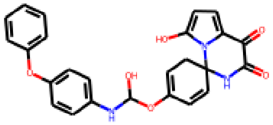	4.0	0.520676	2.336694	2.899742	5	0.820778
**3310**	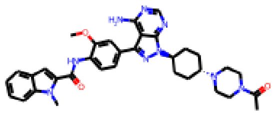	0.328338	Cc1ccc(C(=O)Nc2cccc(C(F)(F)F)c2C)cc1-c1ccc2oc(=O)cccc(F)c2n1	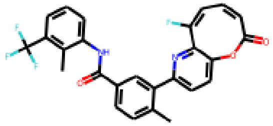	6.0	0.790896	3.985943	2.115897	4	0.783675
**3311**	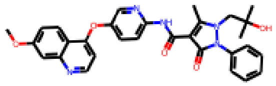	0.328223	COc1ccc2c(=O)cc(CNC(=O)c3c(C)n(C)n(-c4ccccc4)c3=O)nc3cccc2c3c1	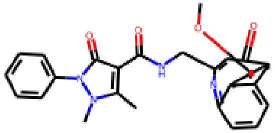	9.0	0.638163	3.995589	2.988471	5	0.774651

	src known mol	similarity	smiles	mol	Class	qed	logP	SAS	rings	kinase inhibition likeliness
**3312**	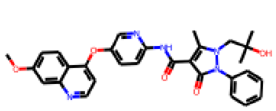	0.328223	COc1ccc2c(=O)cc(CNC(=O)c3c(C)n(C)n(-c4ccccc4)c3=O)nc3cccc2c3c1	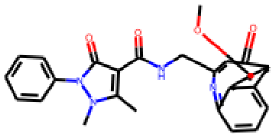	9.0	0.638163	3.995589	2.988471	5	0.774651
**3313**	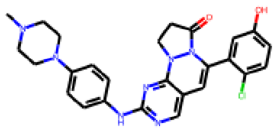	0.328078	O/C(Nc1ccc(Oc2ccccc2)cc1)Oc1ccc2(Cc1)NC(=O)/C(=O)c1ccc(O)n12	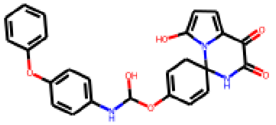	4.0	0.520676	2.336694	2.899742	5	0.820778
**3314**	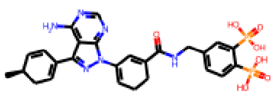	0.327715	C(N1[nH]c(C)c1CCN(c1cc(-c3nn(C)cc3-c3ccc(F)cc3)cc2)nc2C)n1	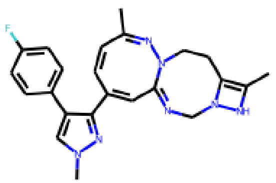	7.0	0.768132	3.608437	2.513170	5	0.754838
**3315**	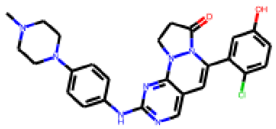	0.327526	Cc1nc(-c2ccc(C(=O)Oc3c(C)cccc3Cl)o2)cc(N2CCNC(=O)C2)n1	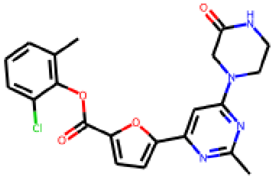	6.0	0.705736	3.500327	0.134263	4	0.764425

	src known mol	similarity	smiles	mol	Class	qed	logP	SAS	rings	kinase inhibition likeliness
**3316**	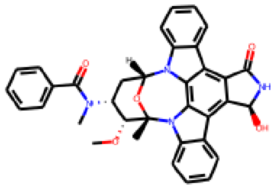	0.326608	COc1cc2c(=O)cc(CNC(=O)c3nn(-c4ccccc4C(C)(F)F)o3)c2o1	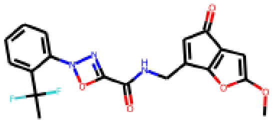	9.0	0.576217	4.086599	3.569904	4	0.783532
**3317**	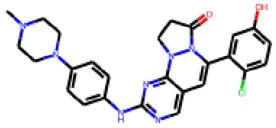	0.325624	O/C(Nc1ccc(Oc2ccccc2)cc1)Oc1ccc2(Cc1)NC(=O)/C(=O)c1ccc(O)n12	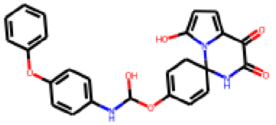	4.0	0.520676	2.336694	2.899742	5	0.820778
**3318**	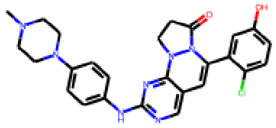	0.325624	O/C(Nc1ccc(Oc2ccccc2)cc1)Oc1ccc2(Cc1)NC(=O)/C(=O)c1ccc(O)n12	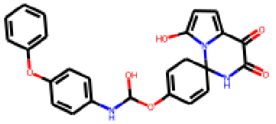	4.0	0.520676	2.336694	2.899742	5	0.820778
**3319**	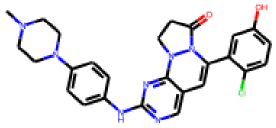	0.324193	O/C(Nc1ccc(Oc2ccccc2)cc1)Oc1ccc2(Cc1)NC(=O)/C(=O)c1ccc(O)n12	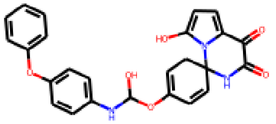	4.0	0.520676	2.336694	2.899742	5	0.820778

	src known mol	similarity	smiles	mol	Class	qed	logP	SAS	rings	kinase inhibition likeliness
**3320**	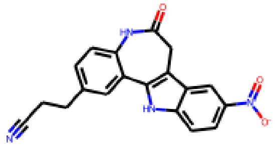	0.323843	CCNC(=O)N1CCC(Nc2n(C(C)c3c(-c4ccccc4)[nH]c4ccccc34)n2)CC1	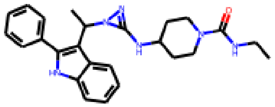	7.0	0.623584	3.603734	4.478009	5	0.804049
**3321**	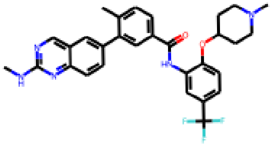	0.322823	Cc1ccc(C(=O)Nc2cccc(C(F)(C)F)c2C)cc1-c1ccc2oc(=O)cccc(F)c2c1	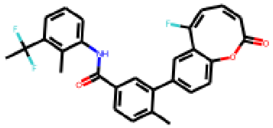	6.0	0.812693	3.141472	1.494671	4	0.774667
**3322**	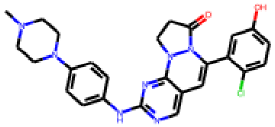	0.322384	O/C(Nc1ccc(Oc2ccccc2)cc1)Oc1ccc2(Cc1)NC(=O)/C(=O)c1ccc(O)n12	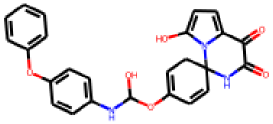	4.0	0.520676	2.336694	2.899742	5	0.820778
**3323**	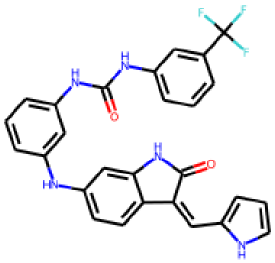	0.320755	COc1ccc(NC(=O)Nc2ccc3c(c2)NC(=O)/C3=C/c2ccc[nH]2)cc1	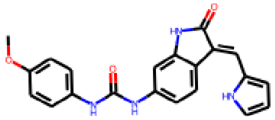	4.0	0.638163	3.995589	2.988471	4	0.768944

	src known mol	similarity	smiles	mol	Class	qed	logP	SAS	rings	kinase inhibition likeliness
**3324**	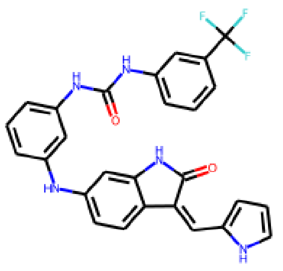	0.320755	COc1ccc(NC(=O)Nc2ccc3c(c2)NC(=O)/C3=C/c2ccc[nH]2)cc1	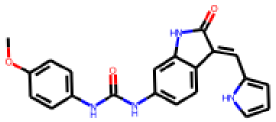	4.0	0.638163	3.995589	2.988471	4	0.768944
**3325**	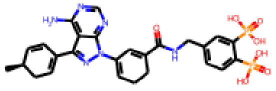	0.320425	C(N1[nH]c(C)c1CCN(c1cc(-c3nn(C)cc3-c3ccc(F)cc3)cc2)nc2C)n1	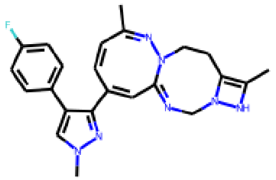	7.0	0.768132	3.608437	2.513170	5	0.754838
**3326**	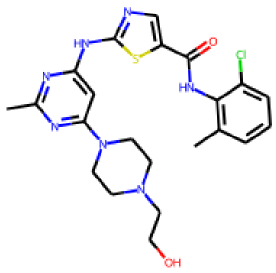	0.319772	Cc1nc(Nc2ccc(C(=O)Nc3c(C)cccc3Cl)s2)cc(N2CCNC(O)CC2)n1	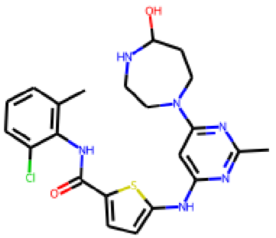	2.0	0.700685	3.487358	0.062602	4	0.770048
**3327**	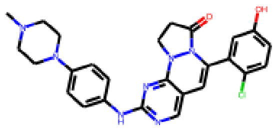	0.319512	O/C(Nc1ccc(Oc2ccccc2)cc1)Oc1ccc2(Cc1)NC(=O)/C(=O)c1ccc(O)n12	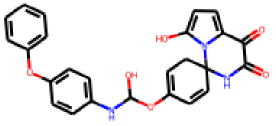	4.0	0.520676	2.336694	2.899742	5	0.820778

	src known mol	similarity	smiles	mol	Class	qed	logP	SAS	rings	kinase inhibition likeliness
**3328**	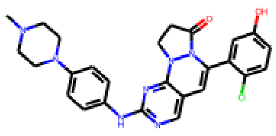	0.318972	Cc1nc(-c2ccc(C(=O)Oc3c(C)cccc3Cl)o2)cc(N2CCNC(=O)C2)n1	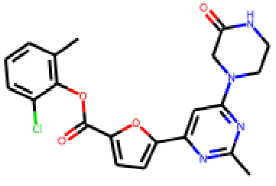	6.0	0.705736	3.500327	0.134263	4	0.764425
**3329**	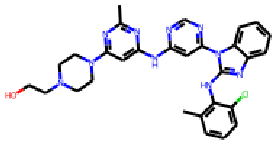	0.318820	Cc1nc(Nc2ccc(C(=O)Oc3c(C)cccc3Cl)s2)cc(N2CCNC(=O)C2=O)n1	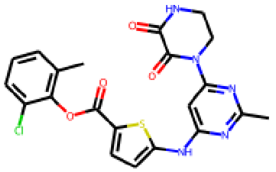	2.0	0.584673	2.312750	2.571988	4	0.770333
**3330**	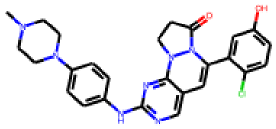	0.318681	O/C(Nc1ccc(Oc2ccccc2)cc1)Oc1ccc2(Cc1)NC(=O)/C(=O)c1ccc(O)n12	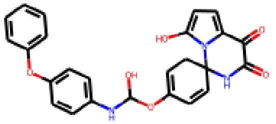	4.0	0.520676	2.336694	2.899742	5	0.820778
**3331**	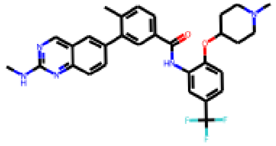	0.315516	Cc1ccc(C(=O)Nc2cccc(C(F)(C)F)c2C)cc1-c1ccc2oc(=O)cccc(F)c2c1	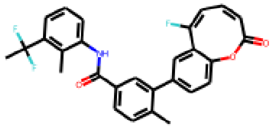	6.0	0.812693	3.141472	1.494671	4	0.774667

	src known mol	similarity	smiles	mol	Class	qed	logP	SAS	rings	kinase inhibition likeliness
**3332**	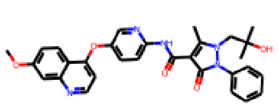	0.312387	COc1ccc2c(=O)cc(CNC(=O)c3c(C)n(C)n(-c4ccccc4)c3=O)nc3cccc3c2n1	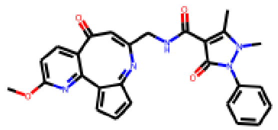	9.0	0.768132	3.608437	2.513170	5	0.823048
**3333**	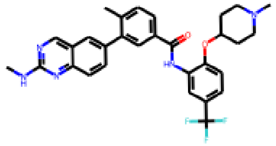	0.311524	Cc1ccc(C(=O)Nc2cccc(C(F)(C)F)c2C)cc1-c1ccc2oc(=O)cccc(F)c2c1	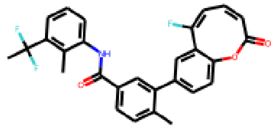	6.0	0.812693	3.141472	1.494671	4	0.774667
**3334**	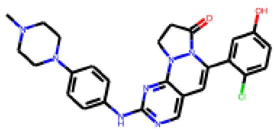	0.311281	Cc1nc(-c2ccc(C(=O)Oc3c(C)cccc3Cl)o2)cc(N2CCNC(=O)C2)n1	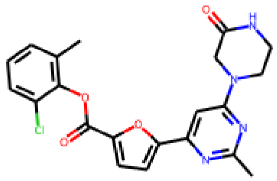	6.0	0.705736	3.500327	0.134263	4	0.764425
**3335**	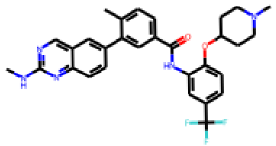	0.310225	Cc1ccc(C(=O)Nc2cccc(C(F)(F)F)c2C)cc1-c1ccc2oc(N)noc2c1	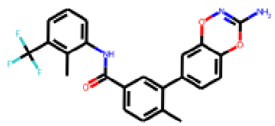	6.0	0.566600	4.200036	5.049812	4	0.784252

	src known mol	similarity	smiles	mol	Class	qed	logP	SAS	rings	kinase inhibition likeliness
**3336**	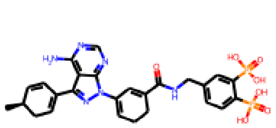	0.309091	C(N1[nH]c(C)c1CCN(c1cc(-c3nn(C)cc3-c3ccc(F)cc3)cc2)nc2C)n1	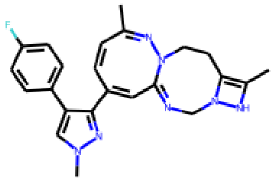	7.0	0.768132	3.608437	2.513170	5	0.754838
**3337**	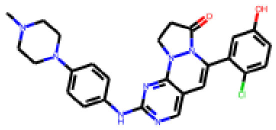	0.308555	Cc1nc(-c2ccc(C(=O)Oc3c(C)cccc3Cl)o2)cc(N2CCNC(=O)C2)n1	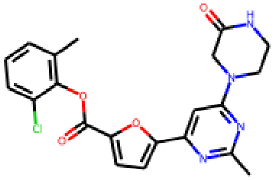	6.0	0.705736	3.500327	0.134263	4	0.764425
**3338**	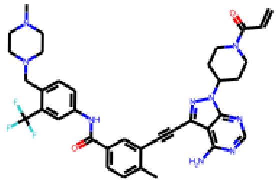	0.307902	Cc1ccc(C(=O)Nc2cccc(C(F)(C)F)c2C)cc1-c1ccc2nc(=O)ccc(C)cn2n1	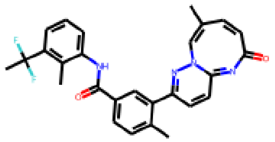	6.0	0.762994	3.476405	2.421118	4	0.764424
**3339**	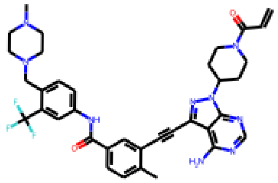	0.307902	Cc1ccc(C(=O)Nc2cccc(C(F)(C)F)c2C)cc1-c1ccc2nc(=O)ccc(C)cn2n1	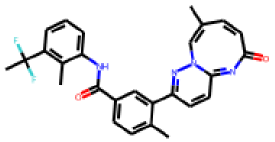	6.0	0.762994	3.476405	2.421118	4	0.764424

	src known mol	similarity	smiles	mol	Class	qed	logP	SAS	rings	kinase inhibition likeliness
**3340**	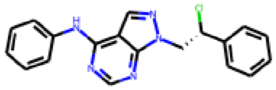	0.307747	Cc1ccc(C(=O)Nc2ccon2)cc1Nc1ncnc2c1cnn2C1cccccc1	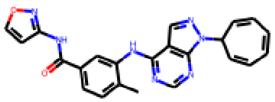	8.0	0.623362	2.525411	2.997881	5	0.770298
**3341**	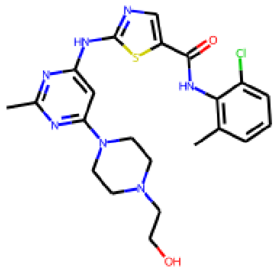	0.307138	Cc1nc(Nc2ccc(C(=O)Nc3c(C)cccc3Cl)s2)cc(N2CCNC(O)CC2)n1	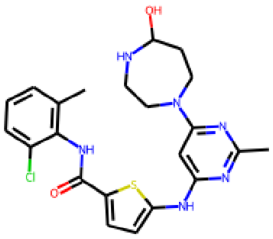	2.0	0.700685	3.487358	0.062602	4	0.770048
**3342**	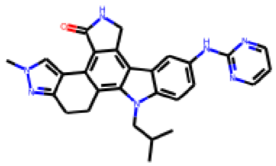	0.305890	Cc1ccc(C(=O)Nc2cccc(C(F)(C)F)c2C)cc1-c1ccc2nc(=O)cccc(F)nc2n1	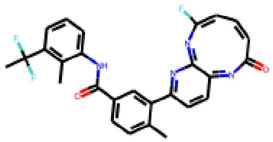	6.0	0.820294	3.038428	1.698172	4	0.833140
**3343**	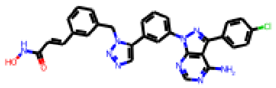	0.301611	Cc1ccc(C(=O)Nc2cscn2)cc1Nc1ncnc2c1cnn2-c1ccccc1	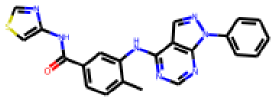	8.0	0.632663	4.308233	4.065316	5	0.804786

	src known mol	similarity	smiles	mol	Class	qed	logP	SAS	rings	kinase inhibition likeliness
**3344**	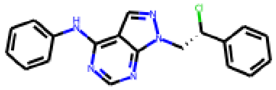	0.301213	Cc1ccc(C(=O)Nc2ccon2)cc1Nc1ncnc2c1cnn2C1cccccc1	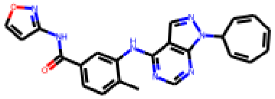	8.0	0.623362	2.525411	2.997881	5	0.770298
**3345**	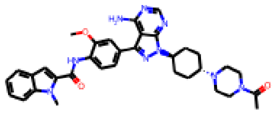	0.300824	Cc1ccc(C(=O)Nc2cccc(C(F)(F)F)c2C)cc1-c1ccc2oc(=O)cccc(F)c2n1	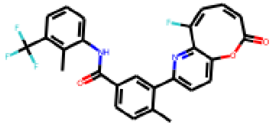	6.0	0.790896	3.985943	2.115897	4	0.783675
**3346**	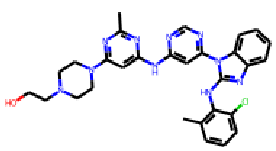	0.300773	Cc1nc(Nc2ccc(C(=O)Oc3c(C)cccc3Cl)s2)cc(N2CCNC(=O)C2=O)n1	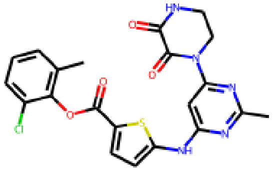	2.0	0.584673	2.312750	2.571988	4	0.770333
**3347**	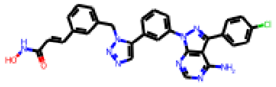	0.295505	Cc1ccc(C(=O)Nc2cscn2)cc1Nc1ncnc2c1cnn2-c1ccccc1	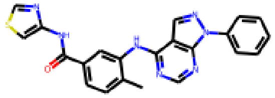	8.0	0.632663	4.308233	4.065316	5	0.804786

	src known mol	similarity	smiles	mol	Class	qed	logP	SAS	rings	kinase inhibition likeliness
**3348**	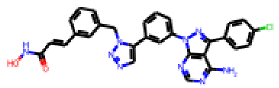	0.295505	Cc1ccc(C(=O)Nc2cscn2)cc1Nc1ncnc2c1cnn2-c1ccccc1	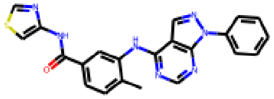	8.0	0.632663	4.308233	4.065316	5	0.804786
**3349**	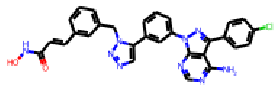	0.294161	Cc1ccc(C(=O)Nc2cscn2)cc1Nc1ncnc2c1cnn2-c1ccccc1	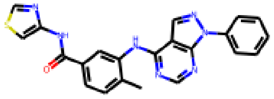	8.0	0.632663	4.308233	4.065316	5	0.804786
**3350**	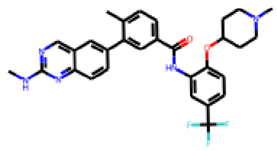	0.292994	Cc1ccc(C(=O)Nc2cccc(C(F)(F)F)c2C)cc1-c1ccc2oc(N)noc2c1	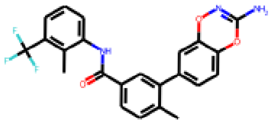	6.0	0.566600	4.200036	5.049812	4	0.784252
**3351**	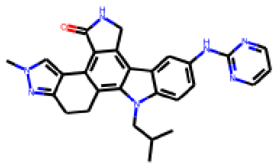	0.292339	C#Cc1ccc(C(=O)OC)cc1-c1C(=O)c-n2nc(-c3ccc(F)cc3F)cc2C1	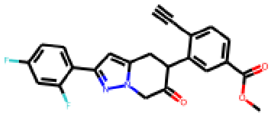	8.0	0.768132	3.608437	2.513170	4	0.763730

	src known mol	similarity	smiles	mol	Class	qed	logP	SAS	rings	kinase inhibition likeliness
**3352**	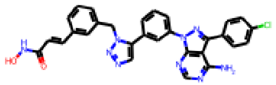	0.289532	Cc1ccc(C(=O)Nc2cscn2)cc1Nc1ncnc2c1cnn2-c1ccccc1	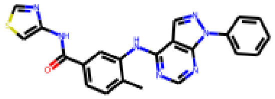	8.0	0.632663	4.308233	4.065316	5	0.804786
**3353**	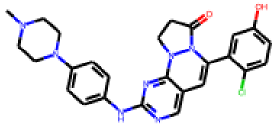	0.287037	Cc1nc(-c2ccc(C(=O)Oc3c(C)cccc3Cl)o2)cc(N2CCNC(=O)C2)n1	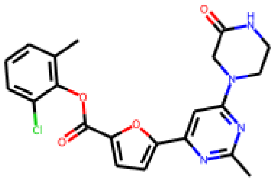	6.0	0.705736	3.500327	0.134263	4	0.764425
**3354**	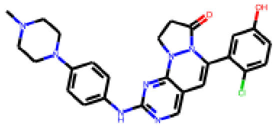	0.283774	Cc1nc(-c2ccc(C(=O)Oc3c(C)cccc3Cl)o2)cc(N2CCNC(=O)C2)n1	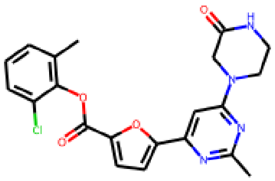	6.0	0.705736	3.500327	0.134263	4	0.764425
**3355**	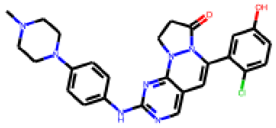	0.280374	Cc1nc(-c2ccc(C(=O)Oc3c(C)cccc3Cl)o2)cc(N2CCNC(=O)C2)n1	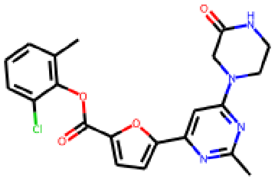	6.0	0.705736	3.500327	0.134263	4	0.764425

	src known mol	similarity	smiles	mol	Class	qed	logP	SAS	rings	kinase inhibition likeliness
**3356**	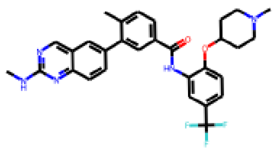	0.279809	Cc1ccc(C(=O)Nc2cccc(C(C)(C)F)c2C)cc1-c1ccc2oc(O)ccccc(F)c2c1	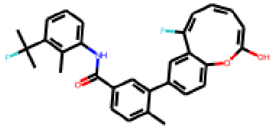	6.0	0.600657	4.044744	5.260394	4	0.788333
**3357**	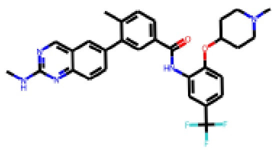	0.279809	Cc1ccc(C(=O)Nc2cccc(C(C)(C)F)c2C)cc1-c1ccc2oc(O)ccccc(F)c2c1	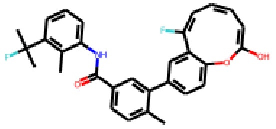	6.0	0.600657	4.044744	5.260394	4	0.788333
**3358**	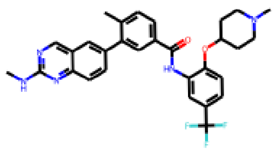	0.278823	Cc1ccc(C(=O)Nc2cccc(C(C)(C)F)c2C)cc1-c1ccc2oc(O)ccccc(F)c2c1	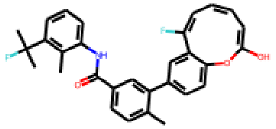	6.0	0.600657	4.044744	5.260394	4	0.788333
**3359**	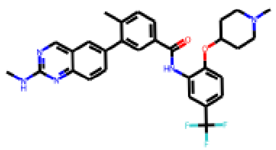	0.278823	Cc1ccc(C(=O)Nc2cccc(C(C)(C)F)c2C)cc1-c1ccc2oc(O)ccccc(F)c2c1	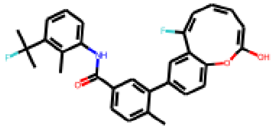	6.0	0.600657	4.044744	5.260394	4	0.788333

	src known mol	similarity	smiles	mol	Class	qed	logP	SAS	rings	kinase inhibition likeliness
**3360**	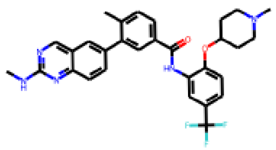	0.273086	Cc1ccc(C(=O)Nc2cccc(C(F)(C)F)c2C)cc1-c1ccc2oc(=O)cccc(F)c2c1	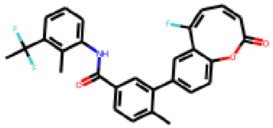	6.0	0.812693	3.141472	1.494671	4	0.774667
**3361**	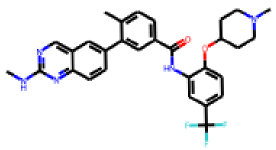	0.249206	Cc1ccc(C(=O)Nc2cccc(C(C)(F)F)c2C)cc1-c1ccc2oc(=O)cccc(F)c2c1	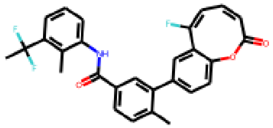	6.0	0.727982	2.869569	2.076397	4	0.768016
**3362**	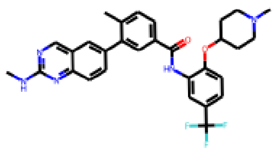	0.242857	Cc1ccc(C(=O)Nc2cccc(C(F)(C)F)c2C)cc1-c1ccc2oc(=O)cccc(F)c2c1	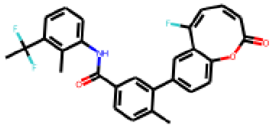	6.0	0.812693	3.141472	1.494671	4	0.774667
**3363**	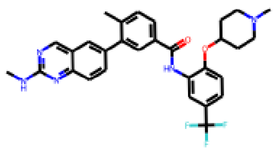	0.239808	Cc1ccc(C(=O)Nc2cccc(C(F)(C)F)c2C)cc1-c1ccc2oc(=O)cccc(F)c2c1	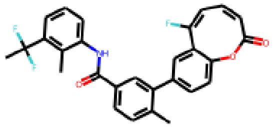	6.0	0.812693	3.141472	1.494671	4	0.774667

	src known mol	similarity	smiles	mol	Class	qed	logP	SAS	rings	kinase inhibition likeliness
**3364**	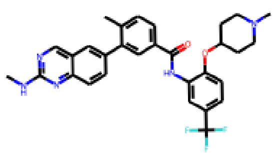	0.235199	Cc1ccc(C(=O)Nc2cccc(C(F)(C)F)c2C)cc1-c1ccc2oc(=O)cccc(F)c2c1	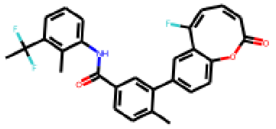	6.0	0.812693	3.141472	1.494671	4	0.774667
**3365**	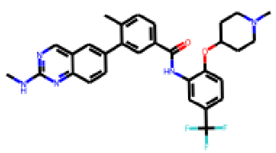	0.230333	Cc1ccc(C(=O)Nc2cccc(C(F)(C)F)c2C)cc1-c1ccc2oc(=O)cccc(F)c2c1	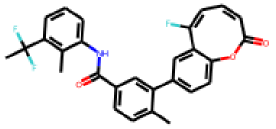	6.0	0.812693	3.141472	1.494671	4	0.774667
**3366**	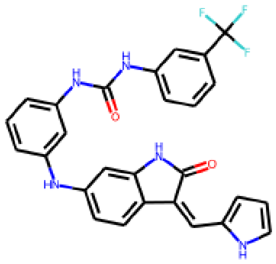	0.226891	COc1ccc(NC(=O)Nc2ccc3c(c2)NC(=O)/C3=C/c2ccc[nH]2)cc1	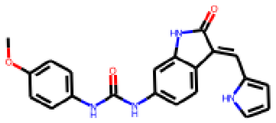	4.0	0.638163	3.995589	2.988471	4	0.768944
