# Supplementary material for: Interpretable Machine Learning Models for Molecular Design of Tyrosine Kinase Inhibitors Using Variational Autoencoders and Perturbation-Based Approach of Chemical Space Exploration
Source: Int J Mol Sci. 2022 Sep 24;23(19):11262. doi: 10.3390/ijms231911262 (PMC9569663; doi:10.3390/ijms231911262)
Supplement: Supplementary file 1 [file ijms-23-11262-s001.zip › SUPPLEMENTARY_MATERIALS/Supplemental_Info_Guide.docx]

**Supplemental Information Guide**

Folders within the Directory:

**Generated_Molecules_With_Similarity**:

- final_similarity_data.pdf: Data File of Generated Molecules originating from SRC Kinase Inhibitors and the known SRC Kinase Inhibitor it is closest to.

Columns: SRC Mol (Known SRC Kinase Inhibitor)

Similarity (similarity score)

Mol (Generated Novel Molecule)

smiles (SMILE string of generated molecule)

qed (QED value of generated molecule)

logP (logP value of generated molecule)

SAS (SAS value of generated molecule)

rings (number of rings for generated molecule)

kinase inhibition likeliness (kinase inhibition score of generated molecule)

- final_similarity_data_kinase_family (1).pdf: Data File of Similarity with Generated Molecules originating from various Kinase Inhibitor families and the known SRC Kinase Inhibitor it is closest to.

Columns: SRC Mol (Known SRC Kinase Inhibitor)

Similarity (similarity score of generated molecule to known SRC Kinase Inhibitor)

smiles (SMILE string of generated molecule)

Mol (Generated Novel Molecule)

Class (Originating Kinase Family):

0: ABL1

2: EGFR

3: CSF1R

4: FLT3

5: KDR

6: LCK

7: MAPK10

8: MAPK14

9: MET

qed (QED value of generated molecule)

logP (logP value of generated molecule)

SAS (SAS value of generated molecule)

rings (number of rings for generated molecule)

kinase inhibition likeliness (kinase inhibition score of generated molecule)

**Original_Set_of_Generated_Molecules:**

- Original_Set.csv: Original set of generated molecules produced from kinase families

Columns: smiles (SMILE strings for generated molecules)

distance (how far the new encoding is from the original encoding)

count (number of each SMILE string produced)

mol (generated molecule image path)

Class (Originating Kinase Family):

0: ABL1

2: EGFR

3: CSF1R

4: FLT3

5: KDR

6: LCK

7: MAPK10

8: MAPK14

9: MET

qed (QED value of generated molecule)

logP (logP value of generated molecule)

SAS (SAS value of generated molecule)

- All_Uniques.csv: unique set of generated molecules produced by originating families— created from Original_Set,csv

Columns: same as above, but includes structural and chemical properties calculated by RDKit

smiles (SMILE strings for generated molecules)

distance (how far the new encoding is from the original encoding)

count (number of each SMILE string produced)

mol (generated molecule image path)

Class (Originating Kinase Family):

0: ABL1

2: EGFR

3: CSF1R

4: FLT3

5: KDR

6: LCK

7: MAPK10

8: MAPK14

9: MET

Characteristics:

rings

weight

csp3

hallkier count

laputeASA

alicarbo

alihetero

alirings

amide bonds

arocarbo

arohetero

arorings

stereocenter

bridgehead

hba

hbd

rotatable

- Over_75_Kinase.csv: generated molecules that have a kinase score of 0.75 or above

Columns: smiles (SMILE strings for generated molecules)

distance (how far the new encoding is from the original encoding)

count (number of each SMILE string produced)

mol (generated molecule image path)

Class (Originating Kinase Family):

0: ABL1

2: EGFR

3: CSF1R

4: FLT3

5: KDR

6: LCK

7: MAPK10

8: MAPK14

9: MET

Characteristics:

rings

weight

csp3

hallkier count

laputeASA

alicarbo

alihetero

alirings

amide bonds

arocarbo

arohetero

arorings

stereocenter

bridgehead

hba

hbd

rotatable

Kinase Inhibition Likeliness

- Top_100_Mol.csv: top 100 generated molecules with highest similarity to known SRC kinase inhibitors

Columns: Known Mol (SRC Known Kinase Inhibitor image path)

similarity (similarity score of generated molecule to known SRC Kinase Inhibitor)

smiles (SMILE strings for generated molecules)

distance (how far the new encoding is from the original encoding)

count (number of each SMILE string produced)

mol (generated molecule image path)

Class (Originating Kinase Family):

0: ABL1

2: EGFR

3: CSF1R

4: FLT3

5: KDR

6: LCK

7: MAPK10

8: MAPK14

9: MET

Characteristics:

rings

weight

csp3

hallkier count

laputeASA

alicarbo

alihetero

alirings

amide bonds

arocarbo

arohetero

arorings

stereocenter

bridgehead

hba

hbd

rotatable

Kinase Inhibition Likeliness

**Prelim_Data_Generated_Molecules_from_SRC_Inhibitors: Initial data from the original experiment- generation of novel molecules from SRC Kinase Inhibitors**

- Top_100_Cluster_1.xlsx: Top 100 Generated Molecules from cluster 1 for initial experiments- generation of novel molecules from SRC Kinase Inhibitors

Columns: Mol (generated molecule image)

smiles (SMILE strings for generated molecules)

distance (how far the new encoding is from the original encoding)

count (number of each SMILE string produced)

Characteristics:

rings

weight

csp3

hallkier count

laputeASA

alicarbo

alihetero

alirings

amide bonds

arocarbo

arohetero

arorings

stereocenter

bridgehead

hba

hbd

rotatable

Kinase Inhibition Likeliness

- Top_100_Cluster_2.xlsx: Top 100 Generated Molecules from cluster 2 for initial experiments- generation of novel molecules from SRC Kinase Inhibitors

Columns: Mol (generated molecule image)

smiles (SMILE strings for generated molecules)

distance (how far the new encoding is from the original encoding)

count (number of each SMILE string produced)

Characteristics:

rings

weight

csp3

hallkier count

laputeASA

alicarbo

alihetero

alirings

amide bonds

arocarbo

arohetero

arorings

stereocenter

bridgehead

hba

hbd

rotatable

Kinase Inhibition Likeliness

- 2_Cluster_Top_100_1.csv & 2_Cluster_Top_100_2.csv: csv files of excel sheets

**Graphs_OF_Chemical_Proprties:** graphs of chemical properties — average values of properties for generated molecules by originating families

- alicarbo_bar: average aliphatic carbocycles of generated molecules by originating family

- alihetero_bar: average aliphatic heterocycles of generated molecules by originating family

- alirings_bar: average aliphatic rings of generated molecules by originating family

- arocarbo_bar: average aromatic carbocycles of generated molecules by originating family

- arohetero_bar: average aromatic heterocycles of generated molecules by originating family

- arorings_bar: average aromatic rings of generated molecules by originating family

- csp3_bar: average CSP3 value of generated molecules by originating family

- hallkier_bar: average hallkier count of generated molecules by originating family

- labuteasa_bar: average labuteasa value of generated molecules by originating family

- weight_bar: average molecular weight of generated molecules by originating family

- rotatable_bar: average rotatable bonds of generated molecules by originating family

**Histogram_Distribution_Of_Chemical_Properties**: Histogram distribution of properties for all molecules in the kinase families used for the training set- initial chemical analysis
