# Supplementary material for: Interpretable Machine Learning Models for Molecular Design of Tyrosine Kinase Inhibitors Using Variational Autoencoders and Perturbation-Based Approach of Chemical Space Exploration
Source: Int J Mol Sci. 2022 Sep 24;23(19):11262. doi: 10.3390/ijms231911262 (PMC9569663; doi:10.3390/ijms231911262)
Supplement: Supplementary file 1 [file ijms-23-11262-s001.zip › SUPPLEMENTARY_MATERIALS/Supplemental_Materials.docx]

Interpretable Machine Learning Models for Molecular Design of Tyrosine Kinase Inhibitors Using Variational Autoencoders and Perturbation-Based Approach of Chemical Space Exploration

Keerthi Krishnan,^1^ Ryan Kassab^1^, Steve Agajanian^1^, Gennady Verkhivker,^1,2^*

^1^ Keck Center for Science and Engineering, Graduate Program in Computational and Data Sciences, Schmid

College of Science and Technology, Chapman University, Orange, CA 92866, United States of America

^2^ Department of Biomedical and Pharmaceutical Sciences, Chapman University School of Pharmacy, Irvine, CA 92618, United States of America

***** Correspondence: verkhivk@chapman.edu; Tel.: +1-714-516-4586 (G.V)

Received: date; Accepted: date; Published: date

**Table S1.** Statistical Distributions of the Kinase Inhibitors for Different Families in the Latent Space

| **Kinase family** | Minimum range | Maximum range | Minimum  average | Maximum average | Minimum std | Maximum std |
| --- | --- | --- | --- | --- | --- | --- |
| **ABL1** | -5.89 | 5.97 | -1.34 | 1.26 | 0.78 | 1.46 |
| **SRC** | -5.89 | 6.20 | -1.38 | 1.30 | 0.86 | 1.63 |
| **CSF1R** | -5.19 | 6.84 | -1.19 | 1.21 | 0.65 | 1.46 |
| **EGFR** | -6.18 | 6.55 | -1.25 | 1.22 | 0.82 | 1.39 |
| **FLT3** | -5.00 | 6.45 | -1.17 | 1.15 | 0.69 | 1.42 |
| **KDR** | -6.15 | 7.05 | -1.37 | 1.32 | 0.80 | 1.35 |
| **LCK** | -6.15 | 6.62 | -1.38 | 1.39 | 0.81 | 1.55 |
| **MAPK10** | -5.08 | 5.98 | -1.16 | 1.14 | 0.68 | 1.29 |
| **MAPK14** | -6.15 | 6.89 | -1.52 | 1.44 | 0.73 | 1.29 |
| **MET** | -6.13 | 6.49 | -1.45 | 1.52 | 0.79 | 1.53 |

**Table S2**. Binary Latent Space-Based Random Forest Classification Model

| Class | Precision | Recall | F1-Score | Specificity | Sensitivity |
| --- | --- | --- | --- | --- | --- |
| Non-SRC molecules (class 0) | 0.98 | 0.99 | 0.99 | 0.99 |  |
| SRC kinase molecules  (class 1) | 0.57 | 0.31 | 0.40 |  | 0.31 |

**Table S3.** Confusion Matrix for Latent Space Binary Classification Model

| **Class** | Non-SRC molecules  (class 0) | SRC kinase molecules  (class 1) |
| --- | --- | --- |
| Non-SRC molecules  (class 0) | TN: 26823 | FN: 497 |
| SRC kinase molecules  (class 1) | FP: 170 | TP: 221 |

Table S4. Binary Chemical Feature-Based Classification Model

| Class | Precision | Recall | F1-Score | Specificity | Sensitivity |
| --- | --- | --- | --- | --- | --- |
| Non-SRC molecules  (class 0) | 0.99 | 0.98 | 0.98 | 0.98 |  |
| SRC kinase molecules (class 1) | 0.73 | 0.81 | 0.77 |  | 0.81 |

Table S5. Confusion Matrix for Binary Chemical Feature-Based Classification Model

| **Class** | Non-SRC molecules  (class 0) | SRC kinase molecules  (class 1) |
| --- | --- | --- |
| Non-SRC molecules  (class 0) | TN: 23052 | FN: 284 |
| SRC kinase molecules  (class 1) | FP: 454 | TP: 1242 |

**Table S6**. Multiclass Classification Latent Space-Based Random Classification Report Results

|  | Precision | Recall | F1-Score |
| --- | --- | --- | --- |
| ABL1 | 0.50 | 0.53 | 0.51 |
| SRC | 0.56 | 0.51 | 0.53 |
| CSF1R | 0.46 | 0.24 | 0.32 |
| EGFR | 0.60 | 0.69 | 0.64 |
| FLT3 | 0.36 | 0.16 | 0.22 |
| KDR | 0.42 | 0.59 | 0.49 |
| LCK | 0.44 | 0.30 | 0.36 |
| MAPK10 | 0.73 | 0.30 | 0.43 |
| MAPK14 | 0.66 | 0.73 | 0.69 |
| MET | 0.76 | 0.55 | 0.63 |

**Table S7**. Multiclass Classification Chemical Feature-Based Classification Report Results

|  | Precision | Recall | F1-Score |
| --- | --- | --- | --- |
| ABL1 | 0.51 | 0.58 | 0.55 |
| SRC | 0.57 | 0.56 | 0.56 |
| CSF1R | 0.69 | 0.54 | 0.61 |
| EGFR | 0.69 | 0.74 | 0.71 |
| FLT3 | 0.55 | 0.46 | 0.50 |
| KDR | 0.58 | 0.59 | 0.58 |
| LCK | 0.47 | 0.41 | 0.44 |
| MAPK10 | 0.77 | 0.55 | 0.64 |
| MAPK14 | 0.75 | 0.80 | 0.78 |
| MET | 0.74 | 0.72 | 0.73 |

**Table S8.** The average values of the physicochemical properties for the generated molecules across kinase families. The five physicochemical properties are as follows: average number of hydrogen bond acceptors (HBA), average number of hydrogen bond donors (HBD), average number of aromatic rings, average molecular weight, and average number of rotatable bonds.

| **Family** | **Number of**  **HBA** | **Number of**  **HBD** | **Number of Aromatic Rings** | **Molecular Weight (Daltons)** | **Number of Rotatable Bonds** |
| --- | --- | --- | --- | --- | --- |
| ABL1 | 6 | 2 | 4 | 347 | 4 |
| CSF1R | 7 | 2 | 3 | 444 | 5 |
| EGFR | 5 | 3 | 3 | 431 | 5 |
| FLT3 | 5 | 3 | 4 | 429 | 4 |
| KDR | 5 | 3 | 4 | 439 | 5 |
| LCK | 6 | 2 | 3 | 436 | 4 |
| MAPK10 | 6 | 2 | 3 | 426 | 6 |
| MAPK14 | 7 | 1 | 4 | 406 | 5 |
| MET | 6 | 1 | 3 | 401 | 4 |
