# Supplementary material for: Interpretable Machine Learning Models for Molecular Design of Tyrosine Kinase Inhibitors Using Variational Autoencoders and Perturbation-Based Approach of Chemical Space Exploration
Source: Int J Mol Sci. 2022 Sep 24;23(19):11262. doi: 10.3390/ijms231911262 (PMC9569663; doi:10.3390/ijms231911262)
Supplement: Supplementary file 1 [file ijms-23-11262-s001.zip › SUPPLEMENTARY_MATERIALS/Supplemental_Materials.pdf]

Interpretable Machine Learning Models for Molecular Design of Tyrosine Kinase Inhibitors Using Variational Autoencoders and Perturbation-Based Approach of Chemical Space Exploration

Keerthi Krishnan,[1] Ryan Kassab[1], Steve Agajanian[1], Gennady Verkhivker,[1,2*]

[1] Keck Center for Science and Engineering, Graduate Program in Computational and Data Sciences, Schmid College of Science and Technology, Chapman University, Orange, CA 92866, United States of America

[2] Department of Biomedical and Pharmaceutical Sciences, Chapman University School of Pharmacy, Irvine, CA 92618, United States of America

* Correspondence: verkhivk@chapman.edu; Tel.: +1-714-516-4586 (G.V)

Received: date; Accepted: date; Published: date

Table S1. Statistical Distributions of the Kinase Inhibitors for Different Families in the Latent Space

**Kinase family**	Minimum range	Maximum range	Minimum average	Maximum average	Minimum std	Maximum std
**ABL1**	-5.89	5.97	-1.34	1.26	0.78	1.46
**SRC**	-5.89	6.20	-1.38	1.30	0.86	1.63
**CSF1R**	-5.19	6.84	-1.19	1.21	0.65	1.46
**EGFR**	-6.18	6.55	-1.25	1.22	0.82	1.39
**FLT3**	-5.00	6.45	-1.17	1.15	0.69	1.42
**KDR**	-6.15	7.05	-1.37	1.32	0.80	1.35
**LCK**	-6.15	6.62	-1.38	1.39	0.81	1.55
**MAPK10**	-5.08	5.98	-1.16	1.14	0.68	1.29
**MAPK14**	-6.15	6.89	-1.52	1.44	0.73	1.29
**MET**	-6.13	6.49	-1.45	1.52	0.79	1.53

Table S2. Binary Latent Space-Based Random Forest Classification Model

Class	Precision	Recall	F1-Score	Specificity	Sensitivity
Non-SRC molecules (class 0)	0.98	0.99	0.99	0.99	
SRC kinase molecules (class 1)	0.57	0.31	0.40		0.31

Table S3. Confusion Matrix for Latent Space Binary Classification Model

**Class**	Non-SRC molecules (class 0)	SRC kinase molecules (class 1)
Non-SRC molecules (class 0)	TN: 26823	FN: 497
SRC kinase molecules (class 1)	FP: 170	TP: 221

Table S4. Binary Chemical Feature-Based Classification Model

**Class**	**Precision**	**Recall**	**F1-Score**	**Specificity**	**Sensitivity**
**Non-SRC molecules (class 0)**	**0.99**	**0.98**	**0.98**	**0.98**	
**SRC kinase molecules (class 1)**	**0.73**	**0.81**	**0.77**		**0.81**

Table S5. Confusion Matrix for Binary Chemical Feature-Based Classification Model

**Class**	Non-SRC molecules (class 0)	SRC kinase molecules (class 1)
Non-SRC molecules (class 0)	TN: 23052	FN: 284
SRC kinase molecules (class 1)	FP: 454	TP: 1242

Table S6. Multiclass Classification Latent Space-Based Random Classification Report Results

	**Precision**	**Recall**	**F1-Score**
**ABL1**	0.50	0.53	0.51
**SRC**	0.56	0.51	0.53
**CSF1R**	0.46	0.24	0.32
**EGFR**	0.60	0.69	0.64
**FLT3**	0.36	0.16	0.22
**KDR**	0.42	0.59	0.49
**LCK**	0.44	0.30	0.36
**MAPK10**	0.73	0.30	0.43
**MAPK14**	0.66	0.73	0.69
**MET**	0.76	0.55	0.63

Table S7. Multiclass Classification Chemical Feature-Based Classification Report Results

	**Precision**	**Recall**	**F1-Score**
**ABL1**	0.51	0.58	0.55
**SRC**	0.57	0.56	0.56
**CSF1R**	0.69	0.54	0.61
**EGFR**	0.69	0.74	0.71
**FLT3**	0.55	0.46	0.50
**KDR**	0.58	0.59	0.58
**LCK**	0.47	0.41	0.44
**MAPK10**	0.77	0.55	0.64
**MAPK14**	0.75	0.80	0.78
**MET**	0.74	0.72	0.73

Table S8. The average values of the physicochemical properties for the generated molecules across kinase families. The five physicochemical properties are as follows: average number of hydrogen bond acceptors (HBA), average number of hydrogen bond donors (HBD), average number of aromatic rings, average molecular weight, and average number of rotatable bonds.

**Family**	**Number of HBA**	**Number of HBD**	**Number of Aromatic Rings**	**Molecular Weight (Daltons)**	**Number of Rotatable Bonds**
ABL1	6	2	4	347	4
CSF1R	7	2	3	444	5
EGFR	5	3	3	431	5
FLT3	5	3	4	429	4
KDR	5	3	4	439	5
LCK	6	2	3	436	4
MAPK10	6	2	3	426	6
MAPK14	7	1	4	406	5
MET	6	1	3	401	4
