# Supplementary figures and images for: Interpretable Machine Learning Models for Molecular Design of Tyrosine Kinase Inhibitors Using Variational Autoencoders and Perturbation-Based Approach of Chemical Space Exploration
Source: Int J Mol Sci. 2022 Sep 24;23(19):11262. doi: 10.3390/ijms231911262 (PMC9569663; doi:10.3390/ijms231911262)

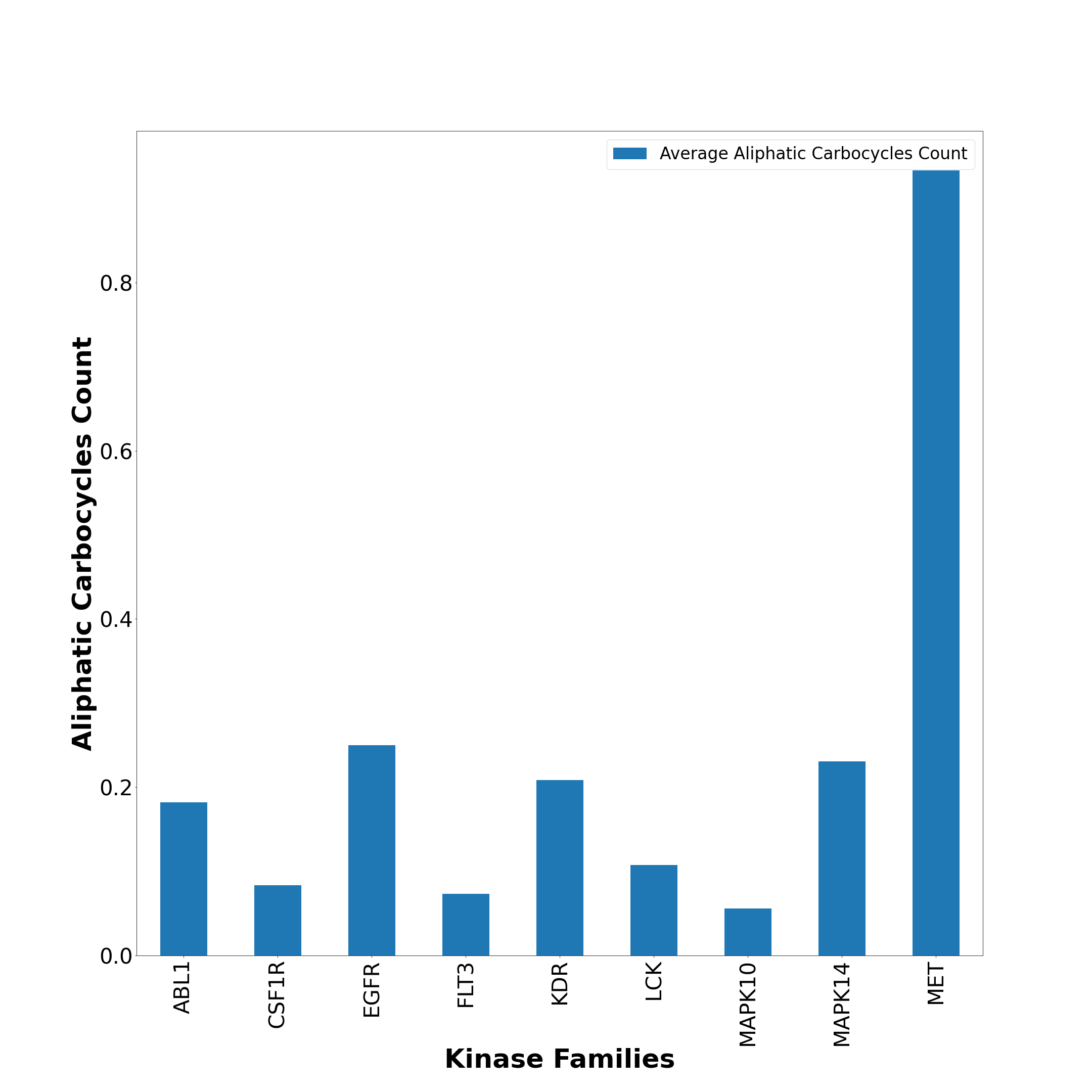

Supplement: Supplementary file 1 [file ijms-23-11262-s001.zip › SUPPLEMENTARY_MATERIALS/Graphs_of_Chemical_Properties/alicarbo_bar.png]

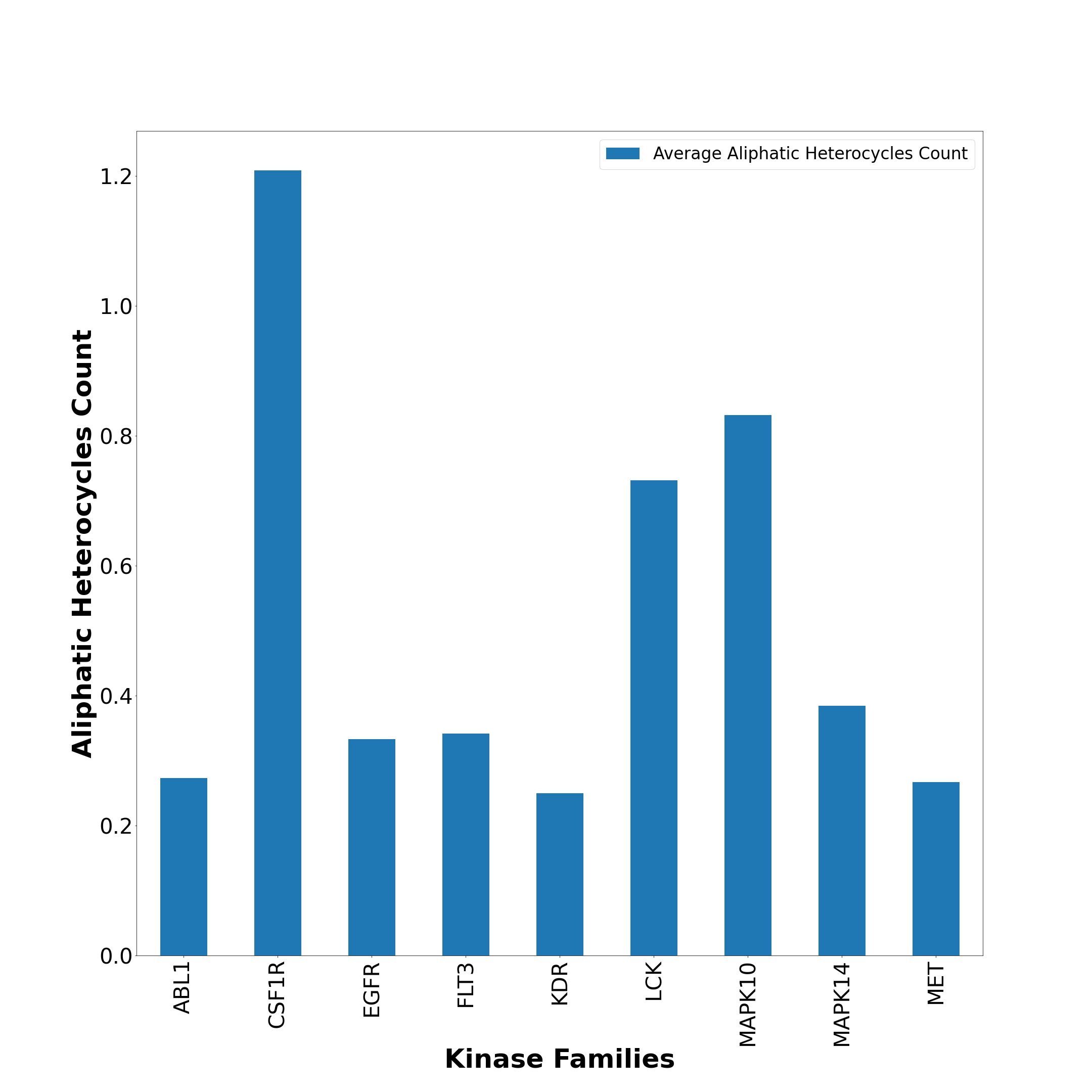

Supplement: Supplementary file 1 [file ijms-23-11262-s001.zip › SUPPLEMENTARY_MATERIALS/Graphs_of_Chemical_Properties/alihetero_bar.png]

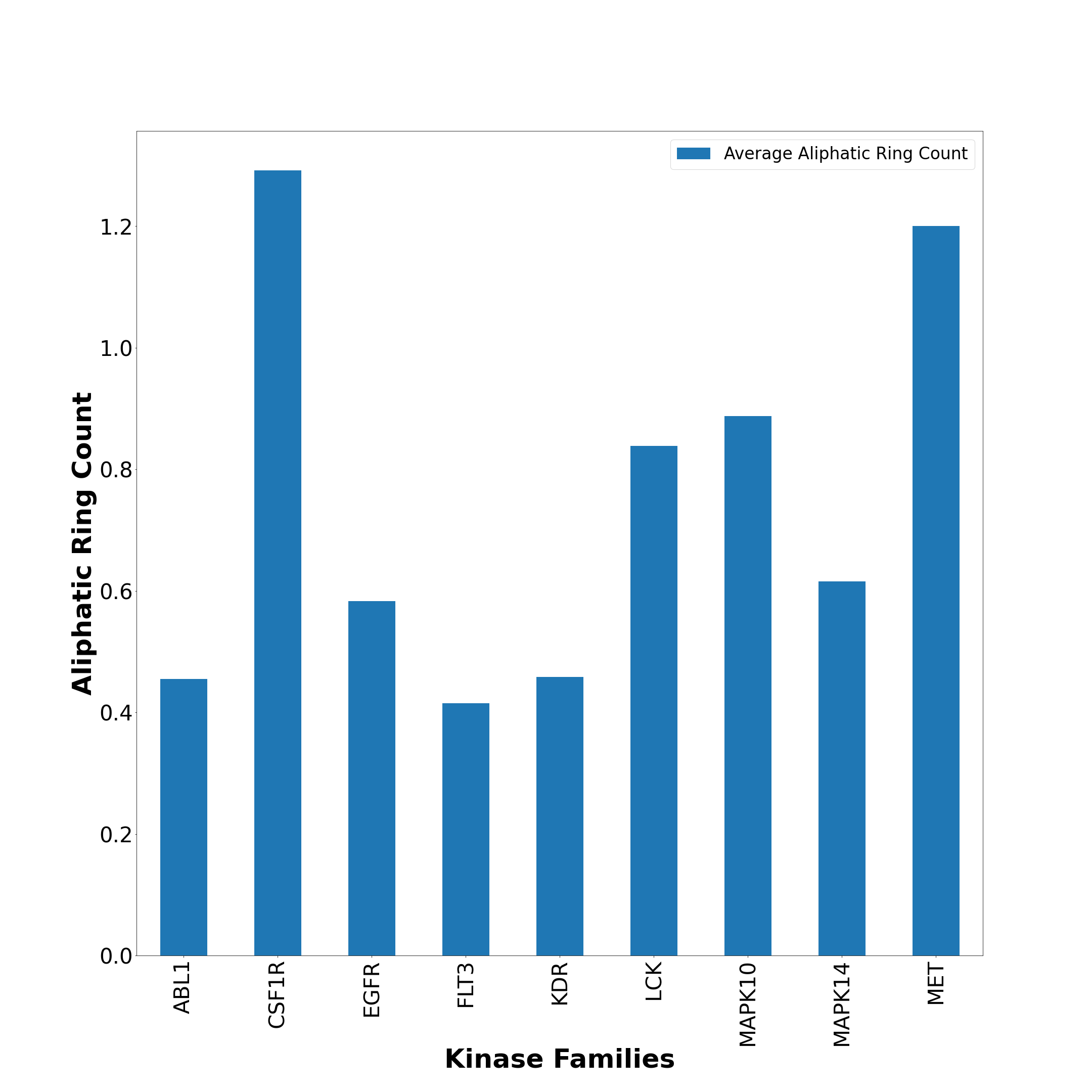

Supplement: Supplementary file 1 [file ijms-23-11262-s001.zip › SUPPLEMENTARY_MATERIALS/Graphs_of_Chemical_Properties/alirings_bar.png]

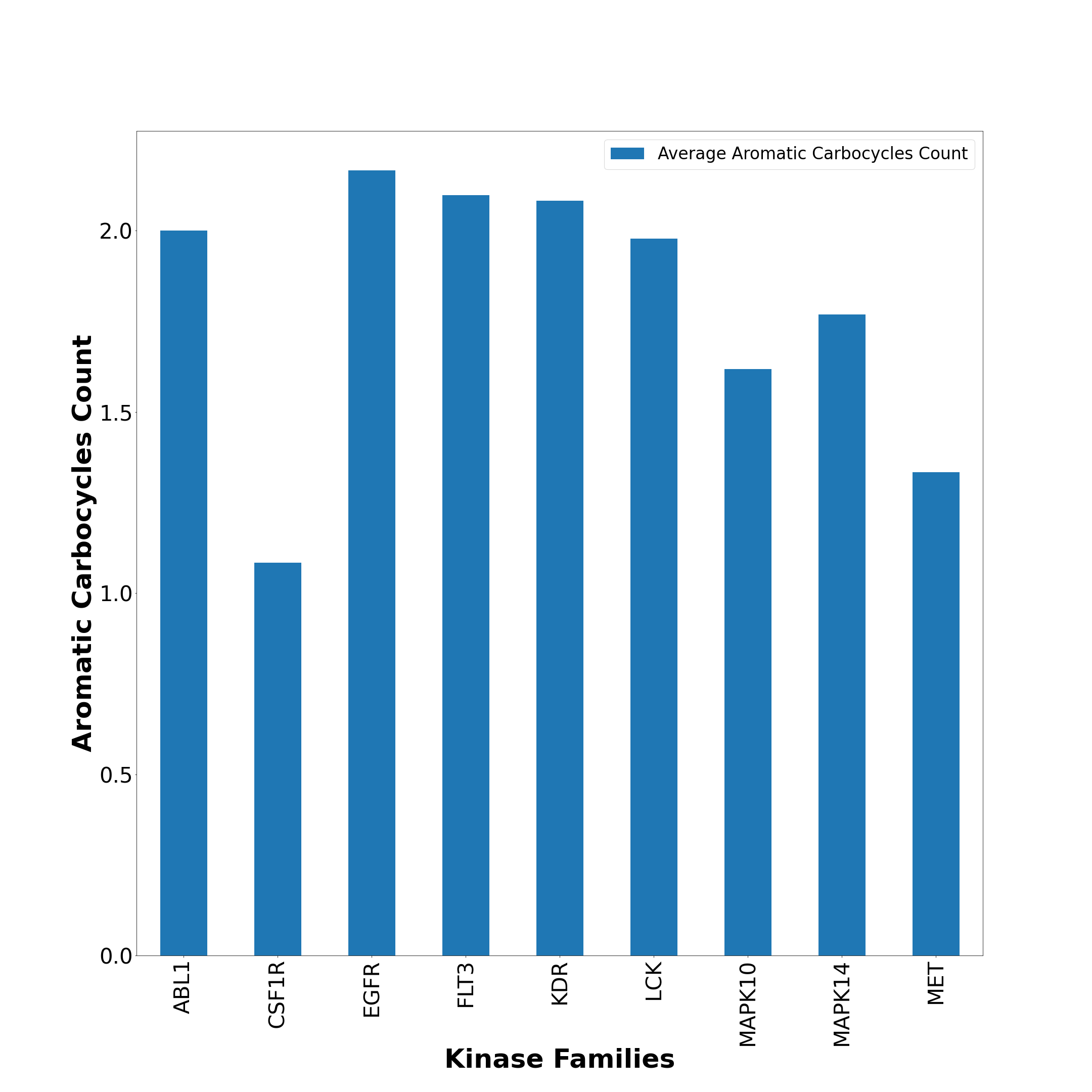

Supplement: Supplementary file 1 [file ijms-23-11262-s001.zip › SUPPLEMENTARY_MATERIALS/Graphs_of_Chemical_Properties/arocarbo_bar.png]

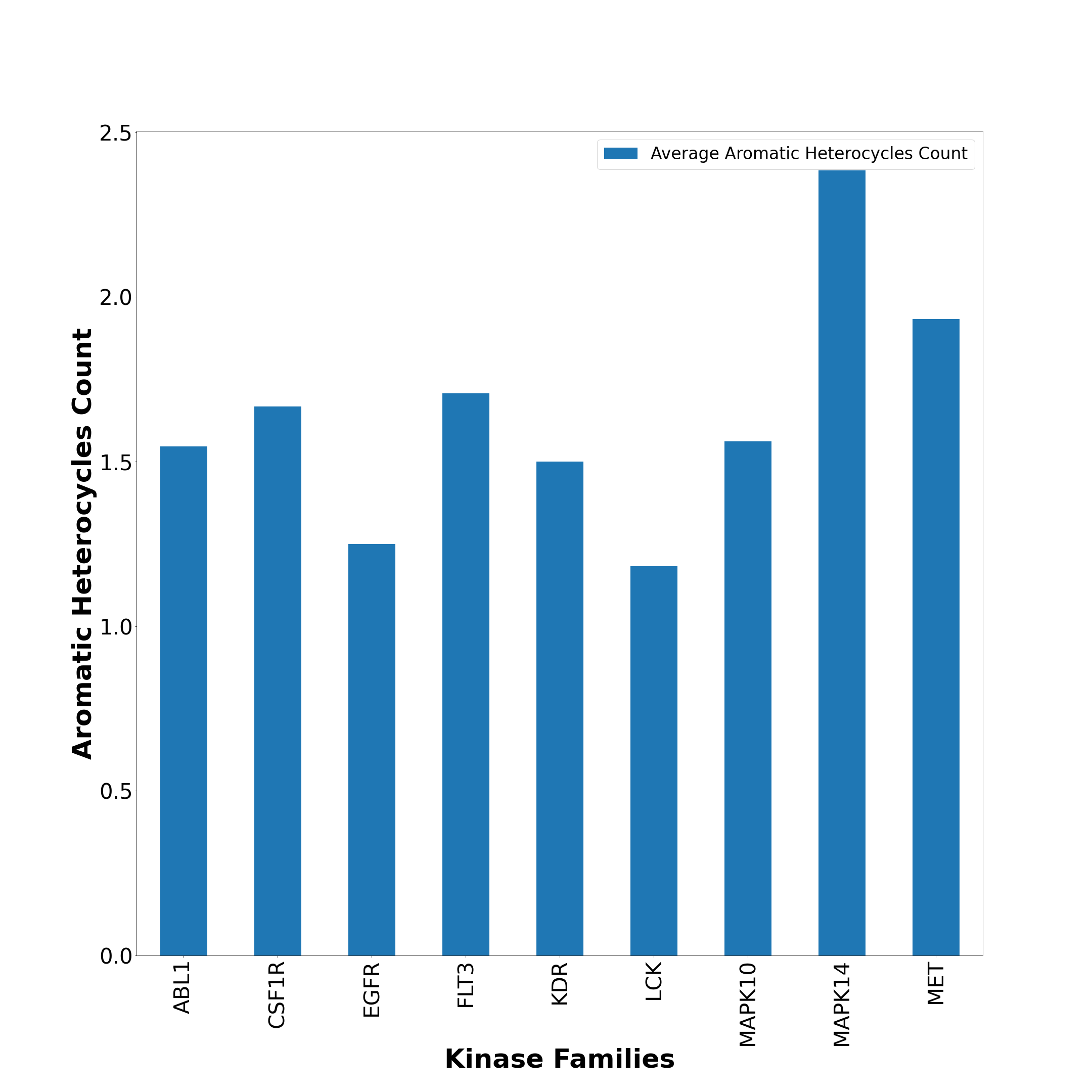

Supplement: Supplementary file 1 [file ijms-23-11262-s001.zip › SUPPLEMENTARY_MATERIALS/Graphs_of_Chemical_Properties/arohetero_bar.png]

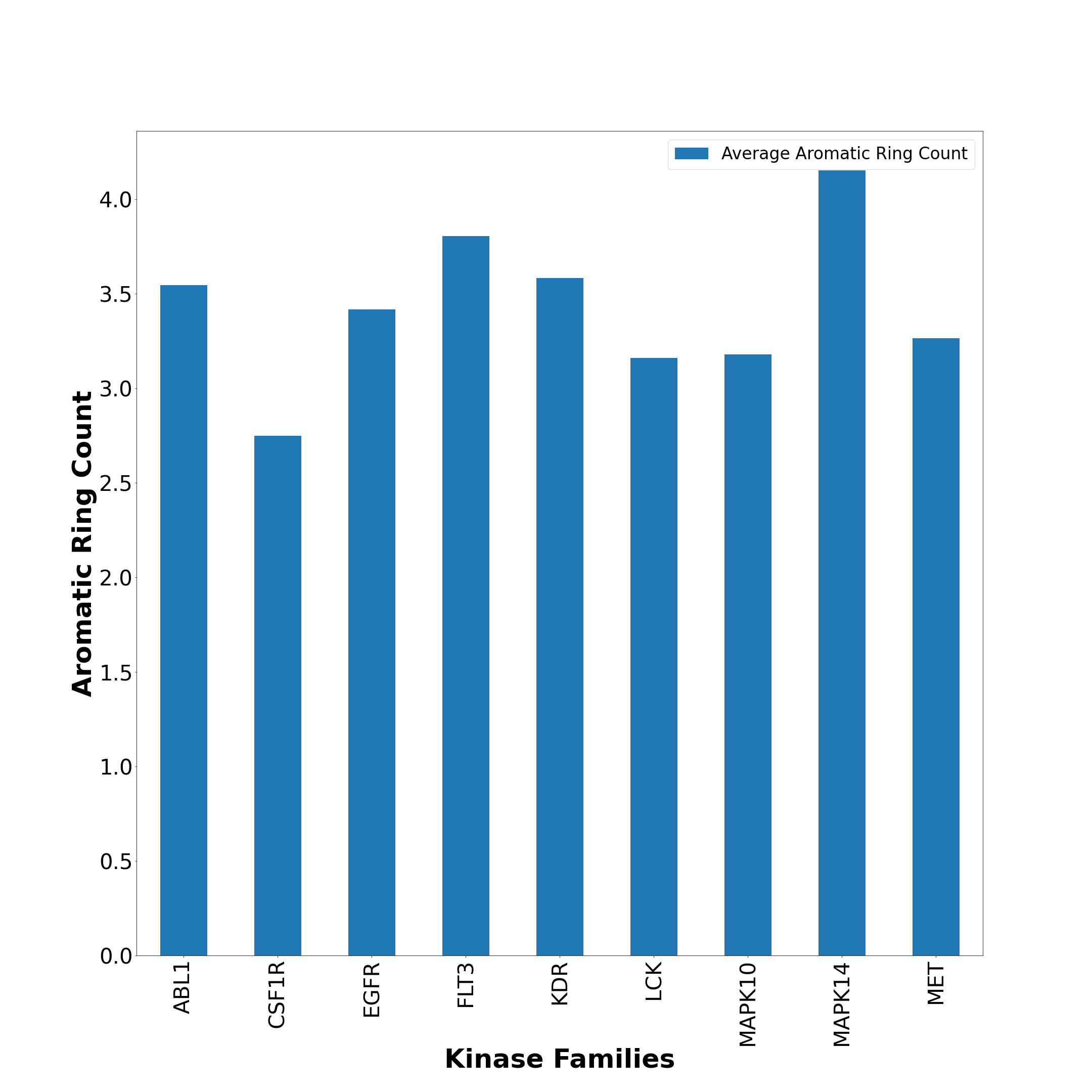

Supplement: Supplementary file 1 [file ijms-23-11262-s001.zip › SUPPLEMENTARY_MATERIALS/Graphs_of_Chemical_Properties/arorings_bar.png]

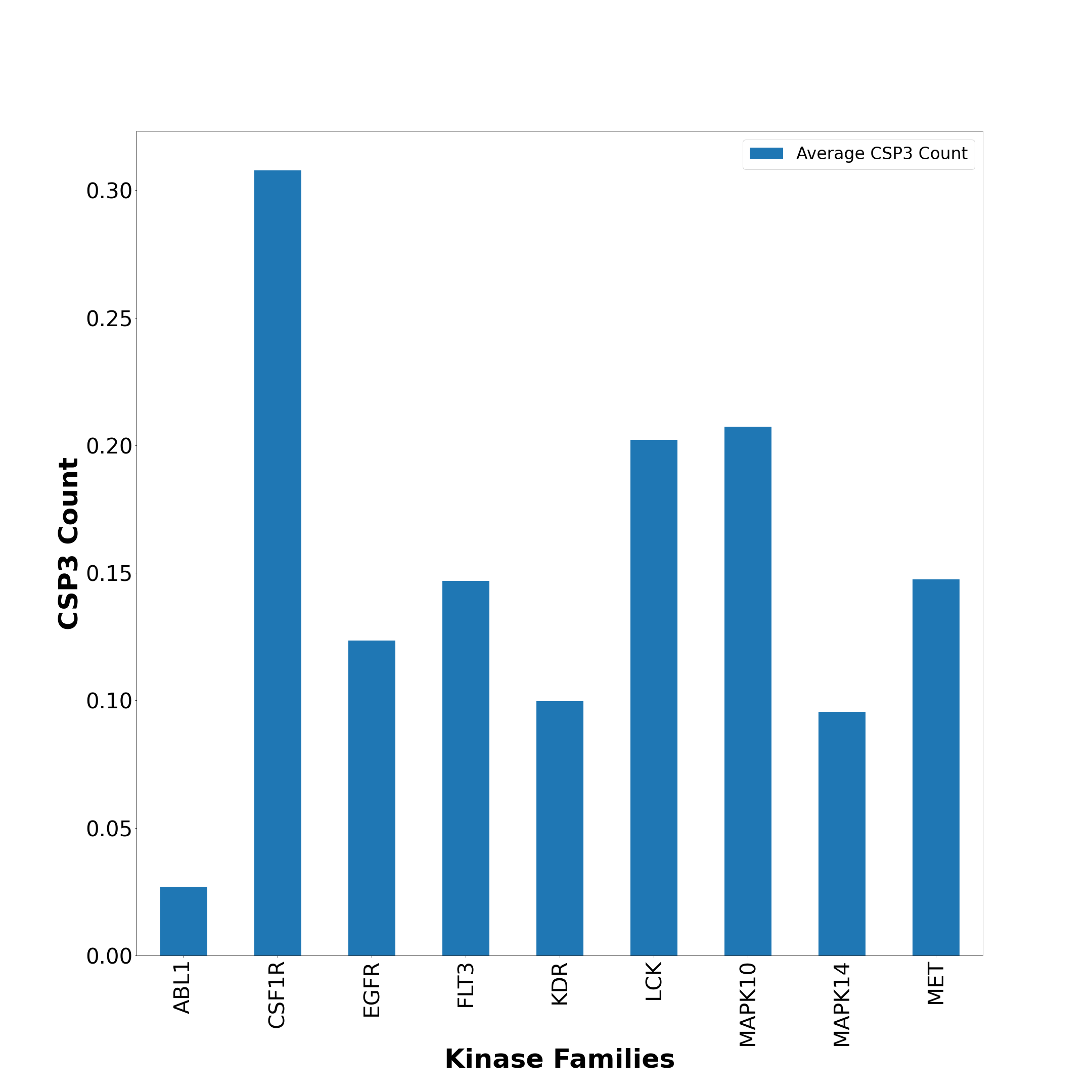

Supplement: Supplementary file 1 [file ijms-23-11262-s001.zip › SUPPLEMENTARY_MATERIALS/Graphs_of_Chemical_Properties/csp3_bar.png]

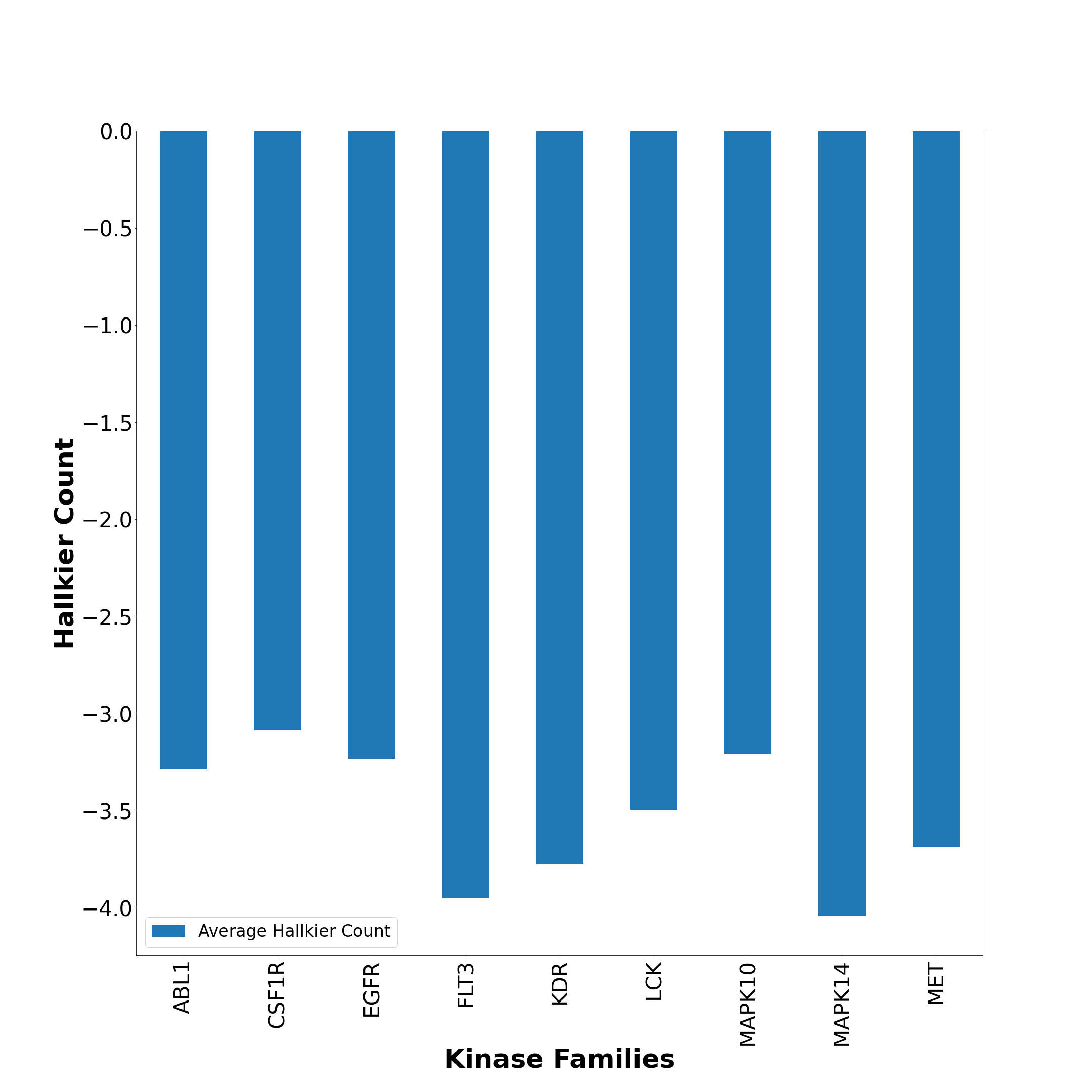

Supplement: Supplementary file 1 [file ijms-23-11262-s001.zip › SUPPLEMENTARY_MATERIALS/Graphs_of_Chemical_Properties/hallkier_bar.png]

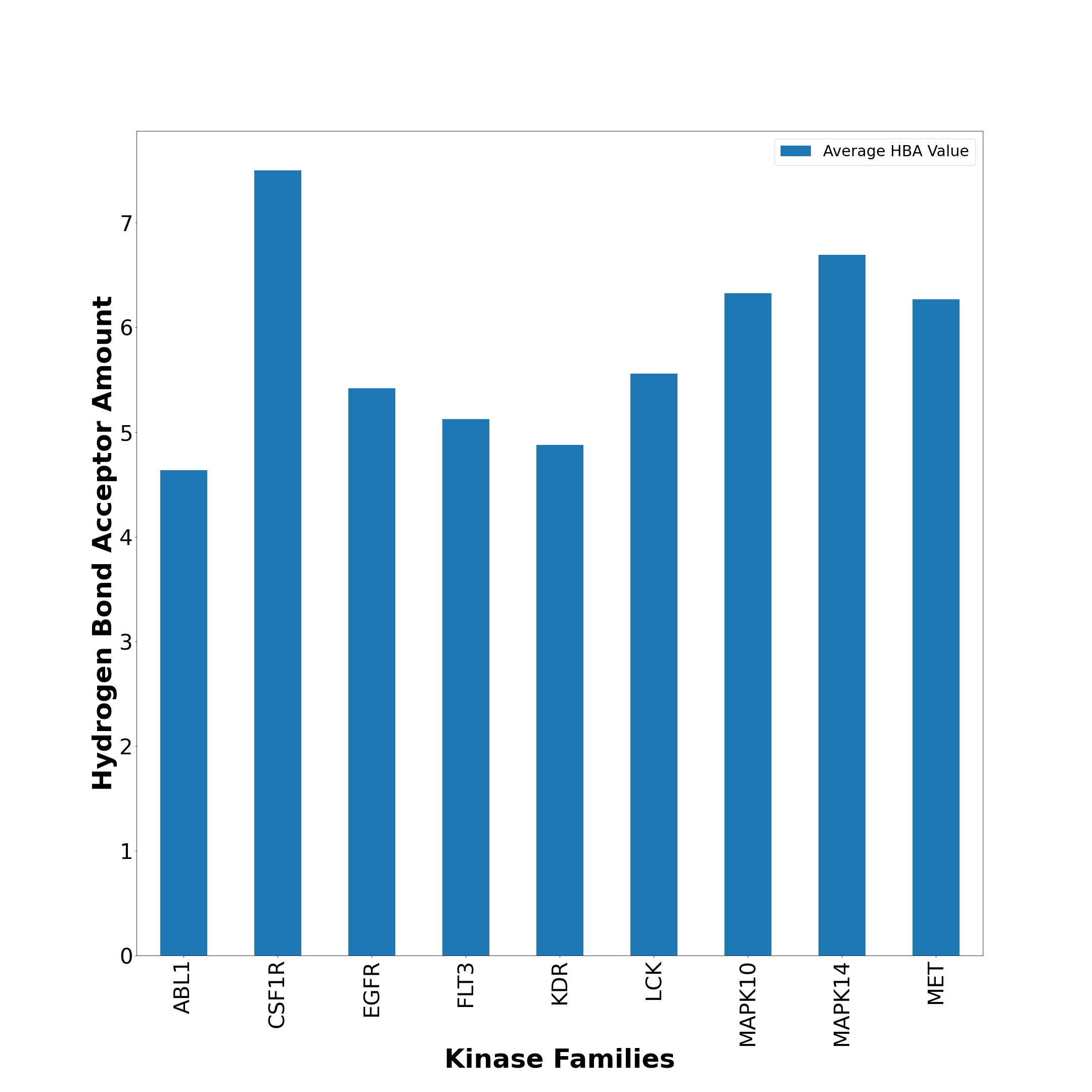

Supplement: Supplementary file 1 [file ijms-23-11262-s001.zip › SUPPLEMENTARY_MATERIALS/Graphs_of_Chemical_Properties/hba_bar.png]

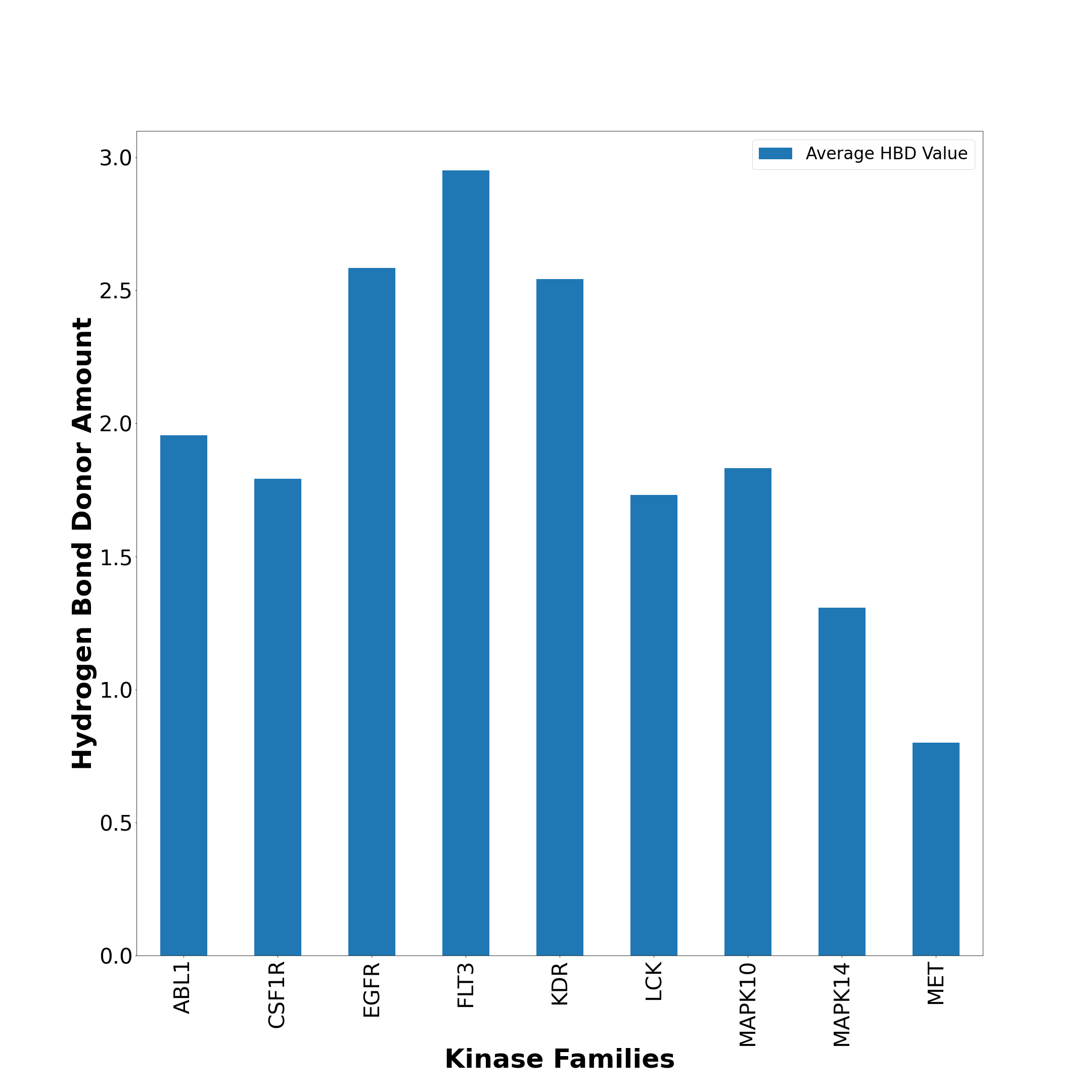

Supplement: Supplementary file 1 [file ijms-23-11262-s001.zip › SUPPLEMENTARY_MATERIALS/Graphs_of_Chemical_Properties/hbd_bar.png]

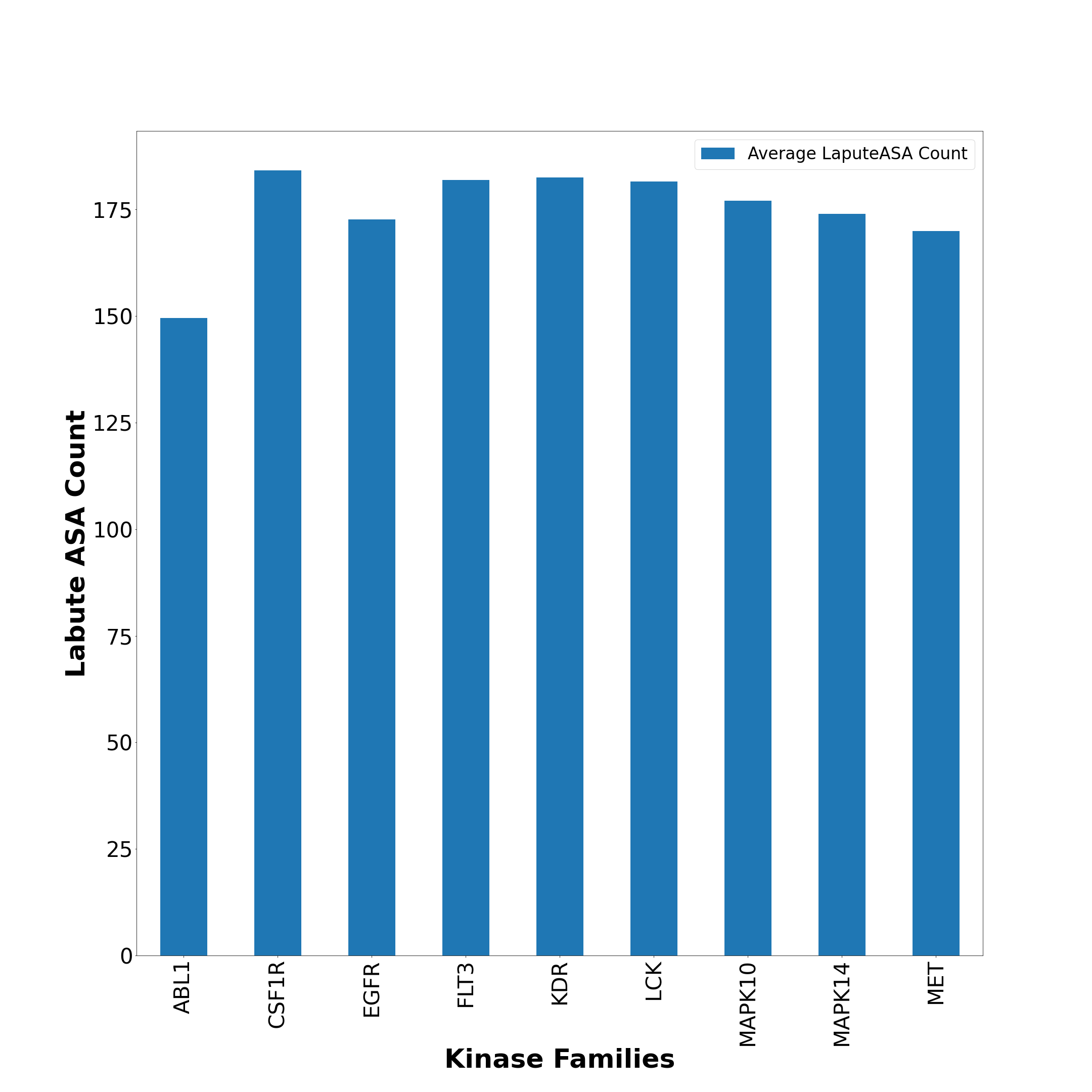

Supplement: Supplementary file 1 [file ijms-23-11262-s001.zip › SUPPLEMENTARY_MATERIALS/Graphs_of_Chemical_Properties/labuteasa_bar.png]

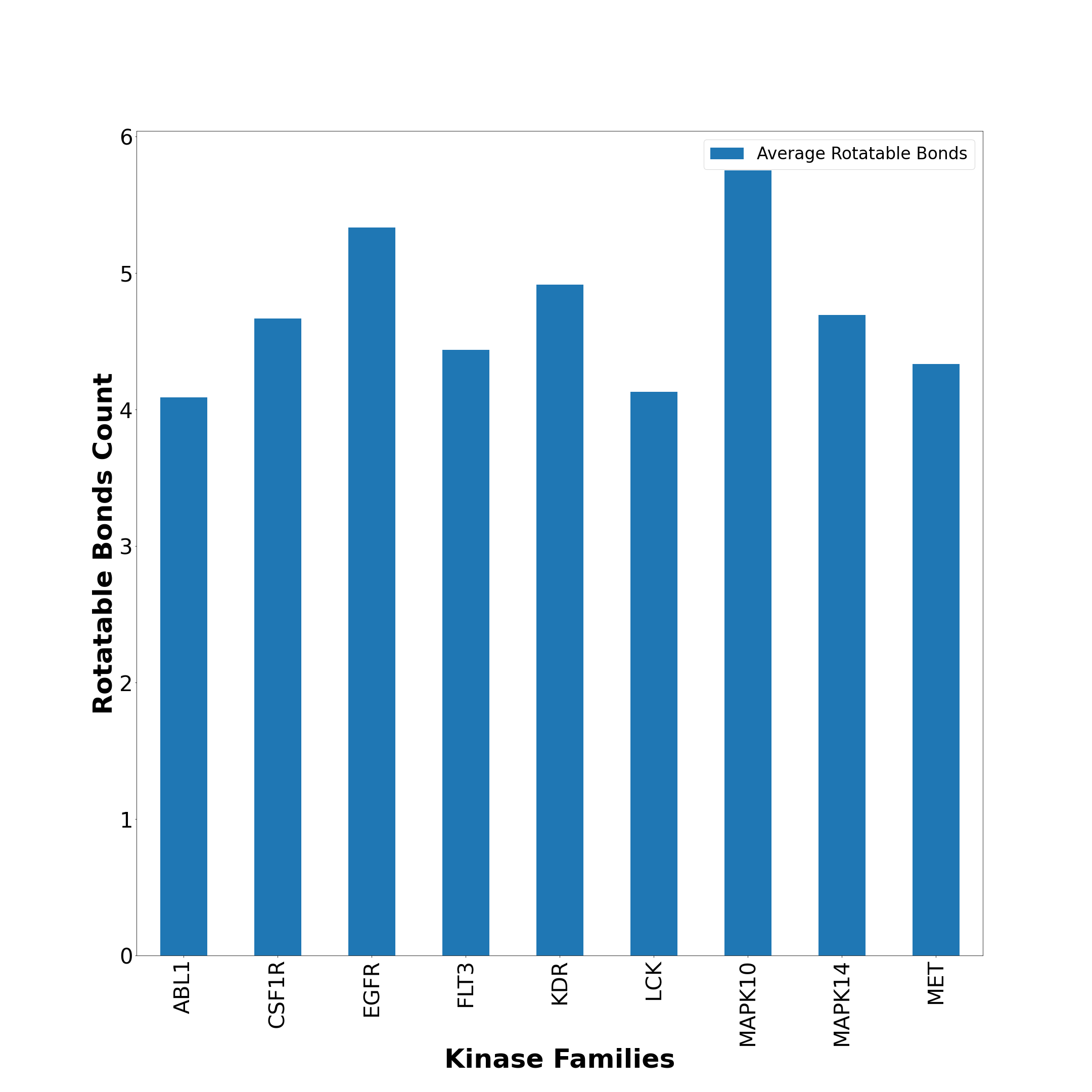

Supplement: Supplementary file 1 [file ijms-23-11262-s001.zip › SUPPLEMENTARY_MATERIALS/Graphs_of_Chemical_Properties/rotatable_bar.png]

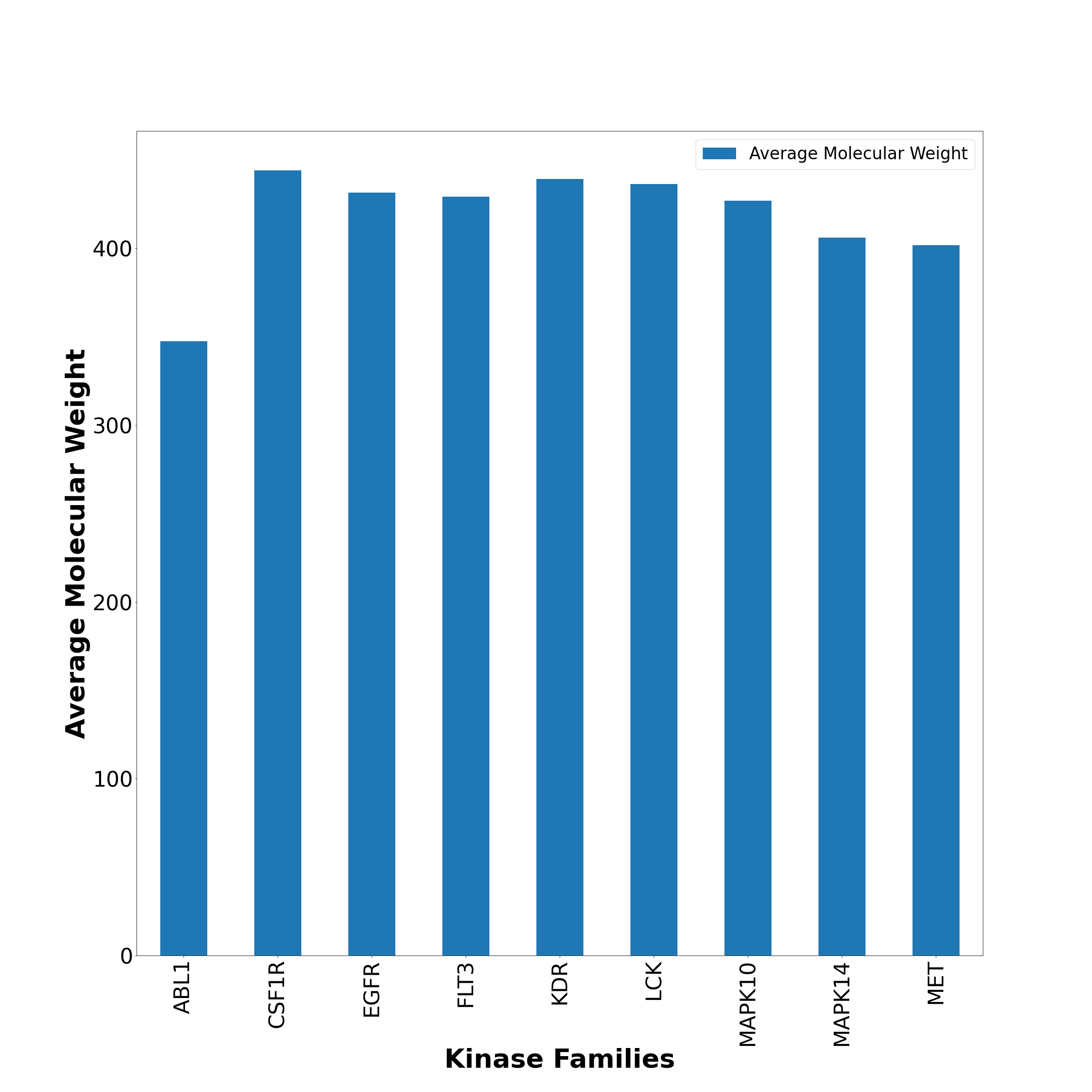

Supplement: Supplementary file 1 [file ijms-23-11262-s001.zip › SUPPLEMENTARY_MATERIALS/Graphs_of_Chemical_Properties/weight_bar.png]
